# Plus ça change – evolutionary sequence divergence predicts protein subcellular localization signals

**DOI:** 10.1186/1471-2164-15-46

**Published:** 2014-01-20

**Authors:** Yoshinori Fukasawa, Ross KK Leung, Stephen KW Tsui, Paul Horton

**Affiliations:** 1 Department of Computational Biology, Graduate School of Frontier Sciences, University of Tokyo, Kashiwa, Japan; 2 Japan Society for the Promotion of Science, Tokyo Chiyoda, Japan; 3 Hong Kong Bioinformatics Centre and School of Biomedical Sciences, Chinese University of Hong Kong, Shatin, China; 4 Computational Biology Research Center, Advanced Industrial Science and Technology, Tokyo, Japan

## Abstract

**Background:**

Protein subcellular localization is a central problem in understanding cell biology and has been the focus of intense research. In order to predict localization from amino acid sequence a myriad of features have been tried: including amino acid composition, sequence similarity, the presence of certain motifs or domains, and many others. Surprisingly, sequence conservation of sorting motifs has not yet been employed, despite its extensive use for tasks such as the prediction of transcription factor binding sites.

**Results:**

Here, we flip the problem around, and present a proof of concept for the idea that the *lack* of sequence conservation can be a novel feature for localization prediction. We show that for yeast, mammal and plant datasets, evolutionary sequence divergence alone has significant power to identify sequences with N-terminal sorting sequences. Moreover sequence divergence is nearly as effective when computed on automatically defined ortholog sets as on hand curated ones. Unfortunately, sequence divergence did not necessarily increase classification performance when combined with some traditional sequence features such as amino acid composition. However a post-hoc analysis of the proteins in which sequence divergence changes the prediction yielded some proteins with atypical (i.e. not MPP-cleaved) matrix targeting signals as well as a few misannotations.

**Conclusion:**

We report the results of the first quantitative study of the effectiveness of evolutionary sequence divergence as a feature for protein subcellular localization prediction. We show that divergence is indeed useful for prediction, but it is not trivial to improve overall accuracy simply by adding this feature to classical sequence features. Nevertheless we argue that sequence divergence is a promising feature and show anecdotal examples in which it succeeds where other features fail.

## Background

Since proper subcellular localization is a prerequisite for protein function, there is a high demand for accurate and complete localization annotation of all proteins [1]. Although proteomics data has allowed large scale determination of protein localization for model organisms [2,3], no experimental evidence is available for the vast majority of organisms. Although sequence similarity can be a good indicator of identical localization site [4], distant similarity is not [5], and thus for many proteins we must rely on computer prediction.

In cells, the localization of proteins is largely determined by “zip-code” like sorting signals, encoded in their amino acid sequence [6]. Unfortunately these sorting signals seem to be only very loosely determined, accepting very diverse sequences, subject to some constraints on their physico-chemical properties [7].

Among those signals, the most well-known sorting signal is the signal peptide of secretory path proteins. A typical signal peptide spans 15–30 amino acids near the N-terminus. Signal peptides typically show three distinct blocks: the n-region containing positively charged residues, the h-region mainly consisting of hydrophobic residues, and the c-region which includes polar uncharged residues and a weakly conserved cleavage motif [8].

Similarly, the targeting signals of mitochondria and chloroplasts are also N-terminally coded [7], and cleaved after import to their final location. In the mitochondria matrix, the N-terminal signal is usually cleaved off by the Mitochondrial Processing Peptidase MPP [9,10], while the corresponding chloroplast targeting N-terminal signals are processed by an analogous protease in the chloroplast stroma [10]. Like signal peptides, these signals are often poorly conserved and difficult to align properly between orthologs [11]. Although some consensus motif has been reported for mitochondrial targeting signals [12,13], it is information poor and produces too many false positives to be used for reliable prediction.

To date, an impressive number of methods have been developed for protein sorting prediction. For example, in 2004 a survey already listed dozens of methods employing fifteen broad categories of features [14]; from commonly used ones such as amino acid composition [15-19] (and many more) to rare categories such as sequence periodicity [20] and mRNA expression level [21]. Sequence similarity as defined by programs such as BLASTP has been explored as a feature for signal peptide detection [22]. Among these features, amino acid composition is attractive due to its simplicity. The significant correlation between amino acid composition and sub-cellular location is partially causative and partially due to indirect effects such as adaption of surface residues to the pH of the protein’s localization site [23].

The one feature conspicuously missing from this list has been evolutionary sequence conservation, despite the fact that it has seen extensive use in sequence analysis from the prediction of transcription factor binding sites [24], to short linear motifs in proteins [25] and functional RNA [26]. Although profile feature methods which indirectly reflect evolutionary conservation have been employed [27], sequence conservation per se has not – presumably because sorting signals are indeed not well conserved at the sequence level. Here, we propose that instead of looking for sequence conservation of sorting signals, a more effective approach is to exploit their high evolutionary sequence *divergence*.

In this paper we first describe our datasets of yeast, animal and plant proteins with their orthologs, divergence and other features we used for classification, and the classifiers we employed. Then, we present a simple statistical feature analysis followed by performance evaluation of localization prediction for various combinations of features, classifiers and datasets. Unfortunately, combining other features with our sequence divergence did not lead to a systematic improvement in overall performance. However we show that consideration of sequence divergence is critical for correct prediction in certain cases and can sometimes flag non-cleaved or misannotated targeting signals. Finally we discuss future directions and conclude.

## Methods

### Sorting signal classes

We mainly focused on the two most common N-terminal sorting signals: *Signal Peptide*s (SP), targeting proteins to the endoplasmic reticulum and *Matrix Targeting Signal*s (MTS) which target proteins to the matrix (inner compartment) of the mitochondria. In the plant dataset, we also consider *Chloroplast Transit Peptide*s (CTP). All of these signals reside near the N-terminus but in general have different properties and are effectively discriminated by the cell. In some cases however, the N-terminal “signal” can be ambiguous. In particular many examples are known in which the same amino acid sequence directs some copies of a protein to the mitochondria and others to the chloroplast [28,29]. Nevertheless these examples still constitute only a small percentage of proteins and therefore we simplify the analysis by treating N-terminal sorting signal identification as a simple three- or four-way classification problem: {MTS, SP, (CTP), no signal}. Other types of N-terminal sorting signals exist, for example the PTS2 signal targeting proteins to the peroxisome [30], but the number of proteins using such signals is much smaller than those using the SP, MTS or CTP signals.

The sorting signal class labels we use in our datasets are partially based on direct experimental evidence. In the dataset of *S.cerevisiae*, we used UniProtKB/Swiss-Prot [31] to assign localization class labels, augmented by MTS containing proteins determined in the proteomics experiment of Vögtle et al. [32]. Because only a small number of SP’s have been directly confirmed experimentally, we also included proteins whose SP is inferred in the database and predicted positive by SignalP [33]. We used proteins annotated to localize to the cytosol or nucleus as proteins without N-terminal signals. To reduce bias in training and accuracy estimation, we used BLASTClust 2.2.22 [34] to remove redundant sequences with a setting of 20% identity. For proteins in human and a few plant species we adopted the dataset of Predotar [35] and for plants augmented that small number by experimental proteomics data determined in the mass spectrometry experiment of Huang et al. [11].

### Dataset

#### Organisms used

We gathered protein sequences from 11 relatively diverse and well annotated representative species of the three phylogenetic divisions: yeast, mammal and plant respectively (Table 1). The 11 mammal species and most of the plant species are annotated reference proteomes in UniProt, but a few of the plant species are only included in UniProt as complete, but not fully annotated, proteomes. Note that our “plant” dataset contains the unicellular green algae *Chlamydomonas reinhardtii*, which is not a typical plant but is classified in the “viridiplantae” kingdom.

**Table 1:** List of species used to define orthologs in each phylogenetic category

***S. cerevisiae***	***H. sapiens***	***A. thaliana***
*Saccharomyces castellii*	*Gorilla gorilla*	*Glycine max*
*Saccharomyces kluyveri*	*Otolemur garnettii*	*Ricinus communis*
*Kluyveromyces waltii*	*Mus musculus*	*Populus trichocarpa*
*Ashbya gossypii*	*Oryctolagus cuniculus*	*Vitis vinifera*
*Candida glabrata*	*Sus scrofa*	*Sorghum bicolor*
*Kluyveromyces lactis*	*Ailuropoda melanoleuca*	*Brachypodium distachyon*
*Zygosaccharomyces rouxii*	*Myotis lucifugus*	*Oryza sativa*
*Kluyveromyces thermotolerans*	*Loxodonta africana*	*Selaginella moellendorffii*
*Saccharomyces bayanus*	*Sarcophilus harrisii*	*Physcomitrella patens*
*Kluyveromyces polysporus*	*Ornithorhynchus anatinus*	*Chlamydomonas reinhardtii*

The species listed at top are the reference species used to determine the subcellular localization site class labels. In the case of plants, one of *G. max*, *O. sativa* and *C. reinhardtii* were used as the reference species for proteins for which no annotation was available in *A. thaliana*.

In each of the three divisions we designated one species as the “reference” species. We used information in proteins from the non-reference species only for computation of sequence divergence (via ortholog multiple sequence alignments). We chose *S.cere.*, *H. sapiens*, and *A. thaliana* as the reference species for yeast, animals and plants respectively, because they have the most complete annotation. However for plants even *A. thaliana* has rather limited annotation of SPs, so in order to increase the plant dataset size we used other species as the reference species in some cases.

#### Ortholog determination

We performed some experiments on hand curated ortholog sets downloaded from the Yeast Gene Order Browser (YGOB) [36], but also computed ortholog sets for each of the three phylogenetic divisions.

Automatic identification of orthologs is a complex subject for which many sophisticated methods have been developed, the most suitable one being application dependent [37]. For this study, we adopted a simple procedure based on reciprocal best hits (RBHs) [38]. Formally, proteins *P* and *P*^′^ from species *S* and *S*^′^ respectively, are RBHs if *P* is more similar to *P*^′^ than any other protein in *S*^′^ and *P*^′^ is more similar to *P* than any other protein in *S*. We define the ortholog set of a reference species protein as all of its RBHs. When computing RBHs it is important that proteins from as many organisms as possible are included; but in the end we only have use for those ortholog sets in which the reference species is annotated, so in general we discarded the rest. However, in the case of plant, we attempted to rescue those discarded sequences by also trying *O. sativa*, *G. max* and *C. reinhardtii* in turn as the reference species.

In computing the similarity scores for RBH we chose to use global alignment rather than local alignment. Our motivation for this was: 1) sorting signals often appear on the N- or C-terminal region of proteins, so differences in those regions may indicate a different localization of the “ortholog”, and 2) for multiple domain proteins, strong similarity in one domain may not imply the same localization site (or signal). We used the heuristic but fast USEARCH [39] program with its default parameters to compute the global similarity scores. Table 2 summarizes the datasets.

**Table 2:** The number of ortholog sets by localization class in each phylogenetic division

**Localization**	***S.cere. *** **curated**	***S.cere. *** **RBH**	***H.sapiens *** **RBH**	**Plants RBH**
**class**	**orthologs**			
MTS	179	219	81	61
SP	53	73	169	15
CTP	N/A	N/A	N/A	97
N-signal-free	450	560	415	99

For each ortholog dataset, the number of ortholog sets in each localization class is listed. RBH orthologs are defined by the reciprocal best hit method.

#### Multiple alignment

**Figure 1:**
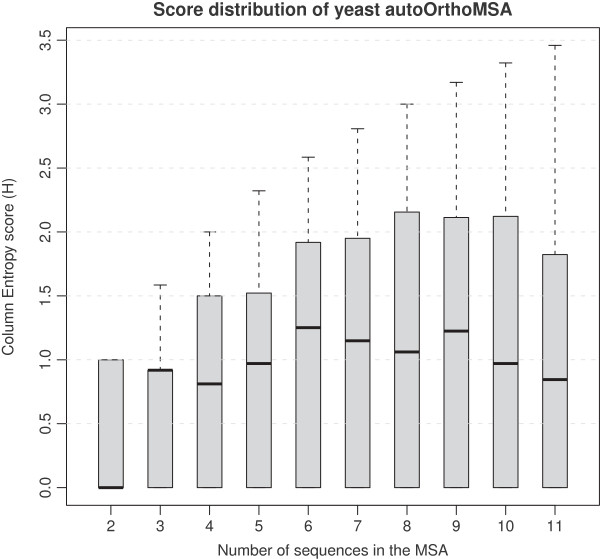
**Relationship between mean divergence score and the number of sequence in MSA’s.** A box plot illustrating the mean, quartiles and range of the column entropy score for MSA’s in the yeast autoOrthoMSA dataset partitioned by the number of sequences in the MSA.

We computed multiple alignments for each of the 4 orthologs sets (1 curated and 3 automatic) by aligning with the MAFFT program [40], using “LINSI”, its most accurate mode. Hereafter, we denote these alignments as “orthoMSA” in general, and as “autoOrthoMSA” when specifically referring to multiple alignments of automatically generated ortholog sets. The number of sequences in the automatically generated ortholog sets generally differs from the YGOB based sets, however, it seems that the distribution of the divergence score stabilizes when the number of sequences exceeds three (Figure 1), therefore we decided to include ortholog sets with at least four sequences.

### Features for classification

#### Column entropy score

Several measures have been suggested for scoring evolutionary sequence conservation (or conversely divergence) [41,42]. Here we adopt a simple Shannon entropy based score. The Shannon entropy *H*(*i*) of the *i*th column of an orthoMSA is defined as:

(1) $$H(i)=-\underset{j\in A}{\sum }F(i,j){\text{log}}_{2}F(i,j).$$

where *A* denotes the set of 20 amino acid characters plus gap characters, and *F*(*i*,*j*) denotes the frequency of character *j* in column *i* of an orthoMSA. Note that when multiple gap characters are present in a column, we consider each to be a unique character. For example, the entropy of an orthoMSA column ‘{L, L, I, -, -}’ is computed as one character (the ‘L’) with frequency 0.4 and three characters with frequency 0.2, because we treat the two ‘-’ characters as distinct. We adopted this treatment of gap characters so that the divergence of orthoMSA columns with many gaps is considered high (we also tried using straight entropy, but the results, not shown, were slightly worse). The range of this divergence score runs from 0 to log2*n*, where *n* is the number of sequences.

#### Divergence based features

For many orthoMSA’s, the entropy often varies widely from column to column. Therefore, we defined a number of evolutionary divergence features based on a smoothed entropy score, ${\bar{H}}_{i,j}$, defined as the average entropy score for columns in the interval [ *i*,*j*]. For example we define the local divergence (LD) of an orthoMSA at position *k* as ${\bar{H}}_{k-10,k+10}$. Another feature we defined is NCdiff, the average difference in divergence between in the first 20 residues and residues 80 to 99. Our motivation for this definition was the hope that subtracting the divergence from residues 80 to 99 would approximately normalize the feature when comparing proteins with different overall rates of evolution. These features are summarized in Table 3.

**Table 3:** List of entropy derived features

**Feature name**	**Quantity**
LD(*i*)	${\bar{H}}_{i-10,i+10}$
*N*_raw_20	${\bar{H}}_{1,20}$
*N*_raw_40	${\bar{H}}_{1,40}$
*N*_raw_80-99	${\bar{H}}_{80,99}$
*μ*_*w*_	Average of ${\bar{H}}_{\text{window}}$ for all length *w* windows
*σ*_*w*_	Standard deviation of ${\bar{H}}_{\text{window}}$ for all length *w* windows
NCdiff	*N*_raw_20−*N*_raw_80-99
*N*20	$\frac{\left(N_{\text{raw}}20-\mu_{20}\right)}{\sigma_{20}}$ (z-score normalized)
*N*40	$\frac{\left(N_{\text{raw}}40-\mu_{40}\right)}{\sigma_{40}}$ (z-score normalized)
*N*80-99	$\frac{\left(N_{\text{raw}}80\text{-}99-\mu_{20}\right)}{\sigma_{20}}$ (z-score normalized)

#### Physico-chemical propensities

To explore the possibility of combining sequence divergence with standard features used in protein localization prediction, we defined three features computed from the first 20 or 40 N-terminal residues of each *S.cere.* protein: 1) the number of positively charged residues (#pos), 2) the number of negatively charged residues (#neg), and 3) the average hydrophobicity as measured by the Kyte-Doolittle [43] index (Hphob).

#### Amino acid composition

Amino acid composition is another standard feature for protein localization. We tested this feature computed on the first 20 residues, the first 40 residues, and the entire protein sequence.

### Classifiers

#### Majority class classifier

The majority class classifier unconditionally predicts all examples to belong to the most common class. Its accuracy is equal to the fraction of examples belonging to the most common class.

#### J48

J48 is a version of the C4.5 decision tree induction algorithm of Quinlan [44,45], implemented in the Weka software package [46]. We used the default value of 0.25 for the confidence factor, which controls the complexity of the induced tree.

#### Support vector machine

The Support Vector Machine (SVM) [47] is perhaps the most popular classifier in current bioinformatics work. In its basic form it is a linear, binary classifier, but it has been extended to non-linear, multiclass classification. In this project, we used the LIBSVM implementation [48]. We used the Gaussian radial basis kernel function with default *γ* value (1.0/# number of features). We used 50.0 for the SVM cost parameter *C*, because with the default cost parameter (1.0) prediction by RBF kernel failed for some features. In our study we conducted binary and 3-class classification. For multiclass discrimination LIBSVM adopts the “one-versus-one” method, in which a separate SVM is learned for each pair of classes, and majority voting among those SVM’s is used when classifying examples [49].

##### Measuring the influence of divergence features

As reported in the Results section, we performed a post-hoc analysis of proteins for which the divergence features greatly influenced the prediction outcome. To do this we needed to compare 6 numbers (three SVM scores {MTS vs SP, MTS vs none, SP vs none} each computed with and without the divergence features) into a measure of how much the divergence features influenced the prediction. Because the SVM scores are not given directly as probabilities and each individual SVM addresses a different subset of classes, it is not trivial to derive a well-principled way to do this. As described in more detail in the Additional file 1, we chose to define this in terms of exponential loss-based decoding [50]. We do not claim that this is necessarily the best measure, but it appears to give reasonable results. Fortunately, for our purposes it is enough that truly large differences are assigned in a roughly suitable order.

### Quantifying feature importance

We used the so called “information gain” to quantify the importance of each feature. Information gain is a simple measure of the predictive power of a feature in isolation (i.e. without consideration of its relationship to other features), defined as:

(2) I(C,F)=H(C)−H(C|F).

where *C* and *F* denote class and feature respectively. *H*(*C*) the denotes information theoretic entropy of the overall distribution of the class labels, while *H*(*C*|*F*) denotes the conditional entropy of the class label when feature F is given. A larger information gain indicates greater predictive power. Because the divergence based features have a large number of possible values, we first binned those values into a smaller number by the method of Fayyad & Irani [51].

### Classification performance evaluation

Accuracy is not always the most meaningful measure of performance for skewed datasets (i.e. datasets with a very uneven number of examples from different classes) [52]. Therefore we report several measures in addition to accuracy.

#### Matthews correlation coefficient

The Matthews correlation coefficient, MCC [53,54], is a measure of performance for binary classification defined as follows:

(3) $$\frac{\text{TP}\times \text{TN}-\text{FP}\times \text{FN}}{\sqrt{\left(\text{TP}+\text{FN})(\text{TP}+\text{FP})(\text{TN}+\text{FP})(\text{TN}+\text{FN}\right)}}$$

where “T” and “F” stand for “true” and “false”, while “N” and “P” stand for “negative” and “positive”. Equivalently, MCC can be defined as the Pearson’s correlation coefficient of the binary vector of class labels compared to the binary vector of predicted class labels. MCC ranges from 1.0 for perfect prediction to -1.0 for perfect inverse prediction. Note that the MCC of the majority class classifier is identically zero, as is the expected value of MCC under random prediction.

#### Area under the ROC curve

The Area under the curve (AUC) for a receiver operating characteristics (ROC) graph is a widely used metric to evaluate binary classification accuracy [55]. The usual way to generate an ROC plot is to rank instances by their predicted scores with increasing threshold values, plotting true positive rate (y-axis) versus false positive rate (x-axis). AUC ranges from 0 to 1.0, with perfect prediction yielding 1.0 and perfectly wrong prediction 0.0. AUC can be interpreted as the probability that a classifier is able to distinguish a randomly chosen positive example from a randomly chosen negative example [56]. For this task, the majority class classifier gives no information over coin flipping and therefore can be considered to yield an AUC of 0.5.

## Results

### Feature analysis

#### N-terminal sorting signals are evolutionary divergent

**Figure 2:**
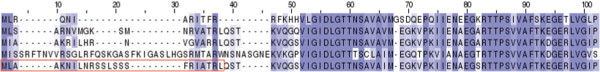
**An example of MTS containing protein.** A multiple sequence alignment of the protein mtHSP70 (UniProt accession P0CS90) and its orthologs from five species of yeast. The red box indicates the cleaved MTS in *S.cere*. Conserved positions are colored by Jalview.

**Figure 3:**
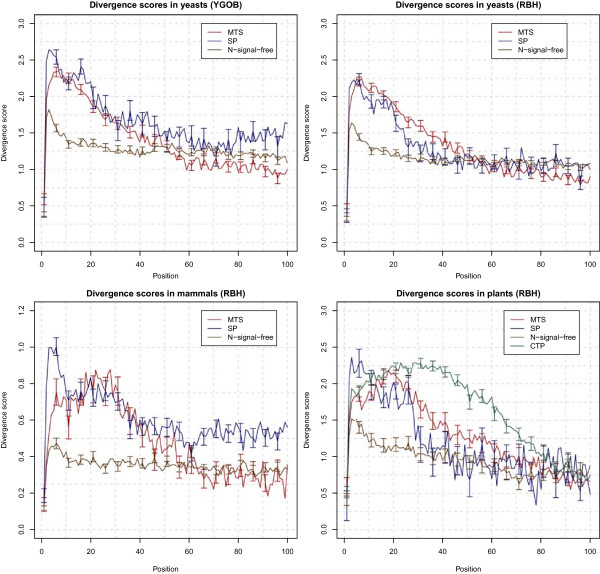
**Local divergence score over N-terminal region.** Average local divergence scores are shown for the 100 residue N-terminal region of: MTS containing, SP containing, and N-signal-free proteins. Top left panel is calculated from orthologs of yeast curated dataset, and the others from automatically collected orthologs. For the plant dataset, CTP containing proteins are also shown. The error bars denote standard error. For clarity, error bars are only shown for every fifth position.

It is well known that N-terminal sorting signals exhibit relatively low sequence conservation [57]. As shown in Figure 2, this phenomenon is particularly clear for the mitochondrial heat shock protein, mtHSP70, in which the main part of the protein is highly conserved but the N-terminal region is highly divergent. Figure 3 quantifies this trend for the proteins in the YGOB ortholog set.

#### Estimate of importance of each feature

**Figure 4:**
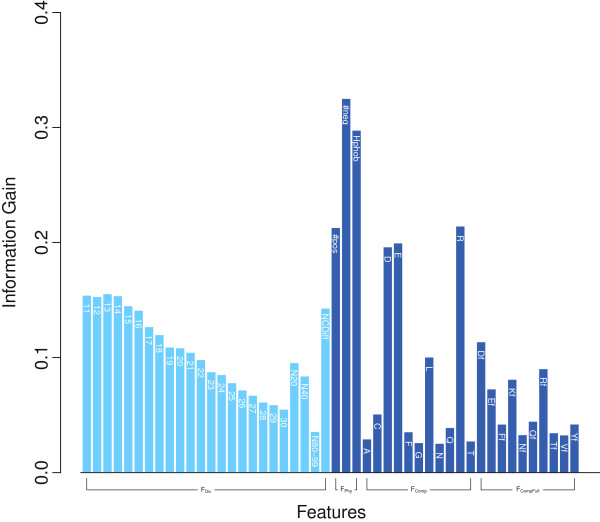
**Importance of each feature.** The importance of each attribute as estimated by information gain is shown for the YGOB ortholog set. At left, the divergence related scores are shown by light blue color lines. For local divergence features LD(*i*), only the residue number *i* is listed. Dark blue colored lines denote standard features of the N-terminal 40 residues such as physico-chemical properties or amino acid composition. The suffix “f” denotes amino acid composition from the full length of the protein.

As a rough estimate of feature importance, we computed the information gain for each feature (Figure 4). The two highest scoring features are the physico-chemical features #neg and Hphob, but the LD features near the N-terminus also show information gain significantly greater than zero.

#### Sequence divergence is not redundant to physico-chemical trends or amino acid composition

**Figure 5:**
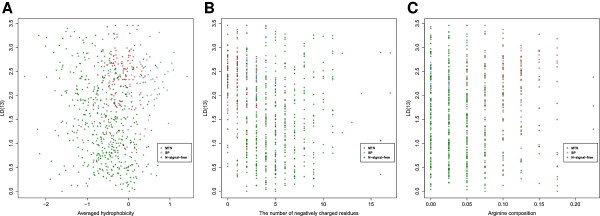
**Correlation between divergence and physico-chemical properties.** Scatter plots of LD(13) (on the vertical axis) vs physico-chemical property **(A)** average hydrophobiciy, **(B)** number of negatively charged residues and **(C)** arginine composition for the YGOB ortholog set (MTS proteins are shown in red, SP in blue and N-signal-free proteins in green).

To be promising as a feature for prediction, it is desirable that evolutionary sequence diversity not be perfectly correlated with other features. To investigate this we plotted LD(13), the divergence feature with the highest information gain, against Hphob, #neg and the arginine composition (the three highest scoring standard features in the 40 residue N-terminal region) (Figure 5). Although there may be some relationship, the feature pairs do not appear highly correlated.

### Divergence predicts the presence of N-terminal signals

We tested whether sequence divergence can be used to distinguish between proteins with an N-terminal localization signal (MTS or SP) and those with none. As shown in Table 4, for this binary classification task, sequence divergence *alone* allows for significantly higher prediction accuracy than randomized control experiments or the majority class fraction (66.0%) in the yeast dataset.

**Table 4:** Performance of N-signal vs N-signal-free protein binary classification

	**Mean accuracy**	**Mean AUC**	**Mean MCC**
J48	72.49±3.30	**0.68**±0.09	**0.40**±0.09
- (randomized)	65.85±0.66	0.50±0.01	0.00±0.03
SVM	**74.64**±2.38	**0.68**±0.03	**0.40**±0.06
- (randomized)	66.19±0.09	0.50±0.00	0.00±0.00
The majority class fraction	65.98*%*	N/A	N/A

Three classification performance measures when using only divergence features are shown for the discrimination of N-signal containing and N-signal-free proteins (yeast curated ortholog sets). AUC denotes the area under the ROC curves. (randomized) indicates the values obtained with the localization class labels randomly shuffled 100 times. For each measure the average and standard deviation is shown over the 5 folds of the cross-validation, or 500 (5 × 100 trials) folds in the case of the randomized data.

### Divergence distinguishes SP vs. MTS vs. N-signal-free

Although the sequence divergence profile of SP’s and MTS’s appear similar when averaged (Figure 3), we found that sequence divergence is still somewhat effective for the three-way classification of SP *vs* MTS *vs* N-signal-free. As shown in Table 5 the performance with divergence features is slightly better than the majority class fraction (66.0%) and also slightly improves the performance when added to the physico-chemical features in N-terminal 40 residues or amino acid composition in either N-terminal 40 or full length (Additional file 1).

**Table 5:** Performance of 3-way classification using SVM classifier

	**Divergence**		**Classical features**		**Combination**	
	**AUC**	**MCC**	**AUC**	**MCC**	**AUC**	**MCC**
MTS	0.67±0.03	0.36±0.06	**0.87**±0.03	0.76±0.05	**0.87**±0.03	**0.77**±0.03
SP	0.50±0.00	0.00±0.00	0.81±0.08	0.70±0.11	**0.90**±0.06	**0.83**±0.07
N-signal-free	0.66±0.02	0.36±0.03	0.85±0.03	0.72±0.05	**0.87**±0.02	**0.77**±0.03
*% accuracy*	70.82±1.61		87.24±1.86		**89.30**±0.66	

The 5-fold cross-validation performance of an SVM classifier using: divergence features only, classical features only, and the two combined; is shown for three-way classification on the yeast curated ortholog dataset. Classical features are computed based on the N-terminal 40 residues.

The ratio of examples in our dataset is 8.5:3.4:1, for N-signal-free, MTS and SP containing proteins respectively. Skewed datasets are known to complicate both learning and performance evaluation [52]. Therefore we also measured performance on a dataset with uniform class occupancy, created by randomly discarding all but 53 proteins from each class. As shown in Table 6, in this experiment the divergence feature only performance (63%) is much higher than the majority class fraction (33%), and the divergence features also contribute more to the performance when combined with the standard features (Table 6).

**Table 6:** Performance on balanced dataset for MTS vs SP vs N-signal-free protein prediction using SVM classifier

	**Divergence**		**Classical features**		**Combination**	
	**AUC**	**MCC**	**AUC**	**MCC**	**AUC**	**MCC**
MTS	0.67±0.10	0.35±0.20	0.84±0.07	0.68±0.13	**0.88**±0.05	**0.78**±0.09
SP	0.71±0.09	0.41±0.16	0.92±0.05	0.85±0.10	**0.94**±0.01	**0.88**±0.03
N-signal-free	0.79±0.07	0.60±0.13	0.78±0.09	0.57±0.18	**0.86**±0.07	**0.74**±0.13
*% accuracy*	62.86±5.84		79.92±5.54		**86.19**±4.67	

The 5-fold cross-validation performance of an SVM classifier using: divergence features only, classical features only, and the two combined; is shown for three-way classification on a balanced dataset (53 proteins from each class, yeast curated orthologs).

We further tested the prediction power of divergence features when combined with classical features computed on a 20 residue N-terminal instead of 40 (which might be too long for the SP class). In this experiment, divergence features improved the performance only slightly when combined with other standard features (Table 7). We also computed the confusion matrix for this dataset (Table 8) and the other datasets investigated in the study (Additional file 1: Tables S14–S25).

**Table 7:** Performance of 3-way classification using SVM classifier (feature length 20)

	**Divergence**		**Classical features**		**Combination**	
	**AUC**	**MCC**	**AUC**	**MCC**	**AUC**	**MCC**
MTS	0.67±0.03	0.36±0.06	**0.89**±0.02	0.80±0.02	**0.89**±0.01	**0.81**±0.02
SP	0.50±0.00	0.00±0.00	0.97±0.03	0.92±0.07	**0.98**±0.03	**0.97**±0.04
N-signal-free	0.66±0.02	0.36±0.03	**0.90**±0.01	0.81±0.02	**0.90**±0.01	**0.83**±0.02
*% accuracy*	70.82±1.61		91.49±1.26		**92.23**±1.25	

The 5-fold cross-validation performance of an SVM classifier using: divergence features only, classical features only, and the two combined; is shown for three-way classification on our entire yeast curated ortholog dataset. Classical features are calculated from N-terminal 20 amino acids.

**Table 8:** Confusion Matrix from 3-way classification using SVM classifier (feature length 20)

	**Divergence**			**Classical features**			**Combination**		
**Predicted →**	**MTS**	**SP**	**N-signal-free**	**MTS**	**SP**	**N-signal-free**	**MTS**	**SP**	**N-signal-free**
MTS	83	0	96	148	1	30	144	0	35
SP	16	0	37	0	50	3	1	51	1
N-signal-free	50	0	400	20	4	426	15	1	434

Confusion matrix of the 5-fold cross-validation performance of an SVM classifier using: divergence features only, classical features only, and the two combined; is shown for three-way classification on our entire yeast curated ortholog dataset. Classical features are calculated from N-terminal 20 amino acids.

### Divergence computed from automatically generated ortholog sets is consistent with the hand curated dataset

Although the YGOB based dataset convincingly demonstrates that the divergence score has discriminative power for N-terminal signal prediction, it covers only 11 yeast species and requires hand curation. Thus as described in the Methods section, in this work we adopted a simple procedure based on reciprocal best hit relationships to obtain automatically generated ortholog sets as well (Table 2).

In yeast, the average divergence score at each positions is similar to the score from the YGOB ortholog set, and the overall tendency looks similar for animals and plants (Figure 3). Interestingly, CTP shows a high and longer region of elevated divergence, consistent with previous observations that CTPs tend to be longer than MTSs [11]. Additionally, we note that the score range of the human autoOrthoMSA’s is significantly different from those of yeast or plants. This is expected because divergence amongst yeast sequences is at least as large as that of the chordates [58], so divergence in mammals should be smaller.

### Divergence computed from autoOrthoMSA also predicts N-terminal signals

First, we confirmed whether or not divergence features can be applied to a simple binary classification: discrimination between N-terminal signal containing proteins and N-signal-free proteins. Although the ratio of positive to negative examples in each dataset differs, the result of prediction by divergence features alone is higher than majority class classifier for all datasets (Table 9).

**Table 9:** Performance of N-signal vs N-signal-free protein binary classification on automatically collected orthologs

**Yeast dataset**	**Mean accuracy**	**Mean AUC**	**Mean MCC**
J48	71.47±5.00	0.67±0.07	0.36±0.12
SVM	**75.35**±3.49	**0.71**±0.04	**0.44**±0.08
The majority class fraction	65.23*%*	N/A	N/A
Human dataset			
J48	69.32±4.10	**0.72**±0.07	**0.43**±0.09
SVM	**72.28**±5.95	**0.72**±0.06	**0.43**±0.12
The majority class fraction	62.41*%*	N/A	N/A
Plant dataset			
J48	79.41±6.03	0.75±0.06	0.55±0.13
SVM	**83.47**±4.01	**0.79**±0.04	**0.64**±0.09
The majority class fraction	63.60*%*	N/A	N/A

Three classification performance measures when using only divergence features are shown for the discrimination of N-signal containing and N-signal-free proteins on automatically collected orthologs. AUC denotes the area under the ROC curves. For each measure the average and standard deviation is shown over the 5 folds of the cross-validation.

Next, we tested the predictive power of divergence in three-way classification on a dataset balanced to have equal class frequency (Table 10). It is evident that on balanced datasets, divergence also shows significant predictive power in distinguishing between the two different kinds of N-terminal signals, even for the relatively closely related mammal species.

**Table 10:** Performance for 3-way classification using SVM classifier on automatically collected orthologs

	***F***_***Div ***_**Yeast (73)**		***F***_***Div ***_**Human (81)**	
	**AUC**	**MCC**	**AUC**	**MCC**
MTS	0.65±0.09	0.31±0.18	0.66±0.05	0.31±0.11
SP	0.60±0.07	0.19±0.14	0.70±0.08	0.40±0.15
N-signal-free	0.66±0.08	0.35±0.15	0.69±0.06	0.39±0.11
*% accuracy*	51.63±7.21		57.61±4.71	

The 5-fold cross-validation performance of an SVM classifier using divergence features is shown for three-way classification on the automatically generated ortholog dataset for yeasts and mammals. The number of examples is given in parenthesis at top.

In plants, the divergence score can also discriminate between the three possible kinds of N-terminal signals better than random. However, there are only 15 experimentally validated SPs in this phylogenetic category (Table 2). Since this small sample size leads to a high statistical variance, we also computed the performance on balanced 3-way classification of MTS vs CTP vs N-signal-free (Table 11).

**Table 11:** Performance on balanced plant dataset using SVM classifier on automatically collected orthologs

	***F***_***Div ***_**Plant 4 classes (15)**		***F***_***Div ***_**Plant 3 classes (61)**	
	**AUC**	**MCC**	**AUC**	**MCC**
MTS	0.62±0.11	0.24±0.21	0.66±0.08	0.35±0.14
SP	0.78±0.11	0.58±0.23	N/A	N/A
CTP	0.73±0.16	0.43±0.31	0.77±0.12	0.51±0.23
N-signal-free	0.80±0.14	0.72±0.20	0.81±0.09	0.67±0.13
*% accuracy*	60.00±9.13		66.22±10.11	

The 5-fold cross-validation performance of an SVM classifier using divergence features is shown for three-way classification on balanced sets of (automatically generated) plant orthologs with or without the SP class. The number of examples is given in parenthesis at top.

In the Additional file 1 we list cross-validated performance estimates on various combinations of datasets and features. From these we draw two conclusions: in most cases divergence features slightly improve prediction when combined with standard features and in general computing standard features on the N-terminal 20 residues leads to higher accuracy than computing on 40 residues.

### Post-hoc analysis of proteins for which divergence strongly influences the prediction result

In this section we discuss proteins for which the use of divergence features strongly affects the results. The ortholog MSA’s of all proteins mentioned in this section are available in the Additional file 2.

#### Divergence features may help flag misannotation

Prior to this work, evolutionary divergence has not been applied systematically to N-terminal signal prediction. However we expected that it might be able to capture interesting examples not revealed by other features. To investigate this, we ranked instances whose SVM prediction changes drastically depending on whether or not divergence features are used. Because of its rich annotation, we focused on *S.cere.*, using the automatically defined ortholog set. The prediction result of 43 proteins changed depending on whether divergence features were added to conventional features. For these 43 proteins, we used the SVM numerical scores to rank the size of the effect as explained in the Additional file 1 (ranked list in Additional file 3: Table S1). The ortholog set multiple sequence alignments for these proteins are also available in the Additional file 2 in html form. In general, prediction differences are observed between the MTS and N-signal-free classes. The most highly affected protein is mitochondrial alanine tRNA ligase, ALA1 (P40825), which is predicted to have an MTS when sequence divergence features are used. Upon closer inspection we discovered that the sequence we used for this protein should in fact have been labeled as an MTS containing protein, but our dataset based on an earlier version of UniProtKB/Swiss-Prot contained mistaken annotation which holds for an alternative translation start site. Thus in this case sequence divergence yields the correct answer.

PTP1 (P25044) is another protein whose prediction changes from N-signal-free to MTS when divergence is considered. Following UniProtKB/Swiss-Prot, we treated it as a cytoplasmic protein, but there is no reference given for this annotation. Moreover PTP1 is identified as a mitochondrial protein by two large-scale experiments. This is suggestive that it may have a mitochondrial localization, although even in that case it would not necessarily have an MTS. Hopefully future work will clarify if this is another case in which divergence features flagged misannotations in our dataset.

#### Divergence features may help detect mitochondrial proteins with non-classical MTS signals

**Figure 6:**
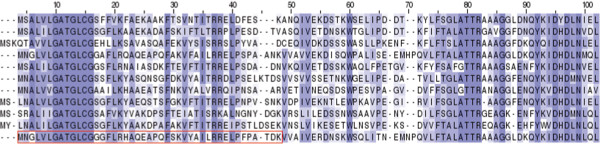
**MSA of FMP52 and its orthologs in 11 yeast species.** Multiple sequence alignment of FMP52 in *S.cerevisiae* and its orthologs in other 10 yeast species. The red boxed region shows annotated MTS of FMP52. The conserved positions are colored by Jalview.

FMP52 (P40008) is a protein included in our dataset for which the SVM with standard features predicts an MTS but the SVM with divergence features predicts N-signal-free. As shown in Figure 6, FMP52’s N-terminal region is not divergent like typical MTS’s, especially very near the N-terminus. FMP52 is indeed a mitochondrial protein, but upon closer scrutiny we discovered a previous report that it strongly associates with the outer membrane [59] — and therefore is unlikely to have a matrix targeting MTS. Moreover, FMP52 is one of the non-MTS containing proteins in the yeast proteomic analysis [32]. Swiss-Prot does annotate FMP52 with an MTS (1-44), but we could not find a reference or supporting information for this MTS annotation; therefore, we conclude that it is unlikely to have MTS. CYM1 (P32898) is another interesting example which has been reported to localize in the intermembrane space and not to be processed by mitochondrial proteases [60]. Since MTS is a cleavable targeting signal for the matrix, the intermembrane space localization and lack of proteolytic cleavage of CYM1 suggests its N-terminal signal is not a typical classical MTS.

MrpL19 (P53875) is another case in which sequence divergence features highlight a ribosomal mitochondrial protein which does not appear to have a classical MTS signal. According to both UniProtKB/Swiss-Prot annotation and a large-scale proteomics experiment [32] MrpL19 has an MTS, but the annotated “MTS” is unusually long and lacks an arginine in position -2, which is normally observed in MPP cleavage sites [9]. Moreover the N-terminal sequence of MrpL19 is very well conserved not only in yeasts but even in bacteria. Indeed the three dimensional structure of rplK, a homolog of MrpL19 in *E.coli*, has been solved and it is evident that the two proteins have a similar structured N-terminal. Taken together the evidence suggests that MrpL19 may not have an N-terminal mitochondrial localization signal, but rather be imported via an alternative pathway.

**Figure 7:**
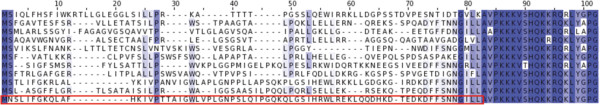
**MSA of MrpL32 and its orthologs in 11 yeast species.** Multiple sequence alignment of MrpL32 in *S.cerevisiae* and its orthologs in 10 other yeast species. The red boxed region shows MTS of MrpL32. The conserved positions are colored by Jalview.

On the other hand, we also observed ribosomal mitochondrial proteins whose N-terminal is poorly conserved. One example is MrpL32 (P25348), which cannot be predicted as having an MTS by standard tools such as TargetP [61] or Predotar [35], nor by our SVM’s trained without divergence features. MrpL32 shows a high divergence in its N-terminal region (Figure 7) and is predicted to have an MTS by our SVM when using divergence features. A literature search revealed that MrpL32 does indeed have an MTS, but it is unusual in the sense that it is cleaved by the protease m-AAA [62,63] instead of MPP. Mrp7 (P12687) is a similar case. Like MrpL32, Mrp7 is also a component of a large ribosomal subunit and is not predicted to have an MTS by TargetP, Predator, nor by our SVM without divergence features, but is predicted to have an MTS when divergence features are used. In UniProtKB/Swiss-Prot, Mrp7 is annotated as having an MTS, and indeed the processing of Mrp7 by MPP has been reported multiple times [32,64]. So in this case high sequence divergence allows an MTS to be correctly predicted.

Another case worth discussing is IMO32 (P53219), which has recently been reported to be processed by the intermediate protease Oct1 (after MPP) in the matrix [65]. It is unusual in that its inferred MPP cleavage site represents a rare exception to the almost invariant presence of arginine at the -2 position. IMO32 is predicted as an MTS by Predator [35] and our SVM when we use divergence, but not by our SVM without divergence features, nor by TargetP [61].

## Discussion

Although strong sequence similarity is a widely used indicator of co-localization, characteristically low sequence conservation in signal sequence regions has not been utilized for prediction. Other authors have noted the low sequence conservation of N-terminal sorting signals such as MTS sequences [66], but our work reported here is the first investigation of the utility of sequence divergence as a predictive feature for N-terminal sorting signals.

Our method requires defining an ortholog set for each gene. The YGOB curated dataset for 11 yeast species is a reliable way to obtain orthologs, but this kind of database is not available for most species. We show that a simple reciprocal best hit method identified orthologs with sufficient reliability for the purposes of computing sequence diversity. One avenue for future research is to relax the requirement of global alignment reciprocal best hit designed to find orthologs, and simply use for (possibly paralogous) homologous sequences. In this study we chose to focus on orthologs because paralogs often have distinct localization sites. For example, Rosso et al. [67] describe the interesting case of the human glutamate dehydrogenases GLUD1 and GLUD2. These paralogs result from a gene duplication event, but GLUD1 localizes to both the cytosol and the mitochondria while GLUD2 localizes exclusively to the mitochondria. Interestingly, the N-terminal region of GLUD2, which functions as an MTS, has evolved faster than GLUD1 [67].

Since we made a few somewhat arbitrary choices when defining divergence features, we performed an *post hoc* analysis to see if simply tuning those parameters would significantly affect the prediction accuracy. Namely, we investigated the effect of the changing the window length and position of the downstream normalizing window used to define NCdiff, but found that prediction accuracy is not strongly dependent on the exact value of these parameters (Additional file 1: Figures S1,S2). Another potential weakness of our method is the simple entropy based definition we used for sequence divergence, which ignores the phylogenetic relationship of the species involved. Many sophisticated measures have been proposed to quantify the degree of sequence conservation [42]. We did experiment with some of them, such as the Jensen-Shannon divergence [68] to try to improve prediction, but without success (results not shown). However we did not extensively explore the possibilities and believe that the simple entropy score employed here probably can be improved upon.

On the other hand, we did provide quantitative evidence that the entropy divergence score has considerable predictive power by itself. The examples ALA1 and FMP52 show that divergence can flag proteins (typically mitochondrial ones) with misannotated MTS information and give a hint regarding which compartment of the mitochondria they localize to. Examples like MrpL32, show that when the predictions of standard predictors are inconsistent with the degree of sequence divergence, non-typical MTS’s, processing proteases or alternative mitochondrial localization pathways may be indicated.

One weakness in our datasets is that many of our SP proteins are not experimentally validated, but rather annotated as SP proteins due to UniProtKB/Swiss-Prot annotation and prediction from amino acid sequence with SignalP [33] in the yeast dataset. This unfortunate circularity (predicting predictions) is unavoidable because: 1) only a handful of SP’s have been experimentally verified, and 2) the presence of SP’s cannot be reliably inferred exclusively from localization site for most *S.cere.* proteins. It may be reasonable to assume that secreted proteins all have SP’s, but *S.cere.* secretes very few proteins (the Swiss-Prot derived WoLF PSORT [69] dataset lists only six). Proteins which localize to the E.R. or Golgi body generally posses SPs, but many proteins annotated as E.R. or Golgi are non-SP containing peripheral membrane proteins, which localize to the periphery of these organelles. However, the risk of incorrect conclusion resulted from employing non-verified SP data is small. First, this problem only applies to the SP class, as recent proteomics data has provided direct measurement of many MTS’s [11,32]. Second, given the intense study of *S.cere.* and the continued scrutiny of UniProtKB/Swiss-Prot by the research community, we find it unlikely that a large fraction of the SP proteins in our dataset are incorrectly labeled. Third, our argument is not completely circular. SignalP prediction is based on physico-chemical features but not divergence (or conservation) for prediction, and the results shown in Figure 5 suggest physico-chemical features do not correlate very closely with sequence divergence.

## Conclusion

We find it rather remarkable that the accuracy of balanced 3-way prediction can be improved to more than 50% just by using simply defined sequence divergence features, while otherwise completely hiding the amino acid sequence of the protein. Although we readily admit the limited scope of this work, it is the first to quantitatively explore sequence divergence as a feature for localization signal prediction. We feel confident that our observation will stand the test of time, as more and more organisms are fully sequenced.

## Note

A preliminary version of this work appeared as a conference proceedings paper [70].

## Competing interests

The authors declare that they have no competing interests.

## Authors’ contributions

YF performed most of the study and wrote much of the manuscript. RL helped with initial attempts at automatic ortholog set determination. PH conceived of the study and wrote some of the manuscript. All authors contributed to discussion and have read and approved the final manuscript.

## Supplementary Material

Additional file 1 **Supplementary Text.** Contains the supplementary text with tables and figures. Click here for file

Additional file 2 **MSA’s of proteins for which sequence divergence changes predicted localization signals.** Contains links to ortholog multiple sequence alignments of each protein in Additional file 3: Table S1. Click here for file

Additional file 3 **List of proteins for which sequence divergence changes predicted localization signals.** A tab separated values file listing proteins and their prediction scores with and without the use of divergence features. Click here for file

## References

[B1] Eisenhaber F Bork P Wanted: subcellular localization of proteins based on sequence Trends Cell Biol 1998 15 169 170 10.1016/S0962-8924(98)01226-4 9695832

[B2] Kumar A Agarwal S Heyman JA Matson S Heidtman M Piccirillo S Umansky L Drawid A Jansen R Liu Y Cheung KH Miller P Gerstein M Roeder GS Snyder M Subcellular localization of the yeast proteome Genes Dev 2002 15 6 707 719 10.1101/gad.970902 11914276 PMC155358

[B3] Huh WK Falvo JV Gerke LG Carroll AS Howson RW Weissman JS O’Shea EK Global analysis of protein localization in budding yeast Nature 2003 15 6959 689 691 10.1038/nature02026 14562095

[B4] Imai K Nakai K Prediction of subcellular locations of proteins: where to proceed? Proteomics 2010 15 22 3970 3983 10.1002/pmic.201000274 21080490

[B5] Nair R Rost B Sequence conserved for subcellular localization Protein Sci 2002 15 12 2836 2847 12441382 10.1110/ps.0207402 PMC2373743

[B6] Blobel G Dobberstein B Transer of proteins across membranes. I. Presence of proteolytically processed and unprocessed nascent immunoglobulin light chains on membrane-bound ribosomes of murine myeloma J Cell Biol 1975 15 3 835 851 10.1083/jcb.67.3.835 811671 PMC2111658

[B7] Schatz G Dobberstein B Common principles of protein translation across membranes Science 1996 15 5255 1519 1526 10.1126/science.271.5255.1519 8599107

[B8] von Heijne G Patterns of amino acids near signal-sequence cleavage sites Eur J Biochem 1983 15 17 21 10.1111/j.1432-1033.1983.tb07424.x 6852022

[B9] Gakh O Cavadini P Isaya G Mitochondrial processing peptidases Biochim Biophys Acta 2002 15 63 77 10.1016/S0167-4889(02)00265-3 12191769

[B10] Teixeira PF Glaser E Processing peptidases in mitochondria and chloroplasts Biochim Biophys Acta 2013 15 2 360 370 10.1016/j.bbamcr.2012.03.012 22495024

[B11] Huang S Taylor NL Whelan J Millar AH Refining the definition of plant mitochondrial presequences through analysis of sorting signals, N-terminal modifications, and cleavage motifs Plant Physiol 2009 15 3 1272 1285 10.1104/pp.109.137885 19474214 PMC2705053

[B12] Saitoh T Igura M Obita T Ose T Kojima R Maenaka K Endo T Kohda D Tom20 recognizes mitochondrial presequences through dynamic equilibrium among multiple bound states EMBO J 2007 15 22 4777 4787 10.1038/sj.emboj.7601888 17948058 PMC2080804

[B13] Yamamoto H Itoh N Kawano S Yatsukawa Y Momose T Makio T Matsunaga M Yokota M Esaki M Shodai T Kohda D Hobbs AE Jensen RE Endo T Dual role of the receptor Tom20 in specificity and efficiency of protein import into mitochondria Proc Natl Acad Sci U S A 2011 15 91 96 10.1073/pnas.1014918108 21173275 PMC3017135

[B14] Horton P Mukai Y Nakai K Wong L Protein localization prediction The Practical Bioinformatician 2004 5 Toh Tuck Link. Singapore 596224: World Scientific 193 215

[B15] Nakashima H Nishikawa K Discrimination of intracellular and extracellular proteins using amino acid composition and residue-pair frequences JMB 1994 15 54 61 10.1006/jmbi.1994.1267 8145256

[B16] Yuan Z Prediction of protein subcellular locations using Markov chain models FEBS Lett 1999 15 23 26 10.1016/S0014-5793(99)00506-2 10356977

[B17] Cedano J Pérez-Ponsa JA Querol E Relation between amino acid composition and cellular location of proteins JMB 1997 15 3 594 600 10.1006/jmbi.1996.0804 9067612

[B18] Reinhardt A Hubbard T Using neural networks for prediction of the subcellular location of proteins Nucleic Acids Res 1998 15 9 2230 2236 10.1093/nar/26.9.2230 9547285 PMC147531

[B19] Park KJ Kanehisa M Prediction of protein subcellular locations by support vector machines using compositions of amino acids and amino acid pairs Bioinformatics 2003 15 13 1656 1663 10.1093/bioinformatics/btg222 12967962

[B20] Sakiyama N Runcong K Sawada R Sonoyama M Mitaku S Nuclear localization of proteins with a charge periodicity of 28 residues Chem-BioInformatics J 2007 15 35 48

[B21] Drawid A Gerstein M A Bayesian system integrating expression data with sequence patterns for localizing proteins: comprehensive application to the yeast genome JMB 2000 15 4 1059 1075 10.1006/jmbi.2000.3968 10966805

[B22] Frank K Sippl MJ High-performance signal peptide prediction based on sequence alignment techniques Bioinformatics 2008 15 19 2172 2176 10.1093/bioinformatics/btn422 18697773

[B23] Andrade MA O’Donoghue SI Rost B Adaptation of protein surfaces to subcellular location J Mol Biol 1998 15 1998 517 525 9512720 10.1006/jmbi.1997.1498

[B24] McCue LA Thompson W Carmack CS Ryan MP Liu JS Derbyshire V Lawrence CE Phylogenetic footprinting of transcription factor binding sites in proteobacterial genomes Nucleic Acids Res 2001 15 3 774 782 10.1093/nar/29.3.774 11160901 PMC30389

[B25] Davey NE Shields DC Edwards RJ Masking residues using context-specific evolutionary conservation significantly improves short linear motif discovery Bioinformatics 2009 15 4 443 450 10.1093/bioinformatics/btn664 19136552

[B26] Martinsen L Johnsen A Venanzetti F Bachmann L Phylogenetic footprinting of non-coding RNA: hammerhead ribozyme sequences in a satellite DNA family of Dolichopoda cave crickets (Orthoptera, Rhaphidophoridae) BMC Evol Biol 2010 15 3 10.1186/1471-2148-10-3 20047671 PMC2837043

[B27] Nair R Rost B Better prediction of sub-cellular localization by combining evolutionary and structural information PROTEINS 2003 15 4 917 930 10.1002/prot.10507 14635133

[B28] Yogev O Pines O Dual targeting of mitochondrial proteins: mechanism, regulation and function Biochim Biophys Acta 2011 15 3 1012 1020 10.1016/j.bbamem.2010.07.004 20637721

[B29] Christopher C Small I A reevaluation of dual-targeting of proteins to mitochondria and chloroplasts Biochim Biophys Acta 2013 15 2 253 259 10.1016/j.bbamcr.2012.05.029 22683762

[B30] Tsukamoto T Hata S Yokota S Miura S Fujiki Y Hijikata M Miyazawa S Hashimoto T Osumi T Characterization of the signal peptide at the amino terminus of the rat peroxisomal 3-ketoacyl-CoA thiolase precursor J Biol Chem 1994 15 8 6001 6010 8119946

[B31] Boutet E Lieberherr D Tognolli M Schneider M Bairoch A UniProtKB/Swiss-Prot Methods Mol Biol 2007 15 89 112 18287689 10.1007/978-1-59745-535-0_4

[B32] Vögtle F Wortelkamp S Zahedi R Becker D Leidhold C Gevaert K Kellermann J Voos W Sickmann A Pfanner N Meisinger C Global analysis of the mitochondrial N-proteome identifies a processing peptidase critical for protein stability Cell 2009 15 2 428 439 10.1016/j.cell.2009.07.045 19837041

[B33] Bendtsen J Nielsen H von Heijne G Brunak S Improved prediction of signal peptides: SignalP 3.0 J Mol Biol 2004 15 4 783 795 10.1016/j.jmb.2004.05.028 15223320

[B34] Dondoshansky I Blastclust (NCBI Software Development Toolkit) 2002

[B35] Small I Peeters N Legeai F Lurin C Predator: a tool for rapidly screening proteomes for N-terminal targeting sequences Proteomics 2004 15 6 1581 1590 10.1002/pmic.200300776 15174128

[B36] Byrne KP Wolfe KH The yeast gene order browser: combining curated homology and syntenic context reveals gene fate in polyploid species Genome Res 2005 15 10 1456 1461 10.1101/gr.3672305 16169922 PMC1240090

[B37] Altenhoff AM Dessimoz C Anisimova M Inferring orthology and paralogy Evolutionary Genomics: Statistics and Computational Methods. Methods in Molecular Biology 2012 USA: Humana Press 259 277 10.1007/978-1-61779-582-4_9 22407712

[B38] Overbeek R Fonstein M D’Souza M Pusch GD Maltsev N The use of gene clusters to infer functional coupling Proc Natl Acad Sci U S A 1999 15 6 2896 2901 10.1073/pnas.96.6.2896 10077608 PMC15866

[B39] Edgar RC Search and clustering orders of magnitude faster than BLAST Bioinformatics 2010 15 19 2460 2461 [USEARCH] 10.1093/bioinformatics/btq461 20709691

[B40] Katoh K Misawa K Kuma K Miyata T MAFFT: a novel method for rapid multiple sequence alignment based on fast Fourier transform Nucleic Acids Res 2002 15 14 3059 3066 10.1093/nar/gkf436 12136088 PMC135756

[B41] Mayrose I Graur D Ben-Tal N Pupko T Comparison of site-specific rate-inference methods for protein sequences: empirical Bayesian methods are superior Mol Biol Evol 2004 15 9 1781 1791 10.1093/molbev/msh194 15201400

[B42] Johansson F Toh H A comparative study of conservation and variation scores BMC Bioinformatics 2010 15 388 10.1186/1471-2105-11-388 20663120 PMC2920274

[B43] Kyte J Doolittle RF A simple method for displaying the hydropathic character of a protein J Mol Biol 1982 15 105 132 10.1016/0022-2836(82)90515-0 7108955

[B44] Quinlan JR Induction of decision trees Mach Learn 1986 15 81 106

[B45] Quinlan JR C4.5: Programs for Machine Learning 1993 San Francisco: Morgan Kaufmann Publishers Inc.

[B46] Hall M Frank E Holmes G Pfahringer B Reutemann P Witten IH The WEKA data mining software: an update ACM SIGKDD Explorations Newsl 2009 15 10 10.1145/1656274.1656278

[B47] Vapnik VN The Nature of Statistical Learning Theory 1995 New York: Springer-Verlag New York, Inc.

[B48] Chang CC Lin CJ LIBSVM: A library for support vector machines ACM Trans Intell Syst Technol 2011 15 3 1 27

[B49] Hsu C Lin C A comparison of methods for multiclass support vector machines Neural Netw, IEEE Trans 2002 15 2 415 425 10.1109/72.991427 18244442

[B50] Allwein EL Schapire RE Singer Y Reducing multiclass to binary: a unifying approach for margin classifiers J Mach Learn Res 2001 15 113 141

[B51] Fayyad UM Irani KB Multi-interval discretization of continuous-valued attributes for classification learning International Joint Conference on Artificial Intelligence 1993 1022 1027

[B52] He H Garcia EA Learning from imbalanced data IEEE Trans Knowl Data Eng 2009 15 9 1263 1284 [http://portal.acm.org/citation.cfm?id=1591901.1592322]

[B53] Matthews BW Comparison of the predicted and observed secondary structure of T4 phage lysozyme Biochim Biophys Acta 1975 15 2 442 451 10.1016/0005-2795(75)90109-9 1180967

[B54] Baldi P Brunak S Chauvin Y Andersen CA Nielsen H Assessing the accuracy of prediction algorithms for classification: an overview Bioinformatics 2000 15 5 412 424 10.1093/bioinformatics/16.5.412 10871264

[B55] Fawcett T An introduction to ROC analysis Pattern Recognit Lett 2006 15 8 861 874 10.1016/j.patrec.2005.10.010

[B56] Argarwal S Graepel T Harbrich R Har-Peled S Roth D Generalization bounds for the area under the ROC curve J Mach Learn Res 2005 15 393 425

[B57] Williams EJ Pal C Hurst LD The molecular evolution of signal peptides Gene 2000 15 2 313 322 10940569 10.1016/s0378-1119(00)00233-x

[B58] Dujon B Yeasts illustrate the molecular mechanisms of eukaryotic genome evolution Trends Genet 2006 15 7 357 387 10.1016/j.tig.2006.05.002 16730849

[B59] Zahedi RP Sickmann A Boehm AM Winkler C Zufall N Schönfisch B Guiard B Pfanner N Meisinger C Proteomic analysis of the yeast mitochondrial outer membrane reveals accumulation of a subclass of preproteins Mol Biol Cell 2006 15 3 1436 1450 16407407 10.1091/mbc.E05-08-0740 PMC1382330

[B60] Kambacheld M Augustin S Tatsuta T Muller S Langer T Role of the novel metallopeptidase Mop112 and saccharolysin for the complete degradation of proteins residing in different subcompartments of mitochondria J Biol Chem 2005 15 20 20132 20139 10.1074/jbc.M500398200 15772085

[B61] Emanuelsson O Brunak S von Heijne G Nielsen H Locating proteins in the cell using TargetP, SignalP and related tools Nat Protoc 2007 15 4 953 971 10.1038/nprot.2007.131 17446895

[B62] Nolden M Ehses S Koppen M Bernacchia A Rugarli EI Langer T The m-AAA protease defective in hereditary spastic paraplegia controls ribosome assembly in mitochondria Cell 2005 15 2 277 289 10.1016/j.cell.2005.08.003 16239145

[B63] Bonn F Tatsua T Petrungaro C Riemer J Langer T Presequence-dependent folding ensures MrpL32 processing by the m-AAA protease in mitochondria EMBO J 2011 15 13 2545 2556 10.1038/emboj.2011.169 21610694 PMC3155303

[B64] Grohmann L Graack HR Kruft V Choli T Goldschmidt-Reisin S Kitakawa M Extended N-terminal sequencing of proteins of the large ribosomal subunit from yeast mitochondria FEBS Lett 1991 15 51 56 10.1016/0014-5793(91)80759-V 2060626

[B65] Vögtle FN Prinz C Kellermann J Lottspeich F Pfanner N Meisinger C Mitochondrial protein turnover: role of the precursor intermediate peptidase Oct1 in protein stabilization Mol Biol Cell 2011 15 13 2135 2143 10.1091/mbc.E11-02-0169 21525245 PMC3128517

[B66] Doyle SR Kasinadhuni NR Chan CK Grant WN Evidence of evolutionary constraints that influences the sequence composition and diversity of mitochondrial matrix targeting signals PLoS ONE 2013 15 6 e67938 10.1371/journal.pone.0067938 23825690 PMC3692466

[B67] Rosso L Marques AC Reichert AS Kaessmann H Mitochondrial targeting adaptation of the hominoid-specific glutamate dehydrogenase driven by positive Darwinian selection PLoS Genetics 2008 15 8 e1000150 10.1371/journal.pgen.1000150 18688271 PMC2478720

[B68] Capra JA Singh M Predicting functionally important residues from sequence conservation Bioinformatics 2007 15 15 1875 1882 10.1093/bioinformatics/btm270 17519246

[B69] Horton P Park KJ Obayashi T Fujita N Harada H Adams-Collier C Nakai K WoLF PSORT: protein localization predictor Nucleic Acids Res 2007 15 Web Server issue W585 W587 17517783 10.1093/nar/gkm259 PMC1933216

[B70] Fukasawa Y Leung RK Tsui SK Horton P Evolutionary sequence divergence predicts protein sub-cellular localization signals Proceedings 5th IEEE International Conference on Systems Biology 2011 IEEE Publishing 307 312

