# Supplementary material for: Plus ça change – evolutionary sequence divergence predicts protein subcellular localization signals
Source: BMC Genomics. 2014 Jan 20;15:46. doi: 10.1186/1471-2164-15-46 (PMC3906766; doi:10.1186/1471-2164-15-46)
Supplement: Additional file 1 — Supplementary Text. Contains the supplementary text with tables and figures. [file 1471-2164-15-46-S1.pdf]

Supplementary material for: Plus ça change – evolutionary sequence divergence predicts protein sub-cellular localization signals

Yoshinori Fukasawa[1,2], Ross KK Leung[3], Stephen KW Tsui[3] and Paul Horton*[1,4]

[1]Department of Computational Biology, Graduate School of Frontier Sciences, University of Tokyo, Kashiwa, Japan
[2]Japan Society for the Promotion of Science, Tokyo Chiyoda
[3]Hong Kong Bioinformatics Centre and School of Biomedical Sciences, Chinese University of Hong Kong, Shatin, China
[4]Computational Biology Research Center, Advanced Industrial Science and Technology Tokyo, Japan

Email: Paul Horton*- horton-p@aist.go.jp;

*Corresponding author

Measure of influence of divergence features:

As reported in the results section, we performed a post-hoc analysis of proteins for which the divergence features greatly influenced the prediction outcome. This requires a concrete, quantitative measure of that influence, which we chose to define in terms of a numerical score known as exponential loss-based decoding [1].

For each protein, and each of two feature sets (with and without divergence features), we compute a probability vector P estimating the probability that the protein is a member of each of the three sorting classes {SP, MTS, N-signal-free}. We then use the Jensen-Shannon divergence as a quantitative measure of how much the two probability vectors (predictions with and without divergence features) differ.

The Jensen-Shannon divergence is a standard measure of distance between two probability distributions. In our notation, the definition is:

$$JSD(P_{\text{div}}||P_{\text{nodiv}}) \;=\; \frac{1}{2}D(P_{\text{div}}||M) + \frac{1}{2}D(P_{\text{nodiv}}||M)$$

where $M = \frac{1}{2}(P_{\text{div}} + P_{\text{nodiv}})$ and $D(P||Q)$ indicates the Kullback-Leibler divergence:

$$D_{\text{KL}}(P||Q) \;=\; \sum_i P(i)\log_2 \frac{P(i)}{Q(i)}$$

The precise method we used to compute P, the probability vector over classes for a given protein, is somewhat involved. We first used all of the yeast YGOB data to train three binary SVM classifiers {0:1, 0:2, 1:2}, where the integers {0,1,2} to denote the three classes {SP, MTS, N-signal-free}. For each protein instance, each SVM classifier outputs a score related to the classification margin, which roughly reflects the confidence of its prediction. Let s_{ij} denote the score for the SVM discriminating between classes i and j, so for example s_{12} denotes the score of the SVM discriminating between MTS and N-signal-free, with a large positive value indicating a strong prediction of MTS, and a large negative value a strong prediction of N-signal-free. Following the exponential loss function described in [1], we define P as:

$$P \;\propto\; e^{s_{01}} + e^{-s_{01}} \;+\; e^{s_{02}} + e^{-s_{02}} \;+\; e^{s_{12}} + e^{-s_{12}}$$

We compute P with this equation and then linearly normalize so that its elements sum to one.

1 Divergence score combined with standard features in N-terminal 40 residues

1.1 S. cerevisiae, curated orthologs (N_{40})

	F_{Div}		F_{Phy}		F_{Comp}	
	AUC	MCC	AUC	MCC	AUC	MCC
MTS	0.67 ± 0.03	0.36 ± 0.06	0.86 ± 0.02	0.72 ± 0.04	0.86 ± 0.05	0.73 ± 0.07
SP	0.50 ± 0.00	0.00 ± 0.00	0.77 ± 0.02	0.62 ± 0.07	0.78 ± 0.11	0.62 ± 0.21
N-signal-free	0.66 ± 0.02	0.36 ± 0.03	0.84 ± 0.02	0.69 ± 0.03	0.85 ± 0.05	0.71 ± 0.07
% accuracy	70.82 ± 1.61		85.19 ± 1.36		85.77 ± 3.15	

	$F_{CompFull}$		F_{Div} & F_{Phy}		F_{Div} & F_{Comp}	
	AUC	MCC	AUC	MCC	AUC	MCC
MTS	0.67 ± 0.02	0.42 ± 0.06	0.84 ± 0.03	0.71 ± 0.05	0.86 ± 0.04	0.77 ± 0.07
SP	0.67 ± 0.11	0.50 ± 0.22	0.90 ± 0.05	0.82 ± 0.07	0.88 ± 0.04	0.79 ± 0.08
N-signal-free	0.67 ± 0.02	0.42 ± 0.04	0.86 ± 0.02	0.73 ± 0.03	$\mathbf{0.87} \pm 0.03$	$\mathbf{0.77} \pm 0.05$
% accuracy	74.78 ± 1.78		87.39 ± 0.95		89.15 ± 1.93	

	F_{Div} & $F_{CompFull}$		F_{Phy} & F_{Comp}		F_{Phy} & $F_{CompFull}$	
	AUC	MCC	AUC	MCC	AUC	MCC
MTS	0.80 ± 0.03	0.65 ± 0.06	$\mathbf{0.87} \pm 0.05$	0.75 ± 0.08	0.84 ± 0.03	0.69 ± 0.06
SP	0.78 ± 0.07	0.66 ± 0.11	0.79 ± 0.12	0.64 ± 0.22	0.82 ± 0.05	0.72 ± 0.10
N-signal-free	0.79 ± 0.02	0.63 ± 0.04	0.85 ± 0.04	0.72 ± 0.07	0.83 ± 0.03	0.68 ± 0.06
% accuracy	82.99 ± 1.66		86.50 ± 3.20		85.04 ± 2.73	

	F_{Comp} & $F_{CompFull}$		F_{Div} & F_{Phy} & F_{Comp}		F_{Div} & F_{Phy} & $F_{CompFull}$	
	AUC	MCC	AUC	MCC	AUC	MCC
MTS	$\mathbf{0.87} \pm 0.03$	$\mathbf{0.78} \pm 0.04$	0.86 ± 0.04	0.76 ± 0.07	0.86 ± 0.01	0.74 ± 0.02
SP	0.80 ± 0.08	0.70 ± 0.09	0.90 ± 0.07	0.79 ± 0.08	0.89 ± 0.06	0.82 ± 0.06
N-signal-free	0.85 ± 0.03	0.74 ± 0.04	$\mathbf{0.87} \pm 0.02$	$\mathbf{0.77} \pm 0.04$	$\mathbf{0.87} \pm 0.02$	0.75 ± 0.03
% accuracy	87.97 ± 1.25		89.15 ± 1.91		88.27 ± 1.29	

	F_{Phy} & F_{Comp} & $F_{CompFull}$		F_{Div} & F_{Comp} & $F_{CompFull}$		ALL	
	AUC	MCC	AUC	MCC	AUC	MCC
MTS	$\mathbf{0.87} \pm 0.03$	0.76 ± 0.05	$\mathbf{0.87} \pm 0.03$	0.77 ± 0.03	$\mathbf{0.87} \pm 0.03$	0.77 ± 0.03
SP	0.81 ± 0.08	0.70 ± 0.11	$\mathbf{0.91} \pm 0.06$	$\mathbf{0.85} \pm 0.06$	0.90 ± 0.06	0.83 ± 0.08
N-signal-free	0.85 ± 0.03	0.72 ± 0.05	$\mathbf{0.87} \pm 0.02$	$\mathbf{0.77} \pm 0.03$	$\mathbf{0.87} \pm 0.02$	$\mathbf{0.77} \pm 0.02$
% accuracy	87.24 ± 1.86		$\mathbf{89.44} \pm 1.12$		89.30 ± 0.66	

Table S2: The 5-fold cross-validation performance of an SVM classifier, using various feature set combinations as listed above each column, is shown for three-way classification on the yeast curated ortholog dataset.

1.2 S. cerevisiae, RBH orthologs (N_{40})

	F_{Div}		F_{Phy}		F_{Comp}	
	AUC	MCC	AUC	MCC	AUC	MCC
MTS	0.65 ± 0.04	0.34 ± 0.08	0.85 ± 0.03	0.72 ± 0.08	0.87 ± 0.03	0.75 ± 0.05
SP	0.50 ± 0.00	0.00 ± 0.00	0.81 ± 0.04	0.66 ± 0.06	0.85 ± 0.04	0.75 ± 0.04
N-signal-free	0.64 ± 0.04	0.33 ± 0.10	0.85 ± 0.02	0.71 ± 0.05	0.87 ± 0.02	0.75 ± 0.04
% accuracy	70.06 ± 3.05		85.44 ± 2.83		87.79 ± 1.83	

	$F_{CompFull}$		F_{Div} & F_{Phy}		F_{Div} & F_{Comp}	
	AUC	MCC	AUC	MCC	AUC	MCC
MTS	0.66 ± 0.03	0.41 ± 0.07	0.85 ± 0.04	0.74 ± 0.08	0.87 ± 0.03	0.77 ± 0.06
SP	0.74 ± 0.08	0.65 ± 0.13	0.85 ± 0.05	0.76 ± 0.09	0.88 ± 0.03	0.80 ± 0.06
N-signal-free	0.68 ± 0.01	0.45 ± 0.03	0.87 ± 0.02	0.77 ± 0.05	0.88 ± 0.03	0.79 ± 0.06
% accuracy	75.94 ± 1.12		88.14 ± 2.03		89.43 ± 2.47	

	F_{Div} & $F_{CompFull}$		F_{Phy} & F_{Comp}		F_{Phy} & $F_{CompFull}$	
	AUC	MCC	AUC	MCC	AUC	MCC
MTS	0.78 ± 0.04	0.61 ± 0.06	0.87 ± 0.03	0.76 ± 0.07	0.86 ± 0.02	0.72 ± 0.05
SP	0.82 ± 0.08	0.75 ± 0.14	0.86 ± 0.03	0.75 ± 0.02	0.87 ± 0.05	0.79 ± 0.06
N-signal-free	0.80 ± 0.04	0.65 ± 0.07	0.87 ± 0.03	0.76 ± 0.06	0.85 ± 0.03	0.71 ± 0.07
% accuracy	83.45 ± 3.23		88.15 ± 2.64		86.62 ± 2.85	

	F_{Comp} & $F_{CompFull}$		F_{Div} & F_{Phy} & F_{Comp}		F_{Div} & F_{Phy} & $F_{CompFull}$	
	AUC	MCC	AUC	MCC	AUC	MCC
MTS	0.87 ± 0.05	0.77 ± 0.09	0.87 ± 0.03	0.76 ± 0.06	0.86 ± 0.05	0.75 ± 0.08
SP	0.90 ± 0.04	0.85 ± 0.06	0.88 ± 0.04	0.78 ± 0.07	0.91 ± 0.03	0.87 ± 0.05
N-signal-free	0.87 ± 0.05	0.77 ± 0.09	0.89 ± 0.03	0.79 ± 0.06	0.88 ± 0.04	0.79 ± 0.06
% accuracy	89.44 ± 3.81		89.20 ± 2.52		89.67 ± 2.72	

	F_{Phy} & F_{Comp} & $F_{CompFull}$		F_{Div} & F_{Comp} & $F_{CompFull}$		ALL	
	AUC	MCC	AUC	MCC	AUC	MCC
MTS	0.87 ± 0.04	0.76 ± 0.07	0.88 ± 0.02	$\mathbf{0.80} \pm 0.04$	$\mathbf{0.89} \pm 0.03$	$\mathbf{0.80} \pm 0.05$
SP	0.89 ± 0.03	0.84 ± 0.07	$\mathbf{0.93} \pm 0.02$	$\mathbf{0.90} \pm 0.02$	$\mathbf{0.93} \pm 0.02$	0.89 ± 0.06
N-signal-free	0.87 ± 0.04	0.76 ± 0.09	$\mathbf{0.90} \pm 0.02$	0.82 ± 0.04	$\mathbf{0.90} \pm 0.02$	$\mathbf{0.83} \pm 0.04$
% accuracy	89.08 ± 3.48		91.31 ± 1.63		$\mathbf{91.67} \pm 1.63$	

Table S3: The 5-fold cross-validation performance of an SVM classifier, using various feature set combinations as listed above each column, is shown for three-way classification on the yeast automatically collected dataset.

1.3 Human, RBH orthologs (N_{40})

	F_{Div}		F_{Phy}		F_{Comp}	
	AUC	MCC	AUC	MCC	AUC	MCC
MTS	0.52 ± 0.03	0.10 ± 0.18	0.79 ± 0.06	0.61 ± 0.16	0.84 ± 0.02	0.69 ± 0.06
SP	0.65 ± 0.05	0.29 ± 0.10	0.82 ± 0.04	0.65 ± 0.07	0.86 ± 0.03	0.74 ± 0.06
N-signal-free	0.66 ± 0.05	0.35 ± 0.09	0.87 ± 0.03	0.74 ± 0.06	0.89 ± 0.03	0.80 ± 0.07
% accuracy	65.11 ± 3.55		83.01 ± 4.17		87.07 ± 3.25	

	$F_{CompFull}$		F_{Div} & F_{Phy}		F_{Div} & F_{Comp}	
	AUC	MCC	AUC	MCC	AUC	MCC
MTS	0.67 ± 0.07	0.43 ± 0.15	0.81 ± 0.03	0.65 ± 0.07	0.82 ± 0.05	0.69 ± 0.08
SP	0.75 ± 0.06	0.52 ± 0.12	0.87 ± 0.03	0.75 ± 0.07	0.89 ± 0.03	0.79 ± 0.07
N-signal-free	0.75 ± 0.06	0.52 ± 0.12	0.88 ± 0.02	0.78 ± 0.06	$\mathbf{0.91} \pm 0.03$	0.82 ± 0.07
% accuracy	74.89 ± 5.49		86.32 ± 3.25		88.42 ± 3.39	

	F_{Div} & $F_{CompFull}$		F_{Phy} & F_{Comp}		F_{Phy} & $F_{CompFull}$	
	AUC	MCC	AUC	MCC	AUC	MCC
MTS	0.73 ± 0.07	0.53 ± 0.11	0.84 ± 0.02	0.69 ± 0.06	0.84 ± 0.05	0.69 ± 0.09
SP	0.81 ± 0.05	0.62 ± 0.06	0.87 ± 0.04	0.75 ± 0.07	0.86 ± 0.05	0.73 ± 0.08
N-signal-free	0.81 ± 0.04	0.64 ± 0.05	0.90 ± 0.04	0.80 ± 0.08	0.89 ± 0.04	0.79 ± 0.09
% accuracy	79.85 ± 1.35		87.22 ± 3.80		86.77 ± 4.47	

	F_{Comp} & $F_{CompFull}$		F_{Div} & F_{Phy} & F_{Comp}		F_{Div} & F_{Phy} & $F_{CompFull}$	
	AUC	MCC	AUC	MCC	AUC	MCC
MTS	0.85 ± 0.02	0.72 ± 0.07	0.83 ± 0.05	0.70 ± 0.08	0.84 ± 0.04	0.69 ± 0.09
SP	0.86 ± 0.04	0.73 ± 0.08	0.89 ± 0.03	0.79 ± 0.07	0.88 ± 0.05	0.77 ± 0.07
N-signal-free	0.88 ± 0.04	0.78 ± 0.08	$\mathbf{0.91} \pm 0.03$	$\mathbf{0.83} \pm 0.08$	0.90 ± 0.03	0.81 ± 0.07
% accuracy	86.92 ± 3.86		88.87 ± 3.62		87.67 ± 3.59	

	F_{Phy} & F_{Comp} & $F_{CompFull}$		F_{Div} & F_{Comp} & $F_{CompFull}$		ALL	
	AUC	MCC	AUC	MCC	AUC	MCC
MTS	$\mathbf{0.87} \pm 0.02$	$\mathbf{0.76} \pm 0.07$	0.84 ± 0.05	0.72 ± 0.06	0.86 ± 0.04	0.74 ± 0.05
SP	0.87 ± 0.03	0.75 ± 0.06	$\mathbf{0.90} \pm 0.04$	$\mathbf{0.80} \pm 0.06$	0.89 ± 0.03	0.79 ± 0.06
N-signal-free	0.90 ± 0.04	0.80 ± 0.09	$\mathbf{0.91} \pm 0.04$	$\mathbf{0.83} \pm 0.08$	$\mathbf{0.91} \pm 0.04$	$\mathbf{0.83} \pm 0.08$
% accuracy	88.12 ± 3.58		89.17 ± 3.51		$\mathbf{89.32} \pm 3.25$	

Table S4: The 5-fold cross-validation performance of an SVM classifier, using various feature set combinations as listed above each column, is shown for three-way classification on the mammal automatically collected dataset.

1.4 Plant model organisms, RBH orthologs (N_{40})

	F_{Div}		F_{Phy}		F_{Comp}	
	AUC	MCC	AUC	MCC	AUC	MCC
MTS	0.61 ± 0.05	0.30 ± 0.15	0.70 ± 0.11	0.40 ± 0.20	$\mathbf{0.81} \pm 0.06$	0.61 ± 0.09
SP	0.50 ± 0.00	0.00 ± 0.00	0.56 ± 0.08	0.12 ± 0.18	0.69 ± 0.19	0.44 ± 0.44
CTP	0.78 ± 0.08	0.54 ± 0.15	0.78 ± 0.05	0.55 ± 0.09	0.85 ± 0.05	0.68 ± 0.10
N-signal-free	0.80 ± 0.05	0.60 ± 0.09	0.85 ± 0.03	0.70 ± 0.05	0.86 ± 0.04	0.73 ± 0.05
% accuracy	65.05 ± 6.00		69.10 ± 4.46		76.81 ± 3.53	

	$F_{CompFull}$		F_{Div} & F_{Phy}		F_{Div} & F_{Comp}	
	AUC	MCC	AUC	MCC	AUC	MCC
MTS	0.58 ± 0.06	0.19 ± 0.15	0.65 ± 0.10	0.32 ± 0.23	0.79 ± 0.08	0.58 ± 0.14
SP	0.69 ± 0.14	0.48 ± 0.29	0.66 ± 0.17	0.36 ± 0.36	0.76 ± 0.25	0.50 ± 0.47
CTP	0.68 ± 0.08	0.35 ± 0.15	0.80 ± 0.05	0.58 ± 0.10	0.84 ± 0.04	0.67 ± 0.09
N-signal-free	0.66 ± 0.05	0.31 ± 0.08	$\mathbf{0.90} \pm 0.02$	$\mathbf{0.82} \pm 0.04$	0.88 ± 0.02	0.79 ± 0.03
% accuracy	53.33 ± 7.70		72.40 ± 4.28		77.93 ± 2.41	

	F_{Div} & $F_{CompFull}$		F_{Phy} & F_{Comp}		F_{Phy} & $F_{CompFull}$	
	AUC	MCC	AUC	MCC	AUC	MCC
MTS	0.63 ± 0.04	0.32 ± 0.11	0.80 ± 0.08	0.59 ± 0.12	0.68 ± 0.11	0.39 ± 0.23
SP	0.70 ± 0.22	0.48 ± 0.46	0.69 ± 0.19	0.41 ± 0.41	0.83 ± 0.20	0.63 ± 0.36
CTP	0.78 ± 0.05	0.53 ± 0.11	0.83 ± 0.04	0.65 ± 0.08	0.75 ± 0.07	0.48 ± 0.14
N-signal-free	0.82 ± 0.06	0.63 ± 0.10	0.87 ± 0.04	0.76 ± 0.06	0.85 ± 0.01	0.72 ± 0.02
% accuracy	67.30 ± 4.19		76.08 ± 3.11		69.51 ± 6.82	

	F_{Comp} & $F_{CompFull}$		F_{Div} & F_{Phy} & F_{Comp}		F_{Div} & F_{Phy} & $F_{CompFull}$	
	AUC	MCC	AUC	MCC	AUC	MCC
MTS	0.77 ± 0.09	0.53 ± 0.15	0.79 ± 0.08	0.57 ± 0.10	0.69 ± 0.04	0.42 ± 0.07
SP	$\mathbf{0.86} \pm 0.21$	$\mathbf{0.68} \pm 0.38$	0.76 ± 0.26	0.52 ± 0.51	0.76 ± 0.25	0.53 ± 0.49
CTP	0.83 ± 0.05	0.66 ± 0.09	0.85 ± 0.05	0.68 ± 0.09	0.77 ± 0.06	0.53 ± 0.11
N-signal-free	0.86 ± 0.02	0.73 ± 0.05	0.89 ± 0.02	0.79 ± 0.04	0.89 ± 0.05	0.79 ± 0.10
% accuracy	76.09 ± 5.59		78.29 ± 3.16		72.76 ± 4.43	

	F_{Phy} & F_{Comp} & $F_{CompFull}$		F_{Div} & F_{Comp} & $F_{CompFull}$		ALL	
	AUC	MCC	AUC	MCC	AUC	MCC
MTS	0.76 ± 0.08	0.52 ± 0.13	0.79 ± 0.07	0.62 ± 0.12	0.80 ± 0.06	$\mathbf{0.63} \pm 0.09$
SP	$\mathbf{0.86} \pm 0.21$	$\mathbf{0.68} \pm 0.38$	0.80 ± 0.22	0.61 ± 0.41	0.83 ± 0.24	0.65 ± 0.44
CTP	0.83 ± 0.05	0.65 ± 0.09	$\mathbf{0.86} \pm 0.03$	$\mathbf{0.70} \pm 0.07$	$\mathbf{0.86} \pm 0.03$	$\mathbf{0.70} \pm 0.06$
N-signal-free	0.86 ± 0.03	0.72 ± 0.05	$\mathbf{0.90} \pm 0.06$	0.81 ± 0.11	0.89 ± 0.03	0.81 ± 0.05
% accuracy	75.73 ± 4.63		80.11 ± 5.87		$\mathbf{80.50} \pm 3.93$	

Table S5: The 5-fold cross-validation performance of an SVM classifier, using various feature set combinations as listed above each column, is shown for three-way classification on the plant automatically collected dataset.

1.5 S. cerevisiae, curated orthologs – classes balanced (N_{40})

	F_{Div}		F_{Phy}		F_{Comp}	
	AUC	MCC	AUC	MCC	AUC	MCC
MTS	0.67 ± 0.10	0.35 ± 0.20	0.85 ± 0.07	0.67 ± 0.15	0.81 ± 0.07	0.61 ± 0.12
SP	0.71 ± 0.09	0.41 ± 0.16	0.88 ± 0.08	0.75 ± 0.15	0.88 ± 0.05	0.76 ± 0.10
N-signal-free	0.79 ± 0.07	0.60 ± 0.13	0.78 ± 0.10	0.60 ± 0.20	0.76 ± 0.11	0.54 ± 0.22
% accuracy	62.86 ± 5.84		78.02 ± 8.75		75.54 ± 7.94	

	$F_{CompFull}$		F_{Div} & F_{Phy}		F_{Div} & F_{Comp}	
	AUC	MCC	AUC	MCC	AUC	MCC
MTS	0.75 ± 0.10	0.49 ± 0.18	$\mathbf{0.90} \pm 0.07$	$\mathbf{0.80} \pm 0.13$	0.83 ± 0.08	0.69 ± 0.15
SP	0.80 ± 0.05	0.61 ± 0.10	0.92 ± 0.03	0.84 ± 0.07	0.93 ± 0.03	0.84 ± 0.07
N-signal-free	0.70 ± 0.06	0.40 ± 0.13	0.86 ± 0.07	0.72 ± 0.14	0.85 ± 0.12	0.70 ± 0.23
% accuracy	66.69 ± 7.71		85.56 ± 6.42		82.44 ± 8.37	

	F_{Div} & $F_{CompFull}$		F_{Phy} & F_{Comp}		F_{Phy} & $F_{CompFull}$	
	AUC	MCC	AUC	MCC	AUC	MCC
MTS	0.80 ± 0.06	0.61 ± 0.14	0.80 ± 0.07	0.60 ± 0.14	0.86 ± 0.09	0.72 ± 0.16
SP	0.85 ± 0.04	0.70 ± 0.08	0.89 ± 0.03	0.77 ± 0.07	0.89 ± 0.03	0.78 ± 0.06
N-signal-free	0.81 ± 0.08	0.63 ± 0.12	0.76 ± 0.12	0.52 ± 0.23	0.76 ± 0.08	0.56 ± 0.16
% accuracy	76.13 ± 6.37		75.52 ± 7.95		78.65 ± 5.90	

	F_{Comp} & $F_{CompFull}$		F_{Div} & F_{Phy} & F_{Comp}		F_{Div} & F_{Phy} & $F_{CompFull}$	
	AUC	MCC	AUC	MCC	AUC	MCC
MTS	0.84 ± 0.07	0.68 ± 0.13	0.84 ± 0.07	0.72 ± 0.12	0.86 ± 0.08	0.73 ± 0.15
SP	0.91 ± 0.06	0.82 ± 0.08	0.93 ± 0.03	0.86 ± 0.07	$\mathbf{0.94} \pm 0.04$	$\mathbf{0.89} \pm 0.06$
N-signal-free	0.78 ± 0.09	0.57 ± 0.16	$\mathbf{0.87} \pm 0.10$	0.73 ± 0.20	0.85 ± 0.10	0.69 ± 0.17
% accuracy	79.27 ± 4.61		84.31 ± 7.59		84.31 ± 6.18	

	F_{Phy} & F_{Comp} & $F_{CompFull}$		F_{Div} & F_{Comp} & $F_{CompFull}$		ALL	
	AUC	MCC	AUC	MCC	AUC	MCC
MTS	0.84 ± 0.07	0.68 ± 0.13	0.86 ± 0.04	0.74 ± 0.08	0.88 ± 0.05	0.78 ± 0.09
SP	0.92 ± 0.05	0.85 ± 0.10	0.93 ± 0.03	0.85 ± 0.06	$\mathbf{0.94} \pm 0.01$	0.88 ± 0.03
N-signal-free	0.78 ± 0.09	0.57 ± 0.18	0.85 ± 0.08	0.72 ± 0.15	0.86 ± 0.07	$\mathbf{0.74} \pm 0.13$
% accuracy	79.92 ± 5.54		84.29 ± 4.35		$\mathbf{86.19} \pm 4.67$	

Table S6: The 5-fold cross-validation performance of an SVM classifier, using various feature set combinations as listed above each column, is shown for three-way classification on the yeast balanced dataset of curated orthologs.

1.6 S. cerevisiae, RBH orthologs – classes balanced (N_{40})

	F_{Div}		F_{Phy}		F_{Comp}	
	AUC	MCC	AUC	MCC	AUC	MCC
MTS	0.65 ± 0.09	0.31 ± 0.18	0.85 ± 0.05	0.70 ± 0.09	0.82 ± 0.06	0.65 ± 0.11
SP	0.60 ± 0.07	0.19 ± 0.14	0.97 ± 0.03	0.94 ± 0.06	0.95 ± 0.04	0.89 ± 0.07
N-signal-free	0.66 ± 0.08	0.35 ± 0.15	0.86 ± 0.05	0.74 ± 0.10	0.84 ± 0.04	0.69 ± 0.08
% accuracy	51.63 ± 7.21		85.87 ± 3.29		82.67 ± 5.16	

	$F_{CompFull}$		F_{Div} & F_{Phy}		F_{Div} & F_{Comp}	
	AUC	MCC	AUC	MCC	AUC	MCC
MTS	0.70 ± 0.06	0.39 ± 0.11	$\mathbf{0.88} \pm 0.03$	0.76 ± 0.03	0.86 ± 0.04	0.74 ± 0.08
SP	0.81 ± 0.09	0.63 ± 0.18	0.98 ± 0.04	0.96 ± 0.06	0.97 ± 0.03	0.93 ± 0.06
N-signal-free	0.69 ± 0.04	0.40 ± 0.10	$\mathbf{0.90} \pm 0.03$	$\mathbf{0.80} \pm 0.07$	0.88 ± 0.03	0.75 ± 0.07
% accuracy	64.40 ± 7.56		89.04 ± 1.05		86.78 ± 3.68	

	F_{Div} & $F_{CompFull}$		F_{Phy} & F_{Comp}		F_{Phy} & $F_{CompFull}$	
	AUC	MCC	AUC	MCC	AUC	MCC
MTS	0.78 ± 0.03	0.55 ± 0.07	0.83 ± 0.06	0.68 ± 0.10	0.86 ± 0.05	0.71 ± 0.09
SP	0.87 ± 0.05	0.76 ± 0.10	0.96 ± 0.04	0.92 ± 0.08	0.98 ± 0.02	0.96 ± 0.04
N-signal-free	0.81 ± 0.04	0.62 ± 0.08	0.87 ± 0.03	0.74 ± 0.07	0.84 ± 0.05	0.71 ± 0.07
% accuracy	75.81 ± 4.63		84.96 ± 5.17		85.87 ± 3.29	

	F_{Comp} & $F_{CompFull}$		F_{Div} & F_{Phy} & F_{Comp}		F_{Div} & F_{Phy} & $F_{CompFull}$	
	AUC	MCC	AUC	MCC	AUC	MCC
MTS	0.84 ± 0.04	0.69 ± 0.05	0.87 ± 0.03	$\mathbf{0.77} \pm 0.06$	0.87 ± 0.07	0.74 ± 0.13
SP	$\mathbf{0.99} \pm 0.01$	$\mathbf{0.98} \pm 0.03$	0.98 ± 0.03	0.97 ± 0.04	0.98 ± 0.02	0.97 ± 0.03
N-signal-free	0.84 ± 0.04	0.69 ± 0.07	$\mathbf{0.90} \pm 0.04$	0.78 ± 0.09	0.89 ± 0.05	0.78 ± 0.10
% accuracy	85.39 ± 2.57		$\mathbf{89.06} \pm 3.32$		88.58 ± 5.80	

	F_{Phy} & F_{Comp} & $F_{CompFull}$		F_{Div} & F_{Comp} & $F_{CompFull}$		ALL	
	AUC	MCC	AUC	MCC	AUC	MCC
MTS	0.83 ± 0.04	0.67 ± 0.06	0.87 ± 0.06	0.74 ± 0.10	$\mathbf{0.88} \pm 0.05$	0.76 ± 0.08
SP	0.98 ± 0.02	0.96 ± 0.04	0.98 ± 0.03	0.96 ± 0.04	0.98 ± 0.03	0.96 ± 0.04
N-signal-free	0.84 ± 0.03	0.69 ± 0.04	0.87 ± 0.07	0.75 ± 0.12	0.88 ± 0.06	0.76 ± 0.11
% accuracy	84.47 ± 2.51		87.67 ± 4.70		88.14 ± 4.34	

Table S7: The 5-fold cross-validation performance of an SVM classifier, using various feature set combinations as listed above each column, is shown for three-way classification on the yeast balanced dataset of automatically collected orthologs.

1.7 Human, RBH orthologs – classes balanced (N_{40})

	F_{Div}		F_{Phy}		F_{Comp}	
	AUC	MCC	AUC	MCC	AUC	MCC
MTS	0.66 ± 0.05	0.31 ± 0.11	0.85 ± 0.05	0.70 ± 0.08	0.86 ± 0.07	0.71 ± 0.14
SP	0.70 ± 0.08	0.40 ± 0.15	0.82 ± 0.05	0.64 ± 0.10	0.82 ± 0.06	0.66 ± 0.11
N-signal-free	0.69 ± 0.06	0.39 ± 0.11	0.86 ± 0.04	0.74 ± 0.09	0.88 ± 0.05	0.78 ± 0.07
% accuracy	57.61 ± 4.71		79.42 ± 2.96		80.68 ± 4.13	

	$F_{CompFull}$		F_{Div} & F_{Phy}		F_{Div} & F_{Comp}	
	AUC	MCC	AUC	MCC	AUC	MCC
MTS	0.81 ± 0.07	0.61 ± 0.13	0.87 ± 0.05	0.72 ± 0.11	0.87 ± 0.06	0.72 ± 0.11
SP	0.72 ± 0.08	0.43 ± 0.15	0.86 ± 0.06	0.73 ± 0.11	0.86 ± 0.05	0.73 ± 0.08
N-signal-free	0.77 ± 0.07	0.54 ± 0.11	0.87 ± 0.07	0.76 ± 0.13	0.88 ± 0.06	0.80 ± 0.10
% accuracy	68.36 ± 6.49		82.30 ± 6.31		82.70 ± 4.04	

	F_{Div} & $F_{CompFull}$		F_{Phy} & F_{Comp}		F_{Phy} & $F_{CompFull}$	
	AUC	MCC	AUC	MCC	AUC	MCC
MTS	0.77 ± 0.05	0.54 ± 0.09	0.87 ± 0.08	0.73 ± 0.14	0.86 ± 0.03	0.73 ± 0.06
SP	0.74 ± 0.06	0.48 ± 0.12	0.83 ± 0.07	0.67 ± 0.14	0.87 ± 0.04	0.73 ± 0.08
N-signal-free	0.77 ± 0.06	0.55 ± 0.10	0.89 ± 0.05	0.79 ± 0.08	0.88 ± 0.05	0.78 ± 0.07
% accuracy	67.92 ± 4.08		81.90 ± 4.64		82.73 ± 3.04	

	F_{Comp} & $F_{CompFull}$		F_{Div} & F_{Phy} & F_{Comp}		F_{Div} & F_{Phy} & $F_{CompFull}$	
	AUC	MCC	AUC	MCC	AUC	MCC
MTS	0.86 ± 0.04	0.72 ± 0.07	0.87 ± 0.06	0.72 ± 0.11	$\mathbf{0.89} \pm 0.05$	$\mathbf{0.77} \pm 0.09$
SP	0.83 ± 0.07	0.67 ± 0.12	0.87 ± 0.04	0.75 ± 0.07	$\mathbf{0.90} \pm 0.03$	$\mathbf{0.81} \pm 0.05$
N-signal-free	0.88 ± 0.03	0.78 ± 0.05	0.88 ± 0.07	0.78 ± 0.11	$\mathbf{0.90} \pm 0.04$	0.82 ± 0.07
% accuracy	81.07 ± 2.66		83.12 ± 4.48		$\mathbf{86.44} \pm 3.37$	

	F_{Phy} & F_{Comp} & $F_{CompFull}$		F_{Div} & F_{Comp} & $F_{CompFull}$		ALL	
	AUC	MCC	AUC	MCC	AUC	MCC
MTS	0.87 ± 0.05	0.73 ± 0.10	0.88 ± 0.03	0.76 ± 0.05	0.88 ± 0.03	0.76 ± 0.05
SP	0.85 ± 0.04	0.71 ± 0.08	0.87 ± 0.04	0.75 ± 0.09	0.88 ± 0.05	0.76 ± 0.09
N-signal-free	0.88 ± 0.03	0.78 ± 0.04	$\mathbf{0.90} \pm 0.05$	0.82 ± 0.05	$\mathbf{0.90} \pm 0.05$	$\mathbf{0.83} \pm 0.07$
% accuracy	82.32 ± 2.99		84.78 ± 3.40		85.20 ± 3.90	

Table S8: The 5-fold cross-validation performance of an SVM classifier, using various feature set combinations as listed above each column, is shown for three-way classification on the mammal balanced dataset of automatically collected orthologs.

1.8 Plant model organisms, RBH orthologs – classes balanced (N_{40})

	F_{Div}		F_{Phy}		F_{Comp}	
	AUC	MCC	AUC	MCC	AUC	MCC
MTS	0.66 ± 0.08	0.35 ± 0.14	0.78 ± 0.07	0.55 ± 0.13	0.76 ± 0.05	0.51 ± 0.09
CTP	0.77 ± 0.12	0.51 ± 0.23	0.79 ± 0.04	0.59 ± 0.10	0.80 ± 0.07	0.61 ± 0.16
N-signal-free	0.81 ± 0.09	0.67 ± 0.13	0.83 ± 0.10	0.69 ± 0.17	0.85 ± 0.05	0.72 ± 0.09
% accuracy	66.22 ± 10.11		73.27 ± 5.63		73.80 ± 4.73	

	$F_{CompFull}$		F_{Div} & F_{Phy}		F_{Div} & F_{Comp}	
	AUC	MCC	AUC	MCC	AUC	MCC
MTS	0.62 ± 0.04	0.23 ± 0.08	0.76 ± 0.09	0.53 ± 0.17	$\mathbf{0.87} \pm 0.03$	0.73 ± 0.07
CTP	0.64 ± 0.03	0.28 ± 0.06	0.77 ± 0.11	0.53 ± 0.22	$\mathbf{0.84} \pm 0.10$	$\mathbf{0.68} \pm 0.18$
N-signal-free	0.66 ± 0.05	0.34 ± 0.09	0.90 ± 0.04	0.81 ± 0.08	0.91 ± 0.05	0.84 ± 0.10
% accuracy	51.94 ± 3.16		74.37 ± 8.44		83.14 ± 6.59	

	F_{Div} & $F_{CompFull}$		F_{Phy} & F_{Comp}		F_{Phy} & $F_{CompFull}$	
	AUC	MCC	AUC	MCC	AUC	MCC
MTS	0.69 ± 0.07	0.38 ± 0.14	0.76 ± 0.05	0.52 ± 0.09	0.75 ± 0.08	0.50 ± 0.14
CTP	0.77 ± 0.09	0.54 ± 0.16	0.79 ± 0.08	0.58 ± 0.18	0.74 ± 0.02	0.48 ± 0.04
N-signal-free	0.79 ± 0.07	0.61 ± 0.12	0.86 ± 0.05	0.74 ± 0.11	0.83 ± 0.07	0.69 ± 0.13
% accuracy	66.74 ± 7.68		73.81 ± 6.02		69.95 ± 5.44	

	F_{Comp} & $F_{CompFull}$		F_{Div} & F_{Phy} & F_{Comp}		F_{Div} & F_{Phy} & $F_{CompFull}$	
	AUC	MCC	AUC	MCC	AUC	MCC
MTS	0.76 ± 0.06	0.51 ± 0.10	$\mathbf{0.87} \pm 0.03$	$\mathbf{0.74} \pm 0.06$	0.73 ± 0.07	0.46 ± 0.10
CTP	0.79 ± 0.07	0.57 ± 0.14	$\mathbf{0.84} \pm 0.10$	$\mathbf{0.68} \pm 0.18$	0.76 ± 0.09	0.52 ± 0.18
N-signal-free	0.78 ± 0.09	0.59 ± 0.17	$\mathbf{0.92} \pm 0.05$	$\mathbf{0.85} \pm 0.10$	0.88 ± 0.05	0.76 ± 0.12
% accuracy	69.97 ± 7.29		$\mathbf{83.68} \pm 6.20$		71.64 ± 4.58	

	F_{Phy} & F_{Comp} & $F_{CompFull}$		F_{Div} & F_{Comp} & $F_{CompFull}$		ALL	
	AUC	MCC	AUC	MCC	AUC	MCC
MTS	0.76 ± 0.03	0.52 ± 0.05	0.82 ± 0.04	0.64 ± 0.08	0.81 ± 0.03	0.61 ± 0.06
CTP	0.78 ± 0.06	0.56 ± 0.13	0.78 ± 0.05	0.57 ± 0.10	0.78 ± 0.05	0.56 ± 0.11
N-signal-free	0.79 ± 0.08	0.62 ± 0.15	0.88 ± 0.06	0.78 ± 0.09	0.89 ± 0.05	0.79 ± 0.07
% accuracy	70.51 ± 5.43		77.09 ± 4.31		76.53 ± 3.91	

Table S9: The 5-fold cross-validation performance of an SVM classifier, using various feature set combinations as listed above each column, is shown for three-way classification on the plant balanced dataset of automatically collected orthologs.

2 Divergence score combined with standard features in the N-terminal 20 residues

2.1 S. cerevisiae, curated orthologs (N_{20})

	F_{Div}		F_{Phy}		F_{Comp}	
	AUC	MCC	AUC	MCC	AUC	MCC
MTS	0.67 ± 0.03	0.36 ± 0.06	0.87 ± 0.03	0.73 ± 0.06	0.87 ± 0.04	0.75 ± 0.07
SP	0.50 ± 0.00	0.00 ± 0.00	0.89 ± 0.04	0.85 ± 0.02	0.94 ± 0.04	0.88 ± 0.05
N-signal-free	0.66 ± 0.02	0.36 ± 0.03	0.89 ± 0.02	0.78 ± 0.03	0.89 ± 0.03	0.80 ± 0.06
% accuracy	70.82 ± 1.61		88.71 ± 1.75		89.88 ± 2.34	

	$F_{CompFull}$		F_{Div} & F_{Phy}		F_{Div} & F_{Comp}	
	AUC	MCC	AUC	MCC	AUC	MCC
MTS	0.67 ± 0.02	0.42 ± 0.06	0.84 ± 0.03	0.70 ± 0.06	0.87 ± 0.03	0.77 ± 0.04
SP	0.67 ± 0.11	0.50 ± 0.22	0.89 ± 0.04	0.85 ± 0.04	0.91 ± 0.02	0.88 ± 0.04
N-signal-free	0.67 ± 0.02	0.42 ± 0.04	0.87 ± 0.03	0.77 ± 0.04	$\mathbf{0.90} \pm 0.02$	$\mathbf{0.83} \pm 0.04$
% accuracy	74.78 ± 1.78		87.98 ± 2.17		90.91 ± 1.34	

	F_{Div} & $F_{CompFull}$		F_{Phy} & F_{Comp}		F_{Phy} & $F_{CompFull}$	
	AUC	MCC	AUC	MCC	AUC	MCC
MTS	0.80 ± 0.03	0.65 ± 0.06	0.87 ± 0.04	0.76 ± 0.06	0.88 ± 0.03	0.75 ± 0.05
SP	0.78 ± 0.07	0.66 ± 0.11	0.95 ± 0.03	0.89 ± 0.05	0.94 ± 0.04	0.91 ± 0.03
N-signal-free	0.79 ± 0.02	0.63 ± 0.04	$\mathbf{0.90} \pm 0.03$	0.80 ± 0.05	0.88 ± 0.03	0.77 ± 0.04
% accuracy	82.99 ± 1.66		90.18 ± 1.76		89.44 ± 1.71	

	F_{Comp} & $F_{CompFull}$		F_{Div} & F_{Phy} & F_{Comp}		F_{Div} & F_{Phy} & $F_{CompFull}$	
	AUC	MCC	AUC	MCC	AUC	MCC
MTS	$\mathbf{0.89} \pm 0.02$	0.80 ± 0.03	0.87 ± 0.02	0.77 ± 0.03	0.88 ± 0.03	0.77 ± 0.04
SP	0.96 ± 0.02	0.92 ± 0.07	0.90 ± 0.04	0.87 ± 0.05	0.95 ± 0.05	0.94 ± 0.07
N-signal-free	$\mathbf{0.90} \pm 0.02$	0.81 ± 0.03	$\mathbf{0.90} \pm 0.02$	0.83 ± 0.02	0.89 ± 0.02	0.79 ± 0.04
% accuracy	91.20 ± 1.58		90.91 ± 0.40		90.47 ± 1.38	

	F_{Phy} & F_{Comp} & $F_{CompFull}$		F_{Div} & F_{Comp} & $F_{CompFull}$		ALL	
	AUC	MCC	AUC	MCC	AUC	MCC
MTS	$\mathbf{0.89} \pm 0.02$	0.80 ± 0.02	$\mathbf{0.89} \pm 0.02$	0.80 ± 0.02	$\mathbf{0.89} \pm 0.01$	$\mathbf{0.81} \pm 0.02$
SP	0.97 ± 0.03	0.92 ± 0.07	$\mathbf{0.98} \pm 0.03$	$\mathbf{0.97} \pm 0.04$	$\mathbf{0.98} \pm 0.03$	$\mathbf{0.97} \pm 0.04$
N-signal-free	$\mathbf{0.90} \pm 0.01$	0.81 ± 0.02	$\mathbf{0.90} \pm 0.01$	0.82 ± 0.03	$\mathbf{0.90} \pm 0.01$	$\mathbf{0.83} \pm 0.02$
% accuracy	91.49 ± 1.26		91.93 ± 1.58		$\mathbf{92.23} \pm 1.25$	

Table S10: The 5-fold cross-validation performance of an SVM classifier, using various feature set combinations as listed above each column, is shown for three-way classification on the yeast curated ortholog dataset.

2.2 S. cerevisiae, RBH orthologs (N_{20})

	F_{Div}		F_{Phy}		F_{Comp}	
	AUC	MCC	AUC	MCC	AUC	MCC
MTS	0.65 ± 0.04	0.34 ± 0.08	0.86 ± 0.04	0.73 ± 0.08	0.87 ± 0.04	0.77 ± 0.08
SP	0.50 ± 0.00	0.00 ± 0.00	0.93 ± 0.04	0.88 ± 0.06	$\mathbf{0.97} \pm 0.03$	0.92 ± 0.05
N-signal-free	0.64 ± 0.04	0.33 ± 0.10	0.90 ± 0.03	0.80 ± 0.06	0.90 ± 0.03	0.81 ± 0.05
% accuracy	70.06 ± 3.05		89.32 ± 2.79		90.84 ± 2.87	

	$F_{CompFull}$		F_{Div} & F_{Phy}		F_{Div} & F_{Comp}	
	AUC	MCC	AUC	MCC	AUC	MCC
MTS	0.66 ± 0.03	0.41 ± 0.07	0.85 ± 0.03	0.72 ± 0.06	0.87 ± 0.03	0.78 ± 0.06
SP	0.74 ± 0.08	0.65 ± 0.13	0.93 ± 0.04	0.86 ± 0.09	0.94 ± 0.03	0.91 ± 0.03
N-signal-free	0.68 ± 0.01	0.45 ± 0.03	0.89 ± 0.04	0.79 ± 0.08	0.91 ± 0.03	0.84 ± 0.05
% accuracy	75.94 ± 1.12		89.20 ± 2.23		91.43 ± 2.34	

	F_{Div} & $F_{CompFull}$		F_{Phy} & F_{Comp}		F_{Phy} & $F_{CompFull}$	
	AUC	MCC	AUC	MCC	AUC	MCC
MTS	0.78 ± 0.04	0.61 ± 0.06	0.87 ± 0.05	0.76 ± 0.08	0.88 ± 0.03	0.74 ± 0.03
SP	0.82 ± 0.08	0.75 ± 0.14	0.96 ± 0.02	0.91 ± 0.04	0.95 ± 0.04	0.92 ± 0.06
N-signal-free	0.80 ± 0.04	0.65 ± 0.07	0.90 ± 0.03	0.81 ± 0.06	0.89 ± 0.02	0.77 ± 0.03
% accuracy	83.45 ± 3.23		90.61 ± 3.10		89.32 ± 1.28	

	F_{Comp} & $F_{CompFull}$		F_{Div} & F_{Phy} & F_{Comp}		F_{Div} & F_{Phy} & $F_{CompFull}$	
	AUC	MCC	AUC	MCC	AUC	MCC
MTS	0.89 ± 0.04	0.79 ± 0.07	0.87 ± 0.03	0.77 ± 0.06	0.87 ± 0.04	0.76 ± 0.07
SP	0.96 ± 0.02	0.95 ± 0.02	0.94 ± 0.03	0.91 ± 0.03	0.96 ± 0.03	0.94 ± 0.02
N-signal-free	0.91 ± 0.04	0.82 ± 0.07	0.91 ± 0.03	0.83 ± 0.05	0.90 ± 0.03	0.81 ± 0.06
% accuracy	91.79 ± 2.90		91.31 ± 2.22		90.73 ± 2.54	

	F_{Phy} & F_{Comp} & $F_{CompFull}$		F_{Div} & F_{Comp} & $F_{CompFull}$		ALL	
	AUC	MCC	AUC	MCC	AUC	MCC
MTS	0.89 ± 0.05	0.80 ± 0.07	$\mathbf{0.90} \pm 0.03$	$\mathbf{0.81} \pm 0.06$	0.89 ± 0.04	$\mathbf{0.81} \pm 0.06$
SP	$\mathbf{0.97} \pm 0.03$	$\mathbf{0.96} \pm 0.03$	0.96 ± 0.02	0.93 ± 0.03	$\mathbf{0.97} \pm 0.03$	0.95 ± 0.02
N-signal-free	0.91 ± 0.04	0.83 ± 0.07	$\mathbf{0.92} \pm 0.03$	0.84 ± 0.05	$\mathbf{0.92} \pm 0.03$	$\mathbf{0.85} \pm 0.05$
% accuracy	92.02 ± 2.85		92.49 ± 2.25		$\mathbf{92.61} \pm 2.14$	

Table S11: The 5-fold cross-validation performance of an SVM classifier, using various feature set combinations as listed above each column, is shown for three-way classification on the yeast automatically collected dataset.

2.3 Human, RBH orthologs (N_{20})

	F_{Div}		F_{Phy}		F_{Comp}	
	AUC	MCC	AUC	MCC	AUC	MCC
MTS	0.52 ± 0.03	0.10 ± 0.18	0.81 ± 0.06	0.64 ± 0.08	0.82 ± 0.08	0.65 ± 0.11
SP	0.65 ± 0.05	0.29 ± 0.10	0.87 ± 0.03	0.76 ± 0.05	0.89 ± 0.02	0.78 ± 0.04
N-signal-free	0.66 ± 0.05	0.35 ± 0.09	0.90 ± 0.01	0.81 ± 0.02	0.93 ± 0.02	0.88 ± 0.05
% accuracy	65.11 ± 3.55		87.37 ± 1.71		89.32 ± 2.09	

	$F_{CompFull}$		F_{Div} & F_{Phy}		F_{Div} & F_{Comp}	
	AUC	MCC	AUC	MCC	AUC	MCC
MTS	0.67 ± 0.07	0.43 ± 0.15	0.81 ± 0.06	0.67 ± 0.08	0.82 ± 0.04	0.68 ± 0.07
SP	0.75 ± 0.06	0.52 ± 0.12	0.89 ± 0.03	0.79 ± 0.05	0.90 ± 0.03	0.80 ± 0.06
N-signal-free	0.75 ± 0.06	0.52 ± 0.12	0.92 ± 0.02	0.85 ± 0.05	0.94 ± 0.04	0.88 ± 0.08
% accuracy	74.89 ± 5.49		89.02 ± 2.74		90.08 ± 2.09	

	F_{Div} & $F_{CompFull}$		F_{Phy} & F_{Comp}		F_{Phy} & $F_{CompFull}$	
	AUC	MCC	AUC	MCC	AUC	MCC
MTS	0.73 ± 0.07	0.53 ± 0.11	0.83 ± 0.04	0.67 ± 0.07	0.82 ± 0.05	0.64 ± 0.03
SP	0.81 ± 0.05	0.62 ± 0.06	0.89 ± 0.03	0.79 ± 0.05	0.88 ± 0.02	0.78 ± 0.02
N-signal-free	0.81 ± 0.04	0.64 ± 0.05	0.93 ± 0.02	0.88 ± 0.04	0.91 ± 0.03	0.82 ± 0.06
% accuracy	79.85 ± 1.35		89.47 ± 1.84		87.82 ± 1.71	

	F_{Comp} & $F_{CompFull}$		F_{Div} & F_{Phy} & F_{Comp}		F_{Div} & F_{Phy} & $F_{CompFull}$	
	AUC	MCC	AUC	MCC	AUC	MCC
MTS	0.81 ± 0.07	0.66 ± 0.09	$\mathbf{0.84} \pm 0.02$	$\mathbf{0.72} \pm 0.02$	$\mathbf{0.84} \pm 0.03$	0.70 ± 0.06
SP	0.89 ± 0.03	0.77 ± 0.06	0.90 ± 0.04	0.80 ± 0.07	$\mathbf{0.91} \pm 0.04$	$\mathbf{0.81} \pm 0.05$
N-signal-free	0.93 ± 0.04	0.87 ± 0.09	0.94 ± 0.04	$\mathbf{0.89} \pm 0.09$	0.93 ± 0.03	0.85 ± 0.06
% accuracy	89.32 ± 3.12		$\mathbf{90.83} \pm 3.42$		89.62 ± 1.95	

	F_{Phy} & F_{Comp} & $F_{CompFull}$		F_{Div} & F_{Comp} & $F_{CompFull}$		ALL	
	AUC	MCC	AUC	MCC	AUC	MCC
MTS	0.83 ± 0.07	0.69 ± 0.07	0.83 ± 0.06	0.71 ± 0.08	$\mathbf{0.84} \pm 0.06$	0.70 ± 0.07
SP	0.89 ± 0.02	0.78 ± 0.03	$\mathbf{0.91} \pm 0.02$	0.80 ± 0.04	0.89 ± 0.02	0.79 ± 0.03
N-signal-free	0.94 ± 0.04	0.88 ± 0.07	0.94 ± 0.04	$\mathbf{0.89} \pm 0.08$	$\mathbf{0.95} \pm 0.04$	$\mathbf{0.89} \pm 0.09$
% accuracy	89.77 ± 1.73		90.68 ± 1.73		90.38 ± 1.87	

Table S12: The 5-fold cross-validation performance of an SVM classifier, using various feature set combinations as listed above each column, is shown for three-way classification on the mammal automatically collected dataset.

2.4 Plant model organisms, RBH orthologs (N_{20})

	F_{Div}		F_{Phy}		F_{Comp}	
	AUC	MCC	AUC	MCC	AUC	MCC
MTS	0.61 ± 0.05	0.30 ± 0.15	$\mathbf{0.88} \pm 0.07$	0.72 ± 0.10	$\mathbf{0.88} \pm 0.02$	0.76 ± 0.05
SP	0.50 ± 0.00	0.00 ± 0.00	0.76 ± 0.09	0.59 ± 0.16	0.83 ± 0.17	0.72 ± 0.25
CTP	0.78 ± 0.08	0.54 ± 0.15	0.82 ± 0.05	0.65 ± 0.10	0.88 ± 0.05	0.75 ± 0.10
N-signal-free	0.80 ± 0.05	0.60 ± 0.09	0.86 ± 0.08	0.73 ± 0.13	0.88 ± 0.04	0.75 ± 0.08
% accuracy	65.05 ± 6.00		78.29 ± 6.40		83.06 ± 4.52	

	$F_{CompFull}$		F_{Div} & F_{Phy}		F_{Div} & F_{Comp}	
	AUC	MCC	AUC	MCC	AUC	MCC
MTS	0.58 ± 0.06	0.19 ± 0.15	0.85 ± 0.09	0.69 ± 0.16	0.87 ± 0.04	0.75 ± 0.08
SP	0.69 ± 0.14	0.48 ± 0.29	0.66 ± 0.17	0.40 ± 0.39	0.80 ± 0.22	0.65 ± 0.39
CTP	0.68 ± 0.08	0.35 ± 0.15	0.88 ± 0.03	0.76 ± 0.07	$\mathbf{0.90} \pm 0.05$	$\mathbf{0.78} \pm 0.10$
N-signal-free	0.66 ± 0.05	0.31 ± 0.08	$\mathbf{0.94} \pm 0.05$	$\mathbf{0.88} \pm 0.10$	0.92 ± 0.05	0.86 ± 0.09
% accuracy	53.33 ± 7.70		83.81 ± 6.23		85.64 ± 4.66	

	F_{Div} & $F_{CompFull}$		F_{Phy} & F_{Comp}		F_{Phy} & $F_{CompFull}$	
	AUC	MCC	AUC	MCC	AUC	MCC
MTS	0.63 ± 0.04	0.32 ± 0.11	$\mathbf{0.88} \pm 0.02$	$\mathbf{0.78} \pm 0.07$	$\mathbf{0.88} \pm 0.06$	0.76 ± 0.08
SP	0.70 ± 0.22	0.48 ± 0.46	0.83 ± 0.12	0.70 ± 0.17	$\mathbf{0.93} \pm 0.09$	$\mathbf{0.83} \pm 0.13$
CTP	0.78 ± 0.05	0.53 ± 0.11	0.89 ± 0.07	0.76 ± 0.13	0.84 ± 0.05	0.68 ± 0.10
N-signal-free	0.82 ± 0.06	0.63 ± 0.10	0.87 ± 0.04	0.75 ± 0.06	0.87 ± 0.06	0.75 ± 0.10
% accuracy	67.30 ± 4.19		83.41 ± 5.93		81.60 ± 4.40	

	F_{Comp} & $F_{CompFull}$		F_{Div} & F_{Phy} & F_{Comp}		F_{Div} & F_{Phy} & $F_{CompFull}$	
	AUC	MCC	AUC	MCC	AUC	MCC
MTS	0.85 ± 0.04	0.73 ± 0.05	$\mathbf{0.88} \pm 0.06$	$\mathbf{0.78} \pm 0.09$	0.84 ± 0.07	0.71 ± 0.10
SP	0.86 ± 0.14	0.72 ± 0.20	0.83 ± 0.20	0.66 ± 0.39	0.86 ± 0.21	0.67 ± 0.40
CTP	0.88 ± 0.04	0.75 ± 0.08	$\mathbf{0.90} \pm 0.05$	$\mathbf{0.78} \pm 0.11$	0.87 ± 0.07	0.72 ± 0.14
N-signal-free	0.87 ± 0.04	0.75 ± 0.06	0.92 ± 0.04	0.85 ± 0.09	0.93 ± 0.03	0.86 ± 0.06
% accuracy	82.33 ± 3.71		$\mathbf{86.36} \pm 5.87$		83.79 ± 6.66	

	F_{Phy} & F_{Comp} & $F_{CompFull}$		F_{Div} & F_{Comp} & $F_{CompFull}$		ALL	
	AUC	MCC	AUC	MCC	AUC	MCC
MTS	0.87 ± 0.05	0.77 ± 0.07	0.85 ± 0.06	0.72 ± 0.07	0.87 ± 0.05	0.77 ± 0.06
SP	0.86 ± 0.14	0.72 ± 0.20	0.86 ± 0.14	0.81 ± 0.16	0.86 ± 0.14	0.81 ± 0.16
CTP	0.88 ± 0.05	0.75 ± 0.09	0.88 ± 0.04	0.75 ± 0.07	0.89 ± 0.05	$\mathbf{0.78} \pm 0.10$
N-signal-free	0.87 ± 0.04	0.76 ± 0.07	0.90 ± 0.06	0.81 ± 0.10	0.91 ± 0.04	0.83 ± 0.06
% accuracy	83.06 ± 4.50		83.80 ± 4.11		85.64 ± 3.64	

Table S13: The 5-fold cross-validation performance of an SVM classifier, using various feature set combinations as listed above each column, is shown for three-way classification on the plant automatically collected dataset.

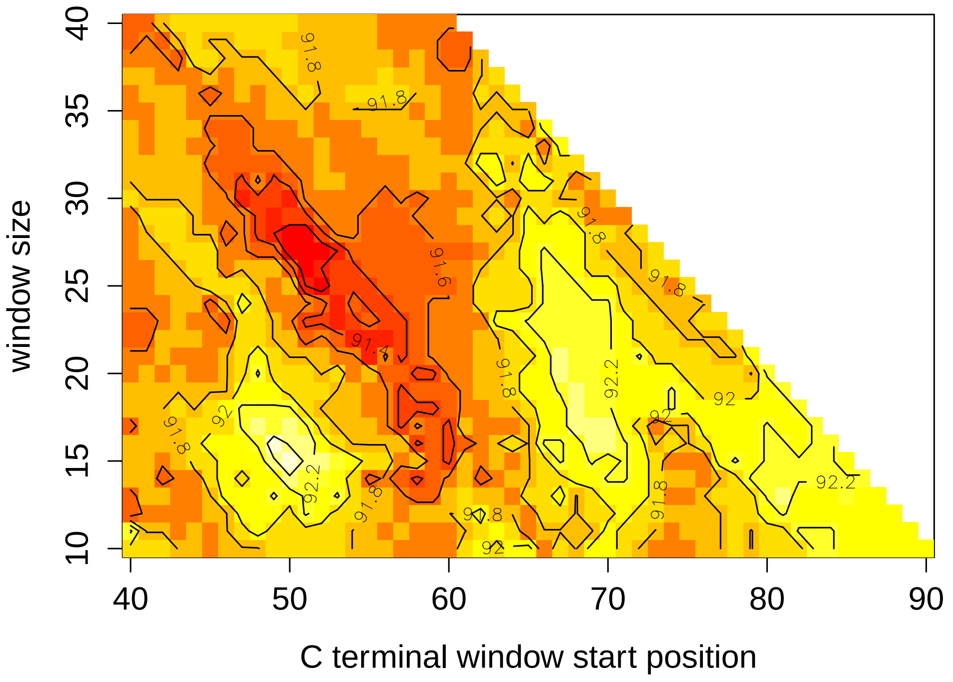

Figure S1: Heat map for two parameters of NCDiff in terms of accuracy based on yeast curated dataset using divergence and classical features in N-terminal 20 residues.

3 Post hoc analysis for NCDiff parameters

Since we made an arbitrary choice when defining divergence features, parameters for NCDiff have been searched with in the yeast curated dataset: window length and normalization start position in C-terminal from 40 to 80 with or without classical N-terminal features of the first 20 amino acids. In the case with the classical features, avearge accuracy for parameter space is 91.82%, and best accuracy is 92.52% with either a combination of window size 15 and start position at 50 or combination 16 and 49. Similarily, in the case without the classical features, average accuracy is 70.45% and best accuracy is 71.99% when window size is 40 and start position is 48. Because of multiple test, this difference does not seem to be significant; however, analysis result is summarized in Figure S1 and S2.

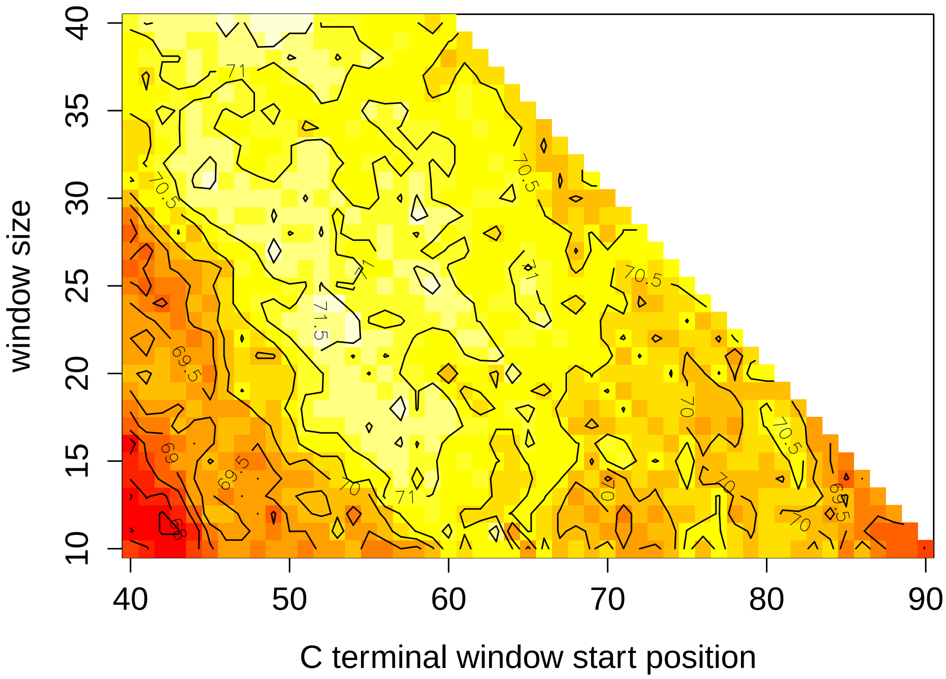

Figure S2: Heat map for two parameters of NCDiff in terms of accuracy based on yeast curated dataset using only divergence features.

4 Divergence score combined with standard features in N-terminal 40 residues

4.1 S. cerevisiae, curated orthologs (N_{40})

	F_{Div}			F_{Phy}			F_{Comp}		
Predicted →	MTS	SP	N-signal-free	MTS	SP	N-signal-free	MTS	SP	N-signal-free
MTS	83	0	96	141	2	36	140	2	37
SP	16	0	37	6	30	17	6	31	16
N-signal-free	50	0	400	30	10	410	26	10	414

	$F_{CompFull}$			F_{Div} & F_{Phy}			F_{Div} & F_{Comp}		
Predicted →	MTS	SP	N-signal-free	MTS	SP	N-signal-free	MTS	SP	N-signal-free
MTS	73	1	105	132	2	45	133	2	44
SP	4	19	30	4	43	6	3	41	9
N-signal-free	29	3	418	23	6	421	10	6	434

	F_{Div} & $F_{CompFull}$			F_{Phy} & F_{Comp}			F_{Phy} & $F_{CompFull}$		
Predicted →	MTS	SP	N-signal-free	MTS	SP	N-signal-free	MTS	SP	N-signal-free
MTS	120	1	58	144	2	33	136	2	41
SP	3	31	19	4	32	17	3	35	15
N-signal-free	28	7	415	26	10	414	35	6	409

	F_{Comp} & $F_{CompFull}$			F_{Div} & F_{Phy} & F_{Comp}			F_{Div} & F_{Phy} & $F_{CompFull}$		
Predicted →	MTS	SP	N-signal-free	MTS	SP	N-signal-free	MTS	SP	N-signal-free
MTS	141	1	37	133	3	43	137	2	40
SP	2	32	19	3	43	7	3	42	8
N-signal-free	18	5	427	11	7	432	22	5	423

	F_{Phy} & F_{Comp} & $F_{CompFull}$			F_{Div} & F_{Comp} & $F_{CompFull}$			ALL		
Predicted →	MTS	SP	N-signal-free	MTS	SP	N-signal-free	MTS	SP	N-signal-free
MTS	140	1	38	137	1	41	137	1	41
SP	2	34	17	2	44	7	2	43	8
N-signal-free	22	7	421	16	5	429	15	6	429

Table S14: Confusion matrix of the 5-fold cross-validation of an SVM classifier, using various feature set combinations as listed above each column, is shown for three-way classification on the yeast curated ortholog dataset.

4.2 S. cerevisiae, RBH orthologs (N_{40})

Predicted →	F_{Div} MTS	F_{Div} SP	F_{Div} N-signal-free	F_{Phy} MTS	F_{Phy} SP	F_{Phy} N-signal-free	F_{Comp} MTS	F_{Comp} SP	F_{Comp} N-signal-free
MTS	91	0	128	168	4	47	172	3	44
SP	18	0	55	8	47	18	7	52	14
N-signal-free	54	0	506	33	14	513	27	9	524

Predicted →	$F_{CompFull}$ MTS	$F_{CompFull}$ SP	$F_{CompFull}$ N-signal-free	F_{Div} & F_{Phy} MTS	F_{Div} & F_{Phy} SP	F_{Div} & F_{Phy} N-signal-free	F_{Div} & F_{Comp} MTS	F_{Div} & F_{Comp} SP	F_{Div} & F_{Comp} N-signal-free
MTS	82	1	136	164	3	52	171	3	45
SP	4	35	34	9	52	12	7	56	10
N-signal-free	29	1	530	19	6	535	19	6	535

Predicted →	F_{Div} & $F_{CompFull}$ MTS	F_{Div} & $F_{CompFull}$ SP	F_{Div} & $F_{CompFull}$ N-signal-free	F_{Phy} & F_{Comp} MTS	F_{Phy} & F_{Comp} SP	F_{Phy} & F_{Comp} N-signal-free	F_{Phy} & $F_{CompFull}$ MTS	F_{Phy} & $F_{CompFull}$ SP	F_{Phy} & $F_{CompFull}$ N-signal-free
MTS	138	4	77	174	2	43	172	3	44
SP	6	48	19	6	53	14	2	54	17
N-signal-free	34	1	525	25	11	524	43	5	512

Predicted →	F_{Comp} & $F_{CompFull}$ MTS	F_{Comp} & $F_{CompFull}$ SP	F_{Comp} & $F_{CompFull}$ N-signal-free	F_{Div} & F_{Phy} & F_{Comp} MTS	F_{Div} & F_{Phy} & F_{Comp} SP	F_{Div} & F_{Phy} & F_{Comp} N-signal-free	F_{Div} & F_{Phy} & $F_{CompFull}$ MTS	F_{Div} & F_{Phy} & $F_{CompFull}$ SP	F_{Div} & F_{Phy} & $F_{CompFull}$ N-signal-free
MTS	172	0	47	171	4	44	168	3	48
SP	3	58	12	9	56	8	5	61	7
N-signal-free	24	4	532	19	8	533	23	2	535

Predicted →	F_{Phy} & F_{Comp} & $F_{CompFull}$ MTS	F_{Phy} & F_{Comp} & $F_{CompFull}$ SP	F_{Phy} & F_{Comp} & $F_{CompFull}$ N-signal-free	F_{Div} & F_{Comp} & $F_{CompFull}$ MTS	F_{Div} & F_{Comp} & $F_{CompFull}$ SP	F_{Div} & F_{Comp} & $F_{CompFull}$ N-signal-free	ALL MTS	ALL SP	ALL N-signal-free
MTS	173	0	46	175	1	43	176	2	41
SP	3	57	13	3	63	7	4	63	6
N-signal-free	26	5	529	18	2	540	16	2	542

Table S15: Confusion matrix of the 5-fold cross-validation of an SVM classifier, using various feature set combinations as listed above each column, is shown for three-way classification on the yeast automatically collected dataset.

4.3 Human, RBH orthologs (N_{40})

	F_{Div}			F_{Phy}			F_{Comp}		
Predicted →	MTS	SP	N-signal-free	MTS	SP	N-signal-free	MTS	SP	N-signal-free
MTS	3	35	43	50	16	15	58	10	13
SP	1	84	84	15	124	30	13	132	24
N-signal-free	2	67	346	10	27	378	8	18	389

	$F_{CompFull}$			F_{Div} & F_{Phy}			F_{Div} & F_{Comp}		
Predicted →	MTS	SP	N-signal-free	MTS	SP	N-signal-free	MTS	SP	N-signal-free
MTS	31	14	36	53	10	18	54	13	14
SP	6	102	61	11	134	24	7	143	19
N-signal-free	13	37	365	10	18	387	8	16	391

	F_{Div} & $F_{CompFull}$			F_{Phy} & F_{Comp}			F_{Phy} & $F_{CompFull}$		
Predicted →	MTS	SP	N-signal-free	MTS	SP	N-signal-free	MTS	SP	N-signal-free
MTS	40	14	27	58	10	13	58	13	10
SP	8	121	40	13	135	21	9	129	31
N-signal-free	11	34	370	9	19	387	12	13	390

	F_{Comp} & $F_{CompFull}$			F_{Div} & F_{Phy} & F_{Comp}			F_{Div} & F_{Phy} & $F_{CompFull}$		
Predicted →	MTS	SP	N-signal-free	MTS	SP	N-signal-free	MTS	SP	N-signal-free
MTS	58	11	12	55	13	13	59	9	13
SP	8	130	31	7	142	20	12	136	21
N-signal-free	7	18	390	7	14	394	12	15	388

	F_{Phy} & F_{Comp} & $F_{CompFull}$			F_{Div} & F_{Comp} & $F_{CompFull}$			ALL		
Predicted →	MTS	SP	N-signal-free	MTS	SP	N-signal-free	MTS	SP	N-signal-free
MTS	63	9	9	58	11	12	60	11	10
SP	9	133	27	7	142	20	8	140	21
N-signal-free	8	17	390	9	13	393	8	13	394

Table S16: Confusion matrix of the 5-fold cross-validation of an SVM classifier, using various feature set combinations as listed above each column, is shown for three-way classification on the mammal automatically collected dataset.

4.4 Plant model organisms, RBH orthologs (N_{40})

	F_{Div}				F_{Phy}				F_{Comp}			
Predicted →	MTS	SP	CTP	N-signal-free	MTS	SP	CTP	N-signal-free	MTS	SP	CTP	N-signal-free
MTS	17	0	35	9	30	0	25	6	44	0	12	5
SP	3	0	4	8	2	2	5	6	1	6	5	3
CTP	3	0	90	6	13	1	78	7	10	2	83	4
N-signal-free	6	0	21	70	7	4	8	78	11	2	8	76

	$F_{CompFull}$				F_{Div} & F_{Phy}				F_{Div} & F_{Comp}			
Predicted →	MTS	SP	CTP	N-signal-free	MTS	SP	CTP	N-signal-free	MTS	SP	CTP	N-signal-free
MTS	16	0	27	18	23	1	31	6	40	0	17	4
SP	0	6	1	8	3	5	5	2	1	8	5	1
CTP	11	1	67	20	11	1	85	2	9	2	85	3
N-signal-free	12	2	27	56	2	2	9	84	8	2	8	79

	F_{Div} & $F_{CompFull}$				F_{Phy} & F_{Comp}				F_{Phy} & $F_{CompFull}$			
Predicted →	MTS	SP	CTP	N-signal-free	MTS	SP	CTP	N-signal-free	MTS	SP	CTP	N-signal-free
MTS	21	0	30	10	43	0	15	3	28	0	26	7
SP	2	6	2	5	1	6	6	2	0	10	3	2
CTP	8	0	81	10	10	3	82	4	17	2	74	6
N-signal-free	8	0	14	75	11	2	8	76	4	1	15	77

	F_{Comp} & $F_{CompFull}$				F_{Div} & F_{Phy} & F_{Comp}				F_{Div} & F_{Phy} & $F_{CompFull}$			
Predicted →	MTS	SP	CTP	N-signal-free	MTS	SP	CTP	N-signal-free	MTS	SP	CTP	N-signal-free
MTS	41	0	13	7	40	0	16	5	29	0	28	4
SP	1	11	3	0	2	8	4	1	0	8	5	2
CTP	13	2	78	6	9	2	84	4	15	0	78	6
N-signal-free	13	1	6	77	8	1	7	81	4	1	9	83

	F_{Phy} & F_{Comp} & $F_{CompFull}$				F_{Div} & F_{Comp} & $F_{CompFull}$				ALL			
Predicted →	MTS	SP	CTP	N-signal-free	MTS	SP	CTP	N-signal-free	MTS	SP	CTP	N-signal-free
MTS	39	0	15	7	40	0	17	4	41	0	17	3
SP	1	11	3	0	1	9	5	0	1	10	4	0
CTP	11	2	79	7	6	1	87	5	6	1	87	5
N-signal-free	12	1	7	77	8	1	6	82	8	1	7	81

Table S17: Confusion matrix of the 5-fold cross-validation of an SVM classifier, using various feature set combinations as listed above each column, is shown for three-way classification on the plant automatically collected dataset.

4.5 S. cerevisiae, curated orthologs – classes balanced (N_{40})

	F_{Div}			F_{Phy}			F_{Comp}		
Predicted →	MTS	SP	N-signal-free	MTS	SP	N-signal-free	MTS	SP	N-signal-free
MTS	31	15	7	46	2	5	40	3	10
SP	16	34	3	6	45	2	4	46	3
N-signal-free	9	9	35	12	8	33	11	8	34

	$F_{CompFull}$			F_{Div} & F_{Phy}			F_{Div} & F_{Comp}		
Predicted →	MTS	SP	N-signal-free	MTS	SP	N-signal-free	MTS	SP	N-signal-free
MTS	37	3	13	45	1	7	39	4	10
SP	8	38	7	2	48	3	2	49	2
N-signal-free	13	9	31	4	6	43	6	4	43

	F_{Div} & $F_{CompFull}$			F_{Phy} & F_{Comp}			F_{Phy} & $F_{CompFull}$		
Predicted →	MTS	SP	N-signal-free	MTS	SP	N-signal-free	MTS	SP	N-signal-free
MTS	38	7	8	39	4	10	45	0	8
SP	5	43	5	3	47	3	3	46	4
N-signal-free	8	5	40	12	7	34	10	9	34

	F_{Comp} & $F_{CompFull}$			F_{Div} & F_{Phy} & F_{Comp}			F_{Div} & F_{Phy} & $F_{CompFull}$		
Predicted →	MTS	SP	N-signal-free	MTS	SP	N-signal-free	MTS	SP	N-signal-free
MTS	43	1	9	40	3	10	43	0	10
SP	2	46	5	2	49	2	3	48	2
N-signal-free	11	5	37	5	3	45	7	3	43

	F_{Phy} & F_{Comp} & $F_{CompFull}$			F_{Div} & F_{Comp} & $F_{CompFull}$			ALL		
Predicted →	MTS	SP	N-signal-free	MTS	SP	N-signal-free	MTS	SP	N-signal-free
MTS	43	0	10	43	3	7	44	2	7
SP	2	48	3	2	49	2	1	50	2
N-signal-free	11	6	36	7	4	42	6	4	43

Table S18: Confusion matrix of the 5-fold cross-validation of an SVM classifier, using various feature set combinations as listed above each column, is shown for three-way classification on the yeast balanced dataset of curated orthologs.

4.6 S. cerevisiae, RBH orthologs – classes balanced (N_{40})

	F_{Div}			F_{Phy}			F_{Comp}		
Predicted →	MTS	SP	N-signal-free	MTS	SP	N-signal-free	MTS	SP	N-signal-free
MTS	36	28	9	59	2	12	55	4	14
SP	18	42	13	3	70	0	4	69	0
N-signal-free	11	27	35	13	1	59	13	3	57

	$F_{CompFull}$			F_{Div} & F_{Phy}			F_{Div} & F_{Comp}		
Predicted →	MTS	SP	N-signal-free	MTS	SP	N-signal-free	MTS	SP	N-signal-free
MTS	47	7	19	61	1	11	57	2	14
SP	13	53	7	3	70	0	2	71	0
N-signal-free	23	9	41	9	0	64	8	3	62

	F_{Div} & $F_{CompFull}$			F_{Phy} & F_{Comp}			F_{Phy} & $F_{CompFull}$		
Predicted →	MTS	SP	N-signal-free	MTS	SP	N-signal-free	MTS	SP	N-signal-free
MTS	55	4	14	56	3	14	61	1	11
SP	12	57	4	4	69	0	1	72	0
N-signal-free	16	3	54	11	1	61	16	2	55

	F_{Comp} & $F_{CompFull}$			F_{Div} & F_{Phy} & F_{Comp}			F_{Div} & F_{Phy} & $F_{CompFull}$		
Predicted →	MTS	SP	N-signal-free	MTS	SP	N-signal-free	MTS	SP	N-signal-free
MTS	58	0	15	59	0	14	59	1	13
SP	1	72	0	2	71	0	2	71	0
N-signal-free	15	1	57	7	1	65	9	0	64

	F_{Phy} & F_{Comp} & $F_{CompFull}$			F_{Div} & F_{Comp} & $F_{CompFull}$			ALL		
Predicted →	MTS	SP	N-signal-free	MTS	SP	N-signal-free	MTS	SP	N-signal-free
MTS	57	1	15	61	0	12	61	0	12
SP	2	71	0	2	70	1	2	70	1
N-signal-free	15	1	57	11	1	61	10	1	62

Table S19: Confusion matrix of the 5-fold cross-validation of an SVM classifier, using various feature set combinations as listed above each column, is shown for three-way classification on the yeast balanced dataset of automatically collected orthologs.

4.7 Human, RBH orthologs – classes balanced (N_{40})

	F_{Div}			F_{Phy}			F_{Comp}		
Predicted →	MTS	SP	N-signal-free	MTS	SP	N-signal-free	MTS	SP	N-signal-free
MTS	45	19	17	66	11	4	69	9	3
SP	21	53	7	11	63	7	14	60	7
N-signal-free	18	21	42	7	10	64	7	7	67

	$F_{CompFull}$			F_{Div} & F_{Phy}			F_{Div} & F_{Comp}		
Predicted →	MTS	SP	N-signal-free	MTS	SP	N-signal-free	MTS	SP	N-signal-free
MTS	61	12	8	69	6	6	71	7	3
SP	16	51	14	11	66	4	13	65	3
N-signal-free	7	20	54	8	8	65	9	7	65

	F_{Div} & $F_{CompFull}$			F_{Phy} & F_{Comp}			F_{Phy} & $F_{CompFull}$		
Predicted →	MTS	SP	N-signal-free	MTS	SP	N-signal-free	MTS	SP	N-signal-free
MTS	57	13	11	70	9	2	67	9	5
SP	16	52	13	12	62	7	9	68	4
N-signal-free	11	14	56	7	7	67	7	8	66

	F_{Comp} & $F_{CompFull}$			F_{Div} & F_{Phy} & F_{Comp}			F_{Div} & F_{Phy} & $F_{CompFull}$		
Predicted →	MTS	SP	N-signal-free	MTS	SP	N-signal-free	MTS	SP	N-signal-free
MTS	66	10	5	70	8	3	71	5	5
SP	11	63	7	10	68	3	8	69	4
N-signal-free	5	8	68	10	7	64	7	4	70

	F_{Phy} & F_{Comp} & $F_{CompFull}$			F_{Div} & F_{Comp} & $F_{CompFull}$			ALL		
Predicted →	MTS	SP	N-signal-free	MTS	SP	N-signal-free	MTS	SP	N-signal-free
MTS	68	8	5	71	6	4	71	6	4
SP	11	65	5	11	66	4	11	67	3
N-signal-free	6	8	67	6	6	69	6	6	69

Table S20: Confusion matrix of the 5-fold cross-validation of an SVM classifier, using various feature set combinations as listed above each column, is shown for three-way classification on the mammal balanced dataset of automatically collected orthologs.

4.8 Plant model organisms, RBH orthologs – classes balanced (N_{40})

	F_{Div}			F_{Phy}			F_{Comp}		
Predicted →	MTS	SP	N-signal-free	MTS	SP	N-signal-free	MTS	SP	N-signal-free
MTS	30	26	5	47	11	3	42	12	7
SP	9	50	2	13	43	5	13	45	3
N-signal-free	11	9	41	13	4	44	8	5	48

	$F_{CompFull}$			F_{Div} & F_{Phy}			F_{Div} & F_{Comp}		
Predicted →	MTS	SP	N-signal-free	MTS	SP	N-signal-free	MTS	SP	N-signal-free
MTS	34	14	13	41	16	4	51	10	0
SP	22	31	8	15	43	3	8	49	4
N-signal-free	17	14	30	4	5	52	5	4	52

	F_{Div} & $F_{CompFull}$			F_{Phy} & F_{Comp}			F_{Phy} & $F_{CompFull}$		
Predicted →	MTS	SP	N-signal-free	MTS	SP	N-signal-free	MTS	SP	N-signal-free
MTS	36	16	9	43	13	5	42	14	5
SP	13	44	4	14	44	3	16	41	4
N-signal-free	13	6	42	8	5	48	7	9	45

	F_{Comp} & $F_{CompFull}$			F_{Div} & F_{Phy} & F_{Comp}			F_{Div} & F_{Phy} & $F_{CompFull}$		
Predicted →	MTS	SP	N-signal-free	MTS	SP	N-signal-free	MTS	SP	N-signal-free
MTS	42	13	6	51	10	0	39	15	7
SP	9	46	6	8	49	4	17	40	4
N-signal-free	12	9	40	4	4	53	6	3	52

	F_{Phy} & F_{Comp} & $F_{CompFull}$			F_{Div} & F_{Comp} & $F_{CompFull}$			ALL		
Predicted →	MTS	SP	N-signal-free	MTS	SP	N-signal-free	MTS	SP	N-signal-free
MTS	42	14	5	48	11	2	46	12	3
SP	9	46	6	13	44	4	13	43	5
N-signal-free	11	9	41	4	8	49	4	6	51

Table S21: Confusion matrix of the 5-fold cross-validation of an SVM classifier, using various feature set combinations as listed above each column, is shown for three-way classification on the plant balanced dataset of automatically collected orthologs.

5 Divergence score combined with standard features in the N-terminal 20 residues

5.1 S. cerevisiae, curated orthologs (N_{20})

	F_{Div}			F_{Phy}			F_{Comp}		
Predicted →	MTS	SP	N-signal-free	MTS	SP	N-signal-free	MTS	SP	N-signal-free
MTS	83	0	96	149	1	29	140	2	37
SP	16	0	37	8	42	3	6	47	0
N-signal-free	50	0	400	34	2	414	20	4	426

	$F_{CompFull}$			F_{Div} & F_{Phy}			F_{Div} & F_{Comp}		
Predicted →	MTS	SP	N-signal-free	MTS	SP	N-signal-free	MTS	SP	N-signal-free
MTS	73	1	105	133	2	44	140	1	38
SP	4	19	30	9	42	2	8	44	1
N-signal-free	29	3	418	24	1	425	13	1	436

	F_{Div} & $F_{CompFull}$			F_{Phy} & F_{Comp}			F_{Phy} & $F_{CompFull}$		
Predicted →	MTS	SP	N-signal-free	MTS	SP	N-signal-free	MTS	SP	N-signal-free
MTS	120	1	58	142	2	35	146	0	33
SP	3	31	19	5	48	0	2	47	4
N-signal-free	28	7	415	21	4	425	30	3	417

	F_{Comp} & $F_{CompFull}$			F_{Div} & F_{Phy} & F_{Comp}			F_{Div} & F_{Phy} & $F_{CompFull}$		
Predicted →	MTS	SP	N-signal-free	MTS	SP	N-signal-free	MTS	SP	N-signal-free
MTS	146	1	32	141	1	37	145	0	34
SP	0	49	4	8	43	2	2	48	3
N-signal-free	20	3	427	13	1	436	25	1	424

	F_{Phy} & F_{Comp} & $F_{CompFull}$			F_{Div} & F_{Comp} & $F_{CompFull}$			ALL		
Predicted →	MTS	SP	N-signal-free	MTS	SP	N-signal-free	MTS	SP	N-signal-free
MTS	148	1	30	144	0	35	144	0	35
SP	0	50	3	0	51	2	1	51	1
N-signal-free	20	4	426	17	1	432	15	1	434

Table S22: Confusion matrix of the 5-fold cross-validation of an SVM classifier, using various feature set combinations as listed above each column, is shown for three-way classification on the yeast curated ortholog dataset.

5.2 S. cerevisiae, RBH orthologs (N_{20})

	F_{Div}			F_{Phy}			F_{Comp}		
Predicted →	MTS	SP	N-signal-free	MTS	SP	N-signal-free	MTS	SP	N-signal-free
MTS	91	0	128	174	6	39	173	3	43
SP	18	0	55	7	64	2	3	69	1
N-signal-free	54	0	506	36	1	523	23	5	532

	$F_{CompFull}$			F_{Div} & F_{Phy}			F_{Div} & F_{Comp}		
Predicted →	MTS	SP	N-signal-free	MTS	SP	N-signal-free	MTS	SP	N-signal-free
MTS	82	1	136	165	5	49	171	2	46
SP	4	35	34	9	63	1	8	65	0
N-signal-free	29	1	530	25	3	532	15	2	543

	F_{Div} & $F_{CompFull}$			F_{Phy} & F_{Comp}			F_{Phy} & $F_{CompFull}$		
Predicted →	MTS	SP	N-signal-free	MTS	SP	N-signal-free	MTS	SP	N-signal-free
MTS	138	4	77	173	4	42	180	0	39
SP	6	48	19	5	68	0	4	66	3
N-signal-free	34	1	525	25	4	531	41	4	515

	F_{Comp} & $F_{CompFull}$			F_{Div} & F_{Phy} & F_{Comp}			F_{Div} & F_{Phy} & $F_{CompFull}$		
Predicted →	MTS	SP	N-signal-free	MTS	SP	N-signal-free	MTS	SP	N-signal-free
MTS	179	0	40	171	2	46	173	1	45
SP	3	68	2	8	65	0	4	67	2
N-signal-free	23	2	535	16	2	542	26	1	533

	F_{Phy} & F_{Comp} & $F_{CompFull}$			F_{Div} & F_{Comp} & $F_{CompFull}$			ALL		
Predicted →	MTS	SP	N-signal-free	MTS	SP	N-signal-free	MTS	SP	N-signal-free
MTS	179	0	40	180	2	37	180	2	37
SP	2	69	2	3	68	2	2	69	2
N-signal-free	23	1	536	17	3	540	19	1	540

Table S23: Confusion matrix of the 5-fold cross-validation of an SVM classifier, using various feature set combinations as listed above each column, is shown for three-way classification on the yeast automatically collected dataset.

5.3 Human, RBH orthologs (N_{20})

	F_{Div}			F_{Phy}			F_{Comp}		
Predicted →	MTS	SP	N-signal-free	MTS	SP	N-signal-free	MTS	SP	N-signal-free
MTS	3	35	43	54	14	13	54	18	9
SP	1	84	84	12	133	24	14	140	15
N-signal-free	2	67	346	12	9	394	7	8	400

	$F_{CompFull}$			F_{Div} & F_{Phy}			F_{Div} & F_{Comp}		
Predicted →	MTS	SP	N-signal-free	MTS	SP	N-signal-free	MTS	SP	N-signal-free
MTS	31	14	36	53	18	10	54	17	10
SP	6	102	61	8	143	18	12	144	13
N-signal-free	13	37	365	8	11	396	5	9	401

	F_{Div} & $F_{CompFull}$			F_{Phy} & F_{Comp}			F_{Phy} & $F_{CompFull}$		
Predicted →	MTS	SP	N-signal-free	MTS	SP	N-signal-free	MTS	SP	N-signal-free
MTS	40	14	27	57	16	8	55	13	13
SP	8	121	40	15	139	15	12	138	19
N-signal-free	11	34	370	8	8	399	13	11	391

	F_{Comp} & $F_{CompFull}$			F_{Div} & F_{Phy} & F_{Comp}			F_{Div} & F_{Phy} & $F_{CompFull}$		
Predicted →	MTS	SP	N-signal-free	MTS	SP	N-signal-free	MTS	SP	N-signal-free
MTS	52	21	8	57	17	7	58	15	8
SP	11	142	16	11	144	14	8	146	15
N-signal-free	5	10	400	4	8	403	12	11	392

	F_{Phy} & F_{Comp} & $F_{CompFull}$			F_{Div} & F_{Comp} & $F_{CompFull}$			ALL		
Predicted →	MTS	SP	N-signal-free	MTS	SP	N-signal-free	MTS	SP	N-signal-free
MTS	56	19	6	56	19	6	57	19	5
SP	13	140	16	9	147	13	12	143	14
N-signal-free	5	9	401	5	10	400	5	9	401

Table S24: Confusion matrix of the 5-fold cross-validation of an SVM classifier, using various feature set combinations as listed above each column, is shown for three-way classification on the mammal automatically collected dataset.

5.4 Plant model organisms, RBH orthologs (N_{20})

	F_{Div}				F_{Phy}				F_{Comp}			
Predicted →	MTS	SP	CTP	N-signal-free	MTS	SP	CTP	N-signal-free	MTS	SP	CTP	N-signal-free
MTS	17	0	35	9	52	0	8	1	49	0	6	6
SP	3	0	4	8	5	8	1	1	1	10	2	2
CTP	3	0	90	6	10	2	77	10	5	1	85	8
N-signal-free	6	0	21	70	5	2	14	76	5	1	9	82

	$F_{CompFull}$				F_{Div} & F_{Phy}				F_{Div} & F_{Comp}			
Predicted →	MTS	SP	CTP	N-signal-free	MTS	SP	CTP	N-signal-free	MTS	SP	CTP	N-signal-free
MTS	16	0	27	18	47	0	12	2	48	0	11	2
SP	0	6	1	8	6	5	4	0	3	9	3	0
CTP	11	1	67	20	6	1	88	4	3	1	91	4
N-signal-free	12	2	27	56	3	1	5	88	5	0	7	85

	F_{Div} & $F_{CompFull}$				F_{Phy} & F_{Comp}				F_{Phy} & $F_{CompFull}$			
Predicted →	MTS	SP	CTP	N-signal-free	MTS	SP	CTP	N-signal-free	MTS	SP	CTP	N-signal-free
MTS	21	0	30	10	49	0	6	6	50	0	9	2
SP	2	6	2	5	1	10	2	2	0	13	1	1
CTP	8	0	81	10	3	2	87	7	7	2	80	10
N-signal-free	8	0	14	75	5	1	10	81	5	1	12	79

	F_{Comp} & $F_{CompFull}$				F_{Div} & F_{Phy} & F_{Comp}				F_{Div} & F_{Phy} & $F_{CompFull}$			
Predicted →	MTS	SP	CTP	N-signal-free	MTS	SP	CTP	N-signal-free	MTS	SP	CTP	N-signal-free
MTS	46	1	7	7	49	0	9	3	45	0	13	3
SP	1	11	2	1	2	10	3	0	2	11	2	0
CTP	4	2	86	7	2	2	90	5	7	2	85	5
N-signal-free	5	1	10	81	4	0	7	86	2	1	7	87

	F_{Phy} & F_{Comp} & $F_{CompFull}$				F_{Div} & F_{Comp} & $F_{CompFull}$				ALL			
Predicted →	MTS	SP	CTP	N-signal-free	MTS	SP	CTP	N-signal-free	MTS	SP	CTP	N-signal-free
MTS	48	0	7	6	46	0	11	4	48	0	9	4
SP	2	11	1	1	3	11	1	0	3	11	1	0
CTP	3	3	87	6	4	1	88	6	3	1	89	6
N-signal-free	4	1	12	80	5	0	9	83	3	0	9	85

Table S25: Confusion matrix of the 5-fold cross-validation of an SVM classifier, using various feature set combinations as listed above each column, is shown for three-way classification on the plant automatically collected dataset.

References

1. Allwein EL, Schapire RE, Singer Y: **Reducing multiclass to binary: A unifying approach for margin classifiers**. *The Journal of Machine Learning Research* 2001, **1**:113–141.
