# Supplementary material for: Plus ça change – evolutionary sequence divergence predicts protein subcellular localization signals
Source: BMC Genomics. 2014 Jan 20;15:46. doi: 10.1186/1471-2164-15-46 (PMC3906766; doi:10.1186/1471-2164-15-46)
Supplement: Additional file 2 — MSA’s of proteins for which sequence divergence changes predicted localization signals. Contains links to ortholog multiple sequence alignments of each protein in Additional file 3: Table S1. [file 1471-2164-15-46-S2.zip › index.html]

List for MSA


# List for MSA whose prediction differ when using divergence

## Color definitions: MTS (red), SP (blue), Non-signal (brown)

## Percentage identity in each columns show in blue color.

## Daker blue shows higher identity.

- P40825|SYA (Ala1)- P13099|RL10- P32504|CBF3A- P53875|RM19- P32523|PRP19- P47123|MOG1- P53219|IMO32- P40957|MAD1- Q02792|XRN2(RAT1)- P38228|TCM62- P12687|RM02(MRP7)- P32324|EF2(EFT1)- Q12019|MDN1- Q01163|RT23(RSM23)- P53727|BUD17- P46672|G4P1(ARC1)- P16862|K6PF2(PFK2)- P09620|KEX1- P25044|PTP1- P32333|MOT1- P25039|EFGM(MEF1)- Q12428|PRPD(PDH1)- P10663|RT02(MRP2)- P41338|THIL(ERG10)- P25348|RM32(MRPL32)- Q03691|ROT1- P39927|PTI1- Q12031|ACEB(ICL2)- P40008|FMP52- P28007|GAR1- P32898|CYM1- P00958|SYMC(MES1)- P39735|SAW1- P36046|MIA40- P35189|TAF14- P40018|RSMB(SMB1)- P43605|ECO1- P61830|H3(HHT[12])- P41805|RL10- P00447|SODM(SOD2)- P36517|RM04(MRPL4)- P38719|DBP8- P08524|FPPS(ERG20)
