# Supplementary material for: Plus ça change – evolutionary sequence divergence predicts protein subcellular localization signals
Source: BMC Genomics. 2014 Jan 20;15:46. doi: 10.1186/1471-2164-15-46 (PMC3906766; doi:10.1186/1471-2164-15-46)
Supplement: Additional file 2 — MSA’s of proteins for which sequence divergence changes predicted localization signals. Contains links to ortholog multiple sequence alignments of each protein in Additional file 3: Table S1. [file 1471-2164-15-46-S2.zip › P00447.html]

|  |  |  |  |  |  |  |  |  |  |  |  |  |  |  |  |  |  |  |  |  |  |  |  |  |  |  |  |  |  |  |  |  |  |  |  |  |  |  |  |  |  |  |  |  |  |  |  |  |  |  |  |  |  |  |  |  |  |  |  |  |  |  |  |  |  |  |  |  |  |  |  |  |  |  |  |  |  |  |  |  |  |  |  |  |  |  |  |  |  |  |  |  |  |  |  |  |  |  |  |  |  |  |  |  |  |  |  |  |  |  |  |  |  |  |  |  |  |  |  |  |  |  |  |  |  |  |  |  |  |  |  |  |  |  |  |  |  |  |  |  |  |  |  |  |  |  |  |  |  |  |  |  |  |  |  |  |  |  |  |  |  |  |  |  |  |  |  |  |  |  |  |  |  |  |  |  |  |  |  |  |  |  |  |  |  |  |  |  |  |  |  |  |  |  |  |  |  |  |  |  |  |  |  |  |  |  |  |  |  |  |  |  |  |  |  |  |  |  |  |  |  |  |  |  |  |  |  |  |  |  |  |  |  |  |  |  |  |  |  |  |  |  |  |  |  |  |  |  |  |  |  |  |  |  |  |  |  |  |  |  |  |  |  |  |  |  |  |  |  |  |  |  |  |  |  |  |  |  |  |  |  |  |  |  |  |  |  |  |  |  |  |  |  |  |  |  |  |  |  |  |  |  |  |  |  |  |  |  |  |  |  |  |  |  |  |  |  |  |  |  |  |  |  |  |  |  |  |  |  |  |  |  |  |  |  |  |  |  |  |  |  |  |  |  |  |  |  |  |  |  |  |  |  |  |  |  |  |  |  |  |  |  |  |  |  |  |  |  |  |  |  |  |  |  |  |  |  |  |  |  |  |  |  |  |  |  |  |  |  |  |  |  |  |  |  |  |  |  |  |  |  |  |  |  |  |  |  |  |  |  |  |  |  |  |  |  |  |  |  |  |  |  |  |  |  |  |  |  |  |  |  |  |  |  |  |  |  |  |  |  |  |  |  |  |  |  |  |  |  |  |  |  |  |  |  |  |  |  |  |  |  |  |  |  |  |  |  |  |  |  |  |  |  |  |  |  |  |  |  |  |  |  |  |  |  |  |  |  |  |  |  |  |  |  |  |  |  |  |  |  |  |  |  |  |  |  |  |  |  |  |  |  |  |  |  |  |  |  |  |  |  |  |  |  |  |  |  |  |  |  |  |  |  |  |  |  |  |  |  |  |  |  |  |  |  |  |  |  |  |  |  |  |  |  |  |  |  |  |  |  |  |  |  |  |  |  |  |  |  |  |  |  |  |  |  |  |  |  |  |  |  |  |  |  |  |  |  |  |  |  |  |  |  |  |  |  |  |  |  |  |  |  |  |  |  |  |  |  |  |  |  |  |  |  |  |  |  |  |  |  |  |  |  |  |  |  |  |  |  |  |  |  |  |  |  |  |  |  |  |  |  |  |  |  |  |  |  |  |  |  |  |  |  |  |  |  |  |  |  |  |  |  |  |  |  |  |  |  |  |  |  |  |  |  |  |  |  |  |  |  |  |  |  |  |  |  |  |  |  |  |  |  |  |  |  |  |  |  |  |  |  |  |  |  |  |  |  |  |  |  |  |  |  |  |  |  |  |  |  |  |  |  |  |  |  |  |  |  |  |  |  |  |  |  |  |  |  |  |  |  |  |  |  |  |  |  |  |  |  |  |  |  |  |  |  |  |  |  |  |  |  |  |  |  |  |  |  |  |  |  |  |  |  |  |  |  |  |  |  |  |  |  |  |  |  |  |  |  |  |  |  |  |  |  |  |  |  |  |  |  |  |  |  |  |  |  |  |  |  |  |  |  |  |  |  |  |  |  |  |  |  |  |  |  |  |  |  |  |  |  |  |  |  |  |  |  |  |  |  |  |  |  |  |  |  |  |  |  |  |  |  |  |  |  |  |  |  |  |  |  |  |  |  |  |  |  |  |  |  |  |  |  |  |  |  |  |  |  |  |  |  |  |  |  |  |  |  |  |  |  |  |  |  |  |  |  |  |  |  |  |  |  |  |  |  |  |  |  |  |  |  |  |  |  |  |  |  |  |  |  |  |  |  |  |  |  |  |  |  |  |  |  |  |  |  |  |  |  |  |  |  |  |  |  |  |  |  |  |  |  |  |  |  |  |  |  |  |  |  |  |  |  |  |  |  |  |  |  |  |  |  |  |  |  |  |  |  |  |  |  |  |  |  |  |  |  |  |  |  |  |  |  |  |  |  |  |  |  |  |  |  |  |  |  |  |  |  |  |  |  |  |  |  |  |  |  |  |  |  |  |  |  |  |  |  |  |  |  |  |  |  |  |  |  |  |  |  |  |  |  |  |  |  |  |  |  |  |  |  |  |  |  |  |  |  |  |  |  |  |  |  |  |  |  |  |  |  |  |  |  |  |  |  |  |  |  |  |  |  |  |  |  |  |  |  |  |  |  |  |  |  |  |  |  |  |  |  |  |  |  |  |  |  |  |  |  |  |  |  |  |  |  |  |  |  |  |  |  |  |  |  |  |  |  |  |  |  |  |  |  |  |  |  |  |  |  |  |  |  |  |  |  |  |  |  |  |  |  |  |  |  |  |  |  |  |  |  |  |  |  |  |  |  |  |  |  |  |  |  |  |  |  |  |  |  |  |  |  |  |  |  |  |  |  |  |  |  |  |  |  |  |  |  |  |  |  |  |  |  |  |  |  |  |  |  |  |  |  |  |  |  |  |  |  |  |  |  |  |  |  |  |  |  |  |  |  |  |  |  |  |  |  |  |  |  |  |  |  |  |  |  |  |  |  |  |  |  |  |  |  |  |  |  |  |  |  |  |  |  |  |  |  |  |  |  |  |  |  |  |  |  |  |  |  |  |  |  |  |  |  |  |  |  |  |  |  |  |  |  |  |  |  |  |  |  |  |  |  |  |  |  |  |  |  |  |  |  |  |  |  |  |  |  |  |  |  |  |  |  |  |  |  |  |  |  |  |  |  |  |  |  |  |  |  |  |  |  |  |  |  |  |  |  |  |  |  |  |  |  |  |  |  |  |  |  |  |  |  |  |  |  |  |  |  |  |  |  |  |  |  |  |  |  |  |  |  |  |  |  |  |  |  |  |  |  |  |  |  |  |  |  |  |  |  |  |  |  |  |  |  |  |  |  |  |  |  |  |  |  |  |  |  |  |  |  |  |  |  |  |  |  |  |  |  |  |  |  |  |  |  |  |  |  |  |  |  |  |  |  |  |  |  |  |  |  |  |  |  |  |  |  |  |  |  |  |  |  |  |  |  |  |  |  |  |  |  |  |  |  |  |  |  |  |  |  |  |  |  |  |  |  |  |  |  |  |  |  |  |  |  |  |  |  |  |  |  |  |  |  |  |  |  |  |  |  |  |  |  |  |  |  |  |  |  |  |  |  |  |  |  |  |  |  |  |  |  |  |  |  |  |  |  |  |  |  |  |  |  |  |  |  |  |  |  |  |  |  |  |  |  |  |  |  |  |  |  |  |  |  |  |  |  |  |  |  |  |  |  |  |  |  |  |  |  |  |  |  |  |  |  |  |  |  |  |  |  |  |  |  |  |  |  |  |  |  |  |  |  |  |  |  |  |  |  |  |  |  |  |  |  |  |  |  |  |  |  |  |  |  |  |  |  |  |  |  |  |  |  |  |  |  |  |  |  |  |  |  |  |  |  |  |  |  |  |  |  |  |  |  |  |  |  |  |  |  |  |  |  |  |  |  |  |  |  |  |  |  |  |  |  |  |  |  |  |  |  |  |  |  |  |  |  |  |  |  |  |  |  |  |  |  |  |  |  |  |  |  |  |  |  |  |  |  |  |  |  |  |  |  |  |  |  |  |  |  |  |  |  |  |  |  |  |  |  |  |  |  |  |  |  |  |  |  |  |  |  |  |  |  |  |  |  |  |  |  |  |  |  |  |  |  |  |  |  |  |  |  |  |  |  |  |  |  |  |  |  |  |  |  |  |  |  |  |  |  |  |  |  |  |  |  |  |  |  |  |  |  |  |  |  |  |  |  |  |  |  |  |  |  |  |  |  |  |  |  |  |  |  |  |  |  |  |  |  |  |  |  |  |  |  |  |  |  |  |  |  |  |  |  |  |  |  |  |  |  |  |  |  |  |  |  |  |  |  |  |  |  |  |  |  |  |  |  |  |  |  |  |  |  |  |  |  |  |  |  |  |  |  |  |  |  |  |  |  |  |  |  |  |  |  |  |  |  |  |  |  |  |  |  |  |  |  |  |  |  |  |  |  |  |  |  |  |  |  |  |  |  |  |  |  |  |  |  |  |  |  |  |  |  |  |  |  |  |  |  |  |  |  |  |  |  |  |  |  |  |  |  |  |  |  |  |  |  |  |  |  |  |  |  |  |  |  |  |  |  |  |  |  |  |  |  |  |  |  |  |  |  |  |  |  |  |  |  |  |  |  |  |  |  |  |  |  |  |  |  |  |  |  |  |  |  |  |  |  |  |  |  |  |  |  |  |  |  |  |  |  |  |  |  |  |  |  |  |  |  |  |  |  |  |  |  |  |  |  |  |  |  |  |  |  |  |  |  |  |  |  |  |  |  |  |  |  |  |  |  |  |  |  |  |  |  |  |  |  |  |  |  |  |  |  |  |  |  |  |  |  |  |  |  |  |  |  |  |  |  |  |  |  |  |  |  |  |  |  |  |  |  |  |  |  |  |  |  |  |  |  |  |  |  |  |  |  |  |  |  |  |  |  |  |  |  |  |  |  |  |  |  |  |  |  |  |  |  |  |  |  |  |  |  |  |  |  |  |  |  |  |  |  |  |  |  |  |  |  |  |  |  |  |  |  |  |  |  |  |  |  |  |  |  |  |  |  |  |  |  |  |  |  |  |  |  |  |  |  |  |  |  |  |  |  |  |  |  |  |  |  |  |  |  |  |  |  |  |  |  |  |  |  |  |  |  |  |  |  |  |  |  |  |  |  |  |  |  |  |  |  |  |  |  |  |  |  |  |  |  |  |  |  |  |  |  |  |  |  |  |  |  |  |  |  |  |  |  |  |  |  |  |  |  |  |  |  |  |  |  |  |  |  |  |  |  |  |  |  |  |  |  |  |  |  |  |  |  |  |  |  |  |  |  |  |  |  |  |  |  |  |  |  |  |  |  |  |  |  |  |  |  |  |  |  |  |  |  |  |  |  |  |  |  |  |  |  |  |  |  |  |  |  |  |  |  |  |  |  |  |  |  |  |  |  |  |  |  |  |  |  |  |  |  |  |  |  |  |  |  |  |  |  |  |  |  |  |  |  |  |  |  |  |  |  |  |  |  |  |  |  |  |  |  |  |  |  |  |  |  |  |  |  |  |  |  |  |  |  |  |  |  |  |  |  |  |  |  |  |  |  |  |  |  |  |  |  |  |  |  |  |  |  |  |  |  |  |  |  |  |  |  |  |  |  |  |  |  |  |  |  |  |  |  |  |  |  |  |  |  |  |  |  |  |  |  |  |  |  |  |  |  |  |  |  |  |  |  |  |  |  |  |  |  |  |  |  |  |  |  |  |  |  |  |  |  |  |  |  |  |  |  |  |  |  |  |  |  |  |  |  |  |  |  |  |  |  |  |  |  |  |  |  |  |  |  |  |  |  |  |  |  |  |  |  |  |  |  |  |  |  |  |  |  |  |  |  |  |  |  |  |  |  |  |  |  |  |  |  |  |  |  |  |  |  |  |  |  |  |  |  |  |  |  |  |  |  |  |  |  |  |  |  |  |  |  |  |  |  |  |  |  |  |  |  |  |  |  |  |  |  |  |  |  |  |  |  |  |  |  |  |  |  |  |  |  |  |  |  |  |  |  |  |  |  |  |  |  |  |  |  |  |  |  |  |  |  |  |  |  |  |  |  |  |  |  |  |  |  |  |  |  |  |  |  |  |  |  |  |  |  |  |  |  |  |  |  |  |  |  |  |  |  |  |  |  |  |  |  |  |  |  |  |  |  |  |  |  |  |  |  |  |  |  |  |  |  |  |  |  |  |  |  |  |  |  |  |  |  |  |  |  |  |  |  |  |  |  |  |  |  |  |  |  |  |  |  |  |  |  |  |  |  |  |  |  |  |  |  |  |  |  |  |  |  |  |  |  |  |  |  |  |  |  |  |  |  |  |  |  |  |  |  |  |  |  |  |  |  |  |  |  |  |  |  |  |  |  |  |  |  |  |  |  |  |  |  |  |  |  |  |  |  |  |  |  |  |  |  |  |  |  |  |  |  |  |  |  |  |  |  |  |  |  |  |  |  |  |  |  |  |  |  |  |  |  |  |  |  |  |  |  |  |  |  |  |  |  |  |  |  |  |  |  |  |  |  |  |  |  |  |  |  |  |  |  |  |  |  |  |  |  |  |  |  |  |  |  |  |  |  |  |  |  |  |  |  |  |  |  |  |  |  |  |  |  |  |  |  |  |  |  |  |  |  |  |  |  |  |  |  |  |  |  |  |  |  |  |  |  |  |  |  |  |  |  |  |  |  |  |  |  |  |  |  |  |  |  |  |  |  |  |  |  |  |  |  |  |  |  |  |  |  |  |  |  |  |  |  |  |  |  |  |  |  |  |  |  |  |  |  |  |  |  |  |  |  |  |  |  |  |  |  |  |  |  |  |  |  |  |  |  |  |  |  |  |  |  |  |  |  |  |  |  |  |  |  |  |  |  |  |  |  |  |  |  |  |  |  |  |  |  |  |  |  |  |  |  |  |  |  |  |  |  |  |  |  |  |  |  |  |  |  |  |  |  |  |  |  |  |  |  |  |  |  |  |  |  |  |  |  |  |  |  |  |  |  |  |  |  |  |  |  |  |  |  |  |  |  |  |  |  |  |  |  |  |  |  |  |  |  |  |  |  |  |  |  |  |  |  |  |  |  |  |  |  |  |  |  |  |  |  |  |  |  |  |  |  |  |  |  |  |  |  |  |  |  |  |  |  |  |  |  |  |  |  |  |  |  |  |  |  |  |  |  |  |  |  |  |  |  |  |  |  |  |  |  |  |  |  |  |  |  |  |  |  |  |  |  |  |  |  |  |  |  |  |  |  |  |  |  |  |  |  |  |  |  |  |  |  |  |  |  |  |  |  |  |  |  |  |  |  |  |  |  |  |  |  |  |  |  |  |  |  |  |  |  |  |  |  |  |  |  |  |  |  |  |  |  |
| --- | --- | --- | --- | --- | --- | --- | --- | --- | --- | --- | --- | --- | --- | --- | --- | --- | --- | --- | --- | --- | --- | --- | --- | --- | --- | --- | --- | --- | --- | --- | --- | --- | --- | --- | --- | --- | --- | --- | --- | --- | --- | --- | --- | --- | --- | --- | --- | --- | --- | --- | --- | --- | --- | --- | --- | --- | --- | --- | --- | --- | --- | --- | --- | --- | --- | --- | --- | --- | --- | --- | --- | --- | --- | --- | --- | --- | --- | --- | --- | --- | --- | --- | --- | --- | --- | --- | --- | --- | --- | --- | --- | --- | --- | --- | --- | --- | --- | --- | --- | --- | --- | --- | --- | --- | --- | --- | --- | --- | --- | --- | --- | --- | --- | --- | --- | --- | --- | --- | --- | --- | --- | --- | --- | --- | --- | --- | --- | --- | --- | --- | --- | --- | --- | --- | --- | --- | --- | --- | --- | --- | --- | --- | --- | --- | --- | --- | --- | --- | --- | --- | --- | --- | --- | --- | --- | --- | --- | --- | --- | --- | --- | --- | --- | --- | --- | --- | --- | --- | --- | --- | --- | --- | --- | --- | --- | --- | --- | --- | --- | --- | --- | --- | --- | --- | --- | --- | --- | --- | --- | --- | --- | --- | --- | --- | --- | --- | --- | --- | --- | --- | --- | --- | --- | --- | --- | --- | --- | --- | --- | --- | --- | --- | --- | --- | --- | --- | --- | --- | --- | --- | --- | --- | --- | --- | --- | --- | --- | --- | --- | --- | --- | --- | --- | --- | --- | --- | --- | --- | --- | --- | --- | --- | --- | --- | --- | --- | --- | --- | --- | --- | --- | --- | --- | --- | --- | --- | --- | --- | --- | --- | --- | --- | --- | --- | --- | --- | --- | --- | --- | --- | --- | --- | --- | --- | --- | --- | --- | --- | --- | --- | --- | --- | --- | --- | --- | --- | --- | --- | --- | --- | --- | --- | --- | --- | --- | --- | --- | --- | --- | --- | --- | --- | --- | --- | --- | --- | --- | --- | --- | --- | --- | --- | --- | --- | --- | --- | --- | --- | --- | --- | --- | --- | --- | --- | --- | --- | --- | --- | --- | --- | --- | --- | --- | --- | --- | --- | --- | --- | --- | --- | --- | --- | --- | --- | --- | --- | --- | --- | --- | --- | --- | --- | --- | --- | --- | --- | --- | --- | --- | --- | --- | --- | --- | --- | --- | --- | --- | --- | --- | --- | --- | --- | --- | --- | --- | --- | --- | --- | --- | --- | --- | --- | --- | --- | --- | --- | --- | --- | --- | --- | --- | --- | --- | --- | --- | --- | --- | --- | --- | --- | --- | --- | --- | --- | --- | --- | --- | --- | --- | --- | --- | --- | --- | --- | --- | --- | --- | --- | --- | --- | --- | --- | --- | --- | --- | --- | --- | --- | --- | --- | --- | --- | --- | --- | --- | --- | --- | --- | --- | --- | --- | --- | --- | --- | --- | --- | --- | --- | --- | --- | --- | --- | --- | --- | --- | --- | --- | --- | --- | --- | --- | --- | --- | --- | --- | --- | --- | --- | --- | --- | --- | --- | --- | --- | --- | --- | --- | --- | --- | --- | --- | --- | --- | --- | --- | --- | --- | --- | --- | --- | --- | --- | --- | --- | --- | --- | --- | --- | --- | --- | --- | --- | --- | --- | --- | --- | --- | --- | --- | --- | --- | --- | --- | --- | --- | --- | --- | --- | --- | --- | --- | --- | --- | --- | --- | --- | --- | --- | --- | --- | --- | --- | --- | --- | --- | --- | --- | --- | --- | --- | --- | --- | --- | --- | --- | --- | --- | --- | --- | --- | --- | --- | --- | --- | --- | --- | --- | --- | --- | --- | --- | --- | --- | --- | --- | --- | --- | --- | --- | --- | --- | --- | --- | --- | --- | --- | --- | --- | --- | --- | --- | --- | --- | --- | --- | --- | --- | --- | --- | --- | --- | --- | --- | --- | --- | --- | --- | --- | --- | --- | --- | --- | --- | --- | --- | --- | --- | --- | --- | --- | --- | --- | --- | --- | --- | --- | --- | --- | --- | --- | --- | --- | --- | --- | --- | --- | --- | --- | --- | --- | --- | --- | --- | --- | --- | --- | --- | --- | --- | --- | --- | --- | --- | --- | --- | --- | --- | --- | --- | --- | --- | --- | --- | --- | --- | --- | --- | --- | --- | --- | --- | --- | --- | --- | --- | --- | --- | --- | --- | --- | --- | --- | --- | --- | --- | --- | --- | --- | --- | --- | --- | --- | --- | --- | --- | --- | --- | --- | --- | --- | --- | --- | --- | --- | --- | --- | --- | --- | --- | --- | --- | --- | --- | --- | --- | --- | --- | --- | --- | --- | --- | --- | --- | --- | --- | --- | --- | --- | --- | --- | --- | --- | --- | --- | --- | --- | --- | --- | --- | --- | --- | --- | --- | --- | --- | --- | --- | --- | --- | --- | --- | --- | --- | --- | --- | --- | --- | --- | --- | --- | --- | --- | --- | --- | --- | --- | --- | --- | --- | --- | --- | --- | --- | --- | --- | --- | --- | --- | --- | --- | --- | --- | --- | --- | --- | --- | --- | --- | --- | --- | --- | --- | --- | --- | --- | --- | --- | --- | --- | --- | --- | --- | --- | --- | --- | --- | --- | --- | --- | --- | --- | --- | --- | --- | --- | --- | --- | --- | --- | --- | --- | --- | --- | --- | --- | --- | --- | --- | --- | --- | --- | --- | --- | --- | --- | --- | --- | --- | --- | --- | --- | --- | --- | --- | --- | --- | --- | --- | --- | --- | --- | --- | --- | --- | --- | --- | --- | --- | --- | --- | --- | --- | --- | --- | --- | --- | --- | --- | --- | --- | --- | --- | --- | --- | --- | --- | --- | --- | --- | --- | --- | --- | --- | --- | --- | --- | --- | --- | --- | --- | --- | --- | --- | --- | --- | --- | --- | --- | --- | --- | --- | --- | --- | --- | --- | --- | --- | --- | --- | --- | --- | --- | --- | --- | --- | --- | --- | --- | --- | --- | --- | --- | --- | --- | --- | --- | --- | --- | --- | --- | --- | --- | --- | --- | --- | --- | --- | --- | --- | --- | --- | --- | --- | --- | --- | --- | --- | --- | --- | --- | --- | --- | --- | --- | --- | --- | --- | --- | --- | --- | --- | --- | --- | --- | --- | --- | --- | --- | --- | --- | --- | --- | --- | --- | --- | --- | --- | --- | --- | --- | --- | --- | --- | --- | --- | --- | --- | --- | --- | --- | --- | --- | --- | --- | --- | --- | --- | --- | --- | --- | --- | --- | --- | --- | --- | --- | --- | --- | --- | --- | --- | --- | --- | --- | --- | --- | --- | --- | --- | --- | --- | --- | --- | --- | --- | --- | --- | --- | --- | --- | --- | --- | --- | --- | --- | --- | --- | --- | --- | --- | --- | --- | --- | --- | --- | --- | --- | --- | --- | --- | --- | --- | --- | --- | --- | --- | --- | --- | --- | --- | --- | --- | --- | --- | --- | --- | --- | --- | --- | --- | --- | --- | --- | --- | --- | --- | --- | --- | --- | --- | --- | --- | --- | --- | --- | --- | --- | --- | --- | --- | --- | --- | --- | --- | --- | --- | --- | --- | --- | --- | --- | --- | --- | --- | --- | --- | --- | --- | --- | --- | --- | --- | --- | --- | --- | --- | --- | --- | --- | --- | --- | --- | --- | --- | --- | --- | --- | --- | --- | --- | --- | --- | --- | --- | --- | --- | --- | --- | --- | --- | --- | --- | --- | --- | --- | --- | --- | --- | --- | --- | --- | --- | --- | --- | --- | --- | --- | --- | --- | --- | --- | --- | --- | --- | --- | --- | --- | --- | --- | --- | --- | --- | --- | --- | --- | --- | --- | --- | --- | --- | --- | --- | --- | --- | --- | --- | --- | --- | --- | --- | --- | --- | --- | --- | --- | --- | --- | --- | --- | --- | --- | --- | --- | --- | --- | --- | --- | --- | --- | --- | --- | --- | --- | --- | --- | --- | --- | --- | --- | --- | --- | --- | --- | --- | --- | --- | --- | --- | --- | --- | --- | --- | --- | --- | --- | --- | --- | --- | --- | --- | --- | --- | --- | --- | --- | --- | --- | --- | --- | --- | --- | --- | --- | --- | --- | --- | --- | --- | --- | --- | --- | --- | --- | --- | --- | --- | --- | --- | --- | --- | --- | --- | --- | --- | --- | --- | --- | --- | --- | --- | --- | --- | --- | --- | --- | --- | --- | --- | --- | --- | --- | --- | --- | --- | --- | --- | --- | --- | --- | --- | --- | --- | --- | --- | --- | --- | --- | --- | --- | --- | --- | --- | --- | --- | --- | --- | --- | --- | --- | --- | --- | --- | --- | --- | --- | --- | --- | --- | --- | --- | --- | --- | --- | --- | --- | --- | --- | --- | --- | --- | --- | --- | --- | --- | --- | --- | --- | --- | --- | --- | --- | --- | --- | --- | --- | --- | --- | --- | --- | --- | --- | --- | --- | --- | --- | --- | --- | --- | --- | --- | --- | --- | --- | --- | --- | --- | --- | --- | --- | --- | --- | --- | --- | --- | --- | --- | --- | --- | --- | --- | --- | --- | --- | --- | --- | --- | --- | --- | --- | --- | --- | --- | --- | --- | --- | --- | --- | --- | --- | --- | --- | --- | --- | --- | --- | --- | --- | --- | --- | --- | --- | --- | --- | --- | --- | --- | --- | --- | --- | --- | --- | --- | --- | --- | --- | --- | --- | --- | --- | --- | --- | --- | --- | --- | --- | --- | --- | --- | --- | --- | --- | --- | --- | --- | --- | --- | --- | --- | --- | --- | --- | --- | --- | --- | --- | --- | --- | --- | --- | --- | --- | --- | --- | --- | --- | --- | --- | --- | --- | --- | --- | --- | --- | --- | --- | --- | --- | --- | --- | --- | --- | --- | --- | --- | --- | --- | --- | --- | --- | --- | --- | --- | --- | --- | --- | --- | --- | --- | --- | --- | --- | --- | --- | --- | --- | --- | --- | --- | --- | --- | --- | --- | --- | --- | --- | --- | --- | --- | --- | --- | --- | --- | --- | --- | --- | --- | --- | --- | --- | --- | --- | --- | --- | --- | --- | --- | --- | --- | --- | --- | --- | --- | --- | --- | --- | --- | --- | --- | --- | --- | --- | --- | --- | --- | --- | --- | --- | --- | --- | --- | --- | --- | --- | --- | --- | --- | --- | --- | --- | --- | --- | --- | --- | --- | --- | --- | --- | --- | --- | --- | --- | --- | --- | --- | --- | --- | --- | --- | --- | --- | --- | --- | --- | --- | --- | --- | --- | --- | --- | --- | --- | --- | --- | --- | --- | --- | --- | --- | --- | --- | --- | --- | --- | --- | --- | --- | --- | --- | --- | --- | --- | --- | --- | --- | --- | --- | --- | --- | --- | --- | --- | --- | --- | --- | --- | --- | --- | --- | --- | --- | --- | --- | --- | --- | --- | --- | --- | --- | --- | --- | --- | --- | --- | --- | --- | --- | --- | --- | --- | --- | --- | --- | --- | --- | --- | --- | --- | --- | --- | --- | --- | --- | --- | --- | --- | --- | --- | --- | --- | --- | --- | --- | --- | --- | --- | --- | --- | --- | --- | --- | --- | --- | --- | --- | --- | --- | --- | --- | --- | --- | --- | --- | --- | --- | --- | --- | --- | --- | --- | --- | --- | --- | --- | --- | --- | --- | --- | --- | --- | --- | --- | --- | --- | --- | --- | --- | --- | --- | --- | --- | --- | --- | --- | --- | --- | --- | --- | --- | --- | --- | --- | --- | --- | --- | --- | --- | --- | --- | --- | --- | --- | --- | --- | --- | --- | --- | --- | --- | --- | --- | --- | --- | --- | --- | --- | --- | --- | --- | --- | --- | --- | --- | --- | --- | --- | --- | --- | --- | --- | --- | --- | --- | --- | --- | --- | --- | --- | --- | --- | --- | --- | --- | --- | --- | --- | --- | --- | --- | --- | --- | --- | --- | --- | --- | --- | --- | --- | --- | --- | --- | --- | --- | --- | --- | --- | --- | --- | --- | --- | --- | --- | --- | --- | --- | --- | --- | --- | --- | --- | --- | --- | --- | --- | --- | --- | --- | --- | --- | --- | --- | --- | --- | --- | --- | --- | --- | --- | --- | --- | --- | --- | --- | --- | --- | --- | --- | --- | --- | --- | --- | --- | --- | --- | --- | --- | --- | --- | --- | --- | --- | --- | --- | --- | --- | --- | --- | --- | --- | --- | --- | --- | --- | --- | --- | --- | --- | --- | --- | --- | --- | --- | --- | --- | --- | --- | --- | --- | --- | --- | --- | --- | --- | --- | --- | --- | --- | --- | --- | --- | --- | --- | --- | --- | --- | --- | --- | --- | --- | --- | --- | --- | --- | --- | --- | --- | --- | --- | --- | --- | --- | --- | --- | --- | --- | --- | --- | --- | --- | --- | --- | --- | --- | --- | --- | --- | --- | --- | --- | --- | --- | --- | --- | --- | --- | --- | --- | --- | --- | --- | --- | --- | --- | --- | --- | --- | --- | --- | --- | --- | --- | --- | --- | --- | --- | --- | --- | --- | --- | --- | --- | --- | --- | --- | --- | --- | --- | --- | --- | --- | --- | --- | --- | --- | --- | --- | --- | --- | --- | --- | --- | --- | --- | --- | --- | --- | --- | --- | --- | --- | --- | --- | --- | --- | --- | --- | --- | --- | --- | --- | --- | --- | --- | --- | --- | --- | --- | --- | --- | --- | --- | --- | --- | --- | --- | --- | --- | --- | --- | --- | --- | --- | --- | --- | --- | --- | --- | --- | --- | --- | --- | --- | --- | --- | --- | --- | --- | --- | --- | --- | --- | --- | --- | --- | --- | --- | --- | --- | --- | --- | --- | --- | --- | --- | --- | --- | --- | --- | --- | --- | --- | --- | --- | --- | --- | --- | --- | --- | --- | --- | --- | --- | --- | --- | --- | --- | --- | --- | --- | --- | --- | --- | --- | --- | --- | --- | --- | --- | --- | --- | --- | --- | --- | --- | --- | --- | --- | --- | --- | --- | --- | --- | --- | --- | --- | --- | --- | --- | --- | --- | --- | --- | --- | --- | --- | --- | --- | --- | --- | --- | --- | --- | --- | --- | --- | --- | --- | --- | --- | --- | --- | --- | --- | --- | --- | --- | --- | --- | --- | --- | --- | --- | --- | --- | --- | --- | --- | --- | --- | --- | --- | --- | --- | --- | --- | --- | --- | --- | --- | --- | --- | --- | --- | --- | --- | --- | --- | --- | --- | --- | --- | --- | --- | --- | --- | --- | --- | --- | --- | --- | --- | --- | --- | --- | --- | --- | --- | --- | --- | --- | --- | --- | --- | --- | --- | --- | --- | --- | --- | --- | --- | --- | --- | --- | --- | --- | --- | --- | --- | --- | --- | --- | --- | --- | --- | --- | --- | --- | --- | --- | --- | --- | --- | --- | --- | --- | --- | --- | --- | --- | --- | --- | --- | --- | --- | --- | --- | --- | --- | --- | --- | --- | --- | --- | --- | --- | --- | --- | --- | --- | --- | --- | --- | --- | --- | --- | --- | --- | --- | --- | --- | --- | --- | --- | --- | --- | --- | --- | --- | --- | --- | --- | --- | --- | --- | --- | --- | --- | --- | --- | --- | --- | --- | --- | --- | --- | --- | --- | --- | --- | --- | --- | --- | --- | --- | --- | --- | --- | --- | --- | --- | --- | --- | --- | --- | --- | --- | --- | --- | --- | --- | --- | --- | --- | --- | --- | --- | --- | --- | --- | --- | --- | --- | --- | --- | --- | --- | --- | --- | --- | --- | --- | --- | --- | --- | --- | --- | --- | --- | --- | --- | --- | --- | --- | --- | --- | --- | --- | --- | --- | --- | --- | --- | --- | --- | --- | --- | --- | --- | --- | --- | --- | --- | --- | --- | --- | --- | --- | --- | --- | --- | --- | --- | --- | --- | --- | --- | --- | --- | --- | --- | --- | --- | --- | --- | --- | --- | --- | --- | --- | --- | --- | --- | --- | --- | --- | --- | --- | --- | --- | --- | --- | --- | --- | --- | --- | --- | --- | --- | --- | --- | --- | --- | --- | --- | --- | --- | --- | --- | --- | --- | --- | --- | --- | --- | --- | --- | --- | --- | --- | --- | --- | --- | --- | --- | --- | --- | --- | --- | --- | --- | --- | --- | --- | --- | --- | --- | --- | --- | --- | --- | --- | --- | --- | --- | --- | --- | --- | --- | --- | --- | --- | --- | --- | --- | --- | --- | --- | --- | --- | --- | --- | --- | --- | --- | --- | --- | --- | --- | --- | --- | --- | --- | --- | --- | --- | --- | --- | --- | --- | --- | --- | --- | --- | --- | --- | --- | --- | --- | --- | --- | --- | --- | --- | --- | --- | --- | --- | --- | --- | --- | --- | --- | --- | --- | --- | --- | --- | --- | --- | --- | --- | --- | --- | --- | --- | --- | --- | --- | --- | --- | --- | --- | --- | --- | --- | --- | --- | --- | --- | --- | --- | --- | --- | --- | --- | --- | --- | --- | --- | --- | --- | --- | --- | --- | --- | --- | --- | --- | --- | --- | --- | --- | --- | --- | --- | --- | --- | --- | --- | --- | --- | --- | --- | --- | --- | --- | --- | --- | --- | --- | --- | --- | --- | --- | --- | --- | --- | --- | --- | --- | --- | --- | --- | --- | --- | --- | --- | --- | --- | --- | --- | --- | --- | --- | --- | --- | --- | --- | --- | --- | --- | --- | --- | --- | --- | --- | --- | --- | --- | --- | --- | --- | --- | --- | --- | --- | --- | --- | --- | --- | --- | --- | --- | --- | --- | --- | --- | --- | --- | --- | --- | --- | --- | --- | --- | --- | --- | --- | --- | --- | --- | --- | --- | --- | --- | --- | --- | --- | --- | --- | --- | --- | --- | --- | --- | --- | --- | --- | --- | --- | --- | --- | --- | --- | --- | --- | --- | --- | --- | --- | --- | --- | --- | --- | --- | --- | --- | --- | --- | --- | --- | --- | --- | --- | --- | --- | --- | --- | --- | --- | --- | --- | --- | --- | --- | --- | --- | --- | --- | --- | --- | --- | --- | --- | --- | --- | --- | --- | --- | --- | --- | --- | --- | --- | --- | --- | --- | --- | --- | --- | --- | --- | --- | --- | --- | --- | --- | --- | --- | --- | --- | --- | --- | --- | --- | --- | --- | --- | --- | --- | --- | --- | --- | --- | --- | --- | --- | --- | --- | --- | --- | --- | --- | --- | --- | --- | --- | --- | --- | --- | --- | --- | --- | --- | --- | --- | --- | --- | --- | --- | --- | --- | --- | --- | --- | --- | --- | --- | --- | --- | --- | --- | --- | --- | --- | --- | --- | --- | --- | --- | --- | --- | --- | --- | --- | --- | --- | --- | --- | --- | --- | --- | --- | --- | --- | --- | --- | --- | --- | --- | --- | --- | --- | --- | --- | --- | --- | --- | --- | --- | --- | --- | --- | --- | --- | --- | --- | --- | --- | --- | --- | --- | --- | --- | --- | --- | --- | --- | --- | --- | --- | --- | --- | --- | --- | --- | --- | --- | --- | --- | --- | --- | --- | --- | --- | --- | --- | --- | --- | --- | --- | --- | --- | --- | --- | --- | --- | --- | --- | --- | --- | --- | --- | --- | --- | --- | --- | --- | --- | --- | --- | --- | --- | --- | --- | --- | --- | --- | --- | --- | --- | --- | --- | --- | --- | --- | --- | --- | --- | --- | --- | --- | --- | --- | --- | --- | --- | --- | --- | --- | --- | --- | --- | --- | --- | --- | --- | --- | --- | --- | --- | --- | --- | --- | --- | --- | --- | --- | --- | --- | --- | --- | --- | --- | --- | --- | --- | --- | --- | --- | --- | --- | --- | --- | --- | --- | --- | --- | --- | --- | --- | --- | --- | --- | --- | --- | --- | --- | --- | --- | --- | --- | --- | --- | --- | --- | --- | --- | --- | --- | --- | --- | --- | --- | --- | --- | --- | --- | --- | --- | --- | --- | --- | --- | --- | --- | --- | --- | --- | --- | --- | --- | --- | --- | --- | --- | --- | --- | --- | --- | --- | --- | --- | --- | --- | --- | --- | --- | --- | --- | --- | --- | --- | --- | --- | --- | --- | --- | --- | --- | --- | --- | --- | --- | --- | --- | --- | --- | --- | --- | --- | --- | --- | --- | --- | --- | --- | --- | --- | --- | --- | --- | --- | --- | --- | --- | --- | --- | --- | --- | --- | --- | --- | --- | --- | --- | --- | --- | --- | --- | --- | --- | --- | --- | --- | --- | --- | --- | --- | --- | --- | --- | --- | --- | --- | --- | --- | --- | --- | --- | --- | --- | --- | --- | --- | --- | --- | --- | --- | --- | --- | --- | --- | --- | --- | --- | --- | --- | --- | --- | --- | --- | --- | --- | --- | --- | --- | --- | --- | --- | --- | --- | --- | --- | --- | --- | --- | --- | --- | --- | --- | --- | --- | --- | --- | --- | --- | --- | --- | --- | --- | --- | --- | --- | --- | --- | --- | --- | --- | --- | --- | --- | --- | --- | --- | --- | --- | --- | --- | --- | --- | --- | --- | --- | --- | --- | --- | --- | --- | --- | --- | --- | --- | --- |
| |  |  |  |  |  |  |  |  |  |  |  |  |  |  |  |  |  |  |  |  |  |  |  |  |  |  |  |  |  |  |  |  |  |  |  |  |  |  |  |  |  |  |  |  |  |  |  |  |  |  |  |  |  |  |  |  |  |  | | --- | --- | --- | --- | --- | --- | --- | --- | --- | --- | --- | --- | --- | --- | --- | --- | --- | --- | --- | --- | --- | --- | --- | --- | --- | --- | --- | --- | --- | --- | --- | --- | --- | --- | --- | --- | --- | --- | --- | --- | --- | --- | --- | --- | --- | --- | --- | --- | --- | --- | --- | --- | --- | --- | --- | --- | --- | --- | | G0VHU8/1-233 | 1 | M | L | - | L | R | S | G | T | A | T | L | R | Q | P | T | T | L | G | K | L | L | S | T | S | A | R | R | T | K | V | T | L | P | D | L | D | W | D | F | G | D | L | E | P | H | I | S | G | Q | I | N | E | L | H | Y | 54 | | Q6CPN2/1-226 | 1 | M | F | - | A | K | S | V | - | - | - | A | R | R | A | - | - | L | Q | L | Q | P | T | L | L | A | K | R | T | K | V | S | L | P | E | L | D | W | D | F | G | A | L | E | P | H | I | S | G | Q | I | N | E | L | H | Y | 49 | | Q6FV67/1-236 | 1 | M | L | S | T | S | R | I | - | - | - | A | F | K | S | T | - | Q | L | R | A | A | S | T | V | L | R | R | T | K | V | T | L | P | E | L | E | W | D | F | G | A | L | E | P | H | I | S | G | Q | I | N | E | L | H | Y | 51 | | Q759W2/1-227 | 1 | M | Y | A | A | R | T | A | - | - | - | L | R | K | S | G | - | S | A | L | S | A | A | A | Y | A | K | R | T | K | V | T | L | P | D | L | D | W | D | F | G | A | L | E | P | H | I | S | G | K | I | N | E | L | H | Y | 51 | | A7TJB7/1-227 | 1 | M | F | - | A | R | S | V | G | K | N | V | L | N | R | - | - | - | - | - | - | - | S | S | V | F | K | R | T | K | V | T | L | P | K | L | E | W | D | F | A | A | L | E | P | F | I | S | G | K | I | N | E | L | H | Y | 47 | | C5DM16/1-228 | 1 | M | F | - | S | R | R | I | - | - | - | V | Q | K | A | - | - | - | P | G | F | A | A | V | L | G | R | R | T | K | V | T | L | P | D | L | D | W | D | F | G | A | L | E | P | H | I | S | G | Q | I | N | E | L | H | Y | 48 | | C5DTA2/1-226 | 1 | M | F | - | A | R | N | V | S | A | T | L | R | Q | T | - | - | - | - | - | - | P | R | F | I | T | K | R | T | K | V | T | L | P | E | L | D | W | D | F | G | A | L | E | P | H | I | S | G | Q | I | N | E | L | H | Y | 48 | | Kwal\_47.18314/1-227 | 1 | M | L | - | A | R | S | V | - | - | - | A | K | K | S | - | - | - | A | V | F | G | T | V | L | A | R | R | T | K | V | T | L | P | D | L | D | W | D | F | G | A | L | E | P | H | I | S | G | Q | I | N | E | L | H | Y | 48 | | Sbay\_668.12/1-233 | 1 | M | F | - | A | K | T | A | A | V | N | L | T | K | K | G | - | G | L | S | L | L | S | T | T | A | R | R | T | K | V | T | L | P | D | L | K | W | D | F | G | A | L | E | P | Y | I | S | G | Q | I | N | E | L | H | Y | 53 | | SAKL0G06380g/1-226 | 1 | M | Y | A | A | R | T | A | - | - | - | L | R | R | T | - | - | A | A | P | A | A | S | F | I | T | K | R | T | K | V | T | L | P | D | L | D | W | D | F | G | D | L | E | P | H | I | S | G | Q | I | N | E | L | H | Y | 50 | | P00447/1-233 | 1 | M | F | - | A | K | T | A | A | A | N | L | T | K | K | G | - | G | L | S | L | L | S | T | T | A | R | R | T | K | V | T | L | P | D | L | K | W | D | F | G | A | L | E | P | Y | I | S | G | Q | I | N | E | L | H | Y | 53 | |  | | G0VHU8/1-233 | 55 | T | K | H | H | Q | T | Y | V | N | G | Y | N | A | A | V | E | Q | F | E | D | L | K | P | R | L | D | T | N | P | - | - | - | V | E | I | S | Q | K | L | I | A | L | Q | Q | N | I | K | F | H | G | G | G | F | T | N | 106 | | Q6CPN2/1-226 | 50 | T | K | H | H | Q | T | Y | V | N | G | F | N | A | A | V | E | Q | F | D | E | L | K | S | K | L | D | N | D | P | - | - | - | - | A | V | A | K | Q | I | V | A | V | Q | Q | N | L | K | F | H | G | G | G | Y | V | N | 100 | | Q6FV67/1-236 | 52 | S | K | H | H | Q | T | Y | V | N | G | L | N | T | A | V | D | Q | F | Q | E | L | T | H | K | L | S | K | D | P | N | - | D | L | Q | A | A | R | D | L | I | Q | V | Q | Q | N | I | K | F | H | G | G | G | Y | T | N | 105 | | Q759W2/1-227 | 52 | T | K | H | H | Q | T | Y | V | N | G | L | N | S | A | L | E | Q | F | H | E | L | S | A | R | V | D | Q | D | S | - | - | - | - | R | V | A | A | Q | L | T | A | L | Q | Q | N | I | K | F | H | G | G | G | Y | R | N | 102 | | A7TJB7/1-227 | 48 | S | K | H | H | Q | T | Y | V | N | G | F | N | T | A | T | E | Q | M | Q | E | L | Q | L | Q | L | S | K | Q | P | E | - | N | L | S | I | A | S | K | I | I | G | V | Q | Q | N | I | K | F | H | G | G | G | F | K | N | 101 | | C5DM16/1-228 | 49 | S | K | H | H | Q | T | Y | V | N | G | F | N | A | A | T | E | Q | F | G | E | L | T | E | K | L | T | G | D | A | Q | Q | D | A | P | L | A | R | K | L | A | A | L | Q | Q | N | I | K | F | H | G | G | G | F | A | N | 103 | | C5DTA2/1-226 | 49 | T | K | H | H | Q | T | Y | V | N | G | F | N | T | A | L | D | Q | L | A | E | L | T | S | S | I | E | K | D | P | - | - | N | P | K | T | A | K | K | I | L | G | V | Q | Q | N | L | K | F | H | G | G | G | Y | T | N | 101 | | Kwal\_47.18314/1-227 | 49 | T | K | H | H | Q | T | Y | V | N | G | F | N | A | A | S | E | Q | F | G | E | L | K | C | K | L | T | G | D | P | K | A | D | V | A | V | V | R | Q | L | V | A | L | Q | Q | N | I | K | F | H | G | G | G | F | S | N | 103 | | Sbay\_668.12/1-233 | 54 | T | K | H | H | Q | T | Y | V | N | G | F | N | T | A | V | E | Q | F | H | E | L | S | N | L | L | A | K | E | P | - | - | S | P | A | N | A | R | K | M | I | A | I | Q | Q | N | I | K | F | H | G | G | G | F | T | N | 106 | | SAKL0G06380g/1-226 | 51 | S | K | H | H | Q | T | Y | V | N | G | L | N | A | A | T | E | Q | F | Q | E | L | N | Q | K | L | A | S | D | P | - | - | - | - | T | V | A | T | K | L | I | A | L | Q | Q | N | I | K | F | H | G | G | G | F | T | N | 101 | | P00447/1-233 | 54 | T | K | H | H | Q | T | Y | V | N | G | F | N | T | A | V | D | Q | F | Q | E | L | S | D | L | L | A | K | E | P | - | - | S | P | A | N | A | R | K | M | I | A | I | Q | Q | N | I | K | F | H | G | G | G | F | T | N | 106 | |  | | G0VHU8/1-233 | 107 | H | C | L | F | W | K | N | L | A | P | E | K | Q | G | G | G | E | P | P | - | - | S | G | S | S | A | L | G | K | Q | I | Q | E | Q | Y | G | S | L | D | N | L | I | K | L | T | N | E | K | L | A | G | V | Q | G | S | 159 | | Q6CPN2/1-226 | 101 | H | C | L | F | W | K | N | L | A | P | T | S | Q | G | G | G | E | A | P | - | - | T | G | - | - | A | L | A | K | Q | I | E | T | Q | F | G | S | L | D | N | L | I | S | L | T | N | A | K | L | A | G | V | Q | G | S | 151 | | Q6FV67/1-236 | 106 | H | C | L | F | W | K | N | L | A | P | E | K | N | G | G | G | E | A | P | S | S | S | S | - | - | A | L | G | Q | Q | I | E | K | Q | Y | G | S | L | D | K | L | I | E | V | T | N | A | K | L | A | G | V | Q | G | S | 158 | | Q759W2/1-227 | 103 | H | C | L | F | W | K | N | L | A | P | A | S | Q | G | G | G | E | P | P | - | - | T | G | - | - | A | L | A | R | Q | I | E | T | Q | F | G | S | L | E | K | L | Q | A | L | T | N | G | K | L | A | G | I | Q | G | S | 153 | | A7TJB7/1-227 | 102 | H | C | L | F | W | A | N | L | S | P | Q | S | A | V | G | G | Q | P | P | - | - | T | G | - | - | A | L | A | A | Q | I | E | K | Q | Y | G | S | L | E | N | L | V | K | I | S | N | D | K | L | L | G | I | Q | G | S | 152 | | C5DM16/1-228 | 104 | H | C | L | F | W | K | N | L | A | P | E | S | Q | G | G | G | E | A | P | - | - | A | G | - | - | A | L | G | R | Q | I | E | S | Q | F | G | S | L | D | K | L | Q | A | L | T | N | A | K | L | A | G | V | Q | G | S | 154 | | C5DTA2/1-226 | 102 | H | C | L | F | W | K | N | L | A | P | A | S | Q | G | G | G | E | P | P | - | - | T | G | - | - | A | L | A | E | Q | I | K | Q | Q | Y | G | S | L | E | K | L | Q | K | V | T | N | A | S | L | A | G | V | Q | G | S | 152 | | Kwal\_47.18314/1-227 | 104 | H | C | L | F | W | K | N | L | A | P | T | S | Q | G | G | G | E | A | P | - | - | T | G | - | - | A | L | G | K | Q | I | E | S | Q | F | G | S | L | D | K | L | I | E | Q | T | N | A | K | L | A | G | V | Q | G | S | 154 | | Sbay\_668.12/1-233 | 107 | H | C | L | F | W | E | N | L | A | P | E | S | Q | G | G | G | E | P | P | - | - | T | G | - | - | A | L | A | K | A | I | D | E | Q | F | G | S | L | D | E | L | I | K | L | T | N | T | K | L | A | G | V | Q | G | S | 157 | | SAKL0G06380g/1-226 | 102 | H | N | L | F | W | K | S | L | A | P | T | S | Q | G | G | G | E | P | P | - | - | T | G | - | - | A | L | G | D | Q | I | N | K | Q | F | G | S | L | D | N | L | I | A | L | T | N | T | K | L | A | G | V | Q | G | S | 152 | | P00447/1-233 | 107 | H | C | L | F | W | E | N | L | A | P | E | S | Q | G | G | G | E | P | P | - | - | T | G | - | - | A | L | A | K | A | I | D | E | Q | F | G | S | L | D | E | L | I | K | L | T | N | T | K | L | A | G | V | Q | G | S | 157 | |  | | G0VHU8/1-233 | 160 | G | W | A | F | I | V | K | N | V | S | N | G | G | K | L | D | V | V | Q | T | Y | N | Q | D | T | V | T | - | G | A | L | K | P | I | V | A | I | D | A | W | E | H | A | Y | Y | L | Q | Y | Q | N | Q | R | A | D | Y | 213 | | Q6CPN2/1-226 | 152 | G | W | A | F | I | V | K | N | V | E | N | G | N | Q | L | E | V | V | Q | T | Y | N | Q | D | T | V | T | - | G | P | L | K | P | L | V | A | I | D | S | W | E | H | A | Y | Y | L | Q | Y | Q | N | Q | K | A | N | Y | 205 | | Q6FV67/1-236 | 159 | G | W | A | F | I | V | K | N | L | E | N | G | G | Q | L | D | V | V | Q | T | Y | N | Q | D | T | V | G | - | N | Q | F | V | P | L | V | A | I | D | A | W | E | H | A | Y | Y | L | Q | Y | Q | N | K | K | V | D | Y | 212 | | Q759W2/1-227 | 154 | G | W | A | F | L | V | K | N | T | D | N | G | G | Q | L | E | L | V | Q | T | Y | N | Q | D | T | V | S | - | G | P | Y | V | P | L | L | A | I | D | A | W | E | H | A | Y | Y | L | Q | Y | Q | N | R | K | A | D | Y | 207 | | A7TJB7/1-227 | 153 | G | W | I | F | I | V | K | N | V | A | N | G | G | T | I | D | V | V | Q | T | Y | N | Q | D | T | I | T | D | G | N | I | I | P | L | V | A | V | D | A | W | E | H | A | Y | Y | L | Q | Y | E | N | R | K | G | E | Y | 207 | | C5DM16/1-228 | 155 | G | W | A | F | I | V | K | N | A | E | N | G | G | Q | I | E | V | V | Q | T | Y | N | Q | D | T | V | S | - | G | P | L | T | P | L | V | A | I | D | A | W | E | H | A | Y | Y | L | Q | Y | Q | N | R | K | A | D | Y | 208 | | C5DTA2/1-226 | 153 | G | W | S | F | I | V | K | D | L | D | N | G | G | K | L | D | V | V | Q | T | Y | N | Q | D | S | V | T | - | G | N | H | V | P | L | V | A | I | D | A | W | E | H | A | Y | Y | L | Q | Y | Q | N | R | K | A | E | Y | 206 | | Kwal\_47.18314/1-227 | 155 | G | W | A | F | I | V | K | N | A | E | N | G | G | Q | L | E | V | V | Q | R | Y | N | Q | D | T | V | T | - | G | P | L | K | P | I | V | A | I | D | A | W | E | H | A | Y | Y | L | Q | Y | Q | N | R | K | A | D | Y | 208 | | Sbay\_668.12/1-233 | 158 | G | W | A | F | I | V | K | N | L | S | N | G | G | K | L | D | V | V | Q | T | Y | N | Q | D | T | V | T | - | G | P | L | I | P | L | V | A | I | D | A | W | E | H | A | Y | Y | L | Q | Y | Q | N | K | K | V | D | Y | 211 | | SAKL0G06380g/1-226 | 153 | G | W | C | F | I | V | K | N | L | E | N | G | G | Q | L | E | V | V | Q | T | Y | N | Q | D | T | V | T | - | G | S | L | K | P | L | L | A | I | D | A | W | E | H | A | Y | Y | L | Q | Y | Q | N | K | K | V | D | Y | 206 | | P00447/1-233 | 158 | G | W | A | F | I | V | K | N | L | S | N | G | G | K | L | D | V | V | Q | T | Y | N | Q | D | T | V | T | - | G | P | L | V | P | L | V | A | I | D | A | W | E | H | A | Y | Y | L | Q | Y | Q | N | K | K | A | D | Y | 211 | |  | | G0VHU8/1-233 | 214 | F | K | A | I | W | N | V | V | N | W | K | E | A | S | R | R | F | D | K | A | - | - | - | - |  | | | | | | | | | | | | | | | | | | | | | | | | | | | | | | | 233 | | Q6CPN2/1-226 | 206 | F | K | A | I | W | N | V | I | N | W | K | E | A | A | K | R | F | E | A | A | A | - | - | - |  | | | | | | | | | | | | | | | | | | | | | | | | | | | | | | | 226 | | Q6FV67/1-236 | 213 | F | K | A | I | W | N | V | I | N | W | K | E | A | A | K | R | Y | E | T | S | K | V | T | K |  | | | | | | | | | | | | | | | | | | | | | | | | | | | | | | | 236 | | Q759W2/1-227 | 208 | F | S | A | I | W | N | V | I | N | W | K | E | A | A | R | R | F | D | A | A | - | - | - | - |  | | | | | | | | | | | | | | | | | | | | | | | | | | | | | | | 227 | | A7TJB7/1-227 | 208 | F | N | A | I | W | N | V | I | N | W | E | E | A | G | R | R | Y | D | S | A | - | - | - | - |  | | | | | | | | | | | | | | | | | | | | | | | | | | | | | | | 227 | | C5DM16/1-228 | 209 | F | K | A | I | W | N | V | I | N | W | K | E | A | E | R | R | F | D | A | A | - | - | - | - |  | | | | | | | | | | | | | | | | | | | | | | | | | | | | | | | 228 | | C5DTA2/1-226 | 207 | F | S | A | I | W | N | V | I | N | W | K | E | A | A | K | R | F | E | S | A | - | - | - | - |  | | | | | | | | | | | | | | | | | | | | | | | | | | | | | | | 226 | | Kwal\_47.18314/1-227 | 209 | F | K | A | I | W | N | V | I | N | W | K | E | A | E | R | R | F | E | A | - | - | - | - | - |  | | | | | | | | | | | | | | | | | | | | | | | | | | | | | | | 227 | | Sbay\_668.12/1-233 | 212 | F | K | A | I | W | N | V | V | N | W | K | E | A | S | R | R | F | D | A | E | K | I | - | - |  | | | | | | | | | | | | | | | | | | | | | | | | | | | | | | | 233 | | SAKL0G06380g/1-226 | 207 | F | K | A | I | W | N | V | I | N | W | K | E | A | A | R | K | F | E | A | A | - | - | - | - |  | | | | | | | | | | | | | | | | | | | | | | | | | | | | | | | 226 | | P00447/1-233 | 212 | F | K | A | I | W | N | V | V | N | W | K | E | A | S | R | R | F | D | A | G | K | I | - | - |  | | | | | | | | | | | | | | | | | | | | | | | | | | | | | | | 233 | |
