# Supplementary material for: Plus ça change – evolutionary sequence divergence predicts protein subcellular localization signals
Source: BMC Genomics. 2014 Jan 20;15:46. doi: 10.1186/1471-2164-15-46 (PMC3906766; doi:10.1186/1471-2164-15-46)
Supplement: Additional file 2 — MSA’s of proteins for which sequence divergence changes predicted localization signals. Contains links to ortholog multiple sequence alignments of each protein in Additional file 3: Table S1. [file 1471-2164-15-46-S2.zip › P08524.html]

|  |  |  |  |  |  |  |  |  |  |  |  |  |  |  |  |  |  |  |  |  |  |  |  |  |  |  |  |  |  |  |  |  |  |  |  |  |  |  |  |  |  |  |  |  |  |  |  |  |  |  |  |  |  |  |  |  |  |  |  |  |  |  |  |  |  |  |  |  |  |  |  |  |  |  |  |  |  |  |  |  |  |  |  |  |  |  |  |  |  |  |  |  |  |  |  |  |  |  |  |  |  |  |  |  |  |  |  |  |  |  |  |  |  |  |  |  |  |  |  |  |  |  |  |  |  |  |  |  |  |  |  |  |  |  |  |  |  |  |  |  |  |  |  |  |  |  |  |  |  |  |  |  |  |  |  |  |  |  |  |  |  |  |  |  |  |  |  |  |  |  |  |  |  |  |  |  |  |  |  |  |  |  |  |  |  |  |  |  |  |  |  |  |  |  |  |  |  |  |  |  |  |  |  |  |  |  |  |  |  |  |  |  |  |  |  |  |  |  |  |  |  |  |  |  |  |  |  |  |  |  |  |  |  |  |  |  |  |  |  |  |  |  |  |  |  |  |  |  |  |  |  |  |  |  |  |  |  |  |  |  |  |  |  |  |  |  |  |  |  |  |  |  |  |  |  |  |  |  |  |  |  |  |  |  |  |  |  |  |  |  |  |  |  |  |  |  |  |  |  |  |  |  |  |  |  |  |  |  |  |  |  |  |  |  |  |  |  |  |  |  |  |  |  |  |  |  |  |  |  |  |  |  |  |  |  |  |  |  |  |  |  |  |  |  |  |  |  |  |  |  |  |  |  |  |  |  |  |  |  |  |  |  |  |  |  |  |  |  |  |  |  |  |  |  |  |  |  |  |  |  |  |  |  |  |  |  |  |  |  |  |  |  |  |  |  |  |  |  |  |  |  |  |  |  |  |  |  |  |  |  |  |  |  |  |  |  |  |  |  |  |  |  |  |  |  |  |  |  |  |  |  |  |  |  |  |  |  |  |  |  |  |  |  |  |  |  |  |  |  |  |  |  |  |  |  |  |  |  |  |  |  |  |  |  |  |  |  |  |  |  |  |  |  |  |  |  |  |  |  |  |  |  |  |  |  |  |  |  |  |  |  |  |  |  |  |  |  |  |  |  |  |  |  |  |  |  |  |  |  |  |  |  |  |  |  |  |  |  |  |  |  |  |  |  |  |  |  |  |  |  |  |  |  |  |  |  |  |  |  |  |  |  |  |  |  |  |  |  |  |  |  |  |  |  |  |  |  |  |  |  |  |  |  |  |  |  |  |  |  |  |  |  |  |  |  |  |  |  |  |  |  |  |  |  |  |  |  |  |  |  |  |  |  |  |  |  |  |  |  |  |  |  |  |  |  |  |  |  |  |  |  |  |  |  |  |  |  |  |  |  |  |  |  |  |  |  |  |  |  |  |  |  |  |  |  |  |  |  |  |  |  |  |  |  |  |  |  |  |  |  |  |  |  |  |  |  |  |  |  |  |  |  |  |  |  |  |  |  |  |  |  |  |  |  |  |  |  |  |  |  |  |  |  |  |  |  |  |  |  |  |  |  |  |  |  |  |  |  |  |  |  |  |  |  |  |  |  |  |  |  |  |  |  |  |  |  |  |  |  |  |  |  |  |  |  |  |  |  |  |  |  |  |  |  |  |  |  |  |  |  |  |  |  |  |  |  |  |  |  |  |  |  |  |  |  |  |  |  |  |  |  |  |  |  |  |  |  |  |  |  |  |  |  |  |  |  |  |  |  |  |  |  |  |  |  |  |  |  |  |  |  |  |  |  |  |  |  |  |  |  |  |  |  |  |  |  |  |  |  |  |  |  |  |  |  |  |  |  |  |  |  |  |  |  |  |  |  |  |  |  |  |  |  |  |  |  |  |  |  |  |  |  |  |  |  |  |  |  |  |  |  |  |  |  |  |  |  |  |  |  |  |  |  |  |  |  |  |  |  |  |  |  |  |  |  |  |  |  |  |  |  |  |  |  |  |  |  |  |  |  |  |  |  |  |  |  |  |  |  |  |  |  |  |  |  |  |  |  |  |  |  |  |  |  |  |  |  |  |  |  |  |  |  |  |  |  |  |  |  |  |  |  |  |  |  |  |  |  |  |  |  |  |  |  |  |  |  |  |  |  |  |  |  |  |  |  |  |  |  |  |  |  |  |  |  |  |  |  |  |  |  |  |  |  |  |  |  |  |  |  |  |  |  |  |  |  |  |  |  |  |  |  |  |  |  |  |  |  |  |  |  |  |  |  |  |  |  |  |  |  |  |  |  |  |  |  |  |  |  |  |  |  |  |  |  |  |  |  |  |  |  |  |  |  |  |  |  |  |  |  |  |  |  |  |  |  |  |  |  |  |  |  |  |  |  |  |  |  |  |  |  |  |  |  |  |  |  |  |  |  |  |  |  |  |  |  |  |  |  |  |  |  |  |  |  |  |  |  |  |  |  |  |  |  |  |  |  |  |  |  |  |  |  |  |  |  |  |  |  |  |  |  |  |  |  |  |  |  |  |  |  |  |  |  |  |  |  |  |  |  |  |  |  |  |  |  |  |  |  |  |  |  |  |  |  |  |  |  |  |  |  |  |  |  |  |  |  |  |  |  |  |  |  |  |  |  |  |  |  |  |  |  |  |  |  |  |  |  |  |  |  |  |  |  |  |  |  |  |  |  |  |  |  |  |  |  |  |  |  |  |  |  |  |  |  |  |  |  |  |  |  |  |  |  |  |  |  |  |  |  |  |  |  |  |  |  |  |  |  |  |  |  |  |  |  |  |  |  |  |  |  |  |  |  |  |  |  |  |  |  |  |  |  |  |  |  |  |  |  |  |  |  |  |  |  |  |  |  |  |  |  |  |  |  |  |  |  |  |  |  |  |  |  |  |  |  |  |  |  |  |  |  |  |  |  |  |  |  |  |  |  |  |  |  |  |  |  |  |  |  |  |  |  |  |  |  |  |  |  |  |  |  |  |  |  |  |  |  |  |  |  |  |  |  |  |  |  |  |  |  |  |  |  |  |  |  |  |  |  |  |  |  |  |  |  |  |  |  |  |  |  |  |  |  |  |  |  |  |  |  |  |  |  |  |  |  |  |  |  |  |  |  |  |  |  |  |  |  |  |  |  |  |  |  |  |  |  |  |  |  |  |  |  |  |  |  |  |  |  |  |  |  |  |  |  |  |  |  |  |  |  |  |  |  |  |  |  |  |  |  |  |  |  |  |  |  |  |  |  |  |  |  |  |  |  |  |  |  |  |  |  |  |  |  |  |  |  |  |  |  |  |  |  |  |  |  |  |  |  |  |  |  |  |  |  |  |  |  |  |  |  |  |  |  |  |  |  |  |  |  |  |  |  |  |  |  |  |  |  |  |  |  |  |  |  |  |  |  |  |  |  |  |  |  |  |  |  |  |  |  |  |  |  |  |  |  |  |  |  |  |  |  |  |  |  |  |  |  |  |  |  |  |  |  |  |  |  |  |  |  |  |  |  |  |  |  |  |  |  |  |  |  |  |  |  |  |  |  |  |  |  |  |  |  |  |  |  |  |  |  |  |  |  |  |  |  |  |  |  |  |  |  |  |  |  |  |  |  |  |  |  |  |  |  |  |  |  |  |  |  |  |  |  |  |  |  |  |  |  |  |  |  |  |  |  |  |  |  |  |  |  |  |  |  |  |  |  |  |  |  |  |  |  |  |  |  |  |  |  |  |  |  |  |  |  |  |  |  |  |  |  |  |  |  |  |  |  |  |  |  |  |  |  |  |  |  |  |  |  |  |  |  |  |  |  |  |  |  |  |  |  |  |  |  |  |  |  |  |  |  |  |  |  |  |  |  |  |  |  |  |  |  |  |  |  |  |  |  |  |  |  |  |  |  |  |  |  |  |  |  |  |  |  |  |  |  |  |  |  |  |  |  |  |  |  |  |  |  |  |  |  |  |  |  |  |  |  |  |  |  |  |  |  |  |  |  |  |  |  |  |  |  |  |  |  |  |  |  |  |  |  |  |  |  |  |  |  |  |  |  |  |  |  |  |  |  |  |  |  |  |  |  |  |  |  |  |  |  |  |  |  |  |  |  |  |  |  |  |  |  |  |  |  |  |  |  |  |  |  |  |  |  |  |  |  |  |  |  |  |  |  |  |  |  |  |  |  |  |  |  |  |  |  |  |  |  |  |  |  |  |  |  |  |  |  |  |  |  |  |  |  |  |  |  |  |  |  |  |  |  |  |  |  |  |  |  |  |  |  |  |  |  |  |  |  |  |  |  |  |  |  |  |  |  |  |  |  |  |  |  |  |  |  |  |  |  |  |  |  |  |  |  |  |  |  |  |  |  |  |  |  |  |  |  |  |  |  |  |  |  |  |  |  |  |  |  |  |  |  |  |  |  |  |  |  |  |  |  |  |  |  |  |  |  |  |  |  |  |  |  |  |  |  |  |  |  |  |  |  |  |  |  |  |  |  |  |  |  |  |  |  |  |  |  |  |  |  |  |  |  |  |  |  |  |  |  |  |  |  |  |  |  |  |  |  |  |  |  |  |  |  |  |  |  |  |  |  |  |  |  |  |  |  |  |  |  |  |  |  |  |  |  |  |  |  |  |  |  |  |  |  |  |  |  |  |  |  |  |  |  |  |  |  |  |  |  |  |  |  |  |  |  |  |  |  |  |  |  |  |  |  |  |  |  |  |  |  |  |  |  |  |  |  |  |  |  |  |  |  |  |  |  |  |  |  |  |  |  |  |  |  |  |  |  |  |  |  |  |  |  |  |  |  |  |  |  |  |  |  |  |  |  |  |  |  |  |  |  |  |  |  |  |  |  |  |  |  |  |  |  |  |  |  |  |  |  |  |  |  |  |  |  |  |  |  |  |  |  |  |  |  |  |  |  |  |  |  |  |  |  |  |  |  |  |  |  |  |  |  |  |  |  |  |  |  |  |  |  |  |  |  |  |  |  |  |  |  |  |  |  |  |  |  |  |  |  |  |  |  |  |  |  |  |  |  |  |  |  |  |  |  |  |  |  |  |  |  |  |  |  |  |  |  |  |  |  |  |  |  |  |  |  |  |  |  |  |  |  |  |  |  |  |  |  |  |  |  |  |  |  |  |  |  |  |  |  |  |  |  |  |  |  |  |  |  |  |  |  |  |  |  |  |  |  |  |  |  |  |  |  |  |  |  |  |  |  |  |  |  |  |  |  |  |  |  |  |  |  |  |  |  |  |  |  |  |  |  |  |  |  |  |  |  |  |  |  |  |  |  |  |  |  |  |  |  |  |  |  |  |  |  |  |  |  |  |  |  |  |  |  |  |  |  |  |  |  |  |  |  |  |  |  |  |  |  |  |  |  |  |  |  |  |  |  |  |  |  |  |  |  |  |  |  |  |  |  |  |  |  |  |  |  |  |  |  |  |  |  |  |  |  |  |  |  |  |  |  |  |  |  |  |  |  |  |  |  |  |  |  |  |  |  |  |  |  |  |  |  |  |  |  |  |  |  |  |  |  |  |  |  |  |  |  |  |  |  |  |  |  |  |  |  |  |  |  |  |  |  |  |  |  |  |  |  |  |  |  |  |  |  |  |  |  |  |  |  |  |  |  |  |  |  |  |  |  |  |  |  |  |  |  |  |  |  |  |  |  |  |  |  |  |  |  |  |  |  |  |  |  |  |  |  |  |  |  |  |  |  |  |  |  |  |  |  |  |  |  |  |  |  |  |  |  |  |  |  |  |  |  |  |  |  |  |  |  |  |  |  |  |  |  |  |  |  |  |  |  |  |  |  |  |  |  |  |  |  |  |  |  |  |  |  |  |  |  |  |  |  |  |  |  |  |  |  |  |  |  |  |  |  |  |  |  |  |  |  |  |  |  |  |  |  |  |  |  |  |  |  |  |  |  |  |  |  |  |  |  |  |  |  |  |  |  |  |  |  |  |  |  |  |  |  |  |  |  |  |  |  |  |  |  |  |  |  |  |  |  |  |  |  |  |  |  |  |  |  |  |  |  |  |  |  |  |  |  |  |  |  |  |  |  |  |  |  |  |  |  |  |  |  |  |  |  |  |  |  |  |  |  |  |  |  |  |  |  |  |  |  |  |  |  |  |  |  |  |  |  |  |  |  |  |  |  |  |  |  |  |  |  |  |  |  |  |  |  |  |  |  |  |  |  |  |  |  |  |  |  |  |  |  |  |  |  |  |  |  |  |  |  |  |  |  |  |  |  |  |  |  |  |  |  |  |  |  |  |  |  |  |  |  |  |  |  |  |  |  |  |  |  |  |  |  |  |  |  |  |  |  |  |  |  |  |  |  |  |  |  |  |  |  |  |  |  |  |  |  |  |  |  |  |  |  |  |  |  |  |  |  |  |  |  |  |  |  |  |  |  |  |  |  |  |  |  |  |  |  |  |  |  |  |  |  |  |  |  |  |  |  |  |  |  |  |  |  |  |  |  |  |  |  |  |  |  |  |  |  |  |  |  |  |  |  |  |  |  |  |  |  |  |  |  |  |  |  |  |  |  |  |  |  |  |  |  |  |  |  |  |  |  |  |  |  |  |  |  |  |  |  |  |  |  |  |  |  |  |  |  |  |  |  |  |  |  |  |  |  |  |  |  |  |  |  |  |  |  |  |  |  |  |  |  |  |  |  |  |  |  |  |  |  |  |  |  |  |  |  |  |  |  |  |  |  |  |  |  |  |  |  |  |  |  |  |  |  |  |  |  |  |  |  |  |  |  |  |  |  |  |  |  |  |  |  |  |  |  |  |  |  |  |  |  |  |  |  |  |  |  |  |  |  |  |  |  |  |  |  |  |  |  |  |  |  |  |  |  |  |  |  |  |  |  |  |  |  |  |  |  |  |  |  |  |  |  |  |  |  |  |  |  |  |  |  |  |  |  |  |  |  |  |  |  |  |  |  |  |  |  |  |  |  |  |  |  |  |  |  |  |  |  |  |  |  |  |  |  |  |  |  |  |  |  |  |  |  |  |  |  |  |  |  |  |  |  |  |  |  |  |  |  |  |  |  |  |  |  |  |  |  |  |  |  |  |  |  |  |  |  |  |  |  |  |  |  |  |  |  |  |  |  |  |  |  |  |  |  |  |  |  |  |  |  |  |  |  |  |  |  |  |  |  |  |  |  |  |  |  |  |  |  |  |  |  |  |  |  |  |  |  |  |  |  |  |  |  |  |  |  |  |  |  |  |  |  |  |  |  |  |  |  |  |  |  |  |  |  |  |  |  |  |  |  |  |  |  |  |  |  |  |  |  |  |  |  |  |  |  |  |  |  |  |  |  |  |  |  |  |  |  |  |  |  |  |  |  |  |  |  |  |  |  |  |  |  |  |  |  |  |  |  |  |  |  |  |  |  |  |  |  |  |  |  |  |  |  |  |  |  |  |  |  |  |  |  |  |  |  |  |  |  |  |  |  |  |  |  |  |  |  |  |  |  |  |  |  |  |  |  |  |  |  |  |  |  |  |  |  |  |  |  |  |  |  |  |  |  |  |  |  |  |  |  |  |  |  |  |  |  |  |  |  |  |  |  |  |  |  |  |  |  |  |  |  |  |  |  |  |  |  |  |  |  |  |  |  |  |  |  |  |  |  |  |  |  |  |  |  |  |  |  |  |  |  |  |  |  |  |  |  |  |  |  |  |  |  |  |  |  |  |  |  |  |  |  |  |  |  |  |  |  |  |  |  |  |  |  |  |  |  |  |  |  |  |  |  |  |  |  |  |  |  |  |  |  |  |  |  |  |  |  |  |  |  |  |  |  |  |  |  |  |  |  |  |  |  |  |  |  |  |  |  |  |  |  |  |  |  |  |  |  |  |  |  |  |  |  |  |  |  |  |  |  |  |  |  |  |  |  |  |  |  |  |  |  |  |  |  |  |  |  |  |  |  |  |  |  |  |  |  |  |  |  |  |  |  |  |  |  |  |  |  |  |  |  |  |  |  |  |  |  |  |  |  |  |  |  |  |  |  |  |  |  |  |  |  |  |  |  |  |  |  |  |  |  |  |  |  |  |  |  |  |  |  |  |  |  |  |  |  |  |  |  |  |  |  |  |  |  |  |  |  |  |  |  |  |  |  |  |  |  |  |  |  |  |  |  |  |  |  |  |  |  |  |  |  |  |  |  |  |  |  |  |  |  |  |  |  |  |  |  |  |  |  |  |  |  |  |  |  |  |  |  |  |  |  |  |  |  |  |  |  |  |  |  |  |  |  |  |  |  |  |  |  |  |  |  |  |  |  |  |  |  |  |  |  |  |  |  |  |  |  |  |  |  |  |  |  |  |  |  |  |  |  |  |  |  |  |  |  |  |  |  |  |  |  |  |  |  |  |  |  |  |  |  |  |  |  |  |  |  |  |  |  |  |  |  |  |  |  |  |  |  |  |  |  |  |  |  |  |  |  |  |  |  |  |  |  |  |  |  |  |  |  |  |  |  |  |  |  |  |  |  |  |  |  |  |  |  |  |  |  |  |  |  |  |  |  |  |  |  |  |  |  |  |  |  |  |  |  |  |  |  |  |  |  |  |  |  |  |  |  |  |  |  |  |  |  |  |  |  |  |  |  |  |  |  |  |  |  |  |  |  |  |  |  |  |  |  |  |  |  |  |  |  |  |  |  |  |  |  |  |  |  |  |  |  |  |  |  |  |  |  |  |  |  |  |  |  |  |  |  |  |  |  |  |  |  |  |  |  |  |  |  |  |  |  |  |  |  |  |  |  |  |  |  |  |  |  |  |  |  |  |  |  |  |  |  |  |  |  |  |  |  |  |  |  |  |  |  |  |  |  |  |  |  |  |  |  |  |  |  |  |  |  |  |  |  |  |  |  |  |  |  |  |  |  |  |  |  |  |  |  |  |  |  |  |  |  |  |  |  |  |  |  |  |  |  |  |  |  |  |  |  |  |  |  |  |  |  |  |  |  |  |  |  |  |  |  |  |  |  |  |  |  |  |  |  |  |  |  |  |  |  |  |  |  |  |  |  |  |  |  |  |  |  |  |  |  |  |  |  |  |  |  |  |  |  |  |  |  |  |  |  |  |  |  |  |  |  |  |  |  |  |  |  |  |  |  |  |  |  |  |  |  |  |  |  |  |  |  |  |  |  |  |  |  |  |  |  |  |  |  |  |  |  |  |  |  |  |  |  |  |  |  |  |  |  |  |  |  |  |  |  |  |  |  |  |  |  |  |  |  |  |  |  |  |  |  |  |  |  |  |  |  |  |  |  |  |  |  |  |  |  |  |  |  |  |  |  |  |  |  |  |  |  |  |  |  |  |  |  |  |  |  |  |  |  |  |  |  |  |  |  |  |  |  |  |  |  |  |  |  |  |  |  |  |  |  |  |  |  |  |  |  |  |  |  |  |  |  |  |  |  |  |  |  |  |  |  |  |  |  |  |  |  |  |  |  |  |  |  |  |  |  |  |  |  |  |  |  |  |  |  |  |  |  |  |  |  |  |  |  |  |  |  |  |  |  |  |  |  |  |  |  |  |  |  |  |  |  |  |  |  |  |  |  |  |  |  |  |  |  |  |  |  |  |  |  |  |  |  |  |  |  |  |  |  |  |  |  |  |  |  |  |  |  |  |  |  |  |  |  |  |  |  |  |  |  |  |  |  |  |  |  |  |  |  |  |  |  |  |  |  |  |  |  |  |  |  |  |  |  |  |  |  |  |  |  |  |  |  |  |  |  |  |  |  |  |  |  |  |  |  |  |  |  |  |  |  |  |  |  |  |  |  |  |  |  |  |  |  |  |  |  |  |  |  |  |  |  |  |  |  |  |  |  |  |  |  |  |  |  |  |  |  |  |  |  |  |  |  |  |  |  |  |  |  |  |  |  |  |  |  |  |  |  |  |  |  |  |  |  |  |  |  |  |  |  |  |  |  |  |  |  |  |  |  |  |  |  |  |  |  |  |  |  |  |  |  |  |  |  |  |  |  |  |  |  |  |  |  |  |  |  |  |  |  |  |  |  |  |  |  |  |  |  |  |  |  |  |  |  |  |  |  |  |  |  |  |  |  |  |  |  |  |  |  |  |  |  |  |  |  |
| --- | --- | --- | --- | --- | --- | --- | --- | --- | --- | --- | --- | --- | --- | --- | --- | --- | --- | --- | --- | --- | --- | --- | --- | --- | --- | --- | --- | --- | --- | --- | --- | --- | --- | --- | --- | --- | --- | --- | --- | --- | --- | --- | --- | --- | --- | --- | --- | --- | --- | --- | --- | --- | --- | --- | --- | --- | --- | --- | --- | --- | --- | --- | --- | --- | --- | --- | --- | --- | --- | --- | --- | --- | --- | --- | --- | --- | --- | --- | --- | --- | --- | --- | --- | --- | --- | --- | --- | --- | --- | --- | --- | --- | --- | --- | --- | --- | --- | --- | --- | --- | --- | --- | --- | --- | --- | --- | --- | --- | --- | --- | --- | --- | --- | --- | --- | --- | --- | --- | --- | --- | --- | --- | --- | --- | --- | --- | --- | --- | --- | --- | --- | --- | --- | --- | --- | --- | --- | --- | --- | --- | --- | --- | --- | --- | --- | --- | --- | --- | --- | --- | --- | --- | --- | --- | --- | --- | --- | --- | --- | --- | --- | --- | --- | --- | --- | --- | --- | --- | --- | --- | --- | --- | --- | --- | --- | --- | --- | --- | --- | --- | --- | --- | --- | --- | --- | --- | --- | --- | --- | --- | --- | --- | --- | --- | --- | --- | --- | --- | --- | --- | --- | --- | --- | --- | --- | --- | --- | --- | --- | --- | --- | --- | --- | --- | --- | --- | --- | --- | --- | --- | --- | --- | --- | --- | --- | --- | --- | --- | --- | --- | --- | --- | --- | --- | --- | --- | --- | --- | --- | --- | --- | --- | --- | --- | --- | --- | --- | --- | --- | --- | --- | --- | --- | --- | --- | --- | --- | --- | --- | --- | --- | --- | --- | --- | --- | --- | --- | --- | --- | --- | --- | --- | --- | --- | --- | --- | --- | --- | --- | --- | --- | --- | --- | --- | --- | --- | --- | --- | --- | --- | --- | --- | --- | --- | --- | --- | --- | --- | --- | --- | --- | --- | --- | --- | --- | --- | --- | --- | --- | --- | --- | --- | --- | --- | --- | --- | --- | --- | --- | --- | --- | --- | --- | --- | --- | --- | --- | --- | --- | --- | --- | --- | --- | --- | --- | --- | --- | --- | --- | --- | --- | --- | --- | --- | --- | --- | --- | --- | --- | --- | --- | --- | --- | --- | --- | --- | --- | --- | --- | --- | --- | --- | --- | --- | --- | --- | --- | --- | --- | --- | --- | --- | --- | --- | --- | --- | --- | --- | --- | --- | --- | --- | --- | --- | --- | --- | --- | --- | --- | --- | --- | --- | --- | --- | --- | --- | --- | --- | --- | --- | --- | --- | --- | --- | --- | --- | --- | --- | --- | --- | --- | --- | --- | --- | --- | --- | --- | --- | --- | --- | --- | --- | --- | --- | --- | --- | --- | --- | --- | --- | --- | --- | --- | --- | --- | --- | --- | --- | --- | --- | --- | --- | --- | --- | --- | --- | --- | --- | --- | --- | --- | --- | --- | --- | --- | --- | --- | --- | --- | --- | --- | --- | --- | --- | --- | --- | --- | --- | --- | --- | --- | --- | --- | --- | --- | --- | --- | --- | --- | --- | --- | --- | --- | --- | --- | --- | --- | --- | --- | --- | --- | --- | --- | --- | --- | --- | --- | --- | --- | --- | --- | --- | --- | --- | --- | --- | --- | --- | --- | --- | --- | --- | --- | --- | --- | --- | --- | --- | --- | --- | --- | --- | --- | --- | --- | --- | --- | --- | --- | --- | --- | --- | --- | --- | --- | --- | --- | --- | --- | --- | --- | --- | --- | --- | --- | --- | --- | --- | --- | --- | --- | --- | --- | --- | --- | --- | --- | --- | --- | --- | --- | --- | --- | --- | --- | --- | --- | --- | --- | --- | --- | --- | --- | --- | --- | --- | --- | --- | --- | --- | --- | --- | --- | --- | --- | --- | --- | --- | --- | --- | --- | --- | --- | --- | --- | --- | --- | --- | --- | --- | --- | --- | --- | --- | --- | --- | --- | --- | --- | --- | --- | --- | --- | --- | --- | --- | --- | --- | --- | --- | --- | --- | --- | --- | --- | --- | --- | --- | --- | --- | --- | --- | --- | --- | --- | --- | --- | --- | --- | --- | --- | --- | --- | --- | --- | --- | --- | --- | --- | --- | --- | --- | --- | --- | --- | --- | --- | --- | --- | --- | --- | --- | --- | --- | --- | --- | --- | --- | --- | --- | --- | --- | --- | --- | --- | --- | --- | --- | --- | --- | --- | --- | --- | --- | --- | --- | --- | --- | --- | --- | --- | --- | --- | --- | --- | --- | --- | --- | --- | --- | --- | --- | --- | --- | --- | --- | --- | --- | --- | --- | --- | --- | --- | --- | --- | --- | --- | --- | --- | --- | --- | --- | --- | --- | --- | --- | --- | --- | --- | --- | --- | --- | --- | --- | --- | --- | --- | --- | --- | --- | --- | --- | --- | --- | --- | --- | --- | --- | --- | --- | --- | --- | --- | --- | --- | --- | --- | --- | --- | --- | --- | --- | --- | --- | --- | --- | --- | --- | --- | --- | --- | --- | --- | --- | --- | --- | --- | --- | --- | --- | --- | --- | --- | --- | --- | --- | --- | --- | --- | --- | --- | --- | --- | --- | --- | --- | --- | --- | --- | --- | --- | --- | --- | --- | --- | --- | --- | --- | --- | --- | --- | --- | --- | --- | --- | --- | --- | --- | --- | --- | --- | --- | --- | --- | --- | --- | --- | --- | --- | --- | --- | --- | --- | --- | --- | --- | --- | --- | --- | --- | --- | --- | --- | --- | --- | --- | --- | --- | --- | --- | --- | --- | --- | --- | --- | --- | --- | --- | --- | --- | --- | --- | --- | --- | --- | --- | --- | --- | --- | --- | --- | --- | --- | --- | --- | --- | --- | --- | --- | --- | --- | --- | --- | --- | --- | --- | --- | --- | --- | --- | --- | --- | --- | --- | --- | --- | --- | --- | --- | --- | --- | --- | --- | --- | --- | --- | --- | --- | --- | --- | --- | --- | --- | --- | --- | --- | --- | --- | --- | --- | --- | --- | --- | --- | --- | --- | --- | --- | --- | --- | --- | --- | --- | --- | --- | --- | --- | --- | --- | --- | --- | --- | --- | --- | --- | --- | --- | --- | --- | --- | --- | --- | --- | --- | --- | --- | --- | --- | --- | --- | --- | --- | --- | --- | --- | --- | --- | --- | --- | --- | --- | --- | --- | --- | --- | --- | --- | --- | --- | --- | --- | --- | --- | --- | --- | --- | --- | --- | --- | --- | --- | --- | --- | --- | --- | --- | --- | --- | --- | --- | --- | --- | --- | --- | --- | --- | --- | --- | --- | --- | --- | --- | --- | --- | --- | --- | --- | --- | --- | --- | --- | --- | --- | --- | --- | --- | --- | --- | --- | --- | --- | --- | --- | --- | --- | --- | --- | --- | --- | --- | --- | --- | --- | --- | --- | --- | --- | --- | --- | --- | --- | --- | --- | --- | --- | --- | --- | --- | --- | --- | --- | --- | --- | --- | --- | --- | --- | --- | --- | --- | --- | --- | --- | --- | --- | --- | --- | --- | --- | --- | --- | --- | --- | --- | --- | --- | --- | --- | --- | --- | --- | --- | --- | --- | --- | --- | --- | --- | --- | --- | --- | --- | --- | --- | --- | --- | --- | --- | --- | --- | --- | --- | --- | --- | --- | --- | --- | --- | --- | --- | --- | --- | --- | --- | --- | --- | --- | --- | --- | --- | --- | --- | --- | --- | --- | --- | --- | --- | --- | --- | --- | --- | --- | --- | --- | --- | --- | --- | --- | --- | --- | --- | --- | --- | --- | --- | --- | --- | --- | --- | --- | --- | --- | --- | --- | --- | --- | --- | --- | --- | --- | --- | --- | --- | --- | --- | --- | --- | --- | --- | --- | --- | --- | --- | --- | --- | --- | --- | --- | --- | --- | --- | --- | --- | --- | --- | --- | --- | --- | --- | --- | --- | --- | --- | --- | --- | --- | --- | --- | --- | --- | --- | --- | --- | --- | --- | --- | --- | --- | --- | --- | --- | --- | --- | --- | --- | --- | --- | --- | --- | --- | --- | --- | --- | --- | --- | --- | --- | --- | --- | --- | --- | --- | --- | --- | --- | --- | --- | --- | --- | --- | --- | --- | --- | --- | --- | --- | --- | --- | --- | --- | --- | --- | --- | --- | --- | --- | --- | --- | --- | --- | --- | --- | --- | --- | --- | --- | --- | --- | --- | --- | --- | --- | --- | --- | --- | --- | --- | --- | --- | --- | --- | --- | --- | --- | --- | --- | --- | --- | --- | --- | --- | --- | --- | --- | --- | --- | --- | --- | --- | --- | --- | --- | --- | --- | --- | --- | --- | --- | --- | --- | --- | --- | --- | --- | --- | --- | --- | --- | --- | --- | --- | --- | --- | --- | --- | --- | --- | --- | --- | --- | --- | --- | --- | --- | --- | --- | --- | --- | --- | --- | --- | --- | --- | --- | --- | --- | --- | --- | --- | --- | --- | --- | --- | --- | --- | --- | --- | --- | --- | --- | --- | --- | --- | --- | --- | --- | --- | --- | --- | --- | --- | --- | --- | --- | --- | --- | --- | --- | --- | --- | --- | --- | --- | --- | --- | --- | --- | --- | --- | --- | --- | --- | --- | --- | --- | --- | --- | --- | --- | --- | --- | --- | --- | --- | --- | --- | --- | --- | --- | --- | --- | --- | --- | --- | --- | --- | --- | --- | --- | --- | --- | --- | --- | --- | --- | --- | --- | --- | --- | --- | --- | --- | --- | --- | --- | --- | --- | --- | --- | --- | --- | --- | --- | --- | --- | --- | --- | --- | --- | --- | --- | --- | --- | --- | --- | --- | --- | --- | --- | --- | --- | --- | --- | --- | --- | --- | --- | --- | --- | --- | --- | --- | --- | --- | --- | --- | --- | --- | --- | --- | --- | --- | --- | --- | --- | --- | --- | --- | --- | --- | --- | --- | --- | --- | --- | --- | --- | --- | --- | --- | --- | --- | --- | --- | --- | --- | --- | --- | --- | --- | --- | --- | --- | --- | --- | --- | --- | --- | --- | --- | --- | --- | --- | --- | --- | --- | --- | --- | --- | --- | --- | --- | --- | --- | --- | --- | --- | --- | --- | --- | --- | --- | --- | --- | --- | --- | --- | --- | --- | --- | --- | --- | --- | --- | --- | --- | --- | --- | --- | --- | --- | --- | --- | --- | --- | --- | --- | --- | --- | --- | --- | --- | --- | --- | --- | --- | --- | --- | --- | --- | --- | --- | --- | --- | --- | --- | --- | --- | --- | --- | --- | --- | --- | --- | --- | --- | --- | --- | --- | --- | --- | --- | --- | --- | --- | --- | --- | --- | --- | --- | --- | --- | --- | --- | --- | --- | --- | --- | --- | --- | --- | --- | --- | --- | --- | --- | --- | --- | --- | --- | --- | --- | --- | --- | --- | --- | --- | --- | --- | --- | --- | --- | --- | --- | --- | --- | --- | --- | --- | --- | --- | --- | --- | --- | --- | --- | --- | --- | --- | --- | --- | --- | --- | --- | --- | --- | --- | --- | --- | --- | --- | --- | --- | --- | --- | --- | --- | --- | --- | --- | --- | --- | --- | --- | --- | --- | --- | --- | --- | --- | --- | --- | --- | --- | --- | --- | --- | --- | --- | --- | --- | --- | --- | --- | --- | --- | --- | --- | --- | --- | --- | --- | --- | --- | --- | --- | --- | --- | --- | --- | --- | --- | --- | --- | --- | --- | --- | --- | --- | --- | --- | --- | --- | --- | --- | --- | --- | --- | --- | --- | --- | --- | --- | --- | --- | --- | --- | --- | --- | --- | --- | --- | --- | --- | --- | --- | --- | --- | --- | --- | --- | --- | --- | --- | --- | --- | --- | --- | --- | --- | --- | --- | --- | --- | --- | --- | --- | --- | --- | --- | --- | --- | --- | --- | --- | --- | --- | --- | --- | --- | --- | --- | --- | --- | --- | --- | --- | --- | --- | --- | --- | --- | --- | --- | --- | --- | --- | --- | --- | --- | --- | --- | --- | --- | --- | --- | --- | --- | --- | --- | --- | --- | --- | --- | --- | --- | --- | --- | --- | --- | --- | --- | --- | --- | --- | --- | --- | --- | --- | --- | --- | --- | --- | --- | --- | --- | --- | --- | --- | --- | --- | --- | --- | --- | --- | --- | --- | --- | --- | --- | --- | --- | --- | --- | --- | --- | --- | --- | --- | --- | --- | --- | --- | --- | --- | --- | --- | --- | --- | --- | --- | --- | --- | --- | --- | --- | --- | --- | --- | --- | --- | --- | --- | --- | --- | --- | --- | --- | --- | --- | --- | --- | --- | --- | --- | --- | --- | --- | --- | --- | --- | --- | --- | --- | --- | --- | --- | --- | --- | --- | --- | --- | --- | --- | --- | --- | --- | --- | --- | --- | --- | --- | --- | --- | --- | --- | --- | --- | --- | --- | --- | --- | --- | --- | --- | --- | --- | --- | --- | --- | --- | --- | --- | --- | --- | --- | --- | --- | --- | --- | --- | --- | --- | --- | --- | --- | --- | --- | --- | --- | --- | --- | --- | --- | --- | --- | --- | --- | --- | --- | --- | --- | --- | --- | --- | --- | --- | --- | --- | --- | --- | --- | --- | --- | --- | --- | --- | --- | --- | --- | --- | --- | --- | --- | --- | --- | --- | --- | --- | --- | --- | --- | --- | --- | --- | --- | --- | --- | --- | --- | --- | --- | --- | --- | --- | --- | --- | --- | --- | --- | --- | --- | --- | --- | --- | --- | --- | --- | --- | --- | --- | --- | --- | --- | --- | --- | --- | --- | --- | --- | --- | --- | --- | --- | --- | --- | --- | --- | --- | --- | --- | --- | --- | --- | --- | --- | --- | --- | --- | --- | --- | --- | --- | --- | --- | --- | --- | --- | --- | --- | --- | --- | --- | --- | --- | --- | --- | --- | --- | --- | --- | --- | --- | --- | --- | --- | --- | --- | --- | --- | --- | --- | --- | --- | --- | --- | --- | --- | --- | --- | --- | --- | --- | --- | --- | --- | --- | --- | --- | --- | --- | --- | --- | --- | --- | --- | --- | --- | --- | --- | --- | --- | --- | --- | --- | --- | --- | --- | --- | --- | --- | --- | --- | --- | --- | --- | --- | --- | --- | --- | --- | --- | --- | --- | --- | --- | --- | --- | --- | --- | --- | --- | --- | --- | --- | --- | --- | --- | --- | --- | --- | --- | --- | --- | --- | --- | --- | --- | --- | --- | --- | --- | --- | --- | --- | --- | --- | --- | --- | --- | --- | --- | --- | --- | --- | --- | --- | --- | --- | --- | --- | --- | --- | --- | --- | --- | --- | --- | --- | --- | --- | --- | --- | --- | --- | --- | --- | --- | --- | --- | --- | --- | --- | --- | --- | --- | --- | --- | --- | --- | --- | --- | --- | --- | --- | --- | --- | --- | --- | --- | --- | --- | --- | --- | --- | --- | --- | --- | --- | --- | --- | --- | --- | --- | --- | --- | --- | --- | --- | --- | --- | --- | --- | --- | --- | --- | --- | --- | --- | --- | --- | --- | --- | --- | --- | --- | --- | --- | --- | --- | --- | --- | --- | --- | --- | --- | --- | --- | --- | --- | --- | --- | --- | --- | --- | --- | --- | --- | --- | --- | --- | --- | --- | --- | --- | --- | --- | --- | --- | --- | --- | --- | --- | --- | --- | --- | --- | --- | --- | --- | --- | --- | --- | --- | --- | --- | --- | --- | --- | --- | --- | --- | --- | --- | --- | --- | --- | --- | --- | --- | --- | --- | --- | --- | --- | --- | --- | --- | --- | --- | --- | --- | --- | --- | --- | --- | --- | --- | --- | --- | --- | --- | --- | --- | --- | --- | --- | --- | --- | --- | --- | --- | --- | --- | --- | --- | --- | --- | --- | --- | --- | --- | --- | --- | --- | --- | --- | --- | --- | --- | --- | --- | --- | --- | --- | --- | --- | --- | --- | --- | --- | --- | --- | --- | --- | --- | --- | --- | --- | --- | --- | --- | --- | --- | --- | --- | --- | --- | --- | --- | --- | --- | --- | --- | --- | --- | --- | --- | --- | --- | --- | --- | --- | --- | --- | --- | --- | --- | --- | --- | --- | --- | --- | --- | --- | --- | --- | --- | --- | --- | --- | --- | --- | --- | --- | --- | --- | --- | --- | --- | --- | --- | --- | --- | --- | --- | --- | --- | --- | --- | --- | --- | --- | --- | --- | --- | --- | --- | --- | --- | --- | --- | --- | --- | --- | --- | --- | --- | --- | --- | --- | --- | --- | --- | --- | --- | --- | --- | --- | --- | --- | --- | --- | --- | --- | --- | --- | --- | --- | --- | --- | --- | --- | --- | --- | --- | --- | --- | --- | --- | --- | --- | --- | --- | --- | --- | --- | --- | --- | --- | --- | --- | --- | --- | --- | --- | --- | --- | --- | --- | --- | --- | --- | --- | --- | --- | --- | --- | --- | --- | --- | --- | --- | --- | --- | --- | --- | --- | --- | --- | --- | --- | --- | --- | --- | --- | --- | --- | --- | --- | --- | --- | --- | --- | --- | --- | --- | --- | --- | --- | --- | --- | --- | --- | --- | --- | --- | --- | --- | --- | --- | --- | --- | --- | --- | --- | --- | --- | --- | --- | --- | --- | --- | --- | --- | --- | --- | --- | --- | --- | --- | --- | --- | --- | --- | --- | --- | --- | --- | --- | --- | --- | --- | --- | --- | --- | --- | --- | --- | --- | --- | --- | --- | --- | --- | --- | --- | --- | --- | --- | --- | --- | --- | --- | --- | --- | --- | --- | --- | --- | --- | --- | --- | --- | --- | --- | --- | --- | --- | --- | --- | --- | --- | --- | --- | --- | --- | --- | --- | --- | --- | --- | --- | --- | --- | --- | --- | --- | --- | --- | --- | --- | --- | --- | --- | --- | --- | --- | --- | --- | --- | --- | --- | --- | --- | --- | --- | --- | --- | --- | --- | --- | --- | --- | --- | --- | --- | --- | --- | --- | --- | --- | --- | --- | --- | --- | --- | --- | --- | --- | --- | --- | --- | --- | --- | --- | --- | --- | --- | --- | --- | --- | --- | --- | --- | --- | --- | --- | --- | --- | --- | --- | --- | --- | --- | --- | --- | --- | --- | --- | --- | --- | --- | --- | --- | --- | --- | --- | --- | --- | --- | --- | --- | --- | --- | --- | --- | --- | --- | --- | --- | --- | --- | --- | --- | --- | --- | --- | --- | --- | --- | --- | --- | --- | --- | --- | --- | --- | --- | --- | --- | --- | --- | --- | --- | --- | --- | --- | --- | --- | --- | --- | --- | --- | --- | --- | --- | --- | --- | --- | --- | --- | --- | --- | --- | --- | --- | --- | --- | --- | --- | --- | --- | --- | --- | --- | --- | --- | --- | --- | --- | --- | --- | --- | --- | --- | --- | --- | --- | --- | --- | --- | --- | --- | --- | --- | --- | --- | --- | --- | --- | --- | --- | --- | --- | --- | --- | --- | --- | --- | --- | --- | --- | --- | --- | --- | --- | --- | --- | --- | --- | --- | --- | --- | --- | --- | --- | --- | --- | --- | --- | --- | --- | --- | --- | --- | --- | --- | --- | --- | --- | --- | --- | --- | --- | --- | --- | --- | --- | --- | --- | --- | --- | --- | --- | --- | --- | --- | --- | --- | --- | --- | --- | --- | --- | --- | --- | --- | --- | --- | --- | --- | --- | --- | --- | --- | --- | --- | --- | --- | --- | --- | --- | --- | --- | --- | --- | --- | --- | --- | --- | --- | --- | --- | --- | --- | --- | --- | --- | --- | --- | --- | --- | --- | --- | --- | --- | --- | --- | --- | --- | --- | --- | --- | --- | --- | --- | --- | --- | --- | --- | --- | --- | --- | --- | --- | --- | --- | --- | --- | --- | --- | --- | --- | --- | --- | --- | --- | --- | --- | --- | --- | --- | --- | --- | --- | --- | --- | --- | --- | --- | --- | --- | --- | --- | --- | --- | --- | --- | --- | --- | --- | --- | --- | --- | --- | --- | --- | --- | --- | --- | --- | --- | --- | --- | --- | --- | --- | --- | --- | --- | --- | --- | --- | --- | --- | --- | --- | --- | --- | --- | --- | --- | --- | --- | --- | --- | --- | --- | --- | --- | --- | --- | --- | --- | --- | --- | --- | --- | --- | --- | --- | --- | --- | --- | --- | --- | --- | --- | --- | --- | --- | --- | --- | --- | --- | --- | --- | --- | --- | --- | --- | --- | --- | --- | --- | --- | --- | --- | --- | --- | --- | --- | --- | --- | --- | --- | --- | --- | --- | --- | --- | --- | --- | --- | --- | --- | --- | --- | --- | --- | --- | --- | --- | --- | --- | --- | --- | --- | --- | --- | --- | --- | --- | --- | --- | --- | --- | --- | --- | --- | --- | --- | --- | --- | --- | --- | --- | --- | --- | --- | --- | --- | --- | --- | --- | --- | --- | --- | --- | --- | --- | --- | --- | --- | --- | --- | --- | --- | --- | --- | --- | --- | --- | --- | --- | --- | --- | --- | --- | --- | --- | --- | --- | --- | --- | --- | --- | --- | --- | --- | --- | --- | --- | --- | --- | --- | --- | --- | --- | --- | --- | --- | --- | --- | --- | --- | --- | --- | --- | --- | --- | --- | --- | --- | --- | --- | --- | --- | --- | --- | --- | --- | --- | --- | --- | --- | --- | --- | --- | --- | --- | --- | --- | --- | --- | --- | --- | --- | --- | --- | --- | --- | --- | --- | --- | --- | --- | --- | --- | --- | --- | --- | --- | --- | --- | --- | --- | --- | --- | --- | --- | --- | --- | --- | --- | --- | --- | --- | --- | --- | --- | --- | --- | --- | --- | --- | --- | --- | --- | --- | --- | --- | --- | --- | --- | --- | --- | --- | --- | --- | --- | --- | --- | --- | --- | --- | --- | --- | --- | --- | --- | --- | --- | --- | --- | --- | --- | --- | --- | --- | --- | --- | --- | --- | --- | --- | --- | --- | --- | --- | --- | --- | --- | --- | --- | --- | --- | --- | --- | --- | --- | --- | --- | --- | --- | --- | --- | --- | --- | --- | --- | --- | --- | --- | --- | --- | --- | --- | --- | --- | --- | --- | --- | --- | --- | --- | --- | --- | --- | --- | --- | --- | --- | --- | --- | --- | --- | --- | --- | --- | --- | --- | --- | --- | --- | --- | --- | --- | --- | --- | --- | --- | --- | --- | --- | --- | --- | --- | --- | --- | --- | --- | --- | --- | --- | --- | --- | --- | --- | --- | --- | --- | --- | --- | --- | --- | --- | --- | --- | --- | --- | --- | --- | --- | --- | --- | --- | --- | --- | --- | --- | --- | --- | --- | --- | --- | --- | --- | --- | --- | --- | --- | --- | --- | --- | --- | --- | --- | --- | --- | --- | --- | --- | --- | --- | --- | --- | --- | --- | --- | --- | --- | --- | --- | --- | --- | --- | --- | --- | --- | --- | --- | --- | --- | --- | --- | --- | --- | --- | --- | --- | --- | --- | --- | --- | --- | --- | --- | --- | --- | --- | --- | --- | --- | --- | --- | --- | --- | --- | --- | --- | --- | --- | --- | --- | --- | --- | --- | --- | --- | --- | --- | --- | --- | --- | --- | --- | --- | --- | --- | --- | --- | --- | --- | --- | --- | --- | --- | --- | --- | --- | --- | --- | --- | --- | --- | --- | --- | --- | --- | --- | --- | --- | --- | --- | --- | --- | --- | --- | --- | --- | --- | --- | --- | --- | --- | --- | --- | --- | --- | --- | --- | --- | --- | --- | --- | --- | --- | --- | --- | --- | --- | --- | --- | --- | --- | --- | --- | --- | --- | --- | --- | --- | --- | --- | --- | --- | --- | --- | --- | --- | --- | --- | --- | --- | --- | --- | --- | --- | --- | --- | --- | --- | --- | --- | --- | --- | --- | --- | --- | --- | --- | --- | --- | --- | --- | --- | --- | --- | --- | --- | --- | --- | --- | --- | --- | --- | --- | --- | --- | --- | --- | --- | --- | --- | --- | --- | --- | --- | --- | --- | --- | --- | --- | --- | --- | --- | --- | --- | --- | --- | --- | --- | --- | --- | --- | --- | --- | --- | --- | --- | --- | --- | --- | --- | --- | --- | --- | --- | --- | --- | --- | --- | --- | --- | --- | --- | --- | --- | --- | --- | --- | --- | --- | --- | --- | --- | --- | --- | --- | --- | --- | --- | --- | --- | --- | --- | --- | --- | --- | --- | --- | --- | --- | --- | --- | --- | --- | --- | --- | --- | --- | --- | --- | --- | --- | --- | --- | --- | --- | --- | --- | --- | --- | --- | --- | --- | --- | --- | --- | --- | --- | --- | --- | --- | --- | --- | --- | --- | --- | --- | --- | --- | --- | --- | --- | --- | --- | --- | --- | --- | --- | --- | --- | --- | --- | --- | --- | --- | --- | --- | --- | --- | --- | --- | --- | --- | --- | --- | --- | --- | --- | --- | --- | --- | --- | --- | --- | --- | --- | --- | --- | --- | --- | --- | --- | --- | --- | --- | --- | --- | --- | --- | --- | --- | --- | --- | --- | --- | --- | --- | --- | --- | --- | --- | --- | --- | --- | --- | --- | --- | --- | --- | --- | --- | --- | --- | --- | --- | --- | --- | --- | --- | --- | --- | --- | --- | --- | --- | --- | --- | --- | --- | --- | --- | --- | --- | --- | --- | --- | --- | --- | --- | --- | --- | --- | --- | --- | --- | --- | --- | --- | --- | --- | --- | --- | --- | --- | --- | --- | --- | --- | --- | --- | --- | --- | --- | --- | --- | --- | --- | --- | --- | --- | --- | --- | --- | --- | --- | --- | --- | --- | --- | --- | --- | --- | --- | --- | --- | --- | --- | --- | --- | --- | --- | --- | --- | --- | --- | --- | --- | --- | --- | --- | --- | --- | --- | --- | --- | --- | --- | --- | --- | --- | --- | --- | --- | --- | --- | --- | --- | --- | --- | --- | --- | --- | --- | --- | --- | --- | --- | --- | --- | --- | --- | --- | --- | --- | --- | --- | --- | --- | --- | --- | --- | --- | --- | --- | --- | --- | --- | --- | --- | --- | --- | --- | --- | --- | --- | --- | --- | --- | --- | --- | --- | --- | --- | --- | --- | --- | --- | --- | --- | --- | --- | --- | --- | --- | --- | --- | --- | --- | --- | --- | --- | --- | --- | --- | --- | --- | --- | --- | --- | --- | --- | --- | --- | --- | --- | --- | --- | --- | --- | --- | --- | --- | --- | --- | --- | --- | --- | --- | --- | --- | --- | --- | --- | --- | --- | --- | --- | --- | --- | --- | --- | --- | --- | --- | --- | --- | --- | --- | --- | --- | --- | --- | --- | --- | --- | --- | --- | --- | --- | --- | --- | --- | --- | --- | --- | --- | --- | --- | --- | --- | --- | --- | --- | --- | --- | --- | --- | --- | --- | --- | --- | --- | --- | --- | --- | --- | --- | --- | --- | --- | --- | --- | --- | --- | --- | --- | --- | --- | --- | --- | --- | --- | --- | --- | --- | --- | --- | --- | --- | --- | --- | --- | --- | --- | --- | --- | --- | --- | --- | --- | --- | --- | --- | --- | --- | --- | --- | --- | --- | --- | --- | --- | --- | --- | --- | --- | --- | --- | --- | --- | --- | --- | --- | --- | --- | --- | --- | --- | --- | --- | --- | --- | --- | --- | --- | --- | --- | --- | --- | --- | --- | --- | --- | --- | --- | --- | --- | --- | --- | --- | --- | --- | --- | --- | --- | --- | --- | --- | --- | --- | --- | --- | --- | --- | --- | --- | --- | --- | --- | --- | --- | --- | --- | --- | --- | --- | --- | --- | --- | --- | --- | --- | --- | --- | --- | --- | --- | --- | --- | --- | --- | --- | --- | --- | --- | --- | --- | --- | --- | --- | --- | --- | --- | --- | --- | --- | --- | --- | --- | --- | --- | --- | --- | --- | --- | --- | --- | --- | --- | --- | --- | --- | --- | --- | --- | --- | --- | --- | --- | --- | --- | --- | --- | --- | --- | --- | --- | --- | --- | --- | --- | --- | --- | --- | --- | --- | --- | --- | --- | --- | --- | --- | --- | --- | --- | --- | --- | --- | --- | --- | --- | --- | --- | --- | --- | --- | --- | --- | --- | --- | --- | --- | --- | --- | --- | --- | --- | --- | --- | --- | --- | --- | --- | --- | --- | --- | --- | --- | --- | --- | --- | --- | --- | --- | --- | --- | --- | --- | --- | --- | --- | --- | --- | --- | --- | --- | --- | --- | --- | --- | --- | --- | --- | --- | --- | --- | --- | --- | --- | --- | --- | --- | --- | --- | --- | --- | --- | --- | --- | --- | --- | --- | --- | --- | --- | --- | --- | --- | --- | --- | --- | --- | --- | --- | --- | --- | --- | --- | --- | --- | --- | --- | --- | --- | --- | --- | --- | --- | --- | --- | --- | --- | --- | --- | --- | --- | --- | --- | --- | --- | --- | --- | --- | --- | --- | --- | --- | --- | --- | --- | --- | --- | --- | --- | --- | --- | --- | --- | --- | --- | --- | --- | --- | --- | --- | --- | --- | --- | --- | --- | --- | --- | --- | --- | --- | --- | --- | --- | --- | --- | --- | --- | --- | --- | --- | --- | --- | --- | --- | --- | --- | --- | --- | --- | --- | --- | --- | --- | --- | --- | --- | --- | --- | --- | --- | --- | --- | --- | --- | --- | --- | --- | --- | --- | --- | --- | --- | --- | --- | --- | --- | --- | --- | --- | --- | --- | --- | --- | --- | --- | --- | --- | --- | --- | --- | --- | --- | --- | --- | --- | --- | --- | --- | --- | --- | --- |
| |  |  |  |  |  |  |  |  |  |  |  |  |  |  |  |  |  |  |  |  |  |  |  |  |  |  |  |  |  |  |  |  |  |  |  |  |  |  |  |  |  |  |  |  |  |  |  |  |  |  |  |  |  |  |  |  |  |  | | --- | --- | --- | --- | --- | --- | --- | --- | --- | --- | --- | --- | --- | --- | --- | --- | --- | --- | --- | --- | --- | --- | --- | --- | --- | --- | --- | --- | --- | --- | --- | --- | --- | --- | --- | --- | --- | --- | --- | --- | --- | --- | --- | --- | --- | --- | --- | --- | --- | --- | --- | --- | --- | --- | --- | --- | --- | --- | | G0VAP1/1-351 | 1 | - | - | M | S | K | E | D | N | R | N | K | F | L | K | E | F | P | D | L | V | V | E | L | K | Q | V | L | A | Q | Y | G | M | P | Q | E | A | I | T | W | Y | E | N | S | L | N | Y | N | T | P | G | G | K | L | N | R | 53 | | P49349/1-349 | 1 | - | - | - | - | M | S | D | N | R | A | Q | F | L | E | V | F | P | S | L | V | Q | E | L | R | D | I | L | A | G | Y | G | M | P | E | E | A | I | E | W | Y | E | K | S | L | N | Y | N | T | P | G | G | K | L | N | R | 51 | | Q6FLV7/1-351 | 1 | - | - | M | S | K | E | A | S | R | Q | K | F | V | D | E | F | P | S | L | V | Q | E | L | R | V | V | L | E | G | Y | G | M | P | K | E | A | I | E | W | Y | E | S | S | L | N | Y | N | T | P | G | G | K | L | N | R | 53 | | Q757Y2/1-351 | 1 | - | - | M | T | K | E | D | N | R | K | K | F | V | D | E | F | P | S | L | V | S | Q | L | T | Q | S | L | R | E | Y | G | I | P | Q | D | A | I | D | W | Y | E | K | S | L | N | Y | N | T | P | G | G | K | L | N | R | 53 | | A7TFI6/1-352 | 1 | - | M | S | S | K | E | A | I | R | S | K | F | V | D | E | F | P | T | L | V | E | E | L | K | Q | V | L | V | K | Y | G | M | P | E | E | A | I | Q | W | Y | E | K | N | L | N | Y | N | T | P | G | G | K | L | N | R | 54 | | C5DKH2/1-351 | 1 | - | - | M | S | K | E | D | K | R | N | K | F | L | S | V | F | P | K | L | V | D | E | L | K | S | I | L | T | G | Y | G | M | T | A | D | A | V | Q | W | Y | E | N | S | L | N | Y | N | T | P | G | G | K | L | N | R | 53 | | C5E173/1-348 | 1 | - | - | - | - | M | S | D | N | K | S | K | F | L | A | E | F | P | K | I | V | D | E | L | K | Q | D | L | A | Q | Y | G | M | P | S | D | A | V | E | W | Y | E | R | S | L | N | Y | N | T | P | G | G | K | L | N | R | 51 | | Kwal\_33.13820/1-351 | 1 | - | - | M | S | K | E | A | K | R | E | K | F | L | S | V | F | P | K | L | V | D | E | L | K | S | I | L | S | G | Y | G | M | T | E | D | A | I | S | W | Y | E | N | S | L | N | Y | N | T | P | G | G | K | L | N | R | 53 | | Sbay\_648.26/1-352 | 1 | M | A | S | E | K | E | I | K | R | E | K | F | L | S | V | F | P | K | L | V | E | E | L | N | A | S | L | L | A | Y | G | M | P | K | E | A | C | D | W | Y | A | H | S | L | N | Y | N | T | P | G | G | K | L | N | R | 55 | | SAKL0C05192g/1-351 | 1 | - | - | M | T | K | Q | D | N | R | T | R | F | V | A | E | F | P | R | L | V | K | E | L | T | S | V | L | D | D | Y | G | M | P | A | D | A | V | E | W | Y | K | K | S | L | T | Y | N | T | P | G | G | K | L | N | R | 53 | | P08524/1-352 | 1 | M | A | S | E | K | E | I | R | R | E | R | F | L | N | V | F | P | K | L | V | E | E | L | N | A | S | L | L | A | Y | G | M | P | K | E | A | C | D | W | Y | A | H | S | L | N | Y | N | T | P | G | G | K | L | N | R | 55 | |  | | G0VAP1/1-351 | 54 | G | L | S | V | V | D | T | Y | A | I | L | K | G | Y | K | S | V | S | E | L | S | Q | E | E | Y | K | K | V | A | L | L | G | W | C | I | E | L | L | Q | A | Y | F | L | V | A | D | D | M | M | D | K | S | I | T | R | 108 | | P49349/1-349 | 52 | G | L | S | V | V | D | T | Y | A | L | L | K | G | Y | K | S | V | S | E | L | S | A | E | E | Y | K | K | V | A | I | L | G | W | C | I | E | L | L | Q | A | Y | F | L | V | A | D | D | M | M | D | Q | S | I | T | R | 106 | | Q6FLV7/1-351 | 54 | G | L | S | V | V | D | T | Y | A | I | L | K | G | Y | E | S | V | D | D | M | K | D | D | E | Y | K | K | V | A | L | L | G | W | C | I | E | L | L | Q | A | Y | F | L | V | A | D | D | M | M | D | K | S | I | T | R | 108 | | Q757Y2/1-351 | 54 | G | L | S | V | V | D | T | Y | V | I | L | K | N | C | K | S | P | L | D | L | P | V | D | E | Y | R | R | V | A | L | L | G | W | C | V | E | L | L | Q | A | Y | F | L | V | A | D | D | M | M | D | K | S | I | T | R | 108 | | A7TFI6/1-352 | 55 | G | L | S | V | V | D | T | Y | A | I | L | K | G | Y | N | S | V | D | D | L | S | K | D | E | Y | R | R | V | A | V | L | G | W | C | V | E | L | L | Q | A | Y | F | L | V | A | D | D | M | M | D | K | S | I | T | R | 109 | | C5DKH2/1-351 | 54 | G | L | S | V | V | D | S | Y | A | I | L | K | G | K | K | S | Y | E | E | L | S | E | E | E | Y | F | K | L | A | T | L | G | W | C | I | E | L | L | Q | A | Y | F | L | V | A | D | D | M | M | D | K | S | I | T | R | 108 | | C5E173/1-348 | 52 | G | L | S | V | V | D | T | F | A | I | L | K | G | - | T | T | A | T | Q | L | P | S | E | Q | Y | S | K | L | A | L | L | G | W | C | I | E | L | L | Q | A | Y | F | L | V | A | D | D | M | M | D | R | S | I | T | R | 105 | | Kwal\_33.13820/1-351 | 54 | G | L | S | V | V | D | T | Y | A | I | L | T | G | K | K | S | Y | E | E | L | S | E | D | E | Y | F | K | L | A | M | L | G | W | C | I | E | L | L | Q | A | Y | F | L | V | A | D | D | M | M | D | K | S | I | T | R | 108 | | Sbay\_648.26/1-352 | 56 | G | L | S | V | V | D | T | Y | A | I | L | S | N | - | K | T | I | D | Q | L | S | Q | E | E | Y | E | K | V | A | I | L | G | W | C | I | E | L | L | Q | A | Y | F | L | V | A | D | D | M | M | D | K | S | I | T | R | 109 | | SAKL0C05192g/1-351 | 54 | G | L | S | V | I | D | T | Y | V | I | L | K | G | L | G | S | H | E | D | L | T | A | E | E | Y | N | K | V | S | T | L | G | W | C | I | E | L | L | Q | A | Y | F | L | V | A | D | D | M | M | D | K | S | I | T | R | 108 | | P08524/1-352 | 56 | G | L | S | V | V | D | T | Y | A | I | L | S | N | - | K | T | V | E | Q | L | G | Q | E | E | Y | E | K | V | A | I | L | G | W | C | I | E | L | L | Q | A | Y | F | L | V | A | D | D | M | M | D | K | S | I | T | R | 109 | |  | | G0VAP1/1-351 | 109 | R | G | Q | P | C | W | Y | K | V | E | N | V | G | D | V | A | I | N | D | A | F | M | L | E | A | A | I | Y | I | L | L | K | K | H | F | R | N | E | S | Y | Y | V | D | L | L | E | L | F | H | D | V | T | F | Q | T | 163 | | P49349/1-349 | 107 | R | G | Q | P | C | W | Y | K | V | E | N | V | G | D | I | A | I | N | D | A | F | M | L | E | G | A | I | Y | C | L | L | K | K | H | F | R | T | E | P | Y | Y | V | D | L | L | E | L | F | H | D | V | T | F | Q | T | 161 | | Q6FLV7/1-351 | 109 | R | G | Q | P | C | W | Y | R | V | K | N | V | N | E | I | A | I | N | D | A | F | M | L | E | A | A | I | Y | V | L | L | K | K | H | F | R | N | D | S | Y | Y | V | D | L | L | E | L | F | H | D | V | T | F | Q | T | 163 | | Q757Y2/1-351 | 109 | R | G | Q | P | C | W | Y | R | V | E | E | V | G | D | M | A | I | N | D | A | F | M | L | E | A | A | I | Y | C | L | L | K | R | N | F | R | D | Q | P | Y | Y | V | D | L | L | E | L | L | H | D | V | T | F | Q | T | 163 | | A7TFI6/1-352 | 110 | R | G | Q | P | C | W | Y | R | V | E | D | V | G | E | I | A | I | N | D | A | F | M | L | E | A | A | I | Y | E | L | L | K | K | Y | F | R | S | E | S | Y | Y | I | D | L | I | E | L | F | H | D | V | T | F | Q | T | 164 | | C5DKH2/1-351 | 109 | R | G | Q | P | C | W | Y | R | V | E | E | V | G | E | I | A | I | N | D | A | F | M | L | E | G | A | I | Y | C | L | L | K | N | H | F | K | Q | D | S | Y | Y | V | E | L | L | E | L | F | H | D | V | T | F | Q | T | 163 | | C5E173/1-348 | 106 | R | G | Q | P | C | W | Y | K | I | E | E | V | N | E | I | A | I | N | D | A | F | M | L | E | A | A | I | Y | K | L | L | K | S | H | F | R | S | E | P | Y | Y | I | D | L | V | E | L | F | H | E | V | T | F | Q | T | 160 | | Kwal\_33.13820/1-351 | 109 | R | G | Q | P | C | W | Y | R | V | E | E | V | G | E | I | A | I | N | D | A | F | M | L | E | G | A | I | Y | C | L | L | K | N | H | F | K | K | E | A | Y | Y | V | D | L | L | E | L | F | H | D | V | T | F | Q | T | 163 | | Sbay\_648.26/1-352 | 110 | R | G | Q | P | C | W | Y | K | V | P | E | V | G | E | I | A | I | N | D | A | F | M | L | E | A | A | I | Y | K | L | L | K | S | H | F | R | N | E | K | Y | Y | I | D | V | T | E | L | F | Q | E | V | T | F | Q | T | 164 | | SAKL0C05192g/1-351 | 109 | R | G | Q | P | C | W | Y | R | V | D | N | V | G | D | I | A | I | N | D | A | F | M | L | E | G | A | I | Y | C | L | L | K | K | N | F | R | G | E | P | F | Y | V | D | L | L | E | L | L | H | D | V | T | F | Q | T | 163 | | P08524/1-352 | 110 | R | G | Q | P | C | W | Y | K | V | P | E | V | G | E | I | A | I | N | D | A | F | M | L | E | A | A | I | Y | K | L | L | K | S | H | F | R | N | E | K | Y | Y | I | D | I | T | E | L | F | H | E | V | T | F | Q | T | 164 | |  | | G0VAP1/1-351 | 164 | E | L | G | Q | L | L | D | L | I | T | A | P | E | D | K | V | D | L | S | K | F | S | M | K | K | H | S | F | I | V | T | F | K | T | A | Y | Y | S | F | Y | L | P | V | A | L | A | M | H | M | A | G | I | N | D | E | 218 | | P49349/1-349 | 162 | E | L | G | Q | L | L | D | L | I | T | A | P | E | D | K | V | D | L | S | K | F | S | L | E | K | H | S | F | I | V | I | F | K | T | A | Y | Y | S | F | Y | L | A | V | A | L | A | M | F | A | A | G | I | T | D | S | 216 | | Q6FLV7/1-351 | 164 | E | L | G | Q | L | L | D | L | I | T | A | P | E | D | H | V | D | L | S | K | F | S | L | S | K | H | S | F | I | V | I | F | K | T | A | Y | Y | S | F | Y | L | P | V | A | L | A | M | Y | A | A | G | V | N | D | S | 218 | | Q757Y2/1-351 | 164 | E | V | G | Q | L | L | D | L | M | T | A | P | E | F | I | V | D | L | N | K | F | S | L | Q | R | H | S | Y | I | V | I | F | K | T | A | Y | Y | S | F | Y | L | P | V | A | L | A | M | Y | A | A | G | V | N | D | E | 218 | | A7TFI6/1-352 | 165 | E | M | G | Q | L | L | D | L | I | T | A | P | E | E | S | V | D | L | S | K | F | S | L | E | K | H | S | F | I | V | I | F | K | T | A | Y | Y | S | F | Y | L | P | V | A | L | A | M | Y | V | A | G | N | H | D | P | 219 | | C5DKH2/1-351 | 164 | E | L | G | Q | L | L | D | L | I | T | A | P | E | D | N | V | D | L | D | K | F | S | L | D | R | H | S | F | I | V | R | F | K | T | A | Y | Y | S | F | Y | L | P | V | A | L | A | M | H | A | A | G | I | S | D | A | 218 | | C5E173/1-348 | 161 | E | L | G | Q | L | L | D | L | I | T | A | P | E | D | K | I | D | L | S | K | F | S | P | A | K | H | S | F | I | V | I | F | K | T | A | Y | Y | S | F | Y | L | P | V | A | L | A | M | Y | V | A | G | I | S | D | P | 215 | | Kwal\_33.13820/1-351 | 164 | E | L | G | Q | L | L | D | L | I | T | A | P | E | D | R | V | D | L | D | K | F | S | L | E | R | H | S | F | I | V | R | F | K | T | A | Y | Y | S | F | Y | L | P | V | V | L | A | M | Y | A | A | G | I | N | D | E | 218 | | Sbay\_648.26/1-352 | 165 | E | L | G | Q | L | M | D | L | I | T | A | P | E | D | S | V | D | L | S | K | F | S | L | K | K | H | S | F | I | V | T | F | K | T | A | Y | Y | S | F | Y | L | P | V | A | L | A | M | F | V | A | G | I | T | D | E | 219 | | SAKL0C05192g/1-351 | 164 | E | L | G | Q | L | L | D | L | I | T | A | P | E | D | H | V | D | L | S | K | F | S | L | D | R | H | S | F | I | V | V | F | K | T | A | Y | Y | S | F | Y | L | P | V | A | L | A | M | F | V | A | G | I | N | D | Q | 218 | | P08524/1-352 | 165 | E | L | G | Q | L | M | D | L | I | T | A | P | E | D | K | V | D | L | S | K | F | S | L | K | K | H | S | F | I | V | T | F | K | T | A | Y | Y | S | F | Y | L | P | V | A | L | A | M | Y | V | A | G | I | T | D | E | 219 | |  | | G0VAP1/1-351 | 219 | R | D | L | K | Q | A | Q | D | V | L | I | P | L | G | E | Y | F | Q | I | Q | D | D | F | L | D | C | F | G | T | P | E | Q | I | G | K | I | G | T | D | I | Q | D | N | K | C | S | W | V | I | N | K | A | L | E | L | 273 | | P49349/1-349 | 217 | K | D | L | K | Q | A | S | D | V | L | I | P | L | G | E | Y | F | Q | I | Q | D | D | F | L | D | C | F | G | K | P | E | D | I | G | K | I | G | T | D | I | Q | D | N | K | C | S | W | V | I | N | V | A | L | K | N | 271 | | Q6FLV7/1-351 | 219 | K | D | L | K | Q | A | Q | D | V | L | I | P | L | G | E | Y | F | Q | I | Q | D | D | Y | L | D | C | F | G | T | P | E | Q | I | G | K | I | G | T | D | I | Q | D | N | K | C | S | W | I | I | N | K | A | L | E | L | 273 | | Q757Y2/1-351 | 219 | R | D | F | A | Q | A | R | E | V | L | L | P | L | G | E | Y | F | Q | I | Q | D | D | Y | L | D | C | F | G | R | P | E | D | I | G | K | I | G | T | D | I | Q | D | N | K | C | S | W | V | I | N | A | A | L | K | L | 273 | | A7TFI6/1-352 | 220 | K | D | L | K | Q | A | Q | D | V | L | I | P | L | G | E | Y | F | Q | I | Q | D | D | Y | L | D | C | F | G | T | P | E | Q | I | G | K | I | G | T | D | I | Q | D | N | K | C | S | W | V | V | N | K | A | L | Q | L | 274 | | C5DKH2/1-351 | 219 | R | D | L | K | Q | A | Q | D | V | L | I | P | L | G | E | Y | F | Q | I | Q | D | D | Y | L | D | C | F | G | K | P | E | D | I | G | K | I | G | T | D | I | Q | D | N | K | C | S | W | V | V | N | T | A | L | K | L | 273 | | C5E173/1-348 | 216 | K | D | L | K | Q | A | Q | D | V | L | I | P | L | G | E | Y | F | Q | I | Q | D | D | Y | L | D | C | Y | G | T | P | E | Q | I | G | K | I | G | T | D | I | Q | D | N | K | C | S | W | V | V | N | K | A | L | E | L | 270 | | Kwal\_33.13820/1-351 | 219 | R | D | L | K | Q | A | Q | D | V | L | I | P | L | G | E | Y | F | Q | I | Q | D | D | Y | L | D | C | F | G | K | P | E | D | I | G | K | I | G | T | D | I | Q | D | N | K | C | S | W | V | V | N | T | A | L | K | L | 273 | | Sbay\_648.26/1-352 | 220 | K | D | L | K | Q | A | K | D | V | L | I | P | L | G | E | Y | F | Q | I | Q | D | D | Y | L | D | C | F | G | T | P | E | Q | I | G | K | I | G | T | D | I | Q | D | N | K | C | S | W | V | I | N | K | A | L | D | L | 274 | | SAKL0C05192g/1-351 | 219 | R | D | L | K | Q | A | K | D | V | L | I | P | L | G | E | Y | F | Q | I | Q | D | D | Y | L | D | C | F | G | K | P | E | D | I | G | K | I | G | T | D | I | Q | D | N | K | C | S | W | V | I | N | T | A | L | K | L | 273 | | P08524/1-352 | 220 | K | D | L | K | Q | A | R | D | V | L | I | P | L | G | E | Y | F | Q | I | Q | D | D | Y | L | D | C | F | G | T | P | E | Q | I | G | K | I | G | T | D | I | Q | D | N | K | C | S | W | V | I | N | K | A | L | E | L | 274 | |  | | G0VAP1/1-351 | 274 | A | T | S | E | Q | R | K | V | L | D | D | N | Y | G | Q | K | N | K | T | A | E | E | K | C | R | K | I | F | Y | D | L | Q | L | Q | K | Y | Y | E | D | Y | E | E | G | I | A | Q | Q | L | K | E | K | I | S | R | I | 328 | | P49349/1-349 | 272 | A | T | K | E | Q | R | D | I | L | D | E | N | Y | G | R | K | D | S | E | K | E | Q | K | C | R | A | V | F | N | E | L | N | I | Q | D | I | Y | H | K | Y | E | E | E | T | A | S | N | L | R | E | K | I | A | N | I | 326 | | Q6FLV7/1-351 | 274 | A | N | E | K | Q | R | K | I | L | D | E | N | Y | G | R | K | D | S | K | C | E | A | N | C | K | A | V | F | S | E | L | K | I | D | I | H | Y | R | E | Y | E | E | A | V | A | N | E | L | K | D | K | I | S | Q | V | 328 | | Q757Y2/1-351 | 274 | C | S | P | E | Q | R | K | I | L | D | E | N | Y | G | R | K | D | P | E | C | E | Q | R | C | K | Q | I | F | Y | D | L | K | L | Q | E | V | Y | E | R | Y | E | E | Q | V | A | A | R | L | R | E | R | I | G | E | I | 328 | | A7TFI6/1-352 | 275 | A | T | P | E | Q | R | K | L | L | D | E | N | Y | G | R | K | N | S | E | M | E | A | K | C | K | K | L | F | N | E | M | G | I | D | K | Y | Y | H | E | Y | E | E | K | V | A | E | D | L | R | A | K | I | A | Q | V | 329 | | C5DKH2/1-351 | 274 | C | S | P | E | Q | R | K | V | L | D | E | N | Y | G | R | K | D | A | Q | C | E | Q | K | C | R | K | I | F | E | D | L | R | I | D | E | R | Y | H | E | Y | E | E | K | I | A | E | D | L | R | A | L | I | A | R | A | 328 | | C5E173/1-348 | 271 | A | N | A | Q | Q | R | K | T | L | D | E | N | Y | G | R | K | N | A | D | S | E | Q | A | C | K | K | V | F | Q | E | L | N | I | Q | Q | H | Y | L | Q | Y | E | D | S | V | A | K | E | L | K | T | R | I | S | Q | V | 325 | | Kwal\_33.13820/1-351 | 274 | C | S | A | D | Q | R | K | V | L | D | E | N | Y | G | R | K | D | S | E | C | E | K | K | C | K | Q | V | F | D | D | L | K | I | D | A | V | Y | H | E | Y | E | E | K | I | A | Q | K | L | Q | G | K | I | S | S | T | 328 | | Sbay\_648.26/1-352 | 275 | A | S | A | E | Q | R | K | T | L | D | E | N | Y | G | R | K | D | S | V | A | E | A | K | C | K | Q | I | F | N | D | L | K | I | D | Q | L | Y | E | E | Y | E | E | S | I | A | K | D | L | K | S | K | I | S | Q | V | 329 | | SAKL0C05192g/1-351 | 274 | C | T | P | E | Q | R | K | V | L | D | E | N | Y | G | R | K | N | A | E | S | E | K | K | C | K | Q | I | F | Y | D | L | D | I | Q | S | A | Y | H | E | Y | E | E | R | V | A | A | E | L | R | D | K | I | S | K | T | 328 | | P08524/1-352 | 275 | A | S | A | E | Q | R | K | T | L | D | E | N | Y | G | K | K | D | S | V | A | E | A | K | C | K | K | I | F | N | D | L | K | I | E | Q | L | Y | H | E | Y | E | E | S | I | A | K | D | L | K | A | K | I | S | Q | V | 329 | |  | | G0VAP1/1-351 | 329 | D | E | S | R | G | F | K | G | D | V | L | T | A | F | L | N | K | V | Y | K | R | T | K |  | | | | | | | | | | | | | | | | | | | | | | | | | | | | | | | | 351 | | P49349/1-349 | 327 | D | E | S | R | G | F | K | A | E | V | L | T | L | F | L | N | K | I | Y | H | R | K | K |  | | | | | | | | | | | | | | | | | | | | | | | | | | | | | | | | 349 | | Q6FLV7/1-351 | 329 | D | E | S | R | G | F | K | K | E | V | L | T | A | F | L | N | K | V | Y | K | R | S | K |  | | | | | | | | | | | | | | | | | | | | | | | | | | | | | | | | 351 | | Q757Y2/1-351 | 329 | D | E | S | R | G | F | R | R | E | V | L | Y | S | F | L | D | K | V | Y | R | R | K | K |  | | | | | | | | | | | | | | | | | | | | | | | | | | | | | | | | 351 | | A7TFI6/1-352 | 330 | D | E | S | R | G | F | K | G | E | V | L | T | A | F | L | N | K | V | Y | K | R | S | K |  | | | | | | | | | | | | | | | | | | | | | | | | | | | | | | | | 352 | | C5DKH2/1-351 | 329 | D | E | S | R | G | F | K | R | D | V | L | T | A | F | L | G | K | V | Y | K | R | Q | K |  | | | | | | | | | | | | | | | | | | | | | | | | | | | | | | | | 351 | | C5E173/1-348 | 326 | D | E | S | R | G | F | K | Q | A | V | L | T | A | F | F | D | K | I | Y | K | R | S | K |  | | | | | | | | | | | | | | | | | | | | | | | | | | | | | | | | 348 | | Kwal\_33.13820/1-351 | 329 | D | E | S | R | G | F | K | S | E | V | L | T | A | F | L | A | K | V | Y | K | R | Q | K |  | | | | | | | | | | | | | | | | | | | | | | | | | | | | | | | | 351 | | Sbay\_648.26/1-352 | 330 | D | E | S | R | G | F | K | A | D | V | L | T | A | F | L | N | K | V | Y | K | R | S | K |  | | | | | | | | | | | | | | | | | | | | | | | | | | | | | | | | 352 | | SAKL0C05192g/1-351 | 329 | D | E | S | R | G | F | K | A | E | V | L | S | V | F | L | D | K | I | Y | K | R | T | K |  | | | | | | | | | | | | | | | | | | | | | | | | | | | | | | | | 351 | | P08524/1-352 | 330 | D | E | S | R | G | F | K | A | D | V | L | T | A | F | L | N | K | V | Y | K | R | S | K |  | | | | | | | | | | | | | | | | | | | | | | | | | | | | | | | | 352 | |
