# Supplementary material for: Plus ça change – evolutionary sequence divergence predicts protein subcellular localization signals
Source: BMC Genomics. 2014 Jan 20;15:46. doi: 10.1186/1471-2164-15-46 (PMC3906766; doi:10.1186/1471-2164-15-46)
Supplement: Additional file 2 — MSA’s of proteins for which sequence divergence changes predicted localization signals. Contains links to ortholog multiple sequence alignments of each protein in Additional file 3: Table S1. [file 1471-2164-15-46-S2.zip › P09620.html]

|  |  |  |  |  |  |  |  |  |  |  |  |  |  |  |  |  |  |  |  |  |  |  |  |  |  |  |  |  |  |  |  |  |  |  |  |  |  |  |  |  |  |  |  |  |  |  |  |  |  |  |  |  |  |  |  |  |  |  |  |  |  |  |  |  |  |  |  |  |  |  |  |  |  |  |  |  |  |  |  |  |  |  |  |  |  |  |  |  |  |  |  |  |  |  |  |  |  |  |  |  |  |  |  |  |  |  |  |  |  |  |  |  |  |  |  |  |  |  |  |  |  |  |  |  |  |  |  |  |  |  |  |  |  |  |  |  |  |  |  |  |  |  |  |  |  |  |  |  |  |  |  |  |  |  |  |  |  |  |  |  |  |  |  |  |  |  |  |  |  |  |  |  |  |  |  |  |  |  |  |  |  |  |  |  |  |  |  |  |  |  |  |  |  |  |  |  |  |  |  |  |  |  |  |  |  |  |  |  |  |  |  |  |  |  |  |  |  |  |  |  |  |  |  |  |  |  |  |  |  |  |  |  |  |  |  |  |  |  |  |  |  |  |  |  |  |  |  |  |  |  |  |  |  |  |  |  |  |  |  |  |  |  |  |  |  |  |  |  |  |  |  |  |  |  |  |  |  |  |  |  |  |  |  |  |  |  |  |  |  |  |  |  |  |  |  |  |  |  |  |  |  |  |  |  |  |  |  |  |  |  |  |  |  |  |  |  |  |  |  |  |  |  |  |  |  |  |  |  |  |  |  |  |  |  |  |  |  |  |  |  |  |  |  |  |  |  |  |  |  |  |  |  |  |  |  |  |  |  |  |  |  |  |  |  |  |  |  |  |  |  |  |  |  |  |  |  |  |  |  |  |  |  |  |  |  |  |  |  |  |  |  |  |  |  |  |  |  |  |  |  |  |  |  |  |  |  |  |  |  |  |  |  |  |  |  |  |  |  |  |  |  |  |  |  |  |  |  |  |  |  |  |  |  |  |  |  |  |  |  |  |  |  |  |  |  |  |  |  |  |  |  |  |  |  |  |  |  |  |  |  |  |  |  |  |  |  |  |  |  |  |  |  |  |  |  |  |  |  |  |  |  |  |  |  |  |  |  |  |  |  |  |  |  |  |  |  |  |  |  |  |  |  |  |  |  |  |  |  |  |  |  |  |  |  |  |  |  |  |  |  |  |  |  |  |  |  |  |  |  |  |  |  |  |  |  |  |  |  |  |  |  |  |  |  |  |  |  |  |  |  |  |  |  |  |  |  |  |  |  |  |  |  |  |  |  |  |  |  |  |  |  |  |  |  |  |  |  |  |  |  |  |  |  |  |  |  |  |  |  |  |  |  |  |  |  |  |  |  |  |  |  |  |  |  |  |  |  |  |  |  |  |  |  |  |  |  |  |  |  |  |  |  |  |  |  |  |  |  |  |  |  |  |  |  |  |  |  |  |  |  |  |  |  |  |  |  |  |  |  |  |  |  |  |  |  |  |  |  |  |  |  |  |  |  |  |  |  |  |  |  |  |  |  |  |  |  |  |  |  |  |  |  |  |  |  |  |  |  |  |  |  |  |  |  |  |  |  |  |  |  |  |  |  |  |  |  |  |  |  |  |  |  |  |  |  |  |  |  |  |  |  |  |  |  |  |  |  |  |  |  |  |  |  |  |  |  |  |  |  |  |  |  |  |  |  |  |  |  |  |  |  |  |  |  |  |  |  |  |  |  |  |  |  |  |  |  |  |  |  |  |  |  |  |  |  |  |  |  |  |  |  |  |  |  |  |  |  |  |  |  |  |  |  |  |  |  |  |  |  |  |  |  |  |  |  |  |  |  |  |  |  |  |  |  |  |  |  |  |  |  |  |  |  |  |  |  |  |  |  |  |  |  |  |  |  |  |  |  |  |  |  |  |  |  |  |  |  |  |  |  |  |  |  |  |  |  |  |  |  |  |  |  |  |  |  |  |  |  |  |  |  |  |  |  |  |  |  |  |  |  |  |  |  |  |  |  |  |  |  |  |  |  |  |  |  |  |  |  |  |  |  |  |  |  |  |  |  |  |  |  |  |  |  |  |  |  |  |  |  |  |  |  |  |  |  |  |  |  |  |  |  |  |  |  |  |  |  |  |  |  |  |  |  |  |  |  |  |  |  |  |  |  |  |  |  |  |  |  |  |  |  |  |  |  |  |  |  |  |  |  |  |  |  |  |  |  |  |  |  |  |  |  |  |  |  |  |  |  |  |  |  |  |  |  |  |  |  |  |  |  |  |  |  |  |  |  |  |  |  |  |  |  |  |  |  |  |  |  |  |  |  |  |  |  |  |  |  |  |  |  |  |  |  |  |  |  |  |  |  |  |  |  |  |  |  |  |  |  |  |  |  |  |  |  |  |  |  |  |  |  |  |  |  |  |  |  |  |  |  |  |  |  |  |  |  |  |  |  |  |  |  |  |  |  |  |  |  |  |  |  |  |  |  |  |  |  |  |  |  |  |  |  |  |  |  |  |  |  |  |  |  |  |  |  |  |  |  |  |  |  |  |  |  |  |  |  |  |  |  |  |  |  |  |  |  |  |  |  |  |  |  |  |  |  |  |  |  |  |  |  |  |  |  |  |  |  |  |  |  |  |  |  |  |  |  |  |  |  |  |  |  |  |  |  |  |  |  |  |  |  |  |  |  |  |  |  |  |  |  |  |  |  |  |  |  |  |  |  |  |  |  |  |  |  |  |  |  |  |  |  |  |  |  |  |  |  |  |  |  |  |  |  |  |  |  |  |  |  |  |  |  |  |  |  |  |  |  |  |  |  |  |  |  |  |  |  |  |  |  |  |  |  |  |  |  |  |  |  |  |  |  |  |  |  |  |  |  |  |  |  |  |  |  |  |  |  |  |  |  |  |  |  |  |  |  |  |  |  |  |  |  |  |  |  |  |  |  |  |  |  |  |  |  |  |  |  |  |  |  |  |  |  |  |  |  |  |  |  |  |  |  |  |  |  |  |  |  |  |  |  |  |  |  |  |  |  |  |  |  |  |  |  |  |  |  |  |  |  |  |  |  |  |  |  |  |  |  |  |  |  |  |  |  |  |  |  |  |  |  |  |  |  |  |  |  |  |  |  |  |  |  |  |  |  |  |  |  |  |  |  |  |  |  |  |  |  |  |  |  |  |  |  |  |  |  |  |  |  |  |  |  |  |  |  |  |  |  |  |  |  |  |  |  |  |  |  |  |  |  |  |  |  |  |  |  |  |  |  |  |  |  |  |  |  |  |  |  |  |  |  |  |  |  |  |  |  |  |  |  |  |  |  |  |  |  |  |  |  |  |  |  |  |  |  |  |  |  |  |  |  |  |  |  |  |  |  |  |  |  |  |  |  |  |  |  |  |  |  |  |  |  |  |  |  |  |  |  |  |  |  |  |  |  |  |  |  |  |  |  |  |  |  |  |  |  |  |  |  |  |  |  |  |  |  |  |  |  |  |  |  |  |  |  |  |  |  |  |  |  |  |  |  |  |  |  |  |  |  |  |  |  |  |  |  |  |  |  |  |  |  |  |  |  |  |  |  |  |  |  |  |  |  |  |  |  |  |  |  |  |  |  |  |  |  |  |  |  |  |  |  |  |  |  |  |  |  |  |  |  |  |  |  |  |  |  |  |  |  |  |  |  |  |  |  |  |  |  |  |  |  |  |  |  |  |  |  |  |  |  |  |  |  |  |  |  |  |  |  |  |  |  |  |  |  |  |  |  |  |  |  |  |  |  |  |  |  |  |  |  |  |  |  |  |  |  |  |  |  |  |  |  |  |  |  |  |  |  |  |  |  |  |  |  |  |  |  |  |  |  |  |  |  |  |  |  |  |  |  |  |  |  |  |  |  |  |  |  |  |  |  |  |  |  |  |  |  |  |  |  |  |  |  |  |  |  |  |  |  |  |  |  |  |  |  |  |  |  |  |  |  |  |  |  |  |  |  |  |  |  |  |  |  |  |  |  |  |  |  |  |  |  |  |  |  |  |  |  |  |  |  |  |  |  |  |  |  |  |  |  |  |  |  |  |  |  |  |  |  |  |  |  |  |  |  |  |  |  |  |  |  |  |  |  |  |  |  |  |  |  |  |  |  |  |  |  |  |  |  |  |  |  |  |  |  |  |  |  |  |  |  |  |  |  |  |  |  |  |  |  |  |  |  |  |  |  |  |  |  |  |  |  |  |  |  |  |  |  |  |  |  |  |  |  |  |  |  |  |  |  |  |  |  |  |  |  |  |  |  |  |  |  |  |  |  |  |  |  |  |  |  |  |  |  |  |  |  |  |  |  |  |  |  |  |  |  |  |  |  |  |  |  |  |  |  |  |  |  |  |  |  |  |  |  |  |  |  |  |  |  |  |  |  |  |  |  |  |  |  |  |  |  |  |  |  |  |  |  |  |  |  |  |  |  |  |  |  |  |  |  |  |  |  |  |  |  |  |  |  |  |  |  |  |  |  |  |  |  |  |  |  |  |  |  |  |  |  |  |  |  |  |  |  |  |  |  |  |  |  |  |  |  |  |  |  |  |  |  |  |  |  |  |  |  |  |  |  |  |  |  |  |  |  |  |  |  |  |  |  |  |  |  |  |  |  |  |  |  |  |  |  |  |  |  |  |  |  |  |  |  |  |  |  |  |  |  |  |  |  |  |  |  |  |  |  |  |  |  |  |  |  |  |  |  |  |  |  |  |  |  |  |  |  |  |  |  |  |  |  |  |  |  |  |  |  |  |  |  |  |  |  |  |  |  |  |  |  |  |  |  |  |  |  |  |  |  |  |  |  |  |  |  |  |  |  |  |  |  |  |  |  |  |  |  |  |  |  |  |  |  |  |  |  |  |  |  |  |  |  |  |  |  |  |  |  |  |  |  |  |  |  |  |  |  |  |  |  |  |  |  |  |  |  |  |  |  |  |  |  |  |  |  |  |  |  |  |  |  |  |  |  |  |  |  |  |  |  |  |  |  |  |  |  |  |  |  |  |  |  |  |  |  |  |  |  |  |  |  |  |  |  |  |  |  |  |  |  |  |  |  |  |  |  |  |  |  |  |  |  |  |  |  |  |  |  |  |  |  |  |  |  |  |  |  |  |  |  |  |  |  |  |  |  |  |  |  |  |  |  |  |  |  |  |  |  |  |  |  |  |  |  |  |  |  |  |  |  |  |  |  |  |  |  |  |  |  |  |  |  |  |  |  |  |  |  |  |  |  |  |  |  |  |  |  |  |  |  |  |  |  |  |  |  |  |  |  |  |  |  |  |  |  |  |  |  |  |  |  |  |  |  |  |  |  |  |  |  |  |  |  |  |  |  |  |  |  |  |  |  |  |  |  |  |  |  |  |  |  |  |  |  |  |  |  |  |  |  |  |  |  |  |  |  |  |  |  |  |  |  |  |  |  |  |  |  |  |  |  |  |  |  |  |  |  |  |  |  |  |  |  |  |  |  |  |  |  |  |  |  |  |  |  |  |  |  |  |  |  |  |  |  |  |  |  |  |  |  |  |  |  |  |  |  |  |  |  |  |  |  |  |  |  |  |  |  |  |  |  |  |  |  |  |  |  |  |  |  |  |  |  |  |  |  |  |  |  |  |  |  |  |  |  |  |  |  |  |  |  |  |  |  |  |  |  |  |  |  |  |  |  |  |  |  |  |  |  |  |  |  |  |  |  |  |  |  |  |  |  |  |  |  |  |  |  |  |  |  |  |  |  |  |  |  |  |  |  |  |  |  |  |  |  |  |  |  |  |  |  |  |  |  |  |  |  |  |  |  |  |  |  |  |  |  |  |  |  |  |  |  |  |  |  |  |  |  |  |  |  |  |  |  |  |  |  |  |  |  |  |  |  |  |  |  |  |  |  |  |  |  |  |  |  |  |  |  |  |  |  |  |  |  |  |  |  |  |  |  |  |  |  |  |  |  |  |  |  |  |  |  |  |  |  |  |  |  |  |  |  |  |  |  |  |  |  |  |  |  |  |  |  |  |  |  |  |  |  |  |  |  |  |  |  |  |  |  |  |  |  |  |  |  |  |  |  |  |  |  |  |  |  |  |  |  |  |  |  |  |  |  |  |  |  |  |  |  |  |  |  |  |  |  |  |  |  |  |  |  |  |  |  |  |  |  |  |  |  |  |  |  |  |  |  |  |  |  |  |  |  |  |  |  |  |  |  |  |  |  |  |  |  |  |  |  |  |  |  |  |  |  |  |  |  |  |  |  |  |  |  |  |  |  |  |  |  |  |  |  |  |  |  |  |  |  |  |  |  |  |  |  |  |  |  |  |  |  |  |  |  |  |  |  |  |  |  |  |  |  |  |  |  |  |  |  |  |  |  |  |  |  |  |  |  |  |  |  |  |  |  |  |  |  |  |  |  |  |  |  |  |  |  |  |  |  |  |  |  |  |  |  |  |  |  |  |  |  |  |  |  |  |  |  |  |  |  |  |  |  |  |  |  |  |  |  |  |  |  |  |  |  |  |  |  |  |  |  |  |  |  |  |  |  |  |  |  |  |  |  |  |  |  |  |  |  |  |  |  |  |  |  |  |  |  |  |  |  |  |  |  |  |  |  |  |  |  |  |  |  |  |  |  |  |  |  |  |  |  |  |  |  |  |  |  |  |  |  |  |  |  |  |  |  |  |  |  |  |  |  |  |  |  |  |  |  |  |  |  |  |  |  |  |  |  |  |  |  |  |  |  |  |  |  |  |  |  |  |  |  |  |  |  |  |  |  |  |  |  |  |  |  |  |  |  |  |  |  |  |  |  |  |  |  |  |  |  |  |  |  |  |  |  |  |  |  |  |  |  |  |  |  |  |  |  |  |  |  |  |  |  |  |  |  |  |  |  |  |  |  |  |  |  |  |  |  |  |  |  |  |  |  |  |  |  |  |  |  |  |  |  |  |  |  |  |  |  |  |  |  |  |  |  |  |  |  |  |  |  |  |  |  |  |  |  |  |  |  |  |  |  |  |  |  |  |  |  |  |  |  |  |  |  |  |  |  |  |  |  |  |  |  |  |  |  |  |  |  |  |  |  |  |  |  |  |  |  |  |  |  |  |  |  |  |  |  |  |  |  |  |  |  |  |  |  |  |  |  |  |  |  |  |  |  |  |  |  |  |  |  |  |  |  |  |  |  |  |  |  |  |  |  |  |  |  |  |  |  |  |  |  |  |  |  |  |  |  |  |  |  |  |  |  |  |  |  |  |  |  |  |  |  |  |  |  |  |  |  |  |  |  |  |  |  |  |  |  |  |  |  |  |  |  |  |  |  |  |  |  |  |  |  |  |  |  |  |  |  |  |  |  |  |  |  |  |  |  |  |  |  |  |  |  |  |  |  |  |  |  |  |  |  |  |  |  |  |  |  |  |  |  |  |  |  |  |  |  |  |  |  |  |  |  |  |  |  |  |  |  |  |  |  |  |  |  |  |  |  |  |  |  |  |  |  |  |  |  |  |  |  |  |  |  |  |  |  |  |  |  |  |  |  |  |  |  |  |  |  |  |  |  |  |  |  |  |  |  |  |  |  |  |  |  |  |  |  |  |  |  |  |  |  |  |  |  |  |  |  |  |  |  |  |  |  |  |  |  |  |  |  |  |  |  |  |  |  |  |  |  |  |  |  |  |  |  |  |  |  |  |  |  |  |  |  |  |  |  |  |  |  |  |  |  |  |  |  |  |  |  |  |  |  |  |  |  |  |  |  |  |  |  |  |  |  |  |  |  |  |  |  |  |  |  |  |  |  |  |  |  |  |  |  |  |  |  |  |  |  |  |  |  |  |  |  |  |  |  |  |  |  |  |  |  |  |  |  |  |  |  |  |  |  |  |  |  |  |  |  |  |  |  |  |  |  |  |  |  |  |  |  |  |  |  |  |  |  |  |  |  |  |  |  |  |  |  |  |  |  |  |  |  |  |  |  |  |  |  |  |  |  |  |  |  |  |  |  |  |  |  |  |  |  |  |  |  |  |  |  |  |  |  |  |  |  |  |  |  |  |  |  |  |  |  |  |  |  |  |  |  |  |  |  |  |  |  |  |  |  |  |  |  |  |  |  |  |  |  |  |  |  |  |  |  |  |  |  |  |  |  |  |  |  |  |  |  |  |  |  |  |  |  |  |  |  |  |  |  |  |  |  |  |  |  |  |  |  |  |  |  |  |  |  |  |  |  |  |  |  |  |  |  |  |  |  |  |  |  |  |  |  |  |  |  |  |  |  |  |  |  |  |  |  |  |  |  |  |  |  |  |  |  |  |  |  |  |  |  |  |  |  |  |  |  |  |  |  |  |  |  |  |  |  |  |  |  |  |  |  |  |  |  |  |  |  |  |  |  |  |  |  |  |  |  |  |  |  |  |  |  |  |  |  |  |  |  |  |  |  |  |  |  |  |  |  |  |  |  |  |  |  |  |  |  |  |  |  |  |  |  |  |  |  |  |  |  |  |  |  |  |  |  |  |  |  |  |  |  |  |  |  |  |  |  |  |  |  |  |  |  |  |  |  |  |  |  |  |  |  |  |  |  |  |  |  |  |  |  |  |  |  |  |  |  |  |  |  |  |  |  |  |  |  |  |  |  |  |  |  |  |  |  |  |  |  |  |  |  |  |  |  |  |  |  |  |  |  |  |  |  |  |  |  |  |  |  |  |  |  |  |  |  |  |  |  |  |  |  |  |  |  |  |  |  |  |  |  |  |  |  |  |  |  |  |  |  |  |  |  |  |  |  |  |  |  |  |  |  |  |  |  |  |  |  |  |  |  |  |  |  |  |  |  |  |  |  |  |  |  |  |  |  |  |  |  |  |  |  |  |  |  |  |  |  |  |  |  |  |  |  |  |  |  |  |  |  |  |  |  |  |  |  |  |  |  |  |  |  |  |  |  |  |  |  |  |  |  |  |  |  |  |  |  |  |  |  |  |  |  |  |  |  |  |  |  |  |  |  |  |  |  |  |  |  |  |  |  |  |  |  |  |  |  |  |  |  |  |  |  |  |  |  |  |  |  |  |  |  |  |  |  |  |  |  |  |  |  |  |  |  |  |  |  |  |  |  |  |  |  |  |  |  |  |  |  |  |  |  |  |  |  |  |  |  |  |  |  |  |  |  |  |  |  |  |  |  |  |  |  |  |  |  |  |  |  |  |  |  |  |  |  |  |  |  |  |  |  |  |  |  |  |  |  |  |  |  |  |  |  |  |  |  |  |  |  |  |  |  |  |  |  |  |  |  |  |  |  |  |  |  |  |  |  |  |  |  |  |  |  |  |  |  |  |  |  |  |  |  |  |  |  |  |  |  |  |  |  |  |  |  |  |  |  |  |  |  |  |  |  |  |  |  |  |  |  |  |  |  |  |  |  |  |  |  |  |  |  |  |  |  |  |  |  |  |  |  |  |  |  |  |  |  |  |  |  |  |  |  |  |  |  |  |  |  |  |  |  |  |  |  |  |  |  |  |  |  |  |  |  |  |  |  |  |  |  |  |  |  |  |  |  |  |  |  |  |  |  |  |  |  |  |  |  |  |  |  |  |  |  |  |  |  |  |  |  |  |  |  |  |  |  |  |  |  |  |  |  |  |  |  |  |  |  |  |  |  |  |  |  |  |  |  |  |  |  |  |  |  |  |  |  |  |  |  |  |  |  |  |  |  |  |  |  |  |  |  |  |  |  |  |  |  |  |  |  |  |  |  |  |  |  |  |  |  |  |  |  |  |  |  |  |  |  |  |  |  |  |  |  |  |  |  |  |  |  |  |  |  |  |  |  |  |  |  |  |  |  |  |  |  |  |  |  |  |  |  |  |  |  |  |  |  |  |  |  |  |  |  |
| --- | --- | --- | --- | --- | --- | --- | --- | --- | --- | --- | --- | --- | --- | --- | --- | --- | --- | --- | --- | --- | --- | --- | --- | --- | --- | --- | --- | --- | --- | --- | --- | --- | --- | --- | --- | --- | --- | --- | --- | --- | --- | --- | --- | --- | --- | --- | --- | --- | --- | --- | --- | --- | --- | --- | --- | --- | --- | --- | --- | --- | --- | --- | --- | --- | --- | --- | --- | --- | --- | --- | --- | --- | --- | --- | --- | --- | --- | --- | --- | --- | --- | --- | --- | --- | --- | --- | --- | --- | --- | --- | --- | --- | --- | --- | --- | --- | --- | --- | --- | --- | --- | --- | --- | --- | --- | --- | --- | --- | --- | --- | --- | --- | --- | --- | --- | --- | --- | --- | --- | --- | --- | --- | --- | --- | --- | --- | --- | --- | --- | --- | --- | --- | --- | --- | --- | --- | --- | --- | --- | --- | --- | --- | --- | --- | --- | --- | --- | --- | --- | --- | --- | --- | --- | --- | --- | --- | --- | --- | --- | --- | --- | --- | --- | --- | --- | --- | --- | --- | --- | --- | --- | --- | --- | --- | --- | --- | --- | --- | --- | --- | --- | --- | --- | --- | --- | --- | --- | --- | --- | --- | --- | --- | --- | --- | --- | --- | --- | --- | --- | --- | --- | --- | --- | --- | --- | --- | --- | --- | --- | --- | --- | --- | --- | --- | --- | --- | --- | --- | --- | --- | --- | --- | --- | --- | --- | --- | --- | --- | --- | --- | --- | --- | --- | --- | --- | --- | --- | --- | --- | --- | --- | --- | --- | --- | --- | --- | --- | --- | --- | --- | --- | --- | --- | --- | --- | --- | --- | --- | --- | --- | --- | --- | --- | --- | --- | --- | --- | --- | --- | --- | --- | --- | --- | --- | --- | --- | --- | --- | --- | --- | --- | --- | --- | --- | --- | --- | --- | --- | --- | --- | --- | --- | --- | --- | --- | --- | --- | --- | --- | --- | --- | --- | --- | --- | --- | --- | --- | --- | --- | --- | --- | --- | --- | --- | --- | --- | --- | --- | --- | --- | --- | --- | --- | --- | --- | --- | --- | --- | --- | --- | --- | --- | --- | --- | --- | --- | --- | --- | --- | --- | --- | --- | --- | --- | --- | --- | --- | --- | --- | --- | --- | --- | --- | --- | --- | --- | --- | --- | --- | --- | --- | --- | --- | --- | --- | --- | --- | --- | --- | --- | --- | --- | --- | --- | --- | --- | --- | --- | --- | --- | --- | --- | --- | --- | --- | --- | --- | --- | --- | --- | --- | --- | --- | --- | --- | --- | --- | --- | --- | --- | --- | --- | --- | --- | --- | --- | --- | --- | --- | --- | --- | --- | --- | --- | --- | --- | --- | --- | --- | --- | --- | --- | --- | --- | --- | --- | --- | --- | --- | --- | --- | --- | --- | --- | --- | --- | --- | --- | --- | --- | --- | --- | --- | --- | --- | --- | --- | --- | --- | --- | --- | --- | --- | --- | --- | --- | --- | --- | --- | --- | --- | --- | --- | --- | --- | --- | --- | --- | --- | --- | --- | --- | --- | --- | --- | --- | --- | --- | --- | --- | --- | --- | --- | --- | --- | --- | --- | --- | --- | --- | --- | --- | --- | --- | --- | --- | --- | --- | --- | --- | --- | --- | --- | --- | --- | --- | --- | --- | --- | --- | --- | --- | --- | --- | --- | --- | --- | --- | --- | --- | --- | --- | --- | --- | --- | --- | --- | --- | --- | --- | --- | --- | --- | --- | --- | --- | --- | --- | --- | --- | --- | --- | --- | --- | --- | --- | --- | --- | --- | --- | --- | --- | --- | --- | --- | --- | --- | --- | --- | --- | --- | --- | --- | --- | --- | --- | --- | --- | --- | --- | --- | --- | --- | --- | --- | --- | --- | --- | --- | --- | --- | --- | --- | --- | --- | --- | --- | --- | --- | --- | --- | --- | --- | --- | --- | --- | --- | --- | --- | --- | --- | --- | --- | --- | --- | --- | --- | --- | --- | --- | --- | --- | --- | --- | --- | --- | --- | --- | --- | --- | --- | --- | --- | --- | --- | --- | --- | --- | --- | --- | --- | --- | --- | --- | --- | --- | --- | --- | --- | --- | --- | --- | --- | --- | --- | --- | --- | --- | --- | --- | --- | --- | --- | --- | --- | --- | --- | --- | --- | --- | --- | --- | --- | --- | --- | --- | --- | --- | --- | --- | --- | --- | --- | --- | --- | --- | --- | --- | --- | --- | --- | --- | --- | --- | --- | --- | --- | --- | --- | --- | --- | --- | --- | --- | --- | --- | --- | --- | --- | --- | --- | --- | --- | --- | --- | --- | --- | --- | --- | --- | --- | --- | --- | --- | --- | --- | --- | --- | --- | --- | --- | --- | --- | --- | --- | --- | --- | --- | --- | --- | --- | --- | --- | --- | --- | --- | --- | --- | --- | --- | --- | --- | --- | --- | --- | --- | --- | --- | --- | --- | --- | --- | --- | --- | --- | --- | --- | --- | --- | --- | --- | --- | --- | --- | --- | --- | --- | --- | --- | --- | --- | --- | --- | --- | --- | --- | --- | --- | --- | --- | --- | --- | --- | --- | --- | --- | --- | --- | --- | --- | --- | --- | --- | --- | --- | --- | --- | --- | --- | --- | --- | --- | --- | --- | --- | --- | --- | --- | --- | --- | --- | --- | --- | --- | --- | --- | --- | --- | --- | --- | --- | --- | --- | --- | --- | --- | --- | --- | --- | --- | --- | --- | --- | --- | --- | --- | --- | --- | --- | --- | --- | --- | --- | --- | --- | --- | --- | --- | --- | --- | --- | --- | --- | --- | --- | --- | --- | --- | --- | --- | --- | --- | --- | --- | --- | --- | --- | --- | --- | --- | --- | --- | --- | --- | --- | --- | --- | --- | --- | --- | --- | --- | --- | --- | --- | --- | --- | --- | --- | --- | --- | --- | --- | --- | --- | --- | --- | --- | --- | --- | --- | --- | --- | --- | --- | --- | --- | --- | --- | --- | --- | --- | --- | --- | --- | --- | --- | --- | --- | --- | --- | --- | --- | --- | --- | --- | --- | --- | --- | --- | --- | --- | --- | --- | --- | --- | --- | --- | --- | --- | --- | --- | --- | --- | --- | --- | --- | --- | --- | --- | --- | --- | --- | --- | --- | --- | --- | --- | --- | --- | --- | --- | --- | --- | --- | --- | --- | --- | --- | --- | --- | --- | --- | --- | --- | --- | --- | --- | --- | --- | --- | --- | --- | --- | --- | --- | --- | --- | --- | --- | --- | --- | --- | --- | --- | --- | --- | --- | --- | --- | --- | --- | --- | --- | --- | --- | --- | --- | --- | --- | --- | --- | --- | --- | --- | --- | --- | --- | --- | --- | --- | --- | --- | --- | --- | --- | --- | --- | --- | --- | --- | --- | --- | --- | --- | --- | --- | --- | --- | --- | --- | --- | --- | --- | --- | --- | --- | --- | --- | --- | --- | --- | --- | --- | --- | --- | --- | --- | --- | --- | --- | --- | --- | --- | --- | --- | --- | --- | --- | --- | --- | --- | --- | --- | --- | --- | --- | --- | --- | --- | --- | --- | --- | --- | --- | --- | --- | --- | --- | --- | --- | --- | --- | --- | --- | --- | --- | --- | --- | --- | --- | --- | --- | --- | --- | --- | --- | --- | --- | --- | --- | --- | --- | --- | --- | --- | --- | --- | --- | --- | --- | --- | --- | --- | --- | --- | --- | --- | --- | --- | --- | --- | --- | --- | --- | --- | --- | --- | --- | --- | --- | --- | --- | --- | --- | --- | --- | --- | --- | --- | --- | --- | --- | --- | --- | --- | --- | --- | --- | --- | --- | --- | --- | --- | --- | --- | --- | --- | --- | --- | --- | --- | --- | --- | --- | --- | --- | --- | --- | --- | --- | --- | --- | --- | --- | --- | --- | --- | --- | --- | --- | --- | --- | --- | --- | --- | --- | --- | --- | --- | --- | --- | --- | --- | --- | --- | --- | --- | --- | --- | --- | --- | --- | --- | --- | --- | --- | --- | --- | --- | --- | --- | --- | --- | --- | --- | --- | --- | --- | --- | --- | --- | --- | --- | --- | --- | --- | --- | --- | --- | --- | --- | --- | --- | --- | --- | --- | --- | --- | --- | --- | --- | --- | --- | --- | --- | --- | --- | --- | --- | --- | --- | --- | --- | --- | --- | --- | --- | --- | --- | --- | --- | --- | --- | --- | --- | --- | --- | --- | --- | --- | --- | --- | --- | --- | --- | --- | --- | --- | --- | --- | --- | --- | --- | --- | --- | --- | --- | --- | --- | --- | --- | --- | --- | --- | --- | --- | --- | --- | --- | --- | --- | --- | --- | --- | --- | --- | --- | --- | --- | --- | --- | --- | --- | --- | --- | --- | --- | --- | --- | --- | --- | --- | --- | --- | --- | --- | --- | --- | --- | --- | --- | --- | --- | --- | --- | --- | --- | --- | --- | --- | --- | --- | --- | --- | --- | --- | --- | --- | --- | --- | --- | --- | --- | --- | --- | --- | --- | --- | --- | --- | --- | --- | --- | --- | --- | --- | --- | --- | --- | --- | --- | --- | --- | --- | --- | --- | --- | --- | --- | --- | --- | --- | --- | --- | --- | --- | --- | --- | --- | --- | --- | --- | --- | --- | --- | --- | --- | --- | --- | --- | --- | --- | --- | --- | --- | --- | --- | --- | --- | --- | --- | --- | --- | --- | --- | --- | --- | --- | --- | --- | --- | --- | --- | --- | --- | --- | --- | --- | --- | --- | --- | --- | --- | --- | --- | --- | --- | --- | --- | --- | --- | --- | --- | --- | --- | --- | --- | --- | --- | --- | --- | --- | --- | --- | --- | --- | --- | --- | --- | --- | --- | --- | --- | --- | --- | --- | --- | --- | --- | --- | --- | --- | --- | --- | --- | --- | --- | --- | --- | --- | --- | --- | --- | --- | --- | --- | --- | --- | --- | --- | --- | --- | --- | --- | --- | --- | --- | --- | --- | --- | --- | --- | --- | --- | --- | --- | --- | --- | --- | --- | --- | --- | --- | --- | --- | --- | --- | --- | --- | --- | --- | --- | --- | --- | --- | --- | --- | --- | --- | --- | --- | --- | --- | --- | --- | --- | --- | --- | --- | --- | --- | --- | --- | --- | --- | --- | --- | --- | --- | --- | --- | --- | --- | --- | --- | --- | --- | --- | --- | --- | --- | --- | --- | --- | --- | --- | --- | --- | --- | --- | --- | --- | --- | --- | --- | --- | --- | --- | --- | --- | --- | --- | --- | --- | --- | --- | --- | --- | --- | --- | --- | --- | --- | --- | --- | --- | --- | --- | --- | --- | --- | --- | --- | --- | --- | --- | --- | --- | --- | --- | --- | --- | --- | --- | --- | --- | --- | --- | --- | --- | --- | --- | --- | --- | --- | --- | --- | --- | --- | --- | --- | --- | --- | --- | --- | --- | --- | --- | --- | --- | --- | --- | --- | --- | --- | --- | --- | --- | --- | --- | --- | --- | --- | --- | --- | --- | --- | --- | --- | --- | --- | --- | --- | --- | --- | --- | --- | --- | --- | --- | --- | --- | --- | --- | --- | --- | --- | --- | --- | --- | --- | --- | --- | --- | --- | --- | --- | --- | --- | --- | --- | --- | --- | --- | --- | --- | --- | --- | --- | --- | --- | --- | --- | --- | --- | --- | --- | --- | --- | --- | --- | --- | --- | --- | --- | --- | --- | --- | --- | --- | --- | --- | --- | --- | --- | --- | --- | --- | --- | --- | --- | --- | --- | --- | --- | --- | --- | --- | --- | --- | --- | --- | --- | --- | --- | --- | --- | --- | --- | --- | --- | --- | --- | --- | --- | --- | --- | --- | --- | --- | --- | --- | --- | --- | --- | --- | --- | --- | --- | --- | --- | --- | --- | --- | --- | --- | --- | --- | --- | --- | --- | --- | --- | --- | --- | --- | --- | --- | --- | --- | --- | --- | --- | --- | --- | --- | --- | --- | --- | --- | --- | --- | --- | --- | --- | --- | --- | --- | --- | --- | --- | --- | --- | --- | --- | --- | --- | --- | --- | --- | --- | --- | --- | --- | --- | --- | --- | --- | --- | --- | --- | --- | --- | --- | --- | --- | --- | --- | --- | --- | --- | --- | --- | --- | --- | --- | --- | --- | --- | --- | --- | --- | --- | --- | --- | --- | --- | --- | --- | --- | --- | --- | --- | --- | --- | --- | --- | --- | --- | --- | --- | --- | --- | --- | --- | --- | --- | --- | --- | --- | --- | --- | --- | --- | --- | --- | --- | --- | --- | --- | --- | --- | --- | --- | --- | --- | --- | --- | --- | --- | --- | --- | --- | --- | --- | --- | --- | --- | --- | --- | --- | --- | --- | --- | --- | --- | --- | --- | --- | --- | --- | --- | --- | --- | --- | --- | --- | --- | --- | --- | --- | --- | --- | --- | --- | --- | --- | --- | --- | --- | --- | --- | --- | --- | --- | --- | --- | --- | --- | --- | --- | --- | --- | --- | --- | --- | --- | --- | --- | --- | --- | --- | --- | --- | --- | --- | --- | --- | --- | --- | --- | --- | --- | --- | --- | --- | --- | --- | --- | --- | --- | --- | --- | --- | --- | --- | --- | --- | --- | --- | --- | --- | --- | --- | --- | --- | --- | --- | --- | --- | --- | --- | --- | --- | --- | --- | --- | --- | --- | --- | --- | --- | --- | --- | --- | --- | --- | --- | --- | --- | --- | --- | --- | --- | --- | --- | --- | --- | --- | --- | --- | --- | --- | --- | --- | --- | --- | --- | --- | --- | --- | --- | --- | --- | --- | --- | --- | --- | --- | --- | --- | --- | --- | --- | --- | --- | --- | --- | --- | --- | --- | --- | --- | --- | --- | --- | --- | --- | --- | --- | --- | --- | --- | --- | --- | --- | --- | --- | --- | --- | --- | --- | --- | --- | --- | --- | --- | --- | --- | --- | --- | --- | --- | --- | --- | --- | --- | --- | --- | --- | --- | --- | --- | --- | --- | --- | --- | --- | --- | --- | --- | --- | --- | --- | --- | --- | --- | --- | --- | --- | --- | --- | --- | --- | --- | --- | --- | --- | --- | --- | --- | --- | --- | --- | --- | --- | --- | --- | --- | --- | --- | --- | --- | --- | --- | --- | --- | --- | --- | --- | --- | --- | --- | --- | --- | --- | --- | --- | --- | --- | --- | --- | --- | --- | --- | --- | --- | --- | --- | --- | --- | --- | --- | --- | --- | --- | --- | --- | --- | --- | --- | --- | --- | --- | --- | --- | --- | --- | --- | --- | --- | --- | --- | --- | --- | --- | --- | --- | --- | --- | --- | --- | --- | --- | --- | --- | --- | --- | --- | --- | --- | --- | --- | --- | --- | --- | --- | --- | --- | --- | --- | --- | --- | --- | --- | --- | --- | --- | --- | --- | --- | --- | --- | --- | --- | --- | --- | --- | --- | --- | --- | --- | --- | --- | --- | --- | --- | --- | --- | --- | --- | --- | --- | --- | --- | --- | --- | --- | --- | --- | --- | --- | --- | --- | --- | --- | --- | --- | --- | --- | --- | --- | --- | --- | --- | --- | --- | --- | --- | --- | --- | --- | --- | --- | --- | --- | --- | --- | --- | --- | --- | --- | --- | --- | --- | --- | --- | --- | --- | --- | --- | --- | --- | --- | --- | --- | --- | --- | --- | --- | --- | --- | --- | --- | --- | --- | --- | --- | --- | --- | --- | --- | --- | --- | --- | --- | --- | --- | --- | --- | --- | --- | --- | --- | --- | --- | --- | --- | --- | --- | --- | --- | --- | --- | --- | --- | --- | --- | --- | --- | --- | --- | --- | --- | --- | --- | --- | --- | --- | --- | --- | --- | --- | --- | --- | --- | --- | --- | --- | --- | --- | --- | --- | --- | --- | --- | --- | --- | --- | --- | --- | --- | --- | --- | --- | --- | --- | --- | --- | --- | --- | --- | --- | --- | --- | --- | --- | --- | --- | --- | --- | --- | --- | --- | --- | --- | --- | --- | --- | --- | --- | --- | --- | --- | --- | --- | --- | --- | --- | --- | --- | --- | --- | --- | --- | --- | --- | --- | --- | --- | --- | --- | --- | --- | --- | --- | --- | --- | --- | --- | --- | --- | --- | --- | --- | --- | --- | --- | --- | --- | --- | --- | --- | --- | --- | --- | --- | --- | --- | --- | --- | --- | --- | --- | --- | --- | --- | --- | --- | --- | --- | --- | --- | --- | --- | --- | --- | --- | --- | --- | --- | --- | --- | --- | --- | --- | --- | --- | --- | --- | --- | --- | --- | --- | --- | --- | --- | --- | --- | --- | --- | --- | --- | --- | --- | --- | --- | --- | --- | --- | --- | --- | --- | --- | --- | --- | --- | --- | --- | --- | --- | --- | --- | --- | --- | --- | --- | --- | --- | --- | --- | --- | --- | --- | --- | --- | --- | --- | --- | --- | --- | --- | --- | --- | --- | --- | --- | --- | --- | --- | --- | --- | --- | --- | --- | --- | --- | --- | --- | --- | --- | --- | --- | --- | --- | --- | --- | --- | --- | --- | --- | --- | --- | --- | --- | --- | --- | --- | --- | --- | --- | --- | --- | --- | --- | --- | --- | --- | --- | --- | --- | --- | --- | --- | --- | --- | --- | --- | --- | --- | --- | --- | --- | --- | --- | --- | --- | --- | --- | --- | --- | --- | --- | --- | --- | --- | --- | --- | --- | --- | --- | --- | --- | --- | --- | --- | --- | --- | --- | --- | --- | --- | --- | --- | --- | --- | --- | --- | --- | --- | --- | --- | --- | --- | --- | --- | --- | --- | --- | --- | --- | --- | --- | --- | --- | --- | --- | --- | --- | --- | --- | --- | --- | --- | --- | --- | --- | --- | --- | --- | --- | --- | --- | --- | --- | --- | --- | --- | --- | --- | --- | --- | --- | --- | --- | --- | --- | --- | --- | --- | --- | --- | --- | --- | --- | --- | --- | --- | --- | --- | --- | --- | --- | --- | --- | --- | --- | --- | --- | --- | --- | --- | --- | --- | --- | --- | --- | --- | --- | --- | --- | --- | --- | --- | --- | --- | --- | --- | --- | --- | --- | --- | --- | --- | --- | --- | --- | --- | --- | --- | --- | --- | --- | --- | --- | --- | --- | --- | --- | --- | --- | --- | --- | --- | --- | --- | --- | --- | --- | --- | --- | --- | --- | --- | --- | --- | --- | --- | --- | --- | --- | --- | --- | --- | --- | --- | --- | --- | --- | --- | --- | --- | --- | --- | --- | --- | --- | --- | --- | --- | --- | --- | --- | --- | --- | --- | --- | --- | --- | --- | --- | --- | --- | --- | --- | --- | --- | --- | --- | --- | --- | --- | --- | --- | --- | --- | --- | --- | --- | --- | --- | --- | --- | --- | --- | --- | --- | --- | --- | --- | --- | --- | --- | --- | --- | --- | --- | --- | --- | --- | --- | --- | --- | --- | --- | --- | --- | --- | --- | --- | --- | --- | --- | --- | --- | --- | --- | --- | --- | --- | --- | --- | --- | --- | --- | --- | --- | --- | --- | --- | --- | --- | --- | --- | --- | --- | --- | --- | --- | --- | --- | --- | --- | --- | --- | --- | --- | --- | --- | --- | --- | --- | --- | --- | --- | --- | --- | --- | --- | --- | --- | --- | --- | --- | --- | --- | --- | --- | --- | --- | --- | --- | --- | --- | --- | --- | --- | --- | --- | --- | --- | --- | --- | --- | --- | --- | --- | --- | --- | --- | --- | --- | --- | --- | --- | --- | --- | --- | --- | --- | --- | --- | --- | --- | --- | --- | --- | --- | --- | --- | --- | --- | --- | --- | --- | --- | --- | --- | --- | --- | --- | --- | --- | --- | --- | --- | --- | --- | --- | --- | --- | --- | --- | --- | --- | --- | --- | --- | --- | --- | --- | --- | --- | --- | --- | --- | --- | --- | --- | --- | --- | --- | --- | --- | --- | --- | --- | --- | --- | --- | --- | --- | --- | --- | --- | --- | --- | --- | --- | --- | --- | --- | --- | --- | --- | --- | --- | --- | --- | --- | --- | --- | --- | --- | --- | --- | --- | --- | --- | --- | --- | --- | --- | --- | --- | --- | --- | --- | --- | --- | --- | --- | --- | --- | --- | --- | --- | --- | --- | --- | --- | --- | --- | --- | --- | --- | --- | --- | --- | --- | --- | --- | --- | --- | --- | --- | --- | --- | --- | --- | --- | --- | --- | --- | --- | --- | --- | --- | --- | --- | --- | --- | --- | --- | --- | --- | --- | --- | --- | --- | --- | --- | --- | --- | --- | --- | --- | --- | --- | --- | --- | --- | --- | --- | --- | --- | --- | --- | --- | --- | --- | --- | --- | --- | --- | --- | --- | --- | --- | --- | --- | --- | --- | --- | --- | --- | --- | --- | --- | --- | --- | --- | --- | --- | --- | --- | --- | --- | --- | --- | --- | --- | --- | --- | --- | --- | --- | --- | --- | --- | --- | --- | --- | --- | --- | --- | --- | --- | --- | --- | --- | --- | --- | --- | --- | --- | --- | --- | --- | --- | --- | --- | --- | --- | --- | --- | --- | --- | --- | --- | --- | --- | --- | --- | --- | --- | --- | --- | --- | --- | --- | --- | --- | --- | --- | --- | --- | --- | --- | --- | --- | --- | --- | --- | --- | --- | --- | --- | --- | --- | --- | --- | --- | --- | --- | --- | --- | --- | --- | --- | --- | --- | --- | --- | --- | --- | --- | --- | --- | --- | --- | --- | --- | --- | --- | --- | --- | --- | --- | --- | --- | --- | --- | --- | --- | --- | --- | --- | --- | --- | --- | --- | --- | --- | --- | --- | --- | --- | --- | --- | --- | --- | --- | --- | --- | --- | --- | --- | --- | --- | --- | --- | --- | --- | --- | --- | --- | --- | --- | --- | --- | --- | --- | --- | --- | --- | --- | --- | --- | --- | --- | --- | --- | --- | --- | --- | --- | --- | --- | --- | --- | --- | --- | --- | --- | --- | --- | --- | --- | --- | --- | --- | --- | --- | --- | --- | --- | --- | --- | --- | --- | --- | --- | --- | --- | --- | --- | --- | --- | --- | --- | --- | --- | --- | --- | --- | --- | --- | --- | --- | --- | --- | --- | --- | --- | --- | --- | --- | --- | --- | --- | --- | --- | --- | --- | --- | --- | --- | --- | --- | --- | --- | --- | --- | --- | --- | --- | --- | --- | --- | --- | --- | --- | --- | --- | --- | --- | --- | --- | --- | --- | --- | --- | --- | --- | --- | --- | --- | --- | --- | --- | --- | --- | --- | --- | --- | --- | --- | --- | --- | --- | --- | --- | --- | --- | --- | --- | --- | --- | --- | --- | --- | --- | --- | --- | --- | --- | --- | --- | --- | --- | --- | --- | --- | --- | --- | --- | --- | --- | --- | --- | --- | --- | --- | --- | --- | --- | --- | --- | --- | --- | --- | --- | --- | --- | --- | --- | --- | --- | --- | --- | --- | --- | --- | --- | --- | --- | --- | --- | --- | --- | --- | --- | --- | --- | --- | --- | --- | --- | --- | --- | --- | --- | --- | --- | --- | --- | --- | --- | --- | --- | --- | --- | --- | --- | --- | --- | --- | --- | --- | --- | --- | --- | --- | --- | --- | --- | --- | --- | --- | --- | --- | --- | --- | --- | --- | --- | --- | --- | --- | --- | --- | --- | --- | --- | --- | --- | --- | --- | --- | --- | --- | --- | --- | --- | --- | --- | --- | --- | --- | --- | --- | --- | --- | --- | --- | --- | --- | --- | --- | --- | --- | --- | --- | --- | --- | --- | --- | --- | --- | --- | --- | --- | --- | --- | --- | --- | --- | --- | --- | --- | --- | --- | --- | --- | --- | --- | --- | --- | --- | --- | --- | --- | --- | --- | --- | --- | --- | --- | --- | --- | --- | --- | --- | --- | --- | --- | --- | --- | --- | --- | --- | --- | --- | --- | --- | --- | --- | --- | --- | --- | --- | --- | --- | --- | --- | --- | --- | --- | --- | --- | --- | --- | --- | --- | --- | --- | --- | --- | --- | --- | --- | --- | --- | --- | --- | --- | --- | --- | --- | --- | --- | --- | --- | --- | --- | --- | --- | --- | --- | --- | --- | --- | --- | --- | --- | --- | --- | --- | --- | --- | --- | --- | --- | --- | --- | --- | --- | --- | --- | --- | --- | --- | --- | --- | --- | --- | --- | --- | --- | --- | --- | --- | --- | --- | --- | --- | --- | --- | --- | --- | --- | --- | --- | --- | --- | --- | --- | --- | --- | --- | --- | --- | --- | --- | --- | --- | --- | --- | --- | --- | --- | --- | --- | --- | --- | --- | --- | --- | --- | --- | --- | --- | --- | --- | --- | --- | --- | --- | --- | --- | --- | --- | --- | --- | --- | --- | --- | --- | --- | --- | --- | --- | --- | --- | --- | --- | --- | --- | --- | --- | --- | --- | --- | --- | --- | --- | --- | --- | --- | --- | --- | --- | --- | --- | --- | --- | --- | --- | --- | --- | --- | --- | --- | --- | --- | --- | --- | --- | --- | --- | --- | --- | --- | --- | --- | --- | --- | --- | --- | --- | --- | --- | --- | --- | --- | --- | --- | --- | --- | --- | --- | --- | --- | --- | --- | --- | --- | --- | --- | --- | --- | --- | --- | --- | --- | --- | --- | --- | --- | --- | --- | --- | --- | --- | --- | --- | --- | --- | --- | --- | --- | --- | --- | --- | --- | --- | --- | --- | --- | --- | --- | --- | --- | --- | --- | --- | --- | --- | --- | --- | --- | --- | --- | --- | --- | --- | --- | --- | --- | --- | --- | --- | --- | --- | --- | --- | --- | --- | --- | --- | --- | --- | --- | --- | --- | --- | --- | --- | --- | --- | --- | --- | --- | --- | --- | --- | --- | --- | --- | --- | --- | --- | --- | --- | --- | --- | --- | --- | --- | --- | --- | --- | --- | --- | --- | --- | --- | --- | --- | --- | --- | --- | --- | --- | --- | --- | --- | --- | --- | --- | --- | --- | --- | --- | --- | --- | --- | --- | --- | --- | --- | --- | --- | --- | --- | --- | --- | --- | --- | --- | --- | --- | --- | --- | --- | --- | --- | --- | --- | --- | --- | --- | --- | --- | --- | --- | --- | --- | --- | --- | --- | --- | --- | --- | --- | --- | --- | --- | --- | --- | --- | --- | --- | --- | --- | --- | --- | --- | --- | --- | --- | --- | --- | --- | --- | --- | --- | --- | --- | --- | --- | --- | --- | --- | --- | --- | --- | --- | --- | --- | --- | --- | --- | --- | --- | --- | --- | --- | --- | --- | --- | --- | --- | --- | --- | --- | --- | --- | --- | --- | --- | --- | --- | --- | --- | --- | --- | --- | --- | --- | --- | --- | --- | --- | --- | --- | --- | --- | --- | --- | --- | --- | --- | --- | --- | --- | --- | --- | --- | --- | --- | --- | --- | --- | --- | --- | --- | --- | --- | --- | --- | --- | --- | --- | --- | --- | --- | --- | --- | --- | --- | --- | --- | --- | --- | --- | --- | --- | --- | --- | --- | --- | --- | --- | --- | --- | --- | --- | --- | --- | --- | --- | --- | --- | --- | --- | --- | --- | --- | --- | --- | --- | --- | --- | --- | --- | --- | --- | --- | --- | --- | --- | --- | --- | --- | --- | --- | --- | --- | --- | --- | --- | --- | --- | --- | --- | --- | --- | --- | --- | --- | --- | --- | --- | --- | --- | --- | --- | --- | --- | --- | --- | --- | --- | --- | --- | --- | --- | --- | --- | --- | --- | --- | --- | --- | --- | --- | --- | --- | --- | --- | --- | --- | --- | --- | --- | --- | --- | --- | --- | --- | --- | --- | --- | --- | --- | --- | --- | --- | --- | --- | --- | --- | --- | --- | --- | --- | --- | --- | --- | --- | --- | --- | --- | --- | --- | --- | --- | --- | --- | --- | --- | --- | --- | --- | --- | --- | --- | --- | --- | --- | --- | --- | --- | --- | --- | --- | --- | --- | --- | --- | --- | --- | --- | --- | --- | --- | --- | --- | --- | --- | --- | --- | --- | --- | --- | --- | --- | --- | --- | --- | --- | --- | --- | --- | --- | --- | --- | --- | --- | --- | --- | --- | --- | --- | --- | --- | --- | --- | --- | --- | --- | --- | --- | --- | --- | --- | --- | --- | --- | --- | --- | --- | --- | --- | --- | --- | --- | --- | --- | --- | --- | --- | --- | --- | --- | --- | --- | --- | --- | --- | --- | --- | --- | --- | --- | --- | --- | --- | --- | --- | --- | --- | --- | --- | --- | --- | --- | --- | --- | --- | --- | --- | --- | --- | --- | --- | --- | --- | --- | --- | --- | --- | --- | --- | --- | --- | --- | --- | --- | --- | --- | --- | --- | --- | --- | --- | --- | --- | --- | --- | --- | --- | --- | --- | --- | --- | --- | --- | --- | --- | --- | --- | --- | --- | --- | --- | --- | --- | --- | --- |
| |  |  |  |  |  |  |  |  |  |  |  |  |  |  |  |  |  |  |  |  |  |  |  |  |  |  |  |  |  |  |  |  |  |  |  |  |  |  |  |  |  |  |  |  |  |  |  |  |  |  |  |  |  |  |  |  |  |  | | --- | --- | --- | --- | --- | --- | --- | --- | --- | --- | --- | --- | --- | --- | --- | --- | --- | --- | --- | --- | --- | --- | --- | --- | --- | --- | --- | --- | --- | --- | --- | --- | --- | --- | --- | --- | --- | --- | --- | --- | --- | --- | --- | --- | --- | --- | --- | --- | --- | --- | --- | --- | --- | --- | --- | --- | --- | --- | | G0VF71/1-703 | 1 | M | V | H | L | S | K | W | L | W | I | L | S | S | T | F | Y | G | T | T | L | V | A | A | L | - | K | S | K | K | D | Y | S | V | A | H | E | L | L | P | G | L | S | S | I | K | D | A | A | L | I | P | E | M | F | A | 54 | | Q752M5/1-599 | 1 | M | - | - | - | - | - | - | L | V | R | K | L | T | L | I | G | A | A | L | A | R | A | A | L | A | L | Q | Q | E | D | F | V | V | N | G | E | L | L | P | G | V | R | E | I | - | D | R | A | E | V | P | E | M | H | A | 48 | | A7TLB3/1-713 | 1 | M | - | M | V | S | Y | K | L | L | S | L | I | T | L | L | F | V | A | Q | C | T | T | G | L | - | L | K | Q | D | D | Y | V | V | R | P | D | L | L | P | G | I | S | S | I | K | D | K | A | L | I | P | K | M | Y | A | 53 | | SAKL0F02134g/1-693 | 1 | M | - | A | L | A | T | V | L | N | A | L | V | A | L | F | L | L | P | H | Y | V | N | S | L | - | L | S | Q | K | D | Y | L | V | S | S | D | L | L | P | G | L | S | S | I | D | D | Q | S | L | I | P | Q | M | Y | A | 53 | | P09620/1-729 | 1 | M | - | F | Y | N | R | W | L | G | T | W | L | A | M | S | A | L | I | R | I | S | V | S | L | - | P | S | S | E | E | Y | K | V | A | Y | E | L | L | P | G | L | S | E | V | P | D | P | S | N | I | P | Q | M | H | A | 53 | |  | | G0VF71/1-703 | 55 | G | Q | I | P | L | - | - | - | - | - | - | - | - | - | - | - | S | D | K | E | D | T | - | N | D | S | K | Y | F | F | W | R | F | H | E | - | - | - | - | - | N | N | K | D | H | D | T | L | I | L | W | L | N | G | G | 92 | | Q752M5/1-599 | 49 | G | L | M | P | L | - | - | - | - | - | - | - | - | - | - | - | - | - | E | E | D | E | - | D | G | R | A | L | F | F | W | R | M | G | E | - | - | - | Q | C | G | K | R | C | S | N | E | L | I | V | W | L | N | G | G | 86 | | A7TLB3/1-713 | 54 | G | H | I | P | L | N | L | Q | K | T | T | D | E | N | E | N | T | D | E | S | D | S | N | S | N | T | N | Y | F | F | W | K | F | Q | H | - | - | - | - | - | Q | S | V | E | S | P | N | L | I | F | W | L | N | G | G | 103 | | SAKL0F02134g/1-693 | 54 | G | H | L | P | L | - | - | - | - | - | - | - | - | - | - | - | G | E | E | E | D | T | - | S | T | K | H | Y | F | F | W | K | F | H | D | T | S | G | L | A | S | S | S | A | A | N | T | L | I | F | W | L | N | G | G | 96 | | P09620/1-729 | 54 | G | H | I | P | L | - | - | - | - | - | - | - | R | S | E | D | A | D | E | Q | D | S | - | S | D | L | E | Y | F | F | W | K | F | T | N | - | - | N | D | S | N | G | N | V | D | R | P | L | I | I | W | L | N | G | G | 98 | |  | | G0VF71/1-703 | 93 | P | G | C | S | S | I | D | G | A | L | V | E | A | G | P | L | R | I | D | S | E | G | K | A | Y | L | N | N | G | S | W | H | T | R | G | D | L | V | F | V | D | Q | P | L | G | T | G | F | S | T | L | D | K | N | K | 147 | | Q752M5/1-599 | 87 | P | G | C | S | S | M | D | G | A | L | M | E | T | G | A | F | R | V | A | E | D | G | K | L | Y | L | N | S | G | S | W | H | T | R | G | T | M | L | F | V | D | Q | P | V | G | T | G | F | S | R | P | G | R | D | G | 141 | | A7TLB3/1-713 | 104 | P | G | C | S | S | M | D | G | A | L | V | E | T | G | P | F | R | V | D | K | N | G | K | L | Y | P | N | E | G | S | W | H | S | R | G | D | L | V | Y | I | D | Q | P | I | G | T | G | L | S | T | S | A | A | I | P | 158 | | SAKL0F02134g/1-693 | 97 | P | G | C | S | S | M | D | G | A | L | M | E | S | G | P | F | R | V | N | D | D | G | K | L | H | L | N | N | G | S | W | H | T | R | G | D | L | V | F | V | D | Q | P | A | G | T | G | F | S | T | V | G | E | D | K | 151 | | P09620/1-729 | 99 | P | G | C | S | S | M | D | G | A | L | V | E | S | G | P | F | R | V | N | S | D | G | K | L | Y | L | N | E | G | S | W | I | S | K | G | D | L | L | F | I | D | Q | P | T | G | T | G | F | S | - | V | E | Q | N | K | 152 | |  | | G0VF71/1-703 | 148 | E | Q | - | - | - | S | D | N | S | F | D | I | D | L | N | D | V | T | N | H | F | M | Q | F | L | E | N | Y | F | Q | V | F | P | E | D | L | P | K | K | L | L | L | A | G | E | S | Y | A | G | Q | Y | I | P | Y | F | 199 | | Q752M5/1-599 | 142 | R | - | - | - | - | - | - | - | - | L | R | T | E | L | S | Q | L | A | D | D | F | L | L | F | M | E | R | Y | Y | A | V | F | P | E | D | R | R | R | T | L | V | L | A | G | E | S | Y | A | G | Q | Y | L | P | Y | F | 188 | | A7TLB3/1-713 | 159 | N | - | - | - | - | - | - | - | - | L | L | D | D | L | K | E | V | S | D | N | F | I | L | F | L | E | N | Y | F | T | I | F | P | N | D | L | D | K | D | I | I | I | A | G | E | S | Y | A | G | Q | Y | I | P | F | F | 205 | | SAKL0F02134g/1-693 | 152 | D | - | - | - | - | - | - | - | - | Y | D | D | D | L | A | L | V | S | Q | R | F | I | A | F | L | E | N | Y | F | T | V | F | P | E | D | Y | S | K | N | I | V | I | S | G | E | S | Y | A | G | Q | F | I | P | F | F | 198 | | P09620/1-729 | 153 | D | E | G | K | I | D | K | N | K | F | D | E | D | L | E | D | V | T | K | H | F | M | D | F | L | E | N | Y | F | K | I | F | P | E | D | L | T | R | K | I | I | L | S | G | E | S | Y | A | G | Q | Y | I | P | F | F | 207 | |  | | G0VF71/1-703 | 200 | A | K | G | I | L | D | H | N | A | K | L | E | H | D | A | I | D | D | Y | E | Y | N | L | E | G | L | L | I | G | N | G | W | I | D | P | S | V | Q | S | L | S | Y | L | P | F | A | V | E | N | K | L | I | D | E | T | 254 | | Q752M5/1-599 | 189 | A | D | A | V | V | R | R | N | A | E | - | - | - | - | R | A | P | E | E | R | Y | K | L | Q | N | V | M | I | G | N | G | W | V | D | P | D | L | Q | S | L | S | Y | V | P | F | V | S | S | R | G | L | F | G | P | E | 239 | | A7TLB3/1-713 | 206 | A | K | A | I | K | E | Y | N | Q | K | - | - | - | I | S | D | N | K | K | K | I | N | L | R | M | L | L | I | G | N | G | W | I | D | P | I | T | Q | S | L | S | Y | L | P | F | A | I | E | K | N | L | V | G | K | D | 257 | | SAKL0F02134g/1-693 | 199 | A | D | A | I | L | D | Y | N | E | E | - | - | - | - | - | N | P | D | S | R | F | N | L | H | S | L | L | I | G | N | G | W | I | D | P | N | S | Q | S | L | S | Y | V | P | F | A | V | D | H | G | L | I | D | K | S | 248 | | P09620/1-729 | 208 | A | N | A | I | L | N | H | N | K | F | - | - | - | S | K | I | D | G | D | T | Y | D | L | K | A | L | L | I | G | N | G | W | I | D | P | N | T | Q | S | L | S | Y | L | P | F | A | M | E | K | K | L | I | D | E | S | 259 | |  | | G0VF71/1-703 | 255 | N | P | N | F | K | N | L | L | N | A | H | E | L | C | Q | K | K | I | N | S | Q | K | T | D | G | N | S | Q | F | S | F | D | E | C | D | K | I | I | N | L | L | L | Q | Y | T | - | - | - | - | K | D | E | S | P | N | 305 | | Q752M5/1-599 | 240 | T | R | N | F | Q | D | I | L | R | D | Q | E | A | C | Q | N | A | I | N | H | G | P | A | K | G | - | - | - | F | S | H | P | E | C | E | G | I | L | P | K | L | L | S | S | I | - | - | - | - | - | - | P | G | P | D | 285 | | A7TLB3/1-713 | 258 | T | P | D | F | E | T | L | L | K | A | H | E | K | C | Q | N | K | I | N | S | I | S | E | D | D | N | S | - | F | S | H | E | E | C | E | S | I | I | N | M | L | V | S | V | T | - | - | - | - | K | D | N | S | P | N | 307 | | SAKL0F02134g/1-693 | 249 | D | S | H | F | K | D | L | L | R | Y | H | E | D | C | Q | N | L | I | N | S | G | T | N | E | G | - | - | - | F | S | Y | D | E | C | E | N | I | L | T | H | L | L | D | Y | T | V | Q | K | K | D | S | E | G | N | K | 300 | | P09620/1-729 | 260 | N | P | N | F | K | H | L | T | N | A | H | E | N | C | Q | N | L | I | N | S | A | S | T | D | E | A | A | H | F | S | Y | Q | E | C | E | N | I | L | N | L | L | L | S | Y | T | - | - | - | - | R | E | S | S | Q | K | 310 | |  | | G0VF71/1-703 | 306 | T | P | A | N | Q | V | C | L | N | V | Y | D | F | Q | L | R | D | S | F | P | Q | C | G | M | N | W | P | A | D | I | S | Y | V | S | K | F | F | N | T | P | G | V | L | E | A | L | N | L | D | S | D | T | T | S | P | 360 | | Q752M5/1-599 | 286 | R | P | V | Q | Q | - | C | I | N | M | Y | D | I | R | L | R | D | V | F | P | S | C | G | M | N | W | P | A | D | L | P | N | V | H | K | F | F | G | T | P | G | V | L | E | A | L | H | V | D | P | Q | V | A | G | P | 339 | | A7TLB3/1-713 | 308 | V | K | S | N | E | V | C | I | N | I | Y | D | F | N | L | R | D | S | F | P | A | C | G | A | N | W | P | I | D | V | S | H | V | A | K | F | F | S | T | P | G | V | I | E | A | L | N | L | N | A | E | E | V | P | R | 362 | | SAKL0F02134g/1-693 | 301 | I | D | D | D | Q | Q | C | L | N | M | Y | D | Y | R | L | R | D | S | Y | P | S | C | G | M | N | W | P | G | D | L | P | N | I | S | K | F | F | S | T | E | G | V | L | E | A | L | N | L | D | S | S | K | V | P | S | 355 | | P09620/1-729 | 311 | G | T | A | D | - | - | C | L | N | M | Y | N | F | N | L | K | D | S | Y | P | S | C | G | M | N | W | P | K | D | I | S | F | V | S | K | F | F | S | T | P | G | V | I | D | S | L | H | L | D | S | D | K | I | D | H | 363 | |  | | G0VF71/1-703 | 361 | W | Q | E | C | N | T | K | V | Q | D | K | L | I | N | P | S | S | K | P | S | I | N | L | L | P | S | I | M | E | S | G | V | E | I | L | L | F | N | G | D | K | D | L | I | C | N | T | M | G | L | Q | N | T | I | D | 415 | | Q752M5/1-599 | 340 | W | V | E | C | K | S | A | V | S | E | A | L | V | N | A | H | S | R | P | S | V | H | L | I | P | G | L | I | E | A | G | V | K | F | V | F | F | N | G | D | Q | D | V | I | C | N | N | M | G | V | E | M | L | I | A | 394 | | A7TLB3/1-713 | 363 | W | K | E | C | N | Y | D | V | L | N | H | L | T | N | P | V | S | K | P | S | V | R | L | L | P | E | L | L | E | S | G | I | E | I | I | L | F | N | G | E | N | D | L | V | C | N | N | K | G | I | T | D | M | I | S | 417 | | SAKL0F02134g/1-693 | 356 | W | R | E | C | D | G | Q | V | S | S | H | L | T | N | P | K | S | K | P | S | I | H | L | L | P | S | L | L | E | S | G | L | Q | I | V | L | F | N | G | D | K | D | I | I | C | N | N | Y | G | V | E | S | L | I | D | 410 | | P09620/1-729 | 364 | W | K | E | C | T | N | S | V | G | T | K | L | S | N | P | I | S | K | P | S | I | H | L | L | P | G | L | L | E | S | G | I | E | I | V | L | F | N | G | D | K | D | L | I | C | N | N | K | G | V | L | D | T | I | D | 418 | |  | | G0VF71/1-703 | 416 | N | L | K | W | G | G | S | T | G | F | S | E | Q | L | E | S | Y | Q | W | S | Y | R | N | Q | Y | D | S | N | S | D | S | N | A | D | N | E | I | T | V | G | S | V | K | Y | D | K | N | L | T | L | V | N | V | Y | N | 470 | | Q752M5/1-599 | 395 | E | L | R | W | R | G | H | M | G | F | S | N | A | T | E | N | F | D | W | Y | H | S | - | - | - | - | - | - | - | D | A | D | A | K | T | L | V | A | A | G | V | V | K | R | D | G | P | V | T | F | I | S | V | F | N | 442 | | A7TLB3/1-713 | 418 | K | L | T | W | N | G | A | T | G | F | S | D | K | V | Q | K | Y | E | W | L | F | R | - | - | - | - | - | - | - | D | L | T | K | D | T | E | E | P | A | G | T | V | T | F | D | R | N | L | T | F | I | S | V | Y | N | 465 | | SAKL0F02134g/1-693 | 411 | S | L | E | W | G | G | V | K | G | F | T | N | E | M | Q | Y | Y | N | W | V | Y | R | - | - | - | - | - | - | - | D | S | D | L | N | F | T | I | P | A | G | S | I | K | Y | E | R | N | L | T | F | I | N | V | Y | N | 458 | | P09620/1-729 | 419 | N | L | K | W | G | G | I | K | G | F | S | D | D | A | V | S | F | D | W | I | H | K | - | - | - | - | - | - | S | K | S | T | D | D | S | E | E | F | S | G | Y | V | K | Y | D | R | N | L | T | F | V | S | V | Y | N | 467 | |  | | G0VF71/1-703 | 471 | A | S | H | M | V | P | H | D | K | G | L | V | S | R | G | I | V | D | I | F | L | K | N | V | V | L | - | E | D | V | D | D | D | V | E | K | L | I | S | S | D | V | P | L | V | S | N | - | E | E | E | E | Q | D | S | 523 | | Q752M5/1-599 | 443 | A | S | H | M | V | P | F | D | V | P | R | I | S | R | G | I | I | D | I | E | R | S | A | T | I | L | G | V | - | - | K | G | D | S | R | M | L | I | T | V | D | T | - | - | - | - | - | - | - | - | - | - | - | - | - | 482 | | A7TLB3/1-713 | 466 | A | S | H | M | V | A | Y | D | K | S | I | V | A | R | G | I | L | D | I | Y | L | D | N | V | M | L | V | E | K | E | T | D | S | P | D | V | L | I | S | T | N | E | P | T | F | S | D | I | E | E | E | E | L | D | G | 520 | | SAKL0F02134g/1-693 | 459 | A | S | H | M | V | P | F | D | N | A | L | V | S | R | G | I | V | D | I | Y | L | N | D | V | Q | L | V | E | - | - | D | G | N | E | D | T | L | V | S | E | G | F | V | K | E | N | K | A | P | E | S | D | I | S | E | 511 | | P09620/1-729 | 468 | A | S | H | M | V | P | F | D | K | S | L | V | S | R | G | I | V | D | I | Y | S | N | D | V | M | I | I | D | - | - | N | N | G | K | N | V | M | I | T | T | D | D | D | S | D | Q | D | A | T | T | E | S | G | D | K | 520 | |  | | G0VF71/1-703 | 524 | Q | K | E | N | - | - | - | - | - | G | T | T | A | G | S | E | E | Q | N | E | - | - | - | - | - | - | - | - | - | - | - | - | - | - | D | D | E | K | H | E | D | E | S | E | E | E | D | E | E | E | E | D | D | D | S | 559 | | Q752M5/1-599 | 483 | - | - | - | - | - | - | - | - | - | - | - | - | - | - | - | - | - | - | - | - | - | - | - | - | - | - | - | - | - | - | - | - | - | - | - | - | - | - | - | - | - | - | - | - | - | - | - | - | - | - | - | - | - | - | - | | A7TLB3/1-713 | 521 | E | K | E | D | E | K | D | G | V | T | E | G | D | G | E | K | S | D | T | D | E | G | K | D | T | D | K | G | K | D | E | K | N | D | D | D | D | D | D | D | D | D | S | D | D | D | S | D | D | D | D | D | D | D | D | 575 | | SAKL0F02134g/1-693 | 512 | G | K | Q | N | - | - | - | - | - | E | K | E | E | E | N | E | E | S | A | S | D | G | D | N | K | D | D | K | D | D | K | - | - | - | D | D | D | D | H | D | D | D | G | D | D | D | G | D | D | D | H | D | D | D | H | 558 | | P09620/1-729 | 521 | P | K | E | N | - | - | - | - | - | L | E | E | E | E | Q | E | A | Q | N | E | E | G | K | E | K | E | G | N | K | D | K | - | - | - | D | G | D | D | - | - | - | - | - | D | N | D | N | D | D | D | D | E | D | D | H | 562 | |  | | G0VF71/1-703 | 560 | D | D | E | E | D | D | D | E | D | D | D | E | D | D | D | D | D | D | D | D | D | E | D | D | N | D | D | - | - | - | - | - | - | - | - | - | E | G | Y | S | - | - | - | - | - | - | - | - | - | - | T | F | R | K | T | 595 | | Q752M5/1-599 | 483 | - | - | - | - | - | - | - | - | E | E | D | V | T | L | S | T | N | A | E | R | N | Q | Q | I | R | H | N | Q | L | K | R | L | N | G | D | T | - | - | - | - | - | - | - | - | - | - | - | - | - | - | - | - | R | K | F | 513 | | A7TLB3/1-713 | 576 | D | D | D | D | D | D | D | D | D | D | D | S | D | D | D | D | D | D | D | D | D | S | D | D | N | E | K | D | D | K | S | E | S | E | T | K | - | - | - | - | - | - | - | - | - | - | - | - | - | - | T | H | P | K | A | 616 | | SAKL0F02134g/1-693 | 559 | D | D | D | G | E | D | D | H | D | D | D | H | D | D | D | G | D | D | E | D | D | N | E | G | E | H | K | K | G | K | H | N | D | S | D | D | S | G | Q | T | - | - | - | - | - | - | - | - | - | - | - | - | G | K | F | 601 | | P09620/1-729 | 563 | N | S | E | G | D | D | D | D | D | D | D | D | D | E | D | D | N | N | E | K | Q | S | N | Q | G | L | E | D | S | R | H | K | S | S | E | Y | E | Q | E | E | E | E | V | E | E | F | A | E | E | I | S | M | Y | K | H | 617 | |  | | G0VF71/1-703 | 596 | T | I | I | V | F | I | V | C | S | L | S | S | C | I | Y | I | I | F | F | Y | G | R | - | S | Q | S | K | N | L | F | S | R | L | K | N | P | - | - | - | - | - | - | - | - | N | K | S | V | T | W | A | D | D | L | E | 641 | | Q752M5/1-599 | 514 | T | I | A | V | F | G | L | T | I | S | S | I | I | G | V | I | V | Y | F | S | M | R | L | H | Y | G | A | K | I | R | A | I | L | T | N | P | - | - | - | - | - | - | - | - | K | S | G | T | S | S | A | D | D | F | S | 560 | | A7TLB3/1-713 | 617 | K | I | A | L | L | L | L | L | F | I | S | V | F | G | I | T | G | S | Q | A | L | R | Q | R | N | F | Q | F | R | R | A | P | L | T | S | N | S | F | S | S | S | S | S | P | N | D | P | S | N | W | D | S | N | D | D | 671 | | SAKL0F02134g/1-693 | 602 | T | M | S | V | I | Y | M | L | L | F | S | V | I | G | V | A | L | Y | Y | F | A | R | E | Y | F | R | P | K | I | R | A | I | L | V | D | P | N | K | R | - | H | D | S | H | K | K | T | V | S | W | A | D | D | L | E | 655 | | P09620/1-729 | 618 | K | A | V | V | V | T | I | V | T | F | L | I | V | V | L | G | V | Y | A | Y | D | R | R | V | R | R | K | A | R | H | T | I | L | V | D | P | N | N | R | Q | H | D | S | P | N | K | T | V | S | W | A | D | D | L | E | 672 | |  | | G0VF71/1-703 | 642 | R | N | A | T | G | T | T | D | E | W | E | D | I | D | D | E | D | N | E | I | T | G | D | V | T | A | G | L | Q | D | D | N | Q | I | S | A | P | P | K | T | P | K | T | K | K | K | G | K | Y | S | S | V | P | A | D | 696 | | Q752M5/1-599 | 561 | M | - | - | - | - | - | - | - | - | - | - | - | - | - | - | - | D | D | E | Y | T | G | T | V | M | G | D | F | H | H | - | - | - | - | - | - | - | - | - | - | - | G | T | H | K | K | G | Q | Y | Y | A | V | P | D | T | 589 | | A7TLB3/1-713 | 672 | F | - | - | - | - | - | - | - | - | - | - | - | - | - | - | - | D | F | D | I | E | N | D | - | - | - | P | L | P | S | T | N | N | - | - | - | - | - | K | H | K | A | A | K | K | K | K | D | Y | V | S | I | P | S | D | 703 | | SAKL0F02134g/1-693 | 656 | Q | - | - | - | - | - | - | - | - | - | - | - | - | - | - | - | - | - | - | - | - | G | D | - | - | A | E | L | Q | E | T | D | T | - | - | - | - | - | - | - | - | K | G | K | K | K | G | G | Y | T | G | I | P | N | T | 680 | | P09620/1-729 | 673 | S | - | G | L | G | A | E | D | D | L | E | Q | - | - | - | - | D | E | Q | L | E | G | G | - | - | A | P | I | S | S | T | S | N | - | - | K | A | G | S | K | L | K | T | K | K | K | K | K | Y | T | S | L | P | N | T | 718 | |  | | G0VF71/1-703 | 697 | E | - | - | - | - | - | - | F | E | M | D | D | F |  | | | | | | | | | | | | | | | | | | | | | | | | | | | | | | | | | | | | | | | | | | 703 | | Q752M5/1-599 | 590 | D | I | - | - | S | S | S | - | E | L | H | S | V |  | | | | | | | | | | | | | | | | | | | | | | | | | | | | | | | | | | | | | | | | | | 599 | | A7TLB3/1-713 | 704 | I | - | - | - | D | E | S | F | E | L | A | E | I |  | | | | | | | | | | | | | | | | | | | | | | | | | | | | | | | | | | | | | | | | | | 713 | | SAKL0F02134g/1-693 | 681 | D | D | T | G | D | D | S | F | E | L | D | D | L |  | | | | | | | | | | | | | | | | | | | | | | | | | | | | | | | | | | | | | | | | | | 693 | | P09620/1-729 | 719 | E | I | - | - | D | E | S | F | E | M | T | D | F |  | | | | | | | | | | | | | | | | | | | | | | | | | | | | | | | | | | | | | | | | | | 729 | |
