# Supplementary material for: Plus ça change – evolutionary sequence divergence predicts protein subcellular localization signals
Source: BMC Genomics. 2014 Jan 20;15:46. doi: 10.1186/1471-2164-15-46 (PMC3906766; doi:10.1186/1471-2164-15-46)
Supplement: Additional file 2 — MSA’s of proteins for which sequence divergence changes predicted localization signals. Contains links to ortholog multiple sequence alignments of each protein in Additional file 3: Table S1. [file 1471-2164-15-46-S2.zip › P13099.html]

|  |  |  |  |  |  |  |  |  |  |  |  |  |  |  |  |  |  |  |  |  |  |  |  |  |  |  |  |  |  |  |  |  |  |  |  |  |  |  |  |  |  |  |  |  |  |  |  |  |  |  |  |  |  |  |  |  |  |  |  |  |  |  |  |  |  |  |  |  |  |  |  |  |  |  |  |  |  |  |  |  |  |  |  |  |  |  |  |  |  |  |  |  |  |  |  |  |  |  |  |  |  |  |  |  |  |  |  |  |  |  |  |  |  |  |  |  |  |  |  |  |  |  |  |  |  |  |  |  |  |  |  |  |  |  |  |  |  |  |  |  |  |  |  |  |  |  |  |  |  |  |  |  |  |  |  |  |  |  |  |  |  |  |  |  |  |  |  |  |  |  |  |  |  |  |  |  |  |  |  |  |  |  |  |  |  |  |  |  |  |  |  |  |  |  |  |  |  |  |  |  |  |  |  |  |  |  |  |  |  |  |  |  |  |  |  |  |  |  |  |  |  |  |  |  |  |  |  |  |  |  |  |  |  |  |  |  |  |  |  |  |  |  |  |  |  |  |  |  |  |  |  |  |  |  |  |  |  |  |  |  |  |  |  |  |  |  |  |  |  |  |  |  |  |  |  |  |  |  |  |  |  |  |  |  |  |  |  |  |  |  |  |  |  |  |  |  |  |  |  |  |  |  |  |  |  |  |  |  |  |  |  |  |  |  |  |  |  |  |  |  |  |  |  |  |  |  |  |  |  |  |  |  |  |  |  |  |  |  |  |  |  |  |  |  |  |  |  |  |  |  |  |  |  |  |  |  |  |  |  |  |  |  |  |  |  |  |  |  |  |  |  |  |  |  |  |  |  |  |  |  |  |  |  |  |  |  |  |  |  |  |  |  |  |  |  |  |  |  |  |  |  |  |  |  |  |  |  |  |  |  |  |  |  |  |  |  |  |  |  |  |  |  |  |  |  |  |  |  |  |  |  |  |  |  |  |  |  |  |  |  |  |  |  |  |  |  |  |  |  |  |  |  |  |  |  |  |  |  |  |  |  |  |  |  |  |  |  |  |  |  |  |  |  |  |  |  |  |  |  |  |  |  |  |  |  |  |  |  |  |  |  |  |  |  |  |  |  |  |  |  |  |  |  |  |  |  |  |  |  |  |  |  |  |  |  |  |  |  |  |  |  |  |  |  |  |  |  |  |  |  |  |  |  |  |  |  |  |  |  |  |  |  |  |  |  |  |  |  |  |  |  |  |  |  |  |  |  |  |  |  |  |  |  |  |  |  |  |  |  |  |  |  |  |  |  |  |  |  |  |  |  |  |  |  |  |  |  |  |  |  |  |  |  |  |  |  |  |  |  |  |  |  |  |  |  |  |  |  |  |  |  |  |  |  |  |  |  |  |  |  |  |  |  |  |  |  |  |  |  |  |  |  |  |  |  |  |  |  |  |  |  |  |  |  |  |  |  |  |  |  |  |  |  |  |  |  |  |  |  |  |  |  |  |  |  |  |  |  |  |  |  |  |  |  |  |  |  |  |  |  |  |  |  |  |  |  |  |  |  |  |  |  |  |  |  |  |  |  |  |  |  |  |  |  |  |  |  |  |  |  |  |  |  |  |  |  |  |  |  |  |  |  |  |  |  |  |  |  |  |  |  |  |  |  |  |  |  |  |  |  |  |  |  |  |  |  |  |  |  |  |  |  |  |  |  |  |  |  |  |  |  |  |  |  |  |  |  |  |  |  |  |  |  |  |  |  |  |  |  |  |  |  |  |  |  |  |  |  |  |  |  |  |  |  |  |  |  |  |  |  |  |  |  |  |  |  |  |  |  |  |  |  |  |  |  |  |  |  |  |  |  |  |  |  |  |  |  |  |  |  |  |  |  |  |  |  |  |  |  |  |  |  |  |  |  |  |  |  |  |  |  |  |  |  |  |  |  |  |  |  |  |  |  |  |  |  |  |  |  |  |  |  |  |  |  |  |  |  |  |  |  |  |  |  |  |  |  |  |  |  |  |  |  |  |  |  |  |  |  |  |  |  |  |  |  |  |  |  |  |  |  |  |  |  |  |  |  |  |  |  |  |  |  |  |  |  |  |  |  |  |  |  |  |  |  |  |  |  |  |  |  |  |  |  |  |  |  |  |  |  |  |  |  |  |  |  |  |  |  |  |  |  |  |  |  |  |  |  |  |  |  |  |  |  |  |  |  |  |  |  |  |  |  |  |  |  |  |  |  |  |  |  |  |  |  |  |  |  |  |  |  |  |  |  |  |  |  |  |  |  |  |  |  |  |  |  |  |  |  |  |  |  |  |  |  |  |  |  |  |  |  |  |  |  |  |  |  |  |  |  |  |  |  |  |  |  |  |  |  |  |  |  |  |  |  |  |  |  |  |  |  |  |  |  |  |  |  |  |  |  |  |  |  |  |  |  |  |  |  |  |  |  |  |  |  |  |  |  |  |  |  |  |  |  |  |  |  |  |  |  |  |  |  |  |  |  |  |  |  |  |  |  |  |  |  |  |  |  |  |  |  |  |  |  |  |  |  |  |  |  |  |  |  |  |  |  |  |  |  |  |  |  |  |  |  |  |  |  |  |  |  |  |  |  |  |  |  |  |  |  |  |  |  |  |  |  |  |  |  |  |  |  |  |  |  |  |  |  |  |  |  |  |  |  |  |  |  |  |  |  |  |  |  |  |  |  |  |  |  |  |  |  |  |  |  |  |  |  |  |  |  |  |  |  |  |  |  |  |  |  |  |  |  |  |  |  |  |  |  |  |  |  |  |  |  |  |  |  |  |  |  |  |  |  |  |  |  |  |  |  |  |  |  |  |  |  |  |  |  |  |  |  |  |  |  |  |  |  |  |  |  |  |  |  |  |  |  |  |  |  |  |  |  |  |  |  |  |  |  |  |  |  |  |  |  |  |  |  |  |  |  |  |  |  |  |  |  |  |  |  |  |  |  |  |  |  |  |  |  |  |  |  |  |  |  |  |  |  |  |  |  |  |  |  |  |  |  |  |  |  |  |  |  |  |  |  |  |  |  |  |  |  |  |  |  |  |  |  |  |  |  |  |  |  |  |  |  |  |  |  |  |  |  |  |  |  |  |  |  |  |  |  |  |  |  |  |  |  |  |  |  |  |  |  |  |  |  |  |  |  |  |  |  |  |  |  |  |  |  |  |  |  |  |  |  |  |  |  |  |  |  |  |  |  |  |  |  |  |  |  |  |  |  |  |  |  |  |  |  |  |  |  |  |  |  |  |  |  |  |  |  |  |  |  |  |  |  |  |  |  |  |  |  |  |  |  |  |  |  |  |  |  |  |  |  |  |  |  |  |  |  |  |  |  |  |  |  |  |  |  |  |  |  |  |  |  |  |  |  |  |  |  |  |  |  |  |  |  |  |  |  |  |  |  |  |  |  |  |  |  |  |  |  |  |  |  |  |  |  |  |  |  |  |  |  |  |  |  |  |  |  |  |  |  |  |  |  |  |  |  |  |  |  |  |  |  |  |  |  |  |  |  |  |  |  |  |  |  |  |  |  |  |  |  |  |  |  |  |  |  |  |  |  |  |  |  |  |  |  |  |  |  |  |  |  |  |  |  |  |  |  |  |  |  |  |  |  |  |  |  |  |  |  |  |  |  |  |  |  |  |  |  |  |  |  |  |  |  |  |  |  |  |  |  |  |  |  |  |  |  |  |  |  |  |  |  |  |  |  |  |  |  |  |  |  |  |  |  |  |  |  |  |  |  |  |  |  |  |  |  |  |  |  |  |  |  |  |  |  |  |  |  |  |  |  |  |  |  |  |  |  |  |  |  |  |  |  |  |  |  |  |  |  |  |  |  |  |  |  |  |  |  |  |  |  |  |  |  |  |  |  |  |  |  |  |  |  |  |  |  |  |  |  |  |  |  |  |  |  |  |  |  |  |  |  |  |  |  |  |  |  |  |  |  |  |  |  |  |  |  |  |  |  |  |  |  |  |  |  |  |  |  |  |  |  |  |  |  |  |  |  |  |  |  |  |  |  |  |  |  |  |  |  |  |  |  |  |  |  |  |  |  |  |  |  |  |  |  |  |  |  |  |  |  |  |  |  |  |  |  |  |  |  |  |  |  |  |  |  |  |  |  |  |  |  |  |  |  |  |  |  |  |  |  |  |  |  |  |  |  |  |  |  |  |  |  |  |  |  |  |  |  |  |  |  |  |  |  |  |  |  |  |  |  |  |  |  |  |  |  |  |  |  |  |  |  |  |  |  |  |  |  |  |  |  |  |  |  |  |  |  |  |  |  |  |  |  |  |  |  |  |  |  |  |  |  |  |  |  |  |  |  |  |  |  |  |  |  |  |  |  |  |  |  |  |  |  |  |  |  |  |  |  |  |  |  |  |  |  |  |  |  |  |  |  |  |  |  |  |  |  |  |  |  |  |  |  |  |  |  |  |  |  |  |  |  |  |  |  |  |  |  |  |  |  |  |  |  |  |  |  |  |  |  |  |  |  |  |  |  |  |  |  |  |  |  |  |  |  |  |  |  |  |  |  |  |  |  |  |  |  |  |  |  |  |  |  |  |  |  |  |  |  |  |  |  |  |  |  |  |  |  |  |  |  |  |  |  |  |  |  |  |  |  |  |  |  |  |  |  |  |  |  |  |  |  |  |  |  |  |  |  |  |  |  |  |  |  |  |  |  |  |  |  |  |  |  |  |  |  |  |  |  |  |  |  |  |  |  |  |  |  |  |  |  |  |  |  |  |  |  |  |  |  |  |  |  |  |  |  |  |  |  |  |  |  |  |  |  |  |  |  |  |  |  |  |  |  |  |  |  |  |  |  |  |  |  |  |  |  |  |  |  |  |  |  |  |  |  |  |  |  |  |  |  |  |  |  |  |  |  |  |  |  |  |  |  |  |  |  |  |  |  |  |  |  |  |  |  |  |  |  |  |  |  |  |  |  |  |  |  |  |  |  |  |  |  |  |  |  |  |  |  |  |  |  |  |  |  |  |  |  |  |  |  |  |  |  |  |  |  |  |  |  |  |  |  |  |  |  |  |  |  |  |  |  |  |  |  |  |  |  |  |  |  |  |  |  |  |  |  |  |  |  |  |  |  |  |  |  |  |  |  |  |  |  |  |  |  |  |  |  |  |  |  |  |  |  |  |  |  |  |  |  |  |  |  |  |  |  |  |  |  |  |  |  |  |  |  |  |  |  |  |  |  |  |  |  |  |  |  |  |  |  |  |  |  |  |  |  |  |  |  |  |  |  |  |  |  |  |  |  |  |  |  |  |  |  |  |  |  |  |  |  |  |  |  |  |  |  |  |  |  |  |  |  |  |  |  |  |  |  |  |  |  |  |  |  |  |  |  |  |  |  |  |  |  |  |  |  |  |  |  |  |  |  |  |  |  |  |  |  |  |  |  |  |  |  |  |  |  |  |  |  |  |  |  |  |  |  |  |  |  |  |  |  |  |  |  |  |  |  |  |  |  |  |  |  |  |  |  |  |  |  |  |  |  |  |  |  |  |  |  |  |  |  |  |  |  |  |  |  |  |  |  |  |  |  |  |  |  |  |  |  |  |  |  |  |  |  |  |  |  |  |  |  |  |  |  |  |  |  |  |  |  |  |  |  |  |  |  |  |  |  |  |  |  |  |  |  |  |  |  |  |  |  |  |  |  |  |  |  |  |  |  |  |  |  |  |  |  |  |  |  |  |  |  |  |  |  |  |  |  |  |  |  |  |  |  |  |  |  |  |  |  |  |  |  |  |  |  |  |  |  |  |  |  |  |  |  |  |  |  |  |  |  |  |  |  |  |  |  |  |  |  |  |  |  |  |  |  |  |  |  |  |  |  |  |  |  |  |  |  |  |  |  |  |  |  |  |  |  |  |  |  |  |  |  |  |  |  |  |  |  |  |  |  |  |  |  |  |  |  |  |  |  |  |  |  |  |  |  |  |  |  |  |  |  |  |  |  |  |  |  |  |  |  |  |  |  |  |  |  |  |  |  |  |  |  |  |  |  |  |  |  |  |  |  |  |  |  |  |  |  |  |  |  |  |  |  |  |  |  |  |  |  |  |  |  |  |  |  |  |  |  |  |  |  |  |  |  |  |  |  |  |  |  |  |  |  |  |  |  |  |  |  |  |  |  |  |  |  |  |  |  |  |  |  |  |  |  |  |  |  |  |  |  |  |  |  |  |  |  |  |  |  |  |  |  |  |  |  |  |  |  |  |  |  |  |  |  |  |  |  |  |  |  |  |  |  |  |  |  |  |  |  |  |  |  |  |  |  |  |  |  |  |  |  |  |  |  |  |  |  |  |  |  |  |  |  |  |  |  |  |  |  |  |  |  |  |  |  |  |  |  |  |  |  |  |  |  |  |  |  |  |  |  |  |  |  |  |  |  |  |  |  |  |  |  |  |  |  |  |  |  |  |  |  |  |  |  |  |  |  |  |  |  |  |  |  |  |  |  |  |  |  |  |  |  |  |  |  |  |  |  |  |  |  |  |  |  |  |  |  |  |  |  |  |  |  |  |  |  |  |  |  |  |  |  |  |  |  |  |  |  |  |  |  |  |  |  |  |  |  |  |  |  |  |  |  |  |  |  |  |  |  |  |  |  |  |  |  |  |  |  |  |  |  |  |  |  |  |  |  |  |  |  |  |  |  |  |  |  |  |  |  |  |  |  |  |  |  |  |  |  |  |  |  |  |  |  |  |  |  |  |  |  |  |  |  |  |  |  |  |  |  |  |  |  |  |  |  |  |  |  |  |  |  |  |  |  |  |  |  |  |  |  |  |  |  |  |  |  |  |  |  |  |  |  |  |  |  |  |  |  |  |  |  |  |  |  |  |  |  |  |  |  |  |  |  |  |  |  |  |  |  |  |  |  |  |  |  |  |  |  |  |  |  |  |  |  |  |  |  |  |  |  |  |  |  |  |  |  |  |  |  |  |  |  |  |  |  |  |  |  |  |  |  |  |  |  |  |  |  |  |  |  |  |  |  |  |  |  |  |  |  |  |  |  |  |  |  |  |  |  |  |  |  |  |  |  |  |  |  |  |  |  |  |  |  |  |  |  |  |  |  |  |  |  |  |  |  |  |  |  |  |  |  |  |  |  |  |  |  |  |  |  |  |  |  |  |  |  |  |  |  |  |  |  |  |  |  |  |  |  |  |  |  |  |  |  |  |  |  |  |  |  |  |  |  |  |  |  |  |  |  |  |  |  |  |  |  |  |  |  |  |  |  |  |  |  |  |  |  |  |  |  |  |  |  |  |  |  |  |  |  |  |  |  |  |  |  |  |  |  |  |  |  |  |  |  |  |  |  |  |  |  |  |  |  |  |  |  |  |  |  |  |  |  |  |  |  |  |  |  |  |  |  |  |  |  |  |  |  |  |  |  |  |  |  |  |  |  |  |  |  |  |  |  |  |  |  |  |  |  |  |  |  |  |  |  |  |  |  |  |  |  |  |  |  |  |  |  |  |  |  |  |  |  |  |  |  |  |  |  |  |  |  |  |  |  |  |  |  |  |  |  |  |  |  |  |  |  |  |  |  |  |  |  |  |  |  |  |  |  |  |  |  |  |  |  |  |  |  |  |  |  |  |  |  |  |  |  |  |  |  |  |  |  |  |  |  |  |  |  |  |  |  |  |  |  |  |  |  |  |  |  |  |  |  |  |  |  |  |  |  |  |  |  |  |  |  |  |  |  |  |  |  |  |  |  |  |  |  |  |  |  |  |  |  |  |  |  |  |  |  |  |  |  |  |  |  |  |  |  |  |  |  |  |  |  |  |  |  |  |  |  |  |  |  |  |  |  |  |  |  |  |  |  |  |  |  |  |  |  |  |  |  |  |  |  |  |  |  |  |  |  |  |  |  |  |  |  |  |  |  |  |  |  |  |  |  |  |  |  |  |  |  |  |  |  |  |  |  |  |  |  |  |  |  |  |  |  |  |  |  |  |  |  |  |  |  |  |  |  |  |  |  |  |  |  |  |  |  |  |  |  |  |  |  |  |  |  |  |  |  |  |  |  |  |  |  |  |  |  |  |  |  |  |  |  |  |  |  |  |  |  |  |  |  |  |  |  |  |  |  |  |  |  |  |  |  |  |  |  |  |  |  |  |  |  |  |  |  |  |  |  |  |  |  |  |  |  |  |  |  |  |  |  |  |  |  |  |  |  |  |  |  |  |  |  |  |  |  |  |  |  |  |  |  |  |  |  |  |  |  |  |  |  |  |  |  |  |  |  |  |  |  |  |  |  |  |  |  |  |  |  |  |  |  |  |  |  |  |  |  |  |  |  |  |  |  |  |  |  |  |  |  |  |  |  |  |  |  |  |  |  |  |  |  |  |  |  |  |  |  |  |  |  |  |  |  |  |  |  |  |  |  |  |  |  |  |  |  |  |  |  |  |  |  |  |  |  |  |  |  |  |  |  |  |  |  |  |  |  |  |  |  |  |  |  |  |  |  |  |  |  |  |  |  |  |  |  |  |  |  |  |  |  |  |  |  |  |  |  |  |  |  |  |  |  |  |  |  |  |  |  |  |  |  |  |  |  |  |  |  |  |  |  |  |  |  |  |  |  |  |  |  |  |  |  |  |  |  |  |  |  |  |  |  |  |  |  |  |  |  |  |  |  |  |  |  |  |  |  |  |  |  |  |  |  |  |  |  |  |  |  |  |  |  |  |  |  |  |  |  |  |  |  |  |  |  |  |  |  |  |  |  |  |  |  |  |  |  |  |  |  |  |  |  |  |  |  |  |  |  |  |  |  |  |  |  |  |  |  |  |  |  |  |  |  |  |  |  |  |  |  |  |  |  |  |  |  |  |  |  |  |  |  |  |  |  |  |  |  |  |  |  |  |  |  |  |  |  |  |  |  |  |  |  |  |  |  |  |  |  |  |  |  |  |  |  |  |  |  |  |  |  |  |  |  |  |  |  |  |  |  |  |  |  |  |  |  |  |  |  |  |  |  |  |  |  |  |  |  |  |  |  |  |  |  |  |  |  |  |  |  |  |  |  |  |  |  |  |  |  |  |  |  |  |  |  |  |  |  |  |  |  |  |  |  |  |  |  |  |  |  |  |  |  |  |  |  |  |  |  |  |  |  |  |  |  |  |  |  |  |  |  |  |  |  |  |  |  |  |  |  |  |  |  |  |  |  |  |  |  |  |  |  |  |  |  |  |  |  |  |  |  |  |  |  |  |  |  |  |  |  |  |  |  |  |  |  |  |  |  |  |  |  |  |  |  |  |  |  |  |  |  |  |  |  |  |  |  |  |  |  |  |  |  |  |  |  |  |  |  |  |  |  |  |  |  |  |  |  |  |  |  |  |  |  |  |  |  |  |  |  |  |  |  |  |  |  |  |  |  |  |  |  |  |  |  |  |  |  |  |  |  |  |  |  |  |  |  |  |  |  |  |  |  |  |  |  |  |  |  |  |  |  |  |  |  |  |  |  |  |  |  |  |  |  |  |  |  |  |  |  |  |  |  |  |  |  |  |  |  |  |  |  |  |  |  |  |  |  |  |  |  |  |  |  |  |  |  |  |  |  |  |  |  |  |  |  |  |  |  |  |  |  |  |  |  |  |  |  |  |  |  |  |  |  |  |  |  |  |  |  |  |  |  |  |  |  |  |  |  |  |  |  |  |  |  |  |  |  |  |  |  |  |  |  |  |  |  |  |  |  |  |  |  |  |  |  |  |  |  |  |  |  |  |  |  |  |  |  |  |  |  |  |  |  |  |  |  |  |  |  |  |  |  |  |  |  |  |  |  |  |  |  |  |  |  |  |  |  |  |  |  |  |  |  |  |  |  |  |  |  |  |  |  |  |  |  |  |  |  |  |  |  |  |  |  |  |  |  |  |  |  |  |  |  |  |  |  |  |  |  |  |  |  |  |  |  |  |  |  |  |  |  |  |  |  |  |  |  |  |  |  |  |  |  |  |  |  |  |  |  |  |  |  |  |  |  |  |  |  |  |  |  |  |  |  |  |  |  |  |  |  |  |  |  |  |  |  |  |  |  |  |  |  |  |  |  |  |  |  |  |  |  |  |  |  |  |  |  |  |  |  |  |  |  |  |  |  |  |  |  |  |  |  |  |  |  |  |  |  |  |  |  |  |  |  |  |  |  |  |  |  |  |  |  |  |  |  |  |  |  |  |  |  |  |  |  |  |  |  |  |  |  |  |  |  |  |  |  |  |  |  |  |  |  |  |  |  |  |  |  |  |  |  |  |  |  |  |  |  |  |  |  |  |  |  |  |  |  |  |  |  |  |  |  |  |  |  |  |  |  |  |  |  |  |  |  |  |  |  |  |  |  |  |  |  |  |  |  |  |  |  |  |  |  |  |  |  |  |  |  |  |  |  |  |  |  |  |  |  |  |  |  |  |  |  |  |  |  |  |  |  |  |  |  |  |  |  |  |  |  |  |  |  |  |  |  |  |  |  |  |  |  |  |  |  |  |  |  |  |  |  |  |  |  |  |  |  |  |  |  |  |  |  |  |  |  |  |  |  |  |  |  |  |  |  |  |  |  |  |  |  |  |  |  |  |  |  |  |  |  |  |  |  |  |  |  |  |  |  |  |  |  |  |  |  |  |  |  |  |  |  |  |  |  |  |  |  |  |  |  |  |  |  |  |  |  |  |  |  |  |  |  |  |  |  |  |  |  |  |  |  |  |  |  |  |  |  |  |  |  |  |  |  |  |  |  |  |  |  |  |  |  |  |  |  |  |  |  |  |  |  |  |  |  |  |  |  |  |  |  |  |  |  |  |  |  |  |  |  |  |  |  |  |  |  |  |  |  |  |  |  |  |  |  |  |  |  |  |  |  |  |  |  |  |  |  |  |  |  |  |  |  |  |  |  |  |  |  |  |  |  |  |  |  |  |  |  |  |  |  |  |  |  |  |  |  |  |  |  |  |  |  |  |  |  |  |  |  |  |  |  |  |  |  |  |  |  |  |  |  |  |  |  |  |  |  |  |  |  |  |  |  |  |  |  |  |  |  |  |  |  |  |  |  |  |  |  |  |  |  |  |  |  |  |  |  |  |  |  |  |  |  |  |  |  |  |  |  |  |  |  |  |  |  |  |  |  |  |  |  |  |  |  |  |  |  |  |  |  |  |  |  |  |  |  |  |  |  |  |  |  |  |  |  |  |  |  |  |  |  |  |  |  |  |  |  |  |  |  |  |  |  |  |  |  |  |  |  |  |  |  |  |  |  |  |  |  |  |  |  |  |  |  |  |  |  |  |  |  |  |  |  |  |  |  |  |  |  |  |  |  |  |  |  |  |  |  |  |  |  |  |  |  |  |  |  |  |  |  |  |  |  |  |  |  |  |  |  |  |  |  |  |  |  |  |  |  |  |  |  |  |  |  |  |  |  |  |  |  |  |  |  |  |  |  |  |  |  |  |  |  |  |  |  |  |  |  |  |  |  |  |  |  |  |  |  |  |  |  |  |  |  |  |  |  |  |  |  |  |  |  |  |  |  |  |  |  |  |  |  |  |  |  |  |  |  |  |  |  |  |  |  |  |  |  |  |  |  |  |  |  |  |  |  |  |  |  |  |  |  |  |  |  |  |  |  |  |  |  |  |  |  |  |  |  |  |  |  |  |  |  |  |  |  |  |  |  |  |  |  |  |  |  |  |  |  |  |  |  |  |  |  |  |  |  |  |  |  |  |  |  |  |  |  |  |  |  |  |  |  |  |  |  |  |  |  |  |  |  |  |  |  |  |  |  |  |  |  |  |  |  |  |  |  |  |  |  |  |  |  |  |  |  |  |  |  |  |  |  |  |  |  |  |  |  |  |  |  |  |  |  |  |  |  |  |  |  |  |  |  |  |  |  |  |  |  |  |  |  |  |  |  |  |  |  |  |  |  |  |  |  |  |  |  |  |  |  |  |  |  |  |  |  |  |  |  |  |  |  |  |  |  |  |  |  |  |  |  |  |  |  |  |  |  |  |  |  |  |  |  |  |  |  |  |  |  |  |  |  |  |  |  |  |  |  |  |  |  |  |  |  |  |  |  |  |  |  |  |  |  |  |  |  |  |  |  |  |  |  |  |  |  |  |  |  |  |  |  |  |  |  |  |  |  |  |  |  |  |  |  |  |  |  |  |  |  |  |  |  |  |  |  |  |  |  |  |  |  |  |  |  |  |  |  |  |  |  |  |  |  |  |  |  |  |  |  |  |  |  |  |  |  |  |  |  |  |  |  |  |  |  |  |  |  |  |  |  |  |  |  |  |  |  |  |  |  |  |  |  |  |  |  |  |  |  |  |  |  |  |  |  |  |  |  |  |  |  |  |  |  |  |  |  |  |  |  |  |  |  |  |  |  |  |  |  |  |  |  |  |  |  |  |  |  |  |  |  |  |  |  |  |  |  |  |  |  |  |  |  |  |  |  |  |  |  |  |  |  |  |  |  |  |  |  |  |  |  |  |  |  |  |  |  |  |  |  |  |  |  |  |  |  |  |  |  |  |  |  |  |  |  |  |  |  |  |  |  |  |  |  |  |  |  |  |  |  |  |  |  |  |  |  |  |  |  |  |  |  |  |  |  |  |  |  |  |  |  |  |  |  |  |  |  |  |  |  |  |  |  |  |  |  |  |  |  |  |  |  |  |  |  |  |  |  |  |  |  |  |  |  |  |  |  |  |  |  |  |  |  |  |  |  |  |  |  |  |  |  |  |  |  |  |  |  |  |  |  |  |  |  |  |  |  |  |  |  |  |  |  |  |  |  |  |  |  |  |  |  |  |  |  |  |  |  |  |  |  |  |  |  |  |  |  |  |  |  |  |  |  |  |  |  |  |  |  |  |  |  |  |  |  |  |  |  |  |  |  |  |  |  |  |  |  |  |  |  |  |  |  |  |  |  |  |  |  |  |  |  |  |  |  |  |  |  |  |  |  |  |  |  |  |  |  |  |  |  |  |  |  |  |  |  |  |  |  |  |  |  |  |  |  |  |  |  |  |  |  |  |  |  |  |  |  |  |  |  |  |  |  |  |  |  |  |  |  |  |  |  |  |  |  |  |  |  |  |  |  |  |  |  |  |  |  |  |  |  |  |  |  |  |  |  |  |  |  |  |  |  |  |  |  |  |  |  |  |  |  |  |  |  |  |  |  |  |  |  |  |  |  |  |  |  |  |  |  |  |  |  |  |  |  |  |  |  |  |  |  |  |  |  |  |  |  |  |  |  |  |  |  |  |  |  |  |  |  |  |  |  |  |  |  |  |  |  |  |  |  |  |  |  |  |  |  |  |  |  |  |  |  |  |  |  |  |  |  |  |  |  |  |  |  |  |  |  |  |  |  |  |  |  |  |  |  |  |  |  |  |  |  |  |  |  |  |  |  |  |  |  |  |  |  |  |  |  |  |  |  |  |  |  |  |  |  |  |  |  |  |  |  |  |  |  |  |  |  |  |  |  |  |  |  |  |  |  |  |  |  |  |  |  |  |  |  |  |  |  |  |  |  |  |  |  |  |  |  |  |  |  |  |  |  |  |  |  |  |  |  |  |  |  |  |  |  |  |  |  |  |  |  |  |  |  |  |  |  |  |  |  |  |  |  |  |  |  |  |  |  |  |  |  |  |  |  |  |  |  |  |  |  |  |  |  |  |  |  |  |  |  |  |  |  |  |  |  |  |  |  |  |  |  |  |  |  |  |  |  |  |  |  |  |  |  |  |  |  |  |  |  |  |  |  |  |  |  |  |  |  |  |  |  |  |  |  |  |  |  |  |  |  |  |  |  |  |  |  |  |  |  |  |  |  |  |  |  |  |  |  |  |  |  |  |  |  |  |  |  |  |  |  |  |  |  |  |  |  |  |  |  |  |  |  |  |  |  |  |  |  |  |  |  |  |  |  |  |  |  |  |  |  |  |  |  |  |  |  |  |  |  |  |  |  |  |  |  |  |  |  |  |  |  |  |  |  |  |  |  |  |  |  |  |  |  |  |  |  |  |  |  |  |  |  |  |  |  |  |  |  |  |  |  |  |  |  |  |  |  |  |  |  |  |  |  |  |  |  |  |  |  |  |  |  |  |  |  |  |  |  |  |  |  |  |  |  |  |  |  |  |  |  |  |  |  |  |  |  |  |  |  |  |  |  |  |  |  |  |  |  |  |  |  |  |  |  |  |  |  |  |  |  |  |  |  |  |  |  |  |  |  |  |  |  |  |  |  |  |  |  |  |  |  |  |  |  |  |  |  |  |  |  |  |  |  |  |  |  |  |  |  |  |  |  |  |  |  |  |  |  |  |  |  |  |  |  |  |  |  |  |  |  |  |  |  |  |  |  |  |  |  |  |  |  |  |  |  |  |  |  |  |  |  |  |  |  |  |  |  |  |  |  |  |  |  |  |  |  |  |  |  |  |  |  |  |  |  |  |  |  |  |  |  |  |  |  |  |  |  |  |  |  |  |  |  |  |  |  |  |  |  |  |  |  |  |  |  |  |  |  |  |  |  |  |  |  |  |  |  |  |  |  |  |  |  |  |  |  |  |  |  |  |  |  |  |  |  |  |  |  |  |  |  |  |  |  |  |  |  |  |  |  |  |  |  |  |  |  |  |  |  |  |  |  |  |  |  |  |  |  |  |  |  |  |  |  |  |  |  |  |  |  |  |  |  |  |  |  |  |  |  |  |  |  |  |  |  |  |  |  |  |  |  |  |  |  |  |  |  |  |  |  |  |  |  |  |  |  |  |  |  |  |  |  |  |  |  |  |  |  |  |  |  |  |  |  |  |  |  |  |  |  |  |  |  |  |  |  |  |  |  |  |  |  |  |  |  |  |  |  |  |  |  |  |  |  |  |  |  |  |  |  |  |  |  |  |  |  |  |  |  |  |  |  |  |  |  |  |  |  |  |  |  |  |  |  |  |  |  |  |  |  |  |  |  |  |  |  |  |  |  |  |  |  |  |  |  |  |  |  |  |  |  |  |  |  |  |  |  |  |  |  |  |  |  |  |  |  |  |  |  |  |  |  |  |  |  |  |  |  |  |  |  |  |  |  |  |  |  |  |  |  |  |  |  |  |  |  |  |  |  |  |  |  |  |  |  |  |  |  |  |  |  |  |  |  |  |  |  |  |  |  |  |  |  |  |  |  |  |  |  |  |  |  |  |  |  |  |  |  |  |  |  |  |  |  |  |  |  |  |  |  |  |  |  |  |  |  |  |  |  |  |  |  |  |  |  |  |  |  |  |  |  |  |  |  |  |  |  |  |  |  |  |  |  |  |  |  |  |  |  |  |  |  |  |  |  |  |  |  |  |  |  |  |  |  |  |  |  |  |  |  |  |  |  |  |  |  |  |  |  |  |  |  |  |  |  |  |  |  |  |  |  |  |  |  |  |  |  |  |  |  |  |  |  |  |  |  |  |  |  |  |  |  |  |  |  |  |  |  |  |  |  |  |  |  |  |  |  |  |  |  |  |  |  |  |  |  |  |  |  |  |  |  |  |  |  |  |  |  |  |  |  |  |  |  |  |  |  |  |  |  |  |  |  |  |  |  |  |  |  |  |  |  |  |  |  |  |  |  |  |  |  |  |  |  |  |  |  |  |  |  |  |  |  |  |  |  |  |  |  |  |  |  |  |  |  |  |  |  |  |  |  |  |  |  |  |  |  |  |  |  |  |  |  |  |  |  |  |  |  |  |  |  |  |  |  |  |  |  |  |  |  |  |  |  |  |  |  |  |  |  |  |  |  |  |  |  |  |  |  |  |  |  |  |  |  |  |  |  |  |  |  |  |  |  |  |  |  |  |  |  |  |  |  |  |  |  |  |  |  |  |  |  |  |  |  |  |  |  |  |  |  |  |  |  |  |  |  |  |  |  |  |  |  |  |  |  |  |  |  |  |  |  |  |  |  |  |  |  |  |  |  |  |  |  |  |  |  |  |  |  |  |  |  |  |  |  |  |  |  |  |  |  |  |  |  |  |  |  |  |  |  |  |  |  |  |  |  |  |  |  |  |  |  |  |  |  |  |  |  |  |  |  |  |  |  |  |  |  |  |  |  |  |  |  |  |  |  |  |  |  |  |  |  |  |  |  |  |  |  |  |  |  |  |  |  |  |  |  |  |  |  |  |  |  |  |  |  |  |  |  |  |  |  |  |  |  |  |  |  |  |  |  |  |  |  |  |  |  |  |  |  |  |  |  |  |  |  |  |  |  |  |  |  |  |  |  |  |  |  |  |  |  |  |  |  |  |  |  |  |  |  |  |  |  |  |  |  |  |  |  |  |  |  |  |  |  |  |  |  |  |  |  |  |  |  |  |  |  |  |  |  |  |  |  |  |  |  |  |  |  |  |  |  |  |  |  |  |  |  |  |  |  |  |  |  |  |  |  |  |  |  |  |  |  |  |  |  |  |  |  |  |  |  |  |  |  |  |  |  |  |  |  |  |  |  |  |  |  |  |  |  |  |  |  |  |  |  |  |  |  |  |  |  |  |  |  |  |  |  |  |  |  |  |  |  |  |  |  |  |  |  |  |  |  |  |  |  |  |  |  |  |  |  |  |  |  |  |  |  |  |  |  |  |  |  |  |  |  |  |  |  |  |  |  |  |  |  |  |  |  |  |  |  |  |  |  |  |  |  |  |  |  |  |  |  |  |  |  |  |  |  |  |  |  |  |  |  |  |  |  |  |  |  |  |  |  |  |  |  |  |  |  |  |  |  |  |  |  |  |  |  |  |  |  |  |  |  |  |  |  |  |  |  |  |  |  |  |  |  |  |  |  |  |  |  |  |  |  |  |  |  |  |  |  |  |  |  |  |  |  |  |  |  |  |  |  |  |  |  |  |  |  |  |  |  |  |  |  |  |  |  |  |  |  |  |  |  |  |  |  |  |  |  |  |  |  |  |  |  |  |  |  |  |  |  |  |  |  |  |  |  |  |  |  |  |  |  |  |  |  |  |  |  |  |  |  |  |  |  |  |  |  |  |  |  |  |  |  |  |  |  |  |  |  |  |  |  |  |  |  |  |  |  |  |  |  |  |  |  |  |  |  |  |  |  |  |  |  |  |  |  |  |  |  |  |  |  |  |  |  |  |  |  |  |  |  |  |  |  |  |  |  |  |  |  |  |  |  |  |  |  |  |  |  |  |  |  |  |  |  |  |  |  |  |  |  |  |  |  |  |  |  |  |  |  |  |  |  |  |  |  |  |  |  |  |  |  |  |  |  |  |  |  |  |  |  |  |  |  |  |  |  |  |  |  |  |  |  |  |  |  |  |  |  |  |  |  |  |  |  |  |  |  |  |  |  |  |  |  |  |  |  |  |  |  |  |  |  |  |  |  |  |  |  |  |  |  |  |  |  |  |  |  |  |  |  |  |  |  |  |  |  |  |  |  |  |  |  |  |  |  |  |  |  |  |  |  |  |  |  |  |  |  |  |  |  |  |  |  |  |  |  |  |  |  |  |  |  |  |  |  |  |  |  |  |  |  |  |  |  |  |  |  |  |  |  |  |  |  |  |  |  |  |  |  |  |  |  |  |  |  |  |  |  |  |  |  |  |  |  |  |  |  |  |  |  |  |  |  |  |  |  |  |  |  |  |  |  |  |  |  |  |  |  |  |  |  |  |  |  |  |  |  |  |  |  |  |  |  |  |  |  |  |  |  |  |  |  |  |  |  |  |  |  |  |  |  |  |  |  |  |  |  |  |  |  |  |  |  |  |  |  |  |  |  |  |  |  |  |  |  |  |  |  |  |  |  |  |  |  |  |  |  |  |  |  |  |  |  |  |  |  |  |  |  |  |  |  |  |  |  |  |  |  |  |  |  |  |  |  |  |  |  |  |  |  |  |  |  |  |  |  |  |  |  |  |  |  |  |  |  |  |  |  |  |  |  |  |  |  |  |  |  |  |  |  |  |  |  |  |  |  |  |  |  |  |  |  |  |  |  |  |  |  |  |  |  |  |  |  |  |  |  |  |  |  |  |  |  |  |  |  |  |  |  |  |  |  |  |  |  |  |  |  |  |  |  |  |  |  |  |  |  |  |  |  |  |  |  |  |  |  |  |  |  |  |  |  |  |  |  |  |  |  |  |  |  |  |  |  |  |  |  |  |  |  |  |  |  |  |  |  |  |  |  |  |  |  |  |  |  |  |  |  |  |  |  |  |  |  |  |  |  |  |  |  |  |  |  |  |  |  |  |  |  |  |  |  |  |  |  |  |  |  |  |  |  |  |  |  |  |  |  |  |  |  |  |  |  |  |  |  |  |  |  |  |  |  |  |  |  |  |  |  |  |  |  |  |  |  |  |  |  |  |  |  |  |  |  |  |  |  |  |  |  |  |  |  |  |  |  |  |  |  |  |  |  |  |  |  |  |  |  |  |  |  |  |  |  |  |  |  |  |  |  |  |  |  |  |  |  |  |  |  |  |  |  |  |  |  |  |  |  |  |  |  |  |  |  |  |  |  |  |  |  |  |  |  |  |  |  |  |  |  |  |  |  |  |  |  |  |  |  |  |  |  |  |  |  |  |  |  |  |  |  |  |  |  |  |  |  |  |  |  |  |  |  |  |  |  |  |  |  |  |  |  |  |  |  |  |  |  |  |  |  |  |  |  |  |  |  |  |  |  |  |  |  |  |  |  |  |  |  |  |  |  |  |  |  |  |  |  |  |  |  |  |  |  |  |  |  |  |  |  |  |  |  |  |  |  |
| --- | --- | --- | --- | --- | --- | --- | --- | --- | --- | --- | --- | --- | --- | --- | --- | --- | --- | --- | --- | --- | --- | --- | --- | --- | --- | --- | --- | --- | --- | --- | --- | --- | --- | --- | --- | --- | --- | --- | --- | --- | --- | --- | --- | --- | --- | --- | --- | --- | --- | --- | --- | --- | --- | --- | --- | --- | --- | --- | --- | --- | --- | --- | --- | --- | --- | --- | --- | --- | --- | --- | --- | --- | --- | --- | --- | --- | --- | --- | --- | --- | --- | --- | --- | --- | --- | --- | --- | --- | --- | --- | --- | --- | --- | --- | --- | --- | --- | --- | --- | --- | --- | --- | --- | --- | --- | --- | --- | --- | --- | --- | --- | --- | --- | --- | --- | --- | --- | --- | --- | --- | --- | --- | --- | --- | --- | --- | --- | --- | --- | --- | --- | --- | --- | --- | --- | --- | --- | --- | --- | --- | --- | --- | --- | --- | --- | --- | --- | --- | --- | --- | --- | --- | --- | --- | --- | --- | --- | --- | --- | --- | --- | --- | --- | --- | --- | --- | --- | --- | --- | --- | --- | --- | --- | --- | --- | --- | --- | --- | --- | --- | --- | --- | --- | --- | --- | --- | --- | --- | --- | --- | --- | --- | --- | --- | --- | --- | --- | --- | --- | --- | --- | --- | --- | --- | --- | --- | --- | --- | --- | --- | --- | --- | --- | --- | --- | --- | --- | --- | --- | --- | --- | --- | --- | --- | --- | --- | --- | --- | --- | --- | --- | --- | --- | --- | --- | --- | --- | --- | --- | --- | --- | --- | --- | --- | --- | --- | --- | --- | --- | --- | --- | --- | --- | --- | --- | --- | --- | --- | --- | --- | --- | --- | --- | --- | --- | --- | --- | --- | --- | --- | --- | --- | --- | --- | --- | --- | --- | --- | --- | --- | --- | --- | --- | --- | --- | --- | --- | --- | --- | --- | --- | --- | --- | --- | --- | --- | --- | --- | --- | --- | --- | --- | --- | --- | --- | --- | --- | --- | --- | --- | --- | --- | --- | --- | --- | --- | --- | --- | --- | --- | --- | --- | --- | --- | --- | --- | --- | --- | --- | --- | --- | --- | --- | --- | --- | --- | --- | --- | --- | --- | --- | --- | --- | --- | --- | --- | --- | --- | --- | --- | --- | --- | --- | --- | --- | --- | --- | --- | --- | --- | --- | --- | --- | --- | --- | --- | --- | --- | --- | --- | --- | --- | --- | --- | --- | --- | --- | --- | --- | --- | --- | --- | --- | --- | --- | --- | --- | --- | --- | --- | --- | --- | --- | --- | --- | --- | --- | --- | --- | --- | --- | --- | --- | --- | --- | --- | --- | --- | --- | --- | --- | --- | --- | --- | --- | --- | --- | --- | --- | --- | --- | --- | --- | --- | --- | --- | --- | --- | --- | --- | --- | --- | --- | --- | --- | --- | --- | --- | --- | --- | --- | --- | --- | --- | --- | --- | --- | --- | --- | --- | --- | --- | --- | --- | --- | --- | --- | --- | --- | --- | --- | --- | --- | --- | --- | --- | --- | --- | --- | --- | --- | --- | --- | --- | --- | --- | --- | --- | --- | --- | --- | --- | --- | --- | --- | --- | --- | --- | --- | --- | --- | --- | --- | --- | --- | --- | --- | --- | --- | --- | --- | --- | --- | --- | --- | --- | --- | --- | --- | --- | --- | --- | --- | --- | --- | --- | --- | --- | --- | --- | --- | --- | --- | --- | --- | --- | --- | --- | --- | --- | --- | --- | --- | --- | --- | --- | --- | --- | --- | --- | --- | --- | --- | --- | --- | --- | --- | --- | --- | --- | --- | --- | --- | --- | --- | --- | --- | --- | --- | --- | --- | --- | --- | --- | --- | --- | --- | --- | --- | --- | --- | --- | --- | --- | --- | --- | --- | --- | --- | --- | --- | --- | --- | --- | --- | --- | --- | --- | --- | --- | --- | --- | --- | --- | --- | --- | --- | --- | --- | --- | --- | --- | --- | --- | --- | --- | --- | --- | --- | --- | --- | --- | --- | --- | --- | --- | --- | --- | --- | --- | --- | --- | --- | --- | --- | --- | --- | --- | --- | --- | --- | --- | --- | --- | --- | --- | --- | --- | --- | --- | --- | --- | --- | --- | --- | --- | --- | --- | --- | --- | --- | --- | --- | --- | --- | --- | --- | --- | --- | --- | --- | --- | --- | --- | --- | --- | --- | --- | --- | --- | --- | --- | --- | --- | --- | --- | --- | --- | --- | --- | --- | --- | --- | --- | --- | --- | --- | --- | --- | --- | --- | --- | --- | --- | --- | --- | --- | --- | --- | --- | --- | --- | --- | --- | --- | --- | --- | --- | --- | --- | --- | --- | --- | --- | --- | --- | --- | --- | --- | --- | --- | --- | --- | --- | --- | --- | --- | --- | --- | --- | --- | --- | --- | --- | --- | --- | --- | --- | --- | --- | --- | --- | --- | --- | --- | --- | --- | --- | --- | --- | --- | --- | --- | --- | --- | --- | --- | --- | --- | --- | --- | --- | --- | --- | --- | --- | --- | --- | --- | --- | --- | --- | --- | --- | --- | --- | --- | --- | --- | --- | --- | --- | --- | --- | --- | --- | --- | --- | --- | --- | --- | --- | --- | --- | --- | --- | --- | --- | --- | --- | --- | --- | --- | --- | --- | --- | --- | --- | --- | --- | --- | --- | --- | --- | --- | --- | --- | --- | --- | --- | --- | --- | --- | --- | --- | --- | --- | --- | --- | --- | --- | --- | --- | --- | --- | --- | --- | --- | --- | --- | --- | --- | --- | --- | --- | --- | --- | --- | --- | --- | --- | --- | --- | --- | --- | --- | --- | --- | --- | --- | --- | --- | --- | --- | --- | --- | --- | --- | --- | --- | --- | --- | --- | --- | --- | --- | --- | --- | --- | --- | --- | --- | --- | --- | --- | --- | --- | --- | --- | --- | --- | --- | --- | --- | --- | --- | --- | --- | --- | --- | --- | --- | --- | --- | --- | --- | --- | --- | --- | --- | --- | --- | --- | --- | --- | --- | --- | --- | --- | --- | --- | --- | --- | --- | --- | --- | --- | --- | --- | --- | --- | --- | --- | --- | --- | --- | --- | --- | --- | --- | --- | --- | --- | --- | --- | --- | --- | --- | --- | --- | --- | --- | --- | --- | --- | --- | --- | --- | --- | --- | --- | --- | --- | --- | --- | --- | --- | --- | --- | --- | --- | --- | --- | --- | --- | --- | --- | --- | --- | --- | --- | --- | --- | --- | --- | --- | --- | --- | --- | --- | --- | --- | --- | --- | --- | --- | --- | --- | --- | --- | --- | --- | --- | --- | --- | --- | --- | --- | --- | --- | --- | --- | --- | --- | --- | --- | --- | --- | --- | --- | --- | --- | --- | --- | --- | --- | --- | --- | --- | --- | --- | --- | --- | --- | --- | --- | --- | --- | --- | --- | --- | --- | --- | --- | --- | --- | --- | --- | --- | --- | --- | --- | --- | --- | --- | --- | --- | --- | --- | --- | --- | --- | --- | --- | --- | --- | --- | --- | --- | --- | --- | --- | --- | --- | --- | --- | --- | --- | --- | --- | --- | --- | --- | --- | --- | --- | --- | --- | --- | --- | --- | --- | --- | --- | --- | --- | --- | --- | --- | --- | --- | --- | --- | --- | --- | --- | --- | --- | --- | --- | --- | --- | --- | --- | --- | --- | --- | --- | --- | --- | --- | --- | --- | --- | --- | --- | --- | --- | --- | --- | --- | --- | --- | --- | --- | --- | --- | --- | --- | --- | --- | --- | --- | --- | --- | --- | --- | --- | --- | --- | --- | --- | --- | --- | --- | --- | --- | --- | --- | --- | --- | --- | --- | --- | --- | --- | --- | --- | --- | --- | --- | --- | --- | --- | --- | --- | --- | --- | --- | --- | --- | --- | --- | --- | --- | --- | --- | --- | --- | --- | --- | --- | --- | --- | --- | --- | --- | --- | --- | --- | --- | --- | --- | --- | --- | --- | --- | --- | --- | --- | --- | --- | --- | --- | --- | --- | --- | --- | --- | --- | --- | --- | --- | --- | --- | --- | --- | --- | --- | --- | --- | --- | --- | --- | --- | --- | --- | --- | --- | --- | --- | --- | --- | --- | --- | --- | --- | --- | --- | --- | --- | --- | --- | --- | --- | --- | --- | --- | --- | --- | --- | --- | --- | --- | --- | --- | --- | --- | --- | --- | --- | --- | --- | --- | --- | --- | --- | --- | --- | --- | --- | --- | --- | --- | --- | --- | --- | --- | --- | --- | --- | --- | --- | --- | --- | --- | --- | --- | --- | --- | --- | --- | --- | --- | --- | --- | --- | --- | --- | --- | --- | --- | --- | --- | --- | --- | --- | --- | --- | --- | --- | --- | --- | --- | --- | --- | --- | --- | --- | --- | --- | --- | --- | --- | --- | --- | --- | --- | --- | --- | --- | --- | --- | --- | --- | --- | --- | --- | --- | --- | --- | --- | --- | --- | --- | --- | --- | --- | --- | --- | --- | --- | --- | --- | --- | --- | --- | --- | --- | --- | --- | --- | --- | --- | --- | --- | --- | --- | --- | --- | --- | --- | --- | --- | --- | --- | --- | --- | --- | --- | --- | --- | --- | --- | --- | --- | --- | --- | --- | --- | --- | --- | --- | --- | --- | --- | --- | --- | --- | --- | --- | --- | --- | --- | --- | --- | --- | --- | --- | --- | --- | --- | --- | --- | --- | --- | --- | --- | --- | --- | --- | --- | --- | --- | --- | --- | --- | --- | --- | --- | --- | --- | --- | --- | --- | --- | --- | --- | --- | --- | --- | --- | --- | --- | --- | --- | --- | --- | --- | --- | --- | --- | --- | --- | --- | --- | --- | --- | --- | --- | --- | --- | --- | --- | --- | --- | --- | --- | --- | --- | --- | --- | --- | --- | --- | --- | --- | --- | --- | --- | --- | --- | --- | --- | --- | --- | --- | --- | --- | --- | --- | --- | --- | --- | --- | --- | --- | --- | --- | --- | --- | --- | --- | --- | --- | --- | --- | --- | --- | --- | --- | --- | --- | --- | --- | --- | --- | --- | --- | --- | --- | --- | --- | --- | --- | --- | --- | --- | --- | --- | --- | --- | --- | --- | --- | --- | --- | --- | --- | --- | --- | --- | --- | --- | --- | --- | --- | --- | --- | --- | --- | --- | --- | --- | --- | --- | --- | --- | --- | --- | --- | --- | --- | --- | --- | --- | --- | --- | --- | --- | --- | --- | --- | --- | --- | --- | --- | --- | --- | --- | --- | --- | --- | --- | --- | --- | --- | --- | --- | --- | --- | --- | --- | --- | --- | --- | --- | --- | --- | --- | --- | --- | --- | --- | --- | --- | --- | --- | --- | --- | --- | --- | --- | --- | --- | --- | --- | --- | --- | --- | --- | --- | --- | --- | --- | --- | --- | --- | --- | --- | --- | --- | --- | --- | --- | --- | --- | --- | --- | --- | --- | --- | --- | --- | --- | --- | --- | --- | --- | --- | --- | --- | --- | --- | --- | --- | --- | --- | --- | --- | --- | --- | --- | --- | --- | --- | --- | --- | --- | --- | --- | --- | --- | --- | --- | --- | --- | --- | --- | --- | --- | --- | --- | --- | --- | --- | --- | --- | --- | --- | --- | --- | --- | --- | --- | --- | --- | --- | --- | --- | --- | --- | --- | --- | --- | --- | --- | --- | --- | --- | --- | --- | --- | --- | --- | --- | --- | --- | --- | --- | --- | --- | --- | --- | --- | --- | --- | --- | --- | --- | --- | --- | --- | --- | --- | --- | --- | --- | --- | --- | --- | --- | --- | --- | --- | --- | --- | --- | --- | --- | --- | --- | --- | --- | --- | --- | --- | --- | --- | --- | --- | --- | --- | --- | --- | --- | --- | --- | --- | --- | --- | --- | --- | --- | --- | --- | --- | --- | --- | --- | --- | --- | --- | --- | --- | --- | --- | --- | --- | --- | --- | --- | --- | --- | --- | --- | --- | --- | --- | --- | --- | --- | --- | --- | --- | --- | --- | --- | --- | --- | --- | --- | --- | --- | --- | --- | --- | --- | --- | --- | --- | --- | --- | --- | --- | --- | --- | --- | --- | --- | --- | --- | --- | --- | --- | --- | --- | --- | --- | --- | --- | --- | --- | --- | --- | --- | --- | --- | --- | --- | --- | --- | --- | --- | --- | --- | --- | --- | --- | --- | --- | --- | --- | --- | --- | --- | --- | --- | --- | --- | --- | --- | --- | --- | --- | --- | --- | --- | --- | --- | --- | --- | --- | --- | --- | --- | --- | --- | --- | --- | --- | --- | --- | --- | --- | --- | --- | --- | --- | --- | --- | --- | --- | --- | --- | --- | --- | --- | --- | --- | --- | --- | --- | --- | --- | --- | --- | --- | --- | --- | --- | --- | --- | --- | --- | --- | --- | --- | --- | --- | --- | --- | --- | --- | --- | --- | --- | --- | --- | --- | --- | --- | --- | --- | --- | --- | --- | --- | --- | --- | --- | --- | --- | --- | --- | --- | --- | --- | --- | --- | --- | --- | --- | --- | --- | --- | --- | --- | --- | --- | --- | --- | --- | --- | --- | --- | --- | --- | --- | --- | --- | --- | --- | --- | --- | --- | --- | --- | --- | --- | --- | --- | --- | --- | --- | --- | --- | --- | --- | --- | --- | --- | --- | --- | --- | --- | --- | --- | --- | --- | --- | --- | --- | --- | --- | --- | --- | --- | --- | --- | --- | --- | --- | --- | --- | --- | --- | --- | --- | --- | --- | --- | --- | --- | --- | --- | --- | --- | --- | --- | --- | --- | --- | --- | --- | --- | --- | --- | --- | --- | --- | --- | --- | --- | --- | --- | --- | --- | --- | --- | --- | --- | --- | --- | --- | --- | --- | --- | --- | --- | --- | --- | --- | --- | --- | --- | --- | --- | --- | --- | --- | --- | --- | --- | --- | --- | --- | --- | --- | --- | --- | --- | --- | --- | --- | --- | --- | --- | --- | --- | --- | --- | --- | --- | --- | --- | --- | --- | --- | --- | --- | --- | --- | --- | --- | --- | --- | --- | --- | --- | --- | --- | --- | --- | --- | --- | --- | --- | --- | --- | --- | --- | --- | --- | --- | --- | --- | --- | --- | --- | --- | --- | --- | --- | --- | --- | --- | --- | --- | --- | --- | --- | --- | --- | --- | --- | --- | --- | --- | --- | --- | --- | --- | --- | --- | --- | --- | --- | --- | --- | --- | --- | --- | --- | --- | --- | --- | --- | --- | --- | --- | --- | --- | --- | --- | --- | --- | --- | --- | --- | --- | --- | --- | --- | --- | --- | --- | --- | --- | --- | --- | --- | --- | --- | --- | --- | --- | --- | --- | --- | --- | --- | --- | --- | --- | --- | --- | --- | --- | --- | --- | --- | --- | --- | --- | --- | --- | --- | --- | --- | --- | --- | --- | --- | --- | --- | --- | --- | --- | --- | --- | --- | --- | --- | --- | --- | --- | --- | --- | --- | --- | --- | --- | --- | --- | --- | --- | --- | --- | --- | --- | --- | --- | --- | --- | --- | --- | --- | --- | --- | --- | --- | --- | --- | --- | --- | --- | --- | --- | --- | --- | --- | --- | --- | --- | --- | --- | --- | --- | --- | --- | --- | --- | --- | --- | --- | --- | --- | --- | --- | --- | --- | --- | --- | --- | --- | --- | --- | --- | --- | --- | --- | --- | --- | --- | --- | --- | --- | --- | --- | --- | --- | --- | --- | --- | --- | --- | --- | --- | --- | --- | --- | --- | --- | --- | --- | --- | --- | --- | --- | --- | --- | --- | --- | --- | --- | --- | --- | --- | --- | --- | --- | --- | --- | --- | --- | --- | --- | --- | --- | --- | --- | --- | --- | --- | --- | --- | --- | --- | --- | --- | --- | --- | --- | --- | --- | --- | --- | --- | --- | --- | --- | --- | --- | --- | --- | --- | --- | --- | --- | --- | --- | --- | --- | --- | --- | --- | --- | --- | --- | --- | --- | --- | --- | --- | --- | --- | --- | --- | --- | --- | --- | --- | --- | --- | --- | --- | --- | --- | --- | --- | --- | --- | --- | --- | --- | --- | --- | --- | --- | --- | --- | --- | --- | --- | --- | --- | --- | --- | --- | --- | --- | --- | --- | --- | --- | --- | --- | --- | --- | --- | --- | --- | --- | --- | --- | --- | --- | --- | --- | --- | --- | --- | --- | --- | --- | --- | --- | --- | --- | --- | --- | --- | --- | --- | --- | --- | --- | --- | --- | --- | --- | --- | --- | --- | --- | --- | --- | --- | --- | --- | --- | --- | --- | --- | --- | --- | --- | --- | --- | --- | --- | --- | --- | --- | --- | --- | --- | --- | --- | --- | --- | --- | --- | --- | --- | --- | --- | --- | --- | --- | --- | --- | --- | --- | --- | --- | --- | --- | --- | --- | --- | --- | --- | --- | --- | --- | --- | --- | --- | --- | --- | --- | --- | --- | --- | --- | --- | --- | --- | --- | --- | --- | --- | --- | --- | --- | --- | --- | --- | --- | --- | --- | --- | --- | --- | --- | --- | --- | --- | --- | --- | --- | --- | --- | --- | --- | --- | --- | --- | --- | --- | --- | --- | --- | --- | --- | --- | --- | --- | --- | --- | --- | --- | --- | --- | --- | --- | --- | --- | --- | --- | --- | --- | --- | --- | --- | --- | --- | --- | --- | --- | --- | --- | --- | --- | --- | --- | --- | --- | --- | --- | --- | --- | --- | --- | --- | --- | --- | --- | --- | --- | --- | --- | --- | --- | --- | --- | --- | --- | --- | --- | --- | --- | --- | --- | --- | --- | --- | --- | --- | --- | --- | --- | --- | --- | --- | --- | --- | --- | --- | --- | --- | --- | --- | --- | --- | --- | --- | --- | --- | --- | --- | --- | --- | --- | --- | --- | --- | --- | --- | --- | --- | --- | --- | --- | --- | --- | --- | --- | --- | --- | --- | --- | --- | --- | --- | --- | --- | --- | --- | --- | --- | --- | --- | --- | --- | --- | --- | --- | --- | --- | --- | --- | --- | --- | --- | --- | --- | --- | --- | --- | --- | --- | --- | --- | --- | --- | --- | --- | --- | --- | --- | --- | --- | --- | --- | --- | --- | --- | --- | --- | --- | --- | --- | --- | --- | --- | --- | --- | --- | --- | --- | --- | --- | --- | --- | --- | --- | --- | --- | --- | --- | --- | --- | --- | --- | --- | --- | --- | --- | --- | --- | --- | --- | --- | --- | --- | --- | --- | --- | --- | --- | --- | --- | --- | --- | --- | --- | --- | --- | --- | --- | --- | --- | --- | --- | --- | --- | --- | --- | --- | --- | --- | --- | --- | --- | --- | --- | --- | --- | --- | --- | --- | --- | --- | --- | --- | --- | --- | --- | --- | --- | --- | --- | --- | --- | --- | --- | --- | --- | --- | --- | --- | --- | --- | --- | --- | --- | --- | --- | --- | --- | --- | --- | --- | --- | --- | --- | --- | --- | --- | --- | --- | --- | --- | --- | --- | --- | --- | --- | --- | --- | --- | --- | --- | --- | --- | --- | --- | --- | --- | --- | --- | --- | --- | --- | --- | --- | --- | --- | --- | --- | --- | --- | --- | --- | --- | --- | --- | --- | --- | --- | --- | --- | --- | --- | --- | --- | --- | --- | --- | --- | --- | --- | --- | --- | --- | --- | --- | --- | --- | --- | --- | --- | --- | --- | --- | --- | --- | --- | --- | --- | --- | --- | --- | --- | --- | --- | --- | --- | --- | --- | --- | --- | --- | --- | --- | --- | --- | --- | --- | --- | --- | --- | --- | --- | --- | --- | --- | --- | --- | --- | --- | --- | --- | --- | --- | --- | --- | --- | --- | --- | --- | --- | --- | --- | --- | --- | --- | --- | --- | --- | --- | --- | --- | --- | --- | --- | --- | --- | --- | --- | --- | --- | --- | --- | --- | --- | --- | --- | --- | --- | --- | --- | --- | --- | --- | --- | --- | --- | --- | --- | --- | --- | --- | --- | --- | --- | --- | --- | --- | --- | --- | --- | --- | --- | --- | --- | --- | --- | --- | --- | --- | --- | --- | --- | --- | --- | --- | --- | --- | --- | --- | --- | --- | --- | --- | --- | --- | --- | --- | --- | --- | --- | --- | --- | --- | --- | --- | --- | --- | --- | --- | --- | --- | --- | --- | --- | --- | --- | --- | --- | --- | --- | --- | --- | --- | --- | --- | --- | --- | --- | --- | --- | --- | --- | --- | --- | --- | --- | --- | --- | --- | --- | --- | --- | --- | --- | --- | --- | --- | --- | --- | --- | --- | --- | --- | --- | --- | --- | --- | --- | --- | --- | --- | --- | --- | --- | --- | --- | --- | --- | --- | --- | --- | --- | --- | --- | --- | --- | --- | --- | --- | --- | --- | --- | --- | --- | --- | --- | --- | --- | --- | --- | --- | --- | --- | --- | --- | --- | --- | --- | --- | --- | --- | --- | --- | --- | --- | --- | --- | --- | --- | --- | --- | --- | --- | --- | --- | --- | --- | --- | --- | --- | --- | --- | --- | --- | --- | --- | --- | --- | --- | --- | --- | --- | --- | --- | --- | --- | --- | --- | --- | --- | --- | --- | --- | --- | --- | --- | --- | --- | --- | --- | --- | --- | --- | --- | --- | --- | --- | --- | --- | --- | --- | --- | --- | --- | --- | --- | --- | --- | --- | --- | --- | --- | --- | --- | --- | --- | --- | --- | --- | --- | --- | --- | --- | --- | --- | --- | --- | --- | --- | --- | --- | --- | --- | --- | --- | --- | --- | --- | --- | --- | --- | --- | --- | --- | --- | --- | --- | --- | --- | --- | --- | --- | --- | --- | --- | --- | --- | --- | --- | --- | --- | --- | --- | --- | --- | --- | --- | --- | --- | --- | --- | --- | --- | --- | --- | --- | --- | --- | --- | --- | --- | --- | --- | --- | --- | --- | --- | --- | --- | --- | --- | --- | --- | --- | --- | --- | --- | --- | --- | --- | --- | --- | --- | --- | --- | --- | --- | --- | --- | --- | --- | --- | --- | --- | --- | --- | --- | --- | --- | --- | --- | --- | --- | --- | --- | --- | --- | --- | --- | --- | --- | --- | --- | --- | --- | --- | --- | --- | --- | --- | --- | --- | --- | --- | --- | --- | --- | --- | --- | --- | --- | --- | --- | --- | --- | --- | --- | --- | --- | --- | --- | --- | --- | --- | --- | --- | --- | --- | --- | --- | --- | --- | --- | --- | --- | --- | --- | --- | --- | --- | --- | --- | --- | --- | --- | --- | --- | --- | --- | --- | --- | --- | --- | --- | --- | --- | --- | --- | --- | --- | --- | --- | --- | --- | --- | --- | --- | --- | --- | --- | --- | --- | --- | --- | --- | --- | --- | --- | --- | --- | --- | --- | --- | --- | --- | --- | --- | --- | --- | --- | --- | --- | --- | --- | --- | --- | --- | --- | --- | --- | --- | --- | --- | --- | --- | --- | --- | --- | --- | --- | --- | --- | --- | --- | --- | --- | --- | --- | --- | --- | --- | --- | --- | --- | --- | --- | --- | --- | --- | --- | --- | --- | --- | --- | --- | --- | --- | --- | --- | --- | --- | --- | --- | --- | --- | --- | --- | --- | --- | --- | --- | --- | --- | --- | --- | --- | --- | --- | --- | --- | --- | --- | --- | --- | --- | --- | --- | --- | --- | --- | --- | --- | --- | --- | --- | --- | --- | --- | --- | --- | --- | --- | --- | --- | --- | --- | --- | --- | --- | --- | --- | --- | --- | --- | --- | --- | --- | --- | --- | --- | --- | --- | --- | --- | --- | --- | --- | --- | --- | --- | --- | --- | --- | --- | --- | --- | --- | --- | --- | --- | --- | --- | --- | --- | --- | --- | --- | --- | --- | --- | --- | --- | --- | --- | --- | --- | --- | --- | --- | --- | --- | --- | --- | --- | --- | --- | --- | --- | --- | --- | --- | --- | --- | --- | --- | --- | --- | --- | --- | --- | --- | --- | --- | --- | --- | --- | --- | --- | --- | --- | --- | --- | --- | --- | --- | --- | --- | --- | --- | --- | --- | --- | --- | --- | --- | --- | --- | --- | --- | --- | --- | --- | --- | --- | --- | --- | --- | --- | --- | --- | --- | --- | --- | --- | --- | --- | --- | --- | --- | --- | --- | --- | --- | --- | --- | --- | --- | --- | --- | --- | --- | --- | --- | --- | --- | --- | --- | --- | --- | --- | --- | --- | --- | --- | --- | --- | --- | --- | --- | --- | --- | --- | --- | --- | --- | --- | --- | --- | --- | --- | --- | --- | --- | --- | --- | --- | --- | --- | --- | --- | --- | --- | --- | --- | --- | --- | --- | --- | --- | --- | --- | --- | --- | --- | --- | --- | --- | --- | --- | --- | --- | --- | --- | --- | --- | --- | --- | --- | --- | --- | --- | --- | --- | --- | --- | --- | --- | --- | --- | --- | --- | --- | --- | --- | --- | --- | --- | --- | --- | --- | --- | --- | --- | --- | --- | --- | --- | --- | --- | --- | --- | --- | --- | --- | --- | --- | --- | --- | --- | --- | --- | --- | --- | --- | --- | --- | --- | --- | --- | --- | --- | --- | --- | --- | --- | --- | --- | --- | --- | --- | --- | --- | --- | --- | --- | --- | --- | --- | --- | --- | --- | --- | --- | --- | --- | --- | --- | --- | --- | --- | --- | --- | --- | --- | --- | --- | --- | --- | --- | --- | --- | --- | --- | --- | --- | --- | --- | --- | --- | --- | --- | --- | --- | --- | --- | --- | --- | --- | --- | --- | --- | --- | --- | --- | --- | --- | --- | --- | --- | --- | --- | --- | --- | --- | --- | --- | --- | --- | --- | --- | --- | --- | --- | --- | --- | --- | --- | --- | --- | --- | --- | --- | --- | --- | --- | --- | --- | --- | --- | --- | --- | --- | --- | --- | --- | --- | --- | --- | --- | --- | --- | --- | --- | --- | --- | --- | --- | --- | --- | --- | --- | --- | --- | --- | --- | --- | --- | --- | --- | --- | --- | --- | --- | --- | --- | --- | --- | --- | --- | --- | --- | --- | --- | --- | --- | --- | --- | --- | --- | --- | --- | --- | --- | --- | --- | --- | --- | --- | --- | --- | --- | --- | --- | --- | --- | --- | --- | --- | --- | --- | --- | --- | --- | --- | --- | --- | --- | --- | --- | --- | --- | --- | --- | --- | --- | --- | --- | --- | --- | --- | --- | --- | --- | --- | --- | --- | --- | --- | --- | --- | --- | --- | --- | --- | --- | --- | --- | --- | --- | --- | --- | --- | --- | --- | --- | --- | --- | --- | --- | --- | --- | --- | --- | --- | --- | --- | --- | --- | --- | --- | --- | --- | --- | --- | --- | --- | --- | --- | --- | --- | --- | --- | --- | --- | --- | --- | --- | --- | --- | --- | --- | --- | --- | --- | --- | --- | --- | --- | --- | --- | --- | --- | --- | --- | --- | --- | --- | --- | --- | --- | --- | --- | --- | --- | --- | --- | --- | --- | --- | --- | --- | --- | --- | --- | --- | --- | --- | --- | --- | --- | --- | --- | --- | --- | --- | --- | --- | --- | --- | --- | --- | --- | --- | --- | --- | --- | --- | --- | --- | --- | --- | --- | --- | --- | --- | --- | --- | --- | --- | --- | --- | --- | --- | --- | --- | --- | --- | --- | --- | --- | --- | --- | --- | --- | --- | --- | --- | --- | --- | --- | --- | --- | --- | --- | --- | --- | --- | --- | --- | --- | --- | --- | --- | --- | --- | --- | --- | --- | --- | --- | --- | --- | --- | --- | --- | --- | --- | --- | --- | --- | --- | --- | --- | --- | --- | --- | --- | --- | --- | --- | --- | --- | --- | --- | --- | --- | --- | --- | --- | --- | --- | --- | --- | --- | --- | --- | --- | --- | --- | --- | --- | --- | --- | --- | --- | --- | --- | --- | --- | --- | --- | --- | --- | --- | --- | --- | --- | --- | --- | --- | --- | --- | --- | --- | --- | --- | --- | --- | --- | --- | --- | --- | --- | --- | --- | --- | --- | --- | --- | --- | --- | --- | --- | --- | --- | --- | --- | --- | --- | --- | --- | --- | --- | --- | --- | --- | --- | --- | --- | --- | --- | --- | --- | --- | --- | --- | --- | --- | --- | --- | --- | --- | --- | --- | --- | --- | --- | --- | --- | --- | --- | --- | --- | --- | --- | --- | --- | --- | --- | --- | --- | --- | --- | --- | --- | --- | --- | --- | --- | --- | --- | --- | --- | --- | --- | --- | --- | --- | --- | --- | --- | --- | --- | --- | --- | --- | --- | --- | --- | --- | --- | --- | --- | --- | --- | --- | --- | --- | --- | --- | --- | --- | --- | --- | --- | --- | --- | --- | --- | --- | --- | --- | --- | --- | --- | --- | --- | --- | --- | --- | --- | --- | --- | --- | --- | --- | --- | --- | --- | --- | --- | --- | --- | --- | --- | --- | --- | --- | --- | --- | --- | --- | --- | --- | --- | --- | --- | --- | --- | --- | --- | --- | --- | --- | --- | --- | --- | --- | --- | --- | --- | --- | --- | --- | --- | --- | --- | --- | --- | --- | --- | --- | --- | --- | --- | --- | --- | --- | --- | --- | --- | --- | --- | --- | --- | --- | --- | --- | --- | --- | --- | --- | --- | --- | --- | --- | --- | --- | --- | --- | --- | --- | --- | --- | --- | --- | --- | --- | --- | --- | --- | --- | --- | --- | --- | --- | --- | --- | --- | --- | --- | --- | --- | --- | --- | --- | --- | --- | --- | --- | --- | --- | --- | --- | --- | --- | --- | --- | --- | --- | --- | --- | --- | --- | --- | --- | --- | --- | --- | --- | --- | --- | --- | --- | --- | --- | --- | --- | --- | --- | --- | --- | --- | --- | --- | --- | --- | --- | --- | --- | --- | --- | --- | --- | --- | --- | --- | --- | --- | --- | --- | --- | --- | --- | --- | --- | --- | --- | --- | --- | --- | --- | --- | --- | --- | --- | --- | --- | --- | --- | --- | --- | --- | --- | --- | --- | --- | --- | --- | --- | --- | --- | --- | --- | --- | --- | --- | --- | --- | --- | --- | --- | --- | --- | --- | --- | --- | --- | --- | --- | --- | --- | --- | --- | --- | --- | --- | --- | --- | --- | --- | --- | --- | --- | --- | --- | --- | --- | --- | --- | --- | --- | --- | --- | --- | --- | --- | --- | --- | --- | --- | --- | --- | --- | --- | --- | --- | --- | --- | --- | --- | --- | --- | --- | --- | --- | --- | --- | --- | --- | --- | --- | --- | --- | --- | --- | --- | --- | --- | --- | --- | --- | --- | --- | --- | --- | --- | --- | --- | --- | --- | --- | --- | --- | --- | --- | --- | --- | --- | --- | --- | --- | --- | --- | --- | --- | --- | --- | --- | --- | --- | --- | --- | --- | --- | --- | --- | --- | --- | --- | --- | --- | --- | --- | --- | --- | --- | --- | --- | --- | --- | --- | --- | --- | --- | --- | --- | --- | --- | --- | --- | --- | --- | --- | --- | --- | --- | --- | --- | --- | --- | --- | --- | --- | --- | --- | --- | --- | --- | --- | --- | --- | --- | --- | --- | --- | --- | --- | --- | --- | --- | --- | --- | --- | --- | --- | --- | --- | --- | --- | --- | --- | --- | --- | --- | --- | --- | --- | --- | --- | --- | --- | --- | --- | --- | --- | --- | --- | --- | --- | --- | --- | --- | --- | --- | --- | --- | --- | --- | --- | --- | --- | --- | --- | --- | --- | --- | --- | --- | --- | --- | --- | --- | --- | --- | --- | --- | --- | --- | --- | --- | --- | --- | --- | --- | --- | --- | --- | --- | --- | --- | --- | --- | --- | --- | --- | --- | --- | --- | --- | --- | --- | --- | --- | --- | --- | --- | --- | --- | --- | --- | --- | --- | --- | --- | --- | --- | --- | --- | --- | --- | --- | --- | --- | --- | --- | --- | --- | --- | --- | --- | --- | --- | --- | --- | --- | --- | --- | --- | --- | --- | --- | --- | --- | --- | --- | --- | --- | --- | --- | --- | --- | --- | --- | --- | --- | --- | --- | --- | --- | --- | --- | --- | --- | --- | --- | --- | --- | --- | --- | --- | --- | --- | --- | --- | --- | --- | --- | --- | --- | --- | --- | --- | --- | --- | --- | --- | --- | --- | --- | --- | --- | --- | --- | --- | --- | --- | --- | --- | --- | --- | --- | --- | --- | --- | --- | --- | --- | --- | --- | --- | --- | --- | --- | --- | --- | --- | --- | --- | --- | --- | --- | --- | --- | --- | --- | --- | --- | --- | --- | --- | --- | --- | --- | --- | --- | --- | --- | --- | --- | --- | --- | --- | --- | --- | --- | --- | --- | --- | --- | --- | --- | --- | --- | --- | --- | --- | --- | --- | --- | --- | --- | --- | --- | --- | --- | --- | --- | --- | --- | --- | --- | --- | --- | --- | --- | --- | --- | --- | --- | --- | --- | --- | --- | --- | --- | --- | --- | --- | --- | --- | --- | --- | --- | --- | --- | --- | --- | --- | --- | --- | --- | --- | --- | --- | --- | --- | --- | --- | --- | --- | --- | --- | --- | --- | --- | --- | --- | --- | --- | --- | --- | --- | --- | --- | --- | --- | --- | --- | --- | --- | --- | --- | --- | --- | --- | --- | --- | --- | --- | --- | --- | --- | --- | --- | --- | --- | --- | --- | --- | --- | --- | --- | --- | --- | --- | --- | --- | --- | --- | --- | --- | --- | --- | --- | --- | --- | --- | --- | --- | --- | --- | --- | --- | --- | --- | --- | --- | --- | --- | --- | --- | --- | --- | --- | --- | --- | --- | --- | --- | --- | --- | --- | --- | --- | --- | --- | --- | --- | --- | --- | --- | --- | --- | --- | --- | --- | --- | --- | --- | --- | --- | --- | --- | --- | --- | --- | --- | --- | --- | --- | --- | --- | --- | --- | --- | --- | --- | --- | --- | --- | --- | --- | --- | --- | --- | --- | --- | --- | --- | --- | --- | --- | --- | --- | --- | --- | --- | --- | --- | --- | --- | --- | --- | --- | --- | --- | --- | --- | --- | --- | --- | --- | --- | --- | --- | --- | --- | --- | --- | --- | --- | --- | --- | --- | --- | --- | --- | --- | --- | --- | --- | --- | --- | --- | --- | --- | --- | --- | --- | --- | --- | --- | --- | --- | --- | --- | --- | --- | --- | --- | --- | --- | --- | --- | --- | --- | --- | --- | --- | --- | --- | --- | --- | --- | --- | --- | --- | --- | --- | --- | --- | --- | --- | --- | --- | --- | --- | --- | --- | --- | --- | --- | --- | --- | --- | --- | --- | --- | --- | --- | --- | --- | --- | --- | --- | --- | --- | --- | --- | --- | --- | --- | --- | --- | --- | --- | --- | --- | --- | --- | --- | --- | --- | --- | --- | --- | --- | --- | --- | --- | --- | --- | --- | --- | --- | --- | --- | --- | --- | --- | --- | --- | --- | --- | --- | --- | --- | --- | --- | --- | --- | --- | --- | --- | --- | --- | --- | --- | --- | --- | --- | --- | --- | --- | --- | --- | --- | --- | --- | --- | --- | --- | --- | --- | --- | --- | --- | --- | --- | --- | --- | --- | --- | --- | --- | --- | --- | --- | --- | --- | --- | --- | --- | --- | --- | --- | --- | --- | --- | --- | --- | --- | --- | --- | --- | --- | --- | --- | --- | --- | --- | --- | --- | --- | --- | --- | --- | --- | --- | --- | --- | --- | --- | --- | --- | --- | --- | --- | --- | --- | --- | --- | --- | --- | --- | --- | --- | --- | --- | --- | --- | --- | --- | --- | --- | --- | --- | --- | --- | --- | --- | --- | --- | --- | --- | --- | --- | --- | --- | --- | --- | --- | --- | --- | --- | --- | --- | --- | --- | --- | --- | --- | --- | --- | --- | --- | --- | --- | --- | --- | --- | --- | --- | --- | --- | --- | --- | --- | --- | --- | --- | --- | --- | --- | --- | --- | --- | --- | --- | --- | --- | --- | --- | --- | --- | --- | --- | --- | --- | --- | --- | --- | --- | --- | --- | --- | --- | --- | --- | --- | --- | --- | --- | --- | --- | --- | --- | --- | --- | --- | --- | --- | --- | --- | --- | --- | --- | --- | --- | --- | --- | --- | --- | --- | --- | --- | --- | --- | --- | --- | --- | --- | --- | --- | --- | --- | --- | --- | --- | --- | --- | --- | --- | --- | --- | --- | --- | --- | --- | --- | --- | --- | --- | --- | --- | --- | --- | --- | --- | --- | --- | --- | --- | --- | --- | --- | --- | --- | --- | --- | --- | --- | --- | --- | --- | --- | --- | --- | --- | --- | --- | --- | --- | --- | --- | --- | --- | --- | --- | --- | --- | --- | --- | --- | --- | --- | --- | --- | --- | --- | --- | --- | --- | --- | --- | --- | --- | --- | --- | --- | --- | --- | --- | --- | --- | --- | --- | --- | --- | --- | --- | --- | --- | --- | --- | --- | --- | --- | --- | --- | --- | --- | --- | --- | --- | --- | --- | --- | --- | --- | --- | --- | --- | --- | --- | --- | --- | --- | --- | --- | --- | --- | --- | --- | --- | --- | --- | --- | --- | --- | --- | --- | --- | --- | --- | --- | --- | --- | --- | --- | --- | --- | --- | --- | --- | --- | --- | --- | --- | --- | --- | --- | --- | --- | --- | --- | --- | --- | --- | --- | --- | --- | --- | --- | --- | --- | --- | --- | --- | --- | --- | --- | --- | --- | --- | --- | --- | --- | --- | --- | --- | --- | --- | --- | --- | --- | --- | --- | --- | --- | --- | --- | --- | --- | --- | --- | --- | --- | --- | --- | --- | --- | --- | --- | --- | --- | --- | --- | --- | --- | --- | --- | --- | --- | --- | --- | --- | --- | --- | --- | --- | --- | --- | --- | --- | --- | --- | --- | --- | --- | --- | --- | --- | --- | --- | --- | --- | --- | --- | --- | --- | --- | --- | --- | --- | --- | --- | --- | --- | --- | --- | --- | --- | --- | --- | --- | --- | --- | --- | --- | --- | --- | --- | --- | --- | --- | --- | --- | --- | --- | --- | --- | --- | --- | --- | --- | --- | --- | --- | --- | --- | --- | --- | --- | --- | --- | --- | --- | --- | --- | --- | --- | --- | --- | --- | --- | --- | --- | --- | --- | --- | --- | --- | --- | --- | --- | --- | --- | --- | --- | --- | --- | --- | --- | --- | --- | --- | --- | --- | --- | --- | --- | --- | --- | --- | --- | --- | --- | --- | --- | --- | --- | --- | --- | --- | --- | --- | --- | --- | --- | --- | --- | --- | --- | --- | --- | --- | --- | --- | --- | --- | --- | --- | --- | --- | --- | --- | --- | --- | --- | --- | --- | --- | --- | --- | --- | --- | --- | --- | --- | --- | --- | --- | --- | --- | --- | --- | --- | --- | --- | --- | --- | --- | --- | --- | --- | --- | --- | --- | --- | --- | --- | --- | --- | --- | --- | --- | --- | --- | --- | --- | --- | --- | --- | --- | --- | --- | --- | --- | --- | --- | --- | --- | --- | --- | --- | --- | --- | --- | --- | --- | --- | --- | --- | --- | --- | --- | --- | --- | --- | --- | --- | --- | --- | --- | --- | --- | --- | --- | --- | --- | --- | --- | --- | --- | --- | --- | --- | --- | --- | --- | --- | --- | --- | --- | --- | --- | --- | --- | --- | --- | --- | --- | --- | --- | --- | --- | --- | --- | --- | --- | --- | --- | --- | --- | --- | --- | --- | --- | --- | --- | --- | --- | --- | --- | --- | --- | --- | --- | --- | --- | --- | --- | --- | --- | --- | --- | --- | --- | --- | --- | --- | --- | --- | --- | --- | --- | --- | --- | --- | --- | --- | --- | --- | --- | --- | --- | --- | --- | --- | --- | --- | --- | --- | --- | --- | --- | --- | --- | --- | --- | --- | --- | --- | --- | --- | --- | --- | --- | --- | --- | --- | --- | --- | --- | --- | --- | --- | --- | --- | --- | --- | --- | --- | --- | --- | --- | --- | --- | --- | --- | --- | --- | --- | --- | --- | --- | --- | --- | --- | --- | --- | --- | --- | --- | --- | --- | --- | --- | --- | --- | --- | --- | --- | --- | --- | --- | --- | --- | --- | --- | --- | --- | --- | --- | --- | --- | --- | --- | --- | --- | --- | --- | --- | --- | --- | --- | --- | --- | --- | --- | --- | --- | --- | --- | --- | --- | --- | --- | --- | --- | --- | --- | --- | --- | --- | --- | --- | --- | --- | --- | --- | --- | --- | --- | --- | --- | --- | --- | --- | --- | --- | --- | --- | --- | --- | --- | --- | --- | --- | --- | --- | --- | --- | --- | --- | --- | --- | --- | --- | --- | --- | --- | --- | --- | --- | --- | --- | --- | --- | --- | --- | --- | --- | --- | --- | --- | --- | --- | --- | --- | --- | --- | --- | --- | --- | --- | --- | --- | --- | --- | --- | --- | --- | --- | --- | --- | --- | --- | --- | --- | --- | --- | --- | --- | --- | --- | --- | --- | --- | --- | --- | --- | --- | --- | --- | --- | --- | --- | --- | --- | --- | --- | --- | --- | --- | --- | --- | --- | --- | --- | --- | --- | --- | --- | --- | --- | --- | --- | --- | --- | --- | --- | --- | --- | --- | --- | --- | --- | --- | --- | --- | --- | --- | --- | --- | --- | --- | --- | --- | --- | --- | --- | --- | --- | --- | --- | --- | --- | --- | --- | --- | --- | --- | --- | --- | --- | --- | --- | --- | --- | --- | --- | --- | --- | --- | --- | --- | --- | --- | --- | --- | --- | --- | --- | --- | --- | --- | --- | --- | --- | --- | --- | --- | --- | --- | --- | --- | --- | --- | --- | --- | --- | --- | --- | --- | --- | --- | --- | --- | --- | --- | --- | --- | --- | --- | --- | --- | --- | --- | --- | --- | --- | --- | --- | --- | --- | --- | --- | --- | --- | --- | --- | --- | --- | --- | --- | --- | --- | --- | --- | --- | --- | --- | --- | --- | --- | --- | --- | --- | --- | --- | --- | --- | --- | --- | --- | --- | --- | --- | --- | --- | --- | --- | --- | --- | --- | --- | --- | --- | --- | --- | --- | --- | --- | --- | --- | --- | --- | --- | --- | --- | --- | --- | --- | --- | --- | --- | --- | --- | --- | --- | --- | --- | --- | --- | --- | --- | --- | --- | --- | --- | --- | --- | --- | --- | --- | --- | --- | --- | --- | --- | --- | --- | --- | --- | --- | --- | --- | --- | --- | --- | --- | --- | --- | --- | --- | --- | --- | --- | --- | --- | --- | --- | --- | --- | --- | --- | --- | --- | --- | --- | --- | --- | --- | --- | --- | --- | --- | --- | --- | --- | --- | --- | --- | --- | --- | --- | --- | --- | --- | --- | --- | --- | --- | --- | --- | --- | --- | --- | --- | --- | --- | --- | --- | --- | --- | --- | --- | --- | --- | --- | --- | --- | --- | --- | --- | --- | --- | --- | --- | --- | --- | --- | --- | --- | --- | --- | --- | --- | --- | --- | --- | --- | --- | --- | --- | --- | --- | --- | --- | --- | --- | --- | --- | --- | --- | --- | --- | --- | --- | --- | --- | --- | --- | --- | --- | --- | --- | --- | --- | --- | --- | --- | --- | --- | --- | --- | --- | --- | --- | --- | --- | --- | --- | --- | --- | --- | --- | --- | --- | --- | --- | --- | --- | --- | --- | --- | --- | --- | --- | --- | --- | --- | --- | --- | --- | --- | --- | --- | --- | --- | --- | --- | --- | --- | --- | --- | --- | --- | --- | --- | --- | --- | --- | --- | --- | --- | --- | --- | --- | --- | --- | --- | --- | --- | --- | --- | --- | --- | --- | --- | --- | --- | --- | --- | --- | --- | --- | --- | --- | --- | --- | --- | --- | --- | --- | --- | --- | --- | --- | --- | --- | --- | --- | --- | --- | --- | --- | --- | --- | --- | --- | --- | --- | --- | --- | --- | --- | --- | --- | --- | --- | --- | --- | --- | --- | --- | --- | --- | --- | --- | --- | --- | --- | --- | --- | --- | --- | --- | --- | --- | --- | --- | --- | --- | --- | --- | --- | --- | --- | --- | --- | --- | --- | --- | --- | --- | --- | --- | --- | --- | --- | --- | --- | --- | --- | --- | --- | --- | --- | --- | --- | --- | --- | --- | --- | --- | --- | --- | --- | --- | --- | --- | --- | --- | --- | --- | --- | --- | --- | --- | --- | --- | --- | --- | --- | --- | --- | --- | --- | --- | --- | --- | --- | --- | --- | --- | --- | --- | --- | --- | --- | --- | --- | --- | --- | --- | --- | --- | --- | --- | --- | --- | --- | --- | --- | --- | --- | --- | --- | --- | --- | --- | --- | --- | --- | --- | --- | --- | --- | --- | --- | --- | --- | --- | --- | --- | --- | --- | --- | --- | --- | --- | --- | --- | --- | --- | --- | --- | --- | --- | --- | --- | --- | --- | --- | --- | --- | --- | --- | --- | --- | --- | --- | --- | --- | --- | --- | --- | --- | --- | --- | --- | --- | --- | --- | --- | --- | --- | --- | --- | --- | --- | --- | --- | --- | --- | --- | --- | --- | --- | --- | --- | --- | --- | --- | --- | --- | --- | --- | --- | --- | --- | --- | --- | --- | --- | --- | --- | --- | --- | --- | --- | --- | --- | --- | --- | --- | --- | --- | --- | --- | --- | --- | --- | --- | --- | --- | --- | --- | --- | --- | --- | --- | --- | --- | --- | --- | --- | --- | --- | --- | --- | --- | --- | --- | --- | --- | --- | --- | --- | --- | --- | --- | --- | --- | --- | --- | --- | --- | --- | --- | --- | --- | --- | --- | --- | --- | --- | --- | --- | --- | --- | --- | --- | --- | --- | --- | --- | --- | --- | --- | --- | --- | --- | --- | --- | --- | --- | --- | --- | --- | --- | --- | --- | --- | --- | --- | --- | --- | --- | --- | --- | --- | --- | --- | --- | --- | --- | --- | --- | --- | --- | --- | --- | --- | --- | --- | --- | --- | --- | --- | --- | --- | --- | --- | --- | --- | --- | --- | --- | --- | --- | --- | --- | --- | --- | --- | --- | --- | --- | --- | --- | --- | --- | --- | --- | --- | --- | --- | --- | --- | --- | --- | --- | --- | --- | --- | --- | --- | --- | --- | --- | --- | --- | --- | --- | --- | --- | --- | --- | --- | --- | --- | --- | --- | --- | --- | --- | --- | --- | --- | --- | --- | --- | --- | --- | --- | --- | --- | --- | --- | --- | --- | --- | --- | --- | --- | --- | --- | --- | --- | --- | --- | --- | --- | --- | --- | --- | --- | --- | --- | --- | --- | --- | --- | --- | --- | --- | --- | --- | --- | --- | --- | --- | --- | --- | --- | --- | --- | --- | --- | --- | --- | --- | --- | --- | --- | --- | --- | --- | --- | --- | --- | --- | --- | --- | --- | --- | --- | --- | --- | --- | --- | --- | --- | --- | --- | --- | --- | --- | --- | --- | --- | --- | --- | --- | --- | --- | --- | --- | --- | --- | --- | --- | --- | --- | --- | --- | --- | --- | --- | --- | --- | --- | --- | --- | --- | --- | --- | --- | --- | --- | --- | --- | --- | --- | --- | --- | --- | --- | --- | --- | --- | --- | --- | --- | --- | --- | --- | --- | --- | --- | --- | --- | --- | --- | --- | --- | --- | --- | --- | --- | --- | --- | --- | --- | --- | --- | --- | --- | --- | --- | --- | --- | --- | --- | --- | --- | --- | --- | --- | --- | --- | --- | --- | --- | --- | --- | --- | --- | --- | --- | --- | --- | --- | --- | --- | --- | --- | --- | --- | --- | --- | --- | --- | --- | --- | --- | --- | --- | --- | --- | --- | --- | --- | --- | --- | --- | --- | --- | --- | --- | --- | --- | --- | --- | --- | --- | --- | --- | --- | --- | --- | --- | --- | --- | --- | --- | --- | --- | --- | --- | --- | --- | --- | --- | --- | --- | --- | --- | --- | --- | --- | --- | --- | --- | --- | --- | --- | --- | --- | --- | --- | --- | --- | --- | --- | --- | --- | --- | --- | --- | --- | --- | --- | --- | --- | --- | --- | --- | --- | --- | --- | --- | --- | --- | --- | --- | --- | --- | --- | --- | --- | --- | --- | --- | --- | --- | --- | --- | --- | --- | --- | --- | --- | --- | --- | --- | --- | --- | --- | --- | --- | --- | --- | --- | --- | --- | --- | --- | --- | --- | --- | --- | --- | --- | --- | --- | --- | --- | --- | --- | --- | --- | --- | --- | --- | --- | --- | --- | --- | --- | --- | --- | --- | --- | --- | --- | --- | --- | --- | --- | --- | --- | --- | --- | --- | --- | --- | --- | --- | --- | --- | --- | --- | --- | --- | --- | --- | --- | --- | --- | --- | --- | --- | --- | --- | --- | --- | --- | --- | --- | --- | --- | --- | --- | --- | --- | --- | --- | --- | --- | --- | --- | --- | --- | --- | --- | --- | --- | --- | --- | --- | --- | --- | --- | --- | --- | --- | --- | --- | --- | --- | --- | --- | --- | --- | --- | --- | --- | --- | --- | --- | --- | --- | --- | --- | --- | --- | --- | --- | --- | --- | --- | --- | --- | --- | --- | --- | --- | --- | --- | --- | --- | --- | --- | --- | --- | --- | --- | --- | --- | --- | --- | --- | --- | --- | --- | --- | --- | --- | --- | --- | --- | --- | --- | --- | --- | --- | --- | --- | --- | --- | --- | --- | --- | --- | --- | --- | --- | --- | --- | --- | --- | --- | --- | --- | --- | --- | --- | --- | --- | --- | --- | --- | --- | --- | --- | --- | --- | --- | --- | --- | --- | --- | --- | --- | --- | --- | --- | --- | --- | --- | --- | --- | --- | --- | --- | --- | --- | --- | --- | --- | --- | --- | --- | --- | --- | --- | --- | --- | --- | --- | --- | --- | --- | --- | --- | --- | --- | --- | --- | --- | --- | --- | --- | --- | --- | --- | --- | --- | --- | --- | --- | --- | --- | --- | --- | --- | --- | --- | --- | --- | --- | --- | --- | --- | --- | --- | --- | --- | --- | --- | --- | --- | --- | --- | --- | --- | --- | --- | --- | --- | --- | --- | --- | --- | --- | --- | --- | --- | --- | --- | --- | --- | --- | --- | --- | --- | --- | --- | --- | --- | --- | --- | --- | --- | --- | --- | --- | --- | --- | --- | --- | --- | --- | --- | --- | --- | --- | --- | --- | --- | --- | --- | --- | --- | --- | --- | --- | --- | --- | --- | --- | --- | --- | --- | --- | --- | --- | --- | --- | --- | --- | --- | --- | --- | --- | --- | --- | --- | --- | --- | --- | --- | --- | --- | --- | --- | --- | --- | --- | --- | --- | --- | --- | --- | --- | --- | --- | --- | --- | --- | --- | --- | --- | --- | --- | --- | --- | --- | --- | --- | --- | --- | --- | --- | --- | --- | --- | --- | --- | --- | --- | --- | --- | --- | --- | --- | --- | --- | --- | --- | --- | --- | --- | --- | --- | --- | --- | --- | --- | --- | --- | --- | --- | --- | --- | --- | --- | --- | --- | --- | --- | --- | --- | --- | --- | --- | --- | --- | --- | --- | --- | --- | --- | --- | --- | --- | --- | --- | --- | --- | --- | --- | --- | --- | --- | --- | --- | --- | --- | --- | --- | --- | --- | --- | --- | --- | --- | --- | --- | --- | --- | --- | --- | --- | --- | --- | --- | --- | --- | --- | --- | --- | --- | --- | --- | --- | --- | --- | --- | --- | --- | --- | --- | --- | --- | --- | --- | --- | --- | --- | --- | --- | --- | --- | --- | --- | --- | --- | --- | --- | --- | --- | --- | --- | --- | --- | --- | --- | --- | --- | --- | --- | --- | --- | --- | --- | --- | --- | --- | --- | --- | --- | --- | --- | --- | --- | --- | --- | --- | --- | --- | --- | --- | --- | --- | --- | --- | --- | --- | --- | --- | --- | --- | --- | --- | --- | --- | --- | --- | --- | --- | --- | --- | --- | --- | --- | --- | --- | --- | --- | --- | --- | --- | --- | --- | --- | --- | --- | --- | --- | --- | --- | --- | --- | --- | --- | --- | --- | --- | --- | --- | --- | --- | --- | --- | --- | --- | --- | --- | --- | --- | --- | --- | --- | --- | --- | --- | --- | --- | --- | --- | --- | --- | --- | --- | --- | --- | --- | --- | --- | --- | --- | --- | --- | --- | --- | --- | --- | --- | --- | --- | --- | --- | --- | --- | --- | --- | --- | --- | --- | --- | --- | --- | --- | --- | --- | --- | --- | --- | --- | --- | --- | --- | --- | --- | --- | --- | --- | --- | --- | --- | --- | --- | --- | --- | --- | --- | --- | --- | --- | --- | --- | --- | --- | --- | --- | --- | --- | --- | --- | --- | --- | --- | --- | --- | --- | --- | --- | --- | --- | --- | --- | --- | --- | --- | --- | --- | --- | --- | --- | --- | --- | --- | --- | --- | --- | --- | --- | --- | --- | --- | --- | --- | --- | --- | --- | --- | --- | --- | --- | --- | --- | --- | --- | --- | --- | --- | --- | --- | --- | --- | --- | --- | --- | --- | --- | --- | --- | --- | --- | --- | --- | --- | --- | --- | --- | --- | --- | --- | --- | --- | --- | --- | --- | --- | --- | --- | --- | --- | --- | --- | --- | --- | --- | --- | --- | --- | --- | --- | --- | --- | --- | --- | --- | --- | --- | --- | --- | --- | --- | --- | --- | --- | --- | --- | --- | --- | --- | --- | --- | --- | --- | --- | --- | --- | --- | --- | --- | --- | --- | --- | --- | --- | --- | --- | --- | --- | --- | --- | --- | --- | --- | --- | --- | --- | --- | --- | --- | --- | --- | --- | --- | --- | --- | --- | --- | --- | --- | --- | --- | --- | --- | --- | --- | --- | --- | --- | --- | --- | --- | --- | --- | --- | --- | --- | --- | --- | --- | --- | --- | --- | --- | --- | --- | --- | --- | --- | --- | --- | --- | --- | --- | --- | --- | --- | --- | --- | --- | --- | --- | --- | --- | --- | --- | --- | --- | --- | --- | --- | --- | --- | --- | --- | --- | --- | --- | --- | --- | --- | --- | --- | --- | --- | --- | --- | --- | --- | --- | --- | --- | --- | --- | --- | --- | --- | --- | --- | --- | --- | --- | --- | --- | --- | --- | --- | --- | --- | --- | --- | --- | --- | --- | --- | --- | --- | --- | --- | --- | --- | --- | --- | --- | --- | --- | --- | --- | --- | --- | --- | --- | --- | --- | --- | --- | --- | --- | --- | --- | --- | --- | --- | --- | --- | --- | --- | --- | --- | --- | --- | --- | --- | --- | --- | --- | --- | --- | --- | --- | --- | --- | --- | --- | --- | --- | --- | --- | --- | --- | --- | --- | --- | --- | --- | --- | --- | --- | --- | --- | --- | --- | --- | --- | --- | --- | --- | --- | --- | --- | --- | --- | --- | --- | --- | --- | --- | --- | --- | --- | --- | --- | --- | --- | --- | --- | --- | --- | --- | --- | --- | --- | --- | --- | --- | --- | --- | --- | --- | --- | --- | --- | --- | --- | --- | --- | --- |
| |  |  |  |  |  |  |  |  |  |  |  |  |  |  |  |  |  |  |  |  |  |  |  |  |  |  |  |  |  |  |  |  |  |  |  |  |  |  |  |  |  |  |  |  |  |  |  |  |  |  |  |  |  |  |  |  |  |  | | --- | --- | --- | --- | --- | --- | --- | --- | --- | --- | --- | --- | --- | --- | --- | --- | --- | --- | --- | --- | --- | --- | --- | --- | --- | --- | --- | --- | --- | --- | --- | --- | --- | --- | --- | --- | --- | --- | --- | --- | --- | --- | --- | --- | --- | --- | --- | --- | --- | --- | --- | --- | --- | --- | --- | --- | --- | --- | | G0VCQ8/1-663 | 1 | - | M | K | V | L | C | V | A | E | K | N | S | I | A | K | S | V | S | Q | I | L | G | G | G | R | S | T | I | R | D | S | T | Y | T | Y | V | K | N | Y | D | F | Q | Y | S | G | F | P | F | G | - | N | G | Q | D | C | 53 | | Q6CJ79/1-659 | 1 | M | V | K | I | L | C | V | A | E | K | N | S | I | A | K | N | V | A | Q | I | L | S | G | G | R | S | S | M | R | P | S | T | S | P | Y | I | K | N | Y | D | F | Q | Y | D | - | F | Q | W | L | - | N | M | G | R | C | 53 | | Q6FSR2/1-650 | 1 | - | M | K | V | L | C | V | A | E | K | N | S | I | A | K | A | V | S | G | I | L | G | G | G | R | L | S | V | R | D | S | G | Y | T | Y | I | K | N | Y | D | F | N | Y | S | G | F | S | F | A | - | G | G | N | D | V | 53 | | Q751B3/1-631 | 1 | - | M | R | V | V | C | V | A | E | K | N | S | I | A | K | A | V | A | G | I | L | G | G | G | N | T | R | T | R | S | S | P | S | K | Y | I | K | N | Y | D | F | T | Y | N | - | F | A | W | A | Q | G | G | E | R | C | 53 | | A7TSV2/1-650 | 1 | - | M | R | V | L | C | V | A | E | K | N | S | I | A | K | A | V | S | Q | I | L | S | G | G | N | S | N | A | R | D | S | G | Y | M | Y | V | K | N | Y | D | F | R | F | N | G | F | P | F | I | - | N | D | R | D | C | 53 | | C5DKW7/1-627 | 1 | M | P | R | V | V | C | V | A | E | K | N | S | I | A | K | A | V | A | G | I | L | S | G | G | R | C | S | R | R | D | S | P | N | K | Y | I | K | N | Y | D | F | T | F | S | - | F | G | F | S | - | - | - | G | S | S | 51 | | C5DT16/1-650 | 1 | - | M | K | V | L | C | V | A | E | K | N | S | I | A | K | A | V | S | Q | T | L | G | G | G | N | C | N | A | R | D | S | G | Y | M | Y | V | K | N | Y | D | F | S | F | D | G | F | P | F | A | Q | G | T | G | S | C | 54 | | Kwal\_23.3190/1-626 | 1 | M | P | R | V | V | C | I | A | E | K | N | S | I | A | K | A | V | A | G | I | L | S | G | G | R | S | S | R | R | D | S | P | N | K | Y | I | K | N | Y | D | F | N | F | E | - | F | P | F | S | - | - | - | G | S | S | 51 | | Sbay\_603.19/1-654 | 1 | - | M | K | V | L | C | V | A | E | K | N | S | I | A | K | A | V | S | Q | I | L | G | G | G | R | S | T | S | R | D | S | G | Y | M | Y | V | K | N | Y | D | F | M | F | S | G | F | P | F | A | R | N | G | G | D | C | 54 | | SAKL0H13310g/1-638 | 1 | - | M | K | V | I | C | I | A | E | K | N | S | I | A | K | A | V | A | D | I | L | G | G | G | R | V | R | T | R | S | S | P | Y | Q | Y | V | K | N | Y | D | F | N | F | N | - | F | S | F | A | - | G | G | S | S | C | 52 | | P13099/1-656 | 1 | - | M | K | V | L | C | V | A | E | K | N | S | I | A | K | A | V | S | Q | I | L | G | G | G | R | S | T | S | R | D | S | G | Y | M | Y | V | K | N | Y | D | F | M | F | S | G | F | P | F | A | R | N | G | A | N | C | 54 | |  | | G0VCQ8/1-663 | 54 | Q | V | T | M | T | S | V | A | G | H | L | T | S | V | D | F | R | G | D | I | Y | G | W | G | K | C | P | I | Y | E | L | F | D | A | P | L | S | T | E | M | D | S | N | Q | K | K | I | A | S | N | I | Q | K | E | A | 108 | | Q6CJ79/1-659 | 54 | N | V | T | M | T | A | V | A | G | H | L | M | N | L | A | F | S | Q | N | E | Y | G | W | G | K | C | D | P | N | L | L | F | D | A | P | T | I | D | E | M | - | - | - | D | D | K | I | V | K | N | L | Q | N | E | G | 105 | | Q6FSR2/1-650 | 54 | Q | V | T | M | T | S | V | A | G | H | L | T | G | V | D | F | A | H | E | Q | Y | G | W | G | N | C | R | I | E | Q | L | F | D | A | P | L | N | E | V | M | D | K | N | Q | Q | K | I | A | A | N | I | R | R | E | A | 108 | | Q751B3/1-631 | 54 | E | V | T | M | T | A | V | A | G | H | L | M | S | Q | D | F | G | - | P | Q | Y | G | W | N | K | C | D | P | R | E | L | F | G | A | E | I | L | E | T | P | - | - | - | T | R | E | I | A | E | N | I | T | R | E | C | 104 | | A7TSV2/1-650 | 54 | D | V | T | M | T | S | V | A | G | H | L | M | G | I | D | F | P | N | D | R | Y | G | W | G | K | C | P | V | H | E | L | F | D | A | P | L | L | Q | V | Y | D | K | N | Q | K | K | I | A | D | N | I | K | K | E | A | 108 | | C5DKW7/1-627 | 52 | S | V | T | M | T | S | V | A | G | H | L | T | G | L | D | L | D | - | Q | A | H | G | W | G | K | C | P | I | P | Q | V | F | D | A | P | I | V | D | T | R | T | K | D | Q | Q | K | I | A | D | N | I | K | K | E | C | 105 | | C5DT16/1-650 | 55 | Q | V | T | M | T | S | V | A | G | H | L | T | G | I | D | F | P | N | E | R | Y | G | W | G | K | C | R | I | D | E | L | F | D | A | P | I | N | E | V | F | D | K | N | Q | K | K | I | A | D | N | I | R | K | E | A | 109 | | Kwal\_23.3190/1-626 | 52 | N | V | T | M | T | S | V | A | G | H | L | T | G | L | D | L | D | - | Q | A | H | G | W | G | K | C | P | I | Q | Q | V | F | E | A | P | I | V | D | T | K | T | K | D | Q | Q | K | I | A | D | N | I | K | K | E | C | 105 | | Sbay\_603.19/1-654 | 55 | Q | V | T | M | T | S | V | A | G | H | L | T | G | I | D | F | S | H | D | S | H | G | W | G | K | C | A | I | Q | E | L | F | D | A | P | L | N | E | I | M | N | N | N | Q | K | K | I | A | S | N | I | K | R | E | A | 109 | | SAKL0H13310g/1-638 | 53 | E | V | T | M | T | S | V | A | G | H | L | M | N | I | D | F | G | S | E | Y | S | N | W | N | Q | C | D | T | R | K | L | F | D | A | P | L | E | D | K | K | S | K | D | Q | Q | K | I | A | K | N | I | E | N | E | S | 107 | | P13099/1-656 | 55 | E | V | T | M | T | S | V | A | G | H | L | T | G | I | D | F | S | H | D | S | H | G | W | G | K | C | A | I | Q | E | L | F | D | A | P | L | N | E | I | M | N | N | N | Q | K | K | I | A | S | N | I | K | R | E | A | 109 | |  | | G0VCQ8/1-663 | 109 | R | N | A | D | F | L | M | I | W | T | D | C | D | R | E | G | E | Y | I | G | W | E | I | L | Q | E | A | E | R | G | N | R | R | L | - | N | E | Q | Q | V | Y | R | A | N | F | S | H | L | E | R | R | H | I | L | D | 162 | | Q6CJ79/1-659 | 106 | R | H | A | T | H | L | M | I | W | T | D | C | D | R | E | G | E | C | I | G | W | E | I | V | Q | A | V | N | R | S | N | S | S | L | V | S | S | D | S | V | Y | R | A | V | F | S | H | L | E | P | S | H | I | I | A | 160 | | Q6FSR2/1-650 | 109 | K | F | A | D | V | L | M | I | W | T | D | C | D | R | E | G | E | Y | I | G | W | E | V | Y | M | E | A | Q | K | S | N | R | R | L | - | N | D | N | Q | V | Y | R | A | V | F | S | H | L | E | R | S | H | I | L | Q | 162 | | Q751B3/1-631 | 105 | R | G | A | E | Y | L | M | I | W | T | D | C | D | R | E | G | E | A | I | G | W | E | I | A | R | V | A | M | Q | A | N | T | R | L | - | V | R | S | R | I | H | R | A | V | F | S | H | L | G | R | E | H | I | M | R | 158 | | A7TSV2/1-650 | 109 | R | N | A | D | F | L | M | I | W | T | D | C | D | R | E | G | E | Y | I | G | W | E | I | F | Q | E | A | K | K | G | N | R | R | L | - | N | E | D | Q | M | F | R | A | N | F | S | H | L | E | R | S | H | I | L | Y | 162 | | C5DKW7/1-627 | 106 | R | N | A | D | Y | L | M | I | W | T | D | C | D | R | E | G | E | Y | I | G | W | E | I | V | Q | T | A | I | Q | A | N | Q | S | L | - | - | Q | A | N | V | Y | R | A | V | F | S | H | L | E | S | G | H | I | M | H | 158 | | C5DT16/1-650 | 110 | R | N | A | N | F | L | M | I | W | T | D | C | D | R | E | G | E | Y | I | G | W | E | I | S | R | E | A | S | K | N | N | S | R | L | - | T | S | N | Q | I | Y | R | A | V | F | S | H | L | E | R | R | H | I | I | D | 163 | | Kwal\_23.3190/1-626 | 106 | R | N | A | D | Y | L | M | I | W | T | D | C | D | R | E | G | E | Y | I | G | W | E | I | V | Q | A | A | L | Q | S | N | R | S | L | - | - | Q | G | N | V | F | R | A | V | F | S | H | L | E | R | A | H | I | L | Q | 158 | | Sbay\_603.19/1-654 | 110 | R | D | A | D | Y | L | M | I | W | T | D | C | D | R | E | G | E | Y | I | G | W | E | I | W | Q | E | A | K | R | G | N | R | R | I | - | E | G | D | Q | V | Y | R | A | V | F | S | H | L | E | R | Q | H | I | L | N | 163 | | SAKL0H13310g/1-638 | 108 | K | Y | A | D | F | L | M | I | W | T | D | C | D | R | E | G | E | Y | I | G | W | E | I | V | Q | A | A | S | K | F | N | R | R | L | - | T | P | S | N | V | Y | R | A | I | F | S | H | L | E | R | Q | H | I | L | N | 161 | | P13099/1-656 | 110 | R | N | A | D | Y | L | M | I | W | T | D | C | D | R | E | G | E | Y | I | G | W | E | I | W | Q | E | A | K | R | G | N | R | L | I | - | Q | N | D | Q | V | Y | R | A | V | F | S | H | L | E | R | Q | H | I | L | N | 163 | |  | | G0VCQ8/1-663 | 163 | A | A | R | R | P | Q | R | L | D | M | K | S | V | S | A | V | R | T | R | M | E | L | D | L | R | T | G | V | T | F | T | R | L | L | T | D | T | M | K | Q | K | V | E | A | S | R | L | Q | V | N | N | N | S | N | N | 217 | | Q6CJ79/1-659 | 161 | S | A | N | R | P | R | K | L | D | Q | R | Q | V | D | A | V | R | A | R | Q | E | I | D | L | R | A | G | L | A | F | T | R | L | L | T | G | N | Y | R | S | V | L | Q | S | D | L | E | Y | K | Q | L | - | - | - | - | 211 | | Q6FSR2/1-650 | 163 | A | A | H | R | P | H | R | L | D | M | R | S | V | N | A | V | R | T | R | I | E | I | D | L | R | A | G | V | T | F | T | R | L | L | T | E | T | M | R | N | K | V | A | S | S | Q | S | E | G | P | S | R | N | - | - | 215 | | Q751B3/1-631 | 159 | A | A | N | S | P | S | Q | I | D | M | N | A | V | D | A | V | K | A | R | S | E | I | D | L | R | A | G | Y | A | F | T | R | L | L | T | A | T | L | R | A | K | V | E | Q | D | M | P | A | - | - | - | - | - | - | - | 206 | | A7TSV2/1-650 | 163 | A | A | R | N | P | R | R | L | D | M | N | S | V | N | A | V | E | T | R | M | E | I | D | L | R | A | G | V | T | F | T | R | L | L | T | D | T | M | K | N | K | V | S | A | T | I | Q | M | N | S | G | P | N | - | - | 215 | | C5DKW7/1-627 | 159 | A | A | R | N | P | K | R | L | D | Q | R | A | V | D | A | V | R | T | R | M | E | V | D | L | R | S | G | V | A | F | T | R | F | L | T | E | L | L | G | S | T | V | G | R | D | S | G | S | - | - | - | - | - | - | - | 206 | | C5DT16/1-650 | 164 | A | A | R | R | P | Q | R | L | D | M | G | S | V | N | A | V | G | T | R | M | E | I | D | L | R | A | G | V | T | F | T | R | L | L | T | E | T | M | R | N | K | V | S | A | N | T | M | G | N | S | Q | H | N | N | N | 218 | | Kwal\_23.3190/1-626 | 159 | S | A | R | N | P | K | R | L | D | Q | R | A | V | D | A | V | R | T | R | M | E | V | D | L | R | S | G | V | A | F | T | R | F | L | T | E | L | L | G | S | T | V | G | R | D | S | G | A | - | - | - | - | - | - | - | 206 | | Sbay\_603.19/1-654 | 164 | A | A | R | N | P | N | R | L | D | M | K | S | V | H | A | V | G | T | R | I | E | I | D | L | R | A | G | V | T | F | T | R | L | L | T | E | T | L | R | N | K | L | K | S | Q | S | A | S | S | T | D | G | G | R | T | 218 | | SAKL0H13310g/1-638 | 162 | A | A | R | N | P | S | R | L | D | Q | K | A | V | D | A | V | R | T | R | M | E | I | D | L | R | A | G | V | T | F | T | R | F | L | T | T | L | L | R | P | K | I | L | T | D | E | T | Y | E | K | T | F | D | D | - | 215 | | P13099/1-656 | 164 | A | A | R | N | P | S | R | L | D | M | K | S | V | H | A | V | G | T | R | I | E | I | D | L | R | A | G | V | T | F | T | R | L | L | T | E | T | L | R | N | K | L | R | N | Q | A | T | M | T | K | D | G | A | K | H | 218 | |  | | G0VCQ8/1-663 | 218 | N | N | I | Q | N | N | Q | N | H | H | K | K | K | K | D | S | P | V | I | V | S | Y | G | A | C | Q | F | P | T | L | G | F | V | V | D | R | F | E | R | I | E | N | F | V | S | E | E | F | W | Y | I | Q | L | Q | I | 272 | | Q6CJ79/1-659 | 212 | - | - | - | - | - | - | - | - | - | - | - | A | K | K | D | D | K | P | V | I | S | Y | G | T | C | Q | F | P | T | L | G | F | V | V | D | R | Y | E | R | V | I | N | F | I | P | E | Q | F | W | H | I | L | L | S | V | 255 | | Q6FSR2/1-650 | 216 | - | - | - | - | - | - | - | - | - | - | - | - | N | R | R | D | M | P | V | I | S | Y | G | T | C | Q | F | P | T | L | G | F | V | V | D | R | F | E | R | I | R | H | F | I | P | E | E | F | W | Y | I | Q | L | N | I | 258 | | Q751B3/1-631 | 207 | - | - | - | - | - | - | - | - | - | - | - | A | N | E | K | K | R | A | L | I | S | Y | G | T | C | Q | F | P | T | L | G | F | V | V | D | R | Y | E | R | I | Q | N | F | V | S | E | G | F | W | Y | L | Q | L | L | I | 250 | | A7TSV2/1-650 | 216 | - | - | - | - | - | - | - | - | - | - | - | - | A | K | K | E | N | V | V | I | S | Y | G | T | C | Q | F | P | T | L | G | F | V | V | D | R | Y | E | R | I | R | H | F | I | P | E | E | F | W | H | I | Q | L | Q | I | 258 | | C5DKW7/1-627 | 207 | - | - | - | - | - | - | - | - | - | - | - | - | K | N | D | Q | R | P | I | I | S | Y | G | T | C | Q | F | P | T | L | G | F | V | V | D | R | F | E | R | I | R | K | F | V | P | E | K | F | W | F | I | Q | I | S | I | 249 | | C5DT16/1-650 | 219 | N | - | - | - | - | - | - | - | - | - | - | R | R | K | D | V | S | T | V | I | S | Y | G | T | C | Q | F | P | T | L | G | F | V | V | D | R | F | E | R | I | R | N | F | I | A | E | E | F | W | Y | I | Q | L | E | I | 263 | | Kwal\_23.3190/1-626 | 207 | - | - | - | - | - | - | - | - | - | - | - | - | - | N | D | Q | R | P | I | I | S | Y | G | T | C | Q | F | P | T | L | G | F | V | V | D | R | F | E | R | I | R | K | F | I | P | E | K | F | W | F | I | Q | I | A | I | 248 | | Sbay\_603.19/1-654 | 219 | R | G | - | - | - | - | - | - | - | - | - | - | G | K | N | D | S | Q | V | V | S | Y | G | T | C | Q | F | P | T | L | G | F | V | V | D | R | F | E | R | I | R | N | F | V | P | E | E | F | W | Y | I | Q | L | V | V | 263 | | SAKL0H13310g/1-638 | 216 | - | - | - | - | - | - | - | - | - | - | - | K | G | R | P | K | D | L | V | V | S | Y | G | T | C | Q | F | P | T | L | G | F | V | V | D | R | Y | E | R | I | R | K | F | V | P | E | E | F | W | Y | L | Q | I | S | V | 259 | | P13099/1-656 | 219 | R | G | - | - | - | - | - | - | - | - | - | G | N | K | N | D | S | Q | V | V | S | Y | G | T | C | Q | F | P | T | L | G | F | V | V | D | R | F | E | R | I | R | N | F | V | P | E | E | F | W | Y | I | Q | L | V | V | 264 | |  | | G0VCQ8/1-663 | 273 | K | N | A | D | T | G | - | - | D | S | T | T | F | Q | W | D | R | G | H | L | F | D | R | L | S | V | L | T | F | Y | E | S | C | I | E | T | A | G | D | K | A | K | V | V | D | L | K | S | K | P | T | S | K | Y | R | 325 | | Q6CJ79/1-659 | 256 | K | D | L | D | G | S | E | - | S | K | V | K | F | Q | W | E | R | G | H | L | F | D | R | L | A | V | L | S | I | Y | E | H | C | I | G | T | C | E | D | R | A | K | V | I | H | V | S | S | K | D | T | K | K | F | R | 309 | | Q6FSR2/1-650 | 259 | K | G | E | E | G | E | S | - | K | P | I | Q | F | Q | W | D | R | G | R | L | F | D | R | F | S | V | L | S | I | Y | E | M | C | L | E | Q | D | G | D | S | A | R | V | S | D | I | K | S | K | P | T | S | K | Y | K | 312 | | Q751B3/1-631 | 251 | E | D | P | Q | C | A | - | - | R | K | V | T | F | S | W | D | R | G | H | L | F | D | R | L | C | V | L | C | L | Y | E | T | C | I | E | A | T | G | N | K | A | L | V | S | S | V | T | A | K | D | T | S | K | Y | R | 303 | | A7TSV2/1-650 | 259 | E | N | K | D | K | N | G | G | E | F | T | T | F | Q | W | E | R | G | H | I | F | D | R | L | T | V | L | T | F | Y | E | V | C | L | E | L | A | G | N | Q | A | K | V | I | D | L | K | S | K | P | T | S | K | Y | R | 313 | | C5DKW7/1-627 | 250 | P | N | D | S | - | - | - | - | E | K | T | V | F | Q | W | E | R | G | H | L | F | D | R | L | A | V | A | V | I | Y | E | L | C | L | E | A | S | G | N | I | A | K | V | A | D | L | K | S | K | P | T | T | K | W | R | 300 | | C5DT16/1-650 | 264 | E | S | S | E | N | - | - | - | G | M | T | A | F | A | W | E | R | G | N | L | F | D | R | L | T | V | F | T | F | Y | E | N | C | I | E | L | A | G | N | R | A | K | V | V | D | V | K | S | R | P | T | S | K | Y | R | 315 | | Kwal\_23.3190/1-626 | 249 | P | N | E | S | - | - | - | - | D | K | T | V | F | Q | W | D | R | G | H | L | F | D | R | F | A | V | A | V | I | Y | E | L | C | I | E | S | A | A | N | V | A | T | V | A | D | I | K | S | K | P | T | S | K | W | R | 299 | | Sbay\_603.19/1-654 | 264 | E | N | K | D | S | G | - | - | G | V | T | T | F | Q | W | D | R | G | H | L | F | D | R | L | S | V | L | T | F | Y | E | T | C | I | E | T | A | G | N | A | A | K | V | V | D | L | R | S | K | P | T | S | K | Y | R | 316 | | SAKL0H13310g/1-638 | 260 | K | N | D | E | - | - | - | - | D | T | T | T | F | Q | W | D | R | G | H | L | F | D | R | L | G | V | A | I | I | Y | D | C | C | V | E | T | S | N | F | Q | A | Q | V | V | D | V | R | S | K | P | A | T | K | Y | R | 310 | | P13099/1-656 | 265 | E | N | K | D | N | G | - | - | G | T | T | T | F | Q | W | D | R | G | H | L | F | D | R | L | S | V | L | T | F | Y | E | T | C | I | E | T | A | G | N | V | A | Q | V | V | D | L | K | S | K | P | T | T | K | Y | R | 317 | |  | | G0VCQ8/1-663 | 326 | P | L | P | L | T | T | V | E | L | Q | R | N | C | A | R | F | L | R | M | N | A | K | K | S | L | A | A | A | E | K | L | Y | Q | K | G | F | V | S | Y | P | R | T | E | T | D | T | F | P | Q | N | M | D | L | K | S | 380 | | Q6CJ79/1-659 | 310 | P | L | P | L | T | T | V | E | L | Q | K | N | C | S | R | F | F | K | L | S | A | K | K | T | L | D | A | A | E | K | L | Y | Q | Q | G | Y | I | S | Y | P | R | T | E | T | D | K | F | P | A | K | T | D | L | K | S | 364 | | Q6FSR2/1-650 | 313 | P | L | P | L | T | T | V | E | L | Q | K | N | C | A | R | F | L | R | I | N | A | K | Q | S | L | D | A | A | E | K | L | Y | Q | R | G | F | I | S | Y | P | R | T | E | T | N | V | F | P | S | T | M | D | L | K | S | 367 | | Q751B3/1-631 | 304 | P | L | P | L | T | T | V | E | L | Q | K | N | C | S | K | F | L | R | M | S | A | K | Q | S | L | D | A | A | E | K | L | Y | Q | K | G | F | I | S | Y | P | R | T | E | T | D | V | F | S | K | Q | A | N | L | Q | G | 358 | | A7TSV2/1-650 | 314 | P | L | P | L | T | T | V | E | L | Q | K | N | C | A | R | F | L | R | L | N | A | K | Q | S | L | D | A | A | E | K | L | Y | Q | K | G | F | I | S | Y | P | R | T | E | T | D | I | F | P | Q | T | M | N | L | K | S | 368 | | C5DKW7/1-627 | 301 | P | L | P | L | T | T | V | E | L | Q | K | N | C | S | R | F | L | K | M | S | A | K | T | S | L | D | A | A | E | K | L | Y | Q | K | G | Y | I | S | Y | P | R | T | E | T | D | I | F | P | P | N | M | D | L | Q | A | 355 | | C5DT16/1-650 | 316 | P | L | P | L | T | T | V | E | L | Q | K | N | C | A | R | F | L | K | M | N | A | K | Q | S | L | D | A | A | E | K | L | Y | Q | K | G | F | I | S | Y | P | R | T | E | T | D | T | F | P | K | N | M | N | L | R | S | 370 | | Kwal\_23.3190/1-626 | 300 | P | L | P | L | T | T | V | E | L | Q | K | N | C | S | R | F | F | R | M | S | A | K | T | S | L | D | A | A | E | K | L | Y | Q | K | G | Y | I | S | Y | P | R | T | E | T | D | M | F | P | S | S | M | D | L | K | S | 354 | | Sbay\_603.19/1-654 | 317 | P | L | P | L | T | T | V | E | L | Q | K | N | C | A | R | Y | L | R | L | N | A | K | Q | S | L | D | A | A | E | K | L | Y | Q | K | G | F | I | S | Y | P | R | T | E | T | D | S | F | P | Q | A | M | D | L | K | S | 371 | | SAKL0H13310g/1-638 | 311 | P | L | P | L | T | T | V | E | L | Q | K | N | C | A | R | F | F | K | M | S | A | K | K | S | L | D | A | A | E | K | L | Y | Q | K | G | F | I | S | Y | P | R | T | E | T | D | T | F | P | Q | K | M | D | L | K | A | 365 | | P13099/1-656 | 318 | P | L | P | L | T | T | V | E | L | Q | K | N | C | A | R | Y | L | R | L | N | A | K | Q | S | L | D | A | A | E | K | L | Y | Q | K | G | F | I | S | Y | P | R | T | E | T | D | T | F | P | H | A | M | D | L | K | S | 372 | |  | | G0VCQ8/1-663 | 381 | L | V | E | K | Q | A | Q | L | D | Q | P | - | G | N | N | K | T | A | W | A | S | Y | A | E | S | L | I | S | E | S | S | - | S | S | N | N | K | F | R | F | P | R | S | G | S | H | D | D | K | A | H | P | P | I | H | 433 | | Q6CJ79/1-659 | 365 | L | L | N | K | H | Q | G | - | - | - | - | - | - | - | - | H | S | E | W | G | P | Y | V | T | N | L | M | N | E | N | S | - | T | S | T | N | K | Y | Q | W | P | R | S | G | N | H | D | D | Q | A | H | P | P | I | H | 410 | | Q6FSR2/1-650 | 368 | L | V | E | K | Q | A | Q | L | D | Q | P | - | A | Q | G | R | S | D | W | A | E | Y | A | A | N | L | L | N | G | S | G | - | S | N | G | N | R | F | M | F | P | R | S | G | N | R | D | D | K | A | H | P | P | I | H | 420 | | Q751B3/1-631 | 359 | L | V | Q | Q | Q | T | E | - | - | - | - | - | - | - | - | H | S | Q | W | G | A | Y | A | S | K | L | L | S | - | - | G | - | E | G | S | N | R | F | Q | W | P | R | E | G | K | H | D | D | Q | A | H | P | P | I | H | 402 | | A7TSV2/1-650 | 369 | L | V | E | K | Q | A | Q | L | D | Q | - | - | G | N | H | R | S | P | W | A | A | Y | A | E | Q | L | I | S | - | D | A | - | P | G | T | N | K | F | K | F | P | R | S | G | T | H | D | D | K | A | H | P | P | I | H | 419 | | C5DKW7/1-627 | 356 | L | V | Q | Q | Q | T | N | - | - | - | - | - | - | - | - | D | R | R | W | G | E | Y | A | Q | N | L | L | N | - | - | G | - | N | G | P | N | C | F | K | W | P | R | A | G | K | N | D | D | K | A | H | P | P | I | H | 399 | | C5DT16/1-650 | 371 | L | I | E | K | Q | A | Q | L | G | Q | - | - | - | E | G | R | S | V | W | A | N | Y | A | E | Q | L | I | S | - | S | S | - | P | G | S | N | K | F | K | T | P | R | S | G | S | H | D | D | K | A | H | P | P | I | H | 420 | | Kwal\_23.3190/1-626 | 355 | L | V | Q | Q | Q | T | T | - | - | - | - | - | - | - | - | D | S | R | W | G | D | Y | A | Q | N | L | L | N | - | - | E | - | N | G | P | N | S | F | Q | W | P | R | A | G | K | N | D | D | K | A | H | P | P | I | H | 398 | | Sbay\_603.19/1-654 | 372 | L | V | E | K | Q | A | Q | L | D | Q | P | G | T | G | G | K | T | A | W | A | E | Y | A | A | S | L | L | L | P | E | N | - | T | S | G | N | R | F | K | P | P | R | S | G | T | H | D | D | K | A | H | P | P | I | H | 425 | | SAKL0H13310g/1-638 | 366 | L | I | E | Q | Q | T | N | - | - | - | - | - | - | - | - | D | A | R | W | S | S | Y | S | Q | K | L | L | T | - | - | G | - | S | G | P | N | K | F | K | W | P | R | S | G | N | H | D | D | K | A | H | P | P | I | H | 409 | | P13099/1-656 | 373 | L | V | E | K | Q | A | Q | L | D | Q | L | A | A | G | G | R | T | A | W | A | S | Y | A | A | S | L | L | Q | P | E | N | T | S | N | N | N | K | F | K | F | P | R | S | G | S | H | D | D | K | A | H | P | P | I | H | 427 | |  | | G0VCQ8/1-663 | 434 | P | I | V | P | V | - | - | - | S | G | N | A | - | N | L | N | I | D | E | K | R | V | Y | E | Y | I | V | R | H | F | L | A | C | C | S | E | D | A | K | G | Q | S | T | T | L | I | L | D | W | A | G | E | K | F | S | 484 | | Q6CJ79/1-659 | 411 | P | I | L | C | V | D | I | A | K | T | D | N | - | R | M | T | S | D | E | K | T | V | Y | N | Y | V | V | K | H | F | L | A | C | C | S | T | D | A | R | G | R | S | T | T | L | K | L | D | W | G | G | E | T | F | Y | 464 | | Q6FSR2/1-650 | 421 | P | I | V | S | V | - | - | - | G | R | D | V | T | S | L | N | P | T | E | K | R | V | Y | E | Y | I | C | R | H | F | L | A | S | C | S | E | D | A | K | G | Q | Q | T | T | I | S | L | D | W | S | G | E | R | F | Y | 472 | | Q751B3/1-631 | 403 | P | V | L | C | A | - | - | - | Q | P | D | A | - | N | L | S | V | D | E | K | R | V | Y | E | Y | V | V | R | H | F | L | A | C | C | S | Q | D | A | K | G | R | S | S | K | I | K | L | K | W | H | T | E | E | F | S | 453 | | A7TSV2/1-650 | 420 | P | I | I | S | L | - | - | - | G | A | K | A | T | N | I | T | P | V | E | R | S | V | Y | E | Y | V | V | R | H | F | L | A | C | C | S | E | D | A | K | G | L | S | T | T | L | T | L | D | W | A | G | E | K | F | T | 471 | | C5DKW7/1-627 | 400 | P | I | V | S | C | - | - | - | G | E | G | L | - | D | - | N | E | N | E | K | K | V | Y | E | Y | V | T | R | H | F | L | A | C | C | S | E | D | S | K | G | H | S | T | T | M | V | L | H | W | G | P | E | A | F | T | 449 | | C5DT16/1-650 | 421 | P | I | I | S | L | - | - | - | G | R | Q | A | - | N | L | S | R | V | E | H | Q | V | Y | E | Y | V | T | R | H | F | L | A | C | C | S | E | D | A | K | G | Q | S | T | T | L | T | L | D | W | G | G | E | R | F | T | 471 | | Kwal\_23.3190/1-626 | 399 | P | I | V | S | S | - | - | - | G | A | G | L | - | D | - | N | E | N | E | R | K | V | Y | E | Y | V | T | R | H | F | L | A | C | C | S | Q | D | A | K | G | H | S | T | T | M | I | L | N | W | G | P | E | S | F | T | 448 | | Sbay\_603.19/1-654 | 426 | P | I | V | S | L | - | - | - | G | P | D | A | - | N | V | S | P | T | E | R | R | I | Y | E | Y | V | A | R | H | F | L | A | S | C | S | E | D | A | R | G | Q | A | T | T | L | V | L | D | W | A | G | E | R | F | T | 476 | | SAKL0H13310g/1-638 | 410 | P | I | V | S | A | - | - | - | A | T | S | A | - | Q | L | T | A | D | E | K | K | I | Y | E | Y | V | V | R | H | F | L | A | C | C | S | E | D | A | R | G | Q | S | S | S | I | A | L | Q | W | G | G | E | K | F | S | 460 | | P13099/1-656 | 428 | P | I | V | S | L | - | - | - | G | P | E | A | - | N | V | S | P | V | E | R | R | V | Y | E | Y | V | A | R | H | F | L | A | C | C | S | E | D | A | K | G | Q | S | M | T | L | V | L | D | W | A | V | E | R | F | S | 478 | |  | | G0VCQ8/1-663 | 485 | A | S | G | L | I | V | L | E | R | N | F | L | D | V | Y | P | W | A | K | W | E | T | T | K | R | L | P | R | L | E | M | N | E | E | C | E | I | S | K | A | D | M | L | S | G | N | T | S | P | P | K | P | M | T | E | 539 | | Q6CJ79/1-659 | 465 | A | N | G | I | Q | V | L | E | R | N | F | L | D | I | Y | Q | W | A | D | W | K | S | N | D | T | L | P | A | Y | Q | M | S | D | D | V | V | V | S | K | T | E | M | K | S | G | Q | T | S | P | P | K | H | M | T | E | 519 | | Q6FSR2/1-650 | 473 | A | N | G | L | M | V | T | E | R | N | F | L | D | V | Y | P | W | A | R | W | E | T | T | R | Q | L | P | R | L | E | I | N | Q | Q | C | K | I | L | K | A | E | M | K | N | G | S | T | S | P | P | K | P | M | T | E | 527 | | Q751B3/1-631 | 454 | A | T | G | L | Q | V | H | E | E | N | F | L | E | I | Y | T | Y | Q | K | W | T | S | S | E | Q | L | P | T | L | P | L | N | S | Q | V | E | F | A | K | A | E | M | K | S | G | K | T | S | P | P | K | Y | I | T | E | 508 | | A7TSV2/1-650 | 472 | A | S | G | L | V | V | L | E | R | N | F | L | D | V | Y | P | W | A | K | W | E | S | T | K | Q | L | P | R | L | E | M | N | E | I | C | T | I | G | K | A | E | M | K | S | G | K | T | S | P | P | K | P | M | T | E | 526 | | C5DKW7/1-627 | 450 | A | N | G | I | V | V | L | E | R | N | F | L | D | V | Y | L | W | M | K | W | E | T | T | K | Q | L | P | N | L | N | V | G | Q | E | V | E | L | L | T | Y | L | M | K | D | G | Q | T | S | P | P | N | Q | M | T | E | 504 | | C5DT16/1-650 | 472 | A | T | G | L | V | V | L | E | R | N | F | L | E | V | Y | P | W | A | K | W | E | T | T | K | R | L | P | Q | L | E | I | N | Q | E | C | Q | I | S | K | A | R | M | K | S | G | S | T | S | P | P | K | P | M | T | E | 526 | | Kwal\_23.3190/1-626 | 449 | A | S | G | I | I | V | L | E | R | N | F | L | D | V | Y | V | W | M | K | W | E | T | T | K | Q | L | P | N | L | N | V | G | E | D | V | N | L | L | S | Y | I | M | K | D | G | Q | T | S | P | P | N | Q | M | T | E | 503 | | Sbay\_603.19/1-654 | 477 | A | A | G | L | V | V | L | E | R | N | F | L | D | V | Y | P | W | A | R | W | E | T | T | K | Q | L | P | R | L | D | L | H | A | S | V | D | I | A | K | A | E | M | K | A | G | A | T | A | P | P | K | P | M | T | E | 531 | | SAKL0H13310g/1-638 | 461 | A | T | G | I | I | V | L | Q | R | N | F | L | E | I | Y | P | W | M | K | W | E | T | T | K | Q | L | P | H | L | E | L | S | Q | T | V | S | L | C | N | T | E | M | K | S | G | K | T | S | P | P | N | H | M | T | E | 515 | | P13099/1-656 | 479 | A | S | G | L | V | V | L | E | R | N | F | L | D | V | Y | P | W | A | R | W | E | T | T | K | Q | L | P | R | L | E | M | N | A | L | V | D | I | A | K | A | E | M | K | A | G | T | T | A | P | P | K | P | M | T | E | 533 | |  | | G0VCQ8/1-663 | 540 | S | E | L | I | M | L | M | D | A | N | G | I | G | T | D | A | T | I | A | D | H | I | D | K | I | Q | V | R | Q | Y | I | R | S | E | - | - | - | - | - | - | K | V | G | K | E | T | F | L | Q | P | T | I | L | G | V | 588 | | Q6CJ79/1-659 | 520 | S | E | L | I | M | L | M | D | A | N | G | I | G | T | D | A | T | I | A | D | H | I | E | K | I | K | S | R | N | Y | I | K | V | Q | T | G | S | G | S | G | A | K | K | T | A | S | V | F | L | P | T | S | L | G | R | 574 | | Q6FSR2/1-650 | 528 | S | E | L | I | V | L | M | D | A | N | G | I | G | T | D | A | T | I | A | E | H | I | E | K | I | Q | M | R | S | Y | V | Q | S | Q | - | - | - | - | - | - | K | V | G | K | E | T | Y | L | Q | P | T | I | L | G | R | 576 | | Q751B3/1-631 | 509 | S | E | L | I | M | L | M | D | A | N | G | I | G | T | D | A | T | I | A | E | H | I | E | K | I | Q | E | R | Q | Y | I | K | A | E | - | - | - | - | - | - | G | T | A | K | N | K | V | F | K | P | T | M | L | G | R | 557 | | A7TSV2/1-650 | 527 | S | E | L | I | S | L | M | D | V | N | G | I | G | T | D | A | T | I | A | E | H | I | E | K | I | Q | V | R | K | Y | I | K | S | E | - | - | - | - | - | - | K | S | G | K | E | T | Y | L | Q | P | T | T | L | G | I | 575 | | C5DKW7/1-627 | 505 | S | E | L | I | L | L | M | D | A | N | G | I | G | T | D | A | T | I | A | E | H | I | E | K | I | Q | Q | R | D | Y | I | K | K | K | - | - | - | - | - | - | K | V | N | K | Q | T | Y | L | I | P | T | T | L | G | I | 553 | | C5DT16/1-650 | 527 | S | E | L | I | M | L | M | D | A | N | G | I | G | T | D | A | T | I | A | E | H | I | E | K | I | Q | T | R | N | Y | V | R | S | E | - | - | - | - | - | - | K | V | G | K | E | T | Y | L | Q | P | T | T | L | G | V | 575 | | Kwal\_23.3190/1-626 | 504 | S | E | L | I | L | L | M | D | A | N | G | I | G | T | D | A | T | I | A | E | H | I | E | K | I | Q | Q | R | D | Y | I | K | K | K | - | - | - | - | - | - | K | V | N | K | Q | T | Y | L | L | P | T | T | L | G | V | 552 | | Sbay\_603.19/1-654 | 532 | S | E | L | I | I | L | M | D | A | N | G | I | G | T | D | A | T | I | A | E | H | I | D | K | I | Q | V | R | N | Y | V | R | S | E | - | - | - | - | - | - | K | A | G | K | E | T | Y | L | Q | P | T | T | L | G | V | 580 | | SAKL0H13310g/1-638 | 516 | S | E | L | I | L | L | M | D | A | N | G | I | G | T | D | A | T | I | A | E | H | I | E | K | I | Q | Q | R | K | Y | I | K | A | E | - | - | - | - | - | - | K | S | G | K | Q | V | V | L | K | P | T | V | L | G | I | 564 | | P13099/1-656 | 534 | S | E | L | I | L | L | M | D | T | N | G | I | G | T | D | A | T | I | A | E | H | I | D | K | I | Q | V | R | N | Y | V | R | S | E | - | - | - | - | - | - | K | V | G | K | E | T | Y | L | Q | P | T | T | L | G | V | 582 | |  | | G0VCQ8/1-663 | 589 | A | L | V | H | G | F | E | T | I | G | L | E | D | S | F | A | K | P | F | Q | R | R | E | M | E | E | D | L | K | K | I | C | E | G | R | L | S | K | E | E | V | I | V | D | I | V | G | K | Y | R | D | Y | W | Q | R | 643 | | Q6CJ79/1-659 | 575 | S | L | V | H | G | F | E | K | I | G | L | E | E | S | F | S | K | P | F | L | R | R | D | L | E | K | D | L | Q | L | I | C | G | G | T | K | D | K | K | D | V | V | S | L | I | I | G | M | Y | R | N | Y | Y | Q | I | 629 | | Q6FSR2/1-650 | 577 | A | L | V | H | G | F | E | A | I | G | L | E | D | S | F | A | K | P | F | Q | R | R | E | M | E | E | M | L | R | Q | I | C | D | G | A | T | D | R | P | R | V | V | S | D | I | L | S | K | F | R | R | Y | W | N | K | 631 | | Q751B3/1-631 | 558 | S | L | V | H | G | F | E | D | I | G | L | E | E | S | F | A | K | P | F | L | R | R | D | M | E | L | D | L | K | R | I | C | E | G | T | K | D | R | N | S | V | L | A | N | L | I | G | M | Y | M | D | Y | Y | D | Q | 612 | | A7TSV2/1-650 | 576 | A | L | V | H | G | F | E | E | I | G | L | E | D | S | F | A | K | P | F | Q | R | R | E | M | E | E | E | L | K | K | I | C | E | G | N | R | T | K | N | E | V | V | V | D | I | V | E | K | Y | K | K | Y | W | I | K | 630 | | C5DKW7/1-627 | 554 | S | L | V | H | G | F | E | A | I | G | L | E | D | S | F | A | K | P | F | L | R | R | E | M | E | L | D | L | K | L | I | C | S | G | N | K | N | R | A | D | V | V | K | D | L | I | N | K | Y | Q | N | Y | Y | G | L | 608 | | C5DT16/1-650 | 576 | A | L | V | H | G | F | E | A | I | G | L | E | D | S | F | A | K | P | F | Q | R | R | E | M | E | V | E | L | K | Q | I | C | E | G | L | R | T | K | N | Q | V | V | D | D | I | V | N | K | Y | R | R | Y | W | A | K | 630 | | Kwal\_23.3190/1-626 | 553 | S | L | V | H | G | F | E | A | I | G | L | E | D | S | F | A | K | P | F | L | R | R | Q | M | E | L | D | L | K | L | I | C | D | G | N | K | N | R | A | D | V | V | K | D | F | I | N | K | Y | Q | D | Y | Y | G | L | 607 | | Sbay\_603.19/1-654 | 581 | S | L | V | H | G | F | E | A | I | G | L | E | D | S | F | A | K | P | F | Q | R | R | E | M | E | Q | D | L | K | K | I | C | D | G | H | A | S | R | A | D | V | V | S | D | M | V | E | K | Y | R | K | Y | W | L | K | 635 | | SAKL0H13310g/1-638 | 565 | S | L | V | H | G | F | E | T | I | G | L | E | D | S | F | A | K | P | F | L | R | R | D | L | E | V | D | L | K | L | I | C | Q | G | E | K | E | K | R | Q | V | V | Y | D | M | V | E | M | Y | R | D | Y | F | N | Q | 619 | | P13099/1-656 | 583 | S | L | V | H | G | F | E | A | I | G | L | E | D | S | F | A | K | P | F | Q | R | R | E | M | E | Q | D | L | K | K | I | C | E | G | H | A | S | K | T | D | V | V | K | D | I | V | E | K | Y | R | K | Y | W | H | K | 637 | |  | | G0VCQ8/1-663 | 644 | T | N | S | A | K | N | T | L | L | E | V | F | D | R | V | R | Q | S | M | - | - | - | - | - | - | - | - | - | - | S |  | | | | | | | | | | | | | | | | | | | | | | | | | 663 | | Q6CJ79/1-659 | 630 | T | T | S | Q | M | R | N | L | I | K | V | Y | N | D | I | K | A | E | S | Q | G | Q | V | T | Q | R | N | R | V | N |  | | | | | | | | | | | | | | | | | | | | | | | | | 659 | | Q6FSR2/1-650 | 632 | T | N | G | S | K | N | T | L | L | E | V | Y | D | R | T | L | R | N | Q | - | - | - | - | - | - | - | - | - | - | - |  | | | | | | | | | | | | | | | | | | | | | | | | | 650 | | Q751B3/1-631 | 613 | T | D | R | Q | R | S | K | L | I | H | C | Y | E | R | I | K | Q | E | S | - | - | - | - | - | - | - | - | - | - | - |  | | | | | | | | | | | | | | | | | | | | | | | | | 631 | | A7TSV2/1-650 | 631 | T | N | G | S | K | N | T | L | L | E | V | F | D | R | V | R | R | T | V | - | - | - | - | - | - | - | - | - | - | I |  | | | | | | | | | | | | | | | | | | | | | | | | | 650 | | C5DKW7/1-627 | 609 | T | S | T | K | Q | V | K | L | I | E | T | Y | T | N | I | K | S | V | M | - | - | - | - | - | - | - | - | - | - | - |  | | | | | | | | | | | | | | | | | | | | | | | | | 627 | | C5DT16/1-650 | 631 | T | N | G | S | K | T | T | L | L | Q | V | Y | D | R | V | K | A | S | M | - | - | - | - | - | - | - | - | - | - | S |  | | | | | | | | | | | | | | | | | | | | | | | | | 650 | | Kwal\_23.3190/1-626 | 608 | T | F | T | K | Q | N | K | L | I | E | T | Y | N | N | I | K | S | V | M | - | - | - | - | - | - | - | - | - | - | - |  | | | | | | | | | | | | | | | | | | | | | | | | | 626 | | Sbay\_603.19/1-654 | 636 | T | N | G | C | K | N | T | L | L | H | V | Y | D | R | V | K | T | S | M | - | - | - | - | - | - | - | - | - | - | - |  | | | | | | | | | | | | | | | | | | | | | | | | | 654 | | SAKL0H13310g/1-638 | 620 | T | M | R | S | K | N | S | L | L | D | V | Y | D | R | V | K | A | N | M | - | - | - | - | - | - | - | - | - | - | - |  | | | | | | | | | | | | | | | | | | | | | | | | | 638 | | P13099/1-656 | 638 | T | N | A | C | K | N | T | L | L | Q | V | Y | D | R | V | K | A | S | M | - | - | - | - | - | - | - | - | - | - | - |  | | | | | | | | | | | | | | | | | | | | | | | | | 656 | |
