# Supplementary material for: Plus ça change – evolutionary sequence divergence predicts protein subcellular localization signals
Source: BMC Genomics. 2014 Jan 20;15:46. doi: 10.1186/1471-2164-15-46 (PMC3906766; doi:10.1186/1471-2164-15-46)
Supplement: Additional file 2 — MSA’s of proteins for which sequence divergence changes predicted localization signals. Contains links to ortholog multiple sequence alignments of each protein in Additional file 3: Table S1. [file 1471-2164-15-46-S2.zip › P25039.html]

|  |  |  |  |  |  |  |  |  |  |  |  |  |  |  |  |  |  |  |  |  |  |  |  |  |  |  |  |  |  |  |  |  |  |  |  |  |  |  |  |  |  |  |  |  |  |  |  |  |  |  |  |  |  |  |  |  |  |  |  |  |  |  |  |  |  |  |  |  |  |  |  |  |  |  |  |  |  |  |  |  |  |  |  |  |  |  |  |  |  |  |  |  |  |  |  |  |  |  |  |  |  |  |  |  |  |  |  |  |  |  |  |  |  |  |  |  |  |  |  |  |  |  |  |  |  |  |  |  |  |  |  |  |  |  |  |  |  |  |  |  |  |  |  |  |  |  |  |  |  |  |  |  |  |  |  |  |  |  |  |  |  |  |  |  |  |  |  |  |  |  |  |  |  |  |  |  |  |  |  |  |  |  |  |  |  |  |  |  |  |  |  |  |  |  |  |  |  |  |  |  |  |  |  |  |  |  |  |  |  |  |  |  |  |  |  |  |  |  |  |  |  |  |  |  |  |  |  |  |  |  |  |  |  |  |  |  |  |  |  |  |  |  |  |  |  |  |  |  |  |  |  |  |  |  |  |  |  |  |  |  |  |  |  |  |  |  |  |  |  |  |  |  |  |  |  |  |  |  |  |  |  |  |  |  |  |  |  |  |  |  |  |  |  |  |  |  |  |  |  |  |  |  |  |  |  |  |  |  |  |  |  |  |  |  |  |  |  |  |  |  |  |  |  |  |  |  |  |  |  |  |  |  |  |  |  |  |  |  |  |  |  |  |  |  |  |  |  |  |  |  |  |  |  |  |  |  |  |  |  |  |  |  |  |  |  |  |  |  |  |  |  |  |  |  |  |  |  |  |  |  |  |  |  |  |  |  |  |  |  |  |  |  |  |  |  |  |  |  |  |  |  |  |  |  |  |  |  |  |  |  |  |  |  |  |  |  |  |  |  |  |  |  |  |  |  |  |  |  |  |  |  |  |  |  |  |  |  |  |  |  |  |  |  |  |  |  |  |  |  |  |  |  |  |  |  |  |  |  |  |  |  |  |  |  |  |  |  |  |  |  |  |  |  |  |  |  |  |  |  |  |  |  |  |  |  |  |  |  |  |  |  |  |  |  |  |  |  |  |  |  |  |  |  |  |  |  |  |  |  |  |  |  |  |  |  |  |  |  |  |  |  |  |  |  |  |  |  |  |  |  |  |  |  |  |  |  |  |  |  |  |  |  |  |  |  |  |  |  |  |  |  |  |  |  |  |  |  |  |  |  |  |  |  |  |  |  |  |  |  |  |  |  |  |  |  |  |  |  |  |  |  |  |  |  |  |  |  |  |  |  |  |  |  |  |  |  |  |  |  |  |  |  |  |  |  |  |  |  |  |  |  |  |  |  |  |  |  |  |  |  |  |  |  |  |  |  |  |  |  |  |  |  |  |  |  |  |  |  |  |  |  |  |  |  |  |  |  |  |  |  |  |  |  |  |  |  |  |  |  |  |  |  |  |  |  |  |  |  |  |  |  |  |  |  |  |  |  |  |  |  |  |  |  |  |  |  |  |  |  |  |  |  |  |  |  |  |  |  |  |  |  |  |  |  |  |  |  |  |  |  |  |  |  |  |  |  |  |  |  |  |  |  |  |  |  |  |  |  |  |  |  |  |  |  |  |  |  |  |  |  |  |  |  |  |  |  |  |  |  |  |  |  |  |  |  |  |  |  |  |  |  |  |  |  |  |  |  |  |  |  |  |  |  |  |  |  |  |  |  |  |  |  |  |  |  |  |  |  |  |  |  |  |  |  |  |  |  |  |  |  |  |  |  |  |  |  |  |  |  |  |  |  |  |  |  |  |  |  |  |  |  |  |  |  |  |  |  |  |  |  |  |  |  |  |  |  |  |  |  |  |  |  |  |  |  |  |  |  |  |  |  |  |  |  |  |  |  |  |  |  |  |  |  |  |  |  |  |  |  |  |  |  |  |  |  |  |  |  |  |  |  |  |  |  |  |  |  |  |  |  |  |  |  |  |  |  |  |  |  |  |  |  |  |  |  |  |  |  |  |  |  |  |  |  |  |  |  |  |  |  |  |  |  |  |  |  |  |  |  |  |  |  |  |  |  |  |  |  |  |  |  |  |  |  |  |  |  |  |  |  |  |  |  |  |  |  |  |  |  |  |  |  |  |  |  |  |  |  |  |  |  |  |  |  |  |  |  |  |  |  |  |  |  |  |  |  |  |  |  |  |  |  |  |  |  |  |  |  |  |  |  |  |  |  |  |  |  |  |  |  |  |  |  |  |  |  |  |  |  |  |  |  |  |  |  |  |  |  |  |  |  |  |  |  |  |  |  |  |  |  |  |  |  |  |  |  |  |  |  |  |  |  |  |  |  |  |  |  |  |  |  |  |  |  |  |  |  |  |  |  |  |  |  |  |  |  |  |  |  |  |  |  |  |  |  |  |  |  |  |  |  |  |  |  |  |  |  |  |  |  |  |  |  |  |  |  |  |  |  |  |  |  |  |  |  |  |  |  |  |  |  |  |  |  |  |  |  |  |  |  |  |  |  |  |  |  |  |  |  |  |  |  |  |  |  |  |  |  |  |  |  |  |  |  |  |  |  |  |  |  |  |  |  |  |  |  |  |  |  |  |  |  |  |  |  |  |  |  |  |  |  |  |  |  |  |  |  |  |  |  |  |  |  |  |  |  |  |  |  |  |  |  |  |  |  |  |  |  |  |  |  |  |  |  |  |  |  |  |  |  |  |  |  |  |  |  |  |  |  |  |  |  |  |  |  |  |  |  |  |  |  |  |  |  |  |  |  |  |  |  |  |  |  |  |  |  |  |  |  |  |  |  |  |  |  |  |  |  |  |  |  |  |  |  |  |  |  |  |  |  |  |  |  |  |  |  |  |  |  |  |  |  |  |  |  |  |  |  |  |  |  |  |  |  |  |  |  |  |  |  |  |  |  |  |  |  |  |  |  |  |  |  |  |  |  |  |  |  |  |  |  |  |  |  |  |  |  |  |  |  |  |  |  |  |  |  |  |  |  |  |  |  |  |  |  |  |  |  |  |  |  |  |  |  |  |  |  |  |  |  |  |  |  |  |  |  |  |  |  |  |  |  |  |  |  |  |  |  |  |  |  |  |  |  |  |  |  |  |  |  |  |  |  |  |  |  |  |  |  |  |  |  |  |  |  |  |  |  |  |  |  |  |  |  |  |  |  |  |  |  |  |  |  |  |  |  |  |  |  |  |  |  |  |  |  |  |  |  |  |  |  |  |  |  |  |  |  |  |  |  |  |  |  |  |  |  |  |  |  |  |  |  |  |  |  |  |  |  |  |  |  |  |  |  |  |  |  |  |  |  |  |  |  |  |  |  |  |  |  |  |  |  |  |  |  |  |  |  |  |  |  |  |  |  |  |  |  |  |  |  |  |  |  |  |  |  |  |  |  |  |  |  |  |  |  |  |  |  |  |  |  |  |  |  |  |  |  |  |  |  |  |  |  |  |  |  |  |  |  |  |  |  |  |  |  |  |  |  |  |  |  |  |  |  |  |  |  |  |  |  |  |  |  |  |  |  |  |  |  |  |  |  |  |  |  |  |  |  |  |  |  |  |  |  |  |  |  |  |  |  |  |  |  |  |  |  |  |  |  |  |  |  |  |  |  |  |  |  |  |  |  |  |  |  |  |  |  |  |  |  |  |  |  |  |  |  |  |  |  |  |  |  |  |  |  |  |  |  |  |  |  |  |  |  |  |  |  |  |  |  |  |  |  |  |  |  |  |  |  |  |  |  |  |  |  |  |  |  |  |  |  |  |  |  |  |  |  |  |  |  |  |  |  |  |  |  |  |  |  |  |  |  |  |  |  |  |  |  |  |  |  |  |  |  |  |  |  |  |  |  |  |  |  |  |  |  |  |  |  |  |  |  |  |  |  |  |  |  |  |  |  |  |  |  |  |  |  |  |  |  |  |  |  |  |  |  |  |  |  |  |  |  |  |  |  |  |  |  |  |  |  |  |  |  |  |  |  |  |  |  |  |  |  |  |  |  |  |  |  |  |  |  |  |  |  |  |  |  |  |  |  |  |  |  |  |  |  |  |  |  |  |  |  |  |  |  |  |  |  |  |  |  |  |  |  |  |  |  |  |  |  |  |  |  |  |  |  |  |  |  |  |  |  |  |  |  |  |  |  |  |  |  |  |  |  |  |  |  |  |  |  |  |  |  |  |  |  |  |  |  |  |  |  |  |  |  |  |  |  |  |  |  |  |  |  |  |  |  |  |  |  |  |  |  |  |  |  |  |  |  |  |  |  |  |  |  |  |  |  |  |  |  |  |  |  |  |  |  |  |  |  |  |  |  |  |  |  |  |  |  |  |  |  |  |  |  |  |  |  |  |  |  |  |  |  |  |  |  |  |  |  |  |  |  |  |  |  |  |  |  |  |  |  |  |  |  |  |  |  |  |  |  |  |  |  |  |  |  |  |  |  |  |  |  |  |  |  |  |  |  |  |  |  |  |  |  |  |  |  |  |  |  |  |  |  |  |  |  |  |  |  |  |  |  |  |  |  |  |  |  |  |  |  |  |  |  |  |  |  |  |  |  |  |  |  |  |  |  |  |  |  |  |  |  |  |  |  |  |  |  |  |  |  |  |  |  |  |  |  |  |  |  |  |  |  |  |  |  |  |  |  |  |  |  |  |  |  |  |  |  |  |  |  |  |  |  |  |  |  |  |  |  |  |  |  |  |  |  |  |  |  |  |  |  |  |  |  |  |  |  |  |  |  |  |  |  |  |  |  |  |  |  |  |  |  |  |  |  |  |  |  |  |  |  |  |  |  |  |  |  |  |  |  |  |  |  |  |  |  |  |  |  |  |  |  |  |  |  |  |  |  |  |  |  |  |  |  |  |  |  |  |  |  |  |  |  |  |  |  |  |  |  |  |  |  |  |  |  |  |  |  |  |  |  |  |  |  |  |  |  |  |  |  |  |  |  |  |  |  |  |  |  |  |  |  |  |  |  |  |  |  |  |  |  |  |  |  |  |  |  |  |  |  |  |  |  |  |  |  |  |  |  |  |  |  |  |  |  |  |  |  |  |  |  |  |  |  |  |  |  |  |  |  |  |  |  |  |  |  |  |  |  |  |  |  |  |  |  |  |  |  |  |  |  |  |  |  |  |  |  |  |  |  |  |  |  |  |  |  |  |  |  |  |  |  |  |  |  |  |  |  |  |  |  |  |  |  |  |  |  |  |  |  |  |  |  |  |  |  |  |  |  |  |  |  |  |  |  |  |  |  |  |  |  |  |  |  |  |  |  |  |  |  |  |  |  |  |  |  |  |  |  |  |  |  |  |  |  |  |  |  |  |  |  |  |  |  |  |  |  |  |  |  |  |  |  |  |  |  |  |  |  |  |  |  |  |  |  |  |  |  |  |  |  |  |  |  |  |  |  |  |  |  |  |  |  |  |  |  |  |  |  |  |  |  |  |  |  |  |  |  |  |  |  |  |  |  |  |  |  |  |  |  |  |  |  |  |  |  |  |  |  |  |  |  |  |  |  |  |  |  |  |  |  |  |  |  |  |  |  |  |  |  |  |  |  |  |  |  |  |  |  |  |  |  |  |  |  |  |  |  |  |  |  |  |  |  |  |  |  |  |  |  |  |  |  |  |  |  |  |  |  |  |  |  |  |  |  |  |  |  |  |  |  |  |  |  |  |  |  |  |  |  |  |  |  |  |  |  |  |  |  |  |  |  |  |  |  |  |  |  |  |  |  |  |  |  |  |  |  |  |  |  |  |  |  |  |  |  |  |  |  |  |  |  |  |  |  |  |  |  |  |  |  |  |  |  |  |  |  |  |  |  |  |  |  |  |  |  |  |  |  |  |  |  |  |  |  |  |  |  |  |  |  |  |  |  |  |  |  |  |  |  |  |  |  |  |  |  |  |  |  |  |  |  |  |  |  |  |  |  |  |  |  |  |  |  |  |  |  |  |  |  |  |  |  |  |  |  |  |  |  |  |  |  |  |  |  |  |  |  |  |  |  |  |  |  |  |  |  |  |  |  |  |  |  |  |  |  |  |  |  |  |  |  |  |  |  |  |  |  |  |  |  |  |  |  |  |  |  |  |  |  |  |  |  |  |  |  |  |  |  |  |  |  |  |  |  |  |  |  |  |  |  |  |  |  |  |  |  |  |  |  |  |  |  |  |  |  |  |  |  |  |  |  |  |  |  |  |  |  |  |  |  |  |  |  |  |  |  |  |  |  |  |  |  |  |  |  |  |  |  |  |  |  |  |  |  |  |  |  |  |  |  |  |  |  |  |  |  |  |  |  |  |  |  |  |  |  |  |  |  |  |  |  |  |  |  |  |  |  |  |  |  |  |  |  |  |  |  |  |  |  |  |  |  |  |  |  |  |  |  |  |  |  |  |  |  |  |  |  |  |  |  |  |  |  |  |  |  |  |  |  |  |  |  |  |  |  |  |  |  |  |  |  |  |  |  |  |  |  |  |  |  |  |  |  |  |  |  |  |  |  |  |  |  |  |  |  |  |  |  |  |  |  |  |  |  |  |  |  |  |  |  |  |  |  |  |  |  |  |  |  |  |  |  |  |  |  |  |  |  |  |  |  |  |  |  |  |  |  |  |  |  |  |  |  |  |  |  |  |  |  |  |  |  |  |  |  |  |  |  |  |  |  |  |  |  |  |  |  |  |  |  |  |  |  |  |  |  |  |  |  |  |  |  |  |  |  |  |  |  |  |  |  |  |  |  |  |  |  |  |  |  |  |  |  |  |  |  |  |  |  |  |  |  |  |  |  |  |  |  |  |  |  |  |  |  |  |  |  |  |  |  |  |  |  |  |  |  |  |  |  |  |  |  |  |  |  |  |  |  |  |  |  |  |  |  |  |  |  |  |  |  |  |  |  |  |  |  |  |  |  |  |  |  |  |  |  |  |  |  |  |  |  |  |  |  |  |  |  |  |  |  |  |  |  |  |  |  |  |  |  |  |  |  |  |  |  |  |  |  |  |  |  |  |  |  |  |  |  |  |  |  |  |  |  |  |  |  |  |  |  |  |  |  |  |  |  |  |  |  |  |  |  |  |  |  |  |  |  |  |  |  |  |  |  |  |  |  |  |  |  |  |  |  |  |  |  |  |  |  |  |  |  |  |  |  |  |  |  |  |  |  |  |  |  |  |  |  |  |  |  |  |  |  |  |  |  |  |  |  |  |  |  |  |  |  |  |  |  |  |  |  |  |  |  |  |  |  |  |  |  |  |  |  |  |  |  |  |  |  |  |  |  |  |  |  |  |  |  |  |  |  |  |  |  |  |  |  |  |  |  |  |  |  |  |  |  |  |  |  |  |  |  |  |  |  |  |  |  |  |  |  |  |  |  |  |  |  |  |  |  |  |  |  |  |  |  |  |  |  |  |  |  |  |  |  |  |  |  |  |  |  |  |  |  |  |  |  |  |  |  |  |  |  |  |  |  |  |  |  |  |  |  |  |  |  |  |  |  |  |  |  |  |  |  |  |  |  |  |  |  |  |  |  |  |  |  |  |  |  |  |  |  |  |  |  |  |  |  |  |  |  |  |  |  |  |  |  |  |  |  |  |  |  |  |  |  |  |  |  |  |  |  |  |  |  |  |  |  |  |  |  |  |  |  |  |  |  |  |  |  |  |  |  |  |  |  |  |  |  |  |  |  |  |  |  |  |  |  |  |  |  |  |  |  |  |  |  |  |  |  |  |  |  |  |  |  |  |  |  |  |  |  |  |  |  |  |  |  |  |  |  |  |  |  |  |  |  |  |  |  |  |  |  |  |  |  |  |  |  |  |  |  |  |  |  |  |  |  |  |  |  |  |  |  |  |  |  |  |  |  |  |  |  |  |  |  |  |  |  |  |  |  |  |  |  |  |  |  |  |  |  |  |  |  |  |  |  |  |  |  |  |  |  |  |  |  |  |  |  |  |  |  |  |  |  |  |  |  |  |  |  |  |  |  |  |  |  |  |  |  |  |  |  |  |  |  |  |  |  |  |  |  |  |  |  |  |  |  |  |  |  |  |  |  |  |  |  |  |  |  |  |  |  |  |  |  |  |  |  |  |  |  |  |  |  |  |  |  |  |  |  |  |  |  |  |  |  |  |  |  |  |  |  |  |  |  |  |  |  |  |  |  |  |  |  |  |  |  |  |  |  |  |  |  |  |  |  |  |  |  |  |  |  |  |  |  |  |  |  |  |  |  |  |  |  |  |  |  |  |  |  |  |  |  |  |  |  |  |  |  |  |  |  |  |  |  |  |  |  |  |  |  |  |  |  |  |  |  |  |  |  |  |  |  |  |  |  |  |  |  |  |  |  |  |  |  |  |  |  |  |  |  |  |  |  |  |  |  |  |  |  |  |  |  |  |  |  |  |  |  |  |  |  |  |  |  |  |  |  |  |  |  |  |  |  |  |  |  |  |  |  |  |  |  |  |  |  |  |  |  |  |  |  |  |  |  |  |  |  |  |  |  |  |  |  |  |  |  |  |  |  |  |  |  |  |  |  |  |  |  |  |  |  |  |  |  |  |  |  |  |  |  |  |  |  |  |  |  |  |  |  |  |  |  |  |  |  |  |  |  |  |  |  |  |  |  |  |  |  |  |  |  |  |  |  |  |  |  |  |  |  |  |  |  |  |  |  |  |  |  |  |  |  |  |  |  |  |  |  |  |  |  |  |  |  |  |  |  |  |  |  |  |  |  |  |  |  |  |  |  |  |  |  |  |  |  |  |  |  |  |  |  |  |  |  |  |  |  |  |  |  |  |  |  |  |  |  |  |  |  |  |  |  |  |  |  |  |  |  |  |  |  |  |  |  |  |  |  |  |  |  |  |  |  |  |  |  |  |  |  |  |  |  |  |  |  |  |  |  |  |  |  |  |  |  |  |  |  |  |  |  |  |  |  |  |  |  |  |  |  |  |  |  |  |  |  |  |  |  |  |  |  |  |  |  |  |  |  |  |  |  |  |  |  |  |  |  |  |  |  |  |  |  |  |  |  |  |  |  |  |  |  |  |  |  |  |  |  |  |  |  |  |  |  |  |  |  |  |  |  |  |  |  |  |  |  |  |  |  |  |  |  |  |  |  |  |  |  |  |  |  |  |  |  |  |  |  |  |  |  |  |  |  |  |  |  |  |  |  |  |  |  |  |  |  |  |  |  |  |  |  |  |  |  |  |  |  |  |  |  |  |  |  |  |  |  |  |  |  |  |  |  |  |  |  |  |  |  |  |  |  |  |  |  |  |  |  |  |  |  |  |  |  |  |  |  |  |  |  |  |  |  |  |  |  |  |  |  |  |  |  |  |  |  |  |  |  |  |  |  |  |  |  |  |  |  |  |  |  |  |  |  |  |  |  |  |  |  |  |  |  |  |  |  |  |  |  |  |  |  |  |  |  |  |  |  |  |  |  |  |  |  |  |  |  |  |  |  |  |  |  |  |  |  |  |  |  |  |  |  |  |  |  |  |  |  |  |  |  |  |  |  |  |  |  |  |  |  |  |  |  |  |  |  |  |  |  |  |  |  |  |  |  |  |  |  |  |  |  |  |  |  |  |  |  |  |  |  |  |  |  |  |  |  |  |  |  |  |  |  |  |  |  |  |  |  |  |  |  |  |  |  |  |  |  |  |  |  |  |  |  |  |  |  |  |  |  |  |  |  |  |  |  |  |  |  |  |  |  |  |  |  |  |  |  |  |  |  |  |  |  |  |  |  |  |  |  |  |  |  |  |  |  |  |  |  |  |  |  |  |  |  |  |  |  |  |  |  |  |  |  |  |  |  |  |  |  |  |  |  |  |  |  |  |  |  |  |  |  |  |  |  |  |  |  |  |  |  |  |  |  |  |  |  |  |  |  |  |  |  |  |  |  |  |  |  |  |  |  |  |  |  |  |  |  |  |  |  |  |  |  |  |  |  |  |  |  |  |  |  |  |  |  |  |  |  |  |  |  |  |  |  |  |  |  |  |  |  |  |  |  |  |  |  |  |  |  |  |  |  |  |  |  |  |  |  |  |  |  |  |  |  |  |  |  |  |  |  |  |  |  |  |  |  |  |  |  |  |  |  |  |  |  |  |  |  |  |  |  |  |  |  |  |  |  |  |  |  |  |  |  |  |  |  |  |  |  |  |  |  |  |  |  |  |  |  |  |  |  |  |  |  |  |  |  |  |  |  |  |  |  |  |  |  |  |  |  |  |  |  |  |  |  |  |  |  |  |  |  |  |  |  |  |  |  |  |  |  |  |  |  |  |  |  |  |  |  |  |  |  |  |  |  |  |  |  |  |  |  |  |  |  |  |  |  |  |  |  |  |  |  |  |  |  |  |  |  |  |  |  |  |  |  |  |  |  |  |  |  |  |  |  |  |  |  |  |  |  |  |  |  |  |  |  |  |  |  |  |  |  |  |  |  |  |  |  |  |  |  |  |  |  |  |  |  |  |  |  |  |  |  |  |  |  |  |  |  |  |  |  |  |  |  |  |  |  |  |  |  |  |  |  |  |  |  |  |  |  |  |  |  |  |  |  |  |  |  |  |  |  |  |  |  |  |  |  |  |  |  |  |  |  |  |  |  |  |  |  |  |  |  |  |  |  |  |  |  |  |  |  |  |  |  |  |  |  |  |  |  |  |  |  |  |  |  |  |  |  |  |  |  |  |  |  |  |  |  |  |  |  |  |  |  |  |  |  |  |  |  |  |  |  |  |  |  |  |  |  |  |  |  |  |  |  |  |  |  |  |  |  |  |  |  |  |  |  |  |  |  |  |  |  |  |  |  |  |  |  |  |  |  |  |  |  |  |  |  |  |  |  |  |  |  |  |  |  |  |  |  |  |  |  |  |  |  |  |  |  |  |  |  |  |  |  |  |  |  |  |  |  |  |  |  |  |  |  |  |  |  |  |  |  |  |  |  |  |  |  |  |  |  |  |  |  |  |  |  |  |  |  |  |  |  |  |  |  |  |  |  |  |  |  |  |  |  |  |  |  |  |  |  |  |  |  |  |  |  |  |  |  |  |  |  |  |  |  |  |  |  |  |  |  |  |  |  |  |  |  |  |  |  |  |  |  |  |  |  |  |  |  |  |  |  |  |  |  |  |  |  |  |  |  |  |  |  |  |  |  |  |  |  |  |  |  |  |  |  |  |  |  |  |  |  |  |  |  |  |  |  |  |  |  |  |  |  |  |  |  |  |  |  |  |  |  |  |  |  |  |  |  |  |  |  |  |  |  |  |  |  |  |  |  |  |  |  |  |  |  |  |  |  |  |  |  |  |  |  |  |  |  |  |  |  |  |  |  |  |  |  |  |  |  |  |  |  |  |  |  |  |  |  |  |  |  |  |  |  |  |  |  |  |  |  |  |  |  |  |  |  |  |  |  |  |  |  |  |  |  |  |  |  |  |  |  |  |  |  |  |  |  |  |  |  |  |  |  |  |  |  |  |  |  |  |  |  |  |  |  |  |  |  |  |  |  |  |  |  |  |  |  |  |  |  |  |  |  |  |  |  |  |  |  |  |  |  |  |  |  |  |  |  |  |  |  |  |  |  |  |  |  |  |  |  |  |  |  |  |  |  |  |  |  |  |  |  |  |  |  |  |  |  |  |  |  |  |  |  |  |  |  |  |  |  |  |  |  |  |  |  |  |  |  |  |  |  |  |  |  |  |  |  |  |  |  |  |  |  |  |  |  |  |  |  |  |  |  |  |  |  |  |  |  |  |  |  |  |  |  |  |  |  |  |  |  |  |  |  |  |  |  |  |  |  |  |  |  |  |  |  |  |  |  |  |  |  |  |  |  |  |  |  |  |  |  |  |  |  |  |  |  |  |  |  |  |  |  |  |  |  |  |  |  |  |  |  |  |  |  |  |  |  |  |  |  |  |  |  |  |  |  |  |  |  |  |  |  |  |  |  |  |  |  |  |  |  |  |  |  |  |  |  |  |  |  |  |  |  |  |  |  |  |  |  |  |  |  |  |  |  |  |  |  |  |  |  |  |  |  |  |  |  |  |  |  |  |  |  |  |  |  |  |  |  |  |  |  |  |  |  |  |  |  |  |  |  |  |  |  |  |  |  |  |  |  |  |  |  |  |  |  |  |  |  |  |  |  |  |  |  |  |  |  |  |  |  |  |  |  |  |  |  |  |  |  |  |  |  |  |  |  |  |  |  |  |  |  |  |  |  |  |  |  |  |  |  |  |  |  |  |  |  |  |  |  |  |  |  |  |  |  |  |  |  |  |  |  |  |  |  |  |  |  |  |  |  |  |  |  |  |  |  |  |  |  |  |  |  |  |  |  |  |  |  |  |  |  |  |  |  |  |  |  |  |  |  |  |  |  |  |  |  |  |  |  |  |  |  |  |  |  |  |  |  |  |  |  |  |  |  |  |  |  |  |  |  |  |  |  |  |  |  |  |  |  |  |  |  |  |  |  |  |  |  |  |  |  |  |  |  |  |  |  |  |  |  |  |  |  |  |  |  |  |  |  |  |  |  |  |  |  |  |  |  |  |  |  |  |  |  |  |  |  |  |  |  |  |  |  |  |  |  |  |  |  |  |  |  |  |  |  |  |  |  |  |  |  |  |  |  |  |  |  |  |  |  |  |  |  |  |  |  |  |  |  |  |  |  |  |  |  |  |  |  |  |  |  |  |  |  |  |  |  |  |  |  |  |  |  |  |  |  |  |  |  |  |  |  |  |  |  |  |  |  |  |  |  |  |  |  |  |  |  |  |  |  |  |  |  |  |  |  |  |  |  |  |  |  |  |  |  |  |  |  |  |  |  |  |  |  |  |  |  |  |  |  |  |  |  |  |  |  |  |  |  |  |  |  |  |  |  |  |  |  |  |  |  |  |  |  |  |  |  |  |  |  |  |  |  |  |  |  |  |  |  |  |  |  |  |  |  |  |  |  |  |  |  |  |  |  |  |  |  |  |  |  |  |  |  |  |  |  |  |  |  |  |  |  |  |  |  |  |  |  |  |  |  |  |  |  |  |  |  |  |  |  |  |  |  |  |  |  |  |  |  |  |  |  |  |  |  |  |  |  |  |  |  |  |  |  |  |  |  |  |  |  |  |  |  |  |  |  |  |  |  |  |  |  |  |  |  |  |  |  |  |  |  |  |  |  |  |  |  |  |  |  |  |  |  |  |  |  |  |  |  |  |  |  |  |  |  |  |  |  |  |  |  |  |  |  |  |  |  |  |  |  |  |  |  |  |  |  |  |  |  |  |  |  |  |  |  |  |  |  |  |  |  |  |  |  |  |  |  |  |  |  |  |  |  |  |  |  |  |  |  |  |  |  |  |  |  |  |  |  |  |  |  |  |  |  |  |  |  |  |  |  |  |  |  |  |  |  |  |  |  |  |  |  |  |  |  |  |  |  |  |  |  |  |  |  |  |  |  |  |  |  |  |  |  |  |  |  |  |  |  |  |  |  |  |  |  |  |  |  |  |  |  |  |  |  |  |  |  |  |  |  |  |  |  |  |  |  |  |  |  |  |  |  |  |  |  |  |  |  |  |  |  |  |  |  |  |  |  |  |  |  |  |  |  |  |  |  |  |  |  |  |  |  |  |  |  |  |  |  |  |  |  |  |  |  |  |  |  |  |  |  |  |  |  |  |  |  |  |  |  |  |  |  |  |  |  |  |  |  |  |  |  |  |  |  |  |  |  |  |  |  |  |  |  |  |  |  |  |  |  |  |  |  |  |  |  |  |  |  |  |  |  |  |  |  |  |  |  |  |  |  |  |  |  |  |  |  |  |  |  |  |  |  |  |  |  |  |  |  |  |  |  |  |  |  |  |  |  |  |  |  |  |  |  |  |  |  |  |  |  |  |  |  |  |  |  |  |  |  |  |  |  |  |  |  |  |  |  |  |  |  |  |  |  |  |  |  |  |  |  |  |  |  |  |  |  |  |  |  |  |  |  |  |  |  |  |  |  |  |  |  |  |  |  |  |  |  |  |  |  |  |  |  |  |  |  |  |  |  |  |  |  |  |  |  |  |  |  |  |  |  |  |  |  |  |  |  |  |  |  |  |  |  |  |  |  |  |  |  |  |  |  |  |  |  |  |  |  |  |  |  |  |  |  |  |  |  |  |  |  |  |  |  |  |  |  |  |  |  |  |  |  |  |  |  |  |  |  |  |  |  |  |  |  |  |  |  |  |  |  |  |  |  |  |  |  |  |  |  |  |  |  |  |  |  |  |  |  |  |  |  |  |  |  |  |  |  |  |  |  |  |  |  |  |  |  |  |  |  |  |  |  |  |  |  |  |  |  |  |  |  |  |  |  |  |  |  |  |  |  |  |  |  |  |  |  |  |  |  |  |  |  |  |  |  |  |  |  |  |  |  |  |  |  |  |  |  |  |  |  |  |  |  |  |  |  |  |  |  |  |  |  |  |  |  |  |  |  |  |  |  |  |  |  |  |  |  |  |  |  |  |  |  |  |  |  |  |  |  |  |  |  |  |  |  |  |  |  |  |  |  |  |  |  |  |  |  |  |  |  |  |  |  |  |  |  |  |  |  |  |  |  |  |  |  |  |  |  |  |  |  |  |  |  |  |  |  |  |  |  |  |  |  |  |  |  |  |  |  |  |  |  |  |  |  |  |  |  |  |  |  |  |  |  |  |  |  |  |  |  |  |  |  |  |  |  |  |  |  |  |  |  |  |  |  |  |  |  |  |  |  |  |  |  |  |  |  |  |  |  |  |  |  |  |  |  |  |  |  |  |  |  |  |  |  |  |  |  |  |  |  |  |  |  |  |  |  |  |  |  |  |  |  |  |  |  |  |  |  |  |  |  |  |  |  |  |  |  |  |  |  |  |  |  |  |  |  |  |  |  |  |  |  |  |  |  |  |  |  |  |  |  |  |  |  |  |  |  |  |  |  |  |  |  |  |  |  |  |  |  |  |  |  |  |  |  |  |  |  |  |  |  |  |  |  |  |  |  |  |  |  |  |  |  |  |  |  |  |  |  |  |  |  |  |  |  |  |  |  |  |  |  |  |  |  |  |  |  |  |  |  |  |  |  |  |  |  |  |  |  |  |  |  |  |  |  |  |  |  |  |  |  |  |  |  |  |  |  |  |  |  |  |  |  |  |  |  |  |  |  |  |  |  |  |  |  |  |  |  |  |  |  |  |  |  |  |  |  |  |  |  |  |  |  |  |  |  |  |  |  |  |  |  |  |  |  |  |  |  |  |  |  |  |  |  |  |  |  |  |  |  |  |  |  |  |  |  |  |  |  |  |  |  |  |  |  |  |  |  |  |  |  |  |  |  |  |  |  |  |  |  |  |  |  |  |  |  |  |  |  |  |  |  |  |  |  |  |  |  |  |  |  |  |  |  |  |  |  |  |  |  |  |  |  |  |  |  |  |  |  |  |  |  |  |  |  |  |  |  |  |  |  |  |  |  |  |  |  |  |  |  |  |  |  |  |  |  |  |  |  |  |  |  |  |  |  |  |  |  |  |  |  |  |  |  |  |  |  |  |  |  |  |  |  |  |  |  |  |  |  |  |  |  |  |  |  |  |  |  |  |  |  |  |  |  |  |  |  |  |  |  |  |  |  |  |  |  |  |  |  |  |  |  |  |  |  |  |  |  |  |  |  |  |  |  |  |  |  |  |  |  |  |  |  |  |  |  |  |  |  |  |  |  |  |  |  |  |  |  |  |  |  |  |  |  |  |  |  |  |  |  |  |  |  |  |  |  |  |  |  |  |  |  |  |  |  |  |  |  |  |  |  |  |  |  |  |  |  |  |  |  |  |  |  |  |  |  |  |  |  |  |  |  |  |  |  |  |  |  |  |  |  |  |  |  |  |  |  |  |  |  |  |  |  |  |  |  |  |  |  |  |  |  |  |  |  |  |  |  |  |  |  |  |  |  |  |  |  |  |  |  |  |  |  |  |  |  |  |  |  |  |  |  |  |  |  |  |  |  |  |  |  |  |  |  |  |  |  |  |  |  |  |  |  |  |  |  |  |  |  |  |  |  |  |  |  |  |  |  |  |  |  |  |  |  |  |  |  |  |  |  |  |  |  |  |  |  |  |  |  |  |  |  |  |  |  |  |  |  |  |  |  |  |  |  |  |  |  |  |  |  |  |  |  |  |  |  |  |  |  |  |  |  |  |  |  |  |  |  |  |  |  |  |  |  |  |  |  |  |  |  |  |  |  |  |  |  |  |  |  |  |  |  |  |  |  |  |  |  |  |  |  |  |  |  |  |  |  |  |  |  |  |  |  |  |  |  |  |  |  |  |  |  |  |  |  |  |  |  |  |  |  |  |  |  |  |  |  |  |  |  |  |  |  |  |  |  |  |  |  |  |  |  |  |  |  |  |  |  |  |  |  |  |  |  |  |  |  |  |  |  |  |  |  |  |  |  |  |  |  |  |  |  |  |  |  |  |  |  |  |  |  |  |  |  |  |  |  |  |  |  |  |  |  |  |  |  |  |  |  |  |  |  |  |  |  |  |  |  |  |  |  |  |  |  |  |  |  |  |  |  |  |  |  |  |  |  |  |  |  |  |  |  |  |  |  |  |  |  |  |  |  |  |  |  |  |  |  |  |  |  |  |  |  |  |  |  |  |  |  |  |  |  |  |  |  |  |  |  |  |  |  |  |  |  |  |  |  |  |  |  |  |  |  |  |  |  |  |  |  |  |  |  |  |  |  |  |  |  |  |  |  |  |  |  |  |  |  |  |  |  |  |  |  |  |  |  |  |  |  |  |  |  |  |  |  |  |  |  |  |  |  |  |  |  |  |  |  |  |  |  |  |  |  |  |  |  |  |  |  |  |  |  |  |  |  |  |  |  |  |  |  |  |  |  |  |  |  |  |  |  |  |  |  |  |  |  |  |  |  |  |  |  |  |  |  |  |  |  |  |  |  |  |  |  |  |  |  |  |  |  |  |  |  |  |  |  |  |  |  |  |  |  |  |  |  |  |  |  |  |  |  |  |  |  |  |  |  |  |  |  |  |  |  |  |  |  |  |  |  |  |  |  |  |  |  |  |  |  |  |  |  |  |  |  |  |  |  |  |  |  |  |  |  |  |  |  |  |  |  |  |  |  |  |  |  |  |  |  |  |  |  |  |  |  |  |  |  |  |  |  |  |  |  |  |  |  |  |  |  |  |  |  |  |  |  |  |  |  |  |  |  |  |  |  |  |  |  |  |  |  |  |  |  |  |  |  |  |  |  |  |  |  |  |  |  |  |  |  |  |  |  |  |  |  |  |  |  |  |  |  |  |  |  |  |  |  |  |  |  |  |  |  |  |  |  |  |  |  |  |  |  |  |  |  |  |  |  |  |  |  |  |  |  |  |  |  |  |  |  |  |  |  |  |  |  |  |  |  |  |  |  |  |  |  |  |  |  |  |  |  |  |  |  |  |  |  |  |  |  |  |  |  |  |  |  |  |  |  |  |  |  |  |  |  |  |  |  |  |  |  |  |  |  |  |  |  |  |  |  |  |  |  |  |  |  |  |  |  |  |  |  |  |  |  |  |  |  |  |  |  |  |  |  |  |  |  |  |  |  |  |  |  |  |  |  |  |  |  |  |  |  |  |  |  |  |  |  |  |  |  |  |  |  |  |  |  |  |  |  |  |  |  |  |  |  |  |  |  |  |  |  |  |  |  |  |  |  |  |  |  |  |  |  |  |  |  |  |  |  |  |  |  |  |  |  |  |  |  |  |  |  |  |  |  |  |  |  |  |  |  |  |  |  |  |  |  |  |  |  |  |  |  |  |  |  |  |  |  |  |  |  |  |  |  |  |  |  |  |  |  |  |  |  |  |  |  |  |  |  |  |  |  |  |  |  |  |  |  |  |  |  |  |  |  |  |  |  |  |  |  |  |  |  |  |  |  |  |  |  |  |  |  |  |  |  |  |  |  |  |  |  |  |  |  |  |  |  |  |  |  |  |  |  |  |  |  |  |  |  |  |  |  |  |  |  |  |  |  |  |  |  |  |  |  |  |  |  |  |  |  |  |  |  |  |  |  |  |  |  |  |  |  |  |  |  |  |  |  |  |  |  |  |  |  |  |  |  |  |  |  |  |  |  |  |  |  |  |  |  |  |  |  |  |  |  |  |  |  |  |  |  |  |  |  |  |  |  |  |  |  |  |  |  |  |  |  |  |  |  |  |  |  |  |  |  |  |  |  |  |  |  |  |  |  |  |  |  |  |  |  |  |  |  |  |  |  |  |  |  |  |  |  |  |  |  |  |  |  |  |  |  |  |  |  |  |  |  |  |  |  |  |  |  |  |  |  |  |  |  |  |  |  |  |  |  |  |  |  |  |  |  |  |  |  |  |  |  |  |  |  |  |  |  |  |  |  |  |  |  |  |  |  |  |  |  |  |  |  |  |  |  |  |  |  |  |  |  |  |  |  |  |  |  |  |  |  |  |  |  |  |  |  |  |  |  |  |  |  |  |  |  |  |  |  |  |  |  |  |  |  |  |  |  |  |  |  |  |  |  |  |  |  |  |  |  |  |  |  |  |  |  |  |  |  |  |  |  |  |  |  |  |  |  |  |  |  |  |  |  |  |  |  |  |  |  |  |  |  |  |  |  |  |  |  |  |  |  |  |  |  |  |  |  |  |  |  |  |  |  |  |  |  |  |  |  |  |  |  |  |  |  |  |  |  |  |  |  |  |  |  |  |  |  |  |  |  |  |  |  |  |  |  |  |  |  |  |  |  |  |  |  |  |  |  |  |  |  |  |  |  |  |  |  |  |  |  |  |  |  |  |  |  |  |  |  |  |  |  |  |  |  |  |  |  |  |  |  |  |  |  |  |  |  |  |  |  |  |  |  |  |  |  |  |  |  |  |  |  |  |  |  |  |  |  |  |  |  |  |  |  |  |  |  |  |  |  |  |  |  |  |  |  |  |  |  |  |  |  |  |  |  |  |  |  |  |  |  |  |  |  |  |  |  |  |  |  |  |  |  |  |  |  |  |  |  |  |  |  |  |  |  |  |  |  |  |  |  |  |  |  |  |  |  |  |  |  |  |  |  |  |  |  |  |  |  |  |  |  |  |  |  |  |  |  |  |  |  |  |  |  |  |  |  |  |  |  |  |  |  |  |  |  |  |  |  |  |  |  |  |  |  |  |  |  |  |  |  |  |  |  |  |  |  |  |  |  |  |  |  |  |  |  |  |  |  |  |  |  |  |  |  |  |  |  |  |  |  |  |  |  |  |  |  |  |  |  |  |  |  |  |  |  |  |  |  |  |  |  |  |  |  |  |  |  |  |  |  |  |  |  |  |  |  |  |  |  |  |  |  |  |  |  |  |  |  |  |  |  |  |  |  |  |  |  |  |  |  |  |  |  |  |  |  |  |  |  |  |  |  |  |  |  |  |  |  |  |  |  |  |  |  |  |  |  |  |  |  |  |  |  |  |  |  |  |  |  |  |  |  |  |  |  |  |  |  |  |  |  |  |  |  |  |  |  |  |  |  |  |  |  |  |  |  |  |  |  |  |  |  |  |  |  |  |  |  |  |  |  |  |  |  |  |  |  |  |  |  |  |  |  |  |  |  |  |  |  |  |  |  |  |  |  |  |  |  |  |  |  |  |  |  |  |  |  |  |  |  |  |  |  |  |  |  |  |  |  |  |  |  |  |  |  |  |  |  |  |  |  |  |  |  |  |  |  |  |  |  |  |  |  |  |  |  |  |  |  |  |  |  |  |  |  |  |  |  |  |  |  |  |  |  |  |  |  |  |  |  |  |  |  |  |  |  |  |  |  |  |  |  |  |  |  |  |  |  |  |  |  |  |  |  |  |  |  |  |  |  |  |  |  |  |  |  |  |  |  |  |  |  |  |  |  |  |  |  |  |  |  |  |  |  |  |  |  |  |  |  |  |  |  |  |  |  |  |  |  |  |  |  |  |  |  |  |  |  |  |  |  |  |  |  |  |  |  |  |  |  |  |  |  |  |  |  |  |  |  |  |  |  |  |  |  |  |  |  |  |  |  |  |  |  |  |  |  |  |  |  |  |  |  |  |  |  |  |  |  |  |  |  |  |  |  |  |  |  |  |  |  |  |  |  |  |  |  |  |  |  |  |  |  |  |  |  |  |  |  |  |  |  |  |  |  |  |  |  |  |  |  |  |  |  |  |  |  |  |  |  |  |  |  |  |  |  |  |  |  |  |  |  |  |  |  |  |  |  |  |  |  |  |  |  |  |  |  |  |  |  |  |  |  |  |  |  |  |  |  |  |  |  |  |  |  |  |  |  |  |  |  |  |  |  |  |  |  |  |  |  |  |  |  |  |  |  |  |  |  |  |  |  |  |  |  |  |  |  |  |  |  |  |  |  |  |  |  |  |  |  |  |  |  |  |  |  |  |  |  |  |  |  |  |  |  |  |  |  |  |  |  |  |  |  |  |  |  |  |  |  |  |  |  |  |  |  |  |  |  |  |  |  |  |  |  |  |  |  |  |  |  |  |  |  |  |  |  |  |  |  |  |  |  |  |  |  |  |  |  |  |  |  |  |  |  |  |  |  |  |  |  |  |  |  |  |  |  |  |  |  |  |  |  |  |  |  |  |  |  |  |  |  |  |  |  |  |  |  |  |  |  |  |  |  |  |  |  |  |  |  |  |  |  |  |  |  |  |  |  |  |  |  |  |  |  |  |  |  |  |  |  |  |  |  |  |  |  |  |  |  |  |  |  |  |  |  |  |  |  |  |  |  |  |  |  |  |  |  |  |  |  |  |  |  |  |  |  |  |  |  |  |  |  |  |  |  |  |  |  |  |  |  |  |  |  |  |  |  |  |  |  |  |  |  |  |  |  |  |  |  |  |  |  |  |  |  |  |  |  |  |  |  |  |  |  |  |  |  |  |  |  |  |  |  |  |  |  |  |  |  |  |  |  |  |  |  |  |  |  |  |  |  |  |  |  |  |  |  |  |  |  |  |  |  |  |  |  |  |  |  |  |  |  |  |  |  |  |  |  |  |  |  |  |  |  |  |  |  |  |  |  |  |  |  |  |  |  |  |  |  |  |  |  |  |  |  |  |  |  |  |  |  |  |  |  |  |  |  |  |  |  |  |  |  |  |  |  |  |  |  |  |  |  |  |  |  |  |  |  |  |  |  |  |  |  |  |  |  |  |  |  |  |  |  |  |  |  |  |  |  |  |  |  |  |  |  |  |  |  |  |  |  |  |  |  |  |  |  |  |  |  |  |  |  |  |  |  |  |  |  |  |  |  |  |  |  |  |  |  |  |  |  |  |  |  |  |  |  |  |  |  |  |  |  |  |  |  |  |  |  |  |  |  |  |  |  |  |  |  |  |  |  |  |  |  |  |  |  |  |  |  |  |  |  |  |  |  |  |  |  |  |  |  |  |  |  |  |  |  |  |  |  |  |  |  |  |  |  |  |  |  |  |  |  |  |  |  |  |  |  |  |  |  |  |  |  |  |  |  |  |  |  |  |  |  |  |  |  |  |  |  |  |  |  |  |  |  |  |  |  |  |  |  |  |  |  |  |  |  |
| --- | --- | --- | --- | --- | --- | --- | --- | --- | --- | --- | --- | --- | --- | --- | --- | --- | --- | --- | --- | --- | --- | --- | --- | --- | --- | --- | --- | --- | --- | --- | --- | --- | --- | --- | --- | --- | --- | --- | --- | --- | --- | --- | --- | --- | --- | --- | --- | --- | --- | --- | --- | --- | --- | --- | --- | --- | --- | --- | --- | --- | --- | --- | --- | --- | --- | --- | --- | --- | --- | --- | --- | --- | --- | --- | --- | --- | --- | --- | --- | --- | --- | --- | --- | --- | --- | --- | --- | --- | --- | --- | --- | --- | --- | --- | --- | --- | --- | --- | --- | --- | --- | --- | --- | --- | --- | --- | --- | --- | --- | --- | --- | --- | --- | --- | --- | --- | --- | --- | --- | --- | --- | --- | --- | --- | --- | --- | --- | --- | --- | --- | --- | --- | --- | --- | --- | --- | --- | --- | --- | --- | --- | --- | --- | --- | --- | --- | --- | --- | --- | --- | --- | --- | --- | --- | --- | --- | --- | --- | --- | --- | --- | --- | --- | --- | --- | --- | --- | --- | --- | --- | --- | --- | --- | --- | --- | --- | --- | --- | --- | --- | --- | --- | --- | --- | --- | --- | --- | --- | --- | --- | --- | --- | --- | --- | --- | --- | --- | --- | --- | --- | --- | --- | --- | --- | --- | --- | --- | --- | --- | --- | --- | --- | --- | --- | --- | --- | --- | --- | --- | --- | --- | --- | --- | --- | --- | --- | --- | --- | --- | --- | --- | --- | --- | --- | --- | --- | --- | --- | --- | --- | --- | --- | --- | --- | --- | --- | --- | --- | --- | --- | --- | --- | --- | --- | --- | --- | --- | --- | --- | --- | --- | --- | --- | --- | --- | --- | --- | --- | --- | --- | --- | --- | --- | --- | --- | --- | --- | --- | --- | --- | --- | --- | --- | --- | --- | --- | --- | --- | --- | --- | --- | --- | --- | --- | --- | --- | --- | --- | --- | --- | --- | --- | --- | --- | --- | --- | --- | --- | --- | --- | --- | --- | --- | --- | --- | --- | --- | --- | --- | --- | --- | --- | --- | --- | --- | --- | --- | --- | --- | --- | --- | --- | --- | --- | --- | --- | --- | --- | --- | --- | --- | --- | --- | --- | --- | --- | --- | --- | --- | --- | --- | --- | --- | --- | --- | --- | --- | --- | --- | --- | --- | --- | --- | --- | --- | --- | --- | --- | --- | --- | --- | --- | --- | --- | --- | --- | --- | --- | --- | --- | --- | --- | --- | --- | --- | --- | --- | --- | --- | --- | --- | --- | --- | --- | --- | --- | --- | --- | --- | --- | --- | --- | --- | --- | --- | --- | --- | --- | --- | --- | --- | --- | --- | --- | --- | --- | --- | --- | --- | --- | --- | --- | --- | --- | --- | --- | --- | --- | --- | --- | --- | --- | --- | --- | --- | --- | --- | --- | --- | --- | --- | --- | --- | --- | --- | --- | --- | --- | --- | --- | --- | --- | --- | --- | --- | --- | --- | --- | --- | --- | --- | --- | --- | --- | --- | --- | --- | --- | --- | --- | --- | --- | --- | --- | --- | --- | --- | --- | --- | --- | --- | --- | --- | --- | --- | --- | --- | --- | --- | --- | --- | --- | --- | --- | --- | --- | --- | --- | --- | --- | --- | --- | --- | --- | --- | --- | --- | --- | --- | --- | --- | --- | --- | --- | --- | --- | --- | --- | --- | --- | --- | --- | --- | --- | --- | --- | --- | --- | --- | --- | --- | --- | --- | --- | --- | --- | --- | --- | --- | --- | --- | --- | --- | --- | --- | --- | --- | --- | --- | --- | --- | --- | --- | --- | --- | --- | --- | --- | --- | --- | --- | --- | --- | --- | --- | --- | --- | --- | --- | --- | --- | --- | --- | --- | --- | --- | --- | --- | --- | --- | --- | --- | --- | --- | --- | --- | --- | --- | --- | --- | --- | --- | --- | --- | --- | --- | --- | --- | --- | --- | --- | --- | --- | --- | --- | --- | --- | --- | --- | --- | --- | --- | --- | --- | --- | --- | --- | --- | --- | --- | --- | --- | --- | --- | --- | --- | --- | --- | --- | --- | --- | --- | --- | --- | --- | --- | --- | --- | --- | --- | --- | --- | --- | --- | --- | --- | --- | --- | --- | --- | --- | --- | --- | --- | --- | --- | --- | --- | --- | --- | --- | --- | --- | --- | --- | --- | --- | --- | --- | --- | --- | --- | --- | --- | --- | --- | --- | --- | --- | --- | --- | --- | --- | --- | --- | --- | --- | --- | --- | --- | --- | --- | --- | --- | --- | --- | --- | --- | --- | --- | --- | --- | --- | --- | --- | --- | --- | --- | --- | --- | --- | --- | --- | --- | --- | --- | --- | --- | --- | --- | --- | --- | --- | --- | --- | --- | --- | --- | --- | --- | --- | --- | --- | --- | --- | --- | --- | --- | --- | --- | --- | --- | --- | --- | --- | --- | --- | --- | --- | --- | --- | --- | --- | --- | --- | --- | --- | --- | --- | --- | --- | --- | --- | --- | --- | --- | --- | --- | --- | --- | --- | --- | --- | --- | --- | --- | --- | --- | --- | --- | --- | --- | --- | --- | --- | --- | --- | --- | --- | --- | --- | --- | --- | --- | --- | --- | --- | --- | --- | --- | --- | --- | --- | --- | --- | --- | --- | --- | --- | --- | --- | --- | --- | --- | --- | --- | --- | --- | --- | --- | --- | --- | --- | --- | --- | --- | --- | --- | --- | --- | --- | --- | --- | --- | --- | --- | --- | --- | --- | --- | --- | --- | --- | --- | --- | --- | --- | --- | --- | --- | --- | --- | --- | --- | --- | --- | --- | --- | --- | --- | --- | --- | --- | --- | --- | --- | --- | --- | --- | --- | --- | --- | --- | --- | --- | --- | --- | --- | --- | --- | --- | --- | --- | --- | --- | --- | --- | --- | --- | --- | --- | --- | --- | --- | --- | --- | --- | --- | --- | --- | --- | --- | --- | --- | --- | --- | --- | --- | --- | --- | --- | --- | --- | --- | --- | --- | --- | --- | --- | --- | --- | --- | --- | --- | --- | --- | --- | --- | --- | --- | --- | --- | --- | --- | --- | --- | --- | --- | --- | --- | --- | --- | --- | --- | --- | --- | --- | --- | --- | --- | --- | --- | --- | --- | --- | --- | --- | --- | --- | --- | --- | --- | --- | --- | --- | --- | --- | --- | --- | --- | --- | --- | --- | --- | --- | --- | --- | --- | --- | --- | --- | --- | --- | --- | --- | --- | --- | --- | --- | --- | --- | --- | --- | --- | --- | --- | --- | --- | --- | --- | --- | --- | --- | --- | --- | --- | --- | --- | --- | --- | --- | --- | --- | --- | --- | --- | --- | --- | --- | --- | --- | --- | --- | --- | --- | --- | --- | --- | --- | --- | --- | --- | --- | --- | --- | --- | --- | --- | --- | --- | --- | --- | --- | --- | --- | --- | --- | --- | --- | --- | --- | --- | --- | --- | --- | --- | --- | --- | --- | --- | --- | --- | --- | --- | --- | --- | --- | --- | --- | --- | --- | --- | --- | --- | --- | --- | --- | --- | --- | --- | --- | --- | --- | --- | --- | --- | --- | --- | --- | --- | --- | --- | --- | --- | --- | --- | --- | --- | --- | --- | --- | --- | --- | --- | --- | --- | --- | --- | --- | --- | --- | --- | --- | --- | --- | --- | --- | --- | --- | --- | --- | --- | --- | --- | --- | --- | --- | --- | --- | --- | --- | --- | --- | --- | --- | --- | --- | --- | --- | --- | --- | --- | --- | --- | --- | --- | --- | --- | --- | --- | --- | --- | --- | --- | --- | --- | --- | --- | --- | --- | --- | --- | --- | --- | --- | --- | --- | --- | --- | --- | --- | --- | --- | --- | --- | --- | --- | --- | --- | --- | --- | --- | --- | --- | --- | --- | --- | --- | --- | --- | --- | --- | --- | --- | --- | --- | --- | --- | --- | --- | --- | --- | --- | --- | --- | --- | --- | --- | --- | --- | --- | --- | --- | --- | --- | --- | --- | --- | --- | --- | --- | --- | --- | --- | --- | --- | --- | --- | --- | --- | --- | --- | --- | --- | --- | --- | --- | --- | --- | --- | --- | --- | --- | --- | --- | --- | --- | --- | --- | --- | --- | --- | --- | --- | --- | --- | --- | --- | --- | --- | --- | --- | --- | --- | --- | --- | --- | --- | --- | --- | --- | --- | --- | --- | --- | --- | --- | --- | --- | --- | --- | --- | --- | --- | --- | --- | --- | --- | --- | --- | --- | --- | --- | --- | --- | --- | --- | --- | --- | --- | --- | --- | --- | --- | --- | --- | --- | --- | --- | --- | --- | --- | --- | --- | --- | --- | --- | --- | --- | --- | --- | --- | --- | --- | --- | --- | --- | --- | --- | --- | --- | --- | --- | --- | --- | --- | --- | --- | --- | --- | --- | --- | --- | --- | --- | --- | --- | --- | --- | --- | --- | --- | --- | --- | --- | --- | --- | --- | --- | --- | --- | --- | --- | --- | --- | --- | --- | --- | --- | --- | --- | --- | --- | --- | --- | --- | --- | --- | --- | --- | --- | --- | --- | --- | --- | --- | --- | --- | --- | --- | --- | --- | --- | --- | --- | --- | --- | --- | --- | --- | --- | --- | --- | --- | --- | --- | --- | --- | --- | --- | --- | --- | --- | --- | --- | --- | --- | --- | --- | --- | --- | --- | --- | --- | --- | --- | --- | --- | --- | --- | --- | --- | --- | --- | --- | --- | --- | --- | --- | --- | --- | --- | --- | --- | --- | --- | --- | --- | --- | --- | --- | --- | --- | --- | --- | --- | --- | --- | --- | --- | --- | --- | --- | --- | --- | --- | --- | --- | --- | --- | --- | --- | --- | --- | --- | --- | --- | --- | --- | --- | --- | --- | --- | --- | --- | --- | --- | --- | --- | --- | --- | --- | --- | --- | --- | --- | --- | --- | --- | --- | --- | --- | --- | --- | --- | --- | --- | --- | --- | --- | --- | --- | --- | --- | --- | --- | --- | --- | --- | --- | --- | --- | --- | --- | --- | --- | --- | --- | --- | --- | --- | --- | --- | --- | --- | --- | --- | --- | --- | --- | --- | --- | --- | --- | --- | --- | --- | --- | --- | --- | --- | --- | --- | --- | --- | --- | --- | --- | --- | --- | --- | --- | --- | --- | --- | --- | --- | --- | --- | --- | --- | --- | --- | --- | --- | --- | --- | --- | --- | --- | --- | --- | --- | --- | --- | --- | --- | --- | --- | --- | --- | --- | --- | --- | --- | --- | --- | --- | --- | --- | --- | --- | --- | --- | --- | --- | --- | --- | --- | --- | --- | --- | --- | --- | --- | --- | --- | --- | --- | --- | --- | --- | --- | --- | --- | --- | --- | --- | --- | --- | --- | --- | --- | --- | --- | --- | --- | --- | --- | --- | --- | --- | --- | --- | --- | --- | --- | --- | --- | --- | --- | --- | --- | --- | --- | --- | --- | --- | --- | --- | --- | --- | --- | --- | --- | --- | --- | --- | --- | --- | --- | --- | --- | --- | --- | --- | --- | --- | --- | --- | --- | --- | --- | --- | --- | --- | --- | --- | --- | --- | --- | --- | --- | --- | --- | --- | --- | --- | --- | --- | --- | --- | --- | --- | --- | --- | --- | --- | --- | --- | --- | --- | --- | --- | --- | --- | --- | --- | --- | --- | --- | --- | --- | --- | --- | --- | --- | --- | --- | --- | --- | --- | --- | --- | --- | --- | --- | --- | --- | --- | --- | --- | --- | --- | --- | --- | --- | --- | --- | --- | --- | --- | --- | --- | --- | --- | --- | --- | --- | --- | --- | --- | --- | --- | --- | --- | --- | --- | --- | --- | --- | --- | --- | --- | --- | --- | --- | --- | --- | --- | --- | --- | --- | --- | --- | --- | --- | --- | --- | --- | --- | --- | --- | --- | --- | --- | --- | --- | --- | --- | --- | --- | --- | --- | --- | --- | --- | --- | --- | --- | --- | --- | --- | --- | --- | --- | --- | --- | --- | --- | --- | --- | --- | --- | --- | --- | --- | --- | --- | --- | --- | --- | --- | --- | --- | --- | --- | --- | --- | --- | --- | --- | --- | --- | --- | --- | --- | --- | --- | --- | --- | --- | --- | --- | --- | --- | --- | --- | --- | --- | --- | --- | --- | --- | --- | --- | --- | --- | --- | --- | --- | --- | --- | --- | --- | --- | --- | --- | --- | --- | --- | --- | --- | --- | --- | --- | --- | --- | --- | --- | --- | --- | --- | --- | --- | --- | --- | --- | --- | --- | --- | --- | --- | --- | --- | --- | --- | --- | --- | --- | --- | --- | --- | --- | --- | --- | --- | --- | --- | --- | --- | --- | --- | --- | --- | --- | --- | --- | --- | --- | --- | --- | --- | --- | --- | --- | --- | --- | --- | --- | --- | --- | --- | --- | --- | --- | --- | --- | --- | --- | --- | --- | --- | --- | --- | --- | --- | --- | --- | --- | --- | --- | --- | --- | --- | --- | --- | --- | --- | --- | --- | --- | --- | --- | --- | --- | --- | --- | --- | --- | --- | --- | --- | --- | --- | --- | --- | --- | --- | --- | --- | --- | --- | --- | --- | --- | --- | --- | --- | --- | --- | --- | --- | --- | --- | --- | --- | --- | --- | --- | --- | --- | --- | --- | --- | --- | --- | --- | --- | --- | --- | --- | --- | --- | --- | --- | --- | --- | --- | --- | --- | --- | --- | --- | --- | --- | --- | --- | --- | --- | --- | --- | --- | --- | --- | --- | --- | --- | --- | --- | --- | --- | --- | --- | --- | --- | --- | --- | --- | --- | --- | --- | --- | --- | --- | --- | --- | --- | --- | --- | --- | --- | --- | --- | --- | --- | --- | --- | --- | --- | --- | --- | --- | --- | --- | --- | --- | --- | --- | --- | --- | --- | --- | --- | --- | --- | --- | --- | --- | --- | --- | --- | --- | --- | --- | --- | --- | --- | --- | --- | --- | --- | --- | --- | --- | --- | --- | --- | --- | --- | --- | --- | --- | --- | --- | --- | --- | --- | --- | --- | --- | --- | --- | --- | --- | --- | --- | --- | --- | --- | --- | --- | --- | --- | --- | --- | --- | --- | --- | --- | --- | --- | --- | --- | --- | --- | --- | --- | --- | --- | --- | --- | --- | --- | --- | --- | --- | --- | --- | --- | --- | --- | --- | --- | --- | --- | --- | --- | --- | --- | --- | --- | --- | --- | --- | --- | --- | --- | --- | --- | --- | --- | --- | --- | --- | --- | --- | --- | --- | --- | --- | --- | --- | --- | --- | --- | --- | --- | --- | --- | --- | --- | --- | --- | --- | --- | --- | --- | --- | --- | --- | --- | --- | --- | --- | --- | --- | --- | --- | --- | --- | --- | --- | --- | --- | --- | --- | --- | --- | --- | --- | --- | --- | --- | --- | --- | --- | --- | --- | --- | --- | --- | --- | --- | --- | --- | --- | --- | --- | --- | --- | --- | --- | --- | --- | --- | --- | --- | --- | --- | --- | --- | --- | --- | --- | --- | --- | --- | --- | --- | --- | --- | --- | --- | --- | --- | --- | --- | --- | --- | --- | --- | --- | --- | --- | --- | --- | --- | --- | --- | --- | --- | --- | --- | --- | --- | --- | --- | --- | --- | --- | --- | --- | --- | --- | --- | --- | --- | --- | --- | --- | --- | --- | --- | --- | --- | --- | --- | --- | --- | --- | --- | --- | --- | --- | --- | --- | --- | --- | --- | --- | --- | --- | --- | --- | --- | --- | --- | --- | --- | --- | --- | --- | --- | --- | --- | --- | --- | --- | --- | --- | --- | --- | --- | --- | --- | --- | --- | --- | --- | --- | --- | --- | --- | --- | --- | --- | --- | --- | --- | --- | --- | --- | --- | --- | --- | --- | --- | --- | --- | --- | --- | --- | --- | --- | --- | --- | --- | --- | --- | --- | --- | --- | --- | --- | --- | --- | --- | --- | --- | --- | --- | --- | --- | --- | --- | --- | --- | --- | --- | --- | --- | --- | --- | --- | --- | --- | --- | --- | --- | --- | --- | --- | --- | --- | --- | --- | --- | --- | --- | --- | --- | --- | --- | --- | --- | --- | --- | --- | --- | --- | --- | --- | --- | --- | --- | --- | --- | --- | --- | --- | --- | --- | --- | --- | --- | --- | --- | --- | --- | --- | --- | --- | --- | --- | --- | --- | --- | --- | --- | --- | --- | --- | --- | --- | --- | --- | --- | --- | --- | --- | --- | --- | --- | --- | --- | --- | --- | --- | --- | --- | --- | --- | --- | --- | --- | --- | --- | --- | --- | --- | --- | --- | --- | --- | --- | --- | --- | --- | --- | --- | --- | --- | --- | --- | --- | --- | --- | --- | --- | --- | --- | --- | --- | --- | --- | --- | --- | --- | --- | --- | --- | --- | --- | --- | --- | --- | --- | --- | --- | --- | --- | --- | --- | --- | --- | --- | --- | --- | --- | --- | --- | --- | --- | --- | --- | --- | --- | --- | --- | --- | --- | --- | --- | --- | --- | --- | --- | --- | --- | --- | --- | --- | --- | --- | --- | --- | --- | --- | --- | --- | --- | --- | --- | --- | --- | --- | --- | --- | --- | --- | --- | --- | --- | --- | --- | --- | --- | --- | --- | --- | --- | --- | --- | --- | --- | --- | --- | --- | --- | --- | --- | --- | --- | --- | --- | --- | --- | --- | --- | --- | --- | --- | --- | --- | --- | --- | --- | --- | --- | --- | --- | --- | --- | --- | --- | --- | --- | --- | --- | --- | --- | --- | --- | --- | --- | --- | --- | --- | --- | --- | --- | --- | --- | --- | --- | --- | --- | --- | --- | --- | --- | --- | --- | --- | --- | --- | --- | --- | --- | --- | --- | --- | --- | --- | --- | --- | --- | --- | --- | --- | --- | --- | --- | --- | --- | --- | --- | --- | --- | --- | --- | --- | --- | --- | --- | --- | --- | --- | --- | --- | --- | --- | --- | --- | --- | --- | --- | --- | --- | --- | --- | --- | --- | --- | --- | --- | --- | --- | --- | --- | --- | --- | --- | --- | --- | --- | --- | --- | --- | --- | --- | --- | --- | --- | --- | --- | --- | --- | --- | --- | --- | --- | --- | --- | --- | --- | --- | --- | --- | --- | --- | --- | --- | --- | --- | --- | --- | --- | --- | --- | --- | --- | --- | --- | --- | --- | --- | --- | --- | --- | --- | --- | --- | --- | --- | --- | --- | --- | --- | --- | --- | --- | --- | --- | --- | --- | --- | --- | --- | --- | --- | --- | --- | --- | --- | --- | --- | --- | --- | --- | --- | --- | --- | --- | --- | --- | --- | --- | --- | --- | --- | --- | --- | --- | --- | --- | --- | --- | --- | --- | --- | --- | --- | --- | --- | --- | --- | --- | --- | --- | --- | --- | --- | --- | --- | --- | --- | --- | --- | --- | --- | --- | --- | --- | --- | --- | --- | --- | --- | --- | --- | --- | --- | --- | --- | --- | --- | --- | --- | --- | --- | --- | --- | --- | --- | --- | --- | --- | --- | --- | --- | --- | --- | --- | --- | --- | --- | --- | --- | --- | --- | --- | --- | --- | --- | --- | --- | --- | --- | --- | --- | --- | --- | --- | --- | --- | --- | --- | --- | --- | --- | --- | --- | --- | --- | --- | --- | --- | --- | --- | --- | --- | --- | --- | --- | --- | --- | --- | --- | --- | --- | --- | --- | --- | --- | --- | --- | --- | --- | --- | --- | --- | --- | --- | --- | --- | --- | --- | --- | --- | --- | --- | --- | --- | --- | --- | --- | --- | --- | --- | --- | --- | --- | --- | --- | --- | --- | --- | --- | --- | --- | --- | --- | --- | --- | --- | --- | --- | --- | --- | --- | --- | --- | --- | --- | --- | --- | --- | --- | --- | --- | --- | --- | --- | --- | --- | --- | --- | --- | --- | --- | --- | --- | --- | --- | --- | --- | --- | --- | --- | --- | --- | --- | --- | --- | --- | --- | --- | --- | --- | --- | --- | --- | --- | --- | --- | --- | --- | --- | --- | --- | --- | --- | --- | --- | --- | --- | --- | --- | --- | --- | --- | --- | --- | --- | --- | --- | --- | --- | --- | --- | --- | --- | --- | --- | --- | --- | --- | --- | --- | --- | --- | --- | --- | --- | --- | --- | --- | --- | --- | --- | --- | --- | --- | --- | --- | --- | --- | --- | --- | --- | --- | --- | --- | --- | --- | --- | --- | --- | --- | --- | --- | --- | --- | --- | --- | --- | --- | --- | --- | --- | --- | --- | --- | --- | --- | --- | --- | --- | --- | --- | --- | --- | --- | --- | --- | --- | --- | --- | --- | --- | --- | --- | --- | --- | --- | --- | --- | --- | --- | --- | --- | --- | --- | --- | --- | --- | --- | --- | --- | --- | --- | --- | --- | --- | --- | --- | --- | --- | --- | --- | --- | --- | --- | --- | --- | --- | --- | --- | --- | --- | --- | --- | --- | --- | --- | --- | --- | --- | --- | --- | --- | --- | --- | --- | --- | --- | --- | --- | --- | --- | --- | --- | --- | --- | --- | --- | --- | --- | --- | --- | --- | --- | --- | --- | --- | --- | --- | --- | --- | --- | --- | --- | --- | --- | --- | --- | --- | --- | --- | --- | --- | --- | --- | --- | --- | --- | --- | --- | --- | --- | --- | --- | --- | --- | --- | --- | --- | --- | --- | --- | --- | --- | --- | --- | --- | --- | --- | --- | --- | --- | --- | --- | --- | --- | --- | --- | --- | --- | --- | --- | --- | --- | --- | --- | --- | --- | --- | --- | --- | --- | --- | --- | --- | --- | --- | --- | --- | --- | --- | --- | --- | --- | --- | --- | --- | --- | --- | --- | --- | --- | --- | --- | --- | --- | --- | --- | --- | --- | --- | --- | --- | --- | --- | --- | --- | --- | --- | --- | --- | --- | --- | --- | --- | --- | --- | --- | --- | --- | --- | --- | --- | --- | --- | --- | --- | --- | --- | --- | --- | --- | --- | --- | --- | --- | --- | --- | --- | --- | --- | --- | --- | --- | --- | --- | --- | --- | --- | --- | --- | --- | --- | --- | --- | --- | --- | --- | --- | --- | --- | --- | --- | --- | --- | --- | --- | --- | --- | --- | --- | --- | --- | --- | --- | --- | --- | --- | --- | --- | --- | --- | --- | --- | --- | --- | --- | --- | --- | --- | --- | --- | --- | --- | --- | --- | --- | --- | --- | --- | --- | --- | --- | --- | --- | --- | --- | --- | --- | --- | --- | --- | --- | --- | --- | --- | --- | --- | --- | --- | --- | --- | --- | --- | --- | --- | --- | --- | --- | --- | --- | --- | --- | --- | --- | --- | --- | --- | --- | --- | --- | --- | --- | --- | --- | --- | --- | --- | --- | --- | --- | --- | --- | --- | --- | --- | --- | --- | --- | --- | --- | --- | --- | --- | --- | --- | --- | --- | --- | --- | --- | --- | --- | --- | --- | --- | --- | --- | --- | --- | --- | --- | --- | --- | --- | --- | --- | --- | --- | --- | --- | --- | --- | --- | --- | --- | --- | --- | --- | --- | --- | --- | --- | --- | --- | --- | --- | --- | --- | --- | --- | --- | --- | --- | --- | --- | --- | --- | --- | --- | --- | --- | --- | --- | --- | --- | --- | --- | --- | --- | --- | --- | --- | --- | --- | --- | --- | --- | --- | --- | --- | --- | --- | --- | --- | --- | --- | --- | --- | --- | --- | --- | --- | --- | --- | --- | --- | --- | --- | --- | --- | --- | --- | --- | --- | --- | --- | --- | --- | --- | --- | --- | --- | --- | --- | --- | --- | --- | --- | --- | --- | --- | --- | --- | --- | --- | --- | --- | --- | --- | --- | --- | --- | --- | --- | --- | --- | --- | --- | --- | --- | --- | --- | --- | --- | --- | --- | --- | --- | --- | --- | --- | --- | --- | --- | --- | --- | --- | --- | --- | --- | --- | --- | --- | --- | --- | --- | --- | --- | --- | --- | --- | --- | --- | --- | --- | --- | --- | --- | --- | --- | --- | --- | --- | --- | --- | --- | --- | --- | --- | --- | --- | --- | --- | --- | --- | --- | --- | --- | --- | --- | --- | --- | --- | --- | --- | --- | --- | --- | --- | --- | --- | --- | --- | --- | --- | --- | --- | --- | --- | --- | --- | --- | --- | --- | --- | --- | --- | --- | --- | --- | --- | --- | --- | --- | --- | --- | --- | --- | --- | --- | --- | --- | --- | --- | --- | --- | --- | --- | --- | --- | --- | --- | --- | --- | --- | --- | --- | --- | --- | --- | --- | --- | --- | --- | --- | --- | --- | --- | --- | --- | --- | --- | --- | --- | --- | --- | --- | --- | --- | --- | --- | --- | --- | --- | --- | --- | --- | --- | --- | --- | --- | --- | --- | --- | --- | --- | --- | --- | --- | --- | --- | --- | --- | --- | --- | --- | --- | --- | --- | --- | --- | --- | --- | --- | --- | --- | --- | --- | --- | --- | --- | --- | --- | --- | --- | --- | --- | --- | --- | --- | --- | --- | --- | --- | --- | --- | --- | --- | --- | --- | --- | --- | --- | --- | --- | --- | --- | --- | --- | --- | --- | --- | --- | --- | --- | --- | --- | --- | --- | --- | --- | --- | --- | --- | --- | --- | --- | --- | --- | --- | --- | --- | --- | --- | --- | --- | --- | --- | --- | --- | --- | --- | --- | --- | --- | --- | --- | --- | --- | --- | --- | --- | --- | --- | --- | --- | --- | --- | --- | --- | --- | --- | --- | --- | --- | --- | --- | --- | --- | --- | --- | --- | --- | --- | --- | --- | --- | --- | --- | --- | --- | --- | --- | --- | --- | --- | --- | --- | --- | --- | --- | --- | --- | --- | --- | --- | --- | --- | --- | --- | --- | --- | --- | --- | --- | --- | --- | --- | --- | --- | --- | --- | --- | --- | --- | --- | --- | --- | --- | --- | --- | --- | --- | --- | --- | --- | --- | --- | --- | --- | --- | --- | --- | --- | --- | --- | --- | --- | --- | --- | --- | --- | --- | --- | --- | --- | --- | --- | --- | --- | --- | --- | --- | --- | --- | --- | --- | --- | --- | --- | --- | --- | --- | --- | --- | --- | --- | --- | --- | --- | --- | --- | --- | --- | --- | --- | --- | --- | --- | --- | --- | --- | --- | --- | --- | --- | --- | --- | --- | --- | --- | --- | --- | --- | --- | --- | --- | --- | --- | --- | --- | --- | --- | --- | --- | --- | --- | --- | --- | --- | --- | --- | --- | --- | --- | --- | --- | --- | --- | --- | --- | --- | --- | --- | --- | --- | --- | --- | --- | --- | --- | --- | --- | --- | --- | --- | --- | --- | --- | --- | --- | --- | --- | --- | --- | --- | --- | --- | --- | --- | --- | --- | --- | --- | --- | --- | --- | --- | --- | --- | --- | --- | --- | --- | --- | --- | --- | --- | --- | --- | --- | --- | --- | --- | --- | --- | --- | --- | --- | --- | --- | --- | --- | --- | --- | --- | --- | --- | --- | --- | --- | --- | --- | --- | --- | --- | --- | --- | --- | --- | --- | --- | --- | --- | --- | --- | --- | --- | --- | --- | --- | --- | --- | --- | --- | --- | --- | --- | --- | --- | --- | --- | --- | --- | --- | --- | --- | --- | --- | --- | --- | --- | --- | --- | --- | --- | --- | --- | --- | --- | --- | --- | --- | --- | --- | --- | --- | --- | --- | --- | --- | --- | --- | --- | --- | --- | --- | --- | --- | --- | --- | --- | --- | --- | --- | --- | --- | --- | --- | --- | --- | --- | --- | --- | --- | --- | --- | --- | --- | --- | --- | --- | --- | --- | --- | --- | --- | --- | --- | --- | --- | --- | --- | --- | --- | --- | --- | --- | --- | --- | --- | --- | --- | --- | --- | --- | --- | --- | --- | --- | --- | --- | --- | --- | --- | --- | --- | --- | --- | --- | --- | --- | --- | --- | --- | --- | --- | --- | --- | --- | --- | --- | --- | --- | --- | --- | --- | --- | --- | --- | --- | --- | --- | --- | --- | --- | --- | --- | --- | --- | --- | --- | --- | --- | --- | --- | --- | --- | --- | --- | --- | --- | --- | --- | --- | --- | --- | --- | --- | --- | --- | --- | --- | --- | --- | --- | --- | --- | --- | --- | --- | --- | --- | --- | --- | --- | --- | --- | --- | --- | --- | --- | --- | --- | --- | --- | --- | --- | --- | --- | --- | --- | --- | --- | --- | --- | --- | --- | --- | --- | --- | --- | --- | --- | --- | --- | --- | --- | --- | --- | --- | --- | --- | --- | --- | --- | --- | --- | --- | --- | --- | --- | --- | --- | --- | --- | --- | --- | --- | --- | --- | --- | --- | --- | --- | --- | --- | --- | --- | --- | --- | --- | --- | --- | --- | --- | --- | --- | --- | --- | --- | --- | --- | --- | --- | --- | --- | --- | --- | --- | --- | --- | --- | --- | --- | --- | --- | --- | --- | --- | --- | --- | --- | --- | --- | --- | --- | --- | --- | --- | --- | --- | --- | --- | --- | --- | --- | --- | --- | --- | --- | --- | --- | --- | --- | --- | --- | --- | --- | --- | --- | --- | --- | --- | --- | --- | --- | --- | --- | --- | --- | --- | --- | --- | --- | --- | --- | --- | --- | --- | --- | --- | --- | --- | --- | --- | --- | --- | --- | --- | --- | --- | --- | --- | --- | --- | --- | --- | --- | --- | --- | --- | --- | --- | --- | --- | --- | --- | --- | --- | --- | --- | --- | --- | --- | --- | --- | --- | --- | --- | --- | --- | --- | --- | --- | --- | --- | --- | --- | --- | --- | --- | --- | --- | --- | --- | --- | --- | --- | --- | --- | --- | --- | --- | --- | --- | --- | --- | --- | --- | --- | --- | --- | --- | --- | --- | --- | --- | --- | --- | --- | --- | --- | --- | --- | --- | --- | --- | --- | --- | --- | --- | --- | --- | --- | --- | --- | --- | --- | --- | --- | --- | --- | --- | --- | --- | --- | --- | --- | --- | --- | --- | --- | --- | --- | --- | --- | --- | --- | --- | --- | --- | --- | --- | --- | --- | --- | --- | --- | --- | --- | --- | --- | --- | --- | --- | --- | --- | --- | --- | --- | --- | --- | --- | --- | --- | --- | --- | --- | --- | --- | --- | --- | --- | --- | --- | --- | --- | --- | --- | --- | --- | --- | --- | --- | --- | --- | --- | --- | --- | --- | --- | --- | --- | --- | --- | --- | --- | --- | --- | --- | --- | --- | --- | --- | --- | --- | --- | --- | --- | --- | --- | --- | --- | --- | --- | --- | --- | --- | --- | --- | --- | --- | --- | --- | --- | --- | --- | --- | --- | --- | --- | --- | --- | --- | --- | --- | --- | --- | --- | --- | --- | --- | --- | --- | --- | --- | --- | --- | --- | --- | --- | --- | --- | --- | --- | --- | --- | --- | --- | --- | --- | --- | --- | --- | --- | --- | --- | --- | --- | --- | --- | --- | --- | --- | --- | --- | --- | --- | --- | --- | --- | --- | --- | --- | --- | --- | --- | --- | --- | --- | --- | --- | --- | --- | --- | --- | --- | --- | --- | --- | --- | --- | --- | --- | --- | --- | --- | --- | --- | --- | --- | --- | --- | --- | --- | --- | --- | --- | --- | --- | --- | --- | --- | --- | --- | --- | --- | --- | --- | --- | --- | --- | --- | --- | --- | --- | --- | --- | --- | --- | --- | --- | --- | --- | --- | --- | --- | --- | --- | --- | --- | --- | --- | --- | --- | --- | --- | --- | --- | --- | --- | --- | --- | --- | --- | --- | --- | --- | --- | --- | --- | --- | --- | --- | --- | --- | --- | --- | --- | --- | --- | --- | --- | --- | --- | --- | --- | --- | --- | --- | --- | --- | --- | --- | --- | --- | --- | --- | --- | --- | --- | --- | --- | --- | --- | --- | --- | --- | --- | --- | --- | --- | --- | --- | --- | --- | --- | --- | --- | --- | --- | --- | --- | --- | --- | --- | --- | --- | --- | --- | --- | --- | --- | --- | --- | --- | --- | --- | --- | --- | --- | --- | --- | --- | --- | --- | --- | --- | --- | --- | --- | --- | --- | --- | --- | --- | --- | --- | --- | --- | --- | --- | --- | --- | --- | --- | --- | --- | --- | --- | --- | --- | --- | --- | --- | --- | --- | --- | --- | --- | --- | --- | --- | --- | --- | --- | --- | --- | --- | --- | --- | --- | --- | --- | --- | --- | --- | --- | --- | --- | --- | --- | --- | --- | --- | --- | --- | --- | --- | --- | --- | --- | --- | --- | --- | --- | --- | --- | --- | --- | --- | --- | --- | --- | --- | --- | --- | --- | --- | --- | --- | --- | --- | --- | --- | --- | --- | --- | --- | --- | --- | --- | --- | --- | --- | --- | --- | --- | --- | --- | --- | --- | --- | --- | --- | --- | --- | --- | --- | --- | --- | --- | --- | --- | --- | --- | --- | --- | --- | --- | --- | --- | --- | --- | --- | --- | --- | --- | --- | --- | --- | --- | --- | --- | --- | --- | --- | --- | --- | --- | --- | --- | --- | --- | --- | --- | --- | --- | --- | --- | --- | --- | --- | --- | --- | --- | --- | --- | --- | --- | --- | --- | --- | --- | --- | --- | --- | --- | --- | --- | --- | --- | --- | --- | --- | --- | --- | --- | --- | --- | --- | --- | --- | --- | --- | --- | --- | --- | --- | --- | --- | --- | --- | --- | --- | --- | --- | --- | --- | --- | --- | --- | --- | --- | --- | --- | --- | --- | --- | --- | --- | --- | --- | --- | --- | --- | --- | --- | --- | --- | --- | --- | --- | --- | --- | --- | --- | --- | --- | --- | --- | --- | --- | --- | --- | --- | --- | --- | --- | --- | --- | --- | --- | --- | --- | --- | --- | --- | --- | --- | --- | --- | --- | --- | --- | --- | --- | --- | --- | --- | --- | --- | --- | --- | --- | --- | --- | --- | --- | --- | --- | --- | --- | --- | --- | --- | --- | --- | --- | --- | --- | --- | --- | --- | --- | --- | --- | --- | --- | --- | --- | --- | --- | --- | --- | --- | --- | --- | --- | --- | --- | --- | --- | --- | --- | --- | --- | --- | --- | --- | --- | --- | --- | --- | --- | --- | --- | --- | --- | --- | --- | --- | --- | --- | --- | --- | --- | --- | --- | --- | --- | --- | --- | --- | --- | --- | --- | --- | --- | --- | --- | --- | --- | --- | --- | --- | --- | --- | --- | --- | --- | --- | --- | --- | --- | --- | --- | --- | --- | --- | --- | --- | --- | --- | --- | --- | --- | --- | --- | --- | --- | --- | --- | --- | --- | --- | --- | --- | --- | --- | --- | --- | --- | --- | --- | --- | --- | --- | --- | --- | --- | --- | --- | --- | --- | --- | --- | --- | --- | --- | --- | --- | --- | --- | --- | --- | --- | --- | --- | --- | --- | --- | --- | --- | --- | --- | --- | --- | --- | --- | --- | --- | --- | --- | --- | --- | --- | --- | --- | --- | --- | --- | --- | --- | --- | --- | --- | --- | --- | --- | --- | --- | --- | --- | --- | --- | --- | --- | --- | --- | --- | --- | --- | --- | --- | --- | --- | --- | --- | --- | --- | --- | --- | --- | --- | --- | --- | --- | --- | --- | --- | --- | --- | --- | --- | --- | --- | --- | --- | --- | --- | --- | --- | --- | --- | --- | --- | --- | --- | --- | --- | --- | --- | --- | --- | --- | --- | --- | --- | --- | --- | --- | --- | --- | --- | --- | --- | --- | --- | --- | --- | --- | --- | --- | --- | --- | --- | --- | --- | --- | --- | --- | --- | --- | --- | --- | --- | --- | --- | --- | --- | --- | --- | --- | --- | --- | --- | --- | --- | --- | --- | --- | --- | --- | --- | --- | --- | --- | --- | --- | --- | --- | --- | --- | --- | --- | --- | --- | --- | --- | --- | --- | --- | --- | --- | --- | --- | --- | --- | --- | --- | --- | --- | --- | --- | --- | --- | --- | --- | --- | --- | --- | --- | --- | --- | --- | --- | --- | --- | --- | --- | --- | --- | --- | --- | --- | --- | --- | --- | --- | --- | --- | --- | --- | --- | --- | --- | --- | --- | --- | --- | --- | --- | --- | --- | --- | --- | --- | --- | --- | --- | --- | --- | --- | --- | --- | --- | --- | --- | --- | --- | --- | --- | --- | --- | --- | --- | --- | --- | --- | --- | --- | --- | --- | --- | --- | --- | --- | --- | --- | --- | --- | --- | --- | --- | --- | --- | --- | --- | --- | --- | --- | --- | --- | --- | --- | --- | --- | --- | --- | --- | --- | --- | --- | --- | --- | --- | --- | --- | --- | --- | --- | --- | --- | --- | --- | --- | --- | --- | --- | --- | --- | --- | --- | --- | --- | --- | --- | --- | --- | --- | --- | --- | --- | --- | --- | --- | --- | --- | --- | --- | --- | --- | --- | --- | --- | --- | --- | --- | --- | --- | --- | --- | --- | --- | --- | --- | --- | --- | --- | --- | --- | --- | --- | --- | --- | --- | --- | --- | --- | --- | --- | --- | --- | --- | --- | --- | --- | --- | --- | --- | --- | --- | --- | --- | --- | --- | --- | --- | --- | --- | --- | --- | --- | --- | --- | --- | --- | --- | --- | --- | --- | --- | --- | --- | --- | --- | --- | --- | --- | --- | --- | --- | --- | --- | --- | --- | --- | --- | --- | --- | --- | --- | --- | --- | --- | --- | --- | --- | --- | --- | --- | --- | --- | --- | --- | --- | --- | --- | --- | --- | --- | --- | --- | --- | --- | --- | --- | --- | --- | --- | --- | --- | --- | --- | --- | --- | --- | --- | --- | --- | --- | --- | --- | --- | --- | --- | --- | --- | --- | --- | --- | --- | --- | --- | --- | --- | --- | --- | --- | --- | --- | --- | --- | --- | --- | --- | --- | --- | --- | --- | --- | --- | --- | --- | --- | --- | --- | --- | --- | --- | --- | --- | --- | --- | --- | --- | --- | --- | --- | --- | --- | --- | --- | --- | --- | --- | --- | --- | --- | --- | --- | --- | --- | --- | --- | --- | --- | --- | --- | --- | --- | --- | --- | --- | --- | --- | --- | --- | --- | --- | --- | --- | --- | --- | --- | --- | --- | --- | --- | --- | --- | --- | --- | --- | --- | --- | --- | --- | --- | --- | --- | --- | --- | --- | --- | --- | --- | --- | --- | --- | --- | --- | --- | --- | --- | --- | --- | --- | --- | --- | --- | --- | --- | --- | --- | --- | --- | --- | --- | --- | --- | --- | --- | --- | --- | --- | --- | --- | --- | --- | --- | --- | --- | --- | --- | --- | --- | --- | --- | --- | --- | --- | --- | --- | --- | --- | --- | --- | --- | --- | --- | --- | --- | --- | --- | --- | --- | --- | --- | --- | --- | --- | --- | --- | --- | --- | --- | --- | --- | --- | --- | --- | --- | --- | --- | --- | --- | --- | --- | --- | --- | --- | --- | --- | --- | --- | --- | --- | --- | --- | --- | --- | --- | --- | --- | --- | --- | --- | --- | --- | --- | --- | --- | --- | --- | --- | --- | --- | --- | --- | --- | --- | --- | --- | --- | --- | --- | --- | --- | --- | --- | --- | --- | --- | --- | --- | --- | --- | --- | --- | --- | --- | --- | --- | --- | --- | --- | --- | --- | --- | --- | --- | --- | --- | --- | --- | --- | --- | --- | --- | --- | --- | --- | --- | --- | --- | --- | --- | --- | --- | --- | --- | --- | --- | --- | --- | --- | --- | --- | --- | --- | --- | --- | --- | --- | --- | --- | --- | --- | --- | --- | --- | --- | --- | --- | --- | --- | --- | --- | --- | --- | --- | --- | --- | --- | --- | --- | --- | --- | --- | --- | --- | --- | --- | --- | --- | --- | --- | --- | --- | --- | --- | --- | --- | --- | --- | --- | --- | --- | --- | --- | --- | --- | --- | --- | --- | --- | --- | --- | --- | --- | --- | --- | --- | --- | --- | --- | --- | --- | --- | --- | --- | --- | --- | --- | --- | --- | --- | --- | --- | --- | --- | --- | --- | --- | --- | --- | --- | --- | --- | --- | --- | --- | --- | --- | --- | --- | --- | --- | --- | --- | --- | --- | --- | --- | --- | --- | --- | --- | --- | --- | --- | --- | --- | --- | --- | --- | --- | --- | --- | --- | --- | --- | --- | --- | --- | --- | --- | --- | --- | --- | --- | --- | --- | --- | --- | --- | --- | --- | --- | --- | --- | --- | --- | --- | --- | --- | --- | --- | --- | --- | --- | --- | --- | --- | --- | --- | --- | --- | --- | --- | --- | --- | --- | --- | --- | --- | --- | --- | --- | --- | --- | --- | --- | --- | --- | --- | --- | --- | --- | --- | --- | --- | --- | --- | --- | --- | --- | --- | --- | --- | --- | --- | --- | --- | --- | --- | --- | --- | --- | --- | --- | --- | --- | --- | --- | --- | --- | --- | --- | --- | --- | --- | --- | --- | --- | --- | --- | --- | --- | --- | --- | --- | --- | --- | --- | --- | --- | --- | --- | --- | --- | --- | --- | --- | --- | --- | --- | --- | --- | --- | --- | --- | --- | --- | --- | --- | --- | --- | --- | --- | --- | --- | --- | --- | --- | --- | --- | --- | --- | --- | --- | --- | --- | --- | --- | --- | --- | --- | --- | --- | --- | --- | --- | --- | --- | --- | --- | --- | --- | --- | --- | --- | --- | --- | --- | --- | --- | --- | --- | --- | --- | --- | --- | --- | --- | --- | --- | --- | --- | --- | --- | --- | --- | --- | --- | --- | --- | --- | --- | --- | --- | --- | --- | --- | --- | --- | --- | --- | --- | --- | --- | --- | --- | --- | --- | --- | --- | --- | --- | --- | --- | --- | --- | --- | --- | --- | --- | --- | --- | --- | --- | --- | --- | --- | --- | --- | --- | --- | --- | --- | --- | --- | --- | --- | --- | --- | --- | --- | --- | --- | --- | --- | --- | --- | --- | --- | --- | --- | --- | --- | --- | --- | --- | --- | --- | --- | --- | --- | --- | --- | --- | --- | --- | --- | --- | --- | --- | --- | --- | --- | --- | --- | --- | --- | --- | --- | --- | --- | --- | --- | --- | --- | --- | --- | --- | --- | --- | --- | --- | --- | --- | --- | --- | --- | --- | --- | --- | --- | --- | --- | --- | --- | --- | --- | --- | --- | --- | --- | --- | --- | --- | --- | --- | --- | --- | --- | --- | --- | --- | --- | --- | --- | --- | --- | --- | --- | --- | --- | --- | --- | --- | --- | --- | --- | --- | --- | --- | --- | --- | --- | --- | --- | --- | --- | --- | --- | --- | --- | --- | --- | --- | --- | --- | --- | --- | --- | --- | --- | --- | --- | --- | --- | --- | --- | --- | --- | --- | --- | --- | --- | --- | --- | --- | --- | --- | --- | --- | --- | --- | --- | --- | --- | --- | --- | --- | --- | --- | --- | --- | --- | --- | --- | --- | --- | --- | --- | --- | --- | --- | --- | --- | --- | --- | --- | --- | --- | --- | --- | --- | --- | --- | --- | --- | --- | --- | --- | --- | --- | --- | --- | --- | --- | --- | --- | --- | --- | --- | --- | --- | --- | --- | --- | --- | --- | --- | --- | --- | --- | --- | --- | --- | --- | --- | --- | --- | --- | --- | --- | --- | --- | --- | --- | --- | --- | --- | --- | --- | --- | --- | --- | --- | --- | --- | --- | --- | --- | --- | --- | --- | --- | --- | --- | --- | --- | --- | --- | --- | --- | --- | --- | --- | --- | --- | --- | --- | --- | --- | --- | --- | --- | --- | --- | --- | --- | --- | --- | --- | --- | --- | --- | --- | --- | --- | --- | --- | --- | --- | --- | --- | --- | --- | --- | --- | --- | --- | --- | --- | --- | --- | --- | --- | --- | --- | --- | --- | --- | --- | --- | --- | --- | --- | --- | --- | --- | --- | --- | --- | --- | --- | --- | --- | --- | --- | --- | --- | --- | --- | --- | --- | --- | --- | --- | --- | --- | --- | --- | --- | --- | --- | --- | --- | --- | --- | --- | --- | --- | --- | --- | --- | --- | --- | --- | --- | --- | --- | --- | --- | --- | --- | --- | --- | --- | --- | --- | --- | --- | --- | --- | --- | --- | --- | --- | --- | --- | --- | --- | --- | --- | --- | --- | --- | --- | --- | --- | --- | --- | --- | --- | --- | --- | --- | --- | --- | --- | --- | --- | --- | --- | --- | --- | --- | --- | --- | --- | --- | --- | --- | --- | --- | --- | --- | --- | --- | --- | --- | --- | --- | --- | --- | --- | --- | --- | --- | --- | --- | --- | --- | --- | --- | --- | --- | --- | --- | --- | --- | --- | --- | --- | --- | --- | --- | --- | --- | --- | --- | --- | --- | --- | --- | --- | --- | --- | --- | --- | --- | --- | --- | --- | --- | --- | --- | --- | --- | --- | --- | --- | --- | --- | --- | --- | --- | --- | --- | --- | --- | --- | --- | --- | --- | --- | --- | --- | --- | --- | --- | --- | --- | --- | --- | --- | --- | --- | --- | --- | --- | --- | --- | --- | --- | --- | --- | --- | --- | --- | --- | --- | --- | --- | --- | --- | --- | --- | --- | --- | --- | --- | --- | --- | --- | --- | --- | --- | --- | --- | --- | --- | --- | --- | --- | --- | --- | --- | --- | --- | --- | --- | --- | --- | --- | --- | --- | --- | --- | --- | --- | --- | --- | --- | --- | --- | --- | --- | --- | --- | --- | --- | --- | --- | --- | --- | --- | --- | --- | --- | --- | --- | --- | --- | --- | --- | --- | --- | --- | --- | --- | --- | --- | --- | --- | --- | --- | --- | --- | --- | --- | --- | --- | --- | --- | --- | --- | --- | --- | --- | --- | --- | --- | --- | --- | --- | --- | --- | --- | --- | --- | --- | --- | --- | --- | --- | --- | --- | --- | --- | --- | --- | --- | --- | --- | --- | --- | --- | --- | --- | --- | --- | --- | --- | --- | --- | --- | --- | --- | --- | --- | --- | --- | --- | --- | --- | --- | --- | --- | --- | --- | --- | --- | --- | --- | --- | --- | --- | --- | --- | --- | --- | --- | --- | --- | --- | --- | --- | --- | --- | --- | --- | --- | --- | --- | --- | --- | --- | --- | --- | --- | --- | --- | --- | --- | --- | --- | --- | --- | --- | --- | --- | --- | --- | --- | --- | --- | --- | --- | --- | --- | --- | --- | --- | --- | --- | --- | --- | --- | --- | --- | --- | --- | --- | --- | --- | --- | --- | --- | --- | --- | --- | --- | --- | --- | --- | --- | --- | --- | --- | --- | --- | --- | --- | --- | --- | --- | --- | --- | --- | --- | --- | --- | --- | --- | --- | --- | --- | --- | --- | --- | --- | --- | --- | --- | --- | --- | --- | --- | --- | --- | --- | --- | --- | --- | --- | --- | --- | --- | --- | --- | --- | --- | --- | --- | --- | --- | --- | --- | --- | --- | --- | --- | --- | --- | --- | --- | --- | --- | --- | --- | --- | --- | --- | --- | --- | --- | --- | --- | --- | --- | --- | --- | --- | --- | --- | --- | --- | --- | --- | --- | --- | --- | --- | --- | --- | --- | --- | --- | --- | --- | --- | --- | --- | --- | --- | --- | --- | --- | --- | --- | --- | --- | --- | --- | --- | --- | --- | --- | --- | --- | --- | --- | --- | --- | --- | --- | --- | --- | --- | --- | --- | --- | --- | --- | --- | --- | --- | --- | --- | --- | --- | --- | --- | --- | --- | --- | --- | --- | --- | --- | --- | --- | --- | --- | --- | --- | --- | --- | --- | --- | --- | --- | --- | --- | --- | --- | --- | --- | --- | --- | --- | --- | --- | --- | --- | --- | --- | --- | --- | --- | --- | --- | --- | --- | --- | --- | --- | --- | --- | --- | --- | --- | --- | --- | --- | --- | --- | --- | --- | --- | --- | --- | --- | --- | --- | --- | --- | --- | --- | --- | --- | --- | --- | --- | --- | --- | --- | --- | --- | --- | --- | --- | --- | --- | --- | --- | --- | --- | --- | --- | --- | --- | --- | --- | --- | --- | --- | --- | --- | --- | --- | --- | --- | --- | --- | --- | --- | --- | --- | --- | --- | --- | --- | --- | --- | --- | --- | --- | --- | --- | --- | --- | --- | --- | --- | --- | --- | --- | --- | --- | --- | --- | --- | --- | --- | --- | --- | --- | --- | --- | --- | --- | --- | --- | --- | --- | --- | --- | --- | --- | --- | --- | --- | --- | --- | --- | --- | --- | --- | --- | --- | --- | --- | --- | --- | --- | --- | --- | --- | --- | --- | --- | --- | --- | --- | --- | --- | --- | --- | --- | --- | --- | --- | --- | --- | --- | --- | --- | --- | --- | --- | --- | --- | --- | --- | --- | --- | --- | --- | --- | --- | --- | --- | --- | --- | --- | --- | --- | --- | --- | --- | --- | --- | --- | --- | --- | --- | --- | --- | --- | --- | --- | --- | --- | --- | --- | --- | --- | --- | --- | --- | --- | --- | --- | --- | --- | --- | --- | --- | --- | --- | --- | --- | --- | --- | --- | --- | --- | --- | --- | --- | --- | --- | --- | --- | --- | --- | --- | --- | --- | --- | --- | --- | --- | --- | --- | --- | --- | --- | --- | --- | --- | --- | --- | --- | --- | --- | --- | --- | --- | --- | --- | --- | --- | --- | --- | --- | --- | --- | --- | --- | --- | --- | --- | --- | --- | --- | --- | --- | --- | --- | --- | --- | --- | --- | --- | --- | --- | --- | --- | --- | --- | --- | --- | --- | --- | --- | --- | --- | --- | --- | --- | --- | --- | --- | --- | --- | --- | --- | --- | --- | --- | --- | --- | --- | --- | --- | --- | --- | --- | --- | --- | --- | --- | --- | --- | --- | --- | --- | --- | --- | --- | --- | --- | --- | --- | --- | --- | --- | --- | --- | --- | --- | --- | --- | --- | --- | --- | --- | --- | --- | --- | --- | --- | --- | --- | --- | --- | --- | --- | --- | --- | --- | --- | --- | --- | --- | --- | --- | --- | --- | --- | --- | --- | --- | --- | --- | --- | --- | --- | --- | --- | --- | --- | --- | --- | --- | --- | --- | --- | --- | --- | --- | --- | --- | --- | --- | --- | --- | --- | --- | --- | --- | --- | --- | --- | --- | --- | --- | --- | --- | --- | --- | --- | --- | --- | --- | --- | --- | --- | --- | --- | --- | --- | --- | --- | --- | --- | --- | --- | --- | --- | --- | --- | --- | --- | --- | --- | --- | --- | --- | --- | --- | --- | --- | --- | --- | --- | --- | --- | --- | --- | --- | --- | --- | --- | --- | --- | --- | --- | --- | --- | --- | --- | --- | --- | --- | --- | --- | --- | --- | --- | --- | --- | --- | --- | --- | --- | --- | --- | --- | --- | --- | --- | --- | --- | --- | --- | --- | --- | --- | --- | --- | --- | --- | --- | --- | --- | --- | --- | --- | --- | --- | --- | --- | --- | --- | --- | --- | --- | --- | --- | --- | --- | --- | --- | --- | --- | --- | --- | --- | --- | --- | --- | --- | --- | --- | --- | --- | --- | --- | --- | --- | --- | --- | --- | --- | --- | --- | --- | --- | --- | --- | --- | --- | --- | --- | --- | --- | --- | --- | --- | --- | --- | --- | --- | --- | --- | --- | --- | --- | --- | --- | --- | --- | --- | --- | --- | --- | --- | --- | --- | --- | --- | --- | --- | --- | --- | --- | --- | --- | --- | --- | --- | --- | --- | --- | --- | --- | --- | --- | --- | --- | --- | --- | --- | --- | --- | --- | --- | --- | --- | --- | --- | --- | --- | --- | --- | --- | --- | --- | --- | --- | --- | --- | --- | --- | --- | --- | --- | --- | --- | --- | --- | --- | --- | --- | --- | --- | --- | --- | --- | --- | --- | --- | --- | --- | --- | --- | --- | --- | --- | --- | --- | --- | --- | --- | --- | --- | --- | --- | --- | --- | --- | --- | --- | --- | --- | --- | --- | --- | --- | --- | --- | --- | --- | --- | --- | --- | --- | --- | --- | --- | --- | --- | --- | --- | --- | --- | --- | --- | --- | --- | --- | --- | --- | --- | --- | --- | --- | --- | --- | --- | --- | --- | --- | --- | --- | --- | --- | --- | --- | --- | --- | --- | --- | --- | --- | --- | --- | --- | --- | --- | --- | --- | --- | --- | --- | --- | --- | --- | --- | --- | --- | --- | --- | --- | --- | --- | --- | --- | --- | --- | --- | --- | --- | --- | --- | --- | --- | --- | --- | --- | --- | --- | --- | --- | --- | --- | --- | --- | --- | --- | --- | --- | --- | --- | --- | --- | --- | --- | --- | --- | --- | --- | --- | --- | --- | --- | --- | --- | --- | --- | --- | --- | --- | --- | --- | --- | --- | --- | --- | --- | --- | --- | --- | --- | --- | --- | --- | --- | --- | --- | --- | --- | --- | --- | --- | --- | --- | --- | --- | --- | --- | --- | --- | --- | --- | --- | --- | --- | --- | --- | --- | --- | --- | --- | --- | --- | --- | --- | --- | --- | --- | --- | --- | --- | --- | --- | --- | --- | --- | --- | --- | --- | --- | --- | --- | --- | --- | --- | --- | --- | --- | --- | --- | --- | --- | --- | --- | --- | --- | --- | --- | --- | --- | --- | --- | --- | --- | --- | --- | --- | --- | --- | --- | --- | --- | --- | --- | --- | --- | --- | --- | --- | --- | --- | --- | --- | --- | --- | --- | --- | --- | --- | --- | --- | --- | --- | --- | --- | --- | --- | --- | --- | --- | --- | --- | --- | --- | --- | --- | --- | --- | --- | --- | --- | --- | --- | --- | --- | --- | --- | --- | --- | --- | --- | --- | --- | --- | --- | --- | --- | --- | --- | --- | --- | --- | --- | --- | --- | --- | --- | --- | --- | --- | --- | --- | --- | --- | --- | --- | --- | --- | --- | --- | --- | --- | --- | --- | --- | --- | --- | --- | --- | --- | --- | --- | --- | --- | --- | --- | --- | --- | --- | --- | --- | --- | --- | --- | --- | --- | --- | --- | --- | --- | --- | --- | --- | --- | --- | --- | --- | --- | --- | --- | --- | --- | --- | --- | --- | --- | --- | --- | --- | --- | --- | --- | --- | --- | --- | --- | --- | --- | --- | --- | --- | --- | --- | --- | --- | --- | --- | --- | --- | --- | --- | --- | --- | --- | --- | --- | --- | --- | --- | --- | --- | --- | --- | --- | --- | --- | --- | --- | --- | --- | --- | --- | --- | --- | --- | --- | --- | --- | --- | --- | --- | --- | --- | --- | --- | --- | --- | --- | --- | --- | --- | --- | --- | --- | --- | --- | --- | --- | --- | --- | --- | --- | --- | --- | --- | --- | --- | --- | --- | --- | --- | --- | --- | --- | --- | --- | --- | --- | --- | --- | --- | --- | --- | --- | --- | --- | --- | --- | --- | --- | --- | --- | --- | --- | --- | --- | --- | --- | --- | --- | --- | --- | --- | --- | --- | --- | --- | --- | --- | --- | --- | --- | --- | --- | --- | --- | --- | --- | --- | --- | --- | --- | --- | --- | --- | --- | --- | --- | --- | --- | --- | --- | --- | --- | --- | --- | --- | --- | --- | --- | --- | --- | --- | --- | --- | --- | --- | --- | --- | --- | --- | --- | --- | --- | --- | --- | --- | --- | --- | --- | --- | --- | --- | --- | --- | --- | --- | --- | --- | --- | --- | --- | --- | --- | --- | --- | --- | --- | --- | --- | --- | --- | --- | --- | --- | --- | --- | --- | --- | --- | --- | --- | --- | --- | --- | --- | --- | --- | --- | --- | --- | --- | --- | --- | --- | --- | --- | --- | --- | --- | --- | --- | --- | --- | --- | --- | --- | --- | --- | --- | --- | --- | --- | --- | --- | --- | --- | --- | --- | --- | --- | --- | --- | --- | --- | --- | --- | --- | --- | --- | --- | --- | --- | --- | --- | --- | --- | --- | --- | --- | --- | --- | --- | --- | --- | --- | --- | --- | --- | --- | --- | --- | --- | --- | --- | --- | --- | --- | --- | --- | --- | --- | --- | --- | --- | --- | --- | --- | --- | --- | --- | --- | --- | --- | --- | --- | --- | --- | --- | --- | --- | --- | --- | --- | --- | --- | --- | --- | --- | --- | --- | --- | --- | --- | --- | --- | --- | --- | --- | --- | --- | --- | --- | --- | --- | --- | --- | --- | --- | --- | --- | --- | --- | --- | --- | --- | --- | --- | --- | --- | --- | --- | --- | --- | --- | --- | --- | --- | --- | --- | --- | --- | --- | --- | --- | --- | --- | --- | --- | --- | --- | --- | --- | --- | --- | --- | --- | --- | --- | --- | --- | --- | --- | --- | --- | --- | --- | --- | --- | --- | --- | --- | --- | --- | --- | --- | --- | --- | --- | --- | --- | --- | --- | --- | --- | --- | --- | --- | --- | --- | --- | --- | --- | --- | --- | --- | --- | --- | --- | --- | --- | --- | --- | --- | --- | --- | --- | --- | --- | --- | --- | --- | --- | --- | --- | --- | --- | --- | --- | --- | --- | --- | --- | --- | --- | --- | --- | --- | --- | --- | --- | --- | --- | --- | --- | --- | --- | --- | --- | --- | --- | --- | --- | --- | --- | --- | --- | --- | --- | --- | --- | --- | --- | --- | --- | --- | --- | --- | --- | --- | --- | --- | --- | --- | --- | --- | --- | --- | --- | --- | --- | --- | --- | --- | --- | --- | --- | --- | --- | --- | --- | --- | --- | --- | --- | --- | --- | --- | --- | --- | --- | --- | --- | --- | --- | --- | --- | --- | --- | --- | --- | --- | --- | --- | --- | --- | --- | --- | --- | --- | --- | --- | --- | --- | --- | --- | --- | --- | --- | --- | --- | --- | --- | --- | --- | --- | --- | --- | --- | --- | --- | --- | --- | --- | --- | --- | --- | --- | --- | --- | --- | --- | --- | --- | --- | --- | --- | --- | --- | --- | --- | --- | --- | --- | --- | --- | --- | --- | --- | --- | --- | --- | --- | --- | --- | --- | --- | --- | --- | --- | --- | --- | --- | --- | --- | --- | --- | --- | --- | --- | --- | --- | --- | --- | --- | --- | --- | --- | --- | --- | --- | --- | --- | --- | --- | --- | --- | --- | --- | --- | --- | --- | --- | --- | --- | --- | --- | --- | --- | --- | --- | --- | --- | --- | --- | --- | --- | --- | --- | --- | --- | --- | --- | --- | --- | --- | --- | --- | --- | --- | --- | --- | --- | --- | --- | --- | --- | --- | --- | --- | --- | --- | --- | --- | --- | --- | --- | --- | --- | --- | --- | --- | --- | --- | --- | --- | --- | --- | --- | --- | --- | --- | --- | --- | --- | --- | --- | --- | --- | --- | --- | --- | --- | --- | --- | --- | --- | --- | --- | --- | --- | --- | --- | --- | --- | --- | --- | --- | --- | --- | --- | --- | --- | --- | --- | --- | --- | --- | --- | --- | --- | --- | --- | --- | --- | --- | --- | --- | --- | --- | --- | --- | --- | --- | --- | --- | --- | --- | --- | --- | --- | --- | --- | --- | --- | --- | --- | --- | --- | --- | --- | --- | --- | --- | --- | --- | --- | --- | --- | --- | --- | --- | --- | --- | --- | --- | --- | --- | --- | --- | --- | --- | --- | --- | --- | --- | --- | --- | --- | --- | --- | --- | --- | --- | --- | --- | --- | --- | --- | --- | --- | --- | --- | --- | --- | --- | --- | --- | --- | --- | --- | --- | --- | --- | --- | --- | --- | --- | --- | --- | --- | --- | --- | --- | --- | --- | --- | --- | --- | --- | --- | --- | --- | --- | --- | --- | --- | --- | --- | --- | --- | --- | --- | --- | --- | --- | --- | --- | --- | --- | --- | --- | --- | --- | --- | --- | --- | --- | --- | --- | --- | --- | --- | --- | --- | --- | --- | --- | --- | --- | --- | --- | --- | --- | --- | --- | --- | --- | --- | --- | --- | --- | --- | --- | --- | --- | --- | --- | --- | --- | --- | --- | --- | --- | --- | --- | --- | --- | --- | --- | --- | --- | --- | --- | --- | --- | --- | --- | --- | --- | --- | --- | --- | --- | --- | --- | --- | --- | --- | --- | --- | --- | --- | --- | --- | --- | --- | --- | --- | --- | --- | --- | --- | --- | --- | --- | --- | --- | --- | --- | --- | --- | --- | --- | --- | --- | --- | --- | --- | --- | --- | --- | --- | --- | --- | --- | --- | --- | --- | --- | --- | --- | --- | --- | --- | --- | --- | --- | --- | --- | --- | --- | --- | --- | --- | --- | --- | --- | --- | --- | --- | --- | --- | --- | --- | --- | --- | --- | --- | --- | --- | --- | --- | --- | --- | --- | --- | --- | --- | --- | --- | --- | --- | --- | --- | --- | --- | --- | --- | --- | --- | --- | --- | --- | --- | --- | --- | --- | --- | --- | --- | --- | --- | --- | --- | --- | --- | --- | --- | --- | --- | --- | --- | --- | --- | --- | --- | --- | --- | --- | --- | --- | --- | --- | --- | --- | --- | --- | --- | --- | --- | --- | --- | --- | --- | --- | --- | --- | --- | --- | --- | --- | --- | --- | --- | --- | --- | --- | --- | --- | --- | --- | --- | --- | --- | --- | --- | --- | --- | --- | --- | --- | --- | --- | --- | --- | --- | --- | --- | --- | --- | --- | --- | --- | --- | --- | --- | --- | --- | --- | --- | --- | --- | --- | --- | --- | --- | --- | --- | --- | --- | --- | --- | --- | --- | --- | --- | --- | --- | --- | --- | --- | --- | --- | --- | --- | --- | --- | --- | --- | --- | --- | --- | --- | --- | --- | --- | --- | --- | --- | --- | --- | --- | --- | --- | --- | --- | --- | --- | --- | --- | --- | --- | --- | --- | --- | --- | --- | --- | --- | --- | --- | --- | --- | --- | --- | --- | --- | --- | --- | --- | --- | --- | --- | --- | --- | --- | --- | --- | --- | --- | --- | --- | --- | --- | --- | --- | --- | --- | --- | --- | --- | --- | --- | --- | --- | --- | --- | --- | --- | --- | --- | --- | --- | --- | --- | --- | --- | --- | --- | --- | --- | --- | --- | --- | --- | --- | --- | --- | --- | --- | --- | --- | --- | --- | --- | --- | --- | --- | --- | --- | --- | --- | --- | --- | --- | --- | --- | --- | --- | --- | --- | --- | --- | --- | --- | --- | --- | --- | --- | --- | --- | --- | --- | --- | --- | --- | --- | --- | --- | --- | --- | --- | --- | --- | --- | --- | --- | --- | --- | --- | --- | --- | --- | --- | --- | --- | --- | --- | --- | --- | --- | --- | --- | --- | --- | --- | --- | --- | --- | --- | --- | --- | --- | --- | --- | --- | --- | --- | --- | --- | --- | --- | --- | --- | --- | --- | --- | --- | --- | --- | --- | --- | --- | --- | --- | --- | --- | --- | --- | --- | --- | --- | --- | --- | --- | --- | --- | --- | --- | --- | --- | --- | --- | --- | --- | --- | --- | --- | --- | --- | --- | --- | --- | --- | --- | --- | --- | --- | --- | --- | --- | --- | --- | --- | --- | --- | --- | --- | --- | --- | --- | --- | --- | --- | --- | --- | --- | --- | --- | --- | --- | --- | --- | --- | --- | --- | --- | --- | --- | --- | --- | --- | --- | --- | --- | --- | --- | --- | --- | --- | --- | --- | --- | --- | --- | --- | --- | --- | --- | --- | --- | --- | --- | --- | --- | --- | --- | --- | --- | --- | --- | --- | --- | --- | --- | --- | --- | --- | --- | --- | --- | --- | --- | --- | --- | --- | --- | --- |
| |  |  |  |  |  |  |  |  |  |  |  |  |  |  |  |  |  |  |  |  |  |  |  |  |  |  |  |  |  |  |  |  |  |  |  |  |  |  |  |  |  |  |  |  |  |  |  |  |  |  |  |  |  |  |  |  |  |  | | --- | --- | --- | --- | --- | --- | --- | --- | --- | --- | --- | --- | --- | --- | --- | --- | --- | --- | --- | --- | --- | --- | --- | --- | --- | --- | --- | --- | --- | --- | --- | --- | --- | --- | --- | --- | --- | --- | --- | --- | --- | --- | --- | --- | --- | --- | --- | --- | --- | --- | --- | --- | --- | --- | --- | --- | --- | --- | | G0VKH7/1-766 | 1 | M | L | F | L | P | R | T | I | T | Q | L | P | L | R | C | V | R | C | - | - | - | - | - | - | - | R | G | I | P | A | - | - | - | T | I | L | S | K | - | T | A | - | Y | Q | L | S | R | K | R | A | F | Q | T | S | T | 43 | | Q6CRY5/1-755 | 1 | M | - | - | F | K | R | V | G | L | I | A | G | - | - | - | - | - | - | - | - | - | - | - | - | - | I | A | G | P | V | - | - | - | A | G | S | S | R | - | F | S | - | A | V | S | F | S | K | R | A | F | S | A | S | S | 35 | | Q6FUQ6/1-757 | 1 | M | L | L | V | P | R | V | P | V | V | M | Q | G | K | C | G | - | - | - | - | - | - | - | - | - | - | - | - | - | - | - | - | - | L | L | K | I | S | - | R | P | - | L | Q | G | S | L | S | R | G | F | H | F | S | R | 36 | | Q75CZ5/1-757 | 1 | M | - | - | L | E | R | A | A | L | L | H | R | L | R | L | P | A | - | - | - | - | - | - | - | - | H | S | L | P | F | - | - | - | I | Y | N | G | - | - | - | A | - | L | F | G | G | A | K | R | S | F | S | A | T | S | 38 | | A7TFN8/1-776 | 1 | M | V | L | L | R | G | V | G | L | L | R | S | S | N | V | T | S | G | G | A | A | A | T | A | S | R | G | G | N | L | F | K | C | L | M | N | A | K | - | R | P | D | G | M | L | V | N | R | R | G | F | H | G | S | M | 54 | | C5DNQ2/1-763 | 1 | M | S | F | L | R | R | A | V | S | G | H | S | Y | G | P | K | T | - | - | - | - | - | - | - | - | F | G | R | M | G | - | - | - | L | L | N | G | S | - | I | M | - | P | I | L | T | T | Q | R | Q | V | H | A | S | S | 42 | | C5DX66/1-769 | 1 | M | N | L | A | R | S | A | G | A | L | R | - | - | - | - | - | - | - | - | - | - | - | T | G | V | Y | L | T | P | R | - | - | - | I | L | G | A | S | C | K | S | I | N | L | T | H | G | V | R | L | F | H | A | N | K | 41 | | Kwal\_14.1090/1-763 | 1 | M | S | L | Y | R | K | N | A | A | G | G | L | H | R | L | A | S | - | - | - | - | - | - | - | - | L | T | R | P | G | - | - | - | L | L | N | G | F | - | K | S | - | H | V | S | Q | T | Y | R | M | I | H | A | S | S | 42 | | Sbay\_672.50/1-761 | 1 | M | S | V | Q | T | M | M | W | V | P | R | - | - | R | V | V | R | - | - | - | - | - | - | - | - | G | S | V | P | F | - | - | - | F | A | N | S | - | - | K | L | - | C | L | G | L | S | R | R | S | F | H | G | S | S | 39 | | SAKL0B00858g/1-761 | 1 | M | S | M | F | R | N | T | A | L | L | S | - | - | R | L | S | S | S | G | L | V | A | S | T | L | F | L | K | P | T | - | - | - | V | S | N | G | - | - | - | - | - | - | - | - | - | F | T | R | N | F | H | N | T | L | 41 | | P25039/1-761 | 1 | M | S | V | Q | K | M | M | W | V | P | R | - | - | K | M | V | G | - | - | - | - | - | - | - | - | G | R | I | P | F | - | - | - | F | T | C | S | - | - | K | V | - | F | S | G | F | S | R | R | S | F | H | E | S | P | 39 | |  | | G0VKH7/1-766 | 44 | R | L | R | S | E | Y | - | E | E | E | K | P | I | L | D | E | I | S | K | S | L | T | T | Q | D | I | E | S | S | K | K | L | R | N | I | G | I | S | A | H | I | D | S | G | K | T | T | F | T | E | R | V | L | Y | Y | 97 | | Q6CRY5/1-755 | 36 | K | - | R | C | T | Y | - | E | E | E | R | A | V | L | D | E | I | Q | P | L | L | S | E | K | D | I | D | A | S | K | K | L | R | N | I | G | I | S | A | H | I | D | S | G | K | T | T | F | T | E | R | V | L | Y | Y | 88 | | Q6FUQ6/1-757 | 37 | A | H | R | S | E | Y | - | D | E | E | K | V | V | I | D | E | I | N | K | K | L | T | P | V | D | I | Q | N | Q | Q | K | L | R | N | I | G | I | S | A | H | I | D | S | G | K | T | T | F | T | E | R | V | L | Y | Y | 90 | | Q75CZ5/1-757 | 39 | K | - | R | C | T | Y | - | E | E | E | K | A | V | L | D | E | L | K | P | Q | L | T | A | D | D | L | K | H | S | K | L | L | R | N | I | G | V | S | A | H | I | D | S | G | K | T | T | F | T | E | R | V | L | Y | Y | 91 | | A7TFN8/1-776 | 55 | V | Y | K | A | A | M | S | E | E | E | A | S | E | L | S | D | V | T | K | Q | L | T | G | A | D | L | Q | A | A | S | K | L | R | N | I | G | I | S | A | H | I | D | S | G | K | T | T | F | T | E | R | V | L | Y | Y | 109 | | C5DNQ2/1-763 | 43 | V | - | L | R | T | Y | - | E | E | E | K | A | V | L | D | E | I | A | P | R | L | T | E | R | D | L | E | T | S | R | K | L | R | N | I | G | I | S | A | H | I | D | S | G | K | T | T | F | T | E | R | V | L | F | Y | 95 | | C5DX66/1-769 | 42 | I | C | R | D | S | Y | - | E | E | E | K | A | T | L | D | E | I | H | K | L | M | K | P | Q | D | F | Q | V | A | D | K | I | R | N | I | G | I | S | A | H | I | D | S | G | K | T | T | F | T | E | R | V | L | Y | Y | 95 | | Kwal\_14.1090/1-763 | 43 | V | - | L | K | S | Y | - | E | D | E | K | V | V | L | D | E | I | A | P | K | L | T | A | H | D | L | E | T | S | R | K | L | R | N | I | G | I | S | A | H | I | D | S | G | K | T | T | F | T | E | R | V | L | F | Y | 95 | | Sbay\_672.50/1-761 | 40 | L | A | R | S | T | Y | - | E | E | E | K | V | L | V | D | E | I | K | Q | Q | L | T | P | A | D | I | E | L | S | K | K | L | R | N | I | G | I | S | A | H | I | D | S | G | K | T | T | F | T | E | R | V | L | Y | Y | 93 | | SAKL0B00858g/1-761 | 42 | A | - | K | R | S | Y | - | E | E | E | K | V | I | L | D | E | I | A | P | N | L | T | E | K | D | L | E | T | S | R | K | L | R | N | I | G | I | S | A | H | I | D | S | G | K | T | T | F | T | E | R | V | L | F | Y | 94 | | P25039/1-761 | 40 | L | A | R | S | T | Y | - | E | E | E | K | V | L | V | D | E | I | K | Q | K | L | T | P | D | D | I | G | R | C | N | K | L | R | N | I | G | I | S | A | H | I | D | S | G | K | T | T | F | T | E | R | V | L | Y | Y | 93 | |  | | G0VKH7/1-766 | 98 | T | G | R | I | K | A | I | H | E | V | R | G | K | D | N | V | G | A | K | M | D | S | M | D | L | E | R | E | K | G | I | T | I | Q | S | A | A | T | Y | C | S | W | D | K | D | G | K | P | Y | H | Y | N | L | I | D | 152 | | Q6CRY5/1-755 | 89 | T | G | R | I | K | A | I | H | E | V | R | G | R | D | N | V | G | A | K | M | D | S | M | D | L | E | R | E | K | G | I | T | I | Q | S | A | A | T | Y | C | S | W | D | K | D | N | E | S | Y | H | F | N | L | I | D | 143 | | Q6FUQ6/1-757 | 91 | T | K | R | I | K | E | I | H | E | V | R | G | R | D | N | V | G | A | T | M | D | F | M | D | L | E | R | E | K | G | I | T | I | Q | S | A | A | T | Y | C | S | W | D | K | D | K | N | S | Y | H | F | N | L | I | D | 145 | | Q75CZ5/1-757 | 92 | T | G | R | I | K | A | I | H | E | V | R | G | R | D | S | V | G | A | K | M | D | H | M | D | L | E | R | E | K | G | I | T | I | Q | S | A | A | T | Y | C | S | W | D | K | D | Q | E | S | Y | H | F | N | L | I | D | 146 | | A7TFN8/1-776 | 110 | T | G | R | I | K | A | I | H | E | V | R | G | R | D | N | V | G | A | K | M | D | S | M | D | L | E | R | E | K | G | I | T | I | Q | S | A | A | T | Y | C | S | W | N | K | D | Q | K | D | Y | H | F | N | L | I | D | 164 | | C5DNQ2/1-763 | 96 | T | G | R | I | K | A | I | H | E | V | R | G | R | D | N | V | G | A | K | M | D | S | M | D | L | E | R | E | K | G | I | T | I | Q | S | A | A | T | Y | C | S | W | D | K | D | G | Q | N | Y | H | F | N | L | I | D | 150 | | C5DX66/1-769 | 96 | T | G | R | I | K | A | I | H | E | V | R | G | R | D | N | V | G | A | K | M | D | H | M | D | L | E | R | E | K | G | I | T | I | Q | S | A | A | T | F | C | S | W | D | K | D | N | K | N | Y | H | F | N | L | I | D | 150 | | Kwal\_14.1090/1-763 | 96 | T | G | R | I | K | A | I | H | E | V | R | G | R | D | N | V | G | A | K | M | D | S | M | D | L | E | R | E | K | G | I | T | I | Q | S | A | A | T | Y | C | S | W | D | K | D | G | Q | N | Y | H | F | N | L | I | D | 150 | | Sbay\_672.50/1-761 | 94 | T | K | R | I | K | A | I | H | E | V | R | G | R | D | N | V | G | A | K | M | D | S | M | D | L | E | R | E | K | G | I | T | I | Q | S | A | A | T | Y | C | S | W | D | K | E | G | K | N | Y | H | F | N | L | I | D | 148 | | SAKL0B00858g/1-761 | 95 | T | G | R | I | K | A | I | H | E | V | R | G | R | D | N | V | G | A | K | M | D | S | M | D | L | E | R | E | K | G | I | T | I | Q | S | A | A | T | Y | C | S | W | D | K | D | N | Q | G | Y | H | F | N | L | I | D | 149 | | P25039/1-761 | 94 | T | K | R | I | K | A | I | H | E | V | R | G | R | D | N | V | G | A | K | M | D | S | M | D | L | E | R | E | K | G | I | T | I | Q | S | A | A | T | Y | C | S | W | D | K | E | G | K | N | Y | H | F | N | L | I | D | 148 | |  | | G0VKH7/1-766 | 153 | T | P | G | H | I | D | F | T | I | E | V | E | R | A | L | R | V | L | D | G | A | V | L | V | V | C | A | V | S | G | V | Q | S | Q | T | V | T | V | D | R | Q | M | R | R | Y | N | I | P | R | I | T | F | I | N | K | 207 | | Q6CRY5/1-755 | 144 | T | P | G | H | I | D | F | T | I | E | V | E | R | A | L | R | I | L | D | G | A | V | L | V | V | C | A | V | S | G | V | Q | S | Q | T | V | T | V | D | R | Q | M | R | R | Y | N | V | P | R | V | T | F | I | N | K | 198 | | Q6FUQ6/1-757 | 146 | T | P | G | H | I | D | F | T | I | E | V | E | R | A | L | R | V | L | D | G | A | V | L | V | V | C | A | V | S | G | V | Q | S | Q | T | V | T | V | D | R | Q | M | R | R | Y | N | V | P | R | V | T | F | I | N | K | 200 | | Q75CZ5/1-757 | 147 | T | P | G | H | I | D | F | T | I | E | V | E | R | A | L | R | V | L | D | G | A | V | L | V | V | C | A | V | S | G | V | Q | S | Q | T | V | T | V | D | R | Q | M | R | R | Y | N | V | P | R | V | T | F | I | N | K | 201 | | A7TFN8/1-776 | 165 | T | P | G | H | I | D | F | T | I | E | V | E | R | A | L | R | V | L | D | G | A | V | L | I | V | C | A | V | S | G | V | Q | S | Q | T | V | T | V | D | R | Q | M | R | R | Y | N | V | P | R | I | T | F | I | N | K | 219 | | C5DNQ2/1-763 | 151 | T | P | G | H | I | D | F | T | I | E | V | E | R | A | L | R | V | L | D | G | A | V | L | V | V | C | A | V | S | G | V | Q | S | Q | T | V | T | V | D | R | Q | M | R | R | Y | N | V | P | R | V | T | F | I | N | K | 205 | | C5DX66/1-769 | 151 | T | P | G | H | I | D | F | T | I | E | V | E | R | A | L | R | V | L | D | G | A | V | L | V | V | C | A | V | S | G | V | Q | S | Q | T | V | T | V | D | R | Q | M | R | R | Y | N | I | P | R | I | T | F | I | N | K | 205 | | Kwal\_14.1090/1-763 | 151 | T | P | G | H | I | D | F | T | I | E | V | E | R | A | L | R | V | L | D | G | A | V | L | V | V | C | A | V | S | G | V | Q | S | Q | T | V | T | V | D | R | Q | M | R | R | Y | N | V | P | R | V | T | F | I | N | K | 205 | | Sbay\_672.50/1-761 | 149 | T | P | G | H | I | D | F | T | I | E | V | E | R | A | L | R | V | L | D | G | A | V | L | V | V | C | A | V | S | G | V | Q | S | Q | T | V | T | V | D | R | Q | M | R | R | Y | N | V | P | R | V | T | F | I | N | K | 203 | | SAKL0B00858g/1-761 | 150 | T | P | G | H | I | D | F | T | I | E | V | E | R | A | L | R | V | L | D | G | A | V | L | V | V | C | A | V | S | G | V | Q | S | Q | T | V | T | V | D | R | Q | M | R | R | Y | N | V | P | R | V | T | F | I | N | K | 204 | | P25039/1-761 | 149 | T | P | G | H | I | D | F | T | I | E | V | E | R | A | L | R | V | L | D | G | A | V | L | V | V | C | A | V | S | G | V | Q | S | Q | T | V | T | V | D | R | Q | M | R | R | Y | N | V | P | R | V | T | F | I | N | K | 203 | |  | | G0VKH7/1-766 | 208 | M | D | R | M | G | S | N | P | F | K | A | I | E | Q | L | N | S | K | L | K | I | P | A | A | A | L | Q | V | P | I | G | A | E | S | G | L | Q | G | V | V | D | I | I | N | R | V | A | L | Y | N | K | G | D | S | G | 262 | | Q6CRY5/1-755 | 199 | M | D | R | M | G | A | N | P | F | R | S | I | E | Q | I | N | N | K | L | R | I | P | A | A | A | I | Q | V | P | I | G | A | E | S | E | L | K | G | V | V | N | I | I | D | R | V | A | I | Y | N | E | G | S | N | G | 253 | | Q6FUQ6/1-757 | 201 | M | D | R | M | G | A | N | P | F | K | A | I | E | Q | L | N | S | K | L | K | L | P | A | A | A | V | Q | V | P | I | G | A | E | S | E | L | K | G | V | V | D | L | L | D | M | K | A | Y | Y | N | K | G | D | N | G | 255 | | Q75CZ5/1-757 | 202 | M | D | R | M | G | A | D | P | F | K | A | I | Q | Q | I | N | T | K | L | R | I | P | A | A | A | V | H | V | P | I | G | S | E | S | D | L | C | G | V | V | D | I | I | N | R | V | A | I | Y | N | E | G | E | N | G | 256 | | A7TFN8/1-776 | 220 | M | D | R | M | G | S | N | P | F | K | A | I | E | Q | I | N | S | K | L | Q | I | S | A | A | A | L | Q | V | P | I | G | S | E | S | N | L | R | G | V | V | D | I | I | N | R | V | A | Y | Y | N | K | G | D | Q | G | 274 | | C5DNQ2/1-763 | 206 | M | D | R | M | G | A | N | P | F | R | A | I | E | Q | I | N | K | K | L | K | T | P | A | A | A | I | Q | V | P | I | G | A | E | S | E | L | K | G | V | V | N | I | I | D | R | V | A | L | Y | N | E | G | A | N | G | 260 | | C5DX66/1-769 | 206 | M | D | R | M | G | A | N | P | F | R | A | V | D | Q | I | H | S | K | L | K | I | P | A | A | V | L | Q | V | P | I | G | L | E | S | D | L | E | G | V | V | D | I | I | N | R | V | A | L | Y | N | K | G | E | H | G | 260 | | Kwal\_14.1090/1-763 | 206 | M | D | R | M | G | A | N | P | F | R | A | I | E | Q | I | N | K | K | L | K | T | P | A | A | A | I | Q | V | P | I | G | S | E | S | E | L | K | G | V | V | N | I | I | D | R | V | A | L | Y | N | E | G | A | K | G | 260 | | Sbay\_672.50/1-761 | 204 | M | D | R | M | G | S | D | P | F | R | A | I | E | Q | L | N | S | K | L | K | I | P | A | A | A | V | Q | I | P | I | G | S | E | S | T | L | S | G | V | V | D | L | I | N | K | V | A | L | Y | N | K | G | D | N | G | 258 | | SAKL0B00858g/1-761 | 205 | M | D | R | M | G | A | N | P | F | K | A | I | E | Q | I | N | S | K | L | K | T | P | A | A | A | I | Q | V | P | I | G | A | E | S | E | L | K | G | A | V | N | I | I | D | R | V | A | L | Y | N | E | G | E | N | G | 259 | | P25039/1-761 | 204 | M | D | R | M | G | S | D | P | F | R | A | I | E | Q | L | N | S | K | L | K | I | P | A | A | A | V | Q | I | P | I | G | S | E | S | S | L | S | G | V | V | D | L | I | N | R | V | A | I | Y | N | K | G | D | N | G | 258 | |  | | G0VKH7/1-766 | 263 | E | I | I | E | K | G | E | V | P | K | D | L | V | D | L | V | E | E | K | R | Q | L | L | I | D | T | L | A | D | V | D | D | E | M | T | E | L | F | L | E | E | K | Q | P | T | V | E | Q | I | K | S | A | I | R | R | 317 | | Q6CRY5/1-755 | 254 | E | K | L | V | T | G | P | V | P | E | D | L | K | D | L | V | E | E | K | R | A | L | L | I | E | T | L | A | D | V | D | D | E | I | A | E | I | F | L | E | E | K | E | P | S | V | D | E | I | K | A | A | I | R | R | 308 | | Q6FUQ6/1-757 | 256 | E | I | I | E | S | G | P | I | P | E | E | L | K | S | L | A | E | E | K | R | Q | V | L | I | E | T | L | A | D | V | D | E | H | M | A | E | I | F | L | E | E | K | E | P | T | I | Q | E | M | K | D | A | I | R | R | 310 | | Q75CZ5/1-757 | 257 | E | V | L | R | K | G | P | V | P | E | E | L | Q | D | L | V | E | E | K | R | L | L | L | V | E | T | L | A | D | V | D | D | E | M | A | E | I | F | L | D | E | Q | E | P | T | V | Q | Q | I | K | D | A | I | R | R | 311 | | A7TFN8/1-776 | 275 | E | I | I | D | K | A | E | V | P | E | D | L | K | D | L | V | E | E | K | R | A | L | L | I | E | K | L | A | D | V | D | D | E | I | A | E | L | F | L | E | E | Q | E | P | T | V | E | Q | I | K | S | A | I | R | R | 329 | | C5DNQ2/1-763 | 261 | E | Q | I | V | E | G | P | V | P | N | E | L | N | E | L | V | E | E | R | R | A | M | L | I | E | T | L | A | D | V | D | D | E | I | A | E | I | F | L | E | E | Q | E | P | S | T | E | Q | I | K | A | A | I | R | R | 315 | | C5DX66/1-769 | 261 | E | E | L | V | P | G | P | V | P | E | N | L | K | D | V | V | E | E | R | R | Q | I | L | V | E | T | L | A | D | V | D | D | E | M | A | E | L | F | L | E | E | Q | E | P | N | V | D | Q | I | K | A | A | I | R | R | 315 | | Kwal\_14.1090/1-763 | 261 | E | Q | I | V | K | G | P | V | P | P | E | L | N | S | L | V | E | E | R | R | A | M | L | I | E | T | L | A | D | V | D | D | E | I | A | E | L | F | L | E | E | Q | E | P | S | V | D | Q | I | K | A | A | I | R | R | 315 | | Sbay\_672.50/1-761 | 259 | E | I | I | E | K | G | P | V | P | E | E | L | K | P | L | M | E | E | K | R | Q | L | L | I | E | T | L | A | D | V | D | D | E | M | A | E | M | F | L | E | E | K | E | P | T | I | E | Q | I | K | A | A | I | R | R | 313 | | SAKL0B00858g/1-761 | 260 | E | I | I | V | S | R | P | V | P | E | E | L | K | E | L | V | E | E | K | R | A | L | L | I | E | T | L | A | D | V | D | D | E | I | A | E | L | Y | L | D | E | K | E | P | S | V | Q | Q | I | K | D | A | I | R | R | 314 | | P25039/1-761 | 259 | E | I | I | E | K | G | P | V | P | E | N | L | K | P | L | M | E | E | K | R | Q | L | L | I | E | T | L | A | D | V | D | D | E | M | A | E | M | F | L | E | E | K | E | P | T | T | Q | Q | I | K | D | A | I | R | R | 313 | |  | | G0VKH7/1-766 | 318 | A | T | I | A | R | K | F | T | P | V | L | M | G | S | A | L | A | N | M | G | I | Q | P | V | L | D | A | I | V | D | Y | L | P | N | P | S | E | V | L | N | T | A | L | D | V | A | K | D | E | A | K | V | N | L | V | 372 | | Q6CRY5/1-755 | 309 | A | T | I | A | R | K | F | S | P | V | L | M | G | S | A | L | A | N | T | G | I | Q | N | V | L | D | A | I | V | E | Y | L | P | N | P | S | E | V | L | N | T | G | L | D | I | A | K | D | E | T | K | V | N | L | I | 363 | | Q6FUQ6/1-757 | 311 | A | T | I | A | R | K | F | T | P | V | L | M | G | S | A | L | A | N | T | G | V | Q | H | V | L | D | A | I | V | D | Y | L | P | N | P | S | E | V | L | N | T | G | L | D | I | A | H | E | E | A | K | V | N | L | I | 365 | | Q75CZ5/1-757 | 312 | A | T | I | A | R | K | F | T | P | V | L | M | G | S | A | L | A | N | T | G | I | Q | N | V | L | D | A | I | V | D | Y | L | P | E | P | S | E | V | L | N | T | A | L | D | V | S | N | N | E | T | K | V | N | L | I | 366 | | A7TFN8/1-776 | 330 | A | T | I | A | R | K | F | T | P | V | M | M | G | T | A | L | G | N | T | G | I | Q | H | V | L | D | A | I | V | D | Y | L | P | N | P | S | E | V | L | N | T | G | L | D | L | S | K | N | E | A | K | V | P | L | V | 384 | | C5DNQ2/1-763 | 316 | A | T | I | A | R | K | F | T | P | V | L | M | G | S | A | L | A | N | R | S | V | Q | P | V | L | D | A | I | V | D | Y | L | P | N | P | S | E | I | L | N | T | G | L | D | I | A | N | N | E | A | K | V | N | L | V | 370 | | C5DX66/1-769 | 316 | A | T | I | A | R | K | F | S | P | V | L | M | G | S | A | L | A | N | T | G | I | Q | P | V | L | D | A | V | V | D | Y | L | P | N | P | S | Q | V | L | N | T | A | L | D | V | A | N | N | E | A | K | V | N | L | V | 370 | | Kwal\_14.1090/1-763 | 316 | A | T | I | A | R | K | F | T | P | V | L | M | G | S | A | L | A | N | R | S | V | Q | P | V | L | D | A | I | V | D | Y | L | P | N | P | S | E | V | L | N | T | G | L | D | I | S | K | G | E | A | K | V | N | L | V | 370 | | Sbay\_672.50/1-761 | 314 | A | T | I | A | R | T | F | T | P | V | L | M | G | S | A | L | A | N | T | G | I | Q | P | V | L | D | A | I | V | D | F | L | P | N | P | S | E | V | L | N | T | A | L | D | V | S | N | D | E | T | T | I | N | L | V | 368 | | SAKL0B00858g/1-761 | 315 | A | T | I | A | R | K | F | T | P | V | L | M | G | S | A | L | A | N | T | G | I | Q | S | V | L | D | A | V | V | D | Y | L | P | N | P | S | E | V | L | N | T | G | L | D | I | A | N | N | E | T | K | V | N | L | I | 369 | | P25039/1-761 | 314 | S | T | I | A | R | S | F | T | P | V | L | M | G | S | A | L | A | N | T | G | I | Q | P | V | L | D | A | I | V | D | Y | L | P | N | P | S | E | V | L | N | T | A | L | D | V | S | N | N | E | A | K | V | N | L | V | 368 | |  | | G0VKH7/1-766 | 373 | P | S | A | Q | K | P | F | V | G | L | A | F | K | L | E | E | G | K | Y | G | Q | L | T | Y | I | R | V | Y | Q | G | R | L | K | K | G | N | Y | I | T | N | V | K | T | G | K | K | V | K | V | S | R | L | V | R | M | 427 | | Q6CRY5/1-755 | 364 | P | S | S | T | Q | P | F | V | G | L | A | F | K | L | E | E | G | K | Y | G | Q | L | T | Y | I | R | V | Y | Q | G | K | M | R | K | G | G | Y | I | T | N | V | K | T | G | K | K | V | K | I | S | R | L | V | R | M | 418 | | Q6FUQ6/1-757 | 366 | P | S | V | Q | Q | P | F | V | G | L | A | F | K | L | E | E | G | K | Y | G | Q | L | T | Y | I | R | V | Y | Q | G | R | L | K | K | G | S | Y | I | T | N | V | K | T | G | K | K | V | K | V | S | R | L | V | R | M | 420 | | Q75CZ5/1-757 | 367 | P | S | S | H | H | P | F | V | G | L | A | F | K | L | E | E | G | N | Y | G | Q | L | T | Y | I | R | V | Y | Q | G | K | L | K | K | G | G | Y | I | T | N | V | R | T | G | K | K | V | K | V | S | R | L | V | R | M | 421 | | A7TFN8/1-776 | 385 | P | S | I | Q | Q | P | F | V | G | L | A | F | K | L | E | E | G | K | Y | G | Q | L | T | Y | I | R | V | Y | Q | G | R | L | R | K | G | N | Y | I | T | N | I | K | T | G | K | K | V | K | V | S | R | L | V | R | M | 439 | | C5DNQ2/1-763 | 371 | P | S | V | Q | E | P | F | V | G | L | A | F | K | L | E | E | G | K | Y | G | Q | L | T | Y | I | R | V | Y | Q | G | R | L | R | K | G | G | Y | I | T | N | V | K | T | G | K | K | I | K | V | S | R | L | V | R | M | 425 | | C5DX66/1-769 | 371 | P | S | V | T | K | P | F | L | G | L | A | F | K | L | E | E | G | Q | Y | G | Q | L | T | Y | I | R | V | Y | Q | G | R | L | R | K | G | T | Y | I | T | N | V | K | S | G | K | K | I | K | V | S | R | L | V | R | M | 425 | | Kwal\_14.1090/1-763 | 371 | P | S | I | Q | E | P | F | V | G | L | A | F | K | L | E | E | G | K | Y | G | Q | L | T | Y | I | R | V | Y | Q | G | R | L | R | K | G | G | Y | I | T | N | V | K | N | K | K | K | I | K | V | S | R | L | V | R | M | 425 | | Sbay\_672.50/1-761 | 369 | P | A | V | Q | Q | P | F | V | G | L | A | F | K | L | E | E | G | K | Y | G | Q | L | T | Y | V | R | V | Y | Q | G | R | L | R | K | G | N | Y | I | T | N | V | K | T | G | K | K | V | K | V | A | R | L | V | R | M | 423 | | SAKL0B00858g/1-761 | 370 | P | S | I | Q | Q | P | F | V | G | L | A | F | K | L | E | E | G | K | Y | G | Q | L | T | Y | I | R | V | Y | Q | G | R | L | R | K | G | G | Y | I | T | N | V | K | T | G | K | K | V | K | V | S | R | L | V | R | M | 424 | | P25039/1-761 | 369 | P | A | V | Q | Q | P | F | V | G | L | A | F | K | L | E | E | G | K | Y | G | Q | L | T | Y | V | R | V | Y | Q | G | R | L | R | K | G | N | Y | I | T | N | V | K | T | G | K | K | V | K | V | A | R | L | V | R | M | 423 | |  | | G0VKH7/1-766 | 428 | H | S | N | D | M | E | D | V | D | E | V | G | S | G | E | I | C | A | T | F | G | I | D | C | S | S | G | D | T | F | T | D | G | T | V | E | Y | S | M | S | S | M | Y | V | P | D | A | V | V | S | L | S | I | S | P | 482 | | Q6CRY5/1-755 | 419 | H | S | N | D | M | E | D | V | D | E | V | G | A | G | E | I | C | A | T | F | G | I | D | C | S | S | G | D | T | F | T | D | G | T | L | K | Y | S | M | S | S | M | F | V | P | D | A | V | I | S | L | S | I | T | P | 473 | | Q6FUQ6/1-757 | 421 | H | S | N | E | M | E | D | V | D | E | V | G | S | G | E | I | C | A | T | F | G | I | D | C | S | S | G | D | T | F | S | D | G | T | L | Q | Y | S | M | S | S | M | F | V | P | D | A | V | V | S | L | S | I | T | P | 475 | | Q75CZ5/1-757 | 422 | H | S | N | E | M | E | D | V | N | E | I | G | A | G | E | I | C | A | T | F | G | I | D | C | S | S | G | D | T | F | T | D | G | K | L | K | Y | S | M | S | S | M | Y | V | P | D | A | V | I | S | L | S | I | S | P | 476 | | A7TFN8/1-776 | 440 | H | S | E | E | M | E | D | V | D | E | I | G | S | G | E | I | C | A | T | F | G | I | D | C | S | S | G | D | T | F | T | D | G | N | V | K | Y | S | M | S | S | M | Y | V | P | D | A | V | V | S | L | S | I | K | P | 494 | | C5DNQ2/1-763 | 426 | H | S | N | D | M | E | D | V | D | E | V | G | S | G | E | I | C | A | T | F | G | I | D | C | A | S | G | D | T | F | T | D | G | T | L | Q | Y | S | M | S | S | M | Y | V | P | D | A | V | I | S | L | S | I | T | P | 480 | | C5DX66/1-769 | 426 | H | S | N | D | M | E | D | V | D | E | V | G | S | G | E | I | C | A | T | F | G | I | D | C | A | S | G | D | T | F | C | D | G | S | V | Q | Y | S | M | S | S | M | Y | V | P | D | A | V | V | S | L | S | V | T | P | 480 | | Kwal\_14.1090/1-763 | 426 | H | S | N | D | M | E | D | V | D | E | V | G | S | G | E | I | C | A | T | F | G | I | D | C | A | S | G | D | T | F | T | D | G | T | L | E | Y | S | M | S | S | M | Y | V | P | D | A | V | I | S | L | S | I | T | P | 480 | | Sbay\_672.50/1-761 | 424 | H | S | N | E | M | E | D | V | D | E | V | G | S | G | E | I | C | A | T | F | G | I | D | C | A | S | G | D | T | F | T | D | G | S | V | Q | Y | S | M | S | S | M | Y | V | P | D | A | V | V | S | L | S | I | T | P | 478 | | SAKL0B00858g/1-761 | 425 | H | S | S | E | M | E | D | V | D | E | V | G | S | G | E | I | C | A | T | F | G | I | D | C | A | S | G | D | T | F | T | D | G | T | L | N | Y | S | M | S | S | M | Y | V | P | D | A | V | I | S | L | S | I | T | P | 479 | | P25039/1-761 | 424 | H | S | S | E | M | E | D | V | D | E | V | G | S | G | E | I | C | A | T | F | G | I | D | C | A | S | G | D | T | F | T | D | G | S | V | Q | Y | S | M | S | S | M | Y | V | P | D | A | V | V | S | L | S | I | T | P | 478 | |  | | G0VKH7/1-766 | 483 | K | G | K | D | V | - | P | N | F | S | K | A | L | N | R | F | Q | K | E | D | P | T | F | R | V | R | F | D | P | E | S | K | E | T | I | I | S | G | M | G | E | L | H | L | E | I | Y | V | E | R | M | K | R | E | Y | 536 | | Q6CRY5/1-755 | 474 | K | S | K | D | S | - | T | N | F | S | K | A | L | N | R | F | Q | K | E | D | P | T | F | R | V | R | F | D | P | E | S | K | E | T | V | I | S | G | M | G | E | L | H | L | E | I | Y | V | E | R | M | R | R | E | Y | 527 | | Q6FUQ6/1-757 | 476 | K | S | K | D | S | - | T | N | F | S | K | A | L | N | R | F | Q | K | E | D | P | T | F | R | V | R | F | D | P | E | S | K | E | T | V | I | S | G | M | G | E | L | H | L | E | I | Y | V | E | R | M | K | R | E | Y | 529 | | Q75CZ5/1-757 | 477 | N | S | K | D | S | A | T | N | F | S | K | A | L | N | R | F | Q | K | E | D | P | T | F | R | V | R | F | D | P | E | S | K | Q | T | I | I | S | G | M | G | E | L | H | L | E | I | Y | V | E | R | M | R | R | E | Y | 531 | | A7TFN8/1-776 | 495 | K | S | K | D | S | - | T | N | F | S | K | A | F | N | R | F | Q | K | E | D | P | T | F | R | V | K | F | D | P | E | S | K | E | T | V | I | S | G | M | G | E | L | H | L | E | I | Y | V | E | R | M | K | R | E | Y | 548 | | C5DNQ2/1-763 | 481 | T | S | R | D | S | - | T | N | F | S | K | A | L | N | R | F | Q | K | E | D | P | T | F | R | V | R | F | D | A | E | S | K | E | T | V | I | S | G | M | G | E | L | H | L | E | I | Y | V | E | R | M | R | R | E | Y | 534 | | C5DX66/1-769 | 481 | N | S | K | D | A | - | G | N | F | S | K | A | L | N | R | F | Q | K | E | D | P | T | F | R | V | K | F | D | P | E | S | K | Q | T | I | V | S | G | M | G | E | L | H | L | E | I | Y | V | E | R | M | R | R | E | Y | 534 | | Kwal\_14.1090/1-763 | 481 | T | S | R | D | S | - | T | N | F | S | K | A | L | N | R | F | Q | K | E | D | P | T | F | R | V | R | F | D | A | E | S | K | E | T | V | I | S | G | M | G | E | L | H | L | E | I | Y | V | E | R | M | R | R | E | Y | 534 | | Sbay\_672.50/1-761 | 479 | K | S | T | D | V | - | S | N | F | S | K | A | L | N | R | F | Q | K | E | D | P | T | F | R | V | K | F | D | P | E | S | K | E | T | I | I | S | G | M | G | E | L | H | L | E | I | Y | V | E | R | M | R | R | E | Y | 532 | | SAKL0B00858g/1-761 | 480 | K | T | K | D | S | - | T | N | F | S | K | A | L | N | R | F | Q | K | E | D | P | T | F | R | V | R | F | D | P | E | S | K | E | T | V | I | S | G | M | G | E | L | H | L | E | I | Y | V | E | R | M | K | R | E | Y | 533 | | P25039/1-761 | 479 | N | S | K | D | A | - | S | N | F | S | K | A | L | N | R | F | Q | K | E | D | P | T | F | R | V | K | F | D | P | E | S | K | E | T | I | I | S | G | M | G | E | L | H | L | E | I | Y | V | E | R | M | R | R | E | Y | 532 | |  | | G0VKH7/1-766 | 537 | N | V | E | C | I | T | G | K | P | Q | V | S | Y | R | E | S | I | T | I | P | A | E | F | D | Y | T | H | K | K | Q | S | G | G | A | G | Q | Y | G | R | V | I | G | T | L | S | P | A | - | E | E | G | G | K | N | N | 590 | | Q6CRY5/1-755 | 528 | N | V | E | C | V | T | G | K | P | Q | V | S | Y | R | E | S | I | Q | S | S | A | E | F | D | Y | T | H | K | K | Q | S | G | G | A | G | Q | Y | G | R | V | M | G | N | L | S | H | I | - | E | N | - | S | N | T | N | 580 | | Q6FUQ6/1-757 | 530 | N | V | E | C | I | T | G | K | P | Q | V | S | Y | R | E | S | I | T | I | P | S | E | F | D | Y | T | H | K | K | Q | S | G | G | A | G | Q | Y | A | R | I | I | G | D | L | S | P | V | - | E | G | G | N | K | S | N | 583 | | Q75CZ5/1-757 | 532 | N | V | A | C | T | T | G | K | P | Q | V | S | Y | R | E | S | I | Q | I | P | A | T | F | D | Y | T | H | K | K | Q | S | G | G | A | G | Q | Y | A | R | V | M | G | N | L | T | P | V | - | A | N | - | S | S | E | N | 584 | | A7TFN8/1-776 | 549 | N | V | E | C | E | T | G | K | P | Q | V | S | Y | R | E | S | I | T | I | P | A | E | F | D | Y | T | H | K | K | Q | S | G | G | A | G | Q | F | G | R | V | I | G | T | M | S | P | V | - | E | G | E | T | R | D | N | 602 | | C5DNQ2/1-763 | 535 | N | V | D | C | V | T | G | K | P | Q | V | S | Y | R | E | S | I | T | I | P | A | E | F | D | Y | T | H | K | K | Q | S | G | G | A | G | Q | Y | G | R | V | M | G | S | L | S | P | L | - | E | G | - | S | N | G | N | 587 | | C5DX66/1-769 | 535 | N | V | E | C | T | T | G | K | P | Q | V | S | Y | R | E | S | V | T | I | P | A | E | F | D | Y | T | H | K | K | Q | S | G | G | A | G | Q | Y | G | R | V | I | G | T | L | A | P | A | P | E | G | N | S | N | S | N | 589 | | Kwal\_14.1090/1-763 | 535 | N | V | D | C | V | T | G | K | P | Q | V | S | Y | R | E | S | I | T | M | P | A | E | F | D | Y | T | H | K | K | Q | S | G | G | A | G | Q | Y | G | R | V | M | G | T | L | T | P | L | - | E | G | - | S | N | G | N | 587 | | Sbay\_672.50/1-761 | 533 | N | V | E | C | V | T | G | K | P | Q | V | S | Y | R | E | S | I | T | I | P | A | D | F | D | Y | T | H | K | K | Q | S | G | G | A | G | Q | Y | G | R | V | I | G | T | L | S | P | V | - | E | D | I | T | K | G | N | 586 | | SAKL0B00858g/1-761 | 534 | N | V | E | C | I | T | G | K | P | Q | V | S | Y | R | E | S | I | T | I | P | A | E | F | D | Y | T | H | K | K | Q | S | G | G | A | G | Q | Y | G | R | V | M | G | N | L | T | P | I | - | E | G | - | S | N | S | N | 586 | | P25039/1-761 | 533 | N | V | D | C | V | T | G | K | P | Q | V | S | Y | R | E | S | I | T | I | P | A | D | F | D | Y | T | H | K | K | Q | S | G | G | A | G | Q | Y | G | R | V | I | G | T | L | S | P | V | - | D | D | I | T | K | G | N | 586 | |  | | G0VKH7/1-766 | 591 | V | F | E | T | A | I | V | G | G | R | I | P | E | K | Y | L | A | A | C | S | K | G | F | E | D | A | C | E | K | G | P | L | I | G | H | K | V | L | N | V | K | M | L | I | N | D | G | A | I | H | A | V | D | S | N | 645 | | Q6CRY5/1-755 | 581 | N | F | E | T | A | I | V | G | G | R | I | P | D | K | Y | L | A | A | C | A | K | G | F | E | E | A | C | E | K | G | P | L | I | G | H | R | V | L | G | V | N | M | L | I | N | D | G | A | I | H | A | V | D | S | N | 635 | | Q6FUQ6/1-757 | 584 | V | F | E | T | H | V | V | G | G | R | I | P | D | K | Y | L | S | A | C | A | K | G | F | D | E | A | C | E | R | G | P | L | I | G | H | K | V | L | N | V | K | M | L | I | N | D | G | A | I | H | S | V | D | S | N | 638 | | Q75CZ5/1-757 | 585 | T | F | T | T | A | V | V | G | G | R | I | P | D | K | Y | L | A | A | C | A | K | G | F | E | E | V | C | E | K | G | P | L | I | G | H | K | V | L | G | I | N | M | L | I | N | D | G | A | I | H | A | V | D | S | N | 639 | | A7TFN8/1-776 | 603 | L | F | E | T | A | I | V | G | G | R | I | P | D | K | Y | L | A | A | C | G | K | G | F | E | E | A | C | E | K | G | P | L | V | G | H | K | V | L | G | V | K | M | L | I | N | D | G | A | I | H | A | V | D | S | N | 657 | | C5DNQ2/1-763 | 588 | K | F | E | T | A | I | V | G | G | R | I | P | D | K | Y | L | A | A | C | G | K | G | F | E | E | A | C | E | K | G | P | L | I | G | H | K | V | L | S | T | H | M | L | I | N | D | G | A | I | H | A | V | D | S | N | 642 | | C5DX66/1-769 | 590 | S | F | E | T | A | I | V | G | G | R | I | P | D | K | Y | L | A | A | C | G | K | G | F | D | E | A | C | E | K | G | P | L | I | G | H | R | V | L | G | V | D | M | L | I | N | D | G | A | I | H | A | V | D | S | N | 644 | | Kwal\_14.1090/1-763 | 588 | K | F | E | T | A | I | V | G | G | R | I | P | D | K | Y | L | A | A | C | G | K | G | F | E | E | A | C | E | K | G | P | L | I | G | H | K | V | L | S | A | H | M | L | I | N | D | G | A | I | H | A | V | D | S | N | 642 | | Sbay\_672.50/1-761 | 587 | I | F | E | T | A | I | V | G | G | R | I | P | D | K | Y | L | A | A | C | A | K | G | F | E | E | V | C | E | K | G | P | L | I | G | H | R | V | L | K | V | E | M | L | I | N | D | G | A | I | H | A | V | D | S | N | 641 | | SAKL0B00858g/1-761 | 587 | K | F | E | T | A | I | V | G | G | R | I | P | D | K | Y | L | A | A | C | A | K | G | F | E | D | S | C | E | K | G | P | L | I | G | H | R | V | L | G | V | N | M | L | I | N | D | G | A | I | H | A | V | D | S | N | 641 | | P25039/1-761 | 587 | I | F | E | T | A | I | V | G | G | R | I | P | D | K | Y | L | A | A | C | G | K | G | F | E | E | V | C | E | K | G | P | L | I | G | H | R | V | L | D | V | K | M | L | I | N | D | G | A | I | H | A | V | D | S | N | 641 | |  | | G0VKH7/1-766 | 646 | E | L | S | F | K | T | A | T | M | G | A | F | R | E | A | F | L | K | A | E | P | V | I | L | E | P | I | M | T | V | T | V | T | S | P | N | E | F | Q | G | N | V | I | G | L | L | N | K | L | Q | A | V | I | Q | D | 700 | | Q6CRY5/1-755 | 636 | E | L | A | F | K | T | A | T | M | A | A | F | R | Q | A | F | L | E | S | Q | P | V | I | L | E | P | I | M | N | V | S | V | T | S | P | N | E | F | Q | G | N | V | I | G | L | M | N | K | L | Q | A | V | I | Q | D | 690 | | Q6FUQ6/1-757 | 639 | E | L | A | F | K | V | A | T | L | T | A | F | R | D | A | F | L | K | A | Q | P | V | I | M | E | P | I | M | I | V | S | V | T | S | P | N | E | F | Q | G | N | V | I | G | L | L | N | K | L | Q | A | V | I | Q | E | 693 | | Q75CZ5/1-757 | 640 | E | M | A | F | K | T | A | T | T | A | A | F | V | Q | S | F | M | Q | A | Q | P | V | V | L | E | P | I | M | T | V | T | V | T | A | P | N | E | F | Q | G | N | V | I | T | L | L | N | K | L | Q | A | V | I | Q | D | 694 | | A7TFN8/1-776 | 658 | E | L | S | F | K | T | A | T | M | A | A | F | R | D | A | F | L | K | S | Q | P | V | V | L | E | P | I | M | N | V | S | V | T | S | P | N | E | F | Q | G | N | V | I | G | L | L | N | K | L | Q | A | V | I | Q | D | 712 | | C5DNQ2/1-763 | 643 | E | L | A | F | K | T | A | T | M | A | A | F | K | E | A | F | L | E | A | K | P | V | I | L | E | P | V | M | N | V | S | V | T | A | P | N | E | F | Q | G | N | V | I | S | L | L | N | K | L | Q | A | V | I | Q | D | 697 | | C5DX66/1-769 | 645 | E | L | S | F | K | T | A | T | M | A | A | F | R | D | S | F | L | K | A | Q | P | V | I | L | E | P | I | M | V | V | T | V | T | S | P | N | E | F | Q | G | N | V | I | G | T | L | N | K | L | Q | A | M | I | Q | D | 699 | | Kwal\_14.1090/1-763 | 643 | E | L | A | F | K | T | A | T | M | A | A | F | K | E | A | F | L | G | A | K | P | V | I | L | E | P | I | M | N | V | S | V | T | A | P | N | E | F | Q | G | N | V | I | S | L | M | N | K | L | Q | A | V | I | Q | D | 697 | | Sbay\_672.50/1-761 | 642 | E | L | S | F | K | T | A | T | M | S | A | F | R | D | A | F | L | R | A | Q | P | V | I | M | E | P | I | M | N | V | S | V | T | S | P | N | E | F | Q | G | N | V | I | G | L | L | N | K | L | Q | A | V | I | Q | D | 696 | | SAKL0B00858g/1-761 | 642 | E | L | A | F | K | T | A | T | M | A | A | F | R | Q | A | F | L | N | A | Q | P | V | I | L | E | P | I | M | N | V | S | V | T | S | P | N | E | F | Q | G | N | I | I | G | L | L | N | K | L | Q | A | V | I | Q | D | 696 | | P25039/1-761 | 642 | E | L | S | F | K | T | A | T | M | S | A | F | R | D | A | F | L | R | A | Q | P | V | I | M | E | P | I | M | N | V | S | V | T | S | P | N | E | F | Q | G | N | V | I | G | L | L | N | K | L | Q | A | V | I | Q | D | 696 | |  | | G0VKH7/1-766 | 701 | T | D | N | G | H | D | E | F | T | L | R | A | E | C | T | L | S | T | M | F | G | F | A | T | S | L | R | A | S | T | Q | G | K | G | E | F | S | L | E | F | N | R | Y | A | P | T | S | P | H | V | Q | K | Q | M | I | 755 | | Q6CRY5/1-755 | 691 | T | E | N | G | Q | D | E | F | T | I | T | A | E | C | P | L | N | T | M | F | G | F | A | T | S | L | R | A | S | T | Q | G | K | G | E | F | S | L | E | F | K | H | Y | A | P | A | S | P | Q | L | Q | K | Q | L | I | 745 | | Q6FUQ6/1-757 | 694 | T | D | N | G | H | D | E | F | T | L | R | A | E | C | S | L | S | T | M | F | G | F | A | S | S | L | R | A | S | T | Q | G | K | G | E | F | S | L | E | F | S | H | Y | A | P | T | A | P | H | V | Q | K | E | L | I | 748 | | Q75CZ5/1-757 | 695 | T | E | N | G | H | D | E | F | T | M | T | S | E | C | S | L | N | T | M | F | C | F | A | T | S | L | R | A | S | T | Q | G | K | G | E | F | S | L | E | F | K | Q | Y | S | P | A | S | P | Q | L | Q | K | Q | L | I | 749 | | A7TFN8/1-776 | 713 | T | E | N | G | H | D | E | F | T | V | K | A | E | C | S | L | S | T | M | F | G | F | A | T | S | L | R | A | S | T | Q | G | K | G | E | F | S | L | E | F | S | H | Y | A | P | T | P | P | H | V | Q | K | E | L | M | 767 | | C5DNQ2/1-763 | 698 | T | E | N | A | Q | D | E | F | T | I | N | A | E | C | S | L | N | T | L | F | G | F | A | T | S | L | R | S | S | T | Q | G | K | G | E | F | S | L | E | F | K | H | Y | S | P | T | S | P | H | L | Q | R | Q | L | I | 752 | | C5DX66/1-769 | 700 | T | E | N | G | Q | D | E | F | T | M | K | A | E | C | P | L | S | N | M | F | G | Y | A | T | S | L | R | A | S | T | Q | G | K | G | E | F | T | L | E | F | S | H | Y | A | P | T | A | P | N | V | Q | R | D | L | I | 754 | | Kwal\_14.1090/1-763 | 698 | T | E | N | A | Q | D | E | F | T | I | T | A | E | C | S | L | N | T | L | F | G | F | A | T | S | L | R | S | S | T | Q | G | K | G | E | F | S | L | E | F | K | H | Y | A | P | C | S | P | H | L | Q | K | Q | L | I | 752 | | Sbay\_672.50/1-761 | 697 | T | E | N | G | H | D | E | F | T | L | K | A | E | C | A | L | S | T | M | F | G | F | A | T | S | L | R | A | S | T | Q | G | K | G | E | F | S | L | E | F | S | H | Y | A | P | T | A | P | H | V | Q | K | E | L | I | 751 | | SAKL0B00858g/1-761 | 697 | T | E | N | G | Q | D | E | F | T | I | T | A | E | C | S | L | N | S | M | F | G | F | A | T | S | L | R | A | S | T | Q | G | K | G | E | F | S | L | E | F | K | H | Y | S | P | A | S | P | H | L | Q | R | Q | L | I | 751 | | P25039/1-761 | 697 | T | E | N | G | H | D | E | F | T | L | K | A | E | C | A | L | S | T | M | F | G | F | A | T | S | L | R | A | S | T | Q | G | K | G | E | F | S | L | E | F | S | H | Y | A | P | T | A | P | H | V | Q | K | E | L | I | 751 | |  | | G0VKH7/1-766 | 756 | Q | E | F | K | E | K | N | L | K | K | K | - | - | - | - |  | | | | | | | | | | | | | | | | | | | | | | | | | | | | | | | | | | | | | | | | 766 | | Q6CRY5/1-755 | 746 | A | D | Y | Q | K | K | Q | Q | Q | K | - | - | - | - | - |  | | | | | | | | | | | | | | | | | | | | | | | | | | | | | | | | | | | | | | | | 755 | | Q6FUQ6/1-757 | 749 | A | E | F | Q | K | K | Q | K | K | - | - | - | - | - | - |  | | | | | | | | | | | | | | | | | | | | | | | | | | | | | | | | | | | | | | | | 757 | | Q75CZ5/1-757 | 750 | E | E | Y | R | K | S | K | K | - | - | - | - | - | - | - |  | | | | | | | | | | | | | | | | | | | | | | | | | | | | | | | | | | | | | | | | 757 | | A7TFN8/1-776 | 768 | A | E | Y | A | K | K | Q | K | K | - | - | - | - | - | - |  | | | | | | | | | | | | | | | | | | | | | | | | | | | | | | | | | | | | | | | | 776 | | C5DNQ2/1-763 | 753 | A | D | F | E | K | K | Q | Q | Q | K | K | - | - | - | - |  | | | | | | | | | | | | | | | | | | | | | | | | | | | | | | | | | | | | | | | | 763 | | C5DX66/1-769 | 755 | A | E | F | Q | K | R | Q | Q | Q | Q | Q | Q | Q | K | K |  | | | | | | | | | | | | | | | | | | | | | | | | | | | | | | | | | | | | | | | | 769 | | Kwal\_14.1090/1-763 | 753 | A | D | F | E | K | K | Q | Q | Q | K | K | - | - | - | - |  | | | | | | | | | | | | | | | | | | | | | | | | | | | | | | | | | | | | | | | | 763 | | Sbay\_672.50/1-761 | 752 | S | E | F | Q | K | K | Q | L | K | K | - | - | - | - | - |  | | | | | | | | | | | | | | | | | | | | | | | | | | | | | | | | | | | | | | | | 761 | | SAKL0B00858g/1-761 | 752 | A | E | Y | E | K | K | Q | Q | K | K | - | - | - | - | - |  | | | | | | | | | | | | | | | | | | | | | | | | | | | | | | | | | | | | | | | | 761 | | P25039/1-761 | 752 | S | E | F | Q | K | K | Q | A | K | K | - | - | - | - | - |  | | | | | | | | | | | | | | | | | | | | | | | | | | | | | | | | | | | | | | | | 761 | |
