# Supplementary material for: Plus ça change – evolutionary sequence divergence predicts protein subcellular localization signals
Source: BMC Genomics. 2014 Jan 20;15:46. doi: 10.1186/1471-2164-15-46 (PMC3906766; doi:10.1186/1471-2164-15-46)
Supplement: Additional file 2 — MSA’s of proteins for which sequence divergence changes predicted localization signals. Contains links to ortholog multiple sequence alignments of each protein in Additional file 3: Table S1. [file 1471-2164-15-46-S2.zip › P25348.html]

|  |  |  |  |  |  |  |  |  |  |  |  |  |  |  |  |  |  |  |  |  |  |  |  |  |  |  |  |  |  |  |  |  |  |  |  |  |  |  |  |  |  |  |  |  |  |  |  |  |  |  |  |  |  |  |  |  |  |  |  |  |  |  |  |  |  |  |  |  |  |  |  |  |  |  |  |  |  |  |  |  |  |  |  |  |  |  |  |  |  |  |  |  |  |  |  |  |  |  |  |  |  |  |  |  |  |  |  |  |  |  |  |  |  |  |  |  |  |  |  |  |  |  |  |  |  |  |  |  |  |  |  |  |  |  |  |  |  |  |  |  |  |  |  |  |  |  |  |  |  |  |  |  |  |  |  |  |  |  |  |  |  |  |  |  |  |  |  |  |  |  |  |  |  |  |  |  |  |  |  |  |  |  |  |  |  |  |  |  |  |  |  |  |  |  |  |  |  |  |  |  |  |  |  |  |  |  |  |  |  |  |  |  |  |  |  |  |  |  |  |  |  |  |  |  |  |  |  |  |  |  |  |  |  |  |  |  |  |  |  |  |  |  |  |  |  |  |  |  |  |  |  |  |  |  |  |  |  |  |  |  |  |  |  |  |  |  |  |  |  |  |  |  |  |  |  |  |  |  |  |  |  |  |  |  |  |  |  |  |  |  |  |  |  |  |  |  |  |  |  |  |  |  |  |  |  |  |  |  |  |  |  |  |  |  |  |  |  |  |  |  |  |  |  |  |  |  |  |  |  |  |  |  |  |  |  |  |  |  |  |  |  |  |  |  |  |  |  |  |  |  |  |  |  |  |  |  |  |  |  |  |  |  |  |  |  |  |  |  |  |  |  |  |  |  |  |  |  |  |  |  |  |  |  |  |  |  |  |  |  |  |  |  |  |  |  |  |  |  |  |  |  |  |  |  |  |  |  |  |  |  |  |  |  |  |  |  |  |  |  |  |  |  |  |  |  |  |  |  |  |  |  |  |  |  |  |  |  |  |  |  |  |  |  |  |  |  |  |  |  |  |  |  |  |  |  |  |  |  |  |  |  |  |  |  |  |  |  |  |  |  |  |  |  |  |  |  |  |  |  |  |  |  |  |  |  |  |  |  |  |  |  |  |  |  |  |  |  |  |  |  |  |  |  |  |  |  |  |  |  |  |  |  |  |  |  |  |  |  |  |  |  |  |  |  |  |  |  |  |  |  |  |  |  |  |  |  |  |  |  |  |  |  |  |  |  |  |  |  |  |  |  |  |  |  |  |  |  |  |  |  |  |  |  |  |  |  |  |  |  |  |  |  |  |  |  |  |  |  |  |  |  |  |  |  |  |  |  |  |  |  |  |  |  |  |  |  |  |  |  |  |  |  |  |  |  |  |  |  |  |  |  |  |  |  |  |  |  |  |  |  |  |  |  |  |  |  |  |  |  |  |  |  |  |  |  |  |  |  |  |  |  |  |  |  |  |  |  |  |  |  |  |  |  |  |  |  |  |  |  |  |  |  |  |  |  |  |  |  |  |  |  |  |  |  |  |  |  |  |  |  |  |  |  |  |  |  |  |  |  |  |  |  |  |  |  |  |  |  |  |  |  |  |  |  |  |  |  |  |  |  |  |  |  |  |  |  |  |  |  |  |  |  |  |  |  |  |  |  |  |  |  |  |  |  |  |  |  |  |  |  |  |  |  |  |  |  |  |  |  |  |  |  |  |  |  |  |  |  |  |  |  |  |  |  |  |  |  |  |  |  |  |  |  |  |  |  |  |  |  |  |  |  |  |  |  |  |  |  |  |  |  |  |  |  |  |  |  |  |  |  |  |  |  |  |  |  |  |  |  |  |  |  |  |  |  |  |  |  |  |  |  |  |  |  |  |  |  |  |  |  |  |  |  |  |  |  |  |  |  |  |  |  |  |  |  |  |  |  |  |  |  |  |  |  |  |  |  |  |  |  |  |  |  |  |  |  |  |  |  |  |  |  |  |  |  |  |  |  |  |  |  |  |  |  |  |  |  |  |  |  |  |  |  |  |  |  |  |  |  |  |  |  |  |  |  |  |  |  |  |  |  |  |  |  |  |  |  |  |  |  |  |  |  |  |  |  |  |  |  |  |  |  |  |  |  |  |  |  |  |  |  |  |  |  |  |  |  |  |  |  |  |  |  |  |  |  |  |  |  |  |  |  |  |  |  |  |  |  |  |  |  |  |  |  |  |  |  |  |  |  |  |  |  |  |  |  |  |  |  |  |  |  |  |  |  |  |  |  |  |  |  |  |  |  |  |  |  |  |  |  |  |  |  |  |  |  |  |  |  |  |  |  |  |  |  |  |  |  |  |  |  |  |  |  |  |  |  |  |  |  |  |  |  |  |  |  |  |  |  |  |  |  |  |  |  |  |  |  |  |  |  |  |  |  |  |  |  |  |  |  |  |  |  |  |  |  |  |  |  |  |  |  |  |  |  |  |  |  |  |  |  |  |  |  |  |  |  |  |  |  |  |  |  |  |  |  |  |  |  |  |  |  |  |  |  |  |  |  |  |  |  |  |  |  |  |  |  |  |  |  |  |  |  |  |  |  |  |  |  |  |  |  |  |  |  |  |  |  |  |  |  |  |  |  |  |  |  |  |  |  |  |  |  |  |  |  |  |  |  |  |  |  |  |  |  |  |  |  |  |  |  |  |  |  |  |  |  |  |  |  |  |  |  |  |  |  |  |  |  |  |  |  |  |  |  |  |  |  |  |  |  |  |  |  |  |  |  |  |  |  |  |  |  |  |  |  |  |  |  |  |  |  |  |  |  |  |  |  |  |  |  |  |  |  |  |  |  |  |  |  |  |  |  |  |  |  |  |  |  |  |  |  |  |  |  |  |  |  |  |  |  |  |  |  |  |  |  |  |  |  |  |  |  |  |  |  |  |  |  |  |  |  |  |  |  |  |  |  |  |  |  |  |  |  |  |  |  |  |  |  |  |  |  |  |  |  |  |  |  |  |  |  |  |  |  |  |  |  |  |  |  |  |  |  |  |  |  |  |  |  |  |  |  |  |  |  |  |  |  |  |  |  |  |  |  |  |  |  |  |  |  |  |  |  |  |  |  |  |  |  |  |  |  |  |  |  |  |  |  |  |  |  |  |  |  |  |  |  |  |  |  |  |  |  |  |  |  |  |  |  |  |  |  |  |  |  |  |  |  |  |  |  |  |  |  |  |  |  |  |  |  |  |  |  |  |  |  |  |  |  |  |  |  |  |  |  |  |  |  |  |  |  |  |  |  |  |  |  |  |  |  |  |  |  |  |  |  |  |  |  |  |  |  |  |  |  |  |  |  |  |  |  |  |  |  |  |  |  |  |  |  |  |  |  |  |  |  |  |  |  |  |  |  |  |  |  |  |  |  |  |  |  |  |  |  |  |  |  |  |  |  |  |  |  |  |  |  |  |  |  |  |  |  |  |  |  |  |  |  |  |  |  |  |  |  |  |  |  |  |  |  |  |  |  |  |  |  |  |  |  |  |  |  |  |  |  |  |  |  |  |  |  |  |  |  |  |  |  |  |  |  |  |  |  |  |  |  |  |  |  |  |  |  |  |  |  |  |  |  |  |  |  |  |  |  |  |  |  |  |  |  |  |  |  |  |  |  |  |  |  |  |  |  |  |  |  |  |  |  |  |  |  |  |  |  |  |  |  |  |  |  |  |  |  |  |  |  |  |  |  |  |  |  |  |  |  |  |  |  |  |  |  |  |  |  |  |  |  |  |  |  |  |  |  |  |  |  |  |  |  |  |  |  |  |  |  |  |  |  |  |  |  |  |  |  |  |  |  |  |  |  |  |  |  |  |  |  |  |  |  |  |  |  |  |  |  |  |  |  |  |  |  |  |  |  |  |  |  |  |  |  |  |  |  |  |  |  |  |  |  |  |  |  |  |  |  |  |  |  |  |  |  |  |  |  |  |  |  |  |  |  |  |  |  |  |  |  |  |  |  |  |  |  |  |  |  |  |  |  |  |  |  |  |  |  |  |  |  |  |  |  |  |  |  |  |  |  |  |  |  |  |  |  |  |  |  |  |  |  |  |  |  |  |  |  |  |  |  |  |  |  |  |  |  |  |  |  |  |  |  |  |  |  |  |  |  |  |  |  |  |  |  |  |  |  |  |  |  |  |  |  |  |  |  |  |  |  |  |  |  |  |  |  |  |  |  |  |  |  |  |  |  |  |  |  |  |  |  |  |  |  |  |  |  |  |  |  |  |  |  |  |  |  |  |  |  |  |  |  |  |  |  |  |  |  |  |  |  |  |  |  |  |  |  |  |  |  |  |  |  |  |  |  |  |  |  |  |  |  |  |  |  |  |  |  |  |  |  |  |  |  |  |  |  |  |  |  |  |  |  |  |  |  |  |  |  |  |  |  |  |  |  |  |  |  |  |  |  |  |  |  |  |  |  |  |  |  |  |  |  |  |  |  |  |  |  |  |  |  |  |  |  |  |  |  |  |  |  |  |  |  |  |  |  |  |  |  |  |  |  |  |  |  |  |  |  |  |  |  |  |  |  |  |  |  |  |  |  |  |  |  |  |  |  |  |  |  |  |  |  |  |  |  |  |  |  |  |  |  |  |  |  |  |  |  |  |  |  |  |  |  |  |  |  |  |  |  |  |  |  |  |  |  |  |  |  |  |  |  |  |  |  |  |  |  |  |  |  |  |  |  |  |  |  |  |  |  |  |  |  |  |  |  |  |  |  |  |  |  |  |  |  |  |  |  |  |  |  |  |  |  |  |  |  |  |  |  |  |  |  |  |  |  |  |  |  |  |  |  |  |  |  |  |  |  |  |  |  |  |  |  |  |  |  |  |  |  |  |  |  |  |  |  |  |  |  |  |  |  |  |  |  |  |  |  |  |  |  |  |  |  |  |  |  |  |  |  |  |  |  |  |  |  |  |  |  |  |  |  |  |  |  |  |  |  |  |  |  |  |  |  |  |  |  |  |  |  |  |  |  |  |  |  |  |  |  |  |  |  |  |  |  |  |  |  |  |  |  |  |  |  |  |  |  |  |  |  |  |  |  |  |  |  |  |  |  |  |  |  |  |  |  |  |  |  |  |  |  |  |  |  |  |  |  |  |  |  |  |  |  |  |  |  |  |  |  |  |  |  |  |  |  |  |  |  |  |  |  |  |  |  |  |  |  |  |  |  |  |  |  |  |  |  |  |  |  |  |  |  |  |  |  |  |  |  |  |  |  |  |  |  |  |  |  |  |  |  |  |  |  |  |  |  |  |  |  |  |  |  |  |  |  |  |  |  |  |  |  |  |  |  |  |  |  |  |  |  |  |  |  |  |  |  |  |  |  |  |  |  |  |  |  |  |  |  |  |  |  |  |  |  |  |  |  |  |  |  |  |  |  |  |  |  |  |  |  |  |  |  |  |  |  |  |  |  |  |  |  |  |  |  |  |  |  |  |  |  |  |  |  |  |  |  |  |  |  |  |  |  |  |  |  |  |  |  |  |  |  |  |  |  |  |  |  |  |  |  |  |  |  |  |  |  |  |  |  |  |  |  |  |  |  |  |  |  |  |  |  |  |  |  |  |  |  |  |  |  |  |  |  |  |  |  |  |  |  |  |  |  |  |  |  |  |  |  |  |  |  |  |  |  |  |  |  |  |  |  |  |  |  |  |  |  |  |  |  |  |  |  |  |  |  |  |  |  |  |  |  |  |  |  |  |  |  |  |  |  |  |  |  |  |  |  |  |  |  |  |  |  |  |  |  |  |  |  |
| --- | --- | --- | --- | --- | --- | --- | --- | --- | --- | --- | --- | --- | --- | --- | --- | --- | --- | --- | --- | --- | --- | --- | --- | --- | --- | --- | --- | --- | --- | --- | --- | --- | --- | --- | --- | --- | --- | --- | --- | --- | --- | --- | --- | --- | --- | --- | --- | --- | --- | --- | --- | --- | --- | --- | --- | --- | --- | --- | --- | --- | --- | --- | --- | --- | --- | --- | --- | --- | --- | --- | --- | --- | --- | --- | --- | --- | --- | --- | --- | --- | --- | --- | --- | --- | --- | --- | --- | --- | --- | --- | --- | --- | --- | --- | --- | --- | --- | --- | --- | --- | --- | --- | --- | --- | --- | --- | --- | --- | --- | --- | --- | --- | --- | --- | --- | --- | --- | --- | --- | --- | --- | --- | --- | --- | --- | --- | --- | --- | --- | --- | --- | --- | --- | --- | --- | --- | --- | --- | --- | --- | --- | --- | --- | --- | --- | --- | --- | --- | --- | --- | --- | --- | --- | --- | --- | --- | --- | --- | --- | --- | --- | --- | --- | --- | --- | --- | --- | --- | --- | --- | --- | --- | --- | --- | --- | --- | --- | --- | --- | --- | --- | --- | --- | --- | --- | --- | --- | --- | --- | --- | --- | --- | --- | --- | --- | --- | --- | --- | --- | --- | --- | --- | --- | --- | --- | --- | --- | --- | --- | --- | --- | --- | --- | --- | --- | --- | --- | --- | --- | --- | --- | --- | --- | --- | --- | --- | --- | --- | --- | --- | --- | --- | --- | --- | --- | --- | --- | --- | --- | --- | --- | --- | --- | --- | --- | --- | --- | --- | --- | --- | --- | --- | --- | --- | --- | --- | --- | --- | --- | --- | --- | --- | --- | --- | --- | --- | --- | --- | --- | --- | --- | --- | --- | --- | --- | --- | --- | --- | --- | --- | --- | --- | --- | --- | --- | --- | --- | --- | --- | --- | --- | --- | --- | --- | --- | --- | --- | --- | --- | --- | --- | --- | --- | --- | --- | --- | --- | --- | --- | --- | --- | --- | --- | --- | --- | --- | --- | --- | --- | --- | --- | --- | --- | --- | --- | --- | --- | --- | --- | --- | --- | --- | --- | --- | --- | --- | --- | --- | --- | --- | --- | --- | --- | --- | --- | --- | --- | --- | --- | --- | --- | --- | --- | --- | --- | --- | --- | --- | --- | --- | --- | --- | --- | --- | --- | --- | --- | --- | --- | --- | --- | --- | --- | --- | --- | --- | --- | --- | --- | --- | --- | --- | --- | --- | --- | --- | --- | --- | --- | --- | --- | --- | --- | --- | --- | --- | --- | --- | --- | --- | --- | --- | --- | --- | --- | --- | --- | --- | --- | --- | --- | --- | --- | --- | --- | --- | --- | --- | --- | --- | --- | --- | --- | --- | --- | --- | --- | --- | --- | --- | --- | --- | --- | --- | --- | --- | --- | --- | --- | --- | --- | --- | --- | --- | --- | --- | --- | --- | --- | --- | --- | --- | --- | --- | --- | --- | --- | --- | --- | --- | --- | --- | --- | --- | --- | --- | --- | --- | --- | --- | --- | --- | --- | --- | --- | --- | --- | --- | --- | --- | --- | --- | --- | --- | --- | --- | --- | --- | --- | --- | --- | --- | --- | --- | --- | --- | --- | --- | --- | --- | --- | --- | --- | --- | --- | --- | --- | --- | --- | --- | --- | --- | --- | --- | --- | --- | --- | --- | --- | --- | --- | --- | --- | --- | --- | --- | --- | --- | --- | --- | --- | --- | --- | --- | --- | --- | --- | --- | --- | --- | --- | --- | --- | --- | --- | --- | --- | --- | --- | --- | --- | --- | --- | --- | --- | --- | --- | --- | --- | --- | --- | --- | --- | --- | --- | --- | --- | --- | --- | --- | --- | --- | --- | --- | --- | --- | --- | --- | --- | --- | --- | --- | --- | --- | --- | --- | --- | --- | --- | --- | --- | --- | --- | --- | --- | --- | --- | --- | --- | --- | --- | --- | --- | --- | --- | --- | --- | --- | --- | --- | --- | --- | --- | --- | --- | --- | --- | --- | --- | --- | --- | --- | --- | --- | --- | --- | --- | --- | --- | --- | --- | --- | --- | --- | --- | --- | --- | --- | --- | --- | --- | --- | --- | --- | --- | --- | --- | --- | --- | --- | --- | --- | --- | --- | --- | --- | --- | --- | --- | --- | --- | --- | --- | --- | --- | --- | --- | --- | --- | --- | --- | --- | --- | --- | --- | --- | --- | --- | --- | --- | --- | --- | --- | --- | --- | --- | --- | --- | --- | --- | --- | --- | --- | --- | --- | --- | --- | --- | --- | --- | --- | --- | --- | --- | --- | --- | --- | --- | --- | --- | --- | --- | --- | --- | --- | --- | --- | --- | --- | --- | --- | --- | --- | --- | --- | --- | --- | --- | --- | --- | --- | --- | --- | --- | --- | --- | --- | --- | --- | --- | --- | --- | --- | --- | --- | --- | --- | --- | --- | --- | --- | --- | --- | --- | --- | --- | --- | --- | --- | --- | --- | --- | --- | --- | --- | --- | --- | --- | --- | --- | --- | --- | --- | --- | --- | --- | --- | --- | --- | --- | --- | --- | --- | --- | --- | --- | --- | --- | --- | --- | --- | --- | --- | --- | --- | --- | --- | --- | --- | --- | --- | --- | --- | --- | --- | --- | --- | --- | --- | --- | --- | --- | --- | --- | --- | --- | --- | --- | --- | --- | --- | --- | --- | --- | --- | --- | --- | --- | --- | --- | --- | --- | --- | --- | --- | --- | --- | --- | --- | --- | --- | --- | --- | --- | --- | --- | --- | --- | --- | --- | --- | --- | --- | --- | --- | --- | --- | --- | --- | --- | --- | --- | --- | --- | --- | --- | --- | --- | --- | --- | --- | --- | --- | --- | --- | --- | --- | --- | --- | --- | --- | --- | --- | --- | --- | --- | --- | --- | --- | --- | --- | --- | --- | --- | --- | --- | --- | --- | --- | --- | --- | --- | --- | --- | --- | --- | --- | --- | --- | --- | --- | --- | --- | --- | --- | --- | --- | --- | --- | --- | --- | --- | --- | --- | --- | --- | --- | --- | --- | --- | --- | --- | --- | --- | --- | --- | --- | --- | --- | --- | --- | --- | --- | --- | --- | --- | --- | --- | --- | --- | --- | --- | --- | --- | --- | --- | --- | --- | --- | --- | --- | --- | --- | --- | --- | --- | --- | --- | --- | --- | --- | --- | --- | --- | --- | --- | --- | --- | --- | --- | --- | --- | --- | --- | --- | --- | --- | --- | --- | --- | --- | --- | --- | --- | --- | --- | --- | --- | --- | --- | --- | --- | --- | --- | --- | --- | --- | --- | --- | --- | --- | --- | --- | --- | --- | --- | --- | --- | --- | --- | --- | --- | --- | --- | --- | --- | --- | --- | --- | --- | --- | --- | --- | --- | --- | --- | --- | --- | --- | --- | --- | --- | --- | --- | --- | --- | --- | --- | --- | --- | --- | --- | --- | --- | --- | --- | --- | --- | --- | --- | --- | --- | --- | --- | --- | --- | --- | --- | --- | --- | --- | --- | --- | --- | --- | --- | --- | --- | --- | --- | --- | --- | --- | --- | --- | --- | --- | --- | --- | --- | --- | --- | --- | --- | --- | --- | --- | --- | --- | --- | --- | --- | --- | --- | --- | --- | --- | --- | --- | --- | --- | --- | --- | --- | --- | --- | --- | --- | --- | --- | --- | --- | --- | --- | --- | --- | --- | --- | --- | --- | --- | --- | --- | --- | --- | --- | --- | --- | --- | --- | --- | --- | --- | --- | --- | --- | --- | --- | --- | --- | --- | --- | --- | --- | --- | --- | --- | --- | --- | --- | --- | --- | --- | --- | --- | --- | --- | --- | --- | --- | --- | --- | --- | --- | --- | --- | --- | --- | --- | --- | --- | --- | --- | --- | --- | --- | --- | --- | --- | --- | --- | --- | --- | --- | --- | --- | --- | --- | --- | --- | --- | --- | --- | --- | --- | --- | --- | --- | --- | --- | --- | --- | --- | --- | --- | --- | --- | --- | --- | --- | --- | --- | --- | --- | --- | --- | --- | --- | --- | --- | --- | --- | --- | --- | --- | --- | --- | --- | --- | --- | --- | --- | --- | --- | --- | --- | --- | --- | --- | --- | --- | --- | --- | --- | --- | --- | --- | --- | --- | --- | --- | --- | --- | --- | --- | --- | --- | --- | --- | --- | --- | --- | --- | --- | --- | --- | --- | --- | --- | --- | --- | --- | --- | --- | --- | --- | --- | --- | --- | --- | --- | --- | --- | --- | --- | --- | --- | --- | --- | --- | --- | --- | --- | --- | --- | --- | --- | --- | --- | --- | --- | --- | --- | --- | --- | --- | --- | --- | --- | --- | --- | --- | --- | --- | --- | --- | --- | --- | --- | --- | --- | --- | --- | --- | --- | --- | --- | --- | --- | --- | --- | --- | --- | --- | --- | --- | --- | --- | --- | --- | --- | --- | --- | --- | --- | --- | --- | --- | --- | --- | --- | --- | --- | --- | --- | --- | --- | --- | --- | --- | --- | --- | --- | --- | --- | --- | --- | --- | --- | --- | --- | --- | --- | --- | --- | --- | --- | --- | --- | --- | --- | --- | --- | --- | --- | --- | --- | --- | --- | --- | --- | --- | --- | --- | --- | --- | --- | --- | --- | --- | --- | --- | --- | --- | --- | --- | --- | --- | --- | --- | --- | --- | --- | --- | --- | --- | --- | --- | --- | --- | --- | --- | --- | --- | --- | --- | --- | --- | --- | --- | --- | --- | --- | --- | --- | --- | --- | --- | --- | --- | --- | --- | --- | --- | --- | --- | --- | --- | --- | --- | --- | --- | --- | --- | --- | --- | --- | --- | --- | --- | --- | --- | --- | --- | --- | --- | --- | --- | --- | --- | --- | --- | --- | --- | --- | --- | --- | --- | --- | --- | --- | --- | --- | --- | --- | --- | --- | --- | --- | --- | --- | --- | --- | --- | --- | --- | --- | --- | --- | --- | --- | --- | --- | --- | --- | --- | --- | --- | --- | --- | --- | --- | --- | --- | --- | --- | --- | --- | --- | --- | --- | --- | --- | --- | --- | --- | --- | --- | --- | --- | --- | --- | --- | --- | --- | --- | --- | --- | --- | --- | --- | --- | --- | --- | --- | --- | --- | --- | --- | --- | --- | --- | --- | --- | --- | --- | --- | --- | --- | --- | --- | --- | --- | --- | --- | --- | --- | --- | --- | --- | --- | --- | --- | --- | --- | --- | --- | --- | --- | --- | --- | --- | --- | --- | --- | --- | --- | --- | --- | --- | --- | --- | --- | --- | --- | --- | --- | --- | --- | --- | --- | --- | --- | --- | --- | --- | --- | --- | --- | --- | --- | --- | --- | --- | --- | --- | --- | --- | --- | --- | --- | --- | --- | --- | --- | --- | --- | --- | --- | --- | --- | --- | --- | --- | --- | --- | --- | --- | --- | --- | --- | --- | --- | --- | --- | --- | --- | --- | --- | --- | --- | --- | --- | --- | --- | --- | --- | --- | --- | --- | --- | --- | --- | --- | --- | --- | --- | --- | --- | --- | --- | --- | --- | --- | --- | --- | --- | --- | --- | --- | --- | --- | --- | --- | --- | --- | --- | --- | --- | --- | --- | --- | --- | --- | --- | --- | --- | --- | --- | --- | --- | --- | --- | --- | --- | --- | --- | --- | --- | --- | --- | --- | --- | --- | --- | --- | --- | --- | --- | --- | --- | --- | --- | --- | --- | --- | --- | --- | --- | --- | --- | --- | --- | --- | --- | --- | --- | --- | --- | --- | --- | --- | --- | --- | --- | --- | --- | --- | --- | --- | --- | --- | --- | --- | --- | --- | --- | --- | --- | --- | --- | --- | --- | --- | --- | --- | --- | --- | --- | --- | --- | --- | --- | --- | --- | --- | --- | --- | --- | --- | --- | --- | --- | --- | --- | --- | --- | --- | --- | --- | --- | --- | --- | --- | --- | --- | --- | --- | --- | --- | --- | --- | --- | --- | --- | --- | --- | --- | --- | --- | --- | --- | --- | --- | --- | --- | --- | --- | --- | --- | --- | --- | --- | --- | --- | --- | --- | --- | --- | --- | --- | --- | --- | --- | --- | --- | --- | --- | --- | --- | --- | --- | --- | --- | --- | --- | --- | --- | --- | --- | --- | --- | --- | --- | --- | --- | --- | --- | --- | --- | --- | --- | --- | --- | --- | --- | --- | --- | --- | --- | --- | --- | --- | --- | --- | --- | --- | --- | --- | --- | --- | --- | --- | --- | --- | --- | --- | --- | --- | --- | --- | --- | --- | --- | --- | --- | --- | --- | --- | --- | --- | --- | --- | --- | --- | --- | --- | --- | --- | --- | --- | --- | --- | --- | --- | --- | --- | --- | --- | --- | --- | --- | --- | --- | --- | --- | --- | --- | --- | --- | --- | --- | --- | --- | --- | --- | --- | --- | --- | --- | --- | --- | --- | --- | --- | --- | --- | --- | --- | --- | --- | --- | --- | --- | --- | --- | --- | --- | --- | --- | --- | --- | --- | --- | --- | --- | --- | --- | --- | --- | --- | --- | --- | --- | --- | --- | --- | --- | --- | --- | --- | --- | --- | --- | --- | --- | --- | --- | --- | --- | --- | --- | --- | --- | --- | --- | --- | --- | --- | --- | --- | --- | --- | --- | --- | --- | --- | --- | --- | --- | --- | --- | --- | --- | --- | --- | --- | --- | --- | --- | --- | --- | --- | --- | --- | --- | --- | --- | --- | --- | --- | --- | --- | --- | --- | --- | --- | --- | --- | --- | --- | --- | --- | --- | --- | --- | --- | --- | --- | --- | --- | --- | --- | --- | --- | --- | --- | --- | --- | --- | --- | --- | --- | --- | --- | --- | --- | --- | --- | --- | --- | --- | --- | --- | --- | --- | --- | --- | --- | --- | --- | --- | --- | --- | --- | --- | --- | --- | --- | --- | --- | --- | --- | --- | --- | --- | --- | --- | --- | --- | --- | --- | --- | --- | --- | --- | --- | --- | --- | --- | --- | --- | --- | --- | --- | --- | --- | --- | --- | --- | --- | --- | --- | --- | --- | --- | --- | --- | --- | --- | --- | --- | --- | --- | --- | --- | --- | --- | --- | --- | --- | --- | --- | --- | --- | --- | --- | --- | --- | --- | --- | --- | --- | --- | --- | --- | --- | --- | --- | --- | --- | --- | --- | --- | --- | --- | --- | --- | --- | --- | --- | --- | --- | --- | --- | --- | --- | --- | --- | --- | --- | --- | --- | --- | --- | --- | --- | --- | --- | --- | --- | --- | --- | --- | --- | --- | --- | --- | --- | --- | --- | --- | --- | --- | --- | --- | --- | --- | --- | --- | --- | --- | --- | --- | --- | --- | --- | --- | --- | --- | --- | --- | --- | --- | --- | --- | --- | --- | --- | --- | --- | --- | --- | --- | --- | --- | --- | --- | --- | --- | --- | --- | --- | --- | --- | --- | --- | --- | --- | --- | --- | --- | --- | --- | --- | --- | --- | --- | --- | --- | --- | --- | --- | --- | --- | --- | --- | --- | --- | --- | --- | --- | --- | --- | --- | --- | --- | --- | --- | --- | --- | --- | --- | --- | --- | --- | --- | --- | --- | --- | --- | --- | --- | --- | --- | --- | --- | --- | --- | --- | --- | --- | --- | --- | --- | --- | --- | --- | --- | --- | --- | --- | --- | --- | --- | --- | --- | --- | --- | --- | --- | --- | --- | --- | --- | --- | --- | --- | --- | --- | --- | --- | --- | --- | --- | --- | --- | --- | --- | --- | --- | --- | --- | --- | --- | --- | --- | --- | --- | --- | --- | --- | --- | --- | --- | --- | --- | --- | --- | --- | --- | --- | --- | --- | --- | --- | --- | --- | --- | --- | --- | --- | --- | --- | --- | --- | --- | --- | --- | --- | --- | --- | --- | --- | --- | --- | --- | --- | --- | --- | --- | --- | --- | --- | --- | --- | --- | --- | --- | --- | --- | --- | --- | --- | --- | --- | --- | --- | --- | --- | --- | --- | --- | --- | --- | --- | --- | --- | --- | --- | --- | --- | --- | --- | --- | --- | --- | --- | --- | --- | --- | --- | --- | --- | --- | --- | --- | --- | --- | --- | --- | --- | --- | --- | --- | --- | --- | --- | --- | --- | --- | --- | --- | --- | --- | --- | --- | --- | --- | --- | --- | --- | --- | --- | --- | --- | --- | --- | --- | --- | --- | --- | --- | --- | --- | --- | --- | --- | --- | --- | --- | --- | --- | --- | --- | --- | --- | --- | --- | --- | --- | --- | --- | --- | --- | --- | --- | --- | --- | --- | --- | --- | --- | --- | --- | --- | --- | --- | --- | --- | --- | --- | --- | --- | --- | --- | --- | --- | --- | --- | --- | --- | --- | --- | --- | --- | --- | --- | --- | --- | --- | --- | --- | --- | --- | --- | --- | --- | --- | --- | --- | --- | --- | --- | --- |
| |  |  |  |  |  |  |  |  |  |  |  |  |  |  |  |  |  |  |  |  |  |  |  |  |  |  |  |  |  |  |  |  |  |  |  |  |  |  |  |  |  |  |  |  |  |  |  |  |  |  |  |  |  |  |  |  |  |  | | --- | --- | --- | --- | --- | --- | --- | --- | --- | --- | --- | --- | --- | --- | --- | --- | --- | --- | --- | --- | --- | --- | --- | --- | --- | --- | --- | --- | --- | --- | --- | --- | --- | --- | --- | --- | --- | --- | --- | --- | --- | --- | --- | --- | --- | --- | --- | --- | --- | --- | --- | --- | --- | --- | --- | --- | --- | --- | | G0VDD1/1-187 | 1 | M | S | I | Q | L | F | H | S | F | I | W | K | R | T | L | L | G | L | E | G | G | L | S | I | L | L | P | R | - | - | - | - | K | A | - | - | - | - | - | T | T | T | T | - | - | - | - | - | P | G | S | S | L | Q | E | 41 | | Q6CK81/1-185 | 1 | M | S | F | G | A | V | T | E | S | F | S | R | - | - | - | - | V | L | L | E | T | A | T | S | I | L | P | R | - | - | - | - | W | S | - | - | T | P | A | A | G | T | A | - | - | - | - | - | L | P | Q | K | L | L | E | 40 | | Q6FSY9/1-182 | 1 | M | S | M | L | A | R | L | S | S | G | Y | I | - | F | A | G | A | G | V | G | S | Q | A | V | V | T | P | - | - | - | - | - | V | T | L | G | L | A | G | V | S | Q | A | - | - | - | - | - | I | P | A | L | L | G | K | 44 | | Q75ES7/1-178 | 1 | M | S | A | Q | A | V | W | G | N | V | G | R | - | - | - | - | A | L | S | E | C | T | A | A | L | F | P | R | - | - | - | - | L | E | - | - | L | G | S | G | S | V | T | - | - | - | - | - | A | P | R | T | L | L | E | 40 | | A7TEQ4/1-175 | 1 | M | S | V | I | K | S | L | F | N | A | N | K | - | - | L | T | T | L | T | E | T | C | N | S | L | V | N | T | V | S | K | I | W | S | - | - | V | E | S | G | R | L | A | - | - | - | - | - | L | P | G | G | Y | - | - | 44 | | C5E3D7/1-180 | 1 | M | S | F | - | - | V | A | T | L | K | K | R | - | - | - | - | - | - | C | L | P | V | F | S | L | L | P | S | W | S | F | S | W | Q | - | - | L | A | P | A | P | T | A | - | - | - | - | - | L | P | R | L | L | H | E | 40 | | C5E4A8/1-186 | 1 | M | S | - | - | S | I | G | F | S | M | S | R | - | - | - | - | - | F | Y | L | S | A | T | T | L | L | P | - | - | - | - | - | W | V | K | P | L | G | G | P | M | I | G | E | L | P | K | Q | L | P | E | S | L | R | K | 43 | | Kwal\_0.287/1-183 | 1 | M | S | F | T | R | L | G | A | F | G | E | R | - | - | - | - | - | - | L | I | T | P | L | A | L | L | P | S | W | S | V | A | W | Q | - | - | V | T | P | V | P | S | I | - | - | - | - | - | L | P | R | L | F | Q | D | 42 | | Sbay\_675.37/1-183 | 1 | M | S | T | L | I | F | G | K | R | L | A | L | - | - | - | - | - | - | - | - | - | - | Y | K | I | V | P | A | N | V | I | G | W | L | A | P | L | G | N | P | P | L | L | A | P | S | Q | K | P | L | G | S | I | H | E | 45 | | SAKL0D08822g/1-183 | 1 | M | S | L | - | A | S | G | F | F | L | G | R | - | - | - | - | T | L | S | A | T | A | I | S | I | L | P | R | - | - | - | - | W | A | - | - | I | G | G | S | A | A | S | I | L | P | Q | Q | L | P | Q | R | L | S | E | 44 | | P25348/1-183 | 1 | M | N | S | L | I | F | G | K | Q | L | A | F | - | - | - | - | - | - | - | - | - | - | H | K | I | V | P | T | T | A | I | G | W | L | V | P | L | G | N | P | S | L | Q | I | P | G | Q | K | Q | L | G | S | I | H | R | 45 | |  | | G0VDD1/1-187 | 42 | W | I | R | R | K | L | L | D | G | P | S | S | T | D | V | P | E | S | N | T | I | D | T | G | V | L | K | A | V | P | K | K | K | V | S | H | Q | K | K | R | Q | R | L | Y | G | P | G | S | K | Q | L | K | M | V | H | 96 | | Q6CK81/1-185 | 41 | L | L | E | R | N | - | - | Q | R | E | K | S | - | S | P | Q | A | D | Y | F | T | N | N | G | I | L | L | A | V | P | K | K | K | V | S | H | Q | K | K | R | Q | K | L | Y | A | P | G | D | K | Q | L | K | M | I | N | 92 | | Q6FSY9/1-182 | 45 | L | L | G | - | - | - | - | D | T | E | A | K | - | - | - | E | E | T | G | F | F | D | N | G | I | L | L | A | A | P | K | K | K | V | S | H | Q | K | K | R | Q | R | L | L | A | P | G | K | K | H | V | N | M | M | N | 92 | | Q75ES7/1-178 | 41 | L | L | R | R | - | - | - | A | G | G | S | Q | - | Q | A | G | T | A | A | V | G | A | D | G | L | V | L | A | V | P | K | K | K | V | S | H | Q | K | R | R | Q | K | L | Y | G | P | G | K | K | Q | L | Q | M | V | H | 91 | | A7TEQ4/1-175 | 45 | - | - | - | - | - | - | - | T | I | E | P | N | - | - | - | N | W | D | I | F | S | N | G | G | M | L | L | A | V | P | K | K | K | V | S | H | Q | K | K | R | Q | R | L | Y | A | P | G | K | K | Q | L | K | F | Q | H | 89 | | C5E3D7/1-180 | 41 | L | L | E | G | - | - | - | Q | V | E | P | Q | L | S | P | D | S | A | S | P | A | K | E | G | I | L | L | A | V | P | K | K | K | V | S | H | Q | K | K | R | Q | K | L | Y | G | P | G | R | K | Q | L | Q | M | I | H | 92 | | C5E4A8/1-186 | 44 | W | I | D | Q | R | T | K | K | N | E | E | G | S | I | V | E | E | D | F | F | S | N | N | G | R | L | L | A | V | P | K | K | K | V | T | H | Q | K | K | R | Q | R | L | Y | A | P | G | K | K | Q | L | K | F | I | H | 98 | | Kwal\_0.287/1-183 | 43 | L | L | D | K | R | G | - | K | G | S | P | S | - | S | P | V | G | E | T | D | I | G | N | G | I | F | L | A | V | P | K | K | K | V | S | H | Q | K | K | R | Q | K | L | Y | G | P | G | R | K | Q | L | Q | M | I | H | 95 | | Sbay\_675.37/1-183 | 46 | W | L | R | K | K | L | L | G | D | G | K | G | - | I | R | D | K | D | F | F | S | N | N | G | I | L | L | A | V | P | K | K | K | V | S | H | Q | K | K | R | Q | K | L | Y | G | P | G | K | K | Q | L | K | M | I | H | 99 | | SAKL0D08822g/1-183 | 45 | L | L | E | K | - | - | - | R | S | E | K | Q | - | T | P | E | Q | D | F | F | S | N | N | G | I | L | L | A | V | P | K | K | K | V | S | H | Q | K | K | R | Q | K | L | Y | G | P | G | K | K | Q | L | K | M | I | H | 95 | | P25348/1-183 | 46 | W | L | R | E | K | L | Q | Q | D | H | K | D | - | T | E | D | K | D | F | F | S | N | N | G | I | L | L | A | V | P | K | K | K | V | S | H | Q | K | K | R | Q | K | L | Y | G | P | G | K | K | Q | L | K | M | I | H | 99 | |  | | G0VDD1/1-187 | 97 | H | L | N | E | C | P | S | C | G | H | Y | K | R | A | N | T | L | C | M | Y | C | V | D | S | I | R | R | I | W | K | A | Q | S | V | E | R | N | S | L | W | K | E | P | E | Q | E | I | G | L | S | D | V | D | K | K | 151 | | Q6CK81/1-185 | 93 | H | L | N | K | C | P | S | C | G | H | Y | K | K | A | H | T | L | C | M | H | C | V | G | E | I | R | H | I | W | K | A | H | T | N | V | E | Q | - | - | - | V | E | P | I | Q | E | Q | E | L | S | E | L | D | K | R | 144 | | Q6FSY9/1-182 | 93 | H | L | N | R | C | P | S | C | G | H | Y | K | R | A | N | T | I | C | M | H | C | F | E | N | V | R | F | L | W | K | S | Y | T | Q | E | Q | R | - | - | - | Q | E | P | I | Q | E | Q | N | L | T | D | L | D | K | R | 144 | | Q75ES7/1-178 | 92 | H | L | G | K | C | P | S | C | G | H | Y | K | R | L | N | T | L | C | M | Y | C | V | G | E | I | R | H | I | W | K | V | Y | T | Q | T | K | P | - | - | - | A | E | P | P | Q | E | Q | D | L | S | E | L | D | K | R | 143 | | A7TEQ4/1-175 | 90 | H | L | N | R | C | P | S | C | G | H | Y | K | L | A | N | T | L | C | T | N | C | V | Q | E | I | R | H | I | W | K | T | H | T | N | K | K | V | - | - | - | E | D | P | I | Q | E | Q | E | L | S | E | L | D | R | R | 141 | | C5E3D7/1-180 | 93 | H | L | N | R | C | P | S | C | G | H | F | K | R | A | N | T | L | C | M | H | C | V | G | E | I | R | H | I | W | K | S | H | T | V | R | K | T | - | - | - | Q | E | P | L | Q | E | Q | E | L | S | N | L | D | K | R | 144 | | C5E4A8/1-186 | 99 | E | L | N | K | C | P | S | C | G | H | Y | K | R | A | N | C | L | C | M | H | C | V | Q | H | V | R | H | I | W | K | T | Q | T | V | K | E | R | - | - | - | Q | E | P | S | Q | E | Q | E | L | S | D | L | D | K | R | 150 | | Kwal\_0.287/1-183 | 96 | H | L | N | R | C | P | A | C | G | H | Y | K | R | A | N | T | L | C | M | H | C | V | G | E | I | R | H | I | W | K | T | H | T | V | Q | K | P | - | - | - | Q | E | P | L | Q | E | Q | N | L | S | D | L | D | K | R | 147 | | Sbay\_675.37/1-183 | 100 | H | L | N | K | C | P | S | C | G | H | Y | K | R | A | N | T | L | C | M | Y | C | V | G | Q | I | R | H | I | W | K | T | H | T | A | K | E | E | - | - | - | I | K | P | R | Q | E | E | D | L | S | E | L | D | Q | R | 151 | | SAKL0D08822g/1-183 | 96 | H | L | N | K | C | P | S | C | G | H | Y | K | R | A | N | T | L | C | M | H | C | V | N | E | I | R | H | I | W | K | T | Q | T | T | E | N | P | - | - | - | Q | E | P | A | Q | E | Q | E | L | S | E | L | D | R | R | 147 | | P25348/1-183 | 100 | H | L | N | K | C | P | S | C | G | H | Y | K | R | A | N | T | L | C | M | Y | C | V | G | Q | I | S | H | I | W | K | T | H | T | A | K | E | E | - | - | - | I | K | P | R | Q | E | E | E | L | S | E | L | D | Q | R | 151 | |  | | G0VDD1/1-187 | 152 | I | I | Y | P | G | K | K | L | T | R | D | M | E | K | L | R | D | K | D | S | Y | L | H | R | K | M | R | T | L | P | T | E | K | S | R | K | - | - | - | - | - |  | | | | | | | | | | | | | | 187 | | Q6CK81/1-185 | 145 | I | L | Y | P | G | R | K | E | T | E | Y | T | K | K | L | K | D | K | D | A | Y | L | E | R | R | M | K | T | L | P | V | E | R | S | E | K | K | E | G | S | H |  | | | | | | | | | | | | | | 185 | | Q6FSY9/1-182 | 145 | V | L | Y | P | G | K | M | D | T | V | Y | E | E | K | L | K | D | K | D | S | Y | L | V | R | R | M | R | T | L | P | K | E | D | P | K | V | E | S | - | - | - |  | | | | | | | | | | | | | | 182 | | Q75ES7/1-178 | 144 | I | L | Y | P | G | R | E | E | T | E | Y | M | K | K | L | K | K | K | D | - | Y | L | E | K | R | M | R | T | L | P | V | D | D | K | G | K | - | - | - | - | - |  | | | | | | | | | | | | | | 178 | | A7TEQ4/1-175 | 142 | L | I | Y | P | G | K | K | E | T | E | Y | E | K | K | L | N | N | K | D | K | Y | L | K | R | T | I | K | T | L | P | V | E | N | K | - | - | - | - | - | - | - |  | | | | | | | | | | | | | | 175 | | C5E3D7/1-180 | 145 | I | L | Y | P | G | K | K | E | T | E | Y | A | K | K | L | K | D | K | D | S | Y | L | D | R | R | M | R | S | L | P | A | G | K | N | D | S | - | - | - | - | - |  | | | | | | | | | | | | | | 180 | | C5E4A8/1-186 | 151 | I | L | Y | P | G | K | K | E | T | E | Y | N | K | K | L | K | D | K | D | S | Y | L | E | R | R | M | R | T | L | P | V | E | E | K | D | K | - | - | - | - | - |  | | | | | | | | | | | | | | 186 | | Kwal\_0.287/1-183 | 148 | I | L | Y | P | G | R | K | E | T | E | Y | Q | K | K | L | K | D | K | D | S | Y | L | D | R | R | M | R | S | L | P | V | K | G | G | E | H | - | - | - | - | - |  | | | | | | | | | | | | | | 183 | | Sbay\_675.37/1-183 | 152 | V | L | Y | P | G | K | K | E | T | K | Y | A | K | D | L | K | D | K | D | K | Y | L | E | R | R | V | R | T | L | K | K | E | - | - | - | - | - | - | - | - | - |  | | | | | | | | | | | | | | 183 | | SAKL0D08822g/1-183 | 148 | I | L | Y | P | G | K | K | D | T | E | Y | M | K | K | L | K | D | K | D | S | Y | L | E | R | R | L | R | S | L | P | V | E | D | K | K | K | - | - | - | - | - |  | | | | | | | | | | | | | | 183 | | P25348/1-183 | 152 | V | L | Y | P | G | R | R | D | T | K | Y | T | K | D | L | K | D | K | D | N | Y | L | E | R | R | V | R | T | L | K | K | D | - | - | - | - | - | - | - | - | - |  | | | | | | | | | | | | | | 183 | |
