# Supplementary material for: Plus ça change – evolutionary sequence divergence predicts protein subcellular localization signals
Source: BMC Genomics. 2014 Jan 20;15:46. doi: 10.1186/1471-2164-15-46 (PMC3906766; doi:10.1186/1471-2164-15-46)
Supplement: Additional file 2 — MSA’s of proteins for which sequence divergence changes predicted localization signals. Contains links to ortholog multiple sequence alignments of each protein in Additional file 3: Table S1. [file 1471-2164-15-46-S2.zip › P32324.html]

|  |  |  |  |  |  |  |  |  |  |  |  |  |  |  |  |  |  |  |  |  |  |  |  |  |  |  |  |  |  |  |  |  |  |  |  |  |  |  |  |  |  |  |  |  |  |  |  |  |  |  |  |  |  |  |  |  |  |  |  |  |  |  |  |  |  |  |  |  |  |  |  |  |  |  |  |  |  |  |  |  |  |  |  |  |  |  |  |  |  |  |  |  |  |  |  |  |  |  |  |  |  |  |  |  |  |  |  |  |  |  |  |  |  |  |  |  |  |  |  |  |  |  |  |  |  |  |  |  |  |  |  |  |  |  |  |  |  |  |  |  |  |  |  |  |  |  |  |  |  |  |  |  |  |  |  |  |  |  |  |  |  |  |  |  |  |  |  |  |  |  |  |  |  |  |  |  |  |  |  |  |  |  |  |  |  |  |  |  |  |  |  |  |  |  |  |  |  |  |  |  |  |  |  |  |  |  |  |  |  |  |  |  |  |  |  |  |  |  |  |  |  |  |  |  |  |  |  |  |  |  |  |  |  |  |  |  |  |  |  |  |  |  |  |  |  |  |  |  |  |  |  |  |  |  |  |  |  |  |  |  |  |  |  |  |  |  |  |  |  |  |  |  |  |  |  |  |  |  |  |  |  |  |  |  |  |  |  |  |  |  |  |  |  |  |  |  |  |  |  |  |  |  |  |  |  |  |  |  |  |  |  |  |  |  |  |  |  |  |  |  |  |  |  |  |  |  |  |  |  |  |  |  |  |  |  |  |  |  |  |  |  |  |  |  |  |  |  |  |  |  |  |  |  |  |  |  |  |  |  |  |  |  |  |  |  |  |  |  |  |  |  |  |  |  |  |  |  |  |  |  |  |  |  |  |  |  |  |  |  |  |  |  |  |  |  |  |  |  |  |  |  |  |  |  |  |  |  |  |  |  |  |  |  |  |  |  |  |  |  |  |  |  |  |  |  |  |  |  |  |  |  |  |  |  |  |  |  |  |  |  |  |  |  |  |  |  |  |  |  |  |  |  |  |  |  |  |  |  |  |  |  |  |  |  |  |  |  |  |  |  |  |  |  |  |  |  |  |  |  |  |  |  |  |  |  |  |  |  |  |  |  |  |  |  |  |  |  |  |  |  |  |  |  |  |  |  |  |  |  |  |  |  |  |  |  |  |  |  |  |  |  |  |  |  |  |  |  |  |  |  |  |  |  |  |  |  |  |  |  |  |  |  |  |  |  |  |  |  |  |  |  |  |  |  |  |  |  |  |  |  |  |  |  |  |  |  |  |  |  |  |  |  |  |  |  |  |  |  |  |  |  |  |  |  |  |  |  |  |  |  |  |  |  |  |  |  |  |  |  |  |  |  |  |  |  |  |  |  |  |  |  |  |  |  |  |  |  |  |  |  |  |  |  |  |  |  |  |  |  |  |  |  |  |  |  |  |  |  |  |  |  |  |  |  |  |  |  |  |  |  |  |  |  |  |  |  |  |  |  |  |  |  |  |  |  |  |  |  |  |  |  |  |  |  |  |  |  |  |  |  |  |  |  |  |  |  |  |  |  |  |  |  |  |  |  |  |  |  |  |  |  |  |  |  |  |  |  |  |  |  |  |  |  |  |  |  |  |  |  |  |  |  |  |  |  |  |  |  |  |  |  |  |  |  |  |  |  |  |  |  |  |  |  |  |  |  |  |  |  |  |  |  |  |  |  |  |  |  |  |  |  |  |  |  |  |  |  |  |  |  |  |  |  |  |  |  |  |  |  |  |  |  |  |  |  |  |  |  |  |  |  |  |  |  |  |  |  |  |  |  |  |  |  |  |  |  |  |  |  |  |  |  |  |  |  |  |  |  |  |  |  |  |  |  |  |  |  |  |  |  |  |  |  |  |  |  |  |  |  |  |  |  |  |  |  |  |  |  |  |  |  |  |  |  |  |  |  |  |  |  |  |  |  |  |  |  |  |  |  |  |  |  |  |  |  |  |  |  |  |  |  |  |  |  |  |  |  |  |  |  |  |  |  |  |  |  |  |  |  |  |  |  |  |  |  |  |  |  |  |  |  |  |  |  |  |  |  |  |  |  |  |  |  |  |  |  |  |  |  |  |  |  |  |  |  |  |  |  |  |  |  |  |  |  |  |  |  |  |  |  |  |  |  |  |  |  |  |  |  |  |  |  |  |  |  |  |  |  |  |  |  |  |  |  |  |  |  |  |  |  |  |  |  |  |  |  |  |  |  |  |  |  |  |  |  |  |  |  |  |  |  |  |  |  |  |  |  |  |  |  |  |  |  |  |  |  |  |  |  |  |  |  |  |  |  |  |  |  |  |  |  |  |  |  |  |  |  |  |  |  |  |  |  |  |  |  |  |  |  |  |  |  |  |  |  |  |  |  |  |  |  |  |  |  |  |  |  |  |  |  |  |  |  |  |  |  |  |  |  |  |  |  |  |  |  |  |  |  |  |  |  |  |  |  |  |  |  |  |  |  |  |  |  |  |  |  |  |  |  |  |  |  |  |  |  |  |  |  |  |  |  |  |  |  |  |  |  |  |  |  |  |  |  |  |  |  |  |  |  |  |  |  |  |  |  |  |  |  |  |  |  |  |  |  |  |  |  |  |  |  |  |  |  |  |  |  |  |  |  |  |  |  |  |  |  |  |  |  |  |  |  |  |  |  |  |  |  |  |  |  |  |  |  |  |  |  |  |  |  |  |  |  |  |  |  |  |  |  |  |  |  |  |  |  |  |  |  |  |  |  |  |  |  |  |  |  |  |  |  |  |  |  |  |  |  |  |  |  |  |  |  |  |  |  |  |  |  |  |  |  |  |  |  |  |  |  |  |  |  |  |  |  |  |  |  |  |  |  |  |  |  |  |  |  |  |  |  |  |  |  |  |  |  |  |  |  |  |  |  |  |  |  |  |  |  |  |  |  |  |  |  |  |  |  |  |  |  |  |  |  |  |  |  |  |  |  |  |  |  |  |  |  |  |  |  |  |  |  |  |  |  |  |  |  |  |  |  |  |  |  |  |  |  |  |  |  |  |  |  |  |  |  |  |  |  |  |  |  |  |  |  |  |  |  |  |  |  |  |  |  |  |  |  |  |  |  |  |  |  |  |  |  |  |  |  |  |  |  |  |  |  |  |  |  |  |  |  |  |  |  |  |  |  |  |  |  |  |  |  |  |  |  |  |  |  |  |  |  |  |  |  |  |  |  |  |  |  |  |  |  |  |  |  |  |  |  |  |  |  |  |  |  |  |  |  |  |  |  |  |  |  |  |  |  |  |  |  |  |  |  |  |  |  |  |  |  |  |  |  |  |  |  |  |  |  |  |  |  |  |  |  |  |  |  |  |  |  |  |  |  |  |  |  |  |  |  |  |  |  |  |  |  |  |  |  |  |  |  |  |  |  |  |  |  |  |  |  |  |  |  |  |  |  |  |  |  |  |  |  |  |  |  |  |  |  |  |  |  |  |  |  |  |  |  |  |  |  |  |  |  |  |  |  |  |  |  |  |  |  |  |  |  |  |  |  |  |  |  |  |  |  |  |  |  |  |  |  |  |  |  |  |  |  |  |  |  |  |  |  |  |  |  |  |  |  |  |  |  |  |  |  |  |  |  |  |  |  |  |  |  |  |  |  |  |  |  |  |  |  |  |  |  |  |  |  |  |  |  |  |  |  |  |  |  |  |  |  |  |  |  |  |  |  |  |  |  |  |  |  |  |  |  |  |  |  |  |  |  |  |  |  |  |  |  |  |  |  |  |  |  |  |  |  |  |  |  |  |  |  |  |  |  |  |  |  |  |  |  |  |  |  |  |  |  |  |  |  |  |  |  |  |  |  |  |  |  |  |  |  |  |  |  |  |  |  |  |  |  |  |  |  |  |  |  |  |  |  |  |  |  |  |  |  |  |  |  |  |  |  |  |  |  |  |  |  |  |  |  |  |  |  |  |  |  |  |  |  |  |  |  |  |  |  |  |  |  |  |  |  |  |  |  |  |  |  |  |  |  |  |  |  |  |  |  |  |  |  |  |  |  |  |  |  |  |  |  |  |  |  |  |  |  |  |  |  |  |  |  |  |  |  |  |  |  |  |  |  |  |  |  |  |  |  |  |  |  |  |  |  |  |  |  |  |  |  |  |  |  |  |  |  |  |  |  |  |  |  |  |  |  |  |  |  |  |  |  |  |  |  |  |  |  |  |  |  |  |  |  |  |  |  |  |  |  |  |  |  |  |  |  |  |  |  |  |  |  |  |  |  |  |  |  |  |  |  |  |  |  |  |  |  |  |  |  |  |  |  |  |  |  |  |  |  |  |  |  |  |  |  |  |  |  |  |  |  |  |  |  |  |  |  |  |  |  |  |  |  |  |  |  |  |  |  |  |  |  |  |  |  |  |  |  |  |  |  |  |  |  |  |  |  |  |  |  |  |  |  |  |  |  |  |  |  |  |  |  |  |  |  |  |  |  |  |  |  |  |  |  |  |  |  |  |  |  |  |  |  |  |  |  |  |  |  |  |  |  |  |  |  |  |  |  |  |  |  |  |  |  |  |  |  |  |  |  |  |  |  |  |  |  |  |  |  |  |  |  |  |  |  |  |  |  |  |  |  |  |  |  |  |  |  |  |  |  |  |  |  |  |  |  |  |  |  |  |  |  |  |  |  |  |  |  |  |  |  |  |  |  |  |  |  |  |  |  |  |  |  |  |  |  |  |  |  |  |  |  |  |  |  |  |  |  |  |  |  |  |  |  |  |  |  |  |  |  |  |  |  |  |  |  |  |  |  |  |  |  |  |  |  |  |  |  |  |  |  |  |  |  |  |  |  |  |  |  |  |  |  |  |  |  |  |  |  |  |  |  |  |  |  |  |  |  |  |  |  |  |  |  |  |  |  |  |  |  |  |  |  |  |  |  |  |  |  |  |  |  |  |  |  |  |  |  |  |  |  |  |  |  |  |  |  |  |  |  |  |  |  |  |  |  |  |  |  |  |  |  |  |  |  |  |  |  |  |  |  |  |  |  |  |  |  |  |  |  |  |  |  |  |  |  |  |  |  |  |  |  |  |  |  |  |  |  |  |  |  |  |  |  |  |  |  |  |  |  |  |  |  |  |  |  |  |  |  |  |  |  |  |  |  |  |  |  |  |  |  |  |  |  |  |  |  |  |  |  |  |  |  |  |  |  |  |  |  |  |  |  |  |  |  |  |  |  |  |  |  |  |  |  |  |  |  |  |  |  |  |  |  |  |  |  |  |  |  |  |  |  |  |  |  |  |  |  |  |  |  |  |  |  |  |  |  |  |  |  |  |  |  |  |  |  |  |  |  |  |  |  |  |  |  |  |  |  |  |  |  |  |  |  |  |  |  |  |  |  |  |  |  |  |  |  |  |  |  |  |  |  |  |  |  |  |  |  |  |  |  |  |  |  |  |  |  |  |  |  |  |  |  |  |  |  |  |  |  |  |  |  |  |  |  |  |  |  |  |  |  |  |  |  |  |  |  |  |  |  |  |  |  |  |  |  |  |  |  |  |  |  |  |  |  |  |  |  |  |  |  |  |  |  |  |  |  |  |  |  |  |  |  |  |  |  |  |  |  |  |  |  |  |  |  |  |  |  |  |  |  |  |  |  |  |  |  |  |  |  |  |  |  |  |  |  |  |  |  |  |  |  |  |  |  |  |  |  |  |  |  |  |  |  |  |  |  |  |  |  |  |  |  |  |  |  |  |  |  |  |  |  |  |  |  |  |  |  |  |  |  |  |  |  |  |  |  |  |  |  |  |  |  |  |  |  |  |  |  |  |  |  |  |  |  |  |  |  |  |  |  |  |  |  |  |  |  |  |  |  |  |  |  |  |  |  |  |  |  |  |  |  |  |  |  |  |  |  |  |  |  |  |  |  |  |  |  |  |  |  |  |  |  |  |  |  |  |  |  |  |  |  |  |  |  |  |  |  |  |  |  |  |  |  |  |  |  |  |  |  |  |  |  |  |  |  |  |  |  |  |  |  |  |  |  |  |  |  |  |  |  |  |  |  |  |  |  |  |  |  |  |  |  |  |  |  |  |  |  |  |  |  |  |  |  |  |  |  |  |  |  |  |  |  |  |  |  |  |  |  |  |  |  |  |  |  |  |  |  |  |  |  |  |  |  |  |  |  |  |  |  |  |  |  |  |  |  |  |  |  |  |  |  |  |  |  |  |  |  |  |  |  |  |  |  |  |  |  |  |  |  |  |  |  |  |  |  |  |  |  |  |  |  |  |  |  |  |  |  |  |  |  |  |  |  |  |  |  |  |  |  |  |  |  |  |  |  |  |  |  |  |  |  |  |  |  |  |  |  |  |  |  |  |  |  |  |  |  |  |  |  |  |  |  |  |  |  |  |  |  |  |  |  |  |  |  |  |  |  |  |  |  |  |  |  |  |  |  |  |  |  |  |  |  |  |  |  |  |  |  |  |  |  |  |  |  |  |  |  |  |  |  |  |  |  |  |  |  |  |  |  |  |  |  |  |  |  |  |  |  |  |  |  |  |  |  |  |  |  |  |  |  |  |  |  |  |  |  |  |  |  |  |  |  |  |  |  |  |  |  |  |  |  |  |  |  |  |  |  |  |  |  |  |  |  |  |  |  |  |  |  |  |  |  |  |  |  |  |  |  |  |  |  |  |  |  |  |  |  |  |  |  |  |  |  |  |  |  |  |  |  |  |  |  |  |  |  |  |  |  |  |  |  |  |  |  |  |  |  |  |  |  |  |  |  |  |  |  |  |  |  |  |  |  |  |  |  |  |  |  |  |  |  |  |  |  |  |  |  |  |  |  |  |  |  |  |  |  |  |  |  |  |  |  |  |  |  |  |  |  |  |  |  |  |  |  |  |  |  |  |  |  |  |  |  |  |  |  |  |  |  |  |  |  |  |  |  |  |  |  |  |  |  |  |  |  |  |  |  |  |  |  |  |  |  |  |  |  |  |  |  |  |  |  |  |  |  |  |  |  |  |  |  |  |  |  |  |  |  |  |  |  |  |  |  |  |  |  |  |  |  |  |  |  |  |  |  |  |  |  |  |  |  |  |  |  |  |  |  |  |  |  |  |  |  |  |  |  |  |  |  |  |  |  |  |  |  |  |  |  |  |  |  |  |  |  |  |  |  |  |  |  |  |  |  |  |  |  |  |  |  |  |  |  |  |  |  |  |  |  |  |  |  |  |  |  |  |  |  |  |  |  |  |  |  |  |  |  |  |  |  |  |  |  |  |  |  |  |  |  |  |  |  |  |  |  |  |  |  |  |  |  |  |  |  |  |  |  |  |  |  |  |  |  |  |  |  |  |  |  |  |  |  |  |  |  |  |  |  |  |  |  |  |  |  |  |  |  |  |  |  |  |  |  |  |  |  |  |  |  |  |  |  |  |  |  |  |  |  |  |  |  |  |  |  |  |  |  |  |  |  |  |  |  |  |  |  |  |  |  |  |  |  |  |  |  |  |  |  |  |  |  |  |  |  |  |  |  |  |  |  |  |  |  |  |  |  |  |  |  |  |  |  |  |  |  |  |  |  |  |  |  |  |  |  |  |  |  |  |  |  |  |  |  |  |  |  |  |  |  |  |  |  |  |  |  |  |  |  |  |  |  |  |  |  |  |  |  |  |  |  |  |  |  |  |  |  |  |  |  |  |  |  |  |  |  |  |  |  |  |  |  |  |  |  |  |  |  |  |  |  |  |  |  |  |  |  |  |  |  |  |  |  |  |  |  |  |  |  |  |  |  |  |  |  |  |  |  |  |  |  |  |  |  |  |  |  |  |  |  |  |  |  |  |  |  |  |  |  |  |  |  |  |  |  |  |  |  |  |  |  |  |  |  |  |  |  |  |  |  |  |  |  |  |  |  |  |  |  |  |  |  |  |  |  |  |  |  |  |  |  |  |  |  |  |  |  |  |  |  |  |  |  |  |  |  |  |  |  |  |  |  |  |  |  |  |  |  |  |  |  |  |  |  |  |  |  |  |  |  |  |  |  |  |  |  |  |  |  |  |  |  |  |  |  |  |  |  |  |  |  |  |  |  |  |  |  |  |  |  |  |  |  |  |  |  |  |  |  |  |  |  |  |  |  |  |  |  |  |  |  |  |  |  |  |  |  |  |  |  |  |  |  |  |  |  |  |  |  |  |  |  |  |  |  |  |  |  |  |  |  |  |  |  |  |  |  |  |  |  |  |  |  |  |  |  |  |  |  |  |  |  |  |  |  |  |  |  |  |  |  |  |  |  |  |  |  |  |  |  |  |  |  |  |  |  |  |  |  |  |  |  |  |  |  |  |  |  |  |  |  |  |  |  |  |  |  |  |  |  |  |  |  |  |  |  |  |  |  |  |  |  |  |  |  |  |  |  |  |  |  |  |  |  |  |  |  |  |  |  |  |  |  |  |  |  |  |  |  |  |  |  |  |  |  |  |  |  |  |  |  |  |  |  |  |  |  |  |  |  |  |  |  |  |  |  |  |  |  |  |  |  |  |  |  |  |  |  |  |  |  |  |  |  |  |  |  |  |  |  |  |  |  |  |  |  |  |  |  |  |  |  |  |  |  |  |  |  |  |  |  |  |  |  |  |  |  |  |  |  |  |  |  |  |  |  |  |  |  |  |  |  |  |  |  |  |  |  |  |  |  |  |  |  |  |  |  |  |  |  |  |  |  |  |  |  |  |  |  |  |  |  |  |  |  |  |  |  |  |  |  |  |  |  |  |  |  |  |  |  |  |  |  |  |  |  |  |  |  |  |  |  |  |  |  |  |  |  |  |  |  |  |  |  |  |  |  |  |  |  |  |  |  |  |  |  |  |  |  |  |  |  |  |  |  |  |  |  |  |  |  |  |  |  |  |  |  |  |  |  |  |  |  |  |  |  |  |  |  |  |  |  |  |  |  |  |  |  |  |  |  |  |  |  |  |  |  |  |  |  |  |  |  |  |  |  |  |  |  |  |  |  |  |  |  |  |  |  |  |  |  |  |  |  |  |  |  |  |  |  |  |  |  |  |  |  |  |  |  |  |  |  |  |  |  |  |  |  |  |  |  |  |  |  |  |  |  |  |  |  |  |  |  |  |  |  |  |  |  |  |  |  |  |  |  |  |  |  |  |  |  |  |  |  |  |  |  |  |  |  |  |  |  |  |  |  |  |  |  |  |  |  |  |  |  |  |  |  |  |  |  |  |  |  |  |  |  |  |  |  |  |  |  |  |  |  |  |  |  |  |  |  |  |  |  |  |  |  |  |  |  |  |  |  |  |  |  |  |  |  |  |  |  |  |  |  |  |  |  |  |  |  |  |  |  |  |  |  |  |  |  |  |  |  |  |  |  |  |  |  |  |  |  |  |  |  |  |  |  |  |  |  |  |  |  |  |  |  |  |  |  |  |  |  |  |  |  |  |  |  |  |  |  |  |  |  |  |  |  |  |  |  |  |  |  |  |  |  |  |  |  |  |  |  |  |  |  |  |  |  |  |  |  |  |  |  |  |  |  |  |  |  |  |  |  |  |  |  |  |  |  |  |  |  |  |  |  |  |  |  |  |  |  |  |  |  |  |  |  |  |  |  |  |  |  |  |  |  |  |  |  |  |  |  |  |  |  |  |  |  |  |  |  |  |  |  |  |  |  |  |  |  |  |  |  |  |  |  |  |  |  |  |  |  |  |  |  |  |  |  |  |  |  |  |  |  |  |  |  |  |  |  |  |  |  |  |  |  |  |  |  |  |  |  |  |  |  |  |  |  |  |  |  |  |  |  |  |  |  |  |  |  |  |  |  |  |  |  |  |  |  |  |  |  |  |  |  |  |  |  |  |  |  |  |  |  |  |  |  |  |  |  |  |  |  |  |  |  |  |  |  |  |  |  |  |  |  |  |  |  |  |  |  |  |  |  |  |  |  |  |  |  |  |  |  |  |  |  |  |  |  |  |  |  |  |  |  |  |  |  |  |  |  |  |  |  |  |  |  |  |  |  |  |  |  |  |  |  |  |  |  |  |  |  |  |  |  |  |  |  |  |  |  |  |  |  |  |  |  |  |  |  |  |  |  |  |  |  |  |  |  |  |  |  |  |  |  |  |  |  |  |  |  |  |  |  |  |  |  |  |  |  |  |  |  |  |  |  |  |  |  |  |  |  |  |  |  |  |  |  |  |  |  |  |  |  |  |  |  |  |  |  |  |  |  |  |  |  |  |  |  |  |  |  |  |  |  |  |  |  |  |  |  |  |  |  |  |  |  |  |  |  |  |  |  |  |  |  |  |  |  |  |  |  |  |  |  |  |  |  |  |  |  |  |  |  |  |  |  |  |  |  |  |  |  |  |  |  |  |  |  |  |  |  |  |  |  |  |  |  |  |  |  |  |  |  |  |  |  |  |  |  |  |  |  |  |  |  |  |  |  |  |  |  |  |  |  |  |  |  |  |  |  |  |  |  |  |  |  |  |  |  |  |  |  |  |  |  |  |  |  |  |  |  |  |  |  |  |  |  |  |  |  |  |  |  |  |  |  |  |  |  |  |  |  |  |  |  |  |  |  |  |  |  |  |  |  |  |  |  |  |  |  |  |  |  |  |  |  |  |  |  |  |  |  |  |  |  |  |  |  |  |  |  |  |  |  |  |  |  |  |  |  |  |  |  |  |  |  |  |  |  |  |  |  |  |  |  |  |  |  |  |  |  |  |  |  |  |  |  |  |  |  |  |  |  |  |  |  |  |  |  |  |  |  |  |  |  |  |  |  |  |  |  |  |  |  |  |  |  |  |  |  |  |  |  |  |  |  |  |  |  |  |  |  |  |  |  |  |  |  |  |  |  |  |  |  |  |  |  |  |  |  |  |  |  |  |  |  |  |  |  |  |  |  |  |  |  |  |  |  |  |  |  |  |  |  |  |  |  |  |  |  |  |  |  |  |  |  |  |  |  |  |  |  |  |  |  |  |  |  |  |  |  |  |  |  |  |  |  |  |  |  |  |  |  |  |  |  |  |  |  |  |  |  |  |  |  |  |  |  |  |  |  |  |  |  |  |  |  |  |  |  |  |  |  |  |  |  |  |  |  |  |  |  |  |  |  |  |  |  |  |  |  |  |  |  |  |  |  |  |  |  |  |  |  |  |  |  |  |  |  |  |  |  |  |  |  |  |  |  |  |  |  |  |  |  |  |  |  |  |  |  |  |  |  |  |  |  |  |  |  |  |  |  |  |  |  |  |  |  |  |  |  |  |  |  |  |  |  |  |  |  |  |  |  |  |  |  |  |  |  |  |  |  |  |  |  |  |  |  |  |  |  |  |  |  |  |  |  |  |  |  |  |  |  |  |  |  |  |  |  |  |  |  |  |  |  |  |  |  |  |  |  |  |  |  |  |  |  |  |  |  |  |  |  |  |  |  |  |  |  |  |  |  |  |  |  |  |  |  |  |  |  |  |  |  |  |  |  |  |  |  |  |  |  |  |  |  |  |  |  |  |  |  |  |  |  |  |  |  |  |  |  |  |  |  |  |  |  |  |  |  |  |  |  |  |  |  |  |  |  |  |  |  |  |  |  |  |  |  |  |  |  |  |  |  |  |  |  |  |  |  |  |  |  |  |  |  |  |  |  |  |  |  |  |  |  |  |  |  |  |  |  |  |  |  |  |  |  |  |  |  |  |  |  |  |  |  |  |  |  |  |  |  |  |  |  |  |  |  |  |  |  |  |  |  |  |  |  |  |  |  |  |  |  |  |  |  |  |  |  |  |  |  |  |  |  |  |  |  |  |  |  |  |  |  |  |  |  |  |  |  |  |  |  |  |  |  |  |  |  |  |  |  |  |  |  |  |  |  |  |  |  |  |  |  |  |  |  |  |  |  |  |  |  |  |  |  |  |  |  |  |  |  |  |  |  |  |  |  |  |  |  |  |  |  |  |  |  |  |  |  |  |  |  |  |  |  |  |  |  |  |  |  |  |  |  |  |  |  |  |  |  |  |  |  |  |  |  |  |  |  |  |  |  |  |  |  |  |  |  |  |  |  |  |  |  |  |  |  |  |  |  |  |  |  |  |  |  |  |  |  |  |  |  |  |  |  |  |  |  |  |  |  |  |  |  |  |  |  |  |  |  |  |  |  |  |  |  |  |  |  |  |  |  |  |  |  |  |  |  |  |  |  |  |  |  |  |  |  |  |  |  |  |  |  |  |  |  |  |  |  |  |  |  |  |  |  |  |  |  |  |  |  |  |  |  |  |  |  |  |  |  |  |  |  |  |  |  |  |  |  |  |  |  |  |  |  |  |  |  |  |  |  |  |  |  |  |  |  |  |  |  |  |  |  |  |  |  |  |  |  |  |  |  |  |  |  |  |  |  |  |  |  |  |  |  |  |  |  |  |  |  |  |  |  |  |  |  |  |  |  |  |  |  |  |  |  |  |  |  |  |  |  |  |  |  |  |  |  |  |  |  |  |  |  |  |  |  |  |  |  |  |  |  |  |  |  |  |  |  |  |  |  |  |  |  |  |  |  |  |  |  |  |  |  |  |  |  |  |  |  |  |  |  |  |  |  |  |  |  |  |  |  |  |  |  |  |  |  |  |  |  |  |  |  |  |  |  |  |  |  |  |  |  |  |  |  |  |  |  |  |  |  |  |  |  |  |  |  |  |  |  |  |  |  |  |  |  |  |  |  |  |  |  |  |  |  |  |  |  |  |  |  |  |  |  |  |  |  |  |  |  |  |  |  |  |  |  |  |  |  |  |  |  |  |  |  |  |  |  |  |  |  |  |  |  |  |  |  |  |  |  |  |  |  |  |  |  |  |  |  |  |  |  |  |  |  |  |  |  |  |  |  |  |  |  |  |  |  |  |  |  |  |  |  |  |  |  |  |  |  |  |  |  |  |  |  |  |  |  |  |  |  |  |  |  |  |  |  |  |  |  |  |  |  |  |  |  |  |  |  |  |  |  |  |  |  |  |  |  |  |  |  |  |  |  |  |  |  |  |  |  |  |  |  |  |  |  |  |  |  |  |  |  |  |  |  |  |  |  |  |  |  |  |  |  |  |  |  |  |  |  |  |  |  |  |  |  |  |  |  |  |  |  |  |  |  |  |  |  |  |  |  |  |  |  |  |  |  |  |  |  |  |  |  |  |  |  |  |  |  |  |  |  |  |  |  |  |  |  |  |  |  |  |  |  |  |  |  |  |  |  |  |  |  |  |  |  |  |  |  |  |  |  |  |  |  |  |  |  |  |  |  |  |  |  |  |  |  |  |  |  |  |  |  |  |  |  |  |  |  |  |  |  |  |  |  |  |  |  |  |  |  |  |  |  |  |  |  |  |  |  |  |  |  |  |  |  |  |  |  |  |  |  |  |  |  |  |  |  |  |  |  |  |  |  |  |  |  |  |  |  |  |  |  |  |  |  |  |  |  |  |  |  |  |  |  |  |  |  |  |  |  |  |  |  |  |  |  |  |  |  |  |  |  |  |  |  |  |  |  |  |  |  |  |  |  |  |  |  |  |  |  |  |  |  |  |  |  |  |  |  |  |  |  |  |  |  |  |  |  |  |  |  |  |  |  |  |  |  |  |  |  |  |  |  |  |  |  |  |  |  |  |  |  |  |  |  |  |  |  |  |  |  |  |  |  |  |  |  |  |  |  |  |  |  |  |  |  |  |  |  |  |  |  |  |  |  |  |  |  |  |  |  |  |  |  |  |  |  |  |  |  |  |  |  |  |  |  |  |  |  |  |  |  |  |  |  |  |  |  |  |  |  |  |  |  |  |  |  |  |  |  |  |  |  |  |  |  |  |  |  |  |  |  |  |  |  |  |  |  |  |  |  |  |  |  |  |  |  |  |  |  |  |  |  |  |  |  |  |  |  |  |  |  |  |  |  |  |  |  |  |  |  |  |  |  |  |  |  |  |  |  |  |  |  |  |  |  |  |  |  |  |  |  |  |  |  |  |  |  |  |  |  |  |  |  |  |  |  |  |  |  |  |  |  |  |  |  |  |  |  |  |  |  |  |  |  |  |  |  |  |  |  |  |  |  |  |  |  |  |  |  |  |  |  |  |  |  |  |  |  |  |  |  |  |  |  |  |  |  |  |  |  |  |  |  |  |  |  |  |  |  |  |  |  |  |  |  |  |  |  |  |  |  |  |  |  |  |  |  |  |  |  |  |  |  |  |  |  |  |  |  |  |  |  |  |  |  |  |  |  |  |  |  |  |  |  |  |  |  |  |  |  |  |  |  |  |  |  |  |  |  |  |  |  |  |  |  |  |  |  |  |  |  |  |  |  |  |  |  |  |  |  |  |  |  |  |  |  |  |  |  |  |  |  |  |  |  |  |  |  |  |  |  |  |  |  |  |  |  |  |  |  |  |  |  |  |  |  |  |  |  |  |  |  |  |  |  |  |  |  |  |  |  |  |  |  |  |  |  |  |  |  |  |  |  |  |  |  |  |  |  |  |  |  |  |  |  |  |  |  |  |  |  |  |  |  |  |  |  |  |  |  |  |  |  |  |  |  |  |  |  |  |  |  |  |  |  |  |  |  |  |  |  |  |  |  |  |  |  |  |  |  |  |  |  |  |  |  |  |  |  |  |  |  |  |  |  |  |  |  |  |  |  |  |  |  |  |  |  |  |  |  |  |  |  |  |  |  |  |  |  |  |  |  |  |  |  |  |  |  |  |  |  |  |  |  |  |  |  |  |  |  |  |  |  |  |  |  |  |  |  |  |  |  |  |  |  |  |  |  |  |  |  |  |  |  |  |  |  |  |  |  |  |  |  |  |  |  |  |  |  |  |  |  |  |  |  |  |  |  |  |  |  |  |  |  |  |  |  |  |  |  |  |  |  |  |  |  |  |  |  |  |  |  |  |  |  |  |  |  |  |  |  |  |  |  |  |  |  |  |  |  |  |  |  |  |  |  |  |  |  |  |  |  |  |  |  |  |  |  |  |  |  |  |  |  |  |  |  |  |  |  |  |  |  |  |  |  |  |  |  |  |  |  |  |  |  |  |  |  |  |  |  |  |  |  |  |  |  |  |  |  |  |  |  |  |  |  |  |  |  |  |  |  |  |  |  |  |  |  |  |  |  |  |  |  |  |  |  |  |  |  |  |  |  |  |  |  |  |  |  |  |  |  |  |  |  |  |  |  |  |  |  |  |  |  |  |  |  |  |  |  |  |  |  |  |  |  |  |  |  |  |  |  |  |  |  |  |  |  |  |  |  |  |  |  |  |  |  |  |  |  |  |  |  |  |  |  |  |  |  |  |  |  |  |  |  |  |  |  |  |  |  |  |  |  |  |  |  |  |  |  |  |  |  |  |  |  |  |  |  |  |  |  |  |  |  |  |  |  |  |  |  |  |  |  |  |  |  |  |  |  |  |  |  |  |  |  |  |  |  |  |  |  |  |  |  |  |  |  |  |  |  |  |  |  |  |  |  |  |  |  |  |  |  |  |  |  |  |  |  |  |  |  |  |  |  |  |  |  |  |  |  |  |  |  |  |  |  |  |  |  |  |  |  |  |  |  |  |  |  |  |  |  |  |  |  |  |  |  |  |  |  |  |  |  |  |  |  |  |  |  |  |  |  |  |  |  |  |  |  |  |  |  |  |  |  |  |  |  |  |  |  |  |  |  |  |  |  |  |  |  |  |  |  |  |  |  |  |  |  |  |  |  |  |  |  |  |  |  |  |  |  |  |  |  |  |  |  |  |  |  |  |  |  |  |  |  |  |  |  |  |  |  |  |  |  |  |  |  |  |  |  |  |  |  |  |  |  |  |  |  |  |  |  |  |  |  |  |  |  |  |  |  |  |  |  |  |  |  |  |  |  |  |  |  |  |  |  |  |  |  |  |  |  |  |  |  |  |  |  |  |  |  |  |  |  |  |  |  |  |  |  |  |  |  |  |  |  |  |  |  |  |  |  |  |  |  |  |  |  |  |  |  |  |  |  |  |  |  |  |  |  |  |  |  |  |  |  |  |  |  |  |  |  |  |  |  |  |  |  |  |  |  |  |  |  |  |  |  |  |  |  |  |  |  |  |  |  |  |  |  |  |  |  |  |  |  |  |  |  |  |  |  |  |  |  |  |  |  |  |  |  |  |  |  |  |  |  |  |  |  |  |  |  |  |  |  |  |  |  |  |  |  |  |  |  |  |  |  |  |  |  |  |  |  |  |  |  |  |  |  |  |  |  |  |  |  |  |  |  |  |  |  |  |  |  |  |  |  |  |  |  |  |  |  |  |  |  |  |  |  |  |  |  |  |  |  |  |  |  |  |  |  |  |  |  |  |  |  |  |  |  |  |  |  |  |  |  |  |  |  |  |  |  |  |  |  |  |  |  |  |  |  |  |  |  |  |  |  |  |  |  |  |  |  |  |  |  |  |  |  |  |  |  |  |  |  |  |  |  |  |  |  |  |  |  |  |  |  |  |  |  |  |  |  |  |  |  |  |  |  |  |  |  |  |  |  |  |  |  |  |  |  |  |  |  |  |  |  |  |  |  |  |  |  |  |  |  |  |  |  |  |  |  |  |  |  |  |  |  |  |  |  |  |  |  |  |  |  |  |  |  |  |  |  |  |  |  |  |  |  |  |  |  |  |  |  |  |  |  |  |  |  |  |  |  |  |  |  |  |  |  |  |  |  |  |  |  |  |  |  |  |  |  |  |  |  |  |  |  |  |  |  |  |  |  |  |  |  |  |  |  |  |  |  |  |  |  |  |  |  |  |  |  |  |  |  |  |  |  |  |  |  |  |  |  |  |  |  |  |  |  |  |  |  |  |  |  |  |  |  |  |  |  |  |  |  |  |  |  |  |  |  |  |  |  |  |  |  |  |  |  |  |  |  |  |  |  |  |  |  |  |  |  |  |  |  |  |  |  |  |  |  |  |  |  |  |  |  |  |  |  |  |  |  |  |  |  |  |  |  |  |  |  |  |  |  |  |  |  |  |  |  |  |  |  |  |  |  |  |  |  |  |  |  |  |  |  |  |  |  |  |  |  |  |  |  |  |  |  |  |  |  |  |  |  |  |  |  |  |  |  |  |  |  |  |  |  |  |  |  |  |  |  |  |  |  |  |  |  |  |  |  |  |  |  |  |  |  |  |  |  |  |  |  |  |  |  |  |  |  |  |  |  |  |  |  |  |  |  |  |  |  |  |  |  |  |  |  |  |  |  |  |  |  |  |  |  |  |  |  |  |  |  |  |  |  |  |  |  |  |  |  |  |  |  |  |  |  |  |  |  |  |  |  |  |  |  |  |  |  |  |  |  |  |  |  |  |  |  |  |  |  |  |  |  |  |  |  |  |  |  |  |  |  |  |  |  |  |  |  |  |  |  |  |  |  |  |  |  |  |  |  |  |  |  |  |  |  |  |  |  |  |  |  |  |  |  |  |  |  |  |  |  |  |  |  |  |  |  |  |  |  |  |  |  |  |  |  |  |  |  |  |  |  |  |  |  |  |  |  |  |  |  |  |  |  |  |  |  |  |  |  |  |  |  |  |  |  |  |  |  |  |  |  |  |  |  |  |  |  |  |  |  |  |  |  |  |  |  |  |  |  |  |  |  |  |  |  |  |  |  |  |  |  |  |  |  |  |  |  |  |  |  |  |  |  |  |  |  |  |  |  |  |  |  |  |  |  |  |  |  |  |  |  |  |  |  |  |  |  |  |  |  |  |  |  |  |  |  |  |  |  |  |  |  |  |  |  |  |  |  |  |  |  |  |  |  |  |  |  |  |  |  |  |  |  |  |  |  |  |  |  |  |  |  |  |  |  |  |  |  |  |  |  |  |  |  |  |  |  |  |  |  |  |  |  |  |  |  |  |  |  |  |  |  |  |  |  |  |  |  |  |  |  |  |  |  |  |  |  |  |  |  |  |  |  |  |  |  |  |  |  |  |  |  |  |  |  |  |  |  |  |  |  |  |  |  |  |  |  |  |  |  |  |  |  |  |  |  |  |  |  |  |  |  |  |  |  |  |  |  |  |  |  |  |  |  |  |  |  |  |  |  |  |  |  |  |  |  |  |  |  |  |  |  |  |  |  |  |  |  |  |  |  |  |  |  |  |  |  |  |  |  |  |  |  |  |  |  |  |  |  |  |  |  |  |  |  |  |  |  |  |  |  |  |  |  |  |  |  |  |  |  |  |  |  |  |  |  |  |  |  |  |  |  |  |  |  |  |  |  |  |  |  |  |  |  |  |  |  |  |  |  |  |  |  |  |  |  |  |  |  |  |  |  |  |  |  |  |  |  |  |  |  |  |  |  |  |  |  |  |  |  |  |  |  |  |  |  |  |  |  |  |  |  |  |  |  |  |  |  |  |  |  |  |  |  |  |  |  |  |  |  |  |  |  |  |  |  |  |  |  |  |  |  |  |  |  |  |  |  |  |  |  |  |  |  |  |  |  |  |  |  |  |  |  |  |  |  |  |  |  |  |  |  |  |  |  |  |  |  |  |  |  |  |  |  |  |  |  |  |  |  |  |  |  |  |  |  |  |  |  |  |  |  |  |  |  |  |  |  |  |  |  |  |  |  |  |  |  |  |  |  |  |  |  |  |  |  |  |  |  |  |  |  |  |  |  |  |  |  |  |  |  |  |  |  |  |  |  |  |  |  |  |  |  |  |  |  |  |  |  |  |  |  |  |  |  |  |  |  |  |  |  |  |  |  |  |  |  |  |  |  |  |  |  |  |  |  |  |  |  |  |  |  |  |  |  |  |  |  |  |  |  |  |  |  |  |  |  |  |  |  |  |  |  |  |  |  |  |  |  |  |  |  |  |  |  |  |  |  |  |  |  |  |  |  |  |  |  |  |  |  |  |  |  |  |  |  |  |  |  |  |  |  |  |  |  |  |  |  |  |  |  |  |  |  |  |  |  |  |  |  |  |  |  |  |  |  |  |  |  |  |  |  |  |  |  |  |  |  |  |  |  |  |  |  |  |  |  |  |  |  |  |  |  |  |  |  |  |  |  |  |  |  |  |  |  |  |  |  |  |  |  |  |  |  |  |  |  |  |  |  |  |  |  |  |  |  |  |  |  |  |  |  |  |  |  |  |  |  |  |  |  |  |  |  |  |  |  |  |  |  |  |  |  |  |  |  |  |  |  |  |  |  |  |  |  |  |  |  |  |  |  |  |  |  |  |  |  |  |  |  |  |  |  |  |  |  |  |  |  |  |  |  |  |  |  |  |  |  |  |  |  |  |  |  |  |  |  |  |  |  |  |  |  |  |  |  |  |  |  |  |  |  |  |  |  |  |  |  |  |  |  |  |  |  |  |  |  |  |  |  |  |  |  |  |  |  |  |  |  |  |  |  |  |  |  |  |  |  |  |  |  |  |  |  |  |  |  |  |  |  |  |  |  |  |  |  |  |  |  |  |  |  |  |  |  |  |  |  |  |  |  |  |  |  |  |  |  |  |  |  |  |  |  |  |  |  |  |  |  |  |  |  |  |  |  |  |  |  |  |  |  |  |  |  |  |  |  |  |  |  |  |  |  |  |  |  |  |  |  |  |  |  |  |  |  |  |  |  |  |  |  |  |  |  |  |  |  |  |  |  |  |  |  |  |  |  |  |  |  |  |  |  |  |  |  |  |  |  |  |  |  |  |  |  |  |  |  |  |  |  |  |  |  |  |  |  |  |  |  |  |  |  |  |  |  |  |  |  |  |  |  |  |  |  |  |  |  |  |  |  |  |  |  |  |  |  |  |  |  |  |  |  |  |  |  |  |  |  |  |  |  |  |  |  |  |  |  |  |  |  |  |  |  |  |  |  |  |  |  |  |  |  |  |  |  |  |  |  |  |  |  |  |  |  |  |  |  |  |  |  |  |  |  |  |  |  |  |  |  |  |  |  |  |  |  |  |  |  |  |  |  |  |  |  |  |  |  |  |  |  |  |  |  |  |  |  |  |  |  |  |  |  |  |  |  |  |  |  |  |  |  |  |  |  |  |  |  |  |  |  |  |  |  |  |  |  |  |  |  |  |  |  |  |  |  |  |  |  |  |  |  |  |  |  |  |  |  |  |  |  |  |  |  |  |  |  |  |  |  |  |  |  |  |  |  |  |  |  |  |  |  |  |  |  |  |  |  |  |  |  |  |  |  |  |  |  |  |  |  |  |  |  |  |  |  |  |  |  |  |  |  |  |  |  |  |  |  |  |  |  |  |  |  |  |  |  |  |  |  |  |  |  |  |  |  |  |  |  |  |  |  |  |  |  |  |  |  |  |  |  |  |  |  |  |  |  |  |  |  |  |  |  |  |  |  |  |  |  |  |  |  |  |  |  |  |  |  |  |  |  |  |  |  |  |  |  |  |  |  |  |  |  |  |  |  |  |  |  |  |  |  |  |  |  |  |  |  |  |  |  |  |  |  |  |  |  |  |  |  |  |  |  |  |  |  |  |  |  |  |  |  |  |  |  |  |  |  |  |  |  |  |  |  |  |  |  |  |  |  |  |  |  |  |  |  |  |  |  |  |  |  |  |  |  |  |  |  |  |  |  |  |  |  |  |  |  |  |  |  |  |  |  |  |  |  |  |  |  |  |  |  |  |  |  |  |  |  |  |  |  |  |  |  |  |  |  |  |  |  |  |  |  |  |  |  |  |  |  |  |  |  |  |  |  |  |  |  |  |  |  |  |  |  |  |  |  |  |  |  |  |  |  |  |  |  |  |  |  |  |  |  |  |  |  |  |  |  |  |  |  |  |  |  |  |  |  |  |  |  |  |  |  |  |  |  |  |  |  |  |  |  |  |  |  |  |  |  |  |  |  |  |  |  |  |  |  |  |  |  |  |  |  |  |  |  |  |  |  |  |  |  |  |  |  |  |  |  |  |  |  |  |  |  |  |  |  |  |  |  |  |  |  |  |  |  |  |  |  |  |  |  |  |  |  |  |  |  |  |  |  |  |  |  |  |  |  |  |  |  |  |  |  |  |  |  |  |  |  |  |  |  |  |  |  |  |  |  |  |  |  |  |  |  |  |  |  |  |  |  |  |  |  |  |  |  |  |  |  |  |  |  |  |  |  |  |  |  |  |  |  |  |  |  |  |  |  |  |  |  |  |  |  |  |  |  |  |  |  |  |  |  |  |  |  |  |  |  |  |  |  |  |  |  |  |  |  |  |  |  |  |  |  |  |  |  |  |  |  |  |  |  |  |  |  |  |  |  |  |  |  |  |  |  |  |  |  |  |  |  |  |  |  |  |  |  |  |  |  |  |  |  |  |  |  |  |  |  |  |  |  |  |  |  |  |  |  |  |  |  |  |  |  |  |  |  |  |  |  |  |  |  |  |  |  |  |  |  |  |  |  |  |  |  |  |  |  |  |  |  |  |  |  |  |  |  |  |  |  |  |  |  |  |  |  |  |  |  |  |  |  |  |  |  |  |  |  |  |  |  |  |  |  |  |  |  |  |  |  |  |  |  |  |  |  |  |  |  |  |  |  |  |  |  |  |  |  |  |  |  |  |  |  |  |  |  |  |  |  |  |  |  |  |  |  |  |  |  |  |  |  |  |  |  |  |  |  |  |  |  |  |  |  |  |  |  |  |  |  |  |  |  |  |  |  |  |  |  |  |  |  |  |  |  |  |  |  |  |  |  |  |  |  |  |  |  |  |  |  |  |  |  |  |  |  |  |  |  |  |  |  |  |  |  |  |  |  |  |  |  |  |  |  |  |  |  |  |  |  |  |  |  |  |  |  |  |  |  |  |  |  |  |  |  |  |  |  |  |  |  |  |  |  |  |  |  |  |  |  |  |  |  |  |  |  |  |  |  |  |  |  |  |  |  |  |  |  |  |  |  |  |  |  |  |  |  |  |  |  |  |  |  |  |  |  |  |  |  |  |  |  |  |  |  |  |  |  |  |  |  |  |  |  |  |  |  |  |  |  |  |  |  |  |  |  |  |  |  |  |  |  |  |  |  |  |  |  |  |  |  |  |  |  |  |  |  |  |  |  |  |  |  |  |  |  |  |  |  |  |  |  |  |  |  |  |  |  |  |  |  |  |  |  |  |  |  |  |  |  |  |  |  |  |  |  |  |  |  |  |  |  |  |  |  |  |  |  |  |  |  |  |  |  |  |  |  |  |  |  |  |  |  |  |  |  |  |  |  |  |  |  |  |  |  |  |  |  |  |  |  |  |  |  |  |  |  |  |  |  |  |  |  |  |  |  |  |  |  |  |  |  |  |  |  |  |  |  |  |  |  |  |  |  |  |  |  |  |  |  |  |  |  |  |  |  |  |  |  |  |  |  |  |  |  |  |  |  |  |  |  |  |  |  |  |  |  |  |  |  |  |  |  |  |  |  |  |  |  |  |  |  |  |  |  |  |  |  |  |  |  |  |  |  |  |  |  |  |  |  |  |  |  |  |  |  |  |  |  |  |  |  |  |  |  |  |  |  |  |  |  |  |  |  |  |  |  |  |  |  |  |  |  |  |  |  |  |  |  |  |  |  |  |  |  |  |  |  |  |  |  |  |  |  |  |  |  |  |  |  |  |  |  |  |  |  |  |  |  |  |  |  |  |  |  |  |  |  |  |  |  |  |  |  |  |  |  |  |  |  |  |  |  |  |  |  |  |  |  |  |  |  |  |  |  |  |  |  |  |  |  |  |  |  |  |  |  |  |  |  |  |  |  |  |  |  |  |  |  |  |  |  |  |  |  |  |  |  |  |  |  |  |  |  |  |  |  |  |  |  |  |  |  |  |  |  |  |  |  |  |  |  |  |  |  |  |  |  |  |  |  |  |  |  |  |  |  |  |  |  |  |  |  |  |  |  |  |  |  |  |  |  |  |  |  |  |  |  |  |  |  |  |  |  |  |  |  |  |  |  |  |  |  |  |  |  |  |  |  |  |  |  |  |  |  |  |  |  |  |  |  |  |  |  |  |  |  |  |  |  |  |  |  |  |  |  |  |  |  |  |  |  |  |  |  |  |  |  |  |  |  |  |  |  |  |  |  |  |  |  |  |  |  |  |  |  |  |  |  |  |  |  |
| --- | --- | --- | --- | --- | --- | --- | --- | --- | --- | --- | --- | --- | --- | --- | --- | --- | --- | --- | --- | --- | --- | --- | --- | --- | --- | --- | --- | --- | --- | --- | --- | --- | --- | --- | --- | --- | --- | --- | --- | --- | --- | --- | --- | --- | --- | --- | --- | --- | --- | --- | --- | --- | --- | --- | --- | --- | --- | --- | --- | --- | --- | --- | --- | --- | --- | --- | --- | --- | --- | --- | --- | --- | --- | --- | --- | --- | --- | --- | --- | --- | --- | --- | --- | --- | --- | --- | --- | --- | --- | --- | --- | --- | --- | --- | --- | --- | --- | --- | --- | --- | --- | --- | --- | --- | --- | --- | --- | --- | --- | --- | --- | --- | --- | --- | --- | --- | --- | --- | --- | --- | --- | --- | --- | --- | --- | --- | --- | --- | --- | --- | --- | --- | --- | --- | --- | --- | --- | --- | --- | --- | --- | --- | --- | --- | --- | --- | --- | --- | --- | --- | --- | --- | --- | --- | --- | --- | --- | --- | --- | --- | --- | --- | --- | --- | --- | --- | --- | --- | --- | --- | --- | --- | --- | --- | --- | --- | --- | --- | --- | --- | --- | --- | --- | --- | --- | --- | --- | --- | --- | --- | --- | --- | --- | --- | --- | --- | --- | --- | --- | --- | --- | --- | --- | --- | --- | --- | --- | --- | --- | --- | --- | --- | --- | --- | --- | --- | --- | --- | --- | --- | --- | --- | --- | --- | --- | --- | --- | --- | --- | --- | --- | --- | --- | --- | --- | --- | --- | --- | --- | --- | --- | --- | --- | --- | --- | --- | --- | --- | --- | --- | --- | --- | --- | --- | --- | --- | --- | --- | --- | --- | --- | --- | --- | --- | --- | --- | --- | --- | --- | --- | --- | --- | --- | --- | --- | --- | --- | --- | --- | --- | --- | --- | --- | --- | --- | --- | --- | --- | --- | --- | --- | --- | --- | --- | --- | --- | --- | --- | --- | --- | --- | --- | --- | --- | --- | --- | --- | --- | --- | --- | --- | --- | --- | --- | --- | --- | --- | --- | --- | --- | --- | --- | --- | --- | --- | --- | --- | --- | --- | --- | --- | --- | --- | --- | --- | --- | --- | --- | --- | --- | --- | --- | --- | --- | --- | --- | --- | --- | --- | --- | --- | --- | --- | --- | --- | --- | --- | --- | --- | --- | --- | --- | --- | --- | --- | --- | --- | --- | --- | --- | --- | --- | --- | --- | --- | --- | --- | --- | --- | --- | --- | --- | --- | --- | --- | --- | --- | --- | --- | --- | --- | --- | --- | --- | --- | --- | --- | --- | --- | --- | --- | --- | --- | --- | --- | --- | --- | --- | --- | --- | --- | --- | --- | --- | --- | --- | --- | --- | --- | --- | --- | --- | --- | --- | --- | --- | --- | --- | --- | --- | --- | --- | --- | --- | --- | --- | --- | --- | --- | --- | --- | --- | --- | --- | --- | --- | --- | --- | --- | --- | --- | --- | --- | --- | --- | --- | --- | --- | --- | --- | --- | --- | --- | --- | --- | --- | --- | --- | --- | --- | --- | --- | --- | --- | --- | --- | --- | --- | --- | --- | --- | --- | --- | --- | --- | --- | --- | --- | --- | --- | --- | --- | --- | --- | --- | --- | --- | --- | --- | --- | --- | --- | --- | --- | --- | --- | --- | --- | --- | --- | --- | --- | --- | --- | --- | --- | --- | --- | --- | --- | --- | --- | --- | --- | --- | --- | --- | --- | --- | --- | --- | --- | --- | --- | --- | --- | --- | --- | --- | --- | --- | --- | --- | --- | --- | --- | --- | --- | --- | --- | --- | --- | --- | --- | --- | --- | --- | --- | --- | --- | --- | --- | --- | --- | --- | --- | --- | --- | --- | --- | --- | --- | --- | --- | --- | --- | --- | --- | --- | --- | --- | --- | --- | --- | --- | --- | --- | --- | --- | --- | --- | --- | --- | --- | --- | --- | --- | --- | --- | --- | --- | --- | --- | --- | --- | --- | --- | --- | --- | --- | --- | --- | --- | --- | --- | --- | --- | --- | --- | --- | --- | --- | --- | --- | --- | --- | --- | --- | --- | --- | --- | --- | --- | --- | --- | --- | --- | --- | --- | --- | --- | --- | --- | --- | --- | --- | --- | --- | --- | --- | --- | --- | --- | --- | --- | --- | --- | --- | --- | --- | --- | --- | --- | --- | --- | --- | --- | --- | --- | --- | --- | --- | --- | --- | --- | --- | --- | --- | --- | --- | --- | --- | --- | --- | --- | --- | --- | --- | --- | --- | --- | --- | --- | --- | --- | --- | --- | --- | --- | --- | --- | --- | --- | --- | --- | --- | --- | --- | --- | --- | --- | --- | --- | --- | --- | --- | --- | --- | --- | --- | --- | --- | --- | --- | --- | --- | --- | --- | --- | --- | --- | --- | --- | --- | --- | --- | --- | --- | --- | --- | --- | --- | --- | --- | --- | --- | --- | --- | --- | --- | --- | --- | --- | --- | --- | --- | --- | --- | --- | --- | --- | --- | --- | --- | --- | --- | --- | --- | --- | --- | --- | --- | --- | --- | --- | --- | --- | --- | --- | --- | --- | --- | --- | --- | --- | --- | --- | --- | --- | --- | --- | --- | --- | --- | --- | --- | --- | --- | --- | --- | --- | --- | --- | --- | --- | --- | --- | --- | --- | --- | --- | --- | --- | --- | --- | --- | --- | --- | --- | --- | --- | --- | --- | --- | --- | --- | --- | --- | --- | --- | --- | --- | --- | --- | --- | --- | --- | --- | --- | --- | --- | --- | --- | --- | --- | --- | --- | --- | --- | --- | --- | --- | --- | --- | --- | --- | --- | --- | --- | --- | --- | --- | --- | --- | --- | --- | --- | --- | --- | --- | --- | --- | --- | --- | --- | --- | --- | --- | --- | --- | --- | --- | --- | --- | --- | --- | --- | --- | --- | --- | --- | --- | --- | --- | --- | --- | --- | --- | --- | --- | --- | --- | --- | --- | --- | --- | --- | --- | --- | --- | --- | --- | --- | --- | --- | --- | --- | --- | --- | --- | --- | --- | --- | --- | --- | --- | --- | --- | --- | --- | --- | --- | --- | --- | --- | --- | --- | --- | --- | --- | --- | --- | --- | --- | --- | --- | --- | --- | --- | --- | --- | --- | --- | --- | --- | --- | --- | --- | --- | --- | --- | --- | --- | --- | --- | --- | --- | --- | --- | --- | --- | --- | --- | --- | --- | --- | --- | --- | --- | --- | --- | --- | --- | --- | --- | --- | --- | --- | --- | --- | --- | --- | --- | --- | --- | --- | --- | --- | --- | --- | --- | --- | --- | --- | --- | --- | --- | --- | --- | --- | --- | --- | --- | --- | --- | --- | --- | --- | --- | --- | --- | --- | --- | --- | --- | --- | --- | --- | --- | --- | --- | --- | --- | --- | --- | --- | --- | --- | --- | --- | --- | --- | --- | --- | --- | --- | --- | --- | --- | --- | --- | --- | --- | --- | --- | --- | --- | --- | --- | --- | --- | --- | --- | --- | --- | --- | --- | --- | --- | --- | --- | --- | --- | --- | --- | --- | --- | --- | --- | --- | --- | --- | --- | --- | --- | --- | --- | --- | --- | --- | --- | --- | --- | --- | --- | --- | --- | --- | --- | --- | --- | --- | --- | --- | --- | --- | --- | --- | --- | --- | --- | --- | --- | --- | --- | --- | --- | --- | --- | --- | --- | --- | --- | --- | --- | --- | --- | --- | --- | --- | --- | --- | --- | --- | --- | --- | --- | --- | --- | --- | --- | --- | --- | --- | --- | --- | --- | --- | --- | --- | --- | --- | --- | --- | --- | --- | --- | --- | --- | --- | --- | --- | --- | --- | --- | --- | --- | --- | --- | --- | --- | --- | --- | --- | --- | --- | --- | --- | --- | --- | --- | --- | --- | --- | --- | --- | --- | --- | --- | --- | --- | --- | --- | --- | --- | --- | --- | --- | --- | --- | --- | --- | --- | --- | --- | --- | --- | --- | --- | --- | --- | --- | --- | --- | --- | --- | --- | --- | --- | --- | --- | --- | --- | --- | --- | --- | --- | --- | --- | --- | --- | --- | --- | --- | --- | --- | --- | --- | --- | --- | --- | --- | --- | --- | --- | --- | --- | --- | --- | --- | --- | --- | --- | --- | --- | --- | --- | --- | --- | --- | --- | --- | --- | --- | --- | --- | --- | --- | --- | --- | --- | --- | --- | --- | --- | --- | --- | --- | --- | --- | --- | --- | --- | --- | --- | --- | --- | --- | --- | --- | --- | --- | --- | --- | --- | --- | --- | --- | --- | --- | --- | --- | --- | --- | --- | --- | --- | --- | --- | --- | --- | --- | --- | --- | --- | --- | --- | --- | --- | --- | --- | --- | --- | --- | --- | --- | --- | --- | --- | --- | --- | --- | --- | --- | --- | --- | --- | --- | --- | --- | --- | --- | --- | --- | --- | --- | --- | --- | --- | --- | --- | --- | --- | --- | --- | --- | --- | --- | --- | --- | --- | --- | --- | --- | --- | --- | --- | --- | --- | --- | --- | --- | --- | --- | --- | --- | --- | --- | --- | --- | --- | --- | --- | --- | --- | --- | --- | --- | --- | --- | --- | --- | --- | --- | --- | --- | --- | --- | --- | --- | --- | --- | --- | --- | --- | --- | --- | --- | --- | --- | --- | --- | --- | --- | --- | --- | --- | --- | --- | --- | --- | --- | --- | --- | --- | --- | --- | --- | --- | --- | --- | --- | --- | --- | --- | --- | --- | --- | --- | --- | --- | --- | --- | --- | --- | --- | --- | --- | --- | --- | --- | --- | --- | --- | --- | --- | --- | --- | --- | --- | --- | --- | --- | --- | --- | --- | --- | --- | --- | --- | --- | --- | --- | --- | --- | --- | --- | --- | --- | --- | --- | --- | --- | --- | --- | --- | --- | --- | --- | --- | --- | --- | --- | --- | --- | --- | --- | --- | --- | --- | --- | --- | --- | --- | --- | --- | --- | --- | --- | --- | --- | --- | --- | --- | --- | --- | --- | --- | --- | --- | --- | --- | --- | --- | --- | --- | --- | --- | --- | --- | --- | --- | --- | --- | --- | --- | --- | --- | --- | --- | --- | --- | --- | --- | --- | --- | --- | --- | --- | --- | --- | --- | --- | --- | --- | --- | --- | --- | --- | --- | --- | --- | --- | --- | --- | --- | --- | --- | --- | --- | --- | --- | --- | --- | --- | --- | --- | --- | --- | --- | --- | --- | --- | --- | --- | --- | --- | --- | --- | --- | --- | --- | --- | --- | --- | --- | --- | --- | --- | --- | --- | --- | --- | --- | --- | --- | --- | --- | --- | --- | --- | --- | --- | --- | --- | --- | --- | --- | --- | --- | --- | --- | --- | --- | --- | --- | --- | --- | --- | --- | --- | --- | --- | --- | --- | --- | --- | --- | --- | --- | --- | --- | --- | --- | --- | --- | --- | --- | --- | --- | --- | --- | --- | --- | --- | --- | --- | --- | --- | --- | --- | --- | --- | --- | --- | --- | --- | --- | --- | --- | --- | --- | --- | --- | --- | --- | --- | --- | --- | --- | --- | --- | --- | --- | --- | --- | --- | --- | --- | --- | --- | --- | --- | --- | --- | --- | --- | --- | --- | --- | --- | --- | --- | --- | --- | --- | --- | --- | --- | --- | --- | --- | --- | --- | --- | --- | --- | --- | --- | --- | --- | --- | --- | --- | --- | --- | --- | --- | --- | --- | --- | --- | --- | --- | --- | --- | --- | --- | --- | --- | --- | --- | --- | --- | --- | --- | --- | --- | --- | --- | --- | --- | --- | --- | --- | --- | --- | --- | --- | --- | --- | --- | --- | --- | --- | --- | --- | --- | --- | --- | --- | --- | --- | --- | --- | --- | --- | --- | --- | --- | --- | --- | --- | --- | --- | --- | --- | --- | --- | --- | --- | --- | --- | --- | --- | --- | --- | --- | --- | --- | --- | --- | --- | --- | --- | --- | --- | --- | --- | --- | --- | --- | --- | --- | --- | --- | --- | --- | --- | --- | --- | --- | --- | --- | --- | --- | --- | --- | --- | --- | --- | --- | --- | --- | --- | --- | --- | --- | --- | --- | --- | --- | --- | --- | --- | --- | --- | --- | --- | --- | --- | --- | --- | --- | --- | --- | --- | --- | --- | --- | --- | --- | --- | --- | --- | --- | --- | --- | --- | --- | --- | --- | --- | --- | --- | --- | --- | --- | --- | --- | --- | --- | --- | --- | --- | --- | --- | --- | --- | --- | --- | --- | --- | --- | --- | --- | --- | --- | --- | --- | --- | --- | --- | --- | --- | --- | --- | --- | --- | --- | --- | --- | --- | --- | --- | --- | --- | --- | --- | --- | --- | --- | --- | --- | --- | --- | --- | --- | --- | --- | --- | --- | --- | --- | --- | --- | --- | --- | --- | --- | --- | --- | --- | --- | --- | --- | --- | --- | --- | --- | --- | --- | --- | --- | --- | --- | --- | --- | --- | --- | --- | --- | --- | --- | --- | --- | --- | --- | --- | --- | --- | --- | --- | --- | --- | --- | --- | --- | --- | --- | --- | --- | --- | --- | --- | --- | --- | --- | --- | --- | --- | --- | --- | --- | --- | --- | --- | --- | --- | --- | --- | --- | --- | --- | --- | --- | --- | --- | --- | --- | --- | --- | --- | --- | --- | --- | --- | --- | --- | --- | --- | --- | --- | --- | --- | --- | --- | --- | --- | --- | --- | --- | --- | --- | --- | --- | --- | --- | --- | --- | --- | --- | --- | --- | --- | --- | --- | --- | --- | --- | --- | --- | --- | --- | --- | --- | --- | --- | --- | --- | --- | --- | --- | --- | --- | --- | --- | --- | --- | --- | --- | --- | --- | --- | --- | --- | --- | --- | --- | --- | --- | --- | --- | --- | --- | --- | --- | --- | --- | --- | --- | --- | --- | --- | --- | --- | --- | --- | --- | --- | --- | --- | --- | --- | --- | --- | --- | --- | --- | --- | --- | --- | --- | --- | --- | --- | --- | --- | --- | --- | --- | --- | --- | --- | --- | --- | --- | --- | --- | --- | --- | --- | --- | --- | --- | --- | --- | --- | --- | --- | --- | --- | --- | --- | --- | --- | --- | --- | --- | --- | --- | --- | --- | --- | --- | --- | --- | --- | --- | --- | --- | --- | --- | --- | --- | --- | --- | --- | --- | --- | --- | --- | --- | --- | --- | --- | --- | --- | --- | --- | --- | --- | --- | --- | --- | --- | --- | --- | --- | --- | --- | --- | --- | --- | --- | --- | --- | --- | --- | --- | --- | --- | --- | --- | --- | --- | --- | --- | --- | --- | --- | --- | --- | --- | --- | --- | --- | --- | --- | --- | --- | --- | --- | --- | --- | --- | --- | --- | --- | --- | --- | --- | --- | --- | --- | --- | --- | --- | --- | --- | --- | --- | --- | --- | --- | --- | --- | --- | --- | --- | --- | --- | --- | --- | --- | --- | --- | --- | --- | --- | --- | --- | --- | --- | --- | --- | --- | --- | --- | --- | --- | --- | --- | --- | --- | --- | --- | --- | --- | --- | --- | --- | --- | --- | --- | --- | --- | --- | --- | --- | --- | --- | --- | --- | --- | --- | --- | --- | --- | --- | --- | --- | --- | --- | --- | --- | --- | --- | --- | --- | --- | --- | --- | --- | --- | --- | --- | --- | --- | --- | --- | --- | --- | --- | --- | --- | --- | --- | --- | --- | --- | --- | --- | --- | --- | --- | --- | --- | --- | --- | --- | --- | --- | --- | --- | --- | --- | --- | --- | --- | --- | --- | --- | --- | --- | --- | --- | --- | --- | --- | --- | --- | --- | --- | --- | --- | --- | --- | --- | --- | --- | --- | --- | --- | --- | --- | --- | --- | --- | --- | --- | --- | --- | --- | --- | --- | --- | --- | --- | --- | --- | --- | --- | --- | --- | --- | --- | --- | --- | --- | --- | --- | --- | --- | --- | --- | --- | --- | --- | --- | --- | --- | --- | --- | --- | --- | --- | --- | --- | --- | --- | --- | --- | --- | --- | --- | --- | --- | --- | --- | --- | --- | --- | --- | --- | --- | --- | --- | --- | --- | --- | --- | --- | --- | --- | --- | --- | --- | --- | --- | --- | --- | --- | --- | --- | --- | --- | --- | --- | --- | --- | --- | --- | --- | --- | --- | --- | --- | --- | --- | --- | --- | --- | --- | --- | --- | --- | --- | --- | --- | --- | --- | --- | --- | --- | --- | --- | --- | --- | --- | --- | --- | --- | --- | --- | --- | --- | --- | --- | --- | --- | --- | --- | --- | --- | --- | --- | --- | --- | --- | --- | --- | --- | --- | --- | --- | --- | --- | --- | --- | --- | --- | --- | --- | --- | --- | --- | --- | --- | --- | --- | --- | --- | --- | --- | --- | --- | --- | --- | --- | --- | --- | --- | --- | --- | --- | --- | --- | --- | --- | --- | --- | --- | --- | --- | --- | --- | --- | --- | --- | --- | --- | --- | --- | --- | --- | --- | --- | --- | --- | --- | --- | --- | --- | --- | --- | --- | --- | --- | --- | --- | --- | --- | --- | --- | --- | --- | --- | --- | --- | --- | --- | --- | --- | --- | --- | --- | --- | --- | --- | --- | --- | --- | --- | --- | --- | --- | --- | --- | --- | --- | --- | --- | --- | --- | --- | --- | --- | --- | --- | --- | --- | --- | --- | --- | --- | --- | --- | --- | --- | --- | --- | --- | --- | --- | --- | --- | --- | --- | --- | --- | --- | --- | --- | --- | --- | --- | --- | --- | --- | --- | --- | --- | --- | --- | --- | --- | --- | --- | --- | --- | --- | --- | --- | --- | --- | --- | --- | --- | --- | --- | --- | --- | --- | --- | --- | --- | --- | --- | --- | --- | --- | --- | --- | --- | --- | --- | --- | --- | --- | --- | --- | --- | --- | --- | --- | --- | --- | --- | --- | --- | --- | --- | --- | --- | --- | --- | --- | --- | --- | --- | --- | --- | --- | --- | --- | --- | --- | --- | --- | --- | --- | --- | --- | --- | --- | --- | --- | --- | --- | --- | --- | --- | --- | --- | --- | --- | --- | --- | --- | --- | --- | --- | --- | --- | --- | --- | --- | --- | --- | --- | --- | --- | --- | --- | --- | --- | --- | --- | --- | --- | --- | --- | --- | --- | --- | --- | --- | --- | --- | --- | --- | --- | --- | --- | --- | --- | --- | --- | --- | --- | --- | --- | --- | --- | --- | --- | --- | --- | --- | --- | --- | --- | --- | --- | --- | --- | --- | --- | --- | --- | --- | --- | --- | --- | --- | --- | --- | --- | --- | --- | --- | --- | --- | --- | --- | --- | --- | --- | --- | --- | --- | --- | --- | --- | --- | --- | --- | --- | --- | --- | --- | --- | --- | --- | --- | --- | --- | --- | --- | --- | --- | --- | --- | --- | --- | --- | --- | --- | --- | --- | --- | --- | --- | --- | --- | --- | --- | --- | --- | --- | --- | --- | --- | --- | --- | --- | --- | --- | --- | --- | --- | --- | --- | --- | --- | --- | --- | --- | --- | --- | --- | --- | --- | --- | --- | --- | --- | --- | --- | --- | --- | --- | --- | --- | --- | --- | --- | --- | --- | --- | --- | --- | --- | --- | --- | --- | --- | --- | --- | --- | --- | --- | --- | --- | --- | --- | --- | --- | --- | --- | --- | --- | --- | --- | --- | --- | --- | --- | --- | --- | --- | --- | --- | --- | --- | --- | --- | --- | --- | --- | --- | --- | --- | --- | --- | --- | --- | --- | --- | --- | --- | --- | --- | --- | --- | --- | --- | --- | --- | --- | --- | --- | --- | --- | --- | --- | --- | --- | --- | --- | --- | --- | --- | --- | --- | --- | --- | --- | --- | --- | --- | --- | --- | --- | --- | --- | --- | --- | --- | --- | --- | --- | --- | --- | --- | --- | --- | --- | --- | --- | --- | --- | --- | --- | --- | --- | --- | --- | --- | --- | --- | --- | --- | --- | --- | --- | --- | --- | --- | --- | --- | --- | --- | --- | --- | --- | --- | --- | --- | --- | --- | --- | --- | --- | --- | --- | --- | --- | --- | --- | --- | --- | --- | --- | --- | --- | --- | --- | --- | --- | --- | --- | --- | --- | --- | --- | --- | --- | --- | --- | --- | --- | --- | --- | --- | --- | --- | --- | --- | --- | --- | --- | --- | --- | --- | --- | --- | --- | --- | --- | --- | --- | --- | --- | --- | --- | --- | --- | --- | --- | --- | --- | --- | --- | --- | --- | --- | --- | --- | --- | --- | --- | --- | --- | --- | --- | --- | --- | --- | --- | --- | --- | --- | --- | --- | --- | --- | --- | --- | --- | --- | --- | --- | --- | --- | --- | --- | --- | --- | --- | --- | --- | --- | --- | --- | --- | --- | --- | --- | --- | --- | --- | --- | --- | --- | --- | --- | --- | --- | --- | --- | --- | --- | --- | --- | --- | --- | --- | --- | --- | --- | --- | --- | --- | --- | --- | --- | --- | --- | --- | --- | --- | --- | --- | --- | --- | --- | --- | --- | --- | --- | --- | --- | --- | --- | --- | --- | --- | --- | --- | --- | --- | --- | --- | --- | --- | --- | --- | --- | --- | --- | --- | --- | --- | --- | --- | --- | --- | --- | --- | --- | --- | --- | --- | --- | --- | --- | --- | --- | --- | --- | --- | --- | --- | --- | --- | --- | --- | --- | --- | --- | --- | --- | --- | --- | --- | --- | --- | --- | --- | --- | --- | --- | --- | --- | --- | --- | --- | --- | --- | --- | --- | --- | --- | --- | --- | --- | --- | --- | --- | --- | --- | --- | --- | --- | --- | --- | --- | --- | --- | --- | --- | --- | --- | --- | --- | --- | --- | --- | --- | --- | --- | --- | --- | --- | --- | --- | --- | --- | --- | --- | --- | --- | --- | --- | --- | --- | --- | --- | --- | --- | --- | --- | --- | --- | --- | --- | --- | --- | --- | --- | --- | --- | --- | --- | --- | --- | --- | --- | --- | --- | --- | --- | --- | --- | --- | --- | --- | --- | --- | --- | --- | --- | --- | --- | --- | --- | --- | --- | --- | --- | --- | --- | --- | --- | --- | --- | --- | --- | --- | --- | --- | --- | --- | --- | --- | --- | --- | --- | --- | --- | --- | --- | --- | --- | --- | --- | --- | --- | --- | --- | --- | --- | --- | --- | --- | --- | --- | --- | --- | --- | --- | --- | --- | --- | --- | --- | --- | --- | --- | --- | --- | --- | --- | --- | --- | --- | --- | --- | --- | --- | --- | --- | --- | --- | --- | --- | --- | --- | --- | --- | --- | --- | --- | --- | --- | --- | --- | --- | --- | --- | --- | --- | --- | --- | --- | --- | --- | --- | --- | --- | --- | --- | --- | --- | --- | --- | --- | --- | --- | --- | --- | --- | --- | --- | --- | --- | --- | --- | --- | --- | --- | --- | --- | --- | --- | --- | --- | --- | --- | --- | --- | --- | --- | --- | --- | --- | --- | --- | --- | --- | --- | --- | --- | --- | --- | --- | --- | --- | --- | --- | --- | --- | --- | --- | --- | --- | --- | --- | --- | --- | --- | --- | --- | --- | --- | --- | --- | --- | --- | --- | --- | --- | --- | --- | --- | --- | --- | --- | --- | --- | --- | --- | --- | --- | --- | --- | --- | --- | --- | --- | --- | --- | --- | --- | --- | --- | --- | --- | --- | --- | --- | --- | --- | --- | --- | --- | --- | --- | --- | --- | --- | --- | --- | --- | --- | --- | --- | --- | --- | --- | --- | --- | --- | --- | --- | --- | --- | --- | --- | --- | --- | --- | --- | --- | --- | --- | --- | --- | --- | --- | --- | --- | --- | --- | --- | --- | --- | --- | --- | --- | --- | --- | --- | --- | --- | --- | --- | --- | --- | --- | --- | --- | --- | --- | --- | --- | --- | --- | --- | --- | --- | --- | --- | --- | --- | --- | --- | --- | --- | --- | --- | --- | --- | --- | --- | --- | --- | --- | --- | --- | --- | --- | --- | --- | --- | --- | --- | --- | --- | --- | --- | --- | --- | --- | --- | --- | --- | --- | --- | --- | --- | --- | --- | --- | --- | --- | --- | --- | --- | --- | --- | --- | --- | --- | --- | --- | --- | --- | --- | --- | --- | --- | --- | --- | --- | --- | --- | --- | --- | --- | --- | --- | --- | --- | --- | --- | --- | --- | --- | --- | --- | --- | --- | --- | --- | --- | --- | --- | --- | --- | --- | --- | --- | --- | --- | --- | --- | --- | --- | --- | --- | --- | --- | --- | --- | --- | --- | --- | --- | --- | --- | --- | --- | --- | --- | --- | --- | --- | --- | --- | --- | --- | --- | --- | --- | --- | --- | --- | --- | --- | --- | --- | --- | --- | --- | --- | --- | --- | --- | --- | --- | --- | --- | --- | --- | --- | --- | --- | --- | --- | --- | --- | --- | --- | --- | --- | --- | --- | --- | --- | --- | --- | --- | --- | --- | --- | --- | --- | --- | --- | --- | --- | --- | --- | --- | --- | --- | --- | --- | --- | --- | --- | --- | --- | --- | --- | --- | --- | --- | --- | --- | --- | --- | --- | --- | --- | --- | --- | --- | --- | --- | --- | --- | --- | --- | --- | --- | --- | --- | --- | --- | --- | --- | --- | --- | --- | --- | --- | --- | --- | --- | --- | --- | --- | --- | --- | --- | --- | --- | --- | --- | --- | --- | --- | --- | --- | --- | --- | --- | --- | --- | --- | --- | --- | --- | --- | --- | --- | --- | --- | --- | --- | --- | --- | --- | --- | --- | --- | --- | --- | --- | --- | --- | --- | --- | --- | --- | --- | --- | --- | --- | --- | --- | --- | --- | --- | --- | --- | --- | --- | --- | --- | --- | --- | --- | --- | --- | --- | --- | --- | --- | --- | --- | --- | --- | --- | --- | --- | --- | --- | --- | --- | --- | --- | --- | --- | --- | --- | --- | --- | --- | --- | --- | --- | --- | --- | --- | --- | --- | --- | --- | --- | --- | --- | --- | --- | --- | --- | --- | --- | --- | --- | --- | --- | --- | --- | --- | --- | --- | --- | --- | --- | --- | --- | --- | --- | --- | --- | --- | --- | --- | --- | --- | --- | --- | --- | --- | --- | --- | --- | --- | --- | --- | --- | --- | --- | --- | --- | --- | --- | --- | --- | --- | --- | --- | --- | --- | --- | --- | --- | --- | --- | --- | --- | --- | --- | --- | --- | --- | --- | --- | --- | --- | --- | --- | --- | --- | --- | --- | --- | --- | --- | --- | --- | --- | --- | --- | --- | --- | --- | --- | --- | --- | --- | --- | --- | --- | --- | --- | --- | --- | --- | --- | --- | --- | --- | --- | --- | --- | --- | --- | --- | --- | --- | --- | --- | --- | --- | --- | --- | --- | --- | --- | --- | --- | --- | --- | --- | --- | --- | --- | --- | --- | --- | --- | --- | --- | --- | --- | --- | --- | --- | --- | --- | --- | --- | --- | --- | --- | --- | --- | --- | --- | --- | --- | --- | --- | --- | --- | --- | --- | --- | --- | --- | --- | --- | --- | --- | --- | --- | --- | --- | --- | --- | --- | --- | --- | --- | --- | --- | --- | --- | --- | --- | --- | --- | --- | --- | --- | --- | --- | --- | --- | --- | --- | --- | --- | --- | --- | --- | --- | --- | --- | --- | --- | --- | --- | --- | --- | --- | --- | --- | --- | --- | --- | --- | --- | --- | --- | --- | --- | --- | --- | --- | --- | --- | --- | --- | --- | --- | --- | --- | --- | --- | --- | --- | --- | --- | --- | --- | --- | --- | --- | --- | --- | --- | --- | --- | --- | --- | --- | --- | --- | --- | --- | --- | --- | --- | --- | --- | --- | --- | --- | --- | --- | --- | --- | --- | --- | --- | --- | --- | --- | --- | --- | --- | --- | --- | --- | --- | --- | --- | --- | --- | --- | --- | --- | --- | --- | --- | --- | --- | --- | --- | --- | --- | --- | --- | --- | --- | --- | --- | --- | --- | --- | --- | --- | --- | --- | --- | --- | --- | --- | --- | --- | --- | --- | --- | --- | --- | --- | --- | --- | --- | --- | --- | --- | --- | --- | --- | --- | --- | --- | --- | --- | --- | --- | --- | --- | --- | --- | --- | --- | --- | --- | --- | --- | --- | --- | --- | --- | --- | --- | --- | --- | --- | --- | --- | --- | --- | --- | --- | --- | --- | --- | --- | --- | --- | --- | --- | --- | --- | --- | --- | --- | --- | --- | --- | --- | --- | --- | --- | --- | --- | --- | --- | --- | --- | --- | --- | --- | --- | --- | --- | --- | --- | --- | --- | --- | --- | --- | --- | --- | --- | --- | --- | --- | --- | --- | --- | --- | --- | --- | --- | --- | --- | --- | --- | --- | --- | --- | --- | --- | --- | --- | --- | --- | --- | --- | --- | --- | --- | --- | --- | --- | --- | --- | --- | --- | --- | --- | --- | --- | --- | --- | --- | --- | --- | --- | --- | --- | --- | --- | --- | --- | --- | --- | --- | --- | --- | --- | --- | --- | --- | --- | --- | --- | --- | --- | --- | --- | --- | --- | --- | --- | --- | --- | --- | --- | --- | --- | --- | --- | --- | --- | --- | --- | --- | --- | --- | --- | --- | --- | --- | --- | --- | --- | --- | --- | --- | --- | --- | --- | --- | --- | --- | --- | --- | --- | --- | --- | --- | --- | --- | --- | --- | --- | --- | --- | --- | --- | --- | --- | --- | --- | --- | --- | --- | --- | --- | --- | --- | --- | --- | --- | --- | --- | --- | --- | --- | --- | --- | --- | --- | --- | --- | --- | --- | --- | --- | --- | --- | --- | --- | --- | --- | --- | --- | --- | --- | --- | --- | --- | --- | --- | --- | --- | --- | --- | --- | --- | --- | --- | --- | --- | --- | --- | --- | --- | --- | --- | --- | --- | --- | --- | --- | --- | --- | --- | --- | --- | --- | --- | --- | --- | --- | --- | --- | --- | --- | --- | --- | --- | --- | --- | --- | --- | --- | --- | --- | --- | --- | --- | --- | --- | --- | --- | --- | --- | --- | --- | --- | --- | --- | --- | --- | --- | --- | --- | --- | --- | --- | --- | --- | --- | --- | --- | --- | --- | --- | --- | --- | --- | --- | --- | --- | --- | --- | --- | --- | --- | --- | --- | --- | --- | --- | --- | --- | --- | --- | --- | --- | --- | --- | --- | --- | --- | --- | --- | --- | --- | --- | --- | --- | --- | --- | --- | --- | --- | --- | --- | --- | --- | --- | --- | --- | --- | --- | --- | --- | --- | --- | --- | --- | --- | --- | --- | --- | --- | --- | --- | --- | --- | --- | --- | --- | --- | --- | --- | --- | --- | --- | --- | --- | --- | --- | --- | --- | --- | --- | --- | --- | --- | --- | --- | --- | --- | --- | --- | --- | --- | --- | --- | --- | --- | --- | --- | --- | --- | --- | --- | --- | --- | --- | --- | --- | --- | --- | --- | --- | --- | --- | --- | --- | --- | --- | --- | --- | --- | --- | --- | --- | --- | --- | --- | --- | --- | --- | --- | --- | --- | --- | --- | --- | --- | --- | --- | --- | --- | --- | --- | --- | --- | --- | --- | --- | --- | --- | --- | --- | --- | --- | --- | --- | --- | --- | --- | --- | --- | --- | --- | --- | --- | --- | --- | --- | --- | --- | --- | --- | --- | --- | --- | --- | --- | --- | --- | --- | --- | --- | --- | --- | --- | --- | --- | --- | --- | --- | --- | --- | --- | --- | --- | --- | --- | --- | --- | --- | --- | --- | --- | --- | --- | --- | --- | --- | --- | --- | --- | --- | --- | --- | --- | --- | --- | --- | --- | --- | --- | --- | --- | --- | --- | --- | --- | --- | --- | --- | --- | --- | --- | --- | --- | --- | --- | --- | --- | --- | --- | --- | --- | --- | --- | --- | --- | --- | --- | --- | --- | --- | --- | --- | --- | --- | --- | --- | --- | --- | --- | --- | --- | --- | --- | --- | --- | --- | --- | --- | --- | --- | --- | --- | --- | --- | --- | --- | --- | --- | --- | --- | --- | --- | --- | --- | --- | --- | --- | --- | --- | --- | --- | --- | --- | --- | --- | --- | --- | --- | --- | --- | --- | --- | --- | --- | --- | --- | --- | --- | --- | --- | --- | --- | --- | --- | --- | --- | --- | --- | --- | --- | --- | --- | --- | --- | --- | --- | --- | --- | --- | --- | --- | --- | --- | --- | --- | --- | --- | --- | --- | --- | --- | --- | --- | --- | --- | --- | --- | --- | --- | --- | --- | --- | --- | --- | --- | --- | --- | --- | --- | --- | --- | --- | --- | --- | --- | --- | --- | --- | --- | --- | --- | --- | --- | --- | --- | --- | --- | --- | --- | --- | --- | --- | --- | --- | --- | --- | --- | --- | --- | --- | --- | --- | --- | --- | --- | --- | --- | --- | --- | --- | --- | --- | --- | --- | --- | --- | --- | --- | --- | --- | --- | --- | --- | --- | --- | --- | --- | --- | --- | --- | --- | --- | --- | --- | --- | --- | --- | --- | --- | --- | --- | --- | --- | --- | --- | --- | --- | --- | --- | --- | --- | --- | --- | --- | --- | --- | --- | --- | --- | --- | --- | --- | --- | --- | --- | --- | --- | --- | --- | --- | --- | --- | --- | --- | --- | --- | --- | --- | --- | --- | --- | --- | --- | --- | --- | --- | --- | --- | --- | --- | --- | --- | --- | --- | --- | --- | --- | --- | --- | --- | --- | --- | --- | --- | --- | --- | --- | --- | --- | --- | --- | --- | --- | --- | --- | --- | --- | --- | --- | --- | --- | --- | --- | --- | --- | --- | --- | --- | --- | --- | --- | --- | --- | --- | --- | --- | --- | --- | --- | --- | --- | --- | --- | --- | --- | --- | --- | --- | --- | --- | --- | --- | --- | --- | --- | --- | --- | --- | --- | --- | --- | --- | --- | --- | --- | --- | --- | --- | --- | --- | --- | --- | --- | --- | --- | --- | --- | --- | --- | --- | --- | --- | --- | --- | --- | --- | --- | --- | --- | --- | --- | --- | --- | --- | --- | --- | --- | --- | --- | --- | --- | --- | --- | --- | --- | --- | --- | --- | --- | --- | --- | --- | --- | --- | --- | --- | --- | --- | --- | --- | --- | --- | --- | --- | --- | --- | --- | --- | --- | --- | --- | --- | --- | --- | --- | --- | --- | --- | --- | --- | --- | --- | --- | --- | --- | --- | --- | --- | --- | --- | --- | --- | --- | --- | --- | --- | --- | --- | --- | --- | --- | --- | --- | --- | --- | --- | --- | --- | --- | --- | --- | --- | --- | --- | --- | --- | --- | --- | --- | --- | --- | --- | --- | --- | --- | --- | --- | --- | --- | --- | --- | --- | --- | --- | --- | --- | --- | --- | --- | --- | --- | --- | --- | --- | --- | --- | --- | --- | --- | --- | --- | --- | --- | --- | --- | --- | --- | --- | --- | --- | --- | --- | --- | --- | --- | --- | --- | --- | --- | --- | --- | --- | --- | --- | --- | --- | --- | --- | --- | --- | --- | --- | --- | --- | --- | --- | --- | --- | --- | --- | --- | --- | --- | --- | --- | --- | --- | --- | --- | --- | --- | --- | --- | --- | --- | --- | --- | --- | --- | --- | --- | --- | --- | --- | --- | --- | --- | --- | --- | --- | --- | --- | --- | --- | --- | --- | --- | --- | --- | --- | --- | --- | --- | --- | --- | --- | --- | --- | --- | --- | --- | --- | --- | --- | --- | --- | --- | --- | --- | --- | --- | --- | --- | --- | --- | --- | --- | --- | --- | --- | --- | --- | --- | --- | --- | --- | --- | --- | --- | --- | --- | --- | --- | --- | --- | --- | --- | --- | --- | --- | --- | --- | --- | --- | --- | --- | --- | --- | --- | --- | --- | --- | --- | --- | --- | --- | --- | --- | --- | --- | --- | --- | --- | --- | --- | --- | --- | --- | --- | --- | --- | --- | --- | --- | --- | --- | --- | --- | --- | --- | --- | --- | --- | --- | --- | --- | --- | --- | --- | --- | --- | --- | --- | --- | --- | --- | --- | --- | --- | --- | --- | --- | --- | --- | --- | --- | --- | --- | --- | --- | --- | --- | --- | --- | --- | --- | --- | --- | --- | --- | --- | --- | --- | --- | --- | --- | --- | --- | --- | --- | --- | --- | --- | --- | --- | --- | --- | --- | --- | --- | --- | --- | --- | --- | --- | --- | --- | --- | --- | --- | --- | --- | --- | --- | --- | --- | --- | --- | --- | --- | --- | --- | --- | --- | --- | --- | --- | --- | --- | --- | --- | --- | --- | --- | --- | --- | --- | --- | --- | --- | --- | --- | --- | --- | --- | --- | --- | --- | --- | --- | --- | --- | --- | --- | --- | --- | --- | --- | --- | --- | --- | --- | --- | --- | --- | --- | --- | --- | --- | --- | --- | --- | --- | --- | --- | --- | --- | --- | --- | --- | --- | --- | --- | --- | --- | --- | --- | --- | --- | --- | --- | --- | --- | --- | --- | --- | --- | --- | --- | --- | --- | --- | --- | --- | --- | --- | --- | --- | --- | --- | --- | --- | --- | --- | --- | --- | --- | --- | --- | --- | --- | --- | --- | --- | --- | --- | --- | --- | --- | --- | --- | --- | --- | --- | --- | --- | --- | --- | --- | --- | --- | --- | --- | --- | --- | --- | --- | --- | --- | --- | --- | --- | --- | --- | --- | --- | --- | --- | --- | --- | --- | --- | --- | --- | --- | --- | --- | --- | --- | --- | --- | --- | --- | --- | --- | --- | --- | --- | --- | --- | --- | --- | --- | --- | --- | --- | --- | --- | --- | --- | --- | --- | --- | --- | --- | --- | --- | --- | --- | --- | --- | --- | --- | --- | --- | --- | --- | --- | --- | --- | --- | --- | --- | --- | --- | --- | --- | --- | --- | --- | --- | --- | --- | --- | --- | --- | --- | --- | --- | --- | --- | --- | --- | --- | --- | --- | --- | --- | --- | --- | --- | --- | --- | --- | --- | --- | --- | --- | --- | --- | --- | --- | --- | --- | --- | --- | --- | --- | --- | --- | --- | --- | --- | --- | --- | --- | --- | --- | --- | --- | --- | --- | --- | --- | --- | --- | --- | --- | --- | --- | --- | --- | --- | --- | --- | --- | --- | --- | --- | --- | --- | --- | --- | --- | --- | --- | --- | --- | --- | --- | --- | --- | --- | --- | --- | --- | --- | --- | --- | --- | --- | --- | --- | --- | --- | --- | --- | --- | --- | --- | --- | --- | --- | --- | --- | --- | --- | --- | --- | --- | --- | --- | --- | --- | --- | --- | --- | --- | --- | --- | --- | --- | --- | --- | --- | --- | --- | --- | --- | --- | --- | --- | --- | --- | --- | --- | --- | --- | --- | --- | --- | --- | --- | --- | --- | --- | --- | --- | --- | --- | --- | --- | --- | --- | --- | --- | --- | --- | --- | --- | --- | --- | --- | --- | --- | --- | --- | --- | --- | --- | --- | --- | --- | --- | --- | --- | --- | --- | --- | --- | --- | --- | --- | --- | --- | --- | --- | --- | --- | --- | --- | --- | --- | --- | --- | --- | --- | --- | --- | --- | --- | --- | --- | --- | --- | --- | --- | --- | --- | --- | --- | --- | --- | --- | --- | --- | --- | --- | --- | --- | --- | --- | --- | --- | --- | --- | --- | --- | --- | --- | --- | --- | --- | --- | --- | --- | --- | --- | --- | --- | --- | --- | --- | --- | --- | --- | --- | --- | --- | --- | --- | --- | --- | --- | --- | --- | --- | --- | --- | --- | --- | --- | --- | --- | --- | --- | --- | --- | --- | --- | --- | --- | --- | --- | --- | --- | --- | --- | --- | --- | --- | --- | --- | --- | --- | --- | --- | --- | --- | --- | --- | --- | --- | --- | --- | --- | --- | --- | --- | --- | --- | --- | --- | --- | --- | --- | --- | --- | --- | --- | --- | --- | --- | --- | --- | --- | --- | --- | --- | --- | --- | --- | --- | --- | --- | --- | --- | --- | --- | --- | --- | --- | --- | --- | --- | --- | --- | --- | --- | --- | --- | --- | --- | --- | --- | --- | --- | --- | --- | --- | --- | --- | --- | --- | --- | --- | --- | --- | --- | --- | --- | --- | --- | --- | --- | --- | --- | --- | --- | --- | --- | --- | --- | --- | --- | --- | --- | --- | --- | --- | --- | --- | --- | --- | --- | --- | --- | --- | --- | --- | --- | --- | --- | --- | --- | --- | --- | --- | --- | --- | --- | --- | --- | --- | --- | --- | --- | --- | --- | --- | --- | --- | --- | --- | --- | --- | --- | --- | --- | --- | --- | --- | --- | --- | --- | --- | --- | --- | --- | --- | --- | --- | --- | --- | --- | --- | --- | --- | --- | --- | --- | --- | --- | --- | --- | --- | --- | --- | --- | --- | --- | --- | --- | --- | --- | --- | --- | --- | --- | --- | --- | --- | --- | --- | --- | --- | --- | --- | --- | --- | --- | --- | --- | --- | --- | --- | --- | --- | --- | --- | --- | --- | --- | --- | --- | --- | --- | --- | --- | --- | --- | --- | --- | --- | --- | --- | --- | --- | --- | --- | --- | --- | --- | --- | --- | --- | --- | --- | --- | --- | --- | --- | --- | --- | --- | --- | --- | --- | --- | --- | --- | --- | --- | --- | --- | --- | --- | --- | --- | --- | --- | --- | --- | --- | --- | --- | --- | --- | --- | --- | --- | --- | --- | --- | --- | --- | --- | --- | --- | --- | --- | --- | --- | --- | --- | --- | --- | --- | --- | --- | --- | --- | --- | --- | --- | --- | --- | --- | --- | --- | --- | --- | --- | --- | --- | --- | --- | --- | --- | --- | --- | --- | --- | --- | --- | --- | --- | --- | --- | --- | --- | --- | --- | --- | --- | --- | --- | --- | --- | --- | --- | --- | --- | --- | --- | --- | --- | --- | --- | --- | --- | --- | --- | --- | --- | --- | --- | --- | --- | --- | --- | --- | --- | --- | --- | --- | --- | --- | --- | --- | --- | --- | --- | --- | --- | --- | --- | --- | --- | --- | --- | --- | --- | --- | --- | --- | --- | --- | --- | --- | --- | --- | --- | --- | --- | --- | --- | --- | --- | --- | --- | --- | --- | --- | --- | --- | --- | --- | --- | --- | --- | --- | --- | --- | --- | --- | --- | --- | --- | --- | --- | --- | --- | --- | --- | --- | --- | --- | --- | --- | --- | --- | --- | --- | --- | --- | --- | --- | --- | --- | --- | --- | --- | --- | --- | --- | --- | --- | --- | --- | --- | --- | --- | --- | --- | --- | --- | --- | --- | --- | --- | --- | --- | --- | --- | --- | --- | --- | --- | --- | --- | --- | --- | --- | --- | --- | --- | --- | --- | --- | --- | --- | --- | --- | --- | --- | --- | --- | --- | --- | --- | --- | --- | --- | --- | --- | --- | --- | --- | --- | --- | --- | --- | --- | --- | --- | --- | --- | --- | --- | --- | --- | --- | --- | --- | --- | --- | --- | --- | --- | --- | --- | --- | --- | --- | --- | --- | --- | --- | --- | --- | --- | --- | --- | --- | --- | --- | --- | --- | --- | --- | --- | --- | --- | --- | --- | --- | --- | --- | --- | --- | --- | --- | --- | --- | --- | --- | --- | --- | --- | --- | --- | --- | --- | --- | --- | --- | --- | --- | --- | --- | --- | --- | --- | --- | --- | --- | --- | --- | --- | --- | --- | --- | --- | --- | --- | --- | --- | --- | --- | --- | --- | --- | --- | --- | --- | --- | --- | --- | --- | --- | --- | --- | --- | --- | --- | --- | --- | --- | --- | --- | --- | --- | --- | --- | --- | --- | --- | --- | --- | --- | --- | --- | --- | --- | --- | --- | --- | --- | --- | --- | --- | --- | --- | --- | --- | --- | --- | --- | --- | --- | --- | --- | --- | --- | --- | --- | --- | --- | --- | --- | --- | --- | --- | --- | --- | --- | --- | --- | --- | --- | --- | --- | --- | --- | --- | --- | --- | --- | --- | --- | --- | --- | --- | --- | --- | --- | --- | --- | --- | --- | --- | --- | --- | --- | --- | --- | --- | --- | --- | --- | --- | --- | --- | --- | --- | --- | --- | --- | --- | --- | --- | --- | --- | --- | --- | --- | --- | --- | --- | --- | --- | --- | --- | --- | --- | --- | --- | --- | --- | --- | --- | --- | --- | --- | --- | --- | --- | --- | --- | --- | --- | --- | --- | --- | --- | --- | --- | --- | --- | --- | --- | --- | --- | --- | --- | --- | --- | --- | --- | --- | --- | --- | --- | --- | --- | --- | --- | --- | --- | --- | --- | --- | --- | --- | --- | --- | --- | --- | --- | --- | --- | --- | --- | --- | --- | --- | --- | --- | --- | --- | --- | --- | --- | --- | --- | --- | --- | --- | --- | --- | --- | --- | --- | --- | --- | --- | --- | --- | --- | --- | --- | --- | --- | --- | --- | --- | --- | --- | --- | --- | --- | --- | --- | --- | --- | --- | --- | --- | --- | --- | --- | --- | --- | --- | --- | --- | --- | --- | --- | --- | --- | --- | --- | --- | --- | --- | --- | --- | --- | --- | --- | --- | --- | --- | --- | --- | --- | --- | --- | --- | --- | --- | --- | --- | --- | --- | --- | --- | --- | --- | --- | --- | --- | --- | --- | --- | --- | --- | --- | --- | --- | --- | --- | --- | --- | --- | --- | --- | --- | --- | --- | --- | --- | --- | --- | --- | --- | --- | --- | --- | --- | --- | --- | --- | --- | --- | --- | --- | --- | --- | --- | --- | --- | --- | --- | --- | --- | --- | --- | --- | --- | --- | --- | --- | --- | --- | --- | --- | --- | --- | --- | --- | --- | --- | --- | --- | --- | --- | --- | --- | --- | --- | --- | --- | --- | --- | --- | --- | --- | --- | --- | --- | --- | --- | --- | --- | --- | --- | --- | --- | --- | --- | --- | --- | --- | --- | --- | --- | --- | --- | --- | --- | --- | --- | --- | --- | --- | --- | --- | --- | --- | --- | --- | --- | --- | --- | --- | --- | --- | --- | --- | --- | --- | --- | --- | --- | --- | --- | --- | --- | --- | --- | --- | --- | --- | --- | --- | --- | --- | --- | --- | --- | --- | --- | --- | --- | --- | --- | --- | --- | --- | --- | --- | --- | --- | --- | --- | --- | --- | --- | --- | --- | --- | --- | --- | --- | --- | --- | --- | --- | --- | --- | --- | --- | --- | --- | --- | --- | --- | --- | --- | --- | --- | --- | --- | --- | --- | --- | --- | --- | --- | --- | --- | --- | --- | --- | --- | --- | --- | --- | --- | --- | --- | --- | --- | --- | --- | --- | --- | --- | --- | --- | --- | --- | --- | --- | --- | --- | --- | --- | --- | --- | --- | --- | --- | --- | --- | --- | --- | --- | --- | --- | --- | --- | --- | --- | --- | --- | --- | --- | --- | --- | --- | --- | --- | --- | --- | --- | --- | --- | --- | --- | --- | --- | --- | --- | --- | --- | --- | --- | --- | --- | --- | --- | --- | --- | --- | --- | --- | --- | --- | --- | --- | --- | --- | --- | --- | --- | --- | --- | --- | --- | --- | --- | --- | --- | --- | --- | --- | --- | --- | --- | --- | --- | --- | --- | --- | --- | --- | --- | --- | --- | --- | --- | --- | --- | --- | --- | --- | --- | --- | --- | --- | --- | --- | --- | --- | --- | --- | --- | --- | --- | --- | --- | --- | --- | --- | --- | --- | --- | --- | --- | --- | --- | --- | --- | --- | --- | --- | --- | --- | --- | --- | --- | --- | --- | --- | --- | --- | --- | --- | --- | --- | --- | --- | --- | --- | --- | --- | --- | --- | --- | --- | --- | --- | --- | --- | --- | --- | --- | --- | --- | --- | --- | --- | --- | --- | --- | --- | --- | --- | --- | --- | --- | --- | --- | --- | --- | --- | --- | --- | --- | --- | --- | --- | --- | --- | --- | --- | --- | --- | --- | --- | --- | --- | --- | --- | --- | --- | --- | --- | --- | --- | --- | --- | --- | --- | --- | --- | --- | --- | --- | --- | --- | --- | --- | --- | --- | --- | --- | --- | --- | --- | --- | --- | --- | --- | --- | --- | --- | --- | --- | --- | --- | --- | --- | --- | --- | --- | --- | --- | --- | --- | --- | --- | --- | --- | --- | --- | --- | --- | --- | --- | --- | --- | --- | --- | --- | --- | --- | --- | --- | --- | --- | --- | --- | --- | --- | --- | --- | --- | --- | --- | --- | --- | --- | --- | --- | --- | --- | --- | --- | --- | --- | --- | --- | --- | --- | --- | --- | --- | --- | --- | --- | --- | --- | --- | --- | --- | --- | --- | --- | --- | --- | --- | --- | --- | --- | --- | --- | --- | --- | --- | --- | --- | --- | --- | --- | --- | --- | --- | --- | --- | --- | --- | --- | --- | --- | --- | --- | --- | --- | --- | --- | --- | --- | --- | --- | --- | --- | --- | --- | --- | --- | --- | --- | --- | --- | --- | --- | --- | --- | --- | --- | --- | --- | --- | --- | --- | --- | --- | --- | --- | --- | --- | --- | --- | --- | --- | --- | --- | --- | --- | --- | --- | --- | --- | --- | --- | --- | --- | --- | --- | --- | --- | --- | --- | --- | --- | --- | --- | --- | --- | --- | --- | --- | --- | --- | --- | --- | --- | --- | --- | --- | --- | --- | --- | --- | --- | --- | --- | --- | --- | --- | --- | --- | --- | --- | --- | --- | --- | --- | --- | --- | --- | --- | --- | --- | --- | --- | --- | --- | --- | --- | --- | --- | --- | --- | --- | --- | --- | --- | --- | --- | --- | --- | --- | --- | --- | --- | --- | --- | --- | --- | --- | --- | --- | --- | --- | --- | --- | --- | --- | --- | --- | --- | --- | --- | --- | --- | --- | --- | --- | --- | --- | --- | --- | --- | --- | --- | --- | --- | --- | --- | --- | --- | --- | --- | --- | --- | --- | --- | --- | --- | --- | --- | --- | --- | --- | --- | --- | --- | --- | --- | --- | --- | --- | --- | --- | --- | --- | --- | --- | --- | --- | --- | --- | --- | --- | --- | --- | --- | --- | --- | --- | --- | --- | --- | --- | --- | --- | --- | --- | --- | --- | --- | --- | --- | --- | --- | --- | --- | --- | --- | --- | --- | --- | --- | --- | --- | --- | --- | --- | --- | --- | --- | --- | --- | --- | --- | --- | --- | --- | --- | --- | --- | --- | --- | --- | --- | --- | --- | --- | --- | --- | --- | --- | --- | --- | --- | --- | --- | --- | --- | --- | --- | --- | --- | --- | --- | --- | --- | --- | --- | --- | --- | --- | --- | --- | --- | --- | --- | --- | --- | --- | --- | --- | --- | --- | --- | --- | --- | --- | --- | --- | --- | --- | --- | --- | --- | --- | --- | --- | --- | --- | --- | --- | --- | --- | --- | --- | --- | --- | --- | --- | --- | --- | --- | --- | --- | --- | --- | --- | --- | --- | --- | --- | --- | --- | --- | --- | --- | --- | --- | --- | --- | --- | --- | --- | --- | --- | --- | --- | --- | --- | --- | --- | --- | --- | --- | --- | --- | --- | --- | --- | --- | --- | --- | --- | --- | --- | --- | --- | --- | --- | --- | --- | --- | --- | --- | --- | --- | --- | --- | --- | --- | --- | --- | --- | --- | --- | --- | --- | --- | --- | --- | --- | --- | --- | --- | --- | --- | --- | --- | --- | --- | --- | --- | --- | --- | --- | --- | --- | --- | --- | --- | --- | --- | --- | --- | --- | --- | --- | --- | --- | --- | --- | --- | --- | --- | --- | --- | --- | --- | --- | --- | --- | --- | --- | --- | --- | --- | --- | --- | --- | --- | --- | --- | --- | --- | --- | --- | --- | --- | --- | --- | --- | --- | --- | --- | --- | --- | --- | --- | --- | --- | --- | --- | --- | --- | --- | --- | --- | --- | --- | --- | --- | --- | --- | --- | --- | --- | --- | --- | --- | --- | --- | --- | --- | --- | --- | --- | --- | --- | --- | --- | --- | --- | --- | --- | --- | --- | --- | --- | --- | --- | --- | --- | --- | --- | --- | --- | --- | --- | --- | --- | --- | --- | --- | --- | --- | --- | --- | --- | --- | --- | --- | --- | --- | --- | --- | --- | --- | --- | --- | --- | --- | --- | --- | --- | --- | --- | --- | --- | --- | --- | --- | --- | --- | --- | --- | --- | --- | --- | --- | --- | --- | --- | --- | --- | --- | --- | --- | --- | --- | --- | --- | --- | --- | --- | --- | --- | --- | --- | --- | --- | --- | --- | --- | --- | --- | --- | --- | --- | --- | --- | --- | --- | --- | --- | --- | --- | --- | --- | --- | --- | --- | --- | --- | --- | --- | --- | --- | --- | --- | --- | --- | --- | --- | --- | --- | --- | --- | --- | --- | --- | --- | --- | --- | --- | --- | --- | --- | --- | --- | --- | --- | --- | --- | --- | --- | --- | --- | --- | --- | --- | --- | --- | --- | --- | --- | --- | --- | --- | --- | --- | --- | --- | --- | --- | --- | --- | --- | --- | --- | --- | --- | --- | --- | --- | --- | --- | --- | --- | --- | --- | --- | --- | --- | --- | --- | --- | --- | --- | --- | --- | --- | --- | --- | --- | --- | --- | --- | --- | --- | --- | --- | --- | --- | --- | --- | --- | --- | --- | --- | --- | --- | --- | --- | --- | --- | --- | --- | --- | --- | --- | --- | --- | --- | --- | --- | --- | --- | --- | --- | --- | --- | --- | --- | --- | --- | --- | --- | --- | --- | --- | --- | --- | --- | --- | --- | --- | --- | --- | --- | --- | --- | --- | --- | --- | --- | --- | --- | --- | --- | --- | --- | --- | --- | --- | --- | --- | --- | --- | --- | --- | --- | --- | --- | --- | --- | --- | --- | --- | --- | --- | --- | --- | --- | --- | --- | --- | --- | --- | --- | --- | --- | --- | --- | --- | --- | --- | --- | --- | --- | --- | --- | --- | --- | --- | --- | --- | --- | --- | --- | --- | --- | --- | --- | --- | --- | --- | --- | --- | --- | --- | --- | --- | --- | --- | --- | --- | --- | --- | --- | --- | --- | --- | --- | --- | --- | --- | --- | --- | --- | --- | --- | --- | --- | --- | --- | --- | --- | --- | --- | --- | --- | --- | --- | --- | --- | --- | --- | --- | --- | --- | --- | --- | --- | --- | --- | --- | --- | --- | --- | --- | --- | --- | --- | --- | --- | --- | --- | --- | --- | --- | --- | --- | --- | --- | --- | --- | --- | --- | --- | --- | --- | --- | --- | --- | --- | --- | --- | --- | --- | --- | --- | --- | --- | --- | --- | --- | --- | --- | --- | --- | --- | --- | --- | --- | --- | --- | --- | --- | --- | --- | --- | --- | --- | --- | --- | --- | --- | --- | --- | --- | --- | --- | --- | --- | --- | --- | --- | --- | --- | --- | --- | --- | --- | --- | --- | --- | --- | --- | --- | --- | --- | --- | --- | --- | --- | --- | --- | --- | --- | --- | --- | --- | --- | --- | --- | --- | --- | --- | --- | --- | --- | --- | --- | --- | --- | --- | --- | --- | --- | --- | --- | --- | --- | --- | --- | --- | --- | --- | --- | --- | --- | --- | --- | --- | --- | --- | --- | --- | --- | --- | --- | --- | --- | --- | --- | --- | --- | --- | --- | --- | --- | --- | --- | --- | --- | --- | --- | --- | --- | --- | --- | --- | --- | --- | --- | --- | --- | --- | --- | --- | --- | --- | --- | --- | --- | --- | --- | --- | --- | --- | --- | --- | --- | --- | --- | --- | --- | --- | --- | --- | --- | --- | --- | --- | --- | --- | --- | --- | --- | --- | --- | --- | --- | --- | --- | --- | --- | --- | --- | --- | --- | --- | --- | --- | --- | --- | --- | --- | --- | --- | --- | --- | --- | --- | --- | --- | --- | --- | --- | --- | --- | --- | --- | --- | --- | --- | --- | --- | --- | --- | --- | --- | --- | --- | --- | --- | --- | --- | --- | --- | --- | --- | --- | --- | --- | --- | --- | --- | --- | --- | --- | --- | --- | --- | --- | --- | --- | --- | --- | --- | --- | --- | --- | --- | --- | --- | --- | --- | --- | --- | --- | --- | --- | --- | --- | --- | --- | --- | --- | --- | --- | --- | --- | --- | --- | --- | --- | --- | --- | --- | --- | --- | --- | --- | --- | --- | --- | --- | --- | --- | --- | --- | --- | --- | --- | --- | --- | --- | --- | --- | --- | --- | --- | --- | --- | --- | --- | --- | --- | --- | --- | --- | --- | --- | --- | --- | --- | --- | --- | --- | --- | --- | --- | --- | --- | --- | --- | --- | --- | --- | --- | --- | --- | --- | --- | --- | --- | --- | --- | --- | --- | --- | --- | --- | --- | --- | --- | --- | --- | --- | --- | --- | --- | --- | --- | --- | --- | --- | --- | --- | --- | --- | --- | --- | --- | --- | --- | --- | --- | --- | --- | --- | --- | --- | --- | --- | --- | --- | --- | --- | --- | --- | --- | --- | --- | --- | --- | --- | --- | --- | --- | --- | --- | --- | --- | --- | --- | --- | --- | --- | --- | --- | --- | --- | --- | --- | --- | --- | --- | --- | --- | --- | --- | --- | --- | --- | --- | --- | --- | --- | --- | --- | --- | --- | --- | --- | --- | --- | --- | --- | --- | --- | --- | --- | --- | --- | --- | --- | --- | --- | --- | --- | --- | --- | --- | --- | --- | --- | --- | --- | --- | --- | --- | --- | --- | --- | --- | --- | --- | --- | --- | --- | --- | --- | --- | --- | --- | --- | --- | --- | --- | --- | --- | --- | --- | --- | --- | --- | --- | --- | --- | --- | --- | --- | --- | --- | --- | --- | --- | --- | --- | --- | --- | --- | --- | --- | --- | --- | --- | --- | --- | --- | --- | --- | --- | --- | --- | --- | --- | --- | --- | --- | --- | --- | --- | --- | --- | --- | --- | --- | --- | --- | --- | --- | --- | --- | --- | --- | --- | --- | --- | --- | --- | --- | --- | --- | --- | --- | --- | --- | --- | --- | --- | --- | --- | --- | --- | --- | --- | --- | --- | --- | --- | --- | --- | --- | --- | --- | --- | --- | --- | --- | --- | --- | --- | --- | --- | --- | --- | --- | --- | --- | --- | --- | --- | --- | --- | --- | --- | --- | --- | --- | --- | --- | --- | --- | --- | --- | --- | --- | --- | --- | --- | --- | --- | --- | --- | --- | --- | --- | --- | --- | --- | --- | --- | --- | --- | --- | --- | --- | --- | --- | --- | --- | --- | --- | --- | --- | --- | --- | --- | --- | --- | --- | --- | --- | --- | --- | --- | --- | --- | --- | --- | --- | --- | --- | --- | --- | --- | --- | --- | --- | --- | --- | --- | --- | --- | --- | --- | --- | --- | --- | --- | --- | --- | --- | --- | --- | --- | --- | --- | --- | --- | --- | --- | --- | --- | --- | --- | --- | --- | --- | --- | --- | --- | --- | --- | --- | --- | --- | --- | --- | --- | --- | --- | --- | --- | --- | --- | --- | --- | --- | --- | --- | --- | --- | --- | --- | --- | --- | --- | --- | --- | --- | --- | --- | --- | --- | --- | --- | --- | --- | --- | --- | --- | --- | --- | --- | --- | --- | --- | --- | --- | --- | --- | --- | --- | --- | --- | --- | --- | --- | --- | --- | --- | --- | --- | --- | --- | --- | --- | --- | --- | --- | --- | --- | --- | --- | --- | --- | --- | --- | --- | --- | --- | --- | --- | --- | --- | --- | --- | --- | --- | --- | --- | --- | --- | --- | --- | --- | --- | --- | --- | --- | --- | --- | --- | --- | --- | --- | --- | --- | --- | --- | --- | --- | --- | --- | --- | --- | --- | --- | --- | --- | --- | --- | --- | --- | --- | --- | --- | --- | --- | --- | --- | --- | --- | --- | --- | --- | --- | --- | --- | --- | --- | --- | --- | --- | --- | --- | --- | --- | --- | --- | --- | --- | --- | --- | --- | --- | --- | --- | --- | --- | --- | --- | --- | --- | --- | --- | --- | --- | --- | --- | --- | --- | --- | --- | --- | --- | --- | --- | --- | --- | --- | --- | --- | --- | --- | --- | --- | --- | --- | --- | --- | --- | --- | --- | --- | --- | --- | --- | --- | --- | --- | --- | --- | --- | --- | --- | --- | --- | --- | --- | --- | --- | --- | --- | --- | --- | --- | --- | --- | --- | --- | --- | --- | --- | --- | --- | --- | --- | --- | --- | --- | --- | --- | --- | --- | --- | --- | --- | --- | --- | --- | --- | --- | --- | --- | --- | --- | --- | --- | --- | --- | --- | --- | --- | --- | --- | --- | --- | --- | --- | --- | --- | --- | --- | --- | --- | --- | --- | --- | --- | --- | --- | --- | --- | --- | --- | --- | --- | --- | --- | --- | --- | --- | --- | --- | --- | --- | --- | --- | --- | --- | --- | --- | --- | --- | --- | --- | --- | --- | --- | --- | --- | --- | --- | --- | --- | --- | --- | --- | --- | --- | --- | --- | --- | --- | --- | --- | --- | --- | --- | --- | --- | --- | --- | --- | --- | --- | --- | --- | --- | --- | --- | --- | --- | --- | --- | --- | --- | --- | --- | --- | --- | --- | --- | --- | --- | --- | --- | --- | --- | --- | --- | --- | --- | --- | --- | --- | --- | --- | --- | --- | --- | --- | --- | --- | --- | --- | --- | --- | --- | --- | --- | --- | --- | --- | --- | --- | --- | --- | --- | --- | --- | --- | --- | --- | --- | --- | --- | --- | --- | --- | --- | --- | --- | --- | --- | --- | --- | --- | --- | --- | --- | --- | --- | --- | --- | --- | --- | --- | --- | --- | --- | --- | --- | --- | --- | --- | --- | --- | --- | --- | --- | --- | --- | --- | --- | --- | --- | --- | --- | --- | --- | --- | --- | --- | --- | --- | --- | --- | --- | --- | --- | --- | --- | --- | --- | --- | --- | --- | --- | --- | --- | --- | --- | --- | --- | --- | --- | --- | --- | --- | --- | --- | --- | --- | --- | --- | --- | --- | --- | --- | --- | --- | --- | --- | --- | --- | --- | --- | --- | --- | --- | --- | --- | --- | --- | --- | --- | --- | --- | --- | --- | --- | --- | --- | --- | --- | --- | --- | --- | --- | --- | --- | --- | --- | --- | --- | --- | --- | --- | --- | --- | --- | --- | --- | --- | --- | --- | --- | --- | --- | --- | --- | --- | --- | --- | --- | --- | --- | --- | --- | --- | --- | --- | --- | --- | --- | --- | --- | --- | --- | --- | --- | --- | --- | --- | --- | --- | --- | --- | --- | --- | --- | --- | --- | --- | --- | --- | --- | --- | --- | --- | --- | --- | --- | --- | --- | --- | --- | --- | --- | --- | --- | --- | --- | --- | --- | --- | --- | --- | --- | --- | --- | --- | --- | --- | --- | --- | --- | --- | --- | --- | --- | --- | --- | --- | --- | --- | --- | --- | --- | --- | --- | --- | --- | --- | --- | --- | --- | --- | --- | --- | --- | --- | --- | --- | --- | --- | --- | --- | --- | --- | --- | --- | --- | --- | --- | --- | --- | --- | --- | --- | --- | --- | --- | --- | --- | --- | --- | --- | --- | --- | --- | --- | --- | --- | --- | --- | --- | --- | --- | --- | --- | --- | --- | --- | --- | --- | --- | --- | --- | --- | --- | --- | --- | --- | --- | --- | --- | --- | --- | --- | --- | --- | --- | --- | --- | --- | --- | --- | --- | --- | --- | --- | --- | --- | --- | --- | --- | --- | --- | --- | --- | --- | --- | --- | --- | --- | --- | --- | --- | --- | --- | --- | --- | --- | --- | --- | --- | --- | --- | --- | --- | --- | --- | --- | --- | --- | --- | --- | --- | --- | --- | --- | --- | --- | --- | --- | --- | --- | --- | --- | --- | --- | --- | --- | --- | --- | --- | --- | --- | --- | --- | --- | --- | --- | --- | --- | --- | --- | --- | --- | --- | --- | --- | --- | --- | --- | --- | --- | --- | --- | --- | --- | --- | --- | --- | --- | --- | --- | --- | --- | --- | --- | --- | --- | --- | --- | --- | --- | --- | --- | --- | --- | --- | --- | --- | --- | --- | --- | --- | --- | --- | --- | --- | --- | --- | --- | --- | --- | --- | --- | --- | --- | --- | --- | --- | --- | --- | --- | --- | --- | --- | --- | --- | --- | --- | --- | --- | --- | --- | --- | --- | --- | --- | --- | --- | --- | --- | --- | --- | --- | --- | --- | --- | --- | --- | --- | --- | --- | --- | --- | --- | --- | --- | --- | --- | --- | --- | --- | --- | --- | --- | --- | --- | --- | --- | --- | --- | --- | --- | --- | --- | --- | --- | --- | --- | --- | --- | --- | --- | --- | --- | --- | --- | --- | --- | --- | --- | --- | --- | --- | --- | --- | --- | --- | --- | --- | --- | --- | --- | --- | --- | --- | --- | --- | --- | --- | --- | --- | --- | --- | --- | --- | --- | --- | --- | --- | --- | --- | --- | --- | --- | --- | --- | --- | --- | --- | --- | --- | --- | --- | --- | --- | --- | --- | --- | --- | --- | --- | --- | --- | --- | --- | --- | --- | --- | --- | --- | --- | --- | --- | --- | --- | --- | --- | --- | --- | --- | --- | --- | --- | --- | --- | --- | --- | --- | --- | --- | --- | --- | --- | --- | --- | --- | --- | --- | --- | --- | --- | --- | --- | --- | --- | --- | --- | --- | --- | --- | --- | --- | --- | --- | --- | --- | --- | --- | --- | --- | --- | --- | --- | --- | --- | --- | --- | --- | --- | --- | --- | --- | --- | --- | --- | --- | --- | --- | --- | --- | --- | --- | --- | --- | --- | --- | --- | --- | --- | --- | --- | --- | --- | --- | --- | --- | --- | --- | --- | --- | --- | --- | --- | --- | --- | --- | --- | --- | --- | --- | --- | --- | --- | --- | --- | --- | --- | --- | --- | --- | --- | --- | --- | --- | --- | --- | --- | --- | --- | --- | --- | --- | --- | --- | --- | --- | --- | --- | --- | --- | --- | --- | --- | --- | --- | --- | --- | --- | --- | --- | --- | --- | --- | --- | --- | --- | --- | --- | --- | --- | --- | --- | --- | --- | --- | --- | --- | --- | --- | --- | --- | --- | --- | --- | --- | --- | --- | --- | --- | --- | --- | --- | --- | --- | --- | --- | --- | --- | --- | --- | --- | --- | --- | --- | --- | --- | --- | --- | --- | --- | --- | --- | --- | --- | --- | --- | --- | --- | --- | --- | --- | --- | --- | --- | --- | --- | --- | --- | --- | --- | --- | --- | --- | --- | --- | --- | --- | --- | --- | --- | --- | --- | --- | --- | --- | --- | --- | --- | --- | --- | --- | --- | --- | --- | --- | --- | --- | --- | --- | --- | --- | --- | --- | --- | --- | --- | --- | --- | --- | --- | --- | --- | --- | --- | --- | --- | --- | --- | --- | --- | --- | --- | --- | --- | --- | --- | --- | --- | --- | --- | --- | --- | --- | --- | --- | --- | --- | --- | --- | --- | --- | --- | --- | --- | --- | --- | --- | --- | --- | --- | --- | --- | --- | --- | --- | --- | --- | --- | --- | --- | --- | --- | --- | --- | --- | --- | --- | --- | --- | --- | --- | --- | --- | --- | --- | --- | --- | --- | --- | --- | --- | --- | --- | --- | --- | --- | --- | --- | --- | --- | --- | --- | --- | --- | --- | --- | --- | --- | --- | --- | --- | --- | --- | --- | --- | --- | --- | --- | --- | --- | --- | --- | --- | --- | --- | --- | --- | --- | --- | --- | --- | --- | --- | --- | --- | --- | --- | --- | --- | --- | --- | --- | --- | --- | --- | --- | --- | --- | --- | --- | --- | --- | --- | --- | --- | --- | --- | --- | --- | --- | --- |
| |  |  |  |  |  |  |  |  |  |  |  |  |  |  |  |  |  |  |  |  |  |  |  |  |  |  |  |  |  |  |  |  |  |  |  |  |  |  |  |  |  |  |  |  |  |  |  |  |  |  |  |  |  |  |  |  |  |  | | --- | --- | --- | --- | --- | --- | --- | --- | --- | --- | --- | --- | --- | --- | --- | --- | --- | --- | --- | --- | --- | --- | --- | --- | --- | --- | --- | --- | --- | --- | --- | --- | --- | --- | --- | --- | --- | --- | --- | --- | --- | --- | --- | --- | --- | --- | --- | --- | --- | --- | --- | --- | --- | --- | --- | --- | --- | --- | | G0V8E9/1-842 | 1 | M | V | A | F | T | V | D | Q | M | R | S | L | M | D | T | V | T | N | V | R | N | M | S | V | I | A | H | V | D | H | G | K | S | T | L | T | D | S | L | V | Q | K | A | G | I | I | S | A | A | K | A | G | E | A | R | 55 | | Q6CPQ9/1-842 | 1 | M | V | A | F | T | V | D | Q | I | R | S | L | M | D | K | V | T | N | V | R | N | M | S | V | I | A | H | V | D | H | G | K | S | T | L | T | D | S | L | V | Q | R | A | G | I | I | S | A | A | K | A | G | E | A | R | 55 | | Q6FYA7/1-842 | 1 | M | V | A | F | T | V | D | Q | M | R | S | L | M | D | K | V | T | N | V | R | N | M | S | V | I | A | H | V | D | H | G | K | S | T | L | T | D | S | L | V | Q | K | A | G | I | I | S | A | A | K | A | G | E | A | R | 55 | | Q754C8/1-842 | 1 | M | V | A | F | T | V | D | Q | I | R | S | L | M | D | K | V | T | N | V | R | N | M | S | V | I | A | H | V | D | H | G | K | S | T | L | T | D | S | L | V | Q | R | A | G | I | I | S | A | A | K | A | G | E | A | R | 55 | | A7THK9/1-842 | 1 | M | V | A | F | T | V | D | Q | M | R | S | L | M | D | T | V | T | N | V | R | N | M | S | V | I | A | H | V | D | H | G | K | S | T | L | T | D | S | L | V | Q | R | A | G | I | I | S | A | A | K | A | G | E | A | R | 55 | | C5DJC0/1-842 | 1 | M | V | A | F | T | V | D | Q | M | R | S | L | M | D | K | V | T | N | V | R | N | M | S | V | I | A | H | V | D | H | G | K | S | T | L | T | D | S | L | V | Q | R | A | G | I | I | S | A | A | K | A | G | E | A | R | 55 | | C5DW13/1-842 | 1 | M | V | A | F | T | V | D | Q | M | R | S | L | M | D | K | V | A | N | V | R | N | M | S | V | I | A | H | V | D | H | G | K | S | T | L | T | D | S | L | V | Q | K | A | G | I | I | S | A | A | K | A | G | E | A | R | 55 | | Kwal\_55.21295/1-842 | 1 | M | V | A | F | T | V | D | Q | M | R | S | L | M | D | K | V | T | N | V | R | N | M | S | V | I | A | H | V | D | H | G | K | S | T | L | T | D | S | L | V | Q | R | A | G | I | I | S | A | A | K | A | G | E | A | R | 55 | | Sbay\_530.4/1-842 | 1 | M | V | A | F | T | V | D | Q | M | R | S | L | M | D | K | V | T | N | V | R | N | M | S | V | I | A | H | V | D | H | G | K | S | T | L | T | D | S | L | V | Q | R | A | G | I | I | S | A | A | K | A | G | E | A | R | 55 | | SAKL0G03388g/1-842 | 1 | M | V | A | F | T | V | D | Q | I | R | S | L | M | D | K | V | T | N | I | R | N | M | S | V | I | A | H | V | D | H | G | K | S | T | L | T | D | S | L | V | Q | R | A | G | I | I | S | A | A | K | A | G | E | A | R | 55 | | P32324/1-842 | 1 | M | V | A | F | T | V | D | Q | M | R | S | L | M | D | K | V | T | N | V | R | N | M | S | V | I | A | H | V | D | H | G | K | S | T | L | T | D | S | L | V | Q | R | A | G | I | I | S | A | A | K | A | G | E | A | R | 55 | |  | | G0V8E9/1-842 | 56 | F | M | D | T | R | K | D | E | Q | E | R | G | I | T | I | K | S | T | A | I | S | L | Y | S | E | M | P | D | E | D | V | K | D | I | A | Q | K | T | E | G | N | A | F | L | I | N | L | I | D | S | P | G | H | V | D | 110 | | Q6CPQ9/1-842 | 56 | F | T | D | T | R | K | D | E | Q | E | R | G | I | T | I | K | S | T | A | I | S | L | F | S | E | M | S | D | D | D | V | K | D | I | K | Q | K | T | D | G | N | A | F | L | I | N | L | I | D | S | P | G | H | V | D | 110 | | Q6FYA7/1-842 | 56 | F | M | D | T | R | K | D | E | Q | E | R | G | I | T | I | K | S | T | A | I | S | L | Y | S | D | L | P | E | E | D | V | K | E | I | P | Q | K | S | D | G | N | S | F | L | I | N | L | I | D | S | P | G | H | V | D | 110 | | Q754C8/1-842 | 56 | F | T | D | T | R | K | D | E | Q | E | R | G | I | T | I | K | S | T | A | I | S | L | F | S | E | M | S | E | E | D | V | K | D | I | K | Q | K | T | E | G | N | S | F | L | I | N | L | I | D | S | P | G | H | V | D | 110 | | A7THK9/1-842 | 56 | F | T | D | T | R | K | D | E | Q | E | R | G | I | T | I | K | S | T | A | I | S | L | Y | S | E | M | S | E | E | D | V | K | D | I | K | Q | K | T | E | G | R | A | F | L | I | N | L | I | D | S | P | G | H | V | D | 110 | | C5DJC0/1-842 | 56 | F | T | D | T | R | K | D | E | Q | E | R | G | I | T | I | K | S | T | A | I | S | L | Y | S | E | M | T | E | D | D | V | K | D | I | K | Q | K | T | I | G | N | S | F | L | I | N | L | I | D | S | P | G | H | V | D | 110 | | C5DW13/1-842 | 56 | F | M | D | T | R | K | D | E | Q | E | R | G | I | T | I | K | S | T | A | I | S | L | F | A | E | M | S | D | T | D | V | K | D | I | K | Q | K | V | D | G | N | S | F | L | V | N | L | I | D | S | P | G | H | V | D | 110 | | Kwal\_55.21295/1-842 | 56 | F | T | D | T | R | K | D | E | Q | E | R | G | I | T | I | K | S | T | A | I | S | L | Y | A | E | M | D | E | E | D | V | K | D | I | K | Q | K | T | I | G | N | S | F | L | I | N | L | I | D | S | P | G | H | V | D | 110 | | Sbay\_530.4/1-842 | 56 | F | T | D | T | R | K | D | E | Q | E | R | G | I | T | I | K | S | T | A | I | S | L | Y | S | E | M | S | D | E | D | V | K | E | I | K | Q | K | T | D | G | N | S | F | L | I | N | L | I | D | S | P | G | H | V | D | 110 | | SAKL0G03388g/1-842 | 56 | F | T | D | T | R | K | D | E | Q | E | R | G | I | T | I | K | S | T | A | I | S | L | F | S | E | M | S | D | D | D | V | K | D | I | K | Q | K | T | E | G | N | S | F | L | I | N | L | I | D | S | P | G | H | V | D | 110 | | P32324/1-842 | 56 | F | T | D | T | R | K | D | E | Q | E | R | G | I | T | I | K | S | T | A | I | S | L | Y | S | E | M | S | D | E | D | V | K | E | I | K | Q | K | T | D | G | N | S | F | L | I | N | L | I | D | S | P | G | H | V | D | 110 | |  | | G0V8E9/1-842 | 111 | F | S | S | E | V | T | A | A | L | R | V | T | D | G | A | L | V | V | V | D | T | V | E | G | V | C | V | Q | T | E | T | V | L | R | Q | A | L | G | E | R | I | K | P | V | V | C | I | N | K | V | D | R | A | L | L | 165 | | Q6CPQ9/1-842 | 111 | F | S | S | E | V | T | A | A | L | R | V | T | D | G | A | L | V | V | V | D | T | V | E | G | V | C | V | Q | T | E | T | V | L | R | Q | S | L | A | E | R | I | K | P | V | V | V | I | N | K | V | D | R | A | L | L | 165 | | Q6FYA7/1-842 | 111 | F | S | S | E | V | T | A | A | L | R | V | T | D | G | A | L | V | V | V | D | T | V | E | G | V | C | V | Q | T | E | T | V | L | R | Q | A | L | G | E | R | I | K | P | V | V | C | I | N | K | V | D | R | A | L | L | 165 | | Q754C8/1-842 | 111 | F | S | S | E | V | T | A | A | L | R | V | T | D | G | A | L | V | V | V | D | T | V | E | G | V | C | V | Q | T | E | T | V | L | R | Q | A | L | G | E | R | I | K | P | V | V | V | I | N | K | V | D | R | A | L | L | 165 | | A7THK9/1-842 | 111 | F | S | S | E | V | T | A | A | L | R | V | T | D | G | A | L | V | V | V | D | T | I | E | G | V | C | V | Q | T | E | T | V | L | R | Q | A | L | G | E | R | I | K | P | V | V | C | I | N | K | V | D | R | A | L | L | 165 | | C5DJC0/1-842 | 111 | F | S | S | E | V | T | A | A | L | R | V | T | D | G | A | L | V | V | V | D | T | V | E | G | V | C | V | Q | T | E | T | V | L | R | Q | A | L | G | E | R | I | K | P | V | V | V | I | N | K | V | D | R | A | L | L | 165 | | C5DW13/1-842 | 111 | F | S | S | E | V | T | A | A | L | R | I | T | D | G | A | L | V | V | V | D | T | V | E | G | V | C | V | Q | T | E | T | V | L | R | Q | A | L | G | E | R | I | K | P | V | V | C | I | N | K | V | D | R | A | L | L | 165 | | Kwal\_55.21295/1-842 | 111 | F | S | S | E | V | T | A | A | L | R | V | T | D | G | A | L | V | V | V | D | T | V | E | G | V | C | V | Q | T | E | T | V | L | R | Q | A | L | G | E | R | I | K | P | V | V | V | I | N | K | V | D | R | A | L | L | 165 | | Sbay\_530.4/1-842 | 111 | F | S | S | E | V | T | A | A | L | R | V | T | D | G | A | L | V | V | V | D | T | I | E | G | V | C | V | Q | T | E | T | V | L | R | Q | A | L | G | E | R | I | K | P | V | V | V | I | N | K | V | D | R | A | L | L | 165 | | SAKL0G03388g/1-842 | 111 | F | S | S | E | V | T | A | A | L | R | V | T | D | G | A | L | V | V | V | D | T | V | E | G | V | C | V | Q | T | E | T | V | L | R | Q | A | L | G | E | R | I | K | P | V | V | C | V | N | K | V | D | R | A | L | L | 165 | | P32324/1-842 | 111 | F | S | S | E | V | T | A | A | L | R | V | T | D | G | A | L | V | V | V | D | T | I | E | G | V | C | V | Q | T | E | T | V | L | R | Q | A | L | G | E | R | I | K | P | V | V | V | I | N | K | V | D | R | A | L | L | 165 | |  | | G0V8E9/1-842 | 166 | E | L | Q | V | S | K | E | D | L | Y | Q | S | F | S | R | T | V | E | S | V | N | V | I | I | S | T | Y | A | D | E | I | L | G | D | V | Q | V | Y | P | S | K | G | T | V | A | F | G | S | G | L | H | G | W | A | F | 220 | | Q6CPQ9/1-842 | 166 | E | L | Q | V | S | K | E | D | L | Y | Q | S | F | S | R | T | V | E | S | V | N | V | I | I | S | T | Y | A | D | E | V | L | G | D | V | Q | V | Y | P | Q | R | G | T | V | A | F | G | S | G | L | H | G | W | A | F | 220 | | Q6FYA7/1-842 | 166 | E | L | Q | V | S | K | E | D | L | Y | Q | S | F | S | R | T | V | E | S | V | N | V | I | I | S | T | Y | S | D | E | V | L | G | D | V | Q | V | Y | P | S | K | G | T | V | A | F | G | S | G | L | H | G | W | A | F | 220 | | Q754C8/1-842 | 166 | E | L | Q | V | S | K | E | D | L | Y | Q | S | F | S | R | T | V | E | S | V | N | V | I | I | S | T | Y | A | D | E | V | L | G | D | V | Q | V | Y | P | Q | K | G | T | V | A | F | G | S | G | L | H | G | W | A | F | 220 | | A7THK9/1-842 | 166 | E | L | Q | V | S | K | E | D | L | Y | Q | T | F | A | R | T | V | E | S | V | N | V | I | I | S | T | Y | A | D | E | V | L | G | D | V | Q | V | Y | P | Q | R | G | T | V | A | F | G | S | G | L | H | G | W | A | F | 220 | | C5DJC0/1-842 | 166 | E | L | Q | V | T | K | E | D | L | Y | Q | S | F | S | R | T | V | E | S | V | N | V | I | V | S | T | Y | A | D | E | V | L | G | D | V | Q | V | F | P | Q | Q | G | T | V | A | F | G | S | G | L | H | G | W | A | F | 220 | | C5DW13/1-842 | 166 | E | L | Q | V | T | K | E | D | L | Y | Q | S | F | S | R | T | V | E | S | V | N | V | I | V | S | T | Y | A | D | E | V | L | G | D | V | Q | V | Y | P | S | Q | G | T | V | A | F | G | S | G | L | H | G | W | A | F | 220 | | Kwal\_55.21295/1-842 | 166 | E | L | Q | V | T | K | E | D | L | Y | Q | T | F | A | R | T | V | E | S | V | N | V | I | I | S | T | Y | A | D | E | A | L | G | D | M | Q | V | F | P | S | K | G | T | V | A | F | G | S | G | L | H | G | W | A | F | 220 | | Sbay\_530.4/1-842 | 166 | E | L | Q | V | S | K | E | D | L | Y | Q | T | F | A | R | T | V | E | S | V | N | V | I | V | S | T | Y | A | D | E | V | L | G | D | V | Q | V | Y | P | A | K | G | T | V | A | F | G | S | G | L | H | G | W | A | F | 220 | | SAKL0G03388g/1-842 | 166 | E | L | Q | V | S | K | E | D | L | Y | Q | S | F | A | R | T | V | E | S | V | N | V | I | I | S | T | Y | A | D | E | V | L | G | D | V | Q | V | Y | P | S | K | G | T | I | A | F | G | S | G | L | H | G | W | A | F | 220 | | P32324/1-842 | 166 | E | L | Q | V | S | K | E | D | L | Y | Q | T | F | A | R | T | V | E | S | V | N | V | I | V | S | T | Y | A | D | E | V | L | G | D | V | Q | V | Y | P | A | R | G | T | V | A | F | G | S | G | L | H | G | W | A | F | 220 | |  | | G0V8E9/1-842 | 221 | T | I | R | Q | F | A | Q | R | Y | A | K | K | F | G | V | D | K | V | K | M | M | E | R | L | W | G | D | S | Y | F | N | P | K | T | K | K | W | T | N | K | E | T | D | A | D | G | K | Q | L | E | R | A | F | N | M | 275 | | Q6CPQ9/1-842 | 221 | T | V | R | Q | F | A | N | R | Y | S | K | K | F | G | V | D | R | E | K | M | M | D | R | L | W | G | D | S | Y | F | N | P | K | T | K | K | W | T | N | K | E | R | D | A | D | G | K | P | L | E | R | A | F | N | M | 275 | | Q6FYA7/1-842 | 221 | T | I | R | Q | F | A | T | R | Y | A | K | K | F | G | V | D | K | Q | K | M | M | E | R | L | W | G | D | S | F | F | N | P | K | T | K | K | W | T | N | K | E | T | D | T | D | G | K | P | L | E | R | A | F | N | M | 275 | | Q754C8/1-842 | 221 | T | I | R | Q | F | A | N | R | Y | S | K | K | F | G | V | D | R | E | K | M | M | E | R | L | W | G | D | S | Y | F | N | P | K | T | K | K | W | T | N | K | D | R | D | A | D | G | K | P | L | E | R | A | F | N | M | 275 | | A7THK9/1-842 | 221 | T | I | R | Q | F | A | N | R | Y | G | K | K | F | G | V | D | K | T | K | M | M | D | R | L | W | G | D | S | F | F | N | P | K | T | K | K | W | S | S | K | D | T | D | A | D | G | K | P | L | E | R | A | F | N | M | 275 | | C5DJC0/1-842 | 221 | T | I | R | Q | F | A | N | R | Y | S | K | K | F | G | V | D | R | Q | K | M | M | D | R | L | W | G | D | S | Y | F | N | P | K | T | K | K | W | T | N | K | E | V | D | A | D | G | K | P | L | E | R | A | F | N | M | 275 | | C5DW13/1-842 | 221 | T | I | R | Q | F | A | N | R | Y | A | K | K | F | G | V | D | K | N | K | M | M | E | K | L | W | G | D | S | Y | F | N | P | K | T | K | K | W | T | N | K | D | T | D | A | D | G | K | P | L | E | R | A | F | N | M | 275 | | Kwal\_55.21295/1-842 | 221 | T | I | R | Q | F | A | S | R | Y | S | K | K | F | G | V | D | K | Q | K | M | M | D | R | L | W | G | D | S | F | F | N | P | K | T | K | K | W | T | N | K | D | T | D | T | D | G | K | P | L | E | R | A | F | N | M | 275 | | Sbay\_530.4/1-842 | 221 | T | I | R | Q | F | A | T | R | Y | A | K | K | F | G | V | D | Q | S | K | M | M | E | R | L | W | G | D | S | F | F | N | P | K | T | K | K | W | T | N | K | D | T | D | A | D | G | K | P | L | E | R | A | F | N | M | 275 | | SAKL0G03388g/1-842 | 221 | T | I | R | Q | F | A | N | R | Y | S | K | K | F | G | V | D | R | E | K | M | M | E | R | L | W | G | D | S | Y | F | N | P | K | T | K | K | W | T | N | K | E | T | D | T | D | G | K | P | L | E | R | A | F | N | M | 275 | | P32324/1-842 | 221 | T | I | R | Q | F | A | T | R | Y | A | K | K | F | G | V | D | K | A | K | M | M | D | R | L | W | G | D | S | F | F | N | P | K | T | K | K | W | T | N | K | D | T | D | A | E | G | K | P | L | E | R | A | F | N | M | 275 | |  | | G0V8E9/1-842 | 276 | F | V | L | D | P | I | F | R | L | F | A | A | I | M | N | F | K | K | D | E | I | P | V | L | L | E | K | L | E | I | N | L | K | G | D | E | K | D | Q | E | G | K | A | L | L | K | T | V | M | K | K | F | L | P | A | 330 | | Q6CPQ9/1-842 | 276 | F | V | L | D | P | I | F | R | L | F | A | A | I | M | N | F | K | K | E | E | I | P | V | L | L | E | K | L | E | I | N | L | K | G | D | E | K | E | L | E | G | K | N | L | L | K | V | V | M | R | K | F | L | P | A | 330 | | Q6FYA7/1-842 | 276 | F | V | L | D | P | I | F | R | L | F | A | A | I | M | N | F | K | K | D | E | I | P | T | L | L | E | K | L | E | I | N | L | K | S | D | E | K | D | L | E | G | K | A | L | L | K | V | V | M | R | K | F | L | P | A | 330 | | Q754C8/1-842 | 276 | F | V | L | D | P | I | F | R | L | F | A | A | I | M | N | F | K | K | D | E | I | P | V | L | L | E | K | L | E | I | A | L | K | S | D | E | R | D | L | E | G | K | A | L | L | K | V | V | M | R | K | F | L | P | A | 330 | | A7THK9/1-842 | 276 | F | V | L | D | P | I | F | R | L | F | S | A | V | M | N | F | K | K | E | E | I | P | V | L | L | E | K | L | E | I | Q | L | K | G | D | E | K | D | L | E | G | K | A | L | L | K | V | V | M | R | K | F | L | P | A | 330 | | C5DJC0/1-842 | 276 | F | V | L | D | P | I | F | R | L | F | A | A | I | M | N | F | K | K | D | E | I | P | V | L | L | E | K | L | E | I | N | L | K | G | D | E | K | D | L | E | G | K | A | L | L | K | V | V | M | R | K | F | L | P | A | 330 | | C5DW13/1-842 | 276 | F | V | L | D | P | I | F | R | L | F | A | A | I | M | N | F | K | K | D | E | I | P | V | L | L | E | K | L | E | I | N | L | K | A | D | E | K | D | L | E | G | K | A | L | L | K | V | V | M | K | K | F | L | P | A | 330 | | Kwal\_55.21295/1-842 | 276 | F | V | L | D | P | I | F | R | L | F | A | A | I | M | N | F | K | K | D | E | I | P | V | L | L | T | K | L | E | I | N | L | K | G | E | E | K | D | L | E | G | K | A | L | L | K | V | V | M | R | K | F | L | P | A | 330 | | Sbay\_530.4/1-842 | 276 | F | I | L | D | P | I | F | R | L | F | T | A | I | M | N | F | K | K | E | E | I | P | V | L | L | E | K | L | E | I | I | L | K | G | D | E | K | D | L | E | G | K | A | L | L | K | V | V | M | R | K | F | L | P | A | 330 | | SAKL0G03388g/1-842 | 276 | F | V | L | D | P | I | F | R | L | F | S | A | I | M | N | F | K | K | D | E | I | P | V | L | L | E | K | L | E | I | N | L | K | G | E | E | K | E | L | E | G | K | A | L | L | K | I | V | M | R | K | F | L | P | A | 330 | | P32324/1-842 | 276 | F | I | L | D | P | I | F | R | L | F | T | A | I | M | N | F | K | K | D | E | I | P | V | L | L | E | K | L | E | I | V | L | K | G | D | E | K | D | L | E | G | K | A | L | L | K | V | V | M | R | K | F | L | P | A | 330 | |  | | G0V8E9/1-842 | 331 | A | D | A | L | L | E | M | I | V | M | N | L | P | S | P | V | T | A | Q | A | Y | R | A | E | Q | L | Y | E | G | P | A | D | D | A | N | C | M | A | I | K | R | C | D | P | K | A | D | L | M | L | Y | V | S | K | M | 385 | | Q6CPQ9/1-842 | 331 | A | D | A | L | L | E | M | I | I | L | H | L | P | S | P | V | T | A | Q | N | Y | R | A | E | Q | L | Y | E | G | P | S | D | D | P | A | C | I | A | I | K | N | C | D | P | K | S | D | L | M | L | Y | V | S | K | M | 385 | | Q6FYA7/1-842 | 331 | A | D | A | L | L | E | M | I | V | M | H | L | P | S | P | V | T | A | Q | N | Y | R | A | E | Q | L | Y | E | G | P | A | D | D | A | N | C | I | A | I | K | K | C | D | P | T | A | D | L | M | L | Y | V | S | K | M | 385 | | Q754C8/1-842 | 331 | A | D | A | L | L | E | M | I | I | M | H | L | P | S | P | V | T | A | Q | N | Y | R | A | E | Q | L | Y | E | G | P | S | D | D | P | A | C | I | A | I | K | N | C | D | P | K | A | D | L | M | L | Y | V | S | K | M | 385 | | A7THK9/1-842 | 331 | A | D | A | L | L | E | M | I | I | M | H | L | P | S | P | V | T | A | Q | E | Y | R | A | E | Q | L | Y | E | G | P | H | D | D | P | S | C | I | A | I | K | N | C | D | P | K | A | D | L | M | L | Y | V | S | K | M | 385 | | C5DJC0/1-842 | 331 | A | D | A | L | L | E | M | I | V | M | H | L | P | S | P | V | T | A | Q | N | Y | R | A | E | Q | L | Y | E | G | P | S | D | D | P | A | C | V | A | I | K | N | C | D | P | T | S | D | L | M | L | Y | V | S | K | M | 385 | | C5DW13/1-842 | 331 | A | D | A | L | M | E | M | I | V | M | H | L | P | S | P | V | T | A | Q | N | Y | R | A | E | Q | L | Y | E | G | P | S | D | D | Q | F | C | Q | A | I | K | K | C | D | P | T | S | D | L | M | L | Y | V | S | K | M | 385 | | Kwal\_55.21295/1-842 | 331 | A | D | A | L | L | E | M | L | V | M | H | L | P | S | P | V | T | A | Q | N | Y | R | A | E | Q | L | Y | E | G | P | A | D | D | A | A | C | V | A | I | K | N | C | D | P | T | S | D | L | M | L | Y | V | S | K | M | 385 | | Sbay\_530.4/1-842 | 331 | A | D | A | L | L | E | M | I | I | L | H | L | P | S | P | V | T | A | Q | A | Y | R | A | E | Q | L | Y | E | G | P | A | D | D | A | S | C | I | A | I | K | N | C | D | P | K | A | D | L | M | L | Y | V | S | K | M | 385 | | SAKL0G03388g/1-842 | 331 | A | D | A | L | L | E | M | I | I | M | H | L | P | S | P | V | T | A | Q | N | Y | R | A | E | Q | L | Y | E | G | P | S | D | D | P | A | C | I | A | I | K | N | C | D | P | K | A | D | L | M | L | Y | V | S | K | M | 385 | | P32324/1-842 | 331 | A | D | A | L | L | E | M | I | V | L | H | L | P | S | P | V | T | A | Q | A | Y | R | A | E | Q | L | Y | E | G | P | A | D | D | A | N | C | I | A | I | K | N | C | D | P | K | A | D | L | M | L | Y | V | S | K | M | 385 | |  | | G0V8E9/1-842 | 386 | V | P | T | S | D | K | G | R | F | Y | A | F | G | R | V | F | A | G | T | V | K | S | G | Q | K | V | R | I | Q | G | P | N | Y | V | P | G | K | K | D | D | L | F | V | K | A | I | Q | R | V | V | L | M | M | G | R | 440 | | Q6CPQ9/1-842 | 386 | V | P | T | S | D | K | G | R | F | Y | A | F | G | R | V | F | A | G | T | V | K | S | G | Q | K | V | R | I | Q | G | P | N | F | I | P | G | K | K | E | D | L | F | I | K | A | I | Q | R | A | V | L | M | M | G | R | 440 | | Q6FYA7/1-842 | 386 | V | P | T | S | D | K | G | R | F | Y | A | F | G | R | V | F | A | G | T | V | K | S | G | Q | K | I | R | I | Q | G | P | N | Y | V | P | G | K | K | D | D | L | F | L | K | A | V | Q | R | V | V | L | M | M | G | S | 440 | | Q754C8/1-842 | 386 | V | P | T | S | D | K | G | R | F | Y | A | F | G | R | V | F | S | G | T | V | K | S | G | Q | K | V | R | I | Q | G | P | S | F | T | V | G | K | K | E | D | L | F | I | K | A | I | Q | R | A | V | L | M | M | G | R | 440 | | A7THK9/1-842 | 386 | V | P | T | S | D | K | G | R | F | Y | A | F | G | R | V | F | A | G | T | V | K | S | G | Q | K | V | R | I | Q | G | P | N | F | V | P | G | K | K | E | D | L | F | I | K | A | I | Q | R | V | V | L | M | M | G | R | 440 | | C5DJC0/1-842 | 386 | V | P | T | S | D | K | G | R | F | Y | A | F | G | R | V | F | A | G | T | V | K | S | G | Q | K | I | R | I | Q | G | P | N | Y | V | P | G | K | K | D | D | L | F | L | K | A | V | Q | R | V | V | L | M | M | G | R | 440 | | C5DW13/1-842 | 386 | I | P | T | S | D | K | G | R | F | Y | A | F | G | R | V | F | A | G | T | V | K | S | G | Q | K | V | R | I | Q | G | P | N | Y | V | P | G | K | K | D | D | L | F | L | K | A | V | Q | R | I | V | L | M | M | G | S | 440 | | Kwal\_55.21295/1-842 | 386 | V | P | T | S | D | K | G | R | F | Y | A | F | G | R | V | F | A | G | T | V | K | S | G | Q | K | I | R | I | Q | G | P | N | Y | I | P | G | K | K | D | D | L | F | I | K | A | V | Q | R | V | V | L | M | M | G | R | 440 | | Sbay\_530.4/1-842 | 386 | V | P | T | S | D | K | G | R | F | Y | A | F | G | R | V | F | A | G | T | V | K | S | G | Q | K | V | R | I | Q | G | P | N | Y | V | P | G | K | K | D | D | L | F | I | K | A | I | Q | R | V | V | L | M | M | G | R | 440 | | SAKL0G03388g/1-842 | 386 | V | P | T | S | D | K | G | R | F | Y | A | F | G | R | V | F | A | G | T | V | K | S | G | Q | K | V | R | I | Q | G | P | N | Y | I | P | G | K | K | D | D | L | F | I | K | A | V | Q | R | A | V | L | M | M | G | R | 440 | | P32324/1-842 | 386 | V | P | T | S | D | K | G | R | F | Y | A | F | G | R | V | F | A | G | T | V | K | S | G | Q | K | V | R | I | Q | G | P | N | Y | V | P | G | K | K | D | D | L | F | I | K | A | I | Q | R | V | V | L | M | M | G | R | 440 | |  | | G0V8E9/1-842 | 441 | F | V | E | P | I | D | D | C | P | A | G | N | I | I | G | L | V | G | I | D | Q | F | L | L | K | S | G | T | L | T | T | D | E | A | A | H | N | M | K | V | M | K | F | S | V | S | P | V | V | Q | V | A | V | E | V | 495 | | Q6CPQ9/1-842 | 441 | F | V | E | P | I | D | D | C | P | A | G | N | I | I | G | L | V | G | I | D | Q | F | L | L | K | T | G | T | L | T | T | F | E | G | A | H | N | M | K | V | M | K | F | S | V | S | P | V | V | Q | V | A | V | E | V | 495 | | Q6FYA7/1-842 | 441 | R | V | E | P | I | D | D | C | P | A | G | N | I | V | G | L | V | G | I | D | Q | F | L | L | K | T | G | T | L | T | T | S | E | T | A | Y | N | M | K | V | M | K | F | S | V | S | P | V | V | Q | V | A | V | D | V | 495 | | Q754C8/1-842 | 441 | F | V | E | P | I | D | D | C | P | A | G | N | I | V | G | L | V | G | I | D | Q | F | L | L | K | T | G | T | L | T | T | F | E | S | A | H | N | M | K | V | M | K | F | S | V | S | P | V | V | Q | V | A | V | E | V | 495 | | A7THK9/1-842 | 441 | F | V | E | P | I | D | D | C | P | A | G | N | I | I | G | L | V | G | I | D | Q | F | L | L | K | T | G | T | L | T | T | S | E | T | A | Y | N | M | K | V | M | K | F | S | V | S | P | V | V | Q | V | A | V | E | V | 495 | | C5DJC0/1-842 | 441 | F | V | E | P | I | D | D | C | P | A | G | N | I | V | G | L | V | G | I | D | Q | F | L | L | K | T | G | T | L | T | T | Y | E | S | A | H | N | M | K | V | M | K | F | S | V | S | P | V | V | Q | V | A | V | E | V | 495 | | C5DW13/1-842 | 441 | R | T | E | P | I | D | D | C | P | A | G | N | I | V | G | L | V | G | I | D | Q | F | L | L | K | T | G | T | L | T | T | N | E | A | A | H | N | M | K | V | M | K | F | S | V | S | P | V | V | Q | V | A | V | E | V | 495 | | Kwal\_55.21295/1-842 | 441 | F | V | E | P | I | D | D | C | P | A | G | N | I | I | G | L | V | G | I | D | Q | F | L | L | K | T | G | T | L | T | T | Y | E | T | A | H | N | M | K | V | M | K | F | S | V | S | P | V | V | Q | V | A | V | E | V | 495 | | Sbay\_530.4/1-842 | 441 | F | V | E | P | I | D | D | C | P | A | G | N | I | I | G | L | V | G | I | D | Q | F | L | L | K | T | G | T | L | T | T | N | E | A | S | H | N | M | K | V | M | K | F | S | V | S | P | V | V | Q | V | A | V | E | V | 495 | | SAKL0G03388g/1-842 | 441 | F | V | E | P | I | D | D | C | P | A | G | N | I | V | G | L | V | G | V | D | Q | F | L | L | K | T | G | T | L | T | T | F | E | G | A | H | N | M | K | V | M | K | F | S | V | S | P | V | V | Q | V | A | V | E | V | 495 | | P32324/1-842 | 441 | F | V | E | P | I | D | D | C | P | A | G | N | I | I | G | L | V | G | I | D | Q | F | L | L | K | T | G | T | L | T | T | S | E | T | A | H | N | M | K | V | M | K | F | S | V | S | P | V | V | Q | V | A | V | E | V | 495 | |  | | G0V8E9/1-842 | 496 | K | N | A | N | D | L | P | K | L | V | E | G | L | K | R | L | S | K | S | D | P | C | V | L | T | Y | M | A | E | T | G | E | H | I | V | A | G | T | G | E | L | H | L | E | I | C | L | Q | D | L | E | N | D | H | A | 550 | | Q6CPQ9/1-842 | 496 | K | N | A | N | D | L | P | K | L | V | E | G | L | K | R | L | S | K | S | D | P | C | V | L | V | S | M | S | E | S | G | E | H | I | V | A | G | T | G | E | L | H | L | E | I | C | L | Q | D | L | E | N | D | H | A | 550 | | Q6FYA7/1-842 | 496 | K | N | A | N | D | L | P | K | L | V | E | G | L | K | R | L | S | K | S | D | P | C | V | L | T | Q | M | S | E | S | G | E | H | I | V | A | G | T | G | E | L | H | L | E | I | C | L | Q | D | L | E | N | E | H | A | 550 | | Q754C8/1-842 | 496 | K | N | A | N | D | L | P | K | L | V | E | G | L | K | R | L | S | K | S | D | P | C | V | L | T | Y | M | S | E | S | G | E | H | I | V | A | G | T | G | E | L | H | L | E | I | C | L | Q | D | L | E | N | D | H | A | 550 | | A7THK9/1-842 | 496 | K | N | A | N | D | L | P | K | L | V | E | G | L | K | R | L | S | K | S | D | P | C | V | L | T | Y | M | S | E | S | G | E | H | I | V | A | G | T | G | E | L | H | L | E | I | C | L | Q | D | L | E | N | D | H | A | 550 | | C5DJC0/1-842 | 496 | K | N | A | N | D | L | P | K | L | V | E | G | L | K | R | L | S | K | S | D | P | C | V | L | C | Y | M | S | E | S | G | E | H | I | V | A | G | T | G | E | L | H | L | E | I | C | L | S | D | L | E | N | D | H | A | 550 | | C5DW13/1-842 | 496 | K | N | A | N | D | L | P | K | L | V | E | G | L | K | R | L | S | K | S | D | P | C | V | M | T | Y | I | S | E | S | G | E | H | I | V | A | G | T | G | E | L | H | L | E | I | C | L | Q | D | L | E | N | D | H | A | 550 | | Kwal\_55.21295/1-842 | 496 | K | N | A | N | D | L | P | K | L | V | E | G | L | K | R | L | S | K | S | D | P | C | V | L | T | Y | M | S | E | S | G | E | H | I | V | A | G | T | G | E | L | H | L | E | I | C | L | S | D | L | E | N | D | H | A | 550 | | Sbay\_530.4/1-842 | 496 | K | N | A | N | D | L | P | K | L | V | E | G | L | K | R | L | S | K | S | D | P | C | V | L | T | Y | M | S | E | S | G | E | H | I | V | A | G | T | G | E | L | H | L | E | I | C | L | Q | D | L | E | Q | D | H | A | 550 | | SAKL0G03388g/1-842 | 496 | K | N | A | N | D | L | P | K | L | V | E | G | L | K | R | L | S | K | S | D | P | C | V | L | T | Y | M | S | E | S | G | E | H | I | V | A | G | T | G | E | L | H | L | E | I | C | L | Q | D | L | E | N | D | H | A | 550 | | P32324/1-842 | 496 | K | N | A | N | D | L | P | K | L | V | E | G | L | K | R | L | S | K | S | D | P | C | V | L | T | Y | M | S | E | S | G | E | H | I | V | A | G | T | G | E | L | H | L | E | I | C | L | Q | D | L | E | H | D | H | A | 550 | |  | | G0V8E9/1-842 | 551 | G | V | P | L | K | I | S | P | P | V | V | A | Y | R | E | T | V | E | A | E | S | S | Q | T | A | L | S | K | S | P | N | K | H | N | R | I | Y | L | K | A | E | P | I | D | E | E | V | S | L | A | I | E | S | G | K | 605 | | Q6CPQ9/1-842 | 551 | G | I | P | L | K | I | S | P | P | V | V | A | Y | R | E | T | V | E | G | E | S | S | Q | T | A | L | S | K | S | P | N | K | H | N | R | I | Y | L | K | A | Q | P | I | D | E | E | V | S | L | A | I | E | G | G | K | 605 | | Q6FYA7/1-842 | 551 | G | I | P | L | K | I | S | P | P | V | V | A | Y | R | E | T | V | E | A | E | S | S | Q | V | A | L | S | K | S | P | N | K | H | N | R | I | Y | L | K | A | E | P | M | D | E | E | V | S | L | A | I | E | Q | G | K | 605 | | Q754C8/1-842 | 551 | G | I | P | L | K | I | S | P | P | V | V | A | Y | R | E | T | V | E | G | E | S | S | Q | V | A | L | S | K | S | P | N | K | H | N | R | I | Y | L | K | A | Q | P | I | D | E | E | V | S | L | A | I | E | G | G | K | 605 | | A7THK9/1-842 | 551 | G | I | P | L | K | I | S | P | P | V | V | A | Y | R | E | T | V | E | G | E | S | S | Q | T | A | L | S | K | S | P | N | K | H | N | R | I | Y | L | K | A | E | P | I | D | E | E | V | S | L | A | I | E | G | G | K | 605 | | C5DJC0/1-842 | 551 | G | I | P | L | K | I | S | P | P | V | V | A | Y | R | E | T | V | E | G | E | S | S | Q | V | A | L | S | K | S | P | N | K | H | N | R | I | Y | L | K | A | E | P | M | D | E | E | C | S | L | A | I | E | D | G | K | 605 | | C5DW13/1-842 | 551 | A | I | P | L | K | I | S | P | P | V | V | A | Y | R | E | T | V | E | G | E | S | S | Q | V | A | L | S | K | S | P | N | K | H | N | R | I | Y | L | K | A | E | P | I | D | E | E | V | S | L | A | I | E | N | G | K | 605 | | Kwal\_55.21295/1-842 | 551 | G | I | P | L | K | I | S | P | P | V | V | A | Y | R | E | T | V | E | G | E | S | S | Q | V | A | L | S | K | S | P | N | K | H | N | R | I | Y | L | K | A | Q | P | I | E | E | E | C | S | L | A | I | E | E | G | K | 605 | | Sbay\_530.4/1-842 | 551 | G | V | P | L | K | I | S | P | P | V | V | A | Y | R | E | T | V | E | T | E | S | S | Q | T | A | L | S | K | S | P | N | K | H | N | R | I | Y | L | K | A | E | P | I | E | E | E | V | S | L | A | I | E | N | G | V | 605 | | SAKL0G03388g/1-842 | 551 | G | I | P | L | K | I | S | P | P | V | V | A | Y | R | E | T | V | E | G | E | S | S | Q | V | A | L | S | K | S | P | N | K | H | N | R | I | Y | L | K | A | E | P | I | D | E | E | V | S | L | A | I | E | A | G | K | 605 | | P32324/1-842 | 551 | G | V | P | L | K | I | S | P | P | V | V | A | Y | R | E | T | V | E | S | E | S | S | Q | T | A | L | S | K | S | P | N | K | H | N | R | I | Y | L | K | A | E | P | I | D | E | E | V | S | L | A | I | E | N | G | I | 605 | |  | | G0V8E9/1-842 | 606 | I | N | P | R | D | D | L | K | A | R | A | R | V | M | A | D | E | F | G | W | D | V | T | D | A | R | K | I | W | C | F | G | P | D | G | N | G | P | N | L | V | V | D | Q | T | K | A | V | Q | Y | L | N | E | I | K | 660 | | Q6CPQ9/1-842 | 606 | I | N | P | R | D | D | F | K | A | R | A | R | I | M | A | D | E | F | G | W | D | V | T | D | A | R | K | I | W | C | F | G | P | D | G | N | G | P | N | L | V | V | D | Q | T | K | A | V | Q | Y | L | N | E | I | K | 660 | | Q6FYA7/1-842 | 606 | I | N | P | R | D | D | F | K | A | R | A | R | V | M | A | D | E | Y | G | W | D | V | T | D | A | R | K | I | W | C | F | G | P | D | G | N | G | P | N | L | V | V | D | Q | T | K | A | V | Q | Y | L | N | E | I | K | 660 | | Q754C8/1-842 | 606 | I | N | P | R | D | D | F | K | A | R | A | R | V | M | A | D | E | Y | G | W | D | V | T | D | A | R | K | I | W | C | F | G | P | D | G | N | G | P | N | L | V | V | D | Q | T | K | A | V | Q | Y | L | N | E | I | K | 660 | | A7THK9/1-842 | 606 | I | N | P | R | D | D | F | K | A | R | A | R | V | M | A | D | E | F | G | W | D | V | T | D | A | R | K | I | W | C | F | G | P | D | G | N | G | P | N | L | V | V | D | Q | T | K | A | V | Q | Y | L | N | E | I | K | 660 | | C5DJC0/1-842 | 606 | I | N | P | R | D | D | F | K | A | R | A | R | V | M | A | D | E | Y | G | W | D | V | T | D | A | R | K | I | W | C | F | G | P | D | G | N | G | P | N | V | V | V | D | Q | T | K | A | V | Q | Y | L | H | E | I | K | 660 | | C5DW13/1-842 | 606 | I | N | P | R | D | D | F | K | A | R | A | R | V | M | A | D | D | Y | G | W | D | V | T | D | A | R | K | I | W | C | F | G | P | D | G | N | G | P | N | L | V | I | D | Q | T | K | A | V | Q | Y | L | N | E | I | K | 660 | | Kwal\_55.21295/1-842 | 606 | I | N | P | R | D | D | F | K | A | R | A | R | I | M | A | D | E | F | G | W | D | V | T | D | A | R | K | I | W | C | F | G | P | D | G | N | G | P | N | V | V | V | D | Q | T | K | A | V | Q | Y | L | N | E | I | K | 660 | | Sbay\_530.4/1-842 | 606 | I | N | P | R | D | D | F | K | A | R | A | R | V | M | A | D | E | F | G | W | D | V | T | D | A | R | K | I | W | C | F | G | P | D | G | N | G | A | N | L | V | V | D | Q | T | K | A | V | Q | Y | L | H | E | I | K | 660 | | SAKL0G03388g/1-842 | 606 | I | N | P | R | D | D | F | K | A | R | A | R | V | M | A | D | D | F | G | W | D | V | T | D | A | R | K | I | W | C | F | G | P | D | G | N | G | P | N | L | V | V | D | Q | T | K | A | V | Q | Y | L | N | E | I | K | 660 | | P32324/1-842 | 606 | I | N | P | R | D | D | F | K | A | R | A | R | I | M | A | D | D | Y | G | W | D | V | T | D | A | R | K | I | W | C | F | G | P | D | G | N | G | P | N | L | V | I | D | Q | T | K | A | V | Q | Y | L | H | E | I | K | 660 | |  | | G0V8E9/1-842 | 661 | D | S | V | V | A | A | F | Q | W | A | T | K | E | G | P | I | F | G | E | Q | M | R | S | V | R | V | N | I | L | D | V | T | L | H | A | D | A | I | H | R | G | G | G | Q | I | I | P | T | M | R | R | A | T | Y | A | 715 | | Q6CPQ9/1-842 | 661 | D | S | V | V | A | A | F | Q | W | A | T | K | E | G | P | I | F | G | E | Q | M | R | S | V | R | V | N | I | L | D | V | T | L | H | A | D | A | I | H | R | G | G | G | Q | I | I | P | T | M | R | R | A | T | Y | A | 715 | | Q6FYA7/1-842 | 661 | D | S | V | V | S | A | F | Q | W | A | T | K | E | G | P | I | L | G | E | T | M | R | S | V | R | V | N | I | L | D | V | T | L | H | A | D | A | I | H | R | G | A | G | Q | I | M | P | T | M | R | R | A | T | Y | A | 715 | | Q754C8/1-842 | 661 | D | S | V | V | S | A | F | Q | W | A | T | K | E | G | P | I | F | G | E | Q | M | R | S | V | R | I | N | L | L | D | V | T | L | H | A | D | A | I | H | R | G | A | G | Q | I | M | P | T | M | R | R | A | T | Y | A | 715 | | A7THK9/1-842 | 661 | D | S | V | V | A | A | F | Q | W | A | T | K | E | G | P | I | F | G | E | Q | M | R | S | V | R | V | N | I | L | D | V | T | L | H | A | D | A | I | H | R | G | G | G | Q | I | I | P | T | M | R | R | A | T | Y | A | 715 | | C5DJC0/1-842 | 661 | D | S | V | V | A | A | F | Q | W | A | S | K | E | G | P | I | F | G | E | Q | M | R | S | V | R | V | N | I | L | D | V | T | L | H | A | D | A | I | H | R | G | G | G | Q | I | I | P | T | M | R | R | A | T | Y | A | 715 | | C5DW13/1-842 | 661 | D | S | V | V | A | A | F | Q | W | A | S | K | E | G | P | I | F | G | E | Q | M | R | S | V | R | V | N | I | L | D | V | T | L | H | A | D | A | I | H | R | G | G | G | Q | I | I | P | T | M | R | R | A | T | Y | A | 715 | | Kwal\_55.21295/1-842 | 661 | D | S | V | V | A | A | F | Q | W | A | S | K | E | G | P | I | F | G | E | Q | M | R | S | V | R | I | N | I | L | D | V | T | L | H | A | D | A | I | H | R | G | G | G | Q | I | I | P | T | M | R | R | A | T | Y | A | 715 | | Sbay\_530.4/1-842 | 661 | D | S | V | V | A | A | F | Q | W | A | T | K | E | G | P | I | F | G | E | E | M | R | S | V | R | V | N | I | L | D | V | T | L | H | A | D | A | I | H | R | G | G | G | Q | I | I | P | T | M | R | R | A | T | Y | A | 715 | | SAKL0G03388g/1-842 | 661 | D | S | V | V | A | A | F | Q | W | A | S | K | E | G | P | I | F | G | E | Q | M | R | S | V | R | V | N | I | L | D | V | T | L | H | A | D | A | I | H | R | G | G | G | Q | I | I | P | T | M | R | R | A | T | Y | A | 715 | | P32324/1-842 | 661 | D | S | V | V | A | A | F | Q | W | A | T | K | E | G | P | I | F | G | E | E | M | R | S | V | R | V | N | I | L | D | V | T | L | H | A | D | A | I | H | R | G | G | G | Q | I | I | P | T | M | R | R | A | T | Y | A | 715 | |  | | G0V8E9/1-842 | 716 | G | F | L | L | A | E | P | K | I | Q | E | P | V | F | L | V | E | I | Q | C | P | E | S | A | V | G | G | I | Y | S | V | L | N | K | K | R | G | Q | V | V | S | E | E | Q | R | P | G | T | P | L | F | T | V | K | A | 770 | | Q6CPQ9/1-842 | 716 | G | F | L | L | A | E | P | K | I | Q | E | P | V | F | L | V | E | I | Q | C | P | E | Q | A | I | G | G | I | Y | S | V | L | N | K | K | R | G | Q | V | V | S | E | E | Q | R | P | G | T | P | L | F | T | V | K | A | 770 | | Q6FYA7/1-842 | 716 | G | F | L | L | A | E | P | K | I | Q | E | P | V | F | L | V | E | I | Q | C | P | E | Q | A | V | G | G | I | Y | S | V | L | N | K | K | R | G | Q | V | V | S | E | E | Q | R | P | G | T | P | L | F | T | V | K | A | 770 | | Q754C8/1-842 | 716 | G | F | L | L | A | E | P | K | I | Q | E | P | V | F | L | V | E | I | Q | C | P | E | Q | A | V | G | G | I | Y | S | V | L | N | R | K | R | G | Q | V | V | S | E | E | Q | R | P | G | T | P | L | F | T | V | K | A | 770 | | A7THK9/1-842 | 716 | G | F | L | L | A | E | P | K | I | Q | E | P | V | F | L | V | E | I | Q | C | P | E | Q | A | V | G | G | I | Y | S | V | L | N | K | K | R | G | Q | V | V | S | E | E | Q | R | P | G | T | P | L | F | T | V | K | A | 770 | | C5DJC0/1-842 | 716 | G | F | L | L | A | E | P | K | I | Q | E | P | V | F | L | V | E | I | Q | C | P | E | Q | A | V | G | G | I | Y | S | V | L | N | K | K | R | G | Q | V | V | S | E | E | Q | R | P | G | T | P | L | F | T | V | K | A | 770 | | C5DW13/1-842 | 716 | G | F | L | L | A | E | P | R | I | Q | E | P | V | F | M | V | E | I | Q | C | P | E | Q | A | V | G | G | I | Y | S | V | L | N | K | R | R | G | Q | V | V | S | E | E | Q | R | P | G | T | P | L | F | T | V | K | A | 770 | | Kwal\_55.21295/1-842 | 716 | G | F | L | L | A | E | P | K | I | Q | E | P | V | F | L | V | E | I | Q | C | P | E | Q | A | V | G | G | I | Y | S | V | L | N | K | K | R | G | Q | V | V | S | E | E | Q | R | P | G | T | P | L | F | T | V | K | A | 770 | | Sbay\_530.4/1-842 | 716 | G | F | L | L | A | E | P | K | I | Q | E | P | V | F | L | V | E | I | Q | C | P | E | Q | A | V | G | G | I | Y | S | V | L | N | K | K | R | G | Q | V | V | S | E | E | Q | R | P | G | T | P | L | F | T | V | K | A | 770 | | SAKL0G03388g/1-842 | 716 | G | F | L | L | A | E | P | K | I | Q | E | P | V | F | L | V | E | I | Q | C | P | E | Q | A | V | G | G | I | Y | S | V | L | N | R | K | R | G | Q | V | V | S | E | E | Q | R | P | G | T | P | L | F | T | V | K | A | 770 | | P32324/1-842 | 716 | G | F | L | L | A | D | P | K | I | Q | E | P | V | F | L | V | E | I | Q | C | P | E | Q | A | V | G | G | I | Y | S | V | L | N | K | K | R | G | Q | V | V | S | E | E | Q | R | P | G | T | P | L | F | T | V | K | A | 770 | |  | | G0V8E9/1-842 | 771 | Y | L | P | V | N | E | S | F | G | F | T | G | E | L | R | Q | A | T | G | G | Q | A | F | P | Q | M | V | F | D | H | W | A | T | L | G | S | D | P | L | D | P | T | S | K | A | G | E | I | V | T | A | A | R | K | R | 825 | | Q6CPQ9/1-842 | 771 | Y | L | P | I | N | E | S | F | G | F | T | G | E | L | R | Q | A | T | G | G | Q | A | F | P | Q | M | V | F | D | H | W | A | T | L | G | T | D | P | L | D | P | S | T | K | A | G | E | I | V | L | A | A | R | K | R | 825 | | Q6FYA7/1-842 | 771 | Y | L | P | V | N | E | S | F | G | F | T | G | E | L | R | Q | A | T | G | G | Q | A | F | P | Q | M | V | F | D | H | W | A | T | L | N | S | D | P | L | D | P | T | S | K | A | G | E | I | V | T | A | A | R | K | R | 825 | | Q754C8/1-842 | 771 | Y | L | P | V | N | E | S | F | G | F | T | G | E | L | R | Q | A | T | G | G | Q | A | F | P | Q | M | V | F | D | H | W | A | T | L | G | T | D | P | L | D | P | T | T | K | A | G | E | I | V | V | E | A | R | K | R | 825 | | A7THK9/1-842 | 771 | Y | L | P | V | N | E | S | F | G | F | T | G | E | L | R | Q | A | T | G | G | Q | A | F | P | Q | M | V | F | D | H | W | A | T | L | G | T | D | P | L | D | P | T | T | K | A | G | E | I | V | A | A | S | R | K | R | 825 | | C5DJC0/1-842 | 771 | Y | L | P | V | N | E | S | F | G | F | T | G | Q | L | R | Q | A | T | G | G | Q | A | F | P | Q | M | V | F | D | H | W | A | T | L | S | A | D | P | L | D | P | S | S | K | A | G | E | I | V | A | A | A | R | K | R | 825 | | C5DW13/1-842 | 771 | H | L | P | V | N | E | S | F | G | F | T | G | E | L | R | Q | A | T | G | G | Q | A | F | P | Q | M | V | F | D | H | W | S | S | L | S | S | D | P | L | D | P | E | T | K | A | G | Q | I | V | T | A | A | R | K | R | 825 | | Kwal\_55.21295/1-842 | 771 | Y | L | P | V | N | E | S | F | G | F | T | G | Q | L | R | Q | A | T | G | G | Q | A | F | P | Q | M | V | F | D | H | W | A | T | L | G | A | D | P | L | D | P | S | S | K | A | G | E | I | V | A | A | A | R | K | R | 825 | | Sbay\_530.4/1-842 | 771 | Y | L | P | V | N | E | S | F | G | F | T | G | E | L | R | Q | A | T | G | G | Q | A | F | P | Q | M | V | F | D | H | W | A | T | L | G | S | D | P | L | D | P | S | S | K | A | G | E | I | V | L | A | A | R | K | R | 825 | | SAKL0G03388g/1-842 | 771 | Y | L | P | V | N | E | S | F | G | F | T | G | E | L | R | Q | A | T | G | G | Q | A | F | P | Q | M | V | F | D | H | W | A | T | L | G | T | D | P | L | D | P | T | T | K | A | G | E | I | V | T | A | A | R | K | R | 825 | | P32324/1-842 | 771 | Y | L | P | V | N | E | S | F | G | F | T | G | E | L | R | Q | A | T | G | G | Q | A | F | P | Q | M | V | F | D | H | W | S | T | L | G | S | D | P | L | D | P | T | S | K | A | G | E | I | V | L | A | A | R | K | R | 825 | |  | | G0V8E9/1-842 | 826 | H | G | M | K | D | V | V | P | G | W | Q | E | Y | Y | D | K | L |  | | | | | | | | | | | | | | | | | | | | | | | | | | | | | | | | | | | | | | 842 | | Q6CPQ9/1-842 | 826 | Q | G | M | K | E | E | V | P | G | W | Q | E | Y | Y | D | K | L |  | | | | | | | | | | | | | | | | | | | | | | | | | | | | | | | | | | | | | | 842 | | Q6FYA7/1-842 | 826 | H | G | M | K | E | E | V | P | G | W | Q | E | Y | Y | D | K | L |  | | | | | | | | | | | | | | | | | | | | | | | | | | | | | | | | | | | | | | 842 | | Q754C8/1-842 | 826 | H | G | L | K | E | N | V | P | G | W | Q | E | Y | Y | D | K | L |  | | | | | | | | | | | | | | | | | | | | | | | | | | | | | | | | | | | | | | 842 | | A7THK9/1-842 | 826 | R | G | M | K | E | E | V | P | G | W | Q | E | Y | Y | D | K | L |  | | | | | | | | | | | | | | | | | | | | | | | | | | | | | | | | | | | | | | 842 | | C5DJC0/1-842 | 826 | H | G | M | K | E | E | V | P | G | W | Q | E | Y | Y | D | K | L |  | | | | | | | | | | | | | | | | | | | | | | | | | | | | | | | | | | | | | | 842 | | C5DW13/1-842 | 826 | H | G | M | K | E | E | V | P | G | W | Q | E | Y | Y | D | K | L |  | | | | | | | | | | | | | | | | | | | | | | | | | | | | | | | | | | | | | | 842 | | Kwal\_55.21295/1-842 | 826 | H | G | M | K | E | E | V | P | G | W | Q | E | Y | Y | D | K | L |  | | | | | | | | | | | | | | | | | | | | | | | | | | | | | | | | | | | | | | 842 | | Sbay\_530.4/1-842 | 826 | H | G | M | K | E | E | V | P | G | W | Q | E | Y | Y | D | K | L |  | | | | | | | | | | | | | | | | | | | | | | | | | | | | | | | | | | | | | | 842 | | SAKL0G03388g/1-842 | 826 | H | G | M | K | E | E | V | P | G | W | Q | E | Y | Y | D | K | L |  | | | | | | | | | | | | | | | | | | | | | | | | | | | | | | | | | | | | | | 842 | | P32324/1-842 | 826 | H | G | M | K | E | E | V | P | G | W | Q | E | Y | Y | D | K | L |  | | | | | | | | | | | | | | | | | | | | | | | | | | | | | | | | | | | | | | 842 | |
