# Supplementary material for: Plus ça change – evolutionary sequence divergence predicts protein subcellular localization signals
Source: BMC Genomics. 2014 Jan 20;15:46. doi: 10.1186/1471-2164-15-46 (PMC3906766; doi:10.1186/1471-2164-15-46)
Supplement: Additional file 2 — MSA’s of proteins for which sequence divergence changes predicted localization signals. Contains links to ortholog multiple sequence alignments of each protein in Additional file 3: Table S1. [file 1471-2164-15-46-S2.zip › P32333.html]

|  |  |  |  |  |  |  |  |  |  |  |  |  |  |  |  |  |  |  |  |  |  |  |  |  |  |  |  |  |  |  |  |  |  |  |  |  |  |  |  |  |  |  |  |  |  |  |  |  |  |  |  |  |  |  |  |  |  |  |  |  |  |  |  |  |  |  |  |  |  |  |  |  |  |  |  |  |  |  |  |  |  |  |  |  |  |  |  |  |  |  |  |  |  |  |  |  |  |  |  |  |  |  |  |  |  |  |  |  |  |  |  |  |  |  |  |  |  |  |  |  |  |  |  |  |  |  |  |  |  |  |  |  |  |  |  |  |  |  |  |  |  |  |  |  |  |  |  |  |  |  |  |  |  |  |  |  |  |  |  |  |  |  |  |  |  |  |  |  |  |  |  |  |  |  |  |  |  |  |  |  |  |  |  |  |  |  |  |  |  |  |  |  |  |  |  |  |  |  |  |  |  |  |  |  |  |  |  |  |  |  |  |  |  |  |  |  |  |  |  |  |  |  |  |  |  |  |  |  |  |  |  |  |  |  |  |  |  |  |  |  |  |  |  |  |  |  |  |  |  |  |  |  |  |  |  |  |  |  |  |  |  |  |  |  |  |  |  |  |  |  |  |  |  |  |  |  |  |  |  |  |  |  |  |  |  |  |  |  |  |  |  |  |  |  |  |  |  |  |  |  |  |  |  |  |  |  |  |  |  |  |  |  |  |  |  |  |  |  |  |  |  |  |  |  |  |  |  |  |  |  |  |  |  |  |  |  |  |  |  |  |  |  |  |  |  |  |  |  |  |  |  |  |  |  |  |  |  |  |  |  |  |  |  |  |  |  |  |  |  |  |  |  |  |  |  |  |  |  |  |  |  |  |  |  |  |  |  |  |  |  |  |  |  |  |  |  |  |  |  |  |  |  |  |  |  |  |  |  |  |  |  |  |  |  |  |  |  |  |  |  |  |  |  |  |  |  |  |  |  |  |  |  |  |  |  |  |  |  |  |  |  |  |  |  |  |  |  |  |  |  |  |  |  |  |  |  |  |  |  |  |  |  |  |  |  |  |  |  |  |  |  |  |  |  |  |  |  |  |  |  |  |  |  |  |  |  |  |  |  |  |  |  |  |  |  |  |  |  |  |  |  |  |  |  |  |  |  |  |  |  |  |  |  |  |  |  |  |  |  |  |  |  |  |  |  |  |  |  |  |  |  |  |  |  |  |  |  |  |  |  |  |  |  |  |  |  |  |  |  |  |  |  |  |  |  |  |  |  |  |  |  |  |  |  |  |  |  |  |  |  |  |  |  |  |  |  |  |  |  |  |  |  |  |  |  |  |  |  |  |  |  |  |  |  |  |  |  |  |  |  |  |  |  |  |  |  |  |  |  |  |  |  |  |  |  |  |  |  |  |  |  |  |  |  |  |  |  |  |  |  |  |  |  |  |  |  |  |  |  |  |  |  |  |  |  |  |  |  |  |  |  |  |  |  |  |  |  |  |  |  |  |  |  |  |  |  |  |  |  |  |  |  |  |  |  |  |  |  |  |  |  |  |  |  |  |  |  |  |  |  |  |  |  |  |  |  |  |  |  |  |  |  |  |  |  |  |  |  |  |  |  |  |  |  |  |  |  |  |  |  |  |  |  |  |  |  |  |  |  |  |  |  |  |  |  |  |  |  |  |  |  |  |  |  |  |  |  |  |  |  |  |  |  |  |  |  |  |  |  |  |  |  |  |  |  |  |  |  |  |  |  |  |  |  |  |  |  |  |  |  |  |  |  |  |  |  |  |  |  |  |  |  |  |  |  |  |  |  |  |  |  |  |  |  |  |  |  |  |  |  |  |  |  |  |  |  |  |  |  |  |  |  |  |  |  |  |  |  |  |  |  |  |  |  |  |  |  |  |  |  |  |  |  |  |  |  |  |  |  |  |  |  |  |  |  |  |  |  |  |  |  |  |  |  |  |  |  |  |  |  |  |  |  |  |  |  |  |  |  |  |  |  |  |  |  |  |  |  |  |  |  |  |  |  |  |  |  |  |  |  |  |  |  |  |  |  |  |  |  |  |  |  |  |  |  |  |  |  |  |  |  |  |  |  |  |  |  |  |  |  |  |  |  |  |  |  |  |  |  |  |  |  |  |  |  |  |  |  |  |  |  |  |  |  |  |  |  |  |  |  |  |  |  |  |  |  |  |  |  |  |  |  |  |  |  |  |  |  |  |  |  |  |  |  |  |  |  |  |  |  |  |  |  |  |  |  |  |  |  |  |  |  |  |  |  |  |  |  |  |  |  |  |  |  |  |  |  |  |  |  |  |  |  |  |  |  |  |  |  |  |  |  |  |  |  |  |  |  |  |  |  |  |  |  |  |  |  |  |  |  |  |  |  |  |  |  |  |  |  |  |  |  |  |  |  |  |  |  |  |  |  |  |  |  |  |  |  |  |  |  |  |  |  |  |  |  |  |  |  |  |  |  |  |  |  |  |  |  |  |  |  |  |  |  |  |  |  |  |  |  |  |  |  |  |  |  |  |  |  |  |  |  |  |  |  |  |  |  |  |  |  |  |  |  |  |  |  |  |  |  |  |  |  |  |  |  |  |  |  |  |  |  |  |  |  |  |  |  |  |  |  |  |  |  |  |  |  |  |  |  |  |  |  |  |  |  |  |  |  |  |  |  |  |  |  |  |  |  |  |  |  |  |  |  |  |  |  |  |  |  |  |  |  |  |  |  |  |  |  |  |  |  |  |  |  |  |  |  |  |  |  |  |  |  |  |  |  |  |  |  |  |  |  |  |  |  |  |  |  |  |  |  |  |  |  |  |  |  |  |  |  |  |  |  |  |  |  |  |  |  |  |  |  |  |  |  |  |  |  |  |  |  |  |  |  |  |  |  |  |  |  |  |  |  |  |  |  |  |  |  |  |  |  |  |  |  |  |  |  |  |  |  |  |  |  |  |  |  |  |  |  |  |  |  |  |  |  |  |  |  |  |  |  |  |  |  |  |  |  |  |  |  |  |  |  |  |  |  |  |  |  |  |  |  |  |  |  |  |  |  |  |  |  |  |  |  |  |  |  |  |  |  |  |  |  |  |  |  |  |  |  |  |  |  |  |  |  |  |  |  |  |  |  |  |  |  |  |  |  |  |  |  |  |  |  |  |  |  |  |  |  |  |  |  |  |  |  |  |  |  |  |  |  |  |  |  |  |  |  |  |  |  |  |  |  |  |  |  |  |  |  |  |  |  |  |  |  |  |  |  |  |  |  |  |  |  |  |  |  |  |  |  |  |  |  |  |  |  |  |  |  |  |  |  |  |  |  |  |  |  |  |  |  |  |  |  |  |  |  |  |  |  |  |  |  |  |  |  |  |  |  |  |  |  |  |  |  |  |  |  |  |  |  |  |  |  |  |  |  |  |  |  |  |  |  |  |  |  |  |  |  |  |  |  |  |  |  |  |  |  |  |  |  |  |  |  |  |  |  |  |  |  |  |  |  |  |  |  |  |  |  |  |  |  |  |  |  |  |  |  |  |  |  |  |  |  |  |  |  |  |  |  |  |  |  |  |  |  |  |  |  |  |  |  |  |  |  |  |  |  |  |  |  |  |  |  |  |  |  |  |  |  |  |  |  |  |  |  |  |  |  |  |  |  |  |  |  |  |  |  |  |  |  |  |  |  |  |  |  |  |  |  |  |  |  |  |  |  |  |  |  |  |  |  |  |  |  |  |  |  |  |  |  |  |  |  |  |  |  |  |  |  |  |  |  |  |  |  |  |  |  |  |  |  |  |  |  |  |  |  |  |  |  |  |  |  |  |  |  |  |  |  |  |  |  |  |  |  |  |  |  |  |  |  |  |  |  |  |  |  |  |  |  |  |  |  |  |  |  |  |  |  |  |  |  |  |  |  |  |  |  |  |  |  |  |  |  |  |  |  |  |  |  |  |  |  |  |  |  |  |  |  |  |  |  |  |  |  |  |  |  |  |  |  |  |  |  |  |  |  |  |  |  |  |  |  |  |  |  |  |  |  |  |  |  |  |  |  |  |  |  |  |  |  |  |  |  |  |  |  |  |  |  |  |  |  |  |  |  |  |  |  |  |  |  |  |  |  |  |  |  |  |  |  |  |  |  |  |  |  |  |  |  |  |  |  |  |  |  |  |  |  |  |  |  |  |  |  |  |  |  |  |  |  |  |  |  |  |  |  |  |  |  |  |  |  |  |  |  |  |  |  |  |  |  |  |  |  |  |  |  |  |  |  |  |  |  |  |  |  |  |  |  |  |  |  |  |  |  |  |  |  |  |  |  |  |  |  |  |  |  |  |  |  |  |  |  |  |  |  |  |  |  |  |  |  |  |  |  |  |  |  |  |  |  |  |  |  |  |  |  |  |  |  |  |  |  |  |  |  |  |  |  |  |  |  |  |  |  |  |  |  |  |  |  |  |  |  |  |  |  |  |  |  |  |  |  |  |  |  |  |  |  |  |  |  |  |  |  |  |  |  |  |  |  |  |  |  |  |  |  |  |  |  |  |  |  |  |  |  |  |  |  |  |  |  |  |  |  |  |  |  |  |  |  |  |  |  |  |  |  |  |  |  |  |  |  |  |  |  |  |  |  |  |  |  |  |  |  |  |  |  |  |  |  |  |  |  |  |  |  |  |  |  |  |  |  |  |  |  |  |  |  |  |  |  |  |  |  |  |  |  |  |  |  |  |  |  |  |  |  |  |  |  |  |  |  |  |  |  |  |  |  |  |  |  |  |  |  |  |  |  |  |  |  |  |  |  |  |  |  |  |  |  |  |  |  |  |  |  |  |  |  |  |  |  |  |  |  |  |  |  |  |  |  |  |  |  |  |  |  |  |  |  |  |  |  |  |  |  |  |  |  |  |  |  |  |  |  |  |  |  |  |  |  |  |  |  |  |  |  |  |  |  |  |  |  |  |  |  |  |  |  |  |  |  |  |  |  |  |  |  |  |  |  |  |  |  |  |  |  |  |  |  |  |  |  |  |  |  |  |  |  |  |  |  |  |  |  |  |  |  |  |  |  |  |  |  |  |  |  |  |  |  |  |  |  |  |  |  |  |  |  |  |  |  |  |  |  |  |  |  |  |  |  |  |  |  |  |  |  |  |  |  |  |  |  |  |  |  |  |  |  |  |  |  |  |  |  |  |  |  |  |  |  |  |  |  |  |  |  |  |  |  |  |  |  |  |  |  |  |  |  |  |  |  |  |  |  |  |  |  |  |  |  |  |  |  |  |  |  |  |  |  |  |  |  |  |  |  |  |  |  |  |  |  |  |  |  |  |  |  |  |  |  |  |  |  |  |  |  |  |  |  |  |  |  |  |  |  |  |  |  |  |  |  |  |  |  |  |  |  |  |  |  |  |  |  |  |  |  |  |  |  |  |  |  |  |  |  |  |  |  |  |  |  |  |  |  |  |  |  |  |  |  |  |  |  |  |  |  |  |  |  |  |  |  |  |  |  |  |  |  |  |  |  |  |  |  |  |  |  |  |  |  |  |  |  |  |  |  |  |  |  |  |  |  |  |  |  |  |  |  |  |  |  |  |  |  |  |  |  |  |  |  |  |  |  |  |  |  |  |  |  |  |  |  |  |  |  |  |  |  |  |  |  |  |  |  |  |  |  |  |  |  |  |  |  |  |  |  |  |  |  |  |  |  |  |  |  |  |  |  |  |  |  |  |  |  |  |  |  |  |  |  |  |  |  |  |  |  |  |  |  |  |  |  |  |  |  |  |  |  |  |  |  |  |  |  |  |  |  |  |  |  |  |  |  |  |  |  |  |  |  |  |  |  |  |  |  |  |  |  |  |  |  |  |  |  |  |  |  |  |  |  |  |  |  |  |  |  |  |  |  |  |  |  |  |  |  |  |  |  |  |  |  |  |  |  |  |  |  |  |  |  |  |  |  |  |  |  |  |  |  |  |  |  |  |  |  |  |  |  |  |  |  |  |  |  |  |  |  |  |  |  |  |  |  |  |  |  |  |  |  |  |  |  |  |  |  |  |  |  |  |  |  |  |  |  |  |  |  |  |  |  |  |  |  |  |  |  |  |  |  |  |  |  |  |  |  |  |  |  |  |  |  |  |  |  |  |  |  |  |  |  |  |  |  |  |  |  |  |  |  |  |  |  |  |  |  |  |  |  |  |  |  |  |  |  |  |  |  |  |  |  |  |  |  |  |  |  |  |  |  |  |  |  |  |  |  |  |  |  |  |  |  |  |  |  |  |  |  |  |  |  |  |  |  |  |  |  |  |  |  |  |  |  |  |  |  |  |  |  |  |  |  |  |  |  |  |  |  |  |  |  |  |  |  |  |  |  |  |  |  |  |  |  |  |  |  |  |  |  |  |  |  |  |  |  |  |  |  |  |  |  |  |  |  |  |  |  |  |  |  |  |  |  |  |  |  |  |  |  |  |  |  |  |  |  |  |  |  |  |  |  |  |  |  |  |  |  |  |  |  |  |  |  |  |  |  |  |  |  |  |  |  |  |  |  |  |  |  |  |  |  |  |  |  |  |  |  |  |  |  |  |  |  |  |  |  |  |  |  |  |  |  |  |  |  |  |  |  |  |  |  |  |  |  |  |  |  |  |  |  |  |  |  |  |  |  |  |  |  |  |  |  |  |  |  |  |  |  |  |  |  |  |  |  |  |  |  |  |  |  |  |  |  |  |  |  |  |  |  |  |  |  |  |  |  |  |  |  |  |  |  |  |  |  |  |  |  |  |  |  |  |  |  |  |  |  |  |  |  |  |  |  |  |  |  |  |  |  |  |  |  |  |  |  |  |  |  |  |  |  |  |  |  |  |  |  |  |  |  |  |  |  |  |  |  |  |  |  |  |  |  |  |  |  |  |  |  |  |  |  |  |  |  |  |  |  |  |  |  |  |  |  |  |  |  |  |  |  |  |  |  |  |  |  |  |  |  |  |  |  |  |  |  |  |  |  |  |  |  |  |  |  |  |  |  |  |  |  |  |  |  |  |  |  |  |  |  |  |  |  |  |  |  |  |  |  |  |  |  |  |  |  |  |  |  |  |  |  |  |  |  |  |  |  |  |  |  |  |  |  |  |  |  |  |  |  |  |  |  |  |  |  |  |  |  |  |  |  |  |  |  |  |  |  |  |  |  |  |  |  |  |  |  |  |  |  |  |  |  |  |  |  |  |  |  |  |  |  |  |  |  |  |  |  |  |  |  |  |  |  |  |  |  |  |  |  |  |  |  |  |  |  |  |  |  |  |  |  |  |  |  |  |  |  |  |  |  |  |  |  |  |  |  |  |  |  |  |  |  |  |  |  |  |  |  |  |  |  |  |  |  |  |  |  |  |  |  |  |  |  |  |  |  |  |  |  |  |  |  |  |  |  |  |  |  |  |  |  |  |  |  |  |  |  |  |  |  |  |  |  |  |  |  |  |  |  |  |  |  |  |  |  |  |  |  |  |  |  |  |  |  |  |  |  |  |  |  |  |  |  |  |  |  |  |  |  |  |  |  |  |  |  |  |  |  |  |  |  |  |  |  |  |  |  |  |  |  |  |  |  |  |  |  |  |  |  |  |  |  |  |  |  |  |  |  |  |  |  |  |  |  |  |  |  |  |  |  |  |  |  |  |  |  |  |  |  |  |  |  |  |  |  |  |  |  |  |  |  |  |  |  |  |  |  |  |  |  |  |  |  |  |  |  |  |  |  |  |  |  |  |  |  |  |  |  |  |  |  |  |  |  |  |  |  |  |  |  |  |  |  |  |  |  |  |  |  |  |  |  |  |  |  |  |  |  |  |  |  |  |  |  |  |  |  |  |  |  |  |  |  |  |  |  |  |  |  |  |  |  |  |  |  |  |  |  |  |  |  |  |  |  |  |  |  |  |  |  |  |  |  |  |  |  |  |  |  |  |  |  |  |  |  |  |  |  |  |  |  |  |  |  |  |  |  |  |  |  |  |  |  |  |  |  |  |  |  |  |  |  |  |  |  |  |  |  |  |  |  |  |  |  |  |  |  |  |  |  |  |  |  |  |  |  |  |  |  |  |  |  |  |  |  |  |  |  |  |  |  |  |  |  |  |  |  |  |  |  |  |  |  |  |  |  |  |  |  |  |  |  |  |  |  |  |  |  |  |  |  |  |  |  |  |  |  |  |  |  |  |  |  |  |  |  |  |  |  |  |  |  |  |  |  |  |  |  |  |  |  |  |  |  |  |  |  |  |  |  |  |  |  |  |  |  |  |  |  |  |  |  |  |  |  |  |  |  |  |  |  |  |  |  |  |  |  |  |  |  |  |  |  |  |  |  |  |  |  |  |  |  |  |  |  |  |  |  |  |  |  |  |  |  |  |  |  |  |  |  |  |  |  |  |  |  |  |  |  |  |  |  |  |  |  |  |  |  |  |  |  |  |  |  |  |  |  |  |  |  |  |  |  |  |  |  |  |  |  |  |  |  |  |  |  |  |  |  |  |  |  |  |  |  |  |  |  |  |  |  |  |  |  |  |  |  |  |  |  |  |  |  |  |  |  |  |  |  |  |  |  |  |  |  |  |  |  |  |  |  |  |  |  |  |  |  |  |  |  |  |  |  |  |  |  |  |  |  |  |  |  |  |  |  |  |  |  |  |  |  |  |  |  |  |  |  |  |  |  |  |  |  |  |  |  |  |  |  |  |  |  |  |  |  |  |  |  |  |  |  |  |  |  |  |  |  |  |  |  |  |  |  |  |  |  |  |  |  |  |  |  |  |  |  |  |  |  |  |  |  |  |  |  |  |  |  |  |  |  |  |  |  |  |  |  |  |  |  |  |  |  |  |  |  |  |  |  |  |  |  |  |  |  |  |  |  |  |  |  |  |  |  |  |  |  |  |  |  |  |  |  |  |  |  |  |  |  |  |  |  |  |  |  |  |  |  |  |  |  |  |  |  |  |  |  |  |  |  |  |  |  |  |  |  |  |  |  |  |  |  |  |  |  |  |  |  |  |  |  |  |  |  |  |  |  |  |  |  |  |  |  |  |  |  |  |  |  |  |  |  |  |  |  |  |  |  |  |  |  |  |  |  |  |  |  |  |  |  |  |  |  |  |  |  |  |  |  |  |  |  |  |  |  |  |  |  |  |  |  |  |  |  |  |  |  |  |  |  |  |  |  |  |  |  |  |  |  |  |  |  |  |  |  |  |  |  |  |  |  |  |  |  |  |  |  |  |  |  |  |  |  |  |  |  |  |  |  |  |  |  |  |  |  |  |  |  |  |  |  |  |  |  |  |  |  |  |  |  |  |  |  |  |  |  |  |  |  |  |  |  |  |  |  |  |  |  |  |  |  |  |  |  |  |  |  |  |  |  |  |  |  |  |  |  |  |  |  |  |  |  |  |  |  |  |  |  |  |  |  |  |  |  |  |  |  |  |  |  |  |  |  |  |  |  |  |  |  |  |  |  |  |  |  |  |  |  |  |  |  |  |  |  |  |  |  |  |  |  |  |  |  |  |  |  |  |  |  |  |  |  |  |  |  |  |  |  |  |  |  |  |  |  |  |  |  |  |  |  |  |  |  |  |  |  |  |  |  |  |  |  |  |  |  |  |  |  |  |  |  |  |  |  |  |  |  |  |  |  |  |  |  |  |  |  |  |  |  |  |  |  |  |  |  |  |  |  |  |  |  |  |  |  |  |  |  |  |  |  |  |  |  |  |  |  |  |  |  |  |  |  |  |  |  |  |  |  |  |  |  |  |  |  |  |  |  |  |  |  |  |  |  |  |  |  |  |  |  |  |  |  |  |  |  |  |  |  |  |  |  |  |  |  |  |  |  |  |  |  |  |  |  |  |  |  |  |  |  |  |  |  |  |  |  |  |  |  |  |  |  |  |  |  |  |  |  |  |  |  |  |  |  |  |  |  |  |  |  |  |  |  |  |  |  |  |  |  |  |  |  |  |  |  |  |  |  |  |  |  |  |  |  |  |  |  |  |  |  |  |  |  |  |  |  |  |  |  |  |  |  |  |  |  |  |  |  |  |  |  |  |  |  |  |  |  |  |  |  |  |  |  |  |  |  |  |  |  |  |  |  |  |  |  |  |  |  |  |  |  |  |  |  |  |  |  |  |  |  |  |  |  |  |  |  |  |  |  |  |  |  |  |  |  |  |  |  |  |  |  |  |  |  |  |  |  |  |  |  |  |  |  |  |  |  |  |  |  |  |  |  |  |  |  |  |  |  |  |  |  |  |  |  |  |  |  |  |  |  |  |  |  |  |  |  |  |  |  |  |  |  |  |  |  |  |  |  |  |  |  |  |  |  |  |  |  |  |  |  |  |  |  |  |  |  |  |  |  |  |  |  |  |  |  |  |  |  |  |  |  |  |  |  |  |  |  |  |  |  |  |  |  |  |  |  |  |  |  |  |  |  |  |  |  |  |  |  |  |  |  |  |  |  |  |  |  |  |  |  |  |  |  |  |  |  |  |  |  |  |  |  |  |  |  |  |  |  |  |  |  |  |  |  |  |  |  |  |  |  |  |  |  |  |  |  |  |  |  |  |  |  |  |  |  |  |  |  |  |  |  |  |  |  |  |  |  |  |  |  |  |  |  |  |  |  |  |  |  |  |  |  |  |  |  |  |  |  |  |  |  |  |  |  |  |  |  |  |  |  |  |  |  |  |  |  |  |  |  |  |  |  |  |  |  |  |  |  |  |  |  |  |  |  |  |  |  |  |  |  |  |  |  |  |  |  |  |  |  |  |  |  |  |  |  |  |  |  |  |  |  |  |  |  |  |  |  |  |  |  |  |  |  |  |  |  |  |  |  |  |  |  |  |  |  |  |  |  |  |  |  |  |  |  |  |  |  |  |  |  |  |  |  |  |  |  |  |  |  |  |  |  |  |  |  |  |  |  |  |  |  |  |  |  |  |  |  |  |  |  |  |  |  |  |  |  |  |  |  |  |  |  |  |  |  |  |  |  |  |  |  |  |  |  |  |  |  |  |  |  |  |  |  |  |  |  |  |  |  |  |  |  |  |  |  |  |  |  |  |  |  |  |  |  |  |  |  |  |  |  |  |  |  |  |  |  |  |  |  |  |  |  |  |  |  |  |  |  |  |  |  |  |  |  |  |  |  |  |  |  |  |  |  |  |  |  |  |  |  |  |  |  |  |  |  |  |  |  |  |  |  |  |  |  |  |  |  |  |  |  |  |  |  |  |  |  |  |  |  |  |  |  |  |  |  |  |  |  |  |  |  |  |  |  |  |  |  |  |  |  |  |  |  |  |  |  |  |  |  |  |  |  |  |  |  |  |  |  |  |  |  |  |  |  |  |  |  |  |  |  |  |  |  |  |  |  |  |  |  |  |  |  |  |  |  |  |  |  |  |  |  |  |  |  |  |  |  |  |  |  |  |  |  |  |  |  |  |  |  |  |  |  |  |  |  |  |  |  |  |  |  |  |  |  |  |  |  |  |  |  |  |  |  |  |  |  |  |  |  |  |  |  |  |  |  |  |  |  |  |  |  |  |  |  |  |  |  |  |  |  |  |  |  |  |  |  |  |  |  |  |  |  |  |  |  |  |  |  |  |  |  |  |  |  |  |  |  |  |  |  |  |  |  |  |  |  |  |  |  |  |  |  |  |  |  |  |  |  |  |  |  |  |  |  |  |  |  |  |  |  |  |  |  |  |  |  |  |  |  |  |  |  |  |  |  |  |  |  |  |  |  |  |  |  |  |  |  |  |  |  |  |  |  |  |  |  |  |  |  |  |  |  |  |  |  |  |  |  |  |  |  |  |  |  |  |  |  |  |  |  |  |  |  |  |  |  |  |  |  |  |  |  |  |  |  |  |  |  |  |  |  |  |  |  |  |  |  |  |  |  |  |  |  |  |  |  |  |  |  |  |  |  |  |  |  |  |  |  |  |  |  |  |  |  |  |  |  |  |  |  |  |  |  |  |  |  |  |  |  |  |  |  |  |  |  |  |  |  |  |  |  |  |  |  |  |  |  |  |  |  |  |  |  |  |  |  |  |  |  |  |  |  |  |  |  |  |  |  |  |  |  |  |  |  |  |  |  |  |  |  |  |  |  |  |  |  |  |  |  |  |  |  |  |  |  |  |  |  |  |  |  |  |  |  |  |  |  |  |  |  |  |  |  |  |  |  |  |  |  |  |  |  |  |  |  |  |  |  |  |  |  |  |  |  |  |  |  |  |  |  |  |  |  |  |  |  |  |  |  |  |  |  |  |  |  |  |  |  |  |  |  |  |  |  |  |  |  |  |  |  |  |  |  |  |  |  |  |  |  |  |  |  |  |  |  |  |  |  |  |  |  |  |  |  |  |  |  |  |  |  |  |  |  |  |  |  |  |  |  |  |  |  |  |  |  |  |  |  |  |  |  |  |  |  |  |  |  |  |  |  |  |  |  |  |  |  |  |  |  |  |  |  |  |  |  |  |  |  |  |  |  |  |  |  |  |  |  |  |  |  |  |  |  |  |  |  |  |  |  |  |  |  |  |  |  |  |  |  |  |  |  |  |  |  |  |  |  |  |  |  |  |  |  |  |  |  |  |  |  |  |  |  |  |  |  |  |  |  |  |  |  |  |  |  |  |  |  |  |  |  |  |  |  |  |  |  |  |  |  |  |  |  |  |  |  |  |  |  |  |  |  |  |  |  |  |  |  |  |  |  |  |  |  |  |  |  |  |  |  |  |  |  |  |  |  |  |  |  |  |  |  |  |  |  |  |  |  |  |  |  |  |  |  |  |  |  |  |  |  |  |  |  |  |  |  |  |  |  |  |  |  |  |  |  |  |  |  |  |  |  |  |  |  |  |  |  |  |  |  |  |  |  |  |  |  |  |  |  |  |  |  |  |  |  |  |  |  |  |  |  |  |  |  |  |  |  |  |  |  |  |  |  |  |  |  |  |  |  |  |  |  |  |  |  |  |  |  |  |  |  |  |  |  |  |  |  |  |  |  |  |  |  |  |  |  |  |  |  |  |  |  |  |  |  |  |  |  |  |  |  |  |  |  |  |  |  |  |  |  |  |  |  |  |  |  |  |  |  |  |  |  |  |  |  |  |  |  |  |  |  |  |  |  |  |  |  |  |  |  |  |  |  |  |  |  |  |  |  |  |  |  |  |  |  |  |  |  |  |  |  |  |  |  |  |  |  |  |  |  |  |  |  |  |  |  |  |  |  |  |  |  |  |  |  |  |  |  |  |  |  |  |  |  |  |  |  |  |  |  |  |  |  |  |  |  |  |  |  |  |  |  |  |  |  |  |  |  |  |  |  |  |  |  |  |  |  |  |  |  |  |  |  |  |  |  |  |  |  |  |  |  |  |  |  |  |  |  |  |  |  |  |  |  |  |  |  |  |  |  |  |  |  |  |  |  |  |  |  |  |  |  |  |  |  |  |  |  |  |  |  |  |  |  |  |  |  |  |  |  |  |  |  |  |  |  |  |  |  |  |  |  |  |  |  |  |  |  |  |  |  |  |  |  |  |  |  |  |  |  |  |  |  |  |  |  |  |  |  |  |  |  |  |  |  |  |  |  |  |  |  |  |  |  |  |  |  |  |  |  |  |  |  |  |  |  |  |  |  |  |  |  |  |  |  |  |  |  |  |  |  |  |  |  |  |  |  |  |  |  |  |  |  |  |  |  |  |  |  |  |  |  |  |  |  |  |  |  |  |  |  |  |  |  |  |  |  |  |  |  |  |  |  |  |  |  |  |  |  |  |  |  |  |  |  |  |  |  |  |  |  |  |  |  |  |  |  |  |  |  |  |  |  |  |  |  |  |  |  |  |  |  |  |  |  |  |  |  |  |  |  |  |  |  |  |  |  |  |  |  |  |  |  |  |  |  |  |  |  |  |  |  |  |  |  |  |  |  |  |  |  |  |  |  |  |  |  |  |  |  |  |  |  |  |  |  |  |  |  |  |  |  |  |  |  |  |  |  |  |  |  |  |  |  |  |  |  |  |  |  |  |  |  |  |  |  |  |  |  |  |  |  |  |  |  |  |  |  |  |  |  |  |  |  |  |  |  |  |  |  |  |  |  |  |  |  |  |  |  |  |  |  |  |  |  |  |  |  |  |  |  |  |  |  |  |  |  |  |  |  |  |  |  |  |  |  |  |  |  |  |  |  |  |  |  |  |  |  |  |  |  |  |  |  |  |  |  |  |  |  |  |  |  |  |  |  |  |  |  |  |  |  |  |  |  |  |  |  |  |  |  |  |  |  |  |  |  |  |  |  |  |  |  |  |  |  |  |  |  |  |  |  |  |  |  |  |  |  |  |  |  |  |  |  |  |  |  |  |  |  |  |  |  |  |  |  |  |  |  |  |  |  |  |  |  |  |  |  |  |  |  |  |  |  |  |  |  |  |  |  |  |  |  |  |  |  |  |  |  |  |  |  |  |  |  |  |  |  |  |  |  |  |  |  |  |  |  |  |  |  |  |  |  |  |  |  |  |  |  |  |  |  |  |  |  |  |  |  |  |  |  |  |  |  |  |  |  |  |  |  |  |  |  |  |  |  |  |  |  |  |  |  |  |  |  |  |  |  |  |  |  |  |  |  |  |  |  |  |  |  |  |  |  |  |  |  |  |  |  |  |  |  |  |  |  |  |  |  |  |  |  |  |  |  |  |  |  |  |  |  |  |  |  |  |  |  |  |  |  |  |  |  |  |  |  |  |  |  |  |  |  |  |  |  |  |  |  |  |  |  |  |  |  |  |  |  |  |  |  |  |  |  |  |  |  |  |  |  |  |  |  |  |  |  |  |  |  |  |  |  |  |  |  |  |  |  |  |  |  |  |  |  |  |  |  |  |  |  |  |  |  |  |  |  |  |  |  |  |  |  |  |  |  |  |  |  |  |  |  |  |  |  |  |  |  |  |  |  |  |  |  |  |  |  |  |  |  |  |  |  |  |  |  |  |  |  |  |  |  |  |  |  |  |  |  |  |  |  |  |  |  |  |  |  |  |  |  |  |  |  |  |  |  |  |  |  |  |  |  |  |  |  |  |  |  |  |  |  |  |  |  |  |  |  |  |  |  |  |  |  |  |  |  |  |  |  |  |  |  |  |  |  |  |  |  |  |  |  |  |  |  |  |  |  |  |  |  |  |  |  |  |  |  |  |  |  |  |  |  |  |  |  |  |  |  |  |  |  |  |  |  |  |  |  |  |  |  |  |  |  |  |  |  |  |  |  |  |  |  |  |  |  |  |  |  |  |  |  |  |  |  |  |  |  |  |  |  |  |  |  |  |  |  |  |  |  |  |  |  |  |  |  |  |  |  |  |  |  |  |  |  |  |  |  |  |  |  |  |  |  |  |  |  |  |  |  |  |  |  |  |  |  |  |  |  |  |  |  |  |  |  |  |  |  |  |  |  |  |  |  |  |  |  |  |  |  |  |  |  |  |  |  |  |  |  |  |  |  |  |  |  |  |  |  |  |  |  |  |  |  |  |  |  |  |  |  |  |  |  |  |  |  |  |  |  |  |  |  |  |  |  |  |  |  |  |  |  |  |  |  |  |  |  |  |  |  |  |  |  |  |  |  |  |  |  |  |  |  |  |  |  |  |  |  |  |  |  |  |  |  |  |  |  |  |  |  |  |  |  |  |  |  |  |  |  |  |  |  |  |  |  |  |  |  |  |  |  |  |  |  |  |  |  |  |  |  |  |  |  |  |  |  |  |  |  |  |  |  |  |  |  |  |  |  |  |  |  |  |  |  |  |  |  |  |  |  |  |  |  |  |  |  |  |  |  |  |  |  |  |  |  |  |  |  |  |  |  |  |  |  |  |  |  |  |  |  |  |  |  |  |  |  |  |  |  |  |  |  |  |  |  |  |  |  |  |  |  |  |  |  |  |  |  |  |  |  |  |  |  |  |  |  |  |  |  |  |  |  |  |  |  |  |  |  |  |  |  |  |  |  |  |  |  |  |  |  |  |  |  |  |  |  |  |  |  |  |  |  |  |  |  |  |  |  |  |  |  |  |  |  |  |  |  |  |  |  |  |  |  |  |  |  |  |  |  |  |  |  |  |  |  |  |  |  |  |  |  |  |  |  |  |  |  |  |  |  |  |  |  |  |  |  |  |  |  |  |  |  |  |  |  |  |  |  |  |  |  |  |  |  |  |  |  |  |  |  |  |  |  |  |  |  |  |  |  |  |  |  |  |  |  |  |  |  |  |  |  |  |  |  |  |  |  |  |  |  |  |  |  |  |  |  |  |  |  |  |  |  |  |  |  |  |  |  |  |  |  |  |  |  |  |  |  |  |  |  |  |  |  |  |  |  |  |  |  |  |  |  |  |  |  |  |  |  |  |  |  |  |  |  |  |  |  |  |  |  |  |  |  |  |  |  |  |  |  |  |  |  |  |  |  |  |  |  |  |  |  |  |  |  |  |  |  |  |  |  |  |  |  |  |  |  |  |  |  |  |  |  |  |  |  |  |  |  |  |  |  |  |  |  |  |  |  |  |  |  |  |  |  |  |  |  |  |  |  |  |  |  |  |  |  |  |  |  |  |  |  |  |  |  |  |  |  |  |  |  |  |  |  |  |  |  |  |  |  |  |  |  |  |  |  |  |  |  |  |  |  |  |  |  |  |  |  |  |  |  |  |  |  |  |  |  |  |  |  |  |  |  |  |  |  |  |  |  |  |  |  |  |  |  |  |  |  |  |  |  |  |  |  |  |  |  |  |  |  |  |  |  |  |  |  |  |  |  |  |  |  |  |  |  |  |  |  |  |  |  |  |  |  |  |  |  |  |  |  |  |  |  |  |  |  |  |  |  |  |  |  |  |  |  |  |  |  |  |  |  |  |  |  |  |  |  |  |  |  |  |  |  |  |  |  |  |  |  |  |  |  |  |  |  |  |  |  |  |  |  |  |  |  |  |  |  |  |  |  |  |  |  |  |  |  |  |  |  |  |  |  |  |  |  |  |  |  |  |  |  |  |  |  |  |  |  |  |  |  |  |  |  |  |  |  |  |  |  |  |  |  |  |  |  |  |  |  |  |  |  |  |  |  |  |  |  |  |  |  |  |  |  |  |  |  |  |  |  |  |  |  |  |  |  |  |  |  |  |  |  |  |  |  |  |  |  |  |  |  |  |  |  |  |  |  |  |  |  |  |  |  |  |  |  |  |  |  |  |  |  |  |  |  |  |  |  |  |  |  |  |  |  |  |  |  |  |  |  |  |  |  |  |  |  |  |  |  |  |  |  |  |  |  |  |  |  |  |  |  |  |  |  |  |  |  |  |  |  |  |  |  |  |  |  |  |  |  |  |  |  |  |  |  |  |  |  |  |  |  |  |  |  |  |  |  |  |  |  |  |  |  |  |  |  |  |  |  |  |  |  |  |  |  |  |  |  |  |  |  |  |  |  |  |  |  |  |  |  |  |  |  |  |  |  |  |  |  |  |  |  |  |  |  |  |  |  |  |  |  |  |  |  |  |  |  |  |  |  |  |  |  |  |  |  |  |  |  |  |  |  |  |  |  |  |  |  |  |  |  |  |  |  |  |  |  |  |  |  |  |  |  |  |  |  |  |  |  |  |  |  |  |  |  |  |  |  |  |  |  |  |  |  |  |  |  |  |  |  |  |  |  |  |  |  |  |  |  |  |  |  |  |  |  |  |  |  |  |  |  |  |  |  |  |  |  |  |  |  |  |  |  |  |  |  |  |  |  |  |  |  |  |  |  |  |  |  |  |  |  |  |  |  |  |  |  |  |  |  |  |  |  |  |  |  |  |  |  |  |  |  |  |  |  |  |  |  |  |  |  |  |  |  |  |  |  |  |  |  |  |  |  |  |  |  |  |  |  |  |  |  |  |  |  |  |  |  |  |  |  |  |  |  |  |  |  |  |  |  |  |  |  |  |  |  |  |  |  |  |  |  |  |  |  |  |  |  |  |  |  |  |  |  |  |  |  |  |  |  |  |  |  |  |  |  |  |  |  |  |  |  |  |  |  |  |  |  |  |  |  |  |  |  |  |  |  |  |  |  |  |  |  |  |  |  |  |  |  |  |  |  |  |  |  |  |  |  |  |  |  |  |  |  |  |  |  |  |  |  |  |  |  |  |  |  |  |  |  |  |  |  |  |  |  |  |  |  |  |  |  |  |  |  |  |  |  |  |  |  |  |  |  |  |  |  |  |  |  |  |  |  |  |  |  |  |  |  |  |  |  |  |  |  |  |  |  |  |  |  |  |  |  |  |  |  |  |  |  |  |  |  |  |  |  |  |  |  |  |  |  |  |  |  |  |  |  |  |  |  |  |  |  |  |  |  |  |  |  |  |  |  |  |  |  |  |  |  |  |  |  |  |  |  |  |  |  |  |  |  |  |  |  |  |  |  |  |  |  |  |  |  |  |  |  |  |  |  |  |  |  |  |  |  |  |  |  |  |  |  |  |  |  |  |  |  |  |  |  |  |  |  |  |  |  |  |  |  |  |  |  |  |  |  |  |  |  |  |  |  |  |  |  |  |  |  |  |  |  |  |  |  |  |  |  |  |  |  |  |  |  |  |  |  |  |  |  |  |  |  |  |  |  |  |  |  |  |  |  |  |  |  |  |  |  |  |  |  |  |  |  |  |  |  |  |  |  |  |  |  |  |  |  |  |  |  |  |  |  |  |  |  |  |  |  |  |  |  |  |  |  |  |  |  |  |  |  |  |  |  |  |  |  |  |  |  |  |  |  |  |  |  |  |  |  |  |  |  |  |  |  |  |  |  |  |  |  |  |  |  |  |  |  |  |  |  |  |  |  |  |  |  |  |  |  |  |  |  |  |  |  |  |  |  |  |  |  |  |  |  |  |  |  |  |  |  |  |  |  |  |  |  |  |  |  |  |  |  |  |  |  |  |  |  |  |  |  |  |  |  |  |  |  |  |  |  |  |  |  |  |  |  |  |  |  |  |  |  |  |  |  |  |  |  |  |  |  |  |  |  |  |  |  |  |  |  |  |  |  |  |  |  |  |  |  |  |  |  |  |  |  |  |  |  |  |  |  |  |  |  |  |  |  |  |  |  |  |  |  |  |  |  |  |  |  |  |  |  |  |  |  |  |  |  |  |  |  |  |  |  |  |  |  |  |  |  |  |  |  |  |  |  |  |  |  |  |  |  |  |  |  |  |  |  |  |  |  |  |  |  |  |  |  |  |  |  |  |  |  |  |  |  |  |  |  |  |  |  |  |  |  |  |  |  |  |  |  |  |  |  |  |  |  |  |  |  |  |  |  |  |  |  |  |  |  |  |  |  |  |  |  |  |  |  |  |  |  |  |  |  |  |  |  |  |  |  |  |  |  |  |  |  |  |  |  |  |  |  |  |  |  |  |  |  |  |  |  |  |  |  |  |  |  |  |  |  |  |  |  |  |  |  |  |  |  |  |  |  |  |  |  |  |  |  |  |  |  |  |  |  |  |  |  |  |  |  |  |  |  |  |  |  |  |  |  |  |  |  |  |  |  |  |  |  |  |  |  |  |  |  |  |  |  |  |  |  |  |  |  |  |  |  |  |  |  |  |  |  |  |  |  |  |  |  |  |  |  |  |  |  |  |  |  |  |  |  |  |  |  |  |  |  |  |  |  |  |  |  |  |  |  |  |  |  |  |  |  |  |  |  |  |  |  |  |  |  |  |  |  |  |  |  |  |  |  |  |  |  |  |  |  |  |  |  |  |  |  |  |  |  |  |  |  |  |  |  |  |  |  |  |  |  |  |  |  |  |  |  |  |  |  |  |  |  |  |  |  |  |  |  |  |  |  |  |  |  |  |  |  |  |  |  |  |  |  |  |  |  |  |  |  |  |  |  |  |  |  |  |  |  |  |  |  |  |  |  |  |  |  |  |  |  |  |  |  |  |  |  |  |  |  |  |  |  |  |  |  |  |  |  |  |  |  |  |  |  |  |  |  |  |  |  |  |  |  |  |  |  |  |  |  |  |  |  |  |  |  |  |  |  |  |  |  |  |  |  |  |  |  |  |  |  |  |  |  |  |  |  |  |  |  |  |  |  |  |  |  |  |  |  |  |  |  |  |  |  |  |  |  |  |  |  |  |  |  |  |  |  |  |  |  |  |  |  |  |  |  |  |  |  |  |  |  |  |  |  |  |  |  |  |  |  |  |  |  |  |  |  |  |  |  |  |  |  |  |  |  |  |  |  |  |  |  |  |  |  |  |  |  |  |  |  |  |  |  |  |  |  |  |  |  |  |  |  |  |  |  |  |  |  |  |  |  |  |  |  |  |  |  |  |  |  |  |  |  |  |  |  |  |  |  |  |  |  |  |  |  |  |  |  |  |  |  |  |  |  |  |  |  |  |  |  |  |  |  |  |  |  |  |  |  |  |  |  |  |  |  |  |  |  |  |  |  |  |  |  |  |  |  |  |  |  |  |  |  |  |  |  |  |  |  |  |  |  |  |  |  |  |  |  |  |  |  |  |  |  |  |  |  |  |  |  |  |  |  |  |  |  |  |  |  |  |  |  |  |  |  |  |  |  |  |  |  |  |  |  |  |  |  |  |  |  |  |  |  |  |  |  |  |  |  |  |  |  |  |  |  |  |  |  |  |  |  |  |  |  |  |  |  |  |  |  |  |  |  |  |  |  |  |  |  |  |  |  |  |  |  |  |  |  |  |  |  |  |  |  |  |  |  |  |  |  |  |  |  |  |  |  |  |  |  |  |  |  |  |  |  |  |  |  |  |  |  |  |  |  |  |  |  |  |  |  |  |  |  |  |  |  |  |  |  |  |  |  |  |  |  |  |  |  |  |  |  |  |  |  |  |  |  |  |  |  |  |  |  |  |  |  |  |  |  |  |  |  |  |  |  |  |  |  |  |  |  |  |  |  |  |  |  |  |  |  |  |  |  |  |  |  |  |  |  |  |  |  |  |  |  |  |  |  |  |  |  |  |  |  |  |  |  |  |  |  |  |  |  |  |  |  |  |  |  |  |  |  |  |  |  |  |  |  |  |  |  |  |  |  |  |  |  |  |  |  |  |  |  |  |  |  |  |  |  |  |  |  |  |  |  |  |  |  |  |  |  |  |  |  |  |  |  |  |  |  |  |  |  |  |  |  |  |  |  |  |  |  |  |  |  |  |  |  |  |  |  |  |  |  |  |  |  |  |  |  |  |  |  |  |  |  |  |  |  |  |  |  |  |  |  |  |  |  |  |  |  |  |  |  |  |  |  |  |  |  |  |  |  |  |  |  |  |  |  |  |  |  |  |  |  |  |  |  |  |  |  |  |  |  |  |  |  |  |  |  |  |  |  |  |  |  |  |  |  |  |  |  |  |  |  |  |  |  |  |  |  |  |  |  |  |  |  |  |  |  |  |  |  |  |  |  |  |  |  |  |  |  |  |  |  |  |  |  |  |  |  |  |  |  |  |  |  |  |  |  |  |  |  |  |  |  |  |  |  |  |  |  |  |  |  |  |  |  |  |  |  |  |  |  |  |  |  |  |  |  |  |  |  |  |  |  |  |  |  |  |  |  |  |  |  |  |  |  |  |  |  |  |  |  |  |  |  |  |  |  |  |  |  |  |  |  |  |  |  |  |  |  |  |  |  |  |  |  |  |  |  |  |  |  |  |  |  |  |  |  |  |  |  |  |  |  |  |  |  |  |  |  |  |  |  |  |  |  |  |  |  |  |  |  |  |  |  |  |  |  |  |  |  |  |  |  |  |  |  |  |  |  |  |  |  |  |  |  |  |  |  |  |  |  |  |  |  |  |  |  |  |  |  |  |  |  |  |  |  |  |  |  |  |  |  |  |  |  |  |  |  |  |  |  |  |  |  |  |  |  |  |  |  |  |  |  |  |  |  |  |  |  |  |  |  |  |  |  |  |  |  |  |  |  |  |  |  |  |  |  |  |  |  |  |  |  |  |  |  |  |  |  |  |  |  |  |  |  |  |  |  |  |  |  |  |  |  |  |  |  |  |  |  |  |  |  |  |  |  |  |  |  |  |  |  |  |  |  |  |  |  |  |  |  |  |  |  |  |  |  |  |  |  |  |  |  |  |  |  |  |  |  |  |  |  |  |  |  |  |  |  |  |  |  |  |  |  |  |  |  |  |  |  |  |  |  |  |  |  |  |  |  |  |  |  |  |  |  |  |  |  |  |  |  |  |  |  |  |  |  |  |  |  |  |  |  |  |  |  |  |  |  |  |  |  |  |  |  |  |  |  |  |  |  |  |  |  |  |  |  |  |  |  |  |  |  |  |  |  |  |  |  |  |  |  |  |  |  |  |  |  |  |  |  |  |  |  |  |  |  |  |  |  |  |  |  |  |  |  |  |  |  |  |  |  |  |  |  |  |  |  |  |  |  |  |  |  |  |  |  |  |  |  |  |  |  |  |  |  |  |  |  |  |  |  |  |  |  |  |  |  |  |  |  |  |  |  |  |  |  |  |  |  |  |  |  |  |  |  |  |  |  |  |  |  |  |  |  |  |  |  |  |  |  |  |  |  |  |  |  |  |  |  |  |  |  |  |  |  |  |  |  |  |  |  |  |  |  |  |  |  |  |  |  |  |  |  |  |  |  |  |  |  |  |  |  |  |  |  |  |  |  |  |  |  |  |  |  |  |  |  |  |  |  |  |  |  |  |  |  |  |  |  |  |  |  |  |  |  |  |  |  |  |  |  |  |  |  |  |  |  |  |  |  |  |  |  |  |  |  |  |  |  |  |  |  |  |  |  |  |  |  |  |  |  |  |  |  |  |  |  |  |  |  |  |  |  |  |  |  |  |  |  |  |  |  |  |  |  |  |  |  |  |  |  |  |  |  |  |  |  |  |  |  |  |  |  |  |  |  |  |  |  |  |  |  |  |  |  |  |  |  |  |  |  |  |  |  |  |  |  |  |  |  |  |  |  |  |  |  |  |  |  |  |  |  |  |  |  |  |  |  |  |  |  |  |  |  |  |  |  |  |  |  |  |  |  |  |  |  |  |  |  |  |  |  |  |  |  |  |  |  |  |  |  |  |  |  |  |  |  |  |  |  |  |  |  |  |  |  |  |  |  |  |  |  |  |  |  |  |  |  |  |  |  |  |  |  |  |  |  |  |  |  |  |  |  |  |  |  |  |  |  |  |  |  |  |  |  |  |  |  |  |  |  |  |  |  |  |  |  |  |  |  |  |  |  |  |  |  |  |  |  |  |  |  |  |  |  |  |  |  |  |  |  |  |  |  |  |  |  |  |  |  |  |  |  |  |  |  |  |  |  |  |  |  |  |  |  |  |  |  |  |  |  |  |  |  |  |  |  |  |  |  |  |  |  |  |  |  |  |  |  |  |  |  |  |  |  |  |  |  |  |  |  |  |  |  |  |  |  |  |  |  |  |  |  |  |  |  |  |  |  |  |  |  |  |  |  |  |  |  |  |  |  |  |  |  |  |  |  |  |  |  |  |  |  |  |  |  |  |  |  |  |  |  |  |  |  |  |  |  |  |  |  |  |  |  |  |  |  |  |  |  |  |  |  |  |  |  |  |  |  |  |  |  |  |  |  |  |  |  |  |  |  |  |  |  |  |  |  |  |  |  |  |  |  |  |  |  |  |  |  |  |  |  |  |  |  |  |  |  |  |  |  |  |  |  |  |  |  |  |  |  |  |  |  |  |  |  |  |  |  |  |  |  |  |  |  |  |  |  |  |  |  |  |  |  |  |  |  |  |  |  |  |  |  |  |  |  |  |  |  |  |  |  |  |  |  |  |  |  |  |  |  |  |  |  |  |  |  |  |  |  |  |  |  |  |  |  |  |  |  |  |  |  |  |  |  |  |  |  |  |  |  |  |  |  |  |  |  |  |  |  |  |  |  |  |  |  |  |  |  |  |  |  |  |  |  |  |  |  |  |  |  |  |  |  |  |  |  |  |  |  |  |  |  |  |  |  |  |  |  |  |  |  |  |  |  |  |  |  |  |  |  |  |  |  |  |  |  |  |  |  |  |  |  |  |  |  |  |  |  |  |  |  |  |  |  |  |  |  |  |  |  |  |  |  |  |  |  |  |  |  |  |  |  |  |  |  |  |  |  |  |  |  |  |  |  |  |  |  |  |  |  |  |  |  |  |  |  |  |  |  |  |  |  |  |  |  |  |  |  |  |  |  |  |  |  |  |  |  |  |  |  |  |  |  |  |  |  |  |  |  |  |  |  |  |  |  |  |  |  |  |  |  |  |  |  |  |  |  |  |  |  |  |  |  |  |  |  |  |  |  |  |  |  |  |  |  |  |  |  |  |  |  |  |  |  |  |  |  |  |  |  |  |  |  |  |  |  |  |  |  |  |  |  |  |  |  |  |  |  |  |  |  |  |  |  |  |  |  |  |  |  |  |  |  |  |  |  |  |  |  |  |  |  |  |  |  |  |  |  |  |  |  |  |  |  |  |  |  |  |  |  |  |  |  |  |  |  |  |  |  |  |  |  |  |  |  |  |  |  |  |  |  |  |  |  |  |  |  |  |  |  |  |  |  |  |  |  |  |  |  |  |  |  |  |  |  |  |  |  |  |  |  |  |  |  |  |  |  |  |  |  |  |  |  |  |  |  |  |  |  |  |  |  |  |  |  |  |  |  |  |  |  |  |  |  |  |  |  |  |  |  |  |  |  |  |  |  |  |  |  |  |  |  |  |  |  |  |  |  |  |  |  |  |  |  |  |  |  |  |  |  |  |  |  |  |  |  |  |  |  |  |  |  |  |  |  |  |  |  |  |  |  |  |  |  |  |  |  |  |  |  |  |  |  |  |  |  |  |  |  |  |  |  |  |  |  |  |  |  |  |  |  |  |  |  |  |  |  |  |  |  |  |  |  |  |  |  |  |  |  |  |  |  |  |  |  |  |  |  |  |  |  |  |  |  |  |  |  |  |  |  |  |  |  |  |  |  |  |  |  |  |  |  |  |  |  |  |  |  |  |  |  |  |  |  |  |  |  |  |  |  |  |  |  |  |  |  |  |  |  |  |  |  |  |  |  |  |  |  |  |  |  |  |  |  |  |  |  |  |  |  |  |  |  |  |  |  |  |  |  |  |  |  |  |  |  |  |  |  |  |  |  |  |  |  |  |  |  |  |  |  |  |  |  |  |  |  |  |  |  |  |  |  |  |  |  |  |  |  |  |  |  |  |  |  |  |  |  |  |  |  |  |  |  |  |  |  |  |  |  |  |  |  |  |  |  |  |  |  |  |  |  |  |  |  |  |  |  |  |  |  |  |  |  |  |  |  |  |  |  |  |  |  |  |  |  |  |  |  |  |  |  |  |  |  |  |  |  |  |  |  |  |  |  |  |  |  |  |  |  |  |  |  |  |  |  |  |  |  |  |  |  |  |  |  |  |  |  |  |  |  |  |  |  |  |  |  |  |  |  |  |  |  |  |  |  |  |  |  |  |  |  |  |  |  |  |  |  |  |  |  |  |  |  |  |  |  |  |  |  |  |  |  |  |  |  |  |  |  |  |  |  |  |  |  |  |  |  |  |  |  |  |  |  |  |  |  |  |  |  |  |  |  |  |  |  |  |  |  |  |  |  |  |  |  |  |  |  |  |  |  |  |  |  |  |  |  |  |  |  |  |  |  |  |  |  |  |  |  |  |  |  |  |  |  |  |  |  |  |  |  |  |  |  |  |  |  |  |  |  |  |  |  |  |  |  |  |  |  |  |  |  |  |  |  |  |  |  |  |  |  |  |  |  |  |  |  |  |  |  |  |  |  |  |  |  |  |  |  |  |  |  |  |  |  |  |  |  |  |  |  |  |  |  |  |  |  |  |  |  |  |  |  |  |  |  |  |  |  |  |  |  |  |  |  |  |  |  |  |  |  |  |  |  |  |  |  |  |  |  |  |  |  |  |  |  |  |  |  |  |  |  |  |  |  |  |  |  |  |  |  |  |  |  |  |  |  |  |  |  |  |  |  |  |  |  |  |  |  |  |  |  |  |  |  |  |  |  |  |  |  |  |  |  |  |  |  |  |  |  |  |  |  |  |  |  |  |  |  |  |  |  |  |  |  |  |  |  |  |  |  |  |  |  |  |  |  |  |  |  |  |  |  |  |  |  |  |  |  |  |  |  |  |  |  |  |  |  |  |  |  |  |  |  |  |  |  |  |  |  |  |  |  |  |  |  |  |  |  |  |  |  |  |  |  |  |  |  |  |  |  |  |  |  |  |  |  |  |  |  |  |  |  |  |  |  |  |  |  |  |  |  |  |  |  |  |  |  |  |  |  |  |  |  |  |  |  |  |  |  |  |  |  |  |  |  |  |  |  |  |  |  |  |  |  |  |  |  |  |  |  |  |  |  |  |  |  |  |  |  |  |  |  |  |  |  |  |  |  |  |  |  |  |  |  |  |  |  |  |  |  |  |  |  |  |  |  |  |  |  |  |  |  |  |  |  |  |  |  |  |  |  |  |  |  |  |  |  |  |  |  |  |  |  |  |  |  |  |  |  |  |  |  |  |  |  |  |  |  |  |  |  |  |  |  |  |  |  |  |  |  |  |  |  |  |  |  |  |  |  |  |  |  |  |  |  |  |  |  |  |  |  |  |  |  |  |  |  |  |  |  |  |  |  |  |  |  |  |  |  |  |  |  |  |  |  |  |  |  |  |  |  |  |  |  |  |  |  |  |  |  |  |  |  |  |  |  |  |  |  |  |  |  |  |  |  |  |  |  |  |  |  |  |  |  |  |  |  |  |  |  |  |  |  |  |  |  |  |  |  |  |  |  |  |  |  |  |  |  |  |  |  |  |  |  |  |  |  |  |  |  |  |  |  |  |  |  |  |  |  |  |  |  |  |  |  |  |  |  |  |  |  |  |  |  |  |  |  |  |  |  |  |  |  |  |  |  |  |  |  |  |  |  |  |  |  |  |  |  |  |  |  |  |  |  |  |  |  |  |  |  |  |  |  |  |  |  |  |  |  |  |  |  |  |  |  |  |  |  |  |  |  |  |  |  |  |  |  |  |  |  |  |  |  |  |  |  |  |  |  |  |  |  |  |  |  |  |  |  |  |  |  |  |  |  |  |  |  |  |  |  |  |  |  |  |  |  |  |  |  |  |  |  |  |  |  |  |  |  |  |  |  |  |  |  |  |  |  |  |  |  |  |  |  |  |  |  |  |  |  |  |  |  |  |  |  |  |  |  |  |  |  |  |  |  |  |  |  |  |  |  |  |  |  |  |  |  |  |  |  |  |  |  |  |  |  |  |  |  |  |  |  |  |  |  |  |  |  |  |  |  |  |  |  |  |  |  |  |  |  |  |  |  |  |  |  |  |  |  |  |  |  |  |  |  |  |  |  |  |  |  |  |  |  |  |  |  |  |  |  |  |  |  |  |  |  |  |  |  |  |  |  |  |  |  |  |  |  |  |  |  |  |  |  |  |  |  |  |  |  |  |  |  |  |  |  |  |  |  |  |  |  |  |  |  |  |  |  |  |  |  |  |  |  |  |  |  |  |  |  |  |  |  |  |  |  |  |  |  |  |  |  |  |  |  |  |  |  |  |  |  |  |  |  |  |  |  |  |  |  |  |  |  |  |  |  |  |  |  |  |  |  |  |  |  |  |  |  |  |  |  |  |  |  |  |  |  |  |  |  |  |  |  |  |  |  |  |  |  |  |  |  |  |  |  |  |  |  |  |  |  |  |  |  |  |  |  |  |  |  |  |  |  |  |  |  |  |  |  |  |  |  |  |  |  |  |  |  |  |  |  |  |  |  |  |  |  |  |  |  |  |  |  |  |  |  |  |  |  |  |  |  |  |  |  |  |  |  |  |  |  |  |  |  |  |  |  |  |  |  |  |  |  |  |  |  |  |  |  |  |  |  |  |  |  |  |  |  |  |  |  |  |  |  |  |  |  |  |  |  |  |  |  |  |  |  |  |  |  |  |  |  |  |  |  |  |  |  |  |  |  |  |  |  |  |  |  |  |  |  |  |  |  |  |  |  |  |  |  |  |  |  |  |  |  |  |  |  |  |  |  |  |  |  |  |  |  |  |  |  |  |  |  |  |  |  |  |  |  |  |  |  |  |  |  |  |  |  |  |  |  |  |  |  |  |  |  |  |  |  |  |  |  |  |  |  |  |  |  |  |  |  |  |  |  |  |  |  |  |  |  |  |  |  |  |  |  |  |  |  |  |  |  |  |  |  |  |  |  |  |  |  |  |  |  |  |  |  |  |  |  |  |  |  |  |  |  |  |  |  |  |  |  |  |  |  |  |  |  |  |  |  |  |  |  |  |  |  |  |  |  |  |  |  |  |  |  |  |  |  |  |  |  |  |  |  |  |  |  |  |  |  |  |  |  |  |  |  |  |  |  |  |  |  |  |  |  |  |  |  |  |  |  |  |  |  |  |  |  |  |  |  |  |  |  |  |  |  |  |  |  |  |  |  |  |  |  |  |  |  |  |  |  |  |  |  |  |  |  |  |  |  |  |  |  |  |  |  |  |  |  |  |  |  |  |  |  |  |  |  |  |  |  |  |  |  |  |  |  |  |  |  |  |  |  |  |  |  |  |  |  |  |  |  |  |  |  |  |  |  |  |  |  |  |  |  |  |  |  |  |  |  |  |  |  |  |  |  |  |  |  |  |  |  |  |  |  |  |  |  |  |  |  |  |  |  |  |  |  |  |  |  |  |  |  |  |  |  |  |  |  |  |  |  |  |  |  |  |  |  |  |  |  |  |  |  |  |  |  |  |  |  |  |  |  |  |  |  |  |  |  |  |  |  |  |  |  |  |  |  |  |  |  |  |  |  |  |  |  |  |  |  |  |  |  |  |  |  |  |  |  |  |  |  |  |  |  |  |  |  |  |  |  |  |  |  |  |  |  |  |  |  |  |  |  |  |  |  |  |  |  |  |  |  |  |  |  |  |  |  |  |  |  |  |  |  |  |  |  |  |  |  |  |  |  |  |  |  |  |  |  |  |  |  |  |  |  |  |  |  |  |  |  |  |  |  |  |  |  |  |  |  |  |  |  |  |  |  |  |  |  |  |  |  |  |  |  |  |  |  |  |  |  |  |  |  |  |  |  |  |  |  |  |  |  |  |  |  |  |  |  |  |  |  |  |  |  |  |  |  |  |  |  |  |  |  |  |  |  |  |  |  |  |  |  |  |  |  |  |  |  |  |  |  |  |  |  |  |  |  |  |  |  |  |  |  |  |  |  |  |  |  |  |  |  |  |  |  |  |  |  |  |  |  |  |  |  |  |  |  |  |  |  |  |  |  |  |  |  |  |  |  |  |  |  |  |  |  |  |  |  |  |  |  |  |  |  |  |  |  |  |  |  |  |  |  |  |  |  |  |  |  |  |  |  |  |  |  |  |  |  |  |  |  |  |  |  |  |  |  |  |  |  |  |  |  |  |  |  |  |  |  |  |  |  |  |  |  |  |  |  |  |  |  |  |  |  |  |  |  |  |  |  |  |  |  |  |  |  |  |  |  |  |  |  |  |  |  |  |  |  |  |  |  |  |  |  |  |  |  |  |  |  |  |  |  |  |  |  |  |  |  |  |  |  |  |  |  |  |  |  |  |  |  |  |  |  |  |  |  |  |  |  |  |  |  |  |  |  |  |  |  |  |  |  |  |  |  |  |  |  |  |  |  |  |  |  |  |  |  |  |  |  |  |  |  |  |  |  |  |  |  |  |  |  |  |  |  |  |  |  |  |  |  |  |  |  |  |  |  |  |  |  |  |  |  |  |  |  |  |  |  |  |  |  |  |  |  |  |  |  |  |  |  |  |  |  |  |  |  |  |  |  |  |  |  |  |  |  |  |  |  |  |  |  |  |  |  |  |  |  |  |  |  |  |  |  |  |  |  |  |  |  |  |  |  |  |  |  |  |  |  |  |  |  |  |  |  |  |  |  |  |  |  |  |  |  |  |  |  |  |  |  |  |  |  |  |  |  |  |  |  |  |  |  |  |  |  |  |  |  |  |  |  |  |  |  |  |  |  |  |  |  |  |  |  |  |  |  |  |  |  |  |  |  |  |  |  |  |  |  |  |  |  |  |  |  |  |  |  |  |  |  |  |  |  |  |  |  |  |  |  |  |  |  |  |  |  |  |  |  |  |  |  |  |  |  |  |  |  |  |  |  |  |  |  |  |  |  |  |  |  |  |  |  |  |  |  |  |  |  |  |  |  |  |  |  |  |  |  |  |  |  |  |  |  |  |  |  |  |  |  |  |  |  |  |  |  |  |  |  |  |  |  |  |  |  |  |  |  |  |  |  |  |  |  |  |  |  |  |  |  |  |  |  |  |  |  |  |  |  |  |  |  |  |  |  |  |  |  |  |  |  |  |  |  |  |  |  |  |  |  |  |  |  |  |  |  |  |  |  |  |  |  |  |  |  |  |  |  |  |  |  |  |  |  |  |  |  |  |  |  |  |  |  |  |  |  |  |  |  |  |  |  |  |  |  |  |  |  |  |  |  |  |  |  |  |  |  |  |  |  |  |  |  |  |  |  |  |  |  |  |  |  |  |  |  |  |  |  |  |  |  |  |  |  |  |  |  |  |  |  |  |  |  |  |  |  |  |  |  |  |  |  |  |  |  |  |  |  |  |  |  |  |  |  |  |  |  |  |  |  |  |  |  |  |  |  |  |  |  |  |  |  |  |  |  |  |  |  |  |  |  |  |  |  |  |  |  |  |  |  |  |  |  |  |  |  |  |  |  |  |  |  |  |  |  |  |  |  |  |  |  |  |  |  |  |  |  |  |  |  |  |  |  |  |  |  |  |  |  |  |  |  |  |  |  |  |  |  |  |  |  |  |  |  |  |  |  |  |  |  |  |  |  |  |  |  |  |  |  |  |  |  |  |  |  |  |  |  |  |  |  |  |  |  |  |  |  |  |  |  |  |  |  |  |  |  |  |  |  |  |  |  |  |  |  |  |  |  |  |  |  |  |  |  |  |  |  |  |  |  |  |  |  |  |  |  |  |  |  |  |  |  |  |  |  |  |  |  |  |  |  |  |  |  |  |  |  |  |  |  |  |  |  |  |  |  |  |  |  |  |  |  |  |  |  |  |  |  |  |  |  |  |  |  |  |  |  |  |  |  |  |  |  |  |  |  |  |  |  |  |  |  |  |  |  |  |  |  |  |  |  |  |  |  |  |  |  |  |  |  |  |  |  |  |  |  |  |  |  |  |  |  |  |  |  |  |  |  |  |  |  |  |  |  |  |  |  |  |  |  |  |  |  |  |  |  |  |  |  |  |  |  |  |  |  |  |  |  |  |  |  |  |  |  |  |  |  |  |  |  |  |  |  |  |  |  |  |  |  |  |  |  |  |  |  |  |  |  |  |  |  |  |  |  |  |  |  |  |  |  |  |  |  |  |  |  |  |  |  |  |  |  |  |  |  |  |  |  |  |  |  |  |  |  |  |  |  |  |  |  |  |  |  |  |  |  |  |  |  |  |  |  |  |  |  |  |  |  |  |  |  |  |  |  |  |  |  |  |  |  |  |  |  |  |  |  |  |  |  |  |  |  |  |  |  |  |  |  |  |  |  |  |  |  |  |  |  |  |  |  |  |  |  |  |  |  |  |  |  |  |  |  |  |  |  |  |  |  |  |  |  |  |  |  |  |  |  |  |  |  |  |  |  |  |  |  |  |  |  |  |  |  |  |  |  |  |  |  |  |  |  |  |  |  |  |  |  |  |  |  |  |  |  |  |  |  |  |  |  |  |  |  |  |  |  |  |  |  |  |  |  |  |  |  |  |  |  |  |  |  |  |  |  |  |  |  |  |  |  |  |  |  |  |  |  |  |  |  |  |  |  |  |  |  |  |  |  |  |  |  |  |  |  |  |  |  |  |  |  |  |  |  |  |  |  |  |  |  |  |  |  |  |  |  |  |  |  |  |  |  |  |  |  |  |  |  |  |  |  |  |  |  |  |  |  |  |  |  |  |  |  |  |  |  |  |  |  |  |  |  |  |  |  |  |  |  |  |  |  |  |  |  |  |  |  |  |  |  |  |  |  |  |  |  |  |  |  |  |  |  |  |  |  |  |  |  |  |  |  |  |  |  |  |  |  |  |  |  |  |  |  |  |  |  |  |  |  |  |  |  |  |  |  |  |  |  |  |  |  |  |  |  |  |  |  |  |  |  |  |  |  |  |  |  |  |  |  |  |  |  |  |  |  |  |  |  |  |  |  |  |  |  |  |  |  |  |  |  |  |  |  |  |  |  |  |  |  |  |  |  |  |  |  |  |  |  |  |  |  |  |  |  |  |  |  |  |  |  |  |  |  |  |  |  |  |  |  |  |  |  |  |  |  |  |  |  |  |  |  |  |  |  |  |  |  |  |  |  |  |  |  |  |  |  |  |  |  |  |  |  |  |  |  |  |  |  |  |  |  |  |  |  |  |  |  |  |  |  |  |  |  |  |  |  |  |  |  |  |  |  |  |  |  |  |  |  |  |  |  |  |  |  |  |  |  |  |  |  |  |  |  |  |  |  |  |  |  |  |  |  |  |  |  |  |  |  |  |  |  |  |  |  |  |  |  |  |  |  |  |  |  |  |  |  |  |  |  |  |  |  |  |  |  |  |  |  |  |  |  |  |  |  |  |  |  |  |  |  |  |  |  |  |  |  |  |  |  |  |  |  |  |  |  |  |  |  |  |  |  |  |  |  |  |  |  |  |  |  |  |  |  |  |  |  |  |  |  |  |  |  |  |  |  |  |  |  |  |  |  |  |  |  |  |  |  |  |  |  |  |  |  |  |  |  |  |  |  |  |  |  |  |  |  |  |  |  |  |  |  |  |  |  |  |  |  |  |  |  |  |  |  |  |  |  |  |  |  |  |  |  |  |  |  |  |  |  |  |  |  |  |  |  |  |  |  |  |  |  |  |  |  |  |  |  |  |  |  |  |  |  |  |  |  |  |  |  |  |  |  |  |  |  |  |  |  |  |  |  |  |  |  |  |  |  |  |  |  |  |  |  |  |  |  |  |  |  |  |  |  |  |  |  |  |  |  |  |  |  |  |  |  |  |  |  |  |  |  |  |  |  |  |  |  |  |  |  |  |  |  |  |  |  |  |  |  |  |  |  |  |  |  |  |  |  |  |  |  |  |  |  |  |  |  |  |  |  |  |  |  |  |  |  |  |  |  |  |  |  |  |  |  |  |  |  |  |  |  |  |  |  |  |  |  |  |  |  |  |  |  |  |  |  |  |  |  |  |  |  |  |  |  |  |  |  |  |  |  |  |  |  |  |  |  |  |  |  |  |  |  |  |  |  |  |  |  |  |  |  |  |  |  |  |  |  |  |  |  |  |  |  |  |  |  |  |  |  |  |  |  |  |  |  |  |  |  |  |  |  |  |  |  |  |  |  |  |  |  |  |  |  |  |  |  |  |  |  |  |  |  |  |  |  |  |  |  |  |  |  |  |  |  |  |  |  |  |  |  |  |  |  |  |  |  |  |  |  |  |  |  |  |  |  |  |  |  |  |  |  |  |  |  |  |  |  |  |  |  |  |  |  |  |  |  |  |  |  |  |  |  |  |  |  |  |  |  |  |  |  |  |  |  |  |  |  |  |  |  |  |  |  |  |  |  |  |  |  |  |  |  |  |  |  |  |  |  |  |  |  |  |  |  |  |  |  |  |  |  |  |  |  |  |  |  |  |  |  |  |  |  |  |  |  |  |  |  |  |  |  |  |  |  |  |  |  |  |  |  |  |  |  |  |  |  |  |  |  |  |  |  |  |  |  |  |  |  |  |  |  |  |  |  |  |  |  |  |  |  |  |  |  |  |  |  |  |  |  |  |  |  |  |  |  |  |  |  |  |  |  |  |  |  |  |  |  |  |  |  |  |  |  |  |  |  |  |  |  |  |  |  |  |  |  |  |  |  |  |  |  |  |  |  |  |  |  |  |  |  |  |  |  |  |  |  |  |  |  |  |  |  |  |  |  |  |  |  |  |  |  |  |  |  |  |  |  |  |  |  |  |  |  |  |  |  |  |  |  |  |  |  |  |  |  |  |  |  |  |  |  |  |  |  |  |  |  |  |  |  |  |  |  |  |  |  |  |  |  |  |  |  |  |  |  |  |  |  |  |  |  |  |  |  |  |  |  |  |  |  |  |  |  |  |  |  |  |  |  |  |  |  |  |  |  |  |  |  |  |  |  |  |  |  |  |  |  |  |  |  |  |  |  |  |  |  |  |  |  |  |  |  |  |  |  |  |  |  |  |  |  |  |  |  |  |  |  |  |  |  |  |  |  |  |  |  |  |  |  |  |  |  |  |  |  |  |  |  |  |  |  |  |  |  |  |  |  |  |  |  |  |  |  |  |  |  |  |  |  |  |  |  |  |  |  |  |  |  |  |  |  |  |  |  |  |  |  |  |  |  |  |  |  |  |  |  |  |  |  |  |  |  |  |  |  |  |  |  |  |  |  |  |  |  |  |  |  |  |  |  |  |  |  |  |  |  |  |  |  |  |  |  |  |  |  |  |  |  |  |  |  |  |  |  |  |  |  |  |  |  |  |  |  |  |  |  |  |  |  |  |  |  |  |  |  |  |  |  |  |  |  |  |  |  |  |  |  |  |  |  |  |  |  |  |  |  |  |  |  |  |  |  |  |  |  |  |  |  |  |  |  |  |  |  |  |  |  |  |  |  |  |  |  |  |  |  |  |  |  |  |  |  |  |  |  |  |  |  |  |  |  |  |  |  |  |  |  |  |  |  |  |  |  |  |  |  |  |  |  |  |  |  |  |  |  |  |  |  |  |  |  |  |  |  |  |  |  |  |  |  |  |  |  |  |  |  |  |  |  |  |  |  |  |  |  |  |  |  |  |  |  |  |  |  |  |  |  |  |  |  |  |  |  |  |  |  |  |  |  |  |  |  |  |  |  |  |  |  |  |  |  |  |  |  |  |  |  |  |  |  |  |  |  |  |  |  |  |  |  |  |  |  |  |  |  |  |  |  |  |  |  |  |  |  |  |  |  |  |  |  |  |  |  |  |  |  |  |  |  |  |  |  |  |  |  |  |  |  |  |  |  |  |  |  |  |  |  |  |  |  |  |  |  |  |  |  |  |  |  |  |  |  |  |  |  |  |  |  |  |  |  |  |  |  |  |  |  |  |  |  |  |  |  |  |  |  |  |  |  |  |  |  |  |  |  |  |  |  |  |  |  |  |  |  |  |  |  |  |  |  |  |  |  |  |  |  |  |  |  |  |  |  |  |  |  |  |  |  |  |  |  |  |  |  |  |  |  |  |  |  |  |  |  |  |  |  |  |  |  |  |  |  |  |  |  |  |  |  |  |  |  |  |  |  |  |  |  |  |  |  |  |  |  |  |  |  |  |  |  |  |  |  |  |  |  |  |  |  |  |  |  |  |  |  |  |  |  |  |  |  |  |  |  |  |  |  |  |  |  |  |  |  |  |  |  |  |  |  |  |  |  |  |  |  |  |  |  |  |  |  |  |  |  |  |  |  |  |  |  |  |  |  |  |  |  |  |  |  |  |  |  |  |  |  |  |  |  |  |  |  |  |  |  |  |  |  |  |  |  |  |  |  |  |  |  |  |  |  |  |  |  |  |  |  |  |  |  |  |  |  |  |  |  |  |  |  |  |  |  |  |  |  |  |  |  |  |  |  |  |  |  |  |  |  |  |  |  |  |  |  |  |  |  |  |  |  |  |  |  |  |  |  |  |  |  |  |  |  |  |  |  |  |  |  |  |  |  |  |  |  |  |  |  |  |  |  |  |  |  |  |  |  |  |  |  |  |  |  |  |  |  |  |  |  |  |  |  |  |  |  |  |  |  |  |  |  |  |  |  |  |  |  |  |  |  |  |  |  |  |  |  |  |  |  |  |  |  |  |  |  |  |  |  |  |  |  |  |  |  |  |  |  |  |  |  |  |  |  |  |  |  |  |  |  |  |  |  |  |  |  |  |  |  |  |  |  |  |  |  |  |  |  |  |  |  |  |  |  |  |  |  |  |  |  |  |  |  |  |  |  |  |  |  |  |  |  |  |  |  |  |  |  |  |  |  |  |  |  |  |  |  |  |  |  |  |  |  |  |  |  |  |  |  |  |  |  |  |  |  |  |  |  |  |  |  |  |  |  |  |  |  |  |  |  |  |  |  |  |  |  |  |  |  |  |  |  |  |  |  |  |  |  |  |  |  |  |  |  |  |  |  |  |  |  |  |  |  |  |  |  |  |  |  |  |  |  |  |  |  |  |  |  |  |  |  |  |  |  |  |  |  |  |  |  |  |  |  |  |  |  |  |  |  |  |  |  |  |  |  |  |  |  |  |  |  |  |  |  |  |  |  |  |  |  |  |  |  |  |  |  |  |  |  |  |  |  |  |  |  |  |  |  |  |  |  |  |  |  |  |  |  |  |  |  |  |  |  |  |  |  |  |  |  |  |  |  |  |  |  |  |  |  |  |  |  |  |  |  |  |  |  |  |  |  |  |  |  |  |  |  |  |  |  |  |  |  |  |  |  |  |  |  |  |  |  |  |  |  |  |  |  |  |  |  |  |  |  |  |  |  |  |  |  |  |  |  |  |  |  |  |  |  |  |  |  |  |  |  |  |  |  |  |  |  |  |  |  |  |  |  |  |  |  |  |  |  |  |  |  |  |  |  |  |  |  |  |  |  |  |  |  |  |  |  |  |  |  |  |  |  |  |  |  |  |  |  |  |  |  |  |  |  |  |  |  |  |  |  |  |  |  |  |  |  |  |  |  |  |  |  |  |  |  |  |  |  |  |  |  |  |  |  |  |  |  |  |  |  |  |  |  |  |  |  |  |  |  |  |  |  |  |  |  |  |  |  |  |  |  |  |  |  |  |  |  |  |  |  |  |  |  |  |  |  |  |  |  |  |  |  |  |  |  |  |  |  |  |  |  |  |  |  |  |  |  |  |  |  |  |  |  |  |  |  |  |  |  |  |  |  |  |  |  |  |  |  |  |  |  |  |  |  |  |  |  |  |  |  |  |  |  |  |  |  |  |  |  |  |  |  |  |  |  |  |  |  |  |  |  |  |  |  |  |  |  |  |  |  |  |  |  |  |  |  |  |  |  |  |  |  |  |  |  |  |  |  |  |  |  |  |  |  |  |  |  |  |  |  |  |  |  |  |  |  |  |  |  |  |  |  |  |  |  |  |  |  |  |  |  |  |  |  |  |  |  |  |  |  |  |  |  |  |  |  |  |  |  |  |  |  |  |  |  |  |  |  |  |  |  |  |  |  |  |  |  |  |  |  |  |  |  |  |  |  |  |  |  |  |  |  |  |  |  |  |  |  |  |  |  |  |  |  |  |  |  |  |  |  |  |  |  |  |  |  |  |  |  |  |  |  |  |  |  |  |  |  |  |  |  |  |  |  |  |  |  |  |  |  |  |  |  |  |  |  |  |  |  |  |  |  |  |  |  |  |  |  |  |  |  |  |  |  |  |  |  |  |  |  |  |  |  |  |  |  |  |  |  |  |  |  |  |  |  |  |  |  |  |  |  |  |  |  |  |  |  |  |  |  |  |  |  |  |  |  |  |  |  |  |  |  |  |  |  |  |  |  |  |  |  |  |  |  |  |  |  |  |  |  |  |  |  |  |  |  |  |  |  |  |  |  |  |  |  |  |  |  |  |  |  |  |  |  |  |  |  |  |  |  |  |  |  |  |  |  |  |  |  |  |  |  |  |  |  |  |  |  |  |  |  |  |  |  |  |  |  |  |  |  |  |  |  |  |  |  |  |  |  |  |  |  |  |  |  |  |  |  |  |  |  |  |  |  |  |  |  |  |  |  |  |  |  |  |  |  |  |  |  |  |  |  |  |  |  |  |  |  |  |  |  |  |  |  |  |  |  |  |  |  |  |  |  |  |  |  |  |  |  |  |  |  |  |  |  |  |  |  |  |  |  |  |  |  |  |  |  |  |  |  |  |  |  |  |  |  |  |  |  |  |  |  |  |  |  |  |  |  |  |  |  |  |  |  |  |  |  |  |  |  |  |  |  |  |  |  |  |  |  |  |  |  |  |  |  |  |  |  |  |  |  |  |  |  |  |  |  |  |  |  |  |  |  |  |  |  |  |  |  |  |  |  |  |  |  |  |  |  |  |  |  |  |  |  |  |  |  |  |  |  |  |  |  |  |  |  |  |  |  |  |  |  |  |  |  |  |  |  |  |  |  |  |  |  |  |  |  |  |  |  |  |  |  |  |  |  |  |  |  |  |  |  |  |  |  |  |  |  |  |  |  |  |  |  |  |  |  |  |  |  |  |  |  |  |  |  |  |  |  |  |  |  |  |  |  |  |  |  |  |  |  |  |  |  |  |  |  |  |  |  |  |  |  |  |  |  |  |  |  |  |  |  |  |  |  |  |  |  |  |  |  |  |  |  |  |  |  |  |  |  |  |  |  |  |  |  |  |  |  |  |  |  |  |  |  |  |  |  |  |  |  |  |  |  |  |  |  |  |  |  |  |  |  |  |  |  |  |  |  |  |  |  |  |  |  |  |  |  |  |  |  |  |  |  |  |  |  |  |  |  |  |  |  |  |  |  |  |  |  |  |  |  |  |  |  |  |  |  |  |  |  |  |  |  |  |  |  |  |  |  |  |  |  |  |  |  |  |  |  |  |  |  |  |  |  |  |  |  |  |  |  |  |  |  |  |  |  |  |  |  |  |  |  |  |  |  |  |  |  |  |  |  |  |  |  |  |  |  |  |  |  |  |  |  |  |  |  |  |  |  |  |  |  |  |  |  |  |  |  |  |  |  |  |  |  |  |  |  |  |  |  |  |  |  |  |  |  |  |  |  |  |  |  |  |  |  |  |  |  |  |  |  |  |  |  |  |  |  |  |  |  |  |  |  |  |  |  |  |  |  |  |  |  |  |  |  |  |  |  |  |  |  |  |  |  |  |  |  |  |  |  |  |  |  |  |  |  |  |  |  |  |  |  |  |  |  |  |  |  |  |  |  |  |  |  |  |  |  |  |  |  |  |  |  |  |  |  |  |  |  |  |  |  |  |  |  |  |  |  |  |  |  |  |  |  |  |  |  |  |  |  |  |  |  |  |  |  |  |  |  |  |  |  |  |  |  |  |  |  |  |  |  |  |  |  |  |  |  |  |  |  |  |  |  |  |  |  |  |  |  |  |  |  |  |  |  |  |  |  |  |  |  |  |  |  |  |  |  |  |  |  |  |  |  |  |  |  |  |  |  |  |  |  |  |  |  |  |  |  |  |  |  |  |  |  |  |  |  |  |  |  |  |  |  |  |  |  |  |  |  |  |  |  |  |  |  |  |  |  |  |  |  |  |  |  |  |  |  |  |  |  |  |  |  |  |  |  |  |  |  |  |  |  |  |  |  |  |  |  |  |  |  |  |  |  |  |  |  |  |  |  |  |  |  |  |  |  |  |  |  |  |  |  |  |  |  |  |  |  |  |  |  |  |  |  |  |  |  |  |  |  |  |  |  |  |  |  |  |  |  |  |  |  |  |  |  |  |  |  |  |  |  |  |  |  |  |  |  |  |  |  |  |  |  |  |  |  |  |  |  |  |  |  |  |  |  |  |  |  |  |  |  |  |  |  |  |  |  |  |  |  |  |  |  |  |  |  |  |  |  |  |  |  |  |  |  |  |  |  |  |  |  |  |  |  |  |  |  |  |  |  |  |  |  |  |  |  |  |  |  |  |  |  |  |  |  |  |  |  |  |  |  |  |  |  |  |  |  |  |  |  |  |  |  |  |  |  |  |  |  |  |  |  |  |  |  |  |  |  |  |  |  |  |  |  |  |  |  |  |  |  |  |  |  |  |  |  |  |  |  |  |  |  |  |  |  |  |  |  |  |  |  |  |  |  |  |  |  |  |  |  |  |  |  |  |  |  |  |  |  |  |  |  |  |  |  |  |  |  |  |  |  |  |  |  |  |  |  |  |  |  |  |  |  |  |  |  |  |  |  |  |  |  |  |  |  |  |  |  |  |  |  |  |  |  |  |  |  |  |  |  |  |  |  |  |  |  |  |  |  |  |  |  |  |  |  |  |  |  |  |  |  |  |  |  |  |  |  |  |  |  |  |  |  |  |  |  |  |  |  |  |  |  |  |  |  |  |  |  |  |  |  |  |  |  |  |  |  |  |  |  |  |  |  |  |  |  |  |  |  |  |  |  |  |  |  |  |  |  |  |  |  |  |  |  |  |  |  |  |  |  |  |  |  |  |  |  |  |  |  |  |  |  |  |  |  |  |  |  |  |  |  |  |  |  |  |  |  |  |  |  |  |  |  |  |  |  |  |  |  |  |  |  |  |  |  |  |  |  |  |  |  |  |  |  |  |  |  |  |  |  |  |  |  |  |  |  |  |  |  |  |  |  |  |  |  |  |  |  |  |  |  |  |  |  |  |  |  |  |  |  |  |  |  |  |  |  |  |  |  |  |  |  |  |  |  |  |  |  |  |  |  |  |  |  |  |  |  |  |  |  |  |  |  |  |  |  |  |  |  |  |  |  |  |  |  |  |  |  |  |  |  |  |  |  |  |  |  |  |  |  |  |  |  |  |  |  |  |  |  |  |  |  |  |  |  |  |  |  |  |  |  |  |  |  |  |  |  |  |  |  |  |  |  |  |  |  |  |  |  |  |  |  |  |  |  |  |  |  |  |  |  |  |  |  |  |  |  |  |  |  |  |  |  |  |  |  |  |  |  |  |  |  |  |  |  |  |  |  |  |  |  |  |  |  |  |  |  |  |  |  |  |  |  |  |  |  |  |  |  |  |  |  |  |  |  |  |  |  |  |  |  |  |  |  |  |  |  |  |  |  |  |  |  |  |  |  |  |  |  |  |  |  |  |  |  |  |  |  |  |  |  |  |  |  |  |  |  |  |  |  |  |  |  |  |  |  |  |  |  |  |  |  |  |  |  |  |  |  |  |  |  |  |  |  |  |  |  |  |  |  |  |  |  |  |  |  |  |  |  |  |  |  |  |  |  |  |  |  |  |  |  |  |  |  |  |  |  |  |  |  |  |  |  |  |  |  |  |  |  |  |  |  |  |  |  |  |  |  |  |  |  |  |  |  |  |  |  |  |  |  |  |  |  |  |  |  |  |  |  |  |  |  |  |  |  |  |  |  |  |  |  |  |  |  |  |  |  |  |  |  |  |  |  |  |  |  |  |  |  |  |  |  |  |  |  |  |  |  |  |  |  |  |  |  |  |  |  |  |  |  |  |  |  |  |  |  |  |  |  |  |  |  |  |  |  |  |  |  |  |  |  |  |  |  |  |  |  |  |  |  |  |  |  |  |  |  |  |  |  |  |  |  |  |  |  |  |  |  |  |  |  |  |  |  |  |  |  |  |  |  |  |  |  |  |  |  |  |  |  |  |  |  |  |  |  |  |  |  |  |  |  |  |  |  |  |  |  |  |  |  |  |  |  |  |  |  |  |  |  |  |  |  |  |  |  |  |  |  |  |  |  |  |  |  |  |  |  |  |  |  |  |  |  |  |  |  |  |  |  |  |  |  |  |  |  |  |  |  |  |  |  |  |  |  |  |  |  |  |  |  |  |  |  |  |  |  |  |  |  |  |  |  |  |  |  |  |  |  |  |  |  |  |  |  |  |  |  |  |  |  |  |  |  |  |  |  |  |  |  |  |  |  |  |  |  |  |  |  |  |  |  |  |  |  |  |  |  |  |  |  |  |  |  |  |  |  |  |  |  |  |  |  |  |  |  |  |  |  |  |  |  |  |  |  |  |  |  |  |  |  |  |  |  |  |  |  |  |  |  |  |  |  |  |  |  |  |  |  |  |  |  |  |  |  |  |  |  |  |  |  |  |  |  |  |  |  |  |  |  |  |  |  |  |  |  |  |  |  |  |  |  |  |  |  |  |  |  |  |  |  |  |  |  |  |  |  |  |  |  |  |  |  |  |  |  |  |  |  |  |  |  |  |  |  |  |  |  |  |  |  |  |  |  |  |  |  |  |  |  |  |  |  |  |  |  |  |  |  |  |  |  |  |  |  |  |  |  |  |  |  |  |  |  |  |  |  |  |  |  |  |  |  |  |  |  |  |  |  |  |  |  |  |  |  |  |  |  |  |  |  |  |  |  |  |  |  |  |  |  |  |  |  |  |  |  |  |  |  |  |  |  |  |  |  |  |  |  |  |  |  |  |  |  |  |  |  |  |  |  |  |  |  |  |  |  |  |  |  |  |  |  |  |  |  |  |  |  |  |  |  |  |  |  |  |  |  |  |  |  |  |  |  |  |  |  |  |  |  |  |  |  |  |  |  |  |  |  |  |  |  |  |  |  |  |  |  |  |  |  |  |  |  |  |  |  |  |  |  |  |  |  |  |  |  |  |  |  |  |  |  |  |  |  |  |  |  |  |  |  |  |  |  |  |  |  |  |  |  |  |  |  |  |  |  |  |  |  |  |  |  |  |  |  |  |  |  |  |  |  |  |  |  |  |  |  |  |  |  |  |  |  |  |  |  |  |  |  |  |  |  |  |  |  |  |  |  |  |  |  |  |  |  |  |  |  |  |  |  |  |  |  |  |  |  |  |  |  |  |  |  |  |  |  |  |  |  |  |  |  |  |  |  |  |  |  |  |  |  |  |  |  |  |  |  |  |  |  |  |  |  |  |  |  |  |  |  |  |  |  |  |  |  |  |  |  |  |  |  |  |  |  |  |  |  |  |  |  |  |  |  |  |  |  |  |  |  |  |  |  |  |  |  |  |  |  |  |  |  |  |  |  |  |  |  |  |  |  |  |  |  |  |  |  |  |  |  |  |  |  |  |  |  |  |  |  |  |  |  |  |  |  |  |  |  |  |  |  |  |  |  |  |  |  |  |  |  |  |  |  |  |  |  |  |  |  |  |  |  |  |  |  |  |  |  |  |  |  |  |  |  |  |  |  |  |  |  |  |  |  |  |  |  |  |  |  |  |  |  |  |  |  |  |  |  |  |  |  |  |  |  |  |  |  |  |  |  |  |  |  |  |  |  |  |  |  |  |  |  |  |  |  |  |  |  |  |  |  |  |  |  |  |  |  |  |  |  |  |  |  |  |  |  |  |  |  |  |  |  |  |  |  |  |  |  |  |  |  |  |  |  |  |  |  |  |  |  |  |  |  |  |  |  |  |  |  |  |  |  |  |  |  |  |  |  |  |  |  |  |  |  |  |  |  |  |  |  |  |  |  |  |  |  |  |  |  |  |  |  |  |  |  |  |  |  |  |  |  |  |  |  |  |  |  |  |  |  |  |  |  |  |  |  |  |  |  |  |  |  |  |  |  |  |  |  |  |  |  |  |  |  |  |  |  |  |  |  |  |  |  |  |  |  |  |  |  |  |  |  |  |  |  |  |  |  |  |  |  |  |  |  |  |  |  |  |  |  |  |  |  |  |  |  |  |  |  |  |  |  |  |  |  |  |  |  |  |  |  |  |  |  |  |  |  |  |  |  |  |  |  |  |  |  |  |  |  |  |  |  |  |  |  |  |  |  |  |  |  |  |  |  |  |  |  |  |  |  |  |  |  |  |  |  |  |  |  |  |  |  |  |  |  |  |  |  |  |  |  |  |  |  |  |  |  |  |  |  |  |  |  |  |  |  |  |  |  |  |  |  |  |  |  |  |  |  |  |  |  |  |  |  |  |  |  |  |  |  |  |  |  |  |  |  |  |  |  |  |  |  |  |  |  |  |  |  |  |  |  |  |  |  |  |  |  |  |  |  |  |  |  |  |  |  |  |  |  |  |  |  |  |  |  |  |  |  |  |  |  |  |  |  |  |  |  |  |  |  |  |  |  |  |  |  |  |  |  |  |  |  |  |  |  |  |  |  |  |  |  |  |  |  |  |  |  |  |  |  |  |  |  |  |  |  |  |  |  |  |  |  |  |  |  |  |  |  |  |  |  |  |  |  |  |  |  |  |  |  |  |  |  |  |  |  |  |  |  |  |  |  |  |  |  |  |  |  |  |  |  |  |  |  |  |  |  |  |  |  |  |  |  |  |  |  |  |  |  |  |  |  |  |  |  |  |  |  |  |  |  |  |  |  |  |  |  |  |  |  |  |  |  |  |  |  |  |  |  |  |  |  |  |  |  |  |  |  |  |  |  |  |  |  |  |  |  |  |  |  |  |  |  |  |  |  |  |  |  |  |  |  |  |  |  |  |  |  |  |  |  |  |  |  |  |  |  |  |  |  |  |  |  |  |  |  |  |  |  |  |  |  |  |  |  |  |  |  |  |  |  |  |  |  |  |  |  |  |  |  |  |  |  |  |  |  |  |  |  |  |  |  |  |  |  |  |  |  |  |  |  |  |  |  |  |  |  |  |  |  |  |  |  |  |  |  |  |  |  |  |  |  |  |  |  |  |  |  |  |  |  |  |  |  |  |  |  |  |  |  |  |  |  |  |  |  |  |  |  |  |  |  |  |  |  |  |  |  |  |  |  |  |  |  |  |  |  |  |  |  |  |  |  |  |  |  |  |  |  |  |  |  |  |  |  |  |  |  |  |  |  |  |  |  |  |  |  |  |  |  |  |  |  |  |  |  |  |  |  |  |  |  |  |  |  |  |  |  |  |  |  |  |  |  |  |  |  |  |  |  |  |  |  |  |  |  |  |  |  |  |  |  |  |  |  |  |  |  |  |  |  |  |  |  |  |  |  |  |  |  |  |  |  |  |  |  |  |  |  |  |  |  |  |  |  |  |  |  |  |  |  |  |  |  |  |  |  |  |  |  |  |  |  |  |  |  |  |  |  |  |  |  |  |  |  |  |  |  |  |  |  |  |  |  |  |  |  |  |  |  |  |  |  |  |  |  |  |  |  |  |  |  |  |  |  |  |  |  |  |  |  |  |  |  |  |  |  |  |  |  |  |  |  |  |  |  |  |  |  |  |  |  |  |  |  |  |  |  |  |  |  |  |  |  |  |  |  |  |  |  |  |  |  |  |  |  |  |  |  |  |  |  |  |  |  |  |  |  |  |  |  |  |  |  |  |  |  |  |  |  |  |  |  |  |  |  |  |  |  |  |  |  |  |  |  |  |  |  |  |  |  |  |  |  |  |  |  |  |  |  |  |  |  |  |  |  |  |  |  |  |  |  |  |  |  |  |  |  |  |  |  |  |  |  |  |  |  |  |  |  |  |  |  |  |  |  |  |  |  |  |  |  |  |  |  |  |  |  |  |  |  |  |  |  |  |  |  |  |  |  |  |  |  |  |  |  |  |  |  |  |  |  |  |  |  |  |  |  |  |  |  |  |  |  |  |  |  |  |  |  |  |  |  |  |  |  |  |  |  |  |  |  |  |  |  |  |  |  |  |  |  |  |  |  |  |  |  |  |  |  |  |  |  |  |  |  |  |  |  |  |  |  |  |  |  |  |  |  |  |  |  |  |  |  |  |  |  |  |  |  |  |  |  |  |  |  |  |  |  |  |  |  |  |  |  |  |  |  |  |  |  |  |  |  |  |  |  |  |  |  |  |  |  |  |  |  |  |  |  |  |  |  |  |  |  |  |  |  |  |  |  |  |  |  |  |  |  |  |  |  |  |  |  |  |  |  |  |  |  |  |  |  |  |  |  |  |  |  |  |  |  |  |  |  |  |  |  |  |  |  |  |  |  |  |  |  |  |  |  |  |  |  |  |  |  |  |  |  |  |  |  |  |  |  |  |  |  |  |  |  |  |  |  |  |  |  |  |  |  |  |  |  |  |  |  |  |  |  |  |  |  |  |  |  |  |  |  |  |  |  |  |  |  |  |  |  |  |  |  |  |  |  |  |  |  |  |  |  |  |  |  |  |  |  |  |  |  |  |  |  |  |  |  |  |  |  |  |  |  |  |  |  |  |  |  |  |  |  |  |  |  |  |  |  |  |  |  |  |  |  |  |  |  |  |  |  |  |  |  |  |  |  |  |  |  |  |  |  |  |  |  |  |  |  |  |  |  |  |  |  |  |  |  |  |  |  |  |  |  |  |  |  |  |  |  |  |  |  |  |  |  |  |  |  |  |  |  |  |  |  |  |  |  |  |  |  |  |  |  |  |  |  |  |  |  |  |  |  |  |  |  |  |  |  |  |  |  |  |  |  |  |  |  |  |  |  |  |  |  |  |  |  |  |  |  |  |  |  |  |  |  |  |  |  |  |  |  |  |  |  |  |  |  |  |  |  |  |  |  |  |  |  |  |  |  |  |  |  |  |  |  |  |  |  |  |  |  |  |  |  |  |  |  |  |  |  |  |  |  |  |  |  |  |  |  |  |  |  |  |  |  |  |  |  |  |  |  |  |  |  |  |  |  |  |  |  |  |  |  |  |  |  |  |  |  |  |  |  |  |  |  |  |  |  |  |  |  |  |  |  |  |  |  |  |  |  |  |  |  |  |  |  |  |  |  |  |  |  |  |  |  |  |  |  |  |  |  |  |  |  |  |  |  |  |  |  |  |  |  |  |  |  |  |  |  |  |  |  |  |  |  |  |  |  |  |  |  |  |  |  |  |  |  |  |  |  |  |  |  |  |  |  |  |  |  |  |  |  |  |  |  |  |  |  |  |  |  |  |  |  |  |  |  |  |  |  |  |  |  |  |  |  |  |  |  |  |  |  |  |  |  |  |  |  |  |  |  |  |  |  |  |  |  |  |  |  |  |  |  |  |  |  |  |  |  |  |  |  |  |  |  |  |  |  |  |  |  |  |  |  |  |  |  |  |  |  |  |  |  |  |  |  |  |  |  |  |  |  |  |  |  |  |  |  |  |  |  |  |  |  |  |  |  |  |  |  |  |  |  |  |  |  |  |  |  |  |  |  |  |  |  |  |  |  |  |  |  |  |  |  |  |  |  |  |  |  |  |  |  |  |  |  |  |  |  |  |  |  |  |  |  |  |  |  |  |  |  |  |  |  |  |  |  |  |  |  |  |  |  |  |  |  |  |  |  |  |  |  |  |  |  |  |  |  |  |  |  |  |  |  |  |  |  |  |  |  |  |  |  |  |  |  |  |  |  |  |  |  |  |  |  |  |  |  |  |  |  |  |  |  |  |  |  |  |  |  |  |  |  |  |  |  |  |  |  |  |  |  |  |  |  |  |  |  |  |  |  |  |  |  |  |  |  |  |  |  |  |  |  |  |  |  |  |  |  |  |  |  |  |  |  |  |  |  |  |  |  |  |  |  |  |  |  |  |  |  |  |  |  |  |  |  |  |  |  |  |  |  |  |  |  |  |  |  |  |  |  |  |  |  |  |  |  |  |  |  |  |  |  |  |  |  |  |  |  |  |  |  |  |  |  |  |  |  |  |  |  |  |  |  |  |  |  |  |  |  |  |  |  |  |  |  |  |  |  |  |  |  |  |  |  |  |  |  |  |  |  |  |  |  |  |  |  |  |  |  |  |  |  |  |  |  |  |  |  |  |  |  |  |  |  |  |  |  |  |  |  |  |  |  |  |  |  |  |  |  |  |  |  |  |  |  |  |  |  |  |  |  |  |  |  |  |  |  |  |  |  |  |  |  |  |  |  |  |  |  |  |  |  |  |  |  |  |  |  |  |  |  |  |  |  |  |  |  |  |  |  |  |  |  |  |  |  |  |  |  |  |  |  |  |  |  |  |  |  |  |  |  |  |  |  |  |  |  |  |  |  |  |  |  |  |  |  |  |  |  |  |  |  |  |  |  |  |  |  |  |  |  |  |  |  |  |  |  |  |  |  |  |  |  |  |  |  |  |  |  |  |  |  |  |  |  |  |  |  |  |  |  |  |  |  |  |  |  |  |  |  |  |  |  |  |  |  |  |  |  |  |  |  |  |  |  |  |  |  |  |  |  |  |  |  |  |  |  |  |  |  |  |  |  |  |  |  |  |  |  |  |  |  |  |  |  |  |  |  |  |  |  |  |  |  |  |  |  |  |  |  |  |  |  |  |  |  |  |  |  |  |  |  |  |  |  |  |  |  |  |  |  |  |  |  |  |  |  |  |  |  |  |  |  |  |  |  |  |  |  |  |  |  |  |  |  |  |  |  |  |  |  |  |  |  |  |  |  |  |  |  |  |  |  |  |  |  |  |  |  |  |  |  |  |  |  |  |  |  |  |  |  |  |  |  |  |  |  |  |  |  |  |  |  |  |  |  |  |  |  |  |  |  |  |  |  |  |  |  |  |  |  |  |  |  |  |  |  |  |  |  |  |  |  |  |  |  |  |  |  |  |  |  |  |  |  |  |  |  |  |  |  |  |  |  |  |  |  |  |  |  |  |  |  |  |  |  |  |  |  |  |  |  |  |  |  |  |  |  |  |  |  |  |  |  |  |  |  |  |  |  |  |  |  |  |  |  |  |  |  |  |  |  |  |  |  |  |  |  |  |  |  |  |  |  |  |  |  |  |  |  |  |  |  |  |  |  |  |  |  |  |  |  |  |  |  |  |  |  |  |  |  |  |  |  |  |  |  |  |  |  |  |  |  |  |  |  |  |  |  |  |  |  |  |  |  |  |  |  |  |  |  |  |  |  |  |  |  |  |  |  |  |  |  |  |  |  |  |  |  |  |  |  |  |  |  |  |  |  |  |  |  |  |  |  |  |  |  |  |  |  |  |  |  |  |  |  |  |  |  |  |  |  |  |  |  |  |  |  |  |  |  |  |  |  |  |  |  |  |  |  |  |  |  |  |  |  |  |  |  |  |  |  |  |  |  |  |  |  |  |  |  |  |  |  |  |  |  |  |  |  |  |  |  |  |  |  |  |  |  |  |  |  |  |  |  |  |  |  |  |  |  |  |  |  |  |  |  |  |  |  |  |  |  |  |  |  |  |  |  |  |  |  |  |  |  |  |  |  |  |  |  |  |  |  |  |  |  |  |  |  |  |  |  |  |  |  |  |  |  |  |  |  |  |  |  |  |  |  |  |  |  |  |  |  |  |  |  |  |  |  |  |  |  |  |  |  |  |  |  |  |  |  |  |  |  |  |  |  |  |  |  |  |  |  |  |  |  |  |  |  |  |  |  |  |  |  |  |  |  |  |  |  |  |  |  |  |  |  |  |  |  |  |  |  |  |  |  |  |  |  |  |  |  |  |  |  |  |  |  |  |  |  |  |  |  |  |  |  |  |  |  |  |  |  |  |  |  |  |  |  |  |  |  |  |  |  |  |  |  |  |  |  |  |  |  |  |  |  |  |  |  |  |  |  |  |  |  |  |  |  |  |  |  |  |  |  |  |  |  |  |  |  |  |  |  |  |  |  |  |  |  |  |  |  |  |  |  |  |  |  |  |  |  |  |  |  |  |  |  |  |  |  |  |  |  |  |  |  |  |  |  |  |  |  |  |  |  |  |  |  |  |  |  |  |  |  |  |  |  |  |  |  |  |  |  |  |  |  |  |  |  |  |  |  |  |  |  |  |  |  |  |  |  |  |  |  |  |  |  |  |  |  |  |  |  |  |  |  |  |  |  |  |  |  |  |  |  |  |  |  |  |  |  |  |  |  |  |  |  |  |  |  |  |  |  |  |  |  |  |  |  |  |  |  |  |  |  |  |  |  |  |  |  |  |  |  |  |  |  |  |  |  |  |  |  |  |  |  |  |  |  |  |  |  |  |  |  |  |  |  |  |  |  |  |  |  |  |  |  |  |  |  |  |  |  |  |  |  |  |  |  |  |  |  |  |  |  |  |  |  |  |  |  |  |  |  |  |  |  |  |  |  |  |  |  |  |  |  |  |  |  |  |  |  |  |  |  |  |  |  |  |  |  |  |  |  |  |  |  |  |  |  |  |  |  |  |  |  |  |  |  |  |  |  |  |  |  |  |  |  |  |  |  |  |  |  |  |  |  |  |  |  |  |  |  |  |  |  |  |  |  |  |  |  |  |  |  |  |  |  |  |  |  |  |  |  |  |  |  |  |  |  |  |  |  |  |  |  |  |  |  |  |  |  |  |  |  |  |  |  |  |  |  |  |  |  |  |  |  |  |  |  |  |  |  |  |  |  |  |  |  |  |  |  |  |  |  |  |  |  |  |  |  |  |  |  |  |  |  |  |  |  |  |  |  |  |  |  |  |  |  |  |  |  |  |  |  |  |  |  |  |  |  |  |  |  |  |  |  |  |  |  |  |  |  |  |  |  |  |  |  |  |  |  |  |  |  |  |  |  |  |  |  |  |  |  |  |  |  |  |  |  |  |  |  |  |  |  |  |  |  |  |  |  |  |  |  |  |  |  |  |  |  |  |  |  |  |  |  |  |  |  |  |  |  |  |  |  |  |  |  |  |  |  |  |  |  |  |  |  |  |  |  |  |  |  |  |  |  |  |  |  |  |  |  |  |  |  |  |  |  |  |  |  |  |  |  |  |  |  |  |  |  |  |  |  |  |  |  |  |  |  |  |  |  |  |  |  |  |  |  |  |  |  |  |  |  |  |  |  |  |  |  |  |  |  |  |  |  |  |  |  |  |  |  |  |  |  |  |  |  |  |  |  |  |  |  |  |  |  |  |  |  |  |  |  |  |  |  |  |  |  |  |  |  |  |  |  |  |  |  |  |  |  |  |  |  |  |  |  |  |  |  |  |  |  |  |  |  |  |  |  |  |  |  |  |  |  |  |  |  |  |  |  |  |  |  |  |  |  |  |  |  |  |  |  |  |  |  |  |  |  |  |  |  |  |  |  |  |  |  |  |  |  |  |  |  |  |  |  |  |  |  |  |  |  |  |  |  |  |  |  |  |  |  |  |  |  |  |  |  |  |  |  |  |  |  |  |  |  |  |  |  |  |  |  |  |  |  |  |  |  |  |  |  |  |  |  |  |  |  |  |  |  |  |  |  |  |  |  |  |  |  |  |  |  |  |  |  |  |  |  |  |  |  |  |  |  |  |  |  |  |  |  |  |  |  |  |  |  |  |  |  |  |  |  |  |  |  |  |  |  |  |  |  |  |  |  |  |  |  |  |  |  |  |  |  |  |  |  |  |  |  |  |  |  |  |  |  |  |  |  |  |  |  |  |  |  |  |  |  |  |  |  |  |  |  |  |  |  |  |  |  |  |  |  |  |  |  |  |  |  |  |  |  |  |  |  |  |  |  |  |  |  |  |  |  |  |  |  |  |  |  |  |  |  |  |  |  |  |  |  |  |  |  |  |  |  |  |  |  |  |  |  |  |  |  |  |  |  |  |  |  |  |  |  |  |  |  |  |  |  |  |  |  |  |  |  |  |  |  |  |  |  |  |  |  |  |  |  |  |  |  |  |  |  |  |  |  |  |  |  |  |  |  |  |  |  |  |  |  |  |  |  |  |  |  |  |  |  |  |  |  |  |  |  |  |  |  |  |  |  |  |  |  |  |  |  |  |  |  |  |  |  |  |  |  |  |  |  |  |  |  |  |  |  |  |  |  |  |  |  |  |  |  |  |  |  |  |  |  |  |  |  |  |  |  |  |  |  |  |  |  |  |  |  |  |  |  |  |  |  |  |  |  |  |  |  |  |  |  |  |  |  |  |  |  |  |  |  |  |  |  |  |  |  |  |  |  |  |  |  |  |  |  |  |  |  |  |  |  |  |  |  |  |  |  |  |  |  |  |  |  |  |  |  |  |  |  |  |  |  |  |  |  |  |  |  |  |  |  |  |  |  |  |  |  |  |  |  |  |  |  |  |  |  |  |  |  |  |  |  |  |  |  |  |  |  |  |  |  |  |  |  |  |  |  |  |  |  |  |  |  |  |  |  |  |  |  |  |  |  |  |  |  |  |  |  |  |  |  |  |  |  |  |  |  |  |  |  |  |  |  |  |  |  |  |  |  |  |  |  |  |  |  |  |  |  |  |  |  |  |  |  |  |  |  |  |  |  |  |  |  |  |  |  |  |  |  |  |  |  |  |  |  |  |  |  |  |  |  |  |  |  |  |  |  |  |  |  |  |  |  |  |  |  |  |  |  |  |  |  |  |  |  |  |  |  |  |  |  |  |  |  |  |  |  |  |  |  |  |  |  |  |  |  |  |  |  |  |  |  |  |  |  |  |  |  |  |  |  |  |  |  |  |  |  |  |  |  |  |  |  |  |  |  |  |  |  |  |  |  |  |  |  |  |  |  |  |  |  |  |  |  |  |  |  |  |  |  |  |  |  |  |  |  |  |  |  |  |  |  |  |  |  |  |  |  |  |  |  |  |  |  |  |  |  |  |  |  |  |  |  |  |  |  |  |  |  |  |  |  |  |  |  |  |  |  |  |  |  |  |  |  |  |  |  |  |  |  |  |  |  |  |  |  |  |  |  |  |  |  |  |  |  |  |  |  |  |  |  |  |  |  |  |  |  |  |  |  |  |  |  |  |  |  |  |  |  |  |  |  |  |  |  |  |  |  |  |  |  |  |  |  |  |  |  |  |  |  |  |  |  |  |  |  |  |  |  |  |  |  |  |  |  |  |  |  |  |  |  |  |  |  |  |  |  |  |  |  |  |  |  |  |  |  |  |  |  |  |  |  |  |  |  |  |  |  |  |  |  |  |  |  |  |  |  |  |  |  |  |  |  |  |  |  |  |  |  |  |  |  |  |  |  |  |  |  |  |  |  |  |  |  |  |  |  |  |  |  |  |  |  |  |  |  |  |  |  |  |  |  |  |  |  |  |  |  |  |  |  |  |  |  |  |  |  |  |  |  |  |  |  |  |  |  |  |  |  |  |  |  |  |  |  |  |  |  |  |  |  |  |  |  |  |  |  |  |  |  |  |  |  |  |  |  |  |  |  |  |  |  |  |  |  |  |  |  |  |  |  |  |  |  |  |  |  |  |  |  |  |  |  |  |  |  |  |  |  |  |  |  |  |  |  |  |  |  |  |  |  |  |  |  |  |  |  |  |  |  |  |  |  |  |  |  |  |  |  |
| --- | --- | --- | --- | --- | --- | --- | --- | --- | --- | --- | --- | --- | --- | --- | --- | --- | --- | --- | --- | --- | --- | --- | --- | --- | --- | --- | --- | --- | --- | --- | --- | --- | --- | --- | --- | --- | --- | --- | --- | --- | --- | --- | --- | --- | --- | --- | --- | --- | --- | --- | --- | --- | --- | --- | --- | --- | --- | --- | --- | --- | --- | --- | --- | --- | --- | --- | --- | --- | --- | --- | --- | --- | --- | --- | --- | --- | --- | --- | --- | --- | --- | --- | --- | --- | --- | --- | --- | --- | --- | --- | --- | --- | --- | --- | --- | --- | --- | --- | --- | --- | --- | --- | --- | --- | --- | --- | --- | --- | --- | --- | --- | --- | --- | --- | --- | --- | --- | --- | --- | --- | --- | --- | --- | --- | --- | --- | --- | --- | --- | --- | --- | --- | --- | --- | --- | --- | --- | --- | --- | --- | --- | --- | --- | --- | --- | --- | --- | --- | --- | --- | --- | --- | --- | --- | --- | --- | --- | --- | --- | --- | --- | --- | --- | --- | --- | --- | --- | --- | --- | --- | --- | --- | --- | --- | --- | --- | --- | --- | --- | --- | --- | --- | --- | --- | --- | --- | --- | --- | --- | --- | --- | --- | --- | --- | --- | --- | --- | --- | --- | --- | --- | --- | --- | --- | --- | --- | --- | --- | --- | --- | --- | --- | --- | --- | --- | --- | --- | --- | --- | --- | --- | --- | --- | --- | --- | --- | --- | --- | --- | --- | --- | --- | --- | --- | --- | --- | --- | --- | --- | --- | --- | --- | --- | --- | --- | --- | --- | --- | --- | --- | --- | --- | --- | --- | --- | --- | --- | --- | --- | --- | --- | --- | --- | --- | --- | --- | --- | --- | --- | --- | --- | --- | --- | --- | --- | --- | --- | --- | --- | --- | --- | --- | --- | --- | --- | --- | --- | --- | --- | --- | --- | --- | --- | --- | --- | --- | --- | --- | --- | --- | --- | --- | --- | --- | --- | --- | --- | --- | --- | --- | --- | --- | --- | --- | --- | --- | --- | --- | --- | --- | --- | --- | --- | --- | --- | --- | --- | --- | --- | --- | --- | --- | --- | --- | --- | --- | --- | --- | --- | --- | --- | --- | --- | --- | --- | --- | --- | --- | --- | --- | --- | --- | --- | --- | --- | --- | --- | --- | --- | --- | --- | --- | --- | --- | --- | --- | --- | --- | --- | --- | --- | --- | --- | --- | --- | --- | --- | --- | --- | --- | --- | --- | --- | --- | --- | --- | --- | --- | --- | --- | --- | --- | --- | --- | --- | --- | --- | --- | --- | --- | --- | --- | --- | --- | --- | --- | --- | --- | --- | --- | --- | --- | --- | --- | --- | --- | --- | --- | --- | --- | --- | --- | --- | --- | --- | --- | --- | --- | --- | --- | --- | --- | --- | --- | --- | --- | --- | --- | --- | --- | --- | --- | --- | --- | --- | --- | --- | --- | --- | --- | --- | --- | --- | --- | --- | --- | --- | --- | --- | --- | --- | --- | --- | --- | --- | --- | --- | --- | --- | --- | --- | --- | --- | --- | --- | --- | --- | --- | --- | --- | --- | --- | --- | --- | --- | --- | --- | --- | --- | --- | --- | --- | --- | --- | --- | --- | --- | --- | --- | --- | --- | --- | --- | --- | --- | --- | --- | --- | --- | --- | --- | --- | --- | --- | --- | --- | --- | --- | --- | --- | --- | --- | --- | --- | --- | --- | --- | --- | --- | --- | --- | --- | --- | --- | --- | --- | --- | --- | --- | --- | --- | --- | --- | --- | --- | --- | --- | --- | --- | --- | --- | --- | --- | --- | --- | --- | --- | --- | --- | --- | --- | --- | --- | --- | --- | --- | --- | --- | --- | --- | --- | --- | --- | --- | --- | --- | --- | --- | --- | --- | --- | --- | --- | --- | --- | --- | --- | --- | --- | --- | --- | --- | --- | --- | --- | --- | --- | --- | --- | --- | --- | --- | --- | --- | --- | --- | --- | --- | --- | --- | --- | --- | --- | --- | --- | --- | --- | --- | --- | --- | --- | --- | --- | --- | --- | --- | --- | --- | --- | --- | --- | --- | --- | --- | --- | --- | --- | --- | --- | --- | --- | --- | --- | --- | --- | --- | --- | --- | --- | --- | --- | --- | --- | --- | --- | --- | --- | --- | --- | --- | --- | --- | --- | --- | --- | --- | --- | --- | --- | --- | --- | --- | --- | --- | --- | --- | --- | --- | --- | --- | --- | --- | --- | --- | --- | --- | --- | --- | --- | --- | --- | --- | --- | --- | --- | --- | --- | --- | --- | --- | --- | --- | --- | --- | --- | --- | --- | --- | --- | --- | --- | --- | --- | --- | --- | --- | --- | --- | --- | --- | --- | --- | --- | --- | --- | --- | --- | --- | --- | --- | --- | --- | --- | --- | --- | --- | --- | --- | --- | --- | --- | --- | --- | --- | --- | --- | --- | --- | --- | --- | --- | --- | --- | --- | --- | --- | --- | --- | --- | --- | --- | --- | --- | --- | --- | --- | --- | --- | --- | --- | --- | --- | --- | --- | --- | --- | --- | --- | --- | --- | --- | --- | --- | --- | --- | --- | --- | --- | --- | --- | --- | --- | --- | --- | --- | --- | --- | --- | --- | --- | --- | --- | --- | --- | --- | --- | --- | --- | --- | --- | --- | --- | --- | --- | --- | --- | --- | --- | --- | --- | --- | --- | --- | --- | --- | --- | --- | --- | --- | --- | --- | --- | --- | --- | --- | --- | --- | --- | --- | --- | --- | --- | --- | --- | --- | --- | --- | --- | --- | --- | --- | --- | --- | --- | --- | --- | --- | --- | --- | --- | --- | --- | --- | --- | --- | --- | --- | --- | --- | --- | --- | --- | --- | --- | --- | --- | --- | --- | --- | --- | --- | --- | --- | --- | --- | --- | --- | --- | --- | --- | --- | --- | --- | --- | --- | --- | --- | --- | --- | --- | --- | --- | --- | --- | --- | --- | --- | --- | --- | --- | --- | --- | --- | --- | --- | --- | --- | --- | --- | --- | --- | --- | --- | --- | --- | --- | --- | --- | --- | --- | --- | --- | --- | --- | --- | --- | --- | --- | --- | --- | --- | --- | --- | --- | --- | --- | --- | --- | --- | --- | --- | --- | --- | --- | --- | --- | --- | --- | --- | --- | --- | --- | --- | --- | --- | --- | --- | --- | --- | --- | --- | --- | --- | --- | --- | --- | --- | --- | --- | --- | --- | --- | --- | --- | --- | --- | --- | --- | --- | --- | --- | --- | --- | --- | --- | --- | --- | --- | --- | --- | --- | --- | --- | --- | --- | --- | --- | --- | --- | --- | --- | --- | --- | --- | --- | --- | --- | --- | --- | --- | --- | --- | --- | --- | --- | --- | --- | --- | --- | --- | --- | --- | --- | --- | --- | --- | --- | --- | --- | --- | --- | --- | --- | --- | --- | --- | --- | --- | --- | --- | --- | --- | --- | --- | --- | --- | --- | --- | --- | --- | --- | --- | --- | --- | --- | --- | --- | --- | --- | --- | --- | --- | --- | --- | --- | --- | --- | --- | --- | --- | --- | --- | --- | --- | --- | --- | --- | --- | --- | --- | --- | --- | --- | --- | --- | --- | --- | --- | --- | --- | --- | --- | --- | --- | --- | --- | --- | --- | --- | --- | --- | --- | --- | --- | --- | --- | --- | --- | --- | --- | --- | --- | --- | --- | --- | --- | --- | --- | --- | --- | --- | --- | --- | --- | --- | --- | --- | --- | --- | --- | --- | --- | --- | --- | --- | --- | --- | --- | --- | --- | --- | --- | --- | --- | --- | --- | --- | --- | --- | --- | --- | --- | --- | --- | --- | --- | --- | --- | --- | --- | --- | --- | --- | --- | --- | --- | --- | --- | --- | --- | --- | --- | --- | --- | --- | --- | --- | --- | --- | --- | --- | --- | --- | --- | --- | --- | --- | --- | --- | --- | --- | --- | --- | --- | --- | --- | --- | --- | --- | --- | --- | --- | --- | --- | --- | --- | --- | --- | --- | --- | --- | --- | --- | --- | --- | --- | --- | --- | --- | --- | --- | --- | --- | --- | --- | --- | --- | --- | --- | --- | --- | --- | --- | --- | --- | --- | --- | --- | --- | --- | --- | --- | --- | --- | --- | --- | --- | --- | --- | --- | --- | --- | --- | --- | --- | --- | --- | --- | --- | --- | --- | --- | --- | --- | --- | --- | --- | --- | --- | --- | --- | --- | --- | --- | --- | --- | --- | --- | --- | --- | --- | --- | --- | --- | --- | --- | --- | --- | --- | --- | --- | --- | --- | --- | --- | --- | --- | --- | --- | --- | --- | --- | --- | --- | --- | --- | --- | --- | --- | --- | --- | --- | --- | --- | --- | --- | --- | --- | --- | --- | --- | --- | --- | --- | --- | --- | --- | --- | --- | --- | --- | --- | --- | --- | --- | --- | --- | --- | --- | --- | --- | --- | --- | --- | --- | --- | --- | --- | --- | --- | --- | --- | --- | --- | --- | --- | --- | --- | --- | --- | --- | --- | --- | --- | --- | --- | --- | --- | --- | --- | --- | --- | --- | --- | --- | --- | --- | --- | --- | --- | --- | --- | --- | --- | --- | --- | --- | --- | --- | --- | --- | --- | --- | --- | --- | --- | --- | --- | --- | --- | --- | --- | --- | --- | --- | --- | --- | --- | --- | --- | --- | --- | --- | --- | --- | --- | --- | --- | --- | --- | --- | --- | --- | --- | --- | --- | --- | --- | --- | --- | --- | --- | --- | --- | --- | --- | --- | --- | --- | --- | --- | --- | --- | --- | --- | --- | --- | --- | --- | --- | --- | --- | --- | --- | --- | --- | --- | --- | --- | --- | --- | --- | --- | --- | --- | --- | --- | --- | --- | --- | --- | --- | --- | --- | --- | --- | --- | --- | --- | --- | --- | --- | --- | --- | --- | --- | --- | --- | --- | --- | --- | --- | --- | --- | --- | --- | --- | --- | --- | --- | --- | --- | --- | --- | --- | --- | --- | --- | --- | --- | --- | --- | --- | --- | --- | --- | --- | --- | --- | --- | --- | --- | --- | --- | --- | --- | --- | --- | --- | --- | --- | --- | --- | --- | --- | --- | --- | --- | --- | --- | --- | --- | --- | --- | --- | --- | --- | --- | --- | --- | --- | --- | --- | --- | --- | --- | --- | --- | --- | --- | --- | --- | --- | --- | --- | --- | --- | --- | --- | --- | --- | --- | --- | --- | --- | --- | --- | --- | --- | --- | --- | --- | --- | --- | --- | --- | --- | --- | --- | --- | --- | --- | --- | --- | --- | --- | --- | --- | --- | --- | --- | --- | --- | --- | --- | --- | --- | --- | --- | --- | --- | --- | --- | --- | --- | --- | --- | --- | --- | --- | --- | --- | --- | --- | --- | --- | --- | --- | --- | --- | --- | --- | --- | --- | --- | --- | --- | --- | --- | --- | --- | --- | --- | --- | --- | --- | --- | --- | --- | --- | --- | --- | --- | --- | --- | --- | --- | --- | --- | --- | --- | --- | --- | --- | --- | --- | --- | --- | --- | --- | --- | --- | --- | --- | --- | --- | --- | --- | --- | --- | --- | --- | --- | --- | --- | --- | --- | --- | --- | --- | --- | --- | --- | --- | --- | --- | --- | --- | --- | --- | --- | --- | --- | --- | --- | --- | --- | --- | --- | --- | --- | --- | --- | --- | --- | --- | --- | --- | --- | --- | --- | --- | --- | --- | --- | --- | --- | --- | --- | --- | --- | --- | --- | --- | --- | --- | --- | --- | --- | --- | --- | --- | --- | --- | --- | --- | --- | --- | --- | --- | --- | --- | --- | --- | --- | --- | --- | --- | --- | --- | --- | --- | --- | --- | --- | --- | --- | --- | --- | --- | --- | --- | --- | --- | --- | --- | --- | --- | --- | --- | --- | --- | --- | --- | --- | --- | --- | --- | --- | --- | --- | --- | --- | --- | --- | --- | --- | --- | --- | --- | --- | --- | --- | --- | --- | --- | --- | --- | --- | --- | --- | --- | --- | --- | --- | --- | --- | --- | --- | --- | --- | --- | --- | --- | --- | --- | --- | --- | --- | --- | --- | --- | --- | --- | --- | --- | --- | --- | --- | --- | --- | --- | --- | --- | --- | --- | --- | --- | --- | --- | --- | --- | --- | --- | --- | --- | --- | --- | --- | --- | --- | --- | --- | --- | --- | --- | --- | --- | --- | --- | --- | --- | --- | --- | --- | --- | --- | --- | --- | --- | --- | --- | --- | --- | --- | --- | --- | --- | --- | --- | --- | --- | --- | --- | --- | --- | --- | --- | --- | --- | --- | --- | --- | --- | --- | --- | --- | --- | --- | --- | --- | --- | --- | --- | --- | --- | --- | --- | --- | --- | --- | --- | --- | --- | --- | --- | --- | --- | --- | --- | --- | --- | --- | --- | --- | --- | --- | --- | --- | --- | --- | --- | --- | --- | --- | --- | --- | --- | --- | --- | --- | --- | --- | --- | --- | --- | --- | --- | --- | --- | --- | --- | --- | --- | --- | --- | --- | --- | --- | --- | --- | --- | --- | --- | --- | --- | --- | --- | --- | --- | --- | --- | --- | --- | --- | --- | --- | --- | --- | --- | --- | --- | --- | --- | --- | --- | --- | --- | --- | --- | --- | --- | --- | --- | --- | --- | --- | --- | --- | --- | --- | --- | --- | --- | --- | --- | --- | --- | --- | --- | --- | --- | --- | --- | --- | --- | --- | --- | --- | --- | --- | --- | --- | --- | --- | --- | --- | --- | --- | --- | --- | --- | --- | --- | --- | --- | --- | --- | --- | --- | --- | --- | --- | --- | --- | --- | --- | --- | --- | --- | --- | --- | --- | --- | --- | --- | --- | --- | --- | --- | --- | --- | --- | --- | --- | --- | --- | --- | --- | --- | --- | --- | --- | --- | --- | --- | --- | --- | --- | --- | --- | --- | --- | --- | --- | --- | --- | --- | --- | --- | --- | --- | --- | --- | --- | --- | --- | --- | --- | --- | --- | --- | --- | --- | --- | --- | --- | --- | --- | --- | --- | --- | --- | --- | --- | --- | --- | --- | --- | --- | --- | --- | --- | --- | --- | --- | --- | --- | --- | --- | --- | --- | --- | --- | --- | --- | --- | --- | --- | --- | --- | --- | --- | --- | --- | --- | --- | --- | --- | --- | --- | --- | --- | --- | --- | --- | --- | --- | --- | --- | --- | --- | --- | --- | --- | --- | --- | --- | --- | --- | --- | --- | --- | --- | --- | --- | --- | --- | --- | --- | --- | --- | --- | --- | --- | --- | --- | --- | --- | --- | --- | --- | --- | --- | --- | --- | --- | --- | --- | --- | --- | --- | --- | --- | --- | --- | --- | --- | --- | --- | --- | --- | --- | --- | --- | --- | --- | --- | --- | --- | --- | --- | --- | --- | --- | --- | --- | --- | --- | --- | --- | --- | --- | --- | --- | --- | --- | --- | --- | --- | --- | --- | --- | --- | --- | --- | --- | --- | --- | --- | --- | --- | --- | --- | --- | --- | --- | --- | --- | --- | --- | --- | --- | --- | --- | --- | --- | --- | --- | --- | --- | --- | --- | --- | --- | --- | --- | --- | --- | --- | --- | --- | --- | --- | --- | --- | --- | --- | --- | --- | --- | --- | --- | --- | --- | --- | --- | --- | --- | --- | --- | --- | --- | --- | --- | --- | --- | --- | --- | --- | --- | --- | --- | --- | --- | --- | --- | --- | --- | --- | --- | --- | --- | --- | --- | --- | --- | --- | --- | --- | --- | --- | --- | --- | --- | --- | --- | --- | --- | --- | --- | --- | --- | --- | --- | --- | --- | --- | --- | --- | --- | --- | --- | --- | --- | --- | --- | --- | --- | --- | --- | --- | --- | --- | --- | --- | --- | --- | --- | --- | --- | --- | --- | --- | --- | --- | --- | --- | --- | --- | --- | --- | --- | --- | --- | --- | --- | --- | --- | --- | --- | --- | --- | --- | --- | --- | --- | --- | --- | --- | --- | --- | --- | --- | --- | --- | --- | --- | --- | --- | --- | --- | --- | --- | --- | --- | --- | --- | --- | --- | --- | --- | --- | --- | --- | --- | --- | --- | --- | --- | --- | --- | --- | --- | --- | --- | --- | --- | --- | --- | --- | --- | --- | --- | --- | --- | --- | --- | --- | --- | --- | --- | --- | --- | --- | --- | --- | --- | --- | --- | --- | --- | --- | --- | --- | --- | --- | --- | --- | --- | --- | --- | --- | --- | --- | --- | --- | --- | --- | --- | --- | --- | --- | --- | --- | --- | --- | --- | --- | --- | --- | --- | --- | --- | --- | --- | --- | --- | --- | --- | --- | --- | --- | --- | --- | --- | --- | --- | --- | --- | --- | --- | --- | --- | --- | --- | --- | --- | --- | --- | --- | --- | --- | --- | --- | --- | --- | --- | --- | --- | --- | --- | --- | --- | --- | --- | --- | --- | --- | --- | --- | --- | --- | --- | --- | --- | --- | --- | --- | --- | --- | --- | --- | --- | --- | --- | --- | --- | --- | --- | --- | --- | --- | --- | --- | --- | --- | --- | --- | --- | --- | --- | --- | --- | --- | --- | --- | --- | --- | --- | --- | --- | --- | --- | --- | --- | --- | --- | --- | --- | --- | --- | --- | --- | --- | --- | --- | --- | --- | --- | --- | --- | --- | --- | --- | --- | --- | --- | --- | --- | --- | --- | --- | --- | --- | --- | --- | --- | --- | --- | --- | --- | --- | --- | --- | --- | --- | --- | --- | --- | --- | --- | --- | --- | --- | --- | --- | --- | --- | --- | --- | --- | --- | --- | --- | --- | --- | --- | --- | --- | --- | --- | --- | --- | --- | --- | --- | --- | --- | --- | --- | --- | --- | --- | --- | --- | --- | --- | --- | --- | --- | --- | --- | --- | --- | --- | --- | --- | --- | --- | --- | --- | --- | --- | --- | --- | --- | --- | --- | --- | --- | --- | --- | --- | --- | --- | --- | --- | --- | --- | --- | --- | --- | --- | --- | --- | --- | --- | --- | --- | --- | --- | --- | --- | --- | --- | --- | --- | --- | --- | --- | --- | --- | --- | --- | --- | --- | --- | --- | --- | --- | --- | --- | --- | --- | --- | --- | --- | --- | --- | --- | --- | --- | --- | --- | --- | --- | --- | --- | --- | --- | --- | --- | --- | --- | --- | --- | --- | --- | --- | --- | --- | --- | --- | --- | --- | --- | --- | --- | --- | --- | --- | --- | --- | --- | --- | --- | --- | --- | --- | --- | --- | --- | --- | --- | --- | --- | --- | --- | --- | --- | --- | --- | --- | --- | --- | --- | --- | --- | --- | --- | --- | --- | --- | --- | --- | --- | --- | --- | --- | --- | --- | --- | --- | --- | --- | --- | --- | --- | --- | --- | --- | --- | --- | --- | --- | --- | --- | --- | --- | --- | --- | --- | --- | --- | --- | --- | --- | --- | --- | --- | --- | --- | --- | --- | --- | --- | --- | --- | --- | --- | --- | --- | --- | --- | --- | --- | --- | --- | --- | --- | --- | --- | --- | --- | --- | --- | --- | --- | --- | --- | --- | --- | --- | --- | --- | --- | --- | --- | --- | --- | --- | --- | --- | --- | --- | --- | --- | --- | --- | --- | --- | --- | --- | --- | --- | --- | --- | --- | --- | --- | --- | --- | --- | --- | --- | --- | --- | --- | --- | --- | --- | --- | --- | --- | --- | --- | --- | --- | --- | --- | --- | --- | --- | --- | --- | --- | --- | --- | --- | --- | --- | --- | --- | --- | --- | --- | --- | --- | --- | --- | --- | --- | --- | --- | --- | --- | --- | --- | --- | --- | --- | --- | --- | --- | --- | --- | --- | --- | --- | --- | --- | --- | --- | --- | --- | --- | --- | --- | --- | --- | --- | --- | --- | --- | --- | --- | --- | --- | --- | --- | --- | --- | --- | --- | --- | --- | --- | --- | --- | --- | --- | --- | --- | --- | --- | --- | --- | --- | --- | --- | --- | --- | --- | --- | --- | --- | --- | --- | --- | --- | --- | --- | --- | --- | --- | --- | --- | --- | --- | --- | --- | --- | --- | --- | --- | --- | --- | --- | --- | --- | --- | --- | --- | --- | --- | --- | --- | --- | --- | --- | --- | --- | --- | --- | --- | --- | --- | --- | --- | --- | --- | --- | --- | --- | --- | --- | --- | --- | --- | --- | --- | --- | --- | --- | --- | --- | --- | --- | --- | --- | --- | --- | --- | --- | --- | --- | --- | --- | --- | --- | --- | --- | --- | --- | --- | --- | --- | --- | --- | --- | --- | --- | --- | --- | --- | --- | --- | --- | --- | --- | --- | --- | --- | --- | --- | --- | --- | --- | --- | --- | --- | --- | --- | --- | --- | --- | --- | --- | --- | --- | --- | --- | --- | --- | --- | --- | --- | --- | --- | --- | --- | --- | --- | --- | --- | --- | --- | --- | --- | --- | --- | --- | --- | --- | --- | --- | --- | --- | --- | --- | --- | --- | --- | --- | --- | --- | --- | --- | --- | --- | --- | --- | --- | --- | --- | --- | --- | --- | --- | --- | --- | --- | --- | --- | --- | --- | --- | --- | --- | --- | --- | --- | --- | --- | --- | --- | --- | --- | --- | --- | --- | --- | --- | --- | --- | --- | --- | --- | --- | --- | --- | --- | --- | --- | --- | --- | --- | --- | --- | --- | --- | --- | --- | --- | --- | --- | --- | --- | --- | --- | --- | --- | --- | --- | --- | --- | --- | --- | --- | --- | --- | --- | --- | --- | --- | --- | --- | --- | --- | --- | --- | --- | --- | --- | --- | --- | --- | --- | --- | --- | --- | --- | --- | --- | --- | --- | --- | --- | --- | --- | --- | --- | --- | --- | --- | --- | --- | --- | --- | --- | --- | --- | --- | --- | --- | --- | --- | --- | --- | --- | --- | --- | --- | --- | --- | --- | --- | --- | --- | --- | --- | --- | --- | --- | --- | --- | --- | --- | --- | --- | --- | --- | --- | --- | --- | --- | --- | --- | --- | --- | --- | --- | --- | --- | --- | --- | --- | --- | --- | --- | --- | --- | --- | --- | --- | --- | --- | --- | --- | --- | --- | --- | --- | --- | --- | --- | --- | --- | --- | --- | --- | --- | --- | --- | --- | --- | --- | --- | --- | --- | --- | --- | --- | --- | --- | --- | --- | --- | --- | --- | --- | --- | --- | --- | --- | --- | --- | --- | --- | --- | --- | --- | --- | --- | --- | --- | --- | --- | --- | --- | --- | --- | --- | --- | --- | --- | --- | --- | --- | --- | --- | --- | --- | --- | --- | --- | --- | --- | --- | --- | --- | --- | --- | --- | --- | --- | --- | --- | --- | --- | --- | --- | --- | --- | --- | --- | --- | --- | --- | --- | --- | --- | --- | --- | --- | --- | --- | --- | --- | --- | --- | --- | --- | --- | --- | --- | --- | --- | --- | --- | --- | --- | --- | --- | --- | --- | --- | --- | --- | --- | --- | --- | --- | --- | --- | --- | --- | --- | --- | --- | --- | --- | --- | --- | --- | --- | --- | --- | --- | --- | --- | --- | --- | --- | --- | --- | --- | --- | --- | --- | --- | --- | --- | --- | --- | --- | --- | --- | --- | --- | --- | --- | --- | --- | --- | --- | --- | --- | --- | --- | --- | --- | --- | --- | --- | --- | --- | --- | --- | --- | --- | --- | --- | --- | --- | --- | --- | --- | --- | --- | --- | --- | --- | --- | --- | --- | --- | --- | --- | --- | --- | --- | --- | --- | --- | --- | --- | --- | --- | --- | --- | --- | --- | --- | --- | --- | --- | --- | --- | --- | --- | --- | --- | --- | --- | --- | --- | --- | --- | --- | --- | --- | --- | --- | --- | --- | --- | --- | --- | --- | --- | --- | --- | --- | --- | --- | --- | --- | --- | --- | --- | --- | --- | --- | --- | --- | --- | --- | --- | --- | --- | --- | --- | --- | --- | --- | --- | --- | --- | --- | --- | --- | --- | --- | --- | --- | --- | --- | --- | --- | --- | --- | --- | --- | --- | --- | --- | --- | --- | --- | --- | --- | --- | --- | --- | --- | --- | --- | --- | --- | --- | --- | --- | --- | --- | --- | --- | --- | --- | --- | --- | --- | --- | --- | --- | --- | --- | --- | --- | --- | --- | --- | --- | --- | --- | --- | --- | --- | --- | --- | --- | --- | --- | --- | --- | --- | --- | --- | --- | --- | --- | --- | --- | --- | --- | --- | --- | --- | --- | --- | --- | --- | --- | --- | --- | --- | --- | --- | --- | --- | --- | --- | --- | --- | --- | --- | --- | --- | --- | --- | --- | --- | --- | --- | --- | --- | --- | --- | --- | --- | --- | --- | --- | --- | --- | --- | --- | --- | --- | --- | --- | --- | --- | --- | --- | --- | --- | --- | --- | --- | --- | --- | --- | --- | --- | --- | --- | --- | --- | --- | --- | --- | --- | --- | --- | --- | --- | --- | --- | --- | --- | --- | --- | --- | --- | --- | --- | --- | --- | --- | --- | --- | --- | --- | --- | --- | --- | --- | --- | --- | --- | --- | --- | --- | --- | --- | --- | --- | --- | --- | --- | --- | --- | --- | --- | --- | --- | --- | --- | --- | --- | --- | --- | --- | --- | --- | --- | --- | --- | --- | --- | --- | --- | --- | --- | --- | --- | --- | --- | --- | --- | --- | --- | --- | --- | --- | --- | --- | --- | --- | --- | --- | --- | --- | --- | --- | --- | --- | --- | --- | --- | --- | --- | --- | --- | --- | --- | --- | --- | --- | --- | --- | --- | --- | --- | --- | --- | --- | --- | --- | --- | --- | --- | --- | --- | --- | --- | --- | --- | --- | --- | --- | --- | --- | --- | --- | --- | --- | --- | --- | --- | --- | --- | --- | --- | --- | --- | --- | --- | --- | --- | --- | --- | --- | --- | --- | --- | --- | --- | --- | --- | --- | --- | --- | --- | --- | --- | --- | --- | --- | --- | --- | --- | --- | --- | --- | --- | --- | --- | --- | --- | --- | --- | --- | --- | --- | --- | --- | --- | --- | --- | --- | --- | --- | --- | --- | --- | --- | --- | --- | --- | --- | --- | --- | --- | --- | --- | --- | --- | --- | --- | --- | --- | --- | --- | --- | --- | --- | --- | --- | --- | --- | --- | --- | --- | --- | --- | --- | --- | --- | --- | --- | --- | --- | --- | --- | --- | --- | --- | --- | --- | --- | --- | --- | --- | --- | --- | --- | --- | --- | --- | --- | --- | --- | --- | --- | --- | --- | --- | --- | --- | --- | --- | --- | --- | --- | --- | --- | --- | --- | --- | --- | --- | --- | --- | --- | --- | --- | --- | --- | --- | --- | --- | --- | --- | --- | --- | --- | --- | --- | --- | --- | --- | --- | --- | --- | --- | --- | --- | --- | --- | --- | --- | --- | --- | --- | --- | --- | --- | --- | --- | --- | --- | --- | --- | --- | --- | --- | --- | --- | --- | --- | --- | --- | --- | --- | --- | --- | --- | --- | --- | --- | --- | --- | --- | --- | --- | --- | --- | --- | --- | --- | --- | --- | --- | --- | --- | --- | --- | --- | --- | --- | --- | --- | --- | --- | --- | --- | --- | --- | --- | --- | --- | --- | --- | --- | --- | --- | --- | --- | --- | --- | --- | --- | --- | --- | --- | --- | --- | --- | --- | --- | --- | --- | --- | --- | --- | --- | --- | --- | --- | --- | --- | --- | --- | --- | --- | --- | --- | --- | --- | --- | --- | --- | --- | --- | --- | --- | --- | --- | --- | --- | --- | --- | --- | --- | --- | --- | --- | --- | --- | --- | --- | --- | --- | --- | --- | --- | --- | --- | --- | --- | --- | --- | --- | --- | --- | --- | --- | --- | --- | --- | --- | --- | --- | --- | --- | --- | --- | --- | --- | --- | --- | --- | --- | --- | --- | --- | --- | --- | --- | --- | --- | --- | --- | --- | --- | --- | --- | --- | --- | --- | --- | --- | --- | --- | --- | --- | --- | --- | --- | --- | --- | --- | --- | --- | --- | --- | --- | --- | --- | --- | --- | --- | --- | --- | --- | --- | --- | --- | --- | --- | --- | --- | --- | --- | --- | --- | --- | --- | --- | --- | --- | --- | --- | --- | --- | --- | --- | --- | --- | --- | --- | --- | --- | --- | --- | --- | --- | --- | --- | --- | --- | --- | --- | --- | --- | --- | --- | --- | --- | --- | --- | --- | --- | --- | --- | --- | --- | --- | --- | --- | --- | --- | --- | --- | --- | --- | --- | --- | --- | --- | --- | --- | --- | --- | --- | --- | --- | --- | --- | --- | --- | --- | --- | --- | --- | --- | --- | --- | --- | --- | --- | --- | --- | --- | --- | --- | --- | --- | --- | --- | --- | --- | --- | --- | --- | --- | --- | --- | --- | --- | --- | --- | --- | --- | --- | --- | --- | --- | --- | --- | --- | --- | --- | --- | --- | --- | --- | --- | --- | --- | --- | --- | --- | --- | --- | --- | --- | --- | --- | --- | --- | --- | --- | --- | --- | --- | --- | --- | --- | --- | --- | --- | --- | --- | --- | --- | --- | --- | --- | --- | --- | --- | --- | --- | --- | --- | --- | --- | --- | --- | --- | --- | --- | --- | --- | --- | --- | --- | --- | --- | --- | --- | --- | --- | --- | --- | --- | --- | --- | --- | --- | --- | --- | --- | --- | --- | --- | --- | --- | --- | --- | --- | --- | --- | --- | --- | --- | --- | --- | --- | --- | --- | --- | --- | --- | --- | --- | --- | --- | --- | --- | --- | --- | --- | --- | --- | --- | --- | --- | --- | --- | --- | --- | --- | --- | --- | --- | --- | --- | --- | --- | --- | --- | --- | --- | --- | --- | --- | --- | --- | --- | --- | --- | --- | --- | --- | --- | --- | --- | --- | --- | --- | --- | --- | --- | --- | --- | --- | --- | --- | --- | --- | --- | --- | --- | --- | --- | --- | --- | --- | --- | --- | --- | --- | --- | --- | --- | --- | --- | --- | --- | --- | --- | --- | --- | --- | --- | --- | --- | --- | --- | --- | --- | --- | --- | --- | --- | --- | --- | --- | --- | --- | --- | --- | --- | --- | --- | --- | --- | --- | --- | --- | --- | --- | --- | --- | --- | --- | --- | --- | --- | --- | --- | --- | --- | --- | --- | --- | --- | --- | --- | --- | --- | --- | --- | --- | --- | --- | --- | --- | --- | --- | --- | --- | --- | --- | --- | --- | --- | --- | --- | --- | --- | --- | --- | --- | --- | --- | --- | --- | --- | --- | --- | --- | --- | --- | --- | --- | --- | --- | --- | --- | --- | --- | --- | --- | --- | --- | --- | --- | --- | --- | --- | --- | --- | --- | --- | --- | --- | --- | --- | --- | --- | --- | --- | --- | --- | --- | --- | --- | --- | --- | --- | --- | --- | --- | --- | --- | --- | --- | --- | --- | --- | --- | --- | --- | --- | --- | --- | --- | --- | --- | --- | --- | --- | --- | --- | --- | --- | --- | --- | --- | --- | --- | --- | --- | --- | --- | --- | --- | --- | --- | --- | --- | --- | --- | --- | --- | --- | --- | --- | --- | --- | --- | --- | --- | --- | --- | --- | --- | --- | --- | --- | --- | --- | --- | --- | --- | --- | --- | --- | --- | --- | --- | --- | --- | --- | --- | --- | --- | --- | --- | --- | --- | --- | --- | --- | --- | --- | --- | --- | --- | --- | --- | --- | --- | --- | --- | --- | --- | --- | --- | --- | --- | --- | --- | --- | --- | --- | --- | --- | --- | --- | --- | --- | --- | --- | --- | --- | --- | --- | --- | --- | --- | --- | --- | --- | --- | --- | --- | --- | --- | --- | --- | --- | --- | --- | --- | --- | --- | --- | --- | --- | --- | --- | --- | --- | --- | --- | --- | --- | --- | --- | --- | --- | --- | --- | --- | --- | --- | --- | --- | --- | --- | --- | --- | --- | --- | --- | --- | --- | --- | --- | --- | --- | --- | --- | --- | --- | --- | --- | --- | --- | --- | --- | --- | --- | --- | --- | --- | --- | --- | --- | --- | --- | --- | --- | --- | --- | --- | --- | --- | --- | --- | --- | --- | --- | --- | --- | --- | --- | --- | --- | --- | --- | --- | --- | --- | --- | --- | --- | --- | --- | --- | --- | --- | --- | --- | --- | --- | --- | --- | --- | --- | --- | --- | --- | --- | --- | --- | --- | --- | --- | --- | --- | --- | --- | --- | --- | --- | --- | --- | --- | --- | --- | --- | --- | --- | --- | --- | --- | --- | --- | --- | --- | --- | --- | --- | --- | --- | --- | --- | --- | --- | --- | --- | --- | --- | --- | --- | --- | --- | --- | --- | --- | --- | --- | --- | --- | --- | --- | --- | --- | --- | --- | --- | --- | --- | --- | --- | --- | --- | --- | --- | --- | --- | --- | --- | --- | --- | --- | --- | --- | --- | --- | --- | --- | --- | --- | --- | --- | --- | --- | --- | --- | --- | --- | --- | --- | --- | --- | --- | --- | --- | --- | --- | --- | --- | --- | --- | --- | --- | --- | --- | --- | --- | --- | --- | --- | --- | --- | --- | --- | --- | --- | --- | --- | --- | --- | --- | --- | --- | --- | --- | --- | --- | --- | --- | --- | --- | --- | --- | --- | --- | --- | --- | --- | --- | --- | --- | --- | --- | --- | --- | --- | --- | --- | --- | --- | --- | --- | --- | --- | --- | --- | --- | --- | --- | --- | --- | --- | --- | --- | --- | --- | --- | --- | --- | --- | --- | --- | --- | --- | --- | --- | --- | --- | --- | --- | --- | --- | --- | --- | --- | --- | --- | --- | --- | --- | --- | --- | --- | --- | --- | --- | --- | --- | --- | --- | --- | --- | --- | --- | --- | --- | --- | --- | --- | --- | --- | --- | --- | --- | --- | --- | --- | --- | --- | --- | --- | --- | --- | --- | --- | --- | --- | --- | --- | --- | --- | --- | --- | --- | --- | --- | --- | --- | --- | --- | --- | --- | --- | --- | --- | --- | --- | --- | --- | --- | --- | --- | --- | --- | --- | --- | --- | --- | --- | --- | --- | --- | --- | --- | --- | --- | --- | --- | --- | --- | --- | --- | --- | --- | --- | --- | --- | --- | --- | --- | --- | --- | --- | --- | --- | --- | --- | --- | --- | --- | --- | --- | --- | --- | --- | --- | --- | --- | --- | --- | --- | --- | --- | --- | --- | --- | --- | --- | --- | --- | --- | --- | --- | --- | --- | --- | --- | --- | --- | --- | --- | --- | --- | --- | --- | --- | --- | --- | --- | --- | --- | --- | --- | --- | --- | --- | --- | --- | --- | --- | --- | --- | --- | --- | --- | --- | --- | --- | --- | --- | --- | --- | --- | --- | --- | --- | --- | --- | --- | --- | --- | --- | --- | --- | --- | --- | --- | --- | --- | --- | --- | --- | --- | --- | --- | --- | --- | --- | --- | --- | --- | --- | --- | --- | --- | --- | --- | --- | --- | --- | --- | --- | --- | --- | --- | --- | --- | --- | --- | --- | --- | --- | --- | --- | --- | --- | --- | --- | --- | --- | --- | --- | --- | --- | --- | --- | --- | --- | --- | --- | --- | --- | --- | --- | --- | --- | --- | --- | --- | --- | --- | --- | --- | --- | --- | --- | --- | --- | --- | --- | --- | --- | --- | --- | --- | --- | --- | --- | --- | --- | --- | --- | --- | --- | --- | --- | --- | --- | --- | --- | --- | --- | --- | --- | --- | --- | --- | --- | --- | --- | --- | --- | --- | --- | --- | --- | --- | --- | --- | --- | --- | --- | --- | --- | --- | --- | --- | --- | --- | --- | --- | --- | --- | --- | --- | --- | --- | --- | --- | --- | --- | --- | --- | --- | --- | --- | --- | --- | --- | --- | --- | --- | --- | --- | --- | --- | --- | --- | --- | --- | --- | --- | --- | --- | --- | --- | --- | --- | --- | --- | --- | --- | --- | --- | --- | --- | --- | --- | --- | --- | --- | --- | --- | --- | --- | --- | --- | --- | --- | --- | --- | --- | --- | --- | --- | --- | --- | --- | --- | --- | --- | --- | --- | --- | --- | --- | --- | --- | --- | --- | --- | --- | --- | --- | --- | --- | --- | --- | --- | --- | --- | --- | --- | --- | --- | --- | --- | --- | --- | --- | --- | --- | --- | --- | --- | --- | --- | --- | --- | --- | --- | --- | --- | --- | --- | --- | --- | --- | --- | --- | --- | --- | --- | --- | --- | --- | --- | --- | --- | --- | --- | --- | --- | --- | --- | --- | --- | --- | --- | --- | --- | --- | --- | --- | --- | --- | --- | --- | --- | --- | --- | --- | --- | --- | --- | --- | --- | --- | --- | --- | --- | --- | --- | --- | --- | --- | --- | --- | --- | --- | --- | --- | --- | --- | --- | --- | --- | --- | --- | --- | --- | --- | --- | --- | --- | --- | --- | --- | --- | --- | --- | --- | --- | --- | --- | --- | --- | --- | --- | --- | --- | --- | --- | --- | --- | --- | --- | --- | --- | --- | --- | --- | --- | --- | --- | --- | --- | --- | --- | --- | --- | --- | --- | --- | --- | --- | --- | --- | --- | --- | --- | --- | --- | --- | --- | --- | --- | --- | --- | --- | --- | --- | --- | --- | --- | --- | --- | --- | --- | --- | --- | --- | --- | --- | --- | --- | --- | --- | --- | --- | --- | --- | --- | --- | --- | --- | --- | --- | --- | --- | --- | --- | --- | --- | --- | --- | --- | --- | --- | --- | --- | --- | --- | --- | --- | --- | --- | --- | --- | --- | --- | --- | --- | --- | --- | --- | --- | --- | --- | --- | --- | --- | --- | --- | --- | --- | --- | --- | --- | --- | --- | --- | --- | --- | --- | --- | --- | --- | --- | --- | --- | --- | --- | --- | --- | --- | --- | --- | --- | --- | --- | --- | --- | --- | --- | --- | --- | --- | --- | --- | --- | --- | --- | --- | --- | --- | --- | --- | --- | --- | --- | --- | --- | --- | --- | --- | --- | --- | --- | --- | --- | --- | --- | --- | --- | --- | --- | --- | --- | --- | --- | --- | --- | --- | --- | --- | --- | --- | --- | --- | --- | --- | --- | --- | --- | --- | --- | --- | --- | --- | --- | --- | --- | --- | --- | --- | --- | --- | --- | --- | --- | --- | --- | --- | --- | --- | --- | --- | --- | --- | --- | --- | --- | --- | --- | --- | --- | --- | --- | --- | --- | --- | --- | --- | --- | --- | --- | --- | --- | --- | --- | --- | --- | --- | --- | --- | --- | --- | --- | --- | --- | --- | --- | --- | --- | --- | --- | --- | --- | --- | --- | --- | --- | --- | --- | --- | --- | --- | --- | --- | --- | --- | --- | --- | --- | --- | --- | --- | --- | --- | --- | --- | --- | --- | --- | --- | --- | --- | --- | --- | --- | --- | --- | --- | --- | --- | --- | --- | --- | --- | --- | --- | --- | --- | --- | --- | --- | --- | --- | --- | --- | --- | --- | --- | --- | --- | --- | --- | --- | --- | --- | --- | --- | --- | --- | --- | --- | --- | --- | --- | --- | --- | --- | --- | --- | --- | --- | --- | --- | --- | --- | --- | --- | --- | --- | --- | --- | --- | --- | --- | --- | --- | --- | --- | --- | --- | --- | --- | --- | --- | --- | --- | --- | --- | --- | --- | --- | --- | --- | --- | --- | --- | --- | --- | --- | --- | --- | --- | --- | --- | --- | --- | --- | --- | --- | --- | --- | --- | --- | --- | --- | --- | --- | --- | --- | --- | --- | --- | --- | --- | --- | --- | --- | --- | --- | --- | --- | --- | --- | --- | --- | --- | --- | --- | --- | --- | --- | --- | --- | --- | --- | --- | --- | --- | --- | --- | --- | --- | --- | --- | --- | --- | --- | --- | --- | --- | --- | --- | --- | --- | --- | --- | --- | --- | --- | --- | --- | --- | --- | --- | --- | --- | --- | --- | --- | --- | --- | --- | --- | --- | --- | --- | --- | --- | --- | --- | --- | --- | --- | --- | --- | --- | --- | --- | --- | --- | --- | --- | --- | --- | --- | --- | --- | --- | --- | --- | --- | --- | --- | --- | --- | --- | --- | --- | --- | --- | --- | --- | --- | --- | --- | --- | --- | --- | --- | --- | --- | --- | --- | --- | --- | --- | --- | --- | --- | --- | --- | --- | --- | --- | --- | --- | --- | --- | --- | --- | --- | --- | --- | --- | --- | --- | --- | --- | --- | --- | --- | --- | --- | --- | --- | --- | --- | --- | --- | --- | --- | --- | --- | --- | --- | --- | --- | --- | --- | --- | --- | --- | --- | --- | --- | --- | --- | --- | --- | --- | --- | --- | --- | --- | --- | --- | --- | --- | --- | --- | --- | --- | --- | --- | --- | --- | --- | --- | --- | --- | --- | --- | --- | --- | --- | --- | --- | --- | --- | --- | --- | --- | --- | --- | --- | --- | --- | --- | --- | --- | --- | --- | --- | --- | --- | --- | --- | --- | --- | --- | --- | --- | --- | --- | --- | --- | --- | --- | --- | --- | --- | --- | --- | --- | --- | --- | --- | --- | --- | --- | --- | --- | --- | --- | --- | --- | --- | --- | --- | --- | --- | --- | --- | --- | --- | --- | --- | --- | --- | --- | --- | --- | --- | --- | --- | --- | --- | --- | --- | --- | --- | --- | --- | --- | --- | --- | --- | --- | --- | --- | --- | --- | --- | --- | --- | --- | --- | --- | --- | --- | --- | --- | --- | --- | --- | --- | --- | --- | --- | --- | --- | --- | --- | --- | --- | --- | --- | --- | --- | --- | --- | --- | --- | --- | --- | --- | --- | --- | --- | --- | --- | --- | --- | --- | --- | --- | --- | --- | --- | --- | --- | --- | --- | --- | --- | --- | --- | --- | --- | --- | --- | --- | --- | --- | --- | --- | --- | --- | --- | --- | --- | --- | --- | --- | --- | --- | --- | --- | --- | --- | --- | --- | --- | --- | --- | --- | --- | --- | --- | --- | --- | --- | --- | --- | --- | --- | --- | --- | --- | --- | --- | --- | --- | --- | --- | --- | --- | --- | --- | --- | --- | --- | --- | --- | --- | --- | --- | --- | --- | --- | --- | --- | --- | --- | --- | --- | --- | --- | --- | --- | --- | --- | --- | --- | --- | --- | --- | --- | --- | --- | --- | --- | --- | --- | --- | --- | --- | --- | --- | --- | --- | --- | --- | --- | --- | --- | --- | --- | --- | --- | --- | --- | --- | --- | --- | --- | --- | --- | --- | --- | --- | --- | --- | --- | --- | --- | --- | --- | --- | --- | --- | --- | --- | --- | --- | --- | --- | --- | --- | --- | --- | --- | --- | --- | --- | --- | --- | --- | --- | --- | --- | --- | --- | --- | --- | --- | --- | --- | --- | --- | --- | --- | --- | --- | --- | --- | --- | --- | --- | --- | --- | --- | --- | --- | --- | --- | --- | --- | --- | --- | --- | --- | --- | --- | --- | --- | --- | --- | --- | --- | --- | --- | --- | --- | --- | --- | --- | --- | --- | --- | --- | --- | --- | --- | --- | --- | --- | --- | --- | --- | --- | --- | --- | --- | --- | --- | --- | --- | --- | --- | --- | --- | --- | --- | --- | --- | --- | --- | --- | --- | --- | --- | --- | --- | --- | --- | --- | --- | --- | --- | --- | --- | --- | --- | --- | --- | --- | --- | --- | --- | --- | --- | --- | --- | --- | --- | --- | --- | --- | --- | --- | --- | --- | --- | --- | --- | --- | --- | --- | --- | --- | --- | --- | --- | --- | --- | --- | --- | --- | --- | --- | --- | --- | --- | --- | --- | --- | --- | --- | --- | --- | --- | --- | --- | --- | --- | --- | --- | --- | --- | --- | --- | --- | --- | --- | --- | --- | --- | --- | --- | --- | --- | --- | --- | --- | --- | --- | --- | --- | --- | --- | --- | --- | --- | --- | --- | --- | --- | --- | --- | --- | --- | --- | --- | --- | --- | --- | --- | --- | --- | --- | --- | --- | --- | --- | --- | --- | --- | --- | --- | --- | --- | --- | --- | --- | --- | --- | --- | --- | --- | --- | --- | --- | --- | --- | --- | --- | --- | --- | --- | --- | --- | --- | --- | --- | --- | --- | --- | --- | --- | --- | --- | --- | --- | --- | --- | --- | --- | --- | --- | --- | --- | --- | --- | --- | --- | --- | --- | --- | --- | --- | --- | --- | --- | --- | --- | --- | --- | --- | --- | --- | --- | --- | --- | --- | --- | --- | --- | --- | --- | --- | --- | --- | --- | --- | --- | --- | --- | --- | --- | --- | --- | --- | --- | --- | --- | --- | --- | --- | --- | --- | --- | --- | --- | --- | --- | --- | --- | --- | --- | --- | --- | --- | --- | --- | --- | --- | --- | --- | --- | --- | --- | --- | --- | --- | --- | --- | --- | --- | --- | --- | --- | --- | --- | --- | --- | --- | --- | --- | --- | --- | --- | --- | --- | --- | --- | --- | --- | --- | --- | --- | --- | --- | --- | --- | --- | --- | --- | --- | --- | --- | --- | --- | --- | --- | --- | --- | --- | --- | --- | --- | --- | --- | --- | --- | --- | --- | --- | --- | --- | --- | --- | --- | --- | --- | --- | --- | --- | --- | --- | --- | --- | --- | --- | --- | --- | --- | --- | --- | --- | --- | --- | --- | --- | --- | --- | --- | --- | --- | --- | --- | --- | --- | --- | --- | --- | --- | --- | --- | --- | --- | --- | --- | --- | --- | --- | --- | --- | --- | --- | --- | --- | --- | --- | --- | --- | --- | --- | --- | --- | --- | --- | --- | --- | --- | --- | --- | --- | --- | --- | --- | --- | --- | --- | --- | --- | --- | --- | --- | --- | --- | --- | --- | --- | --- | --- | --- | --- | --- | --- | --- | --- | --- | --- | --- | --- | --- | --- | --- | --- | --- | --- | --- | --- | --- | --- | --- | --- | --- | --- | --- | --- | --- | --- | --- | --- | --- | --- | --- | --- | --- | --- | --- | --- | --- | --- | --- | --- | --- | --- | --- | --- | --- | --- | --- | --- | --- | --- | --- | --- | --- | --- | --- | --- | --- | --- | --- | --- | --- | --- | --- | --- | --- | --- | --- | --- | --- | --- | --- | --- | --- | --- | --- | --- | --- | --- | --- | --- | --- | --- | --- | --- | --- | --- | --- | --- | --- | --- | --- | --- | --- | --- | --- | --- | --- | --- | --- | --- | --- | --- | --- | --- | --- | --- | --- | --- | --- | --- | --- | --- | --- | --- | --- | --- | --- | --- | --- | --- | --- | --- | --- | --- | --- | --- | --- | --- | --- | --- | --- | --- | --- | --- | --- | --- | --- | --- | --- | --- | --- | --- | --- | --- | --- | --- | --- | --- | --- | --- | --- | --- | --- | --- | --- | --- | --- | --- | --- | --- | --- | --- | --- | --- | --- | --- | --- | --- | --- | --- | --- | --- | --- | --- | --- | --- | --- | --- | --- | --- | --- | --- | --- | --- | --- | --- | --- | --- | --- | --- | --- | --- | --- | --- | --- | --- | --- | --- | --- | --- | --- | --- | --- | --- | --- | --- | --- | --- | --- | --- | --- | --- | --- | --- | --- | --- | --- | --- | --- | --- | --- | --- | --- | --- | --- | --- | --- | --- | --- | --- | --- | --- | --- | --- | --- | --- | --- | --- | --- | --- | --- | --- | --- | --- | --- | --- | --- | --- | --- | --- | --- | --- | --- | --- | --- | --- | --- | --- | --- | --- | --- | --- | --- | --- | --- | --- | --- | --- | --- | --- | --- | --- | --- | --- | --- | --- | --- | --- | --- | --- | --- | --- | --- | --- | --- | --- | --- | --- | --- | --- | --- | --- | --- | --- | --- | --- | --- | --- | --- | --- | --- | --- | --- | --- | --- | --- | --- | --- | --- | --- | --- | --- | --- | --- | --- | --- | --- | --- | --- | --- | --- | --- | --- | --- | --- | --- | --- | --- | --- | --- | --- | --- | --- | --- | --- | --- | --- | --- | --- | --- | --- | --- | --- | --- | --- | --- | --- | --- | --- | --- | --- | --- | --- | --- | --- | --- | --- | --- | --- | --- | --- | --- | --- | --- | --- | --- | --- | --- | --- | --- | --- | --- | --- | --- | --- | --- | --- | --- | --- | --- | --- | --- | --- | --- | --- | --- | --- | --- | --- | --- | --- | --- | --- | --- | --- | --- | --- | --- | --- | --- | --- | --- | --- | --- | --- | --- | --- | --- | --- | --- | --- | --- | --- | --- | --- | --- | --- | --- | --- | --- | --- | --- | --- | --- | --- | --- | --- | --- | --- | --- | --- | --- | --- | --- | --- | --- | --- | --- | --- | --- | --- | --- | --- | --- | --- | --- | --- | --- | --- | --- | --- | --- | --- | --- | --- | --- | --- | --- | --- | --- | --- | --- | --- | --- | --- | --- | --- | --- | --- | --- | --- | --- | --- | --- | --- | --- | --- | --- | --- | --- | --- | --- | --- | --- | --- | --- | --- | --- | --- | --- | --- | --- | --- | --- | --- | --- | --- | --- | --- | --- | --- | --- | --- | --- | --- | --- | --- | --- | --- | --- | --- | --- | --- | --- | --- | --- | --- | --- | --- | --- | --- | --- | --- | --- | --- | --- | --- | --- | --- | --- | --- | --- | --- | --- | --- | --- | --- | --- | --- | --- | --- | --- | --- | --- | --- | --- | --- | --- | --- | --- | --- | --- | --- | --- | --- | --- | --- | --- | --- | --- | --- | --- | --- | --- | --- | --- | --- | --- | --- | --- | --- | --- | --- | --- | --- | --- | --- | --- | --- | --- | --- | --- | --- | --- | --- | --- | --- | --- | --- | --- | --- | --- | --- | --- | --- | --- | --- | --- | --- | --- | --- | --- | --- | --- | --- | --- | --- | --- | --- | --- | --- | --- | --- | --- | --- | --- | --- | --- | --- | --- | --- | --- | --- | --- | --- | --- | --- | --- | --- | --- | --- | --- | --- | --- | --- | --- | --- | --- | --- | --- | --- | --- | --- | --- | --- | --- | --- | --- | --- | --- | --- | --- | --- | --- | --- | --- | --- | --- | --- | --- | --- | --- | --- | --- | --- | --- | --- | --- | --- | --- | --- | --- | --- | --- | --- | --- | --- | --- | --- | --- | --- | --- | --- | --- | --- | --- | --- | --- | --- | --- | --- | --- | --- | --- | --- | --- | --- | --- | --- | --- | --- | --- | --- | --- | --- | --- | --- | --- | --- | --- | --- | --- | --- | --- | --- | --- | --- | --- | --- | --- | --- | --- | --- | --- | --- | --- | --- | --- | --- | --- | --- | --- | --- | --- | --- | --- | --- | --- | --- | --- | --- | --- | --- | --- | --- | --- | --- | --- | --- | --- | --- | --- | --- | --- | --- | --- | --- | --- | --- | --- | --- | --- | --- | --- | --- | --- | --- | --- | --- | --- | --- | --- | --- | --- | --- | --- | --- | --- | --- | --- | --- | --- | --- | --- | --- | --- | --- | --- | --- | --- | --- | --- | --- | --- | --- | --- | --- | --- | --- | --- | --- | --- | --- | --- | --- | --- | --- | --- | --- | --- | --- | --- | --- | --- | --- | --- | --- | --- | --- | --- | --- | --- | --- | --- | --- | --- | --- | --- | --- | --- | --- | --- | --- | --- | --- | --- | --- | --- | --- | --- | --- | --- | --- | --- | --- | --- | --- | --- | --- | --- | --- | --- | --- | --- | --- | --- | --- | --- | --- | --- | --- | --- | --- | --- | --- | --- | --- | --- | --- | --- | --- | --- | --- | --- | --- | --- | --- | --- | --- | --- | --- | --- | --- | --- | --- | --- | --- | --- | --- | --- | --- | --- | --- | --- | --- | --- | --- | --- | --- | --- | --- | --- | --- | --- | --- | --- | --- | --- | --- | --- | --- | --- | --- | --- | --- | --- | --- | --- | --- | --- | --- | --- | --- | --- | --- | --- | --- | --- | --- | --- | --- | --- | --- | --- | --- | --- | --- | --- | --- | --- | --- | --- | --- | --- | --- | --- | --- | --- | --- | --- | --- | --- | --- | --- | --- | --- | --- | --- | --- | --- | --- | --- | --- | --- | --- | --- | --- | --- | --- | --- | --- | --- | --- | --- | --- | --- | --- | --- | --- | --- | --- | --- | --- | --- | --- | --- | --- | --- | --- | --- | --- | --- | --- | --- | --- | --- | --- | --- | --- | --- | --- | --- | --- | --- | --- | --- | --- | --- | --- | --- | --- | --- | --- | --- | --- | --- | --- | --- | --- | --- | --- | --- | --- | --- | --- | --- | --- | --- | --- | --- | --- | --- | --- | --- | --- | --- | --- | --- | --- | --- | --- | --- | --- | --- | --- | --- | --- | --- | --- | --- | --- | --- | --- | --- | --- | --- | --- | --- | --- | --- | --- | --- | --- | --- | --- | --- | --- | --- | --- | --- | --- | --- | --- | --- | --- | --- | --- | --- | --- | --- | --- | --- | --- | --- | --- | --- | --- | --- | --- | --- | --- | --- | --- | --- | --- | --- | --- | --- | --- | --- | --- | --- | --- | --- | --- | --- | --- | --- | --- | --- | --- | --- | --- | --- | --- | --- | --- | --- | --- | --- | --- | --- | --- | --- | --- | --- | --- | --- | --- | --- | --- | --- | --- | --- | --- | --- | --- | --- | --- | --- | --- | --- | --- | --- | --- | --- | --- | --- | --- | --- | --- | --- | --- | --- | --- | --- | --- | --- | --- | --- | --- | --- | --- | --- | --- | --- | --- | --- | --- | --- | --- | --- | --- | --- | --- | --- | --- | --- | --- | --- | --- | --- | --- | --- | --- | --- | --- | --- | --- | --- | --- | --- | --- | --- | --- | --- | --- | --- | --- | --- | --- | --- | --- | --- | --- | --- | --- | --- | --- | --- | --- | --- | --- | --- | --- | --- | --- | --- | --- | --- | --- | --- | --- | --- | --- | --- | --- | --- | --- | --- | --- | --- | --- | --- | --- | --- | --- | --- | --- | --- | --- | --- | --- | --- | --- | --- | --- | --- | --- | --- | --- | --- | --- | --- | --- | --- | --- | --- | --- | --- | --- | --- | --- | --- | --- | --- | --- | --- | --- | --- | --- | --- | --- | --- | --- | --- | --- | --- | --- | --- | --- | --- | --- | --- | --- | --- | --- | --- | --- | --- | --- | --- | --- | --- | --- | --- | --- | --- | --- | --- | --- | --- | --- | --- | --- | --- | --- | --- | --- | --- | --- | --- | --- | --- | --- | --- | --- | --- | --- | --- | --- | --- | --- | --- | --- | --- | --- | --- | --- | --- | --- | --- | --- | --- | --- | --- | --- | --- | --- | --- | --- | --- | --- | --- | --- | --- | --- | --- | --- | --- | --- | --- | --- | --- | --- | --- | --- | --- | --- | --- | --- | --- | --- | --- | --- | --- | --- | --- | --- | --- | --- | --- | --- | --- | --- | --- | --- | --- | --- | --- | --- | --- | --- | --- | --- | --- | --- | --- | --- | --- | --- | --- | --- | --- | --- | --- | --- | --- | --- | --- | --- | --- | --- | --- | --- | --- | --- | --- | --- | --- | --- | --- | --- | --- | --- | --- | --- | --- | --- | --- | --- | --- | --- | --- | --- | --- | --- | --- | --- | --- | --- | --- | --- | --- | --- | --- | --- | --- | --- | --- | --- | --- | --- | --- | --- | --- | --- | --- | --- | --- | --- | --- | --- | --- | --- | --- | --- | --- | --- | --- | --- | --- | --- | --- | --- | --- | --- | --- | --- | --- | --- | --- | --- | --- | --- | --- | --- | --- | --- | --- | --- | --- | --- | --- | --- | --- | --- | --- | --- | --- | --- | --- | --- | --- | --- | --- | --- | --- | --- | --- | --- | --- | --- | --- | --- | --- | --- | --- | --- | --- | --- | --- | --- | --- | --- | --- | --- | --- | --- | --- | --- | --- | --- | --- | --- | --- | --- | --- | --- | --- | --- | --- | --- | --- | --- | --- | --- | --- | --- | --- | --- | --- | --- | --- | --- | --- | --- | --- | --- | --- | --- | --- | --- | --- | --- | --- | --- | --- | --- | --- | --- | --- | --- | --- | --- | --- | --- | --- | --- | --- | --- | --- | --- | --- | --- | --- | --- | --- | --- | --- | --- | --- | --- | --- | --- | --- | --- | --- | --- | --- | --- | --- | --- | --- | --- | --- | --- | --- | --- | --- | --- | --- | --- | --- | --- | --- | --- | --- | --- | --- | --- | --- | --- | --- | --- | --- | --- | --- | --- | --- | --- | --- | --- | --- | --- | --- | --- | --- | --- | --- | --- | --- | --- | --- | --- | --- | --- | --- | --- | --- | --- | --- | --- | --- | --- | --- | --- | --- | --- | --- | --- | --- | --- | --- | --- | --- | --- | --- | --- | --- | --- | --- | --- | --- | --- | --- | --- | --- | --- | --- | --- | --- | --- | --- | --- | --- | --- | --- | --- | --- | --- | --- | --- | --- | --- | --- | --- | --- | --- | --- | --- | --- | --- | --- | --- | --- | --- | --- | --- | --- | --- | --- | --- | --- | --- | --- | --- | --- | --- | --- | --- | --- | --- | --- | --- | --- | --- | --- | --- | --- | --- | --- | --- | --- | --- | --- | --- | --- | --- | --- | --- | --- | --- | --- | --- | --- | --- | --- | --- | --- | --- | --- | --- | --- | --- | --- | --- | --- | --- | --- | --- | --- | --- | --- | --- | --- | --- | --- | --- | --- | --- | --- | --- | --- | --- | --- | --- | --- | --- | --- | --- | --- | --- | --- | --- | --- | --- | --- | --- | --- | --- | --- | --- | --- | --- | --- | --- | --- | --- | --- | --- | --- | --- | --- | --- | --- | --- | --- | --- | --- | --- | --- | --- | --- | --- | --- | --- | --- | --- | --- | --- | --- | --- | --- | --- | --- | --- | --- | --- | --- | --- | --- | --- | --- | --- | --- | --- | --- | --- | --- | --- | --- | --- | --- | --- | --- | --- | --- | --- | --- | --- | --- | --- | --- | --- | --- | --- | --- | --- | --- | --- | --- | --- | --- | --- | --- | --- | --- | --- | --- | --- | --- | --- | --- | --- | --- | --- | --- | --- | --- | --- | --- | --- | --- | --- | --- | --- | --- | --- | --- | --- | --- | --- | --- | --- | --- | --- | --- | --- | --- | --- | --- | --- | --- | --- | --- | --- | --- | --- | --- | --- | --- | --- | --- | --- | --- | --- | --- | --- | --- | --- | --- | --- | --- | --- | --- | --- | --- | --- | --- | --- | --- | --- | --- | --- | --- | --- | --- | --- | --- | --- | --- | --- | --- | --- | --- | --- | --- | --- | --- | --- | --- | --- | --- | --- | --- | --- | --- | --- | --- | --- | --- | --- | --- | --- | --- | --- | --- | --- | --- | --- | --- | --- | --- | --- | --- | --- | --- | --- | --- | --- | --- | --- | --- | --- | --- | --- | --- | --- | --- | --- | --- | --- | --- | --- | --- | --- | --- | --- | --- | --- | --- | --- | --- | --- | --- | --- | --- | --- | --- | --- | --- | --- | --- | --- | --- | --- | --- | --- | --- | --- | --- | --- | --- | --- | --- | --- | --- | --- | --- | --- | --- | --- | --- | --- | --- | --- | --- | --- | --- | --- | --- | --- | --- | --- | --- | --- | --- | --- | --- | --- | --- | --- | --- | --- | --- | --- | --- | --- | --- | --- | --- | --- | --- | --- | --- | --- | --- | --- | --- | --- | --- | --- | --- | --- | --- | --- | --- | --- | --- | --- | --- | --- | --- | --- | --- | --- | --- | --- | --- | --- | --- | --- | --- | --- | --- | --- | --- | --- | --- | --- | --- | --- | --- | --- | --- | --- | --- | --- | --- | --- | --- | --- | --- | --- | --- | --- | --- | --- | --- | --- | --- | --- | --- | --- | --- | --- | --- | --- | --- | --- | --- | --- | --- | --- | --- | --- | --- | --- | --- | --- | --- | --- | --- | --- | --- | --- | --- | --- | --- | --- | --- | --- | --- | --- | --- | --- | --- | --- | --- | --- | --- | --- | --- | --- | --- | --- | --- | --- | --- | --- | --- | --- | --- | --- | --- | --- | --- | --- | --- | --- | --- | --- | --- | --- | --- | --- | --- | --- | --- | --- | --- | --- | --- | --- | --- | --- | --- | --- | --- | --- | --- | --- | --- | --- | --- | --- | --- | --- | --- | --- | --- | --- | --- | --- | --- | --- | --- | --- | --- | --- | --- | --- | --- | --- | --- | --- | --- | --- | --- | --- | --- | --- | --- | --- | --- | --- | --- | --- | --- | --- | --- | --- | --- | --- | --- | --- | --- | --- | --- | --- | --- | --- | --- | --- | --- | --- | --- | --- | --- | --- | --- | --- | --- | --- | --- | --- | --- | --- | --- | --- | --- | --- | --- | --- | --- | --- | --- | --- | --- | --- | --- | --- | --- | --- | --- | --- | --- | --- | --- | --- | --- | --- | --- | --- | --- | --- | --- | --- | --- | --- | --- | --- | --- | --- | --- | --- | --- | --- | --- | --- | --- | --- | --- | --- | --- | --- | --- | --- | --- | --- | --- | --- | --- | --- | --- | --- | --- | --- | --- | --- | --- | --- | --- | --- | --- | --- | --- | --- | --- | --- | --- | --- | --- | --- | --- | --- | --- | --- | --- | --- | --- | --- | --- | --- | --- | --- | --- | --- | --- | --- | --- | --- | --- | --- | --- | --- | --- | --- | --- | --- | --- | --- | --- | --- | --- | --- | --- | --- | --- | --- | --- | --- | --- | --- | --- | --- | --- | --- | --- | --- | --- | --- | --- | --- | --- | --- | --- | --- | --- | --- | --- | --- | --- | --- | --- | --- | --- | --- | --- | --- | --- | --- | --- | --- | --- | --- | --- | --- | --- | --- | --- | --- | --- | --- | --- | --- | --- | --- | --- | --- | --- | --- | --- | --- | --- | --- | --- | --- | --- | --- | --- | --- | --- | --- | --- | --- | --- | --- | --- | --- | --- | --- | --- | --- | --- | --- | --- | --- | --- | --- | --- | --- | --- | --- | --- | --- | --- | --- | --- | --- | --- | --- | --- | --- | --- | --- | --- | --- | --- | --- | --- | --- | --- | --- | --- | --- | --- | --- | --- | --- | --- | --- | --- | --- | --- | --- | --- | --- | --- | --- | --- | --- | --- | --- | --- | --- | --- | --- | --- | --- | --- | --- | --- | --- | --- | --- | --- | --- | --- | --- | --- | --- | --- | --- | --- | --- | --- | --- | --- | --- | --- | --- | --- | --- | --- | --- | --- | --- | --- | --- | --- | --- | --- | --- | --- | --- | --- | --- | --- | --- | --- | --- | --- | --- | --- | --- | --- | --- | --- | --- | --- | --- | --- | --- | --- | --- | --- | --- | --- | --- | --- | --- | --- | --- | --- | --- | --- | --- | --- | --- | --- | --- | --- | --- | --- | --- | --- | --- | --- | --- | --- | --- | --- | --- | --- | --- | --- | --- | --- | --- | --- | --- | --- | --- | --- | --- | --- | --- | --- | --- | --- | --- | --- | --- | --- | --- | --- | --- | --- | --- | --- | --- | --- | --- | --- | --- | --- | --- | --- | --- | --- | --- | --- | --- | --- | --- | --- | --- | --- | --- | --- | --- | --- | --- | --- | --- | --- | --- | --- | --- | --- | --- | --- | --- | --- | --- | --- | --- | --- | --- | --- | --- | --- | --- | --- | --- | --- | --- | --- | --- | --- | --- | --- | --- | --- | --- | --- | --- | --- | --- | --- | --- | --- | --- | --- | --- | --- | --- | --- | --- | --- | --- | --- | --- | --- | --- | --- | --- | --- | --- | --- | --- | --- | --- | --- | --- | --- | --- | --- | --- | --- | --- | --- | --- | --- | --- | --- | --- | --- | --- | --- | --- | --- | --- | --- | --- | --- | --- | --- | --- | --- | --- | --- | --- | --- | --- | --- | --- | --- | --- | --- | --- | --- | --- | --- | --- | --- | --- | --- | --- | --- | --- | --- | --- | --- | --- | --- | --- | --- | --- | --- | --- | --- | --- | --- | --- | --- | --- | --- | --- | --- | --- | --- | --- | --- | --- | --- | --- | --- | --- | --- | --- | --- | --- | --- | --- | --- | --- | --- | --- | --- | --- | --- | --- | --- | --- | --- | --- | --- | --- | --- | --- | --- | --- | --- | --- | --- | --- | --- | --- | --- | --- | --- | --- | --- | --- | --- | --- | --- | --- | --- | --- | --- | --- | --- | --- | --- | --- | --- | --- | --- | --- | --- | --- | --- | --- | --- | --- | --- | --- | --- | --- | --- | --- | --- | --- | --- | --- | --- | --- | --- | --- | --- | --- | --- | --- | --- | --- | --- | --- | --- | --- | --- | --- | --- | --- | --- | --- | --- | --- | --- | --- | --- | --- | --- | --- | --- | --- | --- | --- | --- | --- | --- | --- | --- | --- | --- | --- | --- | --- | --- | --- | --- | --- | --- | --- | --- | --- | --- | --- | --- | --- | --- | --- | --- | --- | --- | --- | --- | --- | --- | --- | --- | --- | --- | --- | --- | --- | --- | --- | --- | --- | --- | --- | --- | --- | --- | --- | --- | --- | --- | --- | --- | --- | --- | --- | --- | --- | --- | --- | --- | --- | --- | --- | --- | --- | --- | --- | --- | --- | --- | --- | --- | --- | --- | --- | --- | --- | --- | --- | --- | --- | --- | --- | --- | --- | --- | --- | --- | --- | --- | --- | --- | --- | --- | --- | --- | --- | --- | --- | --- | --- | --- | --- | --- | --- | --- | --- | --- | --- | --- | --- | --- | --- | --- | --- | --- | --- | --- | --- | --- | --- | --- | --- | --- | --- | --- | --- | --- | --- | --- | --- | --- | --- | --- | --- | --- | --- | --- | --- | --- | --- | --- | --- | --- | --- | --- | --- | --- | --- | --- | --- | --- | --- | --- | --- | --- | --- | --- | --- | --- | --- | --- | --- | --- | --- | --- | --- | --- | --- | --- | --- | --- | --- | --- | --- | --- | --- | --- | --- | --- | --- | --- | --- | --- | --- | --- | --- | --- | --- | --- | --- | --- | --- | --- | --- | --- | --- | --- | --- | --- | --- | --- | --- | --- | --- | --- | --- | --- | --- | --- | --- | --- | --- | --- | --- | --- | --- | --- | --- | --- | --- | --- | --- | --- | --- | --- | --- | --- | --- | --- | --- | --- | --- | --- | --- | --- | --- | --- | --- | --- | --- | --- | --- | --- | --- | --- | --- | --- | --- | --- | --- | --- | --- | --- | --- | --- | --- | --- | --- | --- | --- | --- | --- | --- | --- | --- | --- | --- | --- | --- | --- | --- | --- | --- | --- | --- | --- | --- | --- | --- | --- | --- | --- | --- | --- | --- | --- | --- | --- | --- | --- | --- | --- | --- | --- | --- | --- | --- | --- | --- | --- | --- | --- | --- | --- | --- | --- | --- | --- | --- | --- | --- | --- | --- | --- | --- | --- | --- | --- | --- | --- | --- | --- | --- | --- | --- | --- | --- | --- | --- | --- | --- | --- | --- | --- | --- | --- | --- | --- | --- | --- | --- | --- | --- | --- | --- | --- | --- | --- | --- | --- | --- | --- | --- | --- | --- | --- | --- | --- | --- | --- | --- | --- | --- | --- | --- | --- | --- | --- | --- | --- | --- | --- | --- | --- | --- | --- | --- | --- | --- | --- | --- | --- | --- | --- | --- | --- | --- | --- | --- | --- | --- | --- | --- | --- | --- | --- | --- | --- | --- | --- | --- | --- | --- | --- | --- | --- | --- | --- | --- | --- | --- | --- | --- | --- | --- | --- | --- | --- | --- | --- | --- | --- | --- | --- | --- | --- | --- | --- | --- | --- | --- | --- | --- | --- | --- | --- | --- | --- | --- | --- | --- | --- | --- | --- | --- | --- | --- | --- | --- | --- | --- | --- | --- | --- | --- | --- | --- | --- | --- | --- | --- | --- | --- | --- | --- | --- | --- | --- | --- | --- | --- | --- | --- | --- | --- | --- | --- | --- | --- | --- | --- | --- | --- | --- | --- | --- | --- | --- | --- | --- | --- | --- | --- | --- | --- | --- | --- | --- | --- | --- | --- | --- | --- | --- | --- | --- | --- | --- | --- | --- | --- | --- | --- | --- | --- | --- | --- | --- | --- | --- | --- | --- | --- | --- | --- | --- | --- | --- | --- | --- | --- | --- | --- | --- | --- | --- | --- | --- | --- | --- | --- | --- | --- | --- | --- | --- | --- | --- | --- | --- | --- | --- | --- | --- | --- | --- | --- | --- | --- | --- | --- | --- | --- | --- | --- | --- | --- | --- | --- | --- | --- | --- | --- | --- | --- | --- | --- | --- | --- | --- | --- | --- | --- | --- | --- | --- | --- | --- | --- | --- | --- | --- | --- | --- | --- | --- | --- | --- | --- | --- | --- | --- | --- | --- | --- | --- | --- | --- | --- | --- | --- | --- | --- | --- | --- | --- | --- | --- | --- | --- | --- | --- | --- | --- | --- | --- | --- | --- | --- | --- | --- | --- | --- | --- | --- | --- | --- | --- | --- | --- | --- | --- | --- | --- | --- | --- | --- | --- | --- | --- | --- | --- | --- | --- | --- | --- | --- | --- | --- | --- | --- | --- | --- | --- | --- | --- | --- | --- | --- | --- | --- | --- | --- | --- | --- | --- | --- | --- | --- | --- | --- | --- | --- | --- | --- | --- | --- | --- | --- | --- | --- | --- | --- | --- | --- | --- | --- | --- | --- | --- | --- | --- | --- | --- | --- | --- | --- | --- | --- | --- | --- | --- | --- | --- | --- | --- | --- | --- | --- | --- | --- | --- | --- | --- | --- | --- | --- | --- | --- | --- | --- | --- | --- | --- | --- | --- | --- | --- | --- | --- | --- | --- | --- | --- | --- | --- | --- | --- | --- | --- | --- | --- | --- | --- | --- | --- | --- | --- | --- | --- | --- | --- | --- | --- | --- | --- | --- | --- | --- | --- | --- | --- | --- | --- | --- | --- | --- | --- | --- | --- | --- | --- | --- | --- | --- | --- | --- | --- | --- | --- | --- | --- | --- | --- | --- | --- | --- | --- | --- | --- | --- | --- | --- | --- | --- | --- | --- | --- | --- | --- | --- | --- | --- | --- | --- | --- | --- | --- | --- | --- | --- | --- | --- | --- | --- | --- | --- | --- | --- | --- | --- | --- | --- | --- | --- | --- | --- | --- | --- | --- | --- | --- | --- | --- | --- | --- | --- | --- | --- | --- | --- | --- | --- | --- | --- | --- | --- | --- | --- | --- | --- | --- | --- | --- | --- | --- | --- | --- | --- | --- | --- | --- | --- | --- | --- | --- | --- | --- | --- | --- | --- | --- | --- | --- | --- | --- | --- | --- | --- | --- | --- | --- | --- | --- | --- | --- | --- | --- | --- | --- | --- | --- | --- | --- | --- | --- | --- | --- | --- | --- | --- | --- | --- | --- | --- | --- | --- | --- | --- | --- | --- | --- | --- | --- | --- | --- | --- | --- | --- | --- | --- | --- | --- | --- | --- | --- | --- | --- | --- | --- | --- | --- | --- | --- | --- | --- | --- | --- | --- | --- | --- | --- | --- | --- | --- | --- | --- | --- | --- | --- | --- | --- | --- | --- | --- | --- | --- | --- | --- | --- | --- | --- | --- | --- | --- | --- | --- | --- | --- | --- | --- | --- | --- | --- | --- | --- | --- | --- | --- | --- | --- | --- | --- | --- | --- | --- | --- | --- | --- | --- | --- | --- | --- | --- | --- | --- | --- | --- | --- | --- | --- | --- | --- | --- | --- | --- | --- | --- | --- | --- | --- | --- | --- | --- | --- | --- | --- | --- | --- | --- | --- | --- | --- | --- | --- | --- | --- | --- | --- | --- | --- | --- | --- | --- | --- | --- | --- | --- | --- | --- | --- | --- | --- | --- | --- | --- | --- | --- | --- | --- | --- | --- | --- | --- | --- | --- | --- | --- | --- | --- | --- | --- | --- | --- | --- | --- | --- | --- | --- | --- | --- | --- | --- | --- | --- | --- | --- | --- | --- | --- | --- | --- | --- | --- | --- | --- | --- | --- | --- | --- | --- | --- | --- | --- | --- | --- | --- | --- | --- | --- | --- | --- | --- | --- | --- | --- | --- | --- | --- | --- | --- | --- | --- | --- | --- | --- | --- | --- | --- | --- | --- | --- | --- | --- | --- | --- | --- | --- | --- | --- | --- | --- | --- | --- | --- | --- | --- | --- | --- | --- | --- | --- | --- | --- | --- | --- | --- | --- | --- | --- | --- | --- | --- | --- | --- | --- | --- | --- | --- | --- | --- | --- | --- | --- | --- | --- | --- | --- | --- | --- | --- | --- | --- | --- | --- | --- | --- | --- | --- | --- | --- | --- | --- | --- | --- | --- | --- | --- | --- | --- | --- | --- | --- | --- | --- | --- | --- | --- | --- | --- | --- | --- | --- | --- | --- | --- | --- | --- | --- | --- | --- | --- | --- | --- | --- | --- | --- | --- | --- | --- | --- | --- | --- | --- | --- | --- | --- | --- | --- | --- | --- | --- | --- | --- | --- | --- | --- | --- | --- | --- | --- | --- | --- | --- | --- | --- | --- | --- | --- | --- | --- | --- | --- | --- | --- | --- | --- | --- | --- | --- | --- | --- | --- | --- | --- | --- | --- | --- | --- | --- | --- | --- | --- | --- | --- | --- | --- | --- | --- | --- | --- | --- | --- | --- | --- | --- | --- | --- | --- | --- | --- | --- | --- | --- | --- | --- | --- | --- | --- | --- | --- | --- | --- | --- | --- | --- | --- | --- | --- | --- | --- | --- | --- | --- | --- | --- | --- | --- | --- | --- | --- | --- | --- | --- | --- | --- | --- | --- | --- | --- | --- | --- | --- | --- | --- | --- | --- | --- | --- | --- | --- | --- | --- | --- | --- | --- | --- | --- | --- | --- | --- | --- | --- | --- | --- | --- | --- | --- | --- | --- | --- | --- | --- | --- | --- | --- | --- | --- | --- | --- | --- | --- | --- | --- | --- | --- | --- | --- | --- | --- | --- | --- | --- | --- | --- | --- | --- | --- | --- | --- | --- | --- | --- | --- | --- | --- | --- | --- | --- | --- | --- | --- | --- | --- | --- | --- | --- | --- | --- | --- | --- | --- | --- | --- | --- | --- | --- | --- | --- | --- | --- | --- | --- | --- | --- | --- | --- | --- | --- | --- | --- | --- | --- | --- | --- | --- | --- | --- | --- | --- | --- | --- | --- | --- | --- | --- | --- | --- | --- | --- | --- | --- | --- | --- | --- | --- | --- | --- | --- | --- | --- | --- | --- | --- | --- | --- | --- | --- | --- | --- | --- | --- | --- | --- | --- | --- | --- | --- | --- | --- | --- | --- | --- | --- | --- | --- | --- | --- | --- | --- | --- | --- | --- | --- | --- | --- | --- | --- | --- | --- | --- | --- | --- | --- | --- | --- | --- | --- | --- | --- | --- | --- | --- | --- | --- | --- | --- | --- | --- | --- | --- | --- | --- | --- | --- | --- | --- | --- | --- | --- | --- | --- | --- | --- | --- | --- | --- | --- | --- | --- | --- | --- | --- | --- | --- | --- | --- | --- | --- | --- | --- | --- | --- | --- | --- | --- | --- | --- | --- | --- | --- | --- | --- | --- | --- | --- | --- | --- | --- | --- | --- | --- | --- | --- | --- | --- | --- | --- | --- | --- | --- | --- | --- | --- | --- | --- | --- | --- | --- | --- | --- | --- | --- | --- | --- | --- | --- | --- | --- | --- | --- | --- | --- | --- | --- | --- | --- | --- | --- | --- | --- | --- | --- | --- | --- | --- | --- | --- | --- | --- | --- | --- | --- | --- | --- | --- | --- | --- | --- | --- | --- | --- | --- | --- | --- | --- | --- | --- | --- | --- | --- | --- | --- | --- | --- | --- | --- | --- | --- | --- | --- | --- | --- | --- | --- | --- | --- | --- | --- | --- | --- | --- | --- | --- | --- | --- | --- | --- | --- | --- | --- | --- | --- | --- | --- | --- | --- | --- | --- | --- | --- | --- | --- | --- | --- | --- | --- | --- | --- | --- | --- | --- | --- | --- | --- | --- | --- | --- | --- | --- | --- | --- | --- | --- | --- | --- | --- | --- | --- | --- | --- | --- | --- | --- | --- | --- | --- | --- | --- | --- | --- | --- | --- | --- | --- | --- | --- | --- | --- | --- | --- | --- | --- | --- | --- | --- | --- | --- | --- | --- | --- | --- | --- | --- | --- | --- | --- | --- | --- | --- | --- | --- | --- | --- | --- | --- | --- | --- | --- | --- | --- | --- | --- | --- | --- | --- | --- | --- | --- | --- | --- | --- | --- | --- | --- | --- | --- | --- | --- | --- | --- | --- | --- | --- | --- | --- | --- | --- | --- | --- | --- | --- | --- | --- | --- | --- | --- | --- | --- | --- | --- | --- | --- | --- | --- | --- | --- | --- | --- | --- | --- | --- | --- | --- | --- | --- | --- | --- | --- | --- | --- | --- | --- | --- | --- | --- | --- | --- | --- | --- | --- | --- | --- | --- | --- | --- | --- | --- | --- | --- | --- | --- | --- | --- | --- | --- | --- | --- | --- | --- | --- | --- | --- | --- | --- | --- | --- | --- | --- | --- | --- | --- | --- | --- | --- | --- | --- | --- | --- | --- | --- | --- | --- | --- | --- | --- | --- | --- | --- | --- | --- | --- | --- | --- | --- | --- | --- | --- | --- | --- | --- | --- | --- | --- | --- | --- | --- | --- | --- | --- | --- | --- | --- | --- | --- | --- | --- | --- | --- | --- | --- | --- | --- | --- | --- | --- | --- | --- | --- | --- | --- | --- | --- | --- | --- | --- | --- | --- | --- | --- | --- | --- | --- | --- | --- | --- | --- | --- | --- | --- | --- | --- | --- | --- | --- | --- | --- | --- | --- | --- | --- | --- | --- | --- | --- | --- | --- | --- | --- | --- | --- | --- | --- | --- | --- | --- | --- | --- | --- | --- | --- | --- | --- | --- | --- | --- | --- | --- | --- | --- | --- | --- | --- | --- | --- | --- | --- | --- | --- | --- | --- | --- | --- | --- | --- | --- | --- | --- | --- | --- | --- | --- | --- | --- | --- | --- | --- | --- | --- | --- | --- | --- | --- | --- | --- | --- | --- | --- | --- | --- | --- | --- | --- | --- | --- | --- | --- | --- | --- | --- | --- | --- | --- | --- | --- | --- | --- | --- | --- | --- | --- | --- | --- | --- | --- | --- | --- | --- | --- | --- | --- | --- | --- | --- | --- | --- | --- | --- | --- | --- | --- | --- | --- | --- | --- | --- | --- | --- | --- | --- | --- | --- | --- | --- | --- | --- | --- | --- | --- | --- | --- | --- | --- | --- | --- | --- | --- | --- | --- | --- | --- | --- | --- | --- | --- | --- | --- | --- | --- | --- | --- | --- | --- | --- | --- | --- | --- | --- | --- | --- | --- | --- | --- | --- | --- | --- | --- | --- | --- | --- | --- | --- | --- | --- | --- | --- | --- | --- | --- | --- | --- | --- | --- | --- | --- | --- | --- | --- | --- | --- | --- | --- | --- | --- | --- | --- | --- | --- | --- | --- | --- | --- | --- | --- | --- | --- | --- | --- | --- | --- | --- | --- | --- | --- | --- | --- | --- | --- | --- | --- | --- | --- | --- | --- | --- | --- | --- | --- | --- | --- | --- | --- | --- | --- | --- | --- | --- | --- | --- | --- | --- | --- | --- | --- | --- | --- | --- | --- | --- | --- | --- | --- | --- | --- | --- | --- | --- | --- | --- | --- | --- | --- | --- | --- | --- | --- | --- | --- | --- | --- | --- | --- | --- | --- | --- | --- | --- | --- | --- | --- | --- | --- | --- | --- | --- | --- | --- | --- | --- | --- | --- | --- | --- | --- | --- | --- | --- | --- | --- | --- | --- | --- | --- | --- | --- | --- | --- | --- | --- | --- | --- | --- | --- | --- | --- | --- | --- | --- | --- | --- | --- | --- | --- | --- | --- | --- | --- | --- | --- | --- | --- | --- | --- | --- | --- | --- | --- | --- | --- | --- | --- | --- | --- | --- | --- | --- | --- | --- | --- | --- | --- | --- | --- | --- | --- | --- | --- | --- | --- | --- | --- | --- | --- | --- | --- | --- | --- | --- | --- | --- | --- | --- | --- | --- | --- | --- | --- | --- | --- | --- | --- | --- | --- | --- | --- | --- | --- | --- | --- | --- | --- | --- | --- | --- | --- | --- | --- | --- | --- | --- | --- | --- | --- | --- | --- | --- | --- | --- | --- | --- | --- | --- | --- | --- | --- | --- | --- | --- | --- | --- | --- | --- | --- | --- | --- | --- | --- | --- | --- | --- | --- | --- | --- | --- | --- | --- | --- | --- | --- | --- | --- | --- | --- | --- | --- | --- | --- | --- | --- | --- | --- | --- | --- | --- | --- | --- | --- | --- | --- | --- | --- | --- | --- | --- | --- | --- | --- | --- | --- | --- | --- | --- | --- | --- | --- | --- | --- | --- | --- | --- | --- | --- | --- | --- | --- | --- | --- | --- | --- | --- | --- | --- | --- | --- | --- | --- | --- | --- | --- | --- | --- | --- | --- | --- | --- | --- | --- | --- | --- | --- | --- | --- | --- | --- | --- | --- | --- | --- | --- | --- | --- | --- | --- | --- | --- | --- | --- | --- | --- | --- | --- | --- | --- | --- | --- | --- | --- | --- | --- | --- | --- | --- | --- | --- | --- | --- | --- | --- | --- | --- | --- | --- | --- | --- | --- | --- | --- | --- | --- | --- | --- | --- | --- | --- | --- | --- | --- | --- | --- | --- | --- | --- | --- | --- | --- | --- | --- | --- | --- | --- | --- | --- | --- | --- | --- | --- | --- | --- | --- | --- | --- | --- | --- | --- | --- | --- | --- | --- | --- | --- | --- | --- | --- | --- | --- | --- | --- | --- | --- | --- | --- | --- | --- | --- | --- | --- | --- | --- | --- | --- | --- | --- | --- | --- | --- | --- | --- | --- | --- | --- | --- | --- | --- | --- | --- | --- | --- | --- | --- | --- | --- | --- | --- | --- | --- | --- | --- | --- | --- | --- | --- | --- | --- | --- | --- | --- | --- | --- | --- | --- | --- | --- | --- | --- | --- | --- | --- | --- | --- | --- | --- | --- | --- | --- | --- | --- | --- | --- | --- | --- | --- | --- | --- | --- | --- | --- | --- | --- | --- | --- | --- | --- | --- | --- | --- | --- | --- | --- | --- | --- | --- | --- | --- | --- | --- | --- | --- | --- | --- | --- | --- | --- | --- | --- | --- | --- | --- | --- | --- | --- | --- | --- | --- | --- | --- | --- | --- | --- | --- | --- | --- | --- | --- | --- | --- | --- | --- | --- | --- | --- | --- | --- | --- | --- | --- | --- | --- | --- | --- | --- | --- | --- | --- | --- | --- | --- | --- | --- | --- | --- | --- | --- | --- | --- | --- | --- | --- | --- | --- | --- | --- | --- | --- | --- | --- | --- | --- | --- | --- | --- | --- | --- | --- | --- | --- | --- | --- | --- | --- | --- | --- | --- | --- | --- | --- | --- | --- | --- | --- | --- | --- | --- | --- | --- | --- | --- | --- | --- | --- | --- | --- | --- | --- | --- | --- | --- | --- | --- | --- | --- | --- | --- | --- | --- | --- | --- | --- | --- | --- | --- | --- | --- | --- | --- | --- | --- | --- | --- | --- | --- | --- | --- | --- | --- | --- | --- | --- | --- | --- | --- | --- | --- | --- | --- | --- | --- | --- | --- | --- | --- | --- | --- | --- | --- | --- | --- | --- | --- | --- | --- | --- | --- | --- | --- | --- | --- | --- | --- | --- | --- | --- | --- | --- | --- | --- | --- | --- | --- | --- | --- | --- | --- | --- | --- | --- | --- | --- | --- | --- | --- | --- | --- | --- | --- | --- | --- | --- | --- | --- | --- | --- | --- | --- | --- | --- | --- | --- | --- | --- | --- | --- | --- | --- | --- | --- | --- | --- | --- | --- | --- | --- | --- | --- | --- | --- | --- | --- | --- | --- | --- | --- | --- | --- | --- | --- | --- | --- | --- | --- | --- | --- | --- | --- | --- | --- | --- | --- | --- | --- | --- | --- | --- | --- | --- | --- | --- | --- | --- | --- | --- | --- | --- | --- | --- | --- | --- | --- | --- | --- | --- | --- | --- | --- | --- | --- | --- | --- | --- | --- | --- | --- | --- | --- | --- | --- | --- | --- | --- | --- | --- | --- | --- | --- | --- | --- | --- | --- | --- | --- | --- | --- | --- | --- | --- | --- | --- | --- | --- | --- | --- | --- | --- | --- | --- | --- | --- | --- | --- | --- | --- | --- | --- | --- | --- | --- | --- | --- | --- | --- | --- | --- | --- | --- | --- | --- | --- | --- | --- | --- | --- | --- | --- | --- | --- | --- | --- | --- | --- | --- | --- | --- | --- | --- | --- | --- | --- | --- | --- | --- | --- | --- | --- | --- | --- | --- | --- | --- | --- | --- | --- | --- | --- | --- | --- | --- | --- | --- | --- | --- | --- | --- | --- | --- | --- | --- | --- | --- | --- | --- | --- | --- | --- | --- | --- | --- | --- | --- | --- | --- | --- | --- | --- | --- | --- | --- | --- | --- | --- | --- | --- | --- | --- | --- | --- | --- | --- | --- | --- | --- | --- | --- | --- | --- | --- | --- | --- | --- | --- | --- | --- | --- | --- | --- | --- | --- | --- | --- | --- | --- | --- | --- | --- | --- | --- | --- | --- | --- | --- | --- | --- | --- | --- | --- | --- | --- | --- | --- | --- | --- | --- | --- | --- | --- | --- | --- | --- | --- | --- | --- | --- | --- | --- | --- | --- | --- | --- | --- | --- | --- | --- | --- | --- | --- | --- | --- | --- | --- | --- | --- | --- | --- | --- | --- | --- | --- | --- | --- | --- | --- | --- | --- | --- | --- | --- | --- | --- | --- | --- | --- | --- | --- | --- | --- | --- | --- | --- | --- | --- | --- | --- | --- | --- | --- | --- | --- | --- | --- | --- | --- | --- | --- | --- | --- | --- | --- | --- | --- | --- | --- | --- | --- | --- | --- | --- | --- | --- | --- | --- | --- | --- | --- | --- | --- | --- | --- | --- | --- | --- | --- | --- | --- | --- | --- | --- | --- | --- | --- | --- | --- | --- | --- | --- | --- | --- | --- | --- | --- | --- | --- | --- | --- | --- | --- | --- | --- | --- | --- | --- | --- | --- | --- | --- | --- | --- | --- | --- | --- | --- | --- | --- | --- | --- | --- | --- | --- | --- | --- | --- | --- | --- | --- | --- | --- | --- | --- | --- | --- | --- | --- | --- | --- | --- | --- | --- | --- | --- | --- | --- | --- | --- | --- | --- | --- | --- | --- | --- | --- | --- | --- | --- | --- | --- | --- | --- | --- | --- | --- | --- | --- | --- | --- | --- | --- | --- | --- | --- | --- | --- | --- | --- | --- | --- | --- | --- | --- | --- | --- | --- | --- | --- | --- | --- | --- | --- | --- | --- | --- | --- | --- | --- | --- | --- | --- | --- | --- | --- | --- | --- | --- | --- | --- | --- | --- | --- | --- | --- | --- | --- | --- | --- | --- | --- | --- | --- | --- | --- | --- | --- | --- | --- | --- | --- | --- | --- | --- | --- | --- | --- | --- | --- | --- | --- | --- | --- | --- | --- | --- | --- | --- | --- | --- | --- | --- | --- | --- | --- | --- | --- | --- | --- | --- | --- | --- | --- | --- | --- | --- | --- | --- | --- | --- | --- | --- | --- | --- | --- | --- | --- | --- | --- | --- | --- | --- | --- | --- | --- | --- | --- | --- | --- | --- | --- | --- | --- | --- | --- | --- | --- | --- | --- | --- | --- | --- | --- | --- | --- | --- | --- | --- | --- | --- | --- | --- | --- | --- | --- | --- | --- | --- | --- | --- | --- | --- | --- | --- | --- | --- | --- | --- | --- | --- | --- | --- | --- | --- | --- | --- | --- | --- | --- | --- | --- | --- | --- | --- | --- | --- | --- | --- | --- | --- | --- | --- | --- | --- | --- | --- | --- | --- | --- | --- | --- | --- | --- | --- | --- | --- | --- | --- | --- | --- | --- | --- | --- | --- | --- | --- | --- | --- | --- | --- | --- | --- | --- | --- | --- | --- | --- | --- | --- | --- | --- | --- | --- | --- | --- | --- | --- | --- | --- | --- | --- | --- | --- | --- | --- | --- | --- | --- | --- | --- | --- | --- | --- | --- | --- | --- | --- | --- | --- | --- | --- | --- | --- | --- | --- | --- | --- | --- | --- | --- | --- | --- | --- | --- | --- | --- | --- | --- | --- | --- | --- | --- | --- | --- | --- | --- | --- | --- | --- | --- | --- | --- | --- | --- | --- | --- | --- | --- | --- | --- | --- | --- | --- | --- | --- | --- | --- | --- | --- | --- | --- | --- | --- | --- | --- | --- | --- | --- | --- | --- | --- | --- | --- | --- | --- | --- | --- | --- | --- | --- | --- | --- | --- | --- | --- | --- | --- | --- | --- | --- | --- | --- | --- | --- | --- | --- | --- | --- | --- | --- | --- | --- | --- | --- | --- | --- | --- | --- | --- | --- | --- | --- | --- | --- | --- | --- | --- | --- | --- | --- | --- | --- | --- | --- | --- | --- | --- | --- | --- | --- | --- | --- | --- | --- | --- | --- | --- | --- | --- | --- | --- | --- | --- | --- | --- | --- | --- | --- | --- | --- | --- | --- | --- | --- | --- | --- | --- | --- | --- | --- | --- | --- | --- | --- | --- | --- | --- | --- | --- | --- | --- | --- | --- | --- | --- | --- | --- | --- | --- | --- | --- | --- | --- | --- | --- | --- | --- | --- | --- | --- | --- | --- | --- | --- | --- | --- | --- | --- | --- | --- | --- | --- | --- | --- | --- | --- | --- | --- | --- | --- | --- | --- | --- | --- | --- | --- | --- | --- | --- | --- | --- | --- | --- | --- | --- | --- | --- | --- | --- | --- | --- | --- | --- | --- | --- | --- | --- | --- | --- | --- | --- | --- | --- | --- | --- | --- | --- | --- | --- | --- | --- | --- | --- | --- | --- | --- | --- | --- | --- | --- | --- | --- | --- | --- | --- | --- | --- | --- | --- | --- | --- | --- | --- | --- | --- | --- | --- | --- | --- | --- | --- | --- | --- | --- | --- | --- | --- | --- | --- | --- | --- | --- | --- | --- | --- | --- | --- | --- | --- | --- | --- | --- | --- | --- | --- | --- | --- | --- | --- | --- | --- | --- | --- | --- | --- | --- | --- | --- | --- | --- | --- | --- | --- | --- | --- | --- | --- | --- | --- | --- | --- | --- | --- | --- | --- | --- | --- | --- | --- | --- | --- | --- | --- | --- | --- | --- | --- | --- | --- | --- | --- | --- | --- | --- | --- | --- | --- | --- | --- | --- | --- | --- | --- | --- | --- | --- | --- | --- | --- | --- | --- | --- | --- | --- | --- | --- | --- | --- | --- | --- | --- | --- | --- | --- | --- | --- | --- | --- | --- | --- | --- | --- | --- | --- | --- | --- | --- | --- | --- | --- | --- | --- | --- | --- | --- | --- | --- | --- | --- | --- | --- | --- | --- | --- | --- | --- | --- | --- | --- | --- | --- | --- | --- | --- | --- | --- | --- | --- | --- | --- | --- | --- | --- | --- | --- | --- | --- | --- | --- | --- | --- | --- | --- | --- | --- | --- | --- | --- | --- | --- | --- | --- | --- | --- | --- | --- | --- | --- | --- | --- | --- | --- | --- | --- | --- | --- | --- | --- | --- | --- | --- | --- | --- | --- | --- | --- | --- | --- | --- | --- | --- | --- | --- | --- | --- | --- | --- | --- | --- | --- | --- | --- | --- | --- | --- | --- | --- | --- | --- | --- | --- | --- | --- | --- | --- | --- | --- | --- | --- | --- | --- | --- | --- | --- | --- | --- | --- | --- | --- | --- | --- | --- | --- | --- | --- | --- | --- | --- | --- | --- | --- | --- | --- | --- | --- | --- | --- | --- | --- | --- | --- | --- | --- | --- | --- | --- | --- | --- | --- | --- | --- | --- | --- | --- | --- | --- | --- | --- | --- | --- | --- | --- | --- | --- | --- | --- | --- | --- | --- | --- | --- | --- | --- | --- | --- | --- | --- | --- | --- | --- | --- | --- | --- | --- | --- | --- | --- | --- | --- | --- | --- | --- | --- | --- | --- | --- | --- | --- | --- | --- | --- | --- | --- | --- | --- | --- | --- | --- | --- | --- | --- | --- | --- | --- | --- | --- | --- | --- | --- | --- | --- | --- | --- | --- | --- | --- | --- | --- | --- | --- | --- | --- | --- | --- | --- | --- | --- | --- | --- | --- | --- | --- | --- | --- | --- | --- | --- | --- | --- | --- | --- | --- | --- | --- | --- | --- | --- | --- | --- | --- | --- | --- | --- | --- | --- | --- | --- | --- | --- | --- | --- | --- | --- | --- | --- | --- | --- | --- | --- | --- | --- | --- | --- | --- | --- | --- | --- | --- | --- | --- | --- | --- | --- | --- | --- | --- | --- | --- | --- | --- | --- | --- | --- | --- | --- | --- | --- | --- | --- | --- | --- | --- | --- | --- | --- | --- | --- | --- | --- | --- | --- | --- | --- | --- | --- | --- | --- | --- | --- | --- | --- | --- | --- | --- | --- | --- | --- | --- | --- | --- | --- | --- | --- | --- | --- | --- | --- | --- | --- | --- | --- | --- | --- | --- | --- | --- | --- | --- | --- | --- | --- | --- | --- | --- | --- | --- | --- | --- | --- | --- | --- | --- | --- | --- | --- | --- | --- | --- | --- | --- | --- | --- | --- | --- | --- | --- | --- | --- | --- | --- | --- | --- | --- | --- | --- | --- | --- | --- | --- | --- | --- | --- | --- | --- | --- | --- | --- | --- | --- | --- | --- | --- | --- | --- | --- | --- | --- | --- | --- | --- | --- | --- | --- | --- | --- | --- | --- | --- | --- | --- | --- | --- | --- | --- | --- | --- | --- | --- | --- | --- | --- | --- | --- | --- | --- | --- | --- | --- | --- | --- | --- | --- | --- | --- | --- | --- | --- | --- | --- | --- | --- | --- | --- | --- | --- | --- | --- | --- | --- | --- | --- | --- | --- | --- | --- | --- | --- | --- | --- | --- | --- | --- | --- | --- | --- | --- | --- | --- | --- | --- | --- | --- | --- | --- | --- | --- | --- | --- | --- | --- | --- | --- | --- | --- | --- | --- | --- | --- | --- | --- | --- | --- | --- | --- | --- | --- | --- | --- | --- | --- | --- | --- | --- | --- | --- | --- | --- | --- | --- | --- | --- | --- | --- | --- | --- | --- | --- | --- | --- | --- | --- | --- | --- | --- | --- | --- | --- | --- | --- | --- | --- | --- | --- | --- | --- | --- | --- | --- | --- | --- | --- | --- | --- | --- | --- | --- | --- | --- | --- | --- | --- | --- | --- | --- | --- | --- | --- | --- | --- | --- | --- | --- | --- | --- | --- | --- | --- | --- | --- | --- | --- | --- | --- | --- | --- | --- | --- | --- | --- | --- | --- | --- | --- | --- | --- | --- | --- | --- | --- | --- | --- | --- | --- | --- | --- | --- | --- | --- | --- | --- | --- | --- | --- | --- | --- | --- | --- | --- | --- | --- | --- | --- | --- | --- | --- | --- | --- | --- | --- | --- | --- | --- | --- | --- | --- | --- | --- | --- | --- | --- | --- | --- | --- | --- | --- | --- | --- | --- | --- | --- | --- | --- | --- | --- | --- | --- | --- | --- | --- | --- | --- | --- | --- | --- | --- | --- | --- | --- | --- | --- | --- | --- | --- | --- | --- | --- | --- | --- | --- | --- | --- | --- | --- | --- | --- | --- | --- | --- | --- | --- | --- | --- | --- | --- | --- | --- | --- | --- | --- | --- | --- | --- | --- | --- | --- | --- | --- | --- | --- | --- | --- | --- | --- | --- | --- | --- | --- | --- | --- | --- | --- | --- | --- | --- | --- | --- | --- | --- | --- | --- | --- | --- | --- | --- | --- | --- | --- | --- | --- | --- | --- | --- | --- | --- | --- | --- | --- | --- | --- | --- | --- | --- | --- | --- | --- | --- | --- | --- | --- | --- | --- | --- | --- | --- | --- | --- | --- | --- | --- | --- | --- | --- | --- | --- | --- | --- | --- | --- | --- | --- | --- | --- | --- | --- | --- | --- | --- | --- | --- | --- | --- | --- | --- | --- | --- | --- | --- | --- | --- | --- | --- | --- | --- | --- | --- | --- | --- | --- | --- | --- | --- | --- | --- | --- | --- | --- | --- | --- | --- | --- | --- | --- | --- | --- | --- | --- | --- | --- | --- | --- | --- | --- | --- | --- | --- | --- | --- | --- | --- | --- | --- | --- | --- | --- | --- | --- | --- | --- | --- | --- | --- | --- | --- | --- | --- | --- | --- | --- | --- | --- | --- | --- | --- | --- | --- | --- | --- | --- | --- | --- | --- | --- | --- | --- | --- | --- | --- | --- | --- | --- | --- | --- | --- | --- | --- | --- | --- | --- | --- | --- | --- | --- | --- | --- | --- | --- | --- | --- | --- | --- | --- | --- | --- | --- | --- | --- | --- | --- | --- | --- | --- | --- | --- | --- | --- | --- | --- | --- | --- | --- | --- | --- | --- | --- | --- | --- | --- | --- | --- | --- | --- | --- | --- | --- | --- | --- | --- | --- | --- | --- | --- | --- | --- | --- | --- | --- | --- | --- | --- | --- | --- | --- | --- | --- | --- | --- | --- | --- | --- | --- | --- | --- | --- | --- | --- | --- | --- | --- | --- | --- | --- | --- | --- | --- | --- | --- | --- | --- | --- | --- | --- | --- | --- | --- | --- | --- | --- | --- | --- | --- | --- | --- | --- | --- | --- | --- | --- | --- | --- | --- | --- | --- | --- | --- | --- | --- | --- | --- | --- | --- | --- | --- | --- | --- | --- | --- | --- | --- | --- | --- | --- | --- | --- | --- | --- | --- | --- | --- | --- | --- | --- | --- | --- | --- | --- | --- | --- | --- | --- | --- | --- | --- | --- | --- | --- | --- | --- | --- | --- | --- | --- | --- | --- | --- | --- | --- | --- | --- | --- | --- | --- | --- | --- | --- | --- | --- | --- | --- | --- | --- | --- | --- | --- | --- | --- | --- | --- | --- | --- | --- | --- | --- | --- | --- | --- | --- | --- | --- | --- | --- | --- | --- | --- | --- | --- | --- | --- | --- | --- | --- | --- | --- | --- | --- | --- | --- | --- | --- | --- | --- | --- | --- | --- | --- | --- | --- | --- | --- | --- | --- | --- | --- | --- | --- | --- | --- | --- | --- | --- | --- | --- | --- | --- | --- | --- | --- | --- | --- | --- | --- | --- | --- | --- | --- | --- | --- | --- | --- | --- | --- | --- | --- | --- | --- | --- | --- | --- | --- | --- | --- | --- | --- | --- | --- | --- | --- | --- | --- | --- | --- | --- | --- | --- | --- | --- | --- | --- | --- | --- | --- | --- | --- | --- | --- | --- | --- | --- | --- | --- | --- | --- | --- | --- | --- | --- | --- | --- | --- | --- | --- | --- | --- | --- | --- | --- | --- | --- | --- | --- | --- | --- | --- | --- | --- | --- | --- | --- | --- | --- | --- | --- | --- | --- | --- | --- | --- | --- | --- | --- | --- | --- | --- | --- | --- | --- | --- | --- | --- | --- | --- | --- | --- | --- | --- | --- | --- | --- | --- | --- | --- | --- | --- | --- | --- | --- | --- | --- | --- | --- | --- | --- | --- | --- | --- | --- | --- | --- | --- | --- | --- | --- | --- | --- | --- | --- | --- | --- | --- | --- | --- | --- | --- | --- | --- | --- | --- | --- | --- | --- | --- | --- | --- | --- | --- | --- | --- | --- | --- | --- | --- | --- | --- | --- | --- | --- | --- | --- | --- | --- | --- | --- | --- | --- | --- | --- | --- | --- | --- | --- | --- | --- | --- | --- | --- | --- | --- | --- | --- | --- | --- | --- | --- | --- | --- | --- | --- | --- | --- | --- | --- | --- | --- | --- | --- | --- | --- | --- | --- | --- | --- | --- | --- | --- | --- | --- | --- | --- | --- | --- | --- | --- | --- | --- | --- | --- | --- | --- | --- | --- | --- | --- | --- | --- | --- | --- | --- | --- | --- | --- | --- | --- | --- | --- | --- | --- | --- | --- | --- | --- | --- | --- | --- | --- | --- | --- | --- | --- | --- | --- | --- | --- | --- | --- | --- | --- | --- | --- | --- | --- | --- | --- | --- | --- | --- | --- | --- | --- | --- | --- | --- | --- | --- | --- | --- | --- | --- | --- | --- | --- | --- | --- | --- | --- | --- | --- | --- | --- | --- | --- | --- | --- | --- | --- | --- | --- | --- | --- | --- | --- | --- | --- | --- | --- | --- | --- | --- | --- | --- | --- | --- | --- | --- | --- | --- | --- | --- | --- | --- | --- | --- | --- | --- | --- | --- | --- | --- | --- | --- | --- | --- | --- | --- | --- | --- | --- | --- | --- | --- | --- | --- | --- | --- | --- | --- | --- | --- | --- | --- | --- | --- | --- | --- | --- | --- | --- | --- | --- | --- | --- | --- | --- | --- | --- | --- | --- | --- | --- | --- | --- | --- | --- | --- | --- | --- | --- | --- | --- | --- | --- | --- | --- | --- | --- | --- | --- | --- | --- | --- | --- | --- | --- | --- | --- | --- | --- | --- | --- | --- | --- | --- | --- | --- | --- | --- | --- | --- | --- | --- | --- | --- | --- | --- | --- | --- | --- | --- | --- | --- | --- | --- | --- | --- | --- | --- | --- | --- | --- | --- | --- | --- | --- | --- | --- | --- | --- | --- | --- | --- | --- | --- | --- | --- | --- | --- | --- | --- | --- | --- | --- | --- | --- | --- | --- | --- | --- | --- | --- | --- | --- | --- | --- | --- | --- | --- | --- | --- | --- | --- | --- | --- | --- | --- | --- | --- | --- | --- | --- | --- | --- | --- | --- | --- | --- | --- | --- | --- | --- | --- | --- | --- | --- | --- | --- | --- | --- | --- | --- | --- | --- | --- | --- | --- | --- | --- | --- | --- | --- | --- | --- | --- | --- | --- | --- | --- | --- | --- | --- | --- | --- | --- | --- | --- | --- | --- | --- | --- | --- | --- | --- | --- | --- | --- | --- | --- | --- | --- | --- | --- | --- | --- | --- | --- | --- | --- | --- | --- | --- | --- | --- | --- | --- | --- | --- | --- | --- | --- | --- | --- | --- | --- | --- | --- | --- | --- | --- | --- | --- | --- | --- | --- | --- | --- | --- | --- | --- | --- | --- | --- | --- | --- | --- | --- | --- | --- | --- | --- | --- | --- | --- | --- | --- | --- | --- | --- | --- | --- | --- | --- | --- | --- | --- | --- | --- | --- | --- | --- | --- | --- | --- | --- | --- | --- | --- | --- | --- | --- | --- | --- | --- | --- | --- | --- | --- | --- | --- | --- | --- | --- | --- | --- | --- | --- | --- | --- | --- | --- | --- | --- | --- | --- | --- | --- | --- | --- | --- | --- | --- | --- | --- | --- | --- | --- | --- | --- | --- | --- | --- | --- | --- | --- | --- | --- | --- | --- | --- | --- | --- | --- | --- | --- | --- | --- | --- | --- | --- | --- | --- | --- | --- | --- | --- | --- | --- | --- | --- | --- | --- | --- | --- | --- | --- | --- | --- | --- | --- | --- | --- | --- | --- | --- | --- | --- | --- | --- | --- | --- | --- | --- | --- | --- | --- | --- | --- | --- | --- | --- | --- | --- | --- | --- | --- | --- | --- | --- | --- | --- | --- | --- | --- | --- | --- | --- | --- | --- | --- | --- | --- | --- | --- | --- | --- | --- | --- | --- | --- | --- | --- | --- | --- | --- | --- | --- | --- | --- | --- | --- | --- | --- | --- | --- | --- | --- | --- | --- | --- | --- | --- | --- | --- | --- | --- | --- | --- | --- | --- | --- | --- | --- | --- | --- | --- | --- | --- | --- | --- | --- | --- | --- | --- | --- | --- | --- | --- | --- | --- | --- | --- | --- | --- | --- | --- | --- | --- | --- | --- | --- | --- | --- | --- | --- | --- | --- | --- | --- | --- | --- | --- | --- | --- | --- | --- | --- | --- | --- | --- | --- | --- | --- | --- | --- | --- | --- | --- | --- | --- | --- | --- | --- | --- | --- | --- | --- | --- | --- | --- | --- | --- | --- | --- | --- | --- | --- | --- | --- | --- | --- | --- | --- | --- | --- | --- | --- | --- | --- | --- | --- | --- | --- | --- | --- | --- | --- | --- | --- | --- | --- | --- | --- | --- | --- | --- | --- | --- | --- | --- | --- | --- | --- | --- | --- | --- | --- | --- | --- | --- | --- | --- | --- | --- | --- | --- | --- | --- | --- | --- | --- | --- | --- | --- | --- | --- | --- | --- | --- | --- | --- | --- | --- | --- | --- | --- | --- | --- | --- | --- | --- | --- | --- | --- | --- | --- | --- | --- | --- | --- | --- | --- | --- | --- | --- | --- | --- | --- | --- | --- | --- | --- | --- | --- | --- | --- | --- | --- | --- | --- | --- | --- | --- | --- | --- | --- | --- | --- | --- | --- | --- | --- | --- | --- | --- | --- | --- | --- | --- | --- | --- | --- | --- | --- | --- | --- | --- | --- | --- | --- | --- | --- | --- | --- | --- | --- | --- | --- | --- | --- | --- | --- | --- | --- | --- | --- | --- | --- | --- | --- | --- | --- | --- | --- | --- | --- | --- | --- | --- | --- | --- | --- | --- | --- | --- | --- | --- | --- | --- | --- | --- | --- | --- | --- | --- | --- | --- | --- | --- | --- | --- | --- | --- | --- | --- | --- | --- | --- | --- | --- | --- | --- | --- | --- | --- | --- | --- | --- | --- | --- | --- | --- | --- | --- | --- | --- | --- | --- | --- | --- | --- | --- | --- | --- | --- | --- | --- | --- | --- | --- | --- | --- | --- | --- | --- | --- | --- | --- | --- | --- | --- | --- | --- | --- | --- | --- | --- | --- | --- | --- | --- | --- | --- | --- | --- | --- | --- | --- | --- | --- | --- | --- | --- | --- | --- | --- | --- | --- | --- | --- | --- | --- | --- | --- | --- | --- | --- | --- | --- | --- | --- | --- | --- | --- | --- | --- | --- | --- | --- | --- | --- | --- | --- | --- | --- | --- | --- | --- | --- | --- | --- | --- | --- | --- | --- | --- | --- | --- | --- | --- | --- | --- | --- | --- | --- | --- | --- | --- | --- | --- | --- | --- | --- | --- | --- | --- | --- | --- | --- | --- | --- | --- | --- | --- | --- | --- | --- | --- | --- | --- | --- | --- | --- | --- | --- | --- | --- | --- | --- | --- | --- | --- | --- | --- | --- | --- | --- | --- | --- | --- | --- | --- | --- | --- | --- | --- | --- | --- | --- | --- | --- | --- | --- | --- | --- | --- | --- | --- | --- | --- | --- | --- | --- | --- | --- | --- | --- | --- | --- | --- | --- | --- | --- | --- | --- | --- | --- | --- | --- | --- | --- | --- | --- | --- | --- | --- | --- | --- | --- | --- | --- | --- | --- | --- | --- | --- | --- | --- | --- | --- | --- | --- | --- | --- | --- | --- | --- | --- | --- | --- | --- | --- | --- | --- | --- | --- | --- | --- | --- | --- | --- | --- | --- | --- | --- | --- | --- | --- | --- | --- | --- | --- | --- | --- | --- | --- | --- | --- | --- | --- | --- | --- | --- | --- | --- | --- | --- | --- | --- | --- | --- | --- | --- | --- | --- | --- | --- | --- | --- | --- | --- | --- | --- | --- | --- | --- | --- | --- | --- | --- | --- | --- | --- | --- | --- | --- | --- | --- | --- | --- | --- | --- | --- | --- | --- | --- | --- | --- | --- | --- | --- | --- | --- | --- | --- | --- | --- | --- | --- | --- | --- | --- | --- | --- | --- | --- | --- | --- | --- | --- | --- | --- | --- | --- | --- | --- | --- | --- | --- | --- | --- | --- | --- | --- | --- | --- | --- | --- | --- | --- | --- | --- | --- | --- | --- | --- | --- | --- | --- | --- | --- | --- | --- | --- | --- | --- | --- | --- | --- | --- | --- | --- | --- | --- | --- | --- | --- | --- | --- | --- | --- | --- | --- | --- | --- | --- | --- | --- | --- | --- | --- | --- | --- | --- | --- | --- | --- | --- | --- | --- | --- | --- | --- | --- | --- | --- | --- | --- | --- | --- | --- | --- | --- | --- | --- | --- | --- | --- | --- | --- | --- | --- | --- | --- | --- | --- | --- | --- | --- | --- | --- | --- | --- | --- | --- | --- | --- | --- | --- | --- | --- | --- | --- | --- | --- | --- | --- | --- | --- | --- | --- | --- | --- | --- | --- | --- | --- | --- | --- | --- | --- | --- | --- | --- | --- | --- | --- | --- | --- | --- | --- | --- | --- | --- | --- | --- | --- | --- | --- | --- | --- | --- | --- | --- | --- | --- | --- | --- | --- | --- | --- | --- | --- | --- | --- | --- | --- | --- | --- | --- | --- | --- | --- | --- | --- | --- | --- | --- | --- | --- | --- | --- | --- | --- | --- | --- | --- | --- | --- | --- | --- | --- | --- | --- | --- | --- | --- | --- | --- | --- | --- | --- | --- | --- | --- | --- | --- | --- | --- | --- | --- | --- | --- | --- | --- | --- | --- | --- | --- | --- | --- | --- | --- | --- | --- | --- | --- | --- | --- | --- | --- | --- | --- | --- | --- | --- | --- | --- | --- | --- | --- | --- | --- | --- | --- | --- | --- | --- | --- | --- | --- | --- | --- | --- | --- | --- | --- | --- | --- | --- | --- | --- | --- | --- | --- | --- | --- | --- | --- | --- | --- | --- | --- | --- | --- | --- | --- | --- | --- | --- | --- | --- | --- | --- | --- | --- | --- | --- | --- | --- | --- | --- | --- | --- | --- | --- | --- | --- | --- | --- | --- | --- | --- | --- | --- | --- | --- | --- | --- | --- | --- | --- | --- | --- | --- | --- | --- | --- | --- | --- | --- | --- | --- | --- | --- | --- | --- | --- | --- | --- | --- | --- | --- | --- | --- | --- | --- | --- | --- | --- | --- | --- | --- | --- | --- | --- | --- | --- | --- | --- | --- | --- | --- | --- | --- | --- | --- | --- | --- | --- | --- | --- | --- | --- | --- | --- | --- | --- | --- | --- | --- | --- | --- | --- | --- | --- | --- | --- | --- | --- | --- | --- | --- | --- | --- | --- | --- | --- | --- | --- | --- | --- | --- | --- | --- | --- | --- | --- | --- | --- | --- | --- | --- | --- | --- | --- | --- | --- | --- | --- | --- | --- | --- | --- | --- | --- | --- | --- | --- | --- | --- | --- | --- | --- | --- | --- | --- | --- | --- | --- | --- | --- | --- | --- | --- | --- | --- | --- | --- | --- | --- | --- | --- | --- | --- | --- | --- | --- | --- | --- | --- | --- | --- | --- | --- | --- | --- | --- | --- | --- | --- | --- | --- | --- | --- | --- | --- | --- | --- | --- | --- | --- | --- | --- | --- | --- | --- | --- | --- | --- | --- | --- | --- | --- | --- | --- | --- | --- | --- | --- | --- | --- | --- | --- | --- | --- | --- | --- | --- | --- | --- | --- | --- | --- | --- | --- | --- | --- | --- | --- | --- | --- | --- | --- | --- | --- | --- | --- | --- | --- | --- | --- | --- | --- | --- | --- | --- | --- | --- | --- | --- | --- | --- | --- | --- | --- | --- | --- | --- | --- | --- | --- | --- | --- | --- | --- | --- | --- | --- | --- | --- | --- | --- | --- | --- | --- | --- | --- | --- | --- | --- | --- | --- | --- | --- | --- | --- | --- | --- | --- | --- | --- | --- | --- | --- | --- | --- | --- | --- | --- | --- | --- | --- | --- | --- | --- | --- | --- | --- | --- | --- | --- | --- | --- | --- | --- | --- | --- | --- | --- | --- | --- | --- | --- | --- | --- | --- | --- | --- | --- | --- | --- | --- | --- | --- | --- | --- | --- | --- | --- | --- | --- | --- | --- | --- | --- | --- | --- | --- | --- | --- | --- | --- | --- | --- | --- | --- | --- | --- | --- | --- | --- | --- | --- | --- | --- | --- | --- | --- | --- | --- | --- | --- | --- | --- | --- | --- | --- | --- | --- | --- | --- | --- | --- | --- | --- | --- | --- | --- | --- | --- | --- | --- | --- | --- | --- | --- | --- | --- | --- | --- | --- | --- | --- | --- | --- | --- | --- | --- | --- | --- | --- | --- | --- | --- | --- | --- | --- | --- | --- | --- | --- | --- | --- | --- | --- | --- | --- | --- | --- | --- | --- | --- | --- | --- | --- | --- | --- | --- | --- | --- | --- | --- | --- | --- | --- | --- | --- | --- | --- | --- | --- | --- | --- | --- | --- | --- | --- | --- | --- | --- | --- | --- | --- | --- | --- | --- | --- | --- | --- | --- | --- | --- | --- | --- | --- | --- | --- | --- | --- | --- | --- | --- | --- | --- | --- | --- | --- | --- | --- | --- | --- | --- | --- | --- | --- | --- | --- | --- | --- | --- | --- | --- | --- | --- | --- | --- | --- | --- | --- | --- | --- | --- | --- | --- | --- | --- | --- | --- | --- | --- | --- | --- | --- | --- | --- | --- | --- | --- | --- | --- | --- | --- | --- | --- | --- | --- | --- | --- | --- | --- | --- | --- | --- | --- | --- | --- | --- | --- | --- | --- | --- | --- | --- | --- | --- | --- | --- | --- | --- | --- | --- | --- | --- | --- | --- | --- | --- | --- | --- | --- | --- | --- | --- | --- | --- | --- | --- | --- | --- | --- | --- | --- | --- | --- | --- | --- | --- | --- | --- | --- | --- | --- | --- | --- | --- | --- | --- | --- | --- | --- | --- | --- | --- | --- | --- | --- | --- | --- | --- | --- | --- | --- | --- | --- | --- | --- | --- | --- | --- | --- | --- | --- | --- | --- | --- | --- | --- | --- | --- | --- | --- | --- | --- | --- | --- | --- | --- | --- | --- | --- | --- | --- | --- | --- | --- | --- | --- | --- | --- | --- | --- | --- | --- | --- | --- | --- | --- | --- | --- | --- | --- | --- | --- | --- | --- | --- | --- | --- | --- | --- | --- | --- | --- | --- | --- | --- | --- | --- | --- | --- | --- | --- | --- | --- | --- | --- | --- | --- | --- | --- | --- | --- | --- | --- | --- | --- | --- | --- | --- | --- | --- | --- | --- | --- | --- | --- | --- | --- | --- | --- | --- | --- | --- | --- | --- | --- | --- | --- | --- | --- | --- | --- | --- | --- | --- | --- | --- | --- | --- | --- | --- | --- | --- | --- | --- | --- | --- | --- | --- | --- | --- | --- | --- | --- | --- | --- | --- | --- | --- | --- | --- | --- | --- | --- | --- | --- | --- | --- | --- | --- | --- | --- | --- | --- | --- | --- | --- | --- | --- | --- | --- | --- | --- | --- | --- | --- | --- | --- | --- | --- | --- | --- | --- | --- | --- | --- | --- | --- | --- | --- | --- | --- | --- | --- | --- | --- | --- | --- | --- | --- | --- | --- | --- | --- | --- | --- | --- | --- | --- | --- | --- | --- | --- | --- | --- | --- | --- | --- | --- | --- | --- | --- | --- | --- | --- | --- | --- | --- | --- | --- | --- | --- | --- | --- | --- | --- | --- | --- | --- | --- | --- | --- | --- | --- | --- | --- | --- | --- | --- | --- | --- | --- | --- | --- | --- | --- | --- | --- | --- | --- | --- | --- | --- | --- | --- | --- | --- | --- | --- | --- | --- | --- | --- | --- | --- | --- | --- | --- | --- | --- | --- | --- | --- | --- | --- | --- | --- | --- | --- | --- | --- | --- | --- | --- | --- | --- | --- | --- | --- | --- | --- | --- | --- | --- | --- | --- | --- | --- | --- | --- | --- | --- | --- | --- | --- | --- | --- | --- | --- | --- | --- | --- | --- | --- | --- | --- | --- | --- | --- | --- | --- | --- | --- | --- | --- | --- | --- | --- | --- | --- | --- | --- | --- | --- | --- | --- | --- | --- | --- | --- | --- | --- | --- | --- | --- | --- | --- | --- | --- | --- | --- | --- | --- | --- | --- | --- | --- | --- | --- | --- | --- | --- | --- | --- | --- | --- | --- | --- | --- | --- | --- | --- | --- | --- | --- | --- | --- | --- | --- | --- | --- | --- | --- | --- | --- | --- | --- | --- | --- | --- | --- | --- | --- | --- | --- | --- | --- | --- | --- | --- | --- | --- | --- | --- | --- | --- | --- | --- | --- | --- | --- | --- | --- | --- | --- | --- | --- | --- | --- | --- | --- | --- | --- | --- | --- | --- | --- | --- | --- | --- | --- | --- | --- | --- | --- | --- | --- | --- | --- | --- | --- | --- | --- | --- | --- | --- | --- | --- | --- | --- | --- | --- | --- | --- | --- | --- | --- | --- | --- | --- | --- | --- | --- | --- | --- | --- | --- | --- | --- | --- | --- | --- | --- | --- | --- | --- | --- | --- | --- | --- | --- | --- | --- | --- | --- | --- | --- | --- | --- | --- | --- | --- | --- | --- | --- | --- | --- | --- | --- | --- | --- | --- | --- | --- | --- | --- | --- | --- | --- | --- | --- | --- | --- | --- | --- | --- | --- | --- | --- | --- | --- | --- | --- | --- | --- | --- | --- | --- | --- | --- | --- | --- | --- | --- | --- | --- | --- | --- | --- | --- | --- | --- | --- | --- | --- | --- | --- | --- | --- | --- | --- | --- | --- | --- | --- | --- | --- | --- | --- | --- | --- | --- | --- | --- | --- | --- | --- | --- | --- | --- | --- | --- | --- | --- | --- | --- | --- | --- | --- | --- | --- | --- | --- | --- | --- | --- | --- | --- | --- | --- | --- | --- | --- | --- | --- | --- | --- | --- | --- | --- | --- | --- | --- | --- | --- | --- | --- | --- | --- | --- | --- | --- | --- | --- | --- | --- | --- | --- | --- | --- | --- | --- | --- | --- | --- | --- | --- | --- | --- | --- | --- | --- | --- | --- | --- | --- | --- | --- | --- | --- | --- | --- | --- | --- | --- | --- | --- | --- | --- | --- | --- | --- | --- | --- | --- | --- | --- | --- | --- | --- | --- | --- | --- | --- | --- | --- | --- | --- | --- | --- | --- | --- | --- | --- | --- | --- | --- | --- | --- | --- | --- | --- | --- | --- | --- | --- | --- | --- | --- | --- | --- | --- | --- | --- | --- | --- | --- | --- | --- | --- | --- | --- | --- | --- | --- | --- | --- | --- | --- | --- | --- | --- | --- | --- | --- | --- | --- | --- | --- | --- | --- | --- | --- | --- | --- | --- | --- | --- | --- | --- | --- | --- | --- | --- | --- | --- | --- | --- | --- | --- | --- | --- | --- | --- | --- | --- | --- | --- | --- | --- | --- | --- | --- | --- | --- | --- | --- | --- | --- | --- | --- | --- | --- | --- | --- | --- | --- | --- | --- | --- | --- | --- | --- | --- | --- | --- | --- | --- | --- | --- | --- | --- | --- | --- | --- | --- | --- | --- | --- | --- | --- | --- | --- | --- | --- | --- | --- | --- | --- | --- | --- | --- | --- | --- | --- | --- | --- | --- | --- | --- | --- | --- | --- | --- | --- | --- | --- | --- | --- | --- | --- | --- | --- | --- | --- | --- | --- | --- | --- | --- | --- | --- | --- | --- | --- | --- | --- | --- | --- | --- | --- | --- | --- | --- | --- | --- | --- | --- | --- | --- | --- | --- | --- | --- | --- | --- | --- | --- | --- | --- | --- | --- | --- | --- | --- | --- | --- | --- | --- | --- | --- | --- | --- | --- | --- | --- | --- | --- | --- | --- | --- | --- | --- | --- | --- | --- | --- | --- | --- | --- | --- | --- | --- | --- | --- | --- | --- | --- | --- | --- | --- | --- | --- | --- | --- | --- | --- | --- | --- | --- | --- | --- | --- | --- | --- | --- | --- | --- | --- | --- | --- | --- | --- | --- | --- | --- | --- | --- | --- | --- | --- | --- | --- | --- | --- | --- | --- | --- | --- | --- | --- | --- | --- | --- | --- | --- | --- | --- | --- | --- | --- | --- | --- | --- | --- | --- | --- | --- | --- | --- | --- | --- | --- | --- | --- | --- | --- | --- | --- | --- | --- | --- | --- | --- | --- | --- | --- | --- | --- | --- | --- | --- | --- | --- | --- | --- | --- | --- | --- | --- | --- | --- | --- | --- | --- | --- | --- | --- | --- | --- | --- | --- | --- | --- | --- | --- | --- | --- | --- | --- | --- | --- | --- | --- | --- | --- | --- | --- | --- | --- | --- | --- | --- | --- | --- | --- | --- | --- | --- | --- | --- | --- | --- | --- | --- | --- | --- | --- | --- | --- | --- | --- | --- | --- | --- | --- | --- | --- | --- | --- | --- | --- | --- | --- | --- | --- | --- | --- | --- | --- | --- | --- | --- | --- | --- | --- | --- | --- | --- | --- | --- | --- | --- | --- | --- | --- | --- | --- | --- | --- | --- | --- | --- | --- | --- | --- | --- | --- | --- | --- | --- | --- | --- | --- | --- | --- | --- | --- | --- | --- | --- | --- | --- | --- | --- | --- | --- | --- | --- | --- | --- | --- | --- | --- | --- | --- | --- | --- | --- | --- | --- | --- | --- | --- | --- | --- | --- | --- | --- | --- | --- | --- | --- | --- | --- | --- | --- | --- | --- | --- | --- | --- | --- | --- | --- | --- | --- | --- | --- | --- | --- | --- | --- | --- | --- | --- | --- | --- | --- | --- | --- | --- | --- | --- | --- | --- | --- | --- | --- | --- | --- | --- | --- | --- | --- | --- | --- | --- | --- | --- | --- | --- | --- | --- | --- | --- | --- | --- | --- | --- | --- | --- | --- | --- | --- | --- | --- | --- | --- | --- | --- | --- | --- | --- | --- | --- | --- | --- | --- | --- | --- | --- | --- | --- | --- | --- | --- | --- | --- | --- | --- | --- | --- | --- | --- | --- | --- | --- | --- | --- | --- | --- | --- | --- | --- | --- | --- | --- | --- | --- | --- | --- | --- | --- | --- | --- | --- | --- | --- | --- | --- | --- | --- | --- | --- | --- | --- | --- | --- | --- | --- | --- | --- | --- | --- | --- | --- | --- | --- | --- | --- | --- | --- | --- | --- | --- | --- | --- | --- | --- | --- | --- | --- | --- | --- | --- | --- | --- | --- | --- | --- | --- | --- | --- | --- | --- | --- | --- | --- | --- | --- | --- | --- | --- | --- | --- | --- | --- | --- | --- | --- | --- | --- | --- | --- | --- | --- | --- | --- | --- | --- | --- | --- | --- | --- | --- | --- | --- | --- | --- | --- | --- | --- | --- | --- | --- | --- | --- | --- | --- | --- | --- | --- | --- | --- | --- | --- | --- | --- | --- | --- | --- | --- | --- | --- | --- | --- | --- | --- | --- | --- | --- | --- | --- | --- | --- | --- | --- | --- | --- | --- | --- | --- | --- | --- | --- | --- | --- | --- | --- | --- | --- | --- | --- | --- | --- | --- | --- | --- | --- | --- | --- | --- | --- | --- | --- | --- | --- | --- | --- | --- | --- | --- | --- | --- | --- | --- | --- | --- | --- | --- | --- | --- | --- | --- | --- | --- | --- | --- | --- | --- | --- | --- | --- | --- | --- | --- | --- | --- | --- | --- | --- | --- | --- | --- | --- | --- | --- | --- | --- | --- | --- | --- | --- | --- | --- | --- | --- | --- | --- | --- | --- | --- | --- | --- | --- | --- | --- | --- | --- | --- | --- | --- | --- | --- | --- | --- | --- | --- | --- | --- | --- | --- | --- | --- | --- | --- | --- | --- | --- | --- | --- | --- | --- | --- | --- | --- | --- | --- | --- | --- | --- | --- | --- | --- | --- | --- | --- | --- | --- | --- | --- | --- | --- | --- | --- | --- | --- | --- | --- | --- | --- | --- | --- | --- | --- | --- | --- | --- | --- | --- | --- | --- | --- | --- | --- | --- | --- | --- | --- | --- | --- | --- | --- | --- | --- | --- | --- | --- | --- | --- | --- | --- | --- | --- | --- | --- | --- | --- | --- | --- | --- | --- | --- | --- | --- | --- | --- | --- | --- | --- | --- | --- | --- | --- | --- | --- | --- | --- | --- | --- | --- | --- | --- | --- | --- | --- | --- | --- | --- | --- | --- | --- | --- | --- | --- | --- | --- | --- | --- | --- | --- | --- | --- | --- | --- | --- | --- | --- | --- | --- | --- | --- | --- | --- | --- | --- | --- | --- | --- | --- | --- | --- | --- | --- | --- | --- | --- | --- | --- | --- | --- | --- | --- | --- | --- | --- | --- | --- | --- | --- | --- | --- | --- | --- | --- | --- | --- | --- | --- | --- | --- | --- | --- | --- | --- | --- | --- | --- | --- | --- | --- | --- | --- | --- | --- | --- | --- | --- | --- | --- | --- | --- | --- | --- | --- | --- | --- | --- | --- | --- | --- | --- | --- | --- | --- | --- | --- | --- | --- | --- | --- | --- | --- | --- | --- | --- | --- | --- | --- | --- | --- | --- | --- | --- | --- | --- | --- | --- | --- | --- | --- | --- | --- | --- | --- | --- | --- | --- | --- | --- | --- | --- | --- | --- | --- | --- | --- | --- | --- | --- | --- | --- | --- | --- | --- | --- | --- | --- | --- | --- | --- | --- | --- | --- | --- | --- | --- | --- | --- | --- | --- | --- | --- | --- | --- | --- | --- | --- | --- | --- | --- | --- | --- | --- | --- | --- | --- | --- | --- | --- | --- | --- | --- | --- | --- | --- | --- | --- | --- | --- | --- | --- | --- | --- | --- | --- | --- | --- | --- | --- | --- | --- | --- | --- | --- | --- | --- | --- | --- | --- | --- | --- | --- | --- | --- | --- | --- | --- | --- | --- | --- | --- | --- | --- | --- | --- | --- | --- | --- | --- | --- | --- | --- | --- | --- | --- | --- | --- | --- | --- | --- | --- | --- | --- | --- | --- | --- | --- | --- | --- | --- | --- | --- | --- | --- | --- | --- | --- | --- | --- | --- | --- | --- | --- | --- | --- | --- | --- | --- | --- | --- | --- | --- | --- | --- | --- | --- | --- | --- | --- | --- | --- | --- | --- | --- | --- | --- | --- | --- | --- | --- | --- | --- | --- | --- | --- | --- | --- | --- | --- | --- | --- | --- | --- | --- | --- | --- | --- | --- | --- | --- | --- | --- | --- | --- | --- | --- | --- | --- | --- | --- | --- | --- | --- | --- | --- | --- | --- | --- | --- | --- | --- | --- | --- | --- | --- | --- | --- | --- | --- | --- | --- | --- | --- | --- | --- | --- | --- | --- | --- | --- | --- | --- | --- | --- | --- | --- | --- | --- | --- | --- | --- | --- | --- | --- | --- | --- | --- | --- | --- | --- | --- | --- | --- | --- | --- | --- | --- | --- | --- | --- | --- | --- | --- | --- | --- | --- | --- | --- | --- | --- | --- | --- | --- | --- | --- | --- | --- | --- | --- | --- | --- | --- | --- | --- | --- | --- | --- | --- | --- | --- | --- | --- | --- | --- | --- | --- | --- | --- | --- | --- | --- | --- | --- | --- | --- | --- | --- | --- | --- | --- | --- | --- | --- | --- | --- | --- | --- | --- | --- | --- | --- | --- | --- | --- | --- | --- | --- | --- | --- | --- | --- | --- | --- | --- | --- | --- | --- | --- | --- | --- | --- | --- | --- | --- | --- | --- | --- | --- | --- | --- | --- | --- | --- | --- | --- | --- | --- | --- | --- | --- | --- | --- | --- | --- | --- | --- | --- | --- | --- | --- | --- | --- | --- | --- | --- | --- | --- | --- | --- | --- | --- | --- | --- | --- | --- | --- | --- | --- | --- | --- | --- | --- | --- | --- | --- | --- | --- | --- | --- | --- | --- | --- | --- | --- | --- | --- | --- | --- | --- | --- | --- | --- | --- | --- | --- | --- | --- | --- | --- | --- | --- | --- | --- | --- | --- | --- | --- | --- | --- | --- | --- | --- | --- | --- | --- | --- | --- | --- | --- | --- | --- | --- | --- | --- | --- | --- | --- | --- | --- | --- | --- | --- | --- | --- | --- | --- | --- | --- | --- | --- | --- | --- | --- | --- | --- | --- | --- | --- | --- | --- | --- | --- | --- | --- | --- | --- | --- | --- | --- | --- | --- | --- | --- | --- | --- | --- | --- | --- | --- | --- | --- | --- | --- | --- | --- | --- | --- | --- | --- | --- | --- | --- | --- | --- | --- | --- | --- | --- | --- | --- | --- | --- | --- | --- | --- | --- | --- | --- | --- | --- | --- | --- | --- | --- | --- | --- | --- | --- | --- | --- | --- | --- | --- | --- | --- | --- | --- | --- | --- | --- | --- | --- | --- | --- | --- | --- | --- | --- | --- | --- | --- | --- | --- | --- | --- | --- | --- | --- | --- | --- | --- | --- | --- | --- | --- | --- | --- | --- | --- | --- | --- | --- | --- | --- | --- | --- | --- | --- | --- | --- | --- | --- | --- | --- | --- | --- | --- | --- | --- | --- | --- | --- | --- | --- | --- | --- | --- | --- | --- | --- | --- | --- | --- | --- | --- | --- | --- | --- | --- | --- | --- | --- | --- | --- | --- | --- | --- | --- | --- | --- | --- | --- | --- | --- | --- | --- | --- | --- | --- | --- | --- | --- | --- | --- | --- | --- | --- | --- | --- | --- | --- | --- | --- | --- | --- | --- | --- | --- | --- | --- | --- | --- | --- | --- | --- | --- | --- | --- | --- | --- | --- | --- | --- | --- | --- | --- | --- | --- | --- | --- | --- | --- | --- | --- | --- | --- | --- | --- | --- | --- | --- | --- | --- | --- | --- | --- | --- | --- | --- | --- | --- | --- | --- | --- | --- | --- | --- | --- | --- | --- | --- | --- | --- | --- | --- | --- | --- | --- | --- | --- | --- | --- | --- | --- | --- | --- | --- | --- | --- | --- | --- | --- | --- | --- | --- | --- | --- | --- | --- | --- | --- | --- | --- | --- | --- | --- | --- | --- | --- | --- | --- | --- | --- | --- | --- | --- | --- | --- | --- | --- | --- | --- | --- | --- | --- | --- | --- | --- | --- | --- | --- | --- | --- | --- | --- | --- | --- | --- | --- | --- | --- | --- | --- | --- | --- | --- | --- | --- | --- | --- | --- | --- | --- | --- | --- | --- | --- | --- | --- | --- | --- | --- | --- | --- | --- | --- | --- | --- | --- | --- | --- | --- | --- | --- | --- | --- | --- | --- | --- | --- | --- | --- | --- | --- | --- | --- | --- | --- | --- | --- | --- | --- | --- | --- | --- | --- | --- | --- | --- | --- | --- | --- | --- | --- | --- | --- | --- | --- | --- | --- | --- | --- | --- | --- | --- | --- | --- | --- | --- | --- | --- | --- | --- | --- | --- | --- | --- | --- | --- | --- | --- | --- | --- | --- | --- | --- | --- | --- | --- | --- | --- | --- | --- | --- | --- | --- | --- | --- | --- | --- | --- | --- | --- | --- | --- | --- | --- | --- | --- | --- | --- | --- | --- | --- | --- | --- | --- | --- | --- | --- | --- | --- | --- | --- | --- | --- | --- | --- | --- | --- | --- | --- | --- | --- | --- | --- | --- | --- | --- | --- | --- | --- | --- | --- | --- | --- | --- | --- | --- | --- | --- | --- | --- | --- | --- | --- | --- | --- | --- | --- | --- | --- | --- | --- | --- | --- | --- | --- | --- | --- | --- | --- | --- | --- | --- | --- | --- | --- | --- | --- | --- | --- | --- | --- | --- | --- | --- | --- | --- | --- | --- | --- | --- | --- | --- | --- | --- | --- | --- | --- | --- | --- | --- | --- | --- | --- | --- | --- | --- | --- | --- | --- | --- | --- | --- | --- | --- | --- | --- | --- | --- | --- | --- | --- | --- | --- | --- | --- | --- | --- | --- | --- | --- | --- | --- | --- | --- | --- | --- | --- | --- | --- | --- | --- | --- | --- | --- | --- | --- | --- | --- | --- | --- | --- | --- | --- | --- | --- | --- | --- | --- | --- | --- | --- | --- | --- | --- | --- | --- | --- | --- | --- | --- | --- | --- | --- | --- | --- | --- | --- | --- | --- | --- | --- | --- | --- | --- | --- | --- | --- | --- | --- | --- | --- | --- | --- | --- | --- | --- | --- | --- | --- | --- | --- | --- | --- | --- | --- | --- | --- | --- | --- | --- | --- | --- | --- | --- | --- | --- | --- | --- | --- | --- | --- | --- | --- | --- | --- | --- | --- | --- | --- | --- | --- | --- | --- | --- | --- | --- | --- | --- | --- | --- | --- | --- | --- | --- | --- | --- | --- | --- | --- | --- | --- | --- | --- | --- | --- | --- | --- | --- | --- | --- | --- | --- | --- | --- | --- | --- | --- | --- | --- | --- | --- | --- | --- | --- | --- | --- | --- | --- | --- | --- | --- | --- | --- | --- | --- | --- | --- | --- | --- | --- | --- | --- | --- | --- | --- | --- | --- | --- | --- | --- | --- | --- | --- | --- | --- | --- | --- | --- | --- | --- | --- | --- | --- | --- | --- | --- | --- | --- | --- | --- | --- | --- | --- | --- | --- | --- | --- | --- | --- | --- | --- | --- | --- | --- | --- | --- | --- | --- | --- | --- | --- | --- | --- | --- | --- | --- | --- | --- | --- | --- | --- | --- | --- | --- | --- | --- | --- | --- | --- | --- | --- | --- | --- | --- | --- | --- | --- | --- | --- | --- | --- | --- | --- | --- | --- | --- | --- | --- | --- | --- | --- | --- | --- | --- | --- | --- | --- | --- | --- | --- | --- | --- | --- | --- | --- | --- | --- | --- | --- | --- | --- | --- | --- | --- | --- | --- | --- | --- | --- | --- | --- | --- | --- | --- | --- | --- | --- | --- | --- | --- | --- | --- | --- | --- | --- | --- | --- | --- | --- | --- | --- | --- | --- | --- | --- | --- | --- | --- | --- | --- | --- | --- | --- | --- | --- | --- | --- | --- | --- | --- | --- | --- | --- | --- | --- | --- | --- | --- | --- | --- | --- | --- | --- | --- | --- | --- | --- | --- | --- | --- | --- | --- | --- | --- | --- | --- | --- | --- | --- | --- | --- | --- | --- | --- | --- | --- | --- | --- | --- | --- | --- | --- | --- | --- | --- | --- | --- | --- | --- | --- | --- | --- | --- | --- | --- | --- | --- | --- | --- | --- | --- | --- | --- | --- | --- | --- | --- | --- | --- | --- | --- | --- | --- | --- | --- | --- | --- | --- | --- | --- | --- | --- | --- | --- | --- | --- | --- | --- | --- | --- | --- | --- | --- | --- | --- | --- | --- | --- | --- | --- | --- | --- | --- | --- | --- | --- | --- | --- | --- | --- | --- | --- | --- | --- | --- | --- | --- | --- | --- | --- | --- | --- | --- | --- | --- | --- | --- | --- | --- | --- | --- | --- | --- | --- | --- | --- | --- | --- | --- | --- | --- | --- | --- | --- | --- | --- | --- | --- | --- | --- | --- | --- | --- | --- | --- | --- | --- | --- | --- | --- | --- | --- | --- | --- | --- | --- | --- | --- | --- | --- | --- | --- | --- | --- | --- | --- | --- | --- | --- | --- | --- | --- | --- | --- | --- | --- | --- | --- | --- | --- | --- | --- | --- | --- | --- | --- | --- | --- | --- | --- | --- | --- | --- | --- | --- | --- | --- | --- | --- | --- | --- | --- | --- | --- | --- | --- | --- | --- | --- | --- | --- | --- | --- | --- | --- | --- | --- | --- | --- | --- | --- | --- | --- | --- | --- | --- | --- | --- | --- | --- | --- | --- | --- | --- | --- | --- | --- | --- | --- | --- | --- | --- | --- | --- | --- | --- | --- | --- | --- | --- | --- | --- | --- | --- | --- | --- | --- | --- | --- | --- | --- | --- | --- | --- | --- | --- | --- | --- | --- | --- | --- | --- | --- | --- | --- | --- | --- | --- | --- | --- | --- | --- | --- | --- | --- | --- | --- | --- | --- | --- | --- | --- | --- | --- | --- | --- | --- | --- | --- | --- | --- | --- | --- | --- | --- | --- | --- | --- | --- | --- | --- | --- | --- | --- | --- | --- | --- | --- | --- | --- | --- | --- | --- | --- | --- | --- | --- | --- | --- | --- | --- | --- | --- | --- | --- | --- | --- | --- | --- | --- | --- | --- | --- | --- | --- | --- | --- | --- | --- | --- | --- | --- | --- | --- | --- | --- | --- | --- | --- | --- | --- | --- | --- | --- | --- | --- | --- | --- | --- | --- | --- | --- | --- | --- | --- | --- | --- | --- | --- | --- | --- | --- | --- | --- | --- | --- | --- | --- | --- | --- | --- | --- | --- | --- | --- | --- | --- | --- | --- | --- | --- | --- | --- | --- | --- | --- | --- | --- | --- | --- | --- | --- | --- | --- | --- | --- | --- | --- | --- | --- | --- | --- | --- | --- | --- | --- | --- | --- | --- | --- | --- | --- | --- | --- | --- | --- | --- | --- | --- | --- | --- | --- | --- | --- | --- | --- | --- | --- | --- | --- | --- | --- | --- | --- | --- | --- | --- | --- | --- | --- | --- | --- | --- | --- | --- | --- | --- | --- | --- | --- | --- | --- | --- | --- | --- | --- | --- | --- | --- | --- | --- | --- | --- | --- | --- | --- | --- | --- | --- | --- | --- | --- | --- | --- | --- | --- | --- | --- | --- | --- | --- | --- | --- | --- | --- | --- | --- | --- | --- | --- | --- | --- | --- | --- | --- | --- | --- | --- | --- | --- | --- | --- | --- | --- | --- | --- | --- | --- | --- | --- | --- | --- | --- | --- | --- | --- | --- | --- | --- | --- | --- | --- | --- | --- | --- | --- | --- | --- | --- | --- | --- | --- | --- | --- | --- | --- | --- | --- | --- | --- | --- | --- | --- | --- | --- | --- | --- | --- | --- | --- | --- | --- | --- | --- | --- | --- | --- | --- | --- | --- | --- | --- | --- | --- | --- | --- | --- | --- | --- | --- | --- | --- | --- | --- | --- | --- | --- | --- | --- | --- | --- | --- | --- | --- | --- | --- | --- | --- | --- | --- | --- | --- | --- | --- | --- | --- | --- | --- | --- | --- | --- | --- | --- | --- | --- | --- | --- | --- | --- | --- | --- | --- | --- | --- | --- | --- | --- | --- | --- | --- | --- | --- | --- | --- | --- | --- | --- | --- | --- | --- | --- | --- | --- | --- | --- | --- | --- | --- | --- | --- | --- | --- | --- | --- | --- | --- | --- | --- | --- | --- | --- | --- | --- | --- | --- | --- | --- | --- | --- | --- | --- | --- | --- | --- | --- | --- | --- | --- | --- | --- | --- | --- | --- | --- | --- | --- | --- | --- | --- | --- | --- | --- | --- | --- | --- | --- | --- | --- | --- | --- | --- | --- | --- | --- | --- | --- | --- | --- | --- | --- | --- | --- | --- | --- | --- | --- | --- | --- | --- | --- | --- | --- | --- | --- | --- | --- | --- | --- | --- | --- | --- | --- | --- | --- | --- | --- | --- | --- | --- | --- | --- | --- | --- | --- | --- | --- | --- | --- | --- | --- | --- | --- | --- | --- | --- | --- | --- | --- | --- | --- | --- | --- | --- | --- | --- | --- | --- | --- | --- | --- | --- | --- | --- | --- | --- | --- | --- | --- | --- | --- | --- | --- | --- | --- | --- | --- | --- | --- | --- | --- | --- | --- | --- | --- | --- | --- | --- | --- | --- | --- | --- | --- | --- | --- | --- | --- | --- | --- | --- | --- | --- | --- | --- | --- | --- | --- | --- | --- | --- | --- | --- | --- | --- | --- | --- | --- | --- | --- | --- | --- | --- | --- | --- | --- | --- | --- | --- | --- | --- | --- | --- | --- | --- | --- | --- | --- | --- | --- | --- | --- | --- | --- | --- | --- | --- | --- | --- | --- | --- | --- | --- | --- | --- | --- | --- | --- | --- | --- | --- | --- | --- | --- | --- | --- | --- | --- | --- | --- | --- | --- | --- | --- | --- | --- | --- | --- | --- | --- | --- | --- | --- | --- | --- | --- | --- | --- | --- | --- | --- | --- | --- | --- | --- | --- | --- | --- | --- | --- | --- | --- | --- | --- | --- | --- | --- | --- | --- | --- | --- | --- | --- | --- | --- | --- | --- | --- | --- | --- | --- | --- | --- | --- | --- | --- | --- | --- | --- | --- | --- | --- | --- | --- | --- | --- | --- | --- | --- | --- | --- | --- | --- | --- | --- | --- | --- | --- | --- | --- | --- | --- | --- | --- | --- | --- | --- | --- | --- | --- | --- | --- | --- | --- | --- | --- | --- | --- | --- | --- | --- | --- | --- | --- | --- | --- | --- | --- | --- | --- | --- | --- | --- | --- | --- | --- | --- | --- | --- | --- | --- | --- | --- | --- | --- | --- | --- | --- | --- | --- | --- | --- | --- | --- | --- | --- | --- | --- | --- | --- | --- | --- | --- | --- | --- | --- | --- | --- | --- | --- | --- | --- | --- | --- | --- | --- | --- | --- | --- | --- | --- | --- | --- | --- | --- | --- | --- | --- | --- | --- | --- | --- | --- | --- | --- | --- | --- | --- | --- | --- | --- | --- | --- | --- | --- | --- | --- | --- | --- | --- | --- | --- | --- | --- | --- | --- | --- | --- | --- | --- | --- | --- | --- | --- | --- | --- | --- | --- | --- | --- | --- | --- | --- | --- | --- | --- | --- | --- | --- | --- | --- | --- | --- | --- | --- | --- | --- | --- | --- | --- | --- | --- | --- | --- | --- | --- | --- | --- | --- | --- | --- | --- | --- | --- | --- | --- | --- | --- | --- | --- | --- | --- | --- | --- | --- | --- | --- | --- | --- | --- | --- | --- | --- | --- | --- | --- | --- | --- | --- | --- | --- | --- | --- | --- | --- | --- | --- | --- | --- | --- | --- | --- | --- | --- | --- | --- | --- | --- | --- | --- | --- | --- | --- | --- | --- | --- | --- | --- | --- | --- | --- | --- | --- | --- | --- | --- | --- | --- | --- | --- | --- | --- | --- | --- | --- | --- | --- | --- | --- | --- | --- | --- | --- | --- | --- | --- | --- | --- | --- | --- | --- | --- | --- | --- | --- | --- | --- | --- | --- | --- | --- | --- | --- | --- | --- | --- | --- | --- | --- | --- | --- | --- | --- | --- | --- | --- | --- | --- | --- | --- | --- | --- | --- | --- | --- | --- | --- | --- | --- | --- | --- | --- | --- | --- | --- | --- | --- | --- | --- | --- | --- | --- | --- | --- | --- | --- | --- | --- | --- | --- | --- | --- | --- | --- | --- | --- | --- | --- | --- | --- | --- | --- | --- | --- | --- | --- | --- | --- | --- | --- | --- | --- | --- | --- | --- | --- | --- | --- | --- | --- | --- | --- | --- | --- | --- | --- | --- | --- | --- | --- | --- | --- | --- | --- | --- | --- | --- | --- | --- | --- | --- | --- | --- | --- | --- | --- | --- | --- | --- | --- | --- | --- | --- | --- | --- | --- | --- | --- | --- | --- | --- | --- | --- | --- | --- | --- | --- | --- | --- | --- | --- | --- | --- | --- | --- | --- | --- | --- | --- | --- | --- | --- | --- | --- | --- | --- | --- | --- | --- | --- | --- | --- | --- | --- | --- | --- | --- | --- | --- | --- | --- | --- | --- | --- | --- | --- | --- | --- | --- | --- | --- | --- | --- | --- | --- | --- | --- | --- | --- | --- | --- | --- | --- | --- | --- | --- | --- | --- | --- | --- | --- | --- | --- | --- | --- | --- | --- | --- | --- | --- | --- | --- | --- | --- | --- | --- | --- | --- | --- | --- | --- | --- | --- | --- | --- | --- | --- | --- | --- | --- | --- | --- | --- | --- | --- | --- | --- | --- | --- | --- | --- | --- | --- | --- | --- | --- | --- | --- | --- | --- | --- | --- | --- | --- | --- | --- | --- | --- | --- | --- | --- | --- | --- | --- | --- | --- | --- | --- | --- | --- | --- | --- | --- | --- | --- | --- | --- | --- | --- | --- | --- | --- | --- | --- | --- | --- | --- | --- | --- | --- | --- | --- | --- | --- | --- | --- | --- | --- | --- | --- | --- | --- | --- | --- | --- | --- | --- | --- | --- | --- | --- | --- | --- | --- | --- | --- | --- | --- | --- | --- | --- | --- | --- | --- | --- | --- | --- | --- | --- | --- | --- | --- | --- | --- | --- | --- | --- | --- | --- | --- | --- | --- | --- | --- | --- | --- | --- | --- | --- | --- | --- | --- | --- | --- | --- | --- | --- | --- | --- | --- | --- | --- | --- | --- | --- | --- | --- | --- | --- | --- | --- | --- | --- | --- | --- | --- | --- | --- | --- | --- | --- | --- | --- | --- | --- | --- | --- | --- | --- | --- | --- | --- | --- | --- | --- | --- | --- | --- | --- | --- | --- | --- | --- | --- | --- | --- | --- | --- | --- | --- | --- | --- | --- | --- | --- | --- | --- | --- | --- | --- | --- | --- | --- | --- | --- | --- | --- | --- | --- | --- | --- | --- | --- | --- | --- | --- | --- | --- | --- | --- | --- | --- | --- | --- | --- | --- | --- | --- | --- | --- | --- | --- | --- | --- | --- | --- | --- | --- | --- | --- | --- | --- | --- | --- | --- | --- | --- | --- | --- | --- | --- | --- | --- | --- | --- | --- | --- | --- | --- | --- | --- | --- | --- | --- | --- | --- | --- | --- | --- | --- | --- | --- | --- | --- | --- | --- | --- | --- | --- | --- | --- | --- | --- | --- | --- | --- | --- | --- | --- | --- | --- | --- | --- | --- | --- | --- | --- | --- | --- | --- | --- | --- | --- | --- | --- | --- | --- | --- | --- | --- | --- | --- | --- | --- | --- | --- | --- | --- | --- | --- | --- | --- | --- | --- | --- | --- | --- | --- | --- | --- | --- | --- | --- | --- | --- | --- | --- | --- | --- | --- | --- | --- | --- | --- | --- | --- | --- | --- | --- | --- | --- | --- | --- | --- | --- | --- | --- | --- | --- | --- | --- | --- | --- | --- | --- | --- | --- | --- | --- | --- | --- | --- | --- | --- | --- | --- | --- | --- | --- | --- | --- | --- | --- | --- | --- | --- | --- | --- | --- | --- | --- | --- | --- | --- | --- | --- | --- | --- | --- | --- | --- | --- | --- | --- | --- | --- | --- | --- | --- | --- | --- | --- | --- | --- | --- | --- | --- | --- | --- | --- | --- | --- | --- | --- | --- | --- | --- | --- | --- | --- | --- | --- | --- | --- | --- | --- | --- | --- | --- | --- | --- | --- | --- | --- | --- | --- | --- | --- | --- | --- | --- | --- | --- | --- | --- | --- | --- | --- | --- | --- | --- | --- | --- | --- | --- | --- | --- | --- | --- | --- | --- | --- | --- | --- | --- | --- | --- | --- | --- | --- | --- | --- | --- | --- | --- | --- | --- | --- | --- | --- | --- | --- | --- | --- | --- | --- | --- | --- | --- | --- | --- | --- | --- | --- | --- | --- | --- | --- | --- | --- | --- | --- | --- | --- | --- | --- | --- | --- | --- | --- | --- | --- | --- | --- | --- | --- | --- | --- | --- | --- | --- | --- | --- | --- | --- | --- | --- | --- | --- | --- | --- | --- | --- | --- | --- | --- | --- | --- | --- | --- | --- | --- | --- | --- | --- | --- | --- | --- | --- | --- | --- | --- | --- | --- | --- | --- | --- | --- | --- | --- | --- | --- | --- | --- | --- | --- | --- | --- | --- | --- | --- | --- | --- | --- | --- | --- | --- | --- | --- | --- | --- | --- | --- | --- | --- | --- | --- | --- | --- | --- | --- | --- | --- | --- | --- | --- | --- | --- | --- | --- | --- | --- | --- | --- | --- | --- | --- | --- | --- | --- | --- | --- | --- | --- | --- | --- | --- | --- | --- | --- | --- | --- | --- | --- | --- | --- | --- | --- | --- | --- | --- | --- | --- | --- | --- | --- | --- | --- | --- | --- | --- | --- | --- | --- | --- | --- | --- | --- | --- | --- | --- | --- | --- | --- | --- | --- | --- | --- | --- | --- | --- | --- | --- | --- | --- | --- | --- | --- | --- | --- | --- | --- | --- | --- | --- | --- | --- | --- | --- | --- | --- | --- | --- | --- | --- | --- | --- | --- | --- | --- | --- | --- | --- | --- | --- | --- | --- | --- | --- | --- | --- | --- | --- | --- | --- | --- | --- | --- | --- | --- | --- | --- | --- | --- | --- | --- | --- | --- | --- | --- | --- | --- | --- | --- | --- | --- | --- | --- | --- | --- | --- | --- | --- | --- | --- | --- | --- | --- | --- | --- | --- | --- | --- | --- | --- | --- | --- | --- | --- | --- | --- | --- | --- | --- | --- | --- | --- | --- | --- | --- | --- | --- | --- | --- | --- | --- | --- | --- | --- | --- | --- | --- | --- | --- | --- | --- | --- | --- | --- | --- | --- | --- | --- | --- | --- | --- | --- | --- | --- | --- | --- | --- | --- | --- | --- | --- | --- | --- | --- | --- | --- | --- | --- | --- | --- | --- | --- | --- | --- | --- | --- | --- | --- | --- | --- | --- | --- | --- | --- | --- | --- | --- | --- | --- | --- | --- | --- | --- | --- | --- | --- | --- | --- | --- | --- | --- | --- | --- | --- | --- | --- | --- | --- | --- | --- | --- | --- | --- | --- | --- | --- | --- | --- | --- | --- | --- | --- | --- | --- | --- | --- | --- | --- | --- | --- | --- | --- | --- | --- | --- | --- | --- | --- | --- | --- | --- | --- | --- | --- | --- | --- | --- | --- | --- | --- | --- | --- | --- | --- | --- | --- | --- | --- | --- | --- | --- | --- | --- | --- | --- | --- | --- | --- | --- | --- | --- | --- | --- | --- | --- | --- | --- | --- | --- | --- | --- | --- | --- | --- | --- | --- | --- | --- | --- | --- | --- | --- | --- | --- | --- | --- | --- | --- | --- | --- | --- | --- | --- | --- | --- | --- | --- | --- | --- | --- | --- | --- | --- | --- | --- | --- | --- | --- | --- | --- | --- | --- | --- | --- | --- | --- | --- | --- | --- | --- | --- | --- | --- | --- | --- | --- | --- | --- | --- | --- | --- | --- | --- | --- | --- | --- | --- | --- | --- | --- | --- | --- | --- | --- | --- | --- | --- | --- | --- | --- | --- | --- | --- | --- | --- | --- | --- | --- | --- | --- | --- | --- | --- | --- | --- | --- | --- | --- | --- | --- | --- | --- | --- | --- | --- | --- | --- | --- | --- | --- | --- | --- | --- | --- | --- | --- | --- | --- | --- | --- | --- | --- | --- | --- | --- | --- | --- | --- | --- | --- | --- | --- | --- | --- | --- | --- | --- | --- | --- | --- | --- | --- | --- | --- | --- | --- | --- | --- | --- | --- | --- | --- | --- | --- | --- | --- | --- | --- | --- | --- | --- | --- | --- | --- | --- | --- | --- | --- | --- | --- | --- | --- | --- | --- | --- | --- | --- | --- | --- | --- | --- | --- | --- | --- | --- | --- | --- | --- | --- | --- | --- | --- | --- | --- | --- | --- | --- | --- | --- | --- | --- | --- | --- | --- | --- | --- | --- | --- | --- | --- | --- | --- | --- | --- | --- | --- | --- | --- | --- | --- | --- | --- | --- | --- | --- | --- | --- | --- | --- | --- | --- | --- | --- | --- | --- | --- | --- | --- | --- | --- | --- | --- | --- | --- | --- | --- | --- | --- | --- | --- | --- | --- | --- | --- | --- | --- | --- | --- | --- | --- | --- | --- | --- | --- | --- | --- | --- | --- | --- | --- | --- | --- | --- | --- | --- | --- | --- | --- | --- | --- | --- | --- | --- | --- | --- | --- | --- | --- | --- | --- | --- | --- | --- | --- | --- | --- | --- | --- | --- | --- | --- | --- | --- | --- | --- | --- | --- | --- | --- | --- | --- | --- | --- | --- | --- | --- | --- | --- | --- | --- | --- | --- | --- | --- | --- | --- | --- | --- | --- | --- | --- | --- | --- | --- | --- | --- | --- | --- | --- | --- | --- | --- | --- | --- | --- | --- | --- | --- | --- | --- | --- | --- | --- | --- | --- | --- | --- | --- | --- | --- | --- | --- | --- | --- | --- | --- | --- | --- | --- | --- | --- | --- | --- | --- | --- | --- | --- | --- | --- | --- | --- | --- | --- | --- | --- | --- | --- | --- | --- | --- | --- | --- | --- | --- | --- | --- | --- | --- | --- | --- | --- | --- | --- | --- | --- | --- | --- | --- | --- | --- | --- | --- | --- | --- | --- | --- | --- | --- | --- | --- | --- | --- | --- | --- | --- | --- | --- | --- | --- | --- | --- | --- | --- | --- | --- | --- | --- | --- | --- | --- | --- | --- | --- | --- | --- | --- | --- | --- | --- | --- | --- | --- | --- | --- | --- | --- | --- | --- | --- | --- | --- | --- | --- | --- | --- | --- | --- | --- | --- | --- | --- | --- | --- | --- | --- | --- | --- | --- | --- | --- | --- | --- | --- | --- | --- | --- | --- | --- | --- | --- | --- | --- | --- | --- | --- | --- | --- | --- | --- | --- | --- | --- | --- | --- | --- | --- | --- | --- | --- | --- | --- | --- | --- | --- | --- | --- | --- | --- | --- | --- | --- | --- | --- | --- | --- | --- | --- | --- | --- | --- | --- | --- | --- | --- | --- | --- | --- | --- | --- | --- | --- | --- | --- | --- | --- | --- | --- | --- | --- | --- | --- | --- | --- | --- | --- | --- | --- | --- | --- | --- | --- | --- | --- | --- | --- | --- | --- | --- | --- | --- | --- | --- | --- | --- | --- | --- | --- | --- | --- | --- | --- | --- | --- | --- | --- | --- | --- | --- | --- | --- | --- | --- | --- | --- | --- | --- | --- | --- | --- | --- | --- | --- | --- | --- | --- | --- | --- | --- | --- | --- | --- | --- | --- | --- | --- | --- | --- | --- | --- | --- | --- | --- | --- | --- | --- | --- | --- | --- | --- | --- | --- | --- | --- | --- | --- | --- | --- | --- | --- | --- | --- | --- | --- | --- | --- | --- | --- | --- | --- | --- | --- | --- | --- | --- | --- | --- | --- | --- | --- | --- | --- | --- | --- | --- | --- | --- | --- | --- | --- | --- | --- | --- | --- | --- | --- | --- | --- | --- | --- | --- | --- | --- | --- | --- | --- | --- | --- | --- | --- | --- | --- | --- | --- | --- | --- | --- | --- | --- | --- | --- | --- | --- | --- | --- | --- | --- | --- | --- | --- | --- | --- | --- | --- | --- | --- | --- | --- | --- | --- | --- | --- | --- | --- | --- | --- | --- | --- | --- | --- | --- | --- | --- | --- | --- | --- | --- | --- | --- | --- | --- | --- | --- | --- | --- | --- | --- | --- | --- | --- | --- | --- | --- | --- | --- | --- | --- | --- | --- | --- | --- | --- | --- | --- | --- | --- | --- | --- | --- | --- | --- | --- | --- | --- | --- | --- | --- | --- | --- | --- | --- | --- | --- | --- | --- | --- | --- | --- | --- | --- | --- | --- | --- | --- | --- | --- | --- | --- | --- | --- | --- | --- | --- | --- | --- | --- | --- | --- | --- | --- | --- | --- | --- | --- | --- | --- | --- | --- | --- | --- | --- | --- | --- | --- | --- | --- | --- | --- | --- | --- | --- | --- | --- | --- | --- | --- | --- | --- | --- | --- | --- | --- | --- | --- | --- | --- | --- | --- | --- | --- | --- | --- | --- | --- | --- | --- | --- | --- | --- | --- | --- | --- | --- | --- | --- | --- | --- | --- | --- | --- | --- | --- | --- | --- | --- | --- | --- | --- | --- | --- | --- | --- | --- | --- | --- | --- | --- | --- | --- | --- | --- | --- | --- | --- | --- | --- | --- | --- | --- | --- | --- | --- | --- | --- | --- | --- | --- | --- | --- | --- | --- | --- | --- | --- |
| |  |  |  |  |  |  |  |  |  |  |  |  |  |  |  |  |  |  |  |  |  |  |  |  |  |  |  |  |  |  |  |  |  |  |  |  |  |  |  |  |  |  |  |  |  |  |  |  |  |  |  |  | | --- | --- | --- | --- | --- | --- | --- | --- | --- | --- | --- | --- | --- | --- | --- | --- | --- | --- | --- | --- | --- | --- | --- | --- | --- | --- | --- | --- | --- | --- | --- | --- | --- | --- | --- | --- | --- | --- | --- | --- | --- | --- | --- | --- | --- | --- | --- | --- | --- | --- | --- | --- | | G0VCI3/1-1859 | 1 | M | T | S | K | V | S | R | L | D | R | Q | V | I | L | L | E | T | G | S | T | Q | I | V | R | N | V | A | A | D | Q | M | G | D | L | A | K | Q | H | P | E | D | I | L | N | L | L | S | R | V | 49 | | Q6CM16/1-1873 | 1 | M | S | S | Q | E | S | R | L | D | R | Q | V | I | L | L | E | T | G | S | T | Q | V | V | R | N | M | A | A | D | Q | L | G | D | L | A | K | Q | H | P | E | Q | T | L | P | L | L | S | R | V | 49 | | Q6FRV8/1-1904 | 1 | M | T | S | K | V | S | R | L | D | R | Q | V | I | L | L | E | T | G | S | T | Q | T | I | R | N | V | A | A | D | Q | L | G | E | L | A | K | Q | H | P | E | D | I | L | S | L | L | S | R | V | 49 | | Q758L7/1-1866 | 1 | M | A | S | Q | A | S | R | L | D | R | H | V | I | L | L | E | S | G | S | T | Q | V | V | R | N | V | A | A | D | Q | L | G | D | L | A | R | Q | H | P | D | E | I | L | V | L | L | S | R | V | 49 | | A7TGL6/1-1902 | 1 | M | T | S | Q | V | S | R | L | D | R | Q | V | I | L | L | E | T | G | S | T | Q | V | V | R | S | M | A | A | D | Q | L | G | D | L | A | K | Q | H | P | E | D | I | L | L | L | L | S | R | V | 49 | | C5DII9/1-1880 | 1 | M | T | A | Q | L | S | R | L | D | R | Q | V | I | L | L | E | T | G | S | T | Q | V | V | R | N | M | A | A | D | Q | L | G | D | L | A | K | Q | H | P | E | D | T | L | S | L | L | S | R | V | 49 | | C5DP10/1-1883 | 1 | M | T | S | Q | V | S | R | L | D | R | Q | V | I | L | L | E | T | G | S | T | Q | F | V | R | N | M | A | A | D | Q | M | G | D | L | A | K | E | H | P | E | D | I | L | S | L | L | S | R | V | 49 | | Kwal\_YGOB\_27.12097/1-1546 | 1 | M | S | V | Q | L | S | R | L | D | R | Q | V | I | L | L | E | T | G | S | T | Q | V | V | R | N | M | A | A | D | Q | L | G | D | L | A | K | Q | H | P | E | D | T | L | S | L | L | S | R | V | 49 | | Sbay\_632.22/1-1867 | 1 | M | T | S | R | V | S | R | L | D | R | Q | V | I | L | I | E | T | G | S | T | Q | V | V | R | N | M | A | A | D | Q | M | G | D | L | A | K | Q | H | P | E | D | I | L | S | L | L | S | R | V | 49 | | SAKL0H09724g/1-1862 | 1 | M | T | S | Q | L | S | R | L | D | R | Q | V | I | L | L | E | N | G | S | T | Q | V | V | R | N | V | A | A | D | Q | L | G | D | L | A | K | Q | H | P | E | E | T | L | S | L | L | S | R | V | 49 | | P32333/1-1867 | 1 | M | T | S | R | V | S | R | L | D | R | Q | V | I | L | I | E | T | G | S | T | Q | V | V | R | N | M | A | A | D | Q | M | G | D | L | A | K | Q | H | P | E | D | I | L | S | L | L | S | R | V | 49 | |  | | G0VCI3/1-1859 | 50 | Y | P | F | L | L | V | K | K | W | E | T | R | V | T | A | A | R | A | V | G | G | I | V | K | H | A | T | L | W | D | P | N | E | N | D | - | - | - | D | T | A | M | K | P | T | D | - | - | - | 92 | | Q6CM16/1-1873 | 50 | Y | P | Y | L | L | S | K | Q | W | E | T | R | V | T | A | A | R | A | V | G | G | I | V | S | H | A | P | L | W | D | P | N | A | D | D | - | - | - | E | E | S | K | N | P | Q | - | - | - | - | 91 | | Q6FRV8/1-1904 | 50 | Y | P | F | L | S | S | K | K | W | E | T | R | V | T | A | A | R | A | M | G | G | I | L | S | N | A | S | V | W | D | P | N | E | G | E | V | K | E | E | E | E | S | H | M | N | S | E | I | K | 98 | | Q758L7/1-1866 | 50 | Y | P | Y | L | L | S | R | R | W | E | T | R | V | T | A | A | R | A | V | G | A | I | V | A | H | A | R | L | W | D | P | N | G | E | G | - | - | - | - | - | - | - | - | - | - | - | - | - | - | 84 | | A7TGL6/1-1902 | 50 | Y | P | Y | L | L | S | K | K | W | E | T | R | V | T | A | A | R | A | V | G | G | I | V | A | N | A | E | I | W | N | P | N | K | D | D | - | - | - | E | D | N | N | T | I | D | I | N | V | K | 95 | | C5DII9/1-1880 | 50 | Y | P | F | L | M | A | K | K | W | E | T | R | I | T | T | A | R | A | V | G | G | I | V | S | H | S | P | S | W | D | P | N | E | D | E | - | - | - | V | Q | V | T | G | D | D | N | G | D | E | 95 | | C5DP10/1-1883 | 50 | Y | P | F | L | L | V | K | K | W | E | T | R | V | T | A | A | R | A | V | G | G | I | V | C | H | A | P | V | W | D | P | N | E | N | D | - | - | - | G | E | I | K | T | E | D | - | - | - | - | 91 | | Kwal\_YGOB\_27.12097/1-1546 | 50 | Y | P | F | L | M | A | K | K | W | E | T | R | I | T | T | A | R | A | V | G | G | I | V | S | H | S | P | S | W | D | P | N | E | D | D | - | - | - | - | - | - | - | E | D | D | V | V | K | P | 91 | | Sbay\_632.22/1-1867 | 50 | Y | P | F | L | L | V | K | K | W | E | T | R | V | T | A | A | R | A | V | G | G | I | V | A | H | A | P | S | W | D | P | N | E | S | D | - | - | - | S | V | R | G | - | - | - | - | - | - | - | 88 | | SAKL0H09724g/1-1862 | 50 | Y | P | F | L | L | S | K | K | W | E | T | R | V | T | A | A | R | A | V | G | G | I | V | S | H | S | H | I | W | D | P | N | A | D | D | - | - | - | E | L | D | D | K | I | A | - | - | - | - | 91 | | P32333/1-1867 | 50 | Y | P | F | L | L | V | K | K | W | E | T | R | V | T | A | A | R | A | V | G | G | I | V | A | H | A | P | S | W | D | P | N | E | S | D | - | - | - | L | V | G | G | - | - | - | - | - | - | - | 88 | |  | | G0VCI3/1-1859 | 93 | - | - | - | - | - | - | - | - | - | - | - | - | - | - | - | - | - | - | - | - | - | - | - | - | - | - | - | - | - | - | - | N | T | G | D | A | P | - | - | V | E | T | A | Q | V | K | L | E | Q | 108 | | Q6CM16/1-1873 | 92 | - | - | - | - | - | - | - | - | - | - | - | - | - | - | - | - | - | - | - | - | - | - | - | - | - | - | - | - | - | - | - | T | Q | Q | P | L | G | - | - | P | E | S | A | K | V | K | I | E | E | 107 | | Q6FRV8/1-1904 | 99 | K | E | N | Q | N | N | D | H | A | E | M | E | N | T | V | I | K | E | E | P | D | Q | D | N | G | V | S | V | P | N | H | T | N | N | D | N | N | I | E | T | V | N | A | K | V | K | L | E | Q | 147 | | Q758L7/1-1866 | 85 | - | - | - | - | - | - | - | - | - | - | - | - | - | - | - | - | - | - | - | - | - | - | - | - | - | - | - | - | - | - | - | - | - | - | - | - | - | - | - | - | - | - | G | E | L | A | G | E | E | 91 | | A7TGL6/1-1902 | 96 | N | E | T | D | I | - | - | - | - | - | - | - | - | - | - | - | - | - | - | - | - | - | - | - | - | - | - | - | - | - | - | I | Q | T | D | E | F | - | - | Q | E | N | A | K | I | K | I | E | K | 116 | | C5DII9/1-1880 | 96 | G | Q | A | T | Q | S | A | S | S | A | A | E | - | - | - | - | - | - | - | - | - | - | - | - | - | - | - | - | - | - | - | V | S | S | N | G | D | - | - | G | E | T | A | K | V | K | L | E | Q | 123 | | C5DP10/1-1883 | 92 | - | - | - | - | - | - | - | - | - | - | - | - | - | - | - | - | - | - | - | - | - | - | - | - | - | - | - | - | - | - | - | - | - | A | D | D | E | - | - | E | T | N | A | K | V | K | L | E | H | 105 | | Kwal\_YGOB\_27.12097/1-1546 | 92 | E | E | T | D | A | G | A | N | G | T | T | E | - | - | - | - | - | - | - | - | - | - | - | - | - | - | - | - | - | - | - | A | - | - | - | - | - | - | - | G | E | T | A | K | V | K | L | E | H | 114 | | Sbay\_632.22/1-1867 | 89 | - | - | - | - | - | - | - | - | - | - | - | - | - | - | - | - | - | - | - | - | - | - | - | - | - | - | - | - | - | - | - | T | N | E | G | S | P | - | - | L | D | N | A | Q | V | K | L | E | H | 104 | | SAKL0H09724g/1-1862 | 92 | - | - | - | - | - | - | - | - | - | - | - | - | - | - | - | - | - | - | - | - | - | - | - | - | - | - | - | - | - | - | - | V | E | Q | D | K | L | - | - | E | E | N | A | K | V | K | L | E | N | 107 | | P32333/1-1867 | 89 | - | - | - | - | - | - | - | - | - | - | - | - | - | - | - | - | - | - | - | - | - | - | - | - | - | - | - | - | - | - | - | T | N | E | G | S | P | - | - | L | D | N | A | Q | V | K | L | E | H | 104 | |  | | G0VCI3/1-1859 | 109 | D | I | Q | L | K | L | E | E | F | S | K | S | D | E | T | S | L | L | R | G | N | Q | - | - | - | E | L | Y | S | L | S | Q | W | N | L | N | A | L | F | K | S | G | K | V | L | L | A | S | N | 154 | | Q6CM16/1-1873 | 108 | E | M | K | V | K | L | E | E | L | S | H | T | D | E | W | N | E | L | Q | D | D | T | - | - | - | H | Y | F | T | L | N | S | W | K | I | S | E | L | L | K | S | G | K | S | L | L | A | A | S | 153 | | Q6FRV8/1-1904 | 148 | D | I | Q | L | K | L | E | E | L | I | K | E | D | S | - | S | L | L | D | D | N | K | N | A | D | N | C | L | T | L | S | N | W | H | L | N | E | L | F | K | S | G | K | V | F | L | A | S | S | 195 | | Q758L7/1-1866 | 92 | T | R | Q | L | - | - | - | E | A | L | G | R | Y | E | W | R | G | L | Q | D | D | S | - | - | - | A | L | Y | S | L | A | Q | W | E | L | G | K | I | F | K | A | G | R | T | L | L | A | A | R | 134 | | A7TGL6/1-1902 | 117 | D | M | Q | L | K | L | E | E | I | - | - | - | D | E | D | E | L | L | D | G | D | K | - | - | - | P | Y | Y | S | L | S | N | W | N | L | Y | E | L | F | K | T | G | K | T | L | L | A | S | S | 159 | | C5DII9/1-1880 | 124 | E | I | R | L | K | L | E | E | V | D | N | T | E | E | W | R | S | L | Q | D | D | S | - | - | - | K | L | F | S | L | A | D | W | N | L | T | D | V | L | K | S | G | K | P | L | L | A | A | S | 169 | | C5DP10/1-1883 | 106 | E | M | Q | V | K | L | E | E | A | E | H | D | E | L | L | L | Q | Q | E | D | S | R | - | - | - | Q | T | L | S | L | S | D | W | S | L | H | E | L | L | K | S | K | K | V | L | L | A | S | S | 151 | | Kwal\_YGOB\_27.12097/1-1546 | 115 | E | I | R | L | K | L | E | E | V | D | K | T | D | E | W | R | S | L | Q | D | D | S | - | - | - | N | L | F | S | L | A | S | W | N | L | N | E | I | L | K | S | G | K | T | L | L | A | A | S | 160 | | Sbay\_632.22/1-1867 | 105 | E | M | Q | I | K | L | E | E | A | T | Q | N | N | Q | L | N | L | L | Q | E | D | Q | - | - | - | H | L | S | S | L | T | D | W | K | L | N | E | I | L | K | S | G | K | V | L | L | A | S | S | 150 | | SAKL0H09724g/1-1862 | 108 | D | I | Q | M | K | L | K | E | L | D | Q | S | E | E | W | K | R | L | Q | D | D | S | - | - | - | N | L | F | S | L | S | H | W | R | L | N | E | V | L | K | S | G | R | T | L | L | A | S | S | 153 | | P32333/1-1867 | 105 | E | M | K | I | K | L | E | E | A | T | Q | N | N | Q | L | N | L | L | Q | E | D | H | - | - | - | H | L | S | S | L | S | D | W | K | L | N | E | I | L | K | S | G | K | V | L | L | A | S | S | 150 | |  | | G0VCI3/1-1859 | 155 | M | N | D | F | M | N | N | N | N | I | S | V | N | E | N | P | K | K | Q | L | K | V | - | - | - | - | - | - | - | - | - | - | - | - | - | - | - | - | - | - | - | - | - | - | - | - | - | - | - | 176 | | Q6CM16/1-1873 | 154 | A | N | D | Y | - | - | - | - | - | - | - | E | S | K | A | L | N | I | N | N | N | G | - | - | - | - | S | A | D | I | E | Q | D | D | A | N | N | V | K | H | L | K | K | E | D | S | - | - | F | 189 | | Q6FRV8/1-1904 | 196 | I | N | D | Y | - | - | - | - | - | - | - | D | D | K | K | V | A | S | N | S | P | S | - | - | - | - | - | - | - | T | L | S | S | D | I | E | D | A | - | - | - | - | - | - | - | - | - | - | - | 219 | | Q758L7/1-1866 | 135 | S | D | A | Y | - | - | - | - | - | - | - | A | A | E | E | S | K | K | Q | A | V | L | - | - | - | - | - | - | - | - | A | E | Y | V | L | E | P | A | - | - | - | - | - | - | - | - | - | - | - | 157 | | A7TGL6/1-1902 | 160 | I | N | D | F | - | - | N | D | N | N | D | I | N | A | S | R | K | K | L | I | K | T | - | - | - | - | D | Q | N | N | N | S | N | D | D | D | D | S | N | R | Q | N | S | N | N | N | P | N | I | 202 | | C5DII9/1-1880 | 170 | F | D | D | F | - | - | - | - | - | - | - | P | A | P | L | Q | L | E | Q | P | Q | R | - | - | - | - | Q | P | A | A | K | Q | T | K | I | E | E | T | P | E | F | A | Q | - | - | - | - | - | - | 201 | | C5DP10/1-1883 | 152 | M | N | D | Y | - | - | - | - | - | - | - | G | D | G | N | T | R | K | Q | P | K | K | - | - | - | - | D | L | G | I | N | G | E | F | Y | D | E | L | K | E | I | N | N | G | N | N | - | - | - | 186 | | Kwal\_YGOB\_27.12097/1-1546 | 161 | V | E | D | Y | - | - | - | - | - | - | - | S | A | A | L | Q | L | A | Q | S | D | Q | - | - | - | - | A | S | A | V | K | F | S | K | L | E | D | T | A | E | F | A | Q | - | - | - | - | - | - | 192 | | Sbay\_632.22/1-1867 | 151 | I | N | D | Y | - | - | N | V | L | G | R | A | D | D | N | V | R | K | Q | T | K | A | - | - | - | - | D | - | - | - | - | - | - | - | - | - | - | - | - | - | - | - | - | - | - | - | - | - | - | 171 | | SAKL0H09724g/1-1862 | 154 | I | N | E | Y | - | - | - | - | - | - | - | A | P | E | Q | H | H | Q | Q | Q | Q | Y | H | H | S | D | E | P | L | Q | K | T | V | K | L | E | D | S | V | - | - | - | - | - | - | - | - | - | - | 185 | | P32333/1-1867 | 151 | M | N | D | Y | - | - | N | V | L | G | K | A | D | D | N | I | R | K | Q | A | K | T | - | - | - | - | D | - | - | - | - | - | - | - | - | - | - | - | - | - | - | - | - | - | - | - | - | - | - | 171 | |  | | G0VCI3/1-1859 | 177 | - | - | - | - | - | - | - | - | - | E | T | E | S | N | Q | N | V | K | K | E | Q | G | T | T | T | K | K | S | A | R | M | L | A | M | A | K | R | K | K | K | I | Q | - | A | K | N | A | T | S | 215 | | Q6CM16/1-1873 | 190 | A | D | L | K | K | E | N | S | T | D | S | - | L | A | D | E | D | T | V | N | E | T | K | E | S | K | R | S | A | R | M | L | A | M | A | R | R | K | K | K | I | Q | - | A | K | T | V | N | K | 236 | | Q6FRV8/1-1904 | 220 | - | S | M | K | K | E | N | D | A | E | N | H | C | S | K | S | S | N | I | E | G | G | N | S | S | K | K | S | A | R | M | L | A | M | A | K | R | K | K | K | M | Q | K | S | K | T | T | T | T | 267 | | Q758L7/1-1866 | 158 | - | - | V | K | R | E | - | - | - | - | - | - | - | - | G | V | E | A | P | M | S | P | A | E | S | K | K | S | A | R | M | L | A | M | A | K | R | R | R | K | I | Q | - | A | K | T | T | S | K | 195 | | A7TGL6/1-1902 | 203 | S | D | V | K | Q | E | - | - | - | - | - | - | - | - | Q | Q | Q | Q | P | N | S | N | G | G | S | K | K | S | A | R | M | L | A | M | A | K | R | K | Q | R | M | Q | - | T | K | N | I | S | T | 242 | | C5DII9/1-1880 | 202 | - | - | - | - | - | - | - | - | - | - | - | - | P | Q | P | L | S | G | K | A | Q | S | L | E | S | K | K | S | A | R | M | A | A | M | A | K | R | K | R | K | I | Q | - | A | K | T | T | T | K | 237 | | C5DP10/1-1883 | 187 | - | G | V | K | K | E | E | P | G | D | V | - | P | P | R | Q | S | S | P | S | G | T | S | S | N | K | K | S | A | R | M | L | A | M | A | K | R | K | K | K | M | Q | - | A | K | S | S | S | N | 232 | | Kwal\_YGOB\_27.12097/1-1546 | 193 | - | - | - | - | - | - | - | - | - | - | - | - | P | Q | Q | - | S | S | K | I | P | T | L | E | S | K | K | S | A | R | M | A | A | M | A | K | R | K | R | K | I | Q | - | A | K | T | T | T | K | 227 | | Sbay\_632.22/1-1867 | 172 | - | D | I | K | Q | E | I | P | T | Y | N | - | A | F | D | K | A | N | E | N | K | N | S | A | N | K | K | S | A | R | M | L | A | M | A | R | R | K | K | K | M | I | - | A | K | N | T | S | K | 217 | | SAKL0H09724g/1-1862 | 186 | - | - | - | - | - | - | - | - | - | - | - | - | - | - | K | L | E | G | R | P | A | L | S | E | S | K | K | S | A | R | M | L | A | M | A | K | R | K | K | K | I | Q | - | A | K | Q | T | T | K | 219 | | P32333/1-1867 | 172 | - | D | I | K | Q | E | T | S | M | L | N | - | A | S | D | K | A | N | E | N | K | S | N | A | N | K | K | S | A | R | M | L | A | M | A | R | R | K | K | K | M | S | - | A | K | N | T | P | K | 217 | |  | | G0VCI3/1-1859 | 216 | K | P | V | D | I | T | E | S | S | V | S | K | T | L | L | N | Q | Q | - | - | - | - | - | - | - | - | - | - | - | - | - | - | - | - | - | - | - | - | - | - | N | K | N | N | E | - | - | - | - | 238 | | Q6CM16/1-1873 | 237 | K | P | V | D | L | S | Q | S | S | A | T | R | N | M | I | P | N | K | - | - | - | - | - | - | - | - | - | - | - | - | - | - | - | - | - | - | - | - | - | - | - | - | - | - | E | D | L | S | D | 259 | | Q6FRV8/1-1904 | 268 | M | E | V | N | L | S | E | S | S | L | S | K | K | L | M | D | D | P | - | - | - | - | - | - | - | - | - | - | - | - | - | - | - | - | - | - | - | - | - | - | - | - | - | - | - | - | - | - | S | 286 | | Q758L7/1-1866 | 196 | K | P | L | D | L | S | Q | S | S | V | A | R | N | L | M | N | Q | D | A | A | A | D | G | S | N | E | D | E | D | S | G | N | N | N | N | S | S | N | H | N | N | N | D | N | N | D | S | S | H | 244 | | A7TGL6/1-1902 | 243 | K | P | V | N | L | T | E | S | S | V | S | K | N | L | L | K | S | K | - | - | - | - | - | - | - | - | - | - | - | - | - | - | - | - | - | - | - | - | - | - | N | G | N | N | N | N | - | - | N | 267 | | C5DII9/1-1880 | 238 | K | P | V | D | I | S | Q | S | S | I | S | R | N | L | M | A | Q | E | - | - | - | - | - | - | - | - | - | - | - | - | - | - | - | - | - | - | - | - | - | - | - | - | - | - | - | D | S | S | Q | 259 | | C5DP10/1-1883 | 233 | K | P | V | D | L | S | E | S | S | V | S | K | S | L | M | N | Q | H | - | - | - | - | - | - | - | - | - | - | - | - | - | - | - | - | - | - | - | - | - | - | M | S | K | S | K | E | A | S | M | 259 | | Kwal\_YGOB\_27.12097/1-1546 | 228 | K | P | V | D | I | S | Q | S | S | I | S | R | N | L | M | A | Q | E | - | - | - | - | - | - | - | - | - | - | - | - | - | - | - | - | - | - | - | - | - | - | - | - | - | - | - | D | S | S | Q | 249 | | Sbay\_632.22/1-1867 | 218 | H | P | V | D | I | T | E | S | S | V | S | K | T | L | L | N | E | K | - | - | - | - | - | - | - | - | - | - | - | - | - | - | - | - | - | - | - | - | - | - | N | M | T | S | T | A | A | S | S | 244 | | SAKL0H09724g/1-1862 | 220 | K | P | V | D | L | S | Q | S | T | L | S | K | N | L | M | A | Q | E | - | - | - | - | - | - | - | - | - | - | - | - | - | - | - | - | - | - | - | - | - | - | - | - | - | - | - | H | S | S | H | 241 | | P32333/1-1867 | 218 | H | P | V | D | I | T | E | S | S | V | S | K | T | L | L | N | G | K | - | - | - | - | - | - | - | - | - | - | - | - | - | - | - | - | - | - | - | - | - | - | N | M | T | N | S | A | A | S | L | 244 | |  | | G0VCI3/1-1859 | 239 | - | - | S | T | S | - | - | - | - | P | V | E | L | T | N | P | K | L | E | I | T | E | Q | T | D | P | N | K | I | L | I | E | S | T | M | L | P | I | L | E | Q | Q | E | R | V | A | G | L | V | 281 | | Q6CM16/1-1873 | 260 | S | P | T | P | V | - | - | - | - | P | M | T | L | T | N | P | K | L | E | I | T | E | Q | S | D | E | K | R | L | M | V | E | S | M | V | Q | P | I | L | E | K | H | E | K | I | R | G | F | V | 304 | | Q6FRV8/1-1904 | 287 | A | V | K | S | D | - | - | - | - | A | V | K | M | E | N | P | K | L | A | I | T | D | Q | A | D | P | N | A | I | M | V | E | A | V | V | P | K | I | L | E | K | H | E | K | V | A | G | L | V | 331 | | Q758L7/1-1866 | 245 | S | N | T | A | S | P | Q | Q | V | P | V | Q | L | N | N | P | R | L | E | I | T | E | Q | P | D | S | N | K | I | M | I | E | S | M | V | A | P | L | L | E | K | Q | Q | R | V | S | G | L | V | 293 | | A7TGL6/1-1902 | 268 | N | V | K | N | E | - | - | - | - | S | S | T | L | V | N | P | K | M | E | I | T | E | Q | S | D | S | N | K | I | M | L | E | A | V | V | E | P | I | L | E | K | H | E | R | V | A | G | L | V | 312 | | C5DII9/1-1880 | 260 | S | P | T | P | S | - | - | - | - | P | T | M | L | N | N | P | K | L | E | I | T | E | Q | P | D | S | K | K | I | M | I | E | S | V | M | S | P | I | L | E | K | H | D | S | V | S | G | L | V | 304 | | C5DP10/1-1883 | 260 | S | P | T | T | T | - | - | - | - | P | T | S | L | S | N | P | K | L | E | I | T | E | Q | M | D | N | S | K | I | M | V | E | S | M | M | A | P | I | L | E | K | H | E | K | V | A | G | L | V | 304 | | Kwal\_YGOB\_27.12097/1-1546 | 250 | S | P | T | P | S | - | - | - | - | P | T | M | L | N | N | P | K | L | E | I | T | E | Q | S | D | S | K | K | I | M | I | E | S | M | M | S | P | I | L | E | K | N | D | N | I | A | G | L | V | 294 | | Sbay\_632.22/1-1867 | 245 | S | T | S | P | T | - | - | - | - | - | S | A | Q | P | N | P | K | L | E | I | T | E | Q | A | N | D | N | K | L | M | I | E | S | T | V | R | P | L | L | E | Q | H | E | I | V | A | G | L | V | 288 | | SAKL0H09724g/1-1862 | 242 | S | P | T | P | S | - | - | - | - | P | V | T | A | N | N | P | K | L | E | I | T | E | Q | S | N | S | D | K | I | M | I | E | S | V | M | A | P | I | L | E | K | H | E | R | I | A | G | L | V | 286 | | P32333/1-1867 | 245 | A | T | S | P | T | - | - | - | - | - | S | N | Q | L | N | P | K | L | E | I | T | E | Q | A | D | E | S | K | L | M | I | E | S | T | V | R | P | L | L | E | Q | H | E | I | V | A | G | L | V | 288 | |  | | G0VCI3/1-1859 | 282 | W | Q | F | Q | G | I | Y | E | L | L | L | E | N | L | T | S | D | I | W | E | V | R | H | G | A | A | L | G | L | R | E | L | M | K | K | H | A | S | S | V | S | R | V | K | G | K | S | R | Q | 330 | | Q6CM16/1-1873 | 305 | W | Q | F | Q | G | I | Y | E | L | L | L | D | N | L | M | N | E | Q | W | E | I | R | H | G | A | A | L | G | L | R | E | L | M | K | K | H | A | P | S | V | G | R | L | S | G | K | S | K | Q | 353 | | Q6FRV8/1-1904 | 332 | W | Q | F | Q | G | V | F | E | L | L | L | Q | N | L | M | H | D | N | W | E | V | R | H | G | A | T | L | G | L | R | E | L | M | K | K | H | A | Y | G | I | N | R | I | K | G | K | T | R | K | 380 | | Q758L7/1-1866 | 294 | W | Q | F | Q | G | I | F | E | L | L | L | D | N | L | M | N | D | S | W | E | I | R | H | G | A | A | L | G | I | R | E | I | V | K | R | H | A | T | G | V | G | R | I | K | G | K | T | R | Q | 342 | | A7TGL6/1-1902 | 313 | W | Q | F | Q | G | I | Y | E | L | L | L | K | N | L | A | H | D | N | W | E | I | R | H | G | A | A | L | G | L | R | E | L | M | K | K | H | A | S | S | V | S | R | L | K | G | K | S | K | A | 361 | | C5DII9/1-1880 | 305 | W | Q | F | Q | G | I | Y | E | L | L | L | E | N | L | M | S | D | S | W | E | I | R | H | A | A | A | L | G | L | R | E | I | I | K | K | H | A | K | S | V | A | R | I | K | G | K | S | K | K | 353 | | C5DP10/1-1883 | 305 | W | Q | F | Q | G | I | F | E | L | L | L | D | N | L | I | N | D | S | W | E | V | R | H | G | S | A | L | G | L | R | E | L | L | K | K | H | A | F | S | V | S | R | V | K | G | K | S | R | A | 353 | | Kwal\_YGOB\_27.12097/1-1546 | 295 | W | Q | F | Q | G | I | Y | E | L | L | L | D | S | L | M | S | D | S | W | E | I | R | H | A | A | A | L | G | L | R | E | I | V | K | K | H | A | K | S | V | A | R | I | K | G | K | S | K | K | 343 | | Sbay\_632.22/1-1867 | 289 | W | Q | F | Q | G | I | Y | E | L | L | L | D | N | L | M | S | E | N | W | E | I | R | H | G | A | A | L | G | L | R | E | L | V | K | K | H | A | L | G | V | S | R | V | R | G | R | T | R | D | 337 | | SAKL0H09724g/1-1862 | 287 | W | Q | F | Q | G | I | Y | E | L | L | L | N | N | L | T | N | D | A | W | E | I | R | H | G | A | A | L | G | L | R | E | I | V | K | K | H | A | K | S | V | G | R | I | K | G | K | S | L | K | 335 | | P32333/1-1867 | 289 | W | Q | F | Q | G | I | Y | E | L | L | L | D | N | L | M | S | E | N | W | E | I | R | H | G | A | A | L | G | L | R | E | L | V | K | K | H | A | Y | G | V | S | R | V | K | G | N | T | R | E | 337 | |  | | G0VCI3/1-1859 | 331 | E | N | D | L | Q | N | R | K | S | L | E | D | L | A | T | R | L | L | T | V | F | A | L | D | R | F | G | D | Y | V | Y | D | T | V | V | A | P | V | R | E | S | V | A | Q | T | L | A | A | L | 379 | | Q6CM16/1-1873 | 354 | D | N | D | S | R | N | Y | C | S | L | E | D | L | A | T | R | L | L | T | I | F | A | L | D | R | F | S | D | F | V | N | D | T | A | V | A | P | V | R | E | S | A | A | Q | T | L | A | T | L | 402 | | Q6FRV8/1-1904 | 381 | E | N | N | D | R | N | Y | Q | G | L | E | D | L | A | T | R | L | L | T | I | F | A | L | D | R | F | G | D | Y | I | Y | D | T | V | V | A | P | V | R | E | S | A | A | Q | I | L | A | A | L | 429 | | Q758L7/1-1866 | 343 | E | N | D | L | R | N | K | R | A | L | E | D | L | A | T | R | L | L | T | I | F | A | L | D | R | F | G | D | F | V | N | D | T | V | V | A | P | V | R | E | S | A | A | Q | A | L | A | A | L | 391 | | A7TGL6/1-1902 | 362 | E | N | D | N | R | N | F | A | S | L | D | D | L | A | T | R | L | L | T | I | F | A | L | D | R | F | G | D | Y | I | H | D | T | V | V | A | P | V | R | E | S | A | A | Q | T | L | A | T | L | 410 | | C5DII9/1-1880 | 354 | E | N | N | I | R | N | R | R | A | L | E | D | L | A | T | R | L | L | I | V | F | A | L | D | R | F | G | D | F | V | Y | D | T | V | V | A | P | V | R | E | S | V | A | Q | T | L | A | A | L | 402 | | C5DP10/1-1883 | 354 | E | N | N | A | R | N | K | R | S | L | E | D | I | S | T | R | L | L | T | V | F | A | I | D | R | F | G | D | F | V | Y | D | T | V | V | A | P | V | R | E | S | V | A | Q | T | L | A | A | L | 402 | | Kwal\_YGOB\_27.12097/1-1546 | 344 | E | N | D | L | R | N | R | K | A | L | E | D | L | A | T | R | L | L | T | V | - | - | - | - | - | - | - | - | - | - | - | - | - | - | - | - | - | - | - | - | - | - | - | - | - | - | - | - | - | 363 | | Sbay\_632.22/1-1867 | 338 | E | N | D | I | R | N | S | K | S | L | E | D | L | A | S | R | L | L | T | V | F | A | L | D | R | F | G | D | Y | V | Y | D | T | V | V | A | P | V | R | E | S | I | A | Q | T | L | A | A | L | 386 | | SAKL0H09724g/1-1862 | 336 | E | N | G | H | R | N | S | K | A | L | E | D | L | A | T | R | L | L | T | I | F | A | L | D | R | F | G | D | F | V | Y | D | T | V | V | A | P | V | R | E | S | A | A | Q | T | L | A | A | L | 384 | | P32333/1-1867 | 338 | E | N | N | L | R | N | S | R | S | L | E | D | L | A | S | R | L | L | T | V | F | A | L | D | R | F | G | D | Y | V | Y | D | T | V | V | A | P | V | R | E | S | V | A | Q | T | L | A | A | L | 386 | |  | | G0VCI3/1-1859 | 380 | L | I | H | L | D | D | N | L | S | L | T | I | F | K | T | L | E | Q | L | V | L | Q | D | P | Q | I | T | G | L | P | N | - | - | - | - | - | - | - | - | - | - | - | - | - | - | - | - | - | K | 411 | | Q6CM16/1-1873 | 403 | L | I | H | L | D | D | E | I | A | L | K | V | F | Q | K | L | E | Q | L | V | L | Q | D | P | G | L | T | G | S | P | N | - | - | - | - | - | - | - | - | - | - | - | - | - | - | - | - | - | K | 434 | | Q6FRV8/1-1904 | 430 | L | I | N | L | P | D | D | L | A | L | K | V | Y | M | K | L | E | D | L | V | F | Q | N | P | E | V | T | G | L | P | N | - | - | - | - | - | - | - | - | - | - | - | - | - | - | - | - | - | K | 461 | | Q758L7/1-1866 | 392 | L | I | H | L | E | D | D | L | S | V | K | I | F | S | V | L | E | Q | L | V | L | Q | D | P | R | Y | V | G | S | P | T | - | - | - | - | - | - | - | - | - | - | - | - | - | - | - | - | - | K | 423 | | A7TGL6/1-1902 | 411 | L | I | H | L | N | D | E | L | S | M | K | I | F | N | A | L | Q | Q | L | V | F | Q | D | F | I | N | T | Q | T | Q | N | F | K | P | E | D | D | E | F | Q | Q | P | Q | P | N | I | Q | L | K | 459 | | C5DII9/1-1880 | 403 | L | I | H | L | D | D | D | L | C | I | Q | I | F | G | A | L | E | Q | L | V | L | Q | D | P | K | I | V | G | L | P | N | - | - | - | - | - | - | - | - | - | - | - | - | - | - | - | - | - | K | 434 | | C5DP10/1-1883 | 403 | L | I | H | L | D | D | D | L | C | R | K | I | F | G | A | L | E | Q | L | V | L | Q | D | P | Q | V | V | M | M | P | N | - | - | - | - | - | - | - | - | - | - | - | - | - | - | - | - | - | K | 434 | | Kwal\_YGOB\_27.12097/1-1546 | 364 | - | - | - | - | - | - | - | - | - | - | - | - | - | - | - | - | - | - | - | - | - | - | - | - | - | - | - | - | L | P | N | - | - | - | - | - | - | - | - | - | - | - | - | - | - | - | - | - | K | 367 | | Sbay\_632.22/1-1867 | 387 | L | I | H | L | N | N | N | L | S | I | K | I | F | N | C | L | E | Q | L | V | L | Q | D | P | L | E | T | G | L | P | N | - | - | - | - | - | - | - | - | - | - | - | - | - | - | - | - | - | K | 418 | | SAKL0H09724g/1-1862 | 385 | L | I | H | L | D | N | D | L | S | V | K | I | F | S | A | L | E | Q | L | V | L | Q | D | P | K | V | I | G | L | P | T | - | - | - | - | - | - | - | - | - | - | - | - | - | - | - | - | - | K | 416 | | P32333/1-1867 | 387 | L | I | H | L | D | S | T | L | S | I | K | I | F | N | C | L | E | Q | L | V | L | Q | D | P | L | Q | T | G | L | P | N | - | - | - | - | - | - | - | - | - | - | - | - | - | - | - | - | - | K | 418 | |  | | G0VCI3/1-1859 | 412 | I | W | E | A | T | H | G | G | L | L | G | I | R | Y | F | V | S | I | K | T | D | F | L | L | S | N | N | L | L | D | R | V | V | N | I | V | L | Y | G | L | N | Q | S | D | D | D | V | Q | S | 460 | | Q6CM16/1-1873 | 435 | I | W | Q | A | T | H | G | G | L | L | G | I | R | Y | F | V | S | I | K | T | D | F | L | F | K | H | N | L | L | N | N | V | V | N | I | V | L | Y | G | L | K | E | S | D | D | D | V | Q | S | 483 | | Q6FRV8/1-1904 | 462 | I | W | E | A | T | H | G | G | L | L | G | I | R | Y | F | V | S | I | K | Q | E | F | L | I | E | Q | N | L | L | D | K | V | V | Q | T | V | L | Y | G | L | N | Q | N | D | D | D | V | K | S | 510 | | Q758L7/1-1866 | 424 | I | W | E | A | T | H | G | G | L | L | G | I | R | Y | F | V | S | I | K | T | D | F | L | F | T | N | N | L | L | D | N | V | V | N | I | V | L | Y | G | L | N | E | R | D | D | D | V | Q | S | 472 | | A7TGL6/1-1902 | 460 | V | W | E | A | T | H | G | G | L | L | G | I | L | Y | F | A | S | I | K | Q | D | F | L | K | N | N | S | L | M | D | S | V | V | S | I | V | L | Y | G | L | N | Q | A | D | D | D | V | Q | S | 508 | | C5DII9/1-1880 | 435 | I | W | E | A | T | H | G | G | L | L | G | I | R | Y | F | V | G | I | K | T | D | F | L | F | R | H | N | L | L | E | N | V | V | N | I | V | L | Y | G | L | K | Q | N | N | D | D | V | Q | S | 483 | | C5DP10/1-1883 | 435 | I | W | E | A | T | H | G | G | L | L | G | V | R | Y | F | V | S | I | K | T | D | F | L | L | E | Q | H | L | L | N | N | V | V | N | I | V | L | Y | G | L | N | Q | P | D | D | D | V | Q | S | 483 | | Kwal\_YGOB\_27.12097/1-1546 | 368 | I | W | E | A | T | H | G | G | L | L | G | V | R | Y | F | V | S | I | K | T | D | F | L | F | Q | N | N | L | L | N | N | V | V | N | I | V | L | Y | G | L | K | E | H | N | D | D | V | Q | S | 416 | | Sbay\_632.22/1-1867 | 419 | I | W | E | A | T | H | G | G | L | L | G | I | R | Y | F | V | S | I | K | T | D | F | L | F | S | H | G | L | L | E | N | V | V | R | I | V | L | Y | G | L | N | Q | T | D | D | D | V | Q | S | 467 | | SAKL0H09724g/1-1862 | 417 | I | W | E | A | T | H | G | G | L | L | G | I | R | Y | F | V | S | I | K | A | D | F | L | Y | Q | N | N | L | L | N | N | V | V | N | I | V | L | Y | G | L | N | E | H | D | D | D | V | Q | S | 465 | | P32333/1-1867 | 419 | I | W | E | A | T | H | G | G | L | L | G | I | R | Y | F | V | S | I | K | T | N | F | L | F | A | H | G | L | L | E | N | V | V | R | I | V | L | Y | G | L | N | Q | S | D | D | D | V | Q | S | 467 | |  | | G0VCI3/1-1859 | 461 | V | A | A | S | I | L | T | P | I | T | S | E | F | V | K | M | D | S | E | K | I | D | V | V | L | T | T | I | W | T | S | L | T | H | L | E | D | D | L | S | A | S | V | G | S | V | M | D | L | 509 | | Q6CM16/1-1873 | 484 | V | S | A | A | I | L | S | P | I | T | D | D | F | V | K | L | Q | T | D | T | I | D | L | V | L | T | T | V | W | N | S | L | T | H | L | D | D | D | L | S | S | S | V | S | S | V | M | D | L | 532 | | Q6FRV8/1-1904 | 511 | V | A | A | S | I | L | T | P | I | T | A | E | F | V | K | L | D | T | D | T | L | D | I | V | L | T | T | I | W | N | S | L | I | H | L | D | D | D | L | A | S | S | V | G | S | V | M | D | L | 559 | | Q758L7/1-1866 | 473 | V | A | A | A | I | L | T | P | I | T | S | E | F | I | K | L | E | P | S | T | I | D | L | V | V | S | A | I | W | N | S | L | S | Q | L | E | D | D | L | S | S | S | V | A | S | V | M | D | L | 521 | | A7TGL6/1-1902 | 509 | V | S | A | S | I | L | S | P | I | T | D | D | F | V | K | L | E | T | S | K | I | D | L | L | I | T | T | I | W | N | S | L | T | H | L | D | D | D | L | S | S | S | V | G | S | I | M | D | L | 557 | | C5DII9/1-1880 | 484 | V | A | A | A | I | L | T | P | I | A | V | E | F | V | K | L | E | E | S | T | I | D | L | V | L | S | T | I | W | N | L | L | T | H | L | E | D | D | L | S | S | S | V | G | S | V | M | D | L | 532 | | C5DP10/1-1883 | 484 | V | A | A | A | I | L | S | P | I | T | D | Q | F | V | K | L | D | L | H | T | I | E | L | V | L | T | T | I | W | S | L | L | T | H | L | E | D | D | L | S | S | S | V | G | S | V | M | N | L | 532 | | Kwal\_YGOB\_27.12097/1-1546 | 417 | V | A | A | A | I | L | A | P | I | A | V | E | F | V | K | L | D | T | N | T | I | D | L | V | L | S | T | I | W | N | L | L | T | H | L | E | D | D | L | S | S | S | V | G | S | V | M | D | L | 465 | | Sbay\_632.22/1-1867 | 468 | V | A | A | S | I | L | T | P | I | T | N | E | F | V | K | L | N | T | S | T | I | D | T | L | V | T | T | I | W | S | L | L | A | R | L | D | D | D | I | S | S | S | V | G | S | I | M | N | L | 516 | | SAKL0H09724g/1-1862 | 466 | V | A | A | A | I | L | T | P | I | T | S | E | F | V | T | L | E | A | A | T | I | D | L | V | L | S | T | I | W | N | S | L | S | H | L | D | D | D | L | S | S | S | V | G | A | V | M | N | L | 514 | | P32333/1-1867 | 468 | V | A | A | S | I | L | T | P | I | T | S | E | F | V | K | L | N | N | S | T | I | E | I | L | V | T | T | I | W | S | L | L | A | R | L | D | D | D | I | S | S | S | V | G | S | I | M | D | L | 516 | |  | | G0VCI3/1-1859 | 510 | L | A | N | L | C | K | H | T | E | V | L | D | I | L | K | K | K | A | I | K | Y | P | S | E | W | S | F | K | S | L | V | P | K | L | Y | P | F | L | R | H | S | I | S | S | V | R | I | A | V | 558 | | Q6CM16/1-1873 | 533 | L | A | K | L | C | K | H | E | Q | V | L | A | V | L | K | Q | K | S | I | D | S | P | S | E | W | S | F | K | S | L | V | P | Q | L | Y | P | F | L | R | H | S | I | T | M | V | R | K | S | V | 581 | | Q6FRV8/1-1904 | 560 | L | A | N | L | C | Q | Y | K | E | V | L | D | V | L | K | H | K | A | T | E | Q | P | L | E | W | S | F | K | S | L | V | P | K | L | Y | P | F | L | R | H | S | I | S | S | V | R | K | S | V | 608 | | Q758L7/1-1866 | 522 | L | A | K | L | C | Q | H | Q | E | V | L | D | V | L | H | H | K | A | S | T | H | P | M | E | W | S | F | K | S | L | V | P | K | L | Y | P | F | L | R | N | T | I | T | N | V | R | R | S | V | 570 | | A7TGL6/1-1902 | 558 | L | A | K | L | C | K | Y | E | E | T | L | N | C | V | K | A | K | A | N | Q | Y | P | A | E | W | S | F | K | Y | L | V | P | K | L | F | P | F | L | R | H | S | I | S | S | V | R | Q | S | V | 606 | | C5DII9/1-1880 | 533 | L | A | K | L | C | E | Q | K | E | V | L | D | V | L | R | A | K | A | M | S | H | P | L | E | W | S | F | K | S | L | V | P | K | L | Y | P | F | L | R | H | S | I | T | N | V | R | K | A | V | 581 | | C5DP10/1-1883 | 533 | L | A | K | L | C | K | H | E | E | V | L | H | I | L | K | E | K | A | I | Q | H | P | S | E | W | S | F | K | S | L | V | P | K | L | Y | P | F | L | R | H | S | I | S | S | V | R | K | A | V | 581 | | Kwal\_YGOB\_27.12097/1-1546 | 466 | L | A | K | L | C | E | Q | Q | E | V | L | D | V | L | K | D | K | A | L | S | H | P | L | E | W | S | F | K | S | L | V | P | K | L | Y | P | F | L | R | H | S | I | T | N | V | R | K | S | V | 514 | | Sbay\_632.22/1-1867 | 517 | L | A | K | L | C | Y | H | Q | E | V | L | D | I | L | K | D | K | A | L | E | H | P | S | E | W | S | F | K | S | L | V | P | K | L | Y | P | F | L | R | H | S | I | S | S | V | R | K | A | V | 565 | | SAKL0H09724g/1-1862 | 515 | L | A | K | L | C | E | H | Q | E | V | L | N | V | L | K | D | K | A | I | A | C | P | L | E | W | S | F | K | G | L | V | P | K | L | Y | P | F | L | R | H | S | I | T | N | V | R | K | A | V | 563 | | P32333/1-1867 | 517 | L | A | K | L | C | D | H | Q | E | V | L | D | I | L | K | N | K | A | L | E | H | P | S | E | W | S | F | K | S | L | V | P | K | L | Y | P | F | L | R | H | S | I | S | S | V | R | R | A | V | 565 | |  | | G0VCI3/1-1859 | 559 | L | N | L | L | N | G | F | L | S | I | N | D | D | S | T | K | N | W | L | N | G | K | I | F | R | L | I | F | Q | N | I | L | L | E | Q | N | P | E | I | L | Q | L | S | Y | D | L | Y | G | D | 607 | | Q6CM16/1-1873 | 582 | L | N | L | L | S | A | F | L | S | L | N | D | S | A | T | K | N | W | I | N | G | K | L | F | K | L | I | Y | Q | N | I | L | L | E | Q | N | V | E | V | L | D | L | S | C | K | L | Y | A | A | 630 | | Q6FRV8/1-1904 | 609 | L | N | L | L | Q | S | F | L | S | I | K | D | E | S | T | K | H | W | L | N | G | K | V | F | R | L | V | F | Q | N | I | L | F | E | Q | N | P | D | I | L | N | L | S | Y | E | V | Y | T | S | 657 | | Q758L7/1-1866 | 571 | L | N | L | L | Q | A | F | L | S | I | E | D | E | T | S | K | Q | W | I | N | S | K | I | F | R | L | I | Y | Q | N | I | L | L | E | Q | H | D | D | I | L | E | Q | S | F | H | V | Y | R | T | 619 | | A7TGL6/1-1902 | 607 | L | N | L | L | L | A | F | L | S | I | N | D | D | S | T | K | G | W | I | N | G | K | I | F | R | L | I | F | Q | N | I | I | L | E | Q | N | P | K | I | L | Q | S | S | F | D | V | Y | T | K | 655 | | C5DII9/1-1880 | 582 | L | N | L | L | M | A | F | L | S | I | K | D | D | F | I | K | H | W | I | N | G | K | I | F | R | L | I | F | Q | N | I | I | L | E | Q | N | P | Q | V | L | D | M | S | F | K | V | Y | I | S | 630 | | C5DP10/1-1883 | 582 | L | N | L | L | N | A | F | L | S | I | N | D | D | S | T | K | G | W | L | N | G | K | V | F | R | L | V | F | Q | N | I | I | L | E | Q | N | R | E | I | L | D | L | S | F | Q | V | Y | I | Q | 630 | | Kwal\_YGOB\_27.12097/1-1546 | 515 | L | N | L | L | M | A | F | L | S | I | K | D | D | F | I | K | H | W | I | N | G | K | I | F | R | L | I | F | Q | N | I | I | L | E | Q | N | P | Q | V | L | E | L | S | F | K | V | Y | T | T | 563 | | Sbay\_632.22/1-1867 | 566 | L | N | L | L | I | A | F | L | S | I | K | D | D | S | T | K | N | W | L | N | G | K | V | F | R | L | V | F | Q | N | I | L | L | E | Q | N | P | E | L | L | Q | L | S | F | D | V | Y | T | A | 614 | | SAKL0H09724g/1-1862 | 564 | L | N | L | L | L | A | F | L | S | I | Q | D | D | S | T | K | H | W | I | N | G | K | I | F | R | L | V | Y | Q | N | I | L | L | E | Q | N | K | E | I | L | N | M | S | F | K | V | Y | F | M | 612 | | P32333/1-1867 | 566 | L | N | L | L | I | A | F | L | S | I | K | D | D | S | T | K | N | W | L | N | G | K | V | F | R | L | V | F | Q | N | I | L | L | E | Q | N | P | E | L | L | Q | L | S | F | D | V | Y | V | A | 614 | |  | | G0VCI3/1-1859 | 608 | L | L | S | H | Y | - | K | S | K | H | T | E | K | T | L | D | H | V | F | S | K | H | L | Q | P | I | L | H | L | L | N | T | P | I | G | E | N | G | K | S | Y | S | M | E | A | Q | Y | I | L | 655 | | Q6CM16/1-1873 | 631 | M | L | N | G | Y | V | N | S | K | G | Q | E | K | S | I | D | F | V | F | S | K | Y | L | T | S | I | L | H | L | L | I | T | P | I | G | E | Q | G | K | N | Y | A | M | D | I | Q | H | I | T | 679 | | Q6FRV8/1-1904 | 658 | L | L | T | H | Y | - | Q | L | K | H | T | E | K | T | L | D | H | A | F | C | K | H | L | Q | P | L | L | H | L | L | N | T | P | I | G | E | K | G | K | N | Y | S | M | E | S | H | Y | I | L | 705 | | Q758L7/1-1866 | 620 | M | L | E | I | Y | - | K | S | K | N | P | D | K | S | L | D | H | I | F | S | K | H | L | A | P | I | L | H | L | L | I | T | P | I | G | E | Q | G | K | N | Y | S | M | E | A | Q | Y | L | L | 667 | | A7TGL6/1-1902 | 656 | L | L | N | V | Y | - | K | S | M | E | A | E | K | T | L | D | H | L | L | S | K | H | L | Q | P | M | L | H | L | L | N | T | P | I | G | E | N | D | K | N | Y | S | M | E | S | Q | F | I | L | 703 | | C5DII9/1-1880 | 631 | M | L | E | E | Y | - | K | N | K | N | P | E | K | N | L | D | H | L | F | G | K | H | L | A | P | I | L | H | L | L | I | T | P | I | G | E | H | G | K | S | Y | N | M | E | L | Q | Y | I | L | 678 | | C5DP10/1-1883 | 631 | L | L | R | D | Y | - | R | F | K | H | T | E | K | T | L | D | H | V | F | S | K | H | L | Q | P | I | L | H | L | L | N | T | P | I | G | E | N | G | K | N | Y | S | M | E | S | Q | Y | I | L | 678 | | Kwal\_YGOB\_27.12097/1-1546 | 564 | M | L | A | E | Y | - | K | T | K | N | P | E | K | A | L | D | H | L | F | G | K | H | L | A | P | I | L | H | L | L | I | T | P | I | G | E | H | G | K | S | Y | N | M | E | L | Q | Y | I | L | 611 | | Sbay\_632.22/1-1867 | 615 | L | L | E | H | Y | - | K | V | K | H | T | E | K | T | L | D | H | V | F | S | K | H | L | Q | P | I | L | H | L | L | N | T | P | V | G | E | K | G | K | N | Y | A | M | E | S | Q | Y | I | L | 662 | | SAKL0H09724g/1-1862 | 613 | M | L | E | E | Y | - | R | S | K | N | F | E | K | N | L | D | H | V | F | G | K | H | L | A | P | I | L | H | L | L | I | T | P | I | G | E | Q | G | K | N | Y | N | M | E | L | Q | Y | I | L | 660 | | P32333/1-1867 | 615 | L | L | E | H | Y | - | K | V | K | H | T | E | K | T | L | D | H | V | F | S | K | H | L | Q | P | I | L | H | L | L | N | T | P | V | G | E | K | G | K | N | Y | A | M | E | S | Q | Y | I | L | 662 | |  | | G0VCI3/1-1859 | 656 | K | P | S | Q | H | Y | Q | L | H | P | E | R | K | - | R | S | E | S | S | Q | P | D | D | T | D | I | P | P | P | K | H | L | E | R | I | N | I | D | A | P | M | I | A | G | D | I | T | L | L | 703 | | Q6CM16/1-1873 | 680 | K | P | S | S | S | Y | L | I | N | L | E | R | K | - | R | S | N | A | T | A | - | N | S | S | N | V | - | T | A | T | Y | S | H | R | V | N | I | D | S | P | M | L | A | G | D | V | T | L | I | 725 | | Q6FRV8/1-1904 | 706 | K | P | S | P | R | Y | Q | L | H | P | E | K | K | - | R | S | I | S | E | A | N | N | A | S | D | I | P | N | P | R | P | N | E | N | I | N | I | D | I | P | M | I | N | G | D | V | T | L | L | 753 | | Q758L7/1-1866 | 668 | K | P | S | Q | S | Y | Q | F | N | T | E | R | K | - | R | S | S | M | T | A | L | N | K | S | D | I | P | M | P | L | H | T | E | H | V | N | I | D | A | P | M | I | A | G | D | V | T | L | L | 715 | | A7TGL6/1-1902 | 704 | K | P | S | Q | H | Y | K | L | H | P | D | K | K | - | R | S | L | S | E | A | T | I | E | S | D | I | P | A | P | K | N | S | E | H | V | N | I | D | A | P | M | I | A | G | D | V | M | L | L | 751 | | C5DII9/1-1880 | 679 | K | P | S | P | H | Y | Q | L | Q | S | E | R | K | - | R | G | - | A | A | L | E | P | Q | S | D | I | P | P | P | A | N | S | E | R | V | N | I | D | A | P | M | I | A | G | D | V | T | L | L | 725 | | C5DP10/1-1883 | 679 | K | I | S | Q | H | Y | Q | L | H | P | E | K | K | - | R | S | L | S | E | S | N | F | D | S | D | I | P | A | P | K | N | S | E | R | V | N | I | D | E | P | M | I | A | G | D | I | T | L | L | 726 | | Kwal\_YGOB\_27.12097/1-1546 | 612 | K | P | S | P | H | Y | Q | L | L | S | E | R | K | - | R | N | - | A | A | P | D | V | Q | S | D | I | P | P | P | A | N | S | E | R | V | N | I | D | A | P | M | I | A | G | D | V | T | L | L | 658 | | Sbay\_632.22/1-1867 | 663 | K | P | S | Q | H | Y | Q | L | H | P | E | K | K | - | R | S | I | S | E | A | T | T | D | S | D | I | P | I | P | K | S | N | E | H | I | N | I | D | A | P | M | I | A | G | D | I | T | L | L | 710 | | SAKL0H09724g/1-1862 | 661 | K | P | S | Q | H | Y | Q | L | H | S | E | K | K | R | R | S | S | S | S | S | T | I | K | P | D | I | P | P | P | A | N | N | E | R | I | N | I | D | A | P | M | I | A | G | D | V | N | L | L | 709 | | P32333/1-1867 | 663 | K | P | S | Q | H | Y | Q | L | H | P | E | K | K | - | R | S | I | S | E | T | T | T | D | S | D | I | P | I | P | K | N | N | E | H | I | N | I | D | A | P | M | I | A | G | D | I | T | L | L | 710 | |  | | G0VCI3/1-1859 | 704 | G | P | D | V | I | I | N | T | R | V | M | A | A | R | A | F | G | L | T | L | A | M | F | Q | D | S | T | L | Q | S | F | F | T | N | V | L | V | R | C | L | D | L | P | F | S | T | P | R | M | 752 | | Q6CM16/1-1873 | 726 | G | L | D | I | I | Y | N | T | R | V | K | A | A | K | T | L | G | L | T | L | S | F | F | Q | E | S | T | L | R | S | F | F | E | N | V | L | A | S | C | L | D | L | P | Y | S | T | P | R | M | 774 | | Q6FRV8/1-1904 | 754 | G | E | Q | K | I | M | N | T | R | V | L | A | A | K | A | F | G | F | T | L | A | M | F | Q | E | A | T | V | Q | S | F | F | A | N | V | L | V | R | C | L | D | L | P | Y | A | T | P | R | M | 802 | | Q758L7/1-1866 | 716 | G | A | D | V | I | F | K | T | R | V | L | A | A | K | A | L | G | Y | T | L | A | A | F | Q | E | S | T | V | K | S | F | F | E | T | A | L | L | S | C | L | D | L | P | Y | A | T | P | R | M | 764 | | A7TGL6/1-1902 | 752 | G | K | E | I | I | I | N | T | R | V | M | G | A | K | A | F | G | L | T | L | A | M | L | Q | E | S | T | L | Q | S | F | V | S | N | V | L | V | R | C | L | D | L | P | F | A | T | P | R | M | 800 | | C5DII9/1-1880 | 726 | G | T | D | V | I | F | N | T | R | V | T | G | A | K | A | L | G | I | T | L | S | M | F | Q | K | S | T | L | K | S | F | F | T | N | V | L | L | D | C | L | R | L | P | Y | A | T | P | R | M | 774 | | C5DP10/1-1883 | 727 | G | A | E | V | I | Q | N | T | R | I | M | G | A | E | A | F | G | I | T | L | S | M | F | Q | E | S | T | L | Q | S | F | F | S | N | V | L | V | R | C | F | E | L | S | Y | A | T | P | R | M | 775 | | Kwal\_YGOB\_27.12097/1-1546 | 659 | G | T | E | V | I | F | N | T | R | V | T | G | A | K | A | L | G | I | T | L | S | L | F | Q | E | S | T | L | K | S | F | F | S | N | V | L | L | D | C | L | K | L | P | Y | A | T | P | R | M | 707 | | Sbay\_632.22/1-1867 | 711 | G | L | E | V | I | L | N | T | R | I | M | G | A | K | A | F | A | L | T | L | S | M | F | Q | D | S | T | L | Q | S | F | F | A | N | V | L | A | R | C | L | D | L | P | F | S | T | P | R | M | 759 | | SAKL0H09724g/1-1862 | 710 | G | S | D | V | I | F | N | T | R | V | T | G | A | K | A | L | G | L | T | L | S | M | F | Q | E | S | T | L | K | S | F | F | S | S | V | L | V | G | C | L | Q | L | P | Y | A | T | P | R | M | 758 | | P32333/1-1867 | 711 | G | L | D | V | I | L | N | T | R | I | M | G | A | K | A | F | A | L | T | L | S | M | F | Q | D | S | T | L | Q | S | F | F | T | N | V | L | V | R | C | L | E | L | P | F | S | T | P | R | M | 759 | |  | | G0VCI3/1-1859 | 753 | L | A | A | I | I | I | T | E | F | C | S | S | W | S | K | Q | - | - | H | P | E | Q | D | K | L | P | E | F | V | G | N | I | F | A | P | I | L | N | E | Q | L | S | N | P | E | L | F | P | V | 799 | | Q6CM16/1-1873 | 775 | L | V | A | I | V | L | S | S | F | C | T | N | W | K | E | N | - | - | Q | Q | H | N | P | - | V | P | A | F | M | S | T | L | F | S | S | T | F | L | G | F | L | T | G | A | T | T | L | P | V | 820 | | Q6FRV8/1-1904 | 803 | L | T | A | I | I | V | N | R | F | C | K | F | W | V | D | R | - | - | H | D | D | V | P | E | V | P | K | F | V | S | E | I | F | G | A | T | M | N | E | Q | L | N | N | P | S | K | L | P | V | 849 | | Q758L7/1-1866 | 765 | L | V | S | I | T | V | A | E | Y | C | S | R | W | T | F | L | - | - | H | P | E | T | A | F | P | P | S | F | V | A | D | Y | F | G | P | I | F | M | E | Y | L | F | D | P | S | K | L | P | V | 811 | | A7TGL6/1-1902 | 801 | L | A | G | I | I | L | T | D | I | C | S | N | W | S | L | C | - | - | N | V | N | N | P | T | I | P | S | F | I | F | D | V | F | G | S | V | L | N | E | Q | L | T | N | R | D | K | L | P | P | 847 | | C5DII9/1-1880 | 775 | L | V | A | I | I | I | S | E | F | C | S | N | L | A | N | Q | P | S | E | E | D | A | D | D | L | K | R | F | V | S | E | A | F | G | E | T | F | A | G | Q | L | T | D | P | S | A | L | P | I | 823 | | C5DP10/1-1883 | 776 | I | A | G | I | I | L | S | T | F | C | S | S | W | S | Q | L | - | - | H | P | E | T | - | E | I | P | S | F | I | Q | E | I | F | A | P | V | I | N | P | Q | L | L | N | P | D | D | L | P | T | 821 | | Kwal\_YGOB\_27.12097/1-1546 | 708 | L | V | A | I | I | I | S | D | V | C | A | N | I | S | N | Q | P | T | P | E | E | A | A | N | L | K | A | L | I | S | E | T | F | G | S | I | I | V | G | Q | L | T | D | P | S | A | L | P | T | 756 | | Sbay\_632.22/1-1867 | 760 | L | A | G | I | I | V | T | Q | F | C | S | S | W | L | Q | K | - | - | H | S | E | G | E | E | L | P | S | F | V | S | E | L | F | S | P | V | M | N | K | Q | L | L | N | R | D | G | F | P | V | 806 | | SAKL0H09724g/1-1862 | 759 | L | V | A | I | I | V | S | E | F | C | S | N | W | A | H | Y | - | - | H | P | D | Q | P | T | I | P | S | F | V | P | E | T | F | G | S | T | F | A | E | Y | L | T | E | P | D | K | L | P | T | 805 | | P32333/1-1867 | 760 | L | A | G | I | I | V | S | Q | F | C | S | S | W | L | Q | K | - | - | H | P | E | G | E | K | L | P | S | F | V | S | E | I | F | S | P | V | M | N | K | Q | L | L | N | R | D | E | F | P | V | 806 | |  | | G0VCI3/1-1859 | 800 | F | R | E | L | V | P | S | L | K | A | L | R | T | Q | C | Q | S | L | L | A | T | F | V | D | V | G | M | L | S | Q | Q | K | L | P | S | I | A | I | V | V | Q | G | E | T | E | A | G | P | Q | 848 | | Q6CM16/1-1873 | 821 | F | R | E | L | T | P | I | L | K | A | L | R | T | Q | C | Q | S | L | M | T | T | F | V | D | V | G | M | L | P | P | Q | R | V | P | A | I | A | I | I | V | K | G | E | P | E | A | G | P | E | 869 | | Q6FRV8/1-1904 | 850 | F | R | E | L | I | P | S | L | K | A | L | R | T | S | C | Q | N | L | F | A | T | F | V | D | V | G | M | L | P | Q | H | K | L | P | S | V | A | I | V | V | Q | G | E | S | E | A | G | P | E | 898 | | Q758L7/1-1866 | 812 | F | R | E | L | V | P | S | L | K | A | L | R | T | Q | C | Q | S | L | M | T | T | F | V | E | V | G | M | L | S | P | Q | R | L | P | Q | L | A | I | I | V | K | G | E | A | E | A | G | P | E | 860 | | A7TGL6/1-1902 | 848 | F | R | E | L | V | P | S | L | K | A | L | R | T | Q | C | Q | T | L | F | S | T | F | V | D | V | G | L | L | P | K | H | K | L | P | S | I | A | I | I | V | Q | G | E | T | E | A | G | P | E | 896 | | C5DII9/1-1880 | 824 | F | R | E | L | V | P | S | L | K | A | L | R | T | Q | C | Q | S | L | L | S | T | F | V | D | V | G | M | L | P | P | Q | R | L | P | Q | L | A | I | I | V | Q | G | E | A | E | A | G | P | E | 872 | | C5DP10/1-1883 | 822 | F | R | E | L | V | P | S | L | K | A | L | R | T | Q | C | Q | S | L | M | R | T | F | V | D | V | G | M | L | P | Q | H | K | F | P | N | L | P | I | I | V | Q | G | E | A | E | A | G | P | E | 870 | | Kwal\_YGOB\_27.12097/1-1546 | 757 | F | R | E | L | V | P | S | L | K | A | L | R | T | Q | C | Q | S | L | L | S | T | F | V | D | V | G | M | L | P | P | Q | R | L | P | Q | L | A | I | I | V | Q | G | E | S | E | A | G | P | E | 805 | | Sbay\_632.22/1-1867 | 807 | F | R | E | L | V | P | S | L | K | A | L | R | T | Q | C | Q | S | L | L | A | T | F | V | D | V | G | M | L | P | Q | Y | K | L | P | N | V | A | I | V | V | Q | G | E | T | E | A | G | P | H | 855 | | SAKL0H09724g/1-1862 | 806 | F | R | E | L | V | P | S | L | K | A | L | R | T | Q | C | Q | S | L | M | S | T | F | V | D | V | G | M | L | P | P | Q | K | V | P | Q | I | A | I | I | V | Q | G | E | A | E | S | G | P | E | 854 | | P32333/1-1867 | 807 | F | R | E | L | V | P | S | L | K | A | L | R | T | Q | C | Q | S | L | L | A | T | F | V | D | V | G | M | L | P | Q | Y | K | L | P | N | V | A | I | V | V | Q | G | E | T | E | A | G | P | H | 855 | |  | | G0VCI3/1-1859 | 849 | A | F | S | I | E | T | A | E | K | V | H | N | E | Y | Y | E | K | M | F | R | S | M | N | N | S | Y | K | L | L | A | K | K | P | L | E | D | A | R | Y | R | V | A | L | A | I | E | A | T | K | 897 | | Q6CM16/1-1873 | 870 | A | F | S | I | Q | T | A | E | K | V | Q | T | E | F | Y | A | K | L | F | S | L | L | P | N | A | Q | K | I | L | A | Q | K | P | L | E | D | A | R | Y | R | V | S | L | A | I | E | S | A | K | 918 | | Q6FRV8/1-1904 | 899 | A | F | G | L | E | T | A | E | K | V | Y | H | D | Y | Y | D | K | M | Y | K | N | L | G | N | S | Y | K | L | L | A | K | K | P | L | E | D | A | K | H | R | V | K | Q | S | I | E | S | A | K | 947 | | Q758L7/1-1866 | 861 | A | F | Y | I | E | T | A | E | K | V | C | D | E | H | Y | E | K | L | Y | Q | Y | M | S | N | S | C | K | I | L | A | K | K | P | L | E | D | A | R | Y | R | I | Q | Q | A | I | D | V | A | K | 909 | | A7TGL6/1-1902 | 897 | A | F | G | I | E | T | A | E | K | V | H | N | E | Y | Y | E | K | M | F | R | S | L | G | N | S | Y | K | L | L | A | K | K | P | L | E | D | A | R | Y | R | I | L | L | A | I | E | A | A | K | 945 | | C5DII9/1-1880 | 873 | A | F | G | I | E | T | A | E | K | T | Y | G | E | M | S | D | K | L | F | R | H | L | S | N | S | Y | K | I | L | A | K | K | P | V | D | D | A | K | H | R | V | L | Q | A | I | E | T | A | K | 921 | | C5DP10/1-1883 | 871 | A | F | N | I | S | L | A | E | K | V | H | N | E | Y | Y | D | K | M | F | R | S | M | G | N | S | Y | K | L | L | A | K | K | P | L | E | D | A | K | Y | R | V | F | L | A | I | E | A | A | K | 919 | | Kwal\_YGOB\_27.12097/1-1546 | 806 | A | F | G | I | E | T | A | E | K | T | Y | S | E | T | S | D | K | L | F | R | H | L | S | N | S | Y | K | I | L | A | K | K | P | V | E | D | A | K | H | R | V | L | Q | A | I | E | I | A | K | 854 | | Sbay\_632.22/1-1867 | 856 | A | F | G | I | E | T | A | E | K | V | Y | G | E | Y | Y | D | K | M | F | K | S | M | N | N | S | Y | K | L | L | A | K | K | P | L | E | D | S | K | H | R | V | L | M | A | I | D | A | A | K | 904 | | SAKL0H09724g/1-1862 | 855 | A | F | G | I | E | T | A | D | K | V | C | G | E | Y | Y | E | K | L | F | R | H | L | N | N | S | Y | K | I | L | A | K | K | P | L | E | D | A | R | Y | R | V | Q | Q | A | I | D | A | A | R | 903 | | P32333/1-1867 | 856 | A | F | G | V | E | T | A | E | K | V | Y | G | E | Y | Y | D | K | M | F | K | S | M | N | N | S | Y | K | L | L | A | K | K | P | L | E | D | S | K | H | R | V | L | M | A | I | N | S | A | K | 904 | |  | | G0VCI3/1-1859 | 898 | E | S | R | R | A | H | N | G | S | I | L | S | N | Y | A | S | A | L | L | L | L | K | G | I | P | P | K | L | N | P | V | I | R | A | L | M | D | S | V | K | L | E | K | N | E | K | L | Q | T | 946 | | Q6CM16/1-1873 | 919 | E | S | E | R | E | R | Q | L | E | V | L | S | S | Y | A | S | A | V | M | L | I | D | G | L | P | N | K | L | N | P | L | I | R | S | L | M | D | N | V | K | S | E | K | H | T | I | L | Q | Q | 967 | | Q6FRV8/1-1904 | 948 | E | S | K | Q | K | R | I | C | S | I | L | S | N | Y | A | S | S | V | L | M | F | S | E | L | P | P | K | L | N | P | I | I | R | S | L | M | D | S | V | K | E | E | P | N | E | A | L | Q | K | 996 | | Q758L7/1-1866 | 910 | E | V | R | R | S | R | N | S | N | I | L | A | S | Y | A | S | A | T | L | L | F | D | G | L | P | K | K | L | N | P | F | I | R | S | L | M | D | S | I | K | E | E | Q | C | E | I | L | Q | K | 958 | | A7TGL6/1-1902 | 946 | E | A | K | L | A | R | E | C | S | I | L | S | N | Y | A | S | V | I | I | Q | F | D | G | L | P | E | K | L | N | P | I | I | R | S | L | M | D | S | I | K | E | E | K | N | L | K | L | Q | E | 994 | | C5DII9/1-1880 | 922 | D | A | Q | K | S | R | V | C | N | V | L | A | N | Y | A | S | A | A | L | L | F | D | G | L | P | A | K | L | N | P | F | I | R | A | L | M | D | S | I | K | E | E | R | Y | E | I | L | Q | K | 970 | | C5DP10/1-1883 | 920 | E | S | R | R | A | R | N | S | S | I | L | S | N | Y | A | A | A | S | L | R | F | G | G | L | P | T | K | L | N | P | I | I | R | A | F | M | D | S | I | K | E | E | K | N | G | K | L | Q | R | 968 | | Kwal\_YGOB\_27.12097/1-1546 | 855 | E | A | R | R | S | R | N | C | N | V | L | A | N | Y | A | S | G | A | L | L | F | D | G | L | P | A | K | L | N | P | F | I | R | A | L | M | D | S | I | K | E | E | R | Y | E | I | L | Q | R | 903 | | Sbay\_632.22/1-1867 | 905 | E | S | A | K | L | R | T | G | S | I | L | A | N | Y | A | S | S | I | L | L | F | D | G | L | P | P | K | L | N | P | I | I | R | S | L | M | D | S | V | K | E | E | R | N | E | K | L | Q | K | 953 | | SAKL0H09724g/1-1862 | 904 | E | A | R | R | S | R | A | C | N | V | L | A | N | Y | A | S | A | K | L | L | F | D | G | L | P | K | K | L | N | P | F | I | R | A | L | M | D | S | I | K | E | E | Q | Y | E | I | L | Q | R | 952 | | P32333/1-1867 | 905 | E | S | A | K | L | R | T | G | S | I | L | A | N | Y | A | S | S | I | L | L | F | D | G | L | P | L | K | L | N | P | I | I | R | S | L | M | D | S | V | K | E | E | R | N | E | K | L | Q | T | 953 | |  | | G0VCI3/1-1859 | 947 | M | A | G | D | A | V | I | H | L | I | D | V | L | I | K | N | N | K | G | N | A | A | N | K | I | V | K | N | L | S | G | F | L | C | V | D | T | S | E | V | P | D | F | T | A | N | S | K | Y | 995 | | Q6CM16/1-1873 | 968 | T | S | G | D | S | I | V | N | L | I | S | E | L | V | S | A | Q | K | H | N | V | A | N | K | I | V | K | N | L | C | G | F | I | C | V | D | T | S | E | V | P | E | F | D | G | T | N | - | - | 1014 | | Q6FRV8/1-1904 | 997 | M | S | C | E | S | V | I | Y | L | I | H | E | L | L | K | C | N | K | A | P | V | A | N | K | I | V | K | N | L | C | G | F | L | C | V | D | T | S | E | V | P | D | F | Q | Q | N | L | S | Y | 1045 | | Q758L7/1-1866 | 959 | R | S | G | D | S | V | I | Y | L | I | M | E | L | V | K | N | G | K | V | N | V | A | N | K | V | V | K | N | L | C | G | F | L | C | V | D | T | A | E | V | P | E | F | S | P | N | M | H | Y | 1007 | | A7TGL6/1-1902 | 995 | I | S | G | N | S | V | I | H | L | I | N | K | L | L | S | N | N | K | A | G | V | A | N | K | V | V | K | N | L | C | G | F | L | C | V | D | T | Q | E | V | P | E | F | S | S | N | E | I | Y | 1043 | | C5DII9/1-1880 | 971 | R | S | G | D | A | I | L | N | L | V | V | E | L | V | K | A | K | K | G | N | V | A | N | K | I | V | K | N | L | C | G | F | L | C | V | D | T | S | E | V | P | E | F | S | P | N | S | Q | Y | 1019 | | C5DP10/1-1883 | 969 | L | A | G | D | S | I | I | Y | L | I | K | Q | L | L | E | N | D | R | A | N | V | A | N | K | I | C | K | N | L | C | G | F | L | C | V | D | T | S | E | V | P | E | F | A | A | H | S | Q | L | 1017 | | Kwal\_YGOB\_27.12097/1-1546 | 904 | R | S | G | D | A | I | L | N | L | V | V | E | L | V | K | A | Q | K | G | S | V | A | N | K | I | V | K | N | L | C | G | F | L | C | V | D | T | S | E | V | P | E | F | A | P | N | S | H | Y | 952 | | Sbay\_632.22/1-1867 | 954 | M | A | G | E | S | V | V | H | L | I | Q | Q | L | L | E | N | N | K | V | N | V | S | G | K | I | V | K | N | L | C | G | F | L | C | V | D | T | S | E | V | P | D | F | S | V | N | A | D | F | 1002 | | SAKL0H09724g/1-1862 | 953 | R | S | G | D | S | I | I | H | L | I | T | E | L | V | K | N | D | K | N | V | V | S | N | K | I | V | K | N | L | C | G | F | L | C | V | D | T | S | E | V | P | E | F | L | P | N | I | D | Y | 1001 | | P32333/1-1867 | 954 | M | A | G | E | S | V | V | H | L | I | Q | Q | L | L | E | N | N | K | V | N | V | S | G | K | I | V | K | N | L | C | G | F | L | C | V | D | T | S | E | V | P | D | F | S | V | N | A | E | Y | 1002 | |  | | G0VCI3/1-1859 | 996 | I | D | S | I | L | T | S | I | K | E | G | S | T | L | A | I | Q | E | D | S | N | L | K | K | L | A | G | I | A | Q | L | K | R | K | G | G | L | Y | T | L | G | K | L | L | Q | V | F | G | A | 1044 | | Q6CM16/1-1873 | 1015 | T | T | V | I | T | T | L | V | K | E | A | A | S | L | S | L | Q | E | D | S | E | M | K | K | M | T | E | S | A | R | I | K | R | K | G | G | M | H | T | L | M | K | L | L | I | K | F | Q | E | 1063 | | Q6FRV8/1-1904 | 1046 | Q | D | R | L | Y | T | F | I | K | E | P | D | A | F | V | I | N | E | N | V | E | L | M | K | V | A | E | E | A | R | L | K | R | K | G | G | I | Y | A | M | G | T | L | L | E | I | C | G | S | 1094 | | Q758L7/1-1866 | 1008 | T | D | S | I | L | T | L | I | R | E | A | N | S | L | A | V | Q | D | N | A | S | I | K | L | M | T | K | E | A | Q | I | K | R | R | G | A | M | H | T | L | S | C | L | L | L | Q | L | G | P | 1056 | | A7TGL6/1-1902 | 1044 | K | E | N | I | L | T | L | V | K | E | T | A | G | L | G | L | V | D | D | A | R | L | K | R | I | T | D | E | A | H | I | K | R | R | G | G | L | Y | V | I | G | E | I | F | S | K | F | G | D | 1092 | | C5DII9/1-1880 | 1020 | R | D | S | I | L | T | L | I | R | E | Q | T | A | L | A | F | Q | D | D | A | N | V | K | K | L | A | E | E | A | Q | I | K | R | K | G | A | L | Y | T | L | S | E | F | L | L | R | L | G | P | 1068 | | C5DP10/1-1883 | 1018 | K | D | S | M | L | T | I | V | K | E | N | S | P | P | P | S | P | D | D | V | H | L | Q | R | L | T | E | E | A | Q | I | K | R | R | G | G | L | Y | T | L | G | Q | L | L | Q | L | F | G | S | 1066 | | Kwal\_YGOB\_27.12097/1-1546 | 953 | K | D | S | I | L | T | L | I | R | E | Q | T | A | L | A | F | Q | D | D | A | N | A | K | K | L | A | E | D | A | Q | I | K | R | K | G | A | L | Y | T | L | S | E | F | L | L | R | L | G | P | 1001 | | Sbay\_632.22/1-1867 | 1003 | K | E | K | I | L | T | L | I | K | E | S | N | S | I | A | A | Q | D | D | I | N | L | A | K | M | S | E | E | A | Q | L | K | R | K | G | G | L | I | T | L | K | T | L | F | E | V | L | G | P | 1051 | | SAKL0H09724g/1-1862 | 1002 | K | D | S | I | L | T | L | I | K | E | L | N | V | L | A | L | K | D | D | I | K | I | R | K | M | T | E | E | A | Q | V | K | R | K | G | A | M | Y | V | L | S | E | L | L | Q | K | F | G | T | 1050 | | P32333/1-1867 | 1003 | K | E | K | I | L | T | L | I | K | E | S | N | S | I | A | A | Q | D | D | I | N | L | A | K | M | S | E | E | A | Q | L | K | R | K | G | G | L | I | T | L | K | I | L | F | E | V | L | G | P | 1051 | |  | | G0VCI3/1-1859 | 1045 | N | T | L | V | N | I | P | Q | L | K | T | V | L | F | E | P | L | A | K | A | D | E | V | E | D | E | - | Q | N | T | I | N | N | I | L | G | Q | E | I | V | D | A | L | G | V | L | R | A | V | 1092 | | Q6CM16/1-1873 | 1064 | K | T | L | E | V | V | P | Q | L | K | K | S | L | F | E | P | L | E | K | I | D | I | L | S | S | N | G | D | E | T | T | F | K | K | T | G | Q | E | I | V | D | A | F | G | I | I | R | A | L | 1112 | | Q6FRV8/1-1904 | 1095 | S | A | L | D | D | I | P | Q | L | K | K | V | F | L | E | P | L | E | N | I | S | D | V | S | - | - | - | - | - | S | I | D | V | I | K | G | Q | A | V | T | D | Y | F | G | I | V | R | V | L | 1138 | | Q758L7/1-1866 | 1057 | N | A | L | T | Q | V | P | Q | L | K | A | S | I | F | D | P | L | L | S | F | G | D | L | D | S | D | - | S | D | S | I | E | P | E | V | G | Q | Q | L | V | D | A | L | G | V | L | R | A | L | 1104 | | A7TGL6/1-1902 | 1093 | N | A | F | D | - | I | E | Q | L | K | T | I | I | L | E | P | I | K | E | T | I | K | S | S | - | - | - | D | E | D | I | N | S | K | R | G | Q | S | L | V | D | A | M | G | I | L | R | A | I | 1137 | | C5DII9/1-1880 | 1069 | T | V | L | E | V | V | P | Q | V | K | Q | M | I | F | D | P | L | E | R | V | D | S | E | F | G | - | - | G | V | P | P | D | A | K | A | G | Q | E | V | V | D | A | L | G | V | L | R | A | L | 1115 | | C5DP10/1-1883 | 1067 | E | T | F | Q | R | V | T | Q | F | H | S | C | L | F | E | P | L | D | N | S | S | E | W | K | E | - | - | K | D | S | L | T | D | V | V | G | Q | S | V | V | D | A | L | G | A | L | R | A | T | 1113 | | Kwal\_YGOB\_27.12097/1-1546 | 1002 | S | V | L | E | V | V | P | Q | V | K | Q | L | I | F | D | P | L | E | K | V | D | R | D | F | N | - | - | G | I | P | P | D | A | K | T | G | Q | E | V | V | D | A | L | G | V | L | R | A | L | 1048 | | Sbay\_632.22/1-1867 | 1052 | S | V | L | Q | T | L | P | Q | L | K | A | I | L | F | D | S | L | C | S | H | E | N | V | E | - | - | - | I | E | K | I | D | K | E | Q | G | Q | R | I | V | D | S | F | G | I | L | R | A | L | 1097 | | SAKL0H09724g/1-1862 | 1051 | E | A | L | D | V | V | P | Q | M | K | Q | S | I | F | D | P | L | N | K | L | D | E | L | N | S | - | - | Q | K | D | A | D | G | K | L | G | Q | E | I | V | D | S | F | G | I | L | R | A | L | 1097 | | P32333/1-1867 | 1052 | S | I | L | Q | K | L | P | Q | L | R | S | I | L | F | D | S | L | S | D | H | E | N | E | E | - | - | - | A | S | K | V | D | N | E | Q | G | Q | K | I | V | D | S | F | G | V | L | R | A | L | 1097 | |  | | G0VCI3/1-1859 | 1093 | Y | P | Y | M | D | R | K | L | Q | I | E | E | V | S | T | H | Y | Q | E | I | L | A | I | L | R | S | K | F | S | V | L | R | Y | S | A | A | R | T | L | A | D | F | A | K | I | T | A | V | D | 1141 | | Q6CM16/1-1873 | 1113 | F | T | Y | M | P | E | S | V | Q | D | E | E | V | L | S | R | L | P | V | F | R | D | F | L | R | S | N | V | S | V | I | R | Y | S | A | A | R | T | L | S | E | L | A | C | H | K | P | V | V | 1161 | | Q6FRV8/1-1904 | 1139 | F | P | Y | M | D | Q | S | L | Q | E | S | V | V | I | A | K | F | P | R | M | L | D | F | L | K | C | P | Y | S | V | I | R | Y | S | V | A | R | T | F | A | D | L | A | K | Y | K | P | I | K | 1187 | | Q758L7/1-1866 | 1105 | F | T | Y | M | D | P | V | I | H | L | E | H | V | F | T | R | L | H | D | L | L | K | Y | L | T | S | R | Y | S | V | V | R | Y | S | A | A | R | T | L | A | E | L | A | V | A | N | P | T | Q | 1153 | | A7TGL6/1-1902 | 1138 | Y | M | F | M | S | P | F | I | Q | E | N | Q | V | L | P | C | F | P | N | L | L | T | L | L | R | S | K | Y | S | V | L | R | Y | S | A | A | R | T | F | G | D | L | A | M | I | S | H | V | A | 1186 | | C5DII9/1-1880 | 1116 | I | V | Y | M | S | E | E | L | Q | T | N | E | I | L | P | R | L | P | L | I | L | K | Y | L | R | S | E | L | A | V | F | R | Y | S | A | A | R | T | L | A | D | L | A | N | T | L | T | I | Q | 1164 | | C5DP10/1-1883 | 1114 | Y | S | Y | M | S | G | E | L | K | T | N | E | I | W | P | R | F | P | I | L | L | E | L | L | R | C | R | F | S | V | I | R | Y | S | A | A | R | T | F | A | D | L | A | K | L | S | P | I | P | 1162 | | Kwal\_YGOB\_27.12097/1-1546 | 1049 | I | V | F | M | S | K | E | V | Q | T | N | E | V | L | P | R | L | P | H | L | L | R | Y | L | R | S | D | L | A | V | F | R | Y | S | A | A | R | T | L | A | D | L | A | N | T | L | T | I | Q | 1097 | | Sbay\_632.22/1-1867 | 1098 | F | P | F | M | S | A | S | L | R | S | D | E | V | F | T | R | F | P | I | L | L | T | F | L | R | S | S | L | S | V | F | R | Y | S | A | A | R | T | F | A | D | L | A | K | I | S | S | V | E | 1146 | | SAKL0H09724g/1-1862 | 1098 | F | V | Y | M | D | S | S | L | Q | R | D | E | V | I | S | R | L | P | L | I | L | K | Y | L | R | S | N | F | S | V | F | R | Y | S | A | A | R | T | L | A | D | V | S | S | V | L | P | I | Q | 1146 | | P32333/1-1867 | 1098 | F | P | F | M | S | D | S | L | R | S | S | E | V | F | T | R | F | P | V | L | L | T | F | L | R | S | N | L | S | V | F | R | Y | S | A | A | R | T | F | A | D | L | A | K | I | S | S | V | E | 1146 | |  | | G0VCI3/1-1859 | 1142 | S | M | P | F | I | I | R | E | I | L | P | L | M | N | S | A | G | S | L | P | D | R | Q | G | A | T | E | L | I | Y | H | L | S | L | S | M | R | T | D | I | L | P | Y | V | I | F | L | I | V | 1190 | | Q6CM16/1-1873 | 1162 | L | M | S | Y | V | I | T | D | I | L | P | M | L | N | N | A | G | S | V | T | D | R | Q | G | V | V | E | L | L | Y | H | L | S | I | Q | L | E | S | D | I | L | P | Y | V | V | F | L | I | V | 1210 | | Q6FRV8/1-1904 | 1188 | V | M | P | F | L | I | K | N | V | L | P | M | I | N | D | A | G | S | L | F | N | R | Q | G | A | T | E | L | I | F | H | L | S | V | S | M | E | A | D | I | L | P | Y | V | I | F | L | I | V | 1236 | | Q758L7/1-1866 | 1154 | V | I | P | F | I | I | K | E | V | L | P | L | M | N | N | A | G | S | V | I | A | R | Q | S | G | T | E | L | V | Y | H | L | C | Q | S | M | G | S | D | I | L | P | Y | I | V | F | L | V | V | 1202 | | A7TGL6/1-1902 | 1187 | V | M | T | F | V | I | E | N | V | L | P | L | M | N | N | A | G | S | V | T | D | R | Q | G | A | T | E | L | I | Y | H | L | S | V | S | M | G | T | D | I | L | P | Y | V | I | F | L | I | V | 1235 | | C5DII9/1-1880 | 1165 | V | L | P | F | I | I | Q | T | A | L | P | L | M | S | N | P | S | S | V | T | D | R | Q | G | L | T | E | L | V | Y | H | L | A | L | Y | M | G | S | N | I | L | P | Y | V | V | F | L | I | V | 1213 | | C5DP10/1-1883 | 1163 | V | M | T | F | I | I | R | E | V | L | P | L | L | N | S | A | G | S | T | I | D | R | Q | G | A | T | E | L | V | Y | H | L | S | I | S | M | S | H | D | I | L | P | Y | V | I | F | L | I | V | 1211 | | Kwal\_YGOB\_27.12097/1-1546 | 1098 | V | I | P | F | I | I | Q | M | A | L | P | L | M | N | N | P | S | S | V | T | D | R | Q | G | V | T | E | L | V | Y | H | L | A | L | Y | M | G | S | N | I | L | P | Y | V | V | F | L | I | V | 1146 | | Sbay\_632.22/1-1867 | 1147 | V | M | A | F | T | I | R | E | I | L | P | L | M | N | S | A | G | S | L | S | D | R | Q | G | S | T | E | L | I | Y | H | L | S | L | S | M | E | T | D | V | L | P | Y | V | I | F | L | I | V | 1195 | | SAKL0H09724g/1-1862 | 1147 | V | I | P | F | I | I | K | E | V | L | P | L | M | N | N | A | G | S | V | V | D | R | Q | G | G | T | E | L | V | Y | H | L | S | L | S | M | G | S | N | I | L | P | Y | V | V | F | L | I | V | 1195 | | P32333/1-1867 | 1147 | V | M | A | Y | T | I | R | E | I | L | P | L | M | N | S | A | G | S | L | S | D | R | Q | G | S | T | E | L | I | Y | H | L | S | L | S | M | E | T | D | V | L | P | Y | V | I | F | L | I | V | 1195 | |  | | G0VCI3/1-1859 | 1191 | P | L | L | G | R | M | S | D | S | N | E | D | I | R | N | I | A | T | S | T | F | A | S | I | I | K | L | V | P | L | E | A | G | I | A | D | P | E | S | L | P | K | D | L | V | A | G | R | E | 1239 | | Q6CM16/1-1873 | 1211 | P | L | L | G | R | M | S | D | S | N | E | D | I | R | K | L | A | T | T | T | F | A | S | I | I | K | L | V | P | L | E | A | G | I | A | A | P | V | G | L | S | E | E | L | L | R | G | R | E | 1259 | | Q6FRV8/1-1904 | 1237 | P | L | L | G | R | M | S | D | P | N | E | D | I | R | N | L | A | T | T | T | F | A | S | I | I | K | I | V | P | L | E | E | G | I | K | D | P | E | G | M | P | E | E | L | M | K | G | R | E | 1285 | | Q758L7/1-1866 | 1203 | P | L | L | G | R | M | S | D | P | A | P | D | V | R | S | L | A | T | T | T | F | A | S | I | I | K | L | V | P | L | E | A | G | I | A | D | P | E | G | L | P | E | E | L | L | R | G | R | E | 1251 | | A7TGL6/1-1902 | 1236 | P | L | L | G | R | M | S | D | T | D | P | D | V | R | G | L | A | T | S | T | F | A | S | I | I | K | L | V | P | L | E | E | G | I | A | D | P | E | G | L | P | E | S | L | M | V | G | R | E | 1284 | | C5DII9/1-1880 | 1214 | P | L | L | G | R | M | S | D | S | N | Q | D | I | R | T | L | A | T | S | T | F | A | S | I | I | K | L | V | P | L | E | A | G | I | P | D | P | E | G | L | P | Q | D | L | M | E | G | R | E | 1262 | | C5DP10/1-1883 | 1212 | P | L | L | G | R | M | S | D | S | N | T | D | V | R | S | L | S | T | T | T | F | A | L | I | I | K | L | V | P | L | E | A | G | I | A | D | P | E | G | L | P | E | D | L | M | A | G | R | E | 1260 | | Kwal\_YGOB\_27.12097/1-1546 | 1147 | P | L | L | G | R | M | S | D | S | N | Q | D | I | R | T | L | A | T | S | T | F | A | S | I | I | K | L | V | P | L | E | A | G | I | P | D | P | E | G | L | P | Q | E | L | M | E | G | R | E | 1195 | | Sbay\_632.22/1-1867 | 1196 | P | L | L | G | R | M | S | D | S | N | E | D | V | R | N | L | A | T | T | T | F | A | S | I | I | K | L | V | P | L | E | A | G | I | A | D | P | K | G | L | P | E | E | L | V | A | S | R | E | 1244 | | SAKL0H09724g/1-1862 | 1196 | P | L | L | G | R | M | S | D | S | N | Q | D | V | R | T | L | A | T | T | T | F | A | S | I | I | K | L | V | P | L | E | A | G | I | A | D | P | E | G | L | P | Q | E | L | M | E | G | R | E | 1244 | | P32333/1-1867 | 1196 | P | L | L | G | R | M | S | D | S | N | E | D | V | R | N | L | A | T | T | T | F | A | S | I | I | K | L | V | P | L | E | A | G | I | A | D | P | K | G | L | P | E | E | L | V | A | S | R | E | 1244 | |  | | G0VCI3/1-1859 | 1240 | K | E | R | D | F | I | Q | Q | M | M | D | P | S | K | A | K | P | F | K | L | P | V | A | I | K | A | T | L | R | K | Y | Q | Q | D | G | V | N | W | L | A | F | L | N | K | Y | R | L | H | G | 1288 | | Q6CM16/1-1873 | 1260 | K | E | R | D | F | I | Q | Q | M | M | D | P | S | K | A | K | P | F | K | L | P | V | A | I | K | A | T | L | R | K | Y | Q | Q | D | G | V | N | W | L | A | F | L | N | K | Y | H | L | H | G | 1308 | | Q6FRV8/1-1904 | 1286 | R | E | R | D | F | I | K | Q | M | M | D | P | S | K | A | K | P | F | K | L | P | V | A | I | K | A | T | L | R | K | Y | Q | Q | D | G | I | N | W | L | A | F | L | N | K | Y | H | L | H | G | 1334 | | Q758L7/1-1866 | 1252 | R | E | R | D | F | I | Q | Q | M | M | D | P | S | K | A | K | P | F | S | L | P | V | A | I | K | A | T | L | R | K | Y | Q | Q | E | G | I | N | W | L | A | F | L | N | H | Y | H | L | H | G | 1300 | | A7TGL6/1-1902 | 1285 | R | E | R | D | F | I | Q | Q | M | M | D | P | S | K | A | K | P | F | K | L | P | V | A | I | K | A | T | L | R | K | Y | Q | Q | E | G | V | N | W | L | A | F | L | N | K | Y | H | L | H | G | 1333 | | C5DII9/1-1880 | 1263 | K | E | R | D | F | I | Q | Q | M | M | D | P | S | K | A | K | P | F | Q | C | P | V | A | I | K | A | T | L | R | K | Y | Q | Q | D | G | V | N | W | L | A | F | L | N | K | Y | H | L | H | G | 1311 | | C5DP10/1-1883 | 1261 | R | E | R | D | F | I | Q | Q | M | M | D | S | S | K | A | K | P | F | K | L | P | I | A | I | K | A | T | L | R | K | Y | Q | Q | E | G | V | N | W | L | A | F | L | N | K | Y | H | L | H | G | 1309 | | Kwal\_YGOB\_27.12097/1-1546 | 1196 | R | E | R | D | F | I | Q | Q | M | M | D | P | S | K | A | K | S | F | Q | C | P | V | A | I | K | A | T | L | R | K | Y | Q | Q | D | G | V | N | W | L | A | F | L | N | K | Y | H | L | H | G | 1244 | | Sbay\_632.22/1-1867 | 1245 | R | E | R | D | F | I | Q | Q | M | M | D | P | S | K | A | K | P | F | K | L | P | I | A | I | K | A | T | L | R | K | Y | Q | Q | D | G | V | N | W | L | A | F | L | N | K | Y | H | L | H | G | 1293 | | SAKL0H09724g/1-1862 | 1245 | K | E | R | D | F | I | Q | Q | M | M | D | P | S | K | A | K | P | F | K | L | P | V | A | I | K | A | T | L | R | K | Y | Q | Q | E | G | V | N | W | L | A | F | L | N | K | Y | H | L | H | G | 1293 | | P32333/1-1867 | 1245 | R | E | R | D | F | I | Q | Q | M | M | D | P | S | K | A | K | P | F | K | L | P | I | A | I | K | A | T | L | R | K | Y | Q | Q | D | G | V | N | W | L | A | F | L | N | K | Y | H | L | H | G | 1293 | |  | | G0VCI3/1-1859 | 1289 | I | L | C | D | D | M | G | L | G | K | T | L | Q | T | I | C | I | I | A | S | D | Q | Y | L | R | A | E | D | Y | K | K | T | N | S | V | E | T | R | K | L | P | S | L | I | V | C | P | P | S | 1337 | | Q6CM16/1-1873 | 1309 | I | L | C | D | D | M | G | L | G | K | T | L | Q | T | I | C | I | I | A | S | D | Q | Y | L | R | S | E | D | Y | K | K | T | Q | S | E | K | T | R | P | L | P | S | L | I | I | C | P | P | S | 1357 | | Q6FRV8/1-1904 | 1335 | I | L | C | D | D | M | G | L | G | K | T | L | Q | T | I | C | I | I | A | S | D | Q | Y | L | R | Q | E | E | Y | K | L | S | G | N | I | E | C | R | P | L | P | S | L | I | V | C | P | P | S | 1383 | | Q758L7/1-1866 | 1301 | I | L | C | D | D | M | G | L | G | K | T | L | Q | T | I | C | I | I | A | S | D | Q | Y | L | R | Q | E | D | Y | K | T | T | K | S | V | E | T | R | P | L | P | S | L | I | V | C | P | P | S | 1349 | | A7TGL6/1-1902 | 1334 | I | L | C | D | D | M | G | L | G | K | T | L | Q | T | I | C | I | I | A | S | D | Q | Y | L | R | Q | E | D | F | K | E | T | K | S | V | E | T | R | P | L | P | S | L | I | I | C | P | P | S | 1382 | | C5DII9/1-1880 | 1312 | I | L | C | D | D | M | G | L | G | K | T | L | Q | T | I | C | I | I | A | S | D | Q | Y | M | R | N | E | N | Y | E | K | T | K | A | R | E | S | R | P | L | P | S | L | I | I | C | P | P | S | 1360 | | C5DP10/1-1883 | 1310 | I | L | C | D | D | M | G | L | G | K | T | L | Q | A | I | C | I | I | A | S | D | Q | Y | L | R | R | Q | D | Y | E | K | T | H | S | V | E | T | R | P | L | P | S | L | I | V | C | P | P | S | 1358 | | Kwal\_YGOB\_27.12097/1-1546 | 1245 | I | L | C | D | D | M | G | L | G | K | T | L | Q | T | I | C | I | I | A | S | D | Q | Y | L | R | N | E | N | Y | Q | K | T | K | A | R | E | S | R | P | L | P | S | L | I | V | C | P | P | S | 1293 | | Sbay\_632.22/1-1867 | 1294 | I | L | C | D | D | M | G | L | G | K | T | L | Q | T | I | C | V | I | A | S | D | Q | Y | L | R | K | E | D | Y | E | K | T | H | S | V | E | S | R | P | L | P | S | L | I | I | C | P | P | S | 1342 | | SAKL0H09724g/1-1862 | 1294 | I | L | C | D | D | M | G | L | G | K | T | L | Q | T | I | C | I | I | A | S | D | Q | Y | M | R | Q | E | D | Y | K | I | T | K | S | I | E | T | R | P | L | S | S | L | I | V | C | P | P | S | 1342 | | P32333/1-1867 | 1294 | I | L | C | D | D | M | G | L | G | K | T | L | Q | T | I | C | I | I | A | S | D | Q | Y | L | R | K | E | D | Y | E | K | T | R | S | V | E | S | R | A | L | P | S | L | I | I | C | P | P | S | 1342 | |  | | G0VCI3/1-1859 | 1338 | L | T | G | H | W | E | N | E | F | E | Q | Y | A | P | F | L | K | I | I | V | Y | A | G | G | P | S | M | R | I | P | L | R | D | E | L | G | S | A | D | I | V | I | T | S | Y | D | V | A | R | 1386 | | Q6CM16/1-1873 | 1358 | L | T | G | H | W | E | Q | E | F | Q | Q | Y | S | P | T | L | N | V | L | V | Y | A | G | G | P | S | V | R | Y | P | L | Q | G | Q | V | P | T | A | D | I | V | V | T | S | Y | D | V | A | R | 1406 | | Q6FRV8/1-1904 | 1384 | L | T | G | H | W | E | N | E | F | E | Q | Y | S | P | F | L | K | I | V | V | Y | A | G | G | P | S | V | R | Q | P | L | R | K | Q | L | S | S | A | D | I | I | I | T | S | Y | D | V | A | R | 1432 | | Q758L7/1-1866 | 1350 | L | T | G | H | W | E | Q | E | F | E | Q | Y | A | P | F | L | T | V | L | V | Y | A | G | G | P | S | T | R | Y | P | L | R | G | K | L | G | D | A | D | I | V | V | T | S | Y | D | V | A | R | 1398 | | A7TGL6/1-1902 | 1383 | L | T | G | H | W | E | N | E | F | E | V | Y | S | P | F | L | N | V | V | V | Y | A | G | G | P | S | T | R | Q | S | L | K | E | R | L | A | D | A | D | I | I | V | T | S | Y | D | V | A | R | 1431 | | C5DII9/1-1880 | 1361 | L | T | G | H | W | E | Q | E | F | E | Q | Y | A | P | F | L | K | V | L | V | F | A | G | G | P | S | T | R | Y | P | L | R | D | K | L | G | S | A | D | I | V | I | T | S | Y | D | V | A | R | 1409 | | C5DP10/1-1883 | 1359 | L | T | G | H | W | E | A | E | F | E | E | Y | S | P | F | L | K | I | V | V | Y | A | G | G | P | S | T | R | Y | P | L | R | D | K | L | H | E | A | D | I | I | V | T | S | Y | D | V | A | R | 1407 | | Kwal\_YGOB\_27.12097/1-1546 | 1294 | L | T | G | H | W | E | Q | E | F | E | Q | Y | A | P | F | L | K | V | L | V | F | A | G | G | P | S | A | R | Y | P | L | R | D | K | L | G | D | A | D | I | V | I | T | S | Y | D | V | A | R | 1342 | | Sbay\_632.22/1-1867 | 1343 | L | T | G | H | W | E | N | E | F | D | Q | Y | A | P | F | L | K | V | V | I | Y | A | G | G | P | T | V | R | S | S | L | R | P | Q | L | Q | N | A | D | I | I | V | T | S | Y | D | V | A | R | 1391 | | SAKL0H09724g/1-1862 | 1343 | L | T | G | H | W | E | Q | E | F | E | Q | Y | A | P | F | L | K | V | L | V | Y | A | G | G | P | S | A | R | Y | P | L | R | D | Q | L | D | T | A | D | I | V | I | T | S | Y | D | V | A | R | 1391 | | P32333/1-1867 | 1343 | L | T | G | H | W | E | N | E | F | D | Q | Y | A | P | F | L | K | V | V | V | Y | A | G | G | P | T | V | R | L | T | L | R | P | Q | L | S | D | A | D | I | I | V | T | S | Y | D | V | A | R | 1391 | |  | | G0VCI3/1-1859 | 1387 | N | D | L | S | I | I | T | K | Y | D | F | N | Y | C | V | L | D | E | G | H | I | I | K | N | A | Q | S | K | L | A | K | A | V | K | Q | I | S | A | N | H | R | L | I | L | T | G | T | P | I | 1435 | | Q6CM16/1-1873 | 1407 | N | D | V | D | F | L | K | K | Y | D | Y | N | Y | C | V | L | D | E | G | H | I | I | K | N | S | Q | S | K | L | A | K | A | V | K | L | I | N | S | N | H | R | L | V | L | T | G | T | P | I | 1455 | | Q6FRV8/1-1904 | 1433 | N | D | L | D | T | I | S | S | Y | D | Y | N | Y | C | V | L | D | E | G | H | L | I | K | N | A | Q | S | K | L | A | K | A | V | K | L | I | K | A | N | H | R | L | I | L | T | G | T | P | I | 1481 | | Q758L7/1-1866 | 1399 | N | D | I | D | I | I | S | K | H | D | Y | N | Y | C | V | L | D | E | G | H | I | I | K | N | S | Q | S | K | L | A | K | A | V | K | S | I | R | A | N | H | R | L | I | L | T | G | T | P | I | 1447 | | A7TGL6/1-1902 | 1432 | N | D | L | S | V | I | T | K | Y | D | Y | N | Y | C | V | L | D | E | G | H | I | I | K | N | S | Q | S | K | L | A | K | A | V | K | S | I | A | A | N | H | R | L | I | L | T | G | T | P | I | 1480 | | C5DII9/1-1880 | 1410 | N | D | I | D | V | I | N | N | Y | D | Y | N | Y | C | V | L | D | E | G | H | I | I | K | N | A | Q | S | K | L | A | K | A | V | K | L | V | S | A | N | H | R | L | I | L | T | G | T | P | I | 1458 | | C5DP10/1-1883 | 1408 | N | D | L | S | V | I | A | Q | F | D | Y | N | Y | C | I | L | D | E | G | H | I | I | K | N | A | Q | S | K | L | A | K | A | V | K | Q | F | R | S | N | H | R | L | I | L | T | G | T | P | I | 1456 | | Kwal\_YGOB\_27.12097/1-1546 | 1343 | N | D | I | E | V | I | T | K | Y | D | Y | N | Y | C | V | L | D | E | G | H | I | I | K | N | A | Q | S | K | L | A | K | A | V | K | L | V | S | A | N | H | R | L | I | L | T | G | T | P | I | 1391 | | Sbay\_632.22/1-1867 | 1392 | N | D | L | A | V | L | T | K | T | E | Y | N | Y | C | V | L | D | E | G | H | I | I | K | N | S | Q | S | K | L | A | K | A | V | K | E | I | T | A | N | H | R | L | I | L | T | G | T | P | I | 1440 | | SAKL0H09724g/1-1862 | 1392 | N | D | I | D | I | I | A | K | Y | D | Y | N | Y | C | V | L | D | E | G | H | I | I | K | N | A | Q | S | K | L | S | K | G | V | K | Q | I | R | A | N | H | R | V | V | L | T | G | T | P | I | 1440 | | P32333/1-1867 | 1392 | N | D | L | A | V | L | N | K | T | E | Y | N | Y | C | V | L | D | E | G | H | I | I | K | N | S | Q | S | K | L | A | K | A | V | K | E | I | T | A | N | H | R | L | I | L | T | G | T | P | I | 1440 | |  | | G0VCI3/1-1859 | 1436 | Q | N | N | V | V | E | L | W | S | L | F | D | F | L | M | P | G | F | L | G | T | E | K | M | F | Q | E | K | F | A | K | P | I | A | A | S | R | N | S | K | T | S | S | K | E | Q | E | A | G | 1484 | | Q6CM16/1-1873 | 1456 | Q | N | N | V | V | E | L | W | S | L | F | D | F | L | M | P | G | F | L | G | T | E | K | M | F | Q | E | R | F | A | K | P | I | A | S | S | R | N | S | K | T | S | S | K | E | Q | E | A | G | 1504 | | Q6FRV8/1-1904 | 1482 | Q | N | N | V | V | E | L | W | S | L | F | D | F | L | M | P | G | F | L | G | T | E | K | S | F | Q | E | R | F | A | K | P | I | A | A | S | R | N | S | K | T | S | S | K | E | Q | E | A | G | 1530 | | Q758L7/1-1866 | 1448 | Q | N | N | V | V | E | L | W | S | L | F | D | F | L | M | P | G | F | L | G | S | E | K | A | F | Q | E | R | F | A | K | P | I | A | A | S | R | N | S | K | T | S | S | K | E | Q | E | A | G | 1496 | | A7TGL6/1-1902 | 1481 | Q | N | N | V | V | E | L | W | S | L | F | D | F | L | M | P | G | F | L | G | T | E | K | M | F | Q | E | R | F | A | K | P | I | A | A | S | R | N | S | K | S | S | S | K | E | Q | E | Q | G | 1529 | | C5DII9/1-1880 | 1459 | Q | N | N | V | V | E | L | W | S | L | F | D | F | L | M | P | G | F | L | G | T | E | K | M | F | H | E | R | F | A | K | P | V | A | A | S | R | N | S | K | T | S | S | K | E | Q | E | A | G | 1507 | | C5DP10/1-1883 | 1457 | Q | N | N | V | V | E | L | W | S | L | F | D | F | L | M | P | G | F | L | G | T | E | K | M | F | Q | E | R | F | A | K | P | I | A | A | S | R | N | S | K | T | S | S | K | E | Q | E | A | G | 1505 | | Kwal\_YGOB\_27.12097/1-1546 | 1392 | Q | N | N | V | V | E | L | W | S | L | F | D | F | L | M | P | G | F | L | G | T | E | K | M | F | H | E | R | F | A | K | P | V | A | A | S | R | N | S | K | T | S | S | R | E | Q | E | A | G | 1440 | | Sbay\_632.22/1-1867 | 1441 | Q | N | N | V | L | E | L | W | S | L | F | D | F | L | M | P | G | F | L | G | T | E | K | M | F | Q | E | R | F | A | K | P | I | A | A | S | R | N | S | K | T | S | S | K | E | Q | E | A | G | 1489 | | SAKL0H09724g/1-1862 | 1441 | Q | N | N | V | V | E | L | W | S | L | F | D | F | L | M | P | G | F | L | G | T | E | K | M | F | Q | E | R | F | A | K | P | I | A | A | S | R | N | S | K | T | S | S | R | E | Q | E | A | G | 1489 | | P32333/1-1867 | 1441 | Q | N | N | V | L | E | L | W | S | L | F | D | F | L | M | P | G | F | L | G | T | E | K | M | F | Q | E | R | F | A | K | P | I | A | A | S | R | N | S | K | T | S | S | K | E | Q | E | A | G | 1489 | |  | | G0VCI3/1-1859 | 1485 | V | L | A | L | E | A | L | H | K | Q | V | L | P | F | M | L | R | R | L | K | E | D | V | L | S | D | L | P | P | K | I | I | Q | D | Y | Y | C | E | L | S | D | L | Q | K | Q | L | Y | Q | D | 1533 | | Q6CM16/1-1873 | 1505 | A | L | A | L | E | A | L | H | K | Q | V | L | P | F | M | L | R | R | L | K | E | E | V | L | S | D | L | P | P | K | I | I | Q | D | Y | Y | C | E | L | S | D | L | Q | K | Q | L | Y | N | D | 1553 | | Q6FRV8/1-1904 | 1531 | A | L | A | L | E | A | L | H | K | Q | V | L | P | F | M | L | R | R | L | K | E | D | V | L | S | D | L | P | P | K | I | I | Q | D | Y | Y | C | E | L | S | D | L | Q | K | Q | L | Y | E | D | 1579 | | Q758L7/1-1866 | 1497 | A | L | A | L | E | A | L | H | K | Q | V | L | P | F | M | L | R | R | L | K | E | D | V | L | S | D | L | P | P | K | I | I | Q | D | Y | Y | C | E | L | S | D | L | Q | R | Q | L | Y | K | D | 1545 | | A7TGL6/1-1902 | 1530 | T | L | A | L | E | A | L | H | K | Q | V | L | P | F | M | L | R | R | L | K | E | D | V | L | S | D | L | P | P | K | I | I | Q | D | Y | Y | C | E | L | S | D | L | Q | K | Q | L | Y | K | D | 1578 | | C5DII9/1-1880 | 1508 | A | L | A | L | D | A | L | H | K | Q | V | L | P | F | M | L | R | R | L | K | E | D | V | L | S | D | L | P | P | K | I | I | Q | D | Y | Y | C | E | L | S | D | L | Q | K | Q | L | Y | K | D | 1556 | | C5DP10/1-1883 | 1506 | A | L | A | L | E | A | L | H | K | Q | V | L | P | F | M | L | R | R | L | K | E | D | V | L | S | D | L | P | P | K | I | I | Q | D | Y | H | C | E | L | S | D | L | Q | K | Q | L | Y | E | D | 1554 | | Kwal\_YGOB\_27.12097/1-1546 | 1441 | A | L | A | L | D | A | L | H | K | Q | V | L | P | F | M | L | R | R | L | K | E | D | V | L | S | D | L | P | P | K | I | I | Q | D | Y | Y | C | E | L | S | D | L | Q | K | Q | L | Y | K | D | 1489 | | Sbay\_632.22/1-1867 | 1490 | V | L | A | L | E | A | L | H | K | Q | V | L | P | F | M | L | R | R | L | K | E | D | V | L | S | D | L | P | P | K | I | I | Q | D | Y | Y | C | E | L | G | D | L | Q | K | Q | L | Y | M | D | 1538 | | SAKL0H09724g/1-1862 | 1490 | A | L | A | L | E | A | L | H | K | Q | V | L | P | F | M | L | R | R | L | K | E | D | V | L | S | D | L | P | P | K | I | I | Q | D | Y | Y | C | E | L | S | D | L | Q | K | Q | L | Y | K | D | 1538 | | P32333/1-1867 | 1490 | V | L | A | L | E | A | L | H | K | Q | V | L | P | F | M | L | R | R | L | K | E | D | V | L | S | D | L | P | P | K | I | I | Q | D | Y | Y | C | E | L | G | D | L | Q | K | Q | L | Y | M | D | 1538 | |  | | G0VCI3/1-1859 | 1534 | F | A | K | K | Q | K | N | V | V | E | K | D | I | E | N | T | T | D | T | D | N | S | Q | H | I | F | Q | A | L | Q | Y | M | R | K | L | C | N | H | P | A | L | V | L | S | P | N | H | P | Q | 1582 | | Q6CM16/1-1873 | 1554 | F | V | K | K | Q | K | N | V | V | E | K | D | I | E | N | T | A | E | V | E | N | K | Q | H | I | F | Q | A | L | Q | Y | M | R | K | L | C | N | H | P | S | L | V | L | N | S | S | H | P | Q | 1602 | | Q6FRV8/1-1904 | 1580 | F | A | K | K | Q | K | N | V | V | E | K | D | I | Q | N | T | A | D | V | D | S | K | Q | H | I | F | Q | A | L | Q | Y | M | R | K | L | C | N | H | P | A | L | V | L | S | N | D | H | P | Q | 1628 | | Q758L7/1-1866 | 1546 | F | A | K | K | Q | K | N | I | V | E | R | D | I | E | N | T | M | E | L | E | S | K | N | H | I | F | Q | A | L | Q | Y | M | R | K | L | C | N | H | P | S | L | V | L | S | K | D | H | P | Q | 1594 | | A7TGL6/1-1902 | 1579 | F | A | N | K | Q | K | N | V | V | E | K | D | I | Q | N | T | A | D | V | E | N | K | Q | H | I | F | Q | A | L | Q | Y | M | R | K | L | C | N | H | P | S | L | I | L | S | E | N | H | P | Q | 1627 | | C5DII9/1-1880 | 1557 | F | A | K | K | Q | K | N | N | V | E | Q | D | I | E | N | V | S | E | V | D | N | K | Q | H | I | F | Q | A | L | Q | Y | M | R | K | L | C | N | H | P | S | L | V | V | S | K | D | H | P | Q | 1605 | | C5DP10/1-1883 | 1555 | F | S | R | K | Q | K | T | V | V | Q | K | D | I | E | N | T | A | D | T | D | N | K | Q | H | I | F | Q | A | L | Q | Y | M | R | K | L | C | N | H | P | A | L | V | L | S | P | D | H | P | Q | 1603 | | Kwal\_YGOB\_27.12097/1-1546 | 1490 | F | A | K | K | Q | K | T | N | V | E | Q | D | I | E | N | A | T | E | V | D | N | K | Q | H | I | F | Q | A | L | Q | Y | L | R | K | L | C | N | H | P | S | L | V | V | S | K | D | H | P | Q | 1538 | | Sbay\_632.22/1-1867 | 1539 | F | A | K | K | Q | K | N | V | V | E | K | D | I | E | N | P | E | V | V | D | G | K | Q | H | I | F | Q | A | L | Q | Y | M | R | K | L | C | N | H | P | A | L | V | L | S | P | S | H | P | Q | 1587 | | SAKL0H09724g/1-1862 | 1539 | F | A | K | K | Q | K | N | V | V | E | K | D | I | E | T | T | A | E | V | D | S | K | Q | H | I | F | Q | A | L | Q | Y | M | R | K | L | C | N | H | P | S | L | V | L | S | K | D | H | P | Q | 1587 | | P32333/1-1867 | 1539 | F | T | K | K | Q | K | N | V | V | E | K | D | I | E | N | S | E | I | A | D | G | K | Q | H | I | F | Q | A | L | Q | Y | M | R | K | L | C | N | H | P | A | L | V | L | S | P | N | H | P | Q | 1587 | |  | | G0VCI3/1-1859 | 1583 | L | A | Q | V | Q | D | Y | L | K | Q | T | G | I | D | L | H | D | V | I | N | A | P | K | L | N | A | L | R | T | L | L | F | E | C | G | I | G | E | E | D | M | E | R | K | S | N | P | N | Q | 1631 | | Q6CM16/1-1873 | 1603 | F | Q | Q | V | Q | S | Y | L | S | Q | T | G | M | D | L | H | D | I | G | H | A | P | K | L | E | A | L | K | T | L | L | L | E | C | G | I | G | I | Q | D | V | E | K | K | S | - | - | N | K | 1649 | | Q6FRV8/1-1904 | 1629 | L | K | Q | I | Q | N | Y | L | K | Q | T | G | C | D | L | H | D | I | R | N | A | P | K | L | T | A | L | R | T | L | L | F | E | C | G | I | G | E | A | D | M | D | K | K | V | - | T | G | E | 1676 | | Q758L7/1-1866 | 1595 | Y | N | Q | V | Q | D | Y | L | S | Q | T | G | M | D | I | H | D | I | A | H | A | P | K | L | G | A | L | R | N | L | L | L | E | C | G | I | G | V | Q | D | V | D | Q | N | S | - | - | I | S | 1641 | | A7TGL6/1-1902 | 1628 | L | K | Q | V | D | E | Y | L | K | Q | T | G | L | G | L | H | D | I | V | N | A | P | K | L | G | A | L | R | N | L | L | F | E | C | G | I | G | E | D | D | M | D | K | K | S | - | T | D | Q | 1675 | | C5DII9/1-1880 | 1606 | W | S | Q | V | Q | D | Y | L | K | Q | T | G | F | S | L | H | D | I | T | H | A | P | K | L | G | A | L | K | N | L | L | L | E | C | G | I | G | I | Q | D | V | D | K | K | S | - | K | T | Y | 1653 | | C5DP10/1-1883 | 1604 | L | K | Q | V | E | S | Y | L | K | Q | T | H | M | D | L | H | D | I | S | N | A | P | K | L | L | A | L | R | N | L | L | F | E | C | G | I | G | E | Q | D | I | D | R | K | S | P | V | N | Q | 1652 | | Kwal\_YGOB\_27.12097/1-1546 | 1539 | W | - | - | - | - | - | - | - | - | - | - | - | - | - | - | - | - | - | - | - | - | - | - | - | - | - | - | - | - | - | - | - | - | - | - | - | - | - | - | - | - | - | - | - | - | - | - | - | - | | Sbay\_632.22/1-1867 | 1588 | L | S | Q | V | Q | D | Y | L | K | Q | T | G | L | D | L | H | D | I | I | N | A | P | K | L | S | A | L | R | T | L | L | F | E | C | G | I | G | E | E | D | M | D | K | S | S | C | Q | D | Q | 1636 | | SAKL0H09724g/1-1862 | 1588 | Y | Y | Q | V | Q | E | Y | L | K | Q | T | G | L | S | L | H | D | I | S | H | A | P | K | L | G | A | L | K | N | L | L | L | E | C | G | I | G | T | Q | D | F | E | M | K | N | - | N | H | Q | 1635 | | P32333/1-1867 | 1588 | L | A | Q | V | Q | D | Y | L | K | Q | T | G | L | D | L | H | D | I | I | N | A | P | K | L | S | A | L | R | T | L | L | F | E | C | G | I | G | E | E | D | I | D | K | K | A | S | Q | D | Q | 1636 | |  | | G0VCI3/1-1859 | 1632 | Y | L | T | G | Q | N | V | I | S | Q | H | R | A | L | I | F | C | Q | L | K | D | M | L | D | M | V | E | N | D | L | F | K | R | Y | M | P | S | V | T | Y | M | R | L | D | G | S | V | D | P | 1680 | | Q6CM16/1-1873 | 1650 | N | P | S | I | D | N | V | I | S | Q | H | R | V | L | I | F | C | Q | L | K | D | M | L | D | M | V | E | N | D | L | L | K | K | H | L | P | S | V | T | F | M | R | L | D | G | S | V | D | S | 1698 | | Q6FRV8/1-1904 | 1677 | Q | L | L | T | G | S | V | I | S | Q | H | R | A | L | I | F | C | Q | L | K | D | M | L | D | M | I | E | N | D | L | F | K | R | Y | M | P | S | V | S | Y | M | R | L | D | G | S | V | D | P | 1725 | | Q758L7/1-1866 | 1642 | L | P | S | S | E | N | V | I | S | Q | H | R | A | L | I | F | C | Q | L | K | D | M | L | D | M | I | E | N | D | L | F | K | K | Y | L | P | S | V | T | Y | M | R | L | D | G | S | V | E | S | 1690 | | A7TGL6/1-1902 | 1676 | L | V | P | S | N | T | V | I | S | Q | H | R | A | L | I | F | C | Q | L | K | D | M | L | D | L | V | E | N | D | L | F | K | K | Y | M | P | S | V | T | Y | M | R | L | D | G | S | V | D | P | 1724 | | C5DII9/1-1880 | 1654 | L | P | S | T | E | S | V | I | S | Q | H | R | A | L | I | F | C | Q | L | K | D | M | L | D | M | V | E | N | D | L | F | K | K | Y | M | P | S | V | T | Y | M | R | L | D | G | S | V | E | S | 1702 | | C5DP10/1-1883 | 1653 | L | P | S | T | V | N | V | I | S | Q | H | R | A | L | I | F | C | Q | L | K | D | M | L | D | M | V | E | N | D | L | F | K | R | Y | M | P | S | V | T | Y | M | R | L | D | G | S | V | E | A | 1701 | | Kwal\_YGOB\_27.12097/1-1546 | 1540 | - | - | - | - | - | - | - | - | - | - | - | - | - | - | - | - | - | - | - | - | - | - | - | - | - | - | - | - | - | - | - | - | - | - | - | - | - | V | T | S | S | R | L | P | - | - | - | - | - | 1546 | | Sbay\_632.22/1-1867 | 1637 | S | F | P | I | Q | N | V | I | S | Q | H | R | A | L | I | F | C | Q | L | K | D | M | L | D | M | V | E | N | D | L | F | K | K | Y | M | P | S | V | T | Y | M | R | L | D | G | S | V | D | P | 1685 | | SAKL0H09724g/1-1862 | 1636 | L | P | S | T | E | N | V | I | S | Q | H | R | A | L | I | F | C | Q | L | K | D | M | L | D | M | V | E | N | D | L | L | K | K | C | M | P | S | V | T | Y | M | R | L | D | G | S | V | E | S | 1684 | | P32333/1-1867 | 1637 | N | F | P | I | Q | N | V | I | S | Q | H | R | A | L | I | F | C | Q | L | K | D | M | L | D | M | V | E | N | D | L | F | K | K | Y | M | P | S | V | T | Y | M | R | L | D | G | S | I | D | P | 1685 | |  | | G0VCI3/1-1859 | 1681 | R | D | R | Q | K | V | V | R | K | F | N | E | D | P | S | I | D | C | L | L | L | T | T | K | V | G | G | L | G | L | N | L | T | G | A | D | T | V | I | F | V | E | H | D | W | N | P | M | N | 1729 | | Q6CM16/1-1873 | 1699 | R | D | R | Q | K | V | V | R | K | F | N | E | D | P | S | I | D | C | L | L | L | T | T | K | V | G | G | L | G | L | N | L | T | G | A | D | T | V | I | F | V | E | H | D | W | N | P | M | N | 1747 | | Q6FRV8/1-1904 | 1726 | R | D | R | Q | K | V | V | R | K | F | N | E | D | P | S | I | D | C | L | L | L | T | T | K | V | G | G | L | G | L | N | L | T | G | A | D | T | V | I | F | V | E | H | D | W | N | P | M | N | 1774 | | Q758L7/1-1866 | 1691 | R | D | R | Q | K | V | V | R | K | F | N | E | D | P | S | I | D | C | L | L | L | T | T | K | V | G | G | L | G | L | N | L | T | G | A | D | T | V | I | F | I | E | H | D | W | N | P | M | N | 1739 | | A7TGL6/1-1902 | 1725 | R | D | R | Q | K | V | V | R | K | F | N | E | D | P | S | I | D | C | L | L | L | T | T | K | V | G | G | L | G | L | N | L | T | G | A | D | T | V | I | F | I | E | H | D | W | N | P | M | N | 1773 | | C5DII9/1-1880 | 1703 | R | D | R | Q | A | V | V | R | K | F | N | E | D | P | S | I | D | C | L | L | L | T | T | K | V | G | G | L | G | L | N | L | T | G | A | D | T | V | I | F | I | E | H | D | W | N | P | M | N | 1751 | | C5DP10/1-1883 | 1702 | R | D | R | Q | K | V | V | R | K | F | N | E | D | P | S | I | D | C | L | L | L | T | T | K | V | G | G | L | G | L | N | L | T | G | A | D | T | V | I | F | V | E | H | D | W | N | P | M | N | 1750 | | Kwal\_YGOB\_27.12097/1-1546 |  | - | - | - | - | - | - | - | - | - | - | - | - | - | - | - | - | - | - | - | - | - | - | - | - | - | - | - | - | - | - | - | - | - | - | - | - | - | - | - | - | - | - | - | - | - | - | - | - | - | | Sbay\_632.22/1-1867 | 1686 | R | D | R | Q | N | V | V | R | K | F | N | E | D | P | S | I | D | C | L | L | L | T | T | K | V | G | G | L | G | L | N | L | T | G | A | D | T | V | I | F | V | E | H | D | W | N | P | M | N | 1734 | | SAKL0H09724g/1-1862 | 1685 | R | D | R | Q | K | V | V | R | K | F | N | E | D | P | S | I | D | C | L | L | L | T | T | K | V | G | G | L | G | L | N | L | T | G | A | D | T | V | I | F | I | E | H | D | W | N | P | M | N | 1733 | | P32333/1-1867 | 1686 | R | D | R | Q | K | V | V | R | K | F | N | E | D | P | S | I | D | C | L | L | L | T | T | K | V | G | G | L | G | L | N | L | T | G | A | D | T | V | I | F | V | E | H | D | W | N | P | M | N | 1734 | |  | | G0VCI3/1-1859 | 1730 | D | L | Q | A | M | D | R | A | H | R | L | G | Q | K | K | V | V | N | V | Y | R | I | I | T | K | G | T | L | E | E | K | I | M | G | L | Q | K | F | K | M | N | I | A | S | T | V | V | N | Q | 1778 | | Q6CM16/1-1873 | 1748 | D | L | Q | A | M | D | R | A | H | R | L | G | Q | K | K | V | V | N | V | Y | R | I | I | T | K | G | T | L | E | E | K | I | M | G | L | Q | K | F | K | M | N | I | A | S | T | I | V | N | Q | 1796 | | Q6FRV8/1-1904 | 1775 | D | L | Q | A | M | D | R | A | H | R | L | G | Q | K | K | V | V | N | V | Y | R | I | V | T | K | G | T | L | E | E | K | I | M | G | L | Q | K | F | K | M | N | I | A | S | T | V | V | N | Q | 1823 | | Q758L7/1-1866 | 1740 | D | L | Q | A | M | D | R | A | H | R | L | G | Q | K | K | V | V | N | V | Y | R | I | I | T | K | G | S | L | E | E | K | I | M | G | L | Q | K | F | K | M | N | I | A | S | T | V | V | N | Q | 1788 | | A7TGL6/1-1902 | 1774 | D | L | Q | A | M | D | R | A | H | R | L | G | Q | K | K | V | V | N | V | Y | R | I | I | T | K | G | T | L | E | E | K | I | M | G | L | Q | K | F | K | M | N | I | A | S | T | V | V | N | Q | 1822 | | C5DII9/1-1880 | 1752 | D | L | Q | A | M | D | R | A | H | R | L | G | Q | K | K | V | V | N | V | Y | R | I | I | T | R | G | T | L | E | E | K | I | M | G | L | Q | K | F | K | M | N | I | A | S | T | V | I | N | Q | 1800 | | C5DP10/1-1883 | 1751 | D | L | Q | A | M | D | R | A | H | R | L | G | Q | K | K | V | V | N | V | Y | R | I | V | T | K | G | T | L | E | E | K | I | M | G | L | Q | K | F | K | M | N | I | A | S | T | V | V | N | Q | 1799 | | Kwal\_YGOB\_27.12097/1-1546 |  | - | - | - | - | - | - | - | - | - | - | - | - | - | - | - | - | - | - | - | - | - | - | - | - | - | - | - | - | - | - | - | - | - | - | - | - | - | - | - | - | - | - | - | - | - | - | - | - | - | | Sbay\_632.22/1-1867 | 1735 | D | L | Q | A | M | D | R | A | H | R | I | G | Q | K | K | V | V | N | V | Y | R | I | I | T | K | G | T | L | E | E | K | I | M | G | L | Q | K | F | K | M | N | I | A | S | T | V | V | N | Q | 1783 | | SAKL0H09724g/1-1862 | 1734 | D | L | Q | A | M | D | R | A | H | R | L | G | Q | K | K | V | V | N | V | Y | R | I | I | T | K | G | T | L | E | E | K | I | M | G | L | Q | K | F | K | M | N | I | A | S | T | V | V | N | Q | 1782 | | P32333/1-1867 | 1735 | D | L | Q | A | M | D | R | A | H | R | I | G | Q | K | K | V | V | N | V | Y | R | I | I | T | K | G | T | L | E | E | K | I | M | G | L | Q | K | F | K | M | N | I | A | S | T | V | V | N | Q | 1783 | |  | | G0VCI3/1-1859 | 1779 | Q | N | S | G | L | A | S | M | D | T | H | Q | L | L | D | L | F | D | T | D | D | V | P | S | Q | E | N | E | E | K | Q | Q | S | - | - | - | V | K | P | G | M | D | D | V | A | N | E | T | G | 1824 | | Q6CM16/1-1873 | 1797 | Q | N | A | G | L | G | S | M | N | T | H | Q | L | L | D | L | F | D | A | D | N | I | P | S | Q | E | K | V | E | K | K | K | T | - | - | - | - | - | - | - | V | E | D | V | A | N | E | S | G | 1838 | | Q6FRV8/1-1904 | 1824 | Q | N | S | G | L | A | S | M | D | T | H | Q | L | L | D | L | F | D | T | D | N | V | P | S | N | N | A | G | E | V | A | E | S | - | - | - | G | D | K | L | P | D | D | V | A | N | E | T | G | 1869 | | Q758L7/1-1866 | 1789 | Q | N | A | G | L | A | S | M | D | T | H | Q | L | L | D | L | F | D | T | D | N | S | L | A | Q | V | K | E | E | K | A | G | A | - | - | - | - | - | - | I | S | D | D | V | T | N | E | T | G | 1831 | | A7TGL6/1-1902 | 1823 | Q | N | N | G | L | A | S | M | D | T | H | Q | L | L | D | L | F | D | T | N | N | V | P | N | Q | D | K | E | E | T | P | V | E | - | - | - | - | S | K | A | M | D | D | I | A | N | E | T | G | 1867 | | C5DII9/1-1880 | 1801 | Q | N | S | G | L | A | S | M | D | T | H | Q | L | L | D | L | F | D | T | D | N | V | P | S | Q | E | K | E | T | K | N | S | S | - | - | - | - | D | G | K | I | D | E | I | V | N | E | T | G | 1845 | | C5DP10/1-1883 | 1800 | Q | N | N | G | L | S | S | M | N | T | N | Q | L | L | D | L | F | D | T | D | N | V | P | S | Q | E | K | E | E | K | P | S | Q | A | A | N | G | E | A | G | I | E | E | V | A | N | E | T | G | 1848 | | Kwal\_YGOB\_27.12097/1-1546 |  | - | - | - | - | - | - | - | - | - | - | - | - | - | - | - | - | - | - | - | - | - | - | - | - | - | - | - | - | - | - | - | - | - | - | - | - | - | - | - | - | - | - | - | - | - | - | - | - | - | | Sbay\_632.22/1-1867 | 1784 | Q | N | S | G | L | A | S | M | D | T | H | Q | L | L | D | L | F | D | P | D | N | V | T | S | Q | D | S | E | E | K | N | N | N | D | S | Q | P | A | K | G | M | E | D | I | A | N | E | T | G | 1832 | | SAKL0H09724g/1-1862 | 1783 | Q | N | A | G | L | A | S | M | D | T | H | Q | L | L | D | L | F | D | T | D | N | V | P | G | Q | D | K | E | V | R | Q | S | S | - | - | - | - | N | G | K | T | D | D | I | V | N | E | T | G | 1827 | | P32333/1-1867 | 1784 | Q | N | S | G | L | A | S | M | D | T | H | Q | L | L | D | L | F | D | P | D | N | V | T | S | Q | D | N | E | E | K | N | N | G | D | S | Q | A | A | K | G | M | E | D | I | A | N | E | T | G | 1832 | |  | | G0VCI3/1-1859 | 1825 | L | T | G | K | A | K | E | V | V | G | E | L | K | E | L | W | D | P | S | Q | Y | E | E | E | Y | N | L | D | N | F | I | K | T | L | R |  | | | | | | | | | | | | | | 1859 | | Q6CM16/1-1873 | 1839 | L | T | G | K | A | K | E | A | V | G | E | L | K | E | L | W | D | S | S | Q | Y | E | E | E | Y | N | L | D | N | F | I | K | T | L | R |  | | | | | | | | | | | | | | 1873 | | Q6FRV8/1-1904 | 1870 | L | S | G | K | A | K | E | A | L | G | D | L | K | E | L | W | D | P | S | Q | Y | E | D | E | Y | N | L | D | N | F | I | K | T | L | K |  | | | | | | | | | | | | | | 1904 | | Q758L7/1-1866 | 1832 | L | T | G | K | A | K | E | A | V | S | E | L | K | E | L | W | D | S | S | Q | Y | E | E | E | Y | N | L | D | N | F | I | K | T | L | R |  | | | | | | | | | | | | | | 1866 | | A7TGL6/1-1902 | 1868 | L | S | G | K | A | K | E | A | L | G | G | L | K | E | L | W | D | S | S | Q | Y | E | D | E | Y | N | L | D | N | F | I | K | A | L | R |  | | | | | | | | | | | | | | 1902 | | C5DII9/1-1880 | 1846 | L | T | G | K | A | K | E | A | V | G | E | L | T | E | L | W | D | T | T | Q | Y | E | E | E | Y | N | L | D | N | F | I | K | T | L | R |  | | | | | | | | | | | | | | 1880 | | C5DP10/1-1883 | 1849 | L | S | G | K | A | K | E | A | L | G | E | L | K | E | L | W | D | P | S | Q | Y | E | E | E | Y | N | L | D | N | F | I | K | T | L | R |  | | | | | | | | | | | | | | 1883 | | Kwal\_YGOB\_27.12097/1-1546 |  | - | - | - | - | - | - | - | - | - | - | - | - | - | - | - | - | - | - | - | - | - | - | - | - | - | - | - | - | - | - | - | - | - | - | - |  | | | | | | | | | | | | | | | Sbay\_632.22/1-1867 | 1833 | L | T | G | K | A | K | E | A | L | G | E | L | K | E | L | W | D | P | S | Q | Y | E | E | E | Y | N | L | D | S | F | I | K | T | L | R |  | | | | | | | | | | | | | | 1867 | | SAKL0H09724g/1-1862 | 1828 | L | T | G | K | A | K | E | A | V | G | E | L | K | E | L | W | D | S | S | Q | Y | E | E | E | Y | N | L | D | N | F | I | K | T | L | R |  | | | | | | | | | | | | | | 1862 | | P32333/1-1867 | 1833 | L | T | G | K | A | K | E | A | L | G | E | L | K | E | L | W | D | P | S | Q | Y | E | E | E | Y | N | L | D | T | F | I | K | T | L | R |  | | | | | | | | | | | | | | 1867 | |
