# Supplementary material for: Plus ça change – evolutionary sequence divergence predicts protein subcellular localization signals
Source: BMC Genomics. 2014 Jan 20;15:46. doi: 10.1186/1471-2164-15-46 (PMC3906766; doi:10.1186/1471-2164-15-46)
Supplement: Additional file 2 — MSA’s of proteins for which sequence divergence changes predicted localization signals. Contains links to ortholog multiple sequence alignments of each protein in Additional file 3: Table S1. [file 1471-2164-15-46-S2.zip › P32898.html]

|  |  |  |  |  |  |  |  |  |  |  |  |  |  |  |  |  |  |  |  |  |  |  |  |  |  |  |  |  |  |  |  |  |  |  |  |  |  |  |  |  |  |  |  |  |  |  |  |  |  |  |  |  |  |  |  |  |  |  |  |  |  |  |  |  |  |  |  |  |  |  |  |  |  |  |  |  |  |  |  |  |  |  |  |  |  |  |  |  |  |  |  |  |  |  |  |  |  |  |  |  |  |  |  |  |  |  |  |  |  |  |  |  |  |  |  |  |  |  |  |  |  |  |  |  |  |  |  |  |  |  |  |  |  |  |  |  |  |  |  |  |  |  |  |  |  |  |  |  |  |  |  |  |  |  |  |  |  |  |  |  |  |  |  |  |  |  |  |  |  |  |  |  |  |  |  |  |  |  |  |  |  |  |  |  |  |  |  |  |  |  |  |  |  |  |  |  |  |  |  |  |  |  |  |  |  |  |  |  |  |  |  |  |  |  |  |  |  |  |  |  |  |  |  |  |  |  |  |  |  |  |  |  |  |  |  |  |  |  |  |  |  |  |  |  |  |  |  |  |  |  |  |  |  |  |  |  |  |  |  |  |  |  |  |  |  |  |  |  |  |  |  |  |  |  |  |  |  |  |  |  |  |  |  |  |  |  |  |  |  |  |  |  |  |  |  |  |  |  |  |  |  |  |  |  |  |  |  |  |  |  |  |  |  |  |  |  |  |  |  |  |  |  |  |  |  |  |  |  |  |  |  |  |  |  |  |  |  |  |  |  |  |  |  |  |  |  |  |  |  |  |  |  |  |  |  |  |  |  |  |  |  |  |  |  |  |  |  |  |  |  |  |  |  |  |  |  |  |  |  |  |  |  |  |  |  |  |  |  |  |  |  |  |  |  |  |  |  |  |  |  |  |  |  |  |  |  |  |  |  |  |  |  |  |  |  |  |  |  |  |  |  |  |  |  |  |  |  |  |  |  |  |  |  |  |  |  |  |  |  |  |  |  |  |  |  |  |  |  |  |  |  |  |  |  |  |  |  |  |  |  |  |  |  |  |  |  |  |  |  |  |  |  |  |  |  |  |  |  |  |  |  |  |  |  |  |  |  |  |  |  |  |  |  |  |  |  |  |  |  |  |  |  |  |  |  |  |  |  |  |  |  |  |  |  |  |  |  |  |  |  |  |  |  |  |  |  |  |  |  |  |  |  |  |  |  |  |  |  |  |  |  |  |  |  |  |  |  |  |  |  |  |  |  |  |  |  |  |  |  |  |  |  |  |  |  |  |  |  |  |  |  |  |  |  |  |  |  |  |  |  |  |  |  |  |  |  |  |  |  |  |  |  |  |  |  |  |  |  |  |  |  |  |  |  |  |  |  |  |  |  |  |  |  |  |  |  |  |  |  |  |  |  |  |  |  |  |  |  |  |  |  |  |  |  |  |  |  |  |  |  |  |  |  |  |  |  |  |  |  |  |  |  |  |  |  |  |  |  |  |  |  |  |  |  |  |  |  |  |  |  |  |  |  |  |  |  |  |  |  |  |  |  |  |  |  |  |  |  |  |  |  |  |  |  |  |  |  |  |  |  |  |  |  |  |  |  |  |  |  |  |  |  |  |  |  |  |  |  |  |  |  |  |  |  |  |  |  |  |  |  |  |  |  |  |  |  |  |  |  |  |  |  |  |  |  |  |  |  |  |  |  |  |  |  |  |  |  |  |  |  |  |  |  |  |  |  |  |  |  |  |  |  |  |  |  |  |  |  |  |  |  |  |  |  |  |  |  |  |  |  |  |  |  |  |  |  |  |  |  |  |  |  |  |  |  |  |  |  |  |  |  |  |  |  |  |  |  |  |  |  |  |  |  |  |  |  |  |  |  |  |  |  |  |  |  |  |  |  |  |  |  |  |  |  |  |  |  |  |  |  |  |  |  |  |  |  |  |  |  |  |  |  |  |  |  |  |  |  |  |  |  |  |  |  |  |  |  |  |  |  |  |  |  |  |  |  |  |  |  |  |  |  |  |  |  |  |  |  |  |  |  |  |  |  |  |  |  |  |  |  |  |  |  |  |  |  |  |  |  |  |  |  |  |  |  |  |  |  |  |  |  |  |  |  |  |  |  |  |  |  |  |  |  |  |  |  |  |  |  |  |  |  |  |  |  |  |  |  |  |  |  |  |  |  |  |  |  |  |  |  |  |  |  |  |  |  |  |  |  |  |  |  |  |  |  |  |  |  |  |  |  |  |  |  |  |  |  |  |  |  |  |  |  |  |  |  |  |  |  |  |  |  |  |  |  |  |  |  |  |  |  |  |  |  |  |  |  |  |  |  |  |  |  |  |  |  |  |  |  |  |  |  |  |  |  |  |  |  |  |  |  |  |  |  |  |  |  |  |  |  |  |  |  |  |  |  |  |  |  |  |  |  |  |  |  |  |  |  |  |  |  |  |  |  |  |  |  |  |  |  |  |  |  |  |  |  |  |  |  |  |  |  |  |  |  |  |  |  |  |  |  |  |  |  |  |  |  |  |  |  |  |  |  |  |  |  |  |  |  |  |  |  |  |  |  |  |  |  |  |  |  |  |  |  |  |  |  |  |  |  |  |  |  |  |  |  |  |  |  |  |  |  |  |  |  |  |  |  |  |  |  |  |  |  |  |  |  |  |  |  |  |  |  |  |  |  |  |  |  |  |  |  |  |  |  |  |  |  |  |  |  |  |  |  |  |  |  |  |  |  |  |  |  |  |  |  |  |  |  |  |  |  |  |  |  |  |  |  |  |  |  |  |  |  |  |  |  |  |  |  |  |  |  |  |  |  |  |  |  |  |  |  |  |  |  |  |  |  |  |  |  |  |  |  |  |  |  |  |  |  |  |  |  |  |  |  |  |  |  |  |  |  |  |  |  |  |  |  |  |  |  |  |  |  |  |  |  |  |  |  |  |  |  |  |  |  |  |  |  |  |  |  |  |  |  |  |  |  |  |  |  |  |  |  |  |  |  |  |  |  |  |  |  |  |  |  |  |  |  |  |  |  |  |  |  |  |  |  |  |  |  |  |  |  |  |  |  |  |  |  |  |  |  |  |  |  |  |  |  |  |  |  |  |  |  |  |  |  |  |  |  |  |  |  |  |  |  |  |  |  |  |  |  |  |  |  |  |  |  |  |  |  |  |  |  |  |  |  |  |  |  |  |  |  |  |  |  |  |  |  |  |  |  |  |  |  |  |  |  |  |  |  |  |  |  |  |  |  |  |  |  |  |  |  |  |  |  |  |  |  |  |  |  |  |  |  |  |  |  |  |  |  |  |  |  |  |  |  |  |  |  |  |  |  |  |  |  |  |  |  |  |  |  |  |  |  |  |  |  |  |  |  |  |  |  |  |  |  |  |  |  |  |  |  |  |  |  |  |  |  |  |  |  |  |  |  |  |  |  |  |  |  |  |  |  |  |  |  |  |  |  |  |  |  |  |  |  |  |  |  |  |  |  |  |  |  |  |  |  |  |  |  |  |  |  |  |  |  |  |  |  |  |  |  |  |  |  |  |  |  |  |  |  |  |  |  |  |  |  |  |  |  |  |  |  |  |  |  |  |  |  |  |  |  |  |  |  |  |  |  |  |  |  |  |  |  |  |  |  |  |  |  |  |  |  |  |  |  |  |  |  |  |  |  |  |  |  |  |  |  |  |  |  |  |  |  |  |  |  |  |  |  |  |  |  |  |  |  |  |  |  |  |  |  |  |  |  |  |  |  |  |  |  |  |  |  |  |  |  |  |  |  |  |  |  |  |  |  |  |  |  |  |  |  |  |  |  |  |  |  |  |  |  |  |  |  |  |  |  |  |  |  |  |  |  |  |  |  |  |  |  |  |  |  |  |  |  |  |  |  |  |  |  |  |  |  |  |  |  |  |  |  |  |  |  |  |  |  |  |  |  |  |  |  |  |  |  |  |  |  |  |  |  |  |  |  |  |  |  |  |  |  |  |  |  |  |  |  |  |  |  |  |  |  |  |  |  |  |  |  |  |  |  |  |  |  |  |  |  |  |  |  |  |  |  |  |  |  |  |  |  |  |  |  |  |  |  |  |  |  |  |  |  |  |  |  |  |  |  |  |  |  |  |  |  |  |  |  |  |  |  |  |  |  |  |  |  |  |  |  |  |  |  |  |  |  |  |  |  |  |  |  |  |  |  |  |  |  |  |  |  |  |  |  |  |  |  |  |  |  |  |  |  |  |  |  |  |  |  |  |  |  |  |  |  |  |  |  |  |  |  |  |  |  |  |  |  |  |  |  |  |  |  |  |  |  |  |  |  |  |  |  |  |  |  |  |  |  |  |  |  |  |  |  |  |  |  |  |  |  |  |  |  |  |  |  |  |  |  |  |  |  |  |  |  |  |  |  |  |  |  |  |  |  |  |  |  |  |  |  |  |  |  |  |  |  |  |  |  |  |  |  |  |  |  |  |  |  |  |  |  |  |  |  |  |  |  |  |  |  |  |  |  |  |  |  |  |  |  |  |  |  |  |  |  |  |  |  |  |  |  |  |  |  |  |  |  |  |  |  |  |  |  |  |  |  |  |  |  |  |  |  |  |  |  |  |  |  |  |  |  |  |  |  |  |  |  |  |  |  |  |  |  |  |  |  |  |  |  |  |  |  |  |  |  |  |  |  |  |  |  |  |  |  |  |  |  |  |  |  |  |  |  |  |  |  |  |  |  |  |  |  |  |  |  |  |  |  |  |  |  |  |  |  |  |  |  |  |  |  |  |  |  |  |  |  |  |  |  |  |  |  |  |  |  |  |  |  |  |  |  |  |  |  |  |  |  |  |  |  |  |  |  |  |  |  |  |  |  |  |  |  |  |  |  |  |  |  |  |  |  |  |  |  |  |  |  |  |  |  |  |  |  |  |  |  |  |  |  |  |  |  |  |  |  |  |  |  |  |  |  |  |  |  |  |  |  |  |  |  |  |  |  |  |  |  |  |  |  |  |  |  |  |  |  |  |  |  |  |  |  |  |  |  |  |  |  |  |  |  |  |  |  |  |  |  |  |  |  |  |  |  |  |  |  |  |  |  |  |  |  |  |  |  |  |  |  |  |  |  |  |  |  |  |  |  |  |  |  |  |  |  |  |  |  |  |  |  |  |  |  |  |  |  |  |  |  |  |  |  |  |  |  |  |  |  |  |  |  |  |  |  |  |  |  |  |  |  |  |  |  |  |  |  |  |  |  |  |  |  |  |  |  |  |  |  |  |  |  |  |  |  |  |  |  |  |  |  |  |  |  |  |  |  |  |  |  |  |  |  |  |  |  |  |  |  |  |  |  |  |  |  |  |  |  |  |  |  |  |  |  |  |  |  |  |  |  |  |  |  |  |  |  |  |  |  |  |  |  |  |  |  |  |  |  |  |  |  |  |  |  |  |  |  |  |  |  |  |  |  |  |  |  |  |  |  |  |  |  |  |  |  |  |  |  |  |  |  |  |  |  |  |  |  |  |  |  |  |  |  |  |  |  |  |  |  |  |  |  |  |  |  |  |  |  |  |  |  |  |  |  |  |  |  |  |  |  |  |  |  |  |  |  |  |  |  |  |  |  |  |  |  |  |  |  |  |  |  |  |  |  |  |  |  |  |  |  |  |  |  |  |  |  |  |  |  |  |  |  |  |  |  |  |  |  |  |  |  |  |  |  |  |  |  |  |  |  |  |  |  |  |  |  |  |  |  |  |  |  |  |  |  |  |  |  |  |  |  |  |  |  |  |  |  |  |  |  |  |  |  |  |  |  |  |  |  |  |  |  |  |  |  |  |  |  |  |  |  |  |  |  |  |  |  |  |  |  |  |  |  |  |  |  |  |  |  |  |  |  |  |  |  |  |  |  |  |  |  |  |  |  |  |  |  |  |  |  |  |  |  |  |  |  |  |  |  |  |  |  |  |  |  |  |  |  |  |  |  |  |  |  |  |  |  |  |  |  |  |  |  |  |  |  |  |  |  |  |  |  |  |  |  |  |  |  |  |  |  |  |  |  |  |  |  |  |  |  |  |  |  |  |  |  |  |  |  |  |  |  |  |  |  |  |  |  |  |  |  |  |  |  |  |  |  |  |  |  |  |  |  |  |  |  |  |  |  |  |  |  |  |  |  |  |  |  |  |  |  |  |  |  |  |  |  |  |  |  |  |  |  |  |  |  |  |  |  |  |  |  |  |  |  |  |  |  |  |  |  |  |  |  |  |  |  |  |  |  |  |  |  |  |  |  |  |  |  |  |  |  |  |  |  |  |  |  |  |  |  |  |  |  |  |  |  |  |  |  |  |  |  |  |  |  |  |  |  |  |  |  |  |  |  |  |  |  |  |  |  |  |  |  |  |  |  |  |  |  |  |  |  |  |  |  |  |  |  |  |  |  |  |  |  |  |  |  |  |  |  |  |  |  |  |  |  |  |  |  |  |  |  |  |  |  |  |  |  |  |  |  |  |  |  |  |  |  |  |  |  |  |  |  |  |  |  |  |  |  |  |  |  |  |  |  |  |  |  |  |  |  |  |  |  |  |  |  |  |  |  |  |  |  |  |  |  |  |  |  |  |  |  |  |  |  |  |  |  |  |  |  |  |  |  |  |  |  |  |  |  |  |  |  |  |  |  |  |  |  |  |  |  |  |  |  |  |  |  |  |  |  |  |  |  |  |  |  |  |  |  |  |  |  |  |  |  |  |  |  |  |  |  |  |  |  |  |  |  |  |  |  |  |  |  |  |  |  |  |  |  |  |  |  |  |  |  |  |  |  |  |  |  |  |  |  |  |  |  |  |  |  |  |  |  |  |  |  |  |  |  |  |  |  |  |  |  |  |  |  |  |  |  |  |  |  |  |  |  |  |  |  |  |  |  |  |  |  |  |  |  |  |  |  |  |  |  |  |  |  |  |  |  |  |  |  |  |  |  |  |  |  |  |  |  |  |  |  |  |  |  |  |  |  |  |  |  |  |  |  |  |  |  |  |  |  |  |  |  |  |  |  |  |  |  |  |  |  |  |  |  |  |  |  |  |  |  |  |  |  |  |  |  |  |  |  |  |  |  |  |  |  |  |  |  |  |  |  |  |  |  |  |  |  |  |  |  |  |  |  |  |  |  |  |  |  |  |  |  |  |  |  |  |  |  |  |  |  |  |  |  |  |  |  |  |  |  |  |  |  |  |  |  |  |  |  |  |  |  |  |  |  |  |  |  |  |  |  |  |  |  |  |  |  |  |  |  |  |  |  |  |  |  |  |  |  |  |  |  |  |  |  |  |  |  |  |  |  |  |  |  |  |  |  |  |  |  |  |  |  |  |  |  |  |  |  |  |  |  |  |  |  |  |  |  |  |  |  |  |  |  |  |  |  |  |  |  |  |  |  |  |  |  |  |  |  |  |  |  |  |  |  |  |  |  |  |  |  |  |  |  |  |  |  |  |  |  |  |  |  |  |  |  |  |  |  |  |  |  |  |  |  |  |  |  |  |  |  |  |  |  |  |  |  |  |  |  |  |  |  |  |  |  |  |  |  |  |  |  |  |  |  |  |  |  |  |  |  |  |  |  |  |  |  |  |  |  |  |  |  |  |  |  |  |  |  |  |  |  |  |  |  |  |  |  |  |  |  |  |  |  |  |  |  |  |  |  |  |  |  |  |  |  |  |  |  |  |  |  |  |  |  |  |  |  |  |  |  |  |  |  |  |  |  |  |  |  |  |  |  |  |  |  |  |  |  |  |  |  |  |  |  |  |  |  |  |  |  |  |  |  |  |  |  |  |  |  |  |  |  |  |  |  |  |  |  |  |  |  |  |  |  |  |  |  |  |  |  |  |  |  |  |  |  |  |  |  |  |  |  |  |  |  |  |  |  |  |  |  |  |  |  |  |  |  |  |  |  |  |  |  |  |  |  |  |  |  |  |  |  |  |  |  |  |  |  |  |  |  |  |  |  |  |  |  |  |  |  |  |  |  |  |  |  |  |  |  |  |  |  |  |  |  |  |  |  |  |  |  |  |  |  |  |  |  |  |  |  |  |  |  |  |  |  |  |  |  |  |  |  |  |  |  |  |  |  |  |  |  |  |  |  |  |  |  |  |  |  |  |  |  |  |  |  |  |  |  |  |  |  |  |  |  |  |  |  |  |  |  |  |  |  |  |  |  |  |  |  |  |  |  |  |  |  |  |  |  |  |  |  |  |  |  |  |  |  |  |  |  |  |  |  |  |  |  |  |  |  |  |  |  |  |  |  |  |  |  |  |  |  |  |  |  |  |  |  |  |  |  |  |  |  |  |  |  |  |  |  |  |  |  |  |  |  |  |  |  |  |  |  |  |  |  |  |  |  |  |  |  |  |  |  |  |  |  |  |  |  |  |  |  |  |  |  |  |  |  |  |  |  |  |  |  |  |  |  |  |  |  |  |  |  |  |  |  |  |  |  |  |  |  |  |  |  |  |  |  |  |  |  |  |  |  |  |  |  |  |  |  |  |  |  |  |  |  |  |  |  |  |  |  |  |  |  |  |  |  |  |  |  |  |  |  |  |  |  |  |  |  |  |  |  |  |  |  |  |  |  |  |  |  |  |  |  |  |  |  |  |  |  |  |  |  |  |  |  |  |  |  |  |  |  |  |  |  |  |  |  |  |  |  |  |  |  |  |  |  |  |  |  |  |  |  |  |  |  |  |  |  |  |  |  |  |  |  |  |  |  |  |  |  |  |  |  |  |  |  |  |  |  |  |  |  |  |  |  |  |  |  |  |  |  |  |  |  |  |  |  |  |  |  |  |  |  |  |  |  |  |  |  |  |  |  |  |  |  |  |  |  |  |  |  |  |  |  |  |  |  |  |  |  |  |  |  |  |  |  |  |  |  |  |  |  |  |  |  |  |  |  |  |  |  |  |  |  |  |  |  |  |  |  |  |  |  |  |  |  |  |  |  |  |  |  |  |  |  |  |  |  |  |  |  |  |  |  |  |  |  |  |  |  |  |  |  |  |  |  |  |  |  |  |  |  |  |  |  |  |  |  |  |  |  |  |  |  |  |  |  |  |  |  |  |  |  |  |  |  |  |  |  |  |  |  |  |  |  |  |  |  |  |  |  |  |  |  |  |  |  |  |  |  |  |  |  |  |  |  |  |  |  |  |  |  |  |  |  |  |  |  |  |  |  |  |  |  |  |  |  |  |  |  |  |  |  |  |  |  |  |  |  |  |  |  |  |  |  |  |  |  |  |  |  |  |  |  |  |  |  |  |  |  |  |  |  |  |  |  |  |  |  |  |  |  |  |  |  |  |  |  |  |  |  |  |  |  |  |  |  |  |  |  |  |  |  |  |  |  |  |  |  |  |  |  |  |  |  |  |  |  |  |  |  |  |  |  |  |  |  |  |  |  |  |  |  |  |  |  |  |  |  |  |  |  |  |  |  |  |  |  |  |  |  |  |  |  |  |  |  |  |  |  |  |  |  |  |  |  |  |  |  |  |  |  |  |  |  |  |  |  |  |  |  |  |  |  |  |  |  |  |  |  |  |  |  |  |  |  |  |  |  |  |  |  |  |  |  |  |  |  |  |  |  |  |  |  |  |  |  |  |  |  |  |  |  |  |  |  |  |  |  |  |  |  |  |  |  |  |  |  |  |  |  |  |  |  |  |  |  |  |  |  |  |  |  |  |  |  |  |  |  |  |  |  |  |  |  |  |  |  |  |  |  |  |  |  |  |  |  |  |  |  |  |  |  |  |  |  |  |  |  |  |  |  |  |  |  |  |  |  |  |  |  |  |  |  |  |  |  |  |  |  |  |  |  |  |  |  |  |  |  |  |  |  |  |  |  |  |  |  |  |  |  |  |  |  |  |  |  |  |  |  |  |  |  |  |  |  |  |  |  |  |  |  |  |  |  |  |  |  |  |  |  |  |  |  |  |  |  |  |  |  |  |  |  |  |  |  |  |  |  |  |  |  |  |  |  |  |  |  |  |  |  |  |  |  |  |  |  |  |  |  |  |  |  |  |  |  |  |  |  |  |  |  |  |  |  |  |  |  |  |  |  |  |  |  |  |  |  |  |  |  |  |  |  |  |  |  |  |  |  |  |  |  |  |  |  |  |  |  |  |  |  |  |  |  |  |  |  |  |  |  |  |  |  |  |  |  |  |  |  |  |  |  |  |  |  |  |  |  |  |  |  |  |  |  |  |  |  |  |  |  |  |  |  |  |  |  |  |  |  |  |  |  |  |  |  |  |  |  |  |  |  |  |  |  |  |  |  |  |  |  |  |  |  |  |  |  |  |  |  |  |  |  |  |  |  |  |  |  |  |  |  |  |  |  |  |  |  |  |  |  |  |  |  |  |  |  |  |  |  |  |  |  |  |  |  |  |  |  |  |  |  |  |  |  |  |  |  |  |  |  |  |  |  |  |  |  |  |  |  |  |  |  |  |  |  |  |  |  |  |  |  |  |  |  |  |  |  |  |  |  |  |  |  |  |  |  |  |  |  |  |  |  |  |  |  |  |  |  |  |  |  |  |  |  |  |  |  |  |  |  |  |  |  |  |  |  |  |  |  |  |  |  |  |  |  |  |  |  |  |  |  |  |  |  |  |  |  |  |  |  |  |  |  |  |  |  |  |  |  |  |  |  |  |  |  |  |  |  |  |  |  |  |  |  |  |  |  |  |  |  |  |  |  |  |  |  |  |  |  |  |  |  |  |  |  |  |  |  |  |  |  |  |  |  |  |  |  |  |  |  |  |  |  |  |  |  |  |  |  |  |  |  |  |  |  |  |  |  |  |  |  |  |  |  |  |  |  |  |  |  |  |  |  |  |  |  |  |  |  |  |  |  |  |  |  |  |  |  |  |  |  |  |  |  |  |  |  |  |  |  |  |  |  |  |  |  |  |  |  |  |  |  |  |  |  |  |  |  |  |  |  |  |  |  |  |  |  |  |  |  |  |  |  |  |  |  |  |  |  |  |  |  |  |  |  |  |  |  |  |  |  |  |  |  |  |  |  |  |  |  |  |  |  |  |  |  |  |  |  |  |  |  |  |  |  |  |  |  |  |  |  |  |  |  |  |  |  |  |  |  |  |  |  |  |  |  |  |  |  |  |  |  |  |  |  |  |  |  |  |  |  |  |  |  |  |  |  |  |  |  |  |  |  |  |  |  |  |  |  |  |  |  |  |  |  |  |  |  |  |  |  |  |  |  |  |  |  |  |  |  |  |  |  |  |  |  |  |  |  |  |  |  |  |  |  |  |  |  |  |  |  |  |  |  |  |  |  |  |  |  |  |  |  |  |  |  |  |  |  |  |  |  |  |  |  |  |  |  |  |  |  |  |  |  |  |  |  |  |  |  |  |  |  |  |  |  |  |  |  |  |  |  |  |  |  |  |  |  |  |  |  |  |  |  |  |  |  |  |  |  |  |  |  |  |  |  |  |  |  |  |  |  |  |  |  |  |  |  |  |  |  |  |  |  |  |  |  |  |  |  |  |  |  |  |  |  |  |  |  |  |  |  |  |  |  |  |  |  |  |  |  |  |  |  |  |  |  |  |  |  |  |  |  |  |  |  |  |  |  |  |  |  |  |  |  |  |  |  |  |  |  |  |  |  |  |  |  |  |  |  |  |  |  |  |  |  |  |  |  |  |  |  |  |  |  |  |  |  |  |  |  |  |  |  |  |  |  |  |  |  |  |  |  |  |  |  |  |  |  |  |  |  |  |  |  |  |  |  |  |  |  |  |  |  |  |  |  |  |  |  |  |  |  |  |  |  |  |  |  |  |  |  |  |  |  |  |  |  |  |  |  |  |  |  |  |  |  |  |  |  |  |  |  |  |  |  |  |  |  |  |  |  |  |  |  |  |  |  |  |  |  |  |  |  |  |  |  |  |  |  |  |  |  |  |  |  |  |  |  |  |  |  |  |  |  |  |  |  |  |  |  |  |  |  |  |  |  |  |  |  |  |  |  |  |  |  |  |  |  |  |  |  |  |  |  |  |  |  |  |  |  |  |  |  |  |  |  |  |  |  |  |  |  |  |  |  |  |  |  |  |  |  |  |  |  |  |  |  |  |  |  |  |  |  |  |  |  |  |  |  |  |  |  |  |  |  |  |  |  |  |  |  |  |  |  |  |  |  |  |  |  |  |  |  |  |  |  |  |  |  |  |  |  |  |  |  |  |  |  |  |  |  |  |  |  |  |  |  |  |  |  |  |  |  |  |  |  |  |  |  |  |  |  |  |  |  |  |  |  |  |  |  |  |  |  |  |  |  |  |  |  |  |  |  |  |  |  |  |  |  |  |  |  |  |  |  |  |  |  |  |  |  |  |  |  |  |  |  |  |  |  |  |  |  |  |  |  |  |  |  |  |  |  |  |  |  |  |  |  |  |  |  |  |  |  |  |  |  |  |  |  |  |  |  |  |  |  |  |  |  |  |  |  |  |  |  |  |  |  |  |  |  |  |  |  |  |  |  |  |  |  |  |  |  |  |  |  |  |  |  |  |  |  |  |  |  |  |  |  |  |  |  |  |  |  |  |  |  |  |  |  |  |  |  |  |  |  |  |  |  |  |  |  |  |  |  |  |  |  |  |  |  |  |  |  |  |  |  |  |  |  |  |  |  |  |  |  |  |  |  |  |  |  |  |  |  |  |  |  |  |  |  |  |  |  |  |  |  |  |  |  |  |  |  |  |  |  |  |  |  |  |  |  |  |  |  |  |  |  |  |  |  |  |  |  |  |  |  |  |  |  |  |  |  |  |  |  |  |  |  |  |  |  |  |  |  |  |  |  |  |  |  |  |  |  |  |  |  |  |  |  |  |  |  |  |  |  |  |  |  |  |  |  |  |  |  |  |  |  |  |  |  |  |  |  |  |  |  |  |  |  |  |  |  |  |  |  |  |  |  |  |  |  |  |  |  |  |  |  |  |  |  |  |  |  |  |  |  |  |  |  |  |  |  |  |  |  |  |  |  |  |  |  |  |  |  |  |  |  |  |  |  |  |  |  |  |  |  |  |  |  |  |  |  |  |  |  |  |  |  |  |  |  |  |  |  |  |  |  |  |  |  |  |  |  |  |  |  |  |  |  |  |  |  |  |  |  |  |  |  |  |  |  |  |  |  |  |  |  |  |  |  |  |  |  |  |  |  |  |  |  |  |  |  |  |  |  |  |  |  |  |  |  |  |  |  |  |  |  |  |  |  |  |  |  |  |  |  |  |  |  |  |  |  |  |  |  |  |  |  |  |  |  |  |  |  |  |  |  |  |  |  |  |  |  |  |  |  |  |  |  |  |  |  |  |  |  |  |  |  |  |  |  |  |  |  |  |  |  |  |  |  |  |  |  |  |  |  |  |  |  |  |  |  |  |  |  |  |  |  |  |  |  |  |  |  |  |  |  |  |  |  |  |  |  |  |  |  |  |  |  |  |  |  |  |  |  |  |  |  |  |  |  |  |  |  |  |  |  |  |  |  |  |  |  |  |  |  |  |  |  |  |  |  |  |  |  |  |  |  |  |  |  |  |  |  |  |  |  |  |  |  |  |  |  |  |  |  |  |  |  |  |  |  |  |  |  |  |  |  |  |  |  |  |  |  |  |  |  |  |  |  |  |  |  |  |  |  |  |  |  |  |  |  |  |  |  |  |  |  |  |  |  |  |  |  |  |  |  |  |  |  |  |  |  |  |  |  |  |  |  |  |  |  |  |  |  |  |  |  |  |  |  |  |  |  |  |  |  |  |  |  |  |  |  |  |  |  |  |  |  |  |  |  |  |  |  |  |  |  |  |  |  |  |  |  |  |  |  |  |  |  |  |  |  |  |  |  |  |  |  |  |  |  |  |  |  |  |  |  |  |  |  |  |  |  |  |  |  |  |  |  |  |  |  |  |  |  |  |  |  |  |  |  |  |  |  |  |  |  |  |  |  |  |  |  |  |  |  |  |  |  |  |  |  |  |  |  |  |  |  |  |  |  |  |  |  |  |  |  |  |  |  |  |  |  |  |  |  |  |  |  |  |  |  |  |  |  |  |  |  |  |  |  |  |  |  |  |  |  |  |  |  |  |  |  |  |  |  |  |  |  |  |  |  |  |  |  |  |  |  |  |  |  |  |  |  |  |  |  |  |  |  |  |  |  |  |  |  |  |  |  |  |  |  |  |  |  |  |  |  |  |  |  |  |  |  |  |  |  |  |  |  |  |  |  |  |  |  |  |  |  |  |  |  |  |  |  |  |  |  |  |  |  |  |  |  |  |  |  |  |  |  |  |  |  |  |  |  |  |  |  |  |  |  |  |  |  |  |  |  |  |  |  |  |  |  |  |  |  |  |  |  |  |  |  |  |  |  |  |  |  |  |  |  |  |  |  |  |  |  |  |  |  |  |  |  |  |  |  |  |  |  |  |  |  |  |  |  |  |  |  |  |  |  |  |  |  |  |  |  |  |  |  |  |  |  |  |  |  |  |  |  |  |  |  |  |  |  |  |  |  |  |  |  |  |  |  |  |  |  |  |  |  |  |  |  |  |  |  |  |  |  |  |  |  |  |  |  |  |  |  |  |  |  |  |  |  |  |  |  |  |  |  |  |  |  |  |  |  |  |  |  |  |  |  |  |  |  |  |  |  |  |  |  |  |  |  |  |  |  |  |  |  |  |  |  |  |  |  |  |  |  |  |  |  |  |  |  |  |  |  |  |  |  |  |  |  |  |  |  |  |  |  |  |  |  |  |  |  |  |  |  |  |  |  |  |  |  |  |  |  |  |  |  |  |  |  |  |  |  |  |  |  |  |  |  |  |  |  |  |  |  |  |  |  |  |  |  |  |  |  |  |  |  |  |  |  |  |  |  |  |  |  |  |  |  |  |  |  |  |  |  |  |  |  |  |  |  |  |  |  |  |  |  |  |  |  |  |  |  |  |  |  |  |  |  |  |  |  |  |  |  |  |  |  |  |  |  |  |  |  |  |  |  |  |  |  |  |  |  |  |  |  |  |  |  |  |  |  |  |  |  |  |  |  |  |  |  |  |  |  |  |  |  |  |  |  |  |  |  |  |  |  |  |  |  |  |  |  |  |  |  |  |  |  |  |  |  |  |  |  |  |  |  |  |  |  |  |  |  |  |  |  |  |  |  |  |  |  |  |  |  |  |  |  |  |  |  |  |  |  |  |  |  |  |  |  |  |  |  |  |  |  |  |  |  |  |  |  |  |  |  |  |  |  |  |  |  |  |  |  |  |  |  |  |  |  |  |  |  |  |  |  |  |  |  |  |  |  |  |  |  |  |  |  |  |  |  |  |  |  |  |  |  |  |  |  |  |  |  |  |  |  |  |  |  |  |  |  |  |  |  |  |  |  |  |  |  |  |  |  |  |  |  |  |  |  |  |  |  |  |  |  |  |  |  |  |  |  |  |  |  |  |  |  |  |  |  |  |  |  |  |  |  |  |  |  |  |  |  |  |  |  |  |  |  |  |  |  |  |  |  |  |  |  |  |  |  |  |  |  |  |  |  |  |  |  |  |  |  |  |  |  |  |  |  |  |  |  |  |  |  |  |  |  |  |  |  |  |  |  |  |  |  |  |  |  |  |  |  |  |  |  |  |  |  |  |  |  |  |  |  |  |  |  |  |  |  |  |  |  |  |  |  |  |  |  |  |  |  |  |  |  |  |  |  |  |  |  |  |  |  |  |  |  |  |  |  |  |  |  |  |  |  |  |  |  |  |  |  |  |  |  |  |  |  |  |  |  |  |  |  |  |  |  |  |  |  |  |  |  |  |  |  |  |  |  |  |  |  |  |  |  |  |  |  |  |  |  |  |  |  |  |  |  |  |  |  |  |  |  |  |  |  |  |  |  |  |  |  |  |  |  |  |  |  |  |  |  |  |  |  |  |  |  |  |  |  |  |  |  |  |  |  |  |  |  |  |  |  |  |  |  |  |  |  |  |  |  |  |  |  |  |  |  |  |  |  |  |  |  |  |  |  |  |  |  |  |  |  |  |  |  |  |  |  |  |  |  |  |  |  |  |  |  |  |  |  |  |  |  |  |  |  |  |  |  |  |  |  |  |  |  |  |  |  |  |  |  |  |  |  |  |  |  |  |  |  |  |  |  |  |  |  |  |  |  |  |  |  |  |  |  |  |  |  |  |  |  |  |  |  |  |  |  |  |  |  |  |  |  |  |  |  |  |  |  |  |  |  |  |  |  |  |  |  |  |  |  |  |  |  |  |  |  |  |  |  |  |  |  |  |  |  |  |  |  |  |  |  |  |  |  |  |  |  |  |  |  |  |  |  |  |  |  |  |  |  |  |  |  |  |  |  |  |  |  |  |  |  |  |  |  |  |  |  |  |  |  |  |  |  |  |  |  |  |  |  |  |  |  |  |  |  |  |  |  |  |  |  |  |  |  |  |  |  |  |  |  |  |  |  |  |  |  |  |  |  |  |  |  |  |  |  |  |  |  |  |  |  |  |  |  |  |  |  |  |  |  |  |  |  |  |  |  |  |  |  |  |  |  |  |  |  |  |  |  |  |  |  |  |  |  |  |  |  |  |  |  |  |  |  |  |  |  |  |  |  |  |  |  |  |  |  |  |  |  |  |  |  |  |  |  |  |  |  |  |  |  |  |  |  |  |  |  |  |  |  |  |  |  |  |  |  |  |  |  |  |  |  |  |  |  |  |  |  |  |  |  |  |  |  |  |  |  |  |  |  |  |  |  |  |  |  |  |  |  |  |  |  |  |  |  |  |  |  |  |  |  |  |  |  |  |  |  |  |  |  |  |  |  |  |  |  |  |  |  |  |  |  |  |  |  |  |  |  |  |  |  |  |  |  |  |  |  |  |  |  |  |  |  |  |  |  |  |  |  |  |  |  |  |  |  |  |  |  |  |  |  |  |  |  |  |  |  |  |  |  |  |  |  |  |  |  |  |  |  |  |  |  |  |  |  |  |  |  |  |  |  |  |  |  |  |  |  |  |  |  |  |  |  |  |  |  |  |  |  |  |  |  |  |  |  |  |  |  |  |  |  |  |  |  |  |  |  |  |  |  |  |  |  |  |  |  |  |  |  |  |  |  |  |  |  |  |  |  |  |  |  |  |  |  |  |  |  |  |  |  |  |  |  |  |  |  |  |  |  |  |  |  |  |  |  |  |  |  |  |  |  |  |  |  |  |  |  |  |  |  |  |  |  |  |  |  |  |  |  |  |  |  |  |  |  |  |  |  |  |  |  |  |  |  |  |  |  |  |  |  |  |  |  |  |  |  |  |  |  |  |  |  |  |  |  |  |  |  |  |  |  |  |  |  |  |  |  |  |  |  |  |  |  |  |  |  |  |  |  |  |  |  |  |  |  |  |  |  |  |  |  |  |  |  |  |  |  |  |  |  |  |  |  |  |  |  |  |  |  |  |  |  |  |  |  |  |  |  |  |  |  |  |  |  |  |  |  |  |  |  |  |  |  |  |  |  |  |  |  |  |  |  |  |  |  |  |  |  |  |  |  |  |  |  |  |  |  |  |  |  |  |  |  |  |  |  |  |  |  |  |  |  |  |  |  |  |  |  |  |  |  |  |  |  |  |  |  |  |  |  |  |  |  |  |  |  |  |  |  |  |  |  |  |  |  |  |  |  |  |  |  |  |  |  |  |  |  |  |  |  |  |  |  |  |  |  |  |  |  |  |  |  |  |  |  |  |  |  |  |  |  |  |  |  |  |  |  |  |  |  |  |  |  |  |  |  |  |  |  |  |  |  |  |  |  |  |  |  |  |  |  |  |  |  |  |  |  |  |  |  |  |  |  |  |  |  |  |  |  |  |  |  |  |  |  |  |  |  |  |  |  |  |  |  |  |  |  |  |  |  |  |  |  |  |  |  |  |  |  |  |  |  |  |  |  |  |  |  |  |  |  |  |  |  |  |  |  |  |  |  |  |  |  |  |  |  |  |  |  |  |  |  |  |  |  |  |  |  |  |  |  |  |  |  |  |  |  |  |  |  |  |  |  |  |  |  |  |  |  |  |  |  |  |  |  |  |  |  |  |  |  |  |  |  |  |  |  |  |  |  |  |  |  |  |  |  |  |  |  |  |  |  |  |  |  |  |  |  |  |  |  |  |  |  |  |  |  |  |  |  |  |  |  |  |  |  |  |  |  |  |  |  |  |  |  |  |  |  |  |  |  |  |  |  |  |  |  |  |  |  |  |  |  |  |  |  |  |  |  |  |  |  |  |  |  |  |  |  |  |  |  |  |  |  |  |  |  |  |  |  |  |  |  |  |  |  |  |  |  |  |  |  |  |  |  |  |  |  |  |  |  |  |  |  |  |  |  |  |  |  |  |  |  |  |  |  |  |  |  |  |  |  |  |  |  |  |  |  |  |  |  |  |  |  |  |  |  |  |  |  |  |  |  |  |  |  |  |  |  |  |  |  |  |  |  |  |  |  |  |  |  |  |  |  |  |  |  |  |  |  |  |  |  |  |  |  |  |  |  |  |  |  |  |  |  |  |  |  |  |  |  |  |  |  |  |  |  |  |  |  |  |  |  |  |  |  |  |  |  |  |  |  |  |  |  |  |  |  |  |  |  |  |  |  |  |  |  |  |  |  |  |  |  |  |  |  |  |  |  |  |  |  |  |  |  |  |  |  |  |  |  |  |  |  |  |  |  |  |  |  |  |  |  |  |  |  |  |  |  |  |  |  |  |  |  |  |  |  |  |  |  |  |  |  |  |  |  |  |  |  |  |  |  |  |  |  |  |  |  |  |  |  |  |  |  |  |  |  |  |  |  |  |  |  |  |  |  |  |  |  |  |  |  |  |  |  |  |  |  |  |  |  |  |  |  |  |  |  |  |  |  |  |  |  |  |  |  |  |  |  |  |  |  |  |  |  |  |  |  |  |  |  |  |  |  |  |  |  |  |  |  |  |  |  |  |  |  |  |  |  |  |  |  |  |  |  |  |  |  |  |  |  |  |  |  |  |  |  |  |  |  |  |  |  |  |  |  |  |  |  |  |  |  |  |  |  |  |  |  |  |  |  |  |  |  |  |  |  |  |  |  |  |  |  |  |  |  |  |  |  |  |  |  |  |  |  |  |  |  |  |  |  |  |  |  |  |  |  |  |  |  |  |  |  |  |  |  |  |  |  |  |  |  |  |  |  |  |  |  |  |  |  |  |  |  |  |  |  |  |  |  |  |  |  |  |  |  |  |  |  |  |  |  |  |  |  |  |  |  |  |  |  |  |  |  |  |  |  |  |  |  |  |  |  |  |  |  |  |  |  |  |  |  |  |  |  |  |  |  |  |  |  |  |  |  |  |  |  |  |  |  |  |  |  |  |  |  |  |  |  |  |  |  |  |  |  |  |  |  |  |  |  |  |  |  |  |  |  |  |  |  |  |  |  |  |  |  |  |  |  |  |  |  |  |  |  |  |  |  |  |  |  |  |  |  |  |  |  |  |  |  |  |  |  |  |  |  |  |  |  |  |  |  |  |  |  |  |  |  |  |  |  |  |  |  |  |  |  |  |  |  |  |  |  |  |  |  |  |  |  |  |  |  |  |  |  |  |  |  |  |  |  |  |  |  |  |  |  |  |  |  |  |  |  |  |  |  |  |  |  |  |  |  |  |  |  |  |  |  |  |  |  |  |  |  |  |  |  |  |  |  |  |  |  |  |  |  |  |  |  |  |  |  |  |  |  |  |  |  |  |  |  |  |  |  |  |  |  |  |  |  |  |  |  |  |  |  |  |  |  |  |  |  |  |  |  |  |  |  |  |  |  |  |  |  |  |  |  |  |  |  |  |  |  |  |  |  |  |  |  |  |  |  |  |  |  |  |  |  |  |  |  |  |  |  |  |  |  |  |  |  |  |  |  |  |  |  |  |  |  |  |  |  |  |  |  |  |  |  |  |  |  |  |  |  |  |  |  |  |  |  |  |  |  |  |  |  |  |  |  |  |  |  |  |  |  |  |  |  |  |  |  |  |  |  |  |  |  |  |  |  |  |  |  |  |  |  |  |  |  |  |  |  |  |  |  |  |  |  |  |  |  |  |  |  |  |  |  |  |  |  |  |  |  |  |  |  |  |  |  |  |  |  |  |  |  |  |  |  |  |  |  |  |  |  |  |  |  |  |  |  |  |  |  |  |  |  |  |  |  |  |  |  |  |  |  |  |  |  |  |  |  |  |  |  |  |  |  |  |  |  |  |  |  |  |  |  |  |  |  |  |  |  |  |  |  |  |  |  |  |  |  |  |  |  |  |  |  |  |  |  |  |  |  |  |  |  |  |  |  |  |  |  |  |  |  |  |  |  |  |  |  |  |  |  |  |  |  |  |  |  |  |  |  |  |  |  |  |  |  |  |  |  |  |  |  |  |  |  |  |  |  |  |  |  |  |  |  |  |  |  |  |  |  |  |  |  |  |  |  |  |  |  |  |  |  |  |  |  |  |  |  |  |  |  |  |  |  |  |  |  |  |  |  |  |  |  |  |  |  |  |  |  |  |  |  |  |  |  |  |  |  |  |  |  |  |  |  |  |  |  |  |  |  |  |  |  |  |  |  |  |  |  |  |  |  |  |  |  |  |  |  |  |  |  |  |  |  |  |  |  |  |  |  |  |  |  |  |  |  |  |  |  |  |  |  |  |  |  |  |  |  |  |  |  |  |  |  |  |  |  |  |  |  |  |  |  |  |  |  |  |  |  |  |  |  |  |  |  |  |  |  |  |  |  |  |  |  |  |  |  |  |  |  |  |  |  |  |  |  |  |  |  |  |  |  |  |  |  |  |  |  |  |  |  |  |  |  |  |  |  |  |  |  |  |  |  |  |  |  |  |  |  |  |  |  |  |  |  |  |  |  |  |  |  |  |  |  |  |  |  |  |  |  |  |  |  |  |  |  |  |  |  |  |  |  |  |  |  |  |  |  |  |  |  |  |  |  |  |  |  |  |  |  |  |  |  |  |  |  |  |  |  |  |  |  |  |  |  |  |  |  |  |  |  |  |  |  |  |  |  |  |  |  |  |  |  |  |  |  |  |  |  |  |  |  |  |  |  |  |  |  |  |  |  |  |  |  |  |  |  |  |  |  |  |  |  |  |  |  |  |  |  |  |  |  |  |  |  |  |  |  |  |  |  |  |  |  |  |  |  |  |  |  |  |  |  |  |  |  |  |  |  |  |  |  |  |  |  |  |  |  |  |  |  |  |  |  |  |  |  |  |  |  |  |  |  |  |  |  |  |  |  |  |  |  |  |  |  |  |  |  |  |  |  |  |  |  |  |  |  |  |  |  |  |  |  |  |  |  |  |  |  |  |  |  |  |  |  |  |  |  |  |  |  |  |  |  |  |  |  |  |  |  |  |  |  |  |  |  |  |  |  |  |  |  |  |  |  |  |  |  |  |  |  |  |  |  |  |  |  |  |  |  |  |  |  |  |  |  |  |  |  |  |  |  |  |  |  |  |  |  |  |  |  |  |  |  |  |  |  |  |  |  |  |  |  |  |  |  |  |  |  |  |  |  |  |  |  |  |  |  |  |  |  |  |  |  |  |  |  |  |  |  |  |  |  |  |  |  |  |  |  |  |  |  |  |  |  |  |  |  |  |  |  |  |  |  |  |  |  |  |  |  |  |  |  |  |  |  |  |  |  |  |  |  |  |  |  |  |  |  |  |  |  |  |  |  |  |  |  |  |  |  |  |  |  |  |  |  |  |  |  |  |  |  |  |  |  |  |  |  |  |  |  |  |  |  |  |  |  |  |  |  |  |  |  |  |  |  |  |  |  |  |  |  |  |  |  |  |  |  |  |  |  |  |  |  |  |  |  |  |  |  |  |  |  |  |  |  |  |  |  |  |  |  |  |  |  |  |  |  |  |  |  |  |  |  |  |  |  |  |  |  |  |  |  |  |  |  |  |  |  |  |  |  |  |  |  |  |  |  |  |  |  |  |  |  |  |  |  |  |  |  |  |  |  |  |  |  |  |  |  |  |  |  |  |  |  |  |  |  |  |  |  |  |  |  |  |  |  |  |  |  |  |  |  |  |  |  |  |  |  |  |  |  |  |  |  |  |  |  |  |  |  |  |  |  |  |  |  |  |  |  |  |  |  |  |  |  |  |  |  |  |  |  |  |  |  |  |  |  |  |  |  |  |  |  |  |  |  |  |  |  |  |  |  |  |  |  |  |  |  |  |  |  |  |  |  |  |  |  |  |  |  |  |  |  |  |  |  |  |  |  |  |  |  |  |  |  |  |  |  |  |  |  |  |  |  |  |  |  |  |  |  |  |  |  |  |  |  |  |  |  |  |  |  |  |  |  |  |  |  |  |  |  |  |  |  |  |  |  |  |  |  |  |  |  |  |  |  |  |  |  |  |  |  |  |  |  |  |  |  |  |  |  |  |  |  |  |  |  |  |  |  |  |  |  |  |  |  |  |  |  |  |  |  |  |  |  |  |  |  |  |  |  |  |  |  |  |  |  |  |  |  |  |  |  |  |  |  |  |  |  |  |  |  |  |  |  |  |  |  |  |  |  |  |  |  |  |  |  |  |  |  |  |  |  |  |  |  |  |  |  |  |  |  |  |  |  |  |  |  |  |  |  |  |  |  |  |  |  |  |  |  |  |  |  |  |  |  |  |  |  |  |  |  |  |  |  |  |  |  |  |  |  |  |  |  |  |  |  |  |  |  |  |  |  |  |  |  |  |  |  |  |  |  |  |  |  |  |  |  |  |  |  |  |  |  |  |  |  |  |  |  |  |  |  |  |  |  |  |  |  |  |  |  |  |  |  |  |  |  |  |  |  |  |  |  |  |  |  |  |  |  |  |  |  |  |  |  |  |  |  |  |  |  |  |  |  |  |  |  |  |  |  |  |  |  |  |  |  |  |  |  |  |  |  |  |  |  |  |  |  |  |  |  |  |  |  |  |  |  |  |  |  |  |  |  |  |  |  |  |  |  |  |  |  |  |  |  |  |  |  |  |  |  |  |  |  |  |  |  |  |  |  |  |  |  |  |  |  |  |  |  |  |  |  |  |  |  |  |  |  |  |  |  |  |  |  |  |  |  |  |  |  |  |  |  |  |  |  |  |  |  |  |  |  |  |  |  |  |  |  |  |  |  |  |  |  |  |  |  |  |  |  |  |  |  |  |  |  |  |  |  |  |  |  |  |  |  |  |  |  |  |  |  |  |  |  |  |  |  |  |  |  |  |  |  |  |  |  |  |  |  |  |  |  |  |  |  |  |  |  |  |  |  |  |  |  |  |  |  |  |  |  |  |  |  |  |  |  |  |  |  |  |  |  |  |  |  |  |  |  |  |  |  |  |  |  |  |  |  |  |  |  |  |  |  |  |  |  |  |  |  |  |  |  |  |  |  |  |  |  |  |  |  |  |  |  |  |  |  |  |  |  |  |  |  |  |  |  |  |  |  |  |  |  |  |  |  |  |  |  |  |  |  |  |  |  |  |  |  |  |  |  |  |  |  |  |  |  |  |  |  |  |  |  |  |  |  |  |  |  |  |  |  |  |  |  |  |  |  |  |  |  |  |  |  |  |  |  |  |  |  |  |  |  |  |  |  |  |  |  |  |  |  |  |  |  |  |  |  |  |  |  |  |  |  |  |  |  |  |  |  |  |  |  |  |  |  |  |  |  |  |  |  |  |  |  |  |  |  |  |  |  |  |  |  |  |  |  |  |  |  |  |  |  |  |  |  |  |  |  |  |  |  |  |  |  |  |  |  |  |  |  |  |  |  |  |  |  |  |  |  |  |  |  |  |  |  |  |  |  |  |  |  |  |  |  |  |  |  |  |  |  |  |  |  |  |  |  |  |  |  |  |  |  |  |  |  |  |  |  |  |  |  |  |  |  |  |  |  |  |  |  |  |  |  |  |  |  |  |  |  |  |  |  |  |  |  |  |  |  |  |  |  |  |  |  |  |  |  |  |  |  |  |  |  |  |  |  |  |  |  |  |  |  |  |  |  |  |  |  |  |  |  |  |  |  |  |  |  |  |  |  |  |  |  |  |  |  |  |  |  |  |  |  |  |  |  |  |  |  |  |  |  |  |  |  |  |  |  |  |  |  |  |  |  |  |  |  |  |  |  |  |  |  |  |  |  |  |  |  |  |  |  |  |  |  |  |  |  |  |  |  |  |  |  |  |  |  |  |  |  |  |  |  |  |  |  |  |  |  |  |  |  |  |  |  |  |  |  |  |  |  |  |  |  |  |  |  |  |  |  |  |  |  |  |  |  |  |  |  |  |  |  |  |  |  |  |  |  |  |  |  |  |  |  |  |  |  |  |  |  |  |  |  |  |  |  |  |  |  |  |  |  |  |  |  |  |  |  |  |  |  |  |  |  |  |  |  |  |  |  |  |  |  |  |  |  |  |  |  |  |  |  |  |  |  |  |  |  |  |  |  |  |  |  |  |  |  |  |  |  |  |  |  |  |  |  |  |  |  |  |  |  |  |  |  |  |  |  |  |  |  |  |  |  |  |  |  |  |  |  |  |  |  |  |  |  |  |  |  |  |  |  |  |  |  |  |  |  |  |  |  |  |  |  |  |  |  |  |  |  |  |  |  |  |  |  |  |  |  |  |  |  |  |  |  |  |  |  |  |  |  |  |  |  |  |  |  |  |  |  |  |  |  |  |  |  |  |  |  |  |  |  |  |  |  |  |  |  |  |  |  |  |  |  |  |  |  |  |  |  |  |  |  |  |  |  |  |  |  |  |  |  |  |  |  |  |  |  |  |  |  |  |  |  |  |  |  |  |  |  |  |  |  |  |  |  |  |  |  |  |  |  |  |  |  |  |  |  |  |  |  |  |  |  |  |  |  |  |  |  |  |  |  |  |  |  |  |  |  |  |  |  |  |  |  |  |  |  |  |  |  |  |  |  |  |  |  |  |  |  |  |  |  |  |  |  |  |  |  |  |  |  |  |  |  |  |  |  |  |  |  |  |  |  |  |  |  |  |  |  |  |  |  |  |  |  |  |  |  |  |  |  |  |  |  |  |  |  |  |  |  |  |  |  |  |  |  |  |  |  |  |  |  |  |  |  |  |  |  |  |  |  |  |  |  |  |  |  |  |  |  |  |  |  |  |  |  |  |  |  |  |  |  |  |  |  |  |  |  |  |  |  |  |  |  |  |  |  |  |  |  |  |  |  |  |  |  |  |  |  |  |  |  |  |  |  |  |  |  |  |  |  |  |  |  |  |  |  |  |  |  |  |  |  |  |  |  |  |  |  |  |  |  |  |  |  |  |  |  |  |  |  |  |  |  |  |  |  |  |  |  |  |  |  |  |  |  |  |  |  |  |  |  |  |  |  |  |  |  |  |  |  |  |  |  |  |  |  |  |  |  |  |  |  |  |  |  |  |  |  |  |  |  |  |  |  |  |  |  |  |  |  |  |  |  |  |  |  |  |  |  |  |  |  |  |  |  |  |  |  |  |  |  |  |  |  |  |  |  |  |  |  |  |  |  |  |  |  |  |  |  |  |  |  |  |  |  |  |  |  |  |  |  |  |  |  |  |  |  |  |  |  |  |  |  |  |  |  |  |  |  |  |  |  |  |  |  |  |  |  |  |  |  |  |  |  |  |  |  |  |  |  |  |  |  |  |  |  |  |  |  |  |  |  |  |  |  |  |  |  |  |  |  |  |  |  |  |  |  |  |  |  |  |  |  |  |  |  |  |  |  |  |  |  |  |  |  |  |  |  |  |  |  |  |  |  |  |  |  |  |  |  |  |  |  |  |  |  |  |  |  |  |  |  |  |  |  |  |  |  |  |  |  |  |  |  |  |  |  |  |  |  |  |  |  |  |  |  |  |  |  |  |  |  |  |  |  |  |  |  |  |  |  |  |  |  |  |  |  |  |  |  |  |  |  |  |  |  |  |  |  |  |  |  |  |  |  |  |  |  |  |  |  |  |  |  |  |  |  |  |  |  |  |  |  |  |  |  |  |  |  |  |  |  |  |  |  |  |  |  |  |  |  |  |  |  |  |  |  |  |  |  |  |  |  |  |  |  |  |  |  |  |  |  |  |  |  |  |  |  |  |  |  |  |  |  |  |  |  |  |  |  |  |  |  |  |  |  |  |  |  |  |  |  |  |  |  |  |  |  |  |  |  |  |  |  |  |  |  |  |  |  |  |  |  |  |  |  |  |  |  |  |  |  |  |  |  |  |  |  |  |  |  |  |  |  |  |  |  |  |  |  |  |  |  |  |  |  |  |  |  |  |  |  |  |  |  |  |  |  |  |  |  |  |  |  |  |  |  |  |  |  |  |  |  |  |  |  |  |  |  |  |  |  |  |  |  |  |  |  |  |  |  |  |  |  |  |  |  |  |  |  |  |  |  |  |  |  |  |  |  |  |  |  |  |  |  |  |  |  |  |  |  |  |  |  |  |  |  |  |  |  |  |  |  |  |  |  |  |  |  |  |  |  |  |  |  |  |  |  |  |  |  |  |  |  |  |  |  |  |  |  |  |  |  |  |  |  |  |  |  |  |  |  |  |  |  |  |  |  |  |  |  |  |  |  |  |  |  |  |  |  |  |  |  |  |  |  |  |  |  |  |  |  |  |  |  |  |  |  |  |  |  |  |  |  |  |  |  |  |  |  |  |  |  |  |  |  |  |  |  |  |  |  |  |  |  |  |  |  |  |  |  |  |  |  |  |  |  |  |  |  |  |  |  |  |  |  |  |  |  |  |  |  |  |  |  |  |  |  |  |  |  |  |  |  |  |  |  |  |  |  |  |  |  |  |  |  |  |  |  |  |  |  |  |  |  |  |  |  |  |  |  |  |  |  |  |  |  |  |  |  |  |  |  |  |  |  |  |  |  |  |  |  |  |  |  |  |  |  |  |  |  |  |  |  |  |  |  |  |  |  |  |  |  |  |  |  |  |  |  |  |  |  |  |  |  |  |  |  |  |  |  |  |  |  |  |  |  |  |  |  |  |  |  |  |  |  |  |  |  |  |  |  |  |  |  |  |  |  |  |  |  |  |  |  |  |  |  |  |  |  |  |  |  |  |  |  |  |  |  |  |  |  |  |  |  |  |  |  |  |  |  |  |  |  |  |  |  |  |  |  |  |  |  |  |  |  |  |  |  |  |  |  |  |  |  |  |  |  |  |  |  |  |  |  |  |  |  |  |  |  |  |  |  |  |  |  |  |  |  |  |  |  |  |  |  |  |  |  |  |  |  |  |  |  |  |  |  |  |  |  |  |  |  |  |  |  |  |  |  |  |  |  |  |  |  |  |  |  |  |  |  |  |  |  |  |  |  |  |  |  |  |  |  |  |  |  |  |  |  |  |  |  |  |  |  |  |
| --- | --- | --- | --- | --- | --- | --- | --- | --- | --- | --- | --- | --- | --- | --- | --- | --- | --- | --- | --- | --- | --- | --- | --- | --- | --- | --- | --- | --- | --- | --- | --- | --- | --- | --- | --- | --- | --- | --- | --- | --- | --- | --- | --- | --- | --- | --- | --- | --- | --- | --- | --- | --- | --- | --- | --- | --- | --- | --- | --- | --- | --- | --- | --- | --- | --- | --- | --- | --- | --- | --- | --- | --- | --- | --- | --- | --- | --- | --- | --- | --- | --- | --- | --- | --- | --- | --- | --- | --- | --- | --- | --- | --- | --- | --- | --- | --- | --- | --- | --- | --- | --- | --- | --- | --- | --- | --- | --- | --- | --- | --- | --- | --- | --- | --- | --- | --- | --- | --- | --- | --- | --- | --- | --- | --- | --- | --- | --- | --- | --- | --- | --- | --- | --- | --- | --- | --- | --- | --- | --- | --- | --- | --- | --- | --- | --- | --- | --- | --- | --- | --- | --- | --- | --- | --- | --- | --- | --- | --- | --- | --- | --- | --- | --- | --- | --- | --- | --- | --- | --- | --- | --- | --- | --- | --- | --- | --- | --- | --- | --- | --- | --- | --- | --- | --- | --- | --- | --- | --- | --- | --- | --- | --- | --- | --- | --- | --- | --- | --- | --- | --- | --- | --- | --- | --- | --- | --- | --- | --- | --- | --- | --- | --- | --- | --- | --- | --- | --- | --- | --- | --- | --- | --- | --- | --- | --- | --- | --- | --- | --- | --- | --- | --- | --- | --- | --- | --- | --- | --- | --- | --- | --- | --- | --- | --- | --- | --- | --- | --- | --- | --- | --- | --- | --- | --- | --- | --- | --- | --- | --- | --- | --- | --- | --- | --- | --- | --- | --- | --- | --- | --- | --- | --- | --- | --- | --- | --- | --- | --- | --- | --- | --- | --- | --- | --- | --- | --- | --- | --- | --- | --- | --- | --- | --- | --- | --- | --- | --- | --- | --- | --- | --- | --- | --- | --- | --- | --- | --- | --- | --- | --- | --- | --- | --- | --- | --- | --- | --- | --- | --- | --- | --- | --- | --- | --- | --- | --- | --- | --- | --- | --- | --- | --- | --- | --- | --- | --- | --- | --- | --- | --- | --- | --- | --- | --- | --- | --- | --- | --- | --- | --- | --- | --- | --- | --- | --- | --- | --- | --- | --- | --- | --- | --- | --- | --- | --- | --- | --- | --- | --- | --- | --- | --- | --- | --- | --- | --- | --- | --- | --- | --- | --- | --- | --- | --- | --- | --- | --- | --- | --- | --- | --- | --- | --- | --- | --- | --- | --- | --- | --- | --- | --- | --- | --- | --- | --- | --- | --- | --- | --- | --- | --- | --- | --- | --- | --- | --- | --- | --- | --- | --- | --- | --- | --- | --- | --- | --- | --- | --- | --- | --- | --- | --- | --- | --- | --- | --- | --- | --- | --- | --- | --- | --- | --- | --- | --- | --- | --- | --- | --- | --- | --- | --- | --- | --- | --- | --- | --- | --- | --- | --- | --- | --- | --- | --- | --- | --- | --- | --- | --- | --- | --- | --- | --- | --- | --- | --- | --- | --- | --- | --- | --- | --- | --- | --- | --- | --- | --- | --- | --- | --- | --- | --- | --- | --- | --- | --- | --- | --- | --- | --- | --- | --- | --- | --- | --- | --- | --- | --- | --- | --- | --- | --- | --- | --- | --- | --- | --- | --- | --- | --- | --- | --- | --- | --- | --- | --- | --- | --- | --- | --- | --- | --- | --- | --- | --- | --- | --- | --- | --- | --- | --- | --- | --- | --- | --- | --- | --- | --- | --- | --- | --- | --- | --- | --- | --- | --- | --- | --- | --- | --- | --- | --- | --- | --- | --- | --- | --- | --- | --- | --- | --- | --- | --- | --- | --- | --- | --- | --- | --- | --- | --- | --- | --- | --- | --- | --- | --- | --- | --- | --- | --- | --- | --- | --- | --- | --- | --- | --- | --- | --- | --- | --- | --- | --- | --- | --- | --- | --- | --- | --- | --- | --- | --- | --- | --- | --- | --- | --- | --- | --- | --- | --- | --- | --- | --- | --- | --- | --- | --- | --- | --- | --- | --- | --- | --- | --- | --- | --- | --- | --- | --- | --- | --- | --- | --- | --- | --- | --- | --- | --- | --- | --- | --- | --- | --- | --- | --- | --- | --- | --- | --- | --- | --- | --- | --- | --- | --- | --- | --- | --- | --- | --- | --- | --- | --- | --- | --- | --- | --- | --- | --- | --- | --- | --- | --- | --- | --- | --- | --- | --- | --- | --- | --- | --- | --- | --- | --- | --- | --- | --- | --- | --- | --- | --- | --- | --- | --- | --- | --- | --- | --- | --- | --- | --- | --- | --- | --- | --- | --- | --- | --- | --- | --- | --- | --- | --- | --- | --- | --- | --- | --- | --- | --- | --- | --- | --- | --- | --- | --- | --- | --- | --- | --- | --- | --- | --- | --- | --- | --- | --- | --- | --- | --- | --- | --- | --- | --- | --- | --- | --- | --- | --- | --- | --- | --- | --- | --- | --- | --- | --- | --- | --- | --- | --- | --- | --- | --- | --- | --- | --- | --- | --- | --- | --- | --- | --- | --- | --- | --- | --- | --- | --- | --- | --- | --- | --- | --- | --- | --- | --- | --- | --- | --- | --- | --- | --- | --- | --- | --- | --- | --- | --- | --- | --- | --- | --- | --- | --- | --- | --- | --- | --- | --- | --- | --- | --- | --- | --- | --- | --- | --- | --- | --- | --- | --- | --- | --- | --- | --- | --- | --- | --- | --- | --- | --- | --- | --- | --- | --- | --- | --- | --- | --- | --- | --- | --- | --- | --- | --- | --- | --- | --- | --- | --- | --- | --- | --- | --- | --- | --- | --- | --- | --- | --- | --- | --- | --- | --- | --- | --- | --- | --- | --- | --- | --- | --- | --- | --- | --- | --- | --- | --- | --- | --- | --- | --- | --- | --- | --- | --- | --- | --- | --- | --- | --- | --- | --- | --- | --- | --- | --- | --- | --- | --- | --- | --- | --- | --- | --- | --- | --- | --- | --- | --- | --- | --- | --- | --- | --- | --- | --- | --- | --- | --- | --- | --- | --- | --- | --- | --- | --- | --- | --- | --- | --- | --- | --- | --- | --- | --- | --- | --- | --- | --- | --- | --- | --- | --- | --- | --- | --- | --- | --- | --- | --- | --- | --- | --- | --- | --- | --- | --- | --- | --- | --- | --- | --- | --- | --- | --- | --- | --- | --- | --- | --- | --- | --- | --- | --- | --- | --- | --- | --- | --- | --- | --- | --- | --- | --- | --- | --- | --- | --- | --- | --- | --- | --- | --- | --- | --- | --- | --- | --- | --- | --- | --- | --- | --- | --- | --- | --- | --- | --- | --- | --- | --- | --- | --- | --- | --- | --- | --- | --- | --- | --- | --- | --- | --- | --- | --- | --- | --- | --- | --- | --- | --- | --- | --- | --- | --- | --- | --- | --- | --- | --- | --- | --- | --- | --- | --- | --- | --- | --- | --- | --- | --- | --- | --- | --- | --- | --- | --- | --- | --- | --- | --- | --- | --- | --- | --- | --- | --- | --- | --- | --- | --- | --- | --- | --- | --- | --- | --- | --- | --- | --- | --- | --- | --- | --- | --- | --- | --- | --- | --- | --- | --- | --- | --- | --- | --- | --- | --- | --- | --- | --- | --- | --- | --- | --- | --- | --- | --- | --- | --- | --- | --- | --- | --- | --- | --- | --- | --- | --- | --- | --- | --- | --- | --- | --- | --- | --- | --- | --- | --- | --- | --- | --- | --- | --- | --- | --- | --- | --- | --- | --- | --- | --- | --- | --- | --- | --- | --- | --- | --- | --- | --- | --- | --- | --- | --- | --- | --- | --- | --- | --- | --- | --- | --- | --- | --- | --- | --- | --- | --- | --- | --- | --- | --- | --- | --- | --- | --- | --- | --- | --- | --- | --- | --- | --- | --- | --- | --- | --- | --- | --- | --- | --- | --- | --- | --- | --- | --- | --- | --- | --- | --- | --- | --- | --- | --- | --- | --- | --- | --- | --- | --- | --- | --- | --- | --- | --- | --- | --- | --- | --- | --- | --- | --- | --- | --- | --- | --- | --- | --- | --- | --- | --- | --- | --- | --- | --- | --- | --- | --- | --- | --- | --- | --- | --- | --- | --- | --- | --- | --- | --- | --- | --- | --- | --- | --- | --- | --- | --- | --- | --- | --- | --- | --- | --- | --- | --- | --- | --- | --- | --- | --- | --- | --- | --- | --- | --- | --- | --- | --- | --- | --- | --- | --- | --- | --- | --- | --- | --- | --- | --- | --- | --- | --- | --- | --- | --- | --- | --- | --- | --- | --- | --- | --- | --- | --- | --- | --- | --- | --- | --- | --- | --- | --- | --- | --- | --- | --- | --- | --- | --- | --- | --- | --- | --- | --- | --- | --- | --- | --- | --- | --- | --- | --- | --- | --- | --- | --- | --- | --- | --- | --- | --- | --- | --- | --- | --- | --- | --- | --- | --- | --- | --- | --- | --- | --- | --- | --- | --- | --- | --- | --- | --- | --- | --- | --- | --- | --- | --- | --- | --- | --- | --- | --- | --- | --- | --- | --- | --- | --- | --- | --- | --- | --- | --- | --- | --- | --- | --- | --- | --- | --- | --- | --- | --- | --- | --- | --- | --- | --- | --- | --- | --- | --- | --- | --- | --- | --- | --- | --- | --- | --- | --- | --- | --- | --- | --- | --- | --- | --- | --- | --- | --- | --- | --- | --- | --- | --- | --- | --- | --- | --- | --- | --- | --- | --- | --- | --- | --- | --- | --- | --- | --- | --- | --- | --- | --- | --- | --- | --- | --- | --- | --- | --- | --- | --- | --- | --- | --- | --- | --- | --- | --- | --- | --- | --- | --- | --- | --- | --- | --- | --- | --- | --- | --- | --- | --- | --- | --- | --- | --- | --- | --- | --- | --- | --- | --- | --- | --- | --- | --- | --- | --- | --- | --- | --- | --- | --- | --- | --- | --- | --- | --- | --- | --- | --- | --- | --- | --- | --- | --- | --- | --- | --- | --- | --- | --- | --- | --- | --- | --- | --- | --- | --- | --- | --- | --- | --- | --- | --- | --- | --- | --- | --- | --- | --- | --- | --- | --- | --- | --- | --- | --- | --- | --- | --- | --- | --- | --- | --- | --- | --- | --- | --- | --- | --- | --- | --- | --- | --- | --- | --- | --- | --- | --- | --- | --- | --- | --- | --- | --- | --- | --- | --- | --- | --- | --- | --- | --- | --- | --- | --- | --- | --- | --- | --- | --- | --- | --- | --- | --- | --- | --- | --- | --- | --- | --- | --- | --- | --- | --- | --- | --- | --- | --- | --- | --- | --- | --- | --- | --- | --- | --- | --- | --- | --- | --- | --- | --- | --- | --- | --- | --- | --- | --- | --- | --- | --- | --- | --- | --- | --- | --- | --- | --- | --- | --- | --- | --- | --- | --- | --- | --- | --- | --- | --- | --- | --- | --- | --- | --- | --- | --- | --- | --- | --- | --- | --- | --- | --- | --- | --- | --- | --- | --- | --- | --- | --- | --- | --- | --- | --- | --- | --- | --- | --- | --- | --- | --- | --- | --- | --- | --- | --- | --- | --- | --- | --- | --- | --- | --- | --- | --- | --- | --- | --- | --- | --- | --- | --- | --- | --- | --- | --- | --- | --- | --- | --- | --- | --- | --- | --- | --- | --- | --- | --- | --- | --- | --- | --- | --- | --- | --- | --- | --- | --- | --- | --- | --- | --- | --- | --- | --- | --- | --- | --- | --- | --- | --- | --- | --- | --- | --- | --- | --- | --- | --- | --- | --- | --- | --- | --- | --- | --- | --- | --- | --- | --- | --- | --- | --- | --- | --- | --- | --- | --- | --- | --- | --- | --- | --- | --- | --- | --- | --- | --- | --- | --- | --- | --- | --- | --- | --- | --- | --- | --- | --- | --- | --- | --- | --- | --- | --- | --- | --- | --- | --- | --- | --- | --- | --- | --- | --- | --- | --- | --- | --- | --- | --- | --- | --- | --- | --- | --- | --- | --- | --- | --- | --- | --- | --- | --- | --- | --- | --- | --- | --- | --- | --- | --- | --- | --- | --- | --- | --- | --- | --- | --- | --- | --- | --- | --- | --- | --- | --- | --- | --- | --- | --- | --- | --- | --- | --- | --- | --- | --- | --- | --- | --- | --- | --- | --- | --- | --- | --- | --- | --- | --- | --- | --- | --- | --- | --- | --- | --- | --- | --- | --- | --- | --- | --- | --- | --- | --- | --- | --- | --- | --- | --- | --- | --- | --- | --- | --- | --- | --- | --- | --- | --- | --- | --- | --- | --- | --- | --- | --- | --- | --- | --- | --- | --- | --- | --- | --- | --- | --- | --- | --- | --- | --- | --- | --- | --- | --- | --- | --- | --- | --- | --- | --- | --- | --- | --- | --- | --- | --- | --- | --- | --- | --- | --- | --- | --- | --- | --- | --- | --- | --- | --- | --- | --- | --- | --- | --- | --- | --- | --- | --- | --- | --- | --- | --- | --- | --- | --- | --- | --- | --- | --- | --- | --- | --- | --- | --- | --- | --- | --- | --- | --- | --- | --- | --- | --- | --- | --- | --- | --- | --- | --- | --- | --- | --- | --- | --- | --- | --- | --- | --- | --- | --- | --- | --- | --- | --- | --- | --- | --- | --- | --- | --- | --- | --- | --- | --- | --- | --- | --- | --- | --- | --- | --- | --- | --- | --- | --- | --- | --- | --- | --- | --- | --- | --- | --- | --- | --- | --- | --- | --- | --- | --- | --- | --- | --- | --- | --- | --- | --- | --- | --- | --- | --- | --- | --- | --- | --- | --- | --- | --- | --- | --- | --- | --- | --- | --- | --- | --- | --- | --- | --- | --- | --- | --- | --- | --- | --- | --- | --- | --- | --- | --- | --- | --- | --- | --- | --- | --- | --- | --- | --- | --- | --- | --- | --- | --- | --- | --- | --- | --- | --- | --- | --- | --- | --- | --- | --- | --- | --- | --- | --- | --- | --- | --- | --- | --- | --- | --- | --- | --- | --- | --- | --- | --- | --- | --- | --- | --- | --- | --- | --- | --- | --- | --- | --- | --- | --- | --- | --- | --- | --- | --- | --- | --- | --- | --- | --- | --- | --- | --- | --- | --- | --- | --- | --- | --- | --- | --- | --- | --- | --- | --- | --- | --- | --- | --- | --- | --- | --- | --- | --- | --- | --- | --- | --- | --- | --- | --- | --- | --- | --- | --- | --- | --- | --- | --- | --- | --- | --- | --- | --- | --- | --- | --- | --- | --- | --- | --- | --- | --- | --- | --- | --- | --- | --- | --- | --- | --- | --- | --- | --- | --- | --- | --- | --- | --- | --- | --- | --- | --- | --- | --- | --- | --- | --- | --- | --- | --- | --- | --- | --- | --- | --- | --- | --- | --- | --- | --- | --- | --- | --- | --- | --- | --- | --- | --- | --- | --- | --- | --- | --- | --- | --- | --- | --- | --- | --- | --- | --- | --- | --- | --- | --- | --- | --- | --- | --- | --- | --- | --- | --- | --- | --- | --- | --- | --- | --- | --- | --- | --- | --- | --- | --- | --- | --- | --- | --- | --- | --- | --- | --- | --- | --- | --- | --- | --- | --- | --- | --- | --- | --- | --- | --- | --- | --- | --- | --- | --- | --- | --- | --- | --- | --- | --- | --- | --- | --- | --- | --- | --- | --- | --- | --- | --- | --- | --- | --- | --- | --- | --- | --- | --- | --- | --- | --- | --- | --- | --- | --- | --- | --- | --- | --- | --- | --- | --- | --- | --- | --- | --- | --- | --- | --- | --- | --- | --- | --- | --- | --- | --- | --- | --- | --- | --- | --- | --- | --- | --- | --- | --- | --- | --- | --- | --- | --- | --- | --- | --- | --- | --- | --- | --- | --- | --- | --- | --- | --- | --- | --- | --- | --- | --- | --- | --- | --- | --- | --- | --- | --- | --- | --- | --- | --- | --- | --- | --- | --- | --- | --- | --- | --- | --- | --- | --- | --- | --- | --- | --- | --- | --- | --- | --- | --- | --- | --- | --- | --- | --- | --- | --- | --- | --- | --- | --- | --- | --- | --- | --- | --- | --- | --- | --- | --- | --- | --- | --- | --- | --- | --- | --- | --- | --- | --- | --- | --- | --- | --- | --- | --- | --- | --- | --- | --- | --- | --- | --- | --- | --- | --- | --- | --- | --- | --- | --- | --- | --- | --- | --- | --- | --- | --- | --- | --- | --- | --- | --- | --- | --- | --- | --- | --- | --- | --- | --- | --- | --- | --- | --- | --- | --- | --- | --- | --- | --- | --- | --- | --- | --- | --- | --- | --- | --- | --- | --- | --- | --- | --- | --- | --- | --- | --- | --- | --- | --- | --- | --- | --- | --- | --- | --- | --- | --- | --- | --- | --- | --- | --- | --- | --- | --- | --- | --- | --- | --- | --- | --- | --- | --- | --- | --- | --- | --- | --- | --- | --- | --- | --- | --- | --- | --- | --- | --- | --- | --- | --- | --- | --- | --- | --- | --- | --- | --- | --- | --- | --- | --- | --- | --- | --- | --- | --- | --- | --- | --- | --- | --- | --- | --- | --- | --- | --- | --- | --- | --- | --- | --- | --- | --- | --- | --- | --- | --- | --- | --- | --- | --- | --- | --- | --- | --- | --- | --- | --- | --- | --- | --- | --- | --- | --- | --- | --- | --- | --- | --- | --- | --- | --- | --- | --- | --- | --- | --- | --- | --- | --- | --- | --- | --- | --- | --- | --- | --- | --- | --- | --- | --- | --- | --- | --- | --- | --- | --- | --- | --- | --- | --- | --- | --- | --- | --- | --- | --- | --- | --- | --- | --- | --- | --- | --- | --- | --- | --- | --- | --- | --- | --- | --- | --- | --- | --- | --- | --- | --- | --- | --- | --- | --- | --- | --- | --- | --- | --- | --- | --- | --- | --- | --- | --- | --- | --- | --- | --- | --- | --- | --- | --- | --- | --- | --- | --- | --- | --- | --- | --- | --- | --- | --- | --- | --- | --- | --- | --- | --- | --- | --- | --- | --- | --- | --- | --- | --- | --- | --- | --- | --- | --- | --- | --- | --- | --- | --- | --- | --- | --- | --- | --- | --- | --- | --- | --- | --- | --- | --- | --- | --- | --- | --- | --- | --- | --- | --- | --- | --- | --- | --- | --- | --- | --- | --- | --- | --- | --- | --- | --- | --- | --- | --- | --- | --- | --- | --- | --- | --- | --- | --- | --- | --- | --- | --- | --- | --- | --- | --- | --- | --- | --- | --- | --- | --- | --- | --- | --- | --- | --- | --- | --- | --- | --- | --- | --- | --- | --- | --- | --- | --- | --- | --- | --- | --- | --- | --- | --- | --- | --- | --- | --- | --- | --- | --- | --- | --- | --- | --- | --- | --- | --- | --- | --- | --- | --- | --- | --- | --- | --- | --- | --- | --- | --- | --- | --- | --- | --- | --- | --- | --- | --- | --- | --- | --- | --- | --- | --- | --- | --- | --- | --- | --- | --- | --- | --- | --- | --- | --- | --- | --- | --- | --- | --- | --- | --- | --- | --- | --- | --- | --- | --- | --- | --- | --- | --- | --- | --- | --- | --- | --- | --- | --- | --- | --- | --- | --- | --- | --- | --- | --- | --- | --- | --- | --- | --- | --- | --- | --- | --- | --- | --- | --- | --- | --- | --- | --- | --- | --- | --- | --- | --- | --- | --- | --- | --- | --- | --- | --- | --- | --- | --- | --- | --- | --- | --- | --- | --- | --- | --- | --- | --- | --- | --- | --- | --- | --- | --- | --- | --- | --- | --- | --- | --- | --- | --- | --- | --- | --- | --- | --- | --- | --- | --- | --- | --- | --- | --- | --- | --- | --- | --- | --- | --- | --- | --- | --- | --- | --- | --- | --- | --- | --- | --- | --- | --- | --- | --- | --- | --- | --- | --- | --- | --- | --- | --- | --- | --- | --- | --- | --- | --- | --- | --- | --- | --- | --- | --- | --- | --- | --- | --- | --- | --- | --- | --- | --- | --- | --- | --- | --- | --- | --- | --- | --- | --- | --- | --- | --- | --- | --- | --- | --- | --- | --- | --- | --- | --- | --- | --- | --- | --- | --- | --- | --- | --- | --- | --- | --- | --- | --- | --- | --- | --- | --- | --- | --- | --- | --- | --- | --- | --- | --- | --- | --- | --- | --- | --- | --- | --- | --- | --- | --- | --- | --- | --- | --- | --- | --- | --- | --- | --- | --- | --- | --- | --- | --- | --- | --- | --- | --- | --- | --- | --- | --- | --- | --- | --- | --- | --- | --- | --- | --- | --- | --- | --- | --- | --- | --- | --- | --- | --- | --- | --- | --- | --- | --- | --- | --- | --- | --- | --- | --- | --- | --- | --- | --- | --- | --- | --- | --- | --- | --- | --- | --- | --- | --- | --- | --- | --- | --- | --- | --- | --- | --- | --- | --- | --- | --- | --- | --- | --- | --- | --- | --- | --- | --- | --- | --- | --- | --- | --- | --- | --- | --- | --- | --- | --- | --- | --- | --- | --- | --- | --- | --- | --- | --- | --- | --- | --- | --- | --- | --- | --- | --- | --- | --- | --- | --- | --- | --- | --- | --- | --- | --- | --- | --- | --- | --- | --- | --- | --- | --- | --- | --- | --- | --- | --- | --- | --- | --- | --- | --- | --- | --- | --- | --- | --- | --- | --- | --- | --- | --- | --- | --- | --- | --- | --- | --- | --- | --- | --- | --- | --- | --- | --- | --- | --- | --- | --- | --- | --- | --- | --- | --- | --- | --- | --- | --- | --- | --- | --- | --- | --- | --- | --- | --- | --- | --- | --- | --- | --- | --- | --- | --- | --- | --- | --- | --- | --- | --- | --- | --- | --- | --- | --- | --- | --- | --- | --- | --- | --- | --- | --- | --- | --- | --- | --- | --- | --- | --- | --- | --- | --- | --- | --- | --- | --- | --- | --- | --- | --- | --- | --- | --- | --- | --- | --- | --- | --- | --- | --- | --- | --- | --- | --- | --- | --- | --- | --- | --- | --- | --- | --- | --- | --- | --- | --- | --- | --- | --- | --- | --- | --- | --- | --- | --- | --- | --- | --- | --- | --- | --- | --- | --- | --- | --- | --- | --- | --- | --- | --- | --- | --- | --- | --- | --- | --- | --- | --- | --- | --- | --- | --- | --- | --- | --- | --- | --- | --- | --- | --- | --- | --- | --- | --- | --- | --- | --- | --- | --- | --- | --- | --- | --- | --- | --- | --- | --- | --- | --- | --- | --- | --- | --- | --- | --- | --- | --- | --- | --- | --- | --- | --- | --- | --- | --- | --- | --- | --- | --- | --- | --- | --- | --- | --- | --- | --- | --- | --- | --- | --- | --- | --- | --- | --- | --- | --- | --- | --- | --- | --- | --- | --- | --- | --- | --- | --- | --- | --- | --- | --- | --- | --- | --- | --- | --- | --- | --- | --- | --- | --- | --- | --- | --- | --- | --- | --- | --- | --- | --- | --- | --- | --- | --- | --- | --- | --- | --- | --- | --- | --- | --- | --- | --- | --- | --- | --- | --- | --- | --- | --- | --- | --- | --- | --- | --- | --- | --- | --- | --- | --- | --- | --- | --- | --- | --- | --- | --- | --- | --- | --- | --- | --- | --- | --- | --- | --- | --- | --- | --- | --- | --- | --- | --- | --- | --- | --- | --- | --- | --- | --- | --- | --- | --- | --- | --- | --- | --- | --- | --- | --- | --- | --- | --- | --- | --- | --- | --- | --- | --- | --- | --- | --- | --- | --- | --- | --- | --- | --- | --- | --- | --- | --- | --- | --- | --- | --- | --- | --- | --- | --- | --- | --- | --- | --- | --- | --- | --- | --- | --- | --- | --- | --- | --- | --- | --- | --- | --- | --- | --- | --- | --- | --- | --- | --- | --- | --- | --- | --- | --- | --- | --- | --- | --- | --- | --- | --- | --- | --- | --- | --- | --- | --- | --- | --- | --- | --- | --- | --- | --- | --- | --- | --- | --- | --- | --- | --- | --- | --- | --- | --- | --- | --- | --- | --- | --- | --- | --- | --- | --- | --- | --- | --- | --- | --- | --- | --- | --- | --- | --- | --- | --- | --- | --- | --- | --- | --- | --- | --- | --- | --- | --- | --- | --- | --- | --- | --- | --- | --- | --- | --- | --- | --- | --- | --- | --- | --- | --- | --- | --- | --- | --- | --- | --- | --- | --- | --- | --- | --- | --- | --- | --- | --- | --- | --- | --- | --- | --- | --- | --- | --- | --- | --- | --- | --- | --- | --- | --- | --- | --- | --- | --- | --- | --- | --- | --- | --- | --- | --- | --- | --- | --- | --- | --- | --- | --- | --- | --- | --- | --- | --- | --- | --- | --- | --- | --- | --- | --- | --- | --- | --- | --- | --- | --- | --- | --- | --- | --- | --- | --- | --- | --- | --- | --- | --- | --- | --- | --- | --- | --- | --- | --- | --- | --- | --- | --- | --- | --- | --- | --- | --- | --- | --- | --- | --- | --- | --- | --- | --- | --- | --- | --- | --- | --- | --- | --- | --- | --- | --- | --- | --- | --- | --- | --- | --- | --- | --- | --- | --- | --- | --- | --- | --- | --- | --- | --- | --- | --- | --- | --- | --- | --- | --- | --- | --- | --- | --- | --- | --- | --- | --- | --- | --- | --- | --- | --- | --- | --- | --- | --- | --- | --- | --- | --- | --- | --- | --- | --- | --- | --- | --- | --- | --- | --- | --- | --- | --- | --- | --- | --- | --- | --- | --- | --- | --- | --- | --- | --- | --- | --- | --- | --- | --- | --- | --- | --- | --- | --- | --- | --- | --- | --- | --- | --- | --- | --- | --- | --- | --- | --- | --- | --- | --- | --- | --- | --- | --- | --- | --- | --- | --- | --- | --- | --- | --- | --- | --- | --- | --- | --- | --- | --- | --- | --- | --- | --- | --- | --- | --- | --- | --- | --- | --- | --- | --- | --- | --- | --- | --- | --- | --- | --- | --- | --- | --- | --- | --- | --- | --- | --- | --- | --- | --- | --- | --- | --- | --- | --- | --- | --- | --- | --- | --- | --- | --- | --- | --- | --- | --- | --- | --- | --- | --- | --- | --- | --- | --- | --- | --- | --- | --- | --- | --- | --- | --- | --- | --- | --- | --- | --- | --- | --- | --- | --- | --- | --- | --- | --- | --- | --- | --- | --- | --- | --- | --- | --- | --- | --- | --- | --- | --- | --- | --- | --- | --- | --- | --- | --- | --- | --- | --- | --- | --- | --- | --- | --- | --- | --- | --- | --- | --- | --- | --- | --- | --- | --- | --- | --- | --- | --- | --- | --- | --- | --- | --- | --- | --- | --- | --- | --- | --- | --- | --- | --- | --- | --- | --- | --- | --- | --- | --- | --- | --- | --- | --- | --- | --- | --- | --- | --- | --- | --- | --- | --- | --- | --- | --- | --- | --- | --- | --- | --- | --- | --- | --- | --- | --- | --- | --- | --- | --- | --- | --- | --- | --- | --- | --- | --- | --- | --- | --- | --- | --- | --- | --- | --- | --- | --- | --- | --- | --- | --- | --- | --- | --- | --- | --- | --- | --- | --- | --- | --- | --- | --- | --- | --- | --- | --- | --- | --- | --- | --- | --- | --- | --- | --- | --- | --- | --- | --- | --- | --- | --- | --- | --- | --- | --- | --- | --- | --- | --- | --- | --- | --- | --- | --- | --- | --- | --- | --- | --- | --- | --- | --- | --- | --- | --- | --- | --- | --- | --- | --- | --- | --- | --- | --- | --- | --- | --- | --- | --- | --- | --- | --- | --- | --- | --- | --- | --- | --- | --- | --- | --- | --- | --- | --- | --- | --- | --- | --- | --- | --- | --- | --- | --- | --- | --- | --- | --- | --- | --- | --- | --- | --- | --- | --- | --- | --- | --- | --- | --- | --- | --- | --- | --- | --- | --- | --- | --- | --- | --- | --- | --- | --- | --- | --- | --- | --- | --- | --- | --- | --- | --- | --- | --- | --- | --- | --- | --- | --- | --- | --- | --- | --- | --- | --- | --- | --- | --- | --- | --- | --- | --- | --- | --- | --- | --- | --- | --- | --- | --- | --- | --- | --- | --- | --- | --- | --- | --- | --- | --- | --- | --- | --- | --- | --- | --- | --- | --- | --- | --- | --- | --- | --- | --- | --- | --- | --- | --- | --- | --- | --- | --- | --- | --- | --- | --- | --- | --- | --- | --- | --- | --- | --- | --- | --- | --- | --- | --- | --- | --- | --- | --- | --- | --- | --- | --- | --- | --- | --- | --- | --- | --- | --- | --- | --- | --- | --- | --- | --- | --- | --- | --- | --- | --- | --- | --- | --- | --- | --- | --- | --- | --- | --- | --- | --- | --- | --- | --- | --- | --- | --- | --- | --- | --- | --- | --- | --- | --- | --- | --- | --- | --- | --- | --- | --- | --- | --- | --- | --- | --- | --- | --- | --- | --- | --- | --- | --- | --- | --- | --- | --- | --- | --- | --- | --- | --- | --- | --- | --- | --- | --- | --- | --- | --- | --- | --- | --- | --- | --- | --- | --- | --- | --- | --- | --- | --- | --- | --- | --- | --- | --- | --- | --- | --- | --- | --- | --- | --- | --- | --- | --- | --- | --- | --- | --- | --- | --- | --- | --- | --- | --- | --- | --- | --- | --- | --- | --- | --- | --- | --- | --- | --- | --- | --- | --- | --- | --- | --- | --- | --- | --- | --- | --- | --- | --- | --- | --- | --- | --- | --- | --- | --- | --- | --- | --- | --- | --- | --- | --- | --- | --- | --- | --- | --- | --- | --- | --- | --- | --- | --- | --- | --- | --- | --- | --- | --- | --- | --- | --- | --- | --- | --- | --- | --- | --- | --- | --- | --- | --- | --- | --- | --- | --- | --- | --- | --- | --- | --- | --- | --- | --- | --- | --- | --- | --- | --- | --- | --- | --- | --- | --- | --- | --- | --- | --- | --- | --- | --- | --- | --- | --- | --- | --- | --- | --- | --- | --- | --- | --- | --- | --- | --- | --- | --- | --- | --- | --- | --- | --- | --- | --- | --- | --- | --- | --- | --- | --- | --- | --- | --- | --- | --- | --- | --- | --- | --- | --- | --- | --- | --- | --- | --- | --- | --- | --- | --- | --- | --- | --- | --- | --- | --- | --- | --- | --- | --- | --- | --- | --- | --- | --- | --- | --- | --- | --- | --- | --- | --- | --- | --- | --- | --- | --- | --- | --- | --- | --- | --- | --- | --- | --- | --- | --- | --- | --- | --- | --- | --- | --- | --- | --- | --- | --- | --- | --- | --- | --- | --- | --- | --- | --- | --- | --- | --- | --- | --- | --- | --- | --- | --- | --- | --- | --- | --- | --- | --- | --- | --- | --- | --- | --- | --- | --- | --- | --- | --- | --- | --- | --- | --- | --- | --- | --- | --- | --- | --- | --- | --- | --- | --- | --- | --- | --- | --- | --- | --- | --- | --- | --- | --- | --- | --- | --- | --- | --- | --- | --- | --- | --- | --- | --- | --- | --- | --- | --- | --- | --- | --- | --- | --- | --- | --- | --- | --- | --- | --- | --- | --- | --- | --- | --- | --- | --- | --- | --- | --- | --- | --- | --- | --- | --- | --- | --- | --- | --- | --- | --- | --- | --- | --- | --- | --- | --- | --- | --- | --- | --- | --- | --- | --- | --- | --- | --- | --- | --- | --- | --- | --- | --- | --- | --- | --- | --- | --- | --- | --- | --- | --- | --- | --- | --- | --- | --- | --- | --- | --- | --- | --- | --- | --- | --- | --- | --- | --- | --- | --- | --- | --- | --- | --- | --- | --- | --- | --- | --- | --- | --- | --- | --- | --- | --- | --- | --- | --- | --- | --- | --- | --- | --- | --- | --- | --- | --- | --- | --- | --- | --- | --- | --- | --- | --- | --- | --- | --- | --- | --- | --- | --- | --- | --- | --- | --- | --- | --- | --- | --- | --- | --- | --- | --- | --- | --- | --- | --- | --- | --- | --- | --- | --- | --- | --- | --- | --- | --- | --- | --- | --- | --- | --- | --- | --- | --- | --- | --- | --- | --- | --- | --- | --- | --- | --- | --- | --- | --- | --- | --- | --- | --- | --- | --- | --- | --- | --- | --- | --- | --- | --- | --- | --- | --- | --- | --- | --- | --- | --- | --- | --- | --- | --- | --- | --- | --- | --- | --- | --- | --- | --- | --- | --- | --- | --- | --- | --- | --- | --- | --- | --- | --- | --- | --- | --- | --- | --- | --- | --- | --- | --- | --- | --- | --- | --- | --- | --- | --- | --- | --- | --- | --- | --- | --- | --- | --- | --- | --- | --- | --- | --- | --- | --- | --- | --- | --- | --- | --- | --- | --- | --- | --- | --- | --- | --- | --- | --- | --- | --- | --- | --- | --- | --- | --- | --- | --- | --- | --- | --- | --- | --- | --- | --- | --- | --- | --- | --- | --- | --- | --- | --- | --- | --- | --- | --- | --- | --- | --- | --- | --- | --- | --- | --- | --- | --- | --- | --- | --- | --- | --- | --- | --- | --- | --- | --- | --- | --- | --- | --- | --- | --- | --- | --- | --- | --- | --- | --- | --- | --- | --- | --- | --- | --- | --- | --- | --- | --- | --- | --- | --- | --- | --- | --- | --- | --- | --- | --- | --- | --- | --- | --- | --- | --- | --- | --- | --- | --- | --- | --- | --- | --- | --- | --- | --- | --- | --- | --- | --- | --- | --- | --- | --- | --- | --- | --- | --- | --- | --- | --- | --- | --- | --- | --- | --- | --- | --- | --- | --- | --- | --- | --- | --- | --- | --- | --- | --- | --- | --- | --- | --- | --- | --- | --- | --- | --- | --- | --- | --- | --- | --- | --- | --- | --- | --- | --- | --- | --- | --- | --- | --- | --- | --- | --- | --- | --- | --- | --- | --- | --- | --- | --- | --- | --- | --- | --- | --- | --- | --- | --- | --- | --- | --- | --- | --- | --- | --- | --- | --- | --- | --- | --- | --- | --- | --- | --- | --- | --- | --- | --- | --- | --- | --- | --- | --- | --- | --- | --- | --- | --- | --- | --- | --- | --- | --- | --- | --- | --- | --- | --- | --- | --- | --- | --- | --- | --- | --- | --- | --- | --- | --- | --- | --- | --- | --- | --- | --- | --- | --- | --- | --- | --- | --- | --- | --- | --- | --- | --- | --- | --- | --- | --- | --- | --- | --- | --- | --- | --- | --- | --- | --- | --- | --- | --- | --- | --- | --- | --- | --- | --- | --- | --- | --- | --- | --- | --- | --- | --- | --- | --- | --- | --- | --- | --- | --- | --- | --- | --- | --- | --- | --- | --- | --- | --- | --- | --- | --- | --- | --- | --- | --- | --- | --- | --- | --- | --- | --- | --- | --- | --- | --- | --- | --- | --- | --- | --- | --- | --- | --- | --- | --- | --- | --- | --- | --- | --- | --- | --- | --- | --- | --- | --- | --- | --- | --- | --- | --- | --- | --- | --- | --- | --- | --- | --- | --- | --- | --- | --- | --- | --- | --- | --- | --- | --- | --- | --- | --- | --- | --- | --- | --- | --- | --- | --- | --- | --- | --- | --- | --- | --- | --- | --- | --- | --- | --- | --- | --- | --- | --- | --- | --- | --- | --- | --- | --- | --- | --- | --- | --- | --- | --- | --- | --- | --- | --- | --- | --- | --- | --- | --- | --- | --- | --- | --- | --- | --- | --- | --- | --- | --- | --- | --- | --- | --- | --- | --- | --- | --- | --- | --- | --- | --- | --- | --- | --- | --- | --- | --- | --- | --- | --- | --- | --- | --- | --- | --- | --- | --- | --- | --- | --- | --- | --- | --- | --- | --- | --- | --- | --- | --- | --- | --- | --- | --- | --- | --- | --- | --- | --- | --- | --- | --- | --- | --- | --- | --- | --- | --- | --- | --- | --- | --- | --- | --- | --- | --- | --- | --- | --- | --- | --- | --- | --- | --- | --- | --- | --- | --- | --- | --- | --- | --- | --- | --- | --- | --- | --- | --- | --- | --- | --- | --- | --- | --- | --- | --- | --- | --- | --- | --- | --- | --- | --- | --- | --- | --- | --- | --- | --- | --- | --- | --- | --- | --- | --- | --- | --- | --- | --- | --- | --- | --- | --- | --- | --- | --- | --- | --- | --- | --- | --- | --- | --- | --- | --- | --- | --- | --- | --- | --- | --- | --- | --- | --- | --- | --- | --- | --- | --- | --- | --- | --- | --- | --- | --- | --- | --- | --- | --- | --- | --- | --- | --- | --- | --- | --- | --- | --- | --- | --- | --- | --- | --- | --- | --- | --- | --- | --- | --- | --- | --- | --- | --- | --- | --- | --- | --- | --- | --- | --- | --- | --- | --- | --- | --- | --- | --- | --- | --- | --- | --- | --- | --- | --- | --- | --- | --- | --- | --- | --- | --- | --- | --- | --- | --- | --- | --- | --- | --- | --- | --- | --- | --- | --- | --- | --- | --- | --- | --- | --- | --- | --- | --- | --- | --- | --- | --- | --- | --- | --- | --- | --- | --- | --- | --- | --- | --- | --- | --- | --- | --- | --- | --- | --- | --- | --- | --- | --- | --- | --- | --- | --- | --- | --- | --- | --- | --- | --- | --- | --- | --- | --- | --- | --- | --- | --- | --- | --- | --- | --- | --- | --- | --- | --- | --- | --- | --- | --- | --- | --- | --- | --- | --- | --- | --- | --- | --- | --- | --- | --- | --- | --- | --- | --- | --- | --- | --- | --- | --- | --- | --- | --- | --- | --- | --- | --- | --- | --- | --- | --- | --- | --- | --- | --- | --- | --- | --- | --- | --- | --- | --- | --- | --- | --- | --- | --- | --- | --- | --- | --- | --- | --- | --- | --- | --- | --- | --- | --- | --- | --- | --- | --- | --- | --- | --- | --- | --- | --- | --- | --- | --- | --- | --- | --- | --- | --- | --- | --- | --- | --- | --- | --- | --- | --- | --- | --- | --- | --- | --- | --- | --- | --- | --- | --- | --- | --- | --- | --- | --- | --- | --- | --- | --- | --- | --- | --- | --- | --- | --- | --- | --- | --- | --- | --- | --- | --- | --- | --- | --- | --- | --- | --- | --- | --- | --- | --- | --- | --- | --- | --- | --- | --- | --- | --- | --- | --- | --- | --- | --- | --- | --- | --- | --- | --- | --- | --- | --- | --- | --- | --- | --- | --- | --- | --- | --- | --- | --- | --- | --- | --- | --- | --- | --- | --- | --- | --- | --- | --- | --- | --- | --- | --- | --- | --- | --- | --- | --- | --- | --- | --- | --- | --- | --- | --- | --- | --- | --- | --- | --- | --- | --- | --- | --- | --- | --- | --- | --- | --- | --- | --- | --- | --- | --- | --- | --- | --- | --- | --- | --- | --- | --- | --- | --- | --- | --- | --- | --- | --- | --- | --- | --- | --- | --- | --- | --- | --- | --- | --- | --- | --- | --- | --- | --- | --- | --- | --- | --- | --- | --- | --- | --- | --- | --- | --- | --- | --- | --- | --- | --- | --- | --- | --- | --- | --- | --- | --- | --- | --- | --- | --- | --- | --- | --- | --- | --- | --- | --- | --- | --- | --- | --- | --- | --- | --- | --- | --- | --- | --- | --- | --- | --- | --- | --- | --- | --- | --- | --- | --- | --- | --- | --- | --- | --- | --- | --- | --- | --- | --- | --- | --- | --- | --- | --- | --- | --- | --- | --- | --- | --- | --- | --- | --- | --- | --- | --- | --- | --- | --- | --- | --- | --- | --- | --- | --- | --- | --- | --- | --- | --- | --- | --- | --- | --- | --- | --- | --- | --- | --- | --- | --- | --- | --- | --- | --- | --- | --- | --- | --- | --- | --- | --- | --- | --- | --- | --- | --- | --- | --- | --- | --- | --- | --- | --- | --- | --- | --- | --- | --- | --- | --- | --- | --- | --- | --- | --- | --- | --- | --- | --- | --- | --- | --- | --- | --- | --- | --- | --- | --- | --- | --- | --- | --- | --- | --- | --- | --- | --- | --- | --- | --- | --- | --- | --- | --- | --- | --- | --- | --- | --- | --- | --- | --- | --- | --- | --- | --- | --- | --- | --- | --- | --- | --- | --- | --- | --- | --- | --- | --- | --- | --- | --- | --- | --- | --- | --- | --- | --- | --- | --- | --- | --- | --- | --- | --- | --- | --- | --- | --- | --- | --- | --- | --- | --- | --- | --- | --- | --- | --- | --- | --- | --- | --- | --- | --- | --- | --- | --- | --- | --- | --- | --- | --- | --- | --- | --- | --- | --- | --- | --- | --- | --- | --- | --- | --- | --- | --- | --- | --- | --- | --- | --- | --- | --- | --- | --- | --- | --- | --- | --- | --- | --- | --- | --- | --- | --- | --- | --- | --- | --- | --- | --- | --- | --- | --- | --- | --- | --- | --- | --- | --- | --- | --- | --- | --- | --- | --- | --- | --- | --- | --- | --- | --- | --- | --- | --- | --- | --- | --- | --- | --- | --- | --- | --- | --- | --- | --- | --- | --- | --- | --- | --- | --- | --- | --- | --- | --- | --- | --- | --- | --- | --- | --- | --- | --- | --- | --- | --- | --- | --- | --- | --- | --- | --- | --- | --- | --- | --- | --- | --- | --- | --- | --- | --- | --- | --- | --- | --- | --- | --- | --- | --- | --- | --- | --- | --- | --- | --- | --- | --- | --- | --- | --- | --- | --- | --- | --- | --- | --- | --- | --- | --- | --- | --- | --- | --- | --- | --- | --- | --- | --- | --- | --- | --- | --- | --- | --- | --- | --- | --- | --- | --- | --- | --- | --- | --- | --- | --- | --- | --- | --- | --- | --- | --- | --- | --- | --- | --- | --- | --- | --- | --- | --- | --- | --- | --- | --- | --- | --- | --- | --- | --- | --- | --- | --- | --- | --- | --- | --- | --- | --- | --- | --- | --- | --- | --- | --- | --- | --- | --- | --- | --- | --- | --- | --- | --- | --- | --- | --- | --- | --- | --- | --- | --- | --- | --- | --- | --- | --- | --- | --- | --- | --- | --- | --- | --- | --- | --- | --- | --- | --- | --- | --- | --- | --- | --- | --- | --- | --- | --- | --- | --- | --- | --- | --- | --- | --- | --- | --- | --- | --- | --- | --- | --- | --- | --- | --- | --- | --- | --- | --- | --- | --- | --- | --- | --- | --- | --- | --- | --- | --- | --- | --- | --- | --- | --- | --- | --- | --- | --- | --- | --- | --- | --- | --- | --- | --- | --- | --- | --- | --- | --- | --- | --- | --- | --- | --- | --- | --- | --- | --- | --- | --- | --- | --- | --- | --- | --- | --- | --- | --- | --- | --- | --- | --- | --- | --- | --- | --- | --- | --- | --- | --- | --- | --- | --- | --- | --- | --- | --- | --- | --- | --- | --- | --- | --- | --- | --- | --- | --- | --- | --- | --- | --- | --- | --- | --- | --- | --- | --- | --- | --- | --- | --- | --- | --- | --- | --- | --- | --- | --- | --- | --- | --- | --- | --- | --- | --- | --- | --- | --- | --- | --- | --- | --- | --- | --- | --- | --- | --- | --- | --- | --- | --- | --- | --- | --- | --- | --- | --- | --- | --- | --- | --- | --- | --- | --- | --- | --- | --- | --- | --- | --- | --- | --- | --- | --- | --- | --- | --- | --- | --- | --- | --- | --- | --- | --- | --- | --- | --- | --- | --- | --- | --- | --- | --- | --- | --- | --- | --- | --- | --- | --- | --- | --- | --- | --- | --- | --- | --- | --- | --- | --- | --- | --- | --- | --- | --- | --- | --- | --- | --- | --- | --- | --- | --- | --- | --- | --- | --- | --- | --- | --- | --- | --- | --- | --- | --- | --- | --- | --- | --- | --- | --- | --- | --- | --- | --- | --- | --- | --- | --- | --- | --- | --- | --- | --- | --- | --- | --- | --- | --- | --- | --- | --- | --- | --- | --- | --- | --- | --- | --- | --- | --- | --- | --- | --- | --- | --- | --- | --- | --- | --- | --- | --- | --- | --- | --- | --- | --- | --- | --- | --- | --- | --- | --- | --- | --- | --- | --- | --- | --- | --- | --- | --- | --- | --- | --- | --- | --- | --- | --- | --- | --- | --- | --- | --- | --- | --- | --- | --- | --- | --- | --- | --- | --- | --- | --- | --- | --- | --- | --- | --- | --- | --- | --- | --- | --- | --- | --- | --- | --- | --- | --- | --- | --- | --- | --- | --- | --- | --- | --- | --- | --- | --- | --- | --- | --- | --- | --- | --- | --- | --- | --- | --- | --- | --- | --- | --- | --- | --- | --- | --- | --- | --- | --- | --- | --- | --- | --- | --- | --- | --- | --- | --- | --- | --- | --- | --- | --- | --- | --- | --- | --- | --- | --- | --- | --- | --- | --- | --- | --- | --- | --- | --- | --- | --- | --- | --- | --- | --- | --- | --- | --- | --- | --- | --- | --- | --- | --- | --- | --- | --- | --- | --- | --- | --- | --- | --- | --- | --- | --- | --- | --- | --- | --- | --- | --- | --- | --- | --- | --- | --- | --- | --- | --- | --- | --- | --- | --- | --- | --- | --- | --- | --- | --- | --- | --- | --- | --- | --- | --- | --- | --- | --- | --- | --- | --- | --- | --- | --- | --- | --- | --- | --- | --- | --- | --- | --- | --- | --- | --- | --- | --- | --- | --- | --- | --- | --- | --- | --- | --- | --- | --- | --- | --- | --- | --- | --- | --- | --- | --- | --- | --- | --- | --- | --- | --- | --- | --- | --- | --- | --- | --- | --- | --- | --- | --- | --- | --- | --- | --- | --- | --- | --- | --- | --- | --- | --- | --- | --- | --- | --- | --- | --- | --- | --- | --- | --- | --- | --- | --- | --- | --- | --- | --- | --- | --- | --- | --- | --- | --- | --- | --- | --- | --- | --- | --- | --- | --- | --- | --- | --- | --- | --- | --- | --- | --- | --- | --- | --- | --- | --- | --- | --- | --- | --- | --- | --- | --- | --- | --- | --- | --- | --- | --- | --- | --- | --- | --- | --- | --- | --- | --- | --- | --- | --- | --- | --- | --- | --- | --- | --- | --- | --- | --- | --- | --- | --- | --- | --- | --- | --- | --- | --- | --- | --- | --- | --- | --- | --- | --- | --- | --- | --- | --- | --- | --- | --- | --- | --- | --- | --- | --- | --- | --- | --- | --- | --- | --- | --- | --- | --- | --- | --- | --- | --- | --- | --- | --- | --- | --- | --- | --- | --- | --- | --- | --- | --- | --- | --- | --- | --- | --- | --- | --- | --- | --- | --- | --- | --- | --- | --- | --- | --- | --- | --- | --- | --- | --- | --- | --- | --- | --- | --- | --- | --- | --- | --- | --- | --- | --- | --- | --- | --- | --- | --- | --- | --- | --- | --- | --- | --- | --- | --- | --- | --- | --- | --- | --- | --- | --- | --- | --- | --- | --- | --- | --- | --- | --- | --- | --- | --- | --- | --- | --- | --- | --- | --- | --- | --- | --- | --- | --- | --- | --- | --- | --- | --- | --- | --- | --- | --- | --- | --- | --- | --- | --- | --- | --- | --- | --- | --- | --- | --- | --- | --- | --- | --- | --- | --- | --- | --- | --- | --- | --- | --- | --- | --- | --- | --- | --- | --- | --- | --- | --- | --- | --- | --- | --- | --- | --- | --- | --- | --- | --- | --- | --- | --- | --- | --- | --- | --- | --- | --- | --- | --- | --- | --- | --- | --- | --- | --- | --- | --- | --- | --- | --- | --- | --- | --- | --- | --- | --- | --- | --- | --- | --- | --- | --- | --- | --- | --- | --- | --- | --- | --- | --- | --- | --- | --- | --- | --- | --- | --- | --- | --- | --- | --- | --- | --- | --- | --- | --- | --- | --- | --- | --- | --- | --- | --- | --- | --- | --- | --- | --- | --- | --- | --- | --- | --- | --- | --- | --- | --- | --- | --- | --- | --- | --- | --- | --- | --- | --- | --- | --- | --- | --- | --- | --- | --- | --- | --- | --- | --- | --- | --- | --- | --- | --- | --- | --- | --- | --- | --- | --- | --- | --- | --- | --- | --- | --- | --- | --- | --- | --- | --- | --- | --- | --- | --- | --- | --- | --- | --- | --- | --- | --- | --- | --- | --- | --- | --- | --- | --- | --- | --- | --- | --- | --- | --- | --- | --- | --- | --- | --- | --- | --- | --- | --- | --- | --- | --- | --- | --- | --- | --- | --- | --- | --- | --- | --- | --- | --- | --- | --- | --- | --- | --- | --- | --- | --- | --- | --- | --- | --- | --- | --- | --- | --- | --- | --- | --- | --- | --- | --- | --- | --- | --- | --- | --- | --- | --- | --- | --- | --- | --- | --- | --- | --- | --- | --- | --- | --- | --- | --- | --- | --- | --- | --- | --- | --- | --- | --- | --- | --- | --- | --- | --- | --- | --- | --- | --- | --- | --- | --- | --- | --- | --- | --- | --- | --- | --- | --- | --- | --- | --- | --- | --- | --- | --- | --- | --- | --- | --- | --- | --- | --- | --- | --- | --- | --- | --- | --- | --- | --- | --- | --- | --- | --- | --- | --- | --- | --- | --- | --- | --- | --- | --- | --- | --- | --- | --- | --- | --- | --- | --- | --- | --- | --- | --- | --- | --- | --- | --- | --- | --- | --- | --- | --- | --- | --- | --- | --- | --- | --- | --- | --- | --- | --- | --- | --- | --- | --- | --- | --- | --- | --- | --- | --- | --- | --- | --- | --- | --- | --- | --- | --- | --- | --- | --- | --- | --- | --- | --- | --- | --- | --- | --- | --- | --- | --- | --- | --- | --- | --- | --- | --- | --- | --- | --- | --- | --- | --- | --- | --- | --- | --- | --- | --- | --- | --- | --- | --- | --- | --- | --- | --- | --- | --- | --- | --- | --- | --- | --- | --- | --- | --- | --- | --- | --- | --- | --- | --- | --- | --- | --- | --- | --- | --- | --- | --- | --- | --- | --- | --- | --- | --- | --- | --- | --- | --- | --- | --- | --- | --- | --- | --- | --- | --- | --- | --- | --- | --- | --- | --- | --- | --- | --- | --- | --- | --- | --- | --- | --- | --- | --- | --- | --- | --- | --- | --- | --- | --- | --- | --- | --- | --- | --- | --- | --- | --- | --- | --- | --- | --- | --- | --- | --- | --- | --- | --- | --- | --- | --- | --- | --- | --- | --- | --- | --- | --- | --- | --- | --- | --- | --- | --- | --- | --- | --- | --- | --- | --- | --- | --- | --- | --- | --- | --- | --- | --- | --- | --- | --- | --- | --- | --- | --- | --- | --- | --- | --- | --- | --- | --- | --- | --- | --- | --- | --- | --- | --- | --- | --- | --- | --- | --- | --- | --- | --- | --- | --- | --- | --- | --- | --- | --- | --- | --- | --- | --- | --- | --- | --- | --- | --- | --- | --- | --- | --- | --- | --- | --- | --- | --- | --- | --- | --- | --- | --- | --- | --- | --- | --- | --- | --- | --- | --- | --- | --- | --- | --- | --- | --- | --- | --- | --- | --- | --- | --- | --- | --- | --- | --- | --- | --- | --- | --- | --- | --- | --- | --- | --- | --- | --- | --- | --- | --- | --- | --- | --- | --- | --- | --- | --- | --- | --- | --- | --- | --- | --- | --- | --- | --- | --- | --- | --- | --- | --- | --- | --- | --- | --- | --- | --- | --- | --- | --- | --- | --- | --- | --- | --- | --- | --- | --- | --- | --- | --- | --- | --- | --- | --- | --- | --- | --- | --- | --- | --- | --- | --- | --- | --- | --- | --- | --- | --- | --- | --- | --- | --- | --- | --- | --- | --- | --- | --- | --- | --- | --- | --- | --- | --- | --- | --- | --- | --- | --- | --- | --- | --- | --- | --- | --- | --- | --- | --- | --- | --- | --- | --- | --- | --- | --- | --- | --- | --- | --- | --- | --- | --- | --- | --- | --- | --- | --- | --- | --- | --- | --- | --- | --- | --- | --- | --- | --- | --- | --- | --- | --- | --- | --- | --- | --- | --- | --- | --- | --- | --- | --- | --- | --- | --- | --- | --- | --- | --- | --- | --- | --- | --- | --- | --- | --- | --- | --- | --- | --- | --- | --- | --- | --- | --- | --- | --- | --- | --- | --- | --- | --- | --- | --- | --- | --- | --- | --- | --- | --- | --- | --- | --- | --- | --- | --- | --- | --- | --- | --- | --- | --- | --- | --- | --- | --- | --- | --- | --- | --- | --- | --- | --- | --- | --- | --- | --- | --- | --- | --- | --- | --- | --- | --- | --- | --- | --- | --- | --- | --- | --- | --- | --- | --- | --- | --- | --- | --- | --- | --- | --- | --- | --- | --- | --- | --- | --- | --- | --- | --- | --- | --- | --- | --- | --- | --- | --- | --- | --- | --- | --- | --- | --- | --- | --- | --- | --- | --- | --- | --- | --- | --- | --- | --- | --- | --- | --- | --- | --- | --- | --- | --- | --- | --- | --- | --- | --- | --- | --- | --- | --- | --- | --- | --- | --- | --- | --- | --- | --- | --- | --- | --- | --- | --- | --- | --- | --- | --- | --- | --- | --- | --- | --- | --- | --- | --- | --- | --- | --- | --- | --- | --- | --- | --- | --- | --- | --- | --- | --- | --- | --- | --- | --- | --- | --- | --- | --- | --- | --- | --- | --- | --- | --- | --- | --- | --- | --- | --- | --- | --- | --- | --- | --- | --- | --- | --- | --- | --- | --- | --- | --- | --- | --- | --- | --- | --- | --- | --- | --- | --- | --- | --- | --- | --- | --- | --- | --- | --- | --- | --- | --- | --- | --- | --- | --- | --- | --- | --- | --- | --- | --- | --- | --- | --- | --- | --- | --- | --- | --- | --- | --- | --- | --- | --- | --- | --- | --- | --- | --- | --- | --- | --- | --- | --- | --- | --- | --- | --- | --- | --- | --- | --- | --- | --- | --- | --- | --- | --- | --- | --- | --- | --- | --- | --- | --- | --- | --- | --- | --- | --- | --- | --- | --- | --- | --- | --- | --- | --- | --- | --- | --- | --- | --- | --- | --- | --- | --- | --- | --- | --- | --- | --- | --- | --- | --- | --- | --- | --- | --- | --- | --- | --- | --- | --- | --- | --- | --- | --- | --- | --- | --- | --- | --- | --- | --- | --- | --- | --- | --- | --- | --- | --- | --- | --- | --- | --- | --- | --- | --- | --- | --- | --- | --- | --- | --- | --- | --- | --- | --- | --- | --- | --- | --- | --- | --- | --- | --- | --- | --- | --- | --- | --- | --- | --- | --- | --- | --- | --- | --- | --- | --- | --- | --- | --- | --- | --- | --- | --- | --- | --- | --- | --- | --- | --- | --- | --- | --- | --- | --- | --- | --- | --- | --- | --- | --- | --- | --- | --- | --- | --- | --- | --- | --- | --- | --- | --- | --- | --- | --- | --- | --- | --- | --- | --- | --- | --- | --- | --- | --- | --- | --- | --- | --- | --- | --- | --- | --- | --- | --- | --- | --- | --- | --- | --- | --- | --- | --- | --- | --- | --- | --- | --- | --- | --- | --- | --- | --- | --- | --- | --- | --- | --- | --- | --- | --- | --- | --- | --- | --- | --- | --- | --- | --- | --- | --- | --- | --- | --- | --- | --- | --- | --- | --- | --- | --- | --- | --- | --- | --- | --- | --- | --- | --- | --- | --- | --- | --- | --- | --- | --- | --- | --- | --- | --- | --- | --- | --- | --- | --- | --- | --- | --- | --- | --- | --- | --- | --- | --- | --- | --- | --- | --- | --- | --- | --- | --- | --- | --- | --- | --- | --- | --- | --- | --- | --- | --- | --- | --- | --- | --- | --- | --- | --- | --- | --- | --- | --- | --- | --- | --- | --- | --- | --- | --- | --- | --- | --- | --- | --- | --- | --- | --- | --- | --- | --- | --- | --- | --- | --- | --- | --- | --- | --- | --- | --- | --- | --- | --- | --- | --- | --- | --- | --- | --- | --- | --- | --- | --- | --- | --- | --- | --- | --- | --- | --- | --- | --- | --- | --- | --- | --- | --- | --- | --- | --- | --- | --- | --- | --- | --- | --- | --- | --- | --- | --- | --- | --- | --- | --- | --- | --- | --- | --- | --- | --- | --- | --- | --- | --- | --- | --- | --- | --- | --- | --- | --- | --- | --- | --- | --- | --- | --- | --- | --- | --- | --- | --- | --- | --- | --- | --- | --- | --- | --- | --- | --- | --- | --- | --- | --- | --- | --- | --- | --- | --- | --- | --- | --- | --- | --- | --- | --- | --- | --- | --- | --- | --- | --- | --- | --- | --- | --- | --- | --- | --- | --- | --- | --- | --- | --- | --- | --- | --- | --- | --- | --- | --- | --- | --- | --- | --- | --- | --- | --- | --- | --- | --- | --- | --- | --- | --- | --- | --- | --- | --- | --- | --- | --- | --- | --- | --- | --- | --- | --- | --- | --- | --- | --- | --- | --- | --- | --- | --- | --- | --- | --- | --- | --- | --- | --- | --- | --- | --- | --- | --- | --- | --- | --- | --- | --- | --- | --- | --- | --- | --- | --- | --- | --- | --- | --- | --- | --- | --- | --- | --- | --- | --- | --- | --- | --- | --- | --- | --- | --- | --- | --- | --- | --- | --- | --- | --- | --- | --- | --- | --- | --- | --- | --- | --- | --- | --- | --- | --- | --- | --- | --- | --- | --- | --- | --- | --- | --- | --- | --- | --- | --- | --- | --- | --- | --- | --- | --- | --- | --- | --- | --- | --- | --- | --- | --- | --- | --- | --- | --- | --- | --- | --- | --- | --- | --- | --- | --- | --- | --- | --- | --- | --- | --- | --- | --- | --- | --- | --- | --- | --- | --- | --- | --- | --- | --- | --- | --- | --- | --- | --- | --- | --- | --- | --- | --- | --- | --- | --- | --- | --- | --- | --- | --- | --- | --- | --- | --- | --- | --- | --- | --- | --- | --- | --- | --- | --- | --- | --- | --- | --- | --- | --- | --- | --- | --- | --- | --- | --- | --- | --- | --- | --- | --- | --- | --- | --- | --- | --- | --- | --- | --- | --- | --- | --- | --- | --- | --- | --- | --- | --- | --- | --- | --- | --- | --- | --- | --- | --- | --- | --- | --- | --- | --- | --- | --- | --- | --- | --- | --- | --- | --- | --- | --- | --- | --- | --- | --- | --- | --- | --- | --- | --- | --- | --- | --- | --- | --- | --- | --- | --- | --- | --- | --- | --- | --- | --- | --- | --- | --- | --- | --- | --- | --- | --- | --- | --- | --- | --- | --- | --- | --- | --- | --- | --- | --- | --- | --- | --- | --- | --- | --- | --- | --- | --- | --- | --- | --- | --- | --- | --- | --- | --- | --- | --- | --- | --- | --- | --- | --- | --- | --- | --- | --- | --- | --- | --- | --- | --- | --- | --- | --- | --- | --- | --- | --- | --- | --- | --- | --- | --- | --- | --- | --- | --- | --- | --- | --- | --- | --- | --- | --- | --- | --- | --- | --- | --- | --- | --- | --- | --- | --- | --- | --- | --- | --- | --- | --- | --- | --- | --- | --- | --- | --- | --- | --- | --- | --- | --- | --- | --- | --- | --- | --- | --- | --- | --- | --- | --- | --- | --- | --- | --- | --- | --- | --- | --- | --- | --- | --- | --- | --- | --- | --- | --- | --- | --- | --- | --- | --- | --- | --- | --- | --- | --- | --- | --- | --- | --- | --- | --- | --- | --- | --- | --- | --- | --- | --- | --- | --- | --- | --- | --- | --- | --- | --- | --- | --- | --- | --- | --- | --- | --- | --- | --- | --- | --- | --- | --- | --- | --- | --- | --- | --- | --- | --- | --- | --- | --- | --- | --- | --- | --- | --- | --- | --- | --- | --- | --- | --- | --- | --- | --- | --- | --- | --- | --- | --- | --- | --- | --- | --- | --- | --- | --- | --- | --- | --- | --- | --- | --- | --- | --- | --- | --- | --- | --- | --- | --- | --- | --- | --- | --- | --- | --- | --- | --- | --- | --- | --- | --- | --- | --- | --- | --- | --- | --- | --- | --- | --- | --- | --- | --- | --- | --- | --- | --- | --- | --- | --- | --- | --- | --- | --- | --- | --- | --- | --- | --- | --- | --- | --- | --- | --- | --- | --- | --- | --- | --- | --- | --- | --- | --- | --- | --- | --- | --- | --- | --- | --- | --- | --- | --- | --- | --- | --- | --- | --- | --- | --- | --- | --- | --- | --- | --- | --- | --- | --- | --- | --- | --- | --- | --- | --- | --- | --- | --- | --- | --- | --- | --- | --- | --- | --- | --- | --- | --- | --- | --- | --- | --- | --- | --- | --- | --- | --- | --- | --- | --- | --- | --- | --- | --- | --- | --- | --- | --- | --- | --- | --- | --- | --- | --- | --- | --- | --- | --- | --- | --- | --- | --- | --- | --- | --- | --- | --- | --- | --- | --- | --- | --- | --- | --- | --- | --- | --- | --- | --- | --- | --- | --- | --- | --- | --- | --- | --- | --- | --- | --- | --- | --- | --- | --- | --- | --- | --- | --- | --- | --- | --- | --- | --- | --- | --- | --- | --- | --- | --- | --- | --- | --- | --- | --- | --- | --- | --- | --- | --- | --- | --- | --- | --- | --- | --- | --- | --- | --- | --- | --- | --- | --- | --- | --- | --- | --- | --- | --- | --- | --- | --- | --- | --- | --- | --- | --- | --- | --- | --- | --- | --- | --- | --- | --- | --- | --- | --- | --- | --- | --- | --- | --- | --- | --- | --- | --- | --- | --- | --- | --- | --- | --- | --- | --- | --- | --- | --- | --- | --- | --- | --- | --- | --- | --- | --- | --- | --- | --- | --- | --- | --- | --- | --- | --- | --- | --- | --- | --- | --- | --- | --- | --- | --- | --- | --- | --- | --- | --- | --- | --- | --- | --- | --- | --- | --- | --- | --- | --- | --- | --- | --- | --- | --- | --- | --- | --- | --- | --- | --- | --- | --- | --- | --- | --- | --- | --- | --- | --- | --- | --- | --- | --- | --- | --- | --- | --- | --- | --- | --- | --- | --- | --- | --- | --- | --- | --- | --- | --- | --- | --- | --- | --- | --- | --- | --- | --- | --- | --- | --- | --- | --- | --- | --- | --- | --- | --- | --- | --- | --- | --- | --- | --- | --- | --- | --- | --- | --- | --- | --- | --- | --- | --- | --- | --- | --- | --- | --- | --- | --- | --- | --- | --- | --- | --- | --- | --- | --- | --- | --- | --- | --- | --- | --- | --- | --- | --- | --- | --- | --- | --- | --- | --- | --- | --- | --- | --- | --- | --- | --- | --- | --- | --- | --- | --- | --- | --- | --- | --- | --- | --- | --- | --- | --- | --- | --- | --- | --- | --- | --- | --- | --- | --- | --- | --- | --- | --- | --- | --- | --- | --- | --- | --- | --- | --- | --- | --- | --- | --- | --- | --- | --- | --- | --- | --- | --- | --- | --- | --- | --- | --- | --- | --- | --- | --- | --- | --- | --- | --- | --- | --- | --- | --- | --- | --- | --- | --- | --- | --- | --- | --- | --- | --- | --- | --- | --- | --- | --- | --- | --- | --- | --- | --- | --- | --- | --- | --- | --- | --- | --- | --- | --- | --- | --- | --- | --- | --- | --- | --- | --- | --- | --- | --- | --- | --- | --- | --- | --- | --- | --- | --- | --- | --- | --- | --- | --- | --- | --- | --- | --- | --- | --- | --- | --- | --- | --- | --- | --- | --- | --- | --- | --- | --- | --- | --- | --- | --- | --- | --- | --- | --- | --- | --- | --- | --- | --- | --- | --- | --- | --- | --- | --- | --- | --- | --- | --- | --- | --- | --- | --- | --- | --- | --- | --- | --- | --- | --- | --- | --- | --- | --- | --- | --- | --- | --- | --- | --- | --- | --- | --- | --- | --- | --- | --- | --- | --- | --- | --- | --- | --- | --- | --- | --- | --- | --- | --- | --- | --- | --- | --- | --- | --- | --- | --- | --- | --- | --- | --- | --- | --- | --- | --- | --- | --- | --- | --- | --- | --- | --- | --- | --- | --- | --- | --- | --- | --- | --- | --- | --- | --- | --- | --- | --- | --- | --- | --- | --- | --- | --- | --- | --- | --- | --- | --- | --- | --- | --- | --- | --- | --- | --- | --- | --- | --- | --- | --- | --- | --- | --- | --- | --- | --- | --- | --- | --- | --- | --- | --- | --- | --- | --- | --- | --- | --- | --- | --- | --- | --- | --- | --- | --- | --- | --- | --- | --- | --- | --- | --- | --- | --- | --- | --- | --- | --- | --- | --- | --- | --- | --- | --- | --- | --- | --- | --- | --- | --- | --- | --- | --- | --- | --- | --- | --- | --- | --- | --- | --- | --- | --- | --- | --- | --- | --- | --- | --- | --- | --- | --- | --- | --- | --- | --- | --- | --- | --- | --- | --- | --- | --- | --- | --- | --- | --- | --- | --- | --- | --- | --- | --- | --- | --- | --- | --- | --- | --- | --- | --- | --- | --- | --- | --- | --- | --- | --- | --- | --- | --- | --- | --- | --- | --- | --- | --- | --- | --- | --- | --- | --- | --- | --- | --- | --- | --- | --- | --- | --- | --- | --- | --- | --- | --- | --- | --- | --- | --- | --- | --- | --- | --- | --- | --- | --- | --- | --- | --- | --- | --- | --- | --- | --- | --- | --- | --- | --- | --- | --- | --- | --- | --- | --- | --- | --- | --- | --- | --- | --- | --- | --- | --- | --- | --- | --- | --- | --- | --- | --- | --- | --- | --- | --- | --- | --- | --- | --- | --- | --- | --- | --- | --- | --- | --- | --- | --- | --- | --- | --- | --- | --- | --- | --- | --- | --- | --- | --- | --- | --- | --- | --- | --- | --- | --- | --- | --- | --- | --- | --- | --- | --- | --- | --- | --- | --- | --- | --- | --- | --- | --- | --- | --- | --- | --- | --- | --- | --- | --- | --- | --- | --- | --- | --- | --- | --- | --- | --- | --- | --- | --- | --- | --- | --- | --- | --- | --- | --- | --- | --- | --- | --- | --- | --- | --- | --- | --- | --- | --- | --- | --- | --- | --- | --- | --- | --- | --- | --- | --- | --- | --- | --- | --- | --- | --- | --- | --- | --- | --- | --- | --- | --- | --- | --- | --- | --- | --- | --- | --- | --- | --- | --- | --- | --- | --- | --- | --- | --- | --- | --- | --- | --- | --- | --- | --- | --- | --- | --- | --- | --- | --- | --- | --- | --- | --- | --- | --- | --- | --- | --- | --- | --- | --- | --- | --- | --- | --- | --- | --- | --- | --- | --- | --- | --- | --- | --- | --- | --- | --- | --- | --- | --- | --- | --- | --- | --- | --- | --- | --- | --- | --- | --- | --- | --- | --- | --- | --- | --- | --- | --- | --- | --- | --- | --- | --- | --- | --- | --- | --- | --- | --- | --- | --- | --- | --- | --- | --- | --- | --- | --- | --- | --- | --- | --- | --- | --- | --- | --- | --- | --- | --- | --- | --- | --- | --- | --- | --- | --- | --- | --- | --- | --- | --- | --- | --- | --- | --- | --- | --- | --- | --- | --- | --- | --- | --- | --- | --- | --- | --- | --- | --- | --- | --- | --- | --- | --- | --- | --- | --- | --- | --- | --- | --- | --- | --- | --- | --- | --- | --- | --- | --- | --- | --- | --- | --- | --- | --- | --- | --- | --- | --- | --- | --- | --- | --- | --- | --- | --- | --- | --- | --- | --- | --- | --- | --- | --- | --- | --- | --- | --- | --- | --- | --- | --- | --- | --- | --- | --- | --- | --- | --- | --- | --- | --- | --- | --- | --- | --- | --- | --- | --- | --- | --- | --- | --- | --- | --- | --- | --- | --- | --- | --- | --- | --- | --- | --- | --- | --- | --- | --- | --- | --- | --- | --- | --- | --- | --- | --- | --- | --- | --- | --- | --- | --- | --- | --- | --- | --- | --- | --- | --- | --- | --- | --- | --- | --- | --- | --- | --- | --- | --- | --- | --- | --- | --- | --- | --- | --- | --- | --- | --- | --- | --- | --- | --- | --- | --- | --- | --- | --- | --- | --- | --- | --- | --- | --- | --- | --- | --- | --- | --- | --- | --- | --- | --- | --- | --- | --- | --- | --- | --- | --- | --- | --- | --- | --- | --- | --- | --- | --- | --- | --- | --- | --- | --- | --- | --- | --- | --- | --- | --- | --- | --- | --- | --- | --- | --- | --- | --- | --- | --- | --- | --- | --- | --- | --- | --- | --- | --- | --- | --- | --- | --- | --- | --- | --- | --- | --- | --- | --- | --- | --- | --- | --- | --- | --- | --- | --- | --- | --- | --- | --- | --- | --- | --- | --- | --- | --- | --- | --- | --- | --- | --- | --- | --- | --- | --- | --- | --- | --- | --- | --- | --- | --- | --- | --- | --- | --- | --- | --- | --- | --- | --- | --- | --- | --- | --- | --- | --- | --- | --- | --- | --- | --- | --- | --- | --- | --- | --- | --- | --- | --- | --- | --- | --- | --- | --- | --- | --- | --- | --- | --- | --- | --- | --- | --- | --- | --- | --- | --- | --- | --- | --- | --- | --- | --- | --- | --- | --- | --- | --- | --- | --- | --- | --- | --- | --- | --- | --- | --- | --- | --- | --- | --- | --- | --- | --- | --- | --- | --- | --- | --- | --- | --- | --- | --- | --- | --- | --- | --- | --- | --- | --- | --- | --- | --- | --- | --- | --- | --- | --- | --- | --- | --- | --- | --- | --- | --- | --- | --- | --- | --- | --- | --- | --- | --- | --- | --- | --- | --- | --- | --- | --- | --- | --- | --- | --- | --- | --- | --- | --- | --- | --- | --- | --- | --- | --- | --- | --- | --- | --- | --- | --- | --- | --- | --- | --- | --- | --- | --- | --- | --- | --- | --- | --- | --- | --- | --- | --- | --- | --- | --- | --- | --- | --- | --- | --- | --- | --- | --- | --- | --- | --- | --- | --- | --- | --- | --- | --- | --- | --- | --- | --- | --- | --- | --- | --- | --- | --- | --- | --- | --- | --- | --- | --- | --- | --- | --- | --- | --- | --- | --- | --- | --- | --- | --- | --- | --- | --- | --- | --- | --- | --- | --- | --- | --- | --- | --- | --- | --- | --- | --- | --- | --- | --- | --- | --- | --- | --- | --- | --- | --- | --- | --- | --- | --- | --- | --- | --- | --- | --- | --- | --- | --- | --- | --- | --- | --- | --- | --- | --- | --- | --- | --- | --- | --- | --- | --- | --- | --- | --- | --- | --- | --- | --- | --- | --- | --- | --- | --- | --- | --- | --- | --- | --- | --- | --- | --- | --- | --- | --- | --- | --- | --- | --- | --- | --- | --- | --- | --- | --- | --- | --- | --- | --- | --- | --- | --- | --- | --- | --- | --- | --- | --- | --- | --- | --- | --- | --- | --- | --- | --- | --- | --- | --- | --- | --- | --- | --- | --- | --- | --- | --- | --- | --- | --- | --- | --- | --- | --- | --- | --- | --- | --- | --- | --- | --- | --- | --- | --- | --- | --- | --- | --- | --- | --- | --- | --- | --- | --- | --- | --- | --- | --- | --- | --- | --- | --- | --- | --- | --- | --- | --- | --- | --- | --- | --- | --- | --- | --- | --- | --- | --- | --- | --- | --- | --- | --- | --- | --- | --- | --- | --- | --- | --- | --- | --- | --- | --- | --- | --- | --- | --- | --- | --- | --- | --- | --- | --- | --- | --- | --- | --- | --- | --- | --- | --- | --- | --- | --- | --- | --- | --- | --- | --- | --- | --- | --- | --- | --- | --- | --- | --- | --- | --- | --- | --- | --- | --- | --- | --- | --- | --- | --- | --- | --- | --- | --- | --- | --- | --- | --- | --- | --- | --- | --- | --- | --- | --- | --- | --- | --- | --- | --- | --- | --- | --- | --- | --- | --- | --- | --- | --- | --- | --- | --- | --- | --- | --- | --- | --- | --- | --- | --- | --- | --- | --- | --- | --- | --- | --- | --- | --- | --- | --- | --- | --- | --- | --- | --- | --- | --- | --- | --- | --- | --- | --- | --- | --- | --- | --- | --- | --- | --- | --- | --- | --- | --- | --- | --- | --- | --- | --- | --- | --- | --- | --- | --- | --- | --- | --- | --- | --- | --- | --- | --- | --- | --- | --- | --- | --- | --- | --- | --- | --- | --- | --- | --- | --- | --- | --- | --- | --- | --- | --- | --- | --- | --- | --- | --- | --- | --- | --- | --- | --- | --- | --- | --- | --- | --- | --- | --- | --- | --- | --- | --- | --- | --- | --- | --- | --- | --- | --- | --- | --- | --- | --- | --- | --- | --- | --- | --- | --- | --- | --- | --- | --- | --- | --- | --- | --- | --- | --- | --- | --- | --- | --- | --- | --- | --- | --- | --- | --- | --- | --- | --- | --- | --- | --- | --- | --- | --- | --- | --- | --- | --- | --- | --- | --- | --- | --- | --- | --- | --- | --- | --- | --- | --- | --- | --- | --- | --- | --- | --- | --- | --- | --- | --- | --- | --- | --- | --- | --- | --- | --- | --- | --- | --- | --- | --- | --- | --- | --- | --- | --- | --- | --- | --- | --- | --- | --- | --- | --- | --- | --- | --- | --- | --- | --- | --- | --- | --- | --- | --- | --- | --- | --- | --- | --- | --- | --- | --- | --- | --- | --- | --- | --- | --- | --- | --- | --- | --- | --- | --- | --- | --- | --- | --- | --- | --- | --- | --- | --- | --- | --- | --- | --- | --- | --- | --- | --- | --- | --- | --- | --- | --- | --- | --- | --- | --- | --- | --- | --- | --- | --- | --- | --- | --- | --- | --- | --- | --- | --- | --- | --- | --- | --- | --- | --- | --- | --- | --- | --- | --- | --- | --- | --- | --- | --- | --- | --- | --- | --- | --- | --- | --- | --- | --- | --- | --- | --- | --- | --- | --- | --- | --- | --- | --- | --- | --- | --- | --- | --- | --- | --- | --- | --- | --- | --- | --- | --- | --- | --- | --- | --- | --- | --- | --- | --- | --- | --- | --- | --- | --- | --- | --- | --- | --- | --- | --- | --- | --- | --- | --- | --- | --- | --- | --- | --- | --- | --- | --- | --- | --- | --- | --- | --- | --- | --- | --- | --- | --- | --- | --- | --- | --- | --- | --- | --- | --- | --- | --- | --- | --- | --- | --- | --- | --- | --- | --- | --- | --- | --- | --- | --- | --- | --- | --- | --- | --- | --- | --- | --- | --- | --- | --- | --- | --- | --- | --- | --- | --- | --- | --- | --- | --- | --- | --- | --- | --- | --- | --- | --- | --- | --- | --- | --- | --- | --- | --- | --- | --- | --- | --- | --- | --- | --- | --- | --- | --- | --- | --- | --- | --- | --- | --- | --- | --- | --- | --- | --- | --- | --- | --- | --- | --- | --- | --- | --- | --- | --- | --- | --- | --- | --- | --- | --- | --- | --- | --- | --- | --- | --- | --- | --- | --- | --- | --- | --- | --- | --- | --- | --- | --- | --- | --- | --- | --- | --- | --- | --- | --- | --- | --- | --- | --- | --- | --- | --- | --- | --- | --- | --- | --- | --- | --- | --- | --- | --- | --- | --- | --- | --- | --- | --- | --- | --- | --- | --- | --- | --- | --- | --- | --- | --- | --- | --- | --- | --- | --- | --- | --- | --- | --- | --- | --- | --- | --- | --- | --- | --- | --- | --- | --- | --- | --- | --- | --- | --- | --- | --- | --- | --- | --- | --- | --- | --- | --- | --- | --- | --- | --- | --- | --- | --- | --- | --- | --- | --- | --- | --- | --- | --- | --- | --- | --- | --- | --- | --- | --- | --- | --- | --- | --- | --- | --- | --- | --- | --- | --- | --- | --- | --- | --- | --- | --- | --- | --- | --- | --- | --- | --- | --- | --- | --- | --- | --- | --- | --- | --- | --- | --- | --- | --- | --- | --- | --- | --- | --- | --- | --- | --- | --- | --- | --- | --- | --- | --- | --- | --- | --- | --- | --- | --- | --- | --- | --- | --- | --- | --- | --- | --- | --- | --- | --- | --- | --- | --- | --- | --- | --- | --- | --- | --- | --- | --- | --- | --- | --- | --- | --- | --- | --- | --- | --- | --- | --- | --- | --- | --- | --- | --- | --- | --- | --- | --- | --- | --- | --- | --- | --- | --- | --- | --- | --- | --- | --- | --- | --- | --- | --- | --- | --- | --- | --- | --- | --- | --- | --- | --- | --- | --- | --- | --- | --- | --- | --- | --- | --- | --- | --- | --- | --- | --- | --- | --- | --- | --- | --- | --- | --- | --- | --- | --- | --- | --- | --- | --- | --- | --- | --- | --- | --- | --- | --- | --- | --- | --- | --- | --- | --- | --- | --- | --- | --- | --- | --- | --- | --- | --- | --- | --- | --- | --- | --- | --- | --- | --- | --- | --- | --- | --- | --- | --- | --- | --- | --- | --- | --- | --- | --- | --- | --- | --- | --- | --- | --- | --- | --- | --- | --- | --- | --- | --- | --- | --- | --- | --- | --- | --- | --- | --- | --- | --- | --- | --- | --- | --- | --- | --- | --- | --- | --- | --- | --- | --- | --- | --- | --- | --- | --- | --- | --- | --- | --- | --- | --- | --- | --- | --- | --- | --- | --- | --- | --- | --- | --- | --- | --- | --- | --- | --- | --- | --- | --- | --- | --- | --- | --- | --- | --- | --- | --- | --- | --- | --- | --- | --- | --- | --- | --- | --- | --- | --- | --- | --- | --- | --- | --- | --- | --- | --- | --- | --- | --- | --- | --- | --- | --- | --- | --- | --- | --- | --- | --- | --- | --- | --- | --- | --- | --- | --- | --- | --- | --- | --- | --- | --- | --- | --- | --- | --- | --- | --- | --- | --- | --- | --- | --- | --- | --- | --- | --- | --- | --- | --- | --- | --- | --- | --- | --- | --- | --- | --- | --- | --- | --- | --- | --- | --- | --- | --- | --- | --- | --- | --- | --- | --- | --- | --- | --- | --- | --- | --- | --- | --- | --- | --- | --- | --- | --- | --- | --- | --- | --- | --- | --- | --- | --- | --- | --- | --- | --- | --- | --- | --- | --- | --- | --- | --- | --- | --- | --- | --- | --- | --- | --- | --- | --- | --- | --- | --- | --- | --- | --- | --- | --- | --- | --- | --- | --- | --- | --- | --- | --- | --- | --- | --- | --- | --- | --- | --- | --- | --- | --- | --- | --- | --- | --- | --- | --- | --- | --- | --- | --- | --- | --- | --- | --- | --- | --- | --- | --- | --- | --- | --- | --- | --- | --- | --- | --- | --- | --- | --- | --- | --- | --- | --- | --- | --- | --- | --- | --- | --- | --- | --- | --- | --- | --- | --- | --- | --- | --- | --- | --- | --- | --- | --- | --- | --- | --- | --- | --- | --- | --- | --- | --- | --- | --- | --- | --- | --- | --- | --- | --- | --- | --- | --- | --- | --- | --- | --- | --- | --- | --- | --- | --- | --- | --- | --- | --- | --- | --- | --- | --- | --- | --- | --- | --- | --- | --- | --- | --- | --- | --- | --- | --- | --- | --- | --- | --- | --- | --- | --- | --- | --- | --- | --- | --- | --- | --- | --- | --- | --- | --- | --- | --- | --- | --- | --- | --- | --- | --- | --- | --- | --- | --- | --- | --- | --- | --- | --- | --- | --- | --- | --- | --- | --- | --- | --- | --- | --- | --- | --- | --- | --- | --- | --- | --- | --- | --- | --- | --- | --- | --- | --- | --- | --- | --- | --- | --- | --- | --- | --- | --- | --- | --- | --- | --- | --- | --- | --- | --- | --- | --- | --- | --- | --- | --- | --- | --- | --- | --- | --- | --- | --- | --- | --- | --- | --- | --- | --- | --- | --- | --- | --- | --- | --- | --- | --- | --- | --- | --- | --- | --- | --- | --- | --- | --- | --- | --- | --- | --- | --- | --- | --- | --- | --- | --- | --- | --- | --- | --- | --- | --- | --- | --- | --- | --- | --- | --- | --- | --- | --- | --- | --- | --- | --- | --- | --- | --- | --- | --- | --- | --- | --- | --- | --- | --- | --- | --- | --- | --- | --- | --- | --- | --- | --- | --- | --- | --- | --- | --- | --- | --- | --- | --- | --- | --- | --- | --- | --- | --- | --- | --- | --- | --- | --- | --- | --- | --- | --- | --- | --- | --- | --- | --- | --- | --- | --- | --- | --- | --- | --- | --- | --- | --- | --- | --- | --- | --- | --- | --- | --- | --- | --- | --- | --- | --- | --- | --- | --- | --- | --- | --- | --- | --- | --- | --- | --- | --- | --- | --- | --- | --- | --- | --- | --- | --- | --- | --- | --- | --- | --- | --- | --- | --- | --- | --- | --- | --- |
| |  |  |  |  |  |  |  |  |  |  |  |  |  |  |  |  |  |  |  |  |  |  |  |  |  |  |  |  |  |  |  |  |  |  |  |  |  |  |  |  |  |  |  |  |  |  |  |  |  |  |  |  |  |  |  |  |  |  | | --- | --- | --- | --- | --- | --- | --- | --- | --- | --- | --- | --- | --- | --- | --- | --- | --- | --- | --- | --- | --- | --- | --- | --- | --- | --- | --- | --- | --- | --- | --- | --- | --- | --- | --- | --- | --- | --- | --- | --- | --- | --- | --- | --- | --- | --- | --- | --- | --- | --- | --- | --- | --- | --- | --- | --- | --- | --- | | G0VGH7/1-988 | 1 | M | L | R | F | Q | R | F | A | S | T | Y | A | Q | A | K | V | L | R | K | Y | P | V | G | G | V | F | H | G | Y | E | V | K | R | V | L | A | V | P | E | L | K | L | T | A | V | D | L | V | H | D | Q | T | G | A | E | 55 | | Q6CWW6/1-982 | 1 | M | F | Q | I | R | N | Y | A | T | K | Y | A | Q | S | R | A | L | R | K | Y | P | V | G | G | V | F | H | G | Y | E | V | K | R | L | L | P | V | P | E | L | K | L | T | A | V | D | L | L | H | N | Q | T | G | S | Q | 55 | | Q6FUI7/1-990 | 1 | M | L | R | F | Q | R | F | A | S | T | Y | N | Q | R | A | V | L | R | K | Y | P | V | G | G | I | I | H | G | F | Q | V | R | R | A | V | P | V | P | E | L | K | L | T | A | V | D | L | I | H | E | Q | T | G | A | E | 55 | | Q759T9/1-990 | 1 | M | L | R | F | Q | R | T | V | P | R | V | A | I | R | R | L | A | N | V | Y | S | E | G | A | V | L | H | G | Y | K | V | R | R | A | Q | E | I | P | E | M | R | M | A | A | V | E | L | E | H | E | M | T | G | A | R | 55 | | A7TJ89/1-988 | 1 | M | L | R | F | Q | R | L | A | S | S | Y | A | Q | A | Q | V | L | R | K | Y | P | V | G | G | I | L | H | G | Y | E | V | R | R | V | L | P | V | P | E | L | M | L | T | A | V | D | L | V | H | K | Q | T | G | S | E | 55 | | C5DLW6/1-990 | 1 | M | L | R | F | R | R | Y | A | S | H | F | N | Q | S | K | L | L | R | K | Y | P | V | G | G | V | F | H | G | Y | E | I | N | R | V | L | P | V | P | E | M | R | F | V | A | V | D | L | K | H | L | Q | T | G | A | Q | 55 | | C5DW90/1-986 | 1 | M | L | R | F | Q | R | Y | A | S | S | Y | A | Q | S | R | V | L | R | R | Y | P | V | G | G | L | I | H | G | Y | E | V | R | R | V | L | P | V | P | E | L | K | L | T | A | V | D | L | V | H | E | R | T | G | A | E | 55 | | Kwal\_47.18577/1-987 | 1 | M | L | R | F | R | R | F | A | S | Q | Y | N | Q | A | K | L | L | R | K | Y | P | V | G | K | I | V | H | G | Y | E | I | N | R | V | L | P | V | P | E | M | K | F | V | A | V | D | L | K | H | Q | Q | T | G | A | Q | 55 | | Sbay\_656.22/1-989 | 1 | M | L | R | F | Q | R | F | A | S | S | Y | A | Q | A | Q | A | V | R | R | Y | P | V | G | G | I | L | H | G | Y | E | V | R | R | I | L | P | V | P | E | L | R | L | T | A | V | D | L | V | H | S | Q | T | G | A | E | 55 | | SAKL0G05236g/1-986 | 1 | M | L | R | F | K | R | Y | A | S | R | Y | N | Q | A | K | I | L | R | K | Y | P | V | G | A | V | F | H | G | Y | E | I | K | R | I | L | P | V | Q | E | L | K | L | T | A | V | D | L | I | H | L | Q | T | G | S | Q | 55 | | P32898/1-989 | 1 | M | L | R | F | Q | R | F | A | S | S | Y | A | Q | A | Q | A | V | R | K | Y | P | V | G | G | I | F | H | G | Y | E | V | R | R | I | L | P | V | P | E | L | R | L | T | A | V | D | L | V | H | S | Q | T | G | A | E | 55 | |  | | G0VGH7/1-988 | 56 | H | L | H | I | D | R | D | D | K | N | N | V | F | S | I | S | F | K | T | N | P | P | N | S | T | G | V | P | H | V | L | E | H | T | T | L | C | G | S | K | K | Y | P | V | R | D | P | F | F | K | M | L | N | K | S | 110 | | Q6CWW6/1-982 | 56 | H | L | H | I | D | R | D | D | N | N | N | V | F | S | I | G | F | K | T | N | P | P | D | S | T | G | V | P | H | I | L | E | H | T | T | L | C | G | S | H | K | Y | P | V | R | D | P | F | F | K | M | L | N | R | S | 110 | | Q6FUI7/1-990 | 56 | H | L | H | I | D | R | D | D | K | N | N | V | F | S | I | A | F | R | T | L | P | P | D | A | T | G | V | P | H | I | L | E | H | T | T | L | C | G | S | E | K | Y | P | V | R | D | P | F | F | K | M | L | N | K | S | 110 | | Q759T9/1-990 | 56 | H | L | H | L | E | R | E | D | Q | N | N | V | F | S | V | G | F | R | T | P | P | P | D | A | T | G | V | P | H | I | L | E | H | T | T | L | C | G | S | Q | K | Y | P | V | R | D | P | F | F | K | M | L | N | R | S | 110 | | A7TJ89/1-988 | 56 | H | L | H | I | D | S | A | D | K | N | N | T | F | S | I | S | F | K | T | N | P | P | D | C | T | G | V | P | H | I | L | E | H | T | T | L | C | G | S | Q | K | Y | P | V | R | D | P | F | F | K | M | L | N | R | S | 110 | | C5DLW6/1-990 | 56 | H | L | H | I | D | R | D | D | R | N | N | V | F | S | V | A | F | K | T | N | P | P | D | A | S | G | V | P | H | I | L | E | H | T | T | L | C | G | S | E | K | Y | P | V | R | D | P | F | F | K | M | L | N | R | S | 110 | | C5DW90/1-986 | 56 | H | L | H | I | D | R | D | D | K | N | N | V | F | T | I | G | F | K | T | N | P | P | N | A | T | G | V | P | H | I | L | E | H | T | T | L | C | G | S | V | K | Y | P | V | R | D | P | F | F | K | M | L | N | R | S | 110 | | Kwal\_47.18577/1-987 | 56 | H | L | H | I | D | R | E | D | K | N | N | V | F | S | V | A | F | K | T | N | P | P | D | A | T | G | V | P | H | I | L | E | H | T | T | L | C | G | S | Q | K | Y | P | V | R | D | P | F | F | K | M | L | N | R | S | 110 | | Sbay\_656.22/1-989 | 56 | H | L | H | I | D | R | D | D | K | N | N | V | F | S | I | A | F | K | T | N | P | P | D | A | T | G | V | P | H | I | L | E | H | T | T | L | C | G | S | V | K | Y | P | V | R | D | P | F | F | K | M | L | N | K | S | 110 | | SAKL0G05236g/1-986 | 56 | H | L | H | I | D | R | D | D | K | N | N | V | F | S | V | A | F | K | T | N | P | P | N | A | T | G | V | P | H | I | L | E | H | M | T | L | C | G | S | H | K | Y | P | V | R | D | P | F | F | K | M | L | N | R | S | 110 | | P32898/1-989 | 56 | H | L | H | I | D | R | D | D | K | N | N | V | F | S | I | A | F | K | T | N | P | P | D | S | T | G | V | P | H | I | L | E | H | T | T | L | C | G | S | V | K | Y | P | V | R | D | P | F | F | K | M | L | N | K | S | 110 | |  | | G0VGH7/1-988 | 111 | L | A | N | F | M | N | A | M | T | G | H | D | Y | T | F | F | P | F | A | T | T | N | A | K | D | F | N | N | L | R | D | V | Y | L | D | A | T | L | N | P | L | L | K | Q | E | D | F | Y | Q | E | G | W | R | L | E | 165 | | Q6CWW6/1-982 | 111 | L | A | N | F | M | N | A | M | T | G | H | D | Y | T | F | Y | P | F | A | T | T | N | E | T | D | F | A | N | L | R | D | V | Y | L | D | A | T | L | N | P | L | L | N | Q | Q | D | F | L | Q | E | G | W | R | L | E | 165 | | Q6FUI7/1-990 | 111 | L | A | N | F | M | N | A | M | T | G | P | D | Y | T | F | F | P | F | A | T | T | N | A | R | D | F | V | N | L | R | D | V | Y | L | N | S | T | L | R | P | L | L | K | E | Q | D | F | Y | Q | E | G | W | R | L | E | 165 | | Q759T9/1-990 | 111 | L | A | N | F | M | N | A | M | T | A | H | D | H | T | F | Y | P | F | A | T | T | N | Q | K | D | F | A | N | L | R | D | L | Y | L | D | A | T | L | R | P | L | L | R | H | A | D | F | L | Q | E | G | W | R | L | E | 165 | | A7TJ89/1-988 | 111 | L | S | N | F | M | N | A | M | T | A | H | D | Y | T | F | F | P | F | S | T | T | N | E | T | D | F | K | N | L | R | D | V | Y | I | D | A | T | L | N | P | L | L | K | I | E | D | F | F | Q | E | G | W | R | L | E | 165 | | C5DLW6/1-990 | 111 | L | A | N | F | M | N | A | M | T | G | H | D | Y | T | F | Y | P | F | A | T | A | N | K | A | D | F | A | N | L | R | D | V | Y | L | D | A | T | F | K | P | L | L | K | H | E | D | F | L | Q | E | G | W | R | L | E | 165 | | C5DW90/1-986 | 111 | L | S | N | F | M | N | A | M | T | G | P | D | Y | T | F | Y | P | F | S | T | T | N | R | A | D | F | A | N | L | R | D | V | Y | V | D | A | T | F | N | P | L | L | T | P | E | D | F | Y | Q | E | G | W | R | L | E | 165 | | Kwal\_47.18577/1-987 | 111 | L | A | N | F | M | N | A | M | T | G | H | D | Y | T | F | Y | P | F | A | T | A | N | K | A | D | F | A | N | L | R | D | V | Y | I | D | A | T | F | N | P | L | L | K | H | E | D | F | L | Q | E | G | W | R | L | E | 165 | | Sbay\_656.22/1-989 | 111 | L | A | N | F | M | N | A | M | T | G | P | D | Y | T | F | F | P | F | S | T | T | N | P | Q | D | F | I | N | L | R | G | V | Y | L | D | S | T | L | N | P | L | L | K | Q | E | D | F | D | Q | E | G | W | R | L | E | 165 | | SAKL0G05236g/1-986 | 111 | L | A | N | F | M | N | A | M | T | G | H | D | Y | T | F | Y | P | F | A | T | T | N | K | A | D | F | E | N | L | R | D | V | Y | L | D | A | T | F | N | P | L | L | K | P | E | D | F | Y | Q | E | G | W | R | L | E | 165 | | P32898/1-989 | 111 | L | A | N | F | M | N | A | M | T | G | P | D | Y | T | F | F | P | F | S | T | T | N | P | Q | D | F | A | N | L | R | G | V | Y | L | D | S | T | L | N | P | L | L | K | Q | E | D | F | D | Q | E | G | W | R | L | E | 165 | |  | | G0VGH7/1-988 | 166 | H | D | T | V | T | D | V | T | T | P | I | V | F | K | G | V | V | Y | N | E | M | K | G | Q | V | S | N | A | N | Y | Y | F | W | S | K | Y | Q | E | G | I | Y | P | S | L | N | N | S | G | G | D | P | T | K | I | T | 220 | | Q6CWW6/1-982 | 166 | H | T | K | V | D | D | P | N | S | D | I | G | F | K | G | V | V | Y | N | E | M | K | G | Q | V | S | N | A | N | Y | Y | F | W | I | K | F | Q | E | S | Y | Y | P | S | L | N | N | S | G | G | D | P | T | K | M | T | 220 | | Q6FUI7/1-990 | 166 | H | S | E | V | T | N | P | K | S | D | I | I | F | K | G | V | V | F | N | E | M | K | G | Q | V | S | N | A | D | Y | H | F | W | S | Q | F | Q | Q | N | I | Y | P | S | L | N | N | S | G | G | D | P | Q | K | I | T | 220 | | Q759T9/1-990 | 166 | H | R | D | V | G | D | A | S | S | E | L | V | F | K | G | V | V | Y | N | E | M | K | G | Q | V | S | N | A | D | Y | Y | F | W | I | R | F | Q | E | A | I | Y | P | A | L | H | N | S | G | G | D | P | E | H | I | T | 220 | | A7TJ89/1-988 | 166 | H | S | D | V | T | D | P | K | S | P | I | E | F | K | G | V | V | Y | N | E | M | K | G | Q | I | S | N | A | N | Y | Y | F | W | N | K | F | Q | E | S | I | Y | P | S | L | N | N | S | G | G | D | P | S | K | I | T | 220 | | C5DLW6/1-990 | 166 | H | T | N | L | D | D | P | K | S | D | I | V | F | K | G | V | V | Y | N | E | M | K | G | Q | T | S | S | S | N | Y | Q | F | W | I | K | F | Q | E | N | I | Y | P | S | L | N | N | S | G | G | D | P | Q | K | I | T | 220 | | C5DW90/1-986 | 166 | H | S | D | V | K | D | S | K | S | D | I | V | F | K | G | V | V | Y | N | E | M | K | G | Q | V | S | N | A | N | Y | Y | F | W | I | K | F | Q | E | S | I | Y | P | S | L | N | N | S | G | G | D | P | Q | K | I | T | 220 | | Kwal\_47.18577/1-987 | 166 | H | S | D | L | N | D | P | S | S | D | I | V | F | K | G | V | V | Y | N | E | M | K | G | Q | A | S | S | S | N | Y | Q | F | W | I | K | F | Q | E | S | I | Y | P | S | L | N | N | S | G | G | D | P | Q | K | I | T | 220 | | Sbay\_656.22/1-989 | 166 | H | N | S | I | T | D | P | K | S | N | I | V | F | K | G | V | V | Y | N | E | M | K | G | Q | I | S | N | A | N | Y | Y | F | W | S | K | F | Q | Q | S | I | Y | P | S | L | N | N | S | G | G | D | P | M | K | I | T | 220 | | SAKL0G05236g/1-986 | 166 | H | E | K | T | D | D | P | K | S | P | I | T | F | K | G | V | V | Y | N | E | M | K | G | Q | V | S | N | A | N | Y | Y | F | W | I | K | F | Q | E | S | I | Y | P | S | L | N | N | S | G | G | D | P | K | K | I | T | 220 | | P32898/1-989 | 166 | H | K | N | I | T | D | P | E | S | N | I | V | F | K | G | V | V | Y | N | E | M | K | G | Q | I | S | N | A | N | Y | Y | F | W | S | K | F | Q | Q | S | I | Y | P | S | L | N | N | S | G | G | D | P | M | K | I | T | 220 | |  | | G0VGH7/1-988 | 221 | N | L | K | Y | E | D | L | I | E | F | H | N | K | N | Y | H | P | S | N | C | R | T | F | T | Y | G | N | L | P | L | E | E | T | L | K | R | L | N | E | D | F | I | G | Y | G | K | R | K | V | S | D | Q | V | L | Y | 275 | | Q6CWW6/1-982 | 221 | D | L | Q | Y | E | D | L | I | S | F | H | R | N | N | Y | H | P | S | N | A | K | T | F | T | Y | G | N | F | D | L | N | N | T | L | Q | R | L | N | K | E | Y | Q | G | Y | G | R | R | G | S | K | K | R | E | L | L | 275 | | Q6FUI7/1-990 | 221 | D | L | H | Y | Q | D | L | V | D | F | H | H | A | N | Y | H | P | S | N | A | R | T | F | T | Y | G | S | F | P | L | E | D | T | L | K | K | V | N | E | E | F | R | A | Y | G | K | R | I | I | N | K | K | L | P | K | 275 | | Q759T9/1-990 | 221 | D | L | S | Y | E | D | L | V | A | F | H | Q | N | H | Y | H | P | S | N | A | K | T | F | T | Y | G | N | F | P | L | R | D | T | L | R | K | L | D | D | E | F | R | G | F | G | R | R | A | I | P | Q | M | H | E | K | 275 | | A7TJ89/1-988 | 221 | N | L | T | Y | E | N | L | V | D | F | H | S | K | N | Y | H | P | S | N | S | R | T | Y | T | Y | G | N | L | P | L | E | D | T | L | Q | F | L | N | K | K | F | A | G | Y | G | K | R | N | R | N | K | S | V | L | K | 275 | | C5DLW6/1-990 | 221 | D | L | Q | Y | S | D | L | V | S | F | H | S | K | N | Y | H | P | S | N | A | K | T | F | T | Y | G | S | F | P | L | D | E | T | L | Q | R | L | N | E | E | F | K | L | Y | G | K | R | A | N | R | Y | K | E | L | K | 275 | | C5DW90/1-986 | 221 | D | L | F | Y | Q | D | L | V | D | F | H | H | A | H | Y | H | P | S | N | A | K | T | L | T | Y | G | N | F | P | L | E | E | S | L | Q | R | L | N | E | E | F | E | N | F | G | K | R | S | R | S | D | G | K | L | L | 275 | | Kwal\_47.18577/1-987 | 221 | D | L | Q | Y | S | D | L | V | S | F | H | A | K | N | Y | H | P | S | N | A | K | T | F | T | Y | G | N | L | P | V | E | E | T | L | H | R | L | N | E | E | F | K | L | Y | G | K | R | P | Q | V | S | K | V | L | Q | 275 | | Sbay\_656.22/1-989 | 221 | N | L | K | Y | N | D | L | L | D | F | H | H | R | N | Y | H | P | S | N | A | K | T | F | T | Y | G | N | L | P | L | V | D | T | L | K | Y | L | N | Q | Q | F | A | G | Y | G | K | R | A | R | K | N | K | L | L | L | 275 | | SAKL0G05236g/1-986 | 221 | N | L | Q | Y | P | D | L | V | S | F | H | E | R | N | Y | H | P | S | N | A | K | T | F | T | Y | G | N | F | P | L | Q | E | T | L | A | R | L | D | E | E | F | K | G | F | G | K | R | V | L | S | K | D | I | L | K | 275 | | P32898/1-989 | 221 | D | L | R | Y | G | D | L | L | D | F | H | H | K | N | Y | H | P | S | N | A | K | T | F | T | Y | G | N | L | P | L | V | D | T | L | K | Q | L | N | E | Q | F | S | G | Y | G | K | R | A | R | K | D | K | L | L | M | 275 | |  | | G0VGH7/1-988 | 276 | P | I | K | L | E | K | D | R | T | L | S | M | K | G | Q | I | D | P | M | L | P | P | E | R | Q | L | K | S | S | M | T | W | I | C | G | S | P | A | D | Y | Y | E | T | F | L | L | K | I | I | S | N | M | L | L | D | 330 | | Q6CWW6/1-982 | 276 | P | I | Q | M | K | E | D | V | S | V | E | T | E | G | Q | V | D | P | M | L | P | P | D | K | Q | I | K | T | S | V | T | W | I | C | G | K | P | E | D | T | Y | Q | T | F | L | L | K | I | L | G | N | L | L | L | D | 330 | | Q6FUI7/1-990 | 276 | P | L | E | L | I | E | T | K | E | L | T | L | E | G | Q | I | D | P | M | L | P | A | E | K | Q | T | K | T | S | L | T | W | K | C | G | E | P | T | D | L | Y | E | T | F | L | L | K | I | L | G | N | L | L | L | D | 330 | | Q759T9/1-990 | 276 | P | L | Q | L | R | E | A | V | S | V | E | E | P | C | Q | I | D | P | M | L | P | A | D | K | Q | C | R | T | S | M | T | W | I | C | G | N | P | N | D | V | Y | E | T | F | L | L | K | I | L | G | S | L | L | F | D | 330 | | A7TJ89/1-988 | 276 | P | I | S | L | D | S | I | T | T | I | E | T | T | G | Q | I | D | P | M | L | P | L | D | K | Q | T | K | S | S | M | T | W | L | C | G | S | P | D | N | F | Y | E | T | F | L | L | G | I | I | G | N | L | L | L | D | 330 | | C5DLW6/1-990 | 276 | P | L | E | M | N | S | D | V | R | V | T | E | K | G | Q | V | D | P | M | L | P | P | D | K | Q | L | K | T | S | M | T | W | L | C | G | D | P | A | D | V | Y | G | T | F | L | L | K | V | L | G | N | L | L | M | E | 330 | | C5DW90/1-986 | 276 | P | I | E | L | P | Q | D | L | E | V | R | K | A | G | Q | A | D | P | M | L | P | P | E | R | Q | L | K | T | S | M | T | W | I | C | G | E | P | Q | D | T | Y | E | T | F | L | L | R | V | L | S | N | L | L | M | D | 330 | | Kwal\_47.18577/1-987 | 276 | P | L | Q | L | T | S | D | V | R | V | V | E | K | G | Q | V | D | P | M | L | P | A | D | K | Q | L | K | T | S | M | T | W | L | C | G | D | P | S | D | V | Y | N | S | F | L | L | K | V | L | G | N | L | L | M | E | 330 | | Sbay\_656.22/1-989 | 276 | P | I | E | L | N | K | D | I | D | V | K | L | P | G | Q | V | D | T | M | L | P | P | E | K | Q | I | K | S | S | M | T | W | I | C | G | A | P | H | D | T | Y | E | T | F | L | L | K | V | L | G | N | L | L | M | D | 330 | | SAKL0G05236g/1-986 | 276 | P | L | E | F | K | H | D | I | E | I | K | S | K | G | Q | V | D | P | M | L | P | P | D | K | Q | L | K | T | S | M | T | W | L | C | G | E | P | E | N | T | Y | E | T | F | L | L | K | V | L | G | N | L | L | M | D | 330 | | P32898/1-989 | 276 | P | I | D | L | K | K | D | I | D | V | K | L | L | G | Q | I | D | T | M | L | P | P | E | K | Q | T | K | A | S | M | T | W | I | C | G | A | P | Q | D | T | Y | D | T | F | L | L | K | V | L | G | N | L | L | M | D | 330 | |  | | G0VGH7/1-988 | 331 | G | H | S | S | E | F | Y | Q | A | L | I | - | E | S | S | V | G | F | E | F | S | V | N | T | G | L | E | S | T | T | Q | A | N | L | F | T | I | G | V | Q | G | I | E | N | E | K | V | F | E | E | V | V | R | K | V | 384 | | Q6CWW6/1-982 | 331 | G | H | S | S | P | F | Y | Q | K | L | I | - | E | S | G | L | A | Y | D | F | S | V | N | T | G | V | E | S | Q | T | A | A | N | F | I | T | I | G | V | Q | G | C | D | E | V | D | S | I | Y | E | V | I | N | K | V | 384 | | Q6FUI7/1-990 | 331 | G | H | D | S | I | M | Y | K | G | L | I | - | E | S | G | L | G | H | D | F | S | V | N | T | G | V | E | S | M | T | A | A | N | F | L | T | V | G | I | Q | G | S | Q | N | V | E | E | F | K | S | K | V | F | D | L | 384 | | Q759T9/1-990 | 331 | G | H | S | S | A | F | Y | K | K | L | V | E | E | T | G | L | A | Y | E | L | S | V | N | T | G | V | E | S | Q | T | A | A | N | F | L | T | V | G | V | Q | G | C | T | N | V | G | K | V | H | K | V | I | M | E | T | 385 | | A7TJ89/1-988 | 331 | G | H | S | S | T | L | Y | K | K | L | I | - | E | T | G | L | G | T | D | F | S | V | N | T | G | M | E | T | T | T | E | S | N | F | F | T | V | G | L | N | G | I | S | D | A | A | V | L | K | D | E | V | L | K | T | 384 | | C5DLW6/1-990 | 331 | G | H | S | T | P | L | Y | K | Q | L | I | - | E | S | G | V | G | Y | E | F | S | V | N | S | G | V | E | S | T | T | A | T | N | F | F | T | V | G | V | Q | G | C | S | D | T | I | L | F | E | Q | T | V | K | S | V | 384 | | C5DW90/1-986 | 331 | G | H | S | S | V | F | Y | Q | R | L | I | - | E | S | G | I | G | L | D | F | S | V | N | S | G | L | D | S | A | S | A | A | N | F | V | T | I | G | L | Q | G | V | Q | D | V | D | E | F | R | K | T | V | K | D | I | 384 | | Kwal\_47.18577/1-987 | 331 | G | H | S | S | P | F | Y | Q | K | L | I | - | E | S | G | V | G | Y | D | F | S | V | N | S | G | V | D | A | T | T | A | T | N | F | F | T | V | G | I | Q | G | C | E | N | A | E | K | F | E | T | I | V | K | S | V | 384 | | Sbay\_656.22/1-989 | 331 | G | H | S | S | V | M | Y | Q | K | L | I | - | E | S | G | I | G | L | E | F | S | V | N | S | G | V | E | P | T | T | A | V | N | L | L | T | I | G | V | Q | G | V | H | D | T | Q | L | F | K | D | T | V | N | G | I | 384 | | SAKL0G05236g/1-986 | 331 | G | H | S | S | P | F | Y | Q | K | L | I | - | E | S | G | I | G | Y | E | F | S | V | N | S | G | V | E | S | T | T | A | T | N | F | F | T | I | G | V | Q | G | C | S | D | T | A | Q | F | E | S | T | V | R | E | I | 384 | | P32898/1-989 | 331 | G | H | S | S | V | M | Y | Q | K | L | I | - | E | S | G | I | G | L | E | F | S | V | N | S | G | V | E | P | T | T | A | V | N | L | L | T | V | G | I | Q | G | V | S | D | I | E | I | F | K | D | T | V | N | N | I | 384 | |  | | G0VGH7/1-988 | 385 | F | Q | D | V | M | - | - | K | K | P | F | E | R | K | R | I | D | A | I | I | H | Q | L | E | L | G | K | K | D | Q | K | S | D | F | G | M | Q | I | L | Y | S | I | L | P | G | W | S | N | K | L | D | P | F | D | S | 437 | | Q6CWW6/1-982 | 385 | W | E | E | V | L | - | - | Q | N | P | F | E | E | S | R | I | Q | A | I | I | Q | Q | L | E | L | S | K | K | D | Q | R | A | D | F | G | L | Q | L | L | Y | S | V | L | P | G | W | V | N | K | T | D | P | F | D | S | 437 | | Q6FUI7/1-990 | 385 | F | K | E | F | I | - | - | E | N | D | V | D | S | N | K | V | D | A | I | I | H | Q | L | E | L | S | K | K | D | Q | K | A | D | F | G | L | Q | I | L | Y | S | I | L | P | G | W | T | N | G | I | D | P | I | E | G | 437 | | Q759T9/1-990 | 386 | F | T | A | L | L | - | - | A | Q | P | F | E | K | H | R | V | E | A | I | L | H | Q | L | E | L | S | K | K | D | Q | K | S | D | F | G | L | Q | L | L | Y | G | I | L | P | G | W | V | N | N | T | D | P | F | D | L | 438 | | A7TJ89/1-988 | 385 | L | K | L | I | L | - | - | D | E | P | I | N | N | N | R | V | E | A | I | I | Q | Q | L | E | L | S | K | K | D | Q | K | S | E | F | G | L | L | L | L | Y | S | I | I | P | G | W | S | N | A | R | D | P | F | N | N | 437 | | C5DLW6/1-990 | 385 | F | Q | E | A | L | - | - | E | K | P | F | E | S | D | K | I | E | A | I | I | Q | Q | L | E | L | S | K | K | N | Q | K | A | D | F | G | L | Q | L | L | Y | S | L | V | P | G | W | T | N | K | T | D | P | F | D | H | 437 | | C5DW90/1-986 | 385 | F | A | E | V | A | - | - | N | K | P | F | N | R | D | R | I | E | A | I | M | Q | Q | L | E | L | S | K | K | D | H | K | S | D | F | G | L | Q | L | A | C | S | L | V | P | S | W | T | N | K | V | D | P | F | E | S | 437 | | Kwal\_47.18577/1-987 | 385 | L | Q | E | T | L | - | - | E | K | P | F | E | S | D | K | I | E | A | I | I | Q | Q | L | E | L | S | K | K | N | Q | K | A | D | F | G | L | Q | L | L | Y | S | L | V | P | S | W | V | N | K | T | D | P | F | D | N | 437 | | Sbay\_656.22/1-989 | 385 | F | Q | E | L | L | K | T | D | H | P | F | D | R | K | R | I | D | A | I | I | Q | Q | L | E | L | S | K | K | D | Q | K | A | D | F | G | L | Q | L | L | Y | S | V | L | P | G | W | T | N | E | I | D | P | F | E | N | 439 | | SAKL0G05236g/1-986 | 385 | F | H | E | C | L | - | - | E | K | P | F | E | G | K | K | I | E | A | I | V | Q | Q | L | E | L | S | K | K | D | Q | K | S | D | F | G | L | H | L | L | Y | S | L | L | P | G | W | V | N | K | T | D | P | F | D | G | 437 | | P32898/1-989 | 385 | F | Q | N | L | L | E | T | E | H | P | F | D | R | K | R | I | D | A | I | I | E | Q | L | E | L | S | K | K | D | Q | K | A | D | F | G | L | Q | L | L | Y | S | I | L | P | G | W | T | N | K | I | D | P | F | E | S | 439 | |  | | G0VGH7/1-988 | 438 | L | S | I | D | E | T | L | T | R | F | K | E | D | L | D | K | K | G | D | H | L | F | H | D | L | I | E | K | Y | I | L | D | K | P | C | F | K | F | S | M | R | G | S | E | D | F | S | E | M | L | E | A | E | E | K | 492 | | Q6CWW6/1-982 | 438 | L | L | F | D | E | T | L | E | R | F | Q | E | D | W | A | T | K | G | D | N | L | F | K | D | L | I | K | E | F | V | I | S | K | P | V | F | K | F | T | M | K | G | S | E | T | F | S | Q | K | L | D | A | E | E | Q | 492 | | Q6FUI7/1-990 | 438 | L | E | F | D | E | L | I | G | R | L | K | S | D | F | K | E | N | G | T | K | I | F | K | N | I | L | D | K | Y | I | I | D | Q | P | Y | F | H | F | T | M | K | G | S | E | E | F | S | S | K | L | A | A | E | E | S | 492 | | Q759T9/1-990 | 439 | L | S | L | N | S | A | L | Q | R | F | R | A | D | W | D | R | E | G | D | G | L | F | Q | R | L | L | N | K | Y | V | I | G | K | P | S | F | T | F | T | M | V | G | S | S | D | F | N | Q | V | K | D | Q | N | E | Q | 493 | | A7TJ89/1-988 | 438 | L | L | F | E | E | T | I | A | R | F | R | E | D | W | D | K | K | G | N | E | L | F | K | D | I | I | R | K | Y | I | I | D | K | P | Y | F | Q | F | T | M | K | G | S | E | N | F | S | A | E | L | E | K | E | E | A | 492 | | C5DLW6/1-990 | 438 | L | V | F | D | D | I | L | S | Q | F | R | A | D | W | A | A | K | G | D | E | V | F | R | E | I | I | R | D | Q | I | L | S | K | P | C | F | Y | F | S | M | E | A | D | E | K | Y | S | G | M | L | D | S | E | E | A | 492 | | C5DW90/1-986 | 438 | L | L | F | D | E | I | L | Q | R | F | R | E | D | L | E | N | K | G | D | S | L | F | Q | D | L | L | K | K | Y | I | V | D | K | P | S | F | L | F | T | M | E | P | V | E | D | F | S | Q | R | L | E | V | E | E | K | 492 | | Kwal\_47.18577/1-987 | 438 | L | M | F | D | D | I | I | S | Q | F | R | A | D | W | A | E | K | G | D | E | V | F | R | E | I | V | R | E | Q | I | L | S | K | P | C | F | H | F | S | M | E | A | D | E | N | F | T | A | S | L | E | S | E | E | A | 492 | | Sbay\_656.22/1-989 | 440 | L | L | F | D | E | V | L | Q | R | F | R | S | D | L | E | T | K | G | D | T | L | F | Q | D | L | I | R | K | Y | V | T | N | K | P | C | F | T | F | S | I | E | G | S | E | N | F | S | K | T | L | E | D | E | E | Q | 494 | | SAKL0G05236g/1-986 | 438 | M | L | F | D | E | I | L | E | Q | F | R | E | D | F | E | T | K | G | D | A | I | F | H | N | I | I | N | K | Y | I | L | D | K | P | C | F | K | F | T | M | E | A | S | E | T | F | S | Q | N | L | D | L | E | E | S | 492 | | P32898/1-989 | 440 | L | L | F | E | D | V | L | Q | R | F | R | G | D | L | E | T | K | G | D | T | L | F | Q | D | L | I | R | K | Y | I | V | H | K | P | C | F | T | F | S | I | Q | G | S | E | E | F | S | K | S | L | D | D | E | E | Q | 494 | |  | | G0VGH7/1-988 | 493 | T | R | L | A | D | K | V | S | K | L | N | T | E | D | E | K | V | I | Y | D | R | G | I | V | L | Q | E | M | Q | Q | K | K | E | D | V | S | C | L | P | S | L | Q | I | E | D | I | P | R | I | G | E | K | Y | D | V | 547 | | Q6CWW6/1-982 | 493 | E | R | L | E | R | K | L | K | L | L | D | E | E | D | K | K | V | I | F | E | R | G | K | Q | L | Q | E | L | Q | D | L | K | E | D | L | S | C | L | P | S | L | Q | I | S | A | I | P | R | V | S | K | T | Y | P | L | 547 | | Q6FUI7/1-990 | 493 | T | K | L | D | K | K | L | K | E | L | D | E | T | D | R | K | A | I | F | E | R | G | L | L | L | E | A | A | Q | N | H | K | E | D | L | S | C | L | P | T | L | G | V | A | D | I | S | R | K | V | D | T | Y | D | L | 547 | | Q759T9/1-990 | 494 | S | K | L | K | A | K | V | S | S | L | T | E | S | D | K | E | V | I | Y | K | R | G | L | H | L | Q | E | L | Q | N | S | E | Q | D | L | S | K | L | P | T | L | T | T | A | D | I | P | H | S | S | G | H | Y | F | V | 548 | | A7TJ89/1-988 | 493 | E | R | L | K | A | K | V | T | R | L | D | E | D | D | K | K | I | I | F | K | R | G | A | H | L | Q | A | L | Q | N | E | K | E | D | I | S | C | L | P | S | L | K | V | E | E | I | P | R | T | S | D | V | Y | P | V | 547 | | C5DLW6/1-990 | 493 | R | R | L | E | T | K | V | S | Q | L | D | S | N | D | R | E | V | I | R | E | R | G | K | Q | L | Q | A | K | Q | N | A | T | E | D | L | S | C | L | P | S | L | K | I | K | D | I | P | R | A | G | D | S | Y | E | I | 547 | | C5DW90/1-986 | 493 | E | R | L | G | R | K | L | D | Q | L | D | E | S | D | R | E | V | V | Y | N | R | G | L | L | L | Q | E | K | Q | N | A | K | E | D | L | S | C | L | P | S | V | K | V | S | D | I | P | R | K | D | D | T | F | E | V | 547 | | Kwal\_47.18577/1-987 | 493 | S | R | L | E | E | K | K | S | K | L | D | A | A | D | K | E | T | I | L | A | R | G | K | Q | L | Q | E | N | Q | D | A | T | E | D | L | G | C | L | P | S | L | K | I | S | D | I | P | R | S | G | D | V | Y | T | V | 547 | | Sbay\_656.22/1-989 | 495 | A | R | L | K | K | K | I | A | T | L | D | E | Q | D | K | K | N | I | F | K | R | G | I | L | L | Q | E | K | Q | N | E | K | E | D | L | S | C | L | P | T | L | Q | I | K | D | I | P | R | T | G | D | K | Y | S | I | 549 | | SAKL0G05236g/1-986 | 493 | Q | R | L | K | D | K | I | E | A | M | D | E | T | D | K | E | V | I | Y | K | R | G | L | H | L | Q | E | K | Q | N | A | E | E | D | L | S | L | L | P | S | L | H | I | S | D | I | P | R | T | A | D | V | Y | P | V | 547 | | P32898/1-989 | 495 | T | R | L | R | E | K | I | T | A | L | D | E | Q | D | K | K | N | I | F | K | R | G | I | L | L | Q | E | K | Q | N | E | K | E | D | L | S | C | L | P | T | L | Q | I | K | D | I | P | R | A | G | D | K | Y | S | I | 549 | |  | | G0VGH7/1-988 | 548 | K | K | N | A | - | N | I | K | Y | R | M | T | D | T | N | D | I | T | Y | I | R | A | K | R | S | L | N | N | I | I | P | F | E | L | Y | P | Y | L | P | L | F | A | D | S | L | T | S | L | G | T | A | T | E | S | Y | 601 | | Q6CWW6/1-982 | 548 | L | E | K | D | - | N | V | L | N | R | I | T | D | T | N | G | I | T | Y | V | R | G | K | R | L | L | N | H | H | I | P | R | E | L | Y | P | F | L | P | L | Y | A | D | S | L | T | N | L | G | T | S | T | E | E | F | 601 | | Q6FUI7/1-990 | 548 | N | T | N | A | - | N | I | T | V | R | N | T | A | T | N | G | I | S | Y | I | R | G | K | K | L | I | N | D | M | I | P | L | E | L | Y | P | F | L | S | L | F | A | A | S | L | T | H | L | G | T | K | T | T | P | Y | 601 | | Q759T9/1-990 | 549 | S | R | D | G | - | P | I | T | T | R | Q | T | D | T | N | G | I | T | Y | I | R | M | K | R | P | L | K | G | A | I | P | Y | D | A | Y | P | Y | I | P | L | Y | S | D | G | L | M | N | I | G | T | L | L | E | D | A | 602 | | A7TJ89/1-988 | 548 | V | S | D | S | - | N | I | K | H | R | I | T | E | T | N | G | I | T | Y | L | R | G | K | I | N | L | D | K | K | I | P | E | H | L | F | P | L | L | P | L | F | S | D | C | L | T | H | L | G | T | K | T | E | E | Y | 601 | | C5DLW6/1-990 | 548 | K | R | A | D | - | N | I | F | H | R | I | S | D | T | N | G | I | T | Y | L | R | L | K | R | S | L | D | R | V | I | P | R | D | L | Y | P | Y | L | P | I | F | A | D | C | L | T | S | L | G | T | S | K | E | H | Y | 601 | | C5DW90/1-986 | 548 | R | R | Q | E | P | N | I | W | T | R | I | T | D | T | N | G | I | S | Y | I | R | A | K | R | E | L | S | N | S | I | P | Q | E | L | Y | P | Y | L | P | L | F | A | D | S | L | T | S | L | G | T | S | T | E | D | Y | 602 | | Kwal\_47.18577/1-987 | 548 | A | K | T | A | - | N | V | L | H | R | I | T | G | T | N | G | I | T | Y | L | R | M | K | R | S | L | N | K | S | I | P | F | D | L | Y | P | Y | L | P | I | F | A | D | C | L | T | S | L | G | T | S | K | E | H | Y | 601 | | Sbay\_656.22/1-989 | 550 | E | K | K | N | - | I | S | L | R | R | I | T | D | T | N | G | I | T | Y | I | R | G | K | R | L | L | N | D | I | I | P | Y | E | L | F | P | Y | L | P | L | F | A | E | S | L | T | N | L | G | T | T | K | E | L | F | 603 | | SAKL0G05236g/1-986 | 548 | T | S | R | E | - | N | V | M | N | R | I | T | D | T | N | G | I | T | Y | L | R | A | K | K | S | L | N | K | S | I | P | R | E | L | Y | P | Y | L | S | L | F | A | E | S | L | T | S | L | G | T | S | T | E | D | Y | 601 | | P32898/1-989 | 550 | E | Q | K | N | - | N | T | M | S | R | I | T | D | T | N | G | I | T | Y | V | R | G | K | R | L | L | N | D | I | I | P | F | E | L | F | P | Y | L | P | L | F | A | E | S | L | T | N | L | G | T | T | T | E | S | F | 603 | |  | | G0VGH7/1-988 | 602 | S | D | I | E | D | S | M | K | L | Y | T | G | G | V | S | A | H | I | N | T | S | A | D | P | I | S | L | Q | P | H | L | Y | F | G | F | D | G | W | S | L | N | S | K | S | E | H | I | F | E | I | W | E | K | L | L | 656 | | Q6CWW6/1-982 | 602 | S | T | I | E | E | Q | I | K | L | H | T | G | G | V | S | T | R | V | S | V | N | P | D | A | Q | T | G | K | P | M | L | L | F | Q | V | D | G | W | A | L | N | S | K | T | D | H | I | F | K | F | W | K | K | L | L | 656 | | Q6FUI7/1-990 | 602 | G | A | I | D | N | E | I | K | L | H | T | G | G | I | S | T | N | I | S | V | N | A | D | P | T | T | L | Q | P | N | L | Y | F | D | M | S | G | F | S | L | N | E | K | S | D | Y | I | F | N | F | L | K | T | I | L | 656 | | Q759T9/1-990 | 603 | S | A | I | E | E | Q | I | R | L | H | T | G | G | I | S | V | S | I | G | V | H | P | N | V | E | T | R | L | S | E | L | Y | L | E | I | S | A | C | A | L | N | S | K | T | Q | Y | V | F | D | I | I | N | K | I | M | 657 | | A7TJ89/1-988 | 602 | S | K | I | E | E | A | M | K | L | Y | T | G | G | I | S | T | H | V | D | V | G | S | D | P | I | T | S | H | P | N | L | L | F | R | F | D | G | W | A | L | N | S | K | T | D | K | V | T | E | I | W | E | K | L | L | 656 | | C5DLW6/1-990 | 602 | S | E | I | E | D | E | M | K | L | H | T | G | G | V | S | A | H | V | A | V | H | S | D | P | I | T | C | E | P | S | L | N | F | I | C | E | G | Y | S | L | N | H | K | T | E | R | V | F | D | I | W | R | K | I | L | 656 | | C5DW90/1-986 | 603 | S | D | I | E | D | A | M | K | L | H | T | G | G | I | S | T | M | I | D | V | S | S | D | P | I | T | T | K | P | H | L | Y | F | R | V | S | G | W | S | L | N | S | K | T | E | H | V | F | D | L | W | K | K | L | L | 657 | | Kwal\_47.18577/1-987 | 602 | S | Q | I | E | D | E | M | K | L | H | T | G | G | V | S | T | S | I | S | V | Y | S | N | P | E | T | C | E | P | S | L | E | F | V | C | E | G | Y | S | L | N | H | K | T | E | H | V | L | E | I | W | R | K | L | L | 656 | | Sbay\_656.22/1-989 | 604 | S | E | I | E | D | Q | I | K | L | Y | T | G | G | I | S | T | H | V | E | V | T | S | D | P | N | T | S | E | P | H | L | I | F | G | F | D | G | W | S | L | N | S | K | S | D | H | I | F | K | F | W | S | K | I | L | 658 | | SAKL0G05236g/1-986 | 602 | A | D | I | E | N | E | M | K | L | H | T | G | G | V | S | T | S | V | N | V | H | Q | N | P | V | T | S | V | P | E | L | Q | F | Q | F | D | G | W | C | L | N | S | K | T | E | F | V | F | D | I | W | K | K | L | L | 656 | | P32898/1-989 | 604 | S | E | I | E | D | Q | I | K | L | H | T | G | G | I | S | T | H | V | E | V | T | S | D | P | N | T | T | E | P | R | L | I | F | G | F | D | G | W | S | L | N | S | K | T | D | H | I | F | E | F | W | S | K | I | L | 658 | |  | | G0VGH7/1-988 | 657 | L | N | T | D | F | K | R | N | S | D | K | L | K | T | L | I | R | L | Q | A | T | S | N | T | S | S | V | A | E | S | G | H | L | Y | A | R | G | Y | A | A | A | S | L | D | V | T | K | S | I | N | E | K | L | N | G | 711 | | Q6CWW6/1-982 | 657 | C | E | T | D | F | H | K | H | K | E | K | L | K | V | L | I | R | S | L | A | S | S | N | T | A | S | V | A | E | T | G | H | A | F | A | R | N | F | G | A | A | H | L | S | V | T | K | A | I | N | E | S | L | N | G | 711 | | Q6FUI7/1-990 | 657 | M | E | T | D | F | S | T | H | K | D | K | L | K | V | L | I | N | S | I | A | S | S | N | T | S | H | I | A | D | S | G | H | T | V | A | R | S | F | A | S | G | H | L | S | T | V | A | A | I | Q | E | H | I | S | G | 711 | | Q759T9/1-990 | 658 | N | E | T | A | L | S | V | R | S | E | K | M | K | V | L | I | R | A | A | A | S | S | F | T | S | Y | A | A | E | N | G | H | D | L | A | R | L | H | T | G | A | H | F | S | Q | T | Q | A | I | M | E | Q | T | A | G | 712 | | A7TJ89/1-988 | 657 | V | N | T | D | F | K | Q | N | S | E | K | I | K | V | L | I | K | S | M | V | S | S | N | T | S | S | I | A | E | S | G | H | S | F | A | R | S | Y | S | S | A | H | L | S | T | T | R | A | I | N | E | V | F | H | G | 711 | | C5DLW6/1-990 | 657 | V | E | T | D | I | V | K | H | V | E | K | L | K | V | L | I | R | S | L | A | A | S | N | T | A | S | V | A | E | G | G | H | A | F | A | R | N | F | T | G | A | H | L | T | T | A | K | A | I | N | E | S | L | S | G | 711 | | C5DW90/1-986 | 658 | L | G | T | D | F | Q | K | H | S | E | K | L | K | I | L | I | R | G | L | A | S | N | N | T | S | A | V | A | E | A | G | H | S | F | A | R | G | Y | A | A | A | S | F | S | A | A | R | G | I | S | E | S | F | N | G | 712 | | Kwal\_47.18577/1-987 | 657 | V | D | T | D | I | S | K | H | S | E | K | L | K | V | L | I | R | S | L | A | S | S | N | T | A | S | V | A | E | S | G | H | A | F | A | R | N | Y | T | G | A | H | L | T | T | T | K | S | I | N | E | S | L | N | G | 711 | | Sbay\_656.22/1-989 | 659 | L | E | T | D | F | R | K | N | S | D | K | L | K | V | L | I | R | L | L | A | S | S | N | T | S | S | V | A | D | S | G | H | A | F | A | R | G | Y | S | A | A | H | Y | R | S | S | R | A | I | S | E | T | L | N | G | 713 | | SAKL0G05236g/1-986 | 657 | T | D | T | D | F | N | K | H | K | E | K | L | R | V | L | I | R | S | L | A | S | S | N | T | A | A | V | A | E | S | G | H | S | F | A | R | N | F | T | A | A | H | F | S | N | T | K | A | I | F | E | S | L | A | G | 711 | | P32898/1-989 | 659 | L | E | T | D | F | H | K | N | S | D | K | L | K | V | L | I | R | L | L | A | S | S | N | T | S | S | V | A | D | A | G | H | A | F | A | R | G | Y | S | A | A | H | Y | R | S | S | G | A | I | N | E | T | L | N | G | 713 | |  | | G0VGH7/1-988 | 712 | I | E | Q | L | Q | L | I | N | K | L | N | M | M | L | E | D | E | E | I | F | Q | K | E | V | V | D | K | L | I | R | I | Q | K | L | I | I | N | S | Q | G | L | E | F | F | V | T | T | D | T | E | H | Q | G | G | I | 766 | | Q6CWW6/1-982 | 712 | I | E | Q | L | Q | L | I | N | K | L | S | Q | C | L | D | D | E | A | L | F | E | K | E | V | V | S | K | L | V | E | L | Q | S | Y | I | N | G | S | S | D | M | K | F | M | I | T | S | D | S | Q | V | Q | I | D | A | 766 | | Q6FUI7/1-990 | 712 | V | E | H | Y | K | L | I | S | R | L | C | S | I | M | N | D | D | K | L | F | Q | S | E | V | I | D | K | L | V | M | L | Q | R | I | I | V | N | S | Q | N | M | E | F | F | A | S | V | D | C | Q | A | Q | E | N | K | 766 | | Q759T9/1-990 | 713 | I | E | Q | V | R | H | M | N | N | L | M | S | I | I | E | K | E | A | E | F | N | T | - | V | L | Q | N | L | E | A | M | H | R | K | I | F | V | A | D | G | L | E | V | M | I | T | T | D | N | R | Q | T | S | D | V | 766 | | A7TJ89/1-988 | 712 | I | S | Q | V | Q | L | L | N | K | L | A | M | T | L | E | N | E | E | L | F | Q | T | E | I | V | D | K | L | V | E | L | Q | K | Y | M | M | D | S | N | E | V | Q | F | F | V | T | T | D | S | I | Q | Q | V | A | K | 766 | | C5DLW6/1-990 | 712 | V | E | Q | L | K | L | I | T | Y | L | S | T | I | L | E | K | P | A | L | F | Q | A | E | V | V | D | K | L | N | E | L | K | S | Y | V | I | S | S | E | G | L | E | F | F | T | T | T | D | S | E | K | Q | A | I | K | 766 | | C5DW90/1-986 | 713 | V | E | Q | L | Q | L | I | N | R | L | N | S | L | L | D | D | E | E | S | F | Q | R | E | V | V | D | K | L | V | E | L | K | N | R | I | I | S | S | Q | G | L | E | F | F | I | T | T | D | S | D | P | V | V | S | S | 767 | | Kwal\_47.18577/1-987 | 712 | V | E | Q | L | R | L | I | T | R | L | A | S | I | L | D | S | P | S | L | F | Q | S | E | V | I | D | K | L | N | E | L | K | S | Y | V | I | G | N | D | R | L | E | F | F | V | T | T | D | S | E | R | Q | A | S | S | 766 | | Sbay\_656.22/1-989 | 714 | I | E | Q | L | Q | F | I | N | K | L | H | N | L | L | E | N | E | E | T | F | Q | R | E | V | V | D | K | L | I | E | L | Q | K | Y | I | V | N | A | K | D | M | K | F | F | V | T | S | D | S | D | A | Q | A | Q | I | 768 | | SAKL0G05236g/1-986 | 712 | M | E | Q | L | Q | L | V | T | R | L | S | N | I | L | D | D | D | S | L | F | Q | T | E | V | V | E | K | L | R | E | L | Q | G | Y | V | I | G | S | Q | G | L | G | F | F | V | T | T | D | S | S | N | Q | V | Q | A | 766 | | P32898/1-989 | 714 | I | E | Q | L | Q | F | I | N | R | L | H | S | L | L | D | N | E | E | T | F | Q | R | E | V | V | D | K | L | T | E | L | Q | K | Y | I | V | D | T | N | N | M | N | F | F | I | T | S | D | S | D | V | Q | A | K | T | 768 | |  | | G0VGH7/1-988 | 767 | I | Q | T | Q | V | S | N | F | I | A | K | M | P | H | K | S | P | Q | E | V | S | - | D | N | T | L | N | F | P | L | I | P | S | T | S | G | - | I | S | T | L | I | N | F | P | F | Q | V | H | Y | A | S | E | C | L | 819 | | Q6CWW6/1-982 | 767 | V | H | Q | Q | I | T | G | F | L | S | S | L | P | K | D | S | - | K | P | C | D | - | F | Y | S | E | N | Y | S | M | L | E | N | - | P | G | - | K | P | T | L | L | Q | F | P | F | Q | V | H | Y | T | A | K | C | Y | 817 | | Q6FUI7/1-990 | 767 | I | R | K | E | V | D | Y | F | V | S | T | L | P | N | T | S | S | D | I | S | G | A | I | Q | T | A | C | V | P | R | Y | S | D | - | S | Q | - | V | L | N | L | I | K | F | P | F | Q | V | H | Y | T | A | Q | A | Y | 819 | | Q759T9/1-990 | 767 | V | K | D | Q | A | L | K | F | I | A | G | V | Q | Q | S | A | - | G | A | E | S | - | W | L | P | E | K | Y | S | R | R | A | L | E | K | P | - | Y | P | A | L | L | Q | F | P | F | Q | V | H | Y | T | A | Q | S | T | 818 | | A7TJ89/1-988 | 767 | V | K | Q | E | L | D | T | F | V | G | S | L | S | P | I | E | - | K | N | G | G | - | V | N | I | E | S | Y | P | K | L | A | Q | K | P | G | V | M | S | T | L | I | N | F | P | F | Q | V | H | Y | S | G | S | S | L | 819 | | C5DLW6/1-990 | 767 | T | D | N | E | I | Q | Q | F | V | Q | T | L | P | E | D | S | - | P | S | M | T | - | F | S | S | R | D | Y | P | L | L | D | S | - | K | G | - | K | H | T | L | I | E | F | P | F | Q | V | H | Y | S | A | Q | S | L | 817 | | C5DW90/1-986 | 768 | L | A | K | Q | I | S | Q | F | E | S | Q | L | P | Q | T | L | - | H | N | D | G | - | L | D | P | T | S | F | P | L | L | P | Q | K | - | - | - | P | S | T | L | I | N | F | P | F | Q | V | H | Y | A | A | R | S | L | 817 | | Kwal\_47.18577/1-987 | 767 | A | R | S | Q | I | Q | Q | F | I | Q | A | L | P | E | A | S | - | E | S | A | T | - | F | D | A | E | H | Y | P | L | L | N | S | - | D | A | - | K | A | T | L | I | E | F | P | F | Q | V | H | Y | A | S | Q | S | Y | 817 | | Sbay\_656.22/1-989 | 769 | V | E | S | Q | I | S | K | F | M | E | T | L | P | D | D | K | C | S | L | N | G | - | P | K | T | S | A | Y | P | L | I | K | S | - | E | G | - | K | P | T | L | I | K | F | P | F | Q | V | H | Y | T | S | Q | A | L | 820 | | SAKL0G05236g/1-986 | 767 | V | E | K | Q | I | D | E | F | V | R | A | L | P | S | K | A | - | T | A | Q | N | - | F | C | A | T | N | Y | P | L | L | N | S | - | N | G | - | L | P | T | L | I | D | F | P | F | Q | V | H | Y | A | A | E | S | M | 817 | | P32898/1-989 | 769 | V | E | S | Q | I | S | K | F | M | E | R | L | P | H | G | S | C | L | P | N | G | - | P | K | T | S | D | Y | P | L | I | G | S | - | K | C | - | K | H | T | L | I | K | F | P | F | Q | V | H | Y | T | S | Q | A | L | 820 | |  | | G0VGH7/1-988 | 820 | R | G | V | S | Y | T | H | N | D | G | A | P | L | Q | I | L | S | N | L | L | T | F | K | Y | L | H | R | E | I | R | E | K | G | G | A | Y | G | G | G | A | S | Y | D | A | L | S | G | I | F | G | Y | Y | S | Y | R | 874 | | Q6CWW6/1-982 | 818 | P | G | V | S | Y | T | H | P | D | G | A | K | L | Q | I | L | S | N | M | L | T | H | K | Y | L | H | R | E | I | R | E | K | G | G | A | Y | G | G | G | A | T | Y | S | A | L | D | G | T | F | S | F | Y | S | Y | R | 872 | | Q6FUI7/1-990 | 820 | N | G | V | S | Y | T | H | K | D | G | A | A | L | Q | V | L | A | N | M | L | T | F | K | H | L | H | K | E | I | R | E | K | G | G | A | Y | G | G | G | A | T | F | S | A | L | S | G | I | F | S | Y | Y | S | Y | R | 874 | | Q759T9/1-990 | 819 | Q | G | V | S | Y | T | H | P | D | G | A | H | L | Q | V | L | A | S | L | L | T | F | K | H | L | H | R | E | V | R | E | K | G | G | A | Y | G | G | G | A | T | Y | N | A | T | D | G | I | F | N | F | F | S | Y | R | 873 | | A7TJ89/1-988 | 820 | P | G | V | P | Y | T | H | N | D | G | A | P | L | Q | V | L | S | S | M | L | T | Y | K | Y | L | H | K | E | I | R | E | K | G | G | A | Y | G | G | G | A | T | Y | S | A | L | D | G | I | F | S | F | Y | S | Y | R | 874 | | C5DLW6/1-990 | 818 | V | G | V | P | Y | T | H | R | D | G | A | S | L | Q | V | L | S | N | M | L | T | F | K | H | L | H | R | E | I | R | E | K | G | G | A | Y | G | G | G | A | T | Y | S | A | L | D | G | L | F | S | F | Y | S | Y | R | 872 | | C5DW90/1-986 | 818 | P | G | V | S | Y | T | H | G | D | G | A | P | L | Q | V | L | A | N | L | L | T | F | K | H | L | H | T | E | V | R | E | K | G | G | A | Y | G | G | G | A | S | Y | S | A | L | E | G | M | L | N | F | F | S | Y | R | 872 | | Kwal\_47.18577/1-987 | 818 | L | G | V | P | Y | A | H | E | D | G | A | S | L | Q | V | L | S | N | M | L | T | F | K | H | L | H | R | E | V | R | E | K | G | G | A | Y | G | G | G | A | T | Y | S | A | L | E | G | L | L | A | F | Y | S | Y | R | 872 | | Sbay\_656.22/1-989 | 821 | L | G | V | P | Y | T | H | K | D | G | A | A | L | Q | V | M | S | N | M | L | T | F | K | H | L | H | R | E | V | R | E | K | G | G | A | Y | G | G | G | A | S | Y | S | A | L | A | G | I | F | S | F | Y | S | Y | R | 875 | | SAKL0G05236g/1-986 | 818 | K | G | V | S | Y | T | H | Q | D | G | A | S | L | Q | V | L | A | N | M | L | T | F | K | Y | L | H | R | E | I | R | E | K | G | G | A | Y | G | A | G | A | N | Y | S | A | L | D | G | I | F | N | F | Y | S | Y | R | 872 | | P32898/1-989 | 821 | L | G | V | P | Y | T | H | K | D | G | S | A | L | Q | V | M | S | N | M | L | T | F | K | H | L | H | R | E | V | R | E | K | G | G | A | Y | G | G | G | A | S | Y | S | A | L | A | G | I | F | S | F | Y | S | Y | R | 875 | |  | | G0VGH7/1-988 | 875 | D | P | Q | P | L | R | S | L | K | T | F | K | S | S | A | E | Y | V | L | N | N | A | Q | W | T | K | T | E | L | D | D | A | K | L | T | I | F | Q | Q | I | D | A | P | T | S | R | K | G | E | G | V | T | E | F | L | 929 | | Q6CWW6/1-982 | 873 | D | P | H | A | L | N | S | L | S | T | F | D | S | V | P | E | F | I | L | N | K | S | S | W | G | E | P | D | L | N | E | A | K | L | S | V | F | Q | Q | V | D | S | P | M | S | A | K | N | E | G | T | I | L | F | H | 927 | | Q6FUI7/1-990 | 875 | D | P | N | P | L | A | S | I | Q | T | F | E | K | S | A | S | Y | V | L | N | D | A | K | W | T | Q | S | D | L | D | E | S | K | L | S | I | F | Q | Q | V | D | A | P | I | S | P | K | S | E | G | S | T | F | F | N | 929 | | Q759T9/1-990 | 874 | D | P | Q | P | V | R | S | L | N | I | F | R | N | A | G | K | Y | V | L | N | E | A | R | W | T | A | D | D | L | N | E | A | K | L | S | I | F | Q | R | V | D | A | P | I | S | P | S | S | E | G | L | L | Q | F | R | 928 | | A7TJ89/1-988 | 875 | D | P | D | P | I | R | S | L | E | T | F | H | N | C | G | Q | Y | V | L | E | N | L | K | W | D | T | A | D | L | N | E | A | K | L | T | L | F | Q | Q | V | D | A | P | I | S | R | K | S | E | G | T | I | Y | F | H | 929 | | C5DLW6/1-990 | 873 | D | P | H | P | L | N | S | L | Q | V | F | A | E | S | G | N | Y | V | L | Q | N | S | N | W | K | N | D | D | L | N | E | A | K | L | T | I | F | Q | Q | V | D | A | P | I | S | P | S | S | E | G | S | I | L | F | N | 927 | | C5DW90/1-986 | 873 | D | P | R | P | L | Q | S | L | K | T | F | E | N | A | G | Q | H | A | L | Q | E | A | Q | W | D | S | A | D | L | D | E | A | K | L | T | I | F | Q | R | L | D | A | P | I | S | R | K | S | E | G | V | S | L | F | Y | 927 | | Kwal\_47.18577/1-987 | 873 | D | P | Q | P | L | N | S | L | G | I | F | S | D | S | G | R | Y | V | L | E | D | A | K | W | S | A | S | D | L | D | E | A | K | L | T | I | F | Q | Q | V | D | A | P | T | S | P | S | H | E | G | S | S | L | F | Y | 927 | | Sbay\_656.22/1-989 | 876 | D | P | Q | P | L | N | S | L | E | T | F | K | N | S | G | Q | Y | V | L | D | D | A | K | W | G | T | T | D | L | D | E | A | K | L | T | I | F | Q | Q | V | D | A | P | T | S | P | K | G | E | G | V | T | Y | F | M | 930 | | SAKL0G05236g/1-986 | 873 | D | P | H | A | L | K | S | L | E | T | F | A | K | S | G | E | Y | V | L | N | D | A | K | W | T | A | Q | D | L | D | E | A | K | L | T | I | F | Q | Q | I | D | A | P | I | S | C | K | G | E | G | S | I | L | F | Y | 927 | | P32898/1-989 | 876 | D | P | Q | P | L | K | S | L | E | T | F | K | N | S | G | R | Y | I | L | N | D | A | K | W | G | V | T | D | L | D | E | A | K | L | T | I | F | Q | Q | V | D | A | P | K | S | P | K | G | E | G | V | T | Y | F | M | 930 | |  | | G0VGH7/1-988 | 930 | T | N | V | T | E | E | M | R | Q | T | R | R | E | Q | L | L | D | T | R | L | S | D | I | E | R | V | A | Q | E | Y | L | L | N | - | - | - | K | E | G | V | S | A | V | V | G | P | V | I | E | G | E | T | M | E | P | 981 | | Q6CWW6/1-982 | 928 | Y | D | V | T | D | E | M | K | Q | R | R | R | E | Q | L | L | D | V | N | L | N | D | I | H | Q | V | A | E | E | Y | L | K | Q | - | - | - | D | K | S | I | A | S | I | V | G | P | E | I | - | - | - | - | - | - | P | 973 | | Q6FUI7/1-990 | 930 | L | G | V | T | D | E | M | R | Q | V | R | R | E | Q | L | L | D | T | S | L | L | D | I | H | R | V | A | E | R | Y | I | L | P | - | - | - | N | K | S | I | A | T | V | V | G | P | G | I | D | G | E | T | V | S | P | 981 | | Q759T9/1-990 | 929 | H | N | I | S | D | E | Q | R | D | R | R | R | Q | Q | L | L | K | S | T | L | D | D | V | R | R | V | A | D | I | Y | L | V | Q | P | S | P | S | Q | H | M | S | A | V | V | G | P | E | L | P | R | E | V | W | S | S | 983 | | A7TJ89/1-988 | 930 | S | G | V | T | D | E | M | R | Q | K | R | R | E | Q | L | L | D | T | S | L | I | D | I | H | R | V | A | E | K | Y | L | L | N | - | - | - | K | K | P | I | N | A | V | V | G | P | E | I | E | G | K | T | V | P | P | 981 | | C5DLW6/1-990 | 928 | Y | G | I | T | D | S | M | R | Q | V | R | R | E | Q | L | L | D | V | N | L | E | D | V | R | A | A | A | E | K | Y | L | F | K | - | - | - | K | P | S | V | T | A | V | V | G | P | R | I | E | A | E | M | S | D | G | 979 | | C5DW90/1-986 | 928 | S | G | V | T | D | E | M | R | Q | N | R | R | E | Q | L | L | D | V | T | R | K | D | V | D | R | V | A | E | K | Y | L | L | N | - | - | - | K | D | G | S | N | A | V | V | G | P | E | I | E | G | E | T | V | P | P | 979 | | Kwal\_47.18577/1-987 | 928 | Y | G | V | T | D | S | M | R | Q | K | R | R | E | Q | L | L | D | I | N | L | A | D | V | R | K | A | A | E | K | Y | L | V | N | - | - | - | K | K | S | Y | S | A | V | V | G | P | R | I | E | P | E | M | D | S | A | 979 | | Sbay\_656.22/1-989 | 931 | S | G | V | T | D | D | M | K | Q | A | R | R | E | Q | L | L | D | V | S | L | L | D | V | H | R | V | T | E | K | Y | L | L | N | - | - | - | K | E | G | V | S | T | V | I | G | P | G | I | E | G | K | T | V | S | P | 982 | | SAKL0G05236g/1-986 | 928 | Y | G | I | T | D | Q | M | R | Q | Q | R | R | Q | S | L | L | D | V | Q | L | L | D | I | H | R | A | A | E | K | Y | L | V | G | - | - | - | K | P | S | V | S | A | V | L | G | P | Y | L | D | A | E | M | P | R | D | 979 | | P32898/1-989 | 931 | S | G | V | T | D | D | M | K | Q | A | R | R | E | Q | L | L | D | V | S | L | L | D | V | H | R | V | A | E | K | Y | L | L | N | - | - | - | K | E | G | V | S | T | V | I | G | P | G | I | E | G | K | T | V | S | P | 982 | |  | | G0VGH7/1-988 | 982 | K | W | N | - | - | I | K | N | L | - | - | - | - |  | | | | | | | | | | | | | | | | | | | | | | | | | | | | | | | | | | | | | | | | | | 988 | | Q6CWW6/1-982 | 974 | N | F | D | A | L | V | Q | T | V | - | - | - | - |  | | | | | | | | | | | | | | | | | | | | | | | | | | | | | | | | | | | | | | | | | | 982 | | Q6FUI7/1-990 | 982 | K | W | H | - | - | I | E | D | I | K | V | - | - |  | | | | | | | | | | | | | | | | | | | | | | | | | | | | | | | | | | | | | | | | | | 990 | | Q759T9/1-990 | 984 | Q | W | P | - | - | V | I | K | V | - | - | - | - |  | | | | | | | | | | | | | | | | | | | | | | | | | | | | | | | | | | | | | | | | | | 990 | | A7TJ89/1-988 | 982 | T | W | D | - | - | V | I | N | F | - | - | - | - |  | | | | | | | | | | | | | | | | | | | | | | | | | | | | | | | | | | | | | | | | | | 988 | | C5DLW6/1-990 | 980 | K | W | Q | - | - | I | K | D | L | Q | P | A | K |  | | | | | | | | | | | | | | | | | | | | | | | | | | | | | | | | | | | | | | | | | | 990 | | C5DW90/1-986 | 980 | N | W | Q | - | - | I | K | N | L | - | - | - | - |  | | | | | | | | | | | | | | | | | | | | | | | | | | | | | | | | | | | | | | | | | | 986 | | Kwal\_47.18577/1-987 | 980 | Q | W | Q | - | - | I | K | T | L | N | - | - | - |  | | | | | | | | | | | | | | | | | | | | | | | | | | | | | | | | | | | | | | | | | | 987 | | Sbay\_656.22/1-989 | 983 | K | W | E | - | - | V | K | E | L | - | - | - | - |  | | | | | | | | | | | | | | | | | | | | | | | | | | | | | | | | | | | | | | | | | | 989 | | SAKL0G05236g/1-986 | 980 | Q | W | N | - | - | I | E | K | L | - | - | - | - |  | | | | | | | | | | | | | | | | | | | | | | | | | | | | | | | | | | | | | | | | | | 986 | | P32898/1-989 | 983 | N | W | E | - | - | V | K | E | L | - | - | - | - |  | | | | | | | | | | | | | | | | | | | | | | | | | | | | | | | | | | | | | | | | | | 989 | |
