# Supplementary material for: Plus ça change – evolutionary sequence divergence predicts protein subcellular localization signals
Source: BMC Genomics. 2014 Jan 20;15:46. doi: 10.1186/1471-2164-15-46 (PMC3906766; doi:10.1186/1471-2164-15-46)
Supplement: Additional file 2 — MSA’s of proteins for which sequence divergence changes predicted localization signals. Contains links to ortholog multiple sequence alignments of each protein in Additional file 3: Table S1. [file 1471-2164-15-46-S2.zip › P36517.html]

|  |  |  |  |  |  |  |  |  |  |  |  |  |  |  |  |  |  |  |  |  |  |  |  |  |  |  |  |  |  |  |  |  |  |  |  |  |  |  |  |  |  |  |  |  |  |  |  |  |  |  |  |  |  |  |  |  |  |  |  |  |  |  |  |  |  |  |  |  |  |  |  |  |  |  |  |  |  |  |  |  |  |  |  |  |  |  |  |  |  |  |  |  |  |  |  |  |  |  |  |  |  |  |  |  |  |  |  |  |  |  |  |  |  |  |  |  |  |  |  |  |  |  |  |  |  |  |  |  |  |  |  |  |  |  |  |  |  |  |  |  |  |  |  |  |  |  |  |  |  |  |  |  |  |  |  |  |  |  |  |  |  |  |  |  |  |  |  |  |  |  |  |  |  |  |  |  |  |  |  |  |  |  |  |  |  |  |  |  |  |  |  |  |  |  |  |  |  |  |  |  |  |  |  |  |  |  |  |  |  |  |  |  |  |  |  |  |  |  |  |  |  |  |  |  |  |  |  |  |  |  |  |  |  |  |  |  |  |  |  |  |  |  |  |  |  |  |  |  |  |  |  |  |  |  |  |  |  |  |  |  |  |  |  |  |  |  |  |  |  |  |  |  |  |  |  |  |  |  |  |  |  |  |  |  |  |  |  |  |  |  |  |  |  |  |  |  |  |  |  |  |  |  |  |  |  |  |  |  |  |  |  |  |  |  |  |  |  |  |  |  |  |  |  |  |  |  |  |  |  |  |  |  |  |  |  |  |  |  |  |  |  |  |  |  |  |  |  |  |  |  |  |  |  |  |  |  |  |  |  |  |  |  |  |  |  |  |  |  |  |  |  |  |  |  |  |  |  |  |  |  |  |  |  |  |  |  |  |  |  |  |  |  |  |  |  |  |  |  |  |  |  |  |  |  |  |  |  |  |  |  |  |  |  |  |  |  |  |  |  |  |  |  |  |  |  |  |  |  |  |  |  |  |  |  |  |  |  |  |  |  |  |  |  |  |  |  |  |  |  |  |  |  |  |  |  |  |  |  |  |  |  |  |  |  |  |  |  |  |  |  |  |  |  |  |  |  |  |  |  |  |  |  |  |  |  |  |  |  |  |  |  |  |  |  |  |  |  |  |  |  |  |  |  |  |  |  |  |  |  |  |  |  |  |  |  |  |  |  |  |  |  |  |  |  |  |  |  |  |  |  |  |  |  |  |  |  |  |  |  |  |  |  |  |  |  |  |  |  |  |  |  |  |  |  |  |  |  |  |  |  |  |  |  |  |  |  |  |  |  |  |  |  |  |  |  |  |  |  |  |  |  |  |  |  |  |  |  |  |  |  |  |  |  |  |  |  |  |  |  |  |  |  |  |  |  |  |  |  |  |  |  |  |  |  |  |  |  |  |  |  |  |  |  |  |  |  |  |  |  |  |  |  |  |  |  |  |  |  |  |  |  |  |  |  |  |  |  |  |  |  |  |  |  |  |  |  |  |  |  |  |  |  |  |  |  |  |  |  |  |  |  |  |  |  |  |  |  |  |  |  |  |  |  |  |  |  |  |  |  |  |  |  |  |  |  |  |  |  |  |  |  |  |  |  |  |  |  |  |  |  |  |  |  |  |  |  |  |  |  |  |  |  |  |  |  |  |  |  |  |  |  |  |  |  |  |  |  |  |  |  |  |  |  |  |  |  |  |  |  |  |  |  |  |  |  |  |  |  |  |  |  |  |  |  |  |  |  |  |  |  |  |  |  |  |  |  |  |  |  |  |  |  |  |  |  |  |  |  |  |  |  |  |  |  |  |  |  |  |  |  |  |  |  |  |  |  |  |  |  |  |  |  |  |  |  |  |  |  |  |  |  |  |  |  |  |  |  |  |  |  |  |  |  |  |  |  |  |  |  |  |  |  |  |  |  |  |  |  |  |  |  |  |  |  |  |  |  |  |  |  |  |  |  |  |  |  |  |  |  |  |  |  |  |  |  |  |  |  |  |  |  |  |  |  |  |  |  |  |  |  |  |  |  |  |  |  |  |  |  |  |  |  |  |  |  |  |  |  |  |  |  |  |  |  |  |  |  |  |  |  |  |  |  |  |  |  |  |  |  |  |  |  |  |  |  |  |  |  |  |  |  |  |  |  |  |  |  |  |  |  |  |  |  |  |  |  |  |  |  |  |  |  |  |  |  |  |  |  |  |  |  |  |  |  |  |  |  |  |  |  |  |  |  |  |  |  |  |  |  |  |  |  |  |  |  |  |  |  |  |  |  |  |  |  |  |  |  |  |  |  |  |  |  |  |  |  |  |  |  |  |  |  |  |  |  |  |  |  |  |  |  |  |  |  |  |  |  |  |  |  |  |  |  |  |  |  |  |  |  |  |  |  |  |  |  |  |  |  |  |  |  |  |  |  |  |  |  |  |  |  |  |  |  |  |  |  |  |  |  |  |  |  |  |  |  |  |  |  |  |  |  |  |  |  |  |  |  |  |  |  |  |  |  |  |  |  |  |  |  |  |  |  |  |  |  |  |  |  |  |  |  |  |  |  |  |  |  |  |  |  |  |  |  |  |  |  |  |  |  |  |  |  |  |  |  |  |  |  |  |  |  |  |  |  |  |  |  |  |  |  |  |  |  |  |  |  |  |  |  |  |  |  |  |  |  |  |  |  |  |  |  |  |  |  |  |  |  |  |  |  |  |  |  |  |  |  |  |  |  |  |  |  |  |  |  |  |  |  |  |  |  |  |  |  |  |  |  |  |  |  |  |  |  |  |  |  |  |  |  |  |  |  |  |  |  |  |  |  |  |  |  |  |  |  |  |  |  |  |  |  |  |  |  |  |  |  |  |  |  |  |  |  |  |  |  |  |  |  |  |  |  |  |  |  |  |  |  |  |  |  |  |  |  |  |  |  |  |  |  |  |  |  |  |  |  |  |  |  |  |  |  |  |  |  |  |  |  |  |  |  |  |  |  |  |  |  |  |  |  |  |  |  |  |  |  |  |  |  |  |  |  |  |  |  |  |  |  |  |  |  |  |  |  |  |  |  |  |  |  |  |  |  |  |  |  |  |  |  |  |  |  |  |  |  |  |  |  |  |  |  |  |  |  |  |  |  |  |  |  |  |  |  |  |  |  |  |  |  |  |  |  |  |  |  |  |  |  |  |  |  |  |  |  |  |  |  |  |  |  |  |  |  |  |  |  |  |  |  |  |  |  |  |  |  |  |  |  |  |  |  |  |  |  |  |  |  |  |  |  |  |  |  |  |  |  |  |  |  |  |  |  |  |  |  |  |  |  |  |  |  |  |  |  |  |  |  |  |  |  |  |  |  |  |  |  |  |  |  |  |  |  |  |  |  |  |  |  |  |  |  |  |  |  |  |  |  |  |  |  |  |  |  |  |  |  |  |  |  |  |  |  |  |  |  |  |  |  |  |  |  |  |  |  |  |  |  |  |  |  |  |  |  |  |  |  |  |  |  |  |  |  |  |  |  |  |  |  |  |  |  |  |  |  |  |  |  |  |  |  |  |  |  |  |  |  |  |  |  |  |  |  |  |  |  |  |  |  |  |  |  |  |  |  |  |  |  |  |  |  |  |  |  |  |  |  |  |  |  |  |  |  |  |  |  |  |  |  |  |  |  |  |  |  |  |  |  |  |  |  |  |  |  |  |  |  |  |  |  |  |  |  |  |  |  |  |  |  |  |  |  |  |  |  |  |  |  |  |  |  |  |  |  |  |  |  |  |  |  |  |  |  |  |  |  |  |  |  |  |  |  |  |  |  |  |  |  |  |  |  |  |  |  |  |  |  |  |  |  |  |  |  |  |  |  |  |  |  |  |  |  |  |  |  |  |  |  |  |  |  |  |  |  |  |  |  |  |  |  |  |  |  |  |  |  |  |  |  |  |  |  |  |  |  |  |  |  |  |  |  |  |  |  |  |  |  |  |  |  |  |  |  |  |  |  |  |  |  |  |  |  |  |  |  |  |  |  |  |  |  |  |  |  |  |  |  |  |  |  |  |  |  |  |  |  |  |  |  |  |  |  |  |  |  |  |  |  |  |  |  |  |  |  |  |  |  |  |  |  |  |  |  |  |  |  |  |  |  |  |  |  |  |  |  |  |  |  |  |  |  |  |  |  |  |  |  |  |  |  |  |  |  |  |  |  |  |  |  |  |  |  |  |  |  |  |  |  |  |  |  |  |  |  |  |  |  |  |  |  |  |  |  |  |  |  |  |  |  |  |  |  |  |  |  |  |  |  |  |  |  |  |  |  |  |  |  |  |  |  |  |  |  |  |  |  |  |  |  |  |  |  |  |  |  |  |  |  |  |  |  |  |  |  |  |  |  |  |  |  |  |  |  |  |  |  |  |  |  |  |  |  |  |  |  |  |  |  |  |  |  |  |  |  |  |  |  |  |  |  |  |  |  |  |  |  |  |  |  |  |  |  |  |  |  |  |  |  |  |  |  |  |  |  |  |  |  |  |  |  |  |  |  |  |  |  |  |  |  |  |  |  |  |  |  |  |  |  |  |  |  |  |  |  |  |  |  |  |  |  |  |  |  |  |  |  |  |  |  |  |  |  |  |  |  |  |  |  |  |  |  |  |  |  |  |  |  |  |  |  |  |  |  |  |  |  |  |  |  |  |  |  |  |  |  |  |  |  |  |  |  |  |  |  |  |  |  |  |  |  |  |  |  |  |  |  |  |  |  |  |  |  |  |  |  |  |  |  |  |  |  |  |  |  |  |  |  |  |  |  |  |  |  |  |  |  |  |  |  |  |  |  |  |  |  |  |  |  |  |  |  |  |  |  |  |  |  |  |  |  |  |  |  |  |  |  |  |  |  |  |  |  |  |  |  |  |  |  |  |  |  |  |  |  |  |  |  |  |  |  |  |  |  |  |  |  |  |  |  |  |  |  |  |  |  |  |  |  |  |  |  |  |  |  |  |  |  |  |  |  |  |  |  |  |  |  |  |  |  |  |  |  |  |  |  |  |  |  |  |  |  |  |  |  |  |  |  |  |  |  |  |  |  |  |  |  |  |  |  |  |  |  |  |  |  |  |  |  |  |  |  |  |  |  |  |  |  |  |  |  |  |  |  |  |  |  |  |  |  |  |  |  |  |  |  |  |  |  |  |  |  |  |  |  |  |  |  |  |  |  |  |  |  |  |  |  |  |  |  |  |  |  |  |  |  |  |  |  |  |  |  |  |  |  |  |  |  |  |  |  |  |  |  |  |  |  |  |  |  |  |  |  |  |  |  |  |  |  |  |  |  |  |  |  |  |  |  |  |  |  |  |  |  |  |  |  |  |  |  |  |  |  |  |  |  |  |  |  |  |  |  |  |  |  |  |  |  |  |  |  |  |  |  |  |  |  |  |  |  |  |  |  |  |  |  |  |  |  |  |  |  |  |  |  |  |  |  |  |  |  |  |  |  |  |  |  |  |  |  |  |  |  |  |  |  |  |  |  |  |  |  |  |  |  |  |  |  |  |  |  |  |  |  |  |  |  |  |  |  |  |  |  |  |  |  |  |  |  |  |  |  |  |  |  |  |  |  |  |  |  |  |  |  |  |  |  |  |  |  |  |  |  |  |  |  |  |  |  |  |  |  |  |  |  |  |  |  |  |  |  |  |  |  |  |  |  |  |  |  |  |  |  |  |  |  |  |  |  |  |  |  |  |  |  |  |  |  |  |  |  |  |  |  |  |  |  |  |  |  |  |  |  |  |  |  |  |  |  |  |  |  |  |  |  |  |  |  |  |  |  |  |  |  |  |  |  |  |  |  |  |  |  |  |  |  |  |  |  |  |  |  |  |  |  |  |  |  |  |  |  |  |  |  |  |  |  |  |  |  |  |  |  |  |  |  |  |  |  |  |  |  |  |  |  |  |  |  |  |  |  |  |  |  |  |  |  |  |  |  |  |  |  |  |  |  |  |  |  |  |  |  |  |  |  |  |  |  |  |  |  |  |  |  |  |  |  |  |  |  |  |  |  |  |  |  |  |  |  |  |  |  |  |  |  |  |  |  |  |  |  |  |  |  |  |  |  |  |  |  |  |  |  |  |  |  |  |  |  |  |  |  |  |  |  |  |  |  |  |  |  |  |  |  |  |  |  |  |  |  |  |  |  |  |  |  |  |  |  |  |  |  |  |  |  |  |  |  |  |  |  |  |  |  |  |  |  |  |  |  |  |  |  |  |  |  |  |  |  |  |  |  |  |  |  |  |  |  |  |  |  |  |  |  |  |  |  |  |  |  |  |  |  |  |  |  |  |  |  |  |  |  |  |  |  |  |  |  |  |  |  |  |  |  |  |  |  |  |  |  |  |  |  |  |  |  |  |  |  |  |  |  |  |  |  |  |  |  |  |  |  |  |  |  |  |  |  |  |  |  |  |  |  |  |  |  |  |  |  |  |  |  |  |  |  |  |  |  |  |  |  |  |  |  |  |  |  |  |  |  |  |  |  |  |  |  |  |  |  |  |  |  |  |  |  |  |  |  |  |  |  |  |  |  |  |  |  |  |  |  |  |  |  |  |  |  |  |  |  |  |  |  |  |  |  |  |  |  |  |  |  |  |  |  |  |  |  |  |  |  |  |  |  |  |  |  |  |  |  |  |  |  |  |  |  |  |  |  |  |  |  |  |  |  |  |  |  |  |  |  |  |  |  |  |  |  |  |  |  |  |  |  |  |  |  |  |  |  |  |  |  |  |  |  |  |  |  |  |  |  |  |  |  |  |  |  |  |  |  |  |  |  |  |  |  |  |  |  |  |  |  |  |  |  |  |  |  |  |  |  |  |  |  |  |  |  |  |  |  |  |  |  |  |  |  |  |  |  |  |  |  |  |  |  |  |  |  |  |  |  |  |  |  |  |  |  |  |  |  |  |  |  |  |  |  |  |  |  |  |  |  |  |  |  |  |  |  |  |  |  |  |  |  |  |  |  |  |  |  |  |  |  |  |  |  |  |  |  |  |  |  |  |  |  |  |  |  |  |  |  |  |  |  |  |  |  |  |  |  |  |  |  |  |  |  |  |  |  |  |  |  |  |  |  |  |  |  |  |  |  |  |  |  |  |  |  |  |  |  |  |  |  |  |  |  |  |  |  |  |  |  |  |  |  |  |  |  |  |  |  |  |  |  |  |  |  |  |  |  |  |  |  |  |  |  |  |  |  |  |  |  |  |  |  |  |  |  |  |  |  |  |  |  |  |  |  |  |  |  |  |  |  |  |  |  |  |  |  |  |  |  |  |  |  |  |  |  |  |  |  |  |  |  |  |  |  |  |  |  |  |  |  |  |  |  |  |  |  |  |  |  |  |  |  |  |  |  |  |  |  |  |  |  |  |  |  |  |  |  |  |  |  |  |  |  |  |  |  |  |  |  |  |  |  |  |  |  |  |  |  |  |  |  |  |  |  |  |  |  |  |  |  |  |  |  |  |  |  |  |  |  |  |  |  |  |  |  |  |  |  |  |  |  |  |  |  |  |  |  |  |  |  |  |  |  |  |  |  |  |  |  |  |  |  |  |  |  |  |  |  |  |  |  |  |  |  |  |  |  |  |  |  |  |  |  |  |  |  |  |  |  |  |  |  |  |  |  |  |  |  |  |  |  |  |  |  |  |  |  |  |  |  |  |  |  |  |  |  |  |  |  |  |  |  |  |  |  |  |  |  |  |  |  |  |  |  |  |  |  |  |  |  |  |  |  |  |  |  |  |  |  |  |  |  |  |  |  |  |  |  |  |  |  |  |  |  |  |  |  |  |  |  |  |  |  |  |  |  |  |  |  |  |  |  |  |  |  |  |  |  |  |  |  |  |  |  |  |  |  |  |  |  |  |  |  |  |  |  |  |  |  |  |  |  |  |  |  |  |  |  |  |  |  |  |  |  |  |  |  |  |  |  |  |  |  |  |  |  |  |  |  |  |  |  |  |  |  |  |  |  |  |  |  |  |  |  |  |  |  |  |  |  |  |  |  |  |  |  |  |  |  |  |  |  |  |  |  |  |  |  |  |  |  |  |  |  |  |  |  |  |  |  |  |  |  |  |  |  |  |  |  |  |  |  |  |  |  |  |  |  |  |  |  |  |  |  |  |  |  |  |  |  |  |  |  |  |  |  |  |  |  |  |  |  |  |  |  |  |  |  |  |  |  |  |  |  |  |  |  |  |  |  |  |  |  |  |  |  |  |  |  |  |  |  |  |  |  |  |  |  |  |  |  |  |  |  |  |  |  |  |  |  |  |  |  |  |  |  |  |  |  |  |  |  |  |  |  |  |  |  |  |  |  |  |  |  |  |  |  |  |  |  |  |  |  |  |  |  |  |  |  |  |  |  |  |  |  |  |  |  |  |  |  |  |  |  |  |  |  |  |  |  |  |  |  |  |  |  |  |  |  |  |  |  |  |  |  |  |  |  |  |  |  |  |  |  |  |  |  |  |  |  |  |  |  |  |  |  |  |  |  |  |  |  |  |  |  |  |  |  |  |  |  |  |  |  |  |  |  |  |  |  |  |  |  |  |  |  |  |  |  |  |  |  |  |  |  |  |  |  |  |  |  |  |  |  |  |  |  |  |  |  |  |  |  |  |  |  |  |  |  |  |  |  |  |  |  |  |  |  |  |  |  |  |  |  |  |  |  |  |  |  |  |  |  |  |  |  |  |  |  |  |  |  |  |  |  |  |  |  |  |  |  |  |  |  |  |  |  |  |  |  |  |  |  |  |  |  |  |  |  |  |  |  |  |  |  |  |  |  |  |  |  |  |  |  |  |  |  |  |  |  |  |  |  |  |  |  |  |  |  |  |  |  |  |  |  |  |  |  |  |  |  |  |  |  |  |  |  |  |  |  |  |  |  |  |  |  |  |  |  |  |  |  |  |  |  |  |  |  |  |  |  |  |  |  |  |  |  |  |  |  |  |  |  |  |  |  |  |  |  |  |  |  |  |  |  |  |  |  |  |  |  |  |  |  |  |  |  |  |  |  |  |  |  |  |  |  |  |  |  |  |  |  |  |  |  |  |  |  |  |  |  |  |  |  |  |  |  |  |  |  |  |  |  |  |  |  |  |  |  |  |  |  |  |  |  |  |  |  |  |  |  |  |  |  |  |  |  |  |  |  |  |  |  |  |  |  |  |  |  |  |  |  |  |  |  |  |  |  |  |  |  |  |  |  |  |  |  |  |  |  |  |  |  |  |  |  |  |  |  |  |  |  |  |  |  |  |  |  |  |  |  |  |  |  |  |  |  |  |  |  |  |  |  |  |  |  |  |  |  |  |  |  |  |  |  |  |  |  |  |  |  |  |  |  |  |  |  |  |  |  |  |  |  |  |  |  |  |  |  |  |  |  |  |  |  |  |  |  |  |  |  |  |  |  |  |  |  |  |  |  |  |  |  |  |  |  |  |  |  |  |  |  |  |  |  |  |  |  |  |  |  |  |  |  |  |  |  |  |  |  |  |  |  |  |  |  |  |  |  |  |  |  |  |  |  |  |  |  |  |  |  |  |  |  |  |  |  |  |  |  |  |  |  |  |  |  |  |  |  |  |  |  |  |  |  |  |  |  |  |  |  |  |  |  |  |  |  |  |  |  |  |  |  |  |  |  |  |  |  |  |  |  |  |  |  |  |  |  |  |  |  |  |  |  |  |  |  |  |  |  |  |  |  |  |  |  |  |  |  |  |  |  |  |  |  |  |  |  |  |  |  |  |  |  |  |  |  |  |  |  |  |  |  |  |  |  |  |  |  |  |  |  |  |  |  |  |  |  |  |  |  |  |  |  |  |  |  |  |  |  |  |  |  |  |  |  |  |  |  |  |  |  |  |  |  |  |  |  |  |  |  |  |  |  |  |  |  |  |  |  |  |  |  |  |  |  |  |  |  |  |  |  |  |  |  |  |  |  |  |  |  |  |  |  |  |  |  |  |  |  |  |  |  |  |  |  |  |  |  |  |  |  |  |  |  |  |  |  |  |  |  |  |  |  |  |  |  |  |  |  |  |  |  |  |  |  |  |  |  |  |  |  |  |  |  |  |  |  |  |  |  |  |  |  |  |  |  |  |  |  |  |  |  |
| --- | --- | --- | --- | --- | --- | --- | --- | --- | --- | --- | --- | --- | --- | --- | --- | --- | --- | --- | --- | --- | --- | --- | --- | --- | --- | --- | --- | --- | --- | --- | --- | --- | --- | --- | --- | --- | --- | --- | --- | --- | --- | --- | --- | --- | --- | --- | --- | --- | --- | --- | --- | --- | --- | --- | --- | --- | --- | --- | --- | --- | --- | --- | --- | --- | --- | --- | --- | --- | --- | --- | --- | --- | --- | --- | --- | --- | --- | --- | --- | --- | --- | --- | --- | --- | --- | --- | --- | --- | --- | --- | --- | --- | --- | --- | --- | --- | --- | --- | --- | --- | --- | --- | --- | --- | --- | --- | --- | --- | --- | --- | --- | --- | --- | --- | --- | --- | --- | --- | --- | --- | --- | --- | --- | --- | --- | --- | --- | --- | --- | --- | --- | --- | --- | --- | --- | --- | --- | --- | --- | --- | --- | --- | --- | --- | --- | --- | --- | --- | --- | --- | --- | --- | --- | --- | --- | --- | --- | --- | --- | --- | --- | --- | --- | --- | --- | --- | --- | --- | --- | --- | --- | --- | --- | --- | --- | --- | --- | --- | --- | --- | --- | --- | --- | --- | --- | --- | --- | --- | --- | --- | --- | --- | --- | --- | --- | --- | --- | --- | --- | --- | --- | --- | --- | --- | --- | --- | --- | --- | --- | --- | --- | --- | --- | --- | --- | --- | --- | --- | --- | --- | --- | --- | --- | --- | --- | --- | --- | --- | --- | --- | --- | --- | --- | --- | --- | --- | --- | --- | --- | --- | --- | --- | --- | --- | --- | --- | --- | --- | --- | --- | --- | --- | --- | --- | --- | --- | --- | --- | --- | --- | --- | --- | --- | --- | --- | --- | --- | --- | --- | --- | --- | --- | --- | --- | --- | --- | --- | --- | --- | --- | --- | --- | --- | --- | --- | --- | --- | --- | --- | --- | --- | --- | --- | --- | --- | --- | --- | --- | --- | --- | --- | --- | --- | --- | --- | --- | --- | --- | --- | --- | --- | --- | --- | --- | --- | --- | --- | --- | --- | --- | --- | --- | --- | --- | --- | --- | --- | --- | --- | --- | --- | --- | --- | --- | --- | --- | --- | --- | --- | --- | --- | --- | --- | --- | --- | --- | --- | --- | --- | --- | --- | --- | --- | --- | --- | --- | --- | --- | --- | --- | --- | --- | --- | --- | --- | --- | --- | --- | --- | --- | --- | --- | --- | --- | --- | --- | --- | --- | --- | --- | --- | --- | --- | --- | --- | --- | --- | --- | --- | --- | --- | --- | --- | --- | --- | --- | --- | --- | --- | --- | --- | --- | --- | --- | --- | --- | --- | --- | --- | --- | --- | --- | --- | --- | --- | --- | --- | --- | --- | --- | --- | --- | --- | --- | --- | --- | --- | --- | --- | --- | --- | --- | --- | --- | --- | --- | --- | --- | --- | --- | --- | --- | --- | --- | --- | --- | --- | --- | --- | --- | --- | --- | --- | --- | --- | --- | --- | --- | --- | --- | --- | --- | --- | --- | --- | --- | --- | --- | --- | --- | --- | --- | --- | --- | --- | --- | --- | --- | --- | --- | --- | --- | --- | --- | --- | --- | --- | --- | --- | --- | --- | --- | --- | --- | --- | --- | --- | --- | --- | --- | --- | --- | --- | --- | --- | --- | --- | --- | --- | --- | --- | --- | --- | --- | --- | --- | --- | --- | --- | --- | --- | --- | --- | --- | --- | --- | --- | --- | --- | --- | --- | --- | --- | --- | --- | --- | --- | --- | --- | --- | --- | --- | --- | --- | --- | --- | --- | --- | --- | --- | --- | --- | --- | --- | --- | --- | --- | --- | --- | --- | --- | --- | --- | --- | --- | --- | --- | --- | --- | --- | --- | --- | --- | --- | --- | --- | --- | --- | --- | --- | --- | --- | --- | --- | --- | --- | --- | --- | --- | --- | --- | --- | --- | --- | --- | --- | --- | --- | --- | --- | --- | --- | --- | --- | --- | --- | --- | --- | --- | --- | --- | --- | --- | --- | --- | --- | --- | --- | --- | --- | --- | --- | --- | --- | --- | --- | --- | --- | --- | --- | --- | --- | --- | --- | --- | --- | --- | --- | --- | --- | --- | --- | --- | --- | --- | --- | --- | --- | --- | --- | --- | --- | --- | --- | --- | --- | --- | --- | --- | --- | --- | --- | --- | --- | --- | --- | --- | --- | --- | --- | --- | --- | --- | --- | --- | --- | --- | --- | --- | --- | --- | --- | --- | --- | --- | --- | --- | --- | --- | --- | --- | --- | --- | --- | --- | --- | --- | --- | --- | --- | --- | --- | --- | --- | --- | --- | --- | --- | --- | --- | --- | --- | --- | --- | --- | --- | --- | --- | --- | --- | --- | --- | --- | --- | --- | --- | --- | --- | --- | --- | --- | --- | --- | --- | --- | --- | --- | --- | --- | --- | --- | --- | --- | --- | --- | --- | --- | --- | --- | --- | --- | --- | --- | --- | --- | --- | --- | --- | --- | --- | --- | --- | --- | --- | --- | --- | --- | --- | --- | --- | --- | --- | --- | --- | --- | --- | --- | --- | --- | --- | --- | --- | --- | --- | --- | --- | --- | --- | --- | --- | --- | --- | --- | --- | --- | --- | --- | --- | --- | --- | --- | --- | --- | --- | --- | --- | --- | --- | --- | --- | --- | --- | --- | --- | --- | --- | --- | --- | --- | --- | --- | --- | --- | --- | --- | --- | --- | --- | --- | --- | --- | --- | --- | --- | --- | --- | --- | --- | --- | --- | --- | --- | --- | --- | --- | --- | --- | --- | --- | --- | --- | --- | --- | --- | --- | --- | --- | --- | --- | --- | --- | --- | --- | --- | --- | --- | --- | --- | --- | --- | --- | --- | --- | --- | --- | --- | --- | --- | --- | --- | --- | --- | --- | --- | --- | --- | --- | --- | --- | --- | --- | --- | --- | --- | --- | --- | --- | --- | --- | --- | --- | --- | --- | --- | --- | --- | --- | --- | --- | --- | --- | --- | --- | --- | --- | --- | --- | --- | --- | --- | --- | --- | --- | --- | --- | --- | --- | --- | --- | --- | --- | --- | --- | --- | --- | --- | --- | --- | --- | --- | --- | --- | --- | --- | --- | --- | --- | --- | --- | --- | --- | --- | --- | --- | --- | --- | --- | --- | --- | --- | --- | --- | --- | --- | --- | --- | --- | --- | --- | --- | --- | --- | --- | --- | --- | --- | --- | --- | --- | --- | --- | --- | --- | --- | --- | --- | --- | --- | --- | --- | --- | --- | --- | --- | --- | --- | --- | --- | --- | --- | --- | --- | --- | --- | --- | --- | --- | --- | --- | --- | --- | --- | --- | --- | --- | --- | --- | --- | --- | --- | --- | --- | --- | --- | --- | --- | --- | --- | --- | --- | --- | --- | --- | --- | --- | --- | --- | --- | --- | --- | --- | --- | --- | --- | --- | --- | --- | --- | --- | --- | --- | --- | --- | --- | --- | --- | --- | --- | --- | --- | --- | --- | --- | --- | --- | --- | --- | --- | --- | --- | --- | --- | --- | --- | --- | --- | --- | --- | --- | --- | --- | --- | --- | --- | --- | --- | --- | --- | --- | --- | --- | --- | --- | --- | --- | --- | --- | --- | --- | --- | --- | --- | --- | --- | --- | --- | --- | --- | --- | --- | --- | --- | --- | --- | --- | --- | --- | --- | --- | --- | --- | --- | --- | --- | --- | --- | --- | --- | --- | --- | --- | --- | --- | --- | --- | --- | --- | --- | --- | --- | --- | --- | --- | --- | --- | --- | --- | --- | --- | --- | --- | --- | --- | --- | --- | --- | --- | --- | --- | --- | --- | --- | --- | --- | --- | --- | --- | --- | --- | --- | --- | --- | --- | --- | --- | --- | --- | --- | --- | --- | --- | --- | --- | --- | --- | --- | --- | --- | --- | --- | --- | --- | --- | --- | --- | --- | --- | --- | --- | --- | --- | --- | --- | --- | --- | --- | --- | --- | --- | --- | --- | --- | --- | --- | --- | --- | --- | --- | --- | --- | --- | --- | --- | --- | --- | --- | --- | --- | --- | --- | --- | --- | --- | --- | --- | --- | --- | --- | --- | --- | --- | --- | --- | --- | --- | --- | --- | --- | --- | --- | --- | --- | --- | --- | --- | --- | --- | --- | --- | --- | --- | --- | --- | --- | --- | --- | --- | --- | --- | --- | --- | --- | --- | --- | --- | --- | --- | --- | --- | --- | --- | --- | --- | --- | --- | --- | --- | --- | --- | --- | --- | --- | --- | --- | --- | --- | --- | --- | --- | --- | --- | --- | --- | --- | --- | --- | --- | --- | --- | --- | --- | --- | --- | --- | --- | --- | --- | --- | --- | --- | --- | --- | --- | --- | --- | --- | --- | --- | --- | --- | --- | --- | --- | --- | --- | --- | --- | --- | --- | --- | --- | --- | --- | --- | --- | --- | --- | --- | --- | --- | --- | --- | --- | --- | --- | --- | --- | --- | --- | --- | --- | --- | --- | --- | --- | --- | --- | --- | --- | --- | --- | --- | --- | --- | --- | --- | --- | --- | --- | --- | --- | --- | --- | --- | --- | --- | --- | --- | --- | --- | --- | --- | --- | --- | --- | --- | --- | --- | --- | --- | --- | --- | --- | --- | --- | --- | --- | --- | --- | --- | --- | --- | --- | --- | --- | --- | --- | --- | --- | --- | --- | --- | --- | --- | --- | --- | --- | --- | --- | --- | --- | --- | --- | --- | --- | --- | --- | --- | --- | --- | --- | --- | --- | --- | --- | --- | --- | --- | --- | --- | --- | --- | --- | --- | --- | --- | --- | --- | --- | --- | --- | --- | --- | --- | --- | --- | --- | --- | --- | --- | --- | --- | --- | --- | --- | --- | --- | --- | --- | --- | --- | --- | --- | --- | --- | --- | --- | --- | --- | --- | --- | --- | --- | --- | --- | --- | --- | --- | --- | --- | --- | --- | --- | --- | --- | --- | --- | --- | --- | --- | --- | --- | --- | --- | --- | --- | --- | --- | --- | --- | --- | --- | --- | --- | --- | --- | --- | --- | --- | --- | --- | --- | --- | --- | --- | --- | --- | --- | --- | --- | --- | --- | --- | --- | --- | --- | --- | --- | --- | --- | --- | --- | --- | --- | --- | --- | --- | --- | --- | --- | --- | --- | --- | --- | --- | --- | --- | --- | --- | --- | --- | --- | --- | --- | --- | --- | --- | --- | --- | --- | --- | --- | --- | --- | --- | --- | --- | --- | --- | --- | --- | --- | --- | --- | --- | --- | --- | --- | --- | --- | --- | --- | --- | --- | --- | --- | --- | --- | --- | --- | --- | --- | --- | --- | --- | --- | --- | --- | --- | --- | --- | --- | --- | --- | --- | --- | --- | --- | --- | --- | --- | --- | --- | --- | --- | --- | --- | --- | --- | --- | --- | --- | --- | --- | --- | --- | --- | --- | --- | --- | --- | --- | --- | --- | --- | --- | --- | --- | --- | --- | --- | --- | --- | --- | --- | --- | --- | --- | --- | --- | --- | --- | --- | --- | --- | --- | --- | --- | --- | --- | --- | --- | --- | --- | --- | --- | --- | --- | --- | --- | --- | --- | --- | --- | --- | --- | --- | --- | --- | --- | --- | --- | --- | --- | --- | --- | --- | --- | --- | --- | --- | --- | --- | --- | --- | --- | --- | --- | --- | --- | --- | --- | --- | --- | --- | --- | --- | --- | --- | --- | --- | --- | --- | --- | --- | --- | --- | --- | --- | --- | --- | --- | --- | --- | --- | --- | --- | --- | --- | --- | --- | --- | --- | --- | --- | --- | --- | --- | --- | --- | --- | --- | --- | --- | --- | --- | --- | --- | --- | --- | --- | --- | --- | --- | --- | --- | --- | --- | --- | --- | --- | --- | --- | --- | --- | --- | --- | --- | --- | --- | --- | --- | --- | --- | --- | --- | --- | --- | --- | --- | --- | --- | --- | --- | --- | --- | --- | --- | --- | --- | --- | --- | --- | --- | --- | --- | --- | --- | --- | --- | --- | --- | --- | --- | --- | --- | --- | --- | --- | --- | --- | --- | --- | --- | --- | --- | --- | --- | --- | --- | --- | --- | --- | --- | --- | --- | --- | --- | --- | --- | --- | --- | --- | --- | --- | --- | --- | --- | --- | --- | --- | --- | --- | --- | --- | --- | --- | --- | --- | --- | --- | --- | --- | --- | --- | --- | --- | --- | --- | --- | --- | --- | --- | --- | --- | --- | --- | --- | --- | --- | --- | --- | --- | --- | --- | --- | --- | --- | --- | --- | --- | --- | --- | --- | --- | --- | --- | --- | --- | --- | --- | --- | --- | --- | --- | --- | --- | --- | --- | --- | --- | --- | --- | --- | --- | --- | --- | --- | --- | --- | --- | --- | --- | --- | --- | --- | --- | --- | --- | --- | --- | --- | --- | --- | --- | --- | --- | --- | --- | --- | --- | --- | --- | --- | --- | --- | --- | --- | --- | --- | --- | --- | --- | --- | --- | --- | --- | --- | --- | --- | --- | --- | --- | --- | --- | --- | --- | --- | --- | --- | --- | --- | --- | --- | --- | --- | --- | --- | --- | --- | --- | --- | --- | --- | --- | --- | --- | --- | --- | --- | --- | --- | --- | --- | --- | --- | --- | --- | --- | --- | --- | --- | --- | --- | --- | --- | --- | --- | --- | --- | --- | --- | --- | --- | --- | --- | --- | --- | --- | --- | --- | --- | --- | --- | --- | --- | --- | --- | --- | --- | --- | --- | --- | --- | --- | --- | --- | --- | --- | --- | --- | --- | --- | --- | --- | --- | --- | --- | --- | --- | --- | --- | --- | --- | --- | --- | --- | --- | --- | --- | --- | --- | --- | --- | --- | --- | --- | --- | --- | --- | --- | --- | --- | --- | --- | --- | --- | --- | --- | --- | --- | --- | --- | --- | --- | --- | --- | --- | --- | --- | --- | --- | --- | --- | --- | --- | --- | --- | --- | --- | --- | --- | --- | --- | --- | --- | --- | --- | --- | --- | --- | --- | --- | --- | --- | --- | --- | --- | --- | --- | --- | --- | --- | --- | --- | --- | --- | --- | --- | --- | --- | --- | --- | --- | --- | --- | --- | --- | --- | --- | --- | --- | --- | --- | --- | --- | --- | --- | --- | --- | --- | --- | --- | --- | --- | --- | --- | --- | --- | --- | --- | --- | --- | --- | --- | --- | --- | --- | --- | --- | --- | --- | --- | --- | --- | --- | --- | --- | --- | --- | --- | --- | --- | --- | --- | --- | --- | --- | --- | --- | --- | --- | --- | --- | --- | --- | --- | --- | --- | --- | --- | --- | --- | --- | --- | --- | --- | --- | --- | --- | --- | --- | --- | --- | --- | --- | --- | --- | --- | --- | --- | --- | --- | --- | --- | --- | --- | --- | --- | --- | --- | --- | --- | --- | --- | --- | --- | --- | --- | --- | --- | --- | --- | --- | --- | --- | --- | --- | --- | --- | --- | --- | --- | --- | --- | --- | --- | --- | --- | --- | --- | --- | --- | --- | --- | --- | --- | --- | --- | --- | --- | --- | --- | --- | --- | --- | --- | --- | --- | --- | --- | --- | --- | --- | --- | --- | --- | --- | --- | --- | --- | --- | --- | --- | --- | --- | --- | --- | --- | --- | --- | --- | --- | --- | --- | --- | --- | --- | --- | --- | --- | --- | --- | --- | --- | --- | --- | --- | --- | --- | --- | --- | --- | --- | --- | --- | --- | --- | --- | --- | --- | --- | --- | --- | --- | --- | --- | --- | --- | --- | --- | --- | --- | --- | --- | --- | --- | --- | --- | --- | --- | --- | --- | --- | --- | --- | --- | --- | --- | --- | --- | --- | --- | --- | --- | --- | --- | --- | --- | --- | --- | --- | --- | --- | --- | --- | --- | --- | --- | --- | --- | --- | --- | --- | --- | --- | --- | --- | --- | --- | --- | --- | --- | --- | --- | --- | --- | --- | --- | --- | --- | --- | --- | --- | --- | --- | --- | --- | --- | --- | --- | --- | --- | --- | --- | --- | --- | --- | --- | --- | --- | --- | --- | --- | --- | --- | --- | --- | --- | --- | --- | --- | --- | --- | --- | --- | --- | --- | --- | --- | --- | --- | --- | --- | --- | --- | --- | --- | --- | --- | --- | --- | --- | --- | --- | --- | --- | --- | --- | --- | --- | --- | --- | --- | --- | --- | --- | --- | --- | --- | --- | --- | --- | --- | --- | --- | --- | --- | --- | --- | --- | --- | --- | --- | --- | --- | --- | --- | --- | --- | --- | --- | --- | --- | --- | --- | --- | --- | --- | --- | --- | --- | --- | --- | --- | --- | --- | --- | --- | --- | --- | --- | --- | --- | --- | --- | --- | --- | --- | --- | --- | --- | --- | --- | --- | --- | --- | --- | --- | --- | --- | --- | --- | --- | --- | --- | --- | --- | --- | --- | --- | --- | --- | --- | --- | --- | --- | --- | --- | --- | --- | --- | --- | --- | --- | --- | --- | --- | --- | --- | --- | --- | --- | --- | --- | --- | --- | --- | --- | --- | --- | --- | --- | --- | --- | --- | --- | --- | --- | --- | --- | --- | --- | --- | --- | --- | --- | --- | --- | --- | --- | --- | --- | --- | --- | --- | --- | --- | --- | --- | --- | --- | --- | --- | --- | --- | --- | --- | --- | --- | --- | --- | --- | --- | --- | --- | --- | --- | --- | --- | --- | --- | --- | --- | --- | --- | --- | --- | --- | --- | --- | --- | --- | --- | --- | --- | --- | --- | --- | --- | --- | --- | --- | --- | --- | --- | --- | --- | --- | --- | --- | --- | --- | --- | --- | --- | --- | --- | --- | --- | --- | --- | --- | --- | --- | --- | --- | --- | --- | --- | --- | --- | --- | --- | --- | --- | --- | --- | --- | --- | --- | --- | --- | --- | --- | --- | --- | --- | --- | --- | --- | --- | --- | --- | --- | --- | --- | --- | --- | --- | --- | --- | --- | --- | --- | --- | --- | --- | --- | --- | --- | --- | --- | --- | --- | --- | --- | --- | --- | --- | --- | --- | --- | --- | --- | --- | --- | --- | --- | --- | --- | --- | --- | --- | --- | --- | --- | --- | --- | --- | --- | --- | --- | --- | --- | --- | --- | --- | --- | --- | --- | --- | --- | --- | --- | --- | --- | --- | --- | --- | --- | --- | --- | --- | --- | --- | --- | --- | --- | --- | --- | --- | --- | --- | --- | --- | --- | --- | --- | --- | --- | --- | --- | --- | --- | --- | --- | --- | --- | --- | --- | --- | --- | --- | --- | --- | --- | --- | --- | --- | --- | --- | --- | --- | --- | --- | --- | --- | --- | --- | --- | --- | --- | --- | --- | --- | --- | --- | --- | --- | --- | --- | --- | --- | --- | --- | --- | --- | --- | --- | --- | --- | --- | --- | --- | --- | --- | --- | --- | --- | --- | --- | --- | --- | --- | --- | --- | --- | --- | --- | --- | --- | --- | --- | --- | --- | --- | --- | --- | --- | --- | --- | --- | --- | --- | --- | --- | --- | --- | --- | --- | --- | --- | --- | --- | --- | --- | --- | --- | --- | --- | --- | --- | --- | --- | --- | --- | --- | --- | --- | --- | --- | --- | --- | --- | --- | --- | --- | --- | --- | --- | --- | --- | --- | --- | --- | --- | --- | --- | --- | --- | --- | --- | --- | --- | --- | --- | --- | --- | --- | --- | --- | --- | --- | --- | --- | --- | --- | --- | --- | --- | --- | --- | --- | --- | --- | --- | --- | --- | --- | --- | --- | --- | --- | --- | --- | --- | --- | --- | --- | --- | --- | --- | --- | --- | --- | --- | --- | --- | --- | --- | --- | --- | --- | --- | --- | --- | --- | --- | --- | --- | --- | --- | --- | --- | --- | --- | --- | --- | --- | --- | --- | --- | --- | --- | --- | --- | --- | --- | --- | --- | --- | --- | --- | --- | --- | --- | --- | --- | --- | --- | --- | --- | --- | --- | --- | --- | --- | --- | --- | --- | --- | --- | --- | --- | --- | --- | --- | --- | --- | --- | --- | --- | --- | --- | --- | --- | --- | --- | --- | --- | --- | --- | --- | --- | --- | --- | --- | --- | --- | --- | --- | --- | --- | --- | --- | --- | --- | --- | --- | --- | --- | --- | --- | --- | --- | --- | --- | --- | --- | --- | --- | --- | --- | --- | --- | --- | --- | --- | --- | --- | --- | --- | --- | --- | --- | --- | --- | --- | --- | --- | --- | --- | --- | --- | --- | --- | --- | --- | --- | --- | --- | --- | --- | --- | --- | --- | --- | --- | --- | --- | --- | --- | --- | --- | --- | --- | --- | --- | --- | --- | --- | --- | --- | --- | --- | --- | --- | --- | --- | --- | --- | --- | --- | --- | --- | --- | --- | --- | --- | --- | --- | --- | --- | --- | --- | --- | --- | --- | --- | --- | --- | --- | --- | --- | --- | --- | --- | --- | --- | --- | --- | --- | --- | --- | --- | --- | --- | --- | --- | --- | --- | --- | --- | --- | --- | --- | --- | --- | --- | --- | --- | --- | --- | --- | --- | --- | --- | --- | --- | --- | --- | --- | --- | --- | --- | --- | --- | --- | --- | --- | --- | --- | --- | --- | --- | --- | --- | --- | --- | --- | --- | --- | --- | --- | --- | --- | --- | --- | --- | --- | --- | --- | --- | --- | --- | --- | --- | --- | --- | --- | --- | --- | --- | --- | --- | --- | --- | --- | --- | --- | --- | --- | --- | --- | --- | --- | --- | --- | --- | --- | --- | --- | --- | --- | --- | --- | --- | --- | --- | --- | --- | --- | --- | --- | --- | --- | --- | --- | --- | --- | --- | --- | --- | --- | --- | --- | --- | --- | --- | --- | --- | --- | --- | --- | --- | --- | --- | --- | --- | --- | --- | --- | --- | --- | --- | --- | --- | --- | --- | --- | --- | --- | --- | --- | --- | --- | --- | --- | --- | --- | --- | --- | --- | --- | --- | --- | --- | --- | --- | --- | --- | --- | --- | --- | --- | --- | --- | --- | --- | --- | --- | --- | --- | --- | --- | --- | --- | --- | --- | --- | --- | --- | --- | --- | --- | --- | --- | --- | --- | --- | --- | --- | --- | --- | --- | --- | --- | --- | --- | --- | --- | --- | --- | --- | --- | --- | --- | --- | --- | --- | --- | --- | --- | --- | --- | --- | --- | --- | --- | --- | --- | --- | --- | --- | --- | --- | --- | --- | --- | --- | --- | --- | --- | --- | --- | --- | --- | --- | --- | --- | --- | --- | --- | --- | --- | --- | --- | --- | --- | --- | --- | --- | --- | --- | --- | --- | --- | --- | --- | --- | --- | --- | --- | --- | --- | --- | --- | --- | --- | --- | --- | --- | --- | --- | --- | --- | --- | --- | --- | --- | --- | --- | --- | --- | --- | --- | --- | --- | --- | --- | --- | --- | --- | --- | --- | --- | --- | --- | --- | --- | --- | --- | --- | --- | --- | --- | --- | --- | --- | --- | --- | --- | --- | --- | --- | --- | --- | --- | --- | --- | --- | --- | --- | --- | --- | --- | --- | --- | --- | --- | --- | --- | --- | --- | --- | --- | --- | --- | --- | --- | --- | --- | --- | --- | --- | --- | --- | --- | --- | --- | --- | --- | --- | --- | --- | --- | --- | --- | --- | --- | --- | --- | --- | --- | --- | --- | --- | --- | --- | --- | --- | --- | --- | --- | --- | --- | --- | --- | --- | --- | --- | --- | --- | --- | --- | --- | --- | --- | --- | --- | --- | --- | --- | --- | --- | --- | --- | --- | --- | --- | --- | --- | --- | --- | --- | --- | --- | --- | --- | --- | --- | --- | --- | --- | --- | --- | --- | --- | --- | --- | --- | --- | --- | --- | --- | --- | --- | --- | --- | --- | --- | --- | --- | --- | --- | --- | --- | --- | --- | --- | --- | --- | --- | --- | --- | --- | --- | --- | --- | --- | --- | --- | --- | --- | --- | --- | --- | --- | --- | --- | --- | --- | --- | --- | --- | --- | --- | --- | --- | --- | --- | --- | --- | --- | --- | --- | --- | --- | --- | --- | --- | --- | --- | --- | --- | --- | --- | --- | --- | --- | --- | --- | --- | --- | --- | --- | --- | --- | --- | --- | --- | --- | --- | --- | --- | --- | --- | --- | --- | --- | --- | --- | --- | --- | --- | --- | --- | --- | --- | --- | --- | --- | --- | --- | --- | --- | --- | --- | --- | --- | --- | --- | --- | --- | --- | --- | --- | --- | --- | --- | --- | --- | --- | --- | --- | --- | --- | --- | --- | --- | --- | --- | --- | --- | --- | --- | --- | --- | --- | --- | --- | --- | --- | --- | --- | --- | --- | --- | --- | --- | --- | --- | --- | --- | --- | --- | --- | --- | --- | --- | --- | --- | --- | --- | --- | --- | --- | --- | --- | --- | --- | --- | --- | --- | --- | --- | --- | --- | --- | --- | --- | --- | --- | --- | --- | --- | --- | --- | --- | --- | --- | --- | --- | --- | --- | --- | --- | --- | --- | --- | --- | --- | --- | --- | --- | --- | --- | --- | --- | --- | --- | --- | --- | --- | --- | --- | --- | --- | --- | --- | --- | --- | --- | --- | --- | --- | --- | --- | --- | --- | --- | --- | --- | --- | --- | --- | --- | --- | --- | --- | --- | --- | --- | --- | --- | --- | --- | --- | --- | --- | --- | --- | --- | --- | --- | --- | --- | --- | --- | --- | --- | --- | --- | --- | --- | --- | --- | --- | --- | --- | --- | --- | --- | --- | --- | --- | --- | --- | --- | --- | --- | --- | --- | --- | --- | --- | --- | --- | --- | --- | --- | --- | --- | --- | --- | --- | --- | --- | --- | --- | --- | --- | --- | --- | --- | --- | --- | --- | --- | --- | --- | --- | --- | --- | --- | --- | --- | --- | --- | --- | --- | --- | --- | --- | --- | --- | --- | --- | --- | --- | --- | --- | --- | --- | --- | --- | --- | --- | --- | --- | --- | --- | --- | --- | --- | --- | --- | --- | --- | --- | --- | --- | --- | --- | --- | --- | --- | --- | --- | --- | --- | --- | --- | --- | --- | --- | --- | --- | --- | --- | --- | --- | --- | --- | --- | --- | --- | --- | --- | --- | --- | --- | --- | --- | --- | --- | --- | --- | --- | --- | --- | --- | --- | --- | --- | --- | --- | --- | --- | --- | --- | --- | --- | --- | --- | --- | --- | --- | --- | --- | --- | --- | --- | --- | --- | --- | --- | --- | --- | --- | --- | --- | --- | --- | --- | --- | --- | --- | --- | --- | --- | --- | --- | --- | --- | --- | --- | --- | --- | --- | --- | --- | --- | --- | --- | --- | --- | --- | --- | --- | --- | --- | --- | --- | --- | --- | --- | --- | --- | --- | --- | --- | --- | --- | --- | --- | --- | --- | --- | --- | --- | --- | --- | --- | --- | --- | --- | --- | --- | --- | --- | --- | --- | --- | --- | --- | --- | --- | --- | --- | --- | --- | --- | --- | --- | --- | --- | --- | --- | --- | --- | --- | --- | --- | --- | --- | --- | --- | --- | --- | --- | --- | --- | --- | --- | --- | --- | --- | --- | --- | --- | --- | --- | --- | --- | --- | --- | --- | --- | --- | --- | --- | --- | --- | --- | --- | --- | --- | --- | --- | --- | --- | --- | --- | --- | --- | --- | --- | --- | --- | --- | --- | --- | --- | --- | --- | --- | --- | --- | --- | --- | --- | --- | --- | --- | --- | --- | --- | --- | --- | --- | --- | --- | --- | --- | --- | --- | --- | --- | --- | --- | --- | --- | --- | --- | --- | --- | --- | --- | --- | --- | --- | --- | --- | --- | --- | --- | --- | --- | --- | --- | --- | --- | --- | --- | --- | --- | --- | --- | --- | --- | --- | --- | --- | --- | --- | --- | --- | --- | --- | --- | --- | --- | --- | --- | --- | --- | --- | --- | --- | --- | --- | --- | --- | --- | --- | --- | --- | --- | --- | --- | --- | --- | --- | --- | --- | --- | --- | --- | --- | --- | --- | --- | --- | --- | --- | --- | --- | --- | --- | --- | --- | --- | --- | --- | --- | --- | --- | --- | --- | --- | --- | --- | --- | --- | --- | --- | --- | --- | --- | --- | --- | --- | --- | --- | --- | --- | --- | --- | --- | --- | --- | --- | --- | --- | --- | --- | --- | --- | --- | --- | --- | --- | --- | --- | --- | --- | --- | --- | --- | --- | --- | --- | --- | --- | --- | --- | --- | --- | --- | --- | --- | --- | --- | --- | --- | --- | --- | --- | --- | --- | --- | --- | --- | --- | --- | --- | --- | --- | --- | --- | --- | --- | --- | --- | --- | --- | --- | --- | --- | --- | --- | --- | --- | --- | --- | --- | --- | --- | --- | --- | --- | --- | --- | --- | --- | --- | --- | --- | --- | --- | --- | --- | --- | --- | --- | --- | --- | --- | --- | --- | --- | --- | --- | --- | --- | --- | --- | --- | --- | --- | --- | --- | --- | --- | --- | --- | --- | --- | --- | --- | --- | --- | --- | --- | --- | --- | --- | --- | --- | --- | --- | --- | --- | --- | --- | --- | --- | --- | --- | --- | --- | --- | --- | --- | --- | --- | --- | --- | --- | --- | --- | --- | --- | --- | --- | --- | --- | --- | --- | --- | --- | --- | --- | --- | --- | --- | --- | --- | --- | --- | --- | --- | --- | --- | --- | --- | --- | --- | --- | --- | --- | --- | --- | --- | --- | --- | --- | --- | --- | --- | --- | --- | --- | --- | --- | --- | --- | --- | --- | --- | --- | --- | --- | --- | --- | --- | --- | --- | --- | --- |
| |  |  |  |  |  |  |  |  |  |  |  |  |  |  |  |  |  |  |  |  |  |  |  |  |  |  |  |  |  |  |  |  |  |  |  |  |  |  |  |  |  |  |  |  |  |  |  |  |  |  |  |  |  |  |  |  |  |  | | --- | --- | --- | --- | --- | --- | --- | --- | --- | --- | --- | --- | --- | --- | --- | --- | --- | --- | --- | --- | --- | --- | --- | --- | --- | --- | --- | --- | --- | --- | --- | --- | --- | --- | --- | --- | --- | --- | --- | --- | --- | --- | --- | --- | --- | --- | --- | --- | --- | --- | --- | --- | --- | --- | --- | --- | --- | --- | | G0VKY8/1-331 | 1 | M | L | K | L | S | R | N | F | H | T | R | - | S | P | L | L | V | R | T | R | F | T | K | P | K | P | K | P | E | P | - | - | - | S | K | N | P | R | K | P | T | Q | T | S | H | H | L | N | T | L | K | V | T | A | P | 51 | | Q6CWC4/1-332 | 1 | M | F | P | A | R | R | G | L | H | T | T | - | S | R | A | C | A | R | T | R | F | T | K | P | K | P | K | P | A | K | - | - | - | R | E | N | V | R | L | P | T | Q | R | T | H | H | D | N | D | L | K | I | T | A | P | 51 | | Q6FK61/1-315 | 1 | M | - | - | L | R | R | Q | F | H | S | G | - | C | V | Q | L | A | R | T | R | Y | T | K | P | K | P | K | N | T | E | T | R | A | K | E | Q | I | R | L | P | T | Q | Q | T | H | H | S | N | E | L | R | I | Q | P | P | 52 | | Q756Y8/1-324 | 1 | M | W | V | F | Q | R | G | L | H | S | S | - | R | S | V | L | A | R | T | R | Y | T | K | P | K | P | K | P | P | R | - | - | - | R | S | K | V | R | A | P | T | Q | T | T | H | H | D | T | D | L | K | V | T | A | P | 51 | | A7TNQ2/1-322 | 1 | M | L | N | V | Q | R | G | L | H | T | T | - | V | R | L | S | A | R | T | K | Y | T | K | P | K | P | K | P | Q | A | - | - | - | R | V | I | K | S | E | P | S | Q | V | T | H | H | D | N | N | L | K | I | R | A | P | 51 | | C5DFL5/1-316 | 1 | M | I | S | A | R | R | S | L | H | V | T | - | A | R | A | W | A | R | T | K | Y | T | K | P | K | P | K | P | K | A | - | - | - | R | L | N | V | R | S | P | M | Q | I | T | H | H | D | N | N | L | Q | V | T | A | P | 51 | | C5DS60/1-315 | 1 | M | L | - | F | Q | R | G | L | S | T | T | - | A | R | V | S | A | R | T | K | F | T | R | P | K | P | K | P | P | K | - | - | - | R | Q | N | V | R | P | P | T | Q | T | T | H | H | D | N | T | L | R | I | Q | A | P | 50 | | Kwal\_47.17007/1-316 | 1 | M | L | S | V | K | R | G | L | H | T | T | - | A | F | L | C | A | R | T | K | Y | T | K | P | K | P | K | P | K | S | - | - | - | R | A | N | V | R | S | P | T | Q | S | T | H | H | D | N | N | L | R | V | T | A | P | 51 | | Sbay\_617.21/1-319 | 1 | M | - | - | W | K | R | S | F | H | S | Q | G | G | P | L | R | A | R | T | K | F | T | K | P | K | P | K | Q | P | V | - | L | P | R | D | K | I | R | P | P | T | Q | L | T | H | H | S | R | S | L | Q | I | T | D | P | 52 | | SAKL0G17292g/1-315 | 1 | M | I | S | A | R | R | G | L | H | T | T | - | I | Q | T | C | A | R | T | K | Y | T | K | P | K | P | K | P | K | H | - | - | - | R | T | N | V | R | P | S | T | Q | T | N | H | H | D | N | T | L | K | L | T | A | P | 51 | | P36517/1-319 | 1 | M | - | - | W | K | R | S | F | H | S | Q | G | G | P | L | R | A | R | T | K | F | T | K | P | K | P | K | Q | P | V | - | L | P | K | D | K | I | R | P | P | T | Q | L | T | H | H | S | N | N | L | R | I | T | E | P | 52 | |  | | G0VKY8/1-331 | 52 | I | P | P | T | T | A | N | S | Q | F | I | T | P | E | T | H | P | L | W | Q | F | F | H | D | K | K | F | L | R | S | Q | D | E | L | D | T | K | S | - | - | R | P | W | S | I | P | E | L | R | R | K | S | F | N | D | 104 | | Q6CWC4/1-332 | 52 | I | P | P | A | A | A | N | - | - | L | T | C | P | D | D | H | P | L | W | Q | F | F | S | E | K | K | F | L | R | T | P | E | E | L | D | T | L | S | - | - | R | P | W | T | I | P | E | L | R | R | K | S | F | T | D | 102 | | Q6FK61/1-315 | 53 | I | P | P | S | T | K | N | - | - | I | V | V | P | D | D | H | P | L | W | Q | F | F | S | D | K | K | F | M | R | R | P | E | D | L | D | T | T | S | - | - | R | A | W | S | I | P | E | L | R | R | K | S | F | E | D | 103 | | Q756Y8/1-324 | 52 | I | P | P | A | A | A | N | - | - | L | E | C | N | P | E | H | P | L | W | Q | F | F | D | G | G | R | F | M | R | S | A | E | E | L | D | D | K | S | - | - | R | P | W | T | V | P | E | L | R | R | K | S | F | D | D | 102 | | A7TNQ2/1-322 | 52 | I | P | P | S | A | K | N | - | - | I | V | C | P | E | D | H | P | L | W | Q | F | F | A | D | K | K | F | L | R | D | R | A | D | L | D | N | H | S | - | - | R | P | W | T | I | P | E | L | R | R | K | S | F | E | D | 102 | | C5DFL5/1-316 | 52 | I | P | P | A | A | A | N | - | - | I | T | T | P | D | D | H | P | L | W | Q | F | F | A | D | K | K | Y | L | R | K | F | D | E | L | D | V | D | S | - | - | R | P | W | T | I | P | E | L | R | R | K | S | F | E | D | 102 | | C5DS60/1-315 | 51 | I | P | P | S | A | A | N | - | - | I | Q | C | P | D | D | H | P | L | W | Q | F | F | A | D | K | K | F | M | R | S | P | E | E | L | D | V | H | S | - | - | R | P | W | S | V | P | E | L | R | R | K | S | F | E | D | 101 | | Kwal\_47.17007/1-316 | 52 | I | P | P | A | A | A | N | - | - | I | T | T | P | D | D | H | P | L | W | Q | F | F | A | D | K | K | Y | L | R | K | F | D | E | L | D | N | D | S | - | - | R | A | W | S | I | P | E | L | R | R | K | S | F | E | D | 102 | | Sbay\_617.21/1-319 | 53 | I | P | P | T | A | S | N | - | - | L | R | C | P | A | D | H | P | L | W | Q | F | F | S | N | G | K | F | I | R | T | T | D | D | L | P | P | A | G | H | V | R | P | W | S | I | P | E | L | R | H | K | S | F | N | D | 105 | | SAKL0G17292g/1-315 | 52 | I | P | P | A | A | S | N | - | - | L | K | V | A | D | D | H | P | L | W | Q | F | F | A | D | K | K | F | L | R | K | F | D | E | L | D | T | T | S | - | - | R | P | W | S | I | P | E | L | R | R | K | S | F | D | D | 102 | | P36517/1-319 | 53 | I | P | P | T | T | S | N | - | - | L | R | C | P | D | D | H | P | L | W | Q | F | F | S | N | K | K | F | I | R | S | A | D | D | L | P | P | S | S | H | I | R | P | W | S | I | P | E | L | R | H | K | S | F | N | D | 105 | |  | | G0VKY8/1-331 | 105 | L | H | S | L | W | L | T | A | L | R | E | R | N | V | L | A | R | E | S | H | L | L | H | T | E | S | Q | D | P | T | D | P | F | S | Q | V | A | E | K | C | R | V | T | M | W | R | I | R | H | V | L | S | E | R | H | 159 | | Q6CWC4/1-332 | 103 | L | H | S | L | W | Y | T | C | L | K | E | R | N | V | L | A | R | E | N | H | L | V | Q | F | N | F | E | A | Q | T | E | A | Y | Q | D | I | S | E | K | I | R | T | T | M | W | R | I | R | H | V | L | S | E | R | D | 157 | | Q6FK61/1-315 | 104 | L | H | S | L | W | Y | T | C | L | K | E | R | N | I | L | A | R | E | N | H | L | L | K | N | A | A | K | N | N | R | N | D | Y | E | D | I | S | E | K | I | R | T | T | M | W | R | I | R | H | V | L | S | E | R | D | 158 | | Q756Y8/1-324 | 103 | L | H | S | L | W | Y | A | C | L | K | E | R | N | I | L | A | R | E | M | H | L | R | R | N | - | M | Q | E | E | G | S | A | H | A | Q | L | D | E | R | V | R | T | T | M | W | R | I | R | H | V | L | S | E | R | D | 156 | | A7TNQ2/1-322 | 103 | L | H | S | L | W | Y | T | S | L | K | E | R | N | I | L | A | R | E | N | H | L | L | K | T | A | V | E | S | S | D | D | S | F | E | K | V | A | D | K | V | R | T | T | M | W | R | I | R | H | V | L | S | E | R | D | 157 | | C5DFL5/1-316 | 103 | L | H | S | L | W | Y | T | C | L | K | E | R | N | V | L | A | R | E | N | H | L | L | K | N | D | I | G | S | N | Q | D | S | Y | E | T | V | S | E | K | I | R | T | T | M | W | R | I | R | H | V | L | S | E | R | D | 157 | | C5DS60/1-315 | 102 | L | H | S | L | W | Y | T | C | L | K | E | R | N | I | L | A | R | E | N | H | L | L | R | N | A | V | G | G | Q | Q | E | F | F | E | Q | V | A | E | R | V | R | T | T | M | W | R | I | R | H | V | L | S | E | R | D | 156 | | Kwal\_47.17007/1-316 | 103 | L | H | S | L | W | Y | T | C | L | K | E | R | N | V | L | A | R | E | N | H | L | L | K | N | D | I | G | S | N | Q | D | S | Y | E | A | V | S | E | K | I | R | T | T | M | W | R | I | R | H | V | L | S | E | R | D | 157 | | Sbay\_617.21/1-319 | 106 | L | H | S | L | W | Y | N | C | L | R | E | Q | N | V | L | A | R | E | N | H | L | L | K | N | V | V | G | S | S | H | D | E | F | N | E | L | S | Q | S | I | R | T | T | M | W | Q | I | R | H | V | L | N | E | R | D | 160 | | SAKL0G17292g/1-315 | 103 | L | H | S | L | W | Y | T | C | L | K | E | R | N | V | L | A | R | E | N | H | L | L | R | T | D | I | E | S | N | Q | D | A | Y | E | Q | V | S | E | R | V | R | T | T | M | W | R | I | R | H | V | L | S | E | R | D | 157 | | P36517/1-319 | 106 | L | H | S | L | W | Y | N | C | L | R | E | Q | N | V | L | A | R | E | N | H | L | L | K | N | I | V | G | S | T | H | D | E | F | S | E | L | S | N | S | I | R | T | T | M | W | Q | I | R | H | V | L | N | E | R | E | 160 | |  | | G0VKY8/1-331 | 160 | W | S | Y | V | R | T | K | D | I | V | - | - | - | - | D | P | V | - | - | - | Q | L | Q | E | E | V | A | H | E | L | L | Q | - | - | C | Q | E | E | D | A | F | Y | E | M | L | E | R | C | Q | W | A | L | F | G | I | 205 | | Q6CWC4/1-332 | 158 | W | A | F | K | I | A | N | E | K | F | - | - | A | V | E | K | Q | - | - | - | V | F | I | E | S | F | E | K | D | F | L | E | A | P | A | N | E | D | E | E | I | F | E | S | L | Q | R | F | Q | Y | A | I | Y | G | I | 207 | | Q6FK61/1-315 | 159 | W | A | F | K | R | A | R | E | T | F | - | - | A | Q | N | K | E | L | Q | D | N | L | V | D | D | F | K | K | E | F | L | E | L | D | S | S | E | D | E | Q | A | F | E | M | L | T | R | F | Q | E | A | L | F | G | I | 211 | | Q756Y8/1-324 | 157 | W | A | Y | R | L | A | H | A | E | L | - | - | G | T | Q | Q | A | - | - | - | P | L | L | R | E | V | E | E | A | F | L | A | V | P | E | A | E | D | G | E | A | F | D | M | L | A | R | L | Q | R | A | V | F | G | I | 206 | | A7TNQ2/1-322 | 158 | W | S | Y | R | I | A | N | E | A | F | - | - | V | E | E | V | D | - | - | - | S | F | V | Q | E | F | E | K | D | F | L | L | L | S | Q | D | E | D | E | E | A | F | E | Q | L | S | R | F | Q | K | S | I | F | G | I | 207 | | C5DFL5/1-316 | 158 | W | A | F | K | L | A | Q | Q | E | F | - | - | G | S | E | K | E | - | - | - | V | F | L | Q | E | F | E | A | E | F | L | E | A | P | T | A | E | D | E | E | V | F | E | K | L | S | R | L | Q | Y | S | V | F | G | I | 207 | | C5DS60/1-315 | 157 | W | A | F | R | N | A | Q | E | A | F | - | - | S | V | E | K | E | - | - | - | S | F | V | K | D | F | E | K | E | F | L | E | L | S | Q | E | Q | D | G | E | A | F | E | R | L | S | R | F | Q | H | A | I | F | G | I | 206 | | Kwal\_47.17007/1-316 | 158 | W | A | F | K | L | A | Q | Q | N | F | - | - | E | S | E | K | L | - | - | - | S | L | L | Q | E | F | E | T | E | F | L | D | A | P | A | A | E | D | E | E | S | F | E | K | L | A | R | L | Q | Q | G | V | F | G | I | 207 | | Sbay\_617.21/1-319 | 161 | L | A | H | S | A | S | R | A | S | F | Q | D | Q | S | Q | R | Q | - | - | - | K | F | L | D | T | L | T | N | D | Y | F | L | N | K | D | I | P | D | D | E | V | A | A | M | L | T | R | F | Q | L | A | V | F | G | I | 212 | | SAKL0G17292g/1-315 | 158 | W | A | F | N | I | T | K | E | N | L | - | - | E | P | G | R | Q | - | - | - | E | L | I | Q | E | F | E | S | D | F | L | E | A | P | A | A | E | D | E | E | S | F | E | M | L | S | R | F | Q | N | A | I | F | G | I | 207 | | P36517/1-319 | 161 | L | A | Y | S | A | S | R | E | F | L | Q | D | E | S | E | R | K | - | - | - | K | F | L | D | T | L | A | N | D | Y | F | L | N | K | D | I | P | D | D | E | V | A | S | M | L | T | R | F | Q | L | A | I | F | G | I | 212 | |  | | G0VKY8/1-331 | 206 | S | E | V | V | Q | D | N | L | I | D | A | K | F | V | K | G | V | K | F | V | A | N | L | K | L | Q | W | L | L | K | Q | R | D | I | M | L | Q | D | E | E | N | V | K | K | V | E | K | I | L | - | - | - | Q | G | G | 257 | | Q6CWC4/1-332 | 208 | S | E | Y | I | D | E | N | K | V | D | R | T | F | V | D | G | L | K | A | V | A | T | L | K | L | K | K | F | A | L | R | N | E | - | - | - | - | - | - | - | - | - | - | D | I | K | N | F | L | E | E | - | S | N | N | 251 | | Q6FK61/1-315 | 212 | H | E | V | I | E | D | N | V | V | D | R | K | F | V | D | G | L | K | L | V | A | T | L | K | L | Q | R | F | Q | S | R | N | D | - | - | - | - | - | - | - | - | - | - | S | I | N | A | I | L | E | E | - | S | D | G | 255 | | Q756Y8/1-324 | 207 | S | E | Y | I | E | E | N | L | V | D | R | R | F | V | D | G | L | K | Y | I | A | T | L | K | L | R | R | F | A | P | R | D | A | - | - | - | - | - | - | - | - | - | - | A | V | E | Q | L | L | Q | A | - | S | E | G | 250 | | A7TNQ2/1-322 | 208 | S | E | F | I | D | E | N | V | V | N | K | R | F | I | D | G | M | K | L | I | A | N | L | K | V | K | K | F | S | E | R | N | N | - | - | - | - | - | - | - | - | - | - | D | I | K | T | F | L | D | Q | T | P | N | N | 252 | | C5DFL5/1-316 | 208 | S | E | Y | I | D | E | N | K | V | D | R | A | F | V | D | G | M | K | Y | V | A | T | L | K | L | K | K | F | A | S | R | Q | K | - | - | - | - | - | - | - | - | - | - | E | V | E | D | L | L | Q | Q | - | S | N | N | 251 | | C5DS60/1-315 | 207 | N | E | F | I | D | E | N | L | V | D | R | R | F | V | E | G | L | K | Y | V | A | T | L | K | L | R | K | F | A | S | R | D | E | - | - | - | - | - | - | - | - | - | - | E | L | L | N | F | L | A | Q | C | P | D | Q | 251 | | Kwal\_47.17007/1-316 | 208 | S | E | Y | I | D | E | N | K | V | D | R | P | F | V | D | G | M | K | Y | V | A | T | L | K | L | K | K | F | A | S | R | R | Q | - | - | - | - | - | - | - | - | - | - | S | I | Q | E | L | M | E | Q | - | S | N | N | 251 | | Sbay\_617.21/1-319 | 213 | S | E | T | I | Q | D | N | T | V | D | V | T | F | L | E | G | I | R | F | L | A | N | L | K | L | R | R | F | K | D | S | E | D | - | - | - | - | - | - | - | - | - | - | L | I | S | E | L | - | - | - | - | S | Q | D | 253 | | SAKL0G17292g/1-315 | 208 | S | E | Y | V | D | E | N | K | V | D | R | T | F | V | D | G | L | K | Y | I | A | T | L | K | L | K | K | F | S | S | R | N | A | - | - | - | - | - | - | - | - | - | - | E | I | D | Q | F | L | Q | L | - | S | N | Q | 251 | | P36517/1-319 | 213 | S | E | T | I | Q | D | N | T | V | D | I | N | F | I | D | G | I | K | F | L | A | N | L | K | L | Q | R | F | K | D | S | N | D | - | - | - | - | - | - | - | - | - | - | L | I | S | E | I | - | - | - | - | S | Q | E | 253 | |  | | G0VKY8/1-331 | 258 | P | I | E | D | I | A | E | A | F | V | V | F | T | A | S | N | D | V | N | S | C | V | E | A | C | D | A | I | E | E | L | R | M | D | S | N | N | K | I | H | R | Y | D | E | I | E | T | M | S | K | Y | I | K | Q | L | 312 | | Q6CWC4/1-332 | 252 | T | I | V | D | A | G | E | S | F | L | L | Y | T | S | E | N | T | E | S | A | I | A | E | A | C | Q | A | V | R | E | L | R | E | N | - | G | S | S | V | S | R | Y | D | E | L | E | T | V | Q | E | Y | V | N | K | L | 305 | | Q6FK61/1-315 | 256 | Q | I | N | D | V | G | E | A | F | V | V | F | T | A | D | N | N | L | K | A | V | E | E | A | C | D | A | I | K | E | L | R | Q | G | - | E | N | G | V | S | R | F | D | E | L | P | T | V | R | G | Y | L | K | R | L | 309 | | Q756Y8/1-324 | 251 | G | I | T | D | A | G | E | A | F | V | I | F | T | A | E | N | T | L | A | D | V | K | E | A | A | D | A | V | N | E | L | R | E | Q | - | G | N | K | V | D | R | Y | E | E | I | A | T | V | A | Q | Y | L | K | C | W | 304 | | A7TNQ2/1-322 | 253 | K | I | T | D | A | G | E | A | F | L | V | F | T | C | E | N | T | E | K | D | V | K | E | A | C | E | A | V | L | E | L | R | N | N | - | G | N | A | V | S | R | Y | D | E | L | D | T | V | A | E | Y | V | N | R | L | 306 | | C5DFL5/1-316 | 252 | S | I | S | D | A | G | E | A | F | V | L | F | T | A | E | N | T | L | E | S | V | N | E | A | C | D | V | I | K | E | L | R | A | K | - | N | S | S | V | S | R | Y | E | E | L | E | I | V | G | G | Y | V | K | Q | L | 305 | | C5DS60/1-315 | 252 | G | I | L | D | V | G | E | A | F | V | L | F | T | S | E | H | K | L | E | S | V | K | E | A | C | D | A | I | K | E | L | R | E | R | - | G | N | Y | V | P | R | S | E | E | V | D | T | V | T | Q | Y | I | Q | R | L | 305 | | Kwal\_47.17007/1-316 | 252 | A | I | S | D | A | G | E | A | F | V | L | F | T | A | E | N | T | E | N | S | V | D | E | A | C | E | A | I | K | E | L | R | A | N | - | N | S | S | V | S | R | Y | V | E | L | E | T | V | G | N | Y | V | Q | Q | L | 305 | | Sbay\_617.21/1-319 | 254 | P | I | T | D | V | G | E | S | F | V | L | F | T | S | D | F | D | P | H | A | V | H | D | A | C | V | A | I | K | D | L | R | Q | S | P | E | N | K | V | P | K | L | N | E | L | S | T | V | R | K | Y | L | K | Q | L | 308 | | SAKL0G17292g/1-315 | 252 | A | I | S | D | A | G | E | A | F | V | L | F | T | A | E | N | N | E | S | A | V | K | E | A | C | D | V | V | K | E | L | R | E | K | - | G | A | S | V | S | K | Y | D | E | L | E | T | V | T | E | Y | V | K | Q | L | 305 | | P36517/1-319 | 254 | P | I | T | D | V | G | E | S | F | I | L | F | T | S | D | F | E | P | H | A | V | Q | E | A | C | V | A | I | K | D | L | R | K | S | P | D | N | K | V | P | K | L | D | E | L | P | T | V | R | K | Y | L | K | Q | L | 308 | |  | | G0VKY8/1-331 | 313 | K | K | A | E | S | L | A | E | P | I | L | Q | E | E | G | V | K | S | I | - | - | - | - | - | - | - | - |  | | | | | | | | | | | | | | | | | | | | | | | | | | | | 331 | | Q6CWC4/1-332 | 306 | A | E | A | Q | M | A | K | V | T | E | A | E | I | Q | L | Q | E | E | D | A | K | N | N | A | N | T | L |  | | | | | | | | | | | | | | | | | | | | | | | | | | | | 332 | | Q6FK61/1-315 | 310 | V | S | A | T | T | V | - | - | - | - | - | - | - | - | - | - | - | - | - | - | - | - | - | - | - | - | - |  | | | | | | | | | | | | | | | | | | | | | | | | | | | | 315 | | Q756Y8/1-324 | 305 | P | T | L | R | R | L | L | R | E | P | A | A | L | P | A | D | M | Y | I | Y | - | - | - | - | - | - | - |  | | | | | | | | | | | | | | | | | | | | | | | | | | | | 324 | | A7TNQ2/1-322 | 307 | A | Q | A | Q | S | Q | A | T | Q | P | V | S | E | Q | S | S | - | - | - | - | - | - | - | - | - | - | - |  | | | | | | | | | | | | | | | | | | | | | | | | | | | | 322 | | C5DFL5/1-316 | 306 | A | A | A | Q | D | E | K | T | T | S | A | - | - | - | - | - | - | - | - | - | - | - | - | - | - | - | - |  | | | | | | | | | | | | | | | | | | | | | | | | | | | | 316 | | C5DS60/1-315 | 306 | A | Q | A | Q | L | E | S | P | A | L | - | - | - | - | - | - | - | - | - | - | - | - | - | - | - | - | - |  | | | | | | | | | | | | | | | | | | | | | | | | | | | | 315 | | Kwal\_47.17007/1-316 | 306 | A | A | A | Q | A | E | K | V | T | S | T | - | - | - | - | - | - | - | - | - | - | - | - | - | - | - | - |  | | | | | | | | | | | | | | | | | | | | | | | | | | | | 316 | | Sbay\_617.21/1-319 | 309 | I | R | A | N | S | M | E | Q | A | T | A | - | - | - | - | - | - | - | - | - | - | - | - | - | - | - | - |  | | | | | | | | | | | | | | | | | | | | | | | | | | | | 319 | | SAKL0G17292g/1-315 | 306 | A | A | T | Q | L | E | G | A | N | A | - | - | - | - | - | - | - | - | - | - | - | - | - | - | - | - | - |  | | | | | | | | | | | | | | | | | | | | | | | | | | | | 315 | | P36517/1-319 | 309 | I | H | A | S | S | V | E | Q | A | T | A | - | - | - | - | - | - | - | - | - | - | - | - | - | - | - | - |  | | | | | | | | | | | | | | | | | | | | | | | | | | | | 319 | |
