# Supplementary material for: Plus ça change – evolutionary sequence divergence predicts protein subcellular localization signals
Source: BMC Genomics. 2014 Jan 20;15:46. doi: 10.1186/1471-2164-15-46 (PMC3906766; doi:10.1186/1471-2164-15-46)
Supplement: Additional file 2 — MSA’s of proteins for which sequence divergence changes predicted localization signals. Contains links to ortholog multiple sequence alignments of each protein in Additional file 3: Table S1. [file 1471-2164-15-46-S2.zip › P38228.html]

|  |  |  |  |  |  |  |  |  |  |  |  |  |  |  |  |  |  |  |  |  |  |  |  |  |  |  |  |  |  |  |  |  |  |  |  |  |  |  |  |  |  |  |  |  |  |  |  |  |  |  |  |  |  |  |  |  |  |  |  |  |  |  |  |  |  |  |  |  |  |  |  |  |  |  |  |  |  |  |  |  |  |  |  |  |  |  |  |  |  |  |  |  |  |  |  |  |  |  |  |  |  |  |  |  |  |  |  |  |  |  |  |  |  |  |  |  |  |  |  |  |  |  |  |  |  |  |  |  |  |  |  |  |  |  |  |  |  |  |  |  |  |  |  |  |  |  |  |  |  |  |  |  |  |  |  |  |  |  |  |  |  |  |  |  |  |  |  |  |  |  |  |  |  |  |  |  |  |  |  |  |  |  |  |  |  |  |  |  |  |  |  |  |  |  |  |  |  |  |  |  |  |  |  |  |  |  |  |  |  |  |  |  |  |  |  |  |  |  |  |  |  |  |  |  |  |  |  |  |  |  |  |  |  |  |  |  |  |  |  |  |  |  |  |  |  |  |  |  |  |  |  |  |  |  |  |  |  |  |  |  |  |  |  |  |  |  |  |  |  |  |  |  |  |  |  |  |  |  |  |  |  |  |  |  |  |  |  |  |  |  |  |  |  |  |  |  |  |  |  |  |  |  |  |  |  |  |  |  |  |  |  |  |  |  |  |  |  |  |  |  |  |  |  |  |  |  |  |  |  |  |  |  |  |  |  |  |  |  |  |  |  |  |  |  |  |  |  |  |  |  |  |  |  |  |  |  |  |  |  |  |  |  |  |  |  |  |  |  |  |  |  |  |  |  |  |  |  |  |  |  |  |  |  |  |  |  |  |  |  |  |  |  |  |  |  |  |  |  |  |  |  |  |  |  |  |  |  |  |  |  |  |  |  |  |  |  |  |  |  |  |  |  |  |  |  |  |  |  |  |  |  |  |  |  |  |  |  |  |  |  |  |  |  |  |  |  |  |  |  |  |  |  |  |  |  |  |  |  |  |  |  |  |  |  |  |  |  |  |  |  |  |  |  |  |  |  |  |  |  |  |  |  |  |  |  |  |  |  |  |  |  |  |  |  |  |  |  |  |  |  |  |  |  |  |  |  |  |  |  |  |  |  |  |  |  |  |  |  |  |  |  |  |  |  |  |  |  |  |  |  |  |  |  |  |  |  |  |  |  |  |  |  |  |  |  |  |  |  |  |  |  |  |  |  |  |  |  |  |  |  |  |  |  |  |  |  |  |  |  |  |  |  |  |  |  |  |  |  |  |  |  |  |  |  |  |  |  |  |  |  |  |  |  |  |  |  |  |  |  |  |  |  |  |  |  |  |  |  |  |  |  |  |  |  |  |  |  |  |  |  |  |  |  |  |  |  |  |  |  |  |  |  |  |  |  |  |  |  |  |  |  |  |  |  |  |  |  |  |  |  |  |  |  |  |  |  |  |  |  |  |  |  |  |  |  |  |  |  |  |  |  |  |  |  |  |  |  |  |  |  |  |  |  |  |  |  |  |  |  |  |  |  |  |  |  |  |  |  |  |  |  |  |  |  |  |  |  |  |  |  |  |  |  |  |  |  |  |  |  |  |  |  |  |  |  |  |  |  |  |  |  |  |  |  |  |  |  |  |  |  |  |  |  |  |  |  |  |  |  |  |  |  |  |  |  |  |  |  |  |  |  |  |  |  |  |  |  |  |  |  |  |  |  |  |  |  |  |  |  |  |  |  |  |  |  |  |  |  |  |  |  |  |  |  |  |  |  |  |  |  |  |  |  |  |  |  |  |  |  |  |  |  |  |  |  |  |  |  |  |  |  |  |  |  |  |  |  |  |  |  |  |  |  |  |  |  |  |  |  |  |  |  |  |  |  |  |  |  |  |  |  |  |  |  |  |  |  |  |  |  |  |  |  |  |  |  |  |  |  |  |  |  |  |  |  |  |  |  |  |  |  |  |  |  |  |  |  |  |  |  |  |  |  |  |  |  |  |  |  |  |  |  |  |  |  |  |  |  |  |  |  |  |  |  |  |  |  |  |  |  |  |  |  |  |  |  |  |  |  |  |  |  |  |  |  |  |  |  |  |  |  |  |  |  |  |  |  |  |  |  |  |  |  |  |  |  |  |  |  |  |  |  |  |  |  |  |  |  |  |  |  |  |  |  |  |  |  |  |  |  |  |  |  |  |  |  |  |  |  |  |  |  |  |  |  |  |  |  |  |  |  |  |  |  |  |  |  |  |  |  |  |  |  |  |  |  |  |  |  |  |  |  |  |  |  |  |  |  |  |  |  |  |  |  |  |  |  |  |  |  |  |  |  |  |  |  |  |  |  |  |  |  |  |  |  |  |  |  |  |  |  |  |  |  |  |  |  |  |  |  |  |  |  |  |  |  |  |  |  |  |  |  |  |  |  |  |  |  |  |  |  |  |  |  |  |  |  |  |  |  |  |  |  |  |  |  |  |  |  |  |  |  |  |  |  |  |  |  |  |  |  |  |  |  |  |  |  |  |  |  |  |  |  |  |  |  |  |  |  |  |  |  |  |  |  |  |  |  |  |  |  |  |  |  |  |  |  |  |  |  |  |  |  |  |  |  |  |  |  |  |  |  |  |  |  |  |  |  |  |  |  |  |  |  |  |  |  |  |  |  |  |  |  |  |  |  |  |  |  |  |  |  |  |  |  |  |  |  |  |  |  |  |  |  |  |  |  |  |  |  |  |  |  |  |  |  |  |  |  |  |  |  |  |  |  |  |  |  |  |  |  |  |  |  |  |  |  |  |  |  |  |  |  |  |  |  |  |  |  |  |  |  |  |  |  |  |  |  |  |  |  |  |  |  |  |  |  |  |  |  |  |  |  |  |  |  |  |  |  |  |  |  |  |  |  |  |  |  |  |  |  |  |  |  |  |  |  |  |  |  |  |  |  |  |  |  |  |  |  |  |  |  |  |  |  |  |  |  |  |  |  |  |  |  |  |  |  |  |  |  |  |  |  |  |  |  |  |  |  |  |  |  |  |  |  |  |  |  |  |  |  |  |  |  |  |  |  |  |  |  |  |  |  |  |  |  |  |  |  |  |  |  |  |  |  |  |  |  |  |  |  |  |  |  |  |  |  |  |  |  |  |  |  |  |  |  |  |  |  |  |  |  |  |  |  |  |  |  |  |  |  |  |  |  |  |  |  |  |  |  |  |  |  |  |  |  |  |  |  |  |  |  |  |  |  |  |  |  |  |  |  |  |  |  |  |  |  |  |  |  |  |  |  |  |  |  |  |  |  |  |  |  |  |  |  |  |  |  |  |  |  |  |  |  |  |  |  |  |  |  |  |  |  |  |  |  |  |  |  |  |  |  |  |  |  |  |  |  |  |  |  |  |  |  |  |  |  |  |  |  |  |  |  |  |  |  |  |  |  |  |  |  |  |  |  |  |  |  |  |  |  |  |  |  |  |  |  |  |  |  |  |  |  |  |  |  |  |  |  |  |  |  |  |  |  |  |  |  |  |  |  |  |  |  |  |  |  |  |  |  |  |  |  |  |  |  |  |  |  |  |  |  |  |  |  |  |  |  |  |  |  |  |  |  |  |  |  |  |  |  |  |  |  |  |  |  |  |  |  |  |  |  |  |  |  |  |  |  |  |  |  |  |  |  |  |  |  |  |  |  |  |  |  |  |  |  |  |  |  |  |  |  |  |  |  |  |  |  |  |  |  |  |  |  |  |  |  |  |  |  |  |  |  |  |  |  |  |  |  |  |  |  |  |  |  |  |  |  |  |  |  |  |  |  |  |  |  |  |  |  |  |  |  |  |  |  |  |  |  |  |  |  |  |  |  |  |  |  |  |  |  |  |  |  |  |  |  |  |  |  |  |  |  |  |  |  |  |  |  |  |  |  |  |  |  |  |  |  |  |  |  |  |  |  |  |  |  |  |  |  |  |  |  |  |  |  |  |  |  |  |  |  |  |  |  |  |  |  |  |  |  |  |  |  |  |  |  |  |  |  |  |  |  |  |  |  |  |  |  |  |  |  |  |  |  |  |  |  |  |  |  |  |  |  |  |  |  |  |  |  |  |  |  |  |  |  |  |  |  |  |  |  |  |  |  |  |  |  |  |  |  |  |  |  |  |  |  |  |  |  |  |  |  |  |  |  |  |  |  |  |  |  |  |  |  |  |  |  |  |  |  |  |  |  |  |  |  |  |  |  |  |  |  |  |  |  |  |  |  |  |  |  |  |  |  |  |  |  |  |  |  |  |  |  |  |  |  |  |  |  |  |  |  |  |  |  |  |  |  |  |  |  |  |  |  |  |  |  |  |  |  |  |  |  |  |  |  |  |  |  |  |  |  |  |  |  |  |  |  |  |  |  |  |  |  |  |  |  |  |  |  |  |  |  |  |  |  |  |  |  |  |  |  |  |  |  |  |  |  |  |  |  |  |  |  |  |  |  |  |  |  |  |  |  |  |  |  |  |  |  |  |  |  |  |  |  |  |  |  |  |  |  |  |  |  |  |  |  |  |  |  |  |  |  |  |  |  |  |  |  |  |  |  |  |  |  |  |  |  |  |  |  |  |  |  |  |  |  |  |  |  |  |  |  |  |  |  |  |  |  |  |  |  |  |  |  |  |  |  |  |  |  |  |  |  |  |  |  |  |  |  |  |  |  |  |  |  |  |  |  |  |  |  |  |  |  |  |  |  |  |  |  |  |  |  |  |  |  |  |  |  |  |  |  |  |  |  |  |  |  |  |  |  |  |  |  |  |  |  |  |  |  |  |  |  |  |  |  |  |  |  |  |  |  |  |  |  |  |  |  |  |  |  |  |  |  |  |  |  |  |  |  |  |  |  |  |  |  |  |  |  |  |  |  |  |  |  |  |  |  |  |  |  |  |  |  |  |  |  |  |  |  |  |  |  |  |  |  |  |  |  |  |  |  |  |  |  |  |  |  |  |  |  |  |  |  |  |  |  |  |  |  |  |  |  |  |  |  |  |  |  |  |  |  |  |  |  |  |  |  |  |  |  |  |  |  |  |  |  |  |  |  |  |  |  |  |  |  |  |  |  |  |  |  |  |  |  |  |  |  |  |  |  |  |  |  |  |  |  |  |  |  |  |  |  |  |  |  |  |  |  |  |  |  |  |  |  |  |  |  |  |  |  |  |  |  |  |  |  |  |  |  |  |  |  |  |  |  |  |  |  |  |  |  |  |  |  |  |  |  |  |  |  |  |  |  |  |  |  |  |  |  |  |  |  |  |  |  |  |  |  |  |  |  |  |  |  |  |  |  |  |  |  |  |  |  |  |  |  |  |  |  |  |  |  |  |  |  |  |  |  |  |  |  |  |  |  |  |  |  |  |  |  |  |  |  |  |  |  |  |  |  |  |  |  |  |  |  |  |  |  |  |  |  |  |  |  |  |  |  |  |  |  |  |  |  |  |  |  |  |  |  |  |  |  |  |  |  |  |  |  |  |  |  |  |  |  |  |  |  |  |  |  |  |  |  |  |  |  |  |  |  |  |  |  |  |  |  |  |  |  |  |  |  |  |  |  |  |  |  |  |  |  |  |  |  |  |  |  |  |  |  |  |  |  |  |  |  |  |  |  |  |  |  |  |  |  |  |  |  |  |  |  |  |  |  |  |  |  |  |  |  |  |  |  |  |  |  |  |  |  |  |  |  |  |  |  |  |  |  |  |  |  |  |  |  |  |  |  |  |  |  |  |  |  |  |  |  |  |  |  |  |
| --- | --- | --- | --- | --- | --- | --- | --- | --- | --- | --- | --- | --- | --- | --- | --- | --- | --- | --- | --- | --- | --- | --- | --- | --- | --- | --- | --- | --- | --- | --- | --- | --- | --- | --- | --- | --- | --- | --- | --- | --- | --- | --- | --- | --- | --- | --- | --- | --- | --- | --- | --- | --- | --- | --- | --- | --- | --- | --- | --- | --- | --- | --- | --- | --- | --- | --- | --- | --- | --- | --- | --- | --- | --- | --- | --- | --- | --- | --- | --- | --- | --- | --- | --- | --- | --- | --- | --- | --- | --- | --- | --- | --- | --- | --- | --- | --- | --- | --- | --- | --- | --- | --- | --- | --- | --- | --- | --- | --- | --- | --- | --- | --- | --- | --- | --- | --- | --- | --- | --- | --- | --- | --- | --- | --- | --- | --- | --- | --- | --- | --- | --- | --- | --- | --- | --- | --- | --- | --- | --- | --- | --- | --- | --- | --- | --- | --- | --- | --- | --- | --- | --- | --- | --- | --- | --- | --- | --- | --- | --- | --- | --- | --- | --- | --- | --- | --- | --- | --- | --- | --- | --- | --- | --- | --- | --- | --- | --- | --- | --- | --- | --- | --- | --- | --- | --- | --- | --- | --- | --- | --- | --- | --- | --- | --- | --- | --- | --- | --- | --- | --- | --- | --- | --- | --- | --- | --- | --- | --- | --- | --- | --- | --- | --- | --- | --- | --- | --- | --- | --- | --- | --- | --- | --- | --- | --- | --- | --- | --- | --- | --- | --- | --- | --- | --- | --- | --- | --- | --- | --- | --- | --- | --- | --- | --- | --- | --- | --- | --- | --- | --- | --- | --- | --- | --- | --- | --- | --- | --- | --- | --- | --- | --- | --- | --- | --- | --- | --- | --- | --- | --- | --- | --- | --- | --- | --- | --- | --- | --- | --- | --- | --- | --- | --- | --- | --- | --- | --- | --- | --- | --- | --- | --- | --- | --- | --- | --- | --- | --- | --- | --- | --- | --- | --- | --- | --- | --- | --- | --- | --- | --- | --- | --- | --- | --- | --- | --- | --- | --- | --- | --- | --- | --- | --- | --- | --- | --- | --- | --- | --- | --- | --- | --- | --- | --- | --- | --- | --- | --- | --- | --- | --- | --- | --- | --- | --- | --- | --- | --- | --- | --- | --- | --- | --- | --- | --- | --- | --- | --- | --- | --- | --- | --- | --- | --- | --- | --- | --- | --- | --- | --- | --- | --- | --- | --- | --- | --- | --- | --- | --- | --- | --- | --- | --- | --- | --- | --- | --- | --- | --- | --- | --- | --- | --- | --- | --- | --- | --- | --- | --- | --- | --- | --- | --- | --- | --- | --- | --- | --- | --- | --- | --- | --- | --- | --- | --- | --- | --- | --- | --- | --- | --- | --- | --- | --- | --- | --- | --- | --- | --- | --- | --- | --- | --- | --- | --- | --- | --- | --- | --- | --- | --- | --- | --- | --- | --- | --- | --- | --- | --- | --- | --- | --- | --- | --- | --- | --- | --- | --- | --- | --- | --- | --- | --- | --- | --- | --- | --- | --- | --- | --- | --- | --- | --- | --- | --- | --- | --- | --- | --- | --- | --- | --- | --- | --- | --- | --- | --- | --- | --- | --- | --- | --- | --- | --- | --- | --- | --- | --- | --- | --- | --- | --- | --- | --- | --- | --- | --- | --- | --- | --- | --- | --- | --- | --- | --- | --- | --- | --- | --- | --- | --- | --- | --- | --- | --- | --- | --- | --- | --- | --- | --- | --- | --- | --- | --- | --- | --- | --- | --- | --- | --- | --- | --- | --- | --- | --- | --- | --- | --- | --- | --- | --- | --- | --- | --- | --- | --- | --- | --- | --- | --- | --- | --- | --- | --- | --- | --- | --- | --- | --- | --- | --- | --- | --- | --- | --- | --- | --- | --- | --- | --- | --- | --- | --- | --- | --- | --- | --- | --- | --- | --- | --- | --- | --- | --- | --- | --- | --- | --- | --- | --- | --- | --- | --- | --- | --- | --- | --- | --- | --- | --- | --- | --- | --- | --- | --- | --- | --- | --- | --- | --- | --- | --- | --- | --- | --- | --- | --- | --- | --- | --- | --- | --- | --- | --- | --- | --- | --- | --- | --- | --- | --- | --- | --- | --- | --- | --- | --- | --- | --- | --- | --- | --- | --- | --- | --- | --- | --- | --- | --- | --- | --- | --- | --- | --- | --- | --- | --- | --- | --- | --- | --- | --- | --- | --- | --- | --- | --- | --- | --- | --- | --- | --- | --- | --- | --- | --- | --- | --- | --- | --- | --- | --- | --- | --- | --- | --- | --- | --- | --- | --- | --- | --- | --- | --- | --- | --- | --- | --- | --- | --- | --- | --- | --- | --- | --- | --- | --- | --- | --- | --- | --- | --- | --- | --- | --- | --- | --- | --- | --- | --- | --- | --- | --- | --- | --- | --- | --- | --- | --- | --- | --- | --- | --- | --- | --- | --- | --- | --- | --- | --- | --- | --- | --- | --- | --- | --- | --- | --- | --- | --- | --- | --- | --- | --- | --- | --- | --- | --- | --- | --- | --- | --- | --- | --- | --- | --- | --- | --- | --- | --- | --- | --- | --- | --- | --- | --- | --- | --- | --- | --- | --- | --- | --- | --- | --- | --- | --- | --- | --- | --- | --- | --- | --- | --- | --- | --- | --- | --- | --- | --- | --- | --- | --- | --- | --- | --- | --- | --- | --- | --- | --- | --- | --- | --- | --- | --- | --- | --- | --- | --- | --- | --- | --- | --- | --- | --- | --- | --- | --- | --- | --- | --- | --- | --- | --- | --- | --- | --- | --- | --- | --- | --- | --- | --- | --- | --- | --- | --- | --- | --- | --- | --- | --- | --- | --- | --- | --- | --- | --- | --- | --- | --- | --- | --- | --- | --- | --- | --- | --- | --- | --- | --- | --- | --- | --- | --- | --- | --- | --- | --- | --- | --- | --- | --- | --- | --- | --- | --- | --- | --- | --- | --- | --- | --- | --- | --- | --- | --- | --- | --- | --- | --- | --- | --- | --- | --- | --- | --- | --- | --- | --- | --- | --- | --- | --- | --- | --- | --- | --- | --- | --- | --- | --- | --- | --- | --- | --- | --- | --- | --- | --- | --- | --- | --- | --- | --- | --- | --- | --- | --- | --- | --- | --- | --- | --- | --- | --- | --- | --- | --- | --- | --- | --- | --- | --- | --- | --- | --- | --- | --- | --- | --- | --- | --- | --- | --- | --- | --- | --- | --- | --- | --- | --- | --- | --- | --- | --- | --- | --- | --- | --- | --- | --- | --- | --- | --- | --- | --- | --- | --- | --- | --- | --- | --- | --- | --- | --- | --- | --- | --- | --- | --- | --- | --- | --- | --- | --- | --- | --- | --- | --- | --- | --- | --- | --- | --- | --- | --- | --- | --- | --- | --- | --- | --- | --- | --- | --- | --- | --- | --- | --- | --- | --- | --- | --- | --- | --- | --- | --- | --- | --- | --- | --- | --- | --- | --- | --- | --- | --- | --- | --- | --- | --- | --- | --- | --- | --- | --- | --- | --- | --- | --- | --- | --- | --- | --- | --- | --- | --- | --- | --- | --- | --- | --- | --- | --- | --- | --- | --- | --- | --- | --- | --- | --- | --- | --- | --- | --- | --- | --- | --- | --- | --- | --- | --- | --- | --- | --- | --- | --- | --- | --- | --- | --- | --- | --- | --- | --- | --- | --- | --- | --- | --- | --- | --- | --- | --- | --- | --- | --- | --- | --- | --- | --- | --- | --- | --- | --- | --- | --- | --- | --- | --- | --- | --- | --- | --- | --- | --- | --- | --- | --- | --- | --- | --- | --- | --- | --- | --- | --- | --- | --- | --- | --- | --- | --- | --- | --- | --- | --- | --- | --- | --- | --- | --- | --- | --- | --- | --- | --- | --- | --- | --- | --- | --- | --- | --- | --- | --- | --- | --- | --- | --- | --- | --- | --- | --- | --- | --- | --- | --- | --- | --- | --- | --- | --- | --- | --- | --- | --- | --- | --- | --- | --- | --- | --- | --- | --- | --- | --- | --- | --- | --- | --- | --- | --- | --- | --- | --- | --- | --- | --- | --- | --- | --- | --- | --- | --- | --- | --- | --- | --- | --- | --- | --- | --- | --- | --- | --- | --- | --- | --- | --- | --- | --- | --- | --- | --- | --- | --- | --- | --- | --- | --- | --- | --- | --- | --- | --- | --- | --- | --- | --- | --- | --- | --- | --- | --- | --- | --- | --- | --- | --- | --- | --- | --- | --- | --- | --- | --- | --- | --- | --- | --- | --- | --- | --- | --- | --- | --- | --- | --- | --- | --- | --- | --- | --- | --- | --- | --- | --- | --- | --- | --- | --- | --- | --- | --- | --- | --- | --- | --- | --- | --- | --- | --- | --- | --- | --- | --- | --- | --- | --- | --- | --- | --- | --- | --- | --- | --- | --- | --- | --- | --- | --- | --- | --- | --- | --- | --- | --- | --- | --- | --- | --- | --- | --- | --- | --- | --- | --- | --- | --- | --- | --- | --- | --- | --- | --- | --- | --- | --- | --- | --- | --- | --- | --- | --- | --- | --- | --- | --- | --- | --- | --- | --- | --- | --- | --- | --- | --- | --- | --- | --- | --- | --- | --- | --- | --- | --- | --- | --- | --- | --- | --- | --- | --- | --- | --- | --- | --- | --- | --- | --- | --- | --- | --- | --- | --- | --- | --- | --- | --- | --- | --- | --- | --- | --- | --- | --- | --- | --- | --- | --- | --- | --- | --- | --- | --- | --- | --- | --- | --- | --- | --- | --- | --- | --- | --- | --- | --- | --- | --- | --- | --- | --- | --- | --- | --- | --- | --- | --- | --- | --- | --- | --- | --- | --- | --- | --- | --- | --- | --- | --- | --- | --- | --- | --- | --- | --- | --- | --- | --- | --- | --- | --- | --- | --- | --- | --- | --- | --- | --- | --- | --- | --- | --- | --- | --- | --- | --- | --- | --- | --- | --- | --- | --- | --- | --- | --- | --- | --- | --- | --- | --- | --- | --- | --- | --- | --- | --- | --- | --- | --- | --- | --- | --- | --- | --- | --- | --- | --- | --- | --- | --- | --- | --- | --- | --- | --- | --- | --- | --- | --- | --- | --- | --- | --- | --- | --- | --- | --- | --- | --- | --- | --- | --- | --- | --- | --- | --- | --- | --- | --- | --- | --- | --- | --- | --- | --- | --- | --- | --- | --- | --- | --- | --- | --- | --- | --- | --- | --- | --- | --- | --- | --- | --- | --- | --- | --- | --- | --- | --- | --- | --- | --- | --- | --- | --- | --- | --- | --- | --- | --- | --- | --- | --- | --- | --- | --- | --- | --- | --- | --- | --- | --- | --- | --- | --- | --- | --- | --- | --- | --- | --- | --- | --- | --- | --- | --- | --- | --- | --- | --- | --- | --- | --- | --- | --- | --- | --- | --- | --- | --- | --- | --- | --- | --- | --- | --- | --- | --- | --- | --- | --- | --- | --- | --- | --- | --- | --- | --- | --- | --- | --- | --- | --- | --- | --- | --- | --- | --- | --- | --- | --- | --- | --- | --- | --- | --- | --- | --- | --- | --- | --- | --- | --- | --- | --- | --- | --- | --- | --- | --- | --- | --- | --- | --- | --- | --- | --- | --- | --- | --- | --- | --- | --- | --- | --- | --- | --- | --- | --- | --- | --- | --- | --- | --- | --- | --- | --- | --- | --- | --- | --- | --- | --- | --- | --- | --- | --- | --- | --- | --- | --- | --- | --- | --- | --- | --- | --- | --- | --- | --- | --- | --- | --- | --- | --- | --- | --- | --- | --- | --- | --- | --- | --- | --- | --- | --- | --- | --- | --- | --- | --- | --- | --- | --- | --- | --- | --- | --- | --- | --- | --- | --- | --- | --- | --- | --- | --- | --- | --- | --- | --- | --- | --- | --- | --- | --- | --- | --- | --- | --- | --- | --- | --- | --- | --- | --- | --- | --- | --- | --- | --- | --- | --- | --- | --- | --- | --- | --- | --- | --- | --- | --- | --- | --- | --- | --- | --- | --- | --- | --- | --- | --- | --- | --- | --- | --- | --- | --- | --- | --- | --- | --- | --- | --- | --- | --- | --- | --- | --- | --- | --- | --- | --- | --- | --- | --- | --- | --- | --- | --- | --- | --- | --- | --- | --- | --- | --- | --- | --- | --- | --- | --- | --- | --- | --- | --- | --- | --- | --- | --- | --- | --- | --- | --- | --- | --- | --- | --- | --- | --- | --- | --- | --- | --- | --- | --- | --- | --- | --- | --- | --- | --- | --- | --- | --- | --- | --- | --- | --- | --- | --- | --- | --- | --- | --- | --- | --- | --- | --- | --- | --- | --- | --- | --- | --- | --- | --- | --- | --- | --- | --- | --- | --- | --- | --- | --- | --- | --- | --- | --- | --- | --- | --- | --- | --- | --- | --- | --- | --- | --- | --- | --- | --- | --- | --- | --- | --- | --- | --- | --- | --- | --- | --- | --- | --- | --- | --- | --- | --- | --- | --- | --- | --- | --- | --- | --- | --- | --- | --- | --- | --- | --- | --- | --- | --- | --- | --- | --- | --- | --- | --- | --- | --- | --- | --- | --- | --- | --- | --- | --- | --- | --- | --- | --- | --- | --- | --- | --- | --- | --- | --- | --- | --- | --- | --- | --- | --- | --- | --- | --- | --- | --- | --- | --- | --- | --- | --- | --- | --- | --- | --- | --- | --- | --- | --- | --- | --- | --- | --- | --- | --- | --- | --- | --- | --- | --- | --- | --- | --- | --- | --- | --- | --- | --- | --- | --- | --- | --- | --- | --- | --- | --- | --- | --- | --- | --- | --- | --- | --- | --- | --- | --- | --- | --- | --- | --- | --- | --- | --- | --- | --- | --- | --- | --- | --- | --- | --- | --- | --- | --- | --- | --- | --- | --- | --- | --- | --- | --- | --- | --- | --- | --- | --- | --- | --- | --- | --- | --- | --- | --- | --- | --- | --- | --- | --- | --- | --- | --- | --- | --- | --- | --- | --- | --- | --- | --- | --- | --- | --- | --- | --- | --- | --- | --- | --- | --- | --- | --- | --- | --- | --- | --- | --- | --- | --- | --- | --- | --- | --- | --- | --- | --- | --- | --- | --- | --- | --- | --- | --- | --- | --- | --- | --- | --- | --- | --- | --- | --- | --- | --- | --- | --- | --- | --- | --- | --- | --- | --- | --- | --- | --- | --- | --- | --- | --- | --- | --- | --- | --- | --- | --- | --- | --- | --- | --- | --- | --- | --- | --- | --- | --- | --- | --- | --- | --- | --- | --- | --- | --- | --- | --- | --- | --- | --- | --- | --- | --- | --- | --- | --- | --- | --- | --- | --- | --- | --- | --- | --- | --- | --- | --- | --- | --- | --- | --- | --- | --- | --- | --- | --- | --- | --- | --- | --- | --- | --- | --- | --- | --- | --- | --- | --- | --- | --- | --- | --- | --- | --- | --- | --- | --- | --- | --- | --- | --- | --- | --- | --- | --- | --- | --- | --- | --- | --- | --- | --- | --- | --- | --- | --- | --- | --- | --- | --- | --- | --- | --- | --- | --- | --- | --- | --- | --- | --- | --- | --- | --- | --- | --- | --- | --- | --- | --- | --- | --- | --- | --- | --- | --- | --- | --- | --- | --- | --- | --- | --- | --- | --- | --- | --- | --- | --- | --- | --- | --- | --- | --- | --- | --- | --- | --- | --- | --- | --- | --- | --- | --- | --- | --- | --- | --- | --- | --- | --- | --- | --- | --- | --- | --- | --- | --- | --- | --- | --- | --- | --- | --- | --- | --- | --- | --- | --- | --- | --- | --- | --- | --- | --- | --- | --- | --- | --- | --- | --- | --- | --- | --- | --- | --- | --- | --- | --- | --- | --- | --- | --- | --- | --- | --- | --- | --- | --- | --- | --- | --- | --- | --- | --- | --- | --- | --- | --- | --- | --- | --- | --- | --- | --- | --- | --- | --- | --- | --- | --- | --- | --- | --- | --- | --- | --- | --- | --- | --- | --- | --- | --- | --- | --- | --- | --- | --- | --- | --- | --- | --- | --- | --- | --- | --- | --- | --- | --- | --- | --- | --- | --- | --- | --- | --- | --- | --- | --- | --- | --- | --- | --- | --- | --- | --- | --- | --- | --- | --- | --- | --- | --- | --- | --- | --- | --- | --- | --- | --- | --- | --- | --- | --- | --- | --- | --- | --- | --- | --- | --- | --- | --- | --- | --- | --- | --- | --- | --- | --- | --- | --- | --- | --- | --- | --- | --- | --- | --- | --- | --- | --- | --- | --- | --- | --- | --- | --- | --- | --- | --- | --- | --- | --- | --- | --- | --- | --- | --- | --- | --- | --- | --- | --- | --- | --- | --- | --- | --- | --- | --- | --- | --- | --- | --- | --- | --- | --- | --- | --- | --- | --- | --- | --- | --- | --- | --- | --- | --- | --- | --- | --- | --- | --- | --- | --- | --- | --- | --- | --- | --- | --- | --- | --- |
| |  |  |  |  |  |  |  |  |  |  |  |  |  |  |  |  |  |  |  |  |  |  |  |  |  |  |  |  |  |  |  |  |  |  |  |  |  |  |  |  |  |  |  |  |  |  |  |  |  |  |  |  |  |  |  |  |  |  | | --- | --- | --- | --- | --- | --- | --- | --- | --- | --- | --- | --- | --- | --- | --- | --- | --- | --- | --- | --- | --- | --- | --- | --- | --- | --- | --- | --- | --- | --- | --- | --- | --- | --- | --- | --- | --- | --- | --- | --- | --- | --- | --- | --- | --- | --- | --- | --- | --- | --- | --- | --- | --- | --- | --- | --- | --- | --- | | C5DPH9/1-569 | 1 | M | F | R | R | S | I | H | K | - | S | V | C | Q | S | I | R | T | V | K | T | L | H | T | P | I | Y | E | T | G | Q | L | S | T | K | Q | S | L | M | Q | G | I | K | L | L | D | N | V | V | N | S | T | T | Y | N | K | 54 | | Sbay\_678.180/1-572 | 1 | M | L | R | N | C | L | K | K | L | G | S | H | R | T | R | C | T | I | K | T | L | H | T | P | V | Y | R | M | K | N | L | Q | V | L | R | N | I | L | S | G | I | K | L | L | D | K | I | L | S | S | S | S | Y | N | K | 55 | | SAKL0D04004g/1-558 | 1 | M | I | R | R | - | L | N | K | - | - | - | - | T | S | I | R | T | V | K | T | L | H | T | P | V | Y | D | G | K | Q | F | V | T | R | Q | Y | L | L | H | H | I | A | L | L | D | K | V | L | N | A | T | S | Y | N | K | 50 | | P38228/1-572 | 1 | M | L | R | N | C | L | R | K | L | G | N | H | Q | T | K | C | S | V | K | T | L | H | T | P | I | Y | R | T | K | N | L | Q | V | L | R | D | T | L | S | G | I | K | L | L | E | K | I | I | T | S | S | S | Y | N | K | 55 | |  | | C5DPH9/1-569 | 55 | T | L | L | Q | L | S | K | Y | N | T | N | P | K | F | I | T | S | R | D | S | I | R | L | Q | N | V | V | R | E | L | L | D | G | L | R | M | D | E | V | M | T | G | R | E | R | Q | D | H | Q | S | R | L | A | K | I | 109 | | Sbay\_678.180/1-572 | 56 | T | L | I | Y | E | P | K | Y | K | S | R | P | Q | V | V | S | S | Q | D | T | M | R | L | Q | N | V | L | R | E | F | L | D | S | L | Q | I | D | E | A | T | N | T | E | L | Q | L | D | S | S | R | K | L | G | K | V | 110 | | SAKL0D04004g/1-558 | 51 | S | I | L | Y | T | G | K | Y | K | K | L | P | Q | L | I | T | S | Q | D | S | V | R | V | N | N | V | V | R | D | M | L | E | S | L | Q | M | D | E | A | F | D | Q | Q | R | Q | R | D | P | S | S | K | L | G | K | I | 105 | | P38228/1-572 | 56 | T | L | I | Y | E | P | K | Y | K | S | K | P | Q | V | V | S | S | H | D | T | M | R | L | H | N | V | M | R | E | L | L | D | S | L | Q | V | D | E | A | T | N | T | R | L | Q | S | N | R | P | R | K | L | G | R | V | 110 | |  | | C5DPH9/1-569 | 110 | G | L | Q | L | F | C | E | I | H | E | S | N | I | L | P | I | S | T | S | L | T | L | T | L | M | A | E | Y | A | K | S | P | N | V | T | T | L | R | A | M | L | D | G | L | E | K | V | R | T | F | L | T | E | N | I | 164 | | Sbay\_678.180/1-572 | 111 | G | L | Q | L | F | L | D | C | T | R | D | N | L | T | L | N | S | T | S | L | T | S | S | L | L | E | C | Y | F | K | Y | P | E | K | E | V | V | N | G | I | K | I | G | L | R | Y | I | R | G | F | L | E | Q | N | K | 165 | | SAKL0D04004g/1-558 | 106 | G | L | Q | L | F | M | D | C | H | Q | N | N | I | T | P | V | S | T | S | L | T | K | S | L | T | E | Q | F | N | R | Y | P | E | R | T | T | I | V | G | I | E | E | G | I | S | E | V | R | Q | F | L | R | E | K | K | 160 | | P38228/1-572 | 111 | G | L | Q | L | F | M | D | C | I | Q | D | N | L | T | A | T | S | T | S | L | T | C | S | L | L | E | H | Y | F | K | Y | P | E | K | E | V | T | N | G | I | K | A | G | L | R | Y | I | R | D | F | L | A | K | N | K | 165 | |  | | C5DPH9/1-569 | 165 | I | H | I | S | S | T | V | D | I | D | A | L | V | D | K | L | T | I | L | R | E | D | S | E | T | V | K | Q | V | L | K | A | L | D | Y | K | L | Y | S | D | D | L | V | R | I | V | K | G | R | K | T | T | D | E | I | 219 | | Sbay\_678.180/1-572 | 166 | I | M | V | K | G | Q | N | D | I | D | A | L | V | D | Q | F | T | M | S | S | L | D | S | Q | S | V | K | N | V | L | R | A | V | N | Y | Q | L | F | S | D | D | I | V | R | V | I | N | G | N | K | T | Y | D | E | I | 220 | | SAKL0D04004g/1-558 | 161 | I | Q | L | R | E | P | W | E | I | D | P | L | V | D | K | L | A | H | L | S | K | D | A | E | T | I | K | K | V | L | S | L | L | D | Y | K | L | F | A | D | D | I | V | R | V | N | R | G | K | K | T | T | D | E | I | 215 | | P38228/1-572 | 166 | I | I | V | K | S | Q | N | D | V | D | A | L | V | E | Q | L | T | M | S | S | S | D | S | Q | S | I | K | R | V | L | K | A | I | N | Y | E | L | F | S | D | D | I | V | R | V | I | N | G | N | K | T | Y | D | E | V | 220 | |  | | C5DPH9/1-569 | 220 | D | V | S | K | G | W | K | F | P | A | G | I | L | D | T | N | E | A | Y | L | R | S | I | E | L | P | Q | K | K | L | V | S | V | D | D | E | M | L | V | L | I | Y | D | G | T | L | R | D | A | N | A | V | L | P | T | 274 | | Sbay\_678.180/1-572 | 221 | D | V | S | K | G | W | K | Y | P | A | G | I | L | D | T | N | E | A | Y | L | R | S | L | D | L | P | N | K | K | L | V | S | V | D | K | K | M | L | V | L | M | Y | D | G | T | L | R | D | A | N | K | I | L | P | T | 275 | | SAKL0D04004g/1-558 | 216 | D | I | S | K | G | W | K | Y | P | T | G | I | V | D | T | N | D | A | Y | L | R | S | L | Q | F | S | K | K | N | L | I | T | I | N | R | D | S | L | V | L | V | Y | D | G | T | L | R | D | A | G | R | I | L | P | S | 270 | | P38228/1-572 | 221 | D | V | S | K | G | W | K | Y | P | A | G | I | L | D | S | N | E | A | Y | L | R | S | L | E | L | P | T | K | K | L | V | S | I | D | K | D | M | L | V | L | M | Y | D | G | T | L | R | D | A | N | K | I | L | P | T | 275 | |  | | C5DPH9/1-569 | 275 | L | H | H | A | S | K | S | K | K | S | L | L | L | M | V | T | G | D | C | T | G | D | A | L | A | S | I | V | I | S | N | N | R | N | R | R | Q | G | I | K | S | Q | T | F | I | I | K | Y | D | T | K | A | N | D | G | 329 | | Sbay\_678.180/1-572 | 276 | V | T | Y | A | R | N | L | K | K | S | V | L | L | I | V | K | G | D | C | T | G | D | A | L | T | S | I | T | I | N | N | N | R | N | K | R | E | N | N | E | S | R | I | I | I | M | K | Y | S | N | K | A | N | K | N | 330 | | SAKL0D04004g/1-558 | 271 | L | H | Y | A | A | K | Q | E | K | S | L | L | L | I | V | T | G | D | C | T | G | D | A | L | T | S | I | T | I | N | N | N | K | N | K | R | K | G | I | Q | S | E | T | V | V | M | K | Y | L | A | R | D | H | N | N | 325 | | P38228/1-572 | 276 | I | T | Y | A | R | K | L | R | K | S | V | L | L | I | V | N | G | D | C | T | G | D | A | L | T | S | V | T | I | N | N | N | R | N | K | R | E | N | N | E | S | R | I | V | V | L | K | Y | S | K | K | A | N | N | D | 330 | |  | | C5DPH9/1-569 | 330 | I | S | L | H | E | N | Y | D | L | V | Q | F | L | R | L | P | Q | G | F | A | S | V | Y | S | P | D | Y | S | T | L | V | P | S | K | M | C | A | N | Q | Y | Y | G | K | L | D | S | L | K | A | T | T | G | E | A | F | 384 | | Sbay\_678.180/1-572 | 331 | L | A | L | Q | E | N | H | D | F | V | K | F | L | R | L | P | C | G | Y | D | S | I | Y | S | P | E | Y | S | P | L | V | P | S | K | M | C | A | D | K | Y | Y | G | S | V | E | S | I | K | A | T | T | G | E | A | F | 385 | | SAKL0D04004g/1-558 | 326 | T | Q | L | Q | E | N | L | D | L | I | R | F | L | K | L | P | L | G | L | G | S | V | Y | N | Q | D | F | S | E | Y | V | P | S | A | A | S | A | K | Q | F | F | G | S | V | E | S | I | K | A | T | T | G | E | A | F | 380 | | P38228/1-572 | 331 | L | A | P | Q | E | N | L | D | F | I | K | F | L | R | L | P | C | G | Y | D | S | I | Y | S | P | E | Y | S | P | L | V | P | S | K | M | C | A | D | K | Y | Y | G | S | I | E | S | I | K | A | T | T | G | E | A | F | 385 | |  | | C5DPH9/1-569 | 385 | L | Y | N | P | I | E | W | S | G | E | E | Q | E | - | - | N | P | F | T | K | M | T | V | T | V | N | V | G | A | Q | S | E | I | E | I | D | H | R | R | N | F | L | D | N | L | I | N | N | T | L | C | H | G | L | S | 437 | | Sbay\_678.180/1-572 | 386 | L | Y | N | S | V | D | F | E | S | S | Q | N | E | A | S | Q | S | F | L | Q | Q | T | V | T | L | K | I | G | G | H | N | E | V | E | I | D | Q | R | R | N | T | L | E | N | F | L | N | N | S | L | C | H | G | L | A | 440 | | SAKL0D04004g/1-558 | 381 | L | Y | N | T | Q | D | A | D | - | - | - | D | E | I | N | N | S | S | L | R | T | T | V | T | V | N | V | G | G | E | S | E | F | E | I | D | Q | R | R | A | A | L | D | N | I | I | N | N | I | L | C | H | G | L | A | 432 | | P38228/1-572 | 386 | L | Y | N | S | I | D | A | E | A | I | P | N | K | V | P | K | S | F | L | Q | N | T | V | T | L | S | I | G | G | H | N | E | I | E | I | D | R | R | R | N | A | I | D | N | C | L | N | N | V | L | C | H | G | L | A | 440 | |  | | C5DPH9/1-569 | 438 | K | G | F | I | P | G | Y | N | V | A | L | A | K | A | V | P | L | L | E | K | L | A | F | T | A | Q | D | V | D | T | K | M | G | Y | E | A | V | I | M | A | L | A | Q | S | L | Q | R | S | L | T | N | A | F | G | Y | 492 | | Sbay\_678.180/1-572 | 441 | K | G | F | I | P | S | H | G | I | S | L | L | K | A | V | P | G | L | S | K | L | K | A | N | E | P | N | F | M | T | K | L | G | I | D | A | V | L | S | A | V | V | L | P | S | E | V | A | F | K | N | A | Y | G | Y | 495 | | SAKL0D04004g/1-558 | 433 | Q | G | F | V | P | S | Y | G | V | A | L | A | K | S | I | A | N | L | D | A | G | N | V | D | K | S | N | L | Q | S | - | V | G | K | Y | A | V | M | E | S | L | T | Y | P | L | E | N | A | M | N | N | L | Y | S | L | 486 | | P38228/1-572 | 441 | K | G | F | I | P | G | Y | G | I | S | L | L | K | A | I | P | G | L | N | E | L | K | A | N | E | P | N | F | M | T | K | V | G | I | N | A | V | L | S | A | V | I | L | P | S | E | V | A | F | K | N | A | Y | G | Y | 495 | |  | | C5DPH9/1-569 | 493 | N | K | F | T | S | T | S | L | L | A | E | T | I | K | D | P | N | F | S | T | A | A | I | E | P | M | G | D | L | Q | E | L | S | R | S | G | I | L | D | P | W | D | K | V | D | R | C | L | A | S | I | A | S | F | M | 547 | | Sbay\_678.180/1-572 | 496 | N | H | Y | E | I | S | N | L | I | A | A | A | I | N | E | N | S | F | Q | L | A | K | F | S | S | T | S | E | L | V | D | T | T | K | A | G | S | L | E | P | W | S | K | M | D | S | C | L | A | N | I | A | T | F | I | 550 | | SAKL0D04004g/1-558 | 487 | D | R | F | S | T | A | K | L | I | S | Q | T | I | A | D | S | D | F | T | T | A | Y | L | D | K | P | T | S | M | V | D | - | - | - | S | G | L | L | E | P | W | D | K | L | D | T | C | L | A | N | V | T | N | F | I | 538 | | P38228/1-572 | 496 | N | Y | Y | E | I | N | S | L | I | A | G | A | I | N | E | K | S | F | P | M | A | K | F | S | P | N | S | E | P | V | N | T | V | K | D | G | N | L | E | P | W | S | K | M | D | S | C | L | A | G | V | E | T | F | I | 550 | |  | | C5DPH9/1-569 | 548 | R | I | L | S | S | S | D | K | I | V | A | Q | V | F | E | P | P | K | K | R | Q | P |  | | | | | | | | | | | | | | | | | | | | | | | | | | | | | | | | | 569 | | Sbay\_678.180/1-572 | 551 | K | L | L | T | S | C | N | T | I | I | T | C | I | Y | E | K | P | E | K | R | T | A |  | | | | | | | | | | | | | | | | | | | | | | | | | | | | | | | | | 572 | | SAKL0D04004g/1-558 | 539 | R | L | I | S | S | C | D | V | L | V | T | R | F | F | D | K | P | K | R | K | - | - |  | | | | | | | | | | | | | | | | | | | | | | | | | | | | | | | | | 558 | | P38228/1-572 | 551 | E | L | L | T | S | C | N | T | I | I | T | C | V | Y | K | K | P | E | R | H | K | A |  | | | | | | | | | | | | | | | | | | | | | | | | | | | | | | | | | 572 | |
