# Supplementary material for: Plus ça change – evolutionary sequence divergence predicts protein subcellular localization signals
Source: BMC Genomics. 2014 Jan 20;15:46. doi: 10.1186/1471-2164-15-46 (PMC3906766; doi:10.1186/1471-2164-15-46)
Supplement: Additional file 2 — MSA’s of proteins for which sequence divergence changes predicted localization signals. Contains links to ortholog multiple sequence alignments of each protein in Additional file 3: Table S1. [file 1471-2164-15-46-S2.zip › P38719.html]

|  |  |  |  |  |  |  |  |  |  |  |  |  |  |  |  |  |  |  |  |  |  |  |  |  |  |  |  |  |  |  |  |  |  |  |  |  |  |  |  |  |  |  |  |  |  |  |  |  |  |  |  |  |  |  |  |  |  |  |  |  |  |  |  |  |  |  |  |  |  |  |  |  |  |  |  |  |  |  |  |  |  |  |  |  |  |  |  |  |  |  |  |  |  |  |  |  |  |  |  |  |  |  |  |  |  |  |  |  |  |  |  |  |  |  |  |  |  |  |  |  |  |  |  |  |  |  |  |  |  |  |  |  |  |  |  |  |  |  |  |  |  |  |  |  |  |  |  |  |  |  |  |  |  |  |  |  |  |  |  |  |  |  |  |  |  |  |  |  |  |  |  |  |  |  |  |  |  |  |  |  |  |  |  |  |  |  |  |  |  |  |  |  |  |  |  |  |  |  |  |  |  |  |  |  |  |  |  |  |  |  |  |  |  |  |  |  |  |  |  |  |  |  |  |  |  |  |  |  |  |  |  |  |  |  |  |  |  |  |  |  |  |  |  |  |  |  |  |  |  |  |  |  |  |  |  |  |  |  |  |  |  |  |  |  |  |  |  |  |  |  |  |  |  |  |  |  |  |  |  |  |  |  |  |  |  |  |  |  |  |  |  |  |  |  |  |  |  |  |  |  |  |  |  |  |  |  |  |  |  |  |  |  |  |  |  |  |  |  |  |  |  |  |  |  |  |  |  |  |  |  |  |  |  |  |  |  |  |  |  |  |  |  |  |  |  |  |  |  |  |  |  |  |  |  |  |  |  |  |  |  |  |  |  |  |  |  |  |  |  |  |  |  |  |  |  |  |  |  |  |  |  |  |  |  |  |  |  |  |  |  |  |  |  |  |  |  |  |  |  |  |  |  |  |  |  |  |  |  |  |  |  |  |  |  |  |  |  |  |  |  |  |  |  |  |  |  |  |  |  |  |  |  |  |  |  |  |  |  |  |  |  |  |  |  |  |  |  |  |  |  |  |  |  |  |  |  |  |  |  |  |  |  |  |  |  |  |  |  |  |  |  |  |  |  |  |  |  |  |  |  |  |  |  |  |  |  |  |  |  |  |  |  |  |  |  |  |  |  |  |  |  |  |  |  |  |  |  |  |  |  |  |  |  |  |  |  |  |  |  |  |  |  |  |  |  |  |  |  |  |  |  |  |  |  |  |  |  |  |  |  |  |  |  |  |  |  |  |  |  |  |  |  |  |  |  |  |  |  |  |  |  |  |  |  |  |  |  |  |  |  |  |  |  |  |  |  |  |  |  |  |  |  |  |  |  |  |  |  |  |  |  |  |  |  |  |  |  |  |  |  |  |  |  |  |  |  |  |  |  |  |  |  |  |  |  |  |  |  |  |  |  |  |  |  |  |  |  |  |  |  |  |  |  |  |  |  |  |  |  |  |  |  |  |  |  |  |  |  |  |  |  |  |  |  |  |  |  |  |  |  |  |  |  |  |  |  |  |  |  |  |  |  |  |  |  |  |  |  |  |  |  |  |  |  |  |  |  |  |  |  |  |  |  |  |  |  |  |  |  |  |  |  |  |  |  |  |  |  |  |  |  |  |  |  |  |  |  |  |  |  |  |  |  |  |  |  |  |  |  |  |  |  |  |  |  |  |  |  |  |  |  |  |  |  |  |  |  |  |  |  |  |  |  |  |  |  |  |  |  |  |  |  |  |  |  |  |  |  |  |  |  |  |  |  |  |  |  |  |  |  |  |  |  |  |  |  |  |  |  |  |  |  |  |  |  |  |  |  |  |  |  |  |  |  |  |  |  |  |  |  |  |  |  |  |  |  |  |  |  |  |  |  |  |  |  |  |  |  |  |  |  |  |  |  |  |  |  |  |  |  |  |  |  |  |  |  |  |  |  |  |  |  |  |  |  |  |  |  |  |  |  |  |  |  |  |  |  |  |  |  |  |  |  |  |  |  |  |  |  |  |  |  |  |  |  |  |  |  |  |  |  |  |  |  |  |  |  |  |  |  |  |  |  |  |  |  |  |  |  |  |  |  |  |  |  |  |  |  |  |  |  |  |  |  |  |  |  |  |  |  |  |  |  |  |  |  |  |  |  |  |  |  |  |  |  |  |  |  |  |  |  |  |  |  |  |  |  |  |  |  |  |  |  |  |  |  |  |  |  |  |  |  |  |  |  |  |  |  |  |  |  |  |  |  |  |  |  |  |  |  |  |  |  |  |  |  |  |  |  |  |  |  |  |  |  |  |  |  |  |  |  |  |  |  |  |  |  |  |  |  |  |  |  |  |  |  |  |  |  |  |  |  |  |  |  |  |  |  |  |  |  |  |  |  |  |  |  |  |  |  |  |  |  |  |  |  |  |  |  |  |  |  |  |  |  |  |  |  |  |  |  |  |  |  |  |  |  |  |  |  |  |  |  |  |  |  |  |  |  |  |  |  |  |  |  |  |  |  |  |  |  |  |  |  |  |  |  |  |  |  |  |  |  |  |  |  |  |  |  |  |  |  |  |  |  |  |  |  |  |  |  |  |  |  |  |  |  |  |  |  |  |  |  |  |  |  |  |  |  |  |  |  |  |  |  |  |  |  |  |  |  |  |  |  |  |  |  |  |  |  |  |  |  |  |  |  |  |  |  |  |  |  |  |  |  |  |  |  |  |  |  |  |  |  |  |  |  |  |  |  |  |  |  |  |  |  |  |  |  |  |  |  |  |  |  |  |  |  |  |  |  |  |  |  |  |  |  |  |  |  |  |  |  |  |  |  |  |  |  |  |  |  |  |  |  |  |  |  |  |  |  |  |  |  |  |  |  |  |  |  |  |  |  |  |  |  |  |  |  |  |  |  |  |  |  |  |  |  |  |  |  |  |  |  |  |  |  |  |  |  |  |  |  |  |  |  |  |  |  |  |  |  |  |  |  |  |  |  |  |  |  |  |  |  |  |  |  |  |  |  |  |  |  |  |  |  |  |  |  |  |  |  |  |  |  |  |  |  |  |  |  |  |  |  |  |  |  |  |  |  |  |  |  |  |  |  |  |  |  |  |  |  |  |  |  |  |  |  |  |  |  |  |  |  |  |  |  |  |  |  |  |  |  |  |  |  |  |  |  |  |  |  |  |  |  |  |  |  |  |  |  |  |  |  |  |  |  |  |  |  |  |  |  |  |  |  |  |  |  |  |  |  |  |  |  |  |  |  |  |  |  |  |  |  |  |  |  |  |  |  |  |  |  |  |  |  |  |  |  |  |  |  |  |  |  |  |  |  |  |  |  |  |  |  |  |  |  |  |  |  |  |  |  |  |  |  |  |  |  |  |  |  |  |  |  |  |  |  |  |  |  |  |  |  |  |  |  |  |  |  |  |  |  |  |  |  |  |  |  |  |  |  |  |  |  |  |  |  |  |  |  |  |  |  |  |  |  |  |  |  |  |  |  |  |  |  |  |  |  |  |  |  |  |  |  |  |  |  |  |  |  |  |  |  |  |  |  |  |  |  |  |  |  |  |  |  |  |  |  |  |  |  |  |  |  |  |  |  |  |  |  |  |  |  |  |  |  |  |  |  |  |  |  |  |  |  |  |  |  |  |  |  |  |  |  |  |  |  |  |  |  |  |  |  |  |  |  |  |  |  |  |  |  |  |  |  |  |  |  |  |  |  |  |  |  |  |  |  |  |  |  |  |  |  |  |  |  |  |  |  |  |  |  |  |  |  |  |  |  |  |  |  |  |  |  |  |  |  |  |  |  |  |  |  |  |  |  |  |  |  |  |  |  |  |  |  |  |  |  |  |  |  |  |  |  |  |  |  |  |  |  |  |  |  |  |  |  |  |  |  |  |  |  |  |  |  |  |  |  |  |  |  |  |  |  |  |  |  |  |  |  |  |  |  |  |  |  |  |  |  |  |  |  |  |  |  |  |  |  |  |  |  |  |  |  |  |  |  |  |  |  |  |  |  |  |  |  |  |  |  |  |  |  |  |  |  |  |  |  |  |  |  |  |  |  |  |  |  |  |  |  |  |  |  |  |  |  |  |  |  |  |  |  |  |  |  |  |  |  |  |  |  |  |  |  |  |  |  |  |  |  |  |  |  |  |  |  |  |  |  |  |  |  |  |  |  |  |  |  |  |  |  |  |  |  |  |  |  |  |  |  |  |  |  |  |  |  |  |  |  |  |  |  |  |  |  |  |  |  |  |  |  |  |  |  |  |  |  |  |  |  |  |  |  |  |  |  |  |  |  |  |  |  |  |  |  |  |  |  |  |  |  |  |  |  |  |  |  |  |  |  |  |  |  |  |  |  |  |  |  |  |  |  |  |  |  |  |  |  |  |  |  |  |  |  |  |  |  |  |  |  |  |  |  |  |  |  |  |  |  |  |  |  |  |  |  |  |  |  |  |  |  |  |  |  |  |  |  |  |  |  |  |  |  |  |  |  |  |  |  |  |  |  |  |  |  |  |  |  |  |  |  |  |  |  |  |  |  |  |  |  |  |  |  |  |  |  |  |  |  |  |  |  |  |  |  |  |  |  |  |  |  |  |  |  |  |  |  |  |  |  |  |  |  |  |  |  |  |  |  |  |  |  |  |  |  |  |  |  |  |  |  |  |  |  |  |  |  |  |  |  |  |  |  |  |  |  |  |  |  |  |  |  |  |  |  |  |  |  |  |  |  |  |  |  |  |  |  |  |  |  |  |  |  |  |  |  |  |  |  |  |  |  |  |  |  |  |  |  |  |  |  |  |  |  |  |  |  |  |  |  |  |  |  |  |  |  |  |  |  |  |  |  |  |  |  |  |  |  |  |  |  |  |  |  |  |  |  |  |  |  |  |  |  |  |  |  |  |  |  |  |  |  |  |  |  |  |  |  |  |  |  |  |  |  |  |  |  |  |  |  |  |  |  |  |  |  |  |  |  |  |  |  |  |  |  |  |  |  |  |  |  |  |  |  |  |  |  |  |  |  |  |  |  |  |  |  |  |  |  |  |  |  |  |  |  |  |  |  |  |  |  |  |  |  |  |  |  |  |  |  |  |  |  |  |  |  |  |  |  |  |  |  |  |  |  |  |  |  |  |  |  |  |  |  |  |  |  |  |  |  |  |  |  |  |  |  |  |  |  |  |  |  |  |  |  |  |  |  |  |  |  |  |  |  |  |  |  |  |  |  |  |  |  |  |  |  |  |  |  |  |  |  |  |  |  |  |  |  |  |  |  |  |  |  |  |  |  |  |  |  |  |  |  |  |  |  |  |  |  |  |  |  |  |  |  |  |  |  |  |  |  |  |  |  |  |  |  |  |  |  |  |  |  |  |  |  |  |  |  |  |  |  |  |  |  |  |  |  |  |  |  |  |  |  |  |  |  |  |  |  |  |  |  |  |  |  |  |  |  |  |  |  |  |  |  |  |  |  |  |  |  |  |  |  |  |  |  |  |  |  |  |  |  |  |  |  |  |  |  |  |  |  |  |  |  |  |  |  |  |  |  |  |  |  |  |  |  |  |  |  |  |  |  |  |  |  |  |  |  |  |  |  |  |  |  |  |  |  |  |  |  |  |  |  |  |  |  |  |  |  |  |  |  |  |  |  |  |  |  |  |  |  |  |  |  |  |  |  |  |  |  |  |  |  |  |  |  |  |  |  |  |  |  |  |  |  |  |  |  |  |  |  |  |  |  |  |  |  |  |  |  |  |  |  |  |  |  |  |  |  |  |  |  |  |  |  |  |  |  |  |  |  |  |  |  |  |  |  |  |  |  |  |  |  |  |  |  |  |  |  |  |  |  |  |  |  |  |  |  |  |  |  |  |  |  |  |  |  |  |  |  |  |  |  |  |  |  |  |  |  |  |  |  |  |  |  |  |  |  |  |  |  |  |  |  |  |  |  |  |  |  |  |  |  |  |  |  |  |  |  |  |  |  |  |  |  |  |  |  |  |  |  |  |  |  |  |  |  |  |  |  |  |  |  |  |  |  |  |  |  |  |  |  |  |  |  |  |  |  |  |  |  |  |  |  |  |  |  |  |  |  |  |  |  |  |  |  |  |  |  |  |  |  |  |  |  |  |  |  |  |  |  |  |  |  |  |  |  |  |  |  |  |  |  |  |  |  |  |  |  |  |  |  |  |  |  |  |  |  |  |  |  |  |  |  |  |  |  |  |  |  |  |  |  |  |  |  |  |  |  |  |  |  |  |  |  |  |  |  |  |  |  |  |  |  |  |  |  |  |  |  |  |  |  |  |  |  |  |  |  |  |  |  |  |  |  |  |  |  |  |  |  |  |  |  |  |  |  |  |  |  |  |  |  |  |  |  |  |  |  |  |  |  |  |  |  |  |  |  |  |  |  |  |  |  |  |  |  |  |  |  |  |  |  |  |  |  |  |  |  |  |  |  |  |  |  |  |  |  |  |  |  |  |  |  |  |  |  |  |  |  |  |  |  |  |  |  |  |  |  |  |  |  |  |  |  |  |  |  |  |  |  |  |  |  |  |  |  |  |  |  |  |  |  |  |  |  |  |  |  |  |  |  |  |  |  |  |  |  |  |  |  |  |  |  |  |  |  |  |  |  |  |  |  |  |  |  |  |  |  |  |  |  |  |  |  |  |  |  |  |  |  |  |  |  |  |  |  |  |  |  |  |  |  |  |  |  |  |  |  |  |  |  |  |  |  |  |  |  |  |  |  |  |  |  |  |  |  |  |  |  |  |  |  |  |  |  |  |  |  |  |  |  |  |  |  |  |  |  |  |  |  |  |  |  |  |  |  |  |  |  |  |  |  |  |  |  |  |  |  |  |  |  |  |  |  |  |  |  |  |  |  |  |  |  |  |  |  |  |  |  |  |  |  |  |  |  |  |  |  |  |  |  |  |  |  |  |  |  |  |  |  |  |  |  |  |  |  |  |  |  |  |  |  |  |  |  |  |  |  |  |  |  |  |  |  |  |  |  |  |  |  |  |  |  |  |  |  |  |  |  |  |  |  |  |  |  |  |  |  |  |  |  |  |  |  |  |  |  |  |  |  |  |  |  |  |  |  |  |  |  |  |  |  |  |  |  |  |  |  |  |  |  |  |  |  |  |  |  |  |  |  |  |  |  |  |  |  |  |  |  |  |  |  |  |  |  |  |  |  |  |  |  |  |  |  |  |  |  |  |  |  |  |  |  |  |  |  |  |  |  |  |  |  |  |  |  |  |  |  |  |  |  |  |  |  |  |  |  |  |  |  |  |  |  |  |  |  |  |  |  |  |  |  |  |  |  |  |  |  |  |  |  |  |  |  |  |  |  |  |  |  |  |  |  |  |  |  |  |  |  |  |  |  |  |  |  |  |  |  |  |  |  |  |  |  |  |  |  |  |  |  |  |  |  |  |  |  |  |  |  |  |  |  |  |  |  |  |  |  |  |  |  |  |  |  |  |  |  |  |  |  |  |  |  |  |  |  |  |  |  |  |  |  |  |  |  |  |  |  |  |  |  |  |  |  |  |  |  |  |  |  |  |  |  |  |  |  |  |  |  |  |  |  |  |  |  |  |  |  |  |  |  |  |  |  |  |  |  |  |  |  |  |  |  |  |  |  |  |  |  |  |  |  |  |  |  |  |  |  |  |  |  |  |  |  |  |  |  |  |  |  |  |  |  |  |  |  |  |  |  |  |  |  |  |  |  |  |  |  |  |  |  |  |  |  |  |  |  |  |  |  |  |  |  |  |  |  |  |  |  |  |  |  |  |  |  |  |  |  |  |  |  |  |  |  |  |  |  |  |  |  |  |  |  |  |  |  |  |  |  |  |  |  |  |  |  |  |  |  |  |  |  |  |  |  |  |  |  |  |  |  |  |  |  |  |  |  |  |  |  |  |  |  |  |  |  |  |  |  |  |  |  |  |  |  |  |  |  |  |  |  |  |  |  |  |  |  |  |  |  |  |  |  |  |  |  |  |  |  |  |  |  |  |  |  |  |  |  |  |  |  |  |  |  |  |  |  |  |  |  |  |  |  |  |  |  |  |  |  |  |  |  |  |  |  |  |  |  |  |  |  |  |  |  |  |  |  |  |  |  |  |  |  |  |  |  |  |  |  |  |  |  |  |  |  |  |  |  |  |  |  |  |  |  |  |  |  |  |  |  |  |  |  |  |  |  |  |  |  |  |  |  |  |  |  |  |  |  |  |  |  |  |  |  |  |  |  |  |  |  |  |  |  |  |  |  |  |  |  |  |  |  |  |  |  |  |  |  |  |  |  |  |  |  |  |  |  |  |  |  |  |  |  |  |  |  |  |  |  |  |  |  |  |  |  |  |  |  |  |  |  |  |  |  |  |  |  |  |  |  |  |  |  |  |  |  |  |  |  |  |  |  |  |  |  |  |  |  |  |  |  |  |  |  |  |  |  |  |  |  |  |  |  |  |  |  |  |  |  |  |  |  |  |  |  |  |  |  |  |  |  |  |  |  |  |  |  |  |  |  |  |  |  |  |  |  |  |  |  |  |  |  |  |  |  |  |  |  |  |  |  |  |  |  |  |  |  |  |  |  |  |  |  |  |  |  |  |  |  |  |  |  |  |  |  |  |  |  |  |  |  |  |  |  |  |  |  |  |  |  |  |  |  |  |  |  |  |  |  |  |  |  |  |  |  |  |  |  |  |  |  |  |  |  |  |  |  |  |  |  |  |  |  |  |  |  |  |  |  |  |  |  |  |  |  |  |  |  |  |  |  |  |  |  |  |  |  |  |  |  |  |  |  |  |  |  |  |  |  |  |  |  |  |  |  |  |  |  |  |  |  |  |  |  |  |  |  |  |  |  |  |  |  |  |  |  |  |  |  |  |  |  |  |  |  |  |  |  |  |  |  |  |  |  |  |  |  |  |  |  |  |  |  |  |  |  |  |  |  |  |  |  |  |  |  |  |  |  |  |  |  |  |  |  |  |  |  |  |  |  |  |  |  |  |  |  |  |  |  |  |  |  |  |  |  |  |  |  |  |  |  |  |  |  |  |  |  |  |  |  |  |  |  |  |  |  |  |  |  |  |  |  |  |  |  |  |  |  |  |  |  |  |  |  |  |  |  |  |  |  |  |  |  |  |  |  |  |  |  |  |  |  |  |  |  |  |  |  |  |  |  |  |  |  |  |  |  |  |  |  |  |  |  |  |  |  |  |  |  |  |  |  |  |  |  |  |  |  |  |  |  |  |  |  |  |  |  |  |  |  |  |  |  |  |  |  |  |  |  |  |  |  |  |  |  |  |  |  |  |  |  |  |  |  |  |  |  |  |  |  |  |  |  |  |  |  |  |  |  |  |  |  |  |  |  |  |  |  |  |  |  |  |  |  |  |  |  |  |  |  |  |  |  |  |  |  |  |  |  |  |  |  |  |  |  |  |  |  |  |  |  |  |  |  |  |  |  |  |  |  |  |  |  |  |  |  |  |  |  |  |  |  |  |  |  |  |  |  |  |  |  |  |  |  |  |  |  |  |  |  |  |  |  |  |  |  |  |  |  |  |  |  |  |  |  |  |  |  |  |  |  |  |  |  |  |  |  |  |  |  |  |  |  |  |  |  |  |  |  |  |  |  |  |  |  |  |  |  |  |  |  |  |  |  |  |  |  |  |  |  |  |  |  |  |  |  |  |  |  |  |  |  |  |  |  |  |  |  |  |  |  |  |  |  |  |  |  |  |  |  |  |  |  |  |  |  |  |  |  |  |  |  |  |  |  |  |  |  |  |  |  |  |  |  |  |  |  |  |  |  |  |  |  |  |  |  |  |  |  |  |  |  |  |  |  |  |  |  |  |  |  |  |  |  |  |  |  |  |  |  |  |  |  |  |  |  |  |  |  |  |  |  |  |  |  |  |  |  |  |  |  |  |  |  |  |  |  |  |  |  |  |  |  |  |  |  |  |  |  |  |  |  |  |  |  |  |  |  |  |  |  |  |  |  |  |  |  |  |  |  |  |  |  |  |  |  |  |  |  |  |  |  |  |  |  |  |  |  |  |  |  |  |  |  |  |  |  |  |  |  |  |  |  |  |  |  |  |  |  |  |  |  |  |  |  |  |  |  |  |  |  |  |  |  |  |  |  |  |  |  |  |  |  |  |  |  |  |  |  |  |  |  |  |  |  |  |  |  |  |  |  |  |  |  |  |  |  |  |  |  |  |  |  |  |  |  |  |  |  |  |  |  |  |  |  |  |  |  |  |  |  |  |  |  |  |  |  |  |  |  |  |  |  |  |  |  |  |  |  |  |  |  |  |  |  |  |  |  |  |  |  |  |  |  |  |  |  |  |  |  |  |  |  |  |  |  |  |  |  |  |  |  |  |  |  |  |  |  |  |  |  |  |  |  |  |  |  |  |  |  |  |  |  |  |  |  |  |  |  |  |  |  |  |  |  |  |  |  |  |  |  |  |  |  |  |  |  |  |  |  |  |  |  |  |  |  |  |  |  |  |  |  |  |  |  |  |  |  |  |  |  |  |  |  |  |  |  |  |  |  |  |  |  |  |  |  |  |  |  |  |  |  |  |  |  |  |  |  |  |  |  |  |  |  |  |  |  |  |  |  |  |  |  |  |  |  |  |  |  |  |  |  |  |  |  |  |  |  |  |  |  |  |  |  |  |  |  |  |  |  |  |  |  |  |  |  |  |  |  |  |  |  |  |  |  |  |  |  |  |  |  |  |  |  |  |  |  |  |  |  |  |  |  |  |  |  |  |  |  |  |  |  |  |  |  |  |  |  |  |  |  |  |  |  |  |  |  |  |  |  |  |  |  |  |  |  |  |  |  |  |  |  |  |  |  |  |  |  |  |  |  |  |  |  |  |  |  |  |  |  |  |  |  |  |  |  |  |  |  |  |  |  |  |  |  |  |  |  |  |  |  |  |  |  |  |  |  |  |  |  |  |  |  |  |  |  |  |  |  |  |  |  |  |  |  |  |  |  |  |  |  |  |  |  |  |  |  |  |  |  |  |  |  |  |  |  |  |  |  |  |  |  |  |  |  |  |  |  |  |  |  |  |  |  |  |  |  |  |  |  |  |  |  |  |  |  |  |  |  |  |  |  |  |  |  |  |  |  |  |  |  |  |  |  |  |  |  |  |  |  |  |  |  |  |  |  |  |  |  |  |  |  |  |  |  |  |  |  |  |  |  |  |  |  |  |  |  |  |  |  |  |  |  |  |  |  |  |  |  |  |  |  |  |  |  |  |  |  |  |  |  |  |  |  |  |  |  |  |  |  |  |  |  |  |  |  |  |  |  |  |  |  |  |  |  |  |  |  |  |  |  |  |  |  |  |  |  |  |  |  |  |  |  |  |  |  |  |  |  |  |  |  |  |  |  |  |  |  |  |  |  |  |  |  |  |  |  |  |  |  |  |  |  |  |  |  |  |  |  |  |  |  |  |  |  |  |  |  |  |  |  |  |  |  |  |  |  |  |  |  |  |  |  |  |  |  |  |  |  |  |  |  |  |  |  |  |  |  |  |  |  |  |  |  |  |  |  |  |  |
| --- | --- | --- | --- | --- | --- | --- | --- | --- | --- | --- | --- | --- | --- | --- | --- | --- | --- | --- | --- | --- | --- | --- | --- | --- | --- | --- | --- | --- | --- | --- | --- | --- | --- | --- | --- | --- | --- | --- | --- | --- | --- | --- | --- | --- | --- | --- | --- | --- | --- | --- | --- | --- | --- | --- | --- | --- | --- | --- | --- | --- | --- | --- | --- | --- | --- | --- | --- | --- | --- | --- | --- | --- | --- | --- | --- | --- | --- | --- | --- | --- | --- | --- | --- | --- | --- | --- | --- | --- | --- | --- | --- | --- | --- | --- | --- | --- | --- | --- | --- | --- | --- | --- | --- | --- | --- | --- | --- | --- | --- | --- | --- | --- | --- | --- | --- | --- | --- | --- | --- | --- | --- | --- | --- | --- | --- | --- | --- | --- | --- | --- | --- | --- | --- | --- | --- | --- | --- | --- | --- | --- | --- | --- | --- | --- | --- | --- | --- | --- | --- | --- | --- | --- | --- | --- | --- | --- | --- | --- | --- | --- | --- | --- | --- | --- | --- | --- | --- | --- | --- | --- | --- | --- | --- | --- | --- | --- | --- | --- | --- | --- | --- | --- | --- | --- | --- | --- | --- | --- | --- | --- | --- | --- | --- | --- | --- | --- | --- | --- | --- | --- | --- | --- | --- | --- | --- | --- | --- | --- | --- | --- | --- | --- | --- | --- | --- | --- | --- | --- | --- | --- | --- | --- | --- | --- | --- | --- | --- | --- | --- | --- | --- | --- | --- | --- | --- | --- | --- | --- | --- | --- | --- | --- | --- | --- | --- | --- | --- | --- | --- | --- | --- | --- | --- | --- | --- | --- | --- | --- | --- | --- | --- | --- | --- | --- | --- | --- | --- | --- | --- | --- | --- | --- | --- | --- | --- | --- | --- | --- | --- | --- | --- | --- | --- | --- | --- | --- | --- | --- | --- | --- | --- | --- | --- | --- | --- | --- | --- | --- | --- | --- | --- | --- | --- | --- | --- | --- | --- | --- | --- | --- | --- | --- | --- | --- | --- | --- | --- | --- | --- | --- | --- | --- | --- | --- | --- | --- | --- | --- | --- | --- | --- | --- | --- | --- | --- | --- | --- | --- | --- | --- | --- | --- | --- | --- | --- | --- | --- | --- | --- | --- | --- | --- | --- | --- | --- | --- | --- | --- | --- | --- | --- | --- | --- | --- | --- | --- | --- | --- | --- | --- | --- | --- | --- | --- | --- | --- | --- | --- | --- | --- | --- | --- | --- | --- | --- | --- | --- | --- | --- | --- | --- | --- | --- | --- | --- | --- | --- | --- | --- | --- | --- | --- | --- | --- | --- | --- | --- | --- | --- | --- | --- | --- | --- | --- | --- | --- | --- | --- | --- | --- | --- | --- | --- | --- | --- | --- | --- | --- | --- | --- | --- | --- | --- | --- | --- | --- | --- | --- | --- | --- | --- | --- | --- | --- | --- | --- | --- | --- | --- | --- | --- | --- | --- | --- | --- | --- | --- | --- | --- | --- | --- | --- | --- | --- | --- | --- | --- | --- | --- | --- | --- | --- | --- | --- | --- | --- | --- | --- | --- | --- | --- | --- | --- | --- | --- | --- | --- | --- | --- | --- | --- | --- | --- | --- | --- | --- | --- | --- | --- | --- | --- | --- | --- | --- | --- | --- | --- | --- | --- | --- | --- | --- | --- | --- | --- | --- | --- | --- | --- | --- | --- | --- | --- | --- | --- | --- | --- | --- | --- | --- | --- | --- | --- | --- | --- | --- | --- | --- | --- | --- | --- | --- | --- | --- | --- | --- | --- | --- | --- | --- | --- | --- | --- | --- | --- | --- | --- | --- | --- | --- | --- | --- | --- | --- | --- | --- | --- | --- | --- | --- | --- | --- | --- | --- | --- | --- | --- | --- | --- | --- | --- | --- | --- | --- | --- | --- | --- | --- | --- | --- | --- | --- | --- | --- | --- | --- | --- | --- | --- | --- | --- | --- | --- | --- | --- | --- | --- | --- | --- | --- | --- | --- | --- | --- | --- | --- | --- | --- | --- | --- | --- | --- | --- | --- | --- | --- | --- | --- | --- | --- | --- | --- | --- | --- | --- | --- | --- | --- | --- | --- | --- | --- | --- | --- | --- | --- | --- | --- | --- | --- | --- | --- | --- | --- | --- | --- | --- | --- | --- | --- | --- | --- | --- | --- | --- | --- | --- | --- | --- | --- | --- | --- | --- | --- | --- | --- | --- | --- | --- | --- | --- | --- | --- | --- | --- | --- | --- | --- | --- | --- | --- | --- | --- | --- | --- | --- | --- | --- | --- | --- | --- | --- | --- | --- | --- | --- | --- | --- | --- | --- | --- | --- | --- | --- | --- | --- | --- | --- | --- | --- | --- | --- | --- | --- | --- | --- | --- | --- | --- | --- | --- | --- | --- | --- | --- | --- | --- | --- | --- | --- | --- | --- | --- | --- | --- | --- | --- | --- | --- | --- | --- | --- | --- | --- | --- | --- | --- | --- | --- | --- | --- | --- | --- | --- | --- | --- | --- | --- | --- | --- | --- | --- | --- | --- | --- | --- | --- | --- | --- | --- | --- | --- | --- | --- | --- | --- | --- | --- | --- | --- | --- | --- | --- | --- | --- | --- | --- | --- | --- | --- | --- | --- | --- | --- | --- | --- | --- | --- | --- | --- | --- | --- | --- | --- | --- | --- | --- | --- | --- | --- | --- | --- | --- | --- | --- | --- | --- | --- | --- | --- | --- | --- | --- | --- | --- | --- | --- | --- | --- | --- | --- | --- | --- | --- | --- | --- | --- | --- | --- | --- | --- | --- | --- | --- | --- | --- | --- | --- | --- | --- | --- | --- | --- | --- | --- | --- | --- | --- | --- | --- | --- | --- | --- | --- | --- | --- | --- | --- | --- | --- | --- | --- | --- | --- | --- | --- | --- | --- | --- | --- | --- | --- | --- | --- | --- | --- | --- | --- | --- | --- | --- | --- | --- | --- | --- | --- | --- | --- | --- | --- | --- | --- | --- | --- | --- | --- | --- | --- | --- | --- | --- | --- | --- | --- | --- | --- | --- | --- | --- | --- | --- | --- | --- | --- | --- | --- | --- | --- | --- | --- | --- | --- | --- | --- | --- | --- | --- | --- | --- | --- | --- | --- | --- | --- | --- | --- | --- | --- | --- | --- | --- | --- | --- | --- | --- | --- | --- | --- | --- | --- | --- | --- | --- | --- | --- | --- | --- | --- | --- | --- | --- | --- | --- | --- | --- | --- | --- | --- | --- | --- | --- | --- | --- | --- | --- | --- | --- | --- | --- | --- | --- | --- | --- | --- | --- | --- | --- | --- | --- | --- | --- | --- | --- | --- | --- | --- | --- | --- | --- | --- | --- | --- | --- | --- | --- | --- | --- | --- | --- | --- | --- | --- | --- | --- | --- | --- | --- | --- | --- | --- | --- | --- | --- | --- | --- | --- | --- | --- | --- | --- | --- | --- | --- | --- | --- | --- | --- | --- | --- | --- | --- | --- | --- | --- | --- | --- | --- | --- | --- | --- | --- | --- | --- | --- | --- | --- | --- | --- | --- | --- | --- | --- | --- | --- | --- | --- | --- | --- | --- | --- | --- | --- | --- | --- | --- | --- | --- | --- | --- | --- | --- | --- | --- | --- | --- | --- | --- | --- | --- | --- | --- | --- | --- | --- | --- | --- | --- | --- | --- | --- | --- | --- | --- | --- | --- | --- | --- | --- | --- | --- | --- | --- | --- | --- | --- | --- | --- | --- | --- | --- | --- | --- | --- | --- | --- | --- | --- | --- | --- | --- | --- | --- | --- | --- | --- | --- | --- | --- | --- | --- | --- | --- | --- | --- | --- | --- | --- | --- | --- | --- | --- | --- | --- | --- | --- | --- | --- | --- | --- | --- | --- | --- | --- | --- | --- | --- | --- | --- | --- | --- | --- | --- | --- | --- | --- | --- | --- | --- | --- | --- | --- | --- | --- | --- | --- | --- | --- | --- | --- | --- | --- | --- | --- | --- | --- | --- | --- | --- | --- | --- | --- | --- | --- | --- | --- | --- | --- | --- | --- | --- | --- | --- | --- | --- | --- | --- | --- | --- | --- | --- | --- | --- | --- | --- | --- | --- | --- | --- | --- | --- | --- | --- | --- | --- | --- | --- | --- | --- | --- | --- | --- | --- | --- | --- | --- | --- | --- | --- | --- | --- | --- | --- | --- | --- | --- | --- | --- | --- | --- | --- | --- | --- | --- | --- | --- | --- | --- | --- | --- | --- | --- | --- | --- | --- | --- | --- | --- | --- | --- | --- | --- | --- | --- | --- | --- | --- | --- | --- | --- | --- | --- | --- | --- | --- | --- | --- | --- | --- | --- | --- | --- | --- | --- | --- | --- | --- | --- | --- | --- | --- | --- | --- | --- | --- | --- | --- | --- | --- | --- | --- | --- | --- | --- | --- | --- | --- | --- | --- | --- | --- | --- | --- | --- | --- | --- | --- | --- | --- | --- | --- | --- | --- | --- | --- | --- | --- | --- | --- | --- | --- | --- | --- | --- | --- | --- | --- | --- | --- | --- | --- | --- | --- | --- | --- | --- | --- | --- | --- | --- | --- | --- | --- | --- | --- | --- | --- | --- | --- | --- | --- | --- | --- | --- | --- | --- | --- | --- | --- | --- | --- | --- | --- | --- | --- | --- | --- | --- | --- | --- | --- | --- | --- | --- | --- | --- | --- | --- | --- | --- | --- | --- | --- | --- | --- | --- | --- | --- | --- | --- | --- | --- | --- | --- | --- | --- | --- | --- | --- | --- | --- | --- | --- | --- | --- | --- | --- | --- | --- | --- | --- | --- | --- | --- | --- | --- | --- | --- | --- | --- | --- | --- | --- | --- | --- | --- | --- | --- | --- | --- | --- | --- | --- | --- | --- | --- | --- | --- | --- | --- | --- | --- | --- | --- | --- | --- | --- | --- | --- | --- | --- | --- | --- | --- | --- | --- | --- | --- | --- | --- | --- | --- | --- | --- | --- | --- | --- | --- | --- | --- | --- | --- | --- | --- | --- | --- | --- | --- | --- | --- | --- | --- | --- | --- | --- | --- | --- | --- | --- | --- | --- | --- | --- | --- | --- | --- | --- | --- | --- | --- | --- | --- | --- | --- | --- | --- | --- | --- | --- | --- | --- | --- | --- | --- | --- | --- | --- | --- | --- | --- | --- | --- | --- | --- | --- | --- | --- | --- | --- | --- | --- | --- | --- | --- | --- | --- | --- | --- | --- | --- | --- | --- | --- | --- | --- | --- | --- | --- | --- | --- | --- | --- | --- | --- | --- | --- | --- | --- | --- | --- | --- | --- | --- | --- | --- | --- | --- | --- | --- | --- | --- | --- | --- | --- | --- | --- | --- | --- | --- | --- | --- | --- | --- | --- | --- | --- | --- | --- | --- | --- | --- | --- | --- | --- | --- | --- | --- | --- | --- | --- | --- | --- | --- | --- | --- | --- | --- | --- | --- | --- | --- | --- | --- | --- | --- | --- | --- | --- | --- | --- | --- | --- | --- | --- | --- | --- | --- | --- | --- | --- | --- | --- | --- | --- | --- | --- | --- | --- | --- | --- | --- | --- | --- | --- | --- | --- | --- | --- | --- | --- | --- | --- | --- | --- | --- | --- | --- | --- | --- | --- | --- | --- | --- | --- | --- | --- | --- | --- | --- | --- | --- | --- | --- | --- | --- | --- | --- | --- | --- | --- | --- | --- | --- | --- | --- | --- | --- | --- | --- | --- | --- | --- | --- | --- | --- | --- | --- | --- | --- | --- | --- | --- | --- | --- | --- | --- | --- | --- | --- | --- | --- | --- | --- | --- | --- | --- | --- | --- | --- | --- | --- | --- | --- | --- | --- | --- | --- | --- | --- | --- | --- | --- | --- | --- | --- | --- | --- | --- | --- | --- | --- | --- | --- | --- | --- | --- | --- | --- | --- | --- | --- | --- | --- | --- | --- | --- | --- | --- | --- | --- | --- | --- | --- | --- | --- | --- | --- | --- | --- | --- | --- | --- | --- | --- | --- | --- | --- | --- | --- | --- | --- | --- | --- | --- | --- | --- | --- | --- | --- | --- | --- | --- | --- | --- | --- | --- | --- | --- | --- | --- | --- | --- | --- | --- | --- | --- | --- | --- | --- | --- | --- | --- | --- | --- | --- | --- | --- | --- | --- | --- | --- | --- | --- | --- | --- | --- | --- | --- | --- | --- | --- | --- | --- | --- | --- | --- | --- | --- | --- | --- | --- | --- | --- | --- | --- | --- | --- | --- | --- | --- | --- | --- | --- | --- | --- | --- | --- | --- | --- | --- | --- | --- | --- | --- | --- | --- | --- | --- | --- | --- | --- | --- | --- | --- | --- | --- | --- | --- | --- | --- | --- | --- | --- | --- | --- | --- | --- | --- | --- | --- | --- | --- | --- | --- | --- | --- | --- | --- | --- | --- | --- | --- | --- | --- | --- | --- | --- | --- | --- | --- | --- | --- | --- | --- | --- | --- | --- | --- | --- | --- | --- | --- | --- | --- | --- | --- | --- | --- | --- | --- | --- | --- | --- | --- | --- | --- | --- | --- | --- | --- | --- | --- | --- | --- | --- | --- | --- | --- | --- | --- | --- | --- | --- | --- | --- | --- | --- | --- | --- | --- | --- | --- | --- | --- | --- | --- | --- | --- | --- | --- | --- | --- | --- | --- | --- | --- | --- | --- | --- | --- | --- | --- | --- | --- | --- | --- | --- | --- | --- | --- | --- | --- | --- | --- | --- | --- | --- | --- | --- | --- | --- | --- | --- | --- | --- | --- | --- | --- | --- | --- | --- | --- | --- | --- | --- | --- | --- | --- | --- | --- | --- | --- | --- | --- | --- | --- | --- | --- | --- | --- | --- | --- | --- | --- | --- | --- | --- | --- | --- | --- | --- | --- | --- | --- | --- | --- | --- | --- | --- | --- | --- | --- | --- | --- | --- | --- | --- | --- | --- | --- | --- | --- | --- | --- | --- | --- | --- | --- | --- | --- | --- | --- | --- | --- | --- | --- | --- | --- | --- | --- | --- | --- | --- | --- | --- | --- | --- | --- | --- | --- | --- | --- | --- | --- | --- | --- | --- | --- | --- | --- | --- | --- | --- | --- | --- | --- | --- | --- | --- | --- | --- | --- | --- | --- | --- | --- | --- | --- | --- | --- | --- | --- | --- | --- | --- | --- | --- | --- | --- | --- | --- | --- | --- | --- | --- | --- | --- | --- | --- | --- | --- | --- | --- | --- | --- | --- | --- | --- | --- | --- | --- | --- | --- | --- | --- | --- | --- | --- | --- | --- | --- | --- | --- | --- | --- | --- | --- | --- | --- | --- | --- | --- | --- | --- | --- | --- | --- | --- | --- | --- | --- | --- | --- | --- | --- | --- | --- | --- | --- | --- | --- | --- | --- | --- | --- | --- | --- | --- | --- | --- | --- | --- | --- | --- | --- | --- | --- | --- | --- | --- | --- | --- | --- | --- | --- | --- | --- | --- | --- | --- | --- | --- | --- | --- | --- | --- | --- | --- | --- | --- | --- | --- | --- | --- | --- | --- | --- | --- | --- | --- | --- | --- | --- | --- | --- | --- | --- | --- | --- | --- | --- | --- | --- | --- | --- | --- | --- | --- | --- | --- | --- | --- | --- | --- | --- | --- | --- | --- | --- | --- | --- | --- | --- | --- | --- | --- | --- | --- | --- | --- | --- | --- | --- | --- | --- | --- | --- | --- | --- | --- | --- | --- | --- | --- | --- | --- | --- | --- | --- | --- | --- | --- | --- | --- | --- | --- | --- | --- | --- | --- | --- | --- | --- | --- | --- | --- | --- | --- | --- | --- | --- | --- | --- | --- | --- | --- | --- | --- | --- | --- | --- | --- | --- | --- | --- | --- | --- | --- | --- | --- | --- | --- | --- | --- | --- | --- | --- | --- | --- | --- | --- | --- | --- | --- | --- | --- | --- | --- | --- | --- | --- | --- | --- | --- | --- | --- | --- | --- | --- | --- | --- | --- | --- | --- | --- | --- | --- | --- | --- | --- | --- | --- | --- | --- | --- | --- | --- | --- | --- | --- | --- | --- | --- | --- | --- | --- | --- | --- | --- | --- | --- | --- | --- | --- | --- | --- | --- | --- | --- | --- | --- | --- | --- | --- | --- | --- | --- | --- | --- | --- | --- | --- | --- | --- | --- | --- | --- | --- | --- | --- | --- | --- | --- | --- | --- | --- | --- | --- | --- | --- | --- | --- | --- | --- | --- | --- | --- | --- | --- | --- | --- | --- | --- | --- | --- | --- | --- | --- | --- | --- | --- | --- | --- | --- | --- | --- | --- | --- | --- | --- | --- | --- | --- | --- | --- | --- | --- | --- | --- | --- | --- | --- | --- | --- | --- | --- | --- | --- | --- | --- | --- | --- | --- | --- | --- | --- | --- | --- | --- | --- | --- | --- | --- | --- | --- | --- | --- | --- | --- | --- | --- | --- | --- | --- | --- | --- | --- | --- | --- | --- | --- | --- | --- | --- | --- | --- | --- | --- | --- | --- | --- | --- | --- | --- | --- | --- | --- | --- | --- | --- | --- | --- | --- | --- | --- | --- | --- | --- | --- | --- | --- | --- | --- | --- | --- | --- | --- | --- | --- | --- | --- | --- | --- | --- | --- | --- | --- | --- | --- | --- | --- | --- | --- | --- | --- | --- | --- | --- | --- | --- | --- | --- | --- | --- | --- | --- | --- | --- | --- | --- | --- | --- | --- | --- | --- | --- | --- | --- | --- | --- | --- | --- | --- | --- | --- | --- | --- | --- | --- | --- | --- | --- | --- | --- | --- | --- | --- | --- | --- | --- | --- | --- | --- | --- | --- | --- | --- | --- | --- | --- | --- | --- | --- | --- | --- | --- | --- | --- | --- | --- | --- | --- | --- | --- | --- | --- | --- | --- | --- | --- | --- | --- | --- | --- | --- | --- | --- | --- | --- | --- | --- | --- | --- | --- | --- | --- | --- | --- | --- | --- | --- | --- | --- | --- | --- | --- | --- | --- | --- | --- | --- | --- | --- | --- | --- | --- | --- | --- | --- | --- | --- | --- | --- | --- | --- | --- | --- | --- | --- | --- | --- | --- | --- | --- | --- | --- | --- | --- | --- | --- | --- | --- | --- | --- | --- | --- | --- | --- | --- | --- | --- | --- | --- | --- | --- | --- | --- | --- | --- | --- | --- | --- | --- | --- | --- | --- | --- | --- | --- | --- | --- | --- | --- | --- | --- | --- | --- | --- | --- | --- | --- | --- | --- | --- | --- | --- | --- | --- | --- | --- | --- | --- | --- | --- | --- | --- | --- | --- | --- | --- | --- | --- | --- | --- | --- | --- | --- | --- | --- | --- | --- | --- | --- | --- | --- | --- | --- | --- | --- | --- | --- | --- | --- | --- | --- | --- | --- | --- | --- | --- | --- | --- | --- | --- | --- | --- | --- | --- | --- | --- | --- | --- | --- | --- | --- | --- | --- | --- | --- | --- | --- | --- | --- | --- | --- | --- | --- | --- | --- | --- | --- | --- | --- | --- | --- | --- | --- | --- | --- | --- | --- | --- | --- | --- | --- | --- | --- | --- | --- | --- | --- | --- | --- | --- | --- | --- | --- | --- | --- | --- | --- | --- | --- | --- | --- | --- | --- | --- | --- | --- | --- | --- | --- | --- | --- | --- | --- | --- | --- | --- | --- | --- | --- | --- | --- | --- | --- | --- | --- | --- | --- | --- | --- | --- | --- | --- | --- | --- | --- | --- | --- | --- | --- | --- | --- | --- | --- | --- | --- | --- | --- | --- | --- | --- | --- | --- | --- | --- | --- | --- | --- | --- | --- | --- | --- | --- | --- | --- | --- | --- | --- | --- | --- | --- | --- | --- | --- | --- | --- | --- | --- | --- | --- | --- | --- | --- | --- | --- | --- | --- | --- | --- | --- | --- | --- | --- | --- | --- | --- | --- | --- | --- | --- | --- | --- | --- | --- | --- | --- | --- | --- | --- | --- | --- | --- | --- | --- | --- | --- | --- | --- | --- | --- | --- | --- | --- | --- | --- | --- | --- | --- | --- | --- | --- | --- | --- | --- | --- | --- | --- | --- | --- | --- | --- | --- | --- | --- | --- | --- | --- | --- | --- | --- | --- | --- | --- | --- | --- | --- | --- | --- | --- | --- | --- | --- | --- | --- | --- | --- | --- | --- | --- | --- | --- | --- | --- | --- | --- | --- | --- | --- | --- | --- | --- | --- | --- | --- | --- | --- | --- | --- | --- | --- | --- | --- | --- | --- | --- | --- | --- | --- | --- | --- | --- | --- | --- | --- | --- | --- | --- | --- | --- | --- | --- | --- | --- | --- | --- | --- | --- | --- | --- | --- | --- | --- | --- | --- | --- | --- | --- | --- | --- | --- | --- | --- | --- | --- | --- | --- | --- | --- | --- | --- | --- | --- | --- | --- | --- | --- | --- | --- | --- | --- | --- | --- | --- | --- | --- | --- | --- | --- | --- | --- | --- | --- | --- | --- | --- | --- | --- | --- | --- | --- | --- | --- | --- | --- | --- | --- | --- | --- | --- | --- | --- | --- | --- | --- | --- | --- | --- | --- | --- | --- | --- | --- | --- | --- | --- | --- | --- | --- | --- | --- | --- | --- | --- | --- | --- | --- | --- | --- | --- | --- | --- | --- | --- | --- | --- | --- | --- | --- | --- | --- | --- | --- | --- | --- | --- | --- | --- | --- | --- | --- | --- | --- | --- | --- | --- | --- | --- | --- | --- | --- | --- | --- | --- | --- | --- | --- | --- | --- | --- | --- | --- | --- | --- | --- | --- | --- | --- | --- | --- | --- | --- | --- | --- | --- | --- | --- | --- | --- | --- | --- | --- | --- | --- | --- | --- | --- | --- | --- | --- | --- | --- | --- | --- | --- | --- | --- | --- | --- | --- | --- | --- | --- | --- | --- | --- | --- | --- | --- | --- | --- | --- | --- | --- | --- | --- | --- | --- | --- | --- | --- | --- | --- | --- | --- | --- | --- | --- | --- | --- | --- | --- | --- | --- | --- | --- | --- | --- | --- | --- | --- | --- | --- | --- | --- | --- | --- | --- | --- | --- | --- | --- | --- | --- | --- | --- | --- | --- | --- | --- | --- | --- | --- | --- | --- | --- | --- | --- | --- | --- | --- | --- | --- | --- | --- | --- | --- | --- | --- | --- | --- | --- | --- | --- | --- | --- | --- | --- | --- | --- | --- | --- | --- | --- | --- | --- | --- | --- | --- | --- | --- | --- | --- | --- | --- | --- | --- | --- | --- | --- | --- | --- | --- | --- | --- | --- | --- | --- | --- | --- | --- | --- | --- | --- | --- | --- | --- | --- | --- | --- | --- | --- | --- | --- | --- | --- | --- | --- | --- | --- | --- | --- | --- | --- | --- | --- | --- | --- | --- | --- | --- | --- | --- | --- | --- | --- | --- | --- | --- | --- | --- | --- | --- | --- | --- | --- | --- | --- | --- | --- | --- | --- | --- | --- | --- | --- | --- | --- | --- | --- | --- | --- | --- | --- | --- | --- | --- | --- | --- | --- | --- | --- | --- | --- | --- | --- | --- | --- | --- | --- | --- | --- | --- | --- | --- | --- | --- | --- | --- | --- | --- | --- | --- | --- | --- | --- | --- | --- | --- | --- | --- | --- | --- | --- | --- | --- | --- | --- | --- | --- | --- | --- | --- | --- | --- | --- | --- | --- | --- | --- | --- | --- | --- | --- | --- | --- | --- | --- | --- | --- | --- | --- | --- | --- | --- | --- | --- | --- | --- | --- | --- | --- | --- | --- | --- | --- | --- | --- | --- | --- | --- | --- | --- | --- | --- | --- | --- | --- | --- | --- | --- | --- | --- | --- | --- | --- | --- | --- | --- | --- | --- | --- | --- | --- | --- | --- | --- | --- | --- | --- | --- | --- | --- | --- | --- | --- | --- | --- | --- | --- | --- | --- | --- | --- | --- | --- | --- | --- | --- | --- | --- | --- | --- | --- | --- | --- | --- | --- | --- | --- | --- | --- | --- | --- | --- | --- | --- | --- | --- | --- | --- | --- | --- | --- | --- | --- | --- | --- | --- | --- | --- | --- | --- | --- | --- | --- | --- | --- | --- | --- | --- | --- | --- | --- | --- | --- | --- | --- | --- | --- | --- | --- | --- | --- | --- | --- | --- | --- | --- | --- | --- | --- | --- | --- | --- | --- | --- | --- | --- | --- | --- | --- | --- | --- | --- | --- | --- | --- | --- | --- | --- | --- | --- | --- | --- | --- | --- | --- | --- | --- | --- | --- | --- | --- | --- | --- | --- | --- | --- | --- | --- | --- | --- | --- | --- | --- | --- | --- | --- | --- | --- | --- | --- | --- | --- | --- | --- | --- | --- | --- | --- | --- | --- | --- | --- | --- | --- | --- | --- | --- | --- | --- | --- | --- | --- | --- | --- | --- | --- | --- | --- | --- | --- | --- | --- | --- | --- | --- | --- | --- | --- | --- | --- | --- | --- | --- | --- | --- | --- | --- | --- | --- | --- | --- | --- | --- | --- | --- | --- | --- | --- | --- | --- | --- | --- | --- | --- | --- | --- | --- | --- | --- | --- | --- | --- | --- | --- | --- | --- | --- | --- | --- | --- | --- | --- | --- | --- | --- | --- | --- | --- | --- | --- | --- | --- | --- | --- | --- | --- | --- | --- | --- | --- | --- | --- | --- | --- | --- | --- | --- | --- | --- | --- | --- | --- | --- | --- | --- | --- | --- | --- | --- | --- | --- | --- | --- | --- | --- | --- | --- | --- | --- | --- | --- | --- | --- | --- | --- | --- | --- | --- | --- | --- | --- | --- | --- | --- | --- | --- | --- | --- | --- | --- | --- | --- | --- | --- | --- | --- | --- | --- | --- | --- | --- | --- | --- | --- | --- | --- | --- | --- | --- | --- | --- | --- | --- | --- | --- | --- | --- | --- | --- | --- | --- | --- | --- | --- | --- | --- | --- | --- | --- | --- | --- | --- | --- | --- | --- | --- | --- | --- | --- | --- | --- | --- | --- | --- | --- | --- | --- | --- | --- | --- | --- | --- | --- | --- | --- | --- | --- | --- | --- | --- | --- | --- | --- | --- | --- | --- | --- | --- | --- | --- | --- | --- | --- | --- | --- | --- | --- | --- | --- | --- | --- | --- | --- | --- | --- | --- | --- | --- | --- | --- | --- | --- | --- | --- | --- | --- | --- | --- | --- | --- | --- | --- | --- | --- | --- | --- | --- | --- | --- | --- | --- | --- | --- | --- | --- | --- | --- | --- | --- | --- | --- | --- | --- | --- | --- | --- | --- | --- | --- | --- | --- | --- | --- | --- | --- | --- | --- | --- | --- | --- | --- | --- | --- | --- | --- | --- | --- | --- | --- | --- | --- | --- | --- | --- | --- | --- | --- | --- | --- | --- | --- | --- | --- | --- | --- | --- | --- | --- | --- | --- | --- | --- | --- | --- | --- | --- | --- | --- | --- | --- | --- | --- | --- | --- | --- | --- | --- | --- | --- | --- | --- | --- | --- | --- | --- | --- | --- | --- | --- | --- | --- | --- | --- | --- | --- | --- | --- | --- | --- | --- | --- | --- | --- | --- | --- | --- | --- | --- | --- | --- | --- | --- | --- | --- | --- | --- | --- | --- | --- | --- | --- | --- | --- | --- | --- | --- | --- | --- | --- | --- | --- | --- | --- | --- | --- | --- | --- | --- | --- | --- | --- | --- | --- | --- | --- | --- | --- | --- | --- | --- | --- | --- | --- | --- | --- | --- | --- | --- | --- | --- | --- | --- | --- | --- | --- | --- | --- | --- | --- | --- | --- | --- | --- | --- | --- | --- | --- | --- | --- | --- | --- | --- | --- | --- | --- | --- | --- | --- | --- | --- | --- | --- | --- | --- | --- | --- | --- | --- | --- | --- | --- | --- | --- | --- | --- | --- | --- | --- | --- | --- | --- | --- | --- | --- | --- | --- | --- | --- | --- | --- | --- | --- | --- | --- | --- | --- | --- | --- | --- | --- | --- | --- | --- | --- | --- | --- | --- | --- | --- | --- | --- | --- | --- | --- | --- | --- | --- | --- | --- | --- | --- | --- | --- | --- | --- | --- | --- | --- | --- | --- | --- | --- | --- | --- | --- | --- | --- | --- | --- | --- | --- | --- | --- | --- | --- | --- | --- | --- | --- | --- | --- | --- | --- | --- | --- | --- | --- | --- | --- | --- | --- | --- | --- | --- | --- | --- | --- | --- | --- | --- | --- | --- | --- | --- | --- | --- | --- | --- | --- | --- | --- | --- | --- | --- | --- | --- | --- | --- | --- | --- | --- | --- | --- | --- | --- | --- | --- | --- | --- | --- | --- | --- | --- | --- | --- | --- | --- | --- | --- | --- | --- | --- | --- | --- | --- | --- | --- | --- | --- | --- | --- | --- | --- | --- | --- | --- | --- | --- | --- | --- | --- | --- | --- | --- | --- | --- | --- | --- | --- | --- | --- | --- | --- | --- | --- | --- | --- | --- | --- | --- | --- | --- | --- | --- | --- | --- | --- | --- | --- | --- | --- | --- | --- | --- | --- | --- | --- | --- | --- | --- | --- | --- | --- | --- | --- | --- | --- | --- | --- | --- | --- | --- | --- | --- | --- | --- | --- | --- | --- | --- | --- | --- | --- | --- | --- | --- | --- | --- | --- | --- | --- | --- | --- | --- | --- | --- | --- | --- | --- | --- | --- | --- | --- | --- | --- | --- | --- | --- | --- | --- | --- | --- | --- | --- | --- | --- | --- | --- | --- | --- | --- | --- | --- | --- | --- | --- | --- | --- | --- | --- | --- | --- | --- | --- | --- | --- | --- | --- | --- | --- | --- | --- | --- | --- | --- | --- | --- | --- | --- | --- | --- | --- | --- | --- | --- | --- | --- | --- | --- | --- | --- | --- | --- | --- | --- | --- | --- | --- | --- | --- | --- | --- | --- | --- | --- | --- | --- | --- | --- | --- | --- | --- | --- | --- | --- | --- | --- | --- | --- | --- | --- | --- | --- | --- | --- | --- | --- | --- | --- | --- | --- | --- | --- | --- | --- | --- | --- | --- | --- | --- | --- | --- | --- | --- | --- | --- | --- | --- | --- | --- | --- | --- | --- | --- | --- | --- | --- | --- | --- | --- | --- | --- | --- | --- | --- | --- | --- | --- | --- | --- | --- | --- | --- | --- | --- | --- | --- | --- | --- | --- | --- | --- | --- | --- | --- | --- | --- | --- | --- | --- | --- | --- | --- | --- | --- | --- | --- | --- | --- | --- | --- | --- | --- | --- | --- | --- | --- | --- | --- | --- | --- | --- | --- | --- | --- | --- | --- | --- | --- | --- | --- | --- | --- | --- | --- | --- | --- | --- | --- | --- | --- | --- | --- | --- | --- | --- | --- | --- | --- | --- | --- | --- | --- | --- | --- | --- | --- | --- | --- | --- | --- | --- | --- | --- | --- | --- | --- | --- | --- | --- | --- | --- | --- | --- | --- | --- | --- | --- | --- | --- | --- | --- | --- | --- | --- | --- | --- | --- | --- | --- | --- | --- | --- | --- | --- | --- | --- | --- | --- | --- | --- | --- | --- | --- | --- | --- | --- | --- | --- | --- | --- | --- | --- | --- | --- | --- | --- | --- | --- | --- | --- | --- | --- | --- | --- | --- | --- | --- | --- | --- | --- | --- | --- | --- | --- | --- | --- | --- | --- | --- | --- | --- | --- | --- | --- | --- | --- | --- | --- | --- | --- | --- | --- | --- | --- | --- | --- | --- | --- | --- | --- | --- | --- | --- | --- | --- | --- | --- | --- | --- | --- | --- | --- | --- | --- | --- | --- | --- | --- | --- | --- | --- | --- | --- | --- | --- | --- | --- | --- | --- | --- | --- | --- | --- | --- | --- | --- | --- | --- | --- | --- | --- | --- | --- | --- | --- | --- | --- | --- | --- | --- | --- | --- | --- | --- | --- | --- | --- | --- | --- | --- | --- | --- | --- | --- | --- | --- | --- | --- | --- | --- | --- | --- | --- | --- | --- | --- | --- | --- | --- | --- | --- | --- | --- | --- | --- | --- | --- | --- | --- | --- | --- | --- | --- | --- | --- | --- | --- | --- | --- | --- | --- | --- | --- | --- | --- | --- | --- | --- | --- | --- | --- | --- | --- | --- | --- | --- | --- | --- | --- | --- | --- | --- | --- | --- | --- | --- | --- | --- | --- | --- | --- | --- | --- | --- | --- | --- | --- | --- | --- | --- | --- | --- | --- | --- | --- | --- | --- | --- | --- | --- | --- | --- | --- | --- | --- | --- | --- | --- | --- | --- | --- | --- | --- | --- | --- | --- | --- | --- | --- | --- | --- | --- | --- | --- | --- | --- | --- | --- | --- | --- | --- | --- | --- | --- | --- | --- | --- | --- | --- | --- | --- | --- | --- | --- | --- | --- | --- | --- | --- | --- | --- | --- | --- | --- | --- | --- | --- | --- | --- | --- | --- | --- | --- | --- | --- | --- | --- | --- | --- | --- | --- | --- | --- | --- | --- | --- | --- | --- | --- | --- | --- | --- | --- | --- | --- | --- | --- | --- | --- | --- | --- | --- | --- | --- | --- | --- | --- | --- | --- | --- | --- | --- | --- | --- | --- | --- | --- | --- | --- | --- | --- | --- | --- | --- | --- | --- | --- | --- | --- | --- | --- | --- | --- | --- | --- | --- | --- | --- | --- | --- | --- | --- | --- | --- | --- | --- | --- | --- | --- | --- | --- | --- | --- | --- | --- | --- | --- | --- | --- | --- | --- | --- | --- | --- | --- | --- | --- | --- | --- | --- | --- | --- | --- | --- | --- | --- | --- | --- | --- | --- | --- | --- | --- | --- | --- | --- | --- | --- | --- | --- | --- | --- | --- | --- | --- | --- | --- | --- | --- | --- | --- | --- | --- | --- | --- | --- |
| |  |  |  |  |  |  |  |  |  |  |  |  |  |  |  |  |  |  |  |  |  |  |  |  |  |  |  |  |  |  |  |  |  |  |  |  |  |  |  |  |  |  |  |  |  |  |  |  |  |  |  |  |  |  |  |  |  |  | | --- | --- | --- | --- | --- | --- | --- | --- | --- | --- | --- | --- | --- | --- | --- | --- | --- | --- | --- | --- | --- | --- | --- | --- | --- | --- | --- | --- | --- | --- | --- | --- | --- | --- | --- | --- | --- | --- | --- | --- | --- | --- | --- | --- | --- | --- | --- | --- | --- | --- | --- | --- | --- | --- | --- | --- | --- | --- | | G0VG43/1-437 | 1 | M | S | E | - | - | I | K | N | A | T | F | K | S | L | G | L | S | R | W | L | Q | E | A | L | Q | A | M | K | I | N | Q | P | T | A | I | Q | K | A | C | I | P | E | I | L | K | G | R | D | C | I | G | G | A | K | T | 53 | | Q6CXW0/1-435 | 1 | - | - | - | - | - | M | S | N | S | E | F | K | S | L | G | C | S | K | W | L | V | E | A | L | N | A | M | K | I | V | Q | P | T | A | I | Q | K | A | C | I | P | E | I | L | K | G | R | D | C | I | G | G | A | N | T | 50 | | Q6FQZ0/1-437 | 1 | M | T | E | E | V | S | R | K | Q | N | F | R | Q | L | G | L | S | K | W | L | V | E | S | L | D | A | M | R | I | R | T | P | T | A | I | Q | S | G | C | I | P | E | I | L | K | G | R | D | C | I | G | G | A | K | T | 55 | | Q756G5/1-435 | 1 | - | M | S | - | - | D | S | N | S | T | F | K | D | L | G | V | A | K | W | L | A | E | A | L | N | S | M | K | I | T | Q | P | T | T | I | Q | K | A | C | I | P | E | I | L | A | G | R | D | C | I | G | G | A | K | T | 52 | | A7TK63/1-431 | 1 | - | - | - | - | - | - | - | M | Q | D | F | K | S | L | G | L | S | R | W | L | V | E | S | L | N | A | M | R | I | T | H | P | T | A | I | Q | K | H | C | I | P | E | I | L | K | G | R | D | C | I | G | G | A | K | T | 48 | | C5E1Y4/1-433 | 1 | - | - | - | - | - | - | M | S | S | E | F | A | T | L | G | I | S | K | W | L | V | E | A | L | Q | A | M | K | I | T | Q | P | T | A | I | Q | K | A | C | I | P | Q | I | L | Q | G | K | D | C | I | G | G | A | K | T | 49 | | C5DTV7/1-435 | 1 | M | S | E | - | - | G | S | V | K | S | F | K | I | L | G | V | S | K | W | L | V | E | T | L | N | A | M | K | I | S | Q | P | T | T | I | Q | S | A | C | I | P | E | I | L | K | G | R | D | C | I | G | G | A | K | T | 53 | | Kwal\_56.24760/1-433 | 1 | - | - | - | - | - | - | M | S | D | T | F | S | N | L | G | V | S | K | W | L | T | E | A | L | R | A | M | K | I | D | H | P | T | A | I | Q | K | A | C | I | P | Q | I | L | K | G | K | D | C | I | G | G | A | K | T | 49 | | Sbay\_599.21/1-412 | 1 | - | - | - | - | - | - | - | - | - | - | - | - | - | - | - | - | - | - | - | - | - | - | - | - | - | - | M | K | I | T | Q | P | T | A | I | Q | K | A | C | I | P | K | I | L | E | G | R | D | C | I | G | G | A | K | T | 29 | | SAKL0H02178g/1-435 | 1 | - | M | V | - | - | E | V | P | K | S | F | S | S | L | G | V | A | K | W | L | V | E | A | L | N | A | M | K | I | T | Q | P | T | A | I | Q | R | T | C | I | P | E | I | L | G | G | R | D | C | I | G | G | A | K | T | 52 | | P38719/1-431 | 1 | - | - | - | - | - | - | - | M | A | D | F | K | S | L | G | L | S | K | W | L | T | E | S | L | R | A | M | K | I | T | Q | P | T | A | I | Q | K | A | C | I | P | K | I | L | E | G | R | D | C | I | G | G | A | K | T | 48 | |  | | G0VG43/1-437 | 54 | G | S | G | K | T | V | A | F | A | A | P | M | L | T | K | W | S | E | D | P | S | G | M | F | G | V | V | L | T | P | T | R | E | L | A | M | Q | I | A | E | Q | F | T | A | L | G | S | S | M | N | I | R | V | A | L | 108 | | Q6CXW0/1-435 | 51 | G | S | G | K | T | I | A | F | A | A | P | M | L | T | K | W | S | E | D | P | Q | G | M | F | G | I | V | L | T | P | T | R | E | L | A | M | Q | I | A | E | Q | F | T | A | F | G | S | A | M | N | I | R | V | A | I | 105 | | Q6FQZ0/1-437 | 56 | G | S | G | K | T | I | A | F | A | G | P | M | L | T | Q | W | S | E | D | P | T | G | M | F | G | I | V | L | T | P | T | R | E | L | A | M | Q | I | A | E | Q | F | T | A | L | G | S | Y | M | N | I | R | V | A | L | 110 | | Q756G5/1-435 | 53 | G | S | G | K | T | I | A | F | A | A | P | M | L | T | K | W | S | A | D | P | C | G | M | F | G | I | V | L | T | P | T | R | E | L | A | M | Q | I | A | E | Q | F | T | A | L | G | S | V | M | N | I | R | V | A | L | 107 | | A7TK63/1-431 | 49 | G | S | G | K | T | I | A | F | A | G | P | M | L | S | Q | W | S | D | D | P | S | G | M | F | G | V | V | L | T | P | T | R | E | L | A | I | Q | I | A | E | Q | F | T | A | L | G | S | S | M | N | I | R | V | C | L | 103 | | C5E1Y4/1-433 | 50 | G | S | G | K | T | I | A | F | G | A | P | M | L | T | K | W | S | E | D | P | S | G | M | F | G | V | V | L | T | P | T | R | E | L | A | M | Q | I | A | E | Q | F | T | A | L | G | S | S | M | N | I | R | V | A | L | 104 | | C5DTV7/1-435 | 54 | G | S | G | K | T | I | A | F | G | A | P | M | L | T | K | W | S | E | D | P | C | G | M | F | G | V | V | L | T | P | T | R | E | L | A | M | Q | I | A | E | Q | F | T | A | L | G | S | N | M | N | I | R | V | S | I | 108 | | Kwal\_56.24760/1-433 | 50 | G | S | G | K | T | I | A | F | A | A | P | M | L | T | K | W | S | E | D | P | S | G | M | F | G | V | V | L | T | P | T | R | E | L | A | M | Q | I | A | E | Q | F | T | A | L | G | S | S | M | N | I | R | V | A | L | 104 | | Sbay\_599.21/1-412 | 30 | G | S | G | K | T | I | A | F | A | G | P | M | L | T | K | W | S | E | D | P | C | G | M | F | G | V | V | L | T | P | T | R | E | L | A | M | Q | I | A | E | Q | F | T | A | L | G | S | N | M | N | I | R | V | S | V | 84 | | SAKL0H02178g/1-435 | 53 | G | S | G | K | T | I | A | F | A | A | P | M | L | T | S | W | S | E | D | P | S | G | M | F | G | V | I | L | T | P | T | R | E | L | A | M | Q | I | A | E | Q | F | T | A | L | G | S | S | M | N | I | K | V | A | L | 107 | | P38719/1-431 | 49 | G | S | G | K | T | I | A | F | A | G | P | M | L | T | K | W | S | E | D | P | S | G | M | F | G | V | V | L | T | P | T | R | E | L | A | M | Q | I | A | E | Q | F | T | A | L | G | S | S | M | N | I | R | V | S | V | 103 | |  | | G0VG43/1-437 | 109 | V | V | G | G | E | S | I | V | D | Q | A | I | Q | L | Q | K | K | P | H | F | I | I | A | T | P | G | R | L | A | H | H | I | M | S | S | G | E | D | T | I | G | G | L | K | R | A | K | F | L | V | L | D | E | A | D | 163 | | Q6CXW0/1-435 | 106 | V | V | G | G | E | S | I | V | Q | Q | A | I | E | L | Q | K | R | P | H | F | I | I | A | T | P | G | R | L | A | H | H | V | L | N | S | G | E | D | T | I | G | G | L | K | R | V | K | F | L | V | L | D | E | A | D | 160 | | Q6FQZ0/1-437 | 111 | V | V | G | G | E | S | I | V | D | Q | A | L | Q | L | Q | R | K | P | H | F | I | I | A | T | P | G | R | L | A | H | H | I | L | N | S | G | D | D | T | V | G | G | L | K | R | V | K | Y | L | V | L | D | E | A | D | 165 | | Q756G5/1-435 | 108 | V | V | G | G | E | D | I | V | S | Q | A | L | E | L | Q | R | K | P | H | F | I | I | A | T | P | G | R | L | A | H | H | I | M | H | S | G | E | D | T | I | G | G | L | K | R | V | R | Y | L | V | L | D | E | A | D | 162 | | A7TK63/1-431 | 104 | V | V | G | G | E | S | I | V | K | Q | A | L | E | L | Q | K | K | P | H | F | I | I | A | T | P | G | R | L | A | H | H | I | L | S | S | G | E | E | V | V | G | G | L | S | R | V | K | Y | L | V | L | D | E | A | D | 158 | | C5E1Y4/1-433 | 105 | V | V | G | G | E | S | I | V | D | Q | A | I | N | L | Q | R | K | P | H | F | I | V | A | T | P | G | R | M | A | H | H | I | M | N | S | G | E | D | T | I | G | G | L | K | R | A | K | F | L | V | L | D | E | A | D | 159 | | C5DTV7/1-435 | 109 | I | V | G | G | E | D | I | V | K | Q | G | L | E | L | Q | R | K | P | H | F | I | I | A | T | P | G | R | L | A | H | H | I | L | N | S | G | D | D | T | V | G | G | L | I | R | T | K | F | L | V | L | D | E | A | D | 163 | | Kwal\_56.24760/1-433 | 105 | V | V | G | G | E | S | I | V | D | Q | A | I | A | L | Q | R | K | P | H | F | I | I | A | T | P | G | R | L | A | H | H | V | M | N | S | G | E | D | T | I | G | G | L | K | R | V | K | F | L | V | L | D | E | A | D | 159 | | Sbay\_599.21/1-412 | 85 | V | V | G | G | E | S | I | V | K | Q | A | L | D | L | Q | R | K | P | H | F | I | I | A | T | P | G | R | L | A | H | H | I | M | S | S | G | E | D | T | V | G | G | L | M | R | A | K | Y | L | V | L | D | E | A | D | 139 | | SAKL0H02178g/1-435 | 108 | V | V | G | G | E | S | I | V | Q | Q | A | I | E | L | Q | K | K | P | H | F | I | V | A | T | P | G | R | L | A | H | H | I | M | N | S | G | D | D | T | I | G | G | L | R | R | V | K | Y | L | V | L | D | E | A | D | 162 | | P38719/1-431 | 104 | I | V | G | G | E | S | I | V | Q | Q | A | L | D | L | Q | R | K | P | H | F | I | I | A | T | P | G | R | L | A | H | H | I | M | S | S | G | D | D | T | V | G | G | L | M | R | A | K | Y | L | V | L | D | E | A | D | 158 | |  | | G0VG43/1-437 | 164 | S | L | L | T | D | T | F | A | S | D | L | A | T | C | I | G | A | L | P | S | K | D | K | R | Q | T | L | L | F | T | A | T | I | T | D | Q | V | R | A | L | E | D | A | P | I | Q | E | G | K | P | P | L | F | T | Y | 218 | | Q6CXW0/1-435 | 161 | I | L | L | T | E | T | F | S | K | D | L | A | T | C | V | S | I | L | P | P | K | N | K | R | Q | N | L | L | F | T | A | T | M | T | D | Q | V | K | A | L | S | D | A | P | Q | T | E | G | K | P | P | V | F | T | F | 215 | | Q6FQZ0/1-437 | 166 | I | L | L | T | E | T | F | S | N | D | L | K | T | C | V | G | A | L | P | P | K | E | K | R | Q | T | L | L | F | T | A | T | I | T | D | Q | V | R | A | L | Q | D | A | P | V | Q | K | G | K | Q | P | L | F | C | Y | 220 | | Q756G5/1-435 | 163 | I | L | L | T | D | T | F | S | D | A | L | A | T | C | V | Q | I | L | P | P | K | E | K | R | Q | N | L | L | F | T | A | T | M | T | D | Q | V | L | A | L | K | D | A | P | P | T | S | G | K | P | P | L | F | S | F | 217 | | A7TK63/1-431 | 159 | L | I | L | T | Q | T | F | A | A | D | L | S | T | C | I | A | K | L | P | P | K | Q | K | R | Q | T | L | L | F | T | A | T | I | T | D | Q | V | R | A | L | Q | N | A | P | A | Q | D | S | K | P | P | L | F | A | Y | 213 | | C5E1Y4/1-433 | 160 | I | L | L | T | D | T | F | S | E | H | L | A | T | C | I | S | I | L | P | P | K | E | K | R | Q | T | L | L | F | T | A | T | V | T | D | Q | V | K | A | L | Q | N | A | P | A | A | E | G | K | P | P | L | F | S | Y | 214 | | C5DTV7/1-435 | 164 | S | L | L | T | G | T | F | A | K | D | L | A | I | C | I | G | A | L | P | P | K | N | K | R | Q | T | L | L | F | T | A | T | V | T | D | Q | V | R | A | L | E | N | A | P | - | S | E | G | K | P | P | L | F | T | Y | 217 | | Kwal\_56.24760/1-433 | 160 | I | L | L | T | D | T | F | T | E | H | L | A | I | C | I | G | I | L | P | P | K | E | R | R | Q | N | L | L | F | T | A | T | V | T | D | Q | V | K | M | L | Q | N | A | P | A | T | E | G | K | P | P | L | F | S | Y | 214 | | Sbay\_599.21/1-412 | 140 | I | L | L | T | S | T | F | A | N | H | L | A | T | C | I | G | A | L | P | P | K | D | K | R | Q | T | L | L | F | T | A | T | I | T | D | Q | V | R | S | L | Q | D | A | P | V | Q | G | N | K | P | P | L | F | A | Y | 194 | | SAKL0H02178g/1-435 | 163 | I | L | L | T | N | T | F | S | E | D | L | A | T | C | V | S | I | L | P | P | K | N | K | R | Q | N | L | L | F | T | A | T | M | T | D | Q | V | K | A | L | Q | N | A | P | N | Q | E | G | K | P | P | V | F | S | Y | 217 | | P38719/1-431 | 159 | I | L | L | T | S | T | F | A | D | H | L | A | T | C | I | S | A | L | P | P | K | D | K | R | Q | T | L | L | F | T | A | T | I | T | D | Q | V | K | S | L | Q | N | A | P | V | Q | K | G | K | P | P | L | F | A | Y | 213 | |  | | G0VG43/1-437 | 219 | Q | V | E | S | V | D | K | V | A | I | P | S | T | L | K | T | E | Y | I | L | V | P | E | H | V | K | E | A | Y | L | Y | Q | L | L | T | C | E | T | Y | K | D | S | S | A | I | I | F | V | N | R | T | M | T | A | E | 273 | | Q6CXW0/1-435 | 216 | E | V | E | S | V | D | N | V | A | I | P | K | T | L | E | T | T | Y | L | L | V | P | E | H | V | K | E | S | Y | L | Y | Q | I | L | T | S | E | K | Y | V | K | S | S | C | I | I | F | V | N | R | T | V | T | A | E | 270 | | Q6FQZ0/1-437 | 221 | E | V | E | N | V | D | N | V | A | I | P | S | T | L | N | T | E | Y | V | L | V | P | E | H | V | K | E | A | Y | L | Y | Q | L | L | T | C | E | S | Y | A | N | S | T | A | I | I | F | V | N | R | T | T | A | A | E | 275 | | Q756G5/1-435 | 218 | H | V | E | N | L | D | D | L | A | V | P | A | S | L | Q | T | T | Y | L | L | V | P | E | H | V | K | E | A | Y | L | Y | Q | V | L | S | S | E | E | Y | K | S | K | S | A | I | V | F | V | N | R | T | I | S | A | E | 272 | | A7TK63/1-431 | 214 | E | V | E | N | V | D | N | V | A | V | P | S | T | L | K | L | E | Y | L | L | V | P | E | H | V | K | E | A | Y | L | Y | Q | L | L | T | C | E | D | Y | K | D | S | S | V | I | V | F | V | N | R | T | T | A | A | E | 268 | | C5E1Y4/1-433 | 215 | E | V | E | S | M | D | K | V | A | I | P | S | T | L | K | T | T | Y | L | L | V | P | E | Q | V | K | E | A | Y | L | Y | Q | I | L | T | N | A | T | Y | N | E | S | S | A | I | I | F | V | N | R | T | V | T | A | E | 269 | | C5DTV7/1-435 | 218 | E | V | A | S | M | D | K | V | A | I | P | S | S | L | K | T | E | Y | I | L | V | P | E | Y | V | K | E | A | Y | L | Y | Q | L | L | T | C | E | D | Y | K | D | S | T | A | M | V | F | V | N | R | T | M | A | A | E | 272 | | Kwal\_56.24760/1-433 | 215 | E | V | E | S | M | D | K | V | A | I | P | S | T | L | K | T | S | Y | L | L | V | P | E | Q | V | K | E | A | Y | L | Y | Q | I | L | T | S | A | A | Y | I | D | S | S | A | I | I | F | V | N | R | T | V | T | A | E | 269 | | Sbay\_599.21/1-412 | 195 | Q | V | E | S | V | D | D | V | A | I | P | S | T | L | K | T | E | Y | I | L | V | P | E | H | V | K | E | A | Y | L | Y | Q | L | L | T | C | E | E | Y | E | N | K | T | A | I | V | F | V | N | R | T | M | S | A | E | 249 | | SAKL0H02178g/1-435 | 218 | Q | V | E | S | V | D | K | L | A | I | P | S | T | L | K | T | E | Y | L | L | V | P | E | H | V | K | E | A | Y | L | Y | Q | I | L | T | S | E | D | Y | E | N | S | S | A | I | I | F | V | N | R | T | V | T | A | E | 272 | | P38719/1-431 | 214 | Q | V | E | S | V | D | N | V | A | I | P | S | T | L | K | I | E | Y | I | L | V | P | E | H | V | K | E | A | Y | L | Y | Q | L | L | T | C | E | E | Y | E | N | K | T | A | I | I | F | V | N | R | T | M | T | A | E | 268 | |  | | G0VG43/1-437 | 274 | I | L | R | R | T | L | Y | H | L | E | V | R | V | A | S | L | H | S | Q | M | P | Q | Q | E | R | T | N | S | L | H | R | F | R | A | N | V | A | R | V | L | I | A | T | D | V | A | S | R | G | L | D | I | P | T | V | 328 | | Q6CXW0/1-435 | 271 | I | L | R | R | T | L | K | S | L | D | V | R | V | T | S | L | H | S | Q | M | P | Q | Q | E | R | T | N | S | V | Q | R | F | R | A | Q | A | A | R | V | L | I | A | T | D | V | A | S | R | G | L | D | I | P | I | V | 325 | | Q6FQZ0/1-437 | 276 | V | L | R | R | T | L | K | A | L | D | V | R | V | A | S | L | H | S | Q | M | P | Q | Q | E | R | T | N | S | M | H | R | F | R | A | N | A | A | R | V | L | I | A | T | D | V | A | S | R | G | L | D | I | P | T | V | 330 | | Q756G5/1-435 | 273 | I | L | R | R | M | L | M | Q | L | E | I | R | V | T | S | L | H | S | Q | M | P | Q | R | E | R | T | N | S | L | Q | R | F | R | A | N | A | A | R | V | L | I | A | T | D | V | A | S | R | G | L | D | I | P | A | V | 327 | | A7TK63/1-431 | 269 | V | L | R | R | T | L | R | S | L | E | V | R | V | A | S | L | H | S | Q | M | P | Q | S | E | R | I | N | S | L | Q | R | F | R | A | N | A | A | R | V | L | I | A | T | D | V | A | A | R | G | L | D | I | P | T | V | 323 | | C5E1Y4/1-433 | 270 | I | L | R | R | T | L | K | Q | L | D | V | R | V | A | S | L | H | S | Q | M | P | Q | Q | E | R | T | N | S | L | H | R | F | R | A | N | A | A | R | V | L | I | A | T | D | V | A | S | R | G | L | D | I | P | T | V | 324 | | C5DTV7/1-435 | 273 | I | L | R | R | T | L | Y | A | M | G | V | R | V | T | S | L | H | S | Q | M | P | Q | Q | E | R | T | N | S | L | H | R | F | R | A | N | A | A | R | V | L | I | A | T | D | V | A | S | R | G | L | D | I | P | T | V | 327 | | Kwal\_56.24760/1-433 | 270 | I | L | R | R | T | L | K | Q | L | D | V | R | V | A | S | L | H | S | Q | M | P | Q | Q | E | R | T | N | S | L | H | R | F | R | A | N | A | A | R | V | L | I | A | T | D | V | A | S | R | G | L | D | I | P | T | V | 324 | | Sbay\_599.21/1-412 | 250 | I | L | R | R | T | L | K | Q | L | E | V | R | V | A | S | L | H | S | Q | M | P | Q | Q | E | R | T | N | S | L | H | R | F | R | A | N | A | A | R | V | L | I | A | T | D | V | A | S | R | G | L | D | I | P | T | V | 304 | | SAKL0H02178g/1-435 | 273 | I | L | R | R | T | L | K | G | L | D | V | R | V | A | S | L | H | S | Q | M | P | Q | Q | E | R | T | N | S | L | H | R | F | R | A | N | A | A | R | V | L | I | A | T | D | V | A | S | R | G | L | D | I | P | S | V | 327 | | P38719/1-431 | 269 | I | L | R | R | T | L | K | Q | L | E | V | R | V | A | S | L | H | S | Q | M | P | Q | Q | E | R | T | N | S | L | H | R | F | R | A | N | A | A | R | I | L | I | A | T | D | V | A | S | R | G | L | D | I | P | T | V | 323 | |  | | G0VG43/1-437 | 329 | E | L | V | I | N | Y | D | I | P | A | D | P | D | T | F | I | H | R | A | G | R | T | A | R | A | G | R | S | G | D | A | I | S | F | V | T | Q | R | D | V | S | R | I | E | A | I | E | K | R | I | N | K | K | M | T | 383 | | Q6CXW0/1-435 | 326 | E | L | V | V | N | Y | D | I | P | G | N | P | D | T | F | I | H | R | A | G | R | T | A | R | A | G | R | H | G | E | S | L | C | F | V | T | E | K | D | I | Q | R | V | E | A | I | E | E | R | I | N | K | K | M | E | 380 | | Q6FQZ0/1-437 | 331 | E | L | V | I | N | Y | D | I | P | S | D | P | D | T | F | I | H | R | S | G | R | T | A | R | A | G | R | K | G | D | A | I | S | F | I | T | Q | R | D | V | S | R | I | E | A | I | E | A | R | I | N | M | K | M | T | 385 | | Q756G5/1-435 | 328 | Q | L | V | V | N | Y | D | I | P | A | N | P | D | T | Y | I | H | R | A | G | R | T | A | R | A | G | R | G | G | E | A | L | S | F | I | A | P | K | D | V | S | R | I | Q | A | I | E | E | R | I | G | K | K | M | D | 382 | | A7TK63/1-431 | 324 | E | L | V | I | N | Y | D | I | P | Q | D | P | D | T | F | I | H | R | S | G | R | T | A | R | A | G | R | S | G | D | A | I | S | F | V | T | P | R | D | V | S | R | I | E | A | I | E | E | R | I | N | K | K | M | D | 378 | | C5E1Y4/1-433 | 325 | Q | L | V | I | N | Y | D | I | S | S | N | P | D | T | F | I | H | R | A | G | R | T | A | R | A | G | R | S | G | E | S | I | C | F | V | A | P | R | D | V | S | R | I | E | A | I | E | E | R | I | N | K | K | M | E | 379 | | C5DTV7/1-435 | 328 | Q | L | V | V | N | Y | D | I | P | S | D | P | D | V | F | I | H | R | S | G | R | T | A | R | A | G | R | R | G | D | A | I | S | F | I | T | Q | R | D | I | S | R | I | Q | A | I | E | E | R | I | N | K | K | M | G | 382 | | Kwal\_56.24760/1-433 | 325 | Q | L | V | V | N | Y | D | I | S | A | N | P | D | T | F | I | H | R | A | G | R | T | A | R | A | G | R | K | G | E | S | I | C | F | V | A | Q | R | D | V | S | R | I | Q | A | I | E | E | R | I | N | K | K | M | E | 379 | | Sbay\_599.21/1-412 | 305 | E | L | V | V | N | Y | D | I | P | S | D | P | D | V | F | I | H | R | S | G | R | T | A | R | A | G | R | D | G | D | A | I | S | F | V | T | Q | R | D | V | S | R | I | E | A | I | E | D | R | I | N | K | K | M | T | 359 | | SAKL0H02178g/1-435 | 328 | A | L | V | V | N | Y | D | I | P | A | N | P | D | T | F | I | H | R | A | G | R | T | A | R | A | G | R | S | G | E | S | I | C | F | V | T | Q | R | D | I | T | R | I | E | S | I | E | E | R | I | N | K | K | M | D | 382 | | P38719/1-431 | 324 | E | L | V | V | N | Y | D | I | P | S | D | P | D | V | F | I | H | R | S | G | R | T | A | R | A | G | R | I | G | D | A | I | S | F | V | T | Q | R | D | V | S | R | I | Q | A | I | E | D | R | I | N | K | K | M | T | 378 | |  | | G0VG43/1-437 | 384 | E | S | D | K | V | H | D | T | A | V | I | R | K | A | L | T | K | V | T | K | A | K | R | E | S | L | M | A | M | E | K | E | N | F | G | E | R | K | R | V | Q | K | R | K | E | L | A | S | K | G | F | R | R | - | D | 437 | | Q6CXW0/1-435 | 381 | E | F | T | D | V | G | D | T | A | V | I | R | K | S | L | T | K | V | T | A | A | K | R | E | S | L | M | A | M | D | K | E | G | F | G | E | R | R | K | L | Q | K | R | K | N | E | S | K | E | K | T | H | R | R | T | 435 | | Q6FQZ0/1-437 | 386 | E | C | D | K | V | H | D | T | A | V | I | R | K | A | L | T | K | V | S | K | A | K | R | E | A | L | M | A | M | E | K | E | N | F | G | E | R | R | K | L | Q | K | R | K | T | Q | K | D | G | K | - | R | V | - | - | 437 | | Q756G5/1-435 | 383 | E | F | T | K | V | G | D | T | A | L | I | R | K | S | L | N | K | V | T | V | A | K | R | E | S | L | M | A | M | D | K | E | G | F | G | E | R | R | K | T | Q | R | S | K | G | K | Q | T | K | S | L | H | S | - | - | 435 | | A7TK63/1-431 | 379 | E | C | K | K | V | H | D | T | A | V | I | R | K | A | L | T | K | V | T | K | A | K | R | E | S | L | M | D | M | E | K | A | N | F | G | E | K | R | K | T | Q | K | K | K | N | L | A | E | K | S | L | R | A | - | - | 431 | | C5E1Y4/1-433 | 380 | E | F | K | D | V | H | D | T | A | V | I | R | K | A | L | N | K | V | T | V | A | K | R | E | S | L | M | S | M | E | K | E | N | F | G | E | R | R | K | L | H | L | K | K | S | G | L | S | K | S | Y | R | N | - | N | 433 | | C5DTV7/1-435 | 383 | E | C | D | K | V | H | D | T | A | V | I | R | K | S | L | N | S | V | T | K | A | K | R | E | S | L | M | A | M | E | K | E | N | F | G | E | K | K | I | Q | R | K | K | K | N | - | - | Q | K | G | F | R | S | T | D | 435 | | Kwal\_56.24760/1-433 | 380 | E | F | K | D | V | H | D | T | A | V | I | R | K | A | L | N | K | V | T | A | A | K | R | E | S | L | M | A | M | E | K | E | H | F | G | E | R | R | K | L | H | Q | K | K | N | D | E | G | K | V | Y | R | R | - | N | 433 | | Sbay\_599.21/1-412 | 360 | E | A | N | K | V | H | D | T | A | V | I | R | K | A | L | T | K | V | T | K | A | K | R | E | S | L | M | A | M | Q | K | E | N | F | G | D | R | R | R | L | Q | K | Q | K | Q | M | D | N | K | S | M | R | S | - | - | 412 | | SAKL0H02178g/1-435 | 383 | Q | F | E | K | V | H | D | T | A | V | I | R | K | A | L | N | K | V | T | A | A | K | R | E | S | L | M | A | M | D | K | E | N | F | G | E | K | K | R | I | Q | Q | K | K | H | M | Q | E | K | G | Y | R | K | - | - | 435 | | P38719/1-431 | 379 | E | T | N | K | V | H | D | T | A | V | I | R | K | A | L | T | K | V | T | K | A | K | R | E | S | L | M | A | M | Q | K | E | N | F | G | E | R | K | R | Q | Q | K | K | K | Q | N | D | G | K | S | L | R | S | - | - | 431 | |
