# Supplementary material for: Plus ça change – evolutionary sequence divergence predicts protein subcellular localization signals
Source: BMC Genomics. 2014 Jan 20;15:46. doi: 10.1186/1471-2164-15-46 (PMC3906766; doi:10.1186/1471-2164-15-46)
Supplement: Additional file 2 — MSA’s of proteins for which sequence divergence changes predicted localization signals. Contains links to ortholog multiple sequence alignments of each protein in Additional file 3: Table S1. [file 1471-2164-15-46-S2.zip › P39735.html]

|  |  |  |  |  |  |  |  |  |  |  |  |  |  |  |  |  |  |  |  |  |  |  |  |  |  |  |  |  |  |  |  |  |  |  |  |  |  |  |  |  |  |  |  |  |  |  |  |  |  |  |  |  |  |  |  |  |  |  |  |  |  |  |  |  |  |  |  |  |  |  |  |  |  |  |  |  |  |  |  |  |  |  |  |  |  |  |  |  |  |  |  |  |  |  |  |  |  |  |  |  |  |  |  |  |  |  |  |  |  |  |  |  |  |  |  |  |  |  |  |  |  |  |  |  |  |  |  |  |  |  |  |  |  |  |  |  |  |  |  |  |  |  |  |  |  |  |  |  |  |  |  |  |  |  |  |  |  |  |  |  |  |  |  |  |  |  |  |  |  |  |  |  |  |  |  |  |  |  |  |  |  |  |  |  |  |  |  |  |  |  |  |  |  |  |  |  |  |  |  |  |  |  |  |  |  |  |  |  |  |  |  |  |  |  |  |  |  |  |  |  |  |  |  |  |  |  |  |  |  |  |  |  |  |  |  |  |  |  |  |  |  |  |  |  |  |  |  |  |  |  |  |  |  |  |  |  |  |  |  |  |  |  |  |  |  |  |  |  |  |  |  |  |  |  |  |  |  |  |  |  |  |  |  |  |  |  |  |  |  |  |  |  |  |  |  |  |  |  |  |  |  |  |  |  |  |  |  |  |  |  |  |  |  |  |  |  |  |  |  |  |  |  |  |  |  |  |  |  |  |  |  |  |  |  |  |  |  |  |  |  |  |  |  |  |  |  |  |  |  |  |  |  |  |  |  |  |  |  |  |  |  |  |  |  |  |  |  |  |  |  |  |  |  |  |  |  |  |  |  |  |  |  |  |  |  |  |  |  |  |  |  |  |  |  |  |  |  |  |  |  |  |  |  |  |  |  |  |  |  |  |  |  |  |  |  |  |  |  |  |  |  |  |  |  |  |  |  |  |  |  |  |  |  |  |  |  |  |  |  |  |  |  |  |  |  |  |  |  |  |  |  |  |  |  |  |  |  |  |  |  |  |  |  |  |  |  |  |  |  |  |  |  |  |  |  |  |  |  |  |  |  |  |  |  |  |  |  |  |  |  |  |  |  |  |  |  |  |  |  |  |  |  |  |  |  |  |  |  |  |  |  |  |  |  |  |  |  |  |  |  |  |  |  |  |  |  |  |  |  |  |  |  |  |  |  |  |  |  |  |  |  |  |  |  |  |  |  |  |  |  |  |  |  |  |  |  |  |  |  |  |  |  |  |  |  |  |  |  |  |  |  |  |  |  |  |  |  |  |  |  |  |  |  |  |  |  |  |  |  |  |  |  |  |  |  |  |  |  |  |  |  |  |  |  |  |  |  |  |  |  |  |  |  |  |  |  |  |  |  |  |  |  |  |  |  |  |  |  |  |  |  |  |  |  |  |  |  |  |  |  |  |  |  |  |  |  |  |  |  |  |  |  |  |  |  |  |  |  |  |  |  |  |  |  |  |  |  |  |  |  |  |  |  |  |  |  |  |  |  |  |  |  |  |  |  |  |  |  |  |  |  |  |  |  |  |  |  |  |  |  |  |  |  |  |  |  |  |  |  |  |  |  |  |  |  |  |  |  |  |  |  |  |  |  |  |  |  |  |  |  |  |  |  |  |  |  |  |  |  |  |  |  |  |  |  |  |  |  |  |  |  |  |  |  |  |  |  |  |  |  |  |  |  |  |  |  |  |  |  |  |  |  |  |  |  |  |  |  |  |  |  |  |  |  |  |  |  |  |  |  |  |  |  |  |  |  |  |  |  |  |  |  |  |  |  |  |  |  |  |  |  |  |  |  |  |  |  |  |  |  |  |  |  |  |  |  |  |  |  |  |  |  |  |  |  |  |  |  |  |  |  |  |  |  |  |  |  |  |  |  |  |  |  |  |  |  |  |  |  |  |  |  |  |  |  |  |  |  |  |  |  |  |  |  |  |  |  |  |  |  |  |  |  |  |  |  |  |  |  |  |  |  |  |  |  |  |  |  |  |  |  |  |  |  |  |  |  |  |  |  |  |  |  |  |  |  |  |  |  |  |  |  |  |  |  |  |  |  |  |  |  |  |  |  |  |  |  |  |  |  |  |  |  |  |  |  |  |  |  |  |  |  |  |  |  |  |  |  |  |  |  |  |  |  |  |  |  |  |  |  |  |  |  |  |  |  |  |  |  |  |  |  |  |  |  |  |  |  |  |  |  |  |  |  |  |  |  |  |  |  |  |  |  |  |  |  |  |  |  |  |  |  |  |  |  |  |  |  |  |  |  |  |  |  |  |  |  |  |  |  |  |  |  |  |  |  |  |  |  |  |  |  |  |  |  |  |  |  |  |  |  |  |  |  |  |  |  |  |  |  |  |  |  |  |  |  |  |  |  |  |  |  |  |  |  |  |  |  |  |  |  |  |  |  |  |  |  |  |  |  |  |  |  |  |  |  |  |  |  |  |  |  |  |  |  |  |  |  |  |  |  |  |  |  |  |  |  |  |  |  |  |  |  |  |  |  |  |  |  |  |  |  |  |  |  |  |  |  |  |  |  |  |  |  |  |  |  |  |  |  |  |  |  |  |  |  |  |  |  |  |  |  |  |  |  |  |  |  |  |  |  |  |  |  |  |  |  |  |  |  |  |  |  |  |  |  |  |  |  |  |  |  |  |  |  |  |  |  |  |  |  |  |  |  |  |  |  |  |  |  |  |  |  |  |  |  |  |  |  |  |  |  |  |  |  |  |  |  |  |  |  |  |  |  |  |  |  |  |  |  |  |  |  |  |  |  |  |  |  |  |  |  |  |  |  |  |  |  |  |  |  |  |  |  |  |  |  |  |  |  |  |  |  |  |  |  |  |  |  |  |  |  |  |  |  |  |  |  |  |  |  |  |  |  |  |  |  |  |  |  |  |  |  |  |  |  |  |  |  |  |  |  |  |  |  |  |  |  |  |  |  |  |  |  |  |  |  |  |  |  |  |  |  |  |  |  |  |  |  |  |  |  |  |  |  |  |  |  |  |  |  |  |  |  |  |  |  |  |  |  |  |  |  |  |  |  |  |  |  |  |  |  |  |  |  |  |  |  |  |  |  |  |  |  |  |  |  |  |  |  |  |  |  |  |  |  |  |  |  |  |  |  |  |  |  |  |  |  |  |  |  |  |  |  |  |  |  |  |  |  |  |  |  |  |  |  |  |  |  |  |  |  |  |  |  |  |  |  |  |  |  |  |  |  |  |  |  |  |  |  |  |  |  |  |  |  |  |  |  |  |  |  |  |  |  |  |  |  |  |  |  |  |  |  |  |  |  |  |  |  |  |  |  |  |  |  |  |  |  |  |  |  |  |  |  |  |  |  |  |  |  |  |  |  |  |  |  |  |  |  |  |  |  |  |  |  |  |  |  |  |  |  |  |  |  |  |  |  |  |  |  |  |  |  |  |  |  |  |  |  |  |  |  |  |  |  |  |  |  |  |  |  |  |  |  |  |  |  |  |  |  |  |  |  |  |  |  |  |  |  |  |  |  |  |  |  |  |  |  |  |  |  |  |  |  |  |  |  |  |  |  |  |  |  |  |  |  |  |  |  |  |  |  |  |  |  |  |  |  |  |  |  |  |  |  |  |  |  |  |  |  |  |  |  |  |  |  |  |  |  |  |  |  |  |  |  |  |  |  |  |  |  |  |  |  |  |  |  |  |  |  |  |  |  |  |  |  |  |  |  |  |  |  |  |  |  |  |  |  |  |  |  |  |  |  |  |  |  |  |  |  |  |  |  |  |  |  |  |  |  |  |  |  |  |  |  |  |  |  |  |  |  |  |  |  |  |  |  |  |  |  |  |  |  |  |  |  |  |  |  |  |  |  |  |  |  |  |  |  |  |  |  |  |  |  |  |  |  |  |  |  |  |  |  |  |  |  |  |  |  |  |  |  |  |  |  |  |  |  |  |  |  |  |  |  |  |  |  |  |  |  |  |  |  |  |  |  |  |  |  |  |  |  |  |  |  |  |  |  |  |  |  |  |  |  |  |  |  |  |  |  |  |  |  |
| --- | --- | --- | --- | --- | --- | --- | --- | --- | --- | --- | --- | --- | --- | --- | --- | --- | --- | --- | --- | --- | --- | --- | --- | --- | --- | --- | --- | --- | --- | --- | --- | --- | --- | --- | --- | --- | --- | --- | --- | --- | --- | --- | --- | --- | --- | --- | --- | --- | --- | --- | --- | --- | --- | --- | --- | --- | --- | --- | --- | --- | --- | --- | --- | --- | --- | --- | --- | --- | --- | --- | --- | --- | --- | --- | --- | --- | --- | --- | --- | --- | --- | --- | --- | --- | --- | --- | --- | --- | --- | --- | --- | --- | --- | --- | --- | --- | --- | --- | --- | --- | --- | --- | --- | --- | --- | --- | --- | --- | --- | --- | --- | --- | --- | --- | --- | --- | --- | --- | --- | --- | --- | --- | --- | --- | --- | --- | --- | --- | --- | --- | --- | --- | --- | --- | --- | --- | --- | --- | --- | --- | --- | --- | --- | --- | --- | --- | --- | --- | --- | --- | --- | --- | --- | --- | --- | --- | --- | --- | --- | --- | --- | --- | --- | --- | --- | --- | --- | --- | --- | --- | --- | --- | --- | --- | --- | --- | --- | --- | --- | --- | --- | --- | --- | --- | --- | --- | --- | --- | --- | --- | --- | --- | --- | --- | --- | --- | --- | --- | --- | --- | --- | --- | --- | --- | --- | --- | --- | --- | --- | --- | --- | --- | --- | --- | --- | --- | --- | --- | --- | --- | --- | --- | --- | --- | --- | --- | --- | --- | --- | --- | --- | --- | --- | --- | --- | --- | --- | --- | --- | --- | --- | --- | --- | --- | --- | --- | --- | --- | --- | --- | --- | --- | --- | --- | --- | --- | --- | --- | --- | --- | --- | --- | --- | --- | --- | --- | --- | --- | --- | --- | --- | --- | --- | --- | --- | --- | --- | --- | --- | --- | --- | --- | --- | --- | --- | --- | --- | --- | --- | --- | --- | --- | --- | --- | --- | --- | --- | --- | --- | --- | --- | --- | --- | --- | --- | --- | --- | --- | --- | --- | --- | --- | --- | --- | --- | --- | --- | --- | --- | --- | --- | --- | --- | --- | --- | --- | --- | --- | --- | --- | --- | --- | --- | --- | --- | --- | --- | --- | --- | --- | --- | --- | --- | --- | --- | --- | --- | --- | --- | --- | --- | --- | --- | --- | --- | --- | --- | --- | --- | --- | --- | --- | --- | --- | --- | --- | --- | --- | --- | --- | --- | --- | --- | --- | --- | --- | --- | --- | --- | --- | --- | --- | --- | --- | --- | --- | --- | --- | --- | --- | --- | --- | --- | --- | --- | --- | --- | --- | --- | --- | --- | --- | --- | --- | --- | --- | --- | --- | --- | --- | --- | --- | --- | --- | --- | --- | --- | --- | --- | --- | --- | --- | --- | --- | --- | --- | --- | --- | --- | --- | --- | --- | --- | --- | --- | --- | --- | --- | --- | --- | --- | --- | --- | --- | --- | --- | --- | --- | --- | --- | --- | --- | --- | --- | --- | --- | --- | --- | --- | --- | --- | --- | --- | --- | --- | --- | --- | --- | --- | --- | --- | --- | --- | --- | --- | --- | --- | --- | --- | --- | --- | --- | --- | --- | --- | --- | --- | --- | --- | --- | --- | --- | --- | --- | --- | --- | --- | --- | --- | --- | --- | --- | --- | --- | --- | --- | --- | --- | --- | --- | --- | --- | --- | --- | --- | --- | --- | --- | --- | --- | --- | --- | --- | --- | --- | --- | --- | --- | --- | --- | --- | --- | --- | --- | --- | --- | --- | --- | --- | --- | --- | --- | --- | --- | --- | --- | --- | --- | --- | --- | --- | --- | --- | --- | --- | --- | --- | --- | --- | --- | --- | --- | --- | --- | --- | --- | --- | --- | --- | --- | --- | --- | --- | --- | --- | --- | --- | --- | --- | --- | --- | --- | --- | --- | --- | --- | --- | --- | --- | --- | --- | --- | --- | --- | --- | --- | --- | --- | --- | --- | --- | --- | --- | --- | --- | --- | --- | --- | --- | --- | --- | --- | --- | --- | --- | --- | --- | --- | --- | --- | --- | --- | --- | --- | --- | --- | --- | --- | --- | --- | --- | --- | --- | --- | --- | --- | --- | --- | --- | --- | --- | --- | --- | --- | --- | --- | --- | --- | --- | --- | --- | --- | --- | --- | --- | --- | --- | --- | --- | --- | --- | --- | --- | --- | --- | --- | --- | --- | --- | --- | --- | --- | --- | --- | --- | --- | --- | --- | --- | --- | --- | --- | --- | --- | --- | --- | --- | --- | --- | --- | --- | --- | --- | --- | --- | --- | --- | --- | --- | --- | --- | --- | --- | --- | --- | --- | --- | --- | --- | --- | --- | --- | --- | --- | --- | --- | --- | --- | --- | --- | --- | --- | --- | --- | --- | --- | --- | --- | --- | --- | --- | --- | --- | --- | --- | --- | --- | --- | --- | --- | --- | --- | --- | --- | --- | --- | --- | --- | --- | --- | --- | --- | --- | --- | --- | --- | --- | --- | --- | --- | --- | --- | --- | --- | --- | --- | --- | --- | --- | --- | --- | --- | --- | --- | --- | --- | --- | --- | --- | --- | --- | --- | --- | --- | --- | --- | --- | --- | --- | --- | --- | --- | --- | --- | --- | --- | --- | --- | --- | --- | --- | --- | --- | --- | --- | --- | --- | --- | --- | --- | --- | --- | --- | --- | --- | --- | --- | --- | --- | --- | --- | --- | --- | --- | --- | --- | --- | --- | --- | --- | --- | --- | --- | --- | --- | --- | --- | --- | --- | --- | --- | --- | --- | --- | --- | --- | --- | --- | --- | --- | --- | --- | --- | --- | --- | --- | --- | --- | --- | --- | --- | --- | --- | --- | --- | --- | --- | --- | --- | --- | --- | --- | --- | --- | --- | --- | --- | --- | --- | --- | --- | --- | --- | --- | --- | --- | --- | --- | --- | --- | --- | --- | --- | --- | --- | --- | --- | --- | --- | --- | --- | --- | --- | --- | --- | --- | --- | --- | --- | --- | --- | --- | --- | --- | --- | --- | --- | --- | --- | --- | --- | --- | --- | --- | --- | --- | --- | --- | --- | --- | --- | --- | --- | --- | --- | --- | --- | --- | --- | --- | --- | --- | --- | --- | --- | --- | --- | --- | --- | --- | --- | --- | --- | --- | --- | --- | --- | --- | --- | --- | --- | --- | --- | --- | --- | --- | --- | --- | --- | --- | --- | --- | --- | --- | --- | --- | --- | --- | --- | --- | --- | --- | --- | --- | --- | --- | --- | --- | --- | --- | --- | --- | --- | --- | --- | --- | --- | --- | --- | --- | --- | --- | --- | --- | --- | --- | --- | --- | --- | --- | --- | --- | --- | --- | --- | --- | --- | --- | --- | --- | --- | --- | --- | --- | --- | --- | --- | --- | --- | --- | --- | --- | --- | --- | --- | --- | --- | --- | --- | --- | --- | --- | --- | --- | --- | --- | --- | --- | --- | --- | --- | --- | --- | --- | --- | --- | --- | --- | --- | --- | --- | --- | --- | --- | --- | --- | --- | --- | --- | --- | --- | --- | --- | --- | --- | --- | --- | --- | --- | --- | --- | --- | --- | --- | --- | --- | --- | --- | --- | --- | --- | --- | --- | --- | --- | --- | --- | --- | --- | --- | --- | --- | --- | --- | --- | --- | --- | --- | --- | --- | --- | --- | --- | --- | --- | --- | --- | --- | --- | --- | --- | --- | --- | --- | --- | --- | --- | --- | --- | --- | --- | --- | --- | --- | --- | --- | --- | --- | --- | --- | --- | --- | --- | --- | --- | --- | --- | --- | --- | --- | --- | --- | --- | --- | --- | --- | --- | --- | --- | --- | --- | --- | --- | --- | --- | --- | --- | --- | --- | --- | --- | --- | --- | --- | --- | --- | --- | --- | --- | --- | --- | --- | --- | --- | --- | --- | --- | --- | --- | --- | --- | --- | --- | --- | --- | --- | --- | --- | --- | --- | --- | --- | --- | --- | --- | --- | --- | --- | --- | --- | --- | --- | --- | --- | --- | --- | --- | --- | --- | --- | --- | --- | --- | --- | --- | --- | --- | --- | --- | --- | --- | --- | --- | --- | --- | --- | --- | --- | --- | --- | --- | --- | --- | --- | --- | --- | --- | --- | --- | --- | --- | --- | --- | --- | --- | --- | --- | --- | --- | --- | --- | --- | --- | --- | --- | --- | --- | --- | --- | --- | --- | --- | --- | --- | --- | --- | --- | --- | --- | --- | --- | --- | --- | --- | --- | --- | --- | --- | --- | --- | --- | --- | --- | --- | --- | --- | --- | --- | --- | --- | --- | --- | --- | --- | --- | --- | --- | --- | --- | --- | --- | --- | --- | --- | --- | --- | --- | --- | --- | --- | --- | --- | --- | --- | --- | --- | --- | --- | --- | --- | --- | --- | --- | --- | --- | --- | --- | --- | --- | --- | --- | --- | --- | --- | --- | --- | --- | --- | --- | --- | --- | --- | --- | --- | --- | --- | --- | --- | --- | --- | --- | --- | --- | --- | --- | --- | --- | --- | --- | --- | --- | --- | --- | --- | --- | --- | --- | --- | --- | --- | --- | --- | --- | --- | --- | --- | --- | --- | --- | --- | --- | --- | --- | --- | --- | --- | --- | --- | --- | --- | --- | --- | --- | --- | --- | --- | --- | --- | --- | --- | --- | --- | --- | --- | --- | --- | --- | --- | --- | --- | --- | --- | --- | --- | --- | --- | --- | --- | --- | --- | --- | --- | --- | --- | --- | --- | --- | --- | --- | --- | --- | --- | --- | --- | --- | --- | --- | --- | --- | --- | --- | --- | --- | --- | --- | --- | --- | --- | --- | --- | --- | --- | --- | --- | --- | --- | --- | --- | --- | --- | --- | --- | --- | --- | --- | --- | --- | --- | --- | --- | --- | --- | --- | --- | --- | --- | --- | --- | --- | --- | --- | --- | --- | --- | --- | --- | --- | --- | --- | --- | --- | --- | --- | --- | --- | --- | --- | --- | --- | --- | --- | --- | --- | --- | --- | --- | --- | --- | --- | --- | --- | --- | --- | --- | --- | --- | --- | --- | --- | --- | --- | --- | --- | --- | --- | --- | --- | --- | --- | --- | --- | --- | --- | --- | --- | --- | --- | --- | --- | --- | --- | --- | --- | --- | --- | --- | --- | --- | --- | --- | --- | --- | --- | --- | --- | --- | --- | --- | --- | --- | --- | --- | --- | --- | --- | --- | --- | --- | --- | --- | --- | --- | --- | --- | --- | --- | --- | --- | --- | --- | --- | --- | --- | --- | --- | --- | --- | --- | --- | --- | --- | --- | --- | --- | --- | --- | --- | --- | --- | --- | --- | --- | --- | --- | --- | --- | --- | --- | --- | --- | --- | --- | --- | --- | --- | --- | --- | --- | --- | --- | --- | --- | --- | --- | --- | --- | --- | --- | --- | --- | --- | --- | --- | --- | --- | --- | --- | --- | --- | --- | --- | --- | --- | --- | --- | --- | --- | --- | --- | --- | --- | --- | --- | --- | --- | --- | --- | --- | --- | --- | --- | --- | --- | --- | --- | --- | --- | --- | --- | --- | --- | --- | --- | --- | --- | --- | --- | --- | --- | --- | --- | --- | --- | --- | --- | --- | --- | --- | --- | --- | --- | --- | --- | --- | --- | --- | --- | --- | --- | --- | --- | --- | --- | --- | --- | --- | --- | --- | --- | --- | --- | --- | --- | --- | --- | --- | --- | --- | --- | --- | --- | --- | --- | --- | --- | --- | --- | --- | --- | --- | --- | --- | --- | --- | --- | --- | --- | --- | --- | --- | --- | --- | --- | --- | --- | --- | --- | --- | --- | --- | --- | --- | --- | --- | --- | --- | --- | --- | --- | --- | --- | --- | --- | --- | --- | --- | --- | --- | --- | --- | --- | --- | --- | --- | --- | --- | --- | --- | --- | --- | --- | --- | --- | --- | --- | --- | --- | --- | --- | --- | --- | --- | --- | --- |
| |  |  |  |  |  |  |  |  |  |  |  |  |  |  |  |  |  |  |  |  |  |  |  |  |  |  |  |  |  |  |  |  |  |  |  |  |  |  |  |  |  |  |  |  |  |  |  |  |  |  |  |  |  |  |  |  |  |  |  |  | | --- | --- | --- | --- | --- | --- | --- | --- | --- | --- | --- | --- | --- | --- | --- | --- | --- | --- | --- | --- | --- | --- | --- | --- | --- | --- | --- | --- | --- | --- | --- | --- | --- | --- | --- | --- | --- | --- | --- | --- | --- | --- | --- | --- | --- | --- | --- | --- | --- | --- | --- | --- | --- | --- | --- | --- | --- | --- | --- | --- | | G0VGN5/1-254 | 1 | M | A | S | S | I | A | T | V | R | I | S | K | N | A | I | V | S | L | R | V | F | I | N | R | K | K | L | L | R | N | N | S | R | - | - | - | - | - | E | G | Q | T | F | Q | A | P | L | L | S | N | N | S | I | I | C | L | K | 52 | | Q6FT09/1-253 | 1 | M | V | Q | N | I | A | Y | I | K | V | A | K | D | S | I | L | P | V | R | V | H | I | N | R | R | Q | I | L | S | T | A | T | E | - | - | - | - | - | E | - | S | K | V | Q | A | P | L | L | S | N | N | T | I | V | C | L | K | 51 | | A7TSP3/1-258 | 1 | M | T | L | S | I | A | R | I | K | I | S | D | N | I | I | L | P | L | R | V | F | I | N | R | K | Q | L | L | Y | N | I | T | N | - | - | - | - | - | E | - | P | I | I | Q | A | P | L | L | S | N | N | S | I | V | C | L | K | 51 | | C5DVA4/1-256 | 1 | M | A | P | S | V | A | L | V | K | V | A | S | N | T | V | L | P | I | R | I | F | I | N | R | K | Q | L | L | Q | N | N | S | K | - | - | - | - | - | D | - | T | V | F | H | A | P | L | L | S | N | N | S | I | V | C | I | K | 51 | | Sbay\_669.36/1-248 | 1 | - | - | - | - | - | - | - | - | - | - | - | - | - | M | V | L | P | L | R | I | F | V | N | R | K | H | M | L | Q | S | N | S | N | S | N | N | N | P | D | A | T | I | F | Q | A | P | L | L | S | N | N | S | I | V | C | L | K | 44 | | P39735/1-261 | 1 | M | A | P | S | I | A | T | V | K | I | A | R | D | M | V | L | P | L | R | I | F | V | N | R | K | Q | I | L | Q | T | N | D | K | T | S | N | K | S | N | A | T | I | F | E | A | P | L | L | S | N | N | S | I | I | C | L | K | 57 | |  | | G0VGN5/1-254 | 53 | S | P | L | V | R | I | L | I | S | N | Q | D | M | E | R | L | T | N | E | I | R | D | T | I | I | L | I | V | Y | D | L | S | S | P | E | V | M | E | T | V | L | G | K | L | R | I | G | S | S | A | D | F | E | T | E | I | L | 109 | | Q6FT09/1-253 | 52 | S | P | L | T | K | I | Y | L | S | N | T | D | L | K | N | L | V | T | E | M | E | D | D | I | I | L | I | L | Y | E | L | S | S | P | A | M | K | Q | N | I | F | S | K | I | R | V | G | Q | V | Y | D | F | D | E | D | T | L | 108 | | A7TSP3/1-258 | 52 | S | P | N | T | R | I | F | V | S | N | N | D | L | E | S | L | C | D | E | I | R | D | D | I | L | L | I | I | Y | E | L | T | S | Q | E | V | M | N | Q | T | I | G | K | L | R | V | G | Y | N | L | D | F | K | K | S | V | I | 108 | | C5DVA4/1-256 | 52 | S | P | S | T | R | I | Y | L | S | N | H | D | M | Q | N | L | T | N | D | I | E | T | E | I | L | L | I | V | H | E | L | T | S | P | E | L | M | Q | D | V | L | R | K | I | K | I | G | Q | S | M | D | W | Q | K | D | V | M | 108 | | Sbay\_669.36/1-248 | 45 | S | P | N | T | R | I | Y | L | S | Q | Q | D | K | K | I | L | C | D | E | I | K | E | D | L | L | L | I | L | Y | E | L | A | S | P | E | I | V | T | S | V | L | S | K | I | R | I | G | H | T | I | D | F | Q | T | G | V | L | 101 | | P39735/1-261 | 58 | S | P | N | T | R | I | Y | L | S | Q | Q | D | K | K | N | L | C | D | E | I | K | E | D | L | L | L | I | V | Y | E | L | A | S | P | E | I | I | S | S | V | L | S | K | I | R | V | G | H | S | T | D | F | Q | I | N | V | L | 114 | |  | | G0VGN5/1-254 | 110 | P | K | L | M | D | E | N | T | L | S | K | D | L | V | N | V | P | L | Q | T | V | T | R | V | A | K | F | K | Y | K | L | R | F | K | G | N | W | E | L | D | I | F | I | N | N | M | K | K | L | I | K | I | R | H | F | L | L | 166 | | Q6FT09/1-253 | 109 | K | K | F | P | Q | S | I | - | - | Q | G | S | L | K | E | S | N | L | T | S | L | K | R | V | G | K | M | K | Y | K | L | T | Y | R | K | N | W | D | V | D | I | F | I | S | N | I | N | K | L | E | T | I | R | T | Y | L | I | 163 | | A7TSP3/1-258 | 109 | N | R | F | F | K | D | D | D | T | G | D | Y | Y | I | D | S | N | I | L | M | V | T | R | V | S | K | L | K | Y | K | F | R | F | K | K | N | W | E | V | D | I | F | I | N | N | I | K | K | L | S | D | L | R | D | Y | L | L | 165 | | C5DVA4/1-256 | 109 | P | R | I | F | P | S | E | - | - | T | E | P | L | V | V | S | H | I | Q | S | I | T | R | I | A | K | F | K | Y | K | L | H | F | K | H | N | W | E | L | N | I | F | I | D | N | I | R | A | L | T | H | I | R | A | Y | L | M | 163 | | Sbay\_669.36/1-248 | 102 | P | K | L | F | A | G | A | C | - | T | E | D | T | L | S | S | H | I | Q | T | V | T | R | L | A | K | F | K | Y | K | L | H | Y | K | N | K | W | E | L | D | I | Y | V | N | S | I | K | N | I | A | N | I | R | H | Y | L | M | 157 | | P39735/1-261 | 115 | P | K | L | F | A | G | A | D | - | T | D | N | A | V | T | S | H | I | Q | S | V | T | R | L | A | K | F | K | Y | K | L | H | Y | K | H | K | W | E | L | D | I | F | I | N | S | I | K | K | I | A | N | L | R | H | Y | L | M | 170 | |  | | G0VGN5/1-254 | 167 | F | T | - | D | G | H | M | S | A | A | R | T | L | E | L | P | P | N | R | R | I | L | L | T | E | Q | K | T | S | L | E | S | E | - | - | - | G | S | P | M | I | P | L | E | V | D | G | D | E | M | - | - | I | E | T | T | Q | 217 | | Q6FT09/1-253 | 164 | F | K | - | D | Q | Y | P | Q | W | N | E | Y | P | C | L | Q | K | Q | Q | S | L | L | V | M | G | Q | K | L | - | - | - | S | E | S | T | N | Q | D | P | I | I | L | Q | E | E | E | E | L | N | L | N | Y | E | Q | S | A | E | 216 | | A7TSP3/1-258 | 166 | F | R | H | N | P | N | Q | M | S | S | P | S | I | P | I | N | S | V | K | K | V | L | I | M | E | P | D | D | Y | L | N | I | E | T | G | K | D | Q | T | M | I | L | Q | E | D | E | N | D | S | G | - | I | I | D | Q | Q | S | 221 | | C5DVA4/1-256 | 164 | F | K | - | N | D | L | S | I | I | P | P | V | V | G | L | H | S | K | S | K | L | L | L | H | E | Q | - | - | - | - | - | M | E | T | E | - | K | P | P | Q | V | L | Q | E | D | D | E | P | S | I | P | N | Y | D | E | A | A | 213 | | Sbay\_669.36/1-248 | 158 | F | Q | - | - | - | - | T | L | T | I | N | G | F | S | L | N | A | G | P | K | T | L | L | T | K | K | - | - | - | - | - | I | E | T | Q | P | Q | V | P | K | L | L | V | E | N | E | E | S | D | A | - | - | - | - | L | E | A | 201 | | P39735/1-261 | 171 | F | Q | - | - | - | - | T | L | T | L | N | G | F | S | L | N | A | G | P | K | T | L | L | A | R | K | - | - | - | - | - | I | E | K | Q | P | Q | V | P | N | L | L | I | E | N | G | D | A | D | A | - | - | - | - | L | D | T | 214 | |  | | G0VGN5/1-254 | 218 | D | A | V | E | D | V | K | P | D | I | K | L | K | Y | N | P | I | I | N | L | G | E | C | L | S | I | H | I | L | Q | R | P | R | R | H | K | V | - | - | - | - | - | - | - | - | - | - |  | | | | | | | | | | 254 | | Q6FT09/1-253 | 217 | D | Q | P | N | N | E | K | P | E | I | A | F | T | Y | E | P | Q | L | S | L | I | Q | C | L | D | L | Y | I | L | K | R | P | K | R | K | R | S | - | - | - | - | - | - | - | - | - | - |  | | | | | | | | | | 253 | | A7TSP3/1-258 | 222 | S | Q | D | D | D | S | K | P | I | I | H | Y | R | Y | K | P | T | L | N | L | A | E | L | I | D | I | H | V | L | Q | R | P | R | R | H | K | I | - | - | - | - | - | - | - | - | - | - |  | | | | | | | | | | 258 | | C5DVA4/1-256 | 214 | E | I | Q | E | E | Q | K | P | E | L | K | F | K | Y | S | P | V | V | S | L | G | E | C | I | N | V | H | V | L | Q | R | P | Q | R | N | K | R | - | - | - | - | T | Q | P | T | S | S |  | | | | | | | | | | 256 | | Sbay\_669.36/1-248 | 202 | P | E | E | E | N | I | K | P | V | I | E | F | K | Y | K | P | V | I | N | L | G | E | I | I | D | I | Y | V | L | Q | R | P | R | R | H | K | V | R | T | Q | P | D | Q | R | Q | E | E |  | | | | | | | | | | 248 | | P39735/1-261 | 215 | P | V | E | E | D | I | K | P | V | I | E | F | M | Y | K | P | V | I | N | L | G | E | I | I | D | V | H | V | L | H | R | P | R | R | H | K | V | R | T | Q | S | K | Q | P | Q | E | E |  | | | | | | | | | | 261 | |
