# Supplementary material for: Plus ça change – evolutionary sequence divergence predicts protein subcellular localization signals
Source: BMC Genomics. 2014 Jan 20;15:46. doi: 10.1186/1471-2164-15-46 (PMC3906766; doi:10.1186/1471-2164-15-46)
Supplement: Additional file 2 — MSA’s of proteins for which sequence divergence changes predicted localization signals. Contains links to ortholog multiple sequence alignments of each protein in Additional file 3: Table S1. [file 1471-2164-15-46-S2.zip › P41338.html]

|  |  |  |  |  |  |  |  |  |  |  |  |  |  |  |  |  |  |  |  |  |  |  |  |  |  |  |  |  |  |  |  |  |  |  |  |  |  |  |  |  |  |  |  |  |  |  |  |  |  |  |  |  |  |  |  |  |  |  |  |  |  |  |  |  |  |  |  |  |  |  |  |  |  |  |  |  |  |  |  |  |  |  |  |  |  |  |  |  |  |  |  |  |  |  |  |  |  |  |  |  |  |  |  |  |  |  |  |  |  |  |  |  |  |  |  |  |  |  |  |  |  |  |  |  |  |  |  |  |  |  |  |  |  |  |  |  |  |  |  |  |  |  |  |  |  |  |  |  |  |  |  |  |  |  |  |  |  |  |  |  |  |  |  |  |  |  |  |  |  |  |  |  |  |  |  |  |  |  |  |  |  |  |  |  |  |  |  |  |  |  |  |  |  |  |  |  |  |  |  |  |  |  |  |  |  |  |  |  |  |  |  |  |  |  |  |  |  |  |  |  |  |  |  |  |  |  |  |  |  |  |  |  |  |  |  |  |  |  |  |  |  |  |  |  |  |  |  |  |  |  |  |  |  |  |  |  |  |  |  |  |  |  |  |  |  |  |  |  |  |  |  |  |  |  |  |  |  |  |  |  |  |  |  |  |  |  |  |  |  |  |  |  |  |  |  |  |  |  |  |  |  |  |  |  |  |  |  |  |  |  |  |  |  |  |  |  |  |  |  |  |  |  |  |  |  |  |  |  |  |  |  |  |  |  |  |  |  |  |  |  |  |  |  |  |  |  |  |  |  |  |  |  |  |  |  |  |  |  |  |  |  |  |  |  |  |  |  |  |  |  |  |  |  |  |  |  |  |  |  |  |  |  |  |  |  |  |  |  |  |  |  |  |  |  |  |  |  |  |  |  |  |  |  |  |  |  |  |  |  |  |  |  |  |  |  |  |  |  |  |  |  |  |  |  |  |  |  |  |  |  |  |  |  |  |  |  |  |  |  |  |  |  |  |  |  |  |  |  |  |  |  |  |  |  |  |  |  |  |  |  |  |  |  |  |  |  |  |  |  |  |  |  |  |  |  |  |  |  |  |  |  |  |  |  |  |  |  |  |  |  |  |  |  |  |  |  |  |  |  |  |  |  |  |  |  |  |  |  |  |  |  |  |  |  |  |  |  |  |  |  |  |  |  |  |  |  |  |  |  |  |  |  |  |  |  |  |  |  |  |  |  |  |  |  |  |  |  |  |  |  |  |  |  |  |  |  |  |  |  |  |  |  |  |  |  |  |  |  |  |  |  |  |  |  |  |  |  |  |  |  |  |  |  |  |  |  |  |  |  |  |  |  |  |  |  |  |  |  |  |  |  |  |  |  |  |  |  |  |  |  |  |  |  |  |  |  |  |  |  |  |  |  |  |  |  |  |  |  |  |  |  |  |  |  |  |  |  |  |  |  |  |  |  |  |  |  |  |  |  |  |  |  |  |  |  |  |  |  |  |  |  |  |  |  |  |  |  |  |  |  |  |  |  |  |  |  |  |  |  |  |  |  |  |  |  |  |  |  |  |  |  |  |  |  |  |  |  |  |  |  |  |  |  |  |  |  |  |  |  |  |  |  |  |  |  |  |  |  |  |  |  |  |  |  |  |  |  |  |  |  |  |  |  |  |  |  |  |  |  |  |  |  |  |  |  |  |  |  |  |  |  |  |  |  |  |  |  |  |  |  |  |  |  |  |  |  |  |  |  |  |  |  |  |  |  |  |  |  |  |  |  |  |  |  |  |  |  |  |  |  |  |  |  |  |  |  |  |  |  |  |  |  |  |  |  |  |  |  |  |  |  |  |  |  |  |  |  |  |  |  |  |  |  |  |  |  |  |  |  |  |  |  |  |  |  |  |  |  |  |  |  |  |  |  |  |  |  |  |  |  |  |  |  |  |  |  |  |  |  |  |  |  |  |  |  |  |  |  |  |  |  |  |  |  |  |  |  |  |  |  |  |  |  |  |  |  |  |  |  |  |  |  |  |  |  |  |  |  |  |  |  |  |  |  |  |  |  |  |  |  |  |  |  |  |  |  |  |  |  |  |  |  |  |  |  |  |  |  |  |  |  |  |  |  |  |  |  |  |  |  |  |  |  |  |  |  |  |  |  |  |  |  |  |  |  |  |  |  |  |  |  |  |  |  |  |  |  |  |  |  |  |  |  |  |  |  |  |  |  |  |  |  |  |  |  |  |  |  |  |  |  |  |  |  |  |  |  |  |  |  |  |  |  |  |  |  |  |  |  |  |  |  |  |  |  |  |  |  |  |  |  |  |  |  |  |  |  |  |  |  |  |  |  |  |  |  |  |  |  |  |  |  |  |  |  |  |  |  |  |  |  |  |  |  |  |  |  |  |  |  |  |  |  |  |  |  |  |  |  |  |  |  |  |  |  |  |  |  |  |  |  |  |  |  |  |  |  |  |  |  |  |  |  |  |  |  |  |  |  |  |  |  |  |  |  |  |  |  |  |  |  |  |  |  |  |  |  |  |  |  |  |  |  |  |  |  |  |  |  |  |  |  |  |  |  |  |  |  |  |  |  |  |  |  |  |  |  |  |  |  |  |  |  |  |  |  |  |  |  |  |  |  |  |  |  |  |  |  |  |  |  |  |  |  |  |  |  |  |  |  |  |  |  |  |  |  |  |  |  |  |  |  |  |  |  |  |  |  |  |  |  |  |  |  |  |  |  |  |  |  |  |  |  |  |  |  |  |  |  |  |  |  |  |  |  |  |  |  |  |  |  |  |  |  |  |  |  |  |  |  |  |  |  |  |  |  |  |  |  |  |  |  |  |  |  |  |  |  |  |  |  |  |  |  |  |  |  |  |  |  |  |  |  |  |  |  |  |  |  |  |  |  |  |  |  |  |  |  |  |  |  |  |  |  |  |  |  |  |  |  |  |  |  |  |  |  |  |  |  |  |  |  |  |  |  |  |  |  |  |  |  |  |  |  |  |  |  |  |  |  |  |  |  |  |  |  |  |  |  |  |  |  |  |  |  |  |  |  |  |  |  |  |  |  |  |  |  |  |  |  |  |  |  |  |  |  |  |  |  |  |  |  |  |  |  |  |  |  |  |  |  |  |  |  |  |  |  |  |  |  |  |  |  |  |  |  |  |  |  |  |  |  |  |  |  |  |  |  |  |  |  |  |  |  |  |  |  |  |  |  |  |  |  |  |  |  |  |  |  |  |  |  |  |  |  |  |  |  |  |  |  |  |  |  |  |  |  |  |  |  |  |  |  |  |  |  |  |  |  |  |  |  |  |  |  |  |  |  |  |  |  |  |  |  |  |  |  |  |  |  |  |  |  |  |  |  |  |  |  |  |  |  |  |  |  |  |  |  |  |  |  |  |  |  |  |  |  |  |  |  |  |  |  |  |  |  |  |  |  |  |  |  |  |  |  |  |  |  |  |  |  |  |  |  |  |  |  |  |  |  |  |  |  |  |  |  |  |  |  |  |  |  |  |  |  |  |  |  |  |  |  |  |  |  |  |  |  |  |  |  |  |  |  |  |  |  |  |  |  |  |  |  |  |  |  |  |  |  |  |  |  |  |  |  |  |  |  |  |  |  |  |  |  |  |  |  |  |  |  |  |  |  |  |  |  |  |  |  |  |  |  |  |  |  |  |  |  |  |  |  |  |  |  |  |  |  |  |  |  |  |  |  |  |  |  |  |  |  |  |  |  |  |  |  |  |  |  |  |  |  |  |  |  |  |  |  |  |  |  |  |  |  |  |  |  |  |  |  |  |  |  |  |  |  |  |  |  |  |  |  |  |  |  |  |  |  |  |  |  |  |  |  |  |  |  |  |  |  |  |  |  |  |  |  |  |  |  |  |  |  |  |  |  |  |  |  |  |  |  |  |  |  |  |  |  |  |  |  |  |  |  |  |  |  |  |  |  |  |  |  |  |  |  |  |  |  |  |  |  |  |  |  |  |  |  |  |  |  |  |  |  |  |  |  |  |  |  |  |  |  |  |  |  |  |  |  |  |  |  |  |  |  |  |  |  |  |  |  |  |  |  |  |  |  |  |  |  |  |  |  |  |  |  |  |  |  |  |  |  |  |  |  |  |  |  |  |  |  |  |  |  |  |  |  |  |  |  |  |  |  |  |  |  |  |  |  |  |  |  |  |  |  |  |  |  |  |  |  |  |  |  |  |  |  |  |  |  |  |  |  |  |  |  |  |  |  |  |  |  |  |  |  |  |  |  |  |  |  |  |  |  |  |  |  |  |  |  |  |  |  |  |  |  |  |  |  |  |  |  |  |  |  |  |  |  |  |  |  |  |  |  |  |  |  |  |  |  |  |  |  |  |  |  |  |  |  |  |  |  |  |  |  |  |  |  |  |  |  |  |  |  |  |  |  |  |  |  |  |  |  |  |  |  |  |  |  |  |  |  |  |  |  |  |  |  |  |  |  |  |  |  |  |  |  |  |  |  |  |  |  |  |  |  |  |  |  |  |  |  |  |  |  |  |  |  |  |  |  |  |  |  |  |  |  |  |  |  |  |  |  |  |  |  |  |  |  |  |  |  |  |  |  |  |  |  |  |  |  |  |  |  |  |  |  |  |  |  |  |  |  |  |  |  |  |  |  |  |  |  |  |  |  |  |  |  |  |  |  |  |  |  |  |  |  |  |  |  |  |  |  |  |  |  |  |  |  |  |  |  |  |  |  |  |  |  |  |  |  |  |  |  |  |  |  |  |  |  |  |  |  |  |  |  |  |  |  |  |  |  |  |  |  |  |  |  |  |  |  |  |  |  |  |  |  |  |  |  |  |  |  |  |  |  |  |  |  |  |  |  |  |  |  |  |  |  |  |  |  |  |  |  |  |  |  |  |  |  |  |  |  |  |  |  |  |  |  |  |  |  |  |  |  |  |  |  |  |  |  |  |  |  |  |  |  |  |  |  |  |  |  |  |  |  |  |  |  |  |  |  |  |  |  |  |  |  |  |  |  |  |  |  |  |  |  |  |  |  |  |  |  |  |  |  |  |  |  |  |  |  |  |  |  |  |  |  |  |  |  |  |  |  |  |  |  |  |  |  |  |  |  |  |  |  |  |  |  |  |  |  |  |  |  |  |  |  |  |  |  |  |  |  |  |  |  |  |  |  |  |  |  |  |  |  |  |  |  |  |  |  |  |  |  |  |  |  |  |  |  |  |  |  |  |  |  |  |  |  |  |  |  |  |  |  |  |  |  |  |  |  |  |  |  |  |  |  |  |  |  |  |  |  |  |  |  |  |  |  |  |  |  |  |  |  |  |  |  |  |  |  |  |  |  |  |  |  |  |  |  |  |  |  |  |  |  |  |  |  |  |  |  |  |  |  |  |  |  |  |  |  |  |  |  |  |  |  |  |  |  |  |  |  |  |  |  |  |  |  |  |  |  |  |  |  |  |  |  |  |  |  |  |  |  |  |  |  |  |  |  |  |  |  |  |  |  |  |  |  |  |  |  |  |  |  |  |  |  |  |  |  |  |  |  |  |  |  |  |  |  |  |  |  |  |  |  |  |  |  |  |  |  |  |  |  |  |  |  |  |  |  |  |  |  |  |  |  |  |  |  |  |  |  |  |  |  |  |  |  |  |  |  |  |  |  |  |  |  |  |  |  |  |  |  |  |  |  |  |  |  |  |  |  |  |  |  |  |  |  |  |  |  |  |  |  |  |  |  |  |  |  |  |  |  |  |  |  |  |  |  |  |  |  |  |  |  |  |  |  |  |  |  |  |  |  |  |  |  |  |  |  |  |  |  |  |  |  |  |  |  |  |  |  |  |  |  |  |  |  |  |  |  |  |  |  |  |  |  |  |  |  |  |  |  |  |  |  |  |  |  |  |  |  |  |  |  |  |  |  |  |  |  |  |  |  |  |  |  |  |  |  |  |  |  |  |  |  |  |  |  |  |  |  |  |  |  |  |  |  |  |  |  |  |  |  |  |  |  |  |  |  |  |  |  |  |  |  |  |  |  |  |  |  |  |  |  |  |  |  |  |  |  |  |  |  |  |  |  |  |  |  |  |  |  |  |  |  |  |  |  |  |  |  |  |  |  |  |  |  |  |  |  |  |  |  |  |  |  |  |  |  |  |  |  |  |  |  |  |  |  |  |  |  |  |  |  |  |  |  |  |  |  |  |  |  |  |  |  |  |  |  |  |  |  |  |  |  |  |  |  |  |  |  |  |  |  |  |  |  |  |  |  |  |  |  |  |  |  |  |  |  |  |  |  |  |  |  |  |  |  |  |  |  |  |  |  |  |  |  |  |  |  |  |  |  |  |  |  |  |  |  |  |  |  |  |  |  |  |  |  |  |  |  |  |  |  |  |  |  |  |  |  |  |  |  |  |  |  |  |  |  |  |  |  |  |  |  |  |  |  |  |  |  |  |  |  |  |  |  |  |  |  |  |  |  |  |  |  |  |  |  |  |  |  |  |  |  |  |  |  |  |  |  |  |  |  |  |  |  |  |  |  |  |  |  |  |  |  |  |  |  |  |  |  |  |  |  |  |  |  |  |  |  |  |  |  |  |  |  |  |  |  |  |  |  |  |  |  |  |  |  |  |  |  |  |  |  |  |  |  |  |  |  |  |  |  |  |  |  |  |  |  |  |  |  |  |  |  |  |  |  |  |  |  |  |  |  |  |  |  |  |  |  |  |  |  |  |  |  |  |  |  |  |  |  |  |  |  |  |  |  |  |  |  |  |  |  |  |  |  |  |  |  |  |  |  |  |  |  |  |  |  |  |  |  |  |  |  |  |  |  |  |  |  |  |  |  |  |  |  |  |  |  |  |  |  |  |  |  |  |  |  |  |  |  |  |  |  |  |  |  |  |  |  |  |  |  |  |  |  |  |  |  |  |  |  |  |  |  |  |  |  |  |  |  |  |  |  |  |  |  |  |  |  |  |  |  |  |  |  |  |  |  |  |  |  |  |  |  |  |  |  |  |  |  |  |  |  |  |  |  |  |  |  |  |  |  |  |  |  |  |  |  |  |  |  |  |  |  |  |  |  |  |  |  |  |  |  |  |  |  |  |  |  |  |  |  |  |  |  |  |  |  |  |  |  |  |  |  |  |  |  |  |  |  |  |  |  |  |  |  |  |  |  |  |  |  |  |  |  |  |  |  |  |  |  |  |  |  |  |  |  |  |  |  |  |  |  |  |  |  |  |  |  |  |  |  |  |  |  |  |  |  |  |  |  |  |  |  |  |  |  |  |  |  |  |  |  |  |  |  |  |  |  |  |  |  |  |  |  |  |  |  |  |  |  |  |  |  |  |  |  |  |  |  |  |  |  |  |  |  |  |  |  |  |  |  |  |  |  |  |  |  |  |  |  |  |  |  |  |  |  |  |  |  |  |  |  |  |  |  |  |  |  |  |  |  |  |  |  |  |  |  |  |  |  |  |  |  |  |  |  |  |  |  |  |  |  |  |  |  |  |  |  |  |  |  |  |  |  |  |  |  |  |  |  |  |  |  |  |  |  |  |  |  |  |  |  |  |  |  |  |  |  |  |  |  |  |  |  |  |  |  |  |  |  |  |  |  |  |  |  |  |  |  |  |  |  |  |  |  |  |  |  |  |  |  |  |  |  |  |  |  |  |  |  |  |  |  |  |  |  |  |  |  |  |  |  |  |  |  |  |  |  |  |  |  |  |  |  |  |  |  |  |  |  |  |  |  |  |  |  |  |  |  |  |  |  |  |  |  |  |  |  |  |  |  |  |  |  |  |  |  |  |  |  |  |  |  |  |  |  |  |  |  |  |  |  |  |  |  |  |  |  |  |  |  |  |  |  |  |  |  |  |  |  |  |  |  |  |  |  |  |  |  |  |  |  |  |  |  |  |  |  |  |  |  |  |  |  |  |  |  |  |  |  |  |  |  |  |  |  |  |  |  |  |  |  |  |  |  |  |  |  |  |  |  |  |  |  |  |  |  |  |  |  |  |  |  |  |  |  |  |  |  |  |  |  |  |  |  |  |  |  |  |  |  |  |  |  |  |  |  |  |  |  |  |  |  |  |  |  |  |  |  |  |  |  |  |  |  |  |  |  |  |  |  |  |  |  |  |  |  |  |  |  |  |  |  |  |  |  |  |  |  |  |  |  |  |  |  |  |  |  |  |  |  |  |  |  |  |  |  |  |  |  |  |  |  |  |  |  |  |  |  |  |  |  |  |  |  |  |  |  |  |  |  |  |  |  |  |  |  |  |  |  |  |  |  |  |  |  |  |  |  |  |  |  |  |  |  |  |  |  |  |  |  |  |  |  |  |  |  |  |  |  |  |  |  |  |  |  |  |  |  |  |  |  |  |  |  |  |  |  |  |  |  |  |  |  |  |  |  |  |  |  |  |  |  |  |  |  |  |  |  |  |  |  |  |  |  |  |  |  |  |  |  |  |  |  |  |  |  |  |  |  |  |  |  |  |  |  |  |  |  |  |  |  |  |  |  |  |  |  |  |  |  |  |  |  |  |  |  |  |  |  |  |  |  |  |  |  |  |  |  |  |  |  |  |  |  |  |  |  |  |  |  |  |  |  |  |  |  |  |  |  |  |  |  |  |  |  |  |  |  |  |  |  |  |  |  |  |  |  |  |  |  |  |  |  |  |  |  |  |  |  |  |  |  |  |  |  |  |  |  |  |  |  |  |  |  |  |  |  |  |  |  |  |  |  |  |  |  |  |  |  |  |  |  |  |  |  |  |  |  |  |  |  |  |  |  |  |  |  |  |  |  |  |  |  |  |  |  |  |  |  |  |  |  |  |  |  |  |  |  |  |  |  |  |  |  |  |  |  |  |  |  |  |  |  |  |  |  |  |  |  |  |  |  |  |  |  |  |  |  |  |  |  |  |  |  |  |  |  |  |  |  |  |  |  |  |  |  |  |  |  |  |  |  |  |  |  |  |  |  |  |  |  |  |  |  |  |  |  |  |  |  |  |  |  |  |  |  |  |  |  |  |  |  |  |  |  |  |  |  |  |  |  |  |  |  |  |  |  |  |  |  |  |  |  |  |  |  |  |  |  |  |  |  |  |  |  |  |  |  |  |  |  |  |  |  |  |  |  |  |  |  |  |  |  |  |  |  |  |  |  |  |  |  |  |  |  |  |  |  |  |  |  |  |  |  |  |  |  |  |  |  |  |  |  |  |  |  |  |  |  |  |  |  |  |  |  |  |  |  |  |  |  |  |  |  |  |  |  |  |  |  |  |  |  |  |  |  |  |  |  |  |  |  |  |  |  |  |  |  |  |  |  |  |  |  |  |  |  |  |  |  |  |  |  |  |  |  |  |  |  |  |  |  |  |  |  |  |  |  |  |  |  |  |  |  |  |  |  |  |  |  |  |  |  |  |  |  |  |  |  |  |  |  |  |  |  |  |  |  |  |  |  |  |  |  |  |  |  |  |  |  |  |  |  |  |  |  |  |  |  |  |  |  |  |  |  |  |  |  |  |  |  |  |  |  |  |  |  |  |  |  |  |  |  |  |  |  |  |  |  |  |  |  |  |  |  |  |  |  |  |  |  |  |  |  |  |  |  |  |  |  |  |  |  |  |  |  |  |  |  |  |  |  |  |  |  |  |  |  |  |  |  |  |  |  |  |  |  |  |  |  |  |  |  |  |  |  |  |  |  |  |  |  |  |  |  |  |  |  |  |  |  |  |  |  |  |  |  |  |  |  |  |  |  |  |  |  |  |  |  |  |  |  |  |  |  |  |  |  |  |  |  |  |  |  |  |  |  |  |  |  |  |  |  |  |  |  |  |  |  |  |  |  |  |  |  |  |  |  |  |  |  |  |  |  |  |  |  |  |  |  |  |  |  |  |  |  |  |  |  |  |  |  |  |  |  |  |  |  |  |  |  |  |  |  |  |  |  |  |  |  |  |  |  |  |  |  |  |  |  |  |  |  |  |  |  |  |  |  |  |  |  |  |  |  |  |  |  |  |  |  |  |  |  |  |  |  |  |  |  |  |  |  |  |  |  |  |  |  |  |  |  |  |  |  |  |  |  |  |  |  |  |  |  |  |  |  |  |  |  |  |  |  |  |  |  |  |  |  |  |  |  |  |  |  |  |  |  |  |  |  |  |  |  |  |  |  |  |  |  |  |  |  |  |  |  |  |  |  |  |  |  |  |  |  |  |  |  |  |  |  |  |  |  |  |  |  |  |  |  |  |  |  |  |  |  |  |  |  |  |  |  |  |  |  |  |  |  |  |  |  |  |  |  |  |  |  |  |  |  |  |  |  |  |  |  |  |  |  |  |  |  |  |  |  |  |  |  |  |  |  |  |  |  |  |  |  |  |  |  |  |  |  |  |  |  |  |  |  |  |  |  |  |  |  |  |  |  |  |  |  |  |  |  |  |  |  |  |  |  |  |  |  |  |  |  |  |  |  |  |  |  |  |  |  |  |  |  |  |  |  |  |  |  |  |  |  |  |  |  |  |  |  |  |  |  |  |  |  |  |  |  |  |  |  |  |  |  |  |  |  |  |  |  |  |  |  |  |  |  |  |  |  |  |  |  |  |  |  |  |  |  |  |  |  |  |  |  |  |  |  |  |  |  |  |  |  |  |  |  |  |  |  |  |  |  |  |  |  |  |  |  |  |  |  |  |  |  |  |  |  |  |  |  |  |  |  |  |  |  |  |  |  |  |  |  |  |  |  |  |  |  |  |  |  |  |  |  |  |  |  |  |  |  |  |  |  |  |  |  |  |  |  |  |  |  |  |  |  |  |  |  |  |  |  |  |  |  |  |  |  |  |  |  |  |  |  |  |  |  |  |  |  |  |  |  |  |  |  |  |  |  |  |  |  |  |  |  |  |  |  |  |  |  |  |  |  |  |  |  |  |  |  |  |  |  |  |  |  |  |  |  |  |  |  |  |  |  |  |  |  |  |  |  |  |  |  |  |  |  |  |  |  |  |  |  |  |  |  |  |  |  |  |  |  |  |  |  |  |  |  |  |  |  |  |  |  |  |  |  |  |  |  |  |  |  |  |  |  |  |  |  |  |  |  |  |  |  |  |  |  |  |  |  |  |  |  |  |  |  |  |  |  |  |  |  |  |  |  |  |  |  |  |  |  |  |  |  |  |  |  |  |  |  |  |  |  |  |  |  |  |  |  |  |  |  |  |  |  |  |  |  |  |  |  |  |  |  |  |  |  |  |  |  |  |  |  |  |  |  |  |  |  |  |  |  |  |  |  |  |  |  |  |  |  |  |  |  |  |  |  |  |  |  |  |  |  |  |  |  |  |  |  |  |  |  |  |  |  |  |  |  |  |  |  |  |  |  |  |  |  |  |  |  |  |  |  |  |  |  |  |  |  |  |  |  |  |  |  |  |  |  |  |  |  |  |  |  |  |  |  |  |  |  |  |  |  |  |  |  |  |  |  |  |  |  |  |  |  |  |  |  |  |  |  |  |  |  |  |  |  |  |  |  |  |  |  |  |  |  |  |  |  |  |  |  |  |
| --- | --- | --- | --- | --- | --- | --- | --- | --- | --- | --- | --- | --- | --- | --- | --- | --- | --- | --- | --- | --- | --- | --- | --- | --- | --- | --- | --- | --- | --- | --- | --- | --- | --- | --- | --- | --- | --- | --- | --- | --- | --- | --- | --- | --- | --- | --- | --- | --- | --- | --- | --- | --- | --- | --- | --- | --- | --- | --- | --- | --- | --- | --- | --- | --- | --- | --- | --- | --- | --- | --- | --- | --- | --- | --- | --- | --- | --- | --- | --- | --- | --- | --- | --- | --- | --- | --- | --- | --- | --- | --- | --- | --- | --- | --- | --- | --- | --- | --- | --- | --- | --- | --- | --- | --- | --- | --- | --- | --- | --- | --- | --- | --- | --- | --- | --- | --- | --- | --- | --- | --- | --- | --- | --- | --- | --- | --- | --- | --- | --- | --- | --- | --- | --- | --- | --- | --- | --- | --- | --- | --- | --- | --- | --- | --- | --- | --- | --- | --- | --- | --- | --- | --- | --- | --- | --- | --- | --- | --- | --- | --- | --- | --- | --- | --- | --- | --- | --- | --- | --- | --- | --- | --- | --- | --- | --- | --- | --- | --- | --- | --- | --- | --- | --- | --- | --- | --- | --- | --- | --- | --- | --- | --- | --- | --- | --- | --- | --- | --- | --- | --- | --- | --- | --- | --- | --- | --- | --- | --- | --- | --- | --- | --- | --- | --- | --- | --- | --- | --- | --- | --- | --- | --- | --- | --- | --- | --- | --- | --- | --- | --- | --- | --- | --- | --- | --- | --- | --- | --- | --- | --- | --- | --- | --- | --- | --- | --- | --- | --- | --- | --- | --- | --- | --- | --- | --- | --- | --- | --- | --- | --- | --- | --- | --- | --- | --- | --- | --- | --- | --- | --- | --- | --- | --- | --- | --- | --- | --- | --- | --- | --- | --- | --- | --- | --- | --- | --- | --- | --- | --- | --- | --- | --- | --- | --- | --- | --- | --- | --- | --- | --- | --- | --- | --- | --- | --- | --- | --- | --- | --- | --- | --- | --- | --- | --- | --- | --- | --- | --- | --- | --- | --- | --- | --- | --- | --- | --- | --- | --- | --- | --- | --- | --- | --- | --- | --- | --- | --- | --- | --- | --- | --- | --- | --- | --- | --- | --- | --- | --- | --- | --- | --- | --- | --- | --- | --- | --- | --- | --- | --- | --- | --- | --- | --- | --- | --- | --- | --- | --- | --- | --- | --- | --- | --- | --- | --- | --- | --- | --- | --- | --- | --- | --- | --- | --- | --- | --- | --- | --- | --- | --- | --- | --- | --- | --- | --- | --- | --- | --- | --- | --- | --- | --- | --- | --- | --- | --- | --- | --- | --- | --- | --- | --- | --- | --- | --- | --- | --- | --- | --- | --- | --- | --- | --- | --- | --- | --- | --- | --- | --- | --- | --- | --- | --- | --- | --- | --- | --- | --- | --- | --- | --- | --- | --- | --- | --- | --- | --- | --- | --- | --- | --- | --- | --- | --- | --- | --- | --- | --- | --- | --- | --- | --- | --- | --- | --- | --- | --- | --- | --- | --- | --- | --- | --- | --- | --- | --- | --- | --- | --- | --- | --- | --- | --- | --- | --- | --- | --- | --- | --- | --- | --- | --- | --- | --- | --- | --- | --- | --- | --- | --- | --- | --- | --- | --- | --- | --- | --- | --- | --- | --- | --- | --- | --- | --- | --- | --- | --- | --- | --- | --- | --- | --- | --- | --- | --- | --- | --- | --- | --- | --- | --- | --- | --- | --- | --- | --- | --- | --- | --- | --- | --- | --- | --- | --- | --- | --- | --- | --- | --- | --- | --- | --- | --- | --- | --- | --- | --- | --- | --- | --- | --- | --- | --- | --- | --- | --- | --- | --- | --- | --- | --- | --- | --- | --- | --- | --- | --- | --- | --- | --- | --- | --- | --- | --- | --- | --- | --- | --- | --- | --- | --- | --- | --- | --- | --- | --- | --- | --- | --- | --- | --- | --- | --- | --- | --- | --- | --- | --- | --- | --- | --- | --- | --- | --- | --- | --- | --- | --- | --- | --- | --- | --- | --- | --- | --- | --- | --- | --- | --- | --- | --- | --- | --- | --- | --- | --- | --- | --- | --- | --- | --- | --- | --- | --- | --- | --- | --- | --- | --- | --- | --- | --- | --- | --- | --- | --- | --- | --- | --- | --- | --- | --- | --- | --- | --- | --- | --- | --- | --- | --- | --- | --- | --- | --- | --- | --- | --- | --- | --- | --- | --- | --- | --- | --- | --- | --- | --- | --- | --- | --- | --- | --- | --- | --- | --- | --- | --- | --- | --- | --- | --- | --- | --- | --- | --- | --- | --- | --- | --- | --- | --- | --- | --- | --- | --- | --- | --- | --- | --- | --- | --- | --- | --- | --- | --- | --- | --- | --- | --- | --- | --- | --- | --- | --- | --- | --- | --- | --- | --- | --- | --- | --- | --- | --- | --- | --- | --- | --- | --- | --- | --- | --- | --- | --- | --- | --- | --- | --- | --- | --- | --- | --- | --- | --- | --- | --- | --- | --- | --- | --- | --- | --- | --- | --- | --- | --- | --- | --- | --- | --- | --- | --- | --- | --- | --- | --- | --- | --- | --- | --- | --- | --- | --- | --- | --- | --- | --- | --- | --- | --- | --- | --- | --- | --- | --- | --- | --- | --- | --- | --- | --- | --- | --- | --- | --- | --- | --- | --- | --- | --- | --- | --- | --- | --- | --- | --- | --- | --- | --- | --- | --- | --- | --- | --- | --- | --- | --- | --- | --- | --- | --- | --- | --- | --- | --- | --- | --- | --- | --- | --- | --- | --- | --- | --- | --- | --- | --- | --- | --- | --- | --- | --- | --- | --- | --- | --- | --- | --- | --- | --- | --- | --- | --- | --- | --- | --- | --- | --- | --- | --- | --- | --- | --- | --- | --- | --- | --- | --- | --- | --- | --- | --- | --- | --- | --- | --- | --- | --- | --- | --- | --- | --- | --- | --- | --- | --- | --- | --- | --- | --- | --- | --- | --- | --- | --- | --- | --- | --- | --- | --- | --- | --- | --- | --- | --- | --- | --- | --- | --- | --- | --- | --- | --- | --- | --- | --- | --- | --- | --- | --- | --- | --- | --- | --- | --- | --- | --- | --- | --- | --- | --- | --- | --- | --- | --- | --- | --- | --- | --- | --- | --- | --- | --- | --- | --- | --- | --- | --- | --- | --- | --- | --- | --- | --- | --- | --- | --- | --- | --- | --- | --- | --- | --- | --- | --- | --- | --- | --- | --- | --- | --- | --- | --- | --- | --- | --- | --- | --- | --- | --- | --- | --- | --- | --- | --- | --- | --- | --- | --- | --- | --- | --- | --- | --- | --- | --- | --- | --- | --- | --- | --- | --- | --- | --- | --- | --- | --- | --- | --- | --- | --- | --- | --- | --- | --- | --- | --- | --- | --- | --- | --- | --- | --- | --- | --- | --- | --- | --- | --- | --- | --- | --- | --- | --- | --- | --- | --- | --- | --- | --- | --- | --- | --- | --- | --- | --- | --- | --- | --- | --- | --- | --- | --- | --- | --- | --- | --- | --- | --- | --- | --- | --- | --- | --- | --- | --- | --- | --- | --- | --- | --- | --- | --- | --- | --- | --- | --- | --- | --- | --- | --- | --- | --- | --- | --- | --- | --- | --- | --- | --- | --- | --- | --- | --- | --- | --- | --- | --- | --- | --- | --- | --- | --- | --- | --- | --- | --- | --- | --- | --- | --- | --- | --- | --- | --- | --- | --- | --- | --- | --- | --- | --- | --- | --- | --- | --- | --- | --- | --- | --- | --- | --- | --- | --- | --- | --- | --- | --- | --- | --- | --- | --- | --- | --- | --- | --- | --- | --- | --- | --- | --- | --- | --- | --- | --- | --- | --- | --- | --- | --- | --- | --- | --- | --- | --- | --- | --- | --- | --- | --- | --- | --- | --- | --- | --- | --- | --- | --- | --- | --- | --- | --- | --- | --- | --- | --- | --- | --- | --- | --- | --- | --- | --- | --- | --- | --- | --- | --- | --- | --- | --- | --- | --- | --- | --- | --- | --- | --- | --- | --- | --- | --- | --- | --- | --- | --- | --- | --- | --- | --- | --- | --- | --- | --- | --- | --- | --- | --- | --- | --- | --- | --- | --- | --- | --- | --- | --- | --- | --- | --- | --- | --- | --- | --- | --- | --- | --- | --- | --- | --- | --- | --- | --- | --- | --- | --- | --- | --- | --- | --- | --- | --- | --- | --- | --- | --- | --- | --- | --- | --- | --- | --- | --- | --- | --- | --- | --- | --- | --- | --- | --- | --- | --- | --- | --- | --- | --- | --- | --- | --- | --- | --- | --- | --- | --- | --- | --- | --- | --- | --- | --- | --- | --- | --- | --- | --- | --- | --- | --- | --- | --- | --- | --- | --- | --- | --- | --- | --- | --- | --- | --- | --- | --- | --- | --- | --- | --- | --- | --- | --- | --- | --- | --- | --- | --- | --- | --- | --- | --- | --- | --- | --- | --- | --- | --- | --- | --- | --- | --- | --- | --- | --- | --- | --- | --- | --- | --- | --- | --- | --- | --- | --- | --- | --- | --- | --- | --- | --- | --- | --- | --- | --- | --- | --- | --- | --- | --- | --- | --- | --- | --- | --- | --- | --- | --- | --- | --- | --- | --- | --- | --- | --- | --- | --- | --- | --- | --- | --- | --- | --- | --- | --- | --- | --- | --- | --- | --- | --- | --- | --- | --- | --- | --- | --- | --- | --- | --- | --- | --- | --- | --- | --- | --- | --- | --- | --- | --- | --- | --- | --- | --- | --- | --- | --- | --- | --- | --- | --- | --- | --- | --- | --- | --- | --- | --- | --- | --- | --- | --- | --- | --- | --- | --- | --- | --- | --- | --- | --- | --- | --- | --- | --- | --- | --- | --- | --- | --- | --- | --- | --- | --- | --- | --- | --- | --- | --- | --- | --- | --- | --- | --- | --- | --- | --- | --- | --- | --- | --- | --- | --- | --- | --- | --- | --- | --- | --- | --- | --- | --- | --- | --- | --- | --- | --- | --- | --- | --- | --- | --- | --- | --- | --- | --- | --- | --- | --- | --- | --- | --- | --- | --- | --- | --- | --- | --- | --- | --- | --- | --- | --- | --- | --- | --- | --- | --- | --- | --- | --- | --- | --- | --- | --- | --- | --- | --- | --- | --- | --- | --- | --- | --- | --- | --- | --- | --- | --- | --- | --- | --- | --- | --- | --- | --- | --- | --- | --- | --- | --- | --- | --- | --- | --- | --- | --- | --- | --- | --- | --- | --- | --- | --- | --- | --- | --- | --- | --- | --- | --- | --- | --- | --- | --- | --- | --- | --- | --- | --- | --- | --- | --- | --- | --- | --- | --- | --- | --- | --- | --- | --- | --- | --- | --- | --- | --- | --- | --- | --- | --- | --- | --- | --- | --- | --- | --- | --- | --- | --- | --- | --- | --- | --- | --- | --- | --- | --- | --- | --- | --- | --- | --- | --- | --- | --- | --- | --- | --- | --- | --- | --- | --- | --- | --- | --- | --- | --- | --- | --- | --- | --- | --- | --- | --- | --- | --- | --- | --- | --- | --- | --- | --- | --- | --- | --- | --- | --- | --- | --- | --- | --- | --- | --- | --- | --- | --- | --- | --- | --- | --- | --- | --- | --- | --- | --- | --- | --- | --- | --- | --- | --- | --- | --- | --- | --- | --- | --- | --- | --- | --- | --- | --- | --- | --- | --- | --- | --- | --- | --- | --- | --- | --- | --- | --- | --- | --- | --- | --- | --- | --- | --- | --- | --- | --- | --- | --- | --- | --- | --- | --- | --- | --- | --- | --- | --- | --- | --- | --- | --- | --- | --- | --- | --- | --- | --- | --- | --- | --- | --- | --- | --- | --- | --- | --- | --- | --- | --- | --- | --- | --- | --- | --- | --- | --- | --- | --- | --- | --- | --- | --- | --- | --- | --- | --- | --- | --- | --- | --- | --- | --- | --- | --- | --- | --- | --- | --- | --- | --- | --- | --- | --- | --- | --- | --- | --- | --- | --- | --- | --- | --- | --- | --- | --- | --- | --- | --- | --- | --- | --- | --- | --- | --- | --- | --- | --- | --- | --- | --- | --- | --- | --- | --- | --- | --- | --- | --- | --- | --- | --- | --- | --- | --- | --- | --- | --- | --- | --- | --- | --- | --- | --- | --- | --- | --- | --- | --- | --- | --- | --- | --- | --- | --- | --- | --- | --- | --- | --- | --- | --- | --- | --- | --- | --- | --- | --- | --- | --- | --- | --- | --- | --- | --- | --- | --- | --- | --- | --- | --- | --- | --- | --- | --- | --- | --- | --- | --- | --- | --- | --- | --- | --- | --- | --- | --- | --- | --- | --- | --- | --- | --- | --- | --- | --- | --- | --- | --- | --- | --- | --- | --- | --- | --- | --- | --- | --- | --- | --- | --- | --- | --- | --- | --- | --- | --- | --- | --- | --- | --- | --- | --- | --- | --- | --- | --- | --- | --- | --- | --- | --- | --- | --- | --- | --- | --- | --- | --- | --- | --- | --- | --- | --- | --- | --- | --- | --- | --- | --- | --- | --- | --- | --- | --- | --- | --- | --- | --- | --- | --- | --- | --- | --- | --- | --- | --- | --- | --- | --- | --- | --- | --- | --- | --- | --- | --- | --- | --- | --- | --- | --- | --- | --- | --- | --- | --- | --- | --- | --- | --- | --- | --- | --- | --- | --- | --- | --- | --- | --- | --- | --- | --- | --- | --- | --- | --- | --- | --- | --- | --- | --- | --- | --- | --- | --- | --- | --- | --- | --- | --- | --- | --- | --- | --- | --- | --- | --- | --- | --- | --- | --- | --- | --- | --- | --- | --- | --- | --- | --- | --- | --- | --- | --- | --- | --- | --- | --- | --- | --- | --- | --- | --- | --- | --- | --- | --- | --- | --- | --- | --- | --- | --- | --- | --- | --- | --- | --- | --- | --- | --- | --- | --- | --- | --- | --- | --- | --- | --- | --- | --- | --- | --- | --- | --- | --- | --- | --- | --- | --- | --- | --- | --- | --- | --- | --- | --- | --- | --- | --- | --- | --- | --- | --- | --- | --- | --- | --- | --- | --- | --- | --- | --- | --- | --- | --- | --- | --- | --- | --- | --- | --- | --- | --- | --- | --- | --- | --- | --- | --- | --- | --- | --- | --- | --- | --- | --- | --- | --- | --- | --- | --- | --- | --- | --- | --- | --- | --- | --- | --- | --- | --- | --- | --- | --- | --- | --- | --- | --- | --- | --- | --- | --- | --- | --- | --- | --- | --- | --- | --- | --- | --- | --- | --- | --- | --- | --- | --- | --- | --- | --- | --- | --- | --- | --- | --- | --- | --- | --- | --- | --- | --- | --- | --- | --- | --- | --- | --- | --- | --- | --- | --- | --- | --- | --- | --- | --- | --- | --- | --- | --- | --- | --- | --- | --- | --- | --- | --- | --- | --- | --- | --- | --- | --- | --- | --- | --- | --- | --- | --- | --- | --- | --- | --- | --- | --- | --- | --- | --- | --- | --- | --- | --- | --- | --- | --- | --- | --- | --- | --- | --- | --- | --- | --- | --- | --- | --- | --- | --- | --- | --- | --- | --- | --- | --- | --- | --- | --- | --- | --- | --- | --- | --- | --- | --- | --- | --- | --- | --- | --- | --- | --- | --- | --- | --- | --- | --- | --- | --- | --- | --- | --- | --- | --- | --- | --- | --- | --- | --- | --- | --- | --- | --- | --- | --- | --- | --- | --- | --- | --- | --- | --- | --- | --- | --- | --- | --- | --- | --- | --- | --- | --- | --- | --- | --- | --- | --- | --- | --- | --- | --- | --- | --- | --- | --- | --- | --- | --- | --- | --- | --- | --- | --- | --- | --- | --- | --- | --- | --- | --- | --- | --- | --- | --- | --- | --- | --- | --- | --- | --- | --- | --- | --- | --- | --- | --- | --- | --- | --- | --- | --- | --- | --- | --- | --- | --- | --- | --- | --- | --- | --- | --- | --- | --- | --- | --- | --- | --- | --- | --- | --- | --- | --- | --- | --- | --- | --- | --- | --- | --- | --- | --- | --- | --- | --- | --- | --- | --- | --- | --- | --- | --- | --- | --- | --- | --- | --- | --- | --- | --- | --- | --- | --- | --- | --- | --- | --- | --- | --- | --- | --- | --- | --- | --- | --- | --- | --- | --- | --- | --- | --- | --- | --- | --- | --- | --- | --- | --- | --- | --- | --- | --- | --- | --- | --- | --- | --- | --- | --- | --- | --- | --- | --- | --- | --- | --- | --- | --- | --- | --- | --- | --- | --- | --- | --- | --- | --- | --- | --- | --- | --- | --- | --- | --- | --- | --- | --- | --- | --- | --- | --- | --- | --- | --- | --- | --- | --- | --- | --- | --- | --- | --- | --- | --- | --- | --- | --- | --- | --- | --- | --- | --- | --- | --- | --- | --- | --- | --- | --- | --- | --- | --- | --- | --- | --- | --- | --- | --- | --- | --- | --- | --- | --- | --- | --- | --- | --- | --- | --- | --- | --- | --- | --- | --- | --- | --- | --- | --- | --- | --- | --- | --- | --- | --- | --- | --- | --- | --- | --- | --- | --- | --- | --- | --- | --- | --- | --- | --- | --- | --- | --- | --- | --- | --- | --- | --- | --- | --- | --- | --- | --- | --- | --- | --- | --- | --- | --- | --- | --- | --- | --- | --- | --- | --- | --- | --- | --- | --- | --- | --- | --- | --- | --- | --- | --- | --- | --- | --- | --- | --- | --- | --- | --- | --- | --- | --- | --- | --- | --- | --- | --- | --- | --- | --- | --- | --- | --- | --- | --- | --- | --- | --- | --- | --- | --- | --- | --- | --- | --- | --- | --- | --- | --- | --- | --- | --- | --- | --- | --- | --- | --- | --- | --- | --- | --- | --- | --- | --- | --- | --- | --- | --- | --- | --- | --- | --- | --- | --- | --- | --- | --- | --- | --- | --- | --- | --- | --- | --- | --- | --- | --- | --- | --- | --- | --- | --- | --- | --- | --- | --- | --- | --- | --- | --- | --- | --- | --- | --- | --- | --- | --- | --- | --- | --- | --- | --- | --- | --- | --- | --- | --- | --- | --- | --- | --- | --- | --- | --- | --- | --- | --- | --- | --- | --- | --- | --- | --- | --- | --- | --- | --- | --- | --- | --- | --- | --- | --- | --- | --- | --- | --- | --- | --- | --- | --- | --- | --- | --- | --- | --- | --- | --- | --- | --- | --- | --- | --- | --- | --- | --- | --- | --- | --- | --- | --- | --- | --- | --- | --- | --- | --- | --- | --- | --- | --- | --- | --- | --- | --- | --- | --- | --- | --- | --- | --- | --- | --- | --- | --- | --- | --- | --- | --- | --- | --- | --- | --- | --- | --- | --- | --- | --- | --- | --- | --- | --- | --- | --- | --- | --- | --- | --- | --- | --- | --- | --- | --- | --- | --- | --- | --- | --- | --- | --- | --- | --- | --- | --- | --- | --- | --- | --- | --- | --- | --- | --- | --- | --- | --- | --- | --- | --- | --- | --- | --- | --- | --- | --- | --- | --- | --- | --- | --- | --- | --- | --- | --- | --- | --- | --- | --- | --- | --- | --- | --- | --- | --- | --- | --- | --- | --- | --- | --- | --- | --- | --- | --- | --- | --- | --- | --- | --- | --- | --- | --- | --- | --- | --- | --- | --- | --- | --- | --- | --- | --- | --- | --- | --- | --- | --- | --- | --- | --- | --- | --- | --- | --- | --- | --- | --- | --- | --- | --- | --- | --- | --- | --- | --- | --- | --- | --- | --- | --- | --- | --- | --- | --- | --- | --- | --- | --- | --- | --- | --- | --- | --- | --- | --- | --- | --- | --- | --- | --- | --- | --- | --- | --- | --- | --- | --- | --- | --- | --- | --- | --- | --- | --- | --- | --- | --- | --- | --- | --- | --- | --- | --- | --- | --- | --- | --- | --- | --- | --- | --- | --- | --- | --- | --- | --- | --- | --- | --- | --- | --- | --- | --- | --- | --- | --- | --- | --- | --- | --- | --- | --- | --- | --- | --- | --- | --- | --- | --- | --- | --- | --- | --- | --- | --- | --- | --- | --- | --- | --- | --- | --- | --- | --- | --- | --- | --- | --- | --- | --- | --- | --- | --- | --- | --- | --- | --- | --- | --- | --- | --- | --- | --- | --- | --- | --- | --- | --- | --- | --- | --- | --- | --- | --- | --- | --- | --- | --- | --- | --- | --- | --- | --- | --- | --- | --- | --- | --- | --- | --- | --- | --- | --- | --- | --- | --- | --- | --- | --- | --- | --- | --- | --- | --- | --- | --- | --- | --- | --- | --- | --- | --- | --- | --- | --- | --- | --- | --- | --- | --- | --- | --- | --- | --- | --- | --- | --- | --- | --- | --- | --- | --- | --- | --- | --- | --- | --- | --- | --- | --- | --- | --- | --- | --- | --- | --- | --- | --- | --- | --- | --- | --- | --- | --- | --- | --- | --- | --- | --- | --- | --- | --- | --- | --- | --- | --- | --- | --- | --- | --- | --- | --- | --- | --- | --- | --- | --- | --- | --- | --- | --- | --- | --- | --- | --- | --- | --- | --- | --- | --- | --- | --- | --- | --- | --- | --- | --- | --- | --- | --- | --- | --- | --- | --- | --- | --- | --- | --- | --- | --- | --- | --- | --- | --- | --- | --- | --- | --- | --- | --- | --- | --- | --- | --- | --- | --- | --- | --- | --- | --- | --- | --- | --- | --- | --- | --- | --- | --- | --- | --- | --- | --- | --- | --- | --- | --- | --- | --- | --- | --- | --- | --- | --- | --- | --- | --- | --- | --- | --- | --- | --- | --- | --- | --- | --- | --- | --- | --- | --- | --- | --- | --- | --- | --- | --- | --- | --- | --- | --- | --- | --- | --- | --- | --- | --- | --- | --- | --- | --- | --- | --- | --- | --- | --- | --- | --- | --- | --- | --- | --- | --- | --- | --- | --- | --- | --- | --- | --- | --- | --- | --- | --- | --- | --- | --- | --- | --- | --- | --- | --- | --- | --- | --- | --- | --- | --- | --- | --- | --- | --- | --- | --- | --- | --- | --- | --- | --- | --- | --- | --- | --- | --- | --- | --- | --- | --- | --- | --- | --- | --- | --- | --- | --- | --- | --- | --- | --- | --- | --- | --- | --- | --- | --- | --- | --- | --- | --- | --- | --- | --- | --- | --- | --- | --- | --- | --- | --- | --- | --- | --- | --- | --- | --- | --- | --- | --- | --- | --- | --- | --- | --- | --- | --- | --- | --- | --- | --- | --- | --- | --- | --- | --- | --- | --- | --- | --- | --- | --- | --- | --- | --- | --- | --- | --- | --- | --- | --- | --- | --- | --- | --- | --- | --- | --- | --- | --- | --- | --- | --- | --- | --- | --- | --- | --- | --- | --- | --- | --- | --- | --- | --- | --- | --- | --- | --- | --- | --- | --- | --- | --- | --- | --- | --- | --- | --- | --- | --- | --- | --- | --- | --- | --- | --- | --- | --- | --- | --- | --- | --- | --- | --- | --- | --- | --- | --- | --- | --- | --- | --- | --- | --- | --- | --- | --- | --- | --- | --- | --- | --- | --- | --- | --- | --- | --- | --- | --- | --- | --- | --- | --- | --- | --- | --- | --- | --- | --- | --- | --- | --- | --- | --- | --- | --- | --- | --- | --- | --- | --- | --- | --- | --- | --- | --- | --- | --- | --- | --- | --- | --- | --- | --- | --- | --- | --- | --- | --- | --- | --- | --- | --- | --- | --- | --- | --- | --- | --- | --- | --- | --- | --- | --- | --- | --- | --- | --- | --- | --- | --- | --- | --- | --- | --- | --- | --- | --- | --- | --- | --- | --- | --- | --- | --- | --- | --- | --- | --- | --- | --- | --- | --- | --- | --- | --- | --- | --- | --- | --- | --- | --- | --- | --- | --- | --- | --- | --- | --- | --- | --- | --- | --- | --- | --- | --- | --- | --- | --- | --- | --- | --- | --- | --- | --- | --- | --- | --- | --- | --- | --- | --- | --- | --- | --- | --- | --- | --- | --- | --- | --- | --- | --- | --- | --- | --- | --- | --- | --- | --- | --- | --- | --- | --- | --- | --- | --- | --- | --- | --- | --- | --- | --- | --- | --- | --- | --- | --- | --- | --- | --- | --- | --- | --- | --- | --- | --- | --- | --- | --- | --- | --- | --- | --- | --- | --- | --- | --- | --- | --- | --- | --- | --- | --- | --- | --- | --- | --- | --- | --- | --- | --- | --- | --- | --- | --- | --- | --- | --- | --- | --- | --- | --- | --- | --- | --- | --- | --- | --- | --- | --- | --- | --- | --- | --- | --- | --- | --- | --- | --- | --- | --- | --- | --- | --- | --- | --- | --- | --- | --- | --- | --- | --- | --- | --- | --- | --- | --- | --- | --- | --- | --- | --- | --- | --- | --- | --- | --- | --- | --- | --- | --- | --- | --- | --- | --- | --- | --- | --- | --- | --- | --- | --- | --- | --- | --- | --- | --- | --- | --- | --- | --- | --- | --- | --- | --- | --- | --- | --- | --- | --- | --- | --- | --- | --- | --- | --- | --- | --- | --- | --- | --- | --- | --- | --- | --- | --- | --- | --- | --- | --- | --- | --- | --- | --- | --- | --- | --- | --- | --- | --- | --- | --- | --- | --- | --- | --- | --- | --- | --- | --- | --- | --- | --- | --- | --- | --- | --- | --- | --- | --- | --- | --- | --- | --- | --- | --- | --- | --- | --- | --- | --- | --- | --- | --- | --- | --- | --- | --- | --- | --- | --- | --- | --- | --- | --- | --- | --- | --- | --- | --- | --- | --- | --- | --- | --- | --- | --- | --- | --- | --- | --- | --- | --- | --- | --- | --- | --- | --- | --- | --- | --- | --- | --- | --- | --- | --- | --- | --- | --- | --- | --- | --- | --- | --- | --- | --- | --- | --- | --- | --- | --- | --- | --- | --- | --- | --- | --- | --- | --- | --- | --- | --- | --- | --- | --- | --- | --- | --- | --- | --- | --- | --- | --- | --- | --- | --- | --- | --- | --- | --- | --- | --- | --- | --- | --- | --- | --- | --- | --- | --- | --- | --- | --- | --- | --- | --- | --- | --- | --- | --- | --- | --- | --- | --- | --- | --- | --- | --- | --- | --- | --- | --- | --- | --- | --- | --- | --- | --- | --- | --- | --- | --- | --- | --- | --- | --- | --- | --- | --- | --- | --- | --- | --- | --- | --- | --- | --- | --- | --- | --- | --- | --- | --- | --- | --- | --- | --- | --- | --- | --- | --- | --- | --- | --- | --- | --- | --- | --- | --- | --- | --- | --- | --- | --- | --- | --- | --- | --- | --- | --- | --- | --- | --- | --- | --- | --- | --- | --- | --- | --- | --- | --- | --- | --- | --- | --- | --- | --- | --- | --- | --- | --- | --- | --- | --- | --- | --- | --- | --- | --- | --- | --- | --- | --- | --- | --- | --- | --- | --- | --- | --- | --- | --- | --- | --- | --- | --- | --- | --- | --- | --- | --- | --- | --- | --- | --- | --- | --- | --- | --- | --- | --- | --- | --- | --- | --- | --- | --- | --- | --- | --- | --- | --- | --- | --- | --- | --- | --- | --- | --- | --- | --- | --- | --- | --- | --- | --- | --- | --- | --- | --- | --- | --- | --- | --- | --- | --- | --- | --- | --- | --- | --- | --- | --- | --- | --- | --- | --- | --- | --- | --- | --- | --- | --- | --- | --- | --- | --- | --- | --- | --- | --- | --- | --- | --- | --- | --- | --- | --- | --- | --- | --- | --- | --- | --- | --- | --- | --- | --- | --- | --- | --- | --- | --- | --- | --- | --- | --- | --- | --- | --- | --- | --- | --- | --- | --- | --- | --- | --- | --- | --- | --- | --- | --- | --- | --- | --- | --- | --- | --- | --- | --- | --- | --- | --- | --- | --- | --- | --- | --- | --- | --- | --- | --- | --- | --- | --- | --- | --- | --- | --- | --- | --- | --- | --- | --- | --- | --- | --- | --- | --- | --- | --- | --- | --- | --- | --- | --- | --- | --- | --- | --- | --- | --- | --- | --- | --- | --- | --- | --- | --- | --- | --- | --- | --- | --- | --- | --- | --- | --- | --- | --- | --- | --- | --- | --- | --- | --- | --- | --- | --- | --- | --- | --- | --- | --- | --- | --- | --- | --- | --- | --- | --- | --- | --- | --- | --- | --- | --- | --- | --- | --- | --- | --- | --- | --- | --- | --- | --- | --- | --- | --- | --- | --- | --- | --- | --- | --- | --- | --- | --- | --- | --- | --- | --- | --- | --- | --- | --- | --- | --- | --- | --- | --- | --- | --- | --- | --- | --- | --- | --- | --- | --- | --- | --- | --- | --- | --- | --- | --- | --- | --- | --- | --- | --- | --- | --- | --- | --- | --- | --- | --- | --- | --- | --- | --- | --- | --- | --- | --- | --- | --- | --- | --- | --- | --- | --- | --- | --- | --- | --- | --- | --- | --- | --- | --- | --- | --- | --- | --- | --- | --- | --- | --- | --- | --- | --- | --- | --- | --- | --- | --- | --- | --- | --- | --- | --- | --- | --- | --- | --- | --- | --- | --- | --- | --- | --- | --- | --- | --- | --- | --- | --- | --- | --- | --- | --- | --- | --- | --- | --- | --- | --- | --- | --- | --- | --- | --- | --- | --- | --- | --- | --- | --- | --- | --- | --- | --- | --- | --- | --- | --- | --- | --- | --- | --- | --- | --- | --- | --- | --- | --- | --- | --- | --- | --- | --- | --- | --- | --- | --- | --- | --- | --- | --- | --- | --- | --- | --- | --- | --- | --- | --- | --- | --- | --- | --- | --- | --- | --- | --- | --- | --- | --- | --- | --- | --- | --- | --- | --- | --- | --- | --- | --- | --- | --- | --- | --- | --- | --- | --- | --- | --- | --- | --- | --- | --- | --- | --- | --- | --- | --- | --- | --- | --- | --- | --- | --- | --- | --- | --- | --- | --- | --- | --- | --- | --- | --- | --- | --- | --- | --- | --- | --- | --- | --- | --- | --- | --- | --- | --- | --- | --- | --- | --- | --- | --- | --- | --- | --- | --- | --- | --- | --- | --- | --- | --- | --- | --- | --- | --- | --- | --- | --- | --- | --- | --- | --- | --- | --- | --- | --- | --- | --- | --- | --- | --- | --- | --- | --- | --- | --- | --- | --- | --- | --- | --- | --- | --- | --- | --- | --- | --- | --- | --- | --- | --- | --- | --- | --- | --- | --- | --- | --- | --- | --- | --- | --- | --- | --- | --- | --- | --- | --- | --- | --- | --- | --- | --- | --- | --- | --- | --- | --- | --- | --- | --- | --- | --- | --- | --- | --- | --- | --- | --- | --- | --- | --- | --- | --- | --- | --- | --- | --- | --- | --- | --- | --- | --- | --- | --- | --- | --- | --- | --- | --- | --- | --- | --- | --- | --- | --- | --- | --- | --- | --- | --- | --- | --- | --- | --- | --- | --- | --- | --- | --- | --- | --- | --- | --- | --- | --- | --- | --- | --- | --- | --- | --- | --- | --- | --- | --- | --- | --- | --- | --- | --- | --- | --- | --- | --- | --- | --- | --- | --- | --- | --- | --- | --- | --- | --- | --- | --- | --- | --- | --- | --- | --- | --- | --- | --- | --- | --- | --- | --- | --- | --- | --- | --- | --- | --- | --- | --- | --- | --- | --- | --- | --- | --- | --- | --- | --- | --- | --- | --- | --- | --- | --- | --- | --- | --- | --- | --- | --- | --- | --- | --- | --- | --- | --- | --- | --- | --- | --- | --- | --- | --- | --- | --- | --- | --- | --- | --- | --- | --- | --- | --- | --- | --- | --- | --- | --- | --- | --- | --- | --- | --- | --- | --- | --- | --- | --- | --- | --- | --- | --- | --- | --- | --- | --- | --- | --- | --- | --- | --- | --- | --- | --- | --- | --- | --- | --- | --- | --- | --- | --- | --- | --- | --- | --- | --- | --- | --- | --- | --- | --- | --- | --- | --- | --- | --- | --- | --- | --- | --- | --- | --- | --- | --- | --- | --- | --- | --- | --- | --- | --- | --- | --- | --- | --- | --- | --- | --- | --- | --- | --- | --- | --- | --- | --- | --- | --- | --- | --- | --- | --- | --- | --- | --- | --- | --- | --- | --- | --- | --- | --- | --- | --- | --- | --- | --- | --- | --- | --- | --- | --- | --- | --- | --- | --- | --- | --- | --- | --- | --- | --- | --- | --- | --- | --- | --- | --- | --- | --- | --- | --- | --- | --- | --- | --- | --- | --- | --- | --- | --- | --- | --- | --- | --- | --- | --- | --- | --- | --- | --- | --- | --- | --- | --- | --- | --- | --- | --- | --- | --- | --- | --- | --- | --- | --- | --- | --- | --- | --- | --- | --- | --- | --- | --- | --- | --- | --- | --- | --- | --- | --- | --- | --- | --- | --- | --- | --- | --- | --- | --- | --- | --- | --- | --- | --- | --- | --- | --- | --- | --- | --- | --- | --- | --- | --- | --- | --- | --- | --- | --- | --- | --- | --- | --- | --- | --- | --- | --- | --- | --- | --- | --- | --- | --- | --- | --- | --- | --- | --- | --- | --- | --- | --- | --- | --- | --- | --- | --- | --- | --- | --- | --- | --- | --- | --- | --- | --- | --- | --- | --- | --- | --- | --- | --- | --- | --- | --- | --- | --- | --- | --- | --- | --- | --- | --- | --- | --- | --- | --- | --- | --- | --- | --- | --- | --- | --- | --- | --- | --- | --- | --- | --- | --- | --- | --- | --- | --- | --- | --- | --- | --- | --- | --- | --- | --- | --- | --- | --- | --- | --- | --- | --- | --- | --- | --- | --- | --- | --- | --- | --- | --- | --- | --- | --- | --- | --- | --- | --- | --- | --- | --- | --- | --- | --- | --- | --- | --- | --- | --- | --- | --- | --- | --- | --- | --- | --- | --- | --- | --- | --- |
| |  |  |  |  |  |  |  |  |  |  |  |  |  |  |  |  |  |  |  |  |  |  |  |  |  |  |  |  |  |  |  |  |  |  |  |  |  |  |  |  |  |  |  |  |  |  |  |  |  |  |  |  |  |  |  |  |  |  | | --- | --- | --- | --- | --- | --- | --- | --- | --- | --- | --- | --- | --- | --- | --- | --- | --- | --- | --- | --- | --- | --- | --- | --- | --- | --- | --- | --- | --- | --- | --- | --- | --- | --- | --- | --- | --- | --- | --- | --- | --- | --- | --- | --- | --- | --- | --- | --- | --- | --- | --- | --- | --- | --- | --- | --- | --- | --- | | G0VCN0/1-398 | 1 | - | M | S | N | N | V | Y | I | V | S | A | K | R | T | P | I | G | S | F | Q | G | S | L | A | S | K | T | A | I | D | L | G | A | I | A | L | K | G | A | M | E | Q | V | P | Q | L | N | P | D | S | D | Y | D | E | I | 54 | | Q6CR40/1-398 | 1 | - | M | S | D | N | V | Y | I | V | A | A | A | R | T | P | I | G | S | F | Q | G | S | L | S | S | K | N | C | V | D | L | G | S | A | A | V | K | G | A | L | A | Q | V | P | E | I | D | P | S | - | T | V | E | E | I | 53 | | Q6FKD8/1-398 | 1 | - | M | S | D | T | V | Y | I | V | S | A | A | R | T | P | I | G | S | F | Q | G | A | L | A | S | K | T | A | V | D | L | G | V | I | A | V | K | G | A | M | Q | K | V | P | Q | L | N | P | E | S | D | Y | D | E | V | 54 | | Q759V7/1-397 | 1 | - | M | S | E | N | V | Y | I | V | A | A | A | R | T | P | I | G | S | F | Q | G | S | L | A | S | Q | T | Y | V | D | L | G | A | H | A | V | K | A | A | L | S | Q | V | P | Q | I | D | A | S | - | Q | V | D | E | I | 53 | | A7TRT7/1-397 | 1 | - | M | S | G | K | V | Y | V | V | S | A | V | R | T | P | L | G | S | F | Q | G | G | L | S | P | K | T | A | V | E | L | G | A | T | A | V | K | A | A | A | E | R | V | P | Q | L | N | V | E | K | D | F | D | E | I | 54 | | C5DIB3/1-396 | 1 | - | M | S | D | K | V | Y | I | V | A | A | A | R | T | P | I | G | C | F | L | G | G | L | T | S | K | T | Y | V | D | L | G | S | T | A | V | A | G | A | L | A | Q | V | P | E | I | K | P | N | - | D | V | E | E | I | 53 | | C5DPE6/1-398 | 1 | - | M | S | D | N | V | Y | V | V | A | S | C | R | T | P | V | G | S | F | Q | G | S | L | A | S | K | T | A | I | E | L | G | T | A | A | V | K | G | A | I | S | K | V | P | Q | L | K | P | E | T | D | Y | D | E | I | 54 | | Kwal\_27.11783/1-397 | 1 | M | S | S | D | K | V | Y | I | V | A | A | A | R | T | P | I | G | C | F | Q | G | G | L | A | S | K | T | Y | V | D | L | G | S | A | A | V | V | G | A | L | S | Q | V | P | S | V | K | P | T | - | D | V | E | E | I | 54 | | Sbay\_551.6/1-398 | 1 | - | M | S | Q | N | V | Y | I | V | S | T | A | R | T | P | I | G | S | F | Q | G | S | L | S | S | K | T | A | I | E | L | G | A | A | A | L | K | G | A | L | A | K | V | P | E | L | D | A | S | K | D | F | D | E | I | 54 | | SAKL0H11704g/1-399 | 1 | M | S | S | E | N | V | Y | I | V | A | A | A | R | T | P | I | G | S | F | Q | G | S | L | S | S | K | T | Y | V | D | L | G | S | A | A | V | K | G | A | L | S | Q | V | P | Q | L | N | P | S | K | D | V | E | E | I | 55 | | P41338/1-398 | 1 | - | M | S | Q | N | V | Y | I | V | S | T | A | R | T | P | I | G | S | F | Q | G | S | L | S | S | K | T | A | V | E | L | G | A | V | A | L | K | G | A | L | A | K | V | P | E | L | D | A | S | K | D | F | D | E | I | 54 | |  | | G0VCN0/1-398 | 55 | I | F | G | N | V | L | S | A | N | L | G | Q | A | P | A | R | Q | V | A | L | A | A | G | L | N | N | S | I | V | A | T | T | V | N | K | V | C | A | S | A | M | K | S | I | I | L | A | A | Q | T | I | K | C | G | N | 109 | | Q6CR40/1-398 | 54 | I | F | G | N | V | I | S | A | N | V | G | Q | A | P | A | R | Q | V | A | L | A | A | G | L | G | K | H | I | V | A | S | T | V | N | K | V | C | A | S | G | M | K | A | I | I | L | G | A | Q | A | I | K | T | G | S | 108 | | Q6FKD8/1-398 | 55 | F | F | G | N | V | I | S | A | N | L | G | Q | N | A | A | R | Q | V | A | L | G | A | G | L | K | N | G | I | V | A | T | S | V | N | K | V | C | A | S | A | M | K | S | I | I | L | G | A | Q | A | I | K | C | G | T | 109 | | Q759V7/1-397 | 54 | I | F | G | N | V | L | S | A | N | V | G | Q | A | P | A | R | Q | V | A | L | A | A | G | L | P | K | S | I | V | A | T | T | V | N | K | V | C | A | S | G | M | K | A | L | I | L | A | A | Q | A | I | K | C | G | T | 108 | | A7TRT7/1-397 | 55 | I | F | G | N | V | M | T | A | N | N | G | Q | N | P | A | R | Q | V | A | L | T | A | G | L | D | K | H | I | V | A | T | T | V | N | K | V | C | A | S | G | M | K | A | I | I | L | G | S | Q | T | I | L | C | G | N | 109 | | C5DIB3/1-396 | 54 | F | F | G | N | V | L | S | A | N | V | G | Q | A | P | A | R | Q | V | A | L | A | S | G | L | G | K | H | I | V | A | T | T | V | N | K | V | C | A | S | G | M | K | A | I | I | C | G | A | Q | T | I | K | C | G | N | 108 | | C5DPE6/1-398 | 55 | I | F | G | N | V | L | S | A | N | S | G | Q | A | P | A | R | Q | V | A | L | G | A | G | L | H | N | H | I | V | D | T | T | V | N | K | V | C | A | S | A | L | R | S | I | I | L | G | A | Q | S | I | K | C | G | T | 109 | | Kwal\_27.11783/1-397 | 55 | F | F | G | N | V | L | S | A | N | V | G | Q | A | P | A | R | Q | V | A | L | G | A | G | L | G | K | H | I | V | A | T | T | V | N | K | V | C | A | S | G | M | K | A | I | I | C | A | A | Q | T | I | K | C | G | N | 109 | | Sbay\_551.6/1-398 | 55 | I | F | G | N | V | L | S | A | N | L | G | Q | A | P | A | R | Q | V | A | L | A | A | G | L | G | N | H | I | V | A | S | T | V | N | K | V | C | A | S | A | M | K | A | I | I | L | G | A | Q | S | I | K | C | G | N | 109 | | SAKL0H11704g/1-399 | 56 | F | F | G | N | V | L | S | A | N | V | G | Q | A | P | A | R | Q | V | A | L | A | S | G | L | G | K | H | I | V | A | T | T | V | N | K | V | C | A | S | G | M | K | A | I | I | C | G | A | Q | T | I | K | C | G | N | 110 | | P41338/1-398 | 55 | I | F | G | N | V | L | S | A | N | L | G | Q | A | P | A | R | Q | V | A | L | A | A | G | L | S | N | H | I | V | A | S | T | V | N | K | V | C | A | S | A | M | K | A | I | I | L | G | A | Q | S | I | K | C | G | N | 109 | |  | | G0VCN0/1-398 | 110 | A | D | V | V | I | A | G | G | C | E | S | M | T | N | T | P | Y | Y | M | P | A | A | R | S | G | A | R | F | G | E | T | T | L | V | D | G | V | Q | R | D | G | L | N | D | A | Y | D | G | L | A | M | G | V | H | A | 164 | | Q6CR40/1-398 | 109 | A | D | I | I | V | A | G | G | A | E | S | M | S | N | A | P | Y | Y | L | S | S | H | R | S | G | A | R | F | G | E | S | K | V | I | D | G | I | Q | R | D | G | L | N | D | A | Y | D | G | Q | A | M | G | V | H | A | 163 | | Q6FKD8/1-398 | 110 | A | D | V | V | I | A | G | G | C | E | S | M | T | N | A | P | Y | Y | M | P | A | A | R | A | G | A | R | F | G | E | T | K | M | V | D | G | I | Q | R | D | G | L | N | D | A | Y | D | H | Q | P | M | G | V | H | A | 164 | | Q759V7/1-397 | 109 | A | D | I | V | V | A | G | G | A | E | S | M | T | N | T | P | Y | Y | M | P | A | A | R | G | G | A | R | F | G | E | A | K | L | I | D | G | I | Q | R | D | G | L | N | D | A | Y | D | H | Q | A | M | G | V | H | A | 163 | | A7TRT7/1-397 | 110 | A | D | V | V | V | V | G | G | T | E | S | M | T | N | V | P | Y | Y | V | P | S | A | R | G | G | A | R | F | G | E | T | K | M | I | D | G | I | Q | R | D | G | I | N | D | A | Y | D | G | L | A | M | G | V | H | A | 164 | | C5DIB3/1-396 | 109 | A | D | I | V | V | A | G | G | A | E | S | M | T | N | A | P | Y | Y | M | P | A | A | R | G | G | A | K | F | G | P | S | T | L | V | D | G | I | Q | R | D | G | L | N | D | A | Y | D | N | Q | A | M | G | V | H | A | 163 | | C5DPE6/1-398 | 110 | S | D | V | V | V | A | G | G | C | E | S | M | T | N | T | P | Y | Y | M | P | A | A | R | S | G | A | R | F | G | E | T | K | L | V | D | G | I | Q | R | D | G | L | N | D | A | Y | D | G | L | A | M | G | V | H | A | 164 | | Kwal\_27.11783/1-397 | 110 | A | D | I | V | V | A | G | G | A | E | S | M | T | N | T | P | Y | Y | M | P | A | A | R | G | G | A | K | F | G | P | T | T | L | V | D | G | V | Q | R | D | G | L | N | D | A | Y | D | N | Q | A | M | G | V | H | A | 164 | | Sbay\_551.6/1-398 | 110 | A | D | V | V | V | A | G | G | C | E | S | M | T | N | A | P | Y | Y | M | P | A | A | R | G | G | A | K | F | G | Q | T | V | L | I | D | G | V | E | R | D | G | L | N | D | A | Y | D | G | L | A | M | G | V | H | A | 164 | | SAKL0H11704g/1-399 | 111 | A | D | I | V | V | A | G | G | A | E | S | M | T | N | A | P | Y | Y | L | P | S | A | R | S | G | A | R | F | G | E | T | K | L | I | D | G | I | Q | R | D | G | L | N | D | A | Y | D | G | Q | A | M | G | V | H | A | 165 | | P41338/1-398 | 110 | A | D | V | V | V | A | G | G | C | E | S | M | T | N | A | P | Y | Y | M | P | A | A | R | A | G | A | K | F | G | Q | T | V | L | V | D | G | V | E | R | D | G | L | N | D | A | Y | D | G | L | A | M | G | V | H | A | 164 | |  | | G0VCN0/1-398 | 165 | E | K | C | A | K | D | H | N | V | S | R | E | E | Q | D | Q | F | A | I | D | S | Y | Q | K | A | Q | A | A | Q | N | D | G | K | F | S | S | E | I | V | P | V | T | I | K | G | F | R | G | K | P | D | T | E | V | T | 219 | | Q6CR40/1-398 | 164 | E | K | C | A | S | D | Y | D | F | S | R | E | E | Q | D | D | F | A | I | Q | S | Y | Q | K | A | Q | L | A | Q | S | Q | G | K | F | D | K | E | I | V | P | I | T | I | K | G | F | R | G | K | P | D | T | Q | V | T | 218 | | Q6FKD8/1-398 | 165 | E | K | C | A | R | N | W | E | I | T | R | E | Q | Q | D | D | F | A | I | A | S | Y | Q | K | A | Q | K | A | Q | Q | E | G | K | F | D | N | E | I | V | P | V | T | I | K | G | F | R | G | K | P | D | T | Q | V | T | 219 | | Q759V7/1-397 | 164 | E | K | C | A | S | D | H | S | I | T | R | E | E | Q | D | N | F | A | I | E | S | Y | Q | K | A | Q | K | A | H | A | E | G | K | F | A | A | E | I | A | P | V | T | I | K | G | V | R | G | K | P | D | V | T | V | S | 218 | | A7TRT7/1-397 | 165 | E | K | C | A | R | D | W | E | I | T | R | D | E | Q | D | S | F | A | I | A | S | Y | Q | K | A | Q | K | A | Q | N | D | G | K | F | D | N | E | I | V | P | V | T | I | K | G | F | R | G | K | P | D | T | Q | I | T | 219 | | C5DIB3/1-396 | 164 | E | K | C | A | R | D | Y | Q | I | T | R | E | Q | Q | D | E | Y | A | I | G | S | Y | Q | K | A | Q | K | A | Q | A | E | G | K | F | D | R | E | I | V | P | V | T | I | K | G | F | R | G | K | P | D | T | Y | V | Q | 218 | | C5DPE6/1-398 | 165 | E | K | C | A | R | D | W | D | C | S | R | Q | D | Q | D | E | F | A | I | S | S | Y | Q | K | A | Q | K | A | Q | N | E | G | K | F | D | K | E | I | V | P | I | T | I | K | G | F | R | G | K | P | D | T | Q | V | T | 219 | | Kwal\_27.11783/1-397 | 165 | E | K | C | A | R | D | Y | E | V | T | R | E | Q | Q | D | D | Y | A | I | A | S | Y | Q | K | A | Q | K | A | Q | A | E | G | K | F | D | R | E | I | V | P | L | T | I | K | G | F | R | G | K | P | D | T | V | V | Q | 219 | | Sbay\_551.6/1-398 | 165 | E | K | C | A | R | D | W | D | I | T | R | E | Q | Q | D | T | F | A | I | E | S | Y | Q | K | S | Q | K | S | Q | K | E | G | K | F | N | S | E | I | V | P | V | T | I | K | G | F | R | G | K | P | D | T | Q | V | T | 219 | | SAKL0H11704g/1-399 | 166 | E | K | C | A | R | D | Y | Q | F | T | R | D | D | Q | D | N | F | A | I | N | S | Y | Q | K | A | V | K | A | Q | T | E | G | K | F | E | R | E | I | V | P | V | T | I | K | G | F | R | G | K | P | D | T | Q | V | T | 220 | | P41338/1-398 | 165 | E | K | C | A | R | D | W | D | I | T | R | E | Q | Q | D | N | F | A | I | E | S | Y | Q | K | S | Q | K | S | Q | K | E | G | K | F | D | N | E | I | V | P | V | T | I | K | G | F | R | G | K | P | D | T | Q | V | T | 219 | |  | | G0VCN0/1-398 | 220 | K | D | E | E | P | S | K | L | N | V | E | K | L | R | A | A | R | T | V | F | Q | R | E | - | N | G | T | V | T | A | P | N | A | S | P | I | N | D | G | A | A | A | V | I | L | V | S | E | R | K | L | Q | E | L | K | 273 | | Q6CR40/1-398 | 219 | K | D | E | E | P | S | K | L | N | V | E | K | L | R | S | A | R | A | V | F | A | A | N | G | K | G | T | V | T | A | P | N | A | S | P | I | N | D | G | G | A | A | V | I | L | V | S | E | K | K | L | K | E | L | N | 273 | | Q6FKD8/1-398 | 220 | K | D | E | E | P | T | K | L | N | V | D | K | L | R | S | A | R | T | V | F | Q | K | E | - | N | G | T | V | T | A | P | N | A | S | P | I | N | D | G | A | A | A | V | I | L | V | S | E | K | K | L | K | E | L | N | 273 | | Q759V7/1-397 | 219 | Q | D | E | E | T | T | K | F | N | A | E | K | L | K | A | A | R | P | V | F | K | K | E | - | N | G | T | V | T | A | P | N | A | S | P | I | N | D | G | G | A | A | I | I | L | V | S | E | R | K | L | K | E | L | N | 272 | | A7TRT7/1-397 | 220 | K | D | E | E | P | A | N | L | K | V | D | K | L | R | S | A | R | T | V | F | Q | K | E | - | N | G | T | V | T | A | P | N | A | S | P | L | N | D | G | A | A | A | L | I | L | V | S | E | R | K | L | K | E | L | N | 273 | | C5DIB3/1-396 | 219 | K | D | E | E | P | S | K | L | N | V | E | K | L | K | S | A | R | T | V | F | Q | K | E | - | N | G | T | V | T | A | P | N | A | S | P | I | N | D | G | G | A | A | V | V | L | V | S | E | R | K | L | K | E | L | G | 272 | | C5DPE6/1-398 | 220 | K | D | E | E | P | S | K | L | N | V | E | K | L | K | S | A | R | T | V | F | Q | K | E | - | N | G | T | V | T | A | P | N | A | S | P | L | N | D | G | A | A | A | V | I | L | V | S | E | K | K | L | K | Q | L | G | 273 | | Kwal\_27.11783/1-397 | 220 | K | D | E | E | P | A | K | L | N | V | E | R | L | R | S | A | R | T | V | F | Q | K | E | - | N | G | T | V | T | A | P | N | A | S | P | I | N | D | G | G | A | A | V | V | L | V | S | E | R | K | L | Q | E | L | G | 273 | | Sbay\_551.6/1-398 | 220 | N | D | E | E | P | A | R | L | H | V | E | K | L | K | S | A | R | T | V | F | Q | R | E | - | N | G | T | V | T | A | A | N | A | S | P | I | N | D | G | A | A | A | I | I | L | V | S | E | R | V | L | K | E | K | N | 273 | | SAKL0H11704g/1-399 | 221 | K | D | E | E | P | P | K | L | N | V | E | K | L | K | S | A | R | A | V | F | Q | R | E | - | N | G | T | V | T | A | P | N | A | S | P | I | N | D | G | G | A | A | V | V | L | V | S | E | R | K | L | R | E | L | N | 274 | | P41338/1-398 | 220 | K | D | E | E | P | A | R | L | H | V | E | K | L | R | S | A | R | T | V | F | Q | K | E | - | N | G | T | V | T | A | A | N | A | S | P | I | N | D | G | A | A | A | V | I | L | V | S | E | K | V | L | K | E | K | N | 273 | |  | | G0VCN0/1-398 | 274 | L | T | P | L | A | V | I | R | G | W | G | E | A | A | Q | H | P | A | D | F | T | W | S | P | S | L | A | V | P | K | A | L | K | H | A | G | I | S | D | I | Q | S | V | D | F | F | E | F | N | E | A | F | S | V | V | 328 | | Q6CR40/1-398 | 274 | L | K | P | L | A | L | I | K | G | W | G | E | A | A | H | E | P | A | D | F | T | W | A | P | S | L | A | V | P | K | A | L | K | H | A | G | V | P | D | I | S | S | V | D | F | V | E | L | N | E | A | F | S | V | V | 328 | | Q6FKD8/1-398 | 274 | L | T | P | L | A | T | L | K | G | W | G | E | A | A | H | D | P | A | D | F | T | W | A | P | S | L | A | I | P | K | A | L | K | H | A | G | V | A | D | I | S | Q | V | D | Y | I | E | L | N | E | A | F | S | V | V | 328 | | Q759V7/1-397 | 273 | L | R | P | S | A | L | I | K | G | W | G | E | A | A | H | E | P | A | D | F | T | W | A | P | S | L | A | I | P | K | A | L | K | H | A | G | I | Q | D | I | N | E | V | D | F | V | E | L | N | E | A | F | S | V | V | 327 | | A7TRT7/1-397 | 274 | L | K | P | L | A | T | I | K | G | W | G | E | A | A | H | E | P | A | D | F | T | W | A | P | S | L | A | V | P | K | A | L | T | H | A | G | I | A | Q | - | D | D | V | D | F | F | E | F | N | E | A | F | S | V | V | 327 | | C5DIB3/1-396 | 273 | L | K | P | L | A | L | I | K | G | W | G | E | A | A | H | E | P | E | D | F | T | W | A | P | S | L | A | I | P | K | A | I | K | H | A | G | V | Q | - | L | D | E | I | D | Y | F | E | F | N | E | A | F | S | V | V | 326 | | C5DPE6/1-398 | 274 | L | K | P | I | A | V | I | R | G | W | G | E | A | A | H | E | P | A | D | F | T | W | A | P | S | L | A | V | P | K | A | L | T | H | A | G | I | K | D | I | G | Q | I | D | F | F | E | F | N | E | A | F | S | V | V | 328 | | Kwal\_27.11783/1-397 | 274 | L | K | P | L | A | V | I | K | G | W | G | E | A | A | H | E | P | A | D | F | T | W | A | P | S | L | A | I | P | K | A | V | K | H | A | G | V | Q | - | L | E | D | I | D | F | F | E | F | N | E | A | F | S | V | V | 327 | | Sbay\_551.6/1-398 | 274 | L | K | P | L | A | L | I | K | G | W | G | E | A | A | H | L | P | A | D | F | T | W | A | P | S | L | A | V | P | K | A | L | K | H | A | G | I | E | D | I | N | S | V | D | Y | F | E | F | N | E | A | F | S | V | V | 328 | | SAKL0H11704g/1-399 | 275 | L | T | P | L | A | I | I | K | G | W | G | E | A | A | H | E | P | A | D | F | T | W | A | P | S | L | A | I | P | K | A | L | K | H | A | G | V | E | D | I | N | Q | V | D | F | F | E | L | N | E | A | F | S | V | V | 329 | | P41338/1-398 | 274 | L | K | P | L | A | I | I | K | G | W | G | E | A | A | H | Q | P | A | D | F | T | W | A | P | S | L | A | V | P | K | A | L | K | H | A | G | I | E | D | I | N | S | V | D | Y | F | E | F | N | E | A | F | S | V | V | 328 | |  | | G0VCN0/1-398 | 329 | G | I | A | N | T | K | I | L | K | L | N | P | A | K | V | N | V | Y | G | G | A | V | A | I | G | H | P | L | G | C | S | G | A | R | I | V | V | T | L | L | S | I | L | R | Q | E | K | G | K | L | G | V | A | A | I | 383 | | Q6CR40/1-398 | 329 | G | L | V | N | T | K | K | L | G | I | D | A | A | K | V | N | V | Y | G | G | A | V | A | L | G | H | P | L | G | C | S | G | A | R | V | V | V | T | L | A | N | I | L | N | Q | E | N | G | K | I | G | V | A | G | I | 383 | | Q6FKD8/1-398 | 329 | G | C | A | N | T | K | I | L | K | V | D | P | A | K | V | N | V | Y | G | G | A | V | A | I | G | H | P | L | G | C | S | G | A | R | V | V | V | T | L | L | S | V | L | Q | Q | E | G | G | K | I | G | V | A | G | I | 383 | | Q759V7/1-397 | 328 | G | L | A | N | T | K | L | L | G | L | D | P | S | K | V | N | V | Y | G | G | A | V | A | L | G | H | P | L | G | C | S | G | A | R | I | I | V | T | L | L | S | I | L | Q | Q | E | G | G | K | V | G | V | A | G | I | 382 | | A7TRT7/1-397 | 328 | G | V | A | N | P | K | I | L | K | I | N | Q | D | K | V | N | V | Y | G | G | A | V | A | L | G | H | P | L | G | C | S | G | A | R | V | I | V | T | L | I | S | V | L | H | Q | E | N | G | K | L | G | V | G | A | I | 382 | | C5DIB3/1-396 | 327 | G | I | A | N | P | K | I | L | G | I | P | L | E | K | V | N | P | Y | G | G | A | V | A | I | G | H | P | L | G | C | S | G | A | R | I | V | V | T | L | I | S | V | L | T | Q | E | N | A | K | L | G | A | A | G | I | 381 | | C5DPE6/1-398 | 329 | G | L | A | N | T | K | I | L | K | I | D | P | S | K | V | N | V | Y | G | G | A | V | A | L | G | H | P | L | G | C | S | G | A | R | I | V | V | T | L | L | S | V | L | E | Q | E | N | G | K | L | G | A | A | A | I | 383 | | Kwal\_27.11783/1-397 | 328 | G | I | A | N | P | K | I | L | G | I | P | L | E | K | V | N | P | Y | G | G | A | V | A | L | G | H | P | L | G | C | S | G | A | R | I | V | V | T | L | I | S | V | L | T | Q | E | K | A | N | L | G | A | A | A | I | 382 | | Sbay\_551.6/1-398 | 329 | G | L | V | N | T | K | I | L | K | L | D | P | S | K | V | N | V | Y | G | G | A | V | A | L | G | H | P | L | G | C | S | G | A | R | V | V | V | T | L | L | S | I | L | Q | Q | E | G | G | K | I | G | V | A | A | I | 383 | | SAKL0H11704g/1-399 | 330 | G | L | A | N | T | K | I | L | N | I | A | P | E | K | V | N | V | Y | G | G | A | V | A | I | G | H | P | L | G | C | S | G | A | R | I | I | V | T | L | A | T | V | L | Q | Q | E | G | G | K | L | G | V | A | G | I | 384 | | P41338/1-398 | 329 | G | L | V | N | T | K | I | L | K | L | D | P | S | K | V | N | V | Y | G | G | A | V | A | L | G | H | P | L | G | C | S | G | A | R | V | V | V | T | L | L | S | I | L | Q | Q | E | G | G | K | I | G | V | A | A | I | 383 | |  | | G0VCN0/1-398 | 384 | C | N | G | G | G | G | A | S | S | I | V | I | E | R | L |  | | | | | | | | | | | | | | | | | | | | | | | | | | | | | | | | | | | | | | | | 398 | | Q6CR40/1-398 | 384 | C | N | G | G | G | G | A | S | S | I | V | L | E | R | V |  | | | | | | | | | | | | | | | | | | | | | | | | | | | | | | | | | | | | | | | | 398 | | Q6FKD8/1-398 | 384 | C | N | G | G | G | G | A | S | S | V | V | I | E | R | V |  | | | | | | | | | | | | | | | | | | | | | | | | | | | | | | | | | | | | | | | | 398 | | Q759V7/1-397 | 383 | C | N | G | G | G | G | A | S | S | V | V | L | A | K | L |  | | | | | | | | | | | | | | | | | | | | | | | | | | | | | | | | | | | | | | | | 397 | | A7TRT7/1-397 | 383 | C | N | G | G | G | G | A | S | S | V | V | I | E | K | A |  | | | | | | | | | | | | | | | | | | | | | | | | | | | | | | | | | | | | | | | | 397 | | C5DIB3/1-396 | 382 | C | N | G | G | G | G | A | S | S | I | I | I | E | R | V |  | | | | | | | | | | | | | | | | | | | | | | | | | | | | | | | | | | | | | | | | 396 | | C5DPE6/1-398 | 384 | C | N | G | G | G | G | A | S | S | I | V | L | E | K | L |  | | | | | | | | | | | | | | | | | | | | | | | | | | | | | | | | | | | | | | | | 398 | | Kwal\_27.11783/1-397 | 383 | C | N | G | G | G | G | A | S | S | I | I | I | E | R | A |  | | | | | | | | | | | | | | | | | | | | | | | | | | | | | | | | | | | | | | | | 397 | | Sbay\_551.6/1-398 | 384 | C | N | G | G | G | G | A | S | S | I | V | I | E | K | L |  | | | | | | | | | | | | | | | | | | | | | | | | | | | | | | | | | | | | | | | | 398 | | SAKL0H11704g/1-399 | 385 | C | N | G | G | G | G | A | S | S | I | V | L | E | R | V |  | | | | | | | | | | | | | | | | | | | | | | | | | | | | | | | | | | | | | | | | 399 | | P41338/1-398 | 384 | C | N | G | G | G | G | A | S | S | I | V | I | E | K | I |  | | | | | | | | | | | | | | | | | | | | | | | | | | | | | | | | | | | | | | | | 398 | |
