# Supplementary material for: Plus ça change – evolutionary sequence divergence predicts protein subcellular localization signals
Source: BMC Genomics. 2014 Jan 20;15:46. doi: 10.1186/1471-2164-15-46 (PMC3906766; doi:10.1186/1471-2164-15-46)
Supplement: Additional file 2 — MSA’s of proteins for which sequence divergence changes predicted localization signals. Contains links to ortholog multiple sequence alignments of each protein in Additional file 3: Table S1. [file 1471-2164-15-46-S2.zip › P41805.html]

|  |  |  |  |  |  |  |  |  |  |  |  |  |  |  |  |  |  |  |  |  |  |  |  |  |  |  |  |  |  |  |  |  |  |  |  |  |  |  |  |  |  |  |  |  |  |  |  |  |  |  |  |  |  |  |  |  |  |  |  |  |  |  |  |  |  |  |  |  |  |  |  |  |  |  |  |  |  |  |  |  |  |  |  |  |  |  |  |  |  |  |  |  |  |  |  |  |  |  |  |  |  |  |  |  |  |  |  |  |  |  |  |  |  |  |  |  |  |  |  |  |  |  |  |  |  |  |  |  |  |  |  |  |  |  |  |  |  |  |  |  |  |  |  |  |  |  |  |  |  |  |  |  |  |  |  |  |  |  |  |  |  |  |  |  |  |  |  |  |  |  |  |  |  |  |  |  |  |  |  |  |  |  |  |  |  |  |  |  |  |  |  |  |  |  |  |  |  |  |  |  |  |  |  |  |  |  |  |  |  |  |  |  |  |  |  |  |  |  |  |  |  |  |  |  |  |  |  |  |  |  |  |  |  |  |  |  |  |  |  |  |  |  |  |  |  |  |  |  |  |  |  |  |  |  |  |  |  |  |  |  |  |  |  |  |  |  |  |  |  |  |  |  |  |  |  |  |  |  |  |  |  |  |  |  |  |  |  |  |  |  |  |  |  |  |  |  |  |  |  |  |  |  |  |  |  |  |  |  |  |  |  |  |  |  |  |  |  |  |  |  |  |  |  |  |  |  |  |  |  |  |  |  |  |  |  |  |  |  |  |  |  |  |  |  |  |  |  |  |  |  |  |  |  |  |  |  |  |  |  |  |  |  |  |  |  |  |  |  |  |  |  |  |  |  |  |  |  |  |  |  |  |  |  |  |  |  |  |  |  |  |  |  |  |  |  |  |  |  |  |  |  |  |  |  |  |  |  |  |  |  |  |  |  |  |  |  |  |  |  |  |  |  |  |  |  |  |  |  |  |  |  |  |  |  |  |  |  |  |  |  |  |  |  |  |  |  |  |  |  |  |  |  |  |  |  |  |  |  |  |  |  |  |  |  |  |  |  |  |  |  |  |  |  |  |  |  |  |  |  |  |  |  |  |  |  |  |  |  |  |  |  |  |  |  |  |  |  |  |  |  |  |  |  |  |  |  |  |  |  |  |  |  |  |  |  |  |  |  |  |  |  |  |  |  |  |  |  |  |  |  |  |  |  |  |  |  |  |  |  |  |  |  |  |  |  |  |  |  |  |  |  |  |  |  |  |  |  |  |  |  |  |  |  |  |  |  |  |  |  |  |  |  |  |  |  |  |  |  |  |  |  |  |  |  |  |  |  |  |  |  |  |  |  |  |  |  |  |  |  |  |  |  |  |  |  |  |  |  |  |  |  |  |  |  |  |  |  |  |  |  |  |  |  |  |  |  |  |  |  |  |  |  |  |  |  |  |  |  |  |  |  |  |  |  |  |  |  |  |  |  |  |  |  |  |  |  |  |  |  |  |  |  |  |  |  |  |  |  |  |  |  |  |  |  |  |  |  |  |  |  |  |  |  |  |  |  |  |  |  |  |  |  |  |  |  |  |  |  |  |  |  |  |  |  |  |  |  |  |  |  |  |  |  |  |  |  |  |  |  |  |  |  |  |  |  |  |  |  |  |  |  |  |  |  |  |  |  |  |  |  |  |  |  |  |  |  |  |  |  |  |  |  |  |  |  |  |  |  |  |  |  |  |  |  |  |  |  |  |  |  |  |  |  |  |  |  |  |  |  |  |  |  |  |  |  |  |  |  |  |  |  |  |  |  |  |  |  |  |  |  |  |  |  |  |  |  |  |  |  |  |  |  |  |  |  |  |  |  |  |  |  |  |  |  |  |  |  |  |  |  |  |  |  |  |  |  |  |  |  |  |  |  |  |  |  |  |  |  |  |  |  |  |  |  |  |  |  |  |  |  |  |  |  |  |  |  |  |  |  |  |  |  |  |  |  |  |  |  |  |  |  |  |  |  |  |  |  |  |  |  |  |  |  |  |  |  |  |  |  |  |  |  |  |  |  |  |  |  |  |  |  |  |  |  |  |  |  |  |  |  |  |  |  |  |  |  |  |  |  |  |  |  |  |  |  |  |  |  |  |  |  |  |  |  |  |  |  |  |  |  |  |  |  |  |  |  |  |  |  |  |  |  |  |  |  |  |  |  |  |  |  |  |  |  |  |  |  |  |  |  |  |  |  |  |  |  |  |  |  |  |  |  |  |  |  |  |  |  |  |  |  |  |  |  |  |  |  |  |  |  |  |  |  |  |  |  |  |  |  |  |  |  |  |  |  |  |  |  |  |  |  |  |  |  |  |  |  |  |  |  |  |  |  |  |  |  |  |  |  |  |  |  |  |  |  |  |  |  |  |  |  |  |  |  |  |  |  |  |  |  |  |  |  |  |  |  |  |  |  |  |  |  |  |  |  |  |  |  |  |  |  |  |  |  |  |  |  |  |  |  |  |  |  |  |  |  |  |  |  |  |  |  |  |  |  |  |  |  |  |  |  |  |  |  |  |  |  |  |  |  |  |  |  |  |  |  |  |  |  |  |  |  |  |  |  |  |  |  |  |  |  |  |  |  |  |  |  |  |  |  |  |  |  |  |  |  |  |  |  |  |  |  |  |  |  |  |  |  |  |  |  |  |  |  |  |  |  |  |  |  |  |  |  |  |  |  |  |  |  |  |  |  |  |  |  |  |  |  |  |  |  |  |  |  |  |  |  |  |  |  |  |  |  |  |  |  |  |  |  |  |  |  |  |  |  |  |  |  |  |  |  |  |  |  |  |  |  |  |  |  |  |  |  |  |  |  |  |  |  |  |  |  |  |  |  |  |  |  |  |  |  |  |  |  |  |  |  |  |  |  |  |  |  |  |  |  |  |  |  |  |  |  |  |  |  |  |  |  |  |  |  |  |  |  |  |  |  |  |  |  |  |  |  |  |  |  |  |  |  |  |  |  |  |  |  |  |  |  |  |  |  |  |  |  |  |  |  |  |  |  |  |  |  |  |  |  |  |  |  |  |  |  |  |  |  |  |  |  |  |  |  |  |  |  |  |  |  |  |  |  |  |  |  |  |  |  |  |  |  |  |  |  |  |  |  |  |  |  |  |  |  |  |  |  |  |  |  |  |  |  |  |  |  |  |  |  |  |  |  |  |  |  |  |  |  |  |  |  |  |  |  |  |  |  |  |  |  |  |  |  |  |  |  |  |  |  |  |  |  |  |  |  |  |  |  |  |  |  |  |  |  |  |  |  |  |  |  |  |  |  |  |  |  |  |  |  |  |  |  |  |  |  |  |  |  |  |  |  |  |  |  |  |  |  |  |  |  |  |  |  |  |  |  |  |  |  |  |  |  |  |  |  |  |  |  |  |  |  |  |  |  |  |  |  |  |  |  |  |  |  |  |  |  |  |  |  |  |  |  |  |  |  |  |  |  |  |  |  |  |  |  |  |  |  |  |  |  |  |  |  |  |  |  |  |  |  |  |  |  |  |  |  |  |  |  |  |  |  |  |  |  |  |  |  |  |  |  |  |  |  |  |  |  |  |  |  |  |  |  |  |  |  |  |  |  |  |  |  |  |  |  |  |  |  |  |  |  |  |  |  |  |  |  |  |  |  |  |  |  |  |  |  |  |  |  |  |  |  |  |  |  |  |  |  |  |  |  |  |  |  |  |  |  |  |  |  |  |  |  |  |  |  |  |  |  |  |  |  |  |  |  |  |  |  |  |  |  |  |  |  |  |  |  |  |  |  |  |  |  |  |  |  |  |  |  |  |  |  |  |  |  |  |  |  |  |  |  |  |  |  |  |  |  |  |  |  |  |  |  |  |  |  |  |  |  |  |  |  |  |  |  |  |  |  |  |  |  |  |  |  |  |  |  |  |  |  |  |  |  |  |  |  |  |  |  |  |  |  |  |  |  |  |  |  |  |  |  |  |  |  |  |  |  |  |  |  |  |  |  |  |  |  |  |  |  |  |  |  |  |  |  |  |  |  |  |  |  |  |  |  |  |  |  |  |  |  |  |  |  |  |  |  |  |  |  |  |  |  |  |  |  |  |  |  |  |  |  |  |  |  |  |  |  |  |  |  |  |  |  |  |  |  |  |  |  |  |  |  |  |  |  |  |  |  |  |  |  |  |  |  |  |  |  |  |  |  |  |  |  |  |  |  |  |  |  |  |  |  |  |  |  |  |  |  |  |  |  |  |  |  |  |  |  |  |  |  |  |  |  |  |  |  |  |  |  |  |  |  |  |  |  |  |  |  |  |  |  |  |  |  |  |  |  |  |  |  |  |  |  |  |  |  |  |  |  |  |  |  |  |  |  |  |  |  |  |  |  |  |  |  |  |  |  |  |  |  |  |  |  |  |  |  |  |  |  |  |  |  |  |  |  |  |  |  |  |  |  |  |  |  |  |  |  |  |  |  |  |  |  |  |  |  |  |  |  |  |  |  |  |  |  |  |  |  |  |  |  |  |  |  |  |  |  |  |  |  |  |  |  |  |  |  |  |  |  |  |  |  |  |  |  |  |  |  |  |  |  |  |  |  |  |  |  |  |  |  |  |  |  |  |  |  |  |  |  |  |  |  |  |  |  |  |  |  |  |  |  |  |  |  |  |  |  |  |  |  |  |  |  |  |  |  |  |  |  |  |  |  |  |  |  |  |  |  |  |  |  |  |  |  |  |  |  |  |  |  |  |  |  |  |  |  |  |  |  |  |  |  |  |  |  |  |  |  |  |  |  |  |  |  |  |  |  |  |  |  |  |  |  |  |  |  |  |  |  |  |  |  |  |  |  |  |  |  |  |  |  |  |  |  |  |  |  |  |  |  |  |  |  |  |  |  |  |  |  |  |  |  |  |  |  |  |  |  |  |  |  |  |  |  |  |  |  |  |  |  |  |  |  |  |  |  |  |  |  |  |  |  |  |  |  |  |  |  |  |  |  |  |  |  |  |  |  |  |  |  |  |  |  |  |  |  |  |  |  |  |  |  |  |  |  |  |  |  |  |  |  |  |  |  |  |  |  |  |  |  |  |  |  |  |  |  |  |  |  |  |  |  |  |  |  |  |  |  |  |  |  |  |  |  |  |  |  |  |  |  |  |  |  |  |  |  |  |  |  |  |  |  |  |  |  |  |  |  |  |  |  |  |  |  |  |  |  |  |  |  |  |  |  |  |  |  |  |  |  |  |  |  |  |  |  |  |  |  |  |  |  |  |  |  |  |  |  |  |  |  |  |  |  |  |  |  |  |  |  |  |  |  |  |  |  |  |  |  |  |  |  |  |  |  |  |  |  |  |  |  |  |  |  |  |  |  |  |  |  |  |  |  |  |  |  |  |  |  |  |  |  |  |  |  |  |  |  |  |  |  |  |  |  |  |  |  |  |  |  |  |  |  |  |  |  |  |  |  |  |  |  |  |  |  |  |  |  |  |  |  |  |  |  |  |  |  |  |  |  |  |  |  |  |  |  |  |  |  |  |  |  |  |  |  |  |  |  |  |  |  |  |  |  |  |  |  |  |  |  |  |  |  |  |  |  |  |  |  |  |  |  |  |  |  |  |  |  |  |  |  |  |  |  |  |  |  |  |  |  |  |  |  |  |  |  |  |  |  |  |  |  |  |  |  |  |  |  |  |  |  |  |  |  |  |  |  |  |  |  |  |  |  |  |  |  |  |  |  |  |  |  |  |  |  |  |  |  |  |  |  |  |  |  |  |  |  |  |  |  |  |  |  |  |  |  |  |  |  |  |  |  |  |  |  |  |  |  |  |  |  |  |  |  |  |  |  |  |  |  |  |  |  |  |  |  |  |  |  |  |  |  |  |  |  |  |  |  |  |  |  |  |  |  |  |  |  |  |  |  |  |  |  |  |  |  |  |  |  |  |  |  |  |  |  |  |  |  |  |  |  |  |  |  |  |  |  |  |  |  |  |  |  |  |  |  |  |  |  |  |  |  |  |  |  |  |  |  |  |  |  |  |  |  |  |  |  |  |  |  |  |  |  |  |  |  |  |  |  |  |  |  |  |  |  |  |  |  |  |  |  |  |  |  |  |  |  |  |  |  |  |  |  |  |  |  |  |  |  |  |  |  |  |  |  |  |  |  |  |  |  |  |  |  |  |  |  |  |  |  |  |  |  |  |  |  |  |  |  |  |  |  |  |  |  |  |  |  |  |  |  |  |  |  |  |  |  |  |  |  |  |  |  |  |  |  |  |  |  |  |  |  |  |  |  |  |  |  |  |  |  |  |  |  |  |  |  |  |  |  |  |  |  |  |  |  |  |  |  |  |  |  |  |  |  |  |  |  |  |  |  |  |  |  |  |  |  |  |  |  |  |  |  |  |  |  |  |  |  |  |  |  |  |  |  |  |  |  |  |  |  |  |  |  |  |  |  |  |  |  |  |  |  |  |  |  |  |  |  |  |  |  |  |  |  |  |  |  |  |  |  |  |  |  |  |  |  |  |  |  |  |  |  |  |  |  |  |  |  |  |  |  |  |  |  |  |  |  |  |  |  |  |  |  |  |  |  |  |  |  |  |  |  |  |  |  |  |  |  |  |  |  |  |  |  |  |  |  |  |  |  |  |  |  |  |  |  |  |  |  |  |  |  |  |  |  |  |  |  |  |  |  |  |  |  |  |  |  |  |  |  |  |  |  |  |  |  |  |  |  |  |  |  |  |  |  |  |  |  |  |  |  |  |  |  |  |  |  |  |  |  |  |  |  |  |  |  |  |  |  |  |  |  |  |  |  |  |  |  |  |  |  |  |  |  |  |  |  |  |  |  |  |  |  |  |  |  |  |  |  |  |  |  |  |  |  |  |  |  |  |  |  |  |  |  |  |  |  |  |  |  |  |  |  |  |  |  |  |  |  |  |  |  |  |  |  |  |  |  |  |  |  |  |  |  |  |  |  |  |  |  |  |  |  |  |  |  |  |  |  |  |  |  |  |  |  |  |  |  |  |  |  |  |  |  |  |  |  |  |  |  |  |  |  |  |  |  |  |  |  |  |  |  |  |  |  |  |  |  |  |  |  |  |  |  |  |  |  |  |  |  |  |  |  |  |  |  |  |  |  |  |  |  |  |  |  |  |  |  |  |  |  |  |  |  |  |  |  |  |  |  |  |  |  |  |  |  |  |  |  |  |  |  |  |  |  |  |  |  |
| --- | --- | --- | --- | --- | --- | --- | --- | --- | --- | --- | --- | --- | --- | --- | --- | --- | --- | --- | --- | --- | --- | --- | --- | --- | --- | --- | --- | --- | --- | --- | --- | --- | --- | --- | --- | --- | --- | --- | --- | --- | --- | --- | --- | --- | --- | --- | --- | --- | --- | --- | --- | --- | --- | --- | --- | --- | --- | --- | --- | --- | --- | --- | --- | --- | --- | --- | --- | --- | --- | --- | --- | --- | --- | --- | --- | --- | --- | --- | --- | --- | --- | --- | --- | --- | --- | --- | --- | --- | --- | --- | --- | --- | --- | --- | --- | --- | --- | --- | --- | --- | --- | --- | --- | --- | --- | --- | --- | --- | --- | --- | --- | --- | --- | --- | --- | --- | --- | --- | --- | --- | --- | --- | --- | --- | --- | --- | --- | --- | --- | --- | --- | --- | --- | --- | --- | --- | --- | --- | --- | --- | --- | --- | --- | --- | --- | --- | --- | --- | --- | --- | --- | --- | --- | --- | --- | --- | --- | --- | --- | --- | --- | --- | --- | --- | --- | --- | --- | --- | --- | --- | --- | --- | --- | --- | --- | --- | --- | --- | --- | --- | --- | --- | --- | --- | --- | --- | --- | --- | --- | --- | --- | --- | --- | --- | --- | --- | --- | --- | --- | --- | --- | --- | --- | --- | --- | --- | --- | --- | --- | --- | --- | --- | --- | --- | --- | --- | --- | --- | --- | --- | --- | --- | --- | --- | --- | --- | --- | --- | --- | --- | --- | --- | --- | --- | --- | --- | --- | --- | --- | --- | --- | --- | --- | --- | --- | --- | --- | --- | --- | --- | --- | --- | --- | --- | --- | --- | --- | --- | --- | --- | --- | --- | --- | --- | --- | --- | --- | --- | --- | --- | --- | --- | --- | --- | --- | --- | --- | --- | --- | --- | --- | --- | --- | --- | --- | --- | --- | --- | --- | --- | --- | --- | --- | --- | --- | --- | --- | --- | --- | --- | --- | --- | --- | --- | --- | --- | --- | --- | --- | --- | --- | --- | --- | --- | --- | --- | --- | --- | --- | --- | --- | --- | --- | --- | --- | --- | --- | --- | --- | --- | --- | --- | --- | --- | --- | --- | --- | --- | --- | --- | --- | --- | --- | --- | --- | --- | --- | --- | --- | --- | --- | --- | --- | --- | --- | --- | --- | --- | --- | --- | --- | --- | --- | --- | --- | --- | --- | --- | --- | --- | --- | --- | --- | --- | --- | --- | --- | --- | --- | --- | --- | --- | --- | --- | --- | --- | --- | --- | --- | --- | --- | --- | --- | --- | --- | --- | --- | --- | --- | --- | --- | --- | --- | --- | --- | --- | --- | --- | --- | --- | --- | --- | --- | --- | --- | --- | --- | --- | --- | --- | --- | --- | --- | --- | --- | --- | --- | --- | --- | --- | --- | --- | --- | --- | --- | --- | --- | --- | --- | --- | --- | --- | --- | --- | --- | --- | --- | --- | --- | --- | --- | --- | --- | --- | --- | --- | --- | --- | --- | --- | --- | --- | --- | --- | --- | --- | --- | --- | --- | --- | --- | --- | --- | --- | --- | --- | --- | --- | --- | --- | --- | --- | --- | --- | --- | --- | --- | --- | --- | --- | --- | --- | --- | --- | --- | --- | --- | --- | --- | --- | --- | --- | --- | --- | --- | --- | --- | --- | --- | --- | --- | --- | --- | --- | --- | --- | --- | --- | --- | --- | --- | --- | --- | --- | --- | --- | --- | --- | --- | --- | --- | --- | --- | --- | --- | --- | --- | --- | --- | --- | --- | --- | --- | --- | --- | --- | --- | --- | --- | --- | --- | --- | --- | --- | --- | --- | --- | --- | --- | --- | --- | --- | --- | --- | --- | --- | --- | --- | --- | --- | --- | --- | --- | --- | --- | --- | --- | --- | --- | --- | --- | --- | --- | --- | --- | --- | --- | --- | --- | --- | --- | --- | --- | --- | --- | --- | --- | --- | --- | --- | --- | --- | --- | --- | --- | --- | --- | --- | --- | --- | --- | --- | --- | --- | --- | --- | --- | --- | --- | --- | --- | --- | --- | --- | --- | --- | --- | --- | --- | --- | --- | --- | --- | --- | --- | --- | --- | --- | --- | --- | --- | --- | --- | --- | --- | --- | --- | --- | --- | --- | --- | --- | --- | --- | --- | --- | --- | --- | --- | --- | --- | --- | --- | --- | --- | --- | --- | --- | --- | --- | --- | --- | --- | --- | --- | --- | --- | --- | --- | --- | --- | --- | --- | --- | --- | --- | --- | --- | --- | --- | --- | --- | --- | --- | --- | --- | --- | --- | --- | --- | --- | --- | --- | --- | --- | --- | --- | --- | --- | --- | --- | --- | --- | --- | --- | --- | --- | --- | --- | --- | --- | --- | --- | --- | --- | --- | --- | --- | --- | --- | --- | --- | --- | --- | --- | --- | --- | --- | --- | --- | --- | --- | --- | --- | --- | --- | --- | --- | --- | --- | --- | --- | --- | --- | --- | --- | --- | --- | --- | --- | --- | --- | --- | --- | --- | --- | --- | --- | --- | --- | --- | --- | --- | --- | --- | --- | --- | --- | --- | --- | --- | --- | --- | --- | --- | --- | --- | --- | --- | --- | --- | --- | --- | --- | --- | --- | --- | --- | --- | --- | --- | --- | --- | --- | --- | --- | --- | --- | --- | --- | --- | --- | --- | --- | --- | --- | --- | --- | --- | --- | --- | --- | --- | --- | --- | --- | --- | --- | --- | --- | --- | --- | --- | --- | --- | --- | --- | --- | --- | --- | --- | --- | --- | --- | --- | --- | --- | --- | --- | --- | --- | --- | --- | --- | --- | --- | --- | --- | --- | --- | --- | --- | --- | --- | --- | --- | --- | --- | --- | --- | --- | --- | --- | --- | --- | --- | --- | --- | --- | --- | --- | --- | --- | --- | --- | --- | --- | --- | --- | --- | --- | --- | --- | --- | --- | --- | --- | --- | --- | --- | --- | --- | --- | --- | --- | --- | --- | --- | --- | --- | --- | --- | --- | --- | --- | --- | --- | --- | --- | --- | --- | --- | --- | --- | --- | --- | --- | --- | --- | --- | --- | --- | --- | --- | --- | --- | --- | --- | --- | --- | --- | --- | --- | --- | --- | --- | --- | --- | --- | --- | --- | --- | --- | --- | --- | --- | --- | --- | --- | --- | --- | --- | --- | --- | --- | --- | --- | --- | --- | --- | --- | --- | --- | --- | --- | --- | --- | --- | --- | --- | --- | --- | --- | --- | --- | --- | --- | --- | --- | --- | --- | --- | --- | --- | --- | --- | --- | --- | --- | --- | --- | --- | --- | --- | --- | --- | --- | --- | --- | --- | --- | --- | --- | --- | --- | --- | --- | --- | --- | --- | --- | --- | --- | --- | --- | --- | --- | --- | --- | --- | --- | --- | --- | --- | --- | --- | --- | --- | --- | --- | --- | --- | --- | --- | --- | --- | --- | --- | --- | --- | --- | --- | --- | --- | --- | --- | --- | --- | --- | --- | --- | --- | --- | --- | --- | --- | --- | --- | --- | --- | --- | --- | --- | --- | --- | --- | --- | --- | --- | --- | --- | --- | --- | --- | --- | --- | --- | --- | --- | --- | --- | --- | --- | --- | --- | --- | --- | --- | --- | --- | --- | --- | --- | --- | --- | --- | --- | --- | --- | --- | --- | --- | --- | --- | --- | --- | --- | --- | --- | --- | --- | --- | --- | --- | --- | --- | --- | --- | --- | --- | --- | --- | --- | --- | --- | --- | --- | --- | --- | --- | --- | --- | --- | --- | --- | --- | --- | --- | --- | --- | --- | --- | --- | --- | --- | --- | --- | --- | --- | --- | --- | --- | --- | --- | --- | --- | --- | --- | --- | --- | --- | --- | --- | --- | --- | --- | --- | --- | --- | --- | --- | --- | --- | --- | --- | --- | --- | --- | --- | --- | --- | --- | --- | --- | --- | --- | --- | --- | --- | --- | --- | --- | --- | --- | --- | --- | --- | --- | --- | --- | --- | --- | --- | --- | --- | --- | --- | --- | --- | --- | --- | --- | --- | --- | --- | --- | --- | --- | --- | --- | --- | --- | --- | --- | --- | --- | --- | --- | --- | --- | --- | --- | --- | --- | --- | --- | --- | --- | --- | --- | --- | --- | --- | --- | --- | --- | --- | --- | --- | --- | --- | --- | --- | --- | --- | --- | --- | --- | --- | --- | --- | --- | --- | --- | --- | --- | --- | --- | --- | --- | --- | --- | --- | --- | --- | --- | --- | --- | --- | --- | --- | --- | --- | --- | --- | --- | --- | --- | --- | --- | --- | --- | --- | --- | --- | --- | --- | --- | --- | --- | --- | --- | --- | --- | --- | --- | --- | --- | --- | --- | --- | --- | --- | --- | --- | --- | --- | --- | --- | --- | --- | --- | --- | --- | --- | --- | --- | --- | --- | --- | --- | --- | --- | --- | --- | --- | --- | --- | --- | --- | --- | --- | --- | --- | --- | --- | --- | --- | --- | --- | --- | --- | --- | --- | --- | --- | --- | --- | --- | --- | --- | --- | --- | --- | --- | --- | --- | --- | --- | --- | --- | --- | --- | --- | --- | --- | --- | --- | --- | --- | --- | --- | --- | --- | --- | --- | --- | --- | --- | --- | --- | --- | --- | --- | --- | --- | --- | --- | --- | --- | --- | --- | --- | --- | --- | --- | --- | --- | --- | --- | --- | --- | --- | --- | --- | --- | --- | --- | --- | --- | --- | --- | --- | --- | --- | --- | --- | --- | --- | --- | --- | --- | --- | --- | --- | --- | --- | --- | --- | --- | --- | --- | --- | --- | --- | --- | --- | --- | --- | --- | --- | --- | --- | --- | --- | --- | --- | --- | --- | --- | --- | --- | --- | --- | --- | --- | --- | --- | --- | --- | --- | --- | --- | --- | --- | --- | --- | --- | --- | --- | --- | --- | --- | --- | --- | --- | --- | --- | --- | --- | --- | --- | --- | --- | --- | --- | --- | --- | --- | --- | --- | --- | --- | --- | --- | --- | --- | --- | --- | --- | --- | --- | --- | --- | --- | --- | --- | --- | --- | --- | --- | --- | --- | --- | --- | --- | --- | --- | --- | --- | --- | --- | --- | --- | --- | --- | --- | --- | --- | --- | --- | --- | --- | --- | --- | --- | --- | --- | --- | --- | --- | --- | --- | --- | --- | --- | --- | --- | --- | --- | --- | --- | --- | --- | --- | --- | --- | --- | --- | --- | --- | --- | --- | --- | --- | --- | --- | --- | --- | --- | --- | --- | --- | --- | --- | --- | --- | --- | --- | --- | --- | --- | --- | --- | --- | --- | --- | --- | --- | --- | --- | --- | --- | --- | --- | --- | --- | --- | --- | --- | --- | --- | --- | --- | --- | --- | --- | --- | --- | --- | --- | --- | --- | --- | --- | --- | --- | --- | --- | --- | --- | --- | --- | --- | --- | --- | --- | --- | --- | --- | --- | --- | --- | --- | --- | --- | --- | --- | --- | --- | --- | --- | --- | --- | --- | --- | --- | --- | --- | --- | --- | --- | --- | --- | --- | --- | --- | --- | --- | --- | --- | --- | --- | --- | --- | --- | --- | --- | --- | --- | --- | --- | --- | --- | --- | --- | --- | --- | --- | --- | --- | --- | --- | --- | --- | --- | --- | --- | --- | --- | --- | --- | --- | --- | --- | --- | --- | --- | --- | --- | --- | --- | --- | --- | --- | --- | --- | --- | --- | --- | --- | --- | --- | --- | --- | --- | --- | --- | --- | --- | --- | --- | --- | --- | --- | --- | --- | --- | --- | --- | --- | --- | --- | --- | --- | --- | --- | --- | --- | --- | --- | --- | --- | --- | --- | --- | --- | --- | --- | --- | --- | --- | --- | --- | --- | --- | --- | --- | --- | --- | --- | --- | --- | --- | --- | --- | --- | --- | --- | --- | --- | --- | --- | --- | --- | --- | --- | --- | --- | --- | --- | --- | --- | --- | --- | --- | --- | --- | --- | --- | --- | --- | --- | --- | --- | --- | --- | --- | --- | --- | --- | --- | --- | --- | --- | --- | --- | --- | --- | --- | --- | --- | --- | --- | --- | --- | --- | --- | --- | --- | --- | --- | --- | --- | --- | --- | --- | --- | --- | --- | --- | --- | --- | --- | --- | --- | --- | --- | --- | --- | --- | --- | --- | --- | --- | --- | --- | --- | --- | --- | --- | --- | --- | --- | --- | --- | --- | --- | --- | --- | --- | --- | --- | --- | --- | --- | --- | --- | --- | --- | --- | --- | --- | --- | --- | --- | --- | --- | --- | --- | --- | --- | --- | --- | --- | --- | --- | --- | --- | --- | --- | --- | --- | --- | --- | --- | --- | --- | --- | --- | --- | --- | --- | --- | --- | --- | --- | --- | --- | --- | --- | --- | --- | --- | --- | --- | --- | --- | --- | --- | --- | --- | --- | --- | --- | --- | --- | --- | --- | --- | --- | --- | --- | --- | --- | --- | --- | --- | --- | --- | --- | --- | --- | --- | --- | --- | --- | --- | --- | --- | --- | --- | --- | --- | --- | --- | --- | --- | --- | --- | --- | --- | --- | --- | --- | --- | --- | --- | --- | --- | --- | --- | --- | --- | --- | --- | --- | --- | --- | --- | --- | --- | --- | --- | --- | --- | --- | --- | --- | --- | --- | --- | --- | --- | --- | --- | --- | --- | --- | --- | --- | --- | --- | --- | --- | --- | --- | --- | --- | --- | --- | --- | --- | --- | --- | --- | --- | --- | --- | --- | --- | --- | --- | --- | --- | --- | --- | --- | --- | --- | --- | --- | --- | --- | --- | --- | --- | --- | --- | --- | --- | --- | --- | --- | --- | --- | --- | --- | --- | --- | --- | --- | --- | --- | --- | --- | --- | --- | --- | --- | --- | --- | --- | --- | --- | --- | --- | --- | --- | --- | --- | --- | --- | --- | --- | --- | --- | --- | --- | --- | --- | --- | --- | --- | --- | --- | --- | --- | --- | --- | --- | --- | --- | --- | --- | --- | --- | --- | --- | --- | --- | --- | --- | --- | --- | --- | --- | --- | --- | --- | --- | --- | --- | --- | --- | --- | --- | --- | --- | --- | --- | --- | --- | --- | --- | --- | --- | --- | --- | --- | --- | --- | --- | --- | --- | --- | --- | --- | --- | --- | --- | --- | --- | --- | --- | --- | --- | --- | --- | --- | --- | --- | --- | --- | --- | --- | --- | --- | --- | --- | --- | --- | --- | --- | --- | --- | --- | --- | --- | --- | --- | --- | --- | --- | --- | --- | --- | --- | --- | --- | --- | --- | --- | --- | --- | --- | --- | --- | --- | --- | --- | --- | --- | --- | --- | --- | --- | --- | --- | --- | --- | --- | --- | --- | --- | --- | --- | --- | --- | --- | --- | --- | --- | --- | --- | --- | --- | --- | --- | --- | --- | --- | --- | --- | --- | --- | --- | --- | --- | --- | --- | --- | --- | --- | --- | --- | --- | --- | --- | --- | --- | --- | --- | --- | --- | --- | --- | --- | --- | --- | --- | --- | --- | --- | --- | --- | --- | --- | --- | --- | --- | --- | --- | --- | --- | --- | --- | --- | --- | --- | --- | --- | --- | --- | --- | --- | --- | --- | --- | --- | --- | --- | --- | --- | --- | --- | --- | --- | --- | --- | --- | --- | --- | --- | --- | --- | --- | --- | --- | --- | --- | --- | --- | --- | --- | --- | --- | --- | --- | --- | --- | --- | --- | --- | --- | --- | --- | --- | --- | --- | --- | --- | --- | --- | --- | --- | --- | --- | --- | --- | --- | --- | --- | --- | --- | --- | --- | --- | --- | --- | --- | --- | --- | --- | --- | --- | --- | --- | --- | --- | --- | --- | --- | --- | --- | --- | --- | --- | --- | --- | --- | --- | --- | --- | --- | --- | --- | --- | --- | --- | --- | --- | --- | --- | --- | --- | --- | --- | --- | --- | --- | --- | --- | --- | --- | --- | --- | --- | --- | --- | --- | --- | --- | --- | --- | --- | --- | --- | --- | --- | --- | --- | --- | --- | --- | --- | --- | --- | --- | --- | --- | --- | --- | --- | --- | --- | --- | --- | --- | --- | --- | --- | --- | --- | --- | --- | --- | --- | --- | --- | --- | --- | --- | --- | --- | --- | --- | --- | --- | --- | --- | --- | --- | --- | --- | --- | --- | --- | --- | --- | --- | --- | --- | --- | --- | --- | --- | --- | --- | --- | --- | --- | --- | --- | --- | --- | --- | --- | --- | --- | --- | --- | --- | --- | --- | --- | --- | --- | --- | --- | --- | --- | --- | --- | --- | --- | --- | --- | --- | --- | --- | --- | --- | --- | --- | --- | --- | --- | --- | --- | --- | --- | --- | --- | --- | --- | --- | --- | --- | --- | --- | --- | --- | --- | --- | --- | --- | --- | --- | --- | --- | --- | --- | --- | --- | --- | --- | --- | --- | --- | --- | --- | --- | --- | --- | --- | --- | --- | --- | --- | --- | --- | --- | --- | --- | --- | --- | --- | --- | --- | --- | --- | --- | --- | --- | --- | --- | --- | --- | --- | --- | --- | --- | --- | --- | --- | --- | --- | --- | --- | --- | --- | --- | --- | --- | --- | --- | --- | --- | --- | --- | --- | --- | --- | --- | --- | --- | --- | --- | --- | --- | --- | --- | --- | --- | --- | --- | --- | --- | --- | --- | --- | --- | --- | --- | --- | --- | --- | --- | --- | --- | --- | --- | --- | --- | --- | --- | --- | --- | --- | --- | --- | --- | --- | --- | --- | --- | --- | --- | --- | --- | --- | --- | --- | --- | --- | --- | --- | --- | --- | --- | --- | --- | --- | --- | --- | --- | --- | --- | --- | --- | --- | --- | --- | --- | --- | --- | --- | --- | --- | --- | --- | --- | --- | --- | --- | --- | --- | --- | --- | --- | --- | --- | --- | --- | --- | --- | --- | --- | --- | --- | --- | --- | --- | --- | --- | --- | --- | --- | --- | --- | --- | --- | --- | --- | --- | --- | --- | --- | --- | --- | --- | --- | --- | --- | --- | --- | --- | --- | --- | --- | --- | --- | --- | --- | --- | --- | --- | --- | --- | --- | --- | --- | --- | --- | --- | --- | --- | --- | --- | --- | --- | --- | --- | --- | --- | --- | --- | --- | --- | --- | --- | --- | --- | --- | --- | --- | --- | --- | --- | --- | --- | --- | --- | --- | --- | --- | --- | --- | --- | --- | --- | --- | --- | --- | --- | --- | --- | --- | --- | --- | --- | --- | --- | --- | --- | --- | --- | --- | --- | --- | --- | --- | --- | --- | --- | --- | --- | --- | --- | --- | --- | --- | --- | --- | --- | --- | --- | --- | --- | --- | --- | --- | --- | --- | --- | --- | --- | --- | --- | --- | --- | --- | --- | --- | --- | --- | --- | --- | --- | --- | --- | --- | --- | --- | --- | --- | --- | --- | --- | --- | --- | --- | --- | --- | --- | --- | --- | --- | --- | --- | --- | --- | --- | --- | --- | --- | --- | --- | --- | --- | --- | --- | --- | --- | --- | --- | --- | --- | --- | --- | --- | --- | --- | --- | --- | --- | --- | --- | --- | --- | --- | --- | --- | --- | --- | --- | --- | --- | --- | --- | --- | --- | --- | --- | --- | --- | --- | --- | --- | --- | --- | --- | --- | --- | --- | --- | --- | --- | --- | --- | --- | --- | --- | --- | --- | --- | --- | --- | --- | --- | --- | --- | --- | --- | --- | --- | --- | --- | --- | --- | --- | --- | --- | --- | --- | --- | --- | --- | --- | --- | --- | --- | --- | --- | --- | --- | --- | --- | --- | --- | --- | --- | --- | --- | --- | --- | --- | --- | --- | --- | --- | --- | --- | --- | --- | --- | --- | --- | --- | --- | --- | --- | --- | --- | --- | --- | --- | --- | --- | --- | --- | --- | --- | --- | --- | --- | --- | --- | --- | --- | --- | --- | --- | --- | --- | --- | --- | --- | --- | --- | --- | --- | --- | --- | --- | --- | --- | --- | --- | --- | --- | --- | --- | --- | --- | --- | --- | --- | --- | --- | --- | --- | --- | --- | --- | --- | --- | --- | --- | --- | --- | --- | --- | --- | --- | --- | --- | --- | --- | --- | --- | --- | --- | --- | --- | --- | --- | --- | --- | --- | --- | --- | --- | --- | --- | --- | --- | --- | --- | --- | --- | --- | --- | --- | --- | --- | --- | --- | --- | --- | --- | --- | --- | --- | --- | --- | --- | --- | --- | --- | --- | --- | --- | --- | --- | --- | --- | --- | --- | --- | --- | --- | --- | --- | --- | --- | --- | --- | --- | --- | --- | --- | --- | --- | --- | --- | --- | --- | --- | --- | --- | --- | --- | --- | --- | --- | --- | --- | --- | --- | --- | --- | --- | --- | --- | --- | --- | --- | --- | --- | --- | --- | --- | --- | --- | --- | --- | --- | --- | --- | --- | --- | --- | --- | --- | --- | --- | --- | --- | --- | --- | --- | --- | --- | --- | --- | --- | --- | --- | --- | --- | --- | --- | --- | --- | --- | --- | --- | --- | --- | --- | --- | --- | --- | --- | --- | --- | --- | --- | --- | --- | --- | --- |
| |  |  |  |  |  |  |  |  |  |  |  |  |  |  |  |  |  |  |  |  |  |  |  |  |  |  |  |  |  |  |  |  |  |  |  |  |  |  |  |  |  |  |  |  |  |  |  |  |  |  |  |  |  |  |  |  |  |  | | --- | --- | --- | --- | --- | --- | --- | --- | --- | --- | --- | --- | --- | --- | --- | --- | --- | --- | --- | --- | --- | --- | --- | --- | --- | --- | --- | --- | --- | --- | --- | --- | --- | --- | --- | --- | --- | --- | --- | --- | --- | --- | --- | --- | --- | --- | --- | --- | --- | --- | --- | --- | --- | --- | --- | --- | --- | --- | | G0VKH1/1-220 | 1 | - | - | - | - | - | - | - | - | - | - | - | - | - | - | - | - | - | - | - | M | A | R | R | P | A | R | C | Y | R | Y | Q | K | N | K | P | Y | P | K | S | R | Y | N | R | A | V | P | D | S | K | I | R | I | Y | D | L | 36 | | Q6CRX7/1-220 | 1 | - | - | - | - | - | - | - | - | - | - | - | - | - | - | - | - | - | - | - | M | A | R | R | P | A | R | C | Y | R | Y | Q | K | N | K | P | Y | P | K | S | R | Y | N | R | A | V | P | D | S | K | I | R | I | Y | D | L | 36 | | Q6FLX0/1-221 | 1 | - | - | - | - | - | - | - | - | - | - | - | - | - | - | - | - | - | - | - | M | A | R | R | P | A | R | C | Y | R | Y | Q | K | N | K | P | Y | P | K | S | R | Y | N | R | A | V | P | D | S | K | I | R | I | Y | D | L | 36 | | Q75CY7/1-240 | 1 | M | E | L | R | T | R | P | D | S | A | S | S | E | T | T | T | S | S | K | M | A | R | R | P | A | R | C | Y | R | Y | Q | K | N | K | P | Y | P | K | S | R | Y | N | R | A | V | P | D | S | K | I | R | I | Y | D | L | 55 | | A7TFN4/1-220 | 1 | - | - | - | - | - | - | - | - | - | - | - | - | - | - | - | - | - | - | - | M | A | R | R | P | A | R | C | Y | R | Y | Q | K | N | K | P | Y | P | K | S | R | Y | N | R | A | V | P | D | S | K | I | R | I | Y | D | L | 36 | | C5DNR0/1-220 | 1 | - | - | - | - | - | - | - | - | - | - | - | - | - | - | - | - | - | - | - | M | A | R | R | P | A | R | C | Y | R | Y | Q | K | N | K | P | Y | P | K | S | R | Y | N | R | A | V | P | D | S | K | I | R | I | Y | D | L | 36 | | C5DX59/1-223 | 1 | - | - | - | - | - | - | - | - | - | - | - | - | - | - | - | - | - | - | - | M | A | R | R | P | A | R | C | Y | R | Y | Q | K | N | K | P | Y | P | K | S | R | Y | N | R | A | V | P | D | S | K | I | R | I | Y | D | L | 36 | | Kwal\_14.1060/1-220 | 1 | - | - | - | - | - | - | - | - | - | - | - | - | - | - | - | - | - | - | - | M | A | R | R | P | A | R | C | Y | R | Y | Q | K | N | K | P | Y | P | K | S | R | Y | N | R | A | V | P | D | S | K | I | R | I | Y | D | L | 36 | | Sbay\_672.56/1-221 | 1 | - | - | - | - | - | - | - | - | - | - | - | - | - | - | - | - | - | - | - | M | A | R | R | P | A | R | C | Y | R | Y | Q | K | N | K | P | Y | P | K | S | R | Y | N | R | A | V | P | D | S | K | I | R | I | Y | D | L | 36 | | SAKL0B00682g/1-220 | 1 | - | - | - | - | - | - | - | - | - | - | - | - | - | - | - | - | - | - | - | M | A | R | R | P | A | R | C | Y | R | Y | Q | K | N | K | P | Y | P | K | S | R | Y | N | R | A | V | P | D | S | K | I | R | I | Y | D | L | 36 | | P41805/1-221 | 1 | - | - | - | - | - | - | - | - | - | - | - | - | - | - | - | - | - | - | - | M | A | R | R | P | A | R | C | Y | R | Y | Q | K | N | K | P | Y | P | K | S | R | Y | N | R | A | V | P | D | S | K | I | R | I | Y | D | L | 36 | |  | | G0VKH1/1-220 | 37 | G | K | K | K | A | T | V | D | E | F | P | L | C | V | H | L | V | S | N | E | L | E | Q | L | S | S | E | A | L | E | A | A | R | I | C | A | N | K | Y | V | T | T | V | S | G | R | E | S | F | H | L | R | V | R | V | 91 | | Q6CRX7/1-220 | 37 | G | K | K | K | A | T | V | D | E | F | P | L | C | V | H | L | V | S | N | E | L | E | Q | L | S | S | E | A | L | E | A | A | R | I | T | A | N | K | Y | I | T | K | M | T | G | R | D | S | F | H | L | R | V | R | V | 91 | | Q6FLX0/1-221 | 37 | G | K | K | K | A | T | V | D | E | F | P | L | C | V | H | L | V | S | N | E | L | E | Q | L | S | S | E | A | L | E | A | A | R | I | C | A | N | K | Y | M | T | K | I | S | G | R | D | S | F | H | L | R | V | R | V | 91 | | Q75CY7/1-240 | 56 | G | K | K | K | A | T | V | D | E | F | P | L | C | V | H | L | V | S | N | E | L | E | Q | L | S | S | E | A | L | E | A | A | R | I | C | A | N | K | Y | I | T | K | M | T | G | R | D | S | F | H | L | R | V | R | V | 110 | | A7TFN4/1-220 | 37 | G | K | K | K | A | T | V | D | E | F | P | L | C | V | H | L | V | S | N | E | L | E | Q | L | S | S | E | A | L | E | A | A | R | I | C | A | N | K | Y | M | T | K | I | S | G | R | D | A | F | H | L | R | V | R | V | 91 | | C5DNR0/1-220 | 37 | G | K | K | K | A | T | V | D | E | F | P | L | C | V | H | L | V | S | N | E | L | E | Q | L | S | S | E | A | L | E | A | A | R | I | C | A | N | K | Y | M | T | K | M | S | G | R | D | A | F | H | L | R | V | R | V | 91 | | C5DX59/1-223 | 37 | G | K | K | K | A | T | V | D | E | F | P | L | C | V | H | L | V | S | N | E | L | E | Q | L | S | S | E | A | L | E | A | A | R | I | C | A | N | K | Y | I | T | K | M | S | G | R | D | A | F | H | L | R | V | R | V | 91 | | Kwal\_14.1060/1-220 | 37 | G | K | K | K | A | T | V | D | E | F | P | L | C | V | H | L | V | S | N | E | L | E | Q | L | S | S | E | A | L | E | A | A | R | I | C | A | N | K | Y | M | T | K | V | S | G | R | D | A | F | H | L | R | V | R | V | 91 | | Sbay\_672.56/1-221 | 37 | G | K | K | K | A | T | V | D | E | F | P | L | C | V | H | L | V | S | N | E | L | E | Q | L | S | S | E | A | L | E | A | A | R | I | C | A | N | K | Y | M | T | T | V | S | G | R | D | A | F | H | L | R | V | R | V | 91 | | SAKL0B00682g/1-220 | 37 | G | K | K | K | A | T | V | D | E | F | P | L | C | V | H | L | V | S | N | E | L | E | Q | L | S | S | E | A | L | E | A | A | R | I | C | A | N | K | Y | I | T | K | I | S | G | R | D | A | F | H | L | R | V | R | V | 91 | | P41805/1-221 | 37 | G | K | K | K | A | T | V | D | E | F | P | L | C | V | H | L | V | S | N | E | L | E | Q | L | S | S | E | A | L | E | A | A | R | I | C | A | N | K | Y | M | T | T | V | S | G | R | D | A | F | H | L | R | V | R | V | 91 | |  | | G0VKH1/1-220 | 92 | H | P | F | H | V | L | R | I | N | K | M | L | S | C | A | G | A | D | R | L | Q | Q | G | M | R | G | A | W | G | K | P | H | G | L | A | A | R | V | D | I | G | Q | I | I | F | S | V | R | T | K | D | N | N | K | D | 146 | | Q6CRX7/1-220 | 92 | H | P | F | H | V | L | R | I | N | K | M | L | S | C | A | G | A | D | R | L | Q | Q | G | M | R | G | A | W | G | K | P | H | G | L | A | A | R | V | A | I | G | Q | V | I | F | S | V | R | T | K | D | N | N | K | D | 146 | | Q6FLX0/1-221 | 92 | H | P | F | H | V | L | R | I | N | K | M | L | S | C | A | G | A | D | R | L | Q | Q | G | M | R | G | A | W | G | K | P | H | G | L | A | A | R | V | D | I | G | Q | I | I | F | S | V | R | T | K | D | N | N | K | D | 146 | | Q75CY7/1-240 | 111 | H | P | F | H | V | L | R | I | N | K | M | L | S | C | A | G | A | D | R | L | Q | Q | G | M | R | G | A | W | G | K | P | H | G | L | A | A | R | V | D | I | G | Q | I | I | F | S | V | R | T | K | D | N | N | K | D | 165 | | A7TFN4/1-220 | 92 | H | P | F | H | V | L | R | I | N | K | M | L | S | C | A | G | A | D | R | L | Q | Q | G | M | R | G | A | W | G | K | P | H | G | L | A | A | R | V | D | I | G | Q | I | I | F | S | V | R | T | K | D | N | N | K | D | 146 | | C5DNR0/1-220 | 92 | H | P | F | H | V | L | R | I | N | K | M | L | S | C | A | G | A | D | R | L | Q | Q | G | M | R | G | A | W | G | K | P | H | G | L | A | A | R | V | A | I | G | Q | I | I | F | S | V | R | T | K | D | N | N | K | D | 146 | | C5DX59/1-223 | 92 | H | P | F | H | V | L | R | I | N | K | M | L | S | C | A | G | A | D | R | L | Q | Q | G | M | R | N | A | W | G | K | P | H | G | L | A | A | R | V | A | I | G | Q | I | L | F | S | V | R | T | R | D | N | N | K | D | 146 | | Kwal\_14.1060/1-220 | 92 | H | P | F | H | V | L | R | I | N | K | M | L | S | C | A | G | A | D | R | L | Q | Q | G | M | R | G | A | W | G | K | P | H | G | L | A | A | R | V | A | I | G | Q | I | I | F | S | V | R | T | K | D | S | N | K | D | 146 | | Sbay\_672.56/1-221 | 92 | H | P | F | H | V | L | R | I | N | K | M | L | S | C | A | G | A | D | R | L | Q | Q | G | M | R | G | A | W | G | K | P | H | G | L | A | A | R | V | S | I | G | Q | I | I | F | S | V | R | T | K | D | N | N | K | D | 146 | | SAKL0B00682g/1-220 | 92 | H | P | F | H | V | L | R | I | N | K | M | L | S | C | A | G | A | D | R | L | Q | Q | G | M | R | G | A | W | G | K | P | H | G | L | A | A | R | V | A | I | G | Q | I | I | F | S | V | R | T | K | D | N | N | K | D | 146 | | P41805/1-221 | 92 | H | P | F | H | V | L | R | I | N | K | M | L | S | C | A | G | A | D | R | L | Q | Q | G | M | R | G | A | W | G | K | P | H | G | L | A | A | R | V | D | I | G | Q | I | I | F | S | V | R | T | K | D | S | N | K | D | 146 | |  | | G0VKH1/1-220 | 147 | V | V | I | E | G | L | R | R | A | R | Y | K | F | P | G | Q | Q | K | I | I | L | S | K | K | W | G | F | T | S | L | D | R | A | E | Y | I | K | R | R | D | A | G | E | V | K | D | D | G | S | F | V | K | F | L | S | 201 | | Q6CRX7/1-220 | 147 | T | V | I | E | G | L | R | R | A | R | Y | K | F | P | G | Q | Q | K | I | I | I | S | K | K | W | G | F | T | S | L | N | R | E | E | Y | V | K | K | R | D | A | G | E | I | K | D | D | G | A | F | V | K | F | L | S | 201 | | Q6FLX0/1-221 | 147 | V | V | V | E | G | L | R | R | A | R | Y | K | F | P | G | Q | Q | K | I | I | M | S | K | K | W | G | F | T | N | L | D | R | A | E | Y | V | K | R | R | D | A | G | E | V | K | D | D | G | A | F | V | K | F | L | S | 201 | | Q75CY7/1-240 | 166 | I | V | V | E | A | L | R | R | A | R | Y | K | F | P | G | Q | Q | K | I | I | M | S | K | K | W | G | F | T | N | L | D | R | A | E | Y | V | R | R | R | D | A | G | E | V | K | D | D | G | A | F | V | K | F | L | S | 220 | | A7TFN4/1-220 | 147 | V | V | I | E | G | L | R | R | A | R | Y | K | F | P | G | Q | Q | K | I | I | I | S | K | K | W | G | F | T | N | L | D | R | P | E | Y | V | K | R | R | D | A | G | E | V | K | D | D | G | A | F | V | K | F | L | S | 201 | | C5DNR0/1-220 | 147 | I | V | V | E | G | L | R | R | A | R | Y | K | F | P | G | Q | Q | K | I | I | M | S | K | K | W | G | F | T | N | L | N | R | A | E | Y | I | R | R | R | D | A | G | E | V | K | D | D | G | A | F | V | K | F | L | S | 201 | | C5DX59/1-223 | 147 | I | V | I | E | G | L | R | R | S | R | Y | K | F | P | G | Q | Q | K | I | I | L | S | K | K | W | G | F | T | P | L | D | R | D | E | Y | L | K | R | R | Q | A | G | E | V | K | D | D | G | A | Y | V | K | F | L | T | 201 | | Kwal\_14.1060/1-220 | 147 | V | V | V | E | G | L | R | R | A | R | Y | K | F | P | G | Q | Q | K | I | I | M | S | K | K | W | G | F | T | N | L | N | R | A | E | Y | V | K | R | R | D | A | G | E | V | K | D | D | G | A | F | V | K | F | L | S | 201 | | Sbay\_672.56/1-221 | 147 | V | V | V | E | G | L | R | R | A | R | Y | K | F | P | G | Q | Q | K | I | I | M | S | K | K | W | G | F | T | N | L | D | R | P | D | Y | L | K | K | A | Q | A | G | E | V | K | D | D | G | S | F | V | K | F | L | S | 201 | | SAKL0B00682g/1-220 | 147 | V | V | I | E | G | L | R | R | A | R | Y | K | F | P | G | Q | Q | K | I | I | L | S | K | K | W | G | F | T | N | L | D | R | A | E | Y | V | R | R | R | D | A | G | E | V | K | D | D | G | A | F | V | K | F | L | S | 201 | | P41805/1-221 | 147 | V | V | V | E | G | L | R | R | A | R | Y | K | F | P | G | Q | Q | K | I | I | L | S | K | K | W | G | F | T | N | L | D | R | P | E | Y | L | K | K | R | E | A | G | E | V | K | D | D | G | A | F | V | K | F | L | S | 201 | |  | | G0VKH1/1-220 | 202 | K | K | G | S | L | E | D | N | F | R | E | F | P | E | Y | F | T | K | A | - | - | - |  | | | | | | | | | | | | | | | | | | | | | | | | | | | | | | | | | 220 | | Q6CRX7/1-220 | 202 | K | K | G | S | L | E | E | N | I | R | E | F | P | E | Y | F | A | K | A | - | - | - |  | | | | | | | | | | | | | | | | | | | | | | | | | | | | | | | | | 220 | | Q6FLX0/1-221 | 202 | K | K | G | S | L | E | E | N | F | R | E | F | P | D | Y | F | T | A | Q | A | - | - |  | | | | | | | | | | | | | | | | | | | | | | | | | | | | | | | | | 221 | | Q75CY7/1-240 | 221 | K | K | G | A | L | E | N | N | F | R | E | F | P | E | Y | F | T | A | Q | A | - | - |  | | | | | | | | | | | | | | | | | | | | | | | | | | | | | | | | | 240 | | A7TFN4/1-220 | 202 | K | K | G | S | L | E | D | N | F | R | E | F | P | E | Y | F | T | K | A | - | - | - |  | | | | | | | | | | | | | | | | | | | | | | | | | | | | | | | | | 220 | | C5DNR0/1-220 | 202 | K | K | G | P | L | E | E | N | V | R | E | F | P | E | Y | F | A | K | A | - | - | - |  | | | | | | | | | | | | | | | | | | | | | | | | | | | | | | | | | 220 | | C5DX59/1-223 | 202 | N | K | G | S | L | E | N | N | F | N | N | F | P | D | Y | F | L | S | R | T | Q | V |  | | | | | | | | | | | | | | | | | | | | | | | | | | | | | | | | | 223 | | Kwal\_14.1060/1-220 | 202 | K | K | G | S | L | E | H | N | V | S | E | F | P | E | Y | F | S | K | A | - | - | - |  | | | | | | | | | | | | | | | | | | | | | | | | | | | | | | | | | 220 | | Sbay\_672.56/1-221 | 202 | K | K | G | S | L | E | H | N | V | R | E | F | P | E | Y | F | A | S | Q | A | - | - |  | | | | | | | | | | | | | | | | | | | | | | | | | | | | | | | | | 221 | | SAKL0B00682g/1-220 | 202 | K | K | G | S | L | E | D | N | V | R | E | F | P | E | Y | F | T | K | A | - | - | - |  | | | | | | | | | | | | | | | | | | | | | | | | | | | | | | | | | 220 | | P41805/1-221 | 202 | K | K | G | S | L | E | N | N | I | R | E | F | P | E | Y | F | A | A | Q | A | - | - |  | | | | | | | | | | | | | | | | | | | | | | | | | | | | | | | | | 221 | |
