# Supplementary material for: Plus ça change – evolutionary sequence divergence predicts protein subcellular localization signals
Source: BMC Genomics. 2014 Jan 20;15:46. doi: 10.1186/1471-2164-15-46 (PMC3906766; doi:10.1186/1471-2164-15-46)
Supplement: Additional file 2 — MSA’s of proteins for which sequence divergence changes predicted localization signals. Contains links to ortholog multiple sequence alignments of each protein in Additional file 3: Table S1. [file 1471-2164-15-46-S2.zip › P53727.html]

|  |  |  |  |  |  |  |  |  |  |  |  |  |  |  |  |  |  |  |  |  |  |  |  |  |  |  |  |  |  |  |  |  |  |  |  |  |  |  |  |  |  |  |  |  |  |  |  |  |  |  |  |  |  |  |  |  |  |  |  |  |  |  |  |  |  |  |  |  |  |  |  |  |  |  |  |  |  |  |  |  |  |  |  |  |  |  |  |  |  |  |  |  |  |  |  |  |  |  |  |  |  |  |  |  |  |  |  |  |  |  |  |  |  |  |  |  |  |  |  |  |  |  |  |  |  |  |  |  |  |  |  |  |  |  |  |  |  |  |  |  |  |  |  |  |  |  |  |  |  |  |  |  |  |  |  |  |  |  |  |  |  |  |  |  |  |  |  |  |  |  |  |  |  |  |  |  |  |  |  |  |  |  |  |  |  |  |  |  |  |  |  |  |  |  |  |  |  |  |  |  |  |  |  |  |  |  |  |  |  |  |  |  |  |  |  |  |  |  |  |  |  |  |  |  |  |  |  |  |  |  |  |  |  |  |  |  |  |  |  |  |  |  |  |  |  |  |  |  |  |  |  |  |  |  |  |  |  |  |  |  |  |  |  |  |  |  |  |  |  |  |  |  |  |  |  |  |  |  |  |  |  |  |  |  |  |  |  |  |  |  |  |  |  |  |  |  |  |  |  |  |  |  |  |  |  |  |  |  |  |  |  |  |  |  |  |  |  |  |  |  |  |  |  |  |  |  |  |  |  |  |  |  |  |  |  |  |  |  |  |  |  |  |  |  |  |  |  |  |  |  |  |  |  |  |  |  |  |  |  |  |  |  |  |  |  |  |  |  |  |  |  |  |  |  |  |  |  |  |  |  |  |  |  |  |  |  |  |  |  |  |  |  |  |  |  |  |  |  |  |  |  |  |  |  |  |  |  |  |  |  |  |  |  |  |  |  |  |  |  |  |  |  |  |  |  |  |  |  |  |  |  |  |  |  |  |  |  |  |  |  |  |  |  |  |  |  |  |  |  |  |  |  |  |  |  |  |  |  |  |  |  |  |  |  |  |  |  |  |  |  |  |  |  |  |  |  |  |  |  |  |  |  |  |  |  |  |  |  |  |  |  |  |  |  |  |  |  |  |  |  |  |  |  |  |  |  |  |  |  |  |  |  |  |  |  |  |  |  |  |  |  |  |  |  |  |  |  |  |  |  |  |  |  |  |  |  |  |  |  |  |  |  |  |  |  |  |  |  |  |  |  |  |  |  |  |  |  |  |  |  |  |  |  |  |  |  |  |  |  |  |  |  |  |  |  |  |  |  |  |  |  |  |  |  |  |  |  |  |  |  |  |  |  |  |  |  |  |  |  |  |  |  |  |  |  |  |  |  |  |  |  |  |  |  |  |  |  |  |  |  |  |  |  |  |  |  |  |  |  |  |  |  |  |  |  |  |  |  |  |  |  |  |  |  |  |  |  |  |  |  |  |  |  |  |  |  |  |  |  |  |  |  |  |  |  |  |  |  |  |  |  |  |  |  |  |  |  |  |  |  |  |  |  |  |  |  |  |  |  |  |  |  |  |  |  |  |  |  |  |  |  |  |  |  |  |  |  |  |  |  |  |  |  |  |  |  |  |  |  |  |  |  |  |  |  |  |  |  |  |  |  |  |  |  |  |  |  |  |  |  |  |  |  |  |  |  |  |  |  |  |  |  |  |  |  |  |  |  |  |  |  |  |  |  |  |  |  |  |  |  |  |  |  |  |  |  |  |  |  |  |  |  |  |  |  |  |  |  |  |  |  |  |  |  |  |  |  |  |  |  |  |  |  |  |  |  |  |  |  |  |  |  |  |  |  |  |  |  |  |  |  |  |  |  |  |  |  |  |  |  |  |  |  |  |  |  |  |  |  |  |  |  |  |  |  |  |  |  |  |  |  |  |  |  |  |  |  |  |  |  |  |  |  |  |  |  |  |  |  |  |  |  |  |  |  |  |  |  |  |  |  |  |  |  |  |  |  |  |  |  |  |  |  |  |  |  |  |  |  |  |  |  |  |  |  |  |  |  |  |  |  |  |  |  |  |  |  |  |  |  |  |  |  |  |  |  |  |  |  |  |  |  |  |  |  |  |  |  |  |  |  |  |  |  |  |  |  |  |  |  |  |  |  |  |  |  |  |  |  |  |  |  |  |  |  |  |  |  |  |  |  |  |  |  |  |  |  |  |  |  |  |  |  |  |  |  |  |  |  |  |  |  |  |  |  |  |  |  |  |  |  |  |  |  |  |  |  |  |  |  |  |  |  |  |  |  |  |  |  |  |  |  |  |  |  |  |  |  |  |  |  |  |  |  |  |  |  |  |  |  |  |  |  |  |  |  |  |  |  |  |  |  |  |  |  |  |  |  |  |  |  |  |  |  |  |  |  |  |  |  |  |  |  |  |  |  |  |  |  |  |  |  |  |  |  |  |  |  |  |  |  |  |  |  |  |  |  |  |  |  |  |  |  |  |  |  |  |  |  |  |  |  |  |  |  |  |  |  |  |  |  |  |  |  |  |  |  |  |  |  |  |  |  |  |  |  |  |  |  |  |  |  |  |  |  |  |  |  |  |  |  |  |  |  |  |  |  |  |  |  |  |  |  |  |  |  |  |  |  |  |  |  |  |  |  |  |  |  |  |  |  |  |  |  |  |  |  |  |  |  |  |  |  |  |  |  |  |  |  |  |  |  |  |  |  |  |  |  |  |  |  |  |  |  |  |  |  |  |  |  |  |  |  |  |  |  |  |  |  |  |  |  |  |  |  |  |  |  |  |  |  |  |  |  |  |  |  |  |  |  |  |  |  |  |  |  |  |  |  |  |  |  |  |  |  |  |  |  |  |  |  |  |  |  |  |  |  |  |  |  |  |  |  |  |  |  |  |  |  |  |  |  |  |  |  |  |  |  |  |  |  |  |  |  |  |  |  |  |  |  |  |  |  |  |  |  |  |  |  |  |  |  |  |  |  |  |  |  |  |  |  |  |  |  |  |  |  |  |  |  |  |  |  |  |  |  |  |  |  |  |  |  |  |  |  |  |  |  |  |  |  |  |  |  |  |  |  |  |  |  |  |  |  |  |  |  |  |  |  |  |  |  |  |  |  |  |  |  |  |  |  |  |  |  |  |  |  |  |  |  |  |  |  |  |  |  |  |  |  |  |  |  |  |  |  |  |  |  |  |  |  |  |  |  |  |  |  |  |  |  |  |  |  |  |  |  |  |  |  |  |  |  |  |  |  |  |  |  |  |  |  |  |  |  |  |  |  |  |  |  |  |  |  |  |  |  |  |  |  |  |  |  |  |  |  |  |  |  |  |  |  |  |  |  |  |  |  |  |  |  |  |  |  |  |  |  |  |  |  |  |  |  |  |  |  |  |  |  |  |  |  |  |  |  |  |  |  |  |  |  |  |  |  |  |  |  |  |  |  |  |  |  |  |  |  |  |  |  |  |  |  |  |  |  |  |  |  |  |  |  |  |  |  |  |  |  |  |  |  |  |  |  |  |  |  |  |  |  |  |  |  |  |  |  |  |  |  |  |  |  |  |  |  |  |  |  |  |  |  |  |  |  |  |  |  |  |  |  |  |  |  |  |  |  |  |  |  |  |  |  |  |  |  |  |  |  |  |  |  |  |  |  |  |  |  |  |  |  |  |  |  |  |  |  |  |  |  |  |  |  |  |  |  |  |  |  |  |  |  |  |  |  |  |  |  |  |  |  |  |  |  |  |  |  |  |  |  |  |  |  |  |  |  |  |  |  |  |  |  |  |  |  |  |  |  |  |  |  |  |  |  |  |  |  |  |  |  |  |  |  |  |  |  |  |  |  |  |  |  |  |  |  |  |  |  |  |  |  |  |  |  |  |  |  |  |  |  |  |  |  |  |  |  |  |  |  |  |  |  |  |  |  |  |  |  |  |  |  |  |  |  |  |  |  |  |  |  |  |  |  |  |  |  |  |  |  |  |  |  |  |  |  |  |  |  |  |  |  |  |  |  |  |  |  |  |  |  |  |  |  |  |  |  |  |  |  |  |  |  |  |  |  |  |  |  |  |  |  |  |  |  |  |  |  |  |  |  |  |  |  |  |  |  |  |  |  |  |  |  |  |  |  |  |  |  |  |  |  |  |  |  |  |  |  |  |  |  |  |  |  |  |  |  |  |  |  |  |  |  |  |  |  |  |  |  |  |  |  |  |  |  |  |  |  |  |  |  |  |  |  |  |  |  |  |  |  |  |  |  |  |  |  |  |  |  |  |  |  |  |  |  |  |  |  |  |  |  |  |  |  |  |  |  |  |  |  |  |  |  |  |  |  |  |  |  |  |  |  |  |  |  |  |  |  |  |  |  |  |  |  |  |  |  |  |  |  |  |  |  |  |  |  |  |  |  |  |  |  |  |  |  |  |  |  |  |  |  |  |  |  |  |  |  |  |  |  |  |  |  |  |  |  |  |  |  |  |  |  |  |  |  |  |  |  |  |  |  |  |  |  |  |  |  |  |  |  |  |  |  |  |  |  |  |  |  |  |  |  |  |  |  |  |  |  |  |  |  |  |  |  |  |  |  |  |  |  |  |  |  |  |  |  |  |  |  |  |  |  |  |  |  |  |  |  |  |  |  |  |  |  |  |  |  |  |  |  |  |  |  |  |  |  |  |  |  |  |  |  |  |  |  |  |  |  |  |  |  |  |  |  |  |  |  |  |  |  |  |  |  |  |  |  |  |  |  |  |  |  |  |  |  |  |  |  |  |  |  |  |  |  |  |  |  |  |  |  |  |  |  |  |  |  |  |  |  |  |  |  |  |  |  |  |  |  |  |  |  |  |  |  |  |  |  |  |  |  |  |  |  |  |  |  |  |  |  |  |  |  |  |  |  |  |  |  |  |  |  |  |  |  |  |  |  |  |  |  |  |  |  |  |  |  |  |  |  |  |  |  |  |  |  |  |  |  |  |  |  |  |  |  |  |  |  |  |  |  |  |  |  |  |  |  |  |  |  |  |  |  |  |  |  |  |  |  |  |  |  |  |  |  |  |  |  |  |  |  |  |  |  |  |  |  |  |  |  |  |  |  |  |  |  |  |  |  |  |  |  |  |  |  |  |  |  |  |  |  |  |  |  |  |  |  |  |  |  |  |  |  |  |  |  |  |  |  |  |  |  |  |  |  |  |  |  |  |  |  |  |  |  |  |  |  |  |  |  |  |  |  |  |  |  |  |  |  |  |  |  |  |  |  |  |  |  |  |  |  |  |  |  |  |  |  |  |  |  |  |  |  |  |  |  |  |  |  |  |  |  |  |  |  |  |  |  |  |  |  |  |  |  |  |  |  |  |  |  |  |  |  |  |  |  |  |  |  |  |  |  |  |  |  |  |  |  |  |  |  |  |  |  |  |  |  |  |  |  |  |  |  |  |  |  |  |  |  |  |  |  |  |  |  |  |  |  |  |  |  |  |  |  |  |  |  |  |  |  |  |  |  |  |  |  |  |  |  |  |  |  |  |  |  |  |  |  |  |  |  |  |  |  |  |  |  |  |  |  |  |  |  |  |  |  |  |  |  |  |  |  |  |  |  |  |  |  |  |  |  |  |  |  |  |  |  |  |  |  |  |  |  |  |  |  |  |  |  |  |  |  |  |  |  |  |  |  |  |  |  |  |  |  |  |  |  |  |  |  |  |  |  |  |  |  |  |  |  |  |  |  |  |  |  |  |  |  |  |  |  |  |  |  |  |  |  |  |  |  |  |  |  |  |  |  |  |  |  |  |  |  |  |  |  |  |  |  |  |  |  |  |  |  |  |  |  |  |  |  |  |  |  |  |  |  |  |  |  |  |  |  |  |  |  |  |  |  |  |  |  |  |  |  |  |  |  |  |  |  |  |  |  |  |  |  |  |  |  |  |  |  |  |  |  |  |  |  |  |  |  |  |  |  |  |  |  |  |  |  |  |  |  |  |  |  |  |  |  |  |  |  |  |  |  |  |  |  |  |  |  |  |  |  |  |  |  |  |  |  |  |  |  |  |  |  |  |  |  |  |  |  |  |  |  |  |  |  |  |  |  |  |  |  |  |  |  |  |  |  |  |  |  |  |  |  |  |  |  |  |  |  |  |  |  |  |  |  |  |  |  |  |  |  |  |  |  |  |  |  |  |  |  |  |  |  |  |  |  |  |  |  |  |  |  |  |  |  |  |  |  |  |  |  |  |  |  |  |  |  |  |  |  |  |  |  |  |  |  |  |  |  |  |  |  |  |  |  |  |  |  |  |  |  |  |  |  |  |  |  |  |  |  |  |  |  |  |  |  |  |  |  |  |  |  |  |  |  |  |  |  |  |  |  |  |  |  |  |  |  |  |  |  |  |  |  |  |  |  |  |  |  |  |  |  |  |  |  |  |  |  |  |  |  |  |  |  |  |  |  |  |  |  |  |  |  |  |  |  |  |  |  |  |  |  |  |  |  |  |  |  |  |  |  |  |  |  |  |  |  |  |  |  |  |  |  |  |  |  |  |  |  |  |  |  |  |  |  |  |  |  |  |  |  |  |  |  |  |  |  |  |  |  |  |  |  |  |  |  |  |  |  |  |  |  |  |  |  |  |  |  |  |  |  |  |  |  |  |  |  |  |  |  |  |  |  |  |  |  |  |  |  |  |  |  |  |  |  |  |  |  |  |  |  |  |  |  |  |  |  |  |  |  |  |  |  |  |  |  |  |  |  |  |  |  |  |  |  |  |  |  |  |  |  |  |  |  |  |  |  |  |  |  |  |  |  |  |  |  |  |  |  |  |  |  |  |  |  |  |  |  |  |  |  |  |  |  |  |  |  |  |  |  |  |  |  |  |  |  |  |  |  |  |  |  |  |  |  |  |  |  |  |  |  |  |  |  |  |  |  |  |  |  |  |  |  |  |  |  |  |  |  |  |  |  |  |  |  |  |  |  |  |  |  |  |  |  |  |  |  |  |  |  |  |  |  |  |  |  |  |  |  |  |  |  |  |  |  |  |  |  |  |  |  |  |  |  |  |  |  |  |  |  |  |  |  |  |  |  |  |  |  |  |  |  |  |  |  |  |  |  |  |  |  |  |  |  |  |  |  |  |  |  |  |  |  |  |  |  |  |  |  |  |  |  |  |  |  |  |  |  |  |  |  |  |  |  |  |  |  |  |  |  |  |  |  |  |  |  |  |  |  |  |  |  |  |  |  |  |  |  |  |  |  |  |  |  |  |  |  |  |  |  |  |  |  |  |  |  |  |  |  |  |  |  |  |  |  |  |  |  |  |  |  |  |  |  |  |  |  |  |  |  |  |  |  |  |  |  |  |  |  |  |  |  |  |  |  |  |  |  |  |  |  |  |  |  |  |  |  |  |  |  |  |  |  |  |  |  |  |  |  |  |  |  |  |  |  |  |  |  |  |  |  |  |  |  |  |  |  |  |  |  |  |  |  |  |  |  |  |  |  |  |  |  |  |  |  |  |  |  |  |  |  |  |  |  |  |  |  |  |  |  |  |  |  |  |  |  |  |  |  |  |  |  |  |  |  |  |  |  |  |  |  |  |  |  |  |  |  |  |  |  |  |  |  |  |  |  |  |  |  |  |  |  |  |  |  |  |  |  |  |  |  |  |  |  |  |  |  |  |  |  |  |  |  |  |  |  |  |  |  |  |  |  |  |  |  |  |  |  |  |  |  |  |  |  |  |  |  |  |  |  |  |  |  |  |  |  |  |  |  |  |  |  |  |  |  |  |  |  |  |  |  |  |  |  |  |  |  |  |  |  |  |  |  |  |  |  |  |  |  |  |  |  |  |  |  |  |  |  |  |  |  |  |  |  |  |  |  |  |  |  |  |  |  |  |  |  |  |  |  |  |  |  |  |  |  |  |  |  |  |  |  |  |  |  |  |  |  |  |  |  |  |  |  |  |  |  |  |  |  |  |  |  |  |  |  |  |  |  |  |  |  |  |  |  |  |  |  |  |  |  |  |  |  |  |  |  |  |  |  |  |  |  |  |  |  |  |  |  |  |  |  |  |  |  |  |  |  |  |  |  |  |  |  |  |  |  |  |  |  |  |  |  |  |  |  |  |  |  |  |  |  |  |  |  |  |  |  |  |  |  |  |  |  |  |  |  |  |  |  |  |  |  |  |  |  |  |  |  |  |  |  |  |  |  |  |  |  |  |  |  |  |  |  |  |  |  |  |  |  |  |  |  |  |  |  |  |  |  |  |  |  |  |  |  |  |  |  |  |  |  |  |  |  |  |  |  |  |  |  |  |  |  |  |  |  |  |  |  |  |  |  |  |  |  |  |  |  |  |  |  |  |  |  |  |  |  |  |  |  |  |  |  |  |  |  |  |  |  |  |  |  |  |  |  |  |  |  |  |  |  |  |  |  |  |  |  |  |  |  |  |  |  |  |  |  |  |  |  |  |  |  |  |  |  |  |  |  |  |  |  |  |  |  |  |  |  |  |  |  |  |  |  |  |  |  |  |  |  |  |  |  |  |  |  |  |  |  |  |  |  |  |  |  |  |  |  |  |  |  |  |  |  |  |  |  |  |  |  |  |  |  |  |  |  |  |  |  |  |  |  |  |  |  |  |  |  |  |  |  |  |  |  |  |  |  |  |  |  |  |  |  |  |  |  |  |  |  |  |  |  |  |  |  |  |  |  |  |  |  |  |  |  |  |  |  |  |  |  |  |  |  |  |  |  |  |  |  |  |  |  |  |  |  |  |  |  |  |  |  |  |  |  |  |  |  |  |  |  |  |  |  |  |  |  |  |  |  |  |  |  |  |  |  |  |  |  |  |  |  |  |  |  |  |  |  |  |  |  |  |  |  |  |  |  |  |  |  |  |  |  |  |  |  |  |  |  |  |  |  |  |  |  |  |  |  |  |  |  |  |  |  |  |  |  |  |  |  |  |  |  |  |  |  |  |  |  |  |  |  |  |  |  |  |  |  |  |  |  |  |  |  |  |  |  |  |  |  |  |  |  |  |  |  |  |  |  |  |  |  |  |  |  |  |  |  |  |  |  |  |  |  |  |  |  |  |  |  |  |  |  |  |  |  |  |  |  |  |  |  |  |  |  |  |  |  |  |  |  |  |  |  |  |  |  |  |  |  |  |  |  |  |  |  |  |  |  |  |  |
| --- | --- | --- | --- | --- | --- | --- | --- | --- | --- | --- | --- | --- | --- | --- | --- | --- | --- | --- | --- | --- | --- | --- | --- | --- | --- | --- | --- | --- | --- | --- | --- | --- | --- | --- | --- | --- | --- | --- | --- | --- | --- | --- | --- | --- | --- | --- | --- | --- | --- | --- | --- | --- | --- | --- | --- | --- | --- | --- | --- | --- | --- | --- | --- | --- | --- | --- | --- | --- | --- | --- | --- | --- | --- | --- | --- | --- | --- | --- | --- | --- | --- | --- | --- | --- | --- | --- | --- | --- | --- | --- | --- | --- | --- | --- | --- | --- | --- | --- | --- | --- | --- | --- | --- | --- | --- | --- | --- | --- | --- | --- | --- | --- | --- | --- | --- | --- | --- | --- | --- | --- | --- | --- | --- | --- | --- | --- | --- | --- | --- | --- | --- | --- | --- | --- | --- | --- | --- | --- | --- | --- | --- | --- | --- | --- | --- | --- | --- | --- | --- | --- | --- | --- | --- | --- | --- | --- | --- | --- | --- | --- | --- | --- | --- | --- | --- | --- | --- | --- | --- | --- | --- | --- | --- | --- | --- | --- | --- | --- | --- | --- | --- | --- | --- | --- | --- | --- | --- | --- | --- | --- | --- | --- | --- | --- | --- | --- | --- | --- | --- | --- | --- | --- | --- | --- | --- | --- | --- | --- | --- | --- | --- | --- | --- | --- | --- | --- | --- | --- | --- | --- | --- | --- | --- | --- | --- | --- | --- | --- | --- | --- | --- | --- | --- | --- | --- | --- | --- | --- | --- | --- | --- | --- | --- | --- | --- | --- | --- | --- | --- | --- | --- | --- | --- | --- | --- | --- | --- | --- | --- | --- | --- | --- | --- | --- | --- | --- | --- | --- | --- | --- | --- | --- | --- | --- | --- | --- | --- | --- | --- | --- | --- | --- | --- | --- | --- | --- | --- | --- | --- | --- | --- | --- | --- | --- | --- | --- | --- | --- | --- | --- | --- | --- | --- | --- | --- | --- | --- | --- | --- | --- | --- | --- | --- | --- | --- | --- | --- | --- | --- | --- | --- | --- | --- | --- | --- | --- | --- | --- | --- | --- | --- | --- | --- | --- | --- | --- | --- | --- | --- | --- | --- | --- | --- | --- | --- | --- | --- | --- | --- | --- | --- | --- | --- | --- | --- | --- | --- | --- | --- | --- | --- | --- | --- | --- | --- | --- | --- | --- | --- | --- | --- | --- | --- | --- | --- | --- | --- | --- | --- | --- | --- | --- | --- | --- | --- | --- | --- | --- | --- | --- | --- | --- | --- | --- | --- | --- | --- | --- | --- | --- | --- | --- | --- | --- | --- | --- | --- | --- | --- | --- | --- | --- | --- | --- | --- | --- | --- | --- | --- | --- | --- | --- | --- | --- | --- | --- | --- | --- | --- | --- | --- | --- | --- | --- | --- | --- | --- | --- | --- | --- | --- | --- | --- | --- | --- | --- | --- | --- | --- | --- | --- | --- | --- | --- | --- | --- | --- | --- | --- | --- | --- | --- | --- | --- | --- | --- | --- | --- | --- | --- | --- | --- | --- | --- | --- | --- | --- | --- | --- | --- | --- | --- | --- | --- | --- | --- | --- | --- | --- | --- | --- | --- | --- | --- | --- | --- | --- | --- | --- | --- | --- | --- | --- | --- | --- | --- | --- | --- | --- | --- | --- | --- | --- | --- | --- | --- | --- | --- | --- | --- | --- | --- | --- | --- | --- | --- | --- | --- | --- | --- | --- | --- | --- | --- | --- | --- | --- | --- | --- | --- | --- | --- | --- | --- | --- | --- | --- | --- | --- | --- | --- | --- | --- | --- | --- | --- | --- | --- | --- | --- | --- | --- | --- | --- | --- | --- | --- | --- | --- | --- | --- | --- | --- | --- | --- | --- | --- | --- | --- | --- | --- | --- | --- | --- | --- | --- | --- | --- | --- | --- | --- | --- | --- | --- | --- | --- | --- | --- | --- | --- | --- | --- | --- | --- | --- | --- | --- | --- | --- | --- | --- | --- | --- | --- | --- | --- | --- | --- | --- | --- | --- | --- | --- | --- | --- | --- | --- | --- | --- | --- | --- | --- | --- | --- | --- | --- | --- | --- | --- | --- | --- | --- | --- | --- | --- | --- | --- | --- | --- | --- | --- | --- | --- | --- | --- | --- | --- | --- | --- | --- | --- | --- | --- | --- | --- | --- | --- | --- | --- | --- | --- | --- | --- | --- | --- | --- | --- | --- | --- | --- | --- | --- | --- | --- | --- | --- | --- | --- | --- | --- | --- | --- | --- | --- | --- | --- | --- | --- | --- | --- | --- | --- | --- | --- | --- | --- | --- | --- | --- | --- | --- | --- | --- | --- | --- | --- | --- | --- | --- | --- | --- | --- | --- | --- | --- | --- | --- | --- | --- | --- | --- | --- | --- | --- | --- | --- | --- | --- | --- | --- | --- | --- | --- | --- | --- | --- | --- | --- | --- | --- | --- | --- | --- | --- | --- | --- | --- | --- | --- | --- | --- | --- | --- | --- | --- | --- | --- | --- | --- | --- | --- | --- | --- | --- | --- | --- | --- | --- | --- | --- | --- | --- | --- | --- | --- | --- | --- | --- | --- | --- | --- | --- | --- | --- | --- | --- | --- | --- | --- | --- | --- | --- | --- | --- | --- | --- | --- | --- | --- | --- | --- | --- | --- | --- | --- | --- | --- | --- | --- | --- | --- | --- | --- | --- | --- | --- | --- | --- | --- | --- | --- | --- | --- | --- | --- | --- | --- | --- | --- | --- | --- | --- | --- | --- | --- | --- | --- | --- | --- | --- | --- | --- | --- | --- | --- | --- | --- | --- | --- | --- | --- | --- | --- | --- | --- | --- | --- | --- | --- | --- | --- | --- | --- | --- | --- | --- | --- | --- | --- | --- | --- | --- | --- | --- | --- | --- | --- | --- | --- | --- | --- | --- | --- | --- | --- | --- | --- | --- | --- | --- | --- | --- | --- | --- | --- | --- | --- | --- | --- | --- | --- | --- | --- | --- | --- | --- | --- | --- | --- | --- | --- | --- | --- | --- | --- | --- | --- | --- | --- | --- | --- | --- | --- | --- | --- | --- | --- | --- | --- | --- | --- | --- | --- | --- | --- | --- | --- | --- | --- | --- | --- | --- | --- | --- | --- | --- | --- | --- | --- | --- | --- | --- | --- | --- | --- | --- | --- | --- | --- | --- | --- | --- | --- | --- | --- | --- | --- | --- | --- | --- | --- | --- | --- | --- | --- | --- | --- | --- | --- | --- | --- | --- | --- | --- | --- | --- | --- | --- | --- | --- | --- | --- | --- | --- | --- | --- | --- | --- | --- | --- | --- | --- | --- | --- | --- | --- | --- | --- | --- | --- | --- | --- | --- | --- | --- | --- | --- | --- | --- | --- | --- | --- | --- | --- | --- | --- | --- | --- | --- | --- | --- | --- | --- | --- | --- | --- | --- | --- | --- | --- | --- | --- | --- | --- | --- | --- | --- | --- | --- | --- | --- | --- | --- | --- | --- | --- | --- | --- | --- | --- | --- | --- | --- | --- | --- | --- | --- | --- | --- | --- | --- | --- | --- | --- | --- | --- | --- | --- | --- | --- | --- | --- | --- | --- | --- | --- | --- | --- | --- | --- | --- | --- | --- | --- | --- | --- | --- | --- | --- | --- | --- | --- | --- | --- | --- | --- | --- | --- | --- | --- | --- | --- | --- | --- | --- | --- | --- | --- | --- | --- | --- | --- | --- | --- | --- | --- | --- | --- | --- | --- | --- | --- | --- | --- | --- | --- | --- | --- | --- | --- | --- | --- | --- | --- | --- | --- | --- | --- | --- | --- | --- | --- | --- | --- | --- | --- | --- | --- | --- | --- | --- | --- | --- | --- | --- | --- | --- | --- | --- | --- | --- | --- | --- | --- | --- | --- | --- | --- | --- | --- | --- | --- | --- | --- | --- | --- | --- | --- | --- | --- | --- | --- | --- | --- | --- | --- | --- | --- | --- | --- | --- | --- | --- | --- | --- | --- | --- | --- | --- | --- | --- | --- | --- | --- | --- | --- | --- | --- | --- | --- | --- | --- | --- | --- | --- | --- | --- | --- | --- | --- | --- | --- | --- | --- | --- | --- | --- | --- | --- | --- | --- | --- | --- | --- | --- | --- | --- | --- | --- | --- | --- | --- | --- | --- | --- | --- | --- | --- | --- | --- | --- | --- | --- | --- | --- | --- | --- | --- | --- | --- | --- | --- | --- | --- | --- | --- | --- | --- | --- | --- | --- | --- | --- | --- | --- | --- | --- | --- | --- | --- | --- | --- | --- | --- | --- | --- | --- | --- | --- | --- | --- | --- | --- | --- | --- | --- | --- | --- | --- | --- | --- | --- | --- | --- | --- | --- | --- | --- | --- | --- | --- | --- | --- | --- | --- | --- | --- | --- | --- | --- | --- | --- | --- | --- | --- | --- | --- | --- | --- | --- | --- | --- | --- | --- | --- | --- | --- | --- | --- | --- | --- | --- | --- | --- | --- | --- | --- | --- | --- | --- | --- | --- | --- | --- | --- | --- | --- | --- | --- | --- | --- | --- | --- | --- | --- | --- | --- | --- | --- | --- | --- | --- | --- | --- | --- | --- | --- | --- | --- | --- | --- | --- | --- | --- | --- | --- | --- | --- | --- | --- | --- | --- | --- | --- | --- | --- | --- | --- | --- | --- | --- | --- | --- | --- | --- | --- | --- | --- | --- | --- | --- | --- | --- | --- | --- | --- | --- | --- | --- | --- | --- | --- | --- | --- | --- | --- | --- | --- | --- | --- | --- | --- | --- | --- | --- | --- | --- | --- | --- | --- | --- | --- | --- | --- | --- | --- | --- | --- | --- | --- | --- | --- | --- | --- | --- | --- | --- | --- | --- | --- | --- | --- | --- | --- | --- | --- | --- | --- | --- | --- | --- | --- | --- | --- | --- | --- | --- | --- | --- | --- | --- | --- | --- | --- | --- | --- | --- | --- | --- | --- | --- | --- | --- | --- | --- | --- | --- | --- | --- | --- | --- | --- | --- | --- | --- | --- | --- | --- | --- | --- | --- | --- | --- | --- | --- | --- | --- | --- | --- | --- | --- | --- | --- | --- | --- | --- | --- | --- | --- | --- | --- | --- | --- | --- | --- | --- | --- | --- | --- | --- | --- | --- | --- | --- | --- | --- | --- | --- | --- | --- | --- | --- | --- | --- | --- | --- | --- | --- | --- | --- | --- | --- | --- | --- | --- | --- | --- | --- | --- | --- | --- | --- | --- | --- | --- | --- | --- | --- | --- | --- | --- | --- | --- | --- | --- | --- | --- | --- | --- | --- | --- | --- | --- | --- | --- | --- | --- | --- | --- | --- | --- | --- | --- | --- | --- | --- | --- | --- | --- | --- | --- | --- | --- | --- | --- | --- | --- | --- | --- | --- | --- | --- | --- | --- | --- | --- | --- | --- | --- | --- | --- | --- | --- | --- | --- | --- | --- | --- | --- | --- | --- | --- | --- | --- | --- | --- | --- | --- | --- | --- | --- | --- | --- | --- | --- | --- | --- | --- | --- | --- | --- | --- | --- | --- | --- | --- | --- | --- | --- | --- | --- | --- | --- | --- | --- | --- | --- | --- | --- | --- | --- | --- | --- | --- | --- | --- | --- | --- | --- | --- | --- | --- | --- | --- | --- | --- | --- | --- | --- | --- | --- | --- | --- | --- | --- | --- | --- | --- | --- | --- | --- | --- | --- | --- | --- | --- | --- | --- | --- | --- | --- | --- | --- | --- | --- | --- | --- | --- | --- | --- | --- | --- | --- | --- | --- | --- | --- | --- | --- | --- | --- | --- | --- | --- | --- | --- | --- | --- | --- | --- | --- | --- | --- | --- | --- | --- | --- | --- | --- | --- | --- | --- | --- | --- | --- | --- | --- | --- | --- | --- | --- | --- | --- | --- | --- | --- | --- | --- | --- | --- | --- | --- | --- | --- | --- | --- | --- | --- | --- | --- | --- | --- | --- | --- | --- | --- | --- | --- | --- | --- | --- | --- | --- | --- | --- | --- | --- | --- | --- | --- | --- | --- | --- | --- | --- | --- | --- | --- | --- | --- | --- | --- | --- | --- | --- | --- | --- | --- | --- | --- | --- | --- | --- | --- | --- | --- | --- | --- | --- | --- | --- | --- | --- | --- | --- | --- | --- | --- | --- | --- | --- | --- | --- | --- | --- | --- | --- | --- | --- | --- | --- | --- | --- | --- | --- | --- | --- | --- | --- | --- | --- | --- | --- | --- | --- | --- | --- | --- | --- | --- | --- | --- | --- | --- | --- | --- | --- | --- | --- | --- | --- | --- | --- | --- | --- | --- | --- | --- | --- | --- | --- | --- | --- | --- | --- | --- | --- | --- | --- | --- | --- | --- | --- | --- | --- | --- | --- | --- | --- | --- | --- | --- | --- | --- | --- | --- | --- | --- | --- | --- | --- | --- | --- | --- | --- | --- | --- | --- | --- | --- | --- | --- | --- | --- | --- | --- | --- | --- | --- | --- | --- | --- | --- | --- | --- | --- | --- | --- | --- | --- | --- | --- | --- | --- | --- | --- | --- | --- | --- | --- | --- | --- | --- | --- | --- | --- | --- | --- | --- | --- | --- | --- | --- | --- | --- | --- | --- | --- | --- | --- | --- | --- | --- | --- | --- | --- | --- | --- | --- | --- | --- | --- | --- | --- | --- | --- | --- | --- | --- | --- | --- | --- | --- | --- | --- | --- | --- | --- | --- | --- | --- | --- | --- | --- | --- | --- | --- | --- | --- | --- | --- | --- | --- | --- | --- | --- | --- | --- | --- | --- | --- | --- | --- | --- | --- | --- | --- | --- | --- | --- | --- | --- | --- | --- | --- | --- | --- | --- | --- | --- | --- | --- | --- | --- | --- | --- | --- | --- | --- | --- | --- | --- | --- | --- | --- | --- | --- | --- | --- | --- | --- | --- | --- | --- | --- | --- | --- | --- | --- | --- | --- | --- | --- | --- | --- | --- | --- | --- | --- | --- | --- | --- | --- | --- | --- | --- | --- | --- | --- | --- | --- | --- | --- | --- | --- | --- | --- | --- | --- | --- | --- | --- | --- | --- | --- | --- | --- | --- | --- | --- | --- | --- | --- | --- | --- | --- | --- | --- | --- | --- | --- | --- | --- | --- | --- | --- | --- | --- | --- | --- | --- | --- | --- | --- | --- | --- | --- | --- | --- | --- | --- | --- | --- | --- | --- | --- | --- | --- | --- | --- | --- | --- | --- | --- | --- | --- | --- | --- | --- | --- | --- | --- | --- | --- | --- | --- | --- | --- | --- | --- | --- | --- | --- | --- | --- | --- | --- | --- | --- | --- | --- | --- | --- | --- | --- | --- | --- | --- | --- | --- | --- | --- | --- | --- | --- | --- | --- | --- | --- | --- | --- | --- | --- | --- | --- | --- | --- | --- | --- | --- | --- | --- | --- | --- | --- | --- | --- | --- | --- | --- | --- | --- | --- | --- | --- | --- | --- | --- | --- | --- | --- | --- | --- | --- | --- | --- | --- | --- | --- | --- | --- | --- | --- | --- | --- | --- | --- | --- | --- | --- | --- | --- | --- | --- | --- | --- | --- | --- | --- | --- | --- | --- | --- | --- | --- | --- | --- | --- | --- | --- | --- | --- | --- | --- | --- | --- | --- | --- | --- | --- | --- | --- | --- | --- | --- | --- | --- | --- | --- | --- | --- | --- | --- | --- | --- | --- | --- | --- | --- | --- | --- | --- | --- | --- | --- | --- | --- | --- | --- | --- | --- | --- | --- | --- | --- | --- | --- | --- | --- | --- | --- | --- | --- | --- | --- | --- | --- | --- | --- | --- | --- | --- | --- | --- | --- | --- | --- | --- | --- | --- | --- | --- | --- | --- | --- | --- | --- | --- | --- | --- | --- | --- | --- | --- | --- | --- | --- | --- | --- | --- | --- | --- | --- | --- | --- | --- | --- | --- | --- | --- | --- | --- | --- | --- | --- | --- | --- | --- | --- | --- | --- | --- | --- | --- | --- | --- | --- | --- | --- | --- | --- | --- | --- | --- | --- | --- | --- | --- | --- | --- | --- | --- | --- | --- | --- | --- | --- | --- | --- | --- | --- | --- | --- | --- | --- | --- | --- | --- | --- | --- | --- | --- | --- | --- | --- | --- | --- | --- | --- | --- | --- | --- | --- | --- | --- | --- | --- | --- | --- | --- | --- | --- | --- | --- | --- | --- | --- | --- | --- | --- | --- | --- | --- | --- | --- | --- | --- | --- | --- | --- | --- | --- | --- | --- | --- | --- | --- | --- | --- | --- | --- | --- | --- | --- | --- | --- | --- | --- | --- | --- | --- | --- | --- | --- | --- | --- | --- | --- | --- | --- | --- | --- | --- | --- | --- | --- | --- | --- | --- | --- | --- | --- | --- | --- | --- | --- | --- | --- | --- | --- | --- | --- | --- | --- | --- | --- | --- | --- | --- | --- | --- | --- | --- | --- | --- | --- | --- | --- | --- | --- | --- | --- | --- | --- | --- | --- | --- | --- | --- | --- | --- | --- | --- | --- | --- | --- | --- | --- | --- | --- | --- | --- | --- | --- | --- | --- | --- | --- | --- | --- | --- | --- | --- | --- | --- | --- | --- | --- | --- | --- | --- | --- | --- | --- | --- | --- | --- | --- | --- | --- | --- | --- | --- | --- | --- | --- | --- | --- | --- | --- | --- | --- | --- | --- | --- | --- | --- | --- | --- | --- | --- | --- | --- | --- | --- | --- | --- | --- | --- | --- | --- | --- | --- | --- | --- | --- | --- | --- | --- | --- | --- | --- | --- | --- | --- | --- | --- | --- | --- | --- | --- | --- | --- | --- | --- | --- | --- | --- | --- | --- | --- | --- | --- | --- | --- | --- | --- | --- | --- | --- | --- | --- | --- | --- | --- | --- | --- | --- | --- | --- | --- | --- | --- | --- | --- | --- | --- | --- | --- | --- | --- | --- | --- | --- | --- | --- | --- | --- | --- | --- | --- | --- | --- | --- | --- | --- | --- | --- | --- | --- | --- | --- | --- | --- | --- | --- | --- | --- | --- | --- | --- | --- | --- | --- | --- | --- | --- | --- | --- | --- | --- | --- | --- | --- | --- | --- | --- | --- | --- | --- | --- | --- | --- | --- | --- | --- | --- | --- | --- | --- | --- | --- | --- | --- | --- | --- | --- | --- | --- | --- | --- | --- | --- | --- | --- | --- | --- | --- | --- | --- | --- | --- | --- | --- | --- | --- | --- | --- | --- | --- | --- | --- | --- | --- | --- | --- | --- | --- | --- | --- | --- | --- | --- | --- | --- | --- | --- | --- | --- | --- | --- | --- | --- | --- | --- | --- | --- | --- | --- | --- | --- | --- | --- | --- | --- | --- | --- | --- | --- | --- | --- | --- | --- | --- | --- | --- | --- | --- | --- | --- | --- | --- | --- | --- | --- | --- | --- | --- | --- | --- | --- | --- | --- | --- | --- | --- | --- | --- | --- | --- | --- | --- | --- | --- | --- | --- | --- | --- | --- | --- | --- | --- | --- | --- | --- | --- | --- | --- | --- | --- | --- | --- | --- | --- | --- | --- | --- | --- | --- | --- | --- | --- | --- | --- | --- | --- | --- | --- | --- | --- | --- | --- | --- | --- | --- | --- | --- | --- | --- | --- | --- | --- | --- | --- | --- | --- | --- | --- | --- | --- | --- | --- | --- | --- | --- | --- | --- | --- | --- | --- | --- | --- | --- | --- | --- | --- | --- | --- | --- | --- | --- | --- | --- | --- | --- | --- | --- | --- | --- | --- | --- | --- | --- | --- | --- | --- | --- | --- | --- | --- | --- | --- | --- | --- | --- | --- | --- | --- | --- | --- | --- | --- | --- | --- | --- | --- | --- | --- | --- | --- | --- | --- | --- | --- | --- | --- | --- | --- | --- | --- | --- | --- | --- | --- | --- | --- | --- | --- | --- | --- | --- | --- | --- | --- | --- | --- | --- | --- | --- | --- | --- | --- | --- | --- | --- | --- | --- | --- | --- | --- | --- | --- | --- | --- | --- | --- | --- | --- | --- | --- | --- | --- | --- | --- | --- | --- | --- | --- | --- | --- | --- | --- | --- | --- | --- | --- | --- | --- | --- | --- | --- | --- | --- | --- | --- | --- | --- | --- | --- | --- | --- | --- | --- | --- | --- | --- | --- | --- | --- | --- | --- | --- | --- | --- | --- | --- | --- | --- | --- | --- | --- | --- | --- | --- | --- | --- | --- | --- | --- | --- | --- | --- | --- | --- | --- | --- | --- | --- | --- | --- | --- | --- | --- | --- | --- | --- | --- | --- | --- | --- | --- | --- | --- | --- | --- | --- | --- | --- | --- | --- | --- | --- | --- | --- | --- | --- | --- | --- | --- | --- | --- | --- | --- | --- | --- | --- | --- | --- | --- | --- | --- | --- | --- | --- | --- | --- | --- | --- | --- | --- | --- | --- | --- | --- | --- | --- | --- | --- | --- | --- | --- | --- | --- | --- | --- | --- | --- | --- | --- | --- | --- | --- | --- | --- | --- | --- | --- | --- | --- | --- | --- | --- | --- | --- | --- | --- | --- | --- | --- | --- | --- | --- | --- | --- | --- | --- | --- | --- | --- | --- | --- | --- | --- | --- | --- | --- | --- | --- | --- | --- | --- | --- | --- | --- | --- | --- | --- | --- | --- | --- | --- | --- | --- | --- | --- | --- | --- | --- | --- | --- | --- | --- | --- | --- | --- | --- | --- | --- | --- | --- | --- | --- | --- | --- | --- | --- | --- | --- | --- | --- | --- | --- | --- | --- | --- | --- | --- | --- | --- | --- | --- | --- | --- | --- | --- | --- | --- | --- | --- | --- | --- | --- | --- | --- | --- | --- | --- | --- | --- | --- | --- | --- | --- | --- | --- | --- | --- | --- | --- | --- | --- | --- | --- | --- | --- | --- | --- | --- | --- | --- | --- | --- | --- | --- | --- | --- | --- | --- | --- | --- | --- | --- | --- | --- | --- | --- | --- | --- | --- | --- | --- | --- | --- | --- | --- | --- | --- | --- | --- | --- | --- | --- | --- | --- | --- | --- | --- | --- | --- | --- | --- | --- | --- | --- | --- | --- | --- | --- | --- | --- | --- | --- | --- | --- | --- | --- | --- | --- | --- | --- | --- | --- | --- | --- | --- | --- | --- | --- | --- | --- | --- | --- | --- | --- | --- | --- | --- | --- | --- | --- | --- | --- | --- | --- | --- | --- | --- | --- | --- | --- | --- | --- | --- | --- | --- | --- | --- | --- | --- | --- | --- | --- | --- | --- | --- | --- | --- | --- | --- | --- | --- | --- | --- | --- | --- | --- | --- | --- | --- | --- | --- | --- | --- | --- | --- | --- | --- | --- | --- | --- | --- | --- | --- | --- | --- | --- | --- | --- | --- | --- | --- | --- | --- | --- | --- | --- | --- | --- | --- | --- | --- | --- | --- | --- | --- | --- | --- | --- | --- | --- | --- | --- | --- | --- | --- | --- | --- | --- | --- | --- | --- | --- | --- | --- | --- | --- | --- | --- | --- | --- | --- | --- | --- | --- | --- | --- | --- | --- | --- | --- | --- | --- | --- | --- | --- | --- | --- | --- | --- | --- | --- | --- | --- | --- | --- | --- | --- | --- | --- | --- | --- | --- | --- | --- | --- | --- | --- | --- | --- | --- | --- | --- | --- | --- | --- | --- | --- | --- | --- | --- | --- | --- | --- | --- | --- | --- | --- | --- | --- | --- | --- | --- | --- | --- | --- | --- | --- | --- | --- | --- | --- | --- | --- | --- | --- | --- | --- | --- | --- | --- | --- | --- | --- | --- | --- | --- | --- | --- | --- | --- | --- | --- | --- | --- | --- | --- | --- | --- | --- | --- | --- | --- | --- | --- | --- | --- | --- | --- | --- | --- | --- | --- | --- | --- | --- | --- | --- | --- | --- | --- | --- | --- | --- | --- | --- | --- | --- | --- | --- | --- | --- | --- | --- | --- | --- | --- | --- | --- | --- | --- | --- | --- | --- | --- | --- | --- | --- | --- | --- | --- | --- | --- | --- | --- | --- | --- | --- | --- | --- | --- | --- | --- | --- | --- | --- | --- | --- | --- | --- | --- | --- | --- | --- | --- | --- | --- | --- | --- | --- | --- | --- | --- | --- | --- | --- | --- | --- | --- | --- | --- | --- | --- | --- | --- | --- | --- | --- | --- | --- | --- | --- | --- | --- | --- | --- | --- | --- | --- | --- | --- | --- | --- | --- | --- | --- | --- | --- | --- | --- | --- | --- | --- | --- | --- | --- | --- | --- | --- | --- | --- | --- | --- | --- | --- | --- | --- | --- | --- | --- | --- | --- | --- | --- | --- | --- | --- | --- | --- | --- | --- | --- | --- | --- | --- | --- | --- | --- | --- | --- | --- | --- | --- | --- | --- | --- | --- | --- | --- | --- | --- | --- | --- | --- | --- | --- | --- | --- | --- | --- | --- | --- | --- | --- | --- | --- | --- | --- | --- | --- | --- | --- | --- | --- | --- | --- | --- | --- | --- | --- | --- | --- | --- | --- | --- | --- | --- | --- | --- | --- | --- | --- | --- | --- | --- | --- | --- | --- | --- | --- | --- | --- | --- | --- | --- | --- | --- | --- | --- | --- | --- | --- | --- | --- | --- | --- | --- | --- | --- | --- | --- | --- | --- | --- | --- | --- | --- | --- | --- | --- | --- | --- | --- | --- | --- | --- | --- | --- | --- | --- | --- | --- | --- | --- | --- | --- | --- | --- | --- | --- | --- | --- | --- | --- | --- | --- | --- | --- | --- | --- | --- | --- | --- | --- | --- | --- | --- | --- | --- | --- | --- | --- | --- | --- | --- | --- | --- | --- | --- | --- | --- | --- | --- | --- | --- | --- | --- | --- | --- | --- | --- | --- | --- | --- | --- | --- | --- | --- | --- | --- | --- | --- | --- | --- | --- | --- | --- | --- | --- | --- | --- | --- | --- | --- | --- | --- | --- | --- | --- | --- | --- | --- | --- | --- | --- | --- | --- | --- | --- | --- | --- | --- | --- | --- | --- | --- | --- | --- | --- | --- | --- | --- | --- | --- | --- | --- | --- | --- | --- | --- | --- | --- | --- | --- | --- | --- | --- | --- | --- | --- | --- | --- | --- | --- | --- | --- | --- | --- | --- | --- | --- | --- | --- | --- | --- | --- | --- | --- | --- | --- | --- | --- | --- | --- | --- | --- | --- | --- | --- | --- | --- | --- | --- | --- | --- | --- | --- | --- | --- | --- | --- | --- | --- | --- | --- | --- | --- | --- | --- | --- | --- | --- | --- | --- | --- | --- | --- | --- | --- | --- | --- | --- | --- | --- | --- | --- | --- | --- | --- | --- | --- | --- | --- | --- | --- | --- | --- | --- | --- | --- | --- | --- | --- | --- | --- | --- | --- | --- | --- | --- | --- | --- | --- | --- | --- | --- | --- |
| |  |  |  |  |  |  |  |  |  |  |  |  |  |  |  |  |  |  |  |  |  |  |  |  |  |  |  |  |  |  |  |  |  |  |  |  |  |  |  |  |  |  |  |  |  |  |  |  |  |  |  |  |  |  |  |  |  |  | | --- | --- | --- | --- | --- | --- | --- | --- | --- | --- | --- | --- | --- | --- | --- | --- | --- | --- | --- | --- | --- | --- | --- | --- | --- | --- | --- | --- | --- | --- | --- | --- | --- | --- | --- | --- | --- | --- | --- | --- | --- | --- | --- | --- | --- | --- | --- | --- | --- | --- | --- | --- | --- | --- | --- | --- | --- | --- | | G0V6B9/1-329 | 1 | - | - | - | - | - | - | - | - | - | M | D | Y | T | S | P | I | K | C | R | K | V | L | S | I | Q | S | H | V | I | H | G | Y | V | G | N | K | A | A | T | F | P | L | Q | Y | R | G | W | D | V | D | V | L | N | T | V | 46 | | Q6CU01/1-304 | 1 | - | - | - | - | - | - | - | - | - | - | - | - | - | - | M | V | S | G | K | K | V | L | S | I | Q | S | H | V | V | H | G | Y | V | G | N | K | A | A | T | F | P | L | Q | C | K | G | W | D | V | D | A | L | N | T | V | 41 | | Q6FIY1/1-323 | 1 | - | - | - | - | M | T | G | E | G | S | V | P | G | G | V | S | V | V | G | K | V | L | A | I | Q | S | H | V | V | H | G | Y | V | G | N | R | A | A | T | F | P | L | Q | Y | R | G | W | D | V | D | A | L | N | T | V | 51 | | A7TG14/1-342 | 1 | M | A | V | E | L | S | E | K | L | N | L | D | N | H | L | I | R | T | K | K | V | L | S | I | Q | S | H | V | V | H | G | Y | V | G | N | K | A | S | T | F | S | L | Q | Y | K | G | W | D | V | D | A | L | N | T | V | 55 | | C5DBZ5/1-305 | 1 | - | - | - | - | - | - | - | - | - | - | - | - | - | - | - | - | M | A | R | K | V | L | S | I | Q | S | H | V | V | H | G | Y | V | G | N | K | A | A | T | F | P | L | Q | Y | R | G | W | D | V | D | A | L | N | T | V | 39 | | C5DVP5/1-315 | 1 | - | - | - | - | - | M | V | F | N | R | D | A | M | T | R | I | S | T | R | K | V | L | S | I | Q | S | H | V | V | H | G | Y | V | G | N | K | A | A | T | F | P | L | Q | Y | Q | G | W | D | V | D | A | L | N | T | V | 50 | | Kwal\_23.6296/1-308 | 1 | - | - | - | - | - | - | - | - | - | - | - | - | - | - | - | - | M | A | R | K | V | L | S | I | Q | S | H | V | V | H | G | Y | V | G | N | K | A | A | T | F | P | L | Q | Y | R | G | W | D | V | D | A | L | N | T | V | 39 | | Sbay\_655.42/1-339 | 1 | - | - | - | - | - | - | - | - | - | - | M | T | S | T | L | H | T | T | K | K | V | L | S | I | Q | S | H | V | I | H | G | Y | V | G | N | K | A | A | T | F | P | L | Q | Y | R | G | W | D | V | D | V | L | N | T | V | 45 | | SAKL0A01430g/1-302 | 1 | - | - | - | - | - | - | - | - | - | - | - | - | - | - | - | - | M | V | K | K | V | L | S | I | Q | S | H | V | V | H | G | Y | V | G | N | K | A | A | T | F | P | L | Q | Y | R | G | W | D | V | D | A | L | N | T | V | 39 | | P53727/1-317 | 1 | - | - | - | - | - | - | - | - | - | - | M | T | S | T | L | H | T | T | K | K | V | L | S | I | Q | S | H | V | I | H | G | Y | V | G | N | K | A | A | T | F | P | L | Q | Y | R | G | W | D | V | D | V | L | N | T | V | 45 | |  | | G0V6B9/1-329 | 47 | Q | F | S | N | H | P | G | Y | E | T | F | T | G | Y | K | Y | D | P | K | T | L | Q | D | I | V | E | N | G | L | V | D | S | L | H | I | D | Y | D | A | V | L | T | G | Y | L | P | S | V | E | N | L | Q | N | L | A | 101 | | Q6CU01/1-304 | 42 | Q | F | S | N | H | P | A | Y | G | F | L | S | G | F | K | S | R | S | E | D | L | E | R | I | I | Q | D | G | L | L | S | G | L | K | I | H | Y | D | A | V | L | T | G | Y | L | P | D | T | Q | G | L | K | K | I | G | 96 | | Q6FIY1/1-323 | 52 | Q | Y | S | N | H | L | G | Y | G | Q | A | T | G | F | K | Y | S | G | E | E | L | C | S | V | F | R | D | G | L | L | K | A | M | G | N | R | Y | D | A | I | I | T | G | Y | T | P | S | A | E | V | L | E | D | I | S | 106 | | A7TG14/1-342 | 56 | Q | Y | S | N | H | P | G | Y | G | Q | F | S | G | F | K | T | D | S | K | D | I | S | N | I | F | Q | Q | G | L | I | S | G | L | E | I | Q | Y | D | A | I | I | T | G | Y | I | P | D | I | K | S | L | E | F | L | G | 110 | | C5DBZ5/1-305 | 40 | Q | F | S | N | H | P | G | Y | G | H | F | K | G | F | R | S | E | A | S | D | L | Q | G | I | I | E | K | G | L | L | G | G | L | D | I | Q | Y | D | A | V | L | T | G | Y | L | P | D | I | E | G | L | R | A | I | G | 94 | | C5DVP5/1-315 | 51 | Q | F | S | N | H | P | G | Y | G | H | F | T | G | F | R | Y | D | A | G | H | L | C | E | I | L | E | Q | G | L | A | K | S | L | E | I | Q | Y | D | A | V | L | M | G | Y | L | P | G | V | E | S | L | R | K | I | G | 105 | | Kwal\_23.6296/1-308 | 40 | Q | F | S | N | H | P | G | Y | G | H | F | T | G | Y | R | S | Q | A | T | E | L | S | N | I | L | E | K | G | L | I | G | G | L | Q | I | R | Y | D | A | V | L | T | G | Y | V | P | D | V | A | G | L | K | A | I | A | 94 | | Sbay\_655.42/1-339 | 46 | Q | F | S | N | H | P | G | Y | A | H | F | T | G | F | K | C | S | T | E | E | L | V | E | I | V | E | K | G | L | V | G | S | L | H | I | K | Y | D | A | V | L | S | G | Y | L | P | N | V | Q | A | L | Q | K | M | A | 100 | | SAKL0A01430g/1-302 | 40 | Q | F | S | N | H | P | A | Y | G | H | F | T | G | F | K | S | K | A | Q | E | L | R | D | I | I | E | K | G | L | L | Q | G | L | E | L | K | Y | D | A | V | L | T | G | Y | L | P | D | V | D | G | L | K | A | I | S | 94 | | P53727/1-317 | 46 | Q | F | S | N | H | S | G | Y | A | H | F | T | G | F | K | C | S | T | E | E | L | V | D | I | V | E | K | G | L | I | G | S | L | R | I | K | Y | D | A | V | L | S | G | Y | L | P | N | V | Q | A | L | Q | K | V | A | 100 | |  | | G0V6B9/1-329 | 102 | H | I | I | N | K | M | K | E | T | N | K | H | L | K | W | I | L | D | P | V | L | G | D | N | G | R | L | Y | V | S | G | D | N | V | P | A | Y | K | T | L | L | R | E | N | T | I | Y | L | V | T | P | N | Q | F | E | 156 | | Q6CU01/1-304 | 97 | A | L | L | V | K | L | C | N | D | D | P | S | L | K | W | I | L | D | P | V | L | G | D | N | G | K | L | Y | V | P | E | D | T | V | D | I | Y | K | Q | I | L | K | D | G | S | V | Y | L | A | T | P | N | Q | F | E | 151 | | Q6FIY1/1-323 | 107 | G | I | I | K | N | Q | L | N | Q | Q | Q | D | L | K | W | I | V | D | P | V | L | G | D | N | G | R | L | Y | V | S | E | D | I | V | P | V | Y | K | R | L | L | S | Q | N | K | I | F | L | A | T | P | N | Q | F | E | 161 | | A7TG14/1-342 | 111 | E | E | I | S | S | L | R | E | I | C | D | T | L | K | W | I | L | D | P | V | L | G | D | N | G | K | M | Y | L | A | E | G | I | K | T | T | Y | K | N | I | L | S | S | S | K | I | Y | L | T | T | P | N | Q | F | E | 165 | | C5DBZ5/1-305 | 95 | K | T | L | I | K | L | C | E | K | D | H | R | V | K | W | V | L | D | P | V | L | G | D | N | G | K | L | Y | V | P | E | E | A | I | P | V | Y | R | D | I | L | T | H | G | A | V | Y | L | A | T | P | N | Q | F | E | 149 | | C5DVP5/1-315 | 106 | E | A | V | G | E | M | S | A | R | D | P | D | L | K | W | V | L | D | P | V | L | G | D | N | G | K | L | Y | V | S | G | E | N | V | D | A | Y | K | Q | I | L | R | H | N | K | I | H | L | V | T | P | N | Q | F | E | 160 | | Kwal\_23.6296/1-308 | 95 | Q | T | L | T | N | M | C | E | K | D | R | R | V | K | W | I | L | D | P | V | L | G | D | N | G | K | L | Y | V | A | E | E | A | V | T | V | Y | R | E | I | L | T | N | G | A | V | Y | L | A | T | P | N | Q | F | E | 149 | | Sbay\_655.42/1-339 | 101 | G | I | V | G | R | M | C | E | E | D | D | N | V | K | W | V | L | D | P | V | L | G | D | N | G | K | L | Y | V | E | E | Q | C | V | A | V | Y | Q | D | I | M | H | R | S | K | I | F | L | A | T | P | N | Q | F | E | 155 | | SAKL0A01430g/1-302 | 95 | E | T | V | S | R | L | C | E | S | D | T | S | I | K | W | V | V | D | P | V | L | G | D | N | G | K | L | Y | V | P | E | E | T | V | P | I | Y | R | K | I | L | E | C | G | S | V | F | L | T | T | P | N | Q | F | E | 149 | | P53727/1-317 | 101 | G | I | V | G | Q | L | C | E | G | S | E | N | V | K | W | I | L | D | P | V | L | G | D | N | G | R | L | Y | V | D | R | E | C | V | A | V | Y | Q | D | I | L | Q | N | F | K | I | F | L | A | T | P | N | Q | F | E | 155 | |  | | G0V6B9/1-329 | 157 | M | E | T | L | T | E | I | T | I | S | S | L | E | T | L | R | R | S | F | Q | E | Y | H | R | L | Y | P | K | T | E | R | I | V | V | T | S | L | E | L | N | S | D | D | - | - | - | - | - | - | S | T | Y | I | V | A | 205 | | Q6CU01/1-304 | 152 | L | E | V | L | T | G | T | V | I | A | D | L | N | S | L | K | N | A | L | N | K | F | H | V | L | Y | P | K | V | R | Y | L | V | V | T | S | V | N | W | P | S | S | A | D | D | - | - | - | - | D | S | F | V | S | A | 202 | | Q6FIY1/1-323 | 162 | M | E | L | L | S | E | S | E | L | T | D | L | E | S | A | S | T | A | V | S | K | F | F | Q | L | Y | P | H | V | E | R | L | V | V | T | S | V | V | L | A | G | S | D | - | - | - | - | - | - | D | Y | V | V | I | A | 210 | | A7TG14/1-342 | 166 | M | E | M | L | T | S | M | K | I | N | D | I | S | S | L | R | Q | A | F | V | I | F | H | E | L | Y | P | R | V | E | N | I | V | V | T | G | I | E | I | N | Y | R | E | - | - | - | - | - | - | N | G | Y | I | T | A | 214 | | C5DBZ5/1-305 | 150 | M | E | V | L | T | G | V | Q | I | C | D | L | A | S | L | K | Q | S | I | Q | Q | F | H | E | L | Y | P | R | V | Q | N | V | V | V | T | S | V | S | F | H | S | A | G | D | S | - | - | - | I | S | E | L | I | C | A | 201 | | C5DVP5/1-315 | 161 | M | E | T | L | T | G | V | K | I | Q | D | L | E | S | L | K | S | S | I | E | Q | F | Q | K | L | Y | P | R | V | N | K | I | V | V | T | S | L | E | L | K | - | - | - | - | - | - | - | - | - | N | G | Y | I | C | A | 206 | | Kwal\_23.6296/1-308 | 150 | M | E | V | L | T | D | V | K | I | K | D | L | Q | T | L | K | T | S | I | T | R | F | H | T | L | Y | P | R | V | Q | Y | V | V | I | T | S | M | D | F | A | A | H | E | G | Q | N | N | V | F | S | E | L | I | C | A | 204 | | Sbay\_655.42/1-339 | 156 | M | E | L | L | V | G | M | S | I | R | T | L | D | D | A | K | R | G | F | E | Q | F | H | E | K | F | P | R | V | T | R | V | V | V | T | S | L | E | L | S | D | F | S | N | D | - | - | - | - | N | V | Y | V | V | A | 206 | | SAKL0A01430g/1-302 | 150 | M | E | T | L | T | D | V | K | I | T | D | C | A | S | L | R | S | S | L | I | K | F | H | Q | L | Y | P | T | V | K | Y | V | I | V | T | S | V | V | L | P | N | L | S | - | - | - | - | - | - | D | H | Y | I | T | A | 198 | | P53727/1-317 | 156 | M | E | L | L | V | G | M | S | I | R | T | L | D | D | A | K | Q | A | F | K | L | F | H | K | K | Y | P | R | V | S | R | I | V | V | T | S | L | E | L | S | E | F | L | S | N | - | - | - | - | D | T | Y | V | V | A | 206 | |  | | G0V6B9/1-329 | 206 | C | C | - | - | - | - | - | - | - | - | - | D | T | T | N | K | D | D | D | I | W | F | F | E | V | P | K | I | K | A | H | F | N | G | S | G | D | L | F | A | A | L | L | L | D | I | L | V | P | S | R | S | L | E | V | 251 | | Q6CU01/1-304 | 203 | C | T | - | - | - | - | - | - | - | - | - | - | - | - | - | D | F | T | E | Y | W | Y | F | N | I | P | K | I | N | A | H | F | S | G | S | G | D | L | F | S | A | I | I | M | D | L | L | L | S | S | E | - | - | - | - | 240 | | Q6FIY1/1-323 | 211 | A | D | - | - | - | - | - | - | - | - | - | - | R | T | T | S | P | Q | D | T | I | Y | I | R | S | P | R | I | K | C | H | F | S | G | S | G | D | L | F | T | A | L | L | V | D | A | L | L | R | D | R | E | - | - | - | 252 | | A7TG14/1-342 | 215 | A | C | Y | A | G | D | E | F | T | T | T | N | N | T | S | N | Q | L | H | I | S | G | Y | V | V | P | K | I | P | A | Q | F | S | G | S | G | D | L | F | T | S | L | I | M | N | E | M | I | T | K | S | N | I | A | S | 269 | | C5DBZ5/1-305 | 202 | C | S | - | - | - | - | - | - | - | - | - | - | - | E | Q | E | S | R | R | A | H | Y | F | K | V | P | K | I | D | A | Q | F | S | G | S | G | D | L | C | S | A | L | L | L | D | S | F | L | T | Q | N | P | A | - | - | 243 | | C5DVP5/1-315 | 207 | C | C | - | - | - | - | - | - | - | - | - | - | - | - | - | D | G | G | K | I | Q | Y | A | S | V | P | R | I | N | A | H | F | S | G | T | G | D | L | F | S | A | L | L | L | N | A | L | V | P | P | A | G | - | - | - | 245 | | Kwal\_23.6296/1-308 | 205 | C | S | - | - | - | - | - | - | - | - | - | - | - | E | L | N | S | K | R | A | H | Y | F | V | V | P | K | I | E | A | Q | F | S | G | S | G | D | L | C | S | A | L | L | L | D | V | F | L | S | Q | D | S | E | - | - | 246 | | Sbay\_655.42/1-339 | 207 | G | F | - | - | - | - | - | - | - | - | - | - | D | S | S | L | S | D | E | V | F | F | Y | K | I | P | K | I | N | A | T | F | S | G | S | G | D | L | I | S | A | L | L | T | D | S | L | L | N | G | H | V | - | - | - | 248 | | SAKL0A01430g/1-302 | 199 | C | S | - | - | - | - | - | - | - | - | - | - | - | D | T | E | T | E | T | F | H | Y | F | Q | V | P | K | I | D | A | L | F | S | G | S | G | D | L | F | S | A | L | V | M | D | A | V | L | D | G | T | - | - | - | - | 238 | | P53727/1-317 | 207 | G | F | - | - | - | - | - | - | - | - | - | - | D | C | S | A | S | E | E | I | F | F | Y | E | I | P | K | I | N | A | K | F | S | G | S | G | D | L | I | S | A | M | L | T | D | S | L | L | G | D | R | R | - | - | - | 248 | |  | | G0V6B9/1-329 | 252 | N | Y | P | S | N | H | I | P | L | H | E | A | L | G | Q | T | L | W | L | V | N | E | V | L | E | R | T | F | E | L | A | R | P | L | L | V | N | G | E | N | V | D | Q | E | I | P | K | I | K | D | L | R | L | I | Q | 306 | | Q6CU01/1-304 | 241 | - | - | - | - | - | T | V | E | L | P | L | A | L | N | S | A | L | S | L | V | D | G | V | L | R | R | T | Y | D | L | T | S | K | P | A | Q | S | - | - | - | - | D | D | T | P | F | K | I | N | D | L | K | I | I | Q | 286 | | Q6FIY1/1-323 | 253 | - | - | - | - | - | S | T | K | L | S | Q | A | V | A | K | S | Q | W | M | I | G | S | V | L | Q | R | T | Y | E | Q | A | - | - | - | L | K | S | G | E | L | K | D | Q | D | S | P | V | I | K | D | L | K | L | I | Q | 299 | | A7TG14/1-342 | 270 | T | D | P | - | - | N | E | T | L | S | R | K | L | D | I | A | L | N | Q | T | Q | A | I | L | Q | R | T | Y | N | H | F | N | N | R | I | V | D | G | D | I | N | N | N | S | T | L | K | I | N | D | L | R | L | I | Q | 322 | | C5DBZ5/1-305 | 244 | - | - | - | - | - | A | R | D | L | C | A | A | L | N | Q | V | L | S | L | V | D | S | I | L | L | N | T | F | E | L | Y | K | A | S | I | A | A | - | - | - | - | - | - | P | P | L | K | I | N | D | L | R | L | I | E | 287 | | C5DVP5/1-315 | 246 | - | - | - | Q | T | P | P | T | L | A | Q | A | L | L | L | V | I | S | L | V | D | L | I | L | R | R | T | L | E | L | S | L | S | K | D | - | - | - | - | - | - | D | V | L | P | V | T | I | N | D | L | K | L | I | Q | 291 | | Kwal\_23.6296/1-308 | 247 | - | - | - | - | - | A | R | D | L | S | V | A | L | N | K | V | L | S | L | I | D | S | I | L | R | K | T | F | E | I | H | Q | T | T | V | G | S | - | - | - | - | - | - | H | Q | V | K | I | N | D | L | R | L | I | E | 290 | | Sbay\_655.42/1-339 | 249 | - | - | - | P | I | Q | Q | P | L | S | T | S | L | G | Q | V | L | W | L | V | T | T | I | L | Q | K | T | Y | E | L | N | V | V | D | G | D | P | - | - | - | - | H | D | Q | A | I | R | I | K | D | L | K | L | I | Q | 296 | | SAKL0A01430g/1-302 | 239 | - | - | - | - | - | S | V | S | L | P | L | K | V | N | K | V | L | S | L | V | D | S | I | L | Q | R | T | Y | D | L | T | K | R | C | T | G | N | - | - | - | - | D | A | R | P | I | K | I | N | D | L | K | L | I | E | 284 | | P53727/1-317 | 249 | - | - | - | C | T | Q | L | S | L | S | A | S | L | G | Q | V | L | W | L | V | T | S | I | L | Q | K | T | Y | D | L | N | I | A | E | R | G | P | - | - | - | - | Q | D | S | T | I | D | I | K | D | L | K | L | I | Q | 296 | |  | | G0V6B9/1-329 | 307 | C | R | A | L | F | A | L | D | C | I | P | S | L | T | P | T | R | V | P | S | T | V | V | - | - | - | - | - | - | - | - | - | - | - | - | - | - | - | - | - | - | - | - |  | | | | | | | | | | | | 329 | | Q6CU01/1-304 | 287 | C | K | D | L | F | R | S | Y | P | V | P | N | F | V | A | H | K | L | - | - | - | - | - | - | - | - | - | - | - | - | - | - | - | - | - | - | - | - | - | - | - | - | - |  | | | | | | | | | | | | 304 | | Q6FIY1/1-323 | 300 | C | R | E | L | F | R | L | H | D | I | P | E | I | P | I | T | G | H | I | N | V | P | Q | T | - | - | - | - | - | - | - | - | - | - | - | - | - | - | - | - | - | - | - |  | | | | | | | | | | | | 323 | | A7TG14/1-342 | 323 | S | R | D | I | L | S | N | N | K | F | E | N | F | K | F | E | H | I | T | I | - | - | - | - | - | - | - | - | - | - | - | - | - | - | - | - | - | - | - | - | - | - | - |  | | | | | | | | | | | | 342 | | C5DBZ5/1-305 | 288 | S | R | E | L | L | K | Q | T | T | A | P | K | Y | V | P | I | K | L | - | - | - | - | - | - | - | - | - | - | - | - | - | - | - | - | - | - | - | - | - | - | - | - | - |  | | | | | | | | | | | | 305 | | C5DVP5/1-315 | 292 | C | R | D | L | L | V | G | K | H | Q | N | K | E | L | D | A | Q | I | R | L | V | D | L | N | - | - | - | - | - | - | - | - | - | - | - | - | - | - | - | - | - | - | - |  | | | | | | | | | | | | 315 | | Kwal\_23.6296/1-308 | 291 | S | R | D | L | L | I | Q | D | I | P | L | K | Y | T | S | V | S | L | - | - | - | - | - | - | - | - | - | - | - | - | - | - | - | - | - | - | - | - | - | - | - | - | - |  | | | | | | | | | | | | 308 | | Sbay\_655.42/1-339 | 297 | C | K | E | I | L | K | Q | D | L | V | P | S | I | G | K | P | E | T | F | K | I | N | N | C | V | K | G | Y | T | P | S | P | L | G | H | G | C | C | R | N | R | P | K |  | | | | | | | | | | | | 339 | | SAKL0A01430g/1-302 | 285 | S | R | D | I | L | K | D | K | V | G | P | R | F | T | A | L | K | L | - | - | - | - | - | - | - | - | - | - | - | - | - | - | - | - | - | - | - | - | - | - | - | - | - |  | | | | | | | | | | | | 302 | | P53727/1-317 | 297 | C | R | D | I | L | K | Q | D | L | I | P | S | I | G | K | P | K | T | I | K | I | - | - | - | - | - | - | - | - | - | - | - | - | - | - | - | - | - | - | - | - | - | - |  | | | | | | | | | | | | 317 | |
