# Supplementary material for: Plus ça change – evolutionary sequence divergence predicts protein subcellular localization signals
Source: BMC Genomics. 2014 Jan 20;15:46. doi: 10.1186/1471-2164-15-46 (PMC3906766; doi:10.1186/1471-2164-15-46)
Supplement: Additional file 2 — MSA’s of proteins for which sequence divergence changes predicted localization signals. Contains links to ortholog multiple sequence alignments of each protein in Additional file 3: Table S1. [file 1471-2164-15-46-S2.zip › Q02792.html]

|  |  |  |  |  |  |  |  |  |  |  |  |  |  |  |  |  |  |  |  |  |  |  |  |  |  |  |  |  |  |  |  |  |  |  |  |  |  |  |  |  |  |  |  |  |  |  |  |  |  |  |  |  |  |  |  |  |  |  |  |  |  |  |  |  |  |  |  |  |  |  |  |  |  |  |  |  |  |  |  |  |  |  |  |  |  |  |  |  |  |  |  |  |  |  |  |  |  |  |  |  |  |  |  |  |  |  |  |  |  |  |  |  |  |  |  |  |  |  |  |  |  |  |  |  |  |  |  |  |  |  |  |  |  |  |  |  |  |  |  |  |  |  |  |  |  |  |  |  |  |  |  |  |  |  |  |  |  |  |  |  |  |  |  |  |  |  |  |  |  |  |  |  |  |  |  |  |  |  |  |  |  |  |  |  |  |  |  |  |  |  |  |  |  |  |  |  |  |  |  |  |  |  |  |  |  |  |  |  |  |  |  |  |  |  |  |  |  |  |  |  |  |  |  |  |  |  |  |  |  |  |  |  |  |  |  |  |  |  |  |  |  |  |  |  |  |  |  |  |  |  |  |  |  |  |  |  |  |  |  |  |  |  |  |  |  |  |  |  |  |  |  |  |  |  |  |  |  |  |  |  |  |  |  |  |  |  |  |  |  |  |  |  |  |  |  |  |  |  |  |  |  |  |  |  |  |  |  |  |  |  |  |  |  |  |  |  |  |  |  |  |  |  |  |  |  |  |  |  |  |  |  |  |  |  |  |  |  |  |  |  |  |  |  |  |  |  |  |  |  |  |  |  |  |  |  |  |  |  |  |  |  |  |  |  |  |  |  |  |  |  |  |  |  |  |  |  |  |  |  |  |  |  |  |  |  |  |  |  |  |  |  |  |  |  |  |  |  |  |  |  |  |  |  |  |  |  |  |  |  |  |  |  |  |  |  |  |  |  |  |  |  |  |  |  |  |  |  |  |  |  |  |  |  |  |  |  |  |  |  |  |  |  |  |  |  |  |  |  |  |  |  |  |  |  |  |  |  |  |  |  |  |  |  |  |  |  |  |  |  |  |  |  |  |  |  |  |  |  |  |  |  |  |  |  |  |  |  |  |  |  |  |  |  |  |  |  |  |  |  |  |  |  |  |  |  |  |  |  |  |  |  |  |  |  |  |  |  |  |  |  |  |  |  |  |  |  |  |  |  |  |  |  |  |  |  |  |  |  |  |  |  |  |  |  |  |  |  |  |  |  |  |  |  |  |  |  |  |  |  |  |  |  |  |  |  |  |  |  |  |  |  |  |  |  |  |  |  |  |  |  |  |  |  |  |  |  |  |  |  |  |  |  |  |  |  |  |  |  |  |  |  |  |  |  |  |  |  |  |  |  |  |  |  |  |  |  |  |  |  |  |  |  |  |  |  |  |  |  |  |  |  |  |  |  |  |  |  |  |  |  |  |  |  |  |  |  |  |  |  |  |  |  |  |  |  |  |  |  |  |  |  |  |  |  |  |  |  |  |  |  |  |  |  |  |  |  |  |  |  |  |  |  |  |  |  |  |  |  |  |  |  |  |  |  |  |  |  |  |  |  |  |  |  |  |  |  |  |  |  |  |  |  |  |  |  |  |  |  |  |  |  |  |  |  |  |  |  |  |  |  |  |  |  |  |  |  |  |  |  |  |  |  |  |  |  |  |  |  |  |  |  |  |  |  |  |  |  |  |  |  |  |  |  |  |  |  |  |  |  |  |  |  |  |  |  |  |  |  |  |  |  |  |  |  |  |  |  |  |  |  |  |  |  |  |  |  |  |  |  |  |  |  |  |  |  |  |  |  |  |  |  |  |  |  |  |  |  |  |  |  |  |  |  |  |  |  |  |  |  |  |  |  |  |  |  |  |  |  |  |  |  |  |  |  |  |  |  |  |  |  |  |  |  |  |  |  |  |  |  |  |  |  |  |  |  |  |  |  |  |  |  |  |  |  |  |  |  |  |  |  |  |  |  |  |  |  |  |  |  |  |  |  |  |  |  |  |  |  |  |  |  |  |  |  |  |  |  |  |  |  |  |  |  |  |  |  |  |  |  |  |  |  |  |  |  |  |  |  |  |  |  |  |  |  |  |  |  |  |  |  |  |  |  |  |  |  |  |  |  |  |  |  |  |  |  |  |  |  |  |  |  |  |  |  |  |  |  |  |  |  |  |  |  |  |  |  |  |  |  |  |  |  |  |  |  |  |  |  |  |  |  |  |  |  |  |  |  |  |  |  |  |  |  |  |  |  |  |  |  |  |  |  |  |  |  |  |  |  |  |  |  |  |  |  |  |  |  |  |  |  |  |  |  |  |  |  |  |  |  |  |  |  |  |  |  |  |  |  |  |  |  |  |  |  |  |  |  |  |  |  |  |  |  |  |  |  |  |  |  |  |  |  |  |  |  |  |  |  |  |  |  |  |  |  |  |  |  |  |  |  |  |  |  |  |  |  |  |  |  |  |  |  |  |  |  |  |  |  |  |  |  |  |  |  |  |  |  |  |  |  |  |  |  |  |  |  |  |  |  |  |  |  |  |  |  |  |  |  |  |  |  |  |  |  |  |  |  |  |  |  |  |  |  |  |  |  |  |  |  |  |  |  |  |  |  |  |  |  |  |  |  |  |  |  |  |  |  |  |  |  |  |  |  |  |  |  |  |  |  |  |  |  |  |  |  |  |  |  |  |  |  |  |  |  |  |  |  |  |  |  |  |  |  |  |  |  |  |  |  |  |  |  |  |  |  |  |  |  |  |  |  |  |  |  |  |  |  |  |  |  |  |  |  |  |  |  |  |  |  |  |  |  |  |  |  |  |  |  |  |  |  |  |  |  |  |  |  |  |  |  |  |  |  |  |  |  |  |  |  |  |  |  |  |  |  |  |  |  |  |  |  |  |  |  |  |  |  |  |  |  |  |  |  |  |  |  |  |  |  |  |  |  |  |  |  |  |  |  |  |  |  |  |  |  |  |  |  |  |  |  |  |  |  |  |  |  |  |  |  |  |  |  |  |  |  |  |  |  |  |  |  |  |  |  |  |  |  |  |  |  |  |  |  |  |  |  |  |  |  |  |  |  |  |  |  |  |  |  |  |  |  |  |  |  |  |  |  |  |  |  |  |  |  |  |  |  |  |  |  |  |  |  |  |  |  |  |  |  |  |  |  |  |  |  |  |  |  |  |  |  |  |  |  |  |  |  |  |  |  |  |  |  |  |  |  |  |  |  |  |  |  |  |  |  |  |  |  |  |  |  |  |  |  |  |  |  |  |  |  |  |  |  |  |  |  |  |  |  |  |  |  |  |  |  |  |  |  |  |  |  |  |  |  |  |  |  |  |  |  |  |  |  |  |  |  |  |  |  |  |  |  |  |  |  |  |  |  |  |  |  |  |  |  |  |  |  |  |  |  |  |  |  |  |  |  |  |  |  |  |  |  |  |  |  |  |  |  |  |  |  |  |  |  |  |  |  |  |  |  |  |  |  |  |  |  |  |  |  |  |  |  |  |  |  |  |  |  |  |  |  |  |  |  |  |  |  |  |  |  |  |  |  |  |  |  |  |  |  |  |  |  |  |  |  |  |  |  |  |  |  |  |  |  |  |  |  |  |  |  |  |  |  |  |  |  |  |  |  |  |  |  |  |  |  |  |  |  |  |  |  |  |  |  |  |  |  |  |  |  |  |  |  |  |  |  |  |  |  |  |  |  |  |  |  |  |  |  |  |  |  |  |  |  |  |  |  |  |  |  |  |  |  |  |  |  |  |  |  |  |  |  |  |  |  |  |  |  |  |  |  |  |  |  |  |  |  |  |  |  |  |  |  |  |  |  |  |  |  |  |  |  |  |  |  |  |  |  |  |  |  |  |  |  |  |  |  |  |  |  |  |  |  |  |  |  |  |  |  |  |  |  |  |  |  |  |  |  |  |  |  |  |  |  |  |  |  |  |  |  |  |  |  |  |  |  |  |  |  |  |  |  |  |  |  |  |  |  |  |  |  |  |  |  |  |  |  |  |  |  |  |  |  |  |  |  |  |  |  |  |  |  |  |  |  |  |  |  |  |  |  |  |  |  |  |  |  |  |  |  |  |  |  |  |  |  |  |  |  |  |  |  |  |  |  |  |  |  |  |  |  |  |  |  |  |  |  |  |  |  |  |  |  |  |  |  |  |  |  |  |  |  |  |  |  |  |  |  |  |  |  |  |  |  |  |  |  |  |  |  |  |  |  |  |  |  |  |  |  |  |  |  |  |  |  |  |  |  |  |  |  |  |  |  |  |  |  |  |  |  |  |  |  |  |  |  |  |  |  |  |  |  |  |  |  |  |  |  |  |  |  |  |  |  |  |  |  |  |  |  |  |  |  |  |  |  |  |  |  |  |  |  |  |  |  |  |  |  |  |  |  |  |  |  |  |  |  |  |  |  |  |  |  |  |  |  |  |  |  |  |  |  |  |  |  |  |  |  |  |  |  |  |  |  |  |  |  |  |  |  |  |  |  |  |  |  |  |  |  |  |  |  |  |  |  |  |  |  |  |  |  |  |  |  |  |  |  |  |  |  |  |  |  |  |  |  |  |  |  |  |  |  |  |  |  |  |  |  |  |  |  |  |  |  |  |  |  |  |  |  |  |  |  |  |  |  |  |  |  |  |  |  |  |  |  |  |  |  |  |  |  |  |  |  |  |  |  |  |  |  |  |  |  |  |  |  |  |  |  |  |  |  |  |  |  |  |  |  |  |  |  |  |  |  |  |  |  |  |  |  |  |  |  |  |  |  |  |  |  |  |  |  |  |  |  |  |  |  |  |  |  |  |  |  |  |  |  |  |  |  |  |  |  |  |  |  |  |  |  |  |  |  |  |  |  |  |  |  |  |  |  |  |  |  |  |  |  |  |  |  |  |  |  |  |  |  |  |  |  |  |  |  |  |  |  |  |  |  |  |  |  |  |  |  |  |  |  |  |  |  |  |  |  |  |  |  |  |  |  |  |  |  |  |  |  |  |  |  |  |  |  |  |  |  |  |  |  |  |  |  |  |  |  |  |  |  |  |  |  |  |  |  |  |  |  |  |  |  |  |  |  |  |  |  |  |  |  |  |  |  |  |  |  |  |  |  |  |  |  |  |  |  |  |  |  |  |  |  |  |  |  |  |  |  |  |  |  |  |  |  |  |  |  |  |  |  |  |  |  |  |  |  |  |  |  |  |  |  |  |  |  |  |  |  |  |  |  |  |  |  |  |  |  |  |  |  |  |  |  |  |  |  |  |  |  |  |  |  |  |  |  |  |  |  |  |  |  |  |  |  |  |  |  |  |  |  |  |  |  |  |  |  |  |  |  |  |  |  |  |  |  |  |  |  |  |  |  |  |  |  |  |  |  |  |  |  |  |  |  |  |  |  |  |  |  |  |  |  |  |  |  |  |  |  |  |  |  |  |  |  |  |  |  |  |  |  |  |  |  |  |  |  |  |  |  |  |  |  |  |  |  |  |  |  |  |  |  |  |  |  |  |  |  |  |  |  |  |  |  |  |  |  |  |  |  |  |  |  |  |  |  |  |  |  |  |  |  |  |  |  |  |  |  |  |  |  |  |  |  |  |  |  |  |  |  |  |  |  |  |  |  |  |  |  |  |  |  |  |  |  |  |  |  |  |  |  |  |  |  |  |  |  |  |  |  |  |  |  |  |  |  |  |  |  |  |  |  |  |  |  |  |  |  |  |  |  |  |  |  |  |  |  |  |  |  |  |  |  |  |  |  |  |  |  |  |  |  |  |  |  |  |  |  |  |  |  |  |  |  |  |  |  |  |  |  |  |  |  |  |  |  |  |  |  |  |  |  |  |  |  |  |  |  |  |  |  |  |  |  |  |  |  |  |  |  |  |  |  |  |  |  |  |  |  |  |  |  |  |  |  |  |  |  |  |  |  |  |  |  |  |  |  |  |  |  |  |  |  |  |  |  |  |  |  |  |  |  |  |  |  |  |  |  |  |  |  |  |  |  |  |  |  |  |  |  |  |  |  |  |  |  |  |  |  |  |  |  |  |  |  |  |  |  |  |  |  |  |  |  |  |  |  |  |  |  |  |  |  |  |  |  |  |  |  |  |  |  |  |  |  |  |  |  |  |  |  |  |  |  |  |  |  |  |  |  |  |  |  |  |  |  |  |  |  |  |  |  |  |  |  |  |  |  |  |  |  |  |  |  |  |  |  |  |  |  |  |  |  |  |  |  |  |  |  |  |  |  |  |  |  |  |  |  |  |  |  |  |  |  |  |  |  |  |  |  |  |  |  |  |  |  |  |  |  |  |  |  |  |  |  |  |  |  |  |  |  |  |  |  |  |  |  |  |  |  |  |  |  |  |  |  |  |  |  |  |  |  |  |  |  |  |  |  |  |  |  |  |  |  |  |  |  |  |  |  |  |  |  |  |  |  |  |  |  |  |  |  |  |  |  |  |  |  |  |  |  |  |  |  |  |  |  |  |  |  |  |  |  |  |  |  |  |  |  |  |  |  |  |  |  |  |  |  |  |  |  |  |  |  |  |  |  |  |  |  |  |  |  |  |  |  |  |  |  |  |  |  |  |  |  |  |  |  |  |  |  |  |  |  |  |  |  |  |  |  |  |  |  |  |  |  |  |  |  |  |  |  |  |  |  |  |  |  |  |  |  |  |  |  |  |  |  |  |  |  |  |  |  |  |  |  |  |  |  |  |  |  |  |  |  |  |  |  |  |  |  |  |  |  |  |  |  |  |  |  |  |  |  |  |  |  |  |  |  |  |  |  |  |  |  |  |  |  |  |  |  |  |  |  |  |  |  |  |  |  |  |  |  |  |  |  |  |  |  |  |  |  |  |  |  |  |  |  |  |  |  |  |  |  |  |  |  |  |  |  |  |  |  |  |  |  |  |  |  |  |  |  |  |  |  |  |  |  |  |  |  |  |  |  |  |  |  |  |  |  |  |  |  |  |  |  |  |  |  |  |  |  |  |  |  |  |  |  |  |  |  |  |  |  |  |  |  |  |  |  |  |  |  |  |  |  |  |  |  |  |  |  |  |  |  |  |  |  |  |  |  |  |  |  |  |  |  |  |  |  |  |  |  |  |  |  |  |  |  |  |  |  |  |  |  |  |  |  |  |  |  |  |  |  |  |  |  |  |  |  |  |  |  |  |  |  |  |  |  |  |  |  |  |  |  |  |  |  |  |  |  |  |  |  |  |  |  |  |  |  |  |  |  |  |  |  |  |  |  |  |  |  |  |  |  |  |  |  |  |  |  |  |  |  |  |  |  |  |  |  |  |  |  |  |  |  |  |  |  |  |  |  |  |  |  |  |  |  |  |  |  |  |  |  |  |  |  |  |  |  |  |  |  |  |  |  |  |  |  |  |  |  |  |  |  |  |  |  |  |  |  |  |  |  |  |  |  |  |  |  |  |  |  |  |  |  |  |  |  |  |  |  |  |  |  |  |  |  |  |  |  |  |  |  |  |  |  |  |  |  |  |  |  |  |  |  |  |  |  |  |  |  |  |  |  |  |  |  |  |  |  |  |  |  |  |  |  |  |  |  |  |  |  |  |  |  |  |  |  |  |  |  |  |  |  |  |  |  |  |  |  |  |  |  |  |  |  |  |  |  |  |  |  |  |  |  |  |  |  |  |  |  |  |  |  |  |  |  |  |  |  |  |  |  |  |  |  |  |  |  |  |  |  |  |  |  |  |  |  |  |  |  |  |  |  |  |  |  |  |  |  |  |  |  |  |  |  |  |  |  |  |  |  |  |  |  |  |  |  |  |  |  |  |  |  |  |  |  |  |  |  |  |  |  |  |  |  |  |  |  |  |  |  |  |  |  |  |  |  |  |  |  |  |  |  |  |  |  |  |  |  |  |  |  |  |  |  |  |  |  |  |  |  |  |  |  |  |  |  |  |  |  |  |  |  |  |  |  |  |  |  |  |  |  |  |  |  |  |  |  |  |  |  |  |  |  |  |  |  |  |  |  |  |  |  |  |  |  |  |  |  |  |  |  |  |  |  |  |  |  |  |  |  |  |  |  |  |  |  |  |  |  |  |  |  |  |  |  |  |  |  |  |  |  |  |  |  |  |  |  |  |  |  |  |  |  |  |  |  |  |  |  |  |  |  |  |  |  |  |  |  |  |  |  |  |  |  |  |  |  |  |  |  |  |  |  |  |  |  |  |  |  |  |  |  |  |  |  |  |  |  |  |  |  |  |  |  |  |  |  |  |  |  |  |  |  |  |  |  |  |  |  |  |  |  |  |  |  |  |  |  |  |  |  |  |  |  |  |  |  |  |  |  |  |  |  |  |  |  |  |  |  |  |  |  |  |  |  |  |  |  |  |  |  |  |  |  |  |  |  |  |  |  |  |  |  |  |  |  |  |  |  |  |  |  |  |  |  |  |  |  |  |  |  |  |  |  |  |  |  |  |  |  |  |  |  |  |  |  |  |  |  |  |  |  |  |  |  |  |  |  |  |  |  |  |  |  |  |  |  |  |  |  |  |  |  |  |  |  |  |  |  |  |  |  |  |  |  |  |  |  |  |  |  |  |  |  |  |  |  |  |  |  |  |  |  |  |  |  |  |  |  |  |  |  |  |  |  |  |  |  |  |  |  |  |  |  |  |  |  |  |  |  |  |  |  |  |  |  |  |  |  |  |  |  |  |  |  |  |  |  |  |  |  |  |  |  |  |  |  |  |  |  |  |  |  |  |  |  |  |  |  |  |  |  |  |  |  |  |  |  |  |  |  |  |  |  |  |  |  |  |  |  |  |  |  |  |  |  |  |  |  |  |  |  |  |  |  |  |  |  |  |  |  |  |  |  |  |  |  |  |  |  |  |  |  |  |  |  |  |  |  |  |  |  |  |  |  |  |  |  |  |  |  |  |  |  |  |  |  |  |  |  |  |  |  |  |  |  |  |  |  |  |  |  |  |  |  |  |  |  |  |  |  |  |  |  |  |  |  |  |  |  |  |  |  |  |  |  |  |  |  |  |  |  |  |  |  |  |  |  |  |  |  |  |  |  |  |  |  |  |  |  |  |  |  |  |  |  |  |  |  |  |  |  |  |  |  |  |  |  |  |  |  |  |  |  |  |  |  |  |  |  |  |  |  |  |  |  |  |  |  |  |  |  |  |  |  |  |  |  |  |  |  |  |  |  |  |  |  |  |  |  |  |  |  |  |  |  |  |  |  |  |  |  |  |  |  |  |  |  |  |  |  |  |  |  |  |  |  |  |  |  |  |  |  |  |  |  |  |  |  |  |  |  |  |  |  |  |  |  |  |  |  |  |  |  |  |  |  |  |  |  |  |  |  |  |  |  |  |  |  |  |  |  |  |  |  |  |  |  |  |  |  |  |  |  |  |  |  |  |  |  |  |  |  |  |  |  |  |  |  |  |  |  |  |  |  |  |  |  |  |  |  |  |  |  |  |  |  |  |  |  |  |  |  |  |  |  |  |  |  |  |  |  |  |  |  |  |  |  |  |  |  |  |  |  |  |  |  |  |  |  |  |  |  |  |  |  |  |  |  |  |  |  |  |  |  |  |  |  |  |  |  |  |  |  |  |  |  |  |  |  |  |  |  |  |  |  |  |  |  |  |  |  |  |  |  |  |  |  |  |  |  |  |  |  |  |  |  |  |  |  |  |  |  |  |  |  |  |  |  |  |  |  |  |  |  |  |  |  |  |  |  |  |  |  |  |  |  |  |  |  |  |  |  |  |  |  |  |  |  |  |  |  |  |  |  |  |  |  |  |  |  |  |  |  |  |  |  |  |  |  |  |  |  |  |  |  |  |  |  |  |  |  |  |  |  |  |  |  |  |  |  |  |  |  |  |  |  |  |  |  |  |  |  |  |  |  |  |  |  |  |  |  |  |  |  |  |  |  |  |  |  |  |  |  |  |  |  |  |  |  |  |  |  |  |  |  |  |  |  |  |  |  |  |  |  |  |  |  |  |  |  |  |  |  |  |  |  |  |  |  |  |  |  |  |  |  |  |  |  |  |  |  |  |  |  |  |  |  |  |  |  |  |  |  |  |  |  |  |  |  |  |  |  |  |  |  |  |  |  |  |  |  |  |  |  |  |  |  |  |  |  |  |  |  |  |  |  |  |  |  |  |  |  |  |  |  |  |  |  |  |  |  |  |  |  |  |  |  |  |  |  |  |  |  |  |  |  |  |  |  |  |  |  |  |  |  |  |  |  |  |  |  |  |  |  |  |  |  |  |  |  |  |  |  |  |  |  |  |  |  |  |  |  |  |  |  |  |  |  |  |  |  |  |  |  |  |  |  |  |  |  |  |  |  |  |  |  |  |  |  |  |  |  |  |  |  |  |  |  |  |  |  |  |  |  |  |  |  |  |  |  |  |  |  |  |  |  |  |  |  |  |  |  |  |  |  |  |  |  |  |  |  |  |  |  |  |  |  |  |  |  |  |  |  |  |  |  |  |  |  |  |  |  |  |  |  |  |  |  |  |  |  |  |  |  |  |  |  |  |  |  |  |  |  |  |  |  |  |  |  |  |  |  |  |  |  |  |  |  |  |  |  |  |  |  |  |  |  |  |  |  |  |  |  |  |  |  |  |  |  |  |  |  |  |  |  |  |  |  |  |  |  |  |  |  |  |  |  |  |  |  |  |  |  |  |  |  |  |  |  |  |  |  |  |  |  |  |  |  |  |  |  |  |  |  |  |  |  |  |  |  |  |  |  |  |  |  |  |  |  |  |  |  |  |  |  |  |  |  |  |  |  |  |  |  |  |  |  |  |  |  |  |  |  |  |  |  |  |  |  |  |  |  |  |  |  |  |  |  |  |  |  |  |  |  |  |  |  |  |  |  |  |  |  |  |  |  |  |  |  |  |  |  |  |  |  |  |  |  |  |  |  |  |  |  |  |  |  |  |  |  |  |  |  |  |  |  |  |  |  |  |  |  |  |  |  |  |  |  |  |  |  |  |  |  |  |  |  |  |  |  |  |  |  |  |  |  |  |  |  |  |  |  |  |  |  |  |  |  |  |  |  |  |  |  |  |  |  |  |  |  |  |  |  |  |  |  |  |  |  |  |  |  |  |  |  |  |  |  |  |  |  |  |  |  |  |  |  |  |  |  |  |  |  |  |  |  |  |  |  |  |  |  |  |  |  |  |  |  |  |  |  |  |  |  |  |  |  |  |  |  |  |  |  |  |  |  |  |  |  |  |  |  |  |  |  |  |  |  |  |  |  |  |  |  |  |  |  |  |  |  |  |  |  |  |  |  |  |  |  |  |  |  |  |  |  |  |  |  |  |  |  |  |  |  |  |  |  |  |  |  |  |  |  |  |  |  |  |  |  |  |  |  |  |  |  |  |  |  |  |  |  |  |  |  |  |  |  |  |  |  |  |  |  |  |  |  |  |  |  |  |  |  |  |  |  |  |  |  |  |  |  |  |  |  |  |  |  |  |  |  |  |  |  |  |  |  |  |  |  |  |  |  |  |  |  |  |  |  |  |  |  |  |  |  |  |  |  |  |  |  |  |  |  |  |  |  |  |  |  |  |  |  |  |  |  |  |  |  |  |  |  |  |  |  |  |  |  |  |  |  |  |  |  |  |  |  |  |  |  |  |  |  |  |  |  |  |  |  |  |  |  |  |  |  |  |  |  |  |  |  |  |  |  |  |  |  |  |  |  |  |  |  |  |  |  |  |  |  |  |  |  |  |  |  |  |  |  |  |  |  |  |  |  |  |  |  |  |  |  |  |  |  |  |  |  |  |  |  |  |  |  |  |  |  |  |  |  |  |  |  |  |  |  |  |  |  |  |  |  |  |  |  |  |  |  |  |  |  |  |  |  |  |  |  |  |  |  |  |  |  |  |  |  |  |  |  |  |  |  |  |  |  |  |  |  |  |  |  |  |  |  |  |  |  |  |  |  |  |  |  |  |  |  |  |  |  |  |  |  |  |  |  |  |  |  |  |  |  |  |  |  |  |  |  |  |  |  |  |  |  |  |  |  |  |  |  |  |  |  |  |  |  |  |  |  |  |  |  |  |  |  |  |  |  |  |  |  |  |  |  |  |  |  |  |  |  |  |  |  |  |  |  |  |  |  |  |  |  |  |  |  |  |  |  |  |  |  |  |  |  |  |  |  |  |  |  |  |  |  |  |  |  |  |  |  |  |  |  |  |  |  |  |  |  |  |  |  |  |  |  |  |  |  |  |  |  |  |  |  |  |  |  |  |  |  |  |  |  |  |  |  |  |  |  |  |  |  |  |  |  |  |  |  |  |  |  |  |  |  |  |  |  |  |  |  |  |  |  |  |  |  |  |  |  |  |  |  |  |  |  |  |  |  |  |  |  |  |  |  |  |  |  |  |  |  |  |  |  |  |  |  |  |  |  |  |  |  |  |  |  |  |  |  |  |  |  |  |  |  |  |  |  |  |  |  |  |  |  |  |  |  |  |  |  |  |  |  |  |  |  |  |  |  |  |  |  |  |  |  |  |  |  |  |  |  |  |  |  |  |  |  |  |  |  |  |  |  |  |  |  |  |  |  |  |  |  |  |  |  |  |  |  |  |  |  |  |  |  |  |  |  |  |  |  |  |  |  |  |  |  |  |  |  |  |  |  |  |  |  |  |  |  |  |  |  |  |  |  |  |  |  |  |  |  |  |  |  |  |  |  |  |  |  |  |  |  |  |  |  |  |  |  |  |  |  |  |  |  |  |  |  |  |  |  |  |  |  |  |  |  |  |  |  |  |  |  |  |  |  |  |  |  |  |  |  |  |  |  |  |  |  |  |  |  |  |  |  |  |  |  |  |  |  |  |  |  |  |  |  |  |  |  |  |  |  |  |  |  |  |  |  |  |  |  |  |  |  |  |  |  |  |  |  |  |  |  |  |  |  |  |  |  |  |  |  |  |  |  |  |  |  |  |  |  |  |  |  |  |  |  |  |  |  |  |  |  |  |  |  |  |  |  |  |  |  |  |  |  |  |  |  |  |  |  |  |  |  |  |  |  |  |  |  |  |  |  |  |  |  |  |  |  |  |  |  |  |  |  |  |  |  |  |  |  |  |  |  |  |  |  |  |  |  |  |  |  |  |  |  |  |  |  |  |  |  |  |  |  |  |  |  |  |  |  |  |  |  |  |  |  |  |  |  |  |  |  |  |  |  |  |  |  |  |  |  |  |  |  |  |  |  |  |  |  |  |  |  |  |  |  |  |  |  |  |  |  |  |  |  |  |  |  |  |  |  |  |  |  |  |  |  |  |  |  |  |  |  |  |  |  |  |  |  |  |  |  |  |  |  |  |  |  |  |  |  |  |  |  |  |  |  |  |  |  |  |  |  |  |  |  |  |  |  |  |  |  |  |  |  |  |  |  |  |  |  |  |  |  |  |  |  |  |  |  |  |  |  |  |  |  |  |  |  |  |  |  |  |  |  |  |  |  |  |  |  |  |  |  |  |  |  |  |  |  |  |  |  |  |  |  |  |  |  |  |  |  |  |  |  |  |  |  |  |  |  |  |  |  |  |  |  |  |  |  |  |  |  |  |  |  |  |  |  |  |  |  |  |  |  |  |  |  |  |  |  |  |  |  |  |  |  |  |  |  |  |  |  |  |  |  |  |  |  |  |  |  |  |  |  |  |  |  |  |  |  |  |  |  |  |  |  |  |  |  |  |  |  |  |  |  |  |  |  |  |  |  |  |  |  |  |  |  |  |  |  |  |  |  |  |  |  |  |  |  |  |  |  |  |  |  |  |  |  |  |  |  |  |  |  |  |  |  |  |  |  |  |  |  |  |  |  |  |  |  |  |  |  |  |  |  |  |  |  |  |  |  |  |  |  |  |  |  |  |  |  |  |  |  |  |  |  |  |  |  |  |  |  |  |  |  |  |  |  |  |  |  |  |  |  |  |  |  |  |  |  |  |  |  |  |  |  |  |  |  |  |  |  |  |  |  |  |  |  |  |  |  |  |  |  |  |  |  |  |  |  |  |  |  |  |  |  |  |  |  |  |  |  |  |  |  |  |  |  |  |  |  |  |  |  |  |  |  |  |  |  |  |  |  |  |  |  |  |  |  |  |  |  |  |  |  |  |  |  |  |  |  |  |  |  |  |  |  |  |  |  |  |  |  |  |  |  |  |  |  |  |  |  |  |  |  |  |  |  |  |  |  |  |  |  |  |  |  |  |  |  |  |  |  |  |  |  |  |  |  |  |  |  |  |  |  |  |  |  |  |  |  |  |  |  |  |  |  |  |  |  |  |  |  |  |  |  |  |  |  |  |  |  |  |  |  |  |  |  |  |  |  |  |  |  |  |  |  |  |  |  |  |  |  |  |  |  |  |  |  |  |  |  |  |  |  |  |  |  |  |  |  |  |  |  |  |  |  |  |  |  |  |  |  |  |  |  |  |  |  |  |  |  |  |  |  |  |  |  |  |  |  |  |  |  |  |  |  |  |  |  |  |  |  |  |  |  |  |  |  |  |  |  |  |  |  |  |  |  |  |  |  |  |  |  |  |  |  |  |  |  |  |  |  |  |  |  |  |  |  |  |  |  |  |  |  |  |  |  |  |  |  |  |  |  |  |  |  |  |  |  |  |  |  |  |  |  |  |  |  |  |  |  |  |  |  |  |  |  |  |  |  |  |  |  |  |  |  |  |  |  |  |  |  |  |  |  |  |  |  |  |  |  |  |  |  |  |  |  |  |  |  |  |  |  |  |  |  |  |  |  |  |  |  |  |  |  |  |  |  |  |  |  |  |  |  |  |  |  |  |  |  |  |  |  |  |  |  |  |  |  |  |  |  |  |  |  |  |  |  |  |  |  |  |  |  |  |  |  |  |  |  |  |  |  |  |  |  |  |  |  |  |  |  |  |  |  |  |  |  |  |  |  |  |  |  |  |  |  |  |  |  |  |  |  |  |  |  |  |  |  |  |  |  |  |  |  |  |  |  |  |  |  |  |  |  |  |  |  |  |  |  |  |  |  |  |  |  |  |  |  |  |  |  |  |  |  |  |  |  |  |  |  |  |  |  |  |  |  |  |  |  |  |  |  |  |  |  |  |  |  |  |  |  |  |  |  |  |  |  |  |  |  |  |  |  |  |  |  |  |  |  |  |  |  |  |  |  |  |  |  |  |  |  |  |  |  |  |  |  |  |  |  |  |  |  |  |  |  |  |  |  |  |  |  |  |  |  |  |  |  |  |  |  |  |  |  |  |  |  |  |  |  |  |  |  |  |  |  |  |  |  |  |  |  |  |  |  |  |  |  |  |  |  |  |  |  |  |  |  |  |  |  |  |  |  |  |  |  |  |  |  |  |  |  |  |  |  |  |  |  |  |  |  |  |  |  |  |  |  |  |  |  |  |  |  |  |  |  |  |  |  |  |  |  |  |  |  |  |  |  |  |  |  |  |  |  |  |  |  |  |  |  |  |  |  |  |  |  |  |  |  |  |  |  |  |  |  |  |  |  |  |  |  |  |  |  |  |  |  |  |  |  |  |  |  |  |  |  |  |  |  |  |  |  |  |  |  |  |  |  |  |  |  |  |  |  |  |  |  |  |  |  |  |  |  |  |  |  |  |  |  |  |  |  |  |  |  |  |  |  |  |  |  |  |  |  |  |  |  |  |  |  |  |  |  |  |  |  |  |  |  |  |  |  |  |  |  |  |  |  |  |  |  |  |  |  |  |  |  |  |  |  |  |  |  |  |  |  |  |  |  |  |  |  |  |  |  |  |  |  |  |  |  |  |  |  |  |  |  |  |  |  |  |  |  |  |  |  |  |  |  |  |  |  |  |  |  |  |  |  |  |  |  |  |  |  |  |  |  |  |  |  |  |  |  |  |  |  |  |  |  |  |  |  |  |  |  |  |  |  |  |  |  |  |  |  |  |  |  |  |  |  |  |  |  |  |  |  |  |  |  |  |  |  |  |  |  |  |  |  |  |  |  |  |  |  |  |  |  |  |  |  |  |  |  |  |  |  |  |  |  |  |  |  |  |  |  |  |  |  |  |  |  |  |  |  |  |  |  |  |  |  |  |  |  |  |  |  |  |  |  |  |  |  |  |  |  |  |  |  |  |  |  |  |  |  |  |  |  |  |  |  |  |  |  |  |  |  |  |  |  |  |  |  |  |  |  |  |  |  |  |  |  |  |  |  |  |  |  |  |  |  |  |  |  |  |  |  |  |  |  |  |  |  |  |  |  |  |  |  |  |  |  |  |  |  |  |  |  |  |  |  |  |  |  |  |  |  |  |  |  |  |  |  |  |  |  |  |  |  |  |  |  |  |  |  |  |  |  |  |  |  |  |  |  |  |  |  |  |  |  |  |  |  |  |  |  |  |  |  |  |  |  |  |  |  |  |  |  |  |  |  |  |  |  |  |  |  |  |  |  |  |  |  |  |  |  |  |  |  |  |  |  |  |  |  |  |  |  |  |  |  |  |  |  |  |  |  |  |  |  |  |  |  |  |  |  |  |  |  |  |  |  |  |  |  |  |  |  |  |  |  |  |  |  |  |  |  |  |  |  |  |  |  |  |  |  |  |  |  |  |  |  |  |  |  |  |  |  |  |  |  |  |  |  |  |  |  |  |  |  |  |  |  |  |  |  |  |  |  |  |  |  |  |  |  |  |  |  |  |  |  |  |  |  |  |  |  |  |  |  |  |  |  |  |  |  |  |  |  |  |  |  |  |  |  |  |  |  |  |  |  |  |  |  |  |  |  |  |  |  |  |  |  |  |  |  |  |  |  |  |  |  |  |  |  |  |  |  |  |  |  |  |  |  |  |  |  |  |  |  |  |  |  |  |  |  |  |  |  |  |  |  |  |  |  |  |  |  |  |  |  |  |  |  |  |  |  |  |  |  |  |  |  |  |  |  |  |  |  |  |  |  |  |  |  |  |  |  |  |  |  |  |  |  |  |  |  |  |  |  |  |  |  |  |  |  |  |  |  |  |  |  |  |  |  |  |  |  |  |  |  |  |  |  |  |  |  |  |  |  |  |  |  |  |  |  |  |  |  |  |  |  |  |  |  |  |  |  |  |  |  |  |  |  |  |  |  |  |  |  |  |  |  |  |  |  |  |  |  |  |  |  |  |  |  |  |  |  |  |  |  |  |  |  |  |  |  |  |  |  |  |  |  |  |  |  |  |  |  |  |  |  |  |  |  |  |  |  |  |  |  |  |  |  |  |  |  |  |  |  |  |  |  |  |  |  |  |  |  |  |  |  |  |  |  |  |  |  |  |  |  |  |  |  |  |  |  |  |  |  |  |  |  |  |  |  |  |  |  |  |  |  |  |  |  |  |  |  |  |  |  |  |  |  |  |  |  |  |  |  |  |  |  |  |  |  |  |  |  |  |  |  |  |  |  |  |  |  |  |  |  |  |  |  |  |  |  |  |  |  |  |  |  |  |  |  |  |  |  |  |  |  |  |  |  |  |  |  |  |  |  |  |  |  |  |  |  |  |  |  |  |  |  |  |  |  |  |  |  |  |  |  |  |  |  |  |  |  |  |  |  |  |  |  |  |  |  |  |  |  |  |  |  |  |  |  |  |  |  |  |  |  |  |  |  |  |  |  |  |  |  |  |  |  |  |  |  |  |  |  |  |  |  |  |  |  |  |  |  |  |  |  |  |  |  |  |  |  |  |  |  |  |  |  |  |  |  |  |  |  |  |  |  |  |  |  |  |  |  |  |  |  |  |  |  |  |  |  |  |  |  |  |  |  |  |  |  |  |  |  |  |  |  |  |  |  |  |  |  |  |  |  |  |  |  |  |  |  |  |  |  |  |  |  |  |  |  |  |  |  |  |  |  |  |  |  |  |  |  |  |  |  |  |  |  |  |  |  |  |  |  |  |  |  |  |  |  |  |  |  |  |  |  |  |  |  |  |  |  |  |  |  |  |  |  |  |  |  |  |  |  |  |  |  |  |  |  |  |  |  |  |  |  |  |  |  |  |  |  |  |  |  |  |  |  |  |  |  |  |  |  |  |  |  |  |  |  |  |  |  |  |  |  |  |  |  |  |  |  |  |  |  |  |  |  |  |  |  |  |  |  |  |  |  |  |  |  |  |  |  |  |  |  |  |  |  |  |  |  |  |  |  |  |  |  |  |  |  |  |  |  |  |  |  |  |  |  |  |  |  |  |  |  |  |  |  |  |  |  |  |  |  |  |  |  |  |  |  |  |  |  |  |  |  |  |  |  |  |  |  |  |  |  |  |  |  |  |  |  |  |  |  |  |  |  |  |  |  |  |  |  |  |  |  |  |  |  |  |  |  |  |  |  |  |  |  |  |  |  |  |  |  |  |  |  |  |  |  |  |  |  |  |  |  |  |  |  |  |  |  |  |  |  |  |  |  |  |  |  |  |  |  |  |  |  |  |  |  |  |  |  |  |  |  |  |  |  |  |  |  |  |  |  |  |  |  |  |  |  |  |  |  |  |  |  |  |  |  |  |  |  |  |  |  |  |  |  |  |  |  |  |  |  |  |  |  |  |  |  |  |  |  |  |  |  |  |  |  |  |  |  |  |  |  |  |  |  |  |  |  |  |  |  |  |  |  |  |  |  |  |  |  |  |  |  |  |  |  |  |  |  |  |  |  |  |  |  |  |  |  |  |  |  |  |  |  |  |  |  |  |  |  |  |  |  |  |  |  |  |  |  |  |  |  |  |  |  |  |  |  |  |  |  |  |  |  |  |  |  |  |  |  |  |  |  |  |  |  |  |  |  |  |  |  |  |  |  |  |  |  |  |  |  |  |  |  |  |  |  |  |  |  |  |  |  |  |  |  |  |  |  |  |  |  |  |  |  |  |  |  |  |  |  |  |  |  |  |  |  |  |  |  |  |  |  |  |  |  |  |  |  |  |  |  |  |  |  |  |  |  |  |  |  |  |  |  |  |  |  |  |  |  |  |  |  |  |  |  |  |  |  |  |  |  |  |  |  |  |  |  |  |  |  |  |  |  |  |  |  |  |  |  |  |  |  |  |  |  |  |  |  |  |  |  |  |  |  |  |  |  |  |  |  |  |  |  |  |  |  |  |  |  |  |  |  |  |  |  |  |  |  |  |  |  |  |  |  |  |  |  |  |  |  |  |  |  |  |  |  |  |  |  |  |  |  |  |  |  |  |  |  |  |  |  |  |  |  |  |  |  |  |  |  |  |  |  |  |  |  |  |  |  |  |  |  |  |  |  |  |  |  |  |  |  |  |  |  |  |  |  |  |  |  |  |  |  |  |  |  |  |  |  |  |  |  |  |  |  |  |  |  |  |  |  |  |  |  |  |  |  |  |  |  |  |  |  |  |  |  |  |  |  |  |  |  |  |  |  |  |  |  |  |  |  |  |  |  |  |  |  |  |  |  |  |  |  |  |  |  |  |  |  |  |  |  |  |  |  |  |  |  |  |  |  |  |  |  |  |  |  |  |  |  |  |  |  |  |  |  |  |  |  |  |  |  |  |  |  |  |  |  |  |  |  |  |  |  |  |  |  |  |  |  |  |  |  |  |  |  |  |  |  |  |  |  |  |  |  |  |  |  |  |  |  |  |  |  |  |  |  |  |  |  |  |  |  |  |  |  |  |  |  |  |  |  |  |  |  |  |  |  |  |  |  |  |  |  |  |  |  |  |  |  |  |  |  |  |  |  |  |  |  |  |  |  |  |  |  |  |  |  |  |  |  |  |  |  |  |  |  |  |  |  |  |  |  |  |  |  |  |  |  |  |  |  |  |  |  |  |  |  |  |  |  |  |  |  |  |  |  |  |  |  |  |  |  |  |  |  |  |  |  |  |  |  |  |  |  |  |  |  |  |  |  |  |  |  |  |  |  |  |  |  |  |  |  |  |  |  |  |  |  |  |  |  |  |  |  |  |  |  |  |  |  |  |  |  |  |  |  |  |  |  |  |  |  |  |  |  |  |  |  |  |  |  |  |  |  |  |  |  |  |  |  |  |  |  |  |  |  |  |  |  |  |  |  |  |  |  |  |  |  |  |  |  |  |  |  |  |  |  |  |  |  |  |  |  |  |  |  |  |  |  |  |  |  |  |  |  |  |  |  |  |  |  |  |  |  |  |  |  |  |  |  |  |  |  |  |  |  |  |  |  |  |  |  |  |  |  |  |  |  |  |  |  |  |  |  |  |  |  |  |  |  |  |  |  |  |  |  |  |  |  |  |  |  |  |  |  |  |  |  |  |  |  |  |  |  |  |  |  |  |  |  |  |  |  |  |  |  |  |  |  |  |  |  |  |  |  |  |  |  |  |  |  |  |  |  |  |  |  |  |  |  |  |  |  |  |  |  |  |  |  |  |  |  |  |  |  |  |  |  |  |  |  |  |  |  |  |  |  |  |  |  |  |  |  |  |  |  |  |  |  |  |  |  |  |  |  |  |  |  |  |  |  |  |  |  |  |  |  |  |  |  |  |  |  |  |  |  |  |  |  |  |  |  |  |  |  |  |  |  |  |  |  |  |  |  |  |  |  |  |  |  |  |  |  |  |  |  |  |  |  |  |  |  |  |  |  |  |  |  |  |  |  |  |  |  |  |  |  |  |  |  |  |  |  |  |  |  |  |  |  |  |  |  |  |  |  |  |  |  |  |  |  |  |  |  |  |  |  |  |  |  |  |  |  |  |  |  |  |  |  |  |  |  |  |  |  |  |  |  |  |  |  |  |  |  |  |  |  |  |  |  |  |  |  |  |  |  |  |  |  |  |  |  |  |  |  |  |  |  |  |  |  |  |  |  |  |  |  |  |  |  |  |  |  |  |  |  |  |  |  |  |  |  |  |  |  |  |  |  |  |  |  |  |  |  |  |  |  |  |  |  |  |  |  |  |  |  |  |  |  |  |  |  |  |  |  |  |  |  |  |  |  |  |  |  |  |  |  |  |  |  |  |  |  |  |  |  |  |  |  |  |  |  |  |  |  |  |  |  |  |  |  |  |  |  |  |  |  |  |  |  |  |  |  |  |  |  |  |  |  |  |  |  |  |  |  |  |  |  |  |  |  |  |  |  |  |  |  |  |  |  |  |  |  |  |  |  |  |  |  |  |  |  |  |  |  |  |  |  |  |  |  |  |  |  |  |  |  |  |  |  |  |  |  |  |  |  |  |  |  |  |  |  |  |  |  |  |  |  |  |  |  |  |  |  |  |  |  |  |  |  |  |  |  |  |  |  |  |  |  |  |  |  |  |  |  |  |  |  |  |  |  |  |  |  |  |  |  |  |  |  |  |  |  |  |  |  |  |  |  |  |  |  |  |  |  |  |  |  |  |  |  |  |  |  |  |  |  |  |  |  |  |  |  |  |  |  |  |  |  |  |  |  |  |  |  |  |  |  |  |  |  |  |  |  |  |  |  |  |  |  |  |  |  |  |  |  |  |  |  |  |  |  |  |  |  |  |  |  |  |  |  |  |  |  |  |  |  |  |  |  |  |  |  |  |  |  |  |  |  |  |  |  |  |  |  |  |  |  |  |  |  |  |  |  |  |  |  |  |  |  |  |  |  |  |  |  |  |  |  |  |  |  |  |  |  |  |  |  |  |  |  |  |  |  |  |  |  |  |  |  |  |  |  |  |  |  |  |  |  |  |  |  |  |  |  |  |  |  |  |  |  |  |  |  |  |  |  |  |  |  |  |  |  |  |  |  |  |  |  |  |  |  |  |  |  |  |  |  |  |  |  |  |  |  |  |  |  |  |  |  |  |  |  |  |  |  |  |  |  |  |  |  |  |  |  |  |  |  |  |  |  |  |  |  |  |  |  |  |  |  |  |  |  |  |  |  |  |  |  |  |  |  |  |  |  |  |  |  |  |  |  |  |  |  |  |  |  |  |  |  |  |  |  |  |  |  |  |  |  |  |  |  |  |  |  |  |  |  |  |  |  |  |  |  |  |  |  |  |  |  |  |  |  |  |  |  |  |  |  |  |  |  |  |  |  |  |  |  |  |  |  |  |  |  |  |  |  |  |  |  |  |  |  |  |  |  |  |  |  |  |  |  |  |  |  |  |  |  |  |  |  |  |  |  |  |  |  |  |  |  |  |  |  |  |  |  |  |  |  |  |  |  |  |  |  |  |  |  |  |  |  |  |  |  |  |  |  |  |  |  |  |  |  |  |  |  |  |  |  |  |  |  |  |  |  |  |  |  |  |  |  |  |  |  |  |  |  |  |  |  |  |  |  |  |  |  |  |  |  |  |  |  |  |  |  |  |  |  |  |  |  |  |  |  |  |  |  |  |  |  |  |  |  |  |  |  |  |  |  |  |  |  |  |  |  |  |  |  |  |  |  |  |  |  |  |  |  |  |  |  |  |  |  |  |  |  |  |  |  |  |  |  |  |  |  |  |  |  |  |  |  |  |  |  |  |  |  |  |  |  |  |  |  |  |  |  |  |  |  |  |  |  |  |  |  |  |  |  |  |  |  |  |  |  |  |  |  |  |  |  |  |  |  |  |  |  |  |  |  |  |  |  |  |  |  |  |  |  |  |  |  |  |  |  |  |  |  |  |  |  |  |  |  |  |  |  |  |  |  |  |  |  |  |  |  |  |  |  |  |  |  |  |  |  |  |  |  |  |  |  |  |  |  |  |  |  |  |  |  |  |  |  |  |  |  |  |  |  |  |  |  |  |  |  |  |  |  |  |  |  |  |  |  |  |  |  |  |  |  |  |  |  |  |  |  |  |  |  |  |  |  |  |  |  |  |  |  |  |  |  |  |  |  |  |  |  |  |  |  |  |  |  |  |  |  |  |  |  |  |  |  |  |  |  |  |  |  |  |  |  |  |  |  |  |  |  |  |  |  |  |  |  |  |  |  |  |  |  |  |  |  |  |  |  |  |  |  |  |  |  |  |  |  |  |  |  |  |  |  |  |  |  |  |  |  |  |  |  |  |  |  |  |  |  |  |  |  |  |  |  |  |  |  |  |  |  |  |  |  |  |  |  |  |  |  |  |  |  |  |  |  |  |  |  |  |  |  |  |  |  |  |  |  |  |  |  |  |  |  |  |  |  |  |  |  |  |  |  |  |  |  |  |  |  |  |  |  |  |  |  |  |  |  |  |  |  |  |  |  |  |  |  |  |  |  |  |  |  |  |  |  |  |  |  |  |  |  |  |  |  |  |  |  |  |  |  |  |  |  |  |  |  |  |  |  |  |  |  |  |  |  |  |  |  |  |  |  |  |  |  |  |  |  |  |  |  |  |  |  |  |  |  |  |  |  |  |  |  |  |  |  |  |  |  |  |  |  |  |  |  |  |  |  |  |  |  |  |  |  |  |  |  |  |  |  |  |  |  |  |  |  |  |  |  |  |  |  |  |  |  |  |  |  |  |  |  |  |  |  |  |  |  |  |  |  |  |  |  |  |  |  |  |  |  |  |  |  |  |  |  |  |  |  |  |  |  |  |  |  |  |  |  |  |  |  |  |  |  |  |  |  |  |  |  |  |  |  |  |  |  |  |  |  |  |  |  |  |  |  |  |  |  |  |  |  |  |  |  |  |  |  |  |  |  |  |  |  |  |  |  |  |  |  |  |  |  |  |  |  |  |  |  |  |  |  |  |  |  |  |  |  |  |  |  |  |  |  |  |  |  |  |  |  |  |  |  |  |  |  |  |  |  |  |  |  |  |  |  |  |  |  |  |  |  |  |  |  |  |  |  |  |  |  |  |  |  |  |  |  |  |  |  |  |  |  |  |  |  |  |  |  |  |  |  |  |  |  |  |  |  |  |  |  |  |  |  |  |  |  |  |  |  |  |  |  |  |  |  |  |  |  |  |  |  |  |  |  |  |  |  |  |  |  |  |  |  |  |  |  |  |  |  |  |  |  |  |  |  |  |  |  |  |  |  |  |  |  |  |  |  |  |  |  |  |  |  |  |  |  |  |  |  |  |  |  |  |  |  |  |  |  |  |  |  |  |  |  |  |  |  |  |  |  |  |  |  |  |  |  |  |  |  |  |  |  |  |  |  |  |  |  |  |  |  |  |  |  |  |  |  |  |  |  |  |  |  |  |  |  |  |  |  |  |  |  |  |  |  |  |  |  |  |  |  |  |  |  |  |  |  |  |  |  |  |  |  |  |  |  |  |  |  |  |  |  |  |  |  |  |  |  |  |  |  |  |  |  |  |  |  |  |  |  |  |  |  |  |  |  |  |  |  |  |  |  |  |  |  |  |  |  |  |  |  |  |  |  |  |  |  |  |  |  |  |  |  |  |  |  |  |  |  |  |  |  |  |  |  |  |  |  |  |  |  |  |  |  |  |  |  |  |  |  |  |  |  |  |  |  |  |  |  |  |  |  |  |  |  |  |  |  |  |  |  |  |  |  |  |  |  |  |  |  |  |  |  |  |  |  |  |  |  |  |  |  |  |  |  |  |  |  |  |  |  |  |  |  |  |  |  |  |  |  |  |  |  |  |  |  |  |  |  |  |  |  |  |  |  |  |  |  |  |  |  |  |  |  |  |  |  |  |  |  |  |  |  |  |  |  |  |  |  |  |  |  |  |  |  |  |  |  |  |  |  |  |  |  |  |  |  |  |  |  |  |  |  |  |  |  |  |  |  |  |  |  |  |  |  |  |  |  |  |  |  |  |  |  |  |  |  |  |  |  |  |  |  |  |  |  |  |  |  |  |  |  |  |  |  |  |  |  |  |  |  |  |  |  |  |  |  |  |  |  |  |  |  |  |  |  |  |  |  |  |  |  |  |  |  |  |  |  |  |  |  |  |  |  |  |  |  |  |  |  |  |  |  |  |  |  |  |  |  |  |  |  |  |  |  |  |  |  |  |  |  |  |  |  |  |  |  |  |  |  |  |  |  |  |  |  |  |  |  |  |  |  |  |  |  |  |  |  |  |  |  |  |  |  |  |  |  |  |  |  |  |  |  |  |  |  |  |  |  |  |  |  |  |  |  |  |  |  |  |  |  |  |  |  |  |  |  |  |  |  |  |  |  |  |  |  |  |  |  |  |  |  |  |  |  |  |  |  |  |  |  |  |  |  |  |  |  |  |  |  |  |  |  |  |  |  |  |  |  |  |  |  |  |  |  |  |  |  |  |  |  |  |  |  |  |  |  |  |  |  |  |  |  |  |  |  |  |  |  |  |  |  |  |  |  |  |  |  |  |  |  |  |  |  |  |  |  |  |  |  |  |  |  |  |  |  |  |  |  |  |  |  |  |  |  |  |  |  |  |  |  |  |  |  |  |  |  |  |  |  |  |  |  |  |  |  |  |  |  |  |  |  |  |  |  |  |  |  |  |  |  |  |  |  |  |  |  |  |  |  |  |  |  |  |  |  |  |  |  |  |  |  |  |  |  |  |  |  |  |  |  |  |  |  |  |  |  |  |  |  |  |  |  |  |  |  |  |  |  |  |  |  |  |  |  |  |  |  |  |  |  |  |  |  |  |  |  |  |  |  |  |  |  |  |  |  |  |  |  |  |  |  |  |  |  |  |  |  |  |  |  |  |  |  |  |  |  |  |  |  |  |  |  |  |  |  |  |  |  |  |  |  |  |  |  |  |  |  |  |  |  |  |  |  |  |  |  |  |  |  |  |  |  |  |  |  |  |  |  |  |  |  |  |  |  |  |  |  |  |  |  |  |  |  |  |  |  |  |  |  |  |  |  |  |  |  |  |  |  |  |  |  |  |  |  |  |  |  |  |  |  |  |  |  |  |  |  |  |  |  |  |  |  |  |  |  |  |  |  |  |  |  |  |  |  |  |  |  |  |  |  |  |  |  |  |  |  |  |  |  |  |  |  |  |  |  |  |  |  |  |  |  |  |  |  |  |  |  |  |  |  |  |  |  |  |  |  |  |  |  |  |  |  |  |  |  |  |  |  |  |  |  |  |  |  |  |  |  |  |  |  |  |  |  |  |  |  |  |  |  |  |  |  |  |  |  |  |  |  |  |  |  |  |  |  |  |  |  |  |  |  |  |  |  |  |  |  |  |  |  |  |  |  |  |  |  |  |  |  |  |  |  |  |  |  |  |  |  |  |  |  |  |  |  |  |  |  |  |  |  |  |  |  |  |  |  |  |  |  |  |  |  |  |  |  |  |  |  |  |  |  |  |  |  |  |  |  |  |  |  |  |  |  |  |  |  |  |  |  |  |  |  |  |  |  |  |  |  |  |  |  |  |  |  |  |  |  |  |  |  |  |  |  |  |  |  |  |  |  |  |  |  |  |  |  |  |  |  |  |  |  |  |  |  |  |  |  |  |  |  |  |  |  |  |  |  |  |  |  |  |  |  |  |  |  |  |  |  |  |  |  |  |  |  |  |  |  |  |  |  |  |  |  |  |  |  |  |  |  |  |  |  |  |  |  |  |  |  |  |  |  |  |  |  |  |  |  |  |  |  |  |  |  |  |  |  |  |  |  |  |  |  |  |  |  |  |  |  |  |  |  |  |  |  |  |  |  |  |  |  |  |  |  |  |  |  |  |  |  |  |  |  |  |  |  |  |  |  |  |  |  |  |  |  |  |  |  |  |  |  |  |  |  |  |  |  |  |  |  |  |  |  |  |  |  |  |  |  |  |  |  |  |  |  |  |  |  |  |  |  |  |  |  |  |  |  |  |  |  |  |  |  |  |  |  |  |  |  |  |  |  |  |  |  |  |  |  |  |  |  |  |  |  |  |  |  |  |  |  |  |  |  |  |  |  |  |  |  |  |  |  |  |  |  |  |  |  |  |  |  |  |  |  |  |  |  |  |  |  |  |  |  |  |  |  |  |  |  |  |  |  |  |  |  |  |  |  |  |  |  |  |  |  |  |  |  |  |  |  |  |  |  |  |  |  |  |  |  |  |  |  |  |  |  |  |  |  |  |  |  |  |  |  |  |  |  |  |  |  |  |  |  |  |  |  |  |  |  |  |  |  |  |  |  |  |  |  |  |  |  |  |  |  |  |  |  |  |  |  |  |  |  |  |  |  |  |  |  |  |  |  |  |  |  |  |  |  |  |  |  |  |  |  |  |  |  |  |  |  |  |  |  |  |  |  |  |  |  |  |  |  |  |  |  |  |  |  |  |  |  |  |  |  |  |  |  |  |  |  |  |  |  |  |  |  |  |  |  |  |  |  |  |  |  |  |  |  |  |  |  |  |  |  |  |  |  |  |  |  |  |  |  |  |  |  |  |  |  |  |  |  |  |  |  |  |  |  |  |  |  |  |  |  |  |  |  |  |  |  |  |  |  |  |  |  |  |  |  |  |  |  |  |  |  |  |  |  |  |  |  |  |  |  |  |  |  |  |  |  |  |  |  |  |  |  |  |  |  |  |  |  |  |  |  |  |  |  |  |  |  |  |  |  |  |  |  |  |  |  |  |  |  |  |  |  |  |  |  |  |  |  |  |  |  |  |  |  |  |  |  |  |  |  |  |  |  |  |  |  |  |  |  |  |  |  |  |  |  |  |  |  |  |  |  |  |  |  |  |  |  |  |  |  |  |  |  |  |  |  |  |  |  |  |  |  |  |  |  |  |  |  |  |  |  |  |  |  |  |  |  |  |  |  |  |  |  |  |  |  |  |  |  |  |  |  |  |  |  |  |  |  |  |  |  |  |  |  |  |  |  |  |  |  |  |  |  |  |  |  |  |  |  |  |  |  |  |  |  |  |  |  |  |  |  |  |  |  |  |  |  |  |  |  |  |  |  |  |  |  |  |  |  |  |  |  |  |  |  |  |  |  |  |  |  |  |  |  |  |  |  |  |  |  |  |  |  |  |  |  |  |  |  |  |  |  |  |  |  |  |  |  |  |  |  |  |  |  |  |  |  |  |  |  |  |  |  |  |  |  |  |  |  |  |  |  |  |  |  |  |  |  |  |  |  |  |  |  |  |  |  |  |  |  |  |  |  |  |  |  |  |  |  |  |  |  |  |  |  |  |  |  |  |  |  |  |  |  |  |  |  |  |  |  |  |  |  |  |  |  |  |  |  |  |  |  |  |  |  |  |  |  |  |  |  |  |  |  |  |  |  |  |  |  |  |  |  |  |  |  |  |  |  |  |  |  |  |  |  |  |  |  |  |  |  |  |  |  |  |  |  |  |  |  |  |  |  |  |  |  |  |  |  |  |  |  |  |  |  |  |  |  |  |  |  |  |  |  |  |  |  |  |  |  |  |  |  |  |  |  |  |  |  |  |  |  |  |  |  |  |  |  |  |  |  |  |  |  |  |  |  |  |  |  |  |  |  |  |  |  |  |  |  |  |  |  |  |  |  |  |  |  |  |  |  |  |  |  |  |  |  |  |  |  |  |  |  |  |  |  |  |  |  |  |  |  |  |  |  |  |  |  |  |  |  |  |  |  |  |  |  |  |  |  |  |  |  |  |  |  |  |  |  |  |  |  |  |  |  |  |  |  |  |  |  |  |  |  |  |  |  |  |  |  |  |  |  |  |  |  |  |  |  |  |  |  |  |  |  |  |  |  |  |  |  |  |  |  |  |  |  |  |  |  |  |  |  |  |  |  |  |  |  |  |  |  |  |  |  |  |  |  |  |  |  |  |  |  |  |  |  |  |  |  |  |  |  |  |  |  |  |  |  |  |  |  |  |  |  |  |  |  |  |  |  |  |  |  |  |  |  |  |  |  |  |  |  |  |  |  |  |  |  |  |  |  |  |  |  |  |  |  |  |  |  |  |  |  |  |  |  |  |  |  |  |  |  |  |  |  |  |  |  |  |  |  |  |  |  |  |  |  |  |  |  |  |  |  |  |  |  |  |  |  |  |  |  |  |  |  |  |  |  |  |  |  |  |  |  |  |  |  |  |  |  |  |  |  |  |  |  |  |  |  |  |  |  |  |  |  |  |  |  |  |  |  |  |  |  |  |  |  |  |  |  |  |  |  |  |  |  |  |  |  |  |  |  |  |  |  |  |  |  |  |  |  |  |  |  |  |  |  |  |  |  |  |  |  |  |  |  |  |  |  |  |  |  |  |  |  |  |  |  |  |  |  |  |  |  |  |  |  |  |  |  |  |  |  |  |  |  |  |  |  |  |  |  |  |  |  |  |  |  |  |  |  |  |  |  |  |  |  |  |  |  |  |  |  |  |  |  |  |  |  |  |  |  |  |  |  |  |  |  |  |  |  |  |  |  |  |  |  |  |  |  |  |  |  |  |  |  |  |  |  |  |  |  |  |  |  |  |  |  |  |  |  |  |  |  |  |  |  |  |  |  |  |  |  |  |  |  |  |  |  |  |  |  |  |  |  |  |  |  |  |  |  |  |  |  |  |  |  |  |  |  |  |  |  |  |  |  |  |  |  |  |  |  |  |  |  |  |  |  |  |  |  |  |  |  |  |  |  |  |  |  |  |  |  |  |  |  |  |  |  |  |  |  |  |  |  |  |  |  |  |  |  |  |  |  |  |  |  |  |  |  |  |  |  |  |  |  |  |  |  |  |  |  |  |  |  |  |  |  |  |  |  |  |  |  |  |  |  |  |  |  |  |  |  |  |  |  |  |  |  |  |  |  |  |  |  |  |  |  |  |  |  |  |  |  |  |  |  |  |  |  |  |  |  |  |  |  |  |  |  |  |  |  |  |  |  |  |  |  |  |  |  |  |  |  |  |  |  |  |  |  |  |  |  |  |  |  |  |  |  |  |
| --- | --- | --- | --- | --- | --- | --- | --- | --- | --- | --- | --- | --- | --- | --- | --- | --- | --- | --- | --- | --- | --- | --- | --- | --- | --- | --- | --- | --- | --- | --- | --- | --- | --- | --- | --- | --- | --- | --- | --- | --- | --- | --- | --- | --- | --- | --- | --- | --- | --- | --- | --- | --- | --- | --- | --- | --- | --- | --- | --- | --- | --- | --- | --- | --- | --- | --- | --- | --- | --- | --- | --- | --- | --- | --- | --- | --- | --- | --- | --- | --- | --- | --- | --- | --- | --- | --- | --- | --- | --- | --- | --- | --- | --- | --- | --- | --- | --- | --- | --- | --- | --- | --- | --- | --- | --- | --- | --- | --- | --- | --- | --- | --- | --- | --- | --- | --- | --- | --- | --- | --- | --- | --- | --- | --- | --- | --- | --- | --- | --- | --- | --- | --- | --- | --- | --- | --- | --- | --- | --- | --- | --- | --- | --- | --- | --- | --- | --- | --- | --- | --- | --- | --- | --- | --- | --- | --- | --- | --- | --- | --- | --- | --- | --- | --- | --- | --- | --- | --- | --- | --- | --- | --- | --- | --- | --- | --- | --- | --- | --- | --- | --- | --- | --- | --- | --- | --- | --- | --- | --- | --- | --- | --- | --- | --- | --- | --- | --- | --- | --- | --- | --- | --- | --- | --- | --- | --- | --- | --- | --- | --- | --- | --- | --- | --- | --- | --- | --- | --- | --- | --- | --- | --- | --- | --- | --- | --- | --- | --- | --- | --- | --- | --- | --- | --- | --- | --- | --- | --- | --- | --- | --- | --- | --- | --- | --- | --- | --- | --- | --- | --- | --- | --- | --- | --- | --- | --- | --- | --- | --- | --- | --- | --- | --- | --- | --- | --- | --- | --- | --- | --- | --- | --- | --- | --- | --- | --- | --- | --- | --- | --- | --- | --- | --- | --- | --- | --- | --- | --- | --- | --- | --- | --- | --- | --- | --- | --- | --- | --- | --- | --- | --- | --- | --- | --- | --- | --- | --- | --- | --- | --- | --- | --- | --- | --- | --- | --- | --- | --- | --- | --- | --- | --- | --- | --- | --- | --- | --- | --- | --- | --- | --- | --- | --- | --- | --- | --- | --- | --- | --- | --- | --- | --- | --- | --- | --- | --- | --- | --- | --- | --- | --- | --- | --- | --- | --- | --- | --- | --- | --- | --- | --- | --- | --- | --- | --- | --- | --- | --- | --- | --- | --- | --- | --- | --- | --- | --- | --- | --- | --- | --- | --- | --- | --- | --- | --- | --- | --- | --- | --- | --- | --- | --- | --- | --- | --- | --- | --- | --- | --- | --- | --- | --- | --- | --- | --- | --- | --- | --- | --- | --- | --- | --- | --- | --- | --- | --- | --- | --- | --- | --- | --- | --- | --- | --- | --- | --- | --- | --- | --- | --- | --- | --- | --- | --- | --- | --- | --- | --- | --- | --- | --- | --- | --- | --- | --- | --- | --- | --- | --- | --- | --- | --- | --- | --- | --- | --- | --- | --- | --- | --- | --- | --- | --- | --- | --- | --- | --- | --- | --- | --- | --- | --- | --- | --- | --- | --- | --- | --- | --- | --- | --- | --- | --- | --- | --- | --- | --- | --- | --- | --- | --- | --- | --- | --- | --- | --- | --- | --- | --- | --- | --- | --- | --- | --- | --- | --- | --- | --- | --- | --- | --- | --- | --- | --- | --- | --- | --- | --- | --- | --- | --- | --- | --- | --- | --- | --- | --- | --- | --- | --- | --- | --- | --- | --- | --- | --- | --- | --- | --- | --- | --- | --- | --- | --- | --- | --- | --- | --- | --- | --- | --- | --- | --- | --- | --- | --- | --- | --- | --- | --- | --- | --- | --- | --- | --- | --- | --- | --- | --- | --- | --- | --- | --- | --- | --- | --- | --- | --- | --- | --- | --- | --- | --- | --- | --- | --- | --- | --- | --- | --- | --- | --- | --- | --- | --- | --- | --- | --- | --- | --- | --- | --- | --- | --- | --- | --- | --- | --- | --- | --- | --- | --- | --- | --- | --- | --- | --- | --- | --- | --- | --- | --- | --- | --- | --- | --- | --- | --- | --- | --- | --- | --- | --- | --- | --- | --- | --- | --- | --- | --- | --- | --- | --- | --- | --- | --- | --- | --- | --- | --- | --- | --- | --- | --- | --- | --- | --- | --- | --- | --- | --- | --- | --- | --- | --- | --- | --- | --- | --- | --- | --- | --- | --- | --- | --- | --- | --- | --- | --- | --- | --- | --- | --- | --- | --- | --- | --- | --- | --- | --- | --- | --- | --- | --- | --- | --- | --- | --- | --- | --- | --- | --- | --- | --- | --- | --- | --- | --- | --- | --- | --- | --- | --- | --- | --- | --- | --- | --- | --- | --- | --- | --- | --- | --- | --- | --- | --- | --- | --- | --- | --- | --- | --- | --- | --- | --- | --- | --- | --- | --- | --- | --- | --- | --- | --- | --- | --- | --- | --- | --- | --- | --- | --- | --- | --- | --- | --- | --- | --- | --- | --- | --- | --- | --- | --- | --- | --- | --- | --- | --- | --- | --- | --- | --- | --- | --- | --- | --- | --- | --- | --- | --- | --- | --- | --- | --- | --- | --- | --- | --- | --- | --- | --- | --- | --- | --- | --- | --- | --- | --- | --- | --- | --- | --- | --- | --- | --- | --- | --- | --- | --- | --- | --- | --- | --- | --- | --- | --- | --- | --- | --- | --- | --- | --- | --- | --- | --- | --- | --- | --- | --- | --- | --- | --- | --- | --- | --- | --- | --- | --- | --- | --- | --- | --- | --- | --- | --- | --- | --- | --- | --- | --- | --- | --- | --- | --- | --- | --- | --- | --- | --- | --- | --- | --- | --- | --- | --- | --- | --- | --- | --- | --- | --- | --- | --- | --- | --- | --- | --- | --- | --- | --- | --- | --- | --- | --- | --- | --- | --- | --- | --- | --- | --- | --- | --- | --- | --- | --- | --- | --- | --- | --- | --- | --- | --- | --- | --- | --- | --- | --- | --- | --- | --- | --- | --- | --- | --- | --- | --- | --- | --- | --- | --- | --- | --- | --- | --- | --- | --- | --- | --- | --- | --- | --- | --- | --- | --- | --- | --- | --- | --- | --- | --- | --- | --- | --- | --- | --- | --- | --- | --- | --- | --- | --- | --- | --- | --- | --- | --- | --- | --- | --- | --- | --- | --- | --- | --- | --- | --- | --- | --- | --- | --- | --- | --- | --- | --- | --- | --- | --- | --- | --- | --- | --- | --- | --- | --- | --- | --- | --- | --- | --- | --- | --- | --- | --- | --- | --- | --- | --- | --- | --- | --- | --- | --- | --- | --- | --- | --- | --- | --- | --- | --- | --- | --- | --- | --- | --- | --- | --- | --- | --- | --- | --- | --- | --- | --- | --- | --- | --- | --- | --- | --- | --- | --- | --- | --- | --- | --- | --- | --- | --- | --- | --- | --- | --- | --- | --- | --- | --- | --- | --- | --- | --- | --- | --- | --- | --- | --- | --- | --- | --- | --- | --- | --- | --- | --- | --- | --- | --- | --- | --- | --- | --- | --- | --- | --- | --- | --- | --- | --- | --- | --- | --- | --- | --- | --- | --- | --- | --- | --- | --- | --- | --- | --- | --- | --- | --- | --- | --- | --- | --- | --- | --- | --- | --- | --- | --- | --- | --- | --- | --- | --- | --- | --- | --- | --- | --- | --- | --- | --- | --- | --- | --- | --- | --- | --- | --- | --- | --- | --- | --- | --- | --- | --- | --- | --- | --- | --- | --- | --- | --- | --- | --- | --- | --- | --- | --- | --- | --- | --- | --- | --- | --- | --- | --- | --- | --- | --- | --- | --- | --- | --- | --- | --- | --- | --- | --- | --- | --- | --- | --- | --- | --- | --- | --- | --- | --- | --- | --- | --- | --- | --- | --- | --- | --- | --- | --- | --- | --- | --- | --- | --- | --- | --- | --- | --- | --- | --- | --- | --- | --- | --- | --- | --- | --- | --- | --- | --- | --- | --- | --- | --- | --- | --- | --- | --- | --- | --- | --- | --- | --- | --- | --- | --- | --- | --- | --- | --- | --- | --- | --- | --- | --- | --- | --- | --- | --- | --- | --- | --- | --- | --- | --- | --- | --- | --- | --- | --- | --- | --- | --- | --- | --- | --- | --- | --- | --- | --- | --- | --- | --- | --- | --- | --- | --- | --- | --- | --- | --- | --- | --- | --- | --- | --- | --- | --- | --- | --- | --- | --- | --- | --- | --- | --- | --- | --- | --- | --- | --- | --- | --- | --- | --- | --- | --- | --- | --- | --- | --- | --- | --- | --- | --- | --- | --- | --- | --- | --- | --- | --- | --- | --- | --- | --- | --- | --- | --- | --- | --- | --- | --- | --- | --- | --- | --- | --- | --- | --- | --- | --- | --- | --- | --- | --- | --- | --- | --- | --- | --- | --- | --- | --- | --- | --- | --- | --- | --- | --- | --- | --- | --- | --- | --- | --- | --- | --- | --- | --- | --- | --- | --- | --- | --- | --- | --- | --- | --- | --- | --- | --- | --- | --- | --- | --- | --- | --- | --- | --- | --- | --- | --- | --- | --- | --- | --- | --- | --- | --- | --- | --- | --- | --- | --- | --- | --- | --- | --- | --- | --- | --- | --- | --- | --- | --- | --- | --- | --- | --- | --- | --- | --- | --- | --- | --- | --- | --- | --- | --- | --- | --- | --- | --- | --- | --- | --- | --- | --- | --- | --- | --- | --- | --- | --- | --- | --- | --- | --- | --- | --- | --- | --- | --- | --- | --- | --- | --- | --- | --- | --- | --- | --- | --- | --- | --- | --- | --- | --- | --- | --- | --- | --- | --- | --- | --- | --- | --- | --- | --- | --- | --- | --- | --- | --- | --- | --- | --- | --- | --- | --- | --- | --- | --- | --- | --- | --- | --- | --- | --- | --- | --- | --- | --- | --- | --- | --- | --- | --- | --- | --- | --- | --- | --- | --- | --- | --- | --- | --- | --- | --- | --- | --- | --- | --- | --- | --- | --- | --- | --- | --- | --- | --- | --- | --- | --- | --- | --- | --- | --- | --- | --- | --- | --- | --- | --- | --- | --- | --- | --- | --- | --- | --- | --- | --- | --- | --- | --- | --- | --- | --- | --- | --- | --- | --- | --- | --- | --- | --- | --- | --- | --- | --- | --- | --- | --- | --- | --- | --- | --- | --- | --- | --- | --- | --- | --- | --- | --- | --- | --- | --- | --- | --- | --- | --- | --- | --- | --- | --- | --- | --- | --- | --- | --- | --- | --- | --- | --- | --- | --- | --- | --- | --- | --- | --- | --- | --- | --- | --- | --- | --- | --- | --- | --- | --- | --- | --- | --- | --- | --- | --- | --- | --- | --- | --- | --- | --- | --- | --- | --- | --- | --- | --- | --- | --- | --- | --- | --- | --- | --- | --- | --- | --- | --- | --- | --- | --- | --- | --- | --- | --- | --- | --- | --- | --- | --- | --- | --- | --- | --- | --- | --- | --- | --- | --- | --- | --- | --- | --- | --- | --- | --- | --- | --- | --- | --- | --- | --- | --- | --- | --- | --- | --- | --- | --- | --- | --- | --- | --- | --- | --- | --- | --- | --- | --- | --- | --- | --- | --- | --- | --- | --- | --- | --- | --- | --- | --- | --- | --- | --- | --- | --- | --- | --- | --- | --- | --- | --- | --- | --- | --- | --- | --- | --- | --- | --- | --- | --- | --- | --- | --- | --- | --- | --- | --- | --- | --- | --- | --- | --- | --- | --- | --- | --- | --- | --- | --- | --- | --- | --- | --- | --- | --- | --- | --- | --- | --- | --- | --- | --- | --- | --- | --- | --- | --- | --- | --- | --- | --- | --- | --- | --- | --- | --- | --- | --- | --- | --- | --- | --- | --- | --- | --- | --- | --- | --- | --- | --- | --- | --- | --- | --- | --- | --- | --- | --- | --- | --- | --- | --- | --- | --- | --- | --- | --- | --- | --- | --- | --- | --- | --- | --- | --- | --- | --- | --- | --- | --- | --- | --- | --- | --- | --- | --- | --- | --- | --- | --- | --- | --- | --- | --- | --- | --- | --- | --- | --- | --- | --- | --- | --- | --- | --- | --- | --- | --- | --- | --- | --- | --- | --- | --- | --- | --- | --- | --- | --- | --- | --- | --- | --- | --- | --- | --- | --- | --- | --- | --- | --- | --- | --- | --- | --- | --- | --- | --- | --- | --- | --- | --- | --- | --- | --- | --- | --- | --- | --- | --- | --- | --- | --- | --- | --- | --- | --- | --- | --- | --- | --- | --- | --- | --- | --- | --- | --- | --- | --- | --- | --- | --- | --- | --- | --- | --- | --- | --- | --- | --- | --- | --- | --- | --- | --- | --- | --- | --- | --- | --- | --- | --- | --- | --- | --- | --- | --- | --- | --- | --- | --- | --- | --- | --- | --- | --- | --- | --- | --- | --- | --- | --- | --- | --- | --- | --- | --- | --- | --- | --- | --- | --- | --- | --- | --- | --- | --- | --- | --- | --- | --- | --- | --- | --- | --- | --- | --- | --- | --- | --- | --- | --- | --- | --- | --- | --- | --- | --- | --- | --- | --- | --- | --- | --- | --- | --- | --- | --- | --- | --- | --- | --- | --- | --- | --- | --- | --- | --- | --- | --- | --- | --- | --- | --- | --- | --- | --- | --- | --- | --- | --- | --- | --- | --- | --- | --- | --- | --- | --- | --- | --- | --- | --- | --- | --- | --- | --- | --- | --- | --- | --- | --- | --- | --- | --- | --- | --- | --- | --- | --- | --- | --- | --- | --- | --- | --- | --- | --- | --- | --- | --- | --- | --- | --- | --- | --- | --- | --- | --- | --- | --- | --- | --- | --- | --- | --- | --- | --- | --- | --- | --- | --- | --- | --- | --- | --- | --- | --- | --- | --- | --- | --- | --- | --- | --- | --- | --- | --- | --- | --- | --- | --- | --- | --- | --- | --- | --- | --- | --- | --- | --- | --- | --- | --- | --- | --- | --- | --- | --- | --- | --- | --- | --- | --- | --- | --- | --- | --- | --- | --- | --- | --- | --- | --- | --- | --- | --- | --- | --- | --- | --- | --- | --- | --- | --- | --- | --- | --- | --- | --- | --- | --- | --- | --- | --- | --- | --- | --- | --- | --- | --- | --- | --- | --- | --- | --- | --- | --- | --- | --- | --- | --- | --- | --- | --- | --- | --- | --- | --- | --- | --- | --- | --- | --- | --- | --- | --- | --- | --- | --- | --- | --- | --- | --- | --- | --- | --- | --- | --- | --- | --- | --- | --- | --- | --- | --- | --- | --- | --- | --- | --- | --- | --- | --- | --- | --- | --- | --- | --- | --- | --- | --- | --- | --- | --- | --- | --- | --- | --- | --- | --- | --- | --- | --- | --- | --- | --- | --- | --- | --- | --- | --- | --- | --- | --- | --- | --- | --- | --- | --- | --- | --- | --- | --- | --- | --- | --- | --- | --- | --- | --- | --- | --- | --- | --- | --- | --- | --- | --- | --- | --- | --- | --- | --- | --- | --- | --- | --- | --- | --- | --- | --- | --- | --- | --- | --- | --- | --- | --- | --- | --- | --- | --- | --- | --- | --- | --- | --- | --- | --- | --- | --- | --- | --- | --- | --- | --- | --- | --- | --- | --- | --- | --- | --- | --- | --- | --- | --- | --- | --- | --- | --- | --- | --- | --- | --- | --- | --- | --- | --- | --- | --- | --- | --- | --- | --- | --- | --- | --- | --- | --- | --- | --- | --- | --- | --- | --- | --- | --- | --- | --- | --- | --- | --- | --- | --- | --- | --- | --- | --- | --- | --- | --- | --- | --- | --- | --- | --- | --- | --- | --- | --- | --- | --- | --- | --- | --- | --- | --- | --- | --- | --- | --- | --- | --- | --- | --- | --- | --- | --- | --- | --- | --- | --- | --- | --- | --- | --- | --- | --- | --- | --- | --- | --- | --- | --- | --- | --- | --- | --- | --- | --- | --- | --- | --- | --- | --- | --- | --- | --- | --- | --- | --- | --- | --- | --- | --- | --- | --- | --- | --- | --- | --- | --- | --- | --- | --- | --- | --- | --- | --- | --- | --- | --- | --- | --- | --- | --- | --- | --- | --- | --- | --- | --- | --- | --- | --- | --- | --- | --- | --- | --- | --- | --- | --- | --- | --- | --- | --- | --- | --- | --- | --- | --- | --- | --- | --- | --- | --- | --- | --- | --- | --- | --- | --- | --- | --- | --- | --- | --- | --- | --- | --- | --- | --- | --- | --- | --- | --- | --- | --- | --- | --- | --- | --- | --- | --- | --- | --- | --- | --- | --- | --- | --- | --- | --- | --- | --- | --- | --- | --- | --- | --- | --- | --- | --- | --- | --- | --- | --- | --- | --- | --- | --- | --- | --- | --- | --- | --- | --- | --- | --- | --- | --- | --- | --- | --- | --- | --- | --- | --- | --- | --- | --- | --- | --- | --- | --- | --- | --- | --- | --- | --- | --- | --- | --- | --- | --- | --- | --- | --- | --- | --- | --- | --- | --- | --- | --- | --- | --- | --- | --- | --- | --- | --- | --- | --- | --- | --- | --- | --- | --- | --- | --- | --- | --- | --- | --- | --- | --- | --- | --- | --- | --- | --- | --- | --- | --- | --- | --- | --- | --- | --- | --- | --- | --- | --- | --- | --- | --- | --- | --- | --- | --- | --- | --- | --- | --- | --- | --- | --- | --- | --- | --- | --- | --- | --- | --- | --- | --- | --- | --- | --- | --- | --- | --- | --- | --- | --- | --- | --- | --- | --- | --- | --- | --- | --- | --- | --- | --- | --- | --- | --- | --- | --- | --- | --- | --- | --- | --- | --- | --- | --- | --- | --- | --- | --- | --- | --- | --- | --- | --- | --- | --- | --- | --- | --- | --- | --- | --- | --- | --- | --- | --- | --- | --- | --- | --- | --- | --- | --- | --- | --- | --- | --- | --- | --- | --- | --- | --- | --- | --- | --- | --- | --- | --- | --- | --- | --- | --- | --- | --- | --- | --- | --- | --- | --- | --- | --- | --- | --- | --- | --- | --- | --- | --- | --- | --- | --- | --- | --- | --- | --- | --- | --- | --- | --- | --- | --- | --- | --- | --- | --- | --- | --- | --- | --- | --- | --- | --- | --- | --- | --- | --- | --- | --- | --- | --- | --- | --- | --- | --- | --- | --- | --- | --- | --- | --- | --- | --- | --- | --- | --- | --- | --- | --- | --- | --- | --- | --- | --- | --- | --- | --- | --- | --- | --- | --- | --- | --- | --- | --- | --- | --- | --- | --- | --- | --- | --- | --- | --- | --- | --- | --- | --- | --- | --- | --- | --- | --- | --- | --- | --- | --- | --- | --- | --- | --- | --- | --- | --- | --- | --- | --- | --- | --- | --- | --- | --- | --- | --- | --- | --- | --- | --- | --- | --- | --- | --- | --- | --- | --- | --- | --- | --- | --- | --- | --- | --- | --- | --- | --- | --- | --- | --- | --- | --- | --- | --- | --- | --- | --- | --- | --- | --- | --- | --- | --- | --- | --- | --- | --- | --- | --- | --- | --- | --- | --- | --- | --- | --- | --- | --- | --- | --- | --- | --- | --- | --- | --- | --- | --- | --- | --- | --- | --- | --- | --- | --- | --- | --- | --- | --- | --- | --- | --- | --- | --- | --- | --- | --- | --- | --- | --- | --- | --- | --- | --- | --- | --- | --- | --- | --- | --- | --- | --- | --- | --- | --- | --- | --- | --- | --- | --- | --- | --- | --- | --- | --- | --- | --- | --- | --- | --- | --- | --- | --- | --- | --- | --- | --- | --- | --- | --- | --- | --- | --- | --- | --- | --- | --- | --- | --- | --- | --- | --- | --- | --- | --- | --- | --- | --- | --- | --- | --- | --- | --- | --- | --- | --- | --- | --- | --- | --- | --- | --- | --- | --- | --- | --- | --- | --- | --- | --- | --- | --- | --- | --- | --- | --- | --- | --- | --- | --- | --- | --- | --- | --- | --- | --- | --- | --- | --- | --- | --- | --- | --- | --- | --- | --- | --- | --- | --- | --- | --- | --- | --- | --- | --- | --- | --- | --- | --- | --- | --- | --- | --- | --- | --- | --- | --- | --- | --- | --- | --- | --- | --- | --- | --- | --- | --- | --- | --- | --- | --- | --- | --- | --- | --- | --- | --- | --- | --- | --- | --- | --- | --- | --- | --- | --- | --- | --- | --- | --- | --- | --- | --- | --- | --- | --- | --- | --- | --- | --- | --- | --- | --- | --- | --- | --- | --- | --- | --- | --- | --- | --- | --- | --- | --- | --- | --- | --- | --- | --- | --- | --- | --- | --- | --- | --- | --- | --- | --- | --- | --- | --- | --- | --- | --- | --- | --- | --- | --- | --- | --- | --- | --- | --- | --- | --- | --- | --- | --- | --- | --- | --- | --- | --- | --- | --- | --- | --- | --- | --- | --- | --- | --- | --- | --- | --- | --- | --- | --- | --- | --- | --- | --- | --- | --- | --- | --- | --- | --- | --- | --- | --- | --- | --- | --- | --- | --- | --- | --- | --- | --- | --- | --- | --- | --- | --- | --- | --- | --- | --- | --- | --- | --- | --- | --- | --- | --- | --- | --- | --- | --- | --- | --- | --- | --- | --- | --- | --- | --- | --- | --- | --- | --- | --- | --- | --- | --- | --- | --- | --- | --- | --- | --- | --- | --- | --- | --- | --- | --- | --- | --- | --- | --- | --- | --- | --- | --- | --- | --- | --- | --- | --- | --- | --- | --- | --- | --- | --- | --- | --- | --- | --- | --- | --- | --- | --- | --- | --- | --- | --- | --- | --- | --- | --- | --- | --- | --- | --- | --- | --- | --- | --- | --- | --- | --- | --- | --- | --- | --- | --- | --- | --- | --- | --- | --- | --- | --- | --- | --- | --- | --- | --- | --- | --- | --- | --- | --- | --- | --- | --- | --- | --- | --- | --- | --- | --- | --- | --- | --- | --- | --- | --- | --- | --- | --- | --- | --- | --- | --- | --- | --- | --- | --- | --- | --- | --- | --- | --- | --- | --- | --- | --- | --- | --- | --- | --- | --- | --- | --- | --- | --- | --- | --- | --- | --- | --- | --- | --- | --- | --- | --- | --- | --- | --- | --- | --- | --- | --- | --- | --- | --- | --- | --- | --- | --- | --- | --- | --- | --- | --- | --- | --- | --- | --- | --- | --- | --- | --- | --- | --- | --- | --- | --- | --- | --- | --- | --- | --- | --- | --- | --- | --- | --- | --- | --- | --- | --- | --- | --- | --- | --- | --- | --- | --- | --- | --- | --- | --- | --- | --- | --- | --- | --- | --- | --- | --- | --- | --- | --- | --- | --- | --- | --- | --- | --- | --- | --- | --- | --- | --- | --- | --- | --- | --- | --- | --- | --- | --- | --- | --- | --- | --- | --- | --- | --- | --- | --- | --- | --- | --- | --- | --- | --- | --- | --- | --- | --- | --- | --- | --- | --- | --- | --- | --- | --- | --- | --- | --- | --- | --- | --- | --- | --- | --- | --- | --- | --- | --- | --- | --- | --- | --- | --- | --- | --- | --- | --- | --- | --- | --- | --- | --- | --- | --- | --- | --- | --- | --- | --- | --- | --- | --- | --- | --- | --- | --- | --- | --- | --- | --- | --- | --- | --- | --- | --- | --- | --- | --- | --- | --- | --- | --- | --- | --- | --- | --- | --- | --- | --- | --- | --- | --- | --- | --- | --- | --- | --- | --- | --- | --- | --- | --- | --- | --- | --- | --- | --- | --- | --- | --- | --- | --- | --- | --- | --- | --- | --- | --- | --- | --- | --- | --- | --- | --- | --- | --- | --- | --- | --- | --- | --- | --- | --- | --- | --- | --- | --- | --- | --- | --- | --- | --- | --- | --- | --- | --- | --- | --- | --- | --- | --- | --- | --- | --- | --- | --- | --- | --- | --- | --- | --- | --- | --- | --- | --- | --- | --- | --- | --- | --- | --- | --- | --- | --- | --- | --- | --- | --- | --- | --- | --- | --- | --- | --- | --- | --- | --- | --- | --- | --- | --- | --- | --- | --- | --- | --- | --- | --- | --- | --- | --- | --- | --- | --- | --- | --- | --- | --- | --- | --- | --- | --- | --- | --- | --- | --- | --- | --- | --- | --- | --- | --- | --- | --- | --- | --- | --- | --- | --- | --- | --- | --- | --- | --- | --- | --- | --- | --- | --- | --- | --- | --- | --- | --- | --- | --- | --- | --- | --- | --- | --- | --- | --- | --- | --- | --- | --- | --- | --- | --- | --- | --- | --- | --- | --- | --- | --- | --- | --- | --- | --- | --- | --- | --- | --- | --- | --- | --- | --- | --- | --- | --- | --- | --- | --- | --- | --- | --- | --- | --- | --- | --- | --- | --- | --- | --- | --- | --- | --- | --- | --- | --- | --- | --- | --- | --- | --- | --- | --- | --- | --- | --- | --- | --- | --- | --- | --- | --- | --- | --- | --- | --- | --- | --- | --- | --- | --- | --- | --- | --- | --- | --- | --- | --- | --- | --- | --- | --- | --- | --- | --- | --- | --- | --- | --- | --- | --- | --- | --- | --- | --- | --- | --- | --- | --- | --- | --- | --- | --- | --- | --- | --- | --- | --- | --- | --- | --- | --- | --- | --- | --- | --- | --- | --- | --- | --- | --- | --- | --- | --- | --- | --- | --- | --- | --- | --- | --- | --- | --- | --- | --- | --- | --- | --- | --- | --- | --- | --- | --- | --- | --- | --- | --- | --- | --- | --- | --- | --- | --- | --- | --- | --- | --- | --- | --- | --- | --- | --- | --- | --- | --- | --- | --- | --- | --- | --- | --- | --- | --- | --- | --- | --- | --- | --- | --- | --- | --- | --- | --- | --- | --- | --- | --- | --- | --- | --- | --- | --- | --- | --- | --- | --- | --- | --- | --- | --- | --- | --- | --- | --- | --- | --- | --- | --- | --- | --- | --- | --- | --- | --- | --- | --- | --- | --- | --- | --- | --- | --- | --- | --- | --- | --- | --- | --- | --- | --- | --- | --- | --- | --- | --- | --- | --- | --- | --- | --- | --- | --- | --- | --- | --- | --- | --- | --- | --- | --- | --- | --- | --- | --- | --- | --- | --- | --- | --- | --- | --- | --- | --- | --- | --- | --- | --- | --- | --- | --- | --- | --- | --- | --- | --- | --- | --- | --- | --- | --- | --- | --- | --- | --- | --- | --- | --- | --- | --- | --- | --- | --- | --- | --- | --- | --- | --- | --- | --- | --- | --- | --- | --- | --- | --- | --- | --- | --- | --- | --- | --- | --- | --- | --- | --- | --- | --- | --- | --- | --- | --- | --- | --- | --- | --- | --- | --- | --- | --- | --- | --- | --- | --- | --- | --- | --- | --- | --- | --- | --- | --- | --- | --- | --- | --- | --- | --- | --- | --- | --- | --- | --- | --- | --- | --- | --- | --- | --- | --- | --- | --- | --- | --- | --- | --- | --- | --- | --- | --- | --- | --- | --- | --- | --- | --- | --- | --- | --- | --- | --- | --- | --- | --- | --- | --- | --- | --- | --- | --- | --- | --- | --- | --- | --- | --- | --- | --- | --- | --- | --- | --- | --- | --- | --- | --- | --- | --- | --- | --- | --- | --- | --- | --- | --- | --- | --- | --- | --- | --- | --- | --- | --- | --- | --- | --- | --- | --- | --- | --- | --- | --- | --- | --- | --- | --- | --- | --- | --- | --- | --- | --- | --- | --- | --- | --- | --- | --- | --- | --- | --- | --- | --- | --- | --- | --- | --- | --- | --- | --- | --- | --- | --- | --- | --- | --- | --- | --- | --- | --- | --- | --- | --- | --- | --- | --- | --- | --- | --- | --- | --- | --- | --- | --- | --- | --- | --- | --- | --- | --- | --- | --- | --- | --- | --- | --- | --- | --- | --- | --- | --- | --- | --- | --- | --- | --- | --- | --- | --- | --- | --- | --- | --- | --- | --- | --- | --- | --- | --- | --- | --- | --- | --- | --- | --- | --- | --- | --- | --- | --- | --- | --- | --- | --- | --- | --- | --- | --- | --- | --- | --- | --- | --- | --- | --- | --- | --- | --- | --- | --- | --- | --- | --- | --- | --- | --- | --- | --- | --- | --- | --- | --- | --- | --- | --- | --- | --- | --- | --- | --- | --- | --- | --- | --- | --- | --- | --- | --- | --- | --- | --- | --- | --- | --- | --- | --- | --- | --- | --- | --- | --- | --- | --- | --- | --- | --- | --- | --- | --- | --- | --- | --- | --- | --- | --- | --- | --- | --- | --- | --- | --- | --- | --- | --- | --- | --- | --- | --- | --- | --- | --- | --- | --- | --- | --- | --- | --- | --- | --- | --- | --- | --- | --- | --- | --- | --- | --- | --- | --- | --- | --- | --- | --- | --- | --- | --- | --- | --- | --- | --- | --- | --- | --- | --- | --- | --- | --- | --- | --- | --- | --- | --- | --- | --- | --- | --- | --- | --- | --- | --- | --- | --- | --- | --- | --- | --- | --- | --- | --- | --- | --- | --- | --- | --- | --- | --- | --- | --- | --- | --- | --- | --- | --- | --- | --- | --- | --- | --- | --- | --- | --- | --- | --- | --- | --- | --- | --- | --- | --- | --- | --- | --- | --- | --- | --- | --- | --- | --- | --- | --- | --- | --- | --- | --- | --- | --- | --- | --- | --- | --- | --- | --- | --- | --- | --- | --- | --- | --- | --- | --- | --- | --- | --- | --- | --- | --- | --- | --- | --- | --- | --- | --- | --- | --- | --- | --- | --- | --- | --- | --- | --- | --- | --- | --- | --- | --- | --- | --- | --- | --- | --- | --- | --- | --- | --- | --- | --- | --- | --- | --- | --- | --- | --- | --- | --- | --- | --- | --- | --- | --- | --- | --- | --- | --- | --- | --- | --- | --- | --- | --- | --- | --- | --- | --- | --- | --- | --- | --- | --- | --- | --- | --- | --- | --- | --- | --- | --- | --- | --- | --- | --- | --- | --- | --- | --- | --- | --- | --- | --- | --- | --- | --- | --- | --- | --- | --- | --- | --- | --- | --- | --- | --- | --- | --- | --- | --- | --- | --- | --- | --- | --- | --- | --- | --- | --- | --- | --- | --- | --- | --- | --- | --- | --- | --- | --- | --- | --- | --- | --- | --- | --- | --- | --- | --- | --- | --- | --- | --- | --- | --- | --- | --- | --- | --- | --- | --- | --- | --- | --- | --- | --- | --- | --- | --- | --- | --- | --- | --- | --- | --- | --- | --- | --- | --- | --- | --- | --- | --- | --- | --- | --- | --- | --- | --- | --- | --- | --- | --- | --- | --- | --- | --- | --- | --- | --- | --- | --- | --- | --- | --- | --- | --- | --- | --- | --- | --- | --- | --- | --- | --- | --- | --- | --- | --- | --- | --- | --- | --- | --- | --- | --- | --- | --- | --- | --- | --- | --- | --- | --- | --- | --- | --- | --- | --- | --- | --- | --- | --- | --- | --- | --- | --- | --- | --- | --- | --- | --- | --- | --- | --- | --- | --- | --- | --- | --- | --- | --- | --- | --- | --- | --- | --- | --- | --- | --- | --- | --- | --- | --- | --- | --- | --- | --- | --- | --- | --- | --- | --- | --- | --- | --- | --- | --- | --- | --- | --- | --- | --- | --- | --- | --- | --- | --- | --- | --- | --- | --- | --- | --- | --- | --- | --- | --- | --- | --- | --- | --- | --- | --- | --- | --- | --- | --- | --- | --- | --- | --- | --- | --- | --- | --- | --- | --- | --- | --- | --- | --- | --- | --- | --- | --- | --- | --- | --- | --- | --- | --- | --- | --- | --- | --- | --- | --- | --- | --- | --- | --- | --- | --- | --- | --- | --- | --- | --- | --- | --- | --- | --- | --- | --- | --- | --- | --- | --- | --- | --- | --- | --- | --- | --- | --- | --- | --- | --- | --- | --- | --- | --- | --- | --- | --- | --- | --- | --- | --- | --- | --- | --- | --- | --- | --- | --- | --- | --- | --- | --- | --- | --- | --- | --- | --- | --- | --- | --- | --- | --- | --- | --- | --- | --- | --- | --- | --- | --- | --- | --- | --- | --- | --- | --- | --- | --- | --- | --- | --- | --- | --- | --- | --- | --- | --- | --- | --- | --- | --- | --- | --- | --- | --- | --- | --- | --- | --- | --- | --- | --- | --- | --- | --- | --- | --- | --- | --- | --- | --- | --- | --- | --- | --- | --- | --- | --- | --- | --- | --- | --- | --- | --- | --- | --- | --- | --- | --- | --- | --- | --- | --- | --- | --- | --- | --- | --- | --- | --- | --- | --- | --- | --- | --- | --- | --- | --- | --- | --- | --- | --- | --- | --- | --- | --- | --- | --- | --- | --- | --- | --- | --- | --- | --- | --- | --- | --- | --- | --- | --- | --- | --- | --- | --- | --- | --- | --- | --- | --- | --- | --- | --- | --- | --- | --- | --- | --- | --- | --- | --- | --- | --- | --- | --- | --- | --- | --- | --- | --- | --- | --- | --- | --- | --- | --- | --- | --- | --- | --- | --- | --- | --- | --- | --- | --- | --- | --- | --- | --- | --- | --- | --- | --- | --- | --- | --- | --- | --- | --- | --- | --- | --- | --- | --- | --- | --- | --- | --- | --- | --- | --- | --- | --- | --- | --- | --- | --- | --- | --- | --- | --- | --- | --- | --- | --- | --- | --- | --- | --- | --- | --- | --- | --- | --- | --- | --- | --- | --- | --- | --- | --- | --- | --- | --- | --- | --- | --- | --- | --- | --- | --- | --- | --- | --- | --- | --- | --- | --- | --- | --- | --- | --- | --- | --- | --- | --- | --- | --- | --- | --- | --- | --- | --- | --- | --- | --- | --- | --- | --- | --- | --- | --- | --- | --- | --- | --- | --- | --- | --- | --- | --- | --- | --- | --- | --- | --- | --- | --- | --- | --- | --- | --- | --- | --- | --- | --- | --- | --- | --- | --- | --- | --- | --- | --- | --- | --- | --- | --- | --- | --- | --- | --- | --- | --- | --- | --- | --- | --- | --- | --- | --- | --- | --- | --- | --- | --- | --- | --- | --- | --- | --- | --- | --- | --- | --- | --- | --- | --- | --- | --- | --- | --- | --- | --- | --- | --- | --- | --- | --- | --- | --- | --- | --- | --- | --- | --- | --- | --- | --- | --- | --- | --- | --- | --- | --- | --- | --- | --- | --- | --- | --- | --- | --- | --- | --- | --- | --- | --- | --- | --- | --- | --- | --- | --- | --- | --- | --- | --- | --- | --- | --- | --- | --- | --- | --- | --- | --- | --- | --- | --- | --- | --- | --- | --- | --- | --- | --- | --- | --- | --- | --- | --- | --- | --- | --- | --- | --- | --- | --- | --- | --- | --- | --- | --- | --- | --- | --- | --- | --- | --- | --- | --- | --- | --- | --- | --- | --- | --- | --- | --- | --- | --- | --- | --- | --- | --- | --- | --- | --- | --- | --- | --- | --- | --- | --- | --- | --- | --- | --- | --- | --- | --- | --- | --- | --- | --- | --- | --- | --- | --- | --- | --- | --- | --- | --- | --- | --- | --- | --- | --- | --- | --- | --- | --- | --- | --- | --- | --- | --- | --- | --- | --- | --- | --- | --- | --- | --- | --- | --- | --- | --- | --- | --- | --- | --- | --- | --- | --- | --- | --- | --- | --- | --- | --- | --- | --- | --- | --- | --- | --- | --- | --- | --- | --- | --- | --- | --- | --- | --- | --- | --- | --- | --- | --- | --- | --- | --- | --- | --- | --- | --- | --- | --- | --- | --- | --- | --- | --- | --- | --- | --- | --- | --- | --- | --- | --- | --- | --- | --- | --- | --- | --- | --- | --- | --- | --- | --- | --- | --- | --- | --- | --- | --- | --- | --- | --- | --- | --- | --- | --- | --- | --- | --- | --- | --- | --- | --- | --- | --- | --- | --- | --- | --- | --- | --- | --- | --- | --- | --- | --- | --- | --- | --- | --- | --- | --- | --- | --- | --- | --- | --- | --- | --- | --- | --- | --- | --- | --- | --- | --- | --- | --- | --- | --- | --- | --- | --- | --- | --- | --- | --- | --- | --- | --- | --- | --- | --- | --- | --- | --- | --- | --- | --- | --- | --- | --- | --- | --- | --- | --- | --- | --- | --- | --- | --- | --- | --- | --- | --- | --- | --- | --- | --- | --- | --- | --- | --- | --- | --- | --- | --- | --- | --- | --- | --- | --- | --- | --- | --- | --- | --- | --- | --- | --- | --- | --- | --- | --- | --- | --- | --- | --- | --- | --- | --- | --- | --- | --- | --- | --- | --- | --- | --- | --- | --- | --- | --- | --- | --- | --- | --- | --- | --- | --- | --- | --- | --- | --- | --- | --- | --- | --- | --- | --- | --- | --- | --- | --- | --- | --- | --- | --- | --- | --- | --- | --- | --- | --- | --- | --- | --- | --- | --- | --- | --- | --- | --- | --- | --- | --- | --- | --- | --- | --- | --- | --- | --- | --- | --- | --- | --- | --- | --- | --- | --- | --- | --- | --- | --- | --- | --- | --- | --- | --- | --- | --- | --- | --- | --- | --- | --- | --- | --- | --- | --- | --- | --- | --- | --- | --- | --- | --- | --- | --- | --- | --- | --- | --- | --- | --- | --- | --- | --- | --- | --- | --- | --- | --- | --- | --- | --- | --- | --- | --- | --- | --- | --- | --- | --- | --- | --- | --- | --- | --- | --- | --- | --- | --- | --- | --- | --- | --- | --- | --- | --- | --- | --- | --- | --- | --- | --- | --- | --- | --- | --- | --- | --- | --- | --- | --- | --- | --- | --- | --- | --- | --- | --- | --- | --- | --- | --- | --- | --- | --- | --- | --- | --- | --- | --- | --- | --- | --- | --- | --- | --- | --- | --- | --- | --- | --- | --- | --- | --- | --- | --- | --- | --- | --- | --- | --- | --- | --- | --- | --- | --- | --- | --- | --- | --- | --- | --- | --- | --- | --- | --- | --- | --- | --- | --- | --- | --- | --- | --- | --- | --- | --- | --- | --- | --- | --- | --- | --- | --- | --- | --- | --- | --- | --- | --- | --- | --- | --- | --- | --- | --- | --- | --- | --- | --- | --- | --- | --- | --- | --- | --- | --- | --- | --- | --- | --- | --- | --- | --- | --- | --- | --- | --- | --- | --- | --- | --- | --- | --- | --- | --- | --- | --- | --- | --- | --- | --- | --- | --- | --- | --- | --- | --- | --- | --- | --- | --- | --- | --- | --- | --- | --- | --- | --- | --- | --- | --- | --- | --- | --- | --- | --- | --- | --- | --- | --- | --- | --- | --- | --- | --- | --- | --- | --- | --- | --- | --- | --- | --- | --- | --- | --- | --- | --- | --- | --- | --- | --- | --- | --- | --- | --- | --- | --- | --- | --- | --- | --- | --- | --- | --- | --- | --- | --- | --- | --- | --- | --- | --- | --- | --- | --- | --- | --- | --- | --- | --- | --- | --- | --- | --- | --- | --- | --- | --- | --- | --- | --- | --- | --- | --- | --- | --- | --- | --- | --- | --- | --- | --- | --- | --- | --- | --- | --- | --- | --- | --- | --- | --- | --- | --- | --- | --- | --- | --- | --- | --- | --- | --- | --- | --- | --- | --- | --- | --- | --- | --- | --- | --- | --- | --- | --- | --- | --- | --- | --- | --- | --- | --- | --- | --- | --- | --- | --- | --- | --- | --- | --- | --- | --- | --- | --- | --- | --- | --- | --- | --- | --- | --- | --- | --- | --- | --- | --- | --- | --- | --- | --- | --- | --- | --- | --- | --- | --- | --- | --- | --- | --- | --- | --- | --- | --- | --- | --- | --- | --- | --- | --- | --- | --- | --- | --- | --- | --- | --- | --- | --- | --- | --- | --- | --- | --- | --- | --- | --- | --- | --- | --- | --- | --- | --- | --- | --- | --- | --- | --- | --- | --- | --- | --- | --- | --- | --- | --- | --- | --- | --- | --- | --- | --- | --- | --- | --- | --- | --- | --- | --- | --- | --- | --- | --- | --- | --- | --- | --- | --- | --- | --- | --- | --- | --- | --- | --- | --- | --- | --- | --- | --- | --- | --- | --- | --- | --- | --- | --- | --- | --- | --- | --- | --- | --- | --- | --- | --- | --- | --- | --- | --- | --- | --- | --- | --- | --- | --- | --- | --- | --- | --- | --- | --- | --- | --- | --- | --- | --- | --- | --- | --- | --- | --- | --- | --- | --- | --- | --- | --- | --- | --- | --- | --- | --- | --- | --- | --- | --- | --- | --- | --- | --- | --- | --- | --- | --- | --- | --- | --- | --- | --- | --- | --- | --- | --- | --- | --- | --- | --- | --- | --- | --- | --- | --- | --- | --- | --- | --- | --- | --- | --- | --- | --- | --- | --- | --- | --- | --- | --- | --- | --- | --- | --- | --- | --- | --- | --- | --- | --- | --- | --- | --- | --- | --- | --- | --- | --- | --- | --- | --- | --- | --- | --- | --- | --- | --- | --- | --- | --- | --- | --- | --- | --- | --- | --- | --- | --- | --- | --- | --- | --- | --- | --- | --- | --- | --- | --- | --- | --- | --- | --- | --- | --- | --- | --- | --- | --- | --- | --- | --- | --- | --- | --- | --- | --- | --- | --- | --- | --- | --- | --- | --- | --- | --- | --- | --- | --- | --- | --- | --- | --- | --- | --- | --- | --- | --- | --- | --- | --- | --- | --- | --- | --- | --- | --- | --- | --- | --- | --- | --- | --- | --- | --- | --- | --- | --- | --- | --- | --- | --- | --- | --- | --- | --- | --- | --- | --- | --- | --- | --- | --- | --- | --- | --- | --- | --- | --- | --- | --- | --- | --- | --- | --- | --- | --- | --- | --- | --- | --- | --- | --- | --- | --- | --- | --- | --- | --- | --- | --- | --- | --- | --- | --- | --- | --- | --- | --- | --- | --- | --- | --- | --- | --- | --- | --- | --- | --- | --- | --- | --- | --- | --- | --- | --- | --- | --- | --- | --- | --- | --- | --- | --- | --- | --- | --- | --- | --- | --- | --- | --- | --- | --- | --- | --- | --- | --- | --- | --- | --- | --- | --- | --- | --- | --- | --- | --- | --- | --- | --- | --- | --- | --- | --- | --- | --- | --- | --- | --- | --- | --- | --- | --- | --- | --- | --- | --- | --- | --- | --- | --- | --- | --- | --- | --- | --- | --- | --- | --- | --- | --- | --- | --- | --- | --- | --- | --- | --- | --- | --- | --- | --- | --- | --- | --- | --- | --- | --- | --- | --- | --- | --- | --- | --- | --- | --- | --- | --- | --- | --- | --- | --- | --- | --- | --- | --- | --- | --- | --- | --- | --- | --- | --- | --- | --- | --- | --- | --- | --- | --- | --- | --- | --- | --- | --- | --- | --- | --- | --- | --- | --- | --- | --- | --- | --- | --- | --- | --- | --- | --- | --- | --- | --- | --- | --- | --- | --- | --- | --- | --- | --- | --- | --- | --- | --- | --- | --- | --- | --- | --- | --- | --- | --- | --- | --- | --- | --- | --- | --- | --- | --- | --- | --- | --- | --- | --- | --- | --- | --- | --- | --- | --- | --- | --- | --- | --- | --- | --- | --- | --- | --- | --- | --- | --- | --- | --- | --- | --- | --- | --- | --- | --- | --- | --- | --- | --- | --- | --- | --- | --- | --- | --- | --- | --- | --- | --- | --- | --- | --- | --- | --- | --- | --- | --- | --- | --- | --- | --- | --- | --- | --- | --- | --- | --- | --- | --- | --- | --- | --- | --- | --- | --- | --- | --- | --- | --- | --- | --- | --- | --- | --- | --- | --- | --- | --- | --- | --- | --- | --- | --- | --- | --- | --- | --- | --- | --- | --- | --- | --- | --- | --- | --- | --- | --- | --- | --- | --- | --- | --- | --- | --- | --- | --- | --- | --- | --- | --- | --- | --- | --- | --- | --- | --- | --- | --- | --- | --- | --- | --- | --- | --- | --- | --- | --- | --- | --- | --- | --- | --- | --- | --- | --- | --- | --- | --- | --- | --- | --- | --- | --- | --- | --- | --- | --- | --- | --- | --- | --- | --- | --- | --- | --- | --- | --- | --- | --- | --- | --- | --- | --- | --- | --- | --- | --- | --- | --- | --- | --- | --- | --- | --- | --- | --- | --- | --- | --- | --- | --- | --- | --- | --- | --- | --- | --- | --- | --- | --- | --- | --- | --- | --- | --- | --- | --- | --- | --- | --- | --- | --- | --- | --- | --- | --- | --- | --- | --- | --- | --- | --- | --- | --- | --- | --- | --- | --- | --- | --- | --- | --- | --- | --- | --- | --- | --- | --- | --- | --- | --- | --- | --- | --- | --- | --- | --- | --- | --- | --- | --- | --- | --- | --- | --- | --- | --- | --- | --- | --- | --- | --- | --- | --- | --- | --- | --- | --- | --- | --- | --- | --- | --- | --- | --- | --- | --- | --- | --- | --- | --- | --- | --- | --- | --- | --- | --- | --- | --- | --- | --- | --- | --- | --- | --- | --- | --- | --- | --- | --- | --- | --- | --- | --- | --- | --- | --- | --- | --- | --- | --- | --- | --- | --- | --- | --- | --- | --- | --- | --- | --- | --- | --- | --- | --- | --- | --- | --- | --- | --- | --- | --- | --- | --- | --- | --- | --- | --- | --- | --- | --- | --- | --- | --- | --- | --- | --- | --- | --- | --- | --- | --- | --- | --- | --- | --- | --- | --- | --- | --- | --- | --- | --- | --- | --- | --- | --- | --- | --- | --- | --- | --- | --- | --- | --- | --- | --- | --- | --- | --- | --- | --- | --- | --- | --- | --- | --- | --- | --- | --- | --- | --- | --- | --- | --- | --- | --- | --- | --- | --- | --- | --- | --- | --- | --- | --- | --- | --- | --- | --- | --- | --- | --- | --- | --- | --- | --- | --- | --- | --- | --- | --- | --- | --- | --- | --- | --- | --- | --- | --- | --- | --- | --- | --- | --- | --- | --- | --- | --- | --- | --- | --- | --- | --- | --- | --- | --- | --- | --- | --- | --- | --- | --- | --- | --- | --- | --- | --- | --- | --- | --- | --- | --- | --- | --- | --- | --- | --- | --- | --- | --- | --- | --- | --- | --- | --- | --- | --- | --- | --- | --- | --- | --- | --- | --- | --- | --- | --- | --- | --- | --- | --- | --- | --- | --- | --- | --- | --- | --- | --- | --- | --- | --- | --- | --- | --- | --- | --- | --- | --- | --- | --- | --- | --- | --- | --- | --- | --- | --- | --- | --- | --- | --- | --- | --- | --- | --- | --- | --- | --- | --- | --- | --- | --- | --- | --- | --- | --- | --- | --- | --- | --- | --- | --- | --- | --- | --- | --- | --- | --- | --- | --- | --- | --- | --- | --- | --- | --- | --- | --- | --- | --- | --- | --- | --- | --- | --- | --- | --- | --- | --- | --- | --- | --- | --- | --- | --- | --- | --- | --- | --- | --- | --- | --- | --- | --- | --- | --- | --- | --- | --- | --- | --- | --- | --- | --- | --- | --- | --- | --- | --- | --- | --- | --- | --- | --- | --- | --- | --- | --- | --- | --- | --- | --- | --- | --- | --- | --- | --- | --- | --- | --- | --- | --- | --- | --- | --- | --- | --- | --- | --- | --- | --- | --- | --- | --- | --- | --- | --- | --- | --- | --- | --- | --- | --- | --- | --- | --- | --- | --- | --- | --- | --- | --- | --- | --- | --- | --- | --- | --- | --- | --- | --- | --- | --- | --- | --- | --- | --- | --- | --- | --- | --- | --- | --- | --- | --- | --- | --- | --- | --- | --- | --- | --- | --- | --- | --- | --- | --- | --- | --- | --- | --- | --- | --- | --- | --- | --- | --- | --- | --- | --- | --- | --- | --- | --- | --- | --- | --- | --- | --- | --- | --- | --- | --- | --- | --- | --- | --- | --- | --- | --- | --- | --- | --- | --- | --- | --- | --- | --- | --- | --- | --- | --- | --- | --- | --- | --- | --- | --- | --- | --- | --- | --- | --- | --- | --- | --- | --- | --- | --- | --- | --- | --- | --- | --- | --- | --- | --- | --- | --- | --- | --- | --- | --- | --- | --- | --- | --- | --- | --- | --- | --- | --- | --- | --- | --- | --- | --- | --- | --- | --- | --- | --- | --- | --- | --- | --- | --- | --- | --- | --- | --- | --- | --- | --- | --- | --- | --- | --- | --- | --- | --- | --- | --- | --- | --- | --- | --- | --- | --- | --- | --- | --- | --- | --- | --- | --- | --- | --- | --- | --- | --- | --- | --- | --- | --- | --- | --- | --- | --- | --- | --- | --- | --- | --- | --- | --- | --- | --- | --- | --- | --- | --- | --- | --- | --- | --- | --- | --- | --- | --- | --- | --- | --- | --- | --- | --- | --- | --- | --- | --- | --- | --- | --- | --- | --- | --- | --- | --- | --- | --- | --- | --- | --- | --- | --- | --- | --- | --- | --- | --- | --- | --- | --- | --- | --- | --- | --- | --- | --- | --- | --- | --- | --- | --- | --- | --- | --- | --- | --- | --- | --- | --- | --- | --- | --- | --- | --- | --- | --- | --- | --- | --- | --- | --- | --- | --- | --- | --- | --- | --- | --- | --- | --- | --- | --- | --- | --- | --- | --- | --- | --- | --- | --- | --- | --- | --- | --- | --- | --- | --- | --- | --- | --- | --- | --- | --- | --- | --- | --- | --- | --- | --- | --- | --- | --- | --- | --- | --- | --- | --- | --- | --- | --- | --- | --- | --- | --- | --- | --- | --- | --- | --- | --- | --- | --- | --- | --- | --- | --- | --- | --- | --- | --- | --- | --- | --- | --- | --- | --- | --- | --- | --- | --- | --- | --- | --- | --- | --- | --- | --- | --- | --- | --- | --- | --- | --- | --- | --- | --- | --- | --- | --- | --- | --- | --- | --- | --- | --- | --- | --- | --- | --- | --- | --- | --- | --- | --- | --- | --- | --- | --- | --- | --- | --- | --- | --- | --- | --- | --- | --- | --- | --- | --- | --- | --- | --- | --- | --- | --- | --- | --- | --- | --- | --- | --- | --- | --- | --- | --- | --- | --- | --- | --- | --- | --- | --- | --- | --- | --- | --- | --- | --- | --- | --- | --- | --- | --- | --- | --- | --- | --- | --- | --- | --- | --- | --- | --- | --- | --- | --- | --- | --- | --- | --- | --- | --- | --- | --- | --- | --- | --- | --- | --- | --- | --- | --- | --- | --- | --- | --- | --- | --- | --- | --- | --- | --- | --- | --- | --- | --- | --- | --- | --- | --- | --- | --- | --- | --- | --- | --- | --- | --- | --- | --- | --- | --- | --- | --- | --- | --- | --- | --- | --- | --- | --- | --- | --- | --- | --- | --- | --- | --- | --- | --- | --- | --- | --- | --- | --- | --- | --- | --- | --- | --- | --- | --- | --- | --- | --- | --- | --- | --- | --- | --- | --- | --- | --- | --- | --- | --- | --- | --- | --- | --- | --- | --- | --- | --- | --- | --- | --- | --- | --- | --- | --- | --- | --- | --- | --- | --- | --- | --- | --- | --- | --- | --- | --- | --- | --- | --- | --- | --- | --- | --- | --- | --- | --- | --- | --- | --- | --- | --- | --- | --- | --- | --- | --- | --- | --- | --- | --- | --- | --- | --- | --- | --- | --- | --- | --- | --- | --- | --- | --- | --- | --- | --- | --- | --- | --- | --- | --- | --- | --- | --- | --- | --- | --- | --- | --- | --- | --- | --- | --- | --- | --- | --- | --- | --- | --- | --- | --- | --- | --- | --- | --- | --- | --- | --- | --- | --- | --- | --- | --- | --- | --- | --- | --- | --- | --- | --- | --- | --- | --- | --- | --- | --- | --- | --- | --- | --- | --- | --- | --- | --- | --- | --- | --- | --- | --- | --- | --- | --- | --- | --- | --- | --- | --- | --- | --- | --- | --- | --- | --- | --- | --- | --- | --- | --- | --- | --- | --- | --- | --- | --- | --- | --- | --- | --- | --- | --- | --- | --- | --- | --- | --- | --- | --- | --- | --- | --- | --- | --- | --- | --- | --- | --- | --- | --- | --- | --- | --- | --- | --- | --- | --- | --- | --- | --- | --- | --- | --- | --- | --- | --- | --- | --- | --- | --- | --- | --- | --- | --- | --- | --- | --- | --- | --- | --- | --- | --- | --- | --- | --- | --- | --- | --- | --- | --- | --- | --- | --- | --- | --- | --- | --- | --- | --- | --- | --- | --- | --- | --- | --- | --- | --- | --- | --- | --- | --- | --- | --- | --- | --- | --- | --- | --- | --- | --- | --- | --- | --- | --- | --- | --- | --- | --- | --- | --- | --- | --- | --- | --- | --- | --- | --- | --- | --- | --- | --- | --- | --- | --- | --- | --- | --- | --- | --- | --- | --- | --- | --- | --- | --- | --- | --- | --- | --- | --- | --- | --- | --- | --- | --- | --- | --- | --- | --- | --- | --- | --- | --- | --- | --- | --- | --- | --- | --- | --- | --- | --- | --- | --- | --- | --- | --- | --- | --- | --- | --- | --- | --- | --- | --- | --- | --- | --- | --- | --- | --- | --- | --- | --- | --- | --- | --- | --- | --- | --- | --- | --- | --- | --- | --- | --- | --- | --- | --- | --- | --- | --- | --- | --- | --- | --- | --- | --- | --- | --- | --- | --- | --- | --- | --- | --- | --- | --- | --- | --- | --- | --- | --- | --- | --- | --- | --- | --- | --- | --- | --- | --- | --- | --- | --- | --- | --- | --- | --- | --- | --- | --- | --- | --- | --- | --- | --- | --- | --- | --- | --- | --- | --- | --- | --- | --- | --- | --- | --- | --- | --- | --- | --- | --- | --- | --- | --- | --- | --- | --- | --- | --- | --- | --- | --- | --- | --- | --- | --- | --- | --- | --- | --- | --- | --- | --- | --- | --- | --- | --- | --- | --- | --- | --- | --- | --- | --- | --- | --- | --- | --- | --- | --- | --- | --- | --- | --- | --- | --- | --- | --- | --- | --- | --- | --- | --- | --- | --- | --- | --- | --- | --- | --- | --- | --- | --- | --- | --- | --- | --- | --- | --- | --- | --- | --- | --- | --- | --- | --- | --- | --- | --- | --- | --- | --- | --- | --- | --- | --- | --- | --- | --- | --- | --- | --- | --- | --- | --- | --- | --- | --- | --- | --- | --- | --- | --- | --- | --- | --- | --- | --- | --- | --- | --- | --- | --- | --- | --- | --- | --- | --- | --- | --- | --- | --- | --- | --- | --- | --- | --- | --- | --- | --- | --- | --- | --- | --- | --- | --- | --- | --- | --- | --- | --- | --- | --- | --- | --- | --- | --- | --- | --- | --- | --- | --- | --- | --- | --- | --- | --- | --- | --- | --- | --- | --- | --- | --- | --- | --- | --- | --- | --- | --- | --- | --- | --- | --- | --- | --- | --- | --- | --- | --- | --- | --- | --- | --- | --- | --- | --- | --- | --- | --- | --- | --- | --- | --- | --- | --- | --- | --- | --- | --- | --- | --- | --- | --- | --- | --- | --- | --- | --- | --- | --- | --- | --- | --- | --- | --- | --- | --- | --- | --- | --- | --- | --- | --- | --- | --- | --- | --- | --- | --- | --- | --- | --- | --- | --- | --- | --- | --- | --- | --- | --- | --- | --- | --- | --- | --- | --- | --- | --- | --- | --- | --- | --- | --- | --- | --- | --- | --- | --- | --- | --- | --- | --- | --- | --- | --- | --- | --- | --- | --- | --- | --- | --- | --- | --- | --- | --- | --- | --- | --- | --- | --- | --- | --- | --- | --- | --- | --- | --- | --- | --- | --- | --- | --- | --- | --- | --- | --- | --- | --- | --- | --- | --- | --- | --- | --- | --- | --- | --- | --- | --- | --- | --- | --- | --- | --- | --- | --- | --- | --- | --- | --- | --- | --- | --- | --- | --- | --- | --- | --- | --- | --- | --- | --- | --- | --- | --- | --- | --- | --- | --- | --- | --- | --- | --- | --- | --- | --- | --- | --- | --- | --- | --- | --- | --- | --- | --- | --- | --- | --- | --- | --- | --- | --- | --- | --- | --- | --- | --- | --- | --- | --- | --- | --- | --- | --- | --- | --- | --- | --- | --- | --- | --- | --- | --- | --- | --- | --- | --- | --- | --- | --- | --- | --- | --- | --- | --- | --- | --- | --- | --- | --- | --- | --- | --- | --- | --- | --- | --- | --- | --- | --- | --- | --- | --- | --- | --- | --- | --- | --- | --- | --- | --- | --- | --- | --- | --- | --- | --- | --- | --- | --- | --- | --- | --- | --- | --- | --- | --- | --- | --- | --- | --- | --- | --- | --- | --- | --- | --- | --- | --- | --- | --- | --- | --- | --- | --- | --- | --- | --- | --- | --- | --- | --- | --- | --- | --- | --- | --- | --- | --- | --- | --- | --- | --- | --- | --- | --- | --- | --- | --- | --- | --- | --- | --- | --- | --- | --- | --- | --- | --- | --- | --- | --- | --- | --- | --- | --- | --- | --- | --- | --- | --- | --- | --- | --- | --- | --- | --- | --- | --- | --- | --- | --- | --- | --- | --- | --- | --- | --- | --- | --- | --- | --- | --- | --- | --- | --- | --- | --- | --- | --- | --- | --- | --- | --- | --- | --- | --- | --- | --- | --- | --- | --- | --- | --- | --- | --- | --- | --- | --- | --- | --- | --- | --- | --- | --- | --- | --- | --- | --- | --- | --- | --- | --- | --- | --- | --- | --- | --- | --- | --- | --- | --- | --- | --- | --- | --- | --- | --- | --- | --- | --- | --- | --- | --- | --- | --- | --- | --- | --- | --- | --- | --- | --- | --- | --- | --- | --- | --- | --- | --- | --- | --- | --- | --- | --- | --- | --- | --- | --- | --- | --- | --- | --- | --- | --- | --- | --- | --- | --- | --- | --- | --- | --- | --- | --- | --- | --- | --- | --- | --- | --- | --- | --- | --- | --- | --- | --- | --- | --- | --- | --- | --- | --- | --- | --- | --- | --- | --- | --- | --- | --- | --- | --- | --- | --- | --- | --- | --- | --- | --- | --- | --- | --- | --- | --- | --- | --- | --- | --- | --- | --- | --- | --- | --- | --- | --- | --- | --- | --- | --- | --- | --- | --- | --- | --- | --- | --- | --- | --- | --- | --- | --- | --- | --- | --- | --- | --- | --- | --- | --- | --- | --- | --- | --- | --- | --- | --- | --- | --- | --- | --- | --- | --- | --- | --- | --- | --- | --- | --- | --- | --- | --- | --- | --- | --- | --- | --- | --- | --- | --- | --- | --- | --- | --- | --- | --- | --- | --- | --- | --- | --- | --- | --- | --- | --- | --- | --- | --- | --- | --- | --- | --- | --- | --- | --- | --- | --- | --- | --- | --- | --- | --- | --- | --- | --- | --- | --- | --- | --- | --- | --- | --- | --- | --- | --- | --- | --- | --- | --- | --- | --- | --- | --- | --- | --- | --- | --- | --- | --- | --- | --- | --- | --- | --- | --- | --- | --- | --- | --- | --- | --- | --- | --- | --- | --- | --- | --- | --- | --- | --- | --- | --- | --- | --- | --- | --- | --- | --- | --- | --- | --- | --- | --- | --- | --- | --- | --- | --- | --- | --- | --- | --- | --- | --- | --- | --- | --- | --- | --- | --- | --- | --- | --- | --- | --- | --- | --- | --- | --- | --- | --- | --- | --- | --- | --- | --- | --- | --- | --- | --- | --- | --- | --- | --- | --- | --- | --- | --- | --- | --- | --- | --- | --- | --- | --- | --- | --- | --- | --- | --- | --- | --- | --- | --- | --- | --- | --- | --- | --- | --- | --- | --- | --- | --- | --- | --- | --- | --- | --- | --- | --- | --- | --- | --- | --- | --- | --- | --- | --- | --- | --- | --- | --- | --- | --- | --- | --- | --- | --- | --- | --- | --- | --- | --- | --- | --- | --- | --- | --- | --- | --- | --- | --- | --- | --- | --- | --- | --- | --- | --- | --- | --- | --- | --- | --- | --- | --- | --- | --- | --- | --- | --- | --- | --- | --- | --- | --- | --- | --- | --- | --- | --- | --- | --- | --- | --- | --- | --- | --- | --- | --- | --- | --- | --- | --- | --- | --- | --- | --- | --- | --- | --- | --- | --- | --- | --- | --- | --- | --- | --- | --- | --- | --- | --- | --- | --- | --- | --- | --- | --- | --- | --- | --- | --- | --- | --- | --- | --- | --- | --- | --- | --- | --- | --- | --- | --- | --- | --- | --- | --- | --- | --- | --- | --- | --- | --- | --- | --- | --- | --- | --- | --- | --- | --- | --- | --- | --- | --- | --- | --- | --- | --- | --- | --- | --- | --- | --- | --- | --- | --- | --- | --- | --- | --- | --- | --- | --- | --- | --- | --- | --- | --- | --- | --- | --- | --- | --- | --- | --- | --- | --- | --- | --- | --- | --- | --- | --- | --- | --- | --- | --- | --- | --- | --- | --- | --- | --- | --- | --- | --- | --- | --- | --- | --- | --- | --- | --- | --- | --- | --- | --- | --- | --- | --- | --- | --- | --- | --- | --- | --- | --- | --- | --- | --- | --- | --- | --- | --- | --- | --- | --- | --- | --- | --- | --- | --- | --- | --- | --- | --- | --- | --- | --- | --- | --- | --- | --- | --- | --- | --- | --- | --- | --- | --- | --- | --- | --- | --- | --- | --- | --- | --- | --- | --- | --- | --- | --- | --- | --- | --- | --- | --- | --- | --- | --- | --- | --- | --- | --- | --- | --- | --- | --- | --- | --- | --- | --- | --- | --- | --- | --- | --- | --- | --- | --- | --- | --- | --- | --- | --- | --- | --- | --- | --- | --- | --- | --- | --- | --- | --- | --- | --- | --- | --- | --- | --- | --- | --- | --- | --- | --- | --- | --- | --- | --- | --- | --- | --- | --- | --- | --- | --- | --- | --- | --- | --- | --- | --- | --- | --- | --- | --- | --- | --- | --- | --- | --- | --- | --- | --- | --- | --- | --- | --- | --- | --- | --- | --- | --- | --- | --- | --- | --- | --- | --- | --- | --- | --- | --- | --- | --- | --- | --- | --- | --- | --- | --- | --- | --- | --- | --- | --- | --- | --- | --- | --- | --- | --- | --- | --- | --- | --- | --- | --- | --- | --- | --- | --- | --- | --- | --- | --- | --- | --- | --- | --- | --- | --- | --- | --- | --- | --- | --- | --- | --- | --- | --- | --- | --- | --- | --- | --- | --- | --- | --- | --- | --- | --- | --- | --- | --- | --- | --- | --- | --- | --- | --- | --- | --- | --- | --- | --- | --- | --- | --- | --- | --- | --- | --- | --- | --- | --- | --- | --- | --- | --- | --- | --- | --- | --- | --- | --- | --- | --- | --- | --- | --- | --- | --- | --- | --- | --- | --- | --- | --- | --- | --- | --- | --- | --- | --- | --- | --- | --- | --- | --- | --- | --- | --- | --- | --- | --- | --- | --- | --- | --- | --- | --- | --- | --- | --- | --- | --- | --- | --- | --- | --- | --- | --- | --- | --- | --- | --- | --- | --- | --- | --- | --- | --- | --- | --- | --- | --- | --- | --- | --- | --- | --- | --- | --- | --- | --- | --- | --- | --- | --- | --- | --- | --- | --- | --- | --- | --- | --- | --- | --- | --- | --- | --- | --- | --- | --- | --- | --- | --- | --- | --- | --- | --- | --- | --- | --- | --- | --- | --- | --- | --- | --- | --- | --- | --- | --- | --- | --- | --- | --- | --- | --- | --- | --- | --- | --- | --- | --- | --- | --- | --- | --- | --- | --- | --- | --- | --- | --- | --- | --- | --- | --- | --- | --- | --- | --- | --- | --- | --- | --- | --- | --- | --- | --- | --- | --- | --- | --- | --- | --- | --- | --- | --- | --- | --- | --- | --- | --- | --- | --- | --- | --- | --- | --- | --- | --- | --- | --- | --- | --- | --- | --- | --- | --- | --- | --- | --- | --- | --- | --- | --- | --- | --- | --- | --- | --- | --- | --- | --- | --- | --- | --- | --- | --- | --- | --- | --- | --- | --- | --- | --- | --- | --- | --- | --- | --- | --- | --- | --- | --- | --- | --- | --- | --- | --- | --- | --- | --- | --- | --- | --- | --- | --- | --- | --- | --- | --- | --- | --- | --- | --- | --- | --- | --- | --- | --- | --- | --- | --- | --- | --- | --- | --- | --- | --- | --- | --- | --- | --- | --- | --- | --- | --- | --- | --- | --- | --- | --- | --- | --- | --- | --- | --- | --- | --- | --- | --- | --- | --- | --- | --- | --- | --- | --- | --- | --- | --- | --- | --- | --- | --- | --- | --- | --- | --- | --- | --- | --- | --- | --- | --- | --- | --- | --- | --- | --- | --- | --- | --- | --- | --- | --- | --- | --- | --- | --- | --- | --- | --- | --- | --- | --- | --- | --- | --- | --- | --- | --- | --- | --- | --- | --- | --- | --- | --- | --- | --- | --- | --- | --- | --- | --- | --- | --- | --- | --- | --- | --- | --- | --- | --- | --- | --- | --- | --- | --- | --- | --- | --- | --- | --- | --- | --- | --- | --- | --- | --- | --- | --- | --- | --- | --- | --- | --- | --- | --- | --- | --- | --- | --- | --- | --- | --- | --- | --- | --- | --- | --- | --- | --- | --- | --- | --- | --- | --- | --- | --- | --- | --- | --- | --- | --- | --- | --- | --- | --- | --- | --- | --- | --- | --- | --- | --- | --- | --- | --- | --- | --- | --- | --- | --- | --- | --- | --- | --- | --- | --- | --- | --- | --- | --- | --- | --- | --- | --- | --- | --- | --- | --- | --- | --- | --- | --- | --- | --- | --- | --- | --- | --- | --- | --- | --- | --- | --- | --- | --- | --- | --- | --- | --- | --- | --- | --- | --- | --- | --- | --- | --- | --- | --- | --- | --- | --- | --- | --- | --- | --- | --- | --- | --- | --- | --- | --- | --- | --- | --- | --- | --- | --- | --- | --- | --- | --- | --- | --- | --- | --- | --- | --- | --- | --- | --- | --- | --- | --- | --- | --- | --- | --- | --- | --- | --- | --- | --- | --- | --- | --- | --- | --- | --- | --- | --- | --- | --- | --- | --- | --- | --- | --- | --- | --- | --- | --- | --- | --- | --- | --- | --- | --- | --- | --- | --- | --- | --- | --- | --- | --- | --- | --- | --- | --- | --- | --- | --- | --- | --- | --- | --- | --- | --- | --- | --- | --- | --- | --- | --- | --- | --- | --- | --- | --- | --- | --- | --- | --- | --- | --- | --- | --- | --- | --- | --- | --- | --- | --- | --- | --- | --- | --- | --- | --- | --- | --- | --- | --- | --- | --- | --- | --- | --- | --- | --- | --- | --- | --- | --- | --- | --- | --- | --- | --- | --- | --- | --- | --- | --- | --- | --- | --- | --- | --- | --- | --- | --- | --- | --- | --- | --- | --- | --- | --- | --- | --- | --- | --- | --- | --- | --- | --- | --- | --- | --- | --- | --- | --- | --- | --- | --- | --- | --- | --- | --- | --- | --- | --- | --- | --- | --- | --- | --- | --- | --- | --- | --- | --- | --- | --- | --- | --- | --- | --- | --- | --- | --- | --- | --- | --- | --- | --- | --- | --- | --- | --- | --- | --- | --- | --- | --- | --- | --- | --- | --- | --- | --- | --- | --- | --- | --- | --- | --- | --- | --- | --- | --- | --- | --- | --- | --- | --- | --- | --- | --- | --- | --- | --- | --- | --- | --- | --- | --- | --- | --- | --- | --- | --- | --- | --- | --- | --- | --- | --- | --- | --- | --- | --- | --- | --- | --- | --- | --- | --- | --- | --- | --- | --- | --- | --- | --- | --- | --- | --- | --- | --- | --- | --- | --- | --- | --- | --- | --- | --- | --- | --- | --- | --- | --- | --- | --- | --- | --- | --- | --- | --- | --- | --- | --- | --- | --- | --- | --- | --- | --- | --- | --- | --- | --- | --- | --- | --- | --- | --- | --- | --- | --- | --- | --- | --- | --- | --- | --- | --- | --- | --- | --- | --- | --- | --- | --- | --- | --- | --- | --- | --- | --- | --- | --- | --- | --- | --- | --- | --- | --- | --- | --- | --- | --- | --- | --- | --- | --- | --- | --- | --- | --- | --- | --- | --- | --- | --- | --- | --- | --- | --- | --- | --- | --- | --- | --- | --- | --- | --- | --- | --- | --- | --- | --- | --- | --- | --- | --- | --- | --- | --- | --- | --- | --- | --- | --- | --- | --- | --- | --- | --- | --- | --- | --- | --- | --- | --- | --- | --- | --- | --- | --- | --- | --- | --- | --- | --- | --- | --- | --- | --- | --- | --- | --- | --- | --- | --- | --- | --- | --- | --- | --- | --- | --- | --- | --- | --- | --- | --- | --- | --- | --- | --- | --- | --- | --- | --- | --- | --- | --- | --- | --- | --- | --- | --- | --- | --- | --- | --- | --- | --- | --- | --- | --- | --- | --- | --- | --- | --- | --- | --- | --- | --- | --- | --- | --- | --- | --- | --- | --- | --- | --- | --- | --- | --- | --- | --- | --- | --- | --- | --- | --- | --- | --- | --- | --- | --- | --- | --- | --- | --- | --- | --- | --- | --- | --- | --- | --- | --- | --- | --- | --- | --- | --- | --- | --- | --- | --- | --- | --- | --- | --- | --- | --- | --- | --- | --- | --- | --- | --- | --- | --- | --- | --- | --- | --- | --- | --- | --- | --- | --- | --- | --- | --- | --- | --- | --- | --- | --- | --- | --- | --- | --- | --- | --- | --- | --- | --- | --- | --- | --- | --- | --- | --- | --- | --- | --- | --- | --- | --- | --- | --- | --- | --- | --- | --- | --- | --- | --- | --- | --- | --- | --- | --- | --- | --- | --- | --- | --- | --- | --- | --- | --- | --- | --- | --- | --- | --- | --- | --- | --- | --- | --- | --- | --- | --- | --- | --- | --- | --- | --- | --- | --- | --- | --- | --- | --- | --- | --- | --- | --- | --- | --- | --- | --- | --- | --- | --- | --- | --- | --- | --- | --- | --- | --- | --- | --- | --- | --- | --- | --- | --- | --- | --- | --- | --- | --- | --- | --- | --- | --- | --- | --- | --- | --- | --- | --- | --- | --- | --- | --- | --- | --- | --- | --- | --- | --- | --- | --- | --- | --- | --- | --- | --- | --- | --- | --- | --- | --- | --- | --- | --- | --- | --- | --- | --- | --- | --- | --- | --- | --- | --- | --- | --- | --- | --- | --- | --- | --- | --- | --- | --- | --- | --- | --- | --- | --- | --- | --- | --- | --- | --- | --- | --- | --- | --- | --- | --- | --- | --- | --- | --- | --- | --- | --- | --- | --- | --- | --- | --- | --- | --- | --- | --- | --- | --- | --- | --- | --- | --- | --- | --- | --- | --- | --- | --- | --- | --- | --- | --- | --- | --- | --- | --- | --- | --- | --- | --- | --- | --- | --- | --- | --- | --- | --- | --- | --- | --- | --- | --- | --- | --- | --- | --- | --- | --- | --- | --- | --- | --- | --- | --- | --- | --- | --- | --- | --- | --- | --- | --- | --- | --- | --- | --- | --- | --- | --- | --- | --- | --- | --- | --- | --- | --- | --- | --- | --- | --- | --- | --- | --- | --- | --- | --- | --- | --- | --- | --- | --- | --- | --- | --- | --- | --- | --- | --- | --- | --- | --- | --- | --- | --- | --- | --- | --- | --- | --- | --- | --- | --- | --- | --- | --- | --- | --- | --- | --- | --- | --- | --- | --- | --- | --- | --- | --- | --- | --- | --- | --- | --- | --- | --- | --- | --- | --- | --- | --- | --- | --- | --- | --- | --- | --- | --- | --- | --- | --- | --- | --- | --- | --- | --- | --- | --- | --- | --- | --- | --- | --- | --- | --- | --- | --- | --- | --- | --- | --- | --- | --- | --- | --- | --- | --- | --- | --- | --- | --- | --- | --- | --- | --- | --- | --- | --- | --- | --- | --- | --- | --- | --- | --- | --- | --- | --- | --- | --- | --- | --- | --- | --- | --- | --- | --- | --- | --- | --- | --- | --- | --- | --- | --- | --- | --- | --- | --- | --- | --- | --- | --- | --- | --- | --- | --- | --- | --- | --- | --- | --- | --- | --- | --- | --- | --- | --- | --- | --- | --- | --- | --- | --- | --- | --- | --- | --- | --- | --- | --- | --- | --- | --- | --- | --- | --- | --- | --- | --- | --- | --- | --- | --- | --- | --- | --- | --- | --- | --- | --- | --- | --- | --- | --- | --- | --- | --- | --- | --- | --- | --- | --- | --- | --- | --- | --- | --- | --- | --- | --- | --- | --- | --- | --- | --- | --- | --- | --- | --- | --- | --- | --- | --- | --- | --- | --- | --- | --- | --- | --- | --- | --- | --- | --- | --- | --- | --- | --- | --- | --- | --- | --- | --- | --- | --- | --- | --- | --- | --- | --- | --- | --- | --- | --- | --- | --- | --- | --- | --- | --- | --- | --- | --- | --- | --- | --- | --- | --- | --- | --- | --- | --- | --- | --- | --- | --- | --- | --- | --- | --- | --- | --- | --- | --- | --- | --- | --- | --- | --- | --- | --- | --- | --- | --- | --- | --- | --- | --- | --- | --- | --- | --- | --- | --- | --- | --- | --- | --- | --- | --- | --- | --- | --- | --- | --- | --- | --- | --- | --- | --- | --- | --- | --- | --- | --- | --- | --- | --- | --- | --- | --- | --- | --- | --- | --- | --- | --- | --- | --- | --- | --- | --- | --- | --- | --- | --- | --- | --- | --- | --- | --- | --- | --- | --- | --- | --- | --- | --- | --- | --- | --- | --- | --- | --- | --- | --- | --- | --- | --- | --- | --- | --- | --- | --- | --- | --- | --- | --- | --- | --- | --- | --- | --- | --- | --- | --- | --- | --- | --- | --- | --- | --- | --- | --- | --- | --- | --- | --- | --- | --- | --- | --- | --- | --- | --- | --- | --- | --- | --- | --- | --- | --- | --- | --- | --- | --- | --- | --- | --- | --- | --- | --- | --- | --- | --- | --- | --- | --- | --- | --- | --- | --- | --- | --- | --- | --- | --- | --- | --- | --- | --- | --- | --- | --- | --- | --- | --- | --- | --- | --- | --- | --- | --- | --- | --- | --- | --- | --- | --- | --- | --- | --- | --- | --- | --- | --- | --- | --- | --- | --- | --- | --- | --- | --- | --- | --- | --- | --- | --- | --- | --- | --- | --- | --- | --- | --- | --- | --- | --- | --- | --- | --- | --- | --- | --- | --- | --- | --- | --- | --- | --- | --- | --- | --- | --- | --- | --- | --- | --- | --- | --- | --- | --- | --- | --- | --- | --- | --- | --- | --- | --- | --- | --- | --- | --- | --- | --- | --- | --- | --- | --- | --- | --- | --- | --- | --- | --- | --- | --- | --- | --- | --- | --- | --- | --- | --- | --- | --- | --- | --- | --- | --- | --- | --- | --- | --- | --- | --- | --- | --- | --- | --- | --- | --- | --- | --- | --- | --- | --- | --- | --- | --- | --- | --- | --- | --- | --- | --- | --- | --- | --- | --- | --- | --- | --- | --- | --- | --- | --- | --- | --- | --- | --- | --- | --- | --- | --- | --- | --- | --- | --- | --- | --- | --- | --- | --- | --- | --- | --- | --- | --- | --- | --- | --- | --- | --- | --- | --- | --- | --- | --- | --- | --- | --- | --- | --- | --- | --- | --- | --- | --- | --- | --- | --- | --- | --- | --- | --- | --- | --- | --- | --- | --- | --- | --- | --- | --- | --- | --- | --- | --- | --- | --- | --- | --- | --- | --- | --- | --- | --- | --- | --- | --- | --- | --- | --- | --- | --- | --- | --- | --- | --- | --- | --- | --- | --- | --- | --- | --- | --- | --- | --- | --- | --- | --- | --- | --- | --- | --- | --- | --- | --- | --- | --- | --- | --- | --- | --- | --- | --- | --- | --- | --- | --- | --- | --- | --- | --- | --- | --- | --- | --- | --- | --- | --- | --- | --- | --- | --- | --- | --- | --- | --- | --- | --- | --- | --- | --- | --- | --- | --- | --- | --- | --- | --- | --- | --- | --- | --- | --- | --- | --- | --- | --- | --- | --- | --- | --- | --- | --- | --- | --- | --- | --- | --- | --- | --- | --- | --- | --- | --- | --- | --- | --- | --- | --- | --- | --- | --- | --- | --- | --- | --- | --- | --- | --- | --- | --- | --- | --- | --- | --- | --- | --- | --- | --- | --- | --- | --- | --- | --- | --- | --- | --- | --- | --- | --- | --- | --- | --- | --- | --- | --- | --- | --- | --- | --- | --- | --- | --- | --- | --- | --- | --- | --- | --- | --- | --- | --- | --- | --- | --- | --- | --- | --- | --- | --- | --- | --- | --- | --- | --- | --- | --- | --- | --- | --- | --- | --- | --- | --- | --- | --- | --- | --- | --- | --- | --- | --- | --- | --- | --- | --- | --- | --- | --- | --- | --- | --- | --- | --- | --- | --- | --- | --- | --- | --- | --- | --- | --- | --- | --- | --- | --- | --- | --- | --- | --- | --- | --- | --- | --- | --- | --- | --- | --- | --- | --- | --- | --- | --- | --- | --- | --- | --- | --- | --- | --- | --- | --- | --- | --- | --- | --- | --- | --- | --- | --- | --- | --- | --- | --- | --- | --- | --- | --- | --- | --- | --- | --- | --- | --- | --- | --- | --- | --- | --- | --- | --- | --- | --- | --- | --- | --- | --- | --- | --- | --- | --- | --- | --- | --- | --- | --- | --- | --- | --- | --- | --- | --- | --- | --- | --- | --- | --- | --- | --- | --- | --- | --- | --- | --- | --- | --- | --- | --- | --- | --- | --- | --- | --- | --- | --- | --- | --- | --- | --- | --- | --- | --- | --- | --- | --- | --- | --- | --- | --- | --- | --- | --- | --- | --- | --- | --- | --- | --- | --- | --- | --- | --- | --- | --- | --- | --- | --- | --- | --- | --- | --- | --- | --- | --- | --- | --- | --- | --- | --- | --- | --- | --- | --- | --- | --- | --- | --- | --- | --- | --- | --- | --- | --- | --- | --- | --- | --- | --- | --- | --- | --- | --- | --- | --- | --- | --- | --- | --- | --- | --- | --- | --- | --- | --- | --- | --- | --- | --- | --- | --- | --- | --- | --- | --- | --- | --- | --- | --- | --- | --- | --- | --- | --- | --- | --- | --- | --- | --- | --- | --- | --- | --- | --- | --- | --- | --- | --- | --- | --- | --- | --- | --- | --- | --- | --- | --- | --- | --- | --- | --- | --- | --- | --- | --- | --- | --- | --- | --- | --- | --- | --- | --- | --- | --- | --- | --- | --- | --- | --- | --- | --- | --- | --- | --- | --- | --- | --- | --- | --- | --- | --- | --- | --- | --- | --- | --- | --- | --- | --- | --- | --- | --- | --- | --- | --- | --- | --- | --- | --- | --- | --- | --- | --- | --- | --- | --- | --- | --- | --- | --- | --- | --- | --- | --- | --- | --- | --- | --- | --- | --- | --- | --- | --- | --- | --- | --- | --- | --- | --- | --- | --- | --- | --- | --- | --- | --- | --- | --- | --- | --- | --- | --- | --- | --- | --- | --- | --- | --- | --- | --- | --- | --- | --- | --- | --- | --- | --- | --- | --- | --- | --- | --- | --- | --- | --- | --- | --- | --- | --- | --- | --- | --- | --- | --- | --- | --- | --- | --- | --- | --- | --- | --- | --- | --- | --- | --- | --- | --- | --- | --- | --- | --- | --- | --- | --- | --- | --- | --- | --- | --- | --- | --- | --- | --- | --- | --- | --- | --- | --- | --- | --- | --- | --- | --- | --- | --- | --- | --- | --- | --- | --- | --- | --- | --- | --- | --- | --- | --- | --- | --- | --- | --- | --- | --- | --- | --- | --- | --- | --- | --- | --- | --- | --- | --- | --- | --- | --- | --- | --- | --- | --- | --- | --- | --- | --- | --- | --- | --- | --- | --- | --- | --- | --- | --- | --- | --- | --- | --- | --- | --- | --- | --- | --- | --- | --- | --- | --- | --- | --- | --- | --- | --- | --- | --- | --- | --- | --- | --- | --- | --- | --- | --- | --- | --- | --- | --- | --- | --- | --- | --- | --- | --- | --- | --- | --- | --- | --- | --- | --- | --- | --- | --- | --- | --- | --- | --- | --- | --- | --- | --- | --- | --- | --- | --- | --- | --- | --- | --- | --- | --- | --- | --- | --- | --- | --- | --- | --- | --- | --- | --- | --- | --- | --- | --- | --- | --- | --- | --- | --- | --- | --- | --- | --- | --- | --- | --- | --- | --- | --- | --- | --- | --- | --- | --- | --- | --- | --- | --- | --- | --- | --- | --- | --- | --- | --- | --- | --- | --- | --- | --- | --- | --- | --- | --- | --- | --- | --- | --- | --- | --- | --- | --- | --- | --- | --- | --- | --- | --- | --- | --- | --- | --- | --- | --- | --- | --- | --- | --- | --- | --- | --- | --- | --- | --- | --- | --- | --- | --- | --- | --- | --- | --- | --- | --- | --- | --- | --- | --- | --- | --- | --- | --- | --- | --- | --- | --- | --- | --- | --- | --- | --- | --- | --- | --- | --- | --- | --- | --- | --- | --- | --- | --- | --- | --- | --- | --- | --- | --- | --- | --- | --- | --- | --- | --- | --- | --- | --- | --- | --- | --- | --- | --- | --- | --- | --- | --- | --- | --- | --- | --- | --- | --- | --- | --- | --- | --- | --- | --- | --- | --- | --- | --- | --- | --- | --- | --- | --- | --- | --- | --- | --- | --- | --- | --- | --- | --- | --- | --- | --- | --- | --- | --- | --- | --- | --- | --- | --- | --- | --- | --- | --- | --- | --- | --- | --- | --- | --- | --- | --- | --- | --- | --- | --- | --- | --- | --- | --- | --- | --- | --- | --- | --- | --- | --- | --- | --- | --- | --- | --- | --- | --- | --- | --- | --- | --- | --- | --- | --- | --- | --- | --- | --- | --- | --- | --- | --- | --- | --- | --- | --- | --- | --- | --- | --- | --- | --- | --- | --- | --- | --- | --- | --- | --- | --- | --- | --- | --- | --- | --- | --- | --- | --- | --- | --- | --- | --- | --- | --- | --- | --- | --- | --- | --- | --- | --- | --- | --- | --- | --- | --- | --- | --- | --- | --- | --- | --- | --- | --- | --- | --- | --- | --- | --- | --- | --- | --- | --- | --- | --- | --- | --- | --- | --- | --- | --- | --- | --- | --- | --- | --- | --- | --- | --- | --- | --- | --- | --- | --- | --- | --- | --- | --- | --- | --- | --- | --- | --- | --- | --- | --- | --- | --- | --- | --- | --- | --- | --- | --- | --- | --- | --- | --- | --- | --- | --- | --- | --- | --- | --- | --- | --- | --- | --- | --- | --- | --- | --- | --- | --- | --- | --- | --- | --- | --- | --- | --- | --- | --- | --- | --- | --- | --- | --- | --- | --- | --- | --- | --- | --- | --- | --- | --- | --- | --- | --- | --- | --- | --- | --- | --- | --- | --- | --- | --- | --- | --- | --- | --- | --- | --- | --- | --- | --- | --- | --- | --- | --- | --- | --- | --- | --- | --- | --- | --- | --- | --- | --- | --- | --- | --- | --- | --- | --- | --- | --- | --- | --- | --- | --- | --- | --- | --- | --- | --- | --- | --- | --- | --- | --- | --- | --- | --- | --- | --- | --- | --- | --- | --- | --- | --- | --- | --- | --- | --- | --- | --- | --- | --- | --- | --- | --- | --- | --- | --- | --- | --- | --- | --- | --- | --- | --- | --- | --- | --- | --- | --- | --- | --- | --- | --- | --- | --- | --- | --- | --- | --- | --- | --- | --- | --- | --- | --- | --- | --- | --- | --- | --- | --- | --- | --- | --- | --- | --- | --- | --- | --- | --- | --- | --- | --- | --- | --- | --- | --- | --- | --- | --- | --- | --- | --- | --- | --- | --- | --- | --- | --- | --- | --- | --- | --- | --- | --- | --- | --- | --- | --- | --- | --- | --- | --- | --- | --- | --- | --- | --- | --- | --- | --- | --- | --- | --- | --- | --- | --- | --- | --- | --- | --- | --- | --- | --- | --- | --- | --- | --- | --- | --- | --- | --- | --- | --- | --- | --- | --- | --- | --- | --- | --- | --- | --- | --- | --- | --- | --- | --- | --- | --- | --- | --- | --- | --- | --- | --- | --- | --- | --- | --- | --- | --- | --- | --- | --- | --- | --- | --- | --- | --- | --- | --- | --- | --- | --- | --- | --- | --- | --- | --- | --- | --- | --- | --- | --- | --- | --- | --- | --- | --- | --- | --- | --- | --- | --- | --- | --- | --- | --- | --- | --- | --- | --- | --- | --- | --- | --- | --- | --- | --- | --- | --- | --- | --- | --- | --- | --- | --- | --- | --- | --- | --- | --- | --- | --- | --- | --- | --- | --- | --- | --- | --- | --- | --- | --- | --- | --- | --- | --- | --- | --- | --- | --- | --- | --- | --- | --- | --- | --- | --- | --- | --- | --- | --- | --- | --- | --- | --- | --- | --- | --- | --- | --- | --- | --- | --- | --- | --- | --- | --- | --- | --- | --- | --- | --- | --- | --- | --- | --- | --- | --- | --- | --- | --- | --- | --- | --- | --- | --- | --- | --- | --- | --- | --- | --- | --- | --- | --- | --- | --- | --- | --- | --- | --- | --- | --- | --- | --- | --- | --- | --- | --- | --- | --- | --- | --- | --- | --- | --- | --- | --- | --- | --- | --- | --- | --- | --- | --- | --- | --- | --- | --- | --- | --- | --- | --- | --- | --- | --- | --- | --- | --- | --- | --- | --- | --- | --- | --- | --- | --- | --- | --- | --- | --- | --- | --- | --- | --- | --- | --- | --- | --- | --- | --- | --- | --- | --- | --- | --- | --- | --- | --- | --- | --- | --- | --- | --- | --- | --- | --- | --- | --- | --- | --- | --- | --- | --- | --- | --- | --- | --- | --- | --- | --- | --- | --- | --- | --- | --- | --- | --- | --- | --- | --- | --- | --- | --- | --- | --- | --- | --- | --- | --- | --- | --- | --- | --- | --- | --- | --- | --- | --- | --- | --- | --- | --- | --- | --- | --- | --- | --- | --- | --- | --- | --- | --- | --- | --- | --- | --- | --- | --- | --- | --- | --- | --- | --- | --- | --- | --- | --- | --- | --- | --- | --- | --- | --- | --- | --- | --- | --- | --- | --- | --- | --- | --- | --- | --- | --- | --- | --- | --- | --- | --- | --- | --- | --- | --- | --- | --- | --- | --- | --- | --- | --- | --- | --- | --- | --- | --- | --- | --- | --- | --- | --- | --- | --- | --- | --- | --- | --- | --- | --- | --- | --- | --- | --- | --- | --- | --- | --- | --- | --- | --- | --- | --- | --- | --- | --- | --- | --- | --- | --- | --- | --- | --- | --- | --- | --- | --- | --- | --- | --- | --- | --- | --- | --- | --- | --- | --- | --- | --- | --- | --- | --- | --- | --- | --- | --- | --- | --- | --- | --- | --- | --- | --- | --- | --- | --- | --- | --- | --- | --- | --- | --- | --- | --- | --- | --- | --- | --- | --- | --- | --- | --- | --- | --- | --- | --- | --- | --- | --- | --- | --- | --- | --- | --- | --- | --- | --- | --- | --- | --- | --- | --- | --- | --- | --- | --- | --- | --- | --- | --- | --- | --- | --- | --- | --- | --- | --- | --- | --- | --- | --- | --- | --- | --- | --- | --- | --- | --- | --- | --- | --- | --- | --- | --- | --- | --- | --- | --- | --- | --- | --- | --- | --- | --- | --- | --- | --- | --- | --- | --- | --- | --- | --- | --- | --- | --- | --- | --- | --- | --- | --- | --- | --- | --- | --- | --- | --- | --- | --- |
| |  |  |  |  |  |  |  |  |  |  |  |  |  |  |  |  |  |  |  |  |  |  |  |  |  |  |  |  |  |  |  |  |  |  |  |  |  |  |  |  |  |  |  |  |  |  |  |  |  |  |  |  |  |  |  |  |  | | --- | --- | --- | --- | --- | --- | --- | --- | --- | --- | --- | --- | --- | --- | --- | --- | --- | --- | --- | --- | --- | --- | --- | --- | --- | --- | --- | --- | --- | --- | --- | --- | --- | --- | --- | --- | --- | --- | --- | --- | --- | --- | --- | --- | --- | --- | --- | --- | --- | --- | --- | --- | --- | --- | --- | --- | --- | | G0VHR2/1-1012 | 1 | M | G | V | P | S | F | F | R | W | L | S | R | K | Y | P | K | I | I | S | P | V | L | E | E | T | P | Q | V | I | D | G | V | T | L | P | L | D | Y | S | A | P | N | P | N | G | E | L | D | N | L | Y | L | D | M | 54 | | Q6CKX0/1-992 | 1 | M | G | V | P | S | F | F | R | W | L | S | R | K | Y | P | K | I | I | S | P | V | L | E | E | Y | P | V | I | E | D | G | V | Q | L | P | L | D | Y | S | S | A | N | P | N | G | E | L | D | N | L | Y | L | D | M | 54 | | Q6FKN6/1-1018 | 1 | M | G | V | P | S | F | F | R | W | L | S | R | K | Y | P | K | I | I | S | P | V | L | E | E | P | Q | Q | L | V | D | G | V | A | L | P | I | D | Y | A | G | P | N | P | N | G | E | L | D | N | L | Y | L | D | M | 54 | | Q74ZA0/1-945 | 1 | M | G | V | P | S | F | F | R | W | L | S | R | K | Y | P | K | I | I | S | P | V | I | E | D | T | P | Q | V | V | D | G | V | K | L | P | I | D | Y | S | A | A | N | P | N | G | E | L | D | N | L | Y | L | D | M | 54 | | A7TQ00/1-1057 | 1 | M | G | V | P | S | F | F | R | W | L | S | R | K | Y | P | K | I | I | S | P | V | L | E | D | A | P | Q | V | V | D | G | V | Q | L | P | I | D | Y | S | A | P | N | Q | N | G | E | L | D | N | L | Y | L | D | M | 54 | | C5DJK2/1-982 | 1 | M | G | V | P | S | F | F | R | W | L | S | R | K | Y | P | K | I | I | S | P | V | L | E | D | T | P | Q | I | E | D | G | V | A | L | P | I | D | Y | A | G | P | N | P | N | G | E | L | D | N | L | Y | L | D | M | 54 | | C5DSC0/1-1012 | 1 | M | G | V | P | S | F | F | R | W | L | S | R | K | Y | P | K | I | I | S | P | V | I | E | E | Q | P | Q | V | V | D | G | V | T | L | P | I | D | Y | T | A | P | N | P | N | G | E | L | D | N | L | Y | L | D | M | 54 | | Kwal\_55.21625/1-974 | 1 | M | G | V | P | S | F | F | R | W | L | S | R | K | Y | P | K | I | I | S | P | V | L | E | D | T | P | Q | V | V | D | G | V | A | L | P | I | D | Y | A | G | P | N | P | N | G | E | L | D | N | L | Y | L | D | M | 54 | | Sbay\_668.45/1-1010 | 1 | M | G | V | P | S | F | F | R | W | L | S | R | K | Y | P | K | I | I | S | P | V | L | E | E | Q | P | Q | V | V | D | G | V | I | L | P | L | D | Y | S | A | P | N | P | N | G | E | L | D | N | L | Y | L | D | M | 54 | | SAKL0G01386g/1-950 | 1 | M | G | V | P | S | F | F | R | W | L | S | R | K | Y | P | K | I | I | S | P | V | L | E | D | T | P | Q | I | V | D | G | V | A | L | P | I | D | Y | S | A | P | N | P | N | G | E | L | D | N | L | Y | L | D | M | 54 | | Q02792/1-1006 | 1 | M | G | V | P | S | F | F | R | W | L | S | R | K | Y | P | K | I | I | S | P | V | L | E | E | Q | P | Q | I | V | D | G | V | I | L | P | L | D | Y | S | A | S | N | P | N | G | E | L | D | N | L | Y | L | D | M | 54 | |  | | G0VHR2/1-1012 | 55 | N | G | I | V | H | P | C | S | H | P | E | N | R | P | P | P | E | T | E | D | E | M | L | L | A | V | F | E | Y | T | N | R | V | L | N | M | A | R | P | R | K | V | L | V | M | A | V | D | G | V | A | P | R | A | 108 | | Q6CKX0/1-992 | 55 | N | G | I | V | H | P | C | S | H | P | E | N | K | P | P | P | E | T | E | D | E | M | L | L | A | V | F | E | Y | T | N | R | V | L | N | M | A | R | P | R | K | V | L | M | I | A | V | D | G | V | A | P | R | A | 108 | | Q6FKN6/1-1018 | 55 | N | G | I | V | H | P | C | S | H | P | E | N | K | P | P | P | E | T | E | D | D | M | L | L | A | V | F | E | Y | T | N | R | V | L | N | M | A | R | P | R | K | V | L | V | I | A | V | D | G | V | A | P | R | A | 108 | | Q74ZA0/1-945 | 55 | N | G | I | V | H | P | C | S | H | P | E | N | K | P | A | P | E | T | E | D | E | M | L | L | A | V | F | E | Y | T | N | R | V | L | N | M | A | R | P | R | K | V | L | M | I | A | V | D | G | V | A | P | R | A | 108 | | A7TQ00/1-1057 | 55 | N | G | I | V | H | P | C | S | H | P | E | N | K | P | P | P | E | N | E | D | E | M | L | L | A | V | F | E | Y | T | N | R | V | L | N | M | A | R | P | R | K | V | L | M | I | A | V | D | G | V | A | P | R | A | 108 | | C5DJK2/1-982 | 55 | N | G | I | V | H | P | C | S | H | P | E | N | R | P | P | P | E | T | E | D | E | M | L | L | A | V | F | E | Y | T | N | R | V | L | N | M | A | R | P | R | K | V | L | M | I | A | V | D | G | V | A | P | R | A | 108 | | C5DSC0/1-1012 | 55 | N | G | I | V | H | P | C | S | H | P | E | N | K | P | P | P | E | T | E | D | E | M | L | L | A | V | F | E | Y | T | N | R | V | L | N | M | A | R | P | R | K | V | L | M | I | A | V | D | G | V | A | P | R | A | 108 | | Kwal\_55.21625/1-974 | 55 | N | G | I | V | H | P | C | S | H | P | E | N | R | P | P | P | E | T | E | D | E | M | L | L | A | V | F | E | Y | T | N | R | V | L | N | M | A | R | P | R | K | V | L | M | I | A | V | D | G | V | A | P | R | A | 108 | | Sbay\_668.45/1-1010 | 55 | N | G | I | V | H | P | C | S | H | P | E | N | K | P | P | P | E | T | E | D | E | M | L | L | A | V | F | E | Y | T | N | R | V | L | N | M | A | R | P | R | K | V | L | V | M | A | V | D | G | V | A | P | R | A | 108 | | SAKL0G01386g/1-950 | 55 | N | G | I | V | H | P | C | S | H | P | E | N | K | P | P | P | E | T | E | D | E | M | L | L | A | V | F | E | Y | T | N | R | V | L | N | M | A | R | P | R | K | V | L | M | I | A | V | D | G | V | A | P | R | A | 108 | | Q02792/1-1006 | 55 | N | G | I | V | H | P | C | S | H | P | E | N | K | P | P | P | E | T | E | D | E | M | L | L | A | V | F | E | Y | T | N | R | V | L | N | M | A | R | P | R | K | V | L | V | M | A | V | D | G | V | A | P | R | A | 108 | |  | | G0VHR2/1-1012 | 109 | K | M | N | Q | Q | R | A | R | R | F | R | S | A | R | D | A | A | I | E | N | E | A | R | E | E | I | L | R | Q | K | E | E | I | G | E | V | I | D | E | A | V | K | N | K | K | T | W | D | S | N | A | I | T | P | 162 | | Q6CKX0/1-992 | 109 | K | M | N | Q | Q | R | A | R | R | F | R | S | A | R | D | A | K | L | Q | N | E | A | R | E | Q | V | L | R | E | R | E | D | Y | G | E | T | I | D | E | N | V | K | S | K | K | T | W | D | S | N | A | I | T | P | 162 | | Q6FKN6/1-1018 | 109 | K | M | N | Q | Q | R | S | R | R | F | R | S | A | R | D | A | E | I | E | N | E | A | R | E | E | I | M | R | Q | K | E | Q | L | G | Q | I | I | D | D | S | V | K | N | K | K | T | W | D | S | N | A | I | T | P | 162 | | Q74ZA0/1-945 | 109 | K | M | N | Q | Q | R | S | R | R | F | R | S | A | R | D | A | K | L | A | N | E | E | K | A | R | V | L | A | E | R | E | A | Y | G | E | M | I | D | D | A | V | K | A | K | K | S | W | D | T | N | A | I | T | P | 162 | | A7TQ00/1-1057 | 109 | K | M | N | Q | Q | R | A | R | R | F | R | S | A | R | D | A | K | I | Q | D | E | A | R | E | E | I | M | R | E | K | E | E | I | G | E | H | I | D | E | S | V | R | N | K | K | T | W | D | S | N | A | I | T | P | 162 | | C5DJK2/1-982 | 109 | K | M | N | Q | Q | R | A | R | R | F | R | S | A | R | D | A | Q | I | Q | N | E | E | R | E | R | V | L | L | E | K | E | D | M | G | E | I | I | D | D | S | V | R | N | K | K | T | W | D | S | N | A | I | T | P | 162 | | C5DSC0/1-1012 | 109 | K | M | N | Q | Q | R | A | R | R | F | R | S | A | K | D | A | Q | I | E | N | E | N | R | E | R | I | M | Q | E | R | E | Q | L | G | E | I | I | D | G | A | V | K | T | K | K | T | W | D | S | N | A | I | T | P | 162 | | Kwal\_55.21625/1-974 | 109 | K | M | N | Q | Q | R | A | R | R | F | R | S | A | R | D | A | Q | I | Q | N | E | E | R | E | R | V | L | L | E | K | E | D | L | G | E | V | I | D | E | S | V | R | N | K | K | T | W | D | S | N | A | I | T | P | 162 | | Sbay\_668.45/1-1010 | 109 | K | M | N | Q | Q | R | A | R | R | F | R | S | A | R | D | A | Q | V | E | N | E | A | R | E | E | V | M | R | Q | R | E | E | V | G | E | I | I | D | D | A | V | R | N | K | K | T | W | D | S | N | A | I | T | P | 162 | | SAKL0G01386g/1-950 | 109 | K | M | N | Q | Q | R | A | R | R | F | R | S | A | R | D | A | E | I | Q | N | E | E | R | E | R | V | L | Q | E | R | E | D | F | G | E | I | I | D | D | S | V | R | N | K | K | T | W | D | S | N | A | I | T | P | 162 | | Q02792/1-1006 | 109 | K | M | N | Q | Q | R | A | R | R | F | R | S | A | R | D | A | Q | I | E | N | E | A | R | E | E | I | M | R | Q | R | E | E | V | G | E | I | I | D | D | A | V | R | N | K | K | T | W | D | S | N | A | I | T | P | 162 | |  | | G0VHR2/1-1012 | 163 | G | T | P | F | M | D | K | L | A | A | S | L | R | Y | W | T | A | F | K | L | A | T | D | P | G | W | K | N | L | Q | I | I | I | S | D | A | T | V | P | G | E | G | E | H | K | I | M | N | F | I | R | S | Q | R | 216 | | Q6CKX0/1-992 | 163 | G | T | P | F | M | D | K | L | A | T | A | L | R | Y | W | T | S | F | K | L | A | T | D | P | G | W | K | N | L | Q | I | I | I | S | D | A | T | V | P | G | E | G | E | H | K | I | M | N | F | I | R | S | Q | R | 216 | | Q6FKN6/1-1018 | 163 | G | T | P | F | M | D | K | L | A | I | A | L | R | Y | W | T | A | F | K | L | A | T | D | P | G | W | K | N | L | Q | V | I | I | S | D | A | T | V | P | G | E | G | E | H | K | I | M | N | F | I | R | S | Q | R | 216 | | Q74ZA0/1-945 | 163 | G | T | P | F | M | D | K | L | A | A | A | L | R | Y | W | T | S | F | K | L | A | T | D | P | G | W | K | N | L | Q | V | I | I | S | D | A | T | V | P | G | E | G | E | H | K | I | M | N | F | I | R | S | Q | R | 216 | | A7TQ00/1-1057 | 163 | G | T | P | F | M | D | K | L | A | A | A | L | R | Y | W | T | A | F | K | L | A | T | D | P | G | W | K | N | L | Q | I | I | I | S | D | A | T | V | P | G | E | G | E | H | K | I | M | N | F | I | R | S | Q | R | 216 | | C5DJK2/1-982 | 163 | G | T | P | F | M | D | K | L | A | A | A | L | R | Y | W | T | S | F | K | L | A | T | D | P | G | W | K | N | L | Q | V | I | I | S | D | A | T | V | P | G | E | G | E | H | K | I | M | N | F | V | R | S | Q | R | 216 | | C5DSC0/1-1012 | 163 | G | T | P | F | M | D | K | L | A | A | A | L | R | Y | W | C | S | F | K | L | A | T | D | P | G | W | K | N | L | Q | V | I | I | S | D | A | T | V | P | G | E | G | E | H | K | I | M | N | F | V | R | S | Q | R | 216 | | Kwal\_55.21625/1-974 | 163 | G | T | P | F | M | D | K | L | A | A | A | L | R | Y | W | T | S | F | K | L | S | T | D | P | G | W | K | N | L | Q | V | I | I | S | D | A | T | V | P | G | E | G | E | H | K | I | M | N | F | V | R | S | Q | R | 216 | | Sbay\_668.45/1-1010 | 163 | G | T | P | F | M | D | K | L | A | A | A | L | R | Y | W | T | A | F | K | L | A | T | D | P | G | W | K | N | L | Q | V | I | I | S | D | A | T | V | P | G | E | G | E | H | K | I | M | N | F | I | R | S | Q | R | 216 | | SAKL0G01386g/1-950 | 163 | G | T | P | F | M | D | K | L | A | S | A | L | R | Y | W | T | S | F | K | L | A | T | D | P | G | W | K | G | L | Q | V | I | I | S | D | A | T | V | P | G | E | G | E | H | K | I | M | N | F | I | R | S | Q | R | 216 | | Q02792/1-1006 | 163 | G | T | P | F | M | D | K | L | A | A | A | L | R | Y | W | T | A | F | K | L | A | T | D | P | G | W | K | N | L | Q | V | I | I | S | D | A | T | V | P | G | E | G | E | H | K | I | M | N | F | I | R | S | Q | R | 216 | |  | | G0VHR2/1-1012 | 217 | A | D | P | E | Y | N | P | N | T | T | H | C | I | Y | G | L | D | A | D | L | I | F | L | G | L | A | T | H | E | P | H | F | R | I | L | R | E | D | V | F | A | Q | - | N | N | N | K | R | R | N | F | D | D | T | 269 | | Q6CKX0/1-992 | 217 | A | D | T | Q | Y | N | P | N | T | T | H | C | I | Y | G | L | D | A | D | L | I | F | L | G | L | A | T | H | E | P | H | F | K | I | L | R | E | D | V | F | A | N | - | N | N | Y | K | K | P | K | P | Q | D | M | 269 | | Q6FKN6/1-1018 | 217 | A | D | P | E | Y | N | P | N | T | T | H | C | I | Y | G | L | D | A | D | L | I | F | L | G | L | A | T | H | E | P | H | F | K | I | L | R | E | D | V | F | A | Q | - | D | N | R | K | R | N | N | V | K | D | T | 269 | | Q74ZA0/1-945 | 217 | A | D | V | Q | Y | N | P | N | T | T | H | C | I | Y | G | L | D | A | D | L | I | F | L | G | L | A | T | H | E | P | H | F | K | I | L | R | E | D | V | F | A | Q | - | D | N | R | R | R | P | R | Q | H | D | M | 269 | | A7TQ00/1-1057 | 217 | A | D | P | E | Y | N | P | N | T | T | H | C | I | Y | G | L | D | A | D | L | I | F | L | G | L | A | T | H | E | P | H | F | K | I | L | R | E | D | V | F | A | Q | G | N | N | R | K | R | H | N | F | K | D | N | 270 | | C5DJK2/1-982 | 217 | A | D | P | Q | Y | N | P | N | T | S | H | C | I | Y | G | L | D | A | D | L | I | F | L | G | L | A | T | H | E | P | H | F | K | I | L | R | E | D | V | F | A | Q | - | D | N | R | R | R | Q | R | V | Q | D | T | 269 | | C5DSC0/1-1012 | 217 | A | D | A | Q | Y | N | P | N | T | T | H | C | I | Y | G | L | D | A | D | L | I | F | L | G | L | A | T | H | E | P | H | F | K | V | L | R | E | D | V | F | A | Q | - | G | N | N | K | R | H | N | L | K | D | S | 269 | | Kwal\_55.21625/1-974 | 217 | A | D | P | Q | Y | N | P | N | T | S | H | C | I | Y | G | L | D | A | D | L | I | F | L | G | L | A | T | H | E | P | H | F | K | I | L | R | E | D | V | F | A | Q | - | D | N | R | R | R | Q | R | V | Q | D | T | 269 | | Sbay\_668.45/1-1010 | 217 | A | D | P | E | Y | N | P | N | T | T | H | C | I | Y | G | L | D | A | D | L | I | F | L | G | L | A | T | H | E | P | H | F | K | I | L | R | E | D | V | F | A | Q | - | D | N | R | K | R | N | N | F | K | D | T | 269 | | SAKL0G01386g/1-950 | 217 | S | D | P | Q | Y | N | P | N | T | T | H | C | I | Y | G | L | D | A | D | L | I | F | L | G | L | A | T | H | E | P | H | F | K | I | L | R | E | D | V | F | A | Q | - | D | N | R | R | K | P | N | F | K | D | Q | 269 | | Q02792/1-1006 | 217 | A | D | P | E | Y | N | P | N | T | T | H | C | I | Y | G | L | D | A | D | L | I | F | L | G | L | A | T | H | E | P | H | F | K | I | L | R | E | D | V | F | A | Q | - | D | N | R | K | R | N | N | L | K | D | T | 269 | |  | | G0VHR2/1-1012 | 270 | L | N | M | S | E | E | E | K | Q | L | L | L | A | K | D | S | E | K | P | F | L | W | L | H | I | N | V | L | R | E | Y | L | G | A | E | L | F | I | P | R | L | S | F | P | F | D | L | E | R | A | I | D | D | W | 323 | | Q6CKX0/1-992 | 270 | I | N | L | S | E | E | E | K | Q | Q | L | I | Q | Q | D | S | E | K | P | F | L | W | L | H | I | S | V | L | R | E | Y | L | S | A | E | L | A | I | P | H | L | S | F | Q | F | D | F | E | R | A | I | D | D | W | 323 | | Q6FKN6/1-1018 | 270 | I | D | M | T | D | E | E | K | D | L | I | R | K | Q | D | S | E | K | P | F | L | W | L | H | I | S | V | L | R | E | Y | L | S | A | E | L | W | T | P | K | L | P | F | P | F | D | L | E | R | A | I | D | D | W | 323 | | Q74ZA0/1-945 | 270 | M | D | M | S | A | E | E | K | Q | A | L | I | E | Q | D | A | E | K | P | F | L | W | L | H | V | S | V | L | R | E | Y | L | S | A | E | L | L | V | P | R | L | A | F | H | F | D | L | E | R | A | I | D | D | W | 323 | | A7TQ00/1-1057 | 271 | I | N | M | T | E | E | E | K | Q | I | I | M | K | E | D | S | Q | K | P | F | L | W | L | H | I | S | V | L | R | E | Y | L | S | A | E | L | W | V | Q | R | L | P | F | P | F | D | L | E | R | A | I | D | D | W | 324 | | C5DJK2/1-982 | 270 | I | N | M | S | E | E | D | K | A | A | L | L | Q | Q | D | S | Q | K | P | F | L | W | L | H | I | S | V | L | R | E | Y | L | A | V | E | L | F | V | P | R | L | S | F | E | F | D | T | E | R | A | V | D | D | W | 323 | | C5DSC0/1-1012 | 270 | L | N | M | S | E | E | E | K | Q | M | I | A | K | Q | D | S | E | K | P | F | L | W | L | H | I | S | V | L | R | E | Y | L | S | A | E | L | W | V | P | R | M | P | F | P | F | D | L | E | R | A | I | D | D | W | 323 | | Kwal\_55.21625/1-974 | 270 | I | N | M | T | E | D | E | K | S | A | L | V | Q | Q | D | S | E | K | P | F | L | W | L | H | I | S | V | L | R | E | Y | L | A | V | E | L | F | V | P | R | L | S | F | E | F | D | T | E | R | A | I | D | D | W | 323 | | Sbay\_668.45/1-1010 | 270 | I | N | M | T | E | E | E | K | E | F | L | Q | K | Q | N | S | E | Q | P | F | L | W | L | H | I | N | V | L | R | E | Y | L | S | A | E | L | W | V | P | G | L | P | F | T | F | D | I | E | R | A | I | D | D | W | 323 | | SAKL0G01386g/1-950 | 270 | L | N | M | S | E | E | E | K | Q | I | L | M | K | Q | D | S | E | K | P | F | L | W | L | H | I | S | V | L | R | E | Y | L | S | V | E | L | S | V | P | R | L | S | F | P | F | D | I | E | R | A | V | D | D | W | 323 | | Q02792/1-1006 | 270 | I | N | M | T | E | E | E | K | Q | F | L | Q | K | Q | N | S | E | Q | P | F | L | W | L | H | I | N | V | L | R | E | Y | L | S | A | E | L | W | V | P | G | L | P | F | T | F | D | L | E | R | A | I | D | D | W | 323 | |  | | G0VHR2/1-1012 | 324 | V | F | M | C | F | F | C | G | N | D | F | L | P | H | L | P | S | L | D | V | R | E | N | S | I | D | I | L | L | D | I | W | K | I | V | L | P | N | L | K | T | Y | M | T | C | D | G | E | L | N | L | E | S | V | 377 | | Q6CKX0/1-992 | 324 | V | F | M | C | F | F | C | G | N | D | F | L | P | H | L | P | C | L | D | V | R | E | N | S | I | D | I | L | V | D | I | W | K | T | V | L | P | K | T | K | T | Y | L | T | C | D | G | T | L | N | L | E | P | V | 377 | | Q6FKN6/1-1018 | 324 | V | F | M | C | F | F | C | G | N | D | F | L | P | H | L | P | C | L | D | V | R | E | N | S | I | D | I | L | L | D | I | W | K | S | I | L | P | R | L | K | T | Y | M | T | C | D | G | K | L | N | L | E | S | V | 377 | | Q74ZA0/1-945 | 324 | V | F | M | C | F | F | C | G | N | D | F | L | P | H | L | P | S | L | D | V | R | E | N | S | I | D | I | L | V | D | I | W | K | A | V | L | P | S | L | K | T | Y | L | T | C | D | G | K | L | N | L | K | G | V | 377 | | A7TQ00/1-1057 | 325 | V | F | M | C | F | F | C | G | N | D | F | L | P | H | L | P | S | L | D | V | R | E | N | S | I | D | I | L | L | D | I | W | K | V | V | L | P | N | L | K | D | Y | M | T | C | D | G | E | L | N | L | A | S | V | 378 | | C5DJK2/1-982 | 324 | V | F | M | C | F | F | C | G | N | D | F | L | P | H | L | P | S | L | D | V | R | E | N | S | I | D | I | L | V | E | I | W | K | T | I | L | P | R | L | K | T | Y | M | T | C | D | G | E | L | N | L | D | S | V | 377 | | C5DSC0/1-1012 | 324 | V | F | M | C | F | F | C | G | N | D | F | L | P | H | L | P | C | L | D | V | R | E | N | S | I | D | I | L | L | D | I | W | K | V | V | L | P | T | M | K | T | Y | I | T | C | D | G | E | L | N | L | E | S | V | 377 | | Kwal\_55.21625/1-974 | 324 | V | F | M | C | F | F | C | G | N | D | F | L | P | H | L | P | S | L | D | V | R | E | N | S | I | D | I | L | V | E | I | W | K | A | I | L | P | Q | L | K | T | Y | M | T | C | D | G | E | L | N | L | G | S | V | 377 | | Sbay\_668.45/1-1010 | 324 | V | F | M | C | F | F | C | G | N | D | F | L | P | H | L | P | C | L | D | V | R | E | N | S | I | D | I | L | L | D | I | W | K | V | V | L | P | R | L | K | T | Y | M | T | C | D | G | V | L | N | M | P | S | V | 377 | | SAKL0G01386g/1-950 | 324 | V | F | M | C | F | F | C | G | N | D | F | L | P | H | L | P | S | L | D | V | R | E | N | S | I | D | I | L | V | E | I | W | K | A | V | L | P | R | L | K | D | Y | M | T | C | D | G | E | L | N | L | E | S | V | 377 | | Q02792/1-1006 | 324 | V | F | M | C | F | F | C | G | N | D | F | L | P | H | L | P | C | L | D | V | R | E | N | S | I | D | I | L | L | D | I | W | K | V | V | L | P | K | L | K | T | Y | M | T | C | D | G | V | L | N | L | P | S | V | 377 | |  | | G0VHR2/1-1012 | 378 | E | M | L | L | Q | Q | L | G | S | R | E | G | D | I | F | K | S | R | H | I | Q | E | V | R | K | Q | E | A | Y | Q | R | K | K | L | Q | K | - | N | G | K | F | S | N | G | Q | - | D | R | H | P | T | T | P | N | 429 | | Q6CKX0/1-992 | 378 | E | A | L | L | E | Q | L | G | D | R | E | T | D | L | F | K | K | K | Y | I | Q | E | V | R | K | Q | E | A | H | D | R | R | K | K | L | K | S | N | P | N | V | S | Q | G | K | V | D | R | N | F | M | I | P | L | 431 | | Q6FKN6/1-1018 | 378 | E | M | V | L | K | E | L | G | N | R | E | G | D | I | F | K | T | R | H | I | Q | E | I | R | K | K | E | A | N | E | R | R | K | Q | Q | K | - | Q | Q | N | V | S | T | G | Q | - | D | R | H | P | T | K | F | N | 429 | | Q74ZA0/1-945 | 378 | E | K | V | L | A | Q | L | G | N | R | E | S | D | I | F | K | K | K | Y | E | Q | E | L | R | K | K | E | A | Q | E | R | R | K | M | L | K | N | N | P | N | V | T | K | G | K | V | D | R | N | F | T | V | P | L | 431 | | A7TQ00/1-1057 | 379 | E | K | L | L | Y | Q | L | G | S | R | E | G | D | I | F | K | T | R | H | I | Q | E | I | R | K | Q | E | A | L | Q | R | R | K | L | G | N | - | Q | K | N | L | S | Q | G | Q | - | D | R | H | P | T | Q | F | N | 430 | | C5DJK2/1-982 | 378 | E | L | L | L | Q | H | L | G | A | R | E | P | Q | M | F | K | D | R | F | Q | Q | E | I | R | K | Q | E | A | A | K | R | R | K | M | L | K | S | N | N | N | V | T | K | G | K | T | D | R | N | F | T | I | P | L | 431 | | C5DSC0/1-1012 | 378 | E | K | L | L | S | Q | L | G | S | R | E | A | D | I | F | K | T | R | H | I | Q | E | V | R | K | Q | E | N | F | E | R | K | K | A | Q | R | - | - | - | N | V | S | K | G | Q | - | D | R | H | P | T | K | P | N | 427 | | Kwal\_55.21625/1-974 | 378 | E | L | L | L | Q | H | L | G | A | R | E | P | E | M | F | K | G | R | Y | L | Q | E | I | R | K | Q | E | A | A | K | R | R | K | M | L | K | S | N | N | N | V | T | K | G | K | T | D | R | N | F | T | A | P | L | 431 | | Sbay\_668.45/1-1010 | 378 | E | T | L | L | Q | H | L | G | S | R | E | G | D | I | F | K | T | R | H | I | Q | E | A | R | K | K | E | A | F | E | R | R | K | A | Q | K | - | - | - | N | M | S | K | G | Q | - | D | R | H | P | I | V | A | T | 427 | | SAKL0G01386g/1-950 | 378 | E | L | L | L | Q | Q | L | G | A | R | E | P | E | V | F | K | S | R | H | V | Q | E | V | R | K | Q | E | A | L | K | R | R | K | L | M | K | N | N | N | N | V | T | K | G | K | T | D | R | N | F | T | V | P | L | 431 | | Q02792/1-1006 | 378 | E | T | L | L | Q | H | L | G | S | R | E | G | D | I | F | K | T | R | H | I | Q | E | A | R | K | K | E | A | F | E | R | R | K | A | Q | K | - | - | - | N | M | S | K | G | Q | - | D | R | H | P | T | V | A | T | 427 | |  | | G0VHR2/1-1012 | 430 | E | Q | L | Q | M | Y | D | T | N | G | E | L | A | K | G | S | W | N | L | T | T | H | D | M | V | R | L | K | K | Q | L | M | L | A | N | E | G | D | V | D | A | I | A | V | I | K | A | E | S | E | K | N | D | K | 483 | | Q6CKX0/1-992 | 432 | E | N | M | P | V | Y | D | V | D | G | N | A | A | E | G | S | L | N | L | S | N | K | D | F | A | N | M | R | K | E | I | N | L | A | N | E | G | D | G | E | A | A | K | A | L | K | L | K | S | E | K | N | S | G | 485 | | Q6FKN6/1-1018 | 430 | E | Q | L | Q | M | Y | D | T | N | G | S | L | A | K | G | S | W | N | L | T | T | S | D | M | V | R | Y | K | K | E | L | M | L | A | N | E | G | D | E | N | S | I | K | I | I | E | E | V | S | E | R | N | N | S | 483 | | Q74ZA0/1-945 | 432 | E | S | M | P | V | F | D | V | N | G | N | A | A | A | G | S | L | N | L | T | N | K | D | F | A | N | M | R | K | E | I | T | L | A | N | E | G | N | T | K | A | L | D | A | L | K | R | R | S | D | E | N | R | T | 485 | | A7TQ00/1-1057 | 431 | E | Q | L | Q | L | Y | D | T | N | G | N | L | A | K | G | S | W | N | L | T | T | S | D | M | V | K | F | K | K | E | L | M | L | A | N | E | G | D | E | E | A | I | K | V | I | K | A | Q | S | D | L | N | N | K | 484 | | C5DJK2/1-982 | 432 | E | N | M | P | V | Y | D | V | S | G | N | A | A | D | G | S | L | N | L | S | N | R | D | F | V | K | L | R | K | E | M | N | L | A | N | E | G | D | K | K | S | A | A | A | L | K | I | Q | S | E | Q | N | T | Q | 485 | | C5DSC0/1-1012 | 428 | E | Q | L | Q | L | Y | D | T | N | G | N | L | S | K | S | S | W | N | L | T | T | H | D | M | V | N | L | K | K | E | I | M | L | A | N | E | G | D | A | N | A | I | A | T | V | K | A | Q | S | D | K | N | D | Q | 481 | | Kwal\_55.21625/1-974 | 432 | E | N | L | P | I | Y | D | V | T | G | N | A | A | D | G | S | L | N | L | S | N | R | D | F | V | K | L | R | K | E | M | N | L | A | N | E | G | D | K | E | S | A | A | A | L | K | T | Q | S | A | Q | N | T | K | 485 | | Sbay\_668.45/1-1010 | 428 | E | Q | L | Q | M | Y | D | T | R | G | N | L | A | K | G | S | W | N | L | T | T | S | D | M | V | R | L | R | R | E | L | M | L | A | N | E | G | D | E | K | A | I | A | T | V | K | E | Q | S | D | K | N | N | S | 481 | | SAKL0G01386g/1-950 | 432 | E | N | M | P | L | F | D | V | D | G | N | S | A | K | G | S | L | N | L | S | N | S | D | F | V | K | L | R | K | E | M | N | L | A | N | E | G | D | K | K | S | A | A | A | L | K | S | K | S | E | E | N | T | R | 485 | | Q02792/1-1006 | 428 | E | Q | L | Q | M | Y | D | T | Q | G | N | L | A | K | G | S | W | N | L | T | T | S | D | M | V | R | L | K | K | E | L | M | L | A | N | E | G | N | E | E | A | I | A | K | V | K | Q | Q | S | D | K | N | N | E | 481 | |  | | G0VHR2/1-1012 | 484 | L | - | - | - | - | M | K | - | E | I | T | E | E | Q | M | D | K | V | V | Q | E | A | N | K | S | N | F | T | A | A | E | I | M | K | K | K | L | Q | A | R | K | H | Q | L | E | E | E | - | - | - | - | - | E | - | 526 | | Q6CKX0/1-992 | 486 | V | - | - | - | - | V | - | - | E | F | S | S | E | E | L | K | N | S | V | Q | L | S | K | T | A | N | Y | D | A | A | T | S | L | Q | E | K | L | I | A | K | K | M | A | M | R | D | E | - | - | - | - | - | E | - | 527 | | Q6FKN6/1-1018 | 484 | L | - | - | - | - | M | K | - | E | I | - | Q | S | E | M | T | P | E | D | Y | K | G | N | N | N | N | F | T | A | A | E | L | L | K | K | K | L | N | A | R | K | R | E | L | E | K | E | - | - | - | - | - | K | - | 525 | | Q74ZA0/1-945 | 486 | Q | R | T | S | D | T | R | - | D | F | S | Q | E | E | L | E | S | A | I | E | V | S | K | G | D | N | L | S | A | A | A | S | L | R | E | R | I | L | A | K | R | K | A | A | E | D | P | - | - | - | - | - | E | - | 532 | | A7TQ00/1-1057 | 485 | L | - | - | - | - | E | N | - | E | I | N | K | E | K | E | E | E | A | I | N | Q | G | N | I | A | N | F | S | A | V | D | T | M | K | K | K | L | T | E | R | K | R | Q | L | E | Q | E | - | - | - | - | - | E | - | 527 | | C5DJK2/1-982 | 486 | P | - | - | - | - | T | K | - | D | Y | T | K | E | E | I | S | S | A | V | E | V | A | N | E | A | N | F | S | A | A | S | S | L | K | Q | K | L | I | E | R | K | A | E | A | V | Q | E | - | - | - | - | - | N | - | 528 | | C5DSC0/1-1012 | 482 | L | - | - | - | - | M | N | - | E | L | M | N | E | Q | M | D | K | A | V | D | E | A | N | K | T | N | F | T | A | A | E | V | M | K | K | K | L | V | A | K | K | R | R | L | E | A | E | - | - | - | - | - | E | - | 524 | | Kwal\_55.21625/1-974 | 486 | P | - | - | - | - | T | K | - | V | Y | T | K | E | E | V | S | S | A | V | D | I | A | N | D | A | N | F | S | A | A | A | M | M | K | K | K | L | L | E | Q | A | S | E | A | N | N | G | - | - | - | - | - | D | - | 528 | | Sbay\_668.45/1-1010 | 482 | L | - | - | - | - | M | K | - | D | I | S | K | E | E | I | D | N | A | V | N | K | A | N | K | S | N | F | N | L | A | E | V | M | K | Q | K | L | I | N | K | K | H | H | L | E | K | D | - | - | - | - | - | N | E | 525 | | SAKL0G01386g/1-950 | 486 | G | - | - | - | - | T | S | I | E | F | S | K | E | E | L | N | N | A | I | E | V | A | N | K | D | N | F | S | A | A | A | I | M | K | Q | K | L | L | A | K | K | Q | E | Q | E | Q | E | G | P | G | K | Q | N | - | 534 | | Q02792/1-1006 | 482 | L | - | - | - | - | M | K | - | D | I | S | K | E | E | I | D | D | A | V | S | K | A | N | K | T | N | F | N | L | A | E | V | M | K | Q | K | I | I | N | K | K | H | R | L | E | K | D | - | - | - | - | - | N | - | 524 | |  | | G0VHR2/1-1012 | 527 | K | A | E | A | E | K | D | - | - | - | - | - | V | V | T | K | K | P | L | T | E | E | K | H | L | S | E | E | L | T | N | E | I | E | - | - | - | - | - | - | - | - | - | - | - | - | N | D | V | E | D | E | D | A | 563 | | Q6CKX0/1-992 | 528 | N | A | E | E | L | S | - | - | - | - | - | - | L | K | R | K | S | E | D | V | D | S | A | E | K | E | T | S | N | E | P | E | - | - | - | - | - | - | - | - | - | - | - | - | - | - | D | D | E | A | D | Y | D | E | 561 | | Q6FKN6/1-1018 | 526 | E | N | E | E | Q | E | - | - | - | - | - | - | R | A | S | K | Q | P | K | I | S | E | T | P | D | E | S | D | L | T | E | E | I | E | A | D | V | I | - | - | - | - | - | - | - | - | A | E | V | E | D | E | T | T | 565 | | Q74ZA0/1-945 | 533 | G | A | P | E | P | K | - | - | - | - | - | - | N | P | H | K | A | P | S | A | S | E | T | E | A | A | T | P | A | S | S | E | T | A | - | - | - | - | - | - | - | - | - | - | - | - | - | - | - | - | - | - | - | - | 560 | | A7TQ00/1-1057 | 528 | N | E | D | K | M | K | N | E | - | - | - | - | K | D | T | K | K | P | K | T | E | S | K | E | D | E | - | - | L | T | N | D | I | E | A | E | I | V | A | E | - | V | A | D | E | L | N | D | E | E | G | E | D | G | 574 | | C5DJK2/1-982 | 529 | G | E | D | K | E | N | - | - | - | - | - | - | V | E | E | S | E | K | P | M | N | D | A | I | K | R | K | F | E | E | V | D | D | P | - | - | - | - | - | - | - | - | - | - | - | - | E | E | S | T | S | V | D | A | 564 | | C5DSC0/1-1012 | 525 | E | E | Q | R | Q | E | E | Q | K | E | E | S | R | K | K | K | K | T | E | E | E | A | T | K | E | E | P | N | L | D | D | Q | I | K | A | E | L | E | E | E | R | I | E | E | E | E | E | E | E | E | G | Q | D | Q | 578 | | Kwal\_55.21625/1-974 | 529 | N | V | T | D | E | N | - | - | - | - | - | - | I | E | P | L | R | E | E | G | G | N | D | F | K | R | K | F | E | E | M | E | - | - | - | - | - | - | - | - | - | - | - | - | - | - | - | D | S | P | S | V | D | V | 561 | | Sbay\_668.45/1-1010 | 526 | E | E | E | E | T | T | - | - | - | - | - | - | N | S | S | K | K | T | K | P | E | K | T | K | S | E | S | D | L | D | T | E | I | K | A | E | I | E | - | - | - | - | - | - | - | - | V | D | V | N | D | R | E | D | 565 | | SAKL0G01386g/1-950 | 535 | G | A | E | S | V | G | - | - | - | - | - | - | N | K | E | K | E | E | D | A | G | N | S | L | K | R | N | R | S | D | T | E | T | P | - | - | - | - | - | - | - | - | - | - | - | - | E | D | N | E | G | N | G | G | 570 | | Q02792/1-1006 | 525 | E | E | E | E | I | A | - | - | - | - | - | - | K | D | S | K | K | V | K | T | E | K | A | E | S | E | C | D | L | D | A | E | I | K | D | E | I | V | - | - | - | - | - | - | - | - | A | D | V | N | D | R | E | N | 564 | |  | | G0VHR2/1-1012 | 564 | - | E | E | S | K | - | T | T | S | E | N | E | S | V | P | A | V | S | L | G | P | V | K | S | G | V | I | D | T | D | E | A | V | K | L | Y | E | P | G | F | R | E | R | Y | Y | K | A | K | C | Y | V | S | D | A | 615 | | Q6CKX0/1-992 | 562 | - | - | - | - | - | - | D | E | G | S | T | V | P | P | A | V | I | H | T | G | I | V | K | S | G | F | I | D | T | D | E | S | V | R | L | Y | E | P | G | Y | H | D | R | Y | Y | Q | H | K | F | H | V | P | A | K | 609 | | Q6FKN6/1-1018 | 566 | P | D | E | K | D | - | T | D | S | I | I | T | E | V | P | E | I | S | N | G | G | I | T | S | G | V | I | D | T | D | E | A | V | K | L | F | E | P | G | Y | H | D | R | Y | Y | I | E | K | F | H | I | E | P | N | 618 | | Q74ZA0/1-945 | 561 | - | - | - | - | - | - | - | - | - | S | Q | V | D | I | P | - | I | S | T | G | M | L | T | P | G | F | L | D | T | D | T | G | V | R | L | F | E | P | G | Y | H | D | R | Y | Y | Q | A | K | F | H | V | P | T | G | 604 | | A7TQ00/1-1057 | 575 | - | E | S | G | Q | N | T | E | D | T | N | D | V | V | P | G | I | S | P | F | E | G | T | S | G | V | I | D | T | D | E | S | V | R | L | Y | E | P | G | Y | H | S | R | Y | Y | T | S | K | F | N | I | A | E | E | 627 | | C5DJK2/1-982 | 565 | - | - | - | - | - | - | E | S | Q | T | P | S | S | I | S | V | V | H | T | G | N | V | K | S | G | V | V | D | T | D | E | E | V | K | L | F | E | P | G | Y | H | D | R | Y | Y | T | A | K | F | H | I | D | E | R | 612 | | C5DSC0/1-1012 | 579 | - | E | E | E | Q | - | G | E | K | P | P | D | W | V | P | E | V | S | N | G | G | I | T | S | G | I | V | D | T | D | Q | A | V | K | L | H | E | P | G | Y | H | E | R | Y | Y | T | E | K | F | H | I | S | P | N | 630 | | Kwal\_55.21625/1-974 | 562 | - | - | - | - | - | - | D | S | Q | T | P | A | S | I | T | V | V | H | T | G | N | V | Q | S | G | V | I | D | T | D | E | E | V | K | L | F | E | P | G | Y | H | D | R | Y | Y | T | A | K | F | H | V | N | S | S | 609 | | Sbay\_668.45/1-1010 | 566 | T | E | S | T | E | V | S | R | D | S | P | A | R | N | T | I | V | V | G | E | A | P | Q | N | G | V | F | D | T | D | E | Y | V | R | L | F | E | P | G | Y | H | E | R | Y | Y | T | A | K | F | R | I | A | P | Q | 619 | | SAKL0G01386g/1-950 | 571 | - | - | - | - | - | - | E | E | E | S | T | E | N | T | P | I | I | N | T | P | S | I | K | S | G | V | M | D | T | D | E | M | V | R | L | Y | E | P | G | Y | H | D | R | Y | Y | Q | A | K | F | R | V | S | A | S | 618 | | Q02792/1-1006 | 565 | S | E | T | T | E | V | S | R | D | S | P | V | H | S | T | V | N | V | S | E | G | P | K | N | G | V | F | D | T | D | E | F | V | K | L | F | E | P | G | Y | H | E | R | Y | Y | T | A | K | F | H | V | T | P | Q | 618 | |  | | G0VHR2/1-1012 | 616 | E | I | E | P | L | R | K | G | M | V | R | S | Y | V | E | G | V | S | W | V | L | L | Y | Y | Y | Q | G | C | A | S | W | D | W | Y | Y | P | Y | H | Y | A | P | F | A | S | D | F | F | D | I | T | N | I | K | V | 669 | | Q6CKX0/1-992 | 610 | D | I | P | A | L | Q | K | D | V | I | R | C | Y | V | E | G | I | S | W | V | L | L | Y | Y | Y | Q | G | C | A | S | W | T | W | Y | Y | P | Y | H | Y | A | P | F | A | Q | D | F | K | N | I | K | N | L | D | I | 663 | | Q6FKN6/1-1018 | 619 | Q | I | P | A | L | S | K | H | M | V | K | C | Y | I | E | G | V | S | W | V | L | M | Y | Y | Y | Q | G | C | A | S | W | T | W | Y | Y | P | Y | H | Y | A | P | L | A | E | D | F | V | D | F | H | D | L | D | I | 672 | | Q74ZA0/1-945 | 605 | S | I | D | A | L | R | K | K | V | V | R | S | Y | V | E | G | I | S | W | V | L | L | Y | Y | Y | Q | G | C | P | S | W | T | W | Y | Y | P | Y | H | Y | A | P | F | A | S | D | F | T | G | I | S | D | L | S | V | 658 | | A7TQ00/1-1057 | 628 | N | I | T | P | L | S | R | K | V | V | R | S | Y | I | E | G | V | S | W | V | L | L | Y | Y | Y | Q | G | C | A | S | W | N | W | Y | Y | P | Y | H | Y | A | P | F | A | S | D | F | T | N | I | A | D | M | E | I | 681 | | C5DJK2/1-982 | 613 | D | I | D | Q | L | R | K | E | V | V | R | S | Y | V | E | G | V | S | W | V | L | L | Y | Y | Y | Q | G | C | A | S | W | N | W | Y | Y | P | Y | H | Y | A | P | F | A | S | D | F | K | D | I | K | D | L | K | I | 666 | | C5DSC0/1-1012 | 631 | H | I | D | A | L | R | K | D | L | V | K | C | Y | I | E | G | V | S | W | V | L | L | Y | Y | Y | Q | G | C | A | S | W | T | W | Y | Y | P | Y | H | Y | A | P | L | A | A | D | F | F | G | F | S | D | I | K | V | 684 | | Kwal\_55.21625/1-974 | 610 | D | I | E | Q | L | C | K | E | V | V | R | S | Y | I | E | G | V | S | W | V | L | L | Y | Y | Y | Q | G | C | A | S | W | D | W | Y | Y | P | Y | H | Y | A | P | F | A | S | D | F | K | D | I | K | D | L | E | I | 663 | | Sbay\_668.45/1-1010 | 620 | D | I | D | Q | L | R | K | D | M | V | K | C | Y | I | E | G | V | A | W | V | L | M | Y | Y | Y | Q | G | C | A | S | W | N | W | F | Y | P | Y | H | Y | A | P | L | A | T | D | F | H | G | F | S | H | L | E | I | 673 | | SAKL0G01386g/1-950 | 619 | E | I | E | P | L | R | K | A | L | V | R | S | Y | V | E | G | I | S | W | V | L | L | Y | Y | Y | Q | G | C | A | S | W | N | W | Y | Y | P | Y | H | Y | A | P | F | A | S | D | F | F | D | I | K | D | F | K | I | 672 | | Q02792/1-1006 | 619 | D | I | E | Q | L | R | K | D | M | V | K | C | Y | I | E | G | V | A | W | V | L | M | Y | Y | Y | Q | G | C | A | S | W | N | W | F | Y | P | Y | H | Y | A | P | L | A | T | D | F | H | G | F | S | H | L | E | I | 672 | |  | | G0VHR2/1-1012 | 670 | E | F | E | L | G | K | P | F | L | P | Y | E | Q | L | M | S | V | L | P | A | A | S | G | H | T | L | P | A | I | F | R | P | L | M | S | E | P | D | S | P | I | I | D | F | Y | P | T | E | F | P | I | D | M | N | 723 | | Q6CKX0/1-992 | 664 | H | F | D | L | G | E | P | F | L | P | Y | E | Q | L | M | S | V | L | P | A | A | S | G | H | A | L | P | E | I | F | R | P | L | M | S | D | P | N | S | E | I | I | D | F | Y | P | E | E | F | P | V | D | M | N | 717 | | Q6FKN6/1-1018 | 673 | K | F | E | L | G | E | P | F | L | P | Y | E | Q | L | M | S | V | L | P | A | A | S | G | H | N | L | P | E | V | F | R | P | L | M | S | S | E | D | S | E | I | I | D | F | Y | P | T | E | F | P | I | D | M | N | 726 | | Q74ZA0/1-945 | 659 | T | F | D | L | G | E | P | F | L | P | Y | E | Q | L | M | S | V | L | P | A | A | S | S | H | N | L | P | D | V | F | R | S | L | M | S | D | P | E | S | E | I | Y | D | F | Y | P | E | E | F | P | I | D | M | N | 712 | | A7TQ00/1-1057 | 682 | N | F | E | L | G | E | P | F | L | P | Y | E | Q | L | M | S | V | L | P | A | A | S | G | N | M | L | P | K | V | F | Q | S | L | M | R | N | P | D | S | E | I | V | D | F | Y | P | V | E | F | L | I | D | M | N | 735 | | C5DJK2/1-982 | 667 | T | F | E | K | G | E | P | F | L | P | F | E | Q | L | M | S | V | L | P | A | A | S | G | H | N | L | P | S | I | F | R | P | L | M | S | E | P | D | S | E | I | I | D | F | Y | P | E | E | F | P | I | D | M | N | 720 | | C5DSC0/1-1012 | 685 | E | F | P | P | G | K | P | F | L | P | Y | E | Q | L | M | S | V | L | P | A | A | S | G | H | T | L | P | P | I | F | R | P | L | M | S | S | P | D | S | E | I | I | D | F | Y | P | T | E | F | P | V | D | M | N | 738 | | Kwal\_55.21625/1-974 | 664 | K | F | E | K | G | E | P | F | L | P | F | E | Q | L | M | S | V | L | P | E | A | S | G | H | N | L | P | P | I | F | R | P | L | M | K | E | P | T | S | E | I | I | D | F | Y | P | Q | E | F | P | V | D | M | N | 717 | | Sbay\_668.45/1-1010 | 674 | K | F | E | E | G | T | P | F | L | P | F | E | Q | L | M | S | V | L | P | A | A | S | G | H | T | L | P | S | I | F | R | S | L | M | S | D | P | D | S | E | I | I | D | F | Y | P | E | E | F | P | I | D | M | N | 727 | | SAKL0G01386g/1-950 | 673 | E | F | D | L | G | E | P | F | L | P | Y | E | Q | L | M | S | V | L | P | A | A | S | G | H | N | L | P | P | I | F | R | P | L | M | S | S | P | D | S | E | I | F | D | F | Y | P | E | E | F | P | I | D | M | N | 726 | | Q02792/1-1006 | 673 | K | F | E | E | G | T | P | F | L | P | Y | E | Q | L | M | S | V | L | P | A | A | S | G | H | A | L | P | K | I | F | R | S | L | M | S | E | P | D | S | E | I | I | D | F | Y | P | E | E | F | P | I | D | M | N | 726 | |  | | G0VHR2/1-1012 | 724 | G | K | K | M | S | W | Q | G | I | S | L | L | P | F | I | D | E | K | R | L | L | E | T | V | R | D | Q | Y | P | K | L | T | P | A | E | R | S | R | N | V | C | N | D | P | V | L | L | I | S | N | K | N | A | N | 777 | | Q6CKX0/1-992 | 718 | G | K | K | M | A | W | Q | G | I | A | L | L | P | F | I | D | E | T | R | L | L | K | T | V | R | E | Q | Y | S | K | L | S | D | S | E | K | A | R | N | V | R | K | K | D | A | L | L | I | S | N | K | N | V | N | 771 | | Q6FKN6/1-1018 | 727 | G | K | K | M | S | W | Q | G | I | A | L | L | P | F | I | D | E | T | R | L | L | T | A | T | R | N | Q | Y | K | F | L | S | E | D | E | K | R | R | N | T | R | N | D | P | V | L | L | I | S | N | K | N | V | N | 780 | | Q74ZA0/1-945 | 713 | G | E | K | M | A | W | K | A | L | V | L | L | P | F | I | D | E | T | R | L | L | A | A | V | R | Q | K | Y | P | L | L | S | E | A | E | R | A | R | N | V | R | R | D | E | V | L | L | I | S | N | K | N | T | H | 766 | | A7TQ00/1-1057 | 736 | G | K | K | M | S | W | Q | G | I | A | L | L | P | F | I | D | E | E | R | L | L | K | S | V | R | S | Q | Y | K | V | L | T | D | S | E | K | S | R | N | V | R | N | Q | E | I | V | L | I | S | N | K | N | V | N | 789 | | C5DJK2/1-982 | 721 | G | K | K | M | S | W | Q | G | I | A | L | L | P | F | I | D | E | K | R | L | L | R | V | V | R | D | Q | Y | E | R | L | S | P | E | E | K | A | R | N | V | R | K | Q | E | V | L | L | I | S | N | K | N | V | N | 774 | | C5DSC0/1-1012 | 739 | G | K | K | M | P | W | Q | G | I | A | L | L | P | F | I | D | E | K | R | L | L | Q | V | V | R | A | Q | Y | S | K | L | T | E | E | E | K | S | R | N | V | V | R | N | P | I | L | L | I | S | N | K | N | A | N | 792 | | Kwal\_55.21625/1-974 | 718 | G | K | K | M | S | W | Q | G | I | A | L | L | P | F | I | D | E | K | R | L | L | R | V | V | R | A | Q | Y | E | H | L | G | A | D | E | K | A | R | N | V | R | K | E | A | V | L | L | I | S | N | K | N | A | N | 771 | | Sbay\_668.45/1-1010 | 728 | G | K | K | M | S | W | Q | G | I | A | L | L | P | F | I | D | Q | D | R | L | L | T | A | V | R | A | Q | Y | S | S | L | S | D | A | E | R | A | R | N | V | R | G | R | P | V | L | L | I | S | D | K | N | A | N | 781 | | SAKL0G01386g/1-950 | 727 | G | K | K | M | S | W | Q | G | I | A | L | L | P | F | I | D | E | K | R | L | L | K | V | V | R | D | Q | Y | K | N | L | T | D | D | E | K | R | R | N | V | R | K | K | E | V | L | L | I | S | N | K | N | A | N | 780 | | Q02792/1-1006 | 727 | G | K | K | M | S | W | Q | G | I | A | L | L | P | F | I | D | Q | D | R | L | L | T | A | V | R | A | Q | Y | P | L | L | S | D | A | E | R | A | R | N | I | R | G | E | P | V | L | L | I | S | N | K | N | A | N | 780 | |  | | G0VHR2/1-1012 | 778 | F | E | R | F | T | K | K | F | Y | K | - | T | E | N | E | D | E | V | S | E | F | T | F | H | H | F | K | S | G | L | S | G | V | V | S | A | D | K | E | G | F | K | L | G | S | K | I | V | S | P | V | Q | G | G | 830 | | Q6CKX0/1-992 | 772 | Y | D | L | F | M | K | N | L | Y | G | - | E | N | - | - | - | P | V | N | V | I | E | F | R | H | F | K | S | G | L | S | G | F | V | T | Q | A | E | E | G | F | E | L | N | S | K | L | I | C | P | I | N | G | G | 821 | | Q6FKN6/1-1018 | 781 | Y | E | K | F | A | K | R | L | Y | K | - | K | G | H | E | - | D | N | F | Q | L | V | F | H | H | F | K | S | S | L | A | G | I | V | S | T | D | T | E | G | F | K | L | H | A | K | L | P | C | P | I | Q | S | G | 832 | | Q74ZA0/1-945 | 767 | Y | E | R | F | L | A | R | L | Y | T | - | D | P | - | - | - | A | P | Q | P | V | N | F | R | H | F | K | K | W | P | R | R | H | S | A | V | R | R | G | G | L | P | S | Q | H | Q | D | A | L | S | A | C | R | R | 816 | | A7TQ00/1-1057 | 790 | Y | D | K | F | V | K | E | L | Y | T | - | E | N | L | E | N | P | N | F | E | Q | Y | F | S | H | F | N | S | G | L | S | G | Y | V | G | K | D | K | E | G | F | D | L | N | S | K | I | L | C | P | I | Q | A | G | 842 | | C5DJK2/1-982 | 775 | Y | E | K | F | S | S | K | L | Y | G | - | A | T | - | - | - | P | V | E | V | V | V | F | Q | H | F | R | S | G | L | S | G | Q | A | M | V | D | Q | E | G | F | A | P | N | G | K | L | I | C | P | V | S | G | G | 824 | | C5DSC0/1-1012 | 793 | Y | E | K | F | L | K | K | L | Y | P | - | T | D | V | P | - | P | Q | P | E | L | Y | F | Q | H | F | R | S | G | L | S | G | V | V | S | T | D | H | E | G | Y | R | P | N | S | K | L | S | C | P | I | Q | T | G | 844 | | Kwal\_55.21625/1-974 | 772 | F | E | K | F | S | S | K | L | Y | G | T | S | S | - | - | - | S | V | E | V | V | V | F | Q | H | F | K | S | G | L | S | G | E | A | L | K | D | A | E | G | F | A | P | N | G | K | L | V | S | P | V | - | G | G | 821 | | Sbay\_668.45/1-1010 | 782 | Y | E | R | F | S | K | K | L | Y | S | - | K | D | - | S | N | A | T | I | E | V | K | F | Q | H | F | K | S | G | L | S | G | I | V | S | K | D | V | E | G | F | E | L | N | G | K | M | I | C | P | I | Q | G | G | 833 | | SAKL0G01386g/1-950 | 781 | Y | E | K | F | S | N | K | L | Y | G | - | E | H | - | - | - | P | V | E | V | V | K | F | Q | H | F | K | S | G | L | S | G | E | A | F | P | D | V | E | G | F | K | L | N | S | K | L | T | C | P | V | G | G | G | 830 | | Q02792/1-1006 | 781 | Y | E | R | F | S | K | K | L | Y | S | - | K | E | N | N | N | N | N | V | V | V | K | F | Q | H | F | K | S | G | L | S | G | I | V | S | K | D | V | E | G | F | E | L | N | G | K | I | V | C | P | I | Q | G | G | 833 | |  | | G0VHR2/1-1012 | 831 | G | L | P | D | L | S | T | N | L | L | L | K | L | C | Y | K | L | V | P | L | P | S | R | N | K | S | I | I | L | N | G | Y | I | P | S | E | Q | V | L | D | A | Y | D | L | D | S | V | M | Y | K | Y | N | N | N | 884 | | Q6CKX0/1-992 | 822 | G | L | P | D | L | S | T | N | L | F | L | K | L | S | Y | T | Q | P | V | V | A | G | R | C | K | S | L | V | L | N | G | Y | I | P | P | Q | P | M | L | T | P | Q | D | R | D | C | I | I | Y | K | Y | S | N | - | 874 | | Q6FKN6/1-1018 | 833 | A | L | P | E | L | S | T | N | L | F | L | K | M | Q | Y | K | L | L | P | L | P | S | A | N | K | S | L | I | L | N | G | F | I | P | S | E | P | M | L | T | Q | H | D | F | D | S | I | V | Y | K | Y | G | T | R | 886 | | Q74ZA0/1-945 | 817 | S | L | P | D | I | S | T | N | L | F | L | K | T | A | Y | R | I | P | Q | L | P | A | P | S | K | S | I | L | L | N | G | F | I | P | P | E | A | Q | L | S | P | A | D | R | D | A | I | M | Y | K | Y | P | Q | - | 869 | | A7TQ00/1-1057 | 843 | S | L | P | T | L | S | T | N | L | F | L | K | M | S | Y | R | M | L | P | L | P | T | K | N | K | S | I | I | L | N | G | F | V | A | S | E | P | M | L | T | S | Y | D | L | D | S | I | L | Y | K | Y | N | D | R | 896 | | C5DJK2/1-982 | 825 | G | L | P | D | L | S | T | N | L | F | L | K | V | G | Y | K | M | P | T | L | P | G | P | S | K | S | V | I | L | N | G | F | I | P | P | Q | P | M | L | T | A | Q | D | R | D | A | I | V | Y | R | Y | N | H | - | 877 | | C5DSC0/1-1012 | 845 | S | L | P | E | L | S | T | N | L | F | L | K | L | D | Y | K | L | V | P | L | P | S | K | N | K | S | I | I | L | N | G | F | I | A | S | E | P | V | L | S | P | Y | D | L | D | A | V | M | Y | K | Y | N | N | G | 898 | | Kwal\_55.21625/1-974 | 822 | S | L | P | D | L | S | T | N | L | F | L | K | V | G | Y | K | M | P | E | L | P | G | P | S | K | S | V | I | L | N | G | F | I | P | P | Q | P | M | L | T | P | Q | D | R | D | A | I | M | Y | R | Y | S | H | - | 874 | | Sbay\_668.45/1-1010 | 834 | S | L | P | D | L | S | T | S | L | I | L | K | M | S | Y | R | L | I | P | L | P | S | K | N | K | S | I | I | L | N | G | F | I | P | S | E | Q | V | L | T | T | Y | D | L | D | S | V | M | Y | K | Y | S | N | Q | 887 | | SAKL0G01386g/1-950 | 831 | G | L | P | D | L | S | T | N | L | F | L | K | V | T | Y | K | M | P | S | L | P | G | K | N | K | S | L | I | L | N | G | F | V | P | S | Q | P | M | L | N | N | Q | D | R | E | S | I | M | Y | R | Y | N | Q | - | 883 | | Q02792/1-1006 | 834 | S | L | P | N | L | S | T | T | L | I | L | K | M | S | Y | R | L | I | P | L | P | S | R | N | K | S | I | I | L | N | G | F | I | P | S | E | P | V | L | T | A | Y | D | L | D | S | I | M | Y | K | Y | N | N | Q | 887 | |  | | G0VHR2/1-1012 | 885 | G | R | N | - | - | - | R | W | N | F | G | N | D | M | K | Q | N | K | A | P | V | G | P | Q | G | T | T | Q | Y | K | P | R | V | G | G | Y | R | A | F | F | F | F | G | Q | Q | N | I | S | N | M | N | N | G | - | 934 | | Q6CKX0/1-992 | 875 | - | - | - | - | - | - | R | W | N | - | P | S | M | M | K | Y | N | I | V | P | V | G | P | S | G | I | T | Q | Y | Q | P | R | V | G | G | Y | R | S | F | F | F | H | K | E | Q | T | A | Y | A | Q | Q | P | Q | Q | 921 | | Q6FKN6/1-1018 | 887 | G | F | Q | R | N | - | Q | R | N | F | G | M | E | M | K | Q | N | I | V | P | V | G | P | S | G | T | T | Q | Y | K | P | R | I | G | G | Y | R | S | F | F | Y | F | G | Q | L | N | Q | Q | M | H | Q | H | N | Q | 939 | | Q74ZA0/1-945 | 870 | - | - | - | - | - | - | R | W | N | - | P | H | S | M | K | H | N | L | V | P | V | G | P | A | A | V | T | Q | Y | Q | P | R | I | G | G | Y | R | A | F | L | F | H | Q | Q | L | M | Q | D | R | P | Y | P | P | - | 915 | | A7TQ00/1-1057 | 897 | N | F | Y | R | N | G | N | R | N | F | G | D | D | M | K | Q | N | I | V | P | V | G | P | R | G | V | T | Q | Y | K | P | R | V | G | G | Y | R | A | F | F | H | F | E | Q | M | N | N | G | H | N | N | - | - | - | 947 | | C5DJK2/1-982 | 878 | - | - | - | - | - | - | R | W | N | - | A | M | D | M | K | H | N | I | V | P | V | G | P | C | A | T | T | Q | Y | Q | P | R | M | G | G | Y | K | S | F | L | F | Y | Q | Q | S | H | S | A | Q | P | V | S | R | - | 923 | | C5DSC0/1-1012 | 899 | P | P | R | - | - | - | R | W | N | F | E | N | D | M | R | N | N | V | V | P | V | G | P | C | G | V | T | Q | Y | K | P | R | V | G | G | Y | R | S | F | F | F | F | S | N | M | Q | Q | Q | P | P | T | P | M | Q | 949 | | Kwal\_55.21625/1-974 | 875 | - | - | - | - | - | - | R | W | N | - | A | M | D | M | K | H | N | I | V | P | V | G | P | C | A | T | T | Q | Y | Q | P | R | L | G | G | Y | K | S | F | L | F | Y | Q | Q | S | H | N | A | P | Q | T | S | R | - | 920 | | Sbay\_668.45/1-1010 | 888 | N | N | S | R | - | - | R | W | N | F | G | N | D | L | K | Q | N | I | V | P | V | G | P | K | G | I | T | Q | Y | K | P | R | T | G | G | Y | R | A | F | F | Y | Y | A | E | Q | N | R | N | S | A | Q | P | V | N | 939 | | SAKL0G01386g/1-950 | 884 | - | - | - | - | - | - | R | W | N | - | G | F | S | M | K | N | N | Y | V | P | V | G | P | Y | G | T | T | Q | Y | K | P | R | I | S | G | Y | K | A | F | L | F | H | Q | Q | L | H | E | R | P | P | Q | V | Q | - | 929 | | Q02792/1-1006 | 888 | N | Y | S | R | - | - | R | W | N | F | G | N | D | L | K | Q | N | I | V | P | V | G | P | K | G | I | T | Q | Y | K | P | R | T | G | G | Y | R | A | F | F | Y | F | A | E | L | S | R | N | N | V | Q | P | A | H | 939 | |  | | G0VHR2/1-1012 | 935 | - | - | - | - | - | Y | Q | N | G | G | N | N | A | N | - | - | - | - | Q | N | Q | Y | H | N | Q | Q | N | R | Y | N | S | G | G | R | G | N | - | Y | Q | S | N | S | R | G | S | Y | Q | G | G | S | R | - | Y | G | 977 | | Q6CKX0/1-992 | 922 | N | R | E | Y | M | H | S | Q | Q | R | Q | P | Q | G | - | - | - | - | - | - | - | S | R | Y | Q | Q | S | R | Y | N | N | G | N | Y | N | N | S | N | N | G | Y | S | D | N | N | N | G | N | T | G | K | - | - | - | 965 | | Q6FKN6/1-1018 | 940 | V | - | - | - | - | Y | Q | Q | N | Q | A | P | T | H | - | - | - | - | - | - | - | D | R | Y | R | N | D | R | S | D | R | G | G | R | G | Y | G | R | P | D | H | D | R | G | D | Y | R | G | D | Y | R | - | - | - | 979 | | Q74ZA0/1-945 | 916 | - | - | - | - | - | A | H | M | H | S | H | S | S | G | G | P | P | L | A | Q | G | P | R | V | T | Q | S | R | Y | Q | P | R | R | F | H | - | - | - | - | - | - | - | - | - | - | - | - | - | - | - | - | - | - | - | 945 | | A7TQ00/1-1057 | 948 | - | - | - | - | - | Y | N | N | N | G | T | G | N | T | - | - | - | - | - | - | - | - | S | Y | E | N | N | G | Y | N | R | G | Q | Q | F | N | Q | H | Q | G | G | S | Y | G | R | Y | N | N | S | N | R | N | Y | Q | 988 | | C5DJK2/1-982 | 924 | - | - | - | - | - | F | Q | Q | Q | E | Q | S | G | Y | - | - | - | - | - | - | - | S | R | P | Q | N | S | R | Y | G | G | G | G | G | G | S | S | N | R | G | R | F | G | G | P | H | S | A | P | G | A | - | - | - | 962 | | C5DSC0/1-1012 | 950 | - | - | - | - | - | Y | Q | M | S | G | P | P | - | - | - | - | - | - | - | - | - | - | - | - | I | N | N | R | Y | N | G | G | G | N | G | - | - | - | - | - | - | G | G | S | R | Y | S | G | S | S | R | - | - | - | 977 | | Kwal\_55.21625/1-974 | 921 | - | - | - | - | - | F | Q | Q | R | E | I | A | A | Y | - | - | - | - | - | - | - | S | R | P | Q | N | S | R | Y | G | G | G | - | - | - | - | - | E | R | N | Q | N | R | G | P | P | Q | R | G | G | F | - | - | - | 954 | | Sbay\_668.45/1-1010 | 940 | N | - | - | - | - | Y | S | K | S | G | Y | N | A | Q | - | - | - | - | - | - | - | A | G | F | N | N | S | R | Y | N | G | A | S | N | N | N | - | N | N | N | N | Y | S | S | N | Y | R | Q | N | S | R | - | - | - | 978 | | SAKL0G01386g/1-950 | 930 | - | - | - | - | - | Y | S | R | Y | N | N | N | S | G | - | - | - | - | - | - | - | P | R | P | M | Q | S | - | - | - | - | - | - | - | - | - | - | - | - | - | Y | K | R | G | P | F | - | - | - | - | - | - | - | - | 950 | | Q02792/1-1006 | 940 | N | - | - | - | - | Y | G | R | N | S | Y | N | S | Q | - | - | - | - | - | - | - | P | G | F | N | N | S | R | Y | D | G | G | N | N | - | - | - | - | - | - | - | - | - | - | N | Y | R | Q | N | S | N | - | Y | R | 971 | |  | | G0VHR2/1-1012 | 978 | G | N | N | Y | Q | Q | Q | R | S | G | - | - | S | S | G | S | R | Y | S | G | T | T | G | N | R | P | N | N | R | Y | Q | G | G | P | P | R | R | - | - | - | - | - | - | - | - | - | - | - | - | - | - | - | - | - | 1012 | | Q6CKX0/1-992 | 966 | - | - | - | Y | N | R | H | Y | N | N | S | R | G | N | S | N | R | Y | S | N | S | N | D | R | R | R | E | F | R | R | - | - | - | - | - | - | - | - | - | - | - | - | - | - | - | - | - | - | - | - | - | - | - | - | 992 | | Q6FKN6/1-1018 | 980 | G | D | G | Y | R | G | G | H | R | G | G | Y | R | G | G | S | R | S | R | G | P | S | S | R | G | I | S | S | R | G | Y | S | G | P | S | R | Y | N | R | - | - | - | - | - | - | - | - | - | - | - | - | - | - | - | 1018 | | Q74ZA0/1-945 |  | - | - | - | - | - | - | - | - | - | - | - | - | - | - | - | - | - | - | - | - | - | - | - | - | - | - | - | - | - | - | - | - | - | - | - | - | - | - | - | - | - | - | - | - | - | - | - | - | - | - | - | - | - | - | | A7TQ00/1-1057 | 989 | S | D | N | Y | N | A | N | S | G | G | - | - | S | Y | N | F | R | N | N | G | N | Y | N | N | R | N | R | G | Y | N | N | N | G | Q | E | R | N | D | R | N | T | S | G | H | R | G | G | S | R | Q | Q | S | R | Y | 1040 | | C5DJK2/1-982 | 963 | - | - | - | - | - | Y | N | - | - | S | R | Y | S | A | S | N | R | Q | G | P | P | S | G | R | G | R | Y | - | - | - | - | - | - | - | - | - | - | - | - | - | - | - | - | - | - | - | - | - | - | - | - | - | - | - | 982 | | C5DSC0/1-1012 | 978 | - | - | - | - | - | - | - | - | - | - | - | - | - | - | - | - | - | Y | G | D | P | P | N | N | Y | R | N | Q | N | N | A | P | G | M | S | R | Y | Q | T | G | R | F | P | Q | R | S | G | T | M | R | R | - | - | - | 1011 | | Kwal\_55.21625/1-974 | 955 | - | - | - | - | - | A | N | R | G | S | R | Y | G | A | P | P | R | Q | G | - | - | S | N | R | G | R | Y | - | - | - | - | - | - | - | - | - | - | - | - | - | - | - | - | - | - | - | - | - | - | - | - | - | - | - | 974 | | Sbay\_668.45/1-1010 | 979 | N | N | N | Y | S | G | N | R | N | G | G | Q | Y | G | G | N | S | Y | S | R | N | N | N | Q | - | - | - | - | - | - | - | - | - | - | S | R | Y | D | N | S | R | R | - | - | - | - | - | - | - | - | - | - | - | - | 1010 | | SAKL0G01386g/1-950 |  | - | - | - | - | - | - | - | - | - | - | - | - | - | - | - | - | - | - | - | - | - | - | - | - | - | - | - | - | - | - | - | - | - | - | - | - | - | - | - | - | - | - | - | - | - | - | - | - | - | - | - | - | - | - | | Q02792/1-1006 | 972 | N | N | N | Y | S | G | N | R | N | S | G | Q | Y | S | G | N | S | Y | S | R | N | N | K | Q | - | - | - | - | - | - | - | - | - | - | S | R | Y | D | N | S | R | A | N | R | R | - | - | - | - | - | - | - | - | - | 1006 | |  | | G0VHR2/1-1012 |  | - | - | - | - | - | - | - | - | - | - | - | - | - | - | - | - | - |  | | | | | | | | | | | | | | | | | | | | | | | | | | | | | | | | | | | | | | Q6CKX0/1-992 |  | - | - | - | - | - | - | - | - | - | - | - | - | - | - | - | - | - |  | | | | | | | | | | | | | | | | | | | | | | | | | | | | | | | | | | | | | | Q6FKN6/1-1018 |  | - | - | - | - | - | - | - | - | - | - | - | - | - | - | - | - | - |  | | | | | | | | | | | | | | | | | | | | | | | | | | | | | | | | | | | | | | Q74ZA0/1-945 |  | - | - | - | - | - | - | - | - | - | - | - | - | - | - | - | - | - |  | | | | | | | | | | | | | | | | | | | | | | | | | | | | | | | | | | | | | | A7TQ00/1-1057 | 1041 | N | G | N | W | N | Q | P | H | S | G | N | Y | K | S | R | Y | Q |  | | | | | | | | | | | | | | | | | | | | | | | | | | | | | | | | | | | | | 1057 | | C5DJK2/1-982 |  | - | - | - | - | - | - | - | - | - | - | - | - | - | - | - | - | - |  | | | | | | | | | | | | | | | | | | | | | | | | | | | | | | | | | | | | | | C5DSC0/1-1012 | 1012 | - | - | - | - | - | - | - | - | - | - | - | - | - | - | - | - | H |  | | | | | | | | | | | | | | | | | | | | | | | | | | | | | | | | | | | | | | Kwal\_55.21625/1-974 |  | - | - | - | - | - | - | - | - | - | - | - | - | - | - | - | - | - |  | | | | | | | | | | | | | | | | | | | | | | | | | | | | | | | | | | | | | | Sbay\_668.45/1-1010 |  | - | - | - | - | - | - | - | - | - | - | - | - | - | - | - | - | - |  | | | | | | | | | | | | | | | | | | | | | | | | | | | | | | | | | | | | | | SAKL0G01386g/1-950 |  | - | - | - | - | - | - | - | - | - | - | - | - | - | - | - | - | - |  | | | | | | | | | | | | | | | | | | | | | | | | | | | | | | | | | | | | | | Q02792/1-1006 |  | - | - | - | - | - | - | - | - | - | - | - | - | - | - | - | - | - |  | | | | | | | | | | | | | | | | | | | | | | | | | | | | | | | | | | | | | |
