# Supplementary material for: Plus ça change – evolutionary sequence divergence predicts protein subcellular localization signals
Source: BMC Genomics. 2014 Jan 20;15:46. doi: 10.1186/1471-2164-15-46 (PMC3906766; doi:10.1186/1471-2164-15-46)
Supplement: Additional file 2 — MSA’s of proteins for which sequence divergence changes predicted localization signals. Contains links to ortholog multiple sequence alignments of each protein in Additional file 3: Table S1. [file 1471-2164-15-46-S2.zip › Q03691.html]

|  |  |  |  |  |  |  |  |  |  |  |  |  |  |  |  |  |  |  |  |  |  |  |  |  |  |  |  |  |  |  |  |  |  |  |  |  |  |  |  |  |  |  |  |  |  |  |  |  |  |  |  |  |  |  |  |  |  |  |  |  |  |  |  |  |  |  |  |  |  |  |  |  |  |  |  |  |  |  |  |  |  |  |  |  |  |  |  |  |  |  |  |  |  |  |  |  |  |  |  |  |  |  |  |  |  |  |  |  |  |  |  |  |  |  |  |  |  |  |  |  |  |  |  |  |  |  |  |  |  |  |  |  |  |  |  |  |  |  |  |  |  |  |  |  |  |  |  |  |  |  |  |  |  |  |  |  |  |  |  |  |  |  |  |  |  |  |  |  |  |  |  |  |  |  |  |  |  |  |  |  |  |  |  |  |  |  |  |  |  |  |  |  |  |  |  |  |  |  |  |  |  |  |  |  |  |  |  |  |  |  |  |  |  |  |  |  |  |  |  |  |  |  |  |  |  |  |  |  |  |  |  |  |  |  |  |  |  |  |  |  |  |  |  |  |  |  |  |  |  |  |  |  |  |  |  |  |  |  |  |  |  |  |  |  |  |  |  |  |  |  |  |  |  |  |  |  |  |  |  |  |  |  |  |  |  |  |  |  |  |  |  |  |  |  |  |  |  |  |  |  |  |  |  |  |  |  |  |  |  |  |  |  |  |  |  |  |  |  |  |  |  |  |  |  |  |  |  |  |  |  |  |  |  |  |  |  |  |  |  |  |  |  |  |  |  |  |  |  |  |  |  |  |  |  |  |  |  |  |  |  |  |  |  |  |  |  |  |  |  |  |  |  |  |  |  |  |  |  |  |  |  |  |  |  |  |  |  |  |  |  |  |  |  |  |  |  |  |  |  |  |  |  |  |  |  |  |  |  |  |  |  |  |  |  |  |  |  |  |  |  |  |  |  |  |  |  |  |  |  |  |  |  |  |  |  |  |  |  |  |  |  |  |  |  |  |  |  |  |  |  |  |  |  |  |  |  |  |  |  |  |  |  |  |  |  |  |  |  |  |  |  |  |  |  |  |  |  |  |  |  |  |  |  |  |  |  |  |  |  |  |  |  |  |  |  |  |  |  |  |  |  |  |  |  |  |  |  |  |  |  |  |  |  |  |  |  |  |  |  |  |  |  |  |  |  |  |  |  |  |  |  |  |  |  |  |  |  |  |  |  |  |  |  |  |  |  |  |  |  |  |  |  |  |  |  |  |  |  |  |  |  |  |  |  |  |  |  |  |  |  |  |  |  |  |  |  |  |  |  |  |  |  |  |  |  |  |  |  |  |  |  |  |  |  |  |  |  |  |  |  |  |  |  |  |  |  |  |  |  |  |  |  |  |  |  |  |  |  |  |  |  |  |  |  |  |  |  |  |  |  |  |  |  |  |  |  |  |  |  |  |  |  |  |  |  |  |  |  |  |  |  |  |  |  |  |  |  |  |  |  |  |  |  |  |  |  |  |  |  |  |  |  |  |  |  |  |  |  |  |  |  |  |  |  |  |  |  |  |  |  |  |  |  |  |  |  |  |  |  |  |  |  |  |  |  |  |  |  |  |  |  |  |  |  |  |  |  |  |  |  |  |  |  |  |  |  |  |  |  |  |  |  |  |  |  |  |  |  |  |  |  |  |  |  |  |  |  |  |  |  |  |  |  |  |  |  |  |  |  |  |  |  |  |  |  |  |  |  |  |  |  |  |  |  |  |  |  |  |  |  |  |  |  |  |  |  |  |  |  |  |  |  |  |  |  |  |  |  |  |  |  |  |  |  |  |  |  |  |  |  |  |  |  |  |  |  |  |  |  |  |  |  |  |  |  |  |  |  |  |  |  |  |  |  |  |  |  |  |  |  |  |  |  |  |  |  |  |  |  |  |  |  |  |  |  |  |  |  |  |  |  |  |  |  |  |  |  |  |  |  |  |  |  |  |  |  |  |  |  |  |  |  |  |  |  |  |  |  |  |  |  |  |  |  |  |  |  |  |  |  |  |  |  |  |  |  |  |  |  |  |  |  |  |  |  |  |  |  |  |  |  |  |  |  |  |  |  |  |  |  |  |  |  |  |  |  |  |  |  |  |  |  |  |  |  |  |  |  |  |  |  |  |  |  |  |  |  |  |  |  |  |  |  |  |  |  |  |  |  |  |  |  |  |  |  |  |  |  |  |  |  |  |  |  |  |  |  |  |  |  |  |  |  |  |  |  |  |  |  |  |  |  |  |  |  |  |  |  |  |  |  |  |  |  |  |  |  |  |  |  |  |  |  |  |  |  |  |  |  |  |  |  |  |  |  |  |  |  |  |  |  |  |  |  |  |  |  |  |  |  |  |  |  |  |  |  |  |  |  |  |  |  |  |  |  |  |  |  |  |  |  |  |  |  |  |  |  |  |  |  |  |  |  |  |  |  |  |  |  |  |  |  |  |  |  |  |  |  |  |  |  |  |  |  |  |  |  |  |  |  |  |  |  |  |  |  |  |  |  |  |  |  |  |  |  |  |  |  |  |  |  |  |  |  |  |  |  |  |  |  |  |  |  |  |  |  |  |  |  |  |  |  |  |  |  |  |  |  |  |  |  |  |  |  |  |  |  |  |  |  |  |  |  |  |  |  |  |  |  |  |  |  |  |  |  |  |  |  |  |  |  |  |  |  |  |  |  |  |  |  |  |  |  |  |  |  |  |  |  |  |  |  |  |  |  |  |  |  |  |  |  |  |  |  |  |  |  |  |  |  |  |  |  |  |  |  |  |  |  |  |  |  |  |  |  |  |  |  |  |  |  |  |  |  |  |  |  |  |  |  |  |  |  |  |  |  |  |  |  |  |  |  |  |  |  |  |  |  |  |  |  |  |  |  |  |  |  |  |  |  |  |  |  |  |  |  |  |  |  |  |  |  |  |  |  |  |  |  |  |  |  |  |  |  |  |  |  |  |  |  |  |  |  |  |  |  |  |  |  |  |  |  |  |  |  |  |  |  |  |  |  |  |  |  |  |  |  |  |  |  |  |  |  |  |  |  |  |  |  |  |  |  |  |  |  |  |  |  |  |  |  |  |  |  |  |  |  |  |  |  |  |  |  |  |  |  |  |  |  |  |  |  |  |  |  |  |  |  |  |  |  |  |  |  |  |  |  |  |  |  |  |  |  |  |  |  |  |  |  |  |  |  |  |  |  |  |  |  |  |  |  |  |  |  |  |  |  |  |  |  |  |  |  |  |  |  |  |  |  |  |  |  |  |  |  |  |  |  |  |  |  |  |  |  |  |  |  |  |  |  |  |  |  |  |  |  |  |  |  |  |  |  |  |  |  |  |  |  |  |  |  |  |  |  |  |  |  |  |  |  |  |  |  |  |  |  |  |  |  |  |  |  |  |  |  |  |  |  |  |  |  |  |  |  |  |  |  |  |  |  |  |  |  |  |  |  |  |  |  |  |  |  |  |  |  |  |  |  |  |  |  |  |  |  |  |  |  |  |  |  |  |  |  |  |  |  |  |  |  |  |  |  |  |  |  |  |  |  |  |  |  |  |  |  |  |  |  |  |  |  |  |  |  |  |  |  |  |  |  |  |  |  |  |  |  |  |  |  |  |  |  |  |  |  |  |  |  |  |  |  |  |  |  |  |  |  |  |  |  |  |  |  |  |  |  |  |  |  |  |  |  |  |  |  |  |  |  |  |  |  |  |  |  |  |  |  |  |  |  |  |  |  |  |  |  |  |  |  |  |  |  |  |  |  |  |  |  |  |  |  |  |  |  |  |  |  |  |  |  |  |  |  |  |  |  |  |  |  |  |  |  |  |  |  |  |  |  |  |  |  |  |  |  |  |  |  |  |  |  |  |  |  |  |  |  |  |  |  |  |  |  |  |  |  |  |  |  |  |  |  |  |  |  |  |  |  |  |  |  |  |  |  |  |  |  |  |  |  |  |  |  |  |  |  |  |  |  |  |  |  |  |  |  |  |  |  |  |  |  |  |  |  |  |  |  |  |  |  |  |  |  |  |  |  |  |  |  |  |  |  |  |  |  |  |  |  |  |  |  |  |  |  |  |  |  |  |  |  |  |  |  |  |  |  |  |  |  |  |  |  |  |  |  |  |  |  |  |  |  |  |  |  |  |  |  |  |  |  |  |  |  |  |  |  |  |  |  |  |  |  |  |  |  |  |  |  |  |  |  |  |  |  |  |  |  |  |  |  |  |  |  |  |  |  |  |  |  |  |  |  |  |  |  |  |  |  |  |  |  |  |  |  |  |  |  |  |  |  |  |  |  |  |  |  |  |  |  |  |  |  |  |  |  |  |  |  |  |  |  |  |  |  |  |  |  |  |  |  |  |  |  |  |  |  |  |  |  |  |  |  |  |  |  |  |  |  |  |  |  |  |  |  |  |  |  |  |  |  |  |  |  |  |  |  |  |  |  |  |  |  |  |  |  |  |  |  |  |  |  |  |  |  |  |  |  |  |  |  |  |  |  |  |  |  |  |  |  |  |  |  |  |  |  |  |  |  |  |  |  |  |  |  |  |  |  |  |  |  |  |  |  |  |  |  |  |  |  |  |  |  |  |  |  |  |  |  |  |  |  |  |  |  |  |  |  |  |  |  |  |  |  |  |  |  |  |  |  |  |  |  |  |  |  |  |  |  |  |  |  |  |  |  |  |  |  |  |  |  |  |  |  |  |  |  |  |  |  |  |  |  |  |  |  |  |  |  |  |  |  |  |  |  |  |  |  |  |  |  |  |  |  |  |  |  |  |  |  |  |  |  |  |  |  |  |  |  |  |  |  |  |  |  |  |  |  |  |  |  |  |  |  |  |  |  |  |  |  |  |  |  |  |  |  |  |  |  |  |  |  |  |  |  |  |  |  |  |  |  |  |  |  |  |  |  |  |  |  |  |  |  |  |  |  |  |  |  |  |  |  |  |  |  |  |  |  |  |  |  |  |  |  |  |  |  |  |  |  |  |  |  |  |  |  |  |  |  |  |  |  |  |  |  |  |  |  |  |  |  |  |  |  |  |  |  |  |  |  |  |  |  |  |  |  |  |  |  |  |  |  |  |  |  |  |  |  |  |  |  |  |  |  |  |  |  |  |  |  |  |  |  |  |  |  |  |  |  |  |  |  |  |  |  |  |  |  |  |  |  |  |  |  |  |  |  |  |  |  |  |  |  |  |  |  |  |  |  |  |  |  |  |  |  |  |  |  |  |  |  |  |  |  |  |  |  |  |  |  |  |  |  |  |  |  |  |  |  |  |  |  |  |  |  |  |  |  |  |  |  |  |  |  |  |  |  |  |  |  |  |  |  |  |  |  |  |  |  |  |  |  |  |  |  |  |  |  |  |  |  |  |  |  |  |  |  |  |  |  |  |  |  |  |  |  |  |  |  |  |  |  |  |  |  |  |  |  |  |  |  |  |  |  |  |  |  |  |  |  |  |  |  |  |  |  |  |  |  |  |  |  |  |  |  |  |  |  |  |  |  |  |  |  |  |  |  |  |  |  |  |  |  |  |  |  |  |  |  |  |  |  |  |  |  |  |  |  |  |  |  |  |  |  |  |  |  |  |  |  |  |  |  |  |  |  |  |  |  |  |  |  |  |  |  |  |  |  |  |  |  |  |  |  |  |  |  |  |  |  |  |  |  |  |  |  |  |  |  |  |  |  |  |  |  |  |  |  |  |  |  |  |  |  |  |  |  |  |  |  |  |  |  |  |  |  |  |  |  |  |  |  |  |  |  |  |  |  |  |  |  |  |  |  |  |  |  |  |  |  |  |  |  |  |  |  |  |  |  |  |  |  |  |  |  |  |  |  |  |  |  |  |  |  |  |  |  |  |  |  |  |  |  |  |  |  |  |  |  |  |  |  |  |  |  |  |  |  |  |  |  |  |  |  |  |  |  |  |  |  |  |  |  |  |  |  |  |  |  |  |  |  |  |  |  |  |  |  |  |  |  |  |  |  |  |  |  |  |  |  |  |  |  |  |  |  |  |  |  |  |  |  |  |  |  |  |  |  |  |  |  |  |  |  |  |  |  |  |  |  |  |  |  |  |  |  |  |  |  |  |  |  |  |  |  |  |  |  |  |  |  |  |  |  |  |  |  |  |  |  |  |  |  |  |  |  |  |  |  |  |  |  |  |  |  |  |  |  |  |  |  |  |  |  |  |  |  |  |  |  |  |  |  |  |  |  |  |  |  |  |  |  |  |  |  |  |  |  |  |  |  |  |  |  |  |  |  |  |  |  |  |  |  |  |  |  |  |  |  |  |  |  |  |  |  |  |  |  |  |  |  |  |  |  |  |  |  |  |  |  |  |  |  |  |  |  |  |  |  |  |  |  |  |  |  |  |  |  |  |  |  |  |  |  |  |  |  |  |  |  |  |  |  |  |  |  |  |  |  |  |  |  |  |  |  |  |  |  |  |  |  |  |  |  |  |  |  |  |  |  |  |  |  |  |  |  |  |  |  |  |  |  |  |  |  |  |  |  |  |  |  |  |  |  |  |  |  |  |  |  |  |  |  |  |  |  |  |  |  |  |  |  |  |  |  |  |  |  |  |  |  |  |  |  |  |  |  |  |  |  |  |  |  |  |  |  |  |  |  |  |  |  |  |  |  |  |  |  |  |  |  |  |  |  |  |  |  |  |  |  |  |  |  |  |  |  |  |  |  |  |  |  |  |  |  |  |  |  |  |  |  |  |  |  |  |  |  |  |  |  |  |  |  |  |  |  |  |  |  |  |  |  |  |  |  |  |  |  |  |  |  |  |  |  |  |  |  |  |  |  |  |  |  |  |  |  |  |  |  |  |  |  |  |  |  |  |  |  |  |  |  |  |  |  |  |  |  |  |  |  |  |  |  |  |  |  |  |  |  |  |  |  |  |  |  |  |  |  |  |  |  |  |  |  |  |  |  |  |  |  |  |  |  |  |  |  |  |  |  |  |  |  |  |  |  |  |  |  |  |  |  |  |  |  |  |  |  |  |  |  |  |  |  |  |  |  |  |  |  |  |  |  |  |  |  |  |  |  |  |  |  |  |  |  |  |  |  |  |  |  |  |  |  |  |  |  |  |  |  |  |  |  |  |  |  |  |  |  |  |  |  |  |  |  |  |  |  |  |  |  |  |  |  |  |  |  |  |  |  |  |  |  |  |  |  |  |  |
| --- | --- | --- | --- | --- | --- | --- | --- | --- | --- | --- | --- | --- | --- | --- | --- | --- | --- | --- | --- | --- | --- | --- | --- | --- | --- | --- | --- | --- | --- | --- | --- | --- | --- | --- | --- | --- | --- | --- | --- | --- | --- | --- | --- | --- | --- | --- | --- | --- | --- | --- | --- | --- | --- | --- | --- | --- | --- | --- | --- | --- | --- | --- | --- | --- | --- | --- | --- | --- | --- | --- | --- | --- | --- | --- | --- | --- | --- | --- | --- | --- | --- | --- | --- | --- | --- | --- | --- | --- | --- | --- | --- | --- | --- | --- | --- | --- | --- | --- | --- | --- | --- | --- | --- | --- | --- | --- | --- | --- | --- | --- | --- | --- | --- | --- | --- | --- | --- | --- | --- | --- | --- | --- | --- | --- | --- | --- | --- | --- | --- | --- | --- | --- | --- | --- | --- | --- | --- | --- | --- | --- | --- | --- | --- | --- | --- | --- | --- | --- | --- | --- | --- | --- | --- | --- | --- | --- | --- | --- | --- | --- | --- | --- | --- | --- | --- | --- | --- | --- | --- | --- | --- | --- | --- | --- | --- | --- | --- | --- | --- | --- | --- | --- | --- | --- | --- | --- | --- | --- | --- | --- | --- | --- | --- | --- | --- | --- | --- | --- | --- | --- | --- | --- | --- | --- | --- | --- | --- | --- | --- | --- | --- | --- | --- | --- | --- | --- | --- | --- | --- | --- | --- | --- | --- | --- | --- | --- | --- | --- | --- | --- | --- | --- | --- | --- | --- | --- | --- | --- | --- | --- | --- | --- | --- | --- | --- | --- | --- | --- | --- | --- | --- | --- | --- | --- | --- | --- | --- | --- | --- | --- | --- | --- | --- | --- | --- | --- | --- | --- | --- | --- | --- | --- | --- | --- | --- | --- | --- | --- | --- | --- | --- | --- | --- | --- | --- | --- | --- | --- | --- | --- | --- | --- | --- | --- | --- | --- | --- | --- | --- | --- | --- | --- | --- | --- | --- | --- | --- | --- | --- | --- | --- | --- | --- | --- | --- | --- | --- | --- | --- | --- | --- | --- | --- | --- | --- | --- | --- | --- | --- | --- | --- | --- | --- | --- | --- | --- | --- | --- | --- | --- | --- | --- | --- | --- | --- | --- | --- | --- | --- | --- | --- | --- | --- | --- | --- | --- | --- | --- | --- | --- | --- | --- | --- | --- | --- | --- | --- | --- | --- | --- | --- | --- | --- | --- | --- | --- | --- | --- | --- | --- | --- | --- | --- | --- | --- | --- | --- | --- | --- | --- | --- | --- | --- | --- | --- | --- | --- | --- | --- | --- | --- | --- | --- | --- | --- | --- | --- | --- | --- | --- | --- | --- | --- | --- | --- | --- | --- | --- | --- | --- | --- | --- | --- | --- | --- | --- | --- | --- | --- | --- | --- | --- | --- | --- | --- | --- | --- | --- | --- | --- | --- | --- | --- | --- | --- | --- | --- | --- | --- | --- | --- | --- | --- | --- | --- | --- | --- | --- | --- | --- | --- | --- | --- | --- | --- | --- | --- | --- | --- | --- | --- | --- | --- | --- | --- | --- | --- | --- | --- | --- | --- | --- | --- | --- | --- | --- | --- | --- | --- | --- | --- | --- | --- | --- | --- | --- | --- | --- | --- | --- | --- | --- | --- | --- | --- | --- | --- | --- | --- | --- | --- | --- | --- | --- | --- | --- | --- | --- | --- | --- | --- | --- | --- | --- | --- | --- | --- | --- | --- | --- | --- | --- | --- | --- | --- | --- | --- | --- | --- | --- | --- | --- | --- | --- | --- | --- | --- | --- | --- | --- | --- | --- | --- | --- | --- | --- | --- | --- | --- | --- | --- | --- | --- | --- | --- | --- | --- | --- | --- | --- | --- | --- | --- | --- | --- | --- | --- | --- | --- | --- | --- | --- | --- | --- | --- | --- | --- | --- | --- | --- | --- | --- | --- | --- | --- | --- | --- | --- | --- | --- | --- | --- | --- | --- | --- | --- | --- | --- | --- | --- | --- | --- | --- | --- | --- | --- | --- | --- | --- | --- | --- | --- | --- | --- | --- | --- | --- | --- | --- | --- | --- | --- | --- | --- | --- | --- | --- | --- | --- | --- | --- | --- | --- | --- | --- | --- | --- | --- | --- | --- | --- | --- | --- | --- | --- | --- | --- | --- | --- | --- | --- | --- | --- | --- | --- | --- | --- | --- | --- | --- | --- | --- | --- | --- | --- | --- | --- | --- | --- | --- | --- | --- | --- | --- | --- | --- | --- | --- | --- | --- | --- | --- | --- | --- | --- | --- | --- | --- | --- | --- | --- | --- | --- | --- | --- | --- | --- | --- | --- | --- | --- | --- | --- | --- | --- | --- | --- | --- | --- | --- | --- | --- | --- | --- | --- | --- | --- | --- | --- | --- | --- | --- | --- | --- | --- | --- | --- | --- | --- | --- | --- | --- | --- | --- | --- | --- | --- | --- | --- | --- | --- | --- | --- | --- | --- | --- | --- | --- | --- | --- | --- | --- | --- | --- | --- | --- | --- | --- | --- | --- | --- | --- | --- | --- | --- | --- | --- | --- | --- | --- | --- | --- | --- | --- | --- | --- | --- | --- | --- | --- | --- | --- | --- | --- | --- | --- | --- | --- | --- | --- | --- | --- | --- | --- | --- | --- | --- | --- | --- | --- | --- | --- | --- | --- | --- | --- | --- | --- | --- | --- | --- | --- | --- | --- | --- | --- | --- | --- | --- | --- | --- | --- | --- | --- | --- | --- | --- | --- | --- | --- | --- | --- | --- | --- | --- | --- | --- | --- | --- | --- | --- | --- | --- | --- | --- | --- | --- | --- | --- | --- | --- | --- | --- | --- | --- | --- | --- | --- | --- | --- | --- | --- | --- | --- | --- | --- | --- | --- | --- | --- | --- | --- | --- | --- | --- | --- | --- | --- | --- | --- | --- | --- | --- | --- | --- | --- | --- | --- | --- | --- | --- | --- | --- | --- | --- | --- | --- | --- | --- | --- | --- | --- | --- | --- | --- | --- | --- | --- | --- | --- | --- | --- | --- | --- | --- | --- | --- | --- | --- | --- | --- | --- | --- | --- | --- | --- | --- | --- | --- | --- | --- | --- | --- | --- | --- | --- | --- | --- | --- | --- | --- | --- | --- | --- | --- | --- | --- | --- | --- | --- | --- | --- | --- | --- | --- | --- | --- | --- | --- | --- | --- | --- | --- | --- | --- | --- | --- | --- | --- | --- | --- | --- | --- | --- | --- | --- | --- | --- | --- | --- | --- | --- | --- | --- | --- | --- | --- | --- | --- | --- | --- | --- | --- | --- | --- | --- | --- | --- | --- | --- | --- | --- | --- | --- | --- | --- | --- | --- | --- | --- | --- | --- | --- | --- | --- | --- | --- | --- | --- | --- | --- | --- | --- | --- | --- | --- | --- | --- | --- | --- | --- | --- | --- | --- | --- | --- | --- | --- | --- | --- | --- | --- | --- | --- | --- | --- | --- | --- | --- | --- | --- | --- | --- | --- | --- | --- | --- | --- | --- | --- | --- | --- | --- | --- | --- | --- | --- | --- | --- | --- | --- | --- | --- | --- | --- | --- | --- | --- | --- | --- | --- | --- | --- | --- | --- | --- | --- | --- | --- | --- | --- | --- | --- | --- | --- | --- | --- | --- | --- | --- | --- | --- | --- | --- | --- | --- | --- | --- | --- | --- | --- | --- | --- | --- | --- | --- | --- | --- | --- | --- | --- | --- | --- | --- | --- | --- | --- | --- | --- | --- | --- | --- | --- | --- | --- | --- | --- | --- | --- | --- | --- | --- | --- | --- | --- | --- | --- | --- | --- | --- | --- | --- | --- | --- | --- | --- | --- | --- | --- | --- | --- | --- | --- | --- | --- | --- | --- | --- | --- | --- | --- | --- | --- | --- | --- | --- | --- | --- | --- | --- | --- | --- | --- | --- | --- | --- | --- | --- | --- | --- | --- | --- | --- | --- | --- | --- | --- | --- | --- | --- | --- | --- | --- | --- | --- | --- | --- | --- | --- | --- | --- | --- | --- | --- | --- | --- | --- | --- | --- | --- | --- | --- | --- | --- | --- | --- | --- | --- | --- | --- | --- | --- | --- | --- | --- | --- | --- | --- | --- | --- | --- | --- | --- | --- | --- | --- | --- | --- | --- | --- | --- | --- | --- | --- | --- | --- | --- | --- | --- | --- | --- | --- | --- | --- | --- | --- | --- | --- | --- | --- | --- | --- | --- | --- | --- | --- | --- | --- | --- | --- | --- | --- | --- | --- | --- | --- | --- | --- | --- | --- | --- | --- | --- | --- | --- | --- | --- | --- | --- | --- | --- | --- | --- | --- | --- | --- | --- | --- | --- | --- | --- | --- | --- | --- | --- | --- | --- | --- | --- | --- | --- | --- | --- | --- | --- | --- | --- | --- | --- | --- | --- | --- | --- | --- | --- | --- | --- | --- | --- | --- | --- | --- | --- | --- | --- | --- | --- | --- | --- | --- | --- | --- | --- | --- | --- | --- | --- | --- | --- | --- | --- | --- | --- | --- | --- | --- | --- | --- | --- | --- | --- | --- | --- | --- | --- | --- | --- | --- | --- | --- | --- | --- | --- | --- | --- | --- | --- | --- | --- | --- | --- | --- | --- | --- | --- | --- | --- | --- | --- | --- | --- | --- | --- | --- | --- | --- | --- | --- | --- | --- | --- | --- | --- | --- | --- | --- | --- | --- | --- | --- | --- | --- | --- | --- | --- | --- | --- | --- | --- | --- | --- | --- | --- | --- | --- | --- | --- | --- | --- | --- | --- | --- | --- | --- | --- | --- | --- | --- | --- | --- | --- | --- | --- | --- | --- | --- | --- | --- | --- | --- | --- | --- | --- | --- | --- | --- | --- | --- | --- | --- | --- | --- | --- | --- | --- | --- | --- | --- | --- | --- | --- | --- | --- | --- | --- | --- | --- | --- | --- | --- | --- | --- | --- | --- | --- | --- | --- | --- | --- | --- | --- | --- | --- | --- | --- | --- | --- | --- | --- | --- | --- | --- | --- | --- | --- | --- | --- | --- | --- | --- | --- | --- | --- | --- | --- | --- | --- | --- | --- | --- | --- | --- | --- | --- | --- | --- | --- | --- | --- | --- | --- | --- | --- | --- | --- | --- | --- | --- | --- | --- | --- | --- | --- | --- | --- | --- | --- | --- | --- | --- | --- | --- | --- | --- | --- | --- | --- | --- | --- | --- | --- | --- | --- | --- | --- | --- | --- | --- | --- | --- | --- | --- | --- | --- | --- | --- | --- | --- | --- | --- | --- | --- | --- | --- | --- | --- | --- | --- | --- | --- | --- | --- | --- | --- | --- | --- | --- | --- | --- | --- | --- | --- | --- | --- | --- | --- | --- | --- | --- | --- | --- | --- | --- | --- | --- | --- | --- | --- | --- | --- | --- | --- | --- | --- | --- | --- | --- | --- | --- | --- | --- | --- | --- | --- | --- | --- | --- | --- | --- | --- | --- | --- | --- | --- | --- | --- | --- | --- | --- | --- | --- | --- | --- | --- | --- | --- | --- | --- | --- | --- | --- | --- | --- | --- | --- | --- | --- | --- | --- | --- | --- | --- | --- | --- | --- | --- | --- | --- | --- | --- | --- | --- | --- | --- | --- | --- | --- | --- | --- | --- | --- | --- | --- | --- | --- | --- | --- | --- | --- | --- | --- | --- | --- | --- | --- | --- | --- | --- | --- | --- | --- | --- | --- | --- | --- | --- | --- | --- | --- | --- | --- | --- | --- | --- | --- | --- | --- | --- | --- | --- | --- | --- | --- | --- | --- | --- | --- | --- | --- | --- | --- | --- | --- | --- | --- | --- | --- | --- | --- | --- | --- | --- | --- | --- | --- | --- | --- | --- | --- | --- | --- | --- | --- | --- | --- | --- | --- | --- | --- | --- | --- | --- | --- | --- | --- | --- | --- | --- | --- | --- | --- | --- | --- | --- | --- | --- | --- | --- | --- | --- | --- | --- | --- | --- | --- | --- | --- | --- | --- | --- | --- | --- | --- | --- | --- | --- | --- | --- | --- | --- | --- | --- | --- | --- | --- | --- | --- | --- | --- | --- | --- | --- | --- | --- | --- | --- | --- | --- | --- | --- | --- | --- | --- | --- | --- | --- | --- | --- | --- | --- | --- | --- | --- | --- | --- | --- | --- | --- | --- | --- | --- | --- | --- | --- | --- | --- | --- | --- | --- | --- | --- | --- | --- | --- | --- | --- | --- | --- | --- | --- | --- | --- | --- | --- | --- | --- | --- | --- | --- | --- | --- | --- | --- | --- | --- | --- | --- | --- | --- | --- | --- | --- | --- | --- | --- | --- | --- | --- | --- | --- | --- | --- | --- | --- | --- | --- | --- | --- | --- | --- | --- | --- | --- | --- | --- | --- | --- | --- | --- | --- | --- | --- | --- | --- | --- | --- | --- | --- | --- | --- | --- | --- | --- | --- | --- | --- | --- | --- | --- | --- | --- | --- | --- | --- | --- | --- | --- | --- | --- | --- | --- | --- | --- | --- | --- | --- | --- | --- | --- | --- | --- | --- | --- | --- | --- | --- | --- | --- | --- | --- | --- | --- | --- | --- | --- | --- | --- | --- | --- | --- | --- | --- | --- | --- | --- | --- | --- | --- | --- | --- | --- | --- | --- | --- | --- | --- | --- | --- | --- | --- | --- | --- | --- | --- | --- | --- | --- | --- | --- | --- | --- | --- | --- | --- | --- | --- | --- | --- | --- | --- | --- | --- | --- | --- | --- | --- | --- | --- | --- | --- | --- | --- | --- | --- | --- | --- | --- | --- | --- | --- | --- | --- | --- | --- | --- | --- | --- | --- | --- | --- | --- | --- | --- | --- | --- | --- | --- | --- | --- | --- | --- | --- | --- | --- | --- | --- | --- | --- | --- | --- | --- | --- | --- | --- | --- | --- | --- | --- | --- | --- | --- | --- | --- | --- | --- | --- | --- | --- | --- | --- | --- | --- | --- | --- | --- | --- | --- | --- | --- | --- | --- | --- | --- | --- | --- | --- | --- | --- | --- | --- | --- | --- | --- | --- | --- | --- | --- | --- | --- | --- | --- | --- | --- | --- | --- | --- | --- | --- | --- | --- | --- | --- | --- | --- | --- | --- | --- | --- | --- | --- | --- | --- | --- | --- | --- | --- | --- | --- | --- | --- | --- | --- | --- | --- | --- | --- | --- | --- | --- | --- | --- | --- | --- | --- | --- | --- | --- | --- | --- | --- | --- | --- | --- | --- | --- | --- | --- | --- | --- | --- | --- | --- | --- | --- | --- | --- | --- | --- | --- | --- | --- | --- | --- | --- | --- | --- | --- | --- | --- | --- | --- | --- | --- | --- | --- | --- | --- | --- | --- | --- | --- | --- | --- | --- | --- | --- | --- | --- | --- | --- | --- | --- | --- | --- | --- | --- | --- | --- | --- | --- | --- | --- | --- | --- | --- | --- | --- | --- | --- | --- | --- | --- | --- | --- | --- | --- | --- | --- | --- | --- | --- | --- | --- | --- | --- | --- | --- | --- | --- | --- | --- | --- | --- | --- | --- | --- | --- | --- | --- | --- | --- | --- | --- | --- | --- | --- | --- | --- | --- | --- | --- | --- | --- | --- | --- | --- | --- | --- | --- | --- | --- | --- | --- | --- | --- | --- | --- | --- | --- | --- | --- | --- | --- | --- | --- | --- | --- | --- | --- | --- | --- | --- | --- | --- | --- | --- | --- | --- | --- | --- | --- | --- | --- | --- | --- | --- | --- | --- | --- | --- | --- | --- | --- | --- | --- | --- | --- | --- | --- | --- | --- | --- | --- | --- | --- | --- | --- | --- | --- | --- | --- | --- | --- | --- | --- | --- | --- | --- | --- | --- | --- | --- | --- | --- | --- | --- | --- | --- | --- | --- | --- | --- | --- | --- | --- | --- | --- | --- | --- | --- | --- | --- | --- | --- | --- | --- | --- | --- | --- | --- | --- | --- | --- | --- | --- | --- | --- | --- | --- | --- | --- | --- | --- | --- | --- | --- | --- | --- | --- | --- | --- | --- | --- | --- | --- | --- | --- | --- | --- | --- | --- | --- | --- | --- | --- | --- | --- | --- | --- | --- | --- | --- | --- | --- | --- | --- | --- | --- | --- | --- | --- | --- | --- | --- | --- | --- | --- | --- | --- | --- | --- | --- | --- | --- | --- | --- | --- | --- | --- | --- | --- | --- | --- | --- | --- | --- | --- | --- | --- | --- | --- | --- | --- | --- | --- | --- | --- | --- | --- | --- | --- | --- | --- | --- | --- | --- | --- | --- | --- | --- | --- | --- | --- | --- | --- | --- | --- | --- | --- | --- | --- | --- | --- | --- | --- | --- | --- | --- | --- | --- | --- | --- | --- | --- | --- | --- | --- | --- | --- | --- | --- | --- | --- | --- | --- | --- | --- | --- | --- | --- | --- | --- | --- | --- | --- | --- | --- | --- | --- | --- | --- | --- | --- | --- | --- | --- | --- | --- | --- | --- | --- | --- | --- | --- | --- | --- | --- | --- | --- | --- | --- | --- | --- | --- | --- | --- | --- | --- | --- | --- | --- | --- | --- | --- | --- | --- | --- | --- | --- | --- | --- | --- | --- | --- | --- | --- | --- | --- | --- | --- | --- | --- | --- | --- | --- | --- | --- | --- | --- | --- | --- | --- | --- | --- | --- | --- | --- | --- | --- | --- | --- | --- | --- | --- | --- | --- | --- | --- | --- | --- | --- | --- | --- | --- | --- | --- | --- | --- | --- | --- | --- | --- | --- | --- | --- | --- | --- | --- | --- | --- | --- | --- | --- | --- | --- | --- | --- | --- | --- | --- | --- | --- | --- | --- | --- | --- | --- | --- | --- | --- | --- | --- | --- | --- | --- | --- | --- | --- | --- | --- | --- | --- | --- | --- | --- | --- | --- | --- | --- | --- | --- | --- | --- | --- | --- | --- | --- | --- | --- | --- | --- | --- | --- | --- | --- | --- | --- | --- | --- | --- | --- | --- | --- | --- | --- | --- | --- | --- | --- | --- | --- | --- | --- | --- | --- | --- | --- | --- | --- | --- | --- | --- | --- | --- | --- | --- | --- | --- | --- | --- | --- | --- | --- | --- | --- | --- | --- | --- | --- | --- | --- | --- | --- | --- | --- | --- | --- | --- | --- | --- | --- | --- | --- | --- | --- | --- | --- | --- | --- | --- | --- | --- | --- | --- | --- | --- | --- | --- | --- | --- | --- | --- | --- | --- | --- | --- | --- | --- | --- | --- | --- | --- | --- | --- | --- | --- | --- | --- | --- | --- | --- | --- | --- | --- | --- | --- | --- | --- | --- | --- | --- | --- | --- | --- | --- | --- | --- | --- | --- | --- | --- | --- | --- | --- | --- | --- | --- | --- | --- | --- | --- | --- | --- | --- | --- | --- | --- | --- | --- | --- | --- | --- | --- | --- | --- | --- | --- | --- | --- | --- | --- | --- | --- | --- | --- | --- | --- | --- | --- | --- | --- | --- | --- | --- | --- | --- | --- | --- | --- | --- | --- | --- | --- | --- | --- | --- | --- | --- | --- | --- | --- | --- | --- | --- | --- | --- | --- | --- | --- | --- | --- | --- | --- | --- | --- | --- | --- | --- | --- | --- | --- | --- | --- | --- | --- | --- | --- | --- | --- | --- | --- | --- | --- | --- | --- | --- | --- | --- | --- | --- | --- | --- | --- | --- | --- | --- | --- | --- | --- | --- | --- | --- | --- | --- | --- | --- | --- | --- | --- | --- | --- | --- | --- | --- | --- | --- | --- | --- | --- | --- | --- | --- | --- | --- | --- | --- | --- | --- | --- | --- | --- | --- | --- | --- | --- | --- | --- | --- | --- | --- | --- | --- | --- | --- | --- | --- | --- | --- | --- | --- | --- | --- | --- | --- | --- | --- | --- | --- | --- | --- | --- | --- | --- | --- | --- | --- | --- | --- | --- | --- | --- | --- | --- | --- | --- | --- | --- | --- | --- | --- | --- | --- | --- | --- | --- | --- | --- | --- | --- | --- | --- | --- | --- | --- | --- | --- | --- | --- | --- | --- | --- | --- | --- | --- | --- | --- | --- | --- | --- | --- | --- | --- | --- | --- | --- | --- | --- | --- | --- | --- | --- | --- | --- | --- | --- | --- | --- | --- | --- | --- | --- | --- | --- | --- | --- | --- | --- | --- | --- | --- | --- | --- | --- | --- | --- | --- | --- | --- | --- | --- | --- | --- | --- | --- | --- | --- | --- | --- | --- | --- | --- | --- | --- | --- | --- | --- | --- | --- | --- | --- | --- | --- | --- | --- | --- | --- | --- | --- | --- | --- | --- | --- | --- | --- | --- | --- | --- | --- | --- | --- | --- | --- | --- | --- | --- | --- | --- | --- | --- | --- | --- | --- | --- | --- | --- | --- | --- | --- | --- | --- | --- | --- | --- | --- | --- | --- | --- | --- | --- | --- | --- | --- | --- | --- | --- | --- | --- | --- | --- | --- | --- | --- | --- | --- | --- | --- | --- | --- | --- | --- | --- | --- | --- | --- | --- | --- | --- | --- | --- | --- | --- | --- | --- | --- |
| |  |  |  |  |  |  |  |  |  |  |  |  |  |  |  |  |  |  |  |  |  |  |  |  |  |  |  |  |  |  |  |  |  |  |  |  |  |  |  |  |  |  |  |  |  |  |  |  |  |  |  |  |  |  |  |  |  |  | | --- | --- | --- | --- | --- | --- | --- | --- | --- | --- | --- | --- | --- | --- | --- | --- | --- | --- | --- | --- | --- | --- | --- | --- | --- | --- | --- | --- | --- | --- | --- | --- | --- | --- | --- | --- | --- | --- | --- | --- | --- | --- | --- | --- | --- | --- | --- | --- | --- | --- | --- | --- | --- | --- | --- | --- | --- | --- | | G0VEM1/1-261 | 1 | M | L | S | V | S | - | S | V | A | L | L | L | I | Q | A | S | S | F | F | K | A | G | Y | A | A | S | D | E | S | S | L | Y | G | T | W | S | S | K | S | N | Q | V | F | T | G | P | G | F | Y | D | P | V | D | E | L | 54 | | Q6CL16/1-241 | 1 | M | - | - | - | - | - | - | - | - | R | V | L | I | P | L | S | L | F | A | V | A | V | - | - | S | G | Q | N | S | D | L | E | G | T | W | S | S | K | S | N | Q | V | F | T | G | P | G | F | Y | D | P | I | E | E | L | 45 | | Q6FKL3/1-263 | 1 | M | - | - | - | - | - | - | V | R | S | L | L | F | A | L | L | S | V | A | G | L | V | - | - | A | G | D | A | A | D | L | V | G | T | W | S | S | K | S | N | Q | V | F | T | G | P | G | F | Y | D | P | I | D | E | L | 47 | | Q755J8/1-253 | 1 | M | V | Y | V | S | W | S | A | A | A | L | L | C | A | - | - | S | F | F | S | T | V | V | L | A | Q | S | A | K | D | L | Y | G | T | W | S | A | K | S | N | Q | V | F | T | G | P | G | F | Y | N | P | A | D | E | L | 53 | | A7TDM0/1-255 | 1 | M | - | - | F | G | M | R | L | L | P | F | L | A | N | A | V | I | L | F | N | F | A | I | G | D | N | D | A | A | S | L | S | G | T | W | S | S | K | S | N | Q | V | F | T | G | P | G | F | Y | D | P | V | D | E | L | 53 | | C5DBV5/1-249 | 1 | M | - | - | - | - | - | L | G | A | L | S | L | I | S | L | L | A | F | T | A | T | V | M | G | D | S | D | S | D | A | L | Y | G | T | W | T | S | K | S | N | Q | V | F | T | G | P | G | F | F | D | P | V | D | E | L | 50 | | C5DT53/1-252 | 1 | M | K | L | L | S | - | I | V | S | A | L | L | V | S | - | - | - | - | Y | S | S | Y | V | Q | A | V | D | K | K | D | L | T | G | T | W | S | S | K | S | N | Q | V | F | T | G | P | G | F | Y | D | P | V | D | E | L | 50 | | Kwal\_23.6070/1-251 | 1 | M | - | - | - | - | I | L | Q | A | S | S | L | A | V | L | L | S | L | A | T | R | V | L | C | E | S | T | A | E | S | L | Y | G | T | W | T | S | K | S | Y | Q | V | F | T | G | P | G | F | F | D | P | V | E | E | L | 51 | | Sbay\_651.28/1-263 | 1 | M | W | S | K | K | F | T | L | T | K | L | I | L | S | S | C | V | F | I | Q | K | I | H | C | E | D | S | D | S | S | L | Y | G | T | W | S | S | K | S | N | Q | V | F | T | G | P | G | F | Y | D | P | I | D | E | L | 55 | | SAKL0A02486g/1-252 | 1 | M | - | - | - | - | I | G | L | V | S | L | L | P | L | V | S | S | L | V | G | L | A | T | A | D | S | D | S | N | S | L | Y | G | T | W | S | S | K | S | N | Q | V | F | T | G | P | G | F | Y | D | P | V | D | E | L | 51 | | Q03691/1-256 | 1 | M | W | S | K | K | F | T | L | K | K | L | I | L | G | G | Y | L | F | A | Q | K | V | Y | C | E | D | E | S | N | S | I | Y | G | T | W | S | S | K | S | N | Q | V | F | T | G | P | G | F | Y | D | P | V | D | E | L | 55 | |  | | G0VEM1/1-261 | 55 | L | I | E | P | S | L | P | G | L | S | Y | S | F | T | E | D | G | W | F | E | E | A | T | Y | Q | V | A | G | N | P | K | D | P | T | C | P | E | A | S | L | I | F | Q | H | G | T | Y | E | I | L | S | N | G | T | L | 109 | | Q6CL16/1-241 | 46 | L | I | E | P | A | L | P | G | I | S | Y | S | F | T | A | D | G | Y | F | E | E | A | M | Y | Q | V | S | G | N | P | Q | D | P | K | C | P | V | A | V | L | I | F | Q | H | G | T | Y | E | V | K | D | D | G | Q | L | 100 | | Q6FKL3/1-263 | 48 | L | I | E | P | A | L | P | G | I | S | Y | S | F | T | E | D | G | W | F | E | E | A | S | Y | Q | V | S | G | N | P | K | N | P | A | C | P | K | A | S | L | I | Y | Q | H | G | K | F | E | L | L | D | N | G | T | L | 102 | | Q755J8/1-253 | 54 | L | I | E | P | S | L | P | G | I | S | Y | S | F | T | E | D | G | F | F | E | M | A | T | Y | R | V | S | G | N | P | R | N | L | A | C | P | S | A | V | M | T | F | Q | H | G | K | Y | E | I | L | A | N | G | T | L | 108 | | A7TDM0/1-255 | 54 | L | I | E | P | A | L | P | G | I | S | Y | S | F | T | E | D | G | Y | F | E | E | A | Q | Y | R | V | V | A | N | P | R | N | I | G | C | P | Q | A | V | L | I | Y | Q | H | G | K | Y | E | I | L | T | N | G | T | L | 108 | | C5DBV5/1-249 | 51 | L | I | E | P | S | L | P | G | Y | S | L | S | F | T | K | D | G | Y | F | E | E | A | A | Y | R | V | S | G | N | P | Q | D | P | H | C | P | T | A | V | L | I | Y | Q | H | G | T | Y | E | V | Q | S | N | G | S | L | 105 | | C5DT53/1-252 | 51 | I | I | E | P | S | L | P | G | I | S | Y | S | F | T | E | D | G | H | W | E | M | A | T | Y | L | V | S | G | N | P | K | D | P | T | C | P | S | A | A | I | T | F | Q | H | G | S | Y | D | L | L | D | N | G | T | L | 105 | | Kwal\_23.6070/1-251 | 52 | L | I | E | P | P | L | P | G | L | S | Y | S | F | T | E | D | G | Y | F | E | E | A | A | Y | R | V | Q | G | N | P | K | E | P | G | C | P | V | A | V | L | L | Y | Q | H | G | K | F | E | V | Q | D | N | G | S | L | 106 | | Sbay\_651.28/1-263 | 56 | L | I | E | P | S | L | P | G | I | S | Y | S | F | T | E | D | G | W | Y | E | E | A | S | Y | Q | V | S | G | N | P | R | D | P | T | C | P | M | A | S | L | I | Y | Q | H | G | K | Y | V | I | S | D | N | G | T | L | 110 | | SAKL0A02486g/1-252 | 52 | L | I | E | P | S | L | P | G | I | S | Y | S | F | T | E | D | G | W | F | E | S | A | S | Y | Q | V | K | A | N | P | K | N | P | S | C | P | Q | A | A | I | T | F | Q | H | G | R | F | E | V | S | D | N | G | T | L | 106 | | Q03691/1-256 | 56 | L | I | E | P | S | L | P | G | L | S | Y | S | F | T | E | D | G | W | Y | E | E | A | T | Y | Q | V | S | G | N | P | R | N | P | T | C | P | M | A | S | L | I | Y | Q | H | G | T | Y | N | I | S | E | N | G | T | L | 110 | |  | | G0VEM1/1-261 | 110 | I | L | T | P | I | A | V | D | G | R | Q | L | Y | S | N | P | C | D | D | K | G | V | S | T | Y | T | R | Y | N | Q | T | E | L | F | K | S | F | V | V | E | V | D | A | Y | H | G | V | Y | K | L | Q | L | Y | Q | H | 164 | | Q6CL16/1-241 | 101 | V | L | T | P | I | E | V | D | G | R | Q | L | L | S | Q | P | C | D | D | E | G | V | S | T | Y | S | R | Y | Y | Q | A | E | S | F | K | S | Y | L | V | Q | L | D | T | Y | Y | G | K | Q | S | L | Q | L | Y | G | S | 155 | | Q6FKL3/1-263 | 103 | I | L | H | P | I | E | V | D | G | R | Q | L | F | S | D | P | C | S | D | N | G | I | S | T | Y | T | R | Y | N | Q | T | E | V | F | K | S | F | E | T | F | I | D | P | Y | H | G | V | Y | T | L | Q | L | Y | Q | F | 157 | | Q755J8/1-253 | 109 | I | L | R | P | F | E | V | D | G | R | Q | L | V | S | E | P | C | V | D | K | G | V | S | T | Y | L | R | Y | S | Q | V | E | T | F | Q | R | F | A | V | E | L | D | E | Y | Q | G | K | H | A | L | H | L | F | Q | F | 163 | | A7TDM0/1-255 | 109 | I | L | T | P | F | E | V | D | G | R | Q | L | L | S | D | P | C | N | D | D | G | V | S | T | Y | S | R | Y | N | Q | S | E | T | F | L | S | F | D | I | S | I | D | D | Y | H | G | I | Y | K | L | Q | L | N | Q | F | 163 | | C5DBV5/1-249 | 106 | T | L | T | P | F | E | V | D | G | R | Q | L | L | S | S | P | C | T | D | N | G | T | S | V | Y | S | R | Y | N | Q | T | E | T | F | K | S | F | L | V | Q | L | N | N | Y | H | G | V | K | E | L | Q | L | Y | E | F | 160 | | C5DT53/1-252 | 106 | I | L | N | P | I | A | V | D | G | R | Q | L | V | S | D | P | C | N | D | N | G | V | S | T | Y | N | R | Y | N | Q | T | I | N | F | H | H | F | Y | T | Q | L | D | D | Y | H | G | M | Y | K | L | Q | L | F | Q | F | 160 | | Kwal\_23.6070/1-251 | 107 | V | L | H | P | F | E | V | D | G | R | Q | L | L | S | E | P | C | E | D | R | G | T | S | T | Y | S | R | Y | N | Q | T | E | V | Y | K | S | F | V | V | Q | D | N | E | Y | H | G | V | K | E | L | Q | L | F | A | S | 161 | | Sbay\_651.28/1-263 | 111 | V | L | N | P | I | E | V | D | G | R | Q | L | F | S | D | P | C | N | D | D | G | V | S | T | Y | S | R | Y | N | Q | T | E | T | F | K | E | Y | S | V | G | I | D | A | Y | H | G | I | Y | T | L | Q | L | Y | Q | Y | 165 | | SAKL0A02486g/1-252 | 107 | T | L | N | P | I | P | V | D | G | R | Q | L | F | S | D | P | C | N | D | N | N | I | A | S | Y | N | R | Y | V | Q | V | E | I | F | K | S | F | S | V | S | I | D | S | Y | H | G | K | Y | M | L | Q | L | Y | Q | H | 161 | | Q03691/1-256 | 111 | V | L | N | P | I | E | V | D | G | R | Q | L | F | S | D | P | C | N | D | D | G | V | S | T | Y | S | R | Y | N | Q | T | E | T | F | K | E | Y | A | V | G | I | D | P | Y | H | G | I | Y | T | L | Q | L | Y | Q | Y | 165 | |  | | G0VEM1/1-261 | 165 | D | G | S | P | M | Q | P | L | Y | L | A | Y | K | P | P | M | M | L | P | T | Q | T | L | N | P | T | E | G | A | S | N | T | D | - | S | G | A | S | A | T | D | T | - | - | - | - | - | - | - | - | Q | A | V | N | E | 210 | | Q6CL16/1-241 | 156 | D | G | A | P | L | Q | P | L | F | L | A | Y | K | P | P | V | M | L | P | T | I | T | L | N | P | T | A | S | D | D | A | Q | - | - | - | - | - | - | - | - | - | - | - | - | - | - | - | - | - | - | P | T | S | N | A | 192 | | Q6FKL3/1-263 | 158 | N | G | A | P | L | P | P | L | Y | L | A | Y | R | P | P | M | M | L | P | T | E | T | L | N | P | T | D | G | S | T | G | S | H | P | T | G | S | S | E | S | E | S | Q | E | S | D | D | T | S | K | R | S | L | L | K | 212 | | Q755J8/1-253 | 164 | D | G | S | P | V | Q | P | L | Y | L | A | Y | R | P | P | L | M | L | P | T | I | T | L | N | P | T | D | H | A | G | A | T | A | - | T | A | - | - | - | - | - | - | - | - | - | - | - | - | - | - | G | P | G | H | R | 203 | | A7TDM0/1-255 | 164 | D | G | S | P | L | Q | P | L | Y | L | A | Y | R | P | P | M | M | L | P | T | I | T | L | N | P | T | A | T | N | D | P | T | D | A | V | G | - | - | - | - | - | - | - | - | - | - | - | - | - | - | K | G | S | V | S | 204 | | C5DBV5/1-249 | 161 | D | G | T | A | M | M | P | L | Y | L | A | Y | Q | P | P | V | M | L | P | T | I | T | L | N | P | T | A | S | A | T | S | T | E | - | - | - | - | - | - | - | - | - | - | - | - | - | - | - | - | - | T | G | S | H | N | 198 | | C5DT53/1-252 | 161 | D | E | S | P | L | Q | P | L | Y | L | A | Y | R | P | P | M | M | L | P | T | E | T | L | N | P | T | E | A | A | T | S | D | N | - | A | K | - | - | - | - | - | - | - | - | - | - | - | - | - | - | P | T | G | K | Q | 200 | | Kwal\_23.6070/1-251 | 162 | D | G | S | V | M | V | P | L | Y | L | A | Y | Q | P | P | V | M | L | P | T | I | T | L | N | P | T | K | T | A | S | E | T | S | E | - | - | - | - | - | - | - | - | - | - | - | - | - | - | - | - | T | G | S | K | N | 200 | | Sbay\_651.28/1-263 | 166 | D | G | T | P | M | Q | P | L | Y | L | A | Y | R | P | P | M | M | L | P | T | E | T | L | N | P | T | A | S | A | T | S | S | G | - | A | S | S | S | D | D | T | S | - | - | - | - | - | - | - | - | A | S | S | K | K | 211 | | SAKL0A02486g/1-252 | 162 | D | G | S | P | M | Q | P | L | Y | L | A | Y | K | P | P | L | M | L | P | T | Q | T | L | N | P | T | T | P | V | T | E | S | A | - | S | S | - | - | - | - | - | - | - | - | - | - | - | - | - | - | T | G | S | S | Q | 201 | | Q03691/1-256 | 166 | D | G | T | P | M | Q | P | L | Y | L | A | Y | R | P | P | M | M | L | P | T | E | T | L | N | P | T | S | S | A | T | S | T | D | - | - | - | - | - | - | D | - | - | - | - | - | - | - | - | - | - | P | S | S | N | K | 204 | |  | | G0VEM1/1-261 | 211 | K | R | S | L | R | T | L | V | R | R | S | L | E | N | R | H | K | T | S | A | V | K | V | - | H | K | G | F | W | N | S | D | T | F | W | Y | L | S | A | G | L | L | G | L | G | S | V | M | F | L | Y | S |  | | | 261 | | Q6CL16/1-241 | 193 | K | R | D | L | I | S | H | V | R | R | S | I | E | N | R | H | K | T | N | A | V | K | K | - | - | - | T | A | P | N | I | E | K | Y | W | W | G | S | L | A | L | I | G | V | G | S | I | A | F | L | L | S |  | | | 241 | | Q6FKL3/1-263 | 213 | K | R | S | L | R | E | H | V | K | R | S | L | E | N | R | Y | K | T | N | A | V | K | K | - | S | D | S | I | F | N | A | A | F | V | W | Y | T | S | F | F | L | V | G | A | G | S | L | I | F | I | S | S |  | | | 263 | | Q755J8/1-253 | 204 | K | R | S | L | G | E | L | V | R | A | G | L | Q | D | K | H | K | T | T | A | V | R | - | - | N | P | S | L | F | N | A | A | F | Y | W | W | C | S | A | G | V | I | A | A | G | T | V | L | F | F | M | V |  | | | 253 | | A7TDM0/1-255 | 205 | K | R | S | L | R | Q | I | V | K | R | N | L | E | N | K | H | K | Q | L | A | T | K | K | - | Y | S | G | I | L | V | S | P | Y | L | W | Y | V | C | T | G | V | I | G | V | G | S | A | L | F | L | F | S |  | | | 255 | | C5DBV5/1-249 | 199 | K | R | S | L | R | G | L | V | K | R | G | L | E | N | R | Y | K | T | S | A | V | K | R | - | N | R | E | P | V | N | A | A | A | Y | W | W | T | S | V | G | L | I | A | F | G | S | A | V | F | F | V | F |  | | | 249 | | C5DT53/1-252 | 201 | K | R | S | L | R | D | T | V | R | R | N | L | E | N | R | H | R | T | N | A | V | K | S | K | P | K | S | I | L | N | S | D | L | V | W | Y | L | S | A | G | M | I | G | V | G | S | V | V | F | L | S | S |  | | | 252 | | Kwal\_23.6070/1-251 | 201 | K | R | S | L | R | S | L | V | K | R | G | L | E | N | K | H | K | T | N | A | V | K | R | - | N | K | D | P | A | N | A | A | L | Y | W | W | L | S | A | G | L | V | G | V | G | S | V | A | F | L | A | A |  | | | 251 | | Sbay\_651.28/1-263 | 212 | K | R | S | L | R | S | L | V | R | R | S | L | E | N | R | H | K | T | N | A | V | K | R | S | N | A | S | F | L | T | S | N | A | I | W | Y | I | S | A | G | M | L | G | V | G | S | L | L | F | L | A | F |  | | | 263 | | SAKL0A02486g/1-252 | 202 | K | R | S | L | R | K | M | I | K | R | G | L | E | N | K | H | K | T | N | A | V | K | K | - | S | D | G | H | Y | N | I | A | F | Y | W | W | V | S | A | A | L | I | G | I | G | S | V | A | F | L | V | V |  | | | 252 | | Q03691/1-256 | 205 | K | R | S | L | R | S | L | V | R | R | S | L | E | N | R | H | K | T | N | A | I | K | R | Q | N | T | S | F | L | T | S | N | A | I | W | Y | I | S | A | G | M | L | G | V | G | S | L | L | F | L | A | F |  | | | 256 | |
