# Supplementary material for: Plus ça change – evolutionary sequence divergence predicts protein subcellular localization signals
Source: BMC Genomics. 2014 Jan 20;15:46. doi: 10.1186/1471-2164-15-46 (PMC3906766; doi:10.1186/1471-2164-15-46)
Supplement: Additional file 2 — MSA’s of proteins for which sequence divergence changes predicted localization signals. Contains links to ortholog multiple sequence alignments of each protein in Additional file 3: Table S1. [file 1471-2164-15-46-S2.zip › Q12031.html]

|  |  |  |  |  |  |  |  |  |  |  |  |  |  |  |  |  |  |  |  |  |  |  |  |  |  |  |  |  |  |  |  |  |  |  |  |  |  |  |  |  |  |  |  |  |  |  |  |  |  |  |  |  |  |  |  |  |  |  |  |  |  |  |  |  |  |  |  |  |  |  |  |  |  |  |  |  |  |  |  |  |  |  |  |  |  |  |  |  |  |  |  |  |  |  |  |  |  |  |  |  |  |  |  |  |  |  |  |  |  |  |  |  |  |  |  |  |  |  |  |  |  |  |  |  |  |  |  |  |  |  |  |  |  |  |  |  |  |  |  |  |  |  |  |  |  |  |  |  |  |  |  |  |  |  |  |  |  |  |  |  |  |  |  |  |  |  |  |  |  |  |  |  |  |  |  |  |  |  |  |  |  |  |  |  |  |  |  |  |  |  |  |  |  |  |  |  |  |  |  |  |  |  |  |  |  |  |  |  |  |  |  |  |  |  |  |  |  |  |  |  |  |  |  |  |  |  |  |  |  |  |  |  |  |  |  |  |  |  |  |  |  |  |  |  |  |  |  |  |  |  |  |  |  |  |  |  |  |  |  |  |  |  |  |  |  |  |  |  |  |  |  |  |  |  |  |  |  |  |  |  |  |  |  |  |  |  |  |  |  |  |  |  |  |  |  |  |  |  |  |  |  |  |  |  |  |  |  |  |  |  |  |  |  |  |  |  |  |  |  |  |  |  |  |  |  |  |  |  |  |  |  |  |  |  |  |  |  |  |  |  |  |  |  |  |  |  |  |  |  |  |  |  |  |  |  |  |  |  |  |  |  |  |  |  |  |  |  |  |  |  |  |  |  |  |  |  |  |  |  |  |  |  |  |  |  |  |  |  |  |  |  |  |  |  |  |  |  |  |  |  |  |  |  |  |  |  |  |  |  |  |  |  |  |  |  |  |  |  |  |  |  |  |  |  |  |  |  |  |  |  |  |  |  |  |  |  |  |  |  |  |  |  |  |  |  |  |  |  |  |  |  |  |  |  |  |  |  |  |  |  |  |  |  |  |  |  |  |  |  |  |  |  |  |  |  |  |  |  |  |  |  |  |  |  |  |  |  |  |  |  |  |  |  |  |  |  |  |  |  |  |  |  |  |  |  |  |  |  |  |  |  |  |  |  |  |  |  |  |  |  |  |  |  |  |  |  |  |  |  |  |  |  |  |  |  |  |  |  |  |  |  |  |  |  |  |  |  |  |  |  |  |  |  |  |  |  |  |  |  |  |  |  |  |  |  |  |  |  |  |  |  |  |  |  |  |  |  |  |  |  |  |  |  |  |  |  |  |  |  |  |  |  |  |  |  |  |  |  |  |  |  |  |  |  |  |  |  |  |  |  |  |  |  |  |  |  |  |  |  |  |  |  |  |  |  |  |  |  |  |  |  |  |  |  |  |  |  |  |  |  |  |  |  |  |  |  |  |  |  |  |  |  |  |  |  |  |  |  |  |  |  |  |  |  |  |  |  |  |  |  |  |  |  |  |  |  |  |  |  |  |  |  |  |  |  |  |  |  |  |  |  |  |  |  |  |  |  |  |  |  |  |  |  |  |  |  |  |  |  |  |  |  |  |  |  |  |  |  |  |  |  |  |  |  |  |  |  |  |  |  |  |  |  |  |  |  |  |  |  |  |  |  |  |  |  |  |  |  |  |  |  |  |  |  |  |  |  |  |  |  |  |  |  |  |  |  |  |  |  |  |  |  |  |  |  |  |  |  |  |  |  |  |  |  |  |  |  |  |  |  |  |  |  |  |  |  |  |  |  |  |  |  |  |  |  |  |  |  |  |  |  |  |  |  |  |  |  |  |  |  |  |  |  |  |  |  |  |  |  |  |  |  |  |  |  |  |  |  |  |  |  |  |  |  |  |  |  |  |  |  |  |  |  |  |  |  |  |  |  |  |  |  |  |  |  |  |  |  |  |  |  |  |  |  |  |  |  |  |  |  |  |  |  |  |  |  |  |  |  |  |  |  |  |  |  |  |  |  |  |  |  |  |  |  |  |  |  |  |  |  |  |  |  |  |  |  |  |  |  |  |  |  |  |  |  |  |  |  |  |  |  |  |  |  |  |  |  |  |  |  |  |  |  |  |  |  |  |  |  |  |  |  |  |  |  |  |  |  |  |  |  |  |  |  |  |  |  |  |  |  |  |  |  |  |  |  |  |  |  |  |  |  |  |  |  |  |  |  |  |  |  |  |  |  |  |  |  |  |  |  |  |  |  |  |  |  |  |  |  |  |  |  |  |  |  |  |  |  |  |  |  |  |  |  |  |  |  |  |  |  |  |  |  |  |  |  |  |  |  |  |  |  |  |  |  |  |  |  |  |  |  |  |  |  |  |  |  |  |  |  |  |  |  |  |  |  |  |  |  |  |  |  |  |  |  |  |  |  |  |  |  |  |  |  |  |  |  |  |  |  |  |  |  |  |  |  |  |  |  |  |  |  |  |  |  |  |  |  |  |  |  |  |  |  |  |  |  |  |  |  |  |  |  |  |  |  |  |  |  |  |  |  |  |  |  |  |  |  |  |  |  |  |  |  |  |  |  |  |  |  |  |  |  |  |  |  |  |  |  |  |  |  |  |  |  |  |  |  |  |  |  |  |  |  |  |  |  |  |  |  |  |  |  |  |  |  |  |  |  |  |  |  |  |  |  |  |  |  |  |  |  |  |  |  |  |  |  |  |  |  |  |  |  |  |  |  |  |  |  |  |  |  |  |  |  |  |  |  |  |  |  |  |  |  |  |  |  |  |  |  |  |  |  |  |  |  |  |  |  |  |  |  |  |  |  |  |  |  |  |  |  |  |  |  |  |  |  |  |  |  |  |  |  |  |  |  |  |  |  |  |  |  |  |  |  |  |  |  |  |  |  |  |  |  |  |  |  |  |  |  |  |  |  |  |  |  |  |  |  |  |  |  |  |  |  |  |  |  |  |  |  |  |  |  |  |  |  |  |  |  |  |  |  |  |  |  |  |  |  |  |  |  |  |  |  |  |  |  |  |  |  |  |  |  |  |  |  |  |  |  |  |  |  |  |  |  |  |  |  |  |  |  |  |  |  |  |  |  |  |  |  |  |  |  |  |  |  |  |  |  |  |  |  |  |  |  |  |  |  |  |  |  |  |  |  |  |  |  |  |  |  |  |  |  |  |  |  |  |  |  |  |  |  |  |  |  |  |  |  |  |  |  |  |  |  |  |  |  |  |  |  |  |  |  |  |  |  |  |  |  |  |  |  |  |  |  |  |  |  |  |  |  |  |  |  |  |  |  |  |  |  |  |  |  |  |  |  |  |  |  |  |  |  |  |  |  |  |  |  |  |  |  |  |  |  |  |  |  |  |  |  |  |  |  |  |  |  |  |  |  |  |  |  |  |  |  |  |  |  |  |  |  |  |  |  |  |  |  |  |  |  |  |  |  |  |  |  |  |  |  |  |  |  |  |  |  |  |  |  |  |  |  |  |  |  |  |  |  |  |  |  |  |  |  |  |  |  |  |  |  |  |  |  |  |  |  |  |  |  |  |  |  |  |  |  |  |  |  |  |  |  |  |  |  |  |  |  |  |  |  |  |  |  |  |  |  |  |  |  |  |  |  |  |  |  |  |  |  |  |  |  |  |  |  |  |  |  |  |  |  |  |  |  |  |  |  |  |  |  |  |  |  |  |  |  |  |  |  |  |  |  |  |  |  |  |  |  |  |  |  |  |  |  |  |  |  |  |  |  |  |  |  |  |  |  |  |  |  |  |  |  |  |  |  |  |  |  |  |  |  |  |  |  |  |  |  |  |  |  |  |  |  |  |  |  |  |  |  |  |  |  |  |  |  |  |  |  |  |  |  |  |  |  |  |  |  |  |  |  |  |  |  |  |  |  |  |  |  |  |  |  |  |  |  |  |  |  |  |  |  |  |  |  |  |  |  |  |  |  |  |  |  |  |  |  |  |  |  |  |  |  |  |  |  |  |  |  |  |  |  |  |  |  |  |  |  |  |  |  |  |  |  |  |  |  |  |  |  |  |  |  |  |  |  |  |  |  |  |  |  |  |  |  |  |  |  |  |  |  |  |  |  |  |  |  |  |  |  |  |  |  |  |  |  |  |  |  |  |  |  |  |  |  |  |  |  |  |  |  |  |  |  |  |  |  |  |  |  |  |  |  |  |  |  |  |  |  |  |  |  |  |  |  |  |  |  |  |  |  |  |  |  |  |  |  |  |  |  |  |  |  |  |  |  |  |  |  |  |  |  |  |  |  |  |  |  |  |  |  |  |  |  |  |  |  |  |  |  |  |  |  |  |  |  |  |  |  |  |  |  |  |  |  |  |  |  |  |  |  |  |  |  |  |  |  |  |  |  |  |  |  |  |  |  |  |  |  |  |  |  |  |  |  |  |  |  |  |  |  |  |  |  |  |  |  |  |  |  |  |  |  |  |  |  |  |  |  |  |  |  |  |  |  |  |  |  |  |  |  |  |  |  |  |  |  |  |  |  |  |  |  |  |  |  |  |  |  |  |  |  |  |  |  |  |  |  |  |  |  |  |  |  |  |  |  |  |  |  |  |  |  |  |  |  |  |  |  |  |  |  |  |  |  |  |  |  |  |  |  |  |  |  |  |  |  |  |  |  |  |  |  |  |  |  |  |  |  |  |  |  |  |  |  |  |  |  |  |  |  |  |  |  |  |  |  |  |  |  |  |  |  |  |  |  |  |  |  |  |  |  |  |  |  |  |  |  |  |  |  |  |  |  |  |  |  |  |  |  |  |  |  |  |  |  |  |  |  |  |  |  |  |  |  |  |  |  |  |  |  |  |  |  |  |  |  |  |  |  |  |  |  |  |  |  |  |  |  |  |  |  |  |  |  |  |  |  |  |  |  |  |  |  |  |  |  |  |  |  |  |  |  |  |  |  |  |  |  |  |  |  |  |  |  |  |  |  |  |  |  |  |  |  |  |  |  |  |  |  |  |  |  |  |  |  |  |  |  |  |  |  |  |  |  |  |  |  |  |  |  |  |  |  |  |  |  |  |  |  |  |  |  |  |  |  |  |  |  |  |  |  |  |  |  |  |  |  |  |  |  |  |  |  |  |  |  |  |  |  |  |  |  |  |  |  |  |  |  |  |  |  |  |  |  |  |  |  |  |  |  |  |  |  |  |  |  |  |  |  |  |  |  |  |  |  |  |  |  |  |  |  |  |  |  |  |  |  |  |  |  |  |  |  |  |  |  |  |  |  |  |  |  |  |  |  |  |  |  |  |  |  |  |  |  |  |  |  |  |  |  |  |  |  |  |  |  |  |  |  |  |  |  |  |  |  |  |  |  |  |  |  |  |  |  |  |  |  |  |  |  |  |  |  |  |  |  |  |  |  |  |  |  |  |  |  |  |  |  |  |  |  |  |  |  |  |  |  |  |  |  |  |  |  |  |  |  |  |  |  |  |  |  |  |  |  |  |  |  |  |  |  |  |  |  |  |  |  |  |  |  |  |  |  |  |  |  |  |  |  |  |  |  |  |  |  |  |  |  |  |  |  |  |  |  |  |  |  |  |  |  |  |  |  |  |  |  |  |  |  |  |  |  |  |  |  |  |  |  |  |  |  |  |  |  |  |  |  |  |  |  |  |  |  |  |  |  |  |  |  |  |  |  |  |  |  |  |  |  |  |  |  |  |  |  |  |  |  |  |  |  |  |  |  |  |  |  |  |  |  |  |  |  |  |  |  |  |  |  |  |  |  |  |  |  |  |  |  |  |  |  |  |  |  |  |  |  |  |  |  |  |  |  |  |  |  |  |  |  |  |  |  |  |  |  |  |  |  |  |  |  |  |  |  |  |  |  |  |  |  |  |  |  |  |  |  |  |  |  |  |  |  |  |  |  |  |  |  |  |  |  |  |  |  |  |  |  |  |  |  |  |  |  |  |  |  |  |  |  |  |  |  |  |  |  |  |  |  |  |  |  |  |  |  |  |  |  |  |  |  |  |  |  |  |  |  |  |  |  |  |  |  |  |  |  |  |  |  |  |  |  |  |  |  |  |  |  |  |  |  |  |  |  |  |  |  |  |  |  |  |  |  |  |  |  |  |  |  |  |  |  |  |  |  |  |  |  |  |  |  |  |  |  |  |  |  |  |  |  |  |  |  |  |  |  |  |  |  |  |  |  |  |  |  |  |  |  |  |  |  |  |  |  |  |  |  |  |  |  |  |  |  |  |  |  |  |  |  |  |  |  |  |  |  |  |  |  |  |  |  |  |  |  |  |  |  |  |  |  |  |  |  |  |  |  |  |  |  |  |  |  |  |  |  |  |  |  |  |  |  |  |  |  |  |  |  |  |  |  |  |  |  |  |  |  |  |  |  |  |  |  |  |  |  |  |  |  |  |  |  |  |  |  |  |  |  |  |  |  |  |  |  |  |  |  |  |  |  |  |  |  |  |  |  |  |  |  |  |  |  |  |  |  |  |  |  |  |  |  |  |  |  |  |  |  |  |  |  |  |  |  |  |  |  |  |  |  |  |  |  |  |  |  |  |  |  |  |  |  |  |  |  |  |  |  |  |  |  |  |  |  |  |  |  |  |  |  |  |  |  |  |  |  |  |  |  |  |  |  |  |  |  |  |  |  |  |  |  |  |  |  |  |  |  |  |  |  |  |  |  |  |  |  |  |  |  |  |  |  |  |  |  |  |  |  |  |  |  |  |  |  |  |  |  |  |  |  |  |  |  |  |  |  |  |  |  |  |  |  |  |  |  |  |  |  |  |  |  |  |  |  |  |  |  |  |  |  |  |  |  |  |  |  |  |  |  |  |  |  |  |  |  |  |  |  |  |  |  |  |  |  |  |  |  |  |  |  |  |  |  |  |  |  |  |  |  |  |  |  |  |  |  |  |  |  |  |  |  |  |  |  |  |  |  |  |  |  |  |  |  |  |  |  |  |  |  |  |  |  |  |  |  |  |  |  |  |  |  |  |  |  |  |  |  |  |  |  |  |  |  |  |  |  |  |  |  |  |  |  |  |  |  |  |  |  |  |  |  |  |  |  |  |  |  |  |  |  |  |  |  |  |  |  |  |  |  |  |  |  |  |  |  |  |  |  |  |  |  |  |  |  |  |  |  |  |  |  |  |  |  |  |  |  |  |  |  |  |  |  |  |  |  |  |  |  |  |  |  |  |  |  |  |  |  |  |  |  |  |  |  |  |  |  |  |  |  |  |  |  |  |  |  |  |  |  |  |  |  |  |  |  |  |  |  |  |  |  |  |  |  |  |  |  |  |  |  |  |  |  |  |  |  |  |  |  |  |  |  |  |  |  |  |  |  |  |  |  |  |  |  |  |  |  |  |  |  |  |  |  |  |  |  |  |  |  |  |  |  |  |  |  |  |  |  |  |  |  |  |  |  |  |  |  |  |  |  |  |  |  |  |  |  |  |  |  |  |  |  |  |  |  |  |  |  |  |  |  |  |  |  |  |  |  |  |  |  |  |  |  |  |  |  |  |  |  |  |  |  |  |  |  |  |  |  |  |  |  |  |  |  |  |  |  |  |  |  |  |  |  |  |  |  |  |  |  |  |  |  |  |  |  |  |  |  |  |  |  |  |  |  |  |  |  |  |  |  |  |  |  |  |  |  |  |  |  |  |  |  |  |  |  |  |  |  |  |  |  |  |  |  |  |  |  |  |  |  |  |  |  |  |  |  |  |  |  |  |  |  |  |  |  |  |  |  |  |  |  |  |  |  |  |  |  |  |  |  |  |  |  |  |  |  |  |  |  |  |  |  |  |  |  |  |  |  |  |  |  |  |  |  |  |  |  |  |  |  |  |  |  |  |  |  |  |  |  |  |  |  |  |  |  |  |  |  |  |  |  |  |  |  |  |  |  |  |  |  |  |  |  |  |  |  |  |  |  |  |  |  |  |  |  |  |  |  |  |  |  |  |  |  |  |  |  |  |  |  |  |  |  |  |  |  |  |  |  |  |  |  |  |  |  |  |  |  |  |  |  |  |  |  |  |  |  |  |  |  |  |  |  |  |  |  |  |  |  |  |  |  |  |  |  |  |  |  |  |  |  |  |  |  |  |  |  |  |  |  |  |  |  |  |  |  |  |  |  |  |  |  |  |  |  |  |  |  |  |  |  |  |  |  |  |  |  |  |  |  |  |  |  |  |  |  |  |  |  |  |  |  |  |  |  |  |  |  |  |  |  |  |  |  |  |  |  |  |  |  |  |  |  |  |  |  |  |  |  |  |  |  |  |  |  |  |  |  |  |  |  |  |  |  |  |  |  |  |  |  |  |  |  |  |  |  |  |  |  |  |  |  |  |  |  |  |  |  |  |  |  |  |  |  |  |  |  |  |  |  |  |  |  |  |  |  |  |  |  |  |  |  |  |  |  |  |  |  |  |  |  |  |  |  |  |  |  |  |  |  |  |  |  |  |  |  |  |  |  |  |  |  |  |  |  |  |  |  |  |  |  |  |  |  |  |  |  |  |  |  |  |  |  |  |  |  |  |  |  |  |  |  |  |  |  |  |  |  |  |  |  |  |  |  |  |  |  |  |  |  |  |  |  |  |  |  |  |  |  |  |  |  |  |  |  |  |  |  |  |  |  |  |  |  |  |  |  |  |  |  |  |  |  |  |  |  |  |  |  |  |  |  |  |  |  |  |  |  |  |  |  |  |  |  |  |  |  |  |  |  |  |  |  |  |  |  |  |  |  |  |  |  |  |  |  |  |  |  |  |  |  |  |  |  |  |  |  |  |  |  |  |  |  |  |  |  |  |  |  |  |  |  |  |  |  |  |  |  |  |  |  |  |  |  |  |  |  |  |  |  |  |  |  |  |  |  |  |  |  |  |  |  |  |  |  |  |  |  |  |  |  |  |  |  |  |  |  |  |  |  |  |  |  |  |  |  |  |  |  |  |  |  |  |  |  |  |  |  |  |  |  |  |  |  |  |  |  |  |  |  |  |  |  |  |  |  |  |  |  |  |  |  |  |  |  |  |  |  |  |  |  |  |  |  |  |  |  |  |  |  |  |  |  |  |  |  |  |  |  |  |  |  |  |  |  |  |  |  |  |  |  |  |  |  |  |  |  |  |  |  |  |  |  |  |  |  |  |  |  |  |  |  |  |  |  |  |  |  |  |  |  |  |  |  |  |  |  |  |  |  |  |  |  |  |  |  |  |  |  |  |  |  |  |  |  |  |  |  |  |  |  |  |  |  |  |  |  |  |  |  |  |  |  |  |  |  |  |  |  |  |  |  |  |  |  |  |  |  |  |  |  |  |  |  |  |  |  |  |  |  |  |  |  |  |  |  |  |  |  |  |  |  |  |  |  |  |  |  |  |  |  |  |  |  |  |  |  |  |  |  |  |  |  |  |  |  |  |  |  |  |  |  |  |  |  |  |  |  |  |  |  |  |  |  |  |  |  |  |  |  |  |  |  |  |  |  |  |  |  |  |  |  |  |  |  |  |  |  |  |  |  |  |  |  |  |  |  |  |  |  |  |  |  |  |  |  |  |  |  |  |  |  |  |  |  |  |  |  |  |  |  |  |  |  |  |  |  |  |  |  |  |  |  |  |  |  |  |  |  |  |  |  |  |  |  |  |  |  |  |  |  |  |  |  |  |  |  |  |  |  |  |  |  |  |  |  |  |  |  |  |  |  |  |  |  |  |  |  |  |  |  |  |  |  |  |  |  |  |  |  |  |  |  |  |  |  |  |  |  |  |  |  |  |  |  |  |  |  |  |  |  |  |  |  |  |  |  |  |  |  |  |  |  |  |  |  |  |  |  |  |  |  |  |  |  |  |  |  |  |  |  |  |  |  |  |  |  |  |  |  |  |  |  |  |  |  |  |  |  |  |  |  |  |  |  |  |  |  |  |  |  |  |  |  |  |  |  |  |  |  |  |  |  |  |  |  |  |  |  |  |  |  |  |  |  |  |  |  |  |  |  |  |  |  |  |  |  |  |  |  |  |  |  |  |  |  |  |  |  |  |  |  |  |  |  |  |  |  |  |  |  |  |  |  |  |  |  |  |  |  |  |  |  |  |  |  |  |  |  |  |  |  |  |  |  |  |  |  |  |  |  |  |  |  |  |  |  |  |  |  |  |  |  |  |  |  |  |  |  |  |  |  |  |  |  |  |  |  |  |  |  |  |  |  |  |  |  |  |  |  |  |  |  |  |  |  |  |  |  |  |  |  |  |  |  |  |  |  |  |  |  |  |  |  |  |  |  |  |  |  |  |  |  |  |  |  |  |  |  |  |  |  |  |  |  |  |  |  |  |  |  |  |  |  |  |  |  |  |  |  |  |  |  |  |  |  |  |  |  |  |  |  |  |  |  |  |  |  |  |  |  |  |  |  |  |  |  |  |  |  |  |  |  |  |  |  |  |  |  |  |  |  |  |  |  |  |  |  |  |  |  |  |  |  |  |  |  |  |  |  |  |  |  |  |  |  |  |  |  |  |  |  |  |  |  |  |  |  |  |  |  |  |  |  |  |  |  |  |  |  |  |  |  |  |  |  |  |  |  |  |  |  |  |  |  |  |  |  |  |  |  |  |  |  |  |  |  |  |  |  |  |  |  |  |  |  |  |  |  |  |  |  |  |  |  |  |  |  |  |  |  |  |  |  |  |  |  |  |  |  |  |  |  |  |  |  |  |  |  |  |  |  |  |  |  |  |  |  |  |  |  |  |  |  |  |  |  |  |  |  |  |  |  |  |  |  |  |  |  |  |  |  |  |  |  |  |  |  |  |  |  |  |  |  |  |  |  |  |  |  |  |  |  |  |  |  |  |  |  |  |  |  |  |  |  |  |  |  |  |  |  |  |  |  |  |  |  |  |  |  |  |  |  |  |  |  |  |  |  |  |  |  |  |  |  |  |  |  |  |  |  |  |  |  |  |  |  |  |  |  |  |  |  |  |  |  |  |  |  |  |  |  |  |  |  |  |  |  |  |  |  |  |  |  |  |  |  |  |  |  |  |  |  |  |  |  |  |  |  |  |  |  |  |  |  |  |  |  |  |  |  |  |  |  |  |  |  |  |  |  |  |  |  |  |  |  |  |  |  |  |  |  |  |  |  |  |  |  |  |  |  |  |  |  |  |  |  |  |  |  |  |  |  |  |  |  |  |  |  |  |  |  |  |  |  |  |  |  |  |  |  |  |  |  |  |  |  |  |  |  |  |  |  |  |  |  |  |  |  |  |  |  |  |  |  |  |  |  |  |  |  |  |  |  |  |  |  |  |  |  |  |  |  |  |  |  |  |  |  |  |  |  |  |  |  |  |  |  |  |  |  |  |  |  |  |  |  |  |  |  |  |  |  |  |  |  |  |  |  |  |  |  |  |  |  |  |  |  |  |  |  |  |  |  |  |  |  |  |  |  |  |  |  |  |  |  |  |  |  |  |  |  |  |  |  |  |  |  |  |  |  |  |  |  |  |  |  |  |  |  |  |  |  |  |  |  |  |  |  |  |  |  |  |  |  |  |  |  |  |  |  |  |  |  |  |  |  |  |  |  |  |  |  |  |  |  |  |  |  |  |  |  |  |  |  |  |  |  |  |  |  |  |  |  |  |  |  |  |  |  |  |  |  |  |  |  |  |  |  |  |  |  |  |  |  |  |  |  |  |  |  |  |  |  |  |  |  |  |  |  |  |  |  |  |  |  |  |  |  |  |  |  |  |  |  |  |  |  |  |  |  |  |  |  |  |  |  |  |  |  |  |  |  |  |  |  |  |  |  |  |  |  |  |  |  |  |  |  |  |  |  |  |  |  |  |  |  |  |  |  |  |  |  |  |  |  |  |  |  |  |  |  |  |  |  |  |  |  |  |  |  |  |  |  |  |  |  |  |  |  |  |  |  |  |  |  |  |  |  |  |  |  |  |  |  |  |  |  |  |  |  |  |  |  |  |  |  |  |  |  |  |  |  |  |  |  |  |  |  |  |  |  |  |  |  |  |  |  |  |  |  |  |  |  |  |  |  |  |  |  |  |  |  |  |  |  |  |  |  |  |  |  |  |  |  |  |  |  |  |  |  |  |  |  |  |  |  |  |  |  |  |  |  |  |  |  |  |  |  |  |  |  |  |  |  |  |  |  |  |  |  |  |  |  |  |  |  |  |  |  |  |  |  |  |  |  |  |  |  |  |  |  |  |  |  |  |  |  |  |  |  |  |  |  |  |  |  |  |  |  |  |  |  |  |  |  |  |  |  |  |  |  |  |  |  |  |  |  |  |  |  |  |  |  |  |  |  |  |  |  |  |  |  |  |  |  |  |  |  |  |  |  |  |  |  |  |  |  |  |  |  |  |  |  |  |  |  |  |  |  |  |  |  |  |  |  |  |  |  |  |  |  |  |  |  |  |  |  |  |  |  |  |  |  |  |  |  |  |  |  |  |  |  |  |  |  |  |  |  |  |  |  |  |  |  |  |  |  |  |  |  |  |  |  |  |  |  |  |  |  |  |  |  |  |  |  |  |  |  |  |  |  |  |  |  |  |  |  |  |  |  |  |  |  |  |  |  |  |  |  |  |  |  |  |  |  |  |  |  |  |  |  |  |  |  |  |  |  |  |  |  |  |  |  |  |  |  |  |  |  |  |  |  |  |  |  |  |  |  |  |  |  |  |  |  |  |  |  |  |  |  |  |  |  |  |  |  |  |  |  |  |  |  |  |  |  |  |  |  |  |  |  |  |  |  |  |  |  |  |  |  |  |  |  |  |  |  |  |  |  |  |  |  |  |  |  |  |  |  |  |  |  |  |  |  |  |  |  |  |  |  |  |  |  |  |  |  |  |  |  |  |  |  |  |  |  |  |  |  |  |  |  |  |  |  |  |  |  |  |  |  |  |  |  |  |  |  |  |  |  |  |  |  |  |  |  |  |  |  |  |  |  |  |  |  |  |  |  |
| --- | --- | --- | --- | --- | --- | --- | --- | --- | --- | --- | --- | --- | --- | --- | --- | --- | --- | --- | --- | --- | --- | --- | --- | --- | --- | --- | --- | --- | --- | --- | --- | --- | --- | --- | --- | --- | --- | --- | --- | --- | --- | --- | --- | --- | --- | --- | --- | --- | --- | --- | --- | --- | --- | --- | --- | --- | --- | --- | --- | --- | --- | --- | --- | --- | --- | --- | --- | --- | --- | --- | --- | --- | --- | --- | --- | --- | --- | --- | --- | --- | --- | --- | --- | --- | --- | --- | --- | --- | --- | --- | --- | --- | --- | --- | --- | --- | --- | --- | --- | --- | --- | --- | --- | --- | --- | --- | --- | --- | --- | --- | --- | --- | --- | --- | --- | --- | --- | --- | --- | --- | --- | --- | --- | --- | --- | --- | --- | --- | --- | --- | --- | --- | --- | --- | --- | --- | --- | --- | --- | --- | --- | --- | --- | --- | --- | --- | --- | --- | --- | --- | --- | --- | --- | --- | --- | --- | --- | --- | --- | --- | --- | --- | --- | --- | --- | --- | --- | --- | --- | --- | --- | --- | --- | --- | --- | --- | --- | --- | --- | --- | --- | --- | --- | --- | --- | --- | --- | --- | --- | --- | --- | --- | --- | --- | --- | --- | --- | --- | --- | --- | --- | --- | --- | --- | --- | --- | --- | --- | --- | --- | --- | --- | --- | --- | --- | --- | --- | --- | --- | --- | --- | --- | --- | --- | --- | --- | --- | --- | --- | --- | --- | --- | --- | --- | --- | --- | --- | --- | --- | --- | --- | --- | --- | --- | --- | --- | --- | --- | --- | --- | --- | --- | --- | --- | --- | --- | --- | --- | --- | --- | --- | --- | --- | --- | --- | --- | --- | --- | --- | --- | --- | --- | --- | --- | --- | --- | --- | --- | --- | --- | --- | --- | --- | --- | --- | --- | --- | --- | --- | --- | --- | --- | --- | --- | --- | --- | --- | --- | --- | --- | --- | --- | --- | --- | --- | --- | --- | --- | --- | --- | --- | --- | --- | --- | --- | --- | --- | --- | --- | --- | --- | --- | --- | --- | --- | --- | --- | --- | --- | --- | --- | --- | --- | --- | --- | --- | --- | --- | --- | --- | --- | --- | --- | --- | --- | --- | --- | --- | --- | --- | --- | --- | --- | --- | --- | --- | --- | --- | --- | --- | --- | --- | --- | --- | --- | --- | --- | --- | --- | --- | --- | --- | --- | --- | --- | --- | --- | --- | --- | --- | --- | --- | --- | --- | --- | --- | --- | --- | --- | --- | --- | --- | --- | --- | --- | --- | --- | --- | --- | --- | --- | --- | --- | --- | --- | --- | --- | --- | --- | --- | --- | --- | --- | --- | --- | --- | --- | --- | --- | --- | --- | --- | --- | --- | --- | --- | --- | --- | --- | --- | --- | --- | --- | --- | --- | --- | --- | --- | --- | --- | --- | --- | --- | --- | --- | --- | --- | --- | --- | --- | --- | --- | --- | --- | --- | --- | --- | --- | --- | --- | --- | --- | --- | --- | --- | --- | --- | --- | --- | --- | --- | --- | --- | --- | --- | --- | --- | --- | --- | --- | --- | --- | --- | --- | --- | --- | --- | --- | --- | --- | --- | --- | --- | --- | --- | --- | --- | --- | --- | --- | --- | --- | --- | --- | --- | --- | --- | --- | --- | --- | --- | --- | --- | --- | --- | --- | --- | --- | --- | --- | --- | --- | --- | --- | --- | --- | --- | --- | --- | --- | --- | --- | --- | --- | --- | --- | --- | --- | --- | --- | --- | --- | --- | --- | --- | --- | --- | --- | --- | --- | --- | --- | --- | --- | --- | --- | --- | --- | --- | --- | --- | --- | --- | --- | --- | --- | --- | --- | --- | --- | --- | --- | --- | --- | --- | --- | --- | --- | --- | --- | --- | --- | --- | --- | --- | --- | --- | --- | --- | --- | --- | --- | --- | --- | --- | --- | --- | --- | --- | --- | --- | --- | --- | --- | --- | --- | --- | --- | --- | --- | --- | --- | --- | --- | --- | --- | --- | --- | --- | --- | --- | --- | --- | --- | --- | --- | --- | --- | --- | --- | --- | --- | --- | --- | --- | --- | --- | --- | --- | --- | --- | --- | --- | --- | --- | --- | --- | --- | --- | --- | --- | --- | --- | --- | --- | --- | --- | --- | --- | --- | --- | --- | --- | --- | --- | --- | --- | --- | --- | --- | --- | --- | --- | --- | --- | --- | --- | --- | --- | --- | --- | --- | --- | --- | --- | --- | --- | --- | --- | --- | --- | --- | --- | --- | --- | --- | --- | --- | --- | --- | --- | --- | --- | --- | --- | --- | --- | --- | --- | --- | --- | --- | --- | --- | --- | --- | --- | --- | --- | --- | --- | --- | --- | --- | --- | --- | --- | --- | --- | --- | --- | --- | --- | --- | --- | --- | --- | --- | --- | --- | --- | --- | --- | --- | --- | --- | --- | --- | --- | --- | --- | --- | --- | --- | --- | --- | --- | --- | --- | --- | --- | --- | --- | --- | --- | --- | --- | --- | --- | --- | --- | --- | --- | --- | --- | --- | --- | --- | --- | --- | --- | --- | --- | --- | --- | --- | --- | --- | --- | --- | --- | --- | --- | --- | --- | --- | --- | --- | --- | --- | --- | --- | --- | --- | --- | --- | --- | --- | --- | --- | --- | --- | --- | --- | --- | --- | --- | --- | --- | --- | --- | --- | --- | --- | --- | --- | --- | --- | --- | --- | --- | --- | --- | --- | --- | --- | --- | --- | --- | --- | --- | --- | --- | --- | --- | --- | --- | --- | --- | --- | --- | --- | --- | --- | --- | --- | --- | --- | --- | --- | --- | --- | --- | --- | --- | --- | --- | --- | --- | --- | --- | --- | --- | --- | --- | --- | --- | --- | --- | --- | --- | --- | --- | --- | --- | --- | --- | --- | --- | --- | --- | --- | --- | --- | --- | --- | --- | --- | --- | --- | --- | --- | --- | --- | --- | --- | --- | --- | --- | --- | --- | --- | --- | --- | --- | --- | --- | --- | --- | --- | --- | --- | --- | --- | --- | --- | --- | --- | --- | --- | --- | --- | --- | --- | --- | --- | --- | --- | --- | --- | --- | --- | --- | --- | --- | --- | --- | --- | --- | --- | --- | --- | --- | --- | --- | --- | --- | --- | --- | --- | --- | --- | --- | --- | --- | --- | --- | --- | --- | --- | --- | --- | --- | --- | --- | --- | --- | --- | --- | --- | --- | --- | --- | --- | --- | --- | --- | --- | --- | --- | --- | --- | --- | --- | --- | --- | --- | --- | --- | --- | --- | --- | --- | --- | --- | --- | --- | --- | --- | --- | --- | --- | --- | --- | --- | --- | --- | --- | --- | --- | --- | --- | --- | --- | --- | --- | --- | --- | --- | --- | --- | --- | --- | --- | --- | --- | --- | --- | --- | --- | --- | --- | --- | --- | --- | --- | --- | --- | --- | --- | --- | --- | --- | --- | --- | --- | --- | --- | --- | --- | --- | --- | --- | --- | --- | --- | --- | --- | --- | --- | --- | --- | --- | --- | --- | --- | --- | --- | --- | --- | --- | --- | --- | --- | --- | --- | --- | --- | --- | --- | --- | --- | --- | --- | --- | --- | --- | --- | --- | --- | --- | --- | --- | --- | --- | --- | --- | --- | --- | --- | --- | --- | --- | --- | --- | --- | --- | --- | --- | --- | --- | --- | --- | --- | --- | --- | --- | --- | --- | --- | --- | --- | --- | --- | --- | --- | --- | --- | --- | --- | --- | --- | --- | --- | --- | --- | --- | --- | --- | --- | --- | --- | --- | --- | --- | --- | --- | --- | --- | --- | --- | --- | --- | --- | --- | --- | --- | --- | --- | --- | --- | --- | --- | --- | --- | --- | --- | --- | --- | --- | --- | --- | --- | --- | --- | --- | --- | --- | --- | --- | --- | --- | --- | --- | --- | --- | --- | --- | --- | --- | --- | --- | --- | --- | --- | --- | --- | --- | --- | --- | --- | --- | --- | --- | --- | --- | --- | --- | --- | --- | --- | --- | --- | --- | --- | --- | --- | --- | --- | --- | --- | --- | --- | --- | --- | --- | --- | --- | --- | --- | --- | --- | --- | --- | --- | --- | --- | --- | --- | --- | --- | --- | --- | --- | --- | --- | --- | --- | --- | --- | --- | --- | --- | --- | --- | --- | --- | --- | --- | --- | --- | --- | --- | --- | --- | --- | --- | --- | --- | --- | --- | --- | --- | --- | --- | --- | --- | --- | --- | --- | --- | --- | --- | --- | --- | --- | --- | --- | --- | --- | --- | --- | --- | --- | --- | --- | --- | --- | --- | --- | --- | --- | --- | --- | --- | --- | --- | --- | --- | --- | --- | --- | --- | --- | --- | --- | --- | --- | --- | --- | --- | --- | --- | --- | --- | --- | --- | --- | --- | --- | --- | --- | --- | --- | --- | --- | --- | --- | --- | --- | --- | --- | --- | --- | --- | --- | --- | --- | --- | --- | --- | --- | --- | --- | --- | --- | --- | --- | --- | --- | --- | --- | --- | --- | --- | --- | --- | --- | --- | --- | --- | --- | --- | --- | --- | --- | --- | --- | --- | --- | --- | --- | --- | --- | --- | --- | --- | --- | --- | --- | --- | --- | --- | --- | --- | --- | --- | --- | --- | --- | --- | --- | --- | --- | --- | --- | --- | --- | --- | --- | --- | --- | --- | --- | --- | --- | --- | --- | --- | --- | --- | --- | --- | --- | --- | --- | --- | --- | --- | --- | --- | --- | --- | --- | --- | --- | --- | --- | --- | --- | --- | --- | --- | --- | --- | --- | --- | --- | --- | --- | --- | --- | --- | --- | --- | --- | --- | --- | --- | --- | --- | --- | --- | --- | --- | --- | --- | --- | --- | --- | --- | --- | --- | --- | --- | --- | --- | --- | --- | --- | --- | --- | --- | --- | --- | --- | --- | --- | --- | --- | --- | --- | --- | --- | --- | --- | --- | --- | --- | --- | --- | --- | --- | --- | --- | --- | --- | --- | --- | --- | --- | --- | --- | --- | --- | --- | --- | --- | --- | --- | --- | --- | --- | --- | --- | --- | --- | --- | --- | --- | --- | --- | --- | --- | --- | --- | --- | --- | --- | --- | --- | --- | --- | --- | --- | --- | --- | --- | --- | --- | --- | --- | --- | --- | --- | --- | --- | --- | --- | --- | --- | --- | --- | --- | --- | --- | --- | --- | --- | --- | --- | --- | --- | --- | --- | --- | --- | --- | --- | --- | --- | --- | --- | --- | --- | --- | --- | --- | --- | --- | --- | --- | --- | --- | --- | --- | --- | --- | --- | --- | --- | --- | --- | --- | --- | --- | --- | --- | --- | --- | --- | --- | --- | --- | --- | --- | --- | --- | --- | --- | --- | --- | --- | --- | --- | --- | --- | --- | --- | --- | --- | --- | --- | --- | --- | --- | --- | --- | --- | --- | --- | --- | --- | --- | --- | --- | --- | --- | --- | --- | --- | --- | --- | --- | --- | --- | --- | --- | --- | --- | --- | --- | --- | --- | --- | --- | --- | --- | --- | --- | --- | --- | --- | --- | --- | --- | --- | --- | --- | --- | --- | --- | --- | --- | --- | --- | --- | --- | --- | --- | --- | --- | --- | --- | --- | --- | --- | --- | --- | --- | --- | --- | --- | --- | --- | --- | --- | --- | --- | --- | --- | --- | --- | --- | --- | --- | --- | --- | --- | --- | --- | --- | --- | --- | --- | --- | --- | --- | --- | --- | --- | --- | --- | --- | --- | --- | --- | --- | --- | --- | --- | --- | --- | --- | --- | --- | --- | --- | --- | --- | --- | --- | --- | --- | --- | --- | --- | --- | --- | --- | --- | --- | --- | --- | --- | --- | --- | --- | --- | --- | --- | --- | --- | --- | --- | --- | --- | --- | --- | --- | --- | --- | --- | --- | --- | --- | --- | --- | --- | --- | --- | --- | --- | --- | --- | --- | --- | --- | --- | --- | --- | --- | --- | --- | --- | --- | --- | --- | --- | --- | --- | --- | --- | --- | --- | --- | --- | --- | --- | --- | --- | --- | --- | --- | --- | --- | --- | --- | --- | --- | --- | --- | --- | --- | --- | --- | --- | --- | --- | --- | --- | --- | --- | --- | --- | --- | --- | --- | --- | --- | --- | --- | --- | --- | --- | --- | --- | --- | --- | --- | --- | --- | --- | --- | --- | --- | --- | --- | --- | --- | --- | --- | --- | --- | --- | --- | --- | --- | --- | --- | --- | --- | --- | --- | --- | --- | --- | --- | --- | --- | --- | --- | --- | --- | --- | --- | --- | --- | --- | --- | --- | --- | --- | --- | --- | --- | --- | --- | --- | --- | --- | --- | --- | --- | --- | --- | --- | --- | --- | --- | --- | --- | --- | --- | --- | --- | --- | --- | --- | --- | --- | --- | --- | --- | --- | --- | --- | --- | --- | --- | --- | --- | --- | --- | --- | --- | --- | --- | --- | --- | --- | --- | --- | --- | --- | --- | --- | --- | --- | --- | --- | --- | --- | --- | --- | --- | --- | --- | --- | --- | --- | --- | --- | --- | --- | --- | --- | --- | --- | --- | --- | --- | --- | --- | --- | --- | --- | --- | --- | --- | --- | --- | --- | --- | --- | --- | --- | --- | --- | --- | --- | --- | --- | --- | --- | --- | --- | --- | --- | --- | --- | --- | --- | --- | --- | --- | --- | --- | --- | --- | --- | --- | --- | --- | --- | --- | --- | --- | --- | --- | --- | --- | --- | --- | --- | --- | --- | --- | --- | --- | --- | --- | --- | --- | --- | --- | --- | --- | --- | --- | --- | --- | --- | --- | --- | --- | --- | --- | --- | --- | --- | --- | --- | --- | --- | --- | --- | --- | --- | --- | --- | --- | --- | --- | --- | --- | --- | --- | --- | --- | --- | --- | --- | --- | --- | --- | --- | --- | --- | --- | --- | --- | --- | --- | --- | --- | --- | --- | --- | --- | --- | --- | --- | --- | --- | --- | --- | --- | --- | --- | --- | --- | --- | --- | --- | --- | --- | --- | --- | --- | --- | --- | --- | --- | --- | --- | --- | --- | --- | --- | --- | --- | --- | --- | --- | --- | --- | --- | --- | --- | --- | --- | --- | --- | --- | --- | --- | --- | --- | --- | --- | --- | --- | --- | --- | --- | --- | --- | --- | --- | --- | --- | --- | --- | --- | --- | --- | --- | --- | --- | --- | --- | --- | --- | --- | --- | --- | --- | --- | --- | --- | --- | --- | --- | --- | --- | --- | --- | --- | --- | --- | --- | --- | --- | --- | --- | --- | --- | --- | --- | --- | --- | --- | --- | --- | --- | --- | --- | --- | --- | --- | --- | --- | --- | --- | --- | --- | --- | --- | --- | --- | --- | --- | --- | --- | --- | --- | --- | --- | --- | --- | --- | --- | --- | --- | --- | --- | --- | --- | --- | --- | --- | --- | --- | --- | --- | --- | --- | --- | --- | --- | --- | --- | --- | --- | --- | --- | --- | --- | --- | --- | --- | --- | --- | --- | --- | --- | --- | --- | --- | --- | --- | --- | --- | --- | --- | --- | --- | --- | --- | --- | --- | --- | --- | --- | --- | --- | --- | --- | --- | --- | --- | --- | --- | --- | --- | --- | --- | --- | --- | --- | --- | --- | --- | --- | --- | --- | --- | --- | --- | --- | --- | --- | --- | --- | --- | --- | --- | --- | --- | --- | --- | --- | --- | --- | --- | --- | --- | --- | --- | --- | --- | --- | --- | --- | --- | --- | --- | --- | --- | --- | --- | --- | --- | --- | --- | --- | --- | --- | --- | --- | --- | --- | --- | --- | --- | --- | --- | --- | --- | --- | --- | --- | --- | --- | --- | --- | --- | --- | --- | --- | --- | --- | --- | --- | --- | --- | --- | --- | --- | --- | --- | --- | --- | --- | --- | --- | --- | --- | --- | --- | --- | --- | --- | --- | --- | --- | --- | --- | --- | --- | --- | --- | --- | --- | --- | --- | --- | --- | --- | --- | --- | --- | --- | --- | --- | --- | --- | --- | --- | --- | --- | --- | --- | --- | --- | --- | --- | --- | --- | --- | --- | --- | --- | --- | --- | --- | --- | --- | --- | --- | --- | --- | --- | --- | --- | --- | --- | --- | --- | --- | --- | --- | --- | --- | --- | --- | --- | --- | --- | --- | --- | --- | --- | --- | --- | --- | --- | --- | --- | --- | --- | --- | --- | --- | --- | --- | --- | --- | --- | --- | --- | --- | --- | --- | --- | --- | --- | --- | --- | --- | --- | --- | --- | --- | --- | --- | --- | --- | --- | --- | --- | --- | --- | --- | --- | --- | --- | --- | --- | --- | --- | --- | --- | --- | --- | --- | --- | --- | --- | --- | --- | --- | --- | --- | --- | --- | --- | --- | --- | --- | --- | --- | --- | --- | --- | --- | --- | --- | --- | --- | --- | --- | --- | --- | --- | --- | --- | --- | --- | --- | --- | --- | --- | --- | --- | --- | --- | --- | --- | --- | --- | --- | --- | --- | --- | --- | --- | --- | --- | --- | --- | --- | --- | --- | --- | --- | --- | --- | --- | --- | --- | --- | --- | --- | --- | --- | --- | --- | --- | --- | --- | --- | --- | --- | --- | --- | --- | --- | --- | --- | --- | --- | --- | --- | --- | --- | --- | --- | --- | --- | --- | --- | --- | --- | --- | --- | --- | --- | --- | --- | --- | --- | --- | --- | --- | --- | --- | --- | --- | --- | --- | --- | --- | --- | --- | --- | --- | --- | --- | --- | --- | --- | --- | --- | --- | --- | --- | --- | --- | --- | --- | --- | --- | --- | --- | --- | --- | --- | --- | --- | --- | --- | --- | --- | --- | --- | --- | --- | --- | --- | --- | --- | --- | --- | --- | --- | --- | --- | --- | --- | --- | --- | --- | --- | --- | --- | --- | --- | --- | --- | --- | --- | --- | --- | --- | --- | --- | --- | --- | --- | --- | --- | --- | --- | --- | --- | --- | --- | --- | --- | --- | --- | --- | --- | --- | --- | --- | --- | --- | --- | --- | --- | --- | --- | --- | --- | --- | --- | --- | --- | --- | --- | --- | --- | --- | --- | --- | --- | --- | --- | --- | --- | --- | --- | --- | --- | --- | --- | --- | --- | --- | --- | --- | --- | --- | --- | --- | --- | --- | --- | --- | --- | --- | --- | --- | --- | --- | --- | --- | --- | --- | --- | --- | --- | --- | --- | --- | --- | --- | --- | --- | --- | --- | --- | --- | --- | --- | --- | --- | --- | --- | --- | --- | --- | --- | --- | --- | --- | --- | --- | --- | --- | --- | --- | --- | --- | --- | --- | --- | --- | --- | --- | --- | --- | --- | --- | --- | --- | --- | --- | --- | --- | --- | --- | --- | --- | --- | --- | --- | --- | --- | --- | --- | --- | --- | --- | --- | --- | --- | --- | --- | --- | --- | --- | --- | --- | --- | --- | --- | --- | --- | --- | --- | --- | --- | --- | --- | --- | --- | --- | --- | --- | --- | --- | --- | --- | --- | --- | --- | --- | --- | --- | --- | --- | --- | --- | --- | --- | --- | --- | --- | --- | --- | --- | --- | --- | --- | --- | --- | --- | --- | --- | --- | --- | --- | --- | --- | --- | --- | --- | --- | --- | --- | --- | --- | --- | --- | --- | --- | --- | --- | --- | --- | --- | --- | --- | --- | --- | --- | --- | --- | --- | --- | --- | --- | --- | --- | --- | --- | --- | --- | --- | --- | --- | --- | --- | --- | --- | --- | --- | --- | --- | --- | --- | --- | --- | --- | --- | --- | --- | --- | --- | --- | --- | --- | --- | --- | --- | --- | --- | --- | --- | --- | --- | --- | --- | --- | --- | --- | --- | --- | --- | --- | --- | --- | --- | --- | --- | --- | --- | --- | --- | --- | --- | --- | --- | --- | --- | --- | --- | --- | --- | --- | --- | --- | --- | --- | --- | --- | --- | --- | --- | --- | --- | --- | --- | --- | --- | --- | --- | --- | --- | --- | --- | --- | --- | --- | --- | --- | --- | --- | --- | --- | --- | --- | --- | --- | --- | --- | --- | --- | --- | --- | --- | --- | --- | --- | --- | --- | --- | --- | --- | --- | --- | --- | --- | --- | --- | --- | --- | --- | --- | --- | --- | --- | --- | --- | --- | --- | --- | --- | --- | --- | --- | --- | --- | --- | --- | --- | --- | --- | --- | --- | --- | --- | --- | --- | --- | --- | --- | --- | --- | --- | --- | --- | --- | --- | --- | --- | --- | --- | --- | --- | --- | --- | --- | --- | --- | --- | --- | --- | --- | --- | --- | --- | --- | --- | --- | --- | --- | --- | --- | --- | --- | --- | --- | --- | --- | --- | --- | --- | --- | --- | --- | --- | --- | --- | --- | --- | --- | --- | --- | --- | --- | --- | --- | --- | --- | --- | --- | --- | --- | --- | --- | --- | --- | --- | --- | --- | --- | --- | --- | --- | --- | --- | --- | --- | --- | --- | --- | --- | --- | --- | --- | --- | --- | --- | --- | --- | --- | --- | --- | --- | --- | --- | --- | --- | --- | --- | --- | --- | --- | --- | --- | --- | --- | --- | --- | --- | --- | --- | --- | --- | --- | --- | --- | --- | --- | --- | --- | --- | --- | --- | --- | --- | --- | --- | --- | --- | --- | --- | --- | --- | --- | --- | --- | --- | --- | --- | --- | --- | --- | --- | --- | --- | --- | --- | --- | --- | --- | --- | --- | --- | --- | --- | --- | --- | --- | --- | --- | --- | --- | --- | --- | --- | --- | --- | --- | --- | --- | --- | --- | --- | --- | --- | --- | --- | --- | --- | --- | --- | --- | --- | --- | --- | --- | --- | --- | --- | --- | --- | --- | --- | --- | --- | --- | --- | --- | --- | --- | --- | --- | --- | --- | --- | --- | --- | --- | --- | --- | --- | --- | --- | --- | --- | --- | --- | --- | --- | --- | --- | --- | --- | --- | --- | --- | --- | --- | --- | --- | --- | --- | --- | --- | --- | --- | --- | --- | --- | --- | --- | --- | --- | --- | --- | --- | --- | --- | --- | --- | --- | --- | --- | --- | --- | --- | --- | --- | --- | --- | --- | --- | --- | --- | --- | --- | --- | --- | --- | --- | --- | --- | --- | --- | --- | --- | --- | --- | --- | --- | --- | --- | --- | --- | --- | --- | --- | --- | --- | --- | --- | --- | --- | --- | --- | --- | --- | --- | --- | --- | --- | --- | --- | --- | --- | --- | --- | --- | --- | --- | --- | --- | --- | --- | --- | --- | --- | --- | --- | --- | --- | --- | --- | --- | --- | --- | --- | --- | --- | --- | --- | --- | --- | --- | --- | --- | --- | --- | --- | --- | --- | --- | --- | --- | --- | --- | --- | --- | --- | --- | --- | --- | --- | --- | --- | --- | --- | --- | --- | --- | --- | --- | --- | --- | --- | --- | --- | --- | --- | --- | --- | --- | --- | --- | --- | --- | --- | --- | --- | --- | --- | --- | --- | --- | --- | --- | --- | --- | --- | --- | --- | --- | --- | --- | --- | --- | --- | --- | --- | --- | --- | --- | --- | --- | --- | --- | --- | --- | --- | --- | --- | --- | --- | --- | --- | --- | --- | --- | --- | --- | --- | --- | --- | --- | --- | --- | --- | --- | --- | --- | --- | --- | --- | --- | --- | --- | --- | --- | --- | --- | --- | --- | --- | --- | --- | --- | --- | --- | --- | --- | --- | --- | --- | --- | --- | --- | --- | --- | --- | --- | --- | --- | --- | --- | --- | --- | --- | --- | --- | --- | --- | --- | --- | --- | --- | --- | --- | --- | --- | --- | --- | --- | --- | --- | --- | --- | --- | --- | --- | --- | --- | --- | --- | --- | --- | --- | --- | --- | --- | --- | --- | --- | --- | --- | --- | --- | --- | --- | --- | --- | --- | --- | --- | --- | --- | --- | --- | --- | --- | --- | --- | --- | --- | --- | --- | --- | --- | --- | --- | --- | --- | --- | --- | --- | --- | --- | --- | --- | --- | --- | --- | --- | --- | --- | --- | --- | --- | --- | --- | --- | --- | --- | --- | --- | --- | --- | --- | --- | --- | --- | --- | --- | --- | --- | --- | --- | --- | --- | --- | --- | --- | --- | --- | --- | --- | --- | --- | --- | --- | --- | --- | --- | --- | --- | --- | --- | --- | --- | --- | --- | --- | --- | --- | --- | --- | --- | --- | --- | --- | --- | --- | --- | --- | --- | --- | --- | --- | --- | --- | --- | --- | --- | --- | --- | --- | --- | --- | --- | --- | --- | --- | --- | --- | --- | --- | --- | --- | --- | --- | --- | --- | --- | --- | --- | --- | --- | --- | --- | --- | --- | --- | --- | --- | --- | --- | --- | --- | --- | --- | --- | --- | --- | --- | --- | --- | --- | --- | --- | --- | --- | --- | --- | --- | --- | --- | --- | --- | --- | --- | --- | --- | --- | --- | --- | --- | --- | --- | --- | --- | --- | --- | --- | --- | --- | --- | --- | --- | --- | --- | --- | --- | --- | --- | --- | --- | --- | --- | --- | --- | --- | --- | --- | --- | --- | --- | --- | --- | --- | --- | --- | --- | --- | --- | --- | --- | --- | --- | --- | --- | --- | --- | --- | --- | --- | --- | --- | --- | --- | --- | --- | --- | --- | --- | --- | --- | --- | --- | --- | --- | --- | --- | --- | --- | --- | --- | --- | --- | --- | --- | --- | --- | --- | --- | --- | --- | --- | --- | --- | --- | --- | --- | --- | --- | --- | --- | --- | --- | --- | --- | --- | --- | --- | --- | --- | --- | --- | --- | --- | --- | --- | --- | --- | --- | --- | --- | --- | --- | --- | --- | --- | --- | --- | --- | --- | --- | --- | --- | --- | --- | --- | --- | --- | --- | --- | --- | --- | --- | --- | --- | --- | --- | --- | --- | --- | --- | --- | --- | --- | --- | --- | --- | --- | --- | --- | --- | --- | --- | --- | --- | --- | --- | --- | --- | --- | --- | --- | --- | --- | --- | --- | --- | --- | --- | --- | --- | --- | --- | --- | --- | --- | --- | --- | --- | --- | --- | --- | --- | --- | --- | --- | --- | --- | --- | --- | --- | --- | --- | --- | --- | --- | --- | --- | --- | --- | --- | --- | --- | --- | --- | --- | --- | --- | --- | --- | --- | --- | --- | --- | --- | --- | --- | --- | --- | --- | --- | --- | --- | --- | --- | --- | --- | --- | --- | --- | --- | --- | --- | --- | --- | --- | --- | --- | --- | --- | --- | --- | --- | --- | --- | --- | --- | --- | --- | --- | --- | --- | --- | --- | --- | --- | --- | --- | --- | --- | --- | --- | --- | --- | --- | --- | --- | --- | --- | --- | --- | --- | --- | --- | --- | --- | --- | --- | --- | --- | --- | --- | --- | --- | --- | --- | --- | --- | --- | --- | --- | --- | --- | --- | --- | --- | --- | --- | --- | --- | --- | --- | --- | --- | --- | --- | --- | --- | --- | --- | --- | --- | --- | --- | --- | --- | --- | --- | --- | --- | --- | --- | --- | --- | --- | --- | --- | --- | --- | --- | --- | --- | --- | --- | --- | --- | --- | --- | --- | --- | --- | --- | --- | --- | --- | --- | --- | --- | --- | --- | --- | --- | --- | --- | --- | --- | --- | --- | --- | --- | --- | --- | --- | --- | --- | --- | --- | --- | --- | --- | --- | --- | --- | --- | --- | --- | --- | --- | --- | --- | --- | --- | --- | --- | --- | --- | --- | --- | --- | --- | --- | --- | --- | --- | --- | --- | --- | --- | --- | --- | --- | --- | --- | --- | --- | --- | --- | --- | --- | --- | --- | --- | --- | --- | --- | --- | --- | --- | --- | --- | --- | --- | --- | --- | --- | --- | --- | --- | --- | --- | --- | --- | --- | --- | --- | --- | --- | --- | --- | --- | --- | --- | --- | --- | --- | --- | --- | --- | --- | --- | --- | --- | --- | --- | --- | --- | --- | --- | --- | --- | --- | --- | --- | --- | --- | --- | --- | --- | --- | --- | --- | --- | --- | --- | --- | --- | --- | --- | --- | --- | --- | --- | --- | --- | --- | --- | --- | --- | --- | --- | --- | --- | --- | --- | --- | --- | --- | --- | --- | --- | --- | --- | --- | --- | --- | --- | --- | --- | --- | --- | --- | --- | --- | --- | --- | --- | --- | --- | --- | --- | --- | --- | --- | --- | --- | --- | --- | --- | --- | --- | --- | --- | --- | --- | --- | --- | --- | --- | --- | --- | --- | --- | --- | --- | --- | --- | --- | --- | --- | --- | --- | --- | --- | --- | --- | --- | --- | --- | --- | --- | --- | --- | --- | --- | --- | --- | --- | --- | --- | --- | --- | --- | --- | --- | --- | --- | --- | --- | --- | --- | --- | --- | --- | --- | --- | --- | --- | --- | --- | --- | --- | --- | --- | --- | --- | --- | --- | --- | --- | --- | --- | --- | --- | --- | --- | --- | --- | --- | --- | --- | --- | --- | --- | --- | --- | --- | --- | --- | --- | --- | --- | --- | --- | --- | --- | --- | --- | --- | --- | --- | --- | --- | --- | --- | --- | --- | --- | --- | --- | --- | --- | --- | --- | --- | --- | --- | --- | --- | --- | --- | --- | --- | --- | --- | --- | --- | --- | --- | --- | --- | --- | --- | --- | --- | --- | --- | --- | --- | --- | --- | --- | --- | --- | --- | --- | --- | --- | --- | --- | --- | --- | --- | --- | --- | --- | --- | --- | --- | --- | --- | --- | --- | --- | --- | --- | --- | --- | --- | --- | --- | --- | --- | --- | --- | --- | --- | --- | --- | --- | --- | --- | --- | --- | --- | --- | --- | --- | --- | --- | --- | --- | --- | --- | --- | --- | --- | --- | --- | --- | --- | --- | --- | --- | --- | --- | --- | --- | --- | --- | --- | --- | --- | --- | --- | --- | --- | --- | --- | --- | --- | --- | --- | --- | --- | --- | --- | --- | --- | --- | --- | --- | --- | --- | --- | --- | --- | --- | --- | --- | --- | --- | --- | --- | --- | --- | --- | --- | --- | --- | --- | --- | --- | --- | --- | --- | --- | --- | --- | --- | --- | --- | --- | --- | --- | --- | --- | --- | --- | --- | --- | --- | --- | --- | --- | --- | --- | --- | --- | --- | --- | --- | --- | --- | --- | --- | --- | --- | --- | --- | --- | --- | --- | --- | --- | --- | --- | --- | --- | --- | --- | --- | --- | --- | --- | --- | --- | --- | --- | --- | --- | --- | --- | --- | --- | --- | --- | --- | --- | --- | --- | --- | --- | --- | --- | --- | --- | --- | --- | --- | --- | --- | --- | --- | --- | --- | --- | --- | --- | --- | --- | --- | --- | --- | --- | --- | --- | --- | --- | --- | --- | --- | --- | --- | --- | --- | --- | --- | --- | --- | --- | --- | --- | --- | --- | --- | --- | --- | --- | --- | --- | --- | --- | --- | --- | --- | --- | --- | --- | --- | --- | --- | --- | --- | --- | --- | --- | --- | --- | --- | --- | --- | --- | --- | --- | --- | --- | --- | --- | --- | --- | --- | --- | --- | --- | --- | --- | --- | --- | --- | --- | --- | --- | --- | --- | --- | --- | --- | --- | --- | --- | --- | --- | --- | --- | --- | --- | --- | --- | --- | --- | --- | --- | --- | --- | --- | --- | --- | --- | --- | --- | --- | --- | --- | --- | --- | --- | --- | --- | --- | --- | --- | --- | --- | --- | --- | --- | --- | --- | --- | --- | --- | --- | --- | --- | --- | --- | --- | --- | --- | --- | --- | --- | --- | --- | --- | --- | --- | --- | --- | --- | --- | --- | --- | --- | --- | --- | --- | --- | --- | --- | --- | --- | --- | --- | --- | --- | --- | --- | --- | --- | --- | --- | --- | --- | --- | --- | --- | --- | --- | --- | --- | --- | --- | --- | --- | --- | --- | --- | --- | --- | --- | --- | --- | --- | --- | --- | --- | --- | --- | --- | --- | --- | --- | --- | --- | --- | --- | --- | --- | --- | --- | --- | --- | --- | --- | --- | --- | --- | --- | --- | --- | --- | --- | --- | --- | --- | --- | --- | --- | --- | --- | --- | --- | --- | --- | --- | --- | --- | --- | --- | --- | --- | --- | --- | --- | --- | --- | --- | --- | --- | --- | --- | --- | --- | --- | --- | --- | --- | --- | --- | --- | --- | --- | --- | --- | --- | --- | --- | --- | --- | --- | --- | --- | --- | --- | --- | --- | --- | --- | --- | --- | --- | --- | --- | --- | --- | --- | --- | --- | --- | --- | --- | --- | --- | --- | --- | --- | --- | --- | --- | --- | --- | --- | --- | --- | --- | --- | --- | --- | --- | --- | --- | --- | --- | --- | --- | --- | --- | --- | --- | --- | --- | --- | --- | --- | --- | --- | --- | --- | --- | --- | --- | --- | --- | --- | --- | --- | --- | --- | --- | --- | --- | --- | --- | --- | --- | --- | --- | --- | --- | --- | --- | --- | --- | --- | --- | --- | --- | --- | --- | --- | --- | --- | --- | --- | --- | --- | --- | --- | --- | --- | --- | --- | --- | --- | --- | --- | --- | --- | --- | --- | --- | --- | --- | --- | --- | --- | --- | --- | --- | --- | --- | --- | --- | --- | --- | --- | --- | --- | --- | --- | --- | --- | --- | --- | --- | --- | --- | --- | --- | --- | --- | --- | --- | --- | --- | --- | --- | --- | --- | --- | --- | --- | --- | --- | --- | --- | --- | --- | --- | --- | --- | --- | --- | --- | --- | --- | --- | --- | --- | --- | --- | --- | --- | --- | --- | --- | --- | --- | --- | --- | --- | --- | --- | --- | --- | --- | --- | --- | --- | --- | --- | --- | --- | --- | --- | --- | --- | --- | --- | --- | --- | --- | --- | --- | --- | --- | --- | --- | --- | --- | --- | --- | --- | --- | --- | --- | --- | --- | --- | --- | --- | --- | --- | --- | --- | --- | --- | --- | --- | --- | --- | --- | --- | --- | --- | --- | --- | --- | --- | --- | --- | --- | --- | --- | --- | --- | --- | --- | --- | --- | --- | --- | --- | --- | --- | --- | --- | --- | --- | --- | --- | --- | --- | --- | --- | --- | --- | --- | --- | --- | --- | --- | --- | --- | --- | --- | --- | --- | --- | --- | --- | --- | --- | --- | --- | --- | --- | --- | --- | --- | --- | --- | --- | --- | --- | --- | --- | --- | --- | --- | --- | --- | --- | --- | --- | --- | --- | --- | --- | --- | --- | --- | --- | --- | --- | --- | --- | --- | --- | --- | --- | --- | --- | --- | --- | --- | --- | --- | --- | --- | --- | --- | --- | --- | --- | --- | --- | --- | --- | --- | --- | --- | --- | --- | --- | --- | --- | --- | --- | --- | --- | --- | --- | --- | --- | --- | --- | --- | --- | --- | --- | --- | --- | --- | --- | --- | --- | --- | --- | --- | --- | --- | --- | --- | --- | --- | --- | --- | --- | --- | --- | --- | --- | --- | --- | --- | --- | --- | --- | --- | --- | --- | --- | --- | --- | --- | --- | --- | --- | --- | --- | --- | --- | --- | --- | --- | --- | --- | --- | --- | --- | --- | --- | --- | --- | --- | --- | --- | --- | --- | --- | --- | --- | --- | --- | --- | --- | --- | --- | --- | --- | --- | --- | --- | --- | --- | --- | --- | --- | --- | --- | --- | --- | --- | --- | --- | --- | --- | --- | --- | --- | --- | --- | --- | --- | --- | --- | --- | --- | --- | --- | --- | --- | --- | --- | --- | --- | --- | --- | --- | --- | --- | --- | --- | --- | --- | --- | --- | --- | --- | --- | --- | --- | --- | --- | --- | --- | --- | --- | --- | --- | --- | --- | --- | --- | --- | --- | --- | --- | --- | --- | --- | --- | --- | --- | --- | --- | --- | --- | --- | --- | --- | --- | --- | --- | --- | --- | --- | --- | --- | --- | --- | --- | --- | --- | --- | --- | --- | --- | --- | --- | --- | --- | --- | --- | --- | --- | --- | --- | --- | --- | --- | --- | --- | --- | --- | --- | --- | --- | --- | --- | --- | --- | --- | --- | --- | --- | --- | --- | --- | --- | --- | --- | --- | --- | --- | --- | --- | --- | --- | --- | --- | --- | --- | --- | --- | --- | --- | --- | --- | --- | --- | --- | --- | --- | --- | --- | --- | --- | --- | --- | --- | --- | --- | --- | --- | --- | --- | --- | --- | --- | --- | --- | --- | --- | --- | --- | --- | --- | --- | --- | --- | --- | --- | --- | --- | --- | --- | --- | --- | --- | --- | --- | --- | --- | --- | --- | --- | --- | --- | --- | --- | --- | --- | --- | --- | --- | --- | --- | --- | --- | --- | --- | --- | --- | --- | --- | --- | --- | --- | --- | --- | --- | --- | --- | --- | --- | --- | --- | --- | --- | --- | --- | --- | --- | --- | --- | --- | --- | --- | --- | --- | --- | --- | --- | --- | --- | --- | --- | --- | --- | --- | --- | --- | --- | --- | --- | --- | --- | --- | --- | --- | --- | --- | --- | --- | --- | --- | --- | --- | --- | --- | --- | --- | --- | --- | --- | --- | --- | --- | --- | --- | --- | --- | --- | --- | --- | --- | --- | --- | --- | --- | --- | --- | --- | --- | --- | --- | --- | --- | --- | --- | --- | --- | --- | --- | --- | --- | --- | --- | --- | --- | --- | --- | --- | --- | --- | --- | --- | --- | --- | --- | --- | --- | --- | --- | --- | --- | --- | --- | --- | --- | --- | --- | --- | --- | --- | --- | --- | --- | --- | --- | --- | --- | --- | --- | --- | --- | --- | --- | --- | --- | --- | --- | --- | --- | --- | --- | --- | --- | --- | --- | --- | --- | --- | --- | --- | --- | --- | --- | --- | --- | --- | --- | --- | --- | --- | --- | --- | --- | --- | --- | --- | --- | --- | --- | --- | --- | --- | --- | --- | --- | --- | --- | --- | --- | --- | --- | --- | --- | --- | --- | --- | --- | --- | --- | --- | --- | --- | --- | --- | --- | --- | --- | --- | --- | --- | --- | --- | --- | --- | --- | --- | --- | --- | --- |
| |  |  |  |  |  |  |  |  |  |  |  |  |  |  |  |  |  |  |  |  |  |  |  |  |  |  |  |  |  |  |  |  |  |  |  |  |  |  |  |  |  |  |  |  |  |  |  |  |  |  |  |  |  |  |  |  |  |  | | --- | --- | --- | --- | --- | --- | --- | --- | --- | --- | --- | --- | --- | --- | --- | --- | --- | --- | --- | --- | --- | --- | --- | --- | --- | --- | --- | --- | --- | --- | --- | --- | --- | --- | --- | --- | --- | --- | --- | --- | --- | --- | --- | --- | --- | --- | --- | --- | --- | --- | --- | --- | --- | --- | --- | --- | --- | --- | | G0V632/1-554 | 1 | - | - | - | - | - | - | - | - | - | - | - | - | - | - | - | - | - | - | - | - | - | - | - | - | - | M | L | I | I | K | R | F | Q | H | N | S | S | S | S | G | Y | P | M | R | R | F | F | E | N | E | T | S | E | L | K | 30 | | Q6CNB1/1-572 | 1 | - | - | - | - | M | S | F | R | C | S | V | K | F | G | E | R | R | G | S | F | L | M | S | R | G | L | S | N | I | S | R | F | P | K | P | K | D | A | E | - | - | - | I | E | N | F | V | N | A | Q | A | E | S | I | A | 48 | | Q6FKR5/1-571 | 1 | - | - | - | M | L | R | N | Y | P | K | N | L | I | G | P | R | - | - | - | - | - | - | - | - | - | - | - | T | I | A | R | T | T | H | T | S | C | Y | N | G | G | S | I | K | T | Y | V | E | D | Q | V | K | A | V | T | 41 | | Q750K8/1-557 | 1 | - | - | - | - | - | - | - | - | - | - | - | - | - | - | - | - | - | M | A | S | V | W | R | R | S | L | K | T | L | S | S | Y | P | R | P | S | G | S | S | - | - | - | A | K | R | F | V | E | E | Q | T | T | E | L | E | 35 | | A7TQF1/1-561 | 1 | - | - | - | - | - | - | - | - | - | - | - | - | - | - | M | S | K | M | S | K | L | I | L | T | - | - | - | - | - | R | R | F | N | H | T | T | S | Y | S | - | - | - | - | - | - | - | - | - | K | D | L | A | Q | I | E | 27 | | C5DXF8/1-567 | 1 | - | - | - | - | M | I | N | H | V | T | I | K | G | T | M | Y | - | - | - | - | - | - | - | N | G | T | R | S | L | S | S | S | T | S | - | N | S | S | S | V | L | N | L | K | S | F | V | A | E | E | V | R | K | I | E | 43 | | Sbay\_661.12/1-568 | 1 | - | - | - | - | - | - | - | M | L | F | N | R | T | T | S | R | A | L | K | K | L | V | L | S | P | D | K | S | L | R | A | Y | S | S | - | K | A | K | S | - | - | - | I | E | D | F | L | S | S | E | S | V | K | V | E | 44 | | SAKL0B03212g/1-564 | 1 | - | - | - | M | L | K | S | H | S | K | I | R | M | A | A | K | - | - | - | - | - | - | - | R | M | N | H | V | L | S | V | Y | P | K | P | S | D | Q | E | - | - | - | T | K | K | F | I | T | E | Q | V | S | Q | V | E | 42 | | Q12031/1-575 | 1 | M | I | T | M | I | N | N | K | T | F | N | R | K | T | T | G | T | L | K | K | L | V | L | S | S | D | K | S | L | R | R | S | F | N | - | G | A | S | S | - | - | - | T | K | D | F | V | F | S | E | S | S | K | V | E | 51 | |  | | G0V632/1-554 | 31 | A | W | W | D | S | P | R | F | A | N | V | K | R | P | Y | Q | P | I | D | V | I | K | H | R | G | S | M | P | - | - | L | S | A | T | H | Y | P | S | S | Y | Q | A | G | K | L | F | K | L | L | Q | E | N | F | Q | N | 83 | | Q6CNB1/1-572 | 49 | K | W | W | E | S | D | R | F | K | N | I | K | R | P | Y | T | P | L | D | V | V | K | H | R | G | S | L | G | - | - | D | - | D | V | V | Y | G | S | S | I | Q | A | K | R | L | F | Q | V | L | E | N | K | F | K | N | 100 | | Q6FKR5/1-571 | 42 | E | W | F | G | S | D | R | F | Q | N | I | K | R | T | Y | T | P | L | D | V | V | K | H | R | G | S | I | N | - | - | P | C | E | V | I | Y | P | S | A | F | P | S | R | K | L | F | S | L | V | E | E | H | F | K | D | 94 | | Q750K8/1-557 | 36 | Q | L | W | S | S | P | R | F | Q | E | I | T | R | P | Y | T | P | L | D | V | V | K | H | R | G | S | L | G | - | - | - | - | R | V | G | Y | A | S | S | V | Q | A | E | R | L | H | D | L | L | E | D | K | F | H | K | 86 | | A7TQF1/1-561 | 28 | A | F | F | N | K | K | R | F | K | N | V | K | R | P | Y | K | S | I | D | V | L | K | H | R | G | S | I | N | Y | L | Q | E | N | A | T | P | V | S | S | L | I | S | R | K | L | F | N | L | L | E | T | H | F | K | E | 82 | | C5DXF8/1-567 | 44 | D | W | W | G | Q | P | R | Y | K | D | V | E | R | P | Y | S | S | L | D | V | V | K | H | R | G | S | L | P | - | - | V | E | C | N | R | Y | A | S | S | F | Q | A | Q | K | L | F | R | L | L | E | D | K | F | E | K | 96 | | Sbay\_661.12/1-568 | 45 | K | W | W | A | S | R | R | F | K | D | V | S | R | P | Y | S | A | I | D | V | V | R | H | R | G | S | L | P | - | - | A | N | T | S | I | Y | P | S | S | H | Q | A | R | K | L | F | N | L | L | E | E | N | F | K | R | 97 | | SAKL0B03212g/1-564 | 43 | Q | W | W | G | T | P | R | F | E | N | I | K | R | P | Y | S | A | L | D | V | V | K | H | R | G | S | L | G | - | - | G | - | S | V | S | Y | P | S | S | V | Q | A | Q | R | L | F | R | L | L | Q | E | K | F | S | Q | 94 | | Q12031/1-575 | 52 | E | W | W | E | S | A | R | F | K | N | I | S | R | P | Y | S | A | T | D | V | V | K | H | R | G | S | L | P | - | - | A | N | T | S | I | Y | P | S | S | Y | Q | A | R | K | L | F | N | L | L | E | E | N | F | K | N | 104 | |  | | G0V632/1-554 | 84 | K | T | P | L | H | T | L | G | V | I | D | P | V | Q | M | T | Q | L | S | R | C | E | Q | L | K | V | V | Y | L | S | G | W | A | C | S | S | T | L | V | T | P | S | N | E | V | S | P | D | F | G | D | Y | P | Y | T | 138 | | Q6CNB1/1-572 | 101 | K | L | P | V | H | T | L | G | V | I | D | P | V | Q | M | S | Q | L | A | R | C | D | D | I | E | V | A | Y | V | S | G | W | A | C | S | S | T | M | V | G | S | T | N | E | V | S | P | D | F | G | D | Y | P | Y | D | 155 | | Q6FKR5/1-571 | 95 | K | K | P | L | H | T | L | G | V | L | D | P | V | Q | M | T | Q | L | S | R | C | E | D | L | K | V | A | Y | V | S | G | W | A | C | S | S | T | M | V | G | S | T | N | D | V | S | P | D | F | G | D | Y | P | Y | N | 149 | | Q750K8/1-557 | 87 | R | E | A | V | S | T | L | G | V | I | D | P | V | Q | M | T | Q | L | A | R | C | E | G | I | E | A | A | Y | V | S | G | W | A | C | S | S | T | M | V | G | S | T | N | E | V | S | P | D | F | G | D | Y | P | Y | D | 141 | | A7TQF1/1-561 | 83 | R | K | P | L | H | T | L | G | V | L | D | P | V | Q | M | S | Q | L | A | R | C | E | N | I | K | V | A | Y | V | S | G | W | A | C | S | A | N | - | - | - | A | N | D | N | V | S | P | D | F | G | D | Y | P | Y | D | 134 | | C5DXF8/1-567 | 97 | K | L | P | L | H | T | L | G | V | I | D | P | V | Q | M | T | Q | L | A | R | S | K | E | I | E | V | A | Y | L | S | G | W | A | C | S | S | T | L | V | G | S | T | N | E | V | S | P | D | F | G | D | Y | P | Y | N | 151 | | Sbay\_661.12/1-568 | 98 | G | T | P | L | H | T | L | G | V | I | D | P | V | Q | M | S | Q | L | A | R | C | E | K | I | K | V | A | Y | I | S | G | W | A | C | S | S | T | L | V | G | S | T | N | E | V | S | P | D | F | G | D | Y | P | Y | D | 152 | | SAKL0B03212g/1-564 | 95 | G | E | P | V | H | T | L | G | V | I | D | P | V | Q | M | S | Q | L | S | R | C | D | D | I | H | V | C | Y | I | S | G | W | A | C | S | S | T | M | V | G | S | T | N | D | V | S | P | D | F | G | D | Y | P | Y | D | 149 | | Q12031/1-575 | 105 | G | T | P | L | H | T | L | G | V | I | D | P | V | Q | M | S | Q | L | A | R | C | R | N | I | K | V | A | Y | I | S | G | W | A | C | S | S | T | L | V | G | S | T | N | E | V | S | P | D | F | G | D | Y | P | Y | D | 159 | |  | | G0V632/1-554 | 139 | T | V | P | N | Q | V | E | R | I | F | K | A | Q | Q | L | H | D | R | K | A | F | L | E | S | F | - | - | - | - | E | S | - | - | - | K | T | D | K | M | V | D | Y | L | K | P | I | I | A | D | G | D | M | G | - | G | 185 | | Q6CNB1/1-572 | 156 | T | V | P | N | Q | V | E | R | I | F | K | A | Q | Q | M | H | D | K | K | Q | L | L | Q | F | L | - | - | - | - | E | S | - | - | K | D | T | N | K | R | V | D | Y | L | K | P | I | I | A | D | A | D | M | G | H | G | 204 | | Q6FKR5/1-571 | 150 | T | V | P | N | Q | V | E | R | I | M | K | A | Q | Q | M | H | D | R | K | A | Y | L | E | K | F | M | T | D | Q | K | S | - | - | S | S | N | A | E | F | I | D | Y | L | K | P | I | I | A | D | G | D | M | G | H | G | 202 | | Q750K8/1-557 | 142 | T | V | P | N | Q | V | E | R | I | F | R | A | Q | Q | M | H | D | R | K | A | F | L | A | - | - | - | - | - | - | D | G | - | - | A | E | G | R | A | S | T | D | Y | L | K | P | I | I | A | D | G | D | M | G | H | G | 188 | | A7TQF1/1-561 | 135 | T | V | P | N | Q | V | G | R | I | F | N | A | Q | L | M | H | D | K | K | L | H | L | Q | N | F | - | - | - | - | N | S | G | R | N | N | N | S | D | K | I | D | Y | L | K | P | I | I | A | D | A | D | M | G | H | G | 185 | | C5DXF8/1-567 | 152 | T | V | P | N | Q | V | E | R | I | F | K | A | Q | Q | M | H | D | R | K | L | F | L | E | K | V | - | - | - | - | E | G | - | - | L | T | N | D | D | T | T | D | Y | L | K | P | I | I | A | D | A | D | M | G | H | G | 200 | | Sbay\_661.12/1-568 | 153 | T | V | P | N | Q | V | E | R | I | F | K | A | Q | E | L | H | D | R | K | A | F | L | E | - | - | - | - | - | - | A | S | - | - | I | K | G | S | T | P | V | D | Y | L | K | P | I | I | A | D | A | D | M | G | H | G | 199 | | SAKL0B03212g/1-564 | 150 | T | V | P | N | Q | V | E | R | I | F | K | A | Q | V | M | H | D | K | K | S | L | L | E | N | L | - | - | - | - | N | T | - | - | - | - | G | V | P | R | I | D | Y | L | K | P | I | I | A | D | A | D | T | G | H | G | 196 | | Q12031/1-575 | 160 | T | V | P | N | Q | V | E | R | I | F | K | A | Q | Q | L | H | D | R | K | A | F | L | E | - | - | - | - | - | - | A | S | - | - | I | K | G | S | T | P | V | D | Y | L | K | P | I | I | A | D | A | D | M | G | H | G | 206 | |  | | G0V632/1-554 | 186 | S | P | N | M | C | M | K | L | A | K | L | F | A | E | K | G | A | A | A | I | H | L | E | D | Q | L | L | G | G | K | R | C | G | H | L | G | G | A | V | I | V | P | T | G | D | Q | L | S | R | L | V | A | T | R | F | 240 | | Q6CNB1/1-572 | 205 | G | T | T | T | V | M | K | L | A | K | L | F | A | E | K | G | A | A | G | I | H | L | E | D | Q | L | H | G | G | K | R | C | G | H | L | G | G | A | V | I | V | P | T | S | T | H | I | S | R | L | V | A | T | R | L | 259 | | Q6FKR5/1-571 | 203 | G | P | T | T | V | M | K | V | A | K | L | F | A | E | K | G | A | A | A | V | H | L | E | D | Q | L | V | G | G | K | R | C | G | H | L | S | G | A | V | L | V | P | T | G | A | H | L | S | R | L | I | S | T | R | F | 257 | | Q750K8/1-557 | 189 | G | S | T | T | V | M | K | L | A | K | L | F | A | E | K | G | A | A | A | I | H | L | E | D | Q | M | H | G | G | K | R | C | G | H | L | G | G | A | V | L | A | P | T | Y | V | H | I | S | R | L | I | A | T | R | L | 243 | | A7TQF1/1-561 | 186 | G | I | T | T | V | M | K | L | A | K | L | F | A | E | K | G | A | S | A | I | H | L | E | D | Q | L | V | G | S | K | K | C | G | H | L | G | G | T | V | V | V | P | T | S | T | H | L | Q | R | I | I | A | T | R | F | 240 | | C5DXF8/1-567 | 201 | G | N | T | T | V | M | K | L | A | K | L | F | A | E | K | G | A | A | A | I | H | L | E | D | Q | M | V | G | G | K | R | C | G | H | L | G | G | A | V | I | V | P | S | S | T | Q | L | S | R | L | I | A | T | R | L | 255 | | Sbay\_661.12/1-568 | 200 | G | P | T | T | V | M | K | V | A | K | L | F | A | E | K | G | A | A | A | I | H | L | E | D | Q | M | V | G | G | K | R | C | G | H | L | S | G | A | V | L | V | P | T | A | T | H | L | M | R | L | I | S | T | R | F | 254 | | SAKL0B03212g/1-564 | 197 | G | T | T | A | V | M | K | L | A | K | L | F | A | E | K | G | A | A | A | I | H | L | E | D | Q | L | H | G | G | K | R | C | G | H | L | G | G | A | V | I | V | P | T | S | T | H | T | S | R | L | I | A | T | R | L | 251 | | Q12031/1-575 | 207 | G | P | T | T | V | M | K | V | A | K | L | F | A | E | K | G | A | A | G | I | H | L | E | D | Q | M | V | G | G | K | R | C | G | H | L | S | G | A | V | L | V | P | T | A | T | H | L | M | R | L | I | S | T | R | F | 261 | |  | | G0V632/1-554 | 241 | Q | W | D | I | M | G | T | E | N | L | I | I | A | R | T | D | S | C | N | A | K | L | L | S | S | S | S | D | P | R | D | H | E | F | I | K | G | V | I | - | - | D | P | N | L | T | - | A | W | S | E | E | L | I | D | 292 | | Q6CNB1/1-572 | 260 | Q | W | D | I | M | G | T | E | N | L | V | I | A | R | T | D | S | C | N | A | K | L | L | S | S | N | V | D | P | R | D | H | E | H | I | Q | G | T | I | - | - | N | K | N | V | K | - | P | W | S | D | F | L | S | E | 311 | | Q6FKR5/1-571 | 258 | Q | W | D | I | M | G | T | E | N | L | I | L | A | R | T | D | S | C | N | A | K | L | I | S | S | D | I | D | P | R | D | H | S | F | I | Q | G | I | L | - | - | N | P | S | K | T | D | R | W | A | D | K | L | L | E | 310 | | Q750K8/1-557 | 244 | Q | W | D | I | M | G | T | Q | N | L | L | I | A | R | T | D | S | A | N | A | K | L | I | A | S | S | S | D | P | R | D | H | E | F | I | L | G | T | T | - | - | Q | P | A | A | Q | - | P | W | A | E | L | V | L | D | 295 | | A7TQF1/1-561 | 241 | Q | W | D | L | M | G | A | E | N | L | V | I | A | R | T | D | S | C | N | S | N | L | I | S | S | D | I | D | A | R | D | H | K | F | I | K | G | I | A | V | E | N | N | D | I | R | - | P | V | S | E | V | L | L | E | 294 | | C5DXF8/1-567 | 256 | Q | W | D | I | M | G | T | E | N | L | I | L | A | R | T | D | S | C | N | G | E | L | L | S | S | S | S | D | P | R | D | H | K | F | I | K | G | T | I | - | - | E | P | G | I | T | - | P | W | S | D | R | L | A | G | 307 | | Sbay\_661.12/1-568 | 255 | Q | W | D | I | M | G | T | E | N | L | V | I | A | R | T | D | S | C | N | G | K | L | L | S | S | S | S | D | P | R | D | H | E | F | I | K | G | V | V | - | - | K | G | N | V | V | - | P | W | S | E | K | L | I | E | 306 | | SAKL0B03212g/1-564 | 252 | Q | W | D | I | M | G | T | E | N | M | I | I | A | R | T | D | S | A | N | G | K | L | L | S | S | T | C | D | P | R | D | H | A | Y | I | K | G | I | I | - | - | N | P | K | T | V | - | C | W | T | E | A | L | A | E | 303 | | Q12031/1-575 | 262 | Q | W | D | I | M | G | T | E | N | L | V | I | A | R | T | D | S | C | N | G | K | L | L | S | S | S | S | D | P | R | D | H | E | F | I | R | G | I | I | - | - | R | D | N | V | V | - | P | W | S | E | K | L | I | E | 313 | |  | | G0V632/1-554 | 293 | M | E | T | T | N | - | - | - | - | - | - | T | E | R | S | I | I | Q | E | R | E | L | K | W | Y | N | N | N | Q | L | M | T | F | D | E | A | V | E | M | K | F | N | N | E | E | Y | K | Q | Y | L | T | T | K | K | H | 341 | | Q6CNB1/1-572 | 312 | L | E | F | D | N | - | - | - | - | - | - | A | P | S | T | K | I | S | M | A | E | A | N | W | Y | K | E | N | K | L | Y | T | F | E | E | A | A | K | E | Q | L | S | E | S | Q | F | Q | T | F | K | E | Q | W | N | K | 360 | | Q6FKR5/1-571 | 311 | L | E | R | K | E | - | - | - | - | - | - | V | D | K | S | I | I | A | E | A | E | K | E | W | Y | D | Q | N | K | L | Y | T | Y | E | E | M | I | Q | I | R | F | T | E | S | E | Y | K | D | Y | L | T | R | K | D | K | 359 | | Q750K8/1-557 | 296 | M | E | A | A | G | - | - | - | - | - | - | C | T | A | Q | E | I | A | A | A | E | Q | E | W | F | D | E | C | P | L | L | T | F | D | Q | A | A | E | Q | Q | L | S | S | S | E | Y | G | A | Y | G | A | R | K | K | E | 344 | | A7TQF1/1-561 | 295 | E | E | L | K | Q | E | K | Y | G | H | S | T | S | S | D | S | L | N | S | I | E | Q | E | W | Y | K | E | N | K | L | L | T | F | D | E | Y | M | I | Q | T | L | T | D | S | E | Y | T | N | L | I | K | E | R | S | E | 349 | | C5DXF8/1-567 | 308 | L | E | S | Q | P | - | - | - | - | - | M | V | T | S | D | Q | I | A | I | E | E | A | E | W | Y | D | K | H | H | V | F | T | F | D | E | A | L | Q | K | Q | V | S | Q | T | E | Y | E | R | Y | L | E | L | K | N | K | 357 | | Sbay\_661.12/1-568 | 307 | M | E | E | K | K | - | - | - | - | - | - | V | S | N | S | T | I | S | T | M | E | Q | E | W | Y | H | D | N | E | L | F | T | F | E | E | A | L | A | K | T | L | T | S | G | E | F | K | T | Y | K | A | K | K | E | D | 355 | | SAKL0B03212g/1-564 | 304 | L | E | A | S | G | - | - | - | - | - | - | S | N | N | Q | T | I | S | N | A | E | A | K | W | Y | N | E | N | E | L | F | T | F | D | E | A | V | E | R | Q | V | N | K | D | E | Y | E | E | Y | L | E | Q | K | G | K | 352 | | Q12031/1-575 | 314 | M | E | D | K | K | - | - | - | - | - | - | I | P | N | S | A | I | A | D | M | E | K | E | W | Y | H | E | N | E | L | F | T | F | E | E | A | L | E | K | Q | F | T | A | S | E | F | E | S | Y | K | E | K | K | E | D | 362 | |  | | G0V632/1-554 | 342 | M | M | D | K | E | L | K | R | P | Y | L | S | L | S | E | L | K | M | I | A | K | R | V | S | P | S | K | E | I | Y | F | N | W | D | I | P | R | T | K | E | G | Y | F | M | F | K | G | C | M | E | A | A | T | R | R | 396 | | Q6CNB1/1-572 | 361 | N | L | - | K | L | G | K | R | G | Y | L | S | I | D | E | L | K | S | L | V | R | S | V | N | G | G | S | D | I | V | F | N | W | D | A | P | R | T | K | E | G | F | Y | M | F | K | G | G | M | K | P | A | I | Q | R | 414 | | Q6FKR5/1-571 | 360 | Y | L | - | Q | T | Q | G | K | N | F | L | S | V | R | E | M | K | N | I | A | T | E | V | N | S | T | K | R | L | E | F | C | W | M | A | P | R | T | K | E | G | Y | Y | M | F | K | G | G | M | E | P | A | I | R | R | 413 | | Q750K8/1-557 | 345 | L | C | - | S | Q | L | G | R | P | Y | L | A | L | R | E | M | R | H | I | A | E | A | V | A | P | H | K | S | V | N | F | D | W | D | A | P | R | T | R | E | G | H | H | M | L | H | G | C | M | E | L | A | V | R | R | 398 | | A7TQF1/1-561 | 350 | L | L | - | K | K | L | G | R | R | Y | L | S | I | S | E | M | K | Q | L | A | N | K | I | S | P | R | K | N | L | T | F | N | W | Y | Y | P | R | T | K | E | G | H | F | M | F | N | G | C | L | E | A | A | I | E | R | 403 | | C5DXF8/1-567 | 358 | H | L | - | K | E | - | - | K | P | F | V | S | L | K | E | M | K | A | L | A | S | Q | A | S | P | A | T | T | I | E | F | D | W | D | A | P | R | T | K | E | G | Y | Y | L | Y | N | G | G | M | E | A | A | I | E | R | 409 | | Sbay\_661.12/1-568 | 356 | L | M | T | N | K | L | N | R | T | Y | L | S | L | R | E | M | K | L | L | A | Q | E | V | A | P | S | K | N | V | I | F | D | W | D | A | P | K | T | K | E | G | Y | Y | M | F | K | G | C | I | E | A | A | I | R | R | 410 | | SAKL0B03212g/1-564 | 353 | Y | M | - | R | K | L | G | R | E | Y | L | S | L | R | E | C | R | E | V | V | K | I | V | A | P | D | K | E | V | Y | F | D | W | D | A | P | R | T | K | E | G | Y | Y | M | L | K | N | C | I | E | A | A | I | Q | R | 406 | | Q12031/1-575 | 363 | L | M | V | N | K | L | G | R | A | Y | L | S | L | R | E | M | K | L | L | A | Q | E | V | T | P | L | K | K | I | I | F | D | W | D | A | P | R | T | K | E | G | Y | Y | M | F | N | G | C | I | E | A | A | I | R | R | 417 | |  | | G0V632/1-554 | 397 | S | L | V | F | A | P | Y | S | D | M | T | W | L | E | T | K | T | P | D | L | V | Q | A | K | D | F | A | R | N | I | H | D | V | Y | P | W | V | K | L | V | Y | N | L | S | P | S | F | N | W | T | Q | N | G | F | S | 451 | | Q6CNB1/1-572 | 415 | S | L | A | F | A | P | Y | A | D | L | L | W | L | E | T | K | S | P | D | L | K | Q | A | Q | A | F | S | K | E | I | H | D | V | Y | P | E | A | K | L | V | Y | N | L | S | P | S | F | N | W | S | A | H | G | F | D | 469 | | Q6FKR5/1-571 | 414 | S | L | V | F | A | P | Y | S | D | M | I | W | L | E | T | K | T | P | D | L | E | Q | A | K | S | F | S | K | E | I | H | N | T | Y | P | H | V | K | F | V | Y | N | L | S | P | S | F | N | W | T | A | H | G | F | N | 468 | | Q750K8/1-557 | 399 | T | L | A | F | A | P | Y | S | D | L | L | W | L | E | T | K | T | P | D | L | R | Q | A | S | A | F | A | A | A | I | H | R | A | Y | P | H | A | K | L | V | Y | N | L | S | P | S | F | N | W | T | A | H | G | Y | D | 453 | | A7TQF1/1-561 | 404 | S | L | L | F | S | Y | Y | S | D | L | I | W | L | E | T | K | T | P | N | L | L | Q | A | K | N | F | A | M | T | I | H | E | K | N | P | N | I | K | F | V | Y | N | L | S | P | S | F | N | W | E | A | Q | G | F | N | 458 | | C5DXF8/1-567 | 410 | S | L | Y | F | A | P | Y | A | D | M | I | W | L | E | T | K | T | P | D | L | K | Q | A | I | S | F | S | S | R | I | H | E | V | Y | P | H | V | K | L | V | Y | N | L | S | P | S | F | N | W | S | A | H | G | Y | T | 464 | | Sbay\_661.12/1-568 | 411 | S | L | V | F | A | P | Y | S | D | M | I | W | L | E | T | K | T | P | D | L | E | Q | A | R | S | F | S | S | K | I | H | K | L | F | P | A | T | K | L | V | Y | N | L | S | P | S | F | N | W | S | A | H | G | F | D | 465 | | SAKL0B03212g/1-564 | 407 | S | L | E | Y | A | P | Y | A | D | M | I | W | L | E | T | K | T | P | D | L | E | Q | A | R | N | F | S | R | K | I | H | R | V | Y | P | H | V | K | L | V | Y | N | L | S | P | S | F | N | W | S | A | Q | G | Y | S | 461 | | Q12031/1-575 | 418 | S | L | V | F | A | P | Y | S | D | M | I | W | L | E | T | K | T | P | D | L | E | Q | A | R | S | F | S | R | K | I | H | K | Q | L | P | A | T | K | L | V | Y | N | L | S | P | S | F | N | W | S | A | H | G | F | D | 472 | |  | | G0V632/1-554 | 452 | E | T | E | L | K | S | F | I | W | D | L | A | K | E | G | F | I | L | Q | L | V | S | L | A | G | L | H | V | N | A | L | S | F | W | E | L | A | Q | G | F | Q | S | G | G | M | D | A | Y | V | K | Q | I | Q | Q | R | 506 | | Q6CNB1/1-572 | 470 | D | E | Q | L | Q | S | F | I | W | D | L | A | K | E | G | F | V | L | Q | L | V | S | L | A | G | L | H | S | D | A | S | S | F | W | K | L | A | K | R | Y | Q | T | E | G | M | K | A | Y | V | E | E | V | Q | K | V | 524 | | Q6FKR5/1-571 | 469 | G | D | K | L | K | S | F | I | W | D | L | A | K | E | G | F | V | L | Q | L | V | S | L | A | G | L | H | T | N | A | A | S | F | W | D | L | A | K | N | F | Q | K | D | G | M | K | A | Y | V | D | K | V | Q | K | I | 523 | | Q750K8/1-557 | 454 | D | R | Q | L | R | A | F | I | P | S | L | A | A | A | G | F | V | L | Q | L | V | S | L | A | G | L | H | S | D | A | L | A | F | W | Q | L | A | A | A | F | P | A | D | G | M | L | A | Y | V | Q | H | V | Q | A | P | 508 | | A7TQF1/1-561 | 459 | Q | E | T | L | K | S | F | I | W | D | L | A | K | H | G | F | V | L | Q | L | V | S | L | A | G | L | H | A | N | G | L | G | T | W | K | L | A | K | E | F | Q | Y | D | G | M | K | A | Y | V | N | N | I | Q | N | V | 513 | | C5DXF8/1-567 | 465 | E | E | N | L | K | G | F | I | W | D | L | A | K | H | G | F | V | L | Q | L | V | S | L | A | G | L | H | V | N | G | L | S | F | W | Q | L | V | K | S | F | G | Q | N | G | M | K | A | Y | V | E | Q | V | Q | R | L | 519 | | Sbay\_661.12/1-568 | 466 | D | K | A | L | K | S | F | I | W | D | L | A | K | E | G | F | T | L | Q | L | V | S | L | A | G | L | H | S | D | G | V | S | F | W | E | L | A | N | S | F | Q | N | D | G | M | K | A | Y | V | E | Q | V | Q | R | R | 520 | | SAKL0B03212g/1-564 | 462 | K | E | D | L | Q | S | F | I | W | D | L | A | R | E | G | F | V | L | Q | L | V | S | L | A | G | L | H | V | D | A | V | S | F | W | Q | L | A | Q | K | F | Q | T | E | G | M | Q | A | Y | V | S | Q | I | Q | D | R | 516 | | Q12031/1-575 | 473 | D | K | A | L | K | S | F | V | W | D | L | A | K | E | G | F | T | L | Q | L | V | S | L | A | G | L | H | S | D | G | V | S | F | W | E | L | A | N | S | F | Q | S | D | G | M | K | A | Y | V | E | K | V | Q | K | R | 527 | |  | | G0V632/1-554 | 507 | E | K | E | T | D | C | D | V | L | T | H | Q | K | W | S | G | A | E | Y | I | D | S | V | L | Q | V | I | Q | N | G | S | S | S | H | T | S | T | T | S | G | E | S | Y | T | E | S | Q | F | - |  | | | | | | 554 | | Q6CNB1/1-572 | 525 | E | K | E | N | G | C | D | V | L | T | H | Q | Q | W | S | G | A | E | Y | V | D | S | I | M | Q | V | V | Q | N | G | S | S | S | Q | T | L | S | T | T | G | E | S | F | T | E | T | Q | F | - |  | | | | | | 572 | | Q6FKR5/1-571 | 524 | E | K | Q | L | D | C | D | V | L | T | H | Q | K | W | S | G | A | E | Y | M | D | S | L | L | N | V | V | Q | N | G | S | S | S | Q | T | M | S | T | S | G | E | S | F | T | E | N | Q | F | - |  | | | | | | 571 | | Q750K8/1-557 | 509 | E | R | R | S | G | C | D | V | L | L | H | Q | R | W | S | G | V | D | Y | V | D | S | L | G | N | L | V | Q | N | G | A | S | S | H | T | R | G | A | G | G | D | S | F | T | E | S | Q | F | Q |  | | | | | | 557 | | A7TQF1/1-561 | 514 | E | R | Q | T | N | S | D | M | L | T | H | Q | K | W | S | G | I | D | Y | I | E | S | I | S | N | V | I | Q | N | G | Q | S | S | M | T | S | S | T | S | G | S | G | F | T | E | K | H | F | - |  | | | | | | 561 | | C5DXF8/1-567 | 520 | E | K | L | E | N | S | D | V | L | T | H | Q | K | W | S | G | A | E | Y | V | D | S | I | I | K | V | L | Q | N | G | S | S | S | Q | T | L | S | T | S | G | D | S | F | T | E | N | Q | F | - |  | | | | | | 567 | | Sbay\_661.12/1-568 | 521 | E | K | K | S | G | C | D | V | L | T | H | Q | L | W | S | G | A | E | Y | V | D | S | L | M | K | V | V | Q | N | G | A | S | S | Q | T | L | S | T | S | G | E | S | F | T | E | T | Q | F | - |  | | | | | | 568 | | SAKL0B03212g/1-564 | 517 | E | R | D | S | Q | C | D | V | L | T | H | Q | R | W | S | G | A | E | Y | V | D | S | V | M | K | V | V | Q | N | G | S | S | S | Q | T | L | S | T | A | G | D | S | F | T | E | T | Q | F | - |  | | | | | | 564 | | Q12031/1-575 | 528 | E | K | E | T | N | C | D | I | M | T | H | Q | L | W | S | G | A | E | Y | V | D | S | L | M | K | V | V | Q | N | G | A | S | S | Q | T | L | S | T | S | G | E | S | F | T | E | T | Q | F | - |  | | | | | | 575 | |
