# Supplementary material for: Plus ça change – evolutionary sequence divergence predicts protein subcellular localization signals
Source: BMC Genomics. 2014 Jan 20;15:46. doi: 10.1186/1471-2164-15-46 (PMC3906766; doi:10.1186/1471-2164-15-46)
Supplement: Additional file 2 — MSA’s of proteins for which sequence divergence changes predicted localization signals. Contains links to ortholog multiple sequence alignments of each protein in Additional file 3: Table S1. [file 1471-2164-15-46-S2.zip › Q12428.html]

|  |  |  |  |  |  |  |  |  |  |  |  |  |  |  |  |  |  |  |  |  |  |  |  |  |  |  |  |  |  |  |  |  |  |  |  |  |  |  |  |  |  |  |  |  |  |  |  |  |  |  |  |  |  |  |  |  |  |  |  |  |  |  |  |  |  |  |  |  |  |  |  |  |  |  |  |  |  |  |  |  |  |  |  |  |  |  |  |  |  |  |  |  |  |  |  |  |  |  |  |  |  |  |  |  |  |  |  |  |  |  |  |  |  |  |  |  |  |  |  |  |  |  |  |  |  |  |  |  |  |  |  |  |  |  |  |  |  |  |  |  |  |  |  |  |  |  |  |  |  |  |  |  |  |  |  |  |  |  |  |  |  |  |  |  |  |  |  |  |  |  |  |  |  |  |  |  |  |  |  |  |  |  |  |  |  |  |  |  |  |  |  |  |  |  |  |  |  |  |  |  |  |  |  |  |  |  |  |  |  |  |  |  |  |  |  |  |  |  |  |  |  |  |  |  |  |  |  |  |  |  |  |  |  |  |  |  |  |  |  |  |  |  |  |  |  |  |  |  |  |  |  |  |  |  |  |  |  |  |  |  |  |  |  |  |  |  |  |  |  |  |  |  |  |  |  |  |  |  |  |  |  |  |  |  |  |  |  |  |  |  |  |  |  |  |  |  |  |  |  |  |  |  |  |  |  |  |  |  |  |  |  |  |  |  |  |  |  |  |  |  |  |  |  |  |  |  |  |  |  |  |  |  |  |  |  |  |  |  |  |  |  |  |  |  |  |  |  |  |  |  |  |  |  |  |  |  |  |  |  |  |  |  |  |  |  |  |  |  |  |  |  |  |  |  |  |  |  |  |  |  |  |  |  |  |  |  |  |  |  |  |  |  |  |  |  |  |  |  |  |  |  |  |  |  |  |  |  |  |  |  |  |  |  |  |  |  |  |  |  |  |  |  |  |  |  |  |  |  |  |  |  |  |  |  |  |  |  |  |  |  |  |  |  |  |  |  |  |  |  |  |  |  |  |  |  |  |  |  |  |  |  |  |  |  |  |  |  |  |  |  |  |  |  |  |  |  |  |  |  |  |  |  |  |  |  |  |  |  |  |  |  |  |  |  |  |  |  |  |  |  |  |  |  |  |  |  |  |  |  |  |  |  |  |  |  |  |  |  |  |  |  |  |  |  |  |  |  |  |  |  |  |  |  |  |  |  |  |  |  |  |  |  |  |  |  |  |  |  |  |  |  |  |  |  |  |  |  |  |  |  |  |  |  |  |  |  |  |  |  |  |  |  |  |  |  |  |  |  |  |  |  |  |  |  |  |  |  |  |  |  |  |  |  |  |  |  |  |  |  |  |  |  |  |  |  |  |  |  |  |  |  |  |  |  |  |  |  |  |  |  |  |  |  |  |  |  |  |  |  |  |  |  |  |  |  |  |  |  |  |  |  |  |  |  |  |  |  |  |  |  |  |  |  |  |  |  |  |  |  |  |  |  |  |  |  |  |  |  |  |  |  |  |  |  |  |  |  |  |  |  |  |  |  |  |  |  |  |  |  |  |  |  |  |  |  |  |  |  |  |  |  |  |  |  |  |  |  |  |  |  |  |  |  |  |  |  |  |  |  |  |  |  |  |  |  |  |  |  |  |  |  |  |  |  |  |  |  |  |  |  |  |  |  |  |  |  |  |  |  |  |  |  |  |  |  |  |  |  |  |  |  |  |  |  |  |  |  |  |  |  |  |  |  |  |  |  |  |  |  |  |  |  |  |  |  |  |  |  |  |  |  |  |  |  |  |  |  |  |  |  |  |  |  |  |  |  |  |  |  |  |  |  |  |  |  |  |  |  |  |  |  |  |  |  |  |  |  |  |  |  |  |  |  |  |  |  |  |  |  |  |  |  |  |  |  |  |  |  |  |  |  |  |  |  |  |  |  |  |  |  |  |  |  |  |  |  |  |  |  |  |  |  |  |  |  |  |  |  |  |  |  |  |  |  |  |  |  |  |  |  |  |  |  |  |  |  |  |  |  |  |  |  |  |  |  |  |  |  |  |  |  |  |  |  |  |  |  |  |  |  |  |  |  |  |  |  |  |  |  |  |  |  |  |  |  |  |  |  |  |  |  |  |  |  |  |  |  |  |  |  |  |  |  |  |  |  |  |  |  |  |  |  |  |  |  |  |  |  |  |  |  |  |  |  |  |  |  |  |  |  |  |  |  |  |  |  |  |  |  |  |  |  |  |  |  |  |  |  |  |  |  |  |  |  |  |  |  |  |  |  |  |  |  |  |  |  |  |  |  |  |  |  |  |  |  |  |  |  |  |  |  |  |  |  |  |  |  |  |  |  |  |  |  |  |  |  |  |  |  |  |  |  |  |  |  |  |  |  |  |  |  |  |  |  |  |  |  |  |  |  |  |  |  |  |  |  |  |  |  |  |  |  |  |  |  |  |  |  |  |  |  |  |  |  |  |  |  |  |  |  |  |  |  |  |  |  |  |  |  |  |  |  |  |  |  |  |  |  |  |  |  |  |  |  |  |  |  |  |  |  |  |  |  |  |  |  |  |  |  |  |  |  |  |  |  |  |  |  |  |  |  |  |  |  |  |  |  |  |  |  |  |  |  |  |  |  |  |  |  |  |  |  |  |  |  |  |  |  |  |  |  |  |  |  |  |  |  |  |  |  |  |  |  |  |  |  |  |  |  |  |  |  |  |  |  |  |  |  |  |  |  |  |  |  |  |  |  |  |  |  |  |  |  |  |  |  |  |  |  |  |  |  |  |  |  |  |  |  |  |  |  |  |  |  |  |  |  |  |  |  |  |  |  |  |  |  |  |  |  |  |  |  |  |  |  |  |  |  |  |  |  |  |  |  |  |  |  |  |  |  |  |  |  |  |  |  |  |  |  |  |  |  |  |  |  |  |  |  |  |  |  |  |  |  |  |  |  |  |  |  |  |  |  |  |  |  |  |  |  |  |  |  |  |  |  |  |  |  |  |  |  |  |  |  |  |  |  |  |  |  |  |  |  |  |  |  |  |  |  |  |  |  |  |  |  |  |  |  |  |  |  |  |  |  |  |  |  |  |  |  |  |  |  |  |  |  |  |  |  |  |  |  |  |  |  |  |  |  |  |  |  |  |  |  |  |  |  |  |  |  |  |  |  |  |  |  |  |  |  |  |  |  |  |  |  |  |  |  |  |  |  |  |  |  |  |  |  |  |  |  |  |  |  |  |  |  |  |  |  |  |  |  |  |  |  |  |  |  |  |  |  |  |  |  |  |  |  |  |  |  |  |  |  |  |  |  |  |  |  |  |  |  |  |  |  |  |  |  |  |  |  |  |  |  |  |  |  |  |  |  |  |  |  |  |  |  |  |  |  |  |  |  |  |  |  |  |  |  |  |  |  |  |  |  |  |  |  |  |  |  |  |  |  |  |  |  |  |  |  |  |  |  |  |  |  |  |  |  |  |  |  |  |  |  |  |  |  |  |  |  |  |  |  |  |  |  |  |  |  |  |  |  |  |  |  |  |  |  |  |  |  |  |  |  |  |  |  |  |  |  |  |  |  |  |  |  |  |  |  |  |  |  |  |  |  |  |  |  |  |  |  |  |  |  |  |  |  |  |  |  |  |  |  |  |  |  |  |  |  |  |  |  |  |  |  |  |  |  |  |  |  |  |  |  |  |  |  |  |  |  |  |  |  |  |  |  |  |  |  |  |  |  |  |  |  |  |  |  |  |  |  |  |  |  |  |  |  |  |  |  |  |  |  |  |  |  |  |  |  |  |  |  |  |  |  |  |  |  |  |  |  |  |  |  |  |  |  |  |  |  |  |  |  |  |  |  |  |  |  |  |  |  |  |  |  |  |  |  |  |  |  |  |  |  |  |  |  |  |  |  |  |  |  |  |  |  |  |  |  |  |  |  |  |  |  |  |  |  |  |  |  |  |  |  |  |  |  |  |  |  |  |  |  |  |  |  |  |  |  |  |  |  |  |  |  |  |  |  |  |  |  |  |  |  |  |  |  |  |  |  |  |  |  |  |  |  |  |  |  |  |  |  |  |  |  |  |  |  |  |  |  |  |  |  |  |  |  |  |  |  |  |  |  |  |  |  |  |  |  |  |  |  |  |  |  |  |  |  |  |  |  |  |  |  |  |  |  |  |  |  |  |  |  |  |  |  |  |  |  |  |  |  |  |  |  |  |  |  |  |  |  |  |  |  |  |  |  |  |  |  |  |  |  |  |  |  |  |  |  |  |  |  |  |  |  |  |  |  |  |  |  |  |  |  |  |  |  |  |  |  |  |  |  |  |  |  |  |  |  |  |  |  |  |  |  |  |  |  |  |  |  |  |  |  |  |  |  |  |  |  |  |  |  |  |  |  |  |  |  |  |  |  |  |  |  |  |  |  |  |  |  |  |  |  |  |  |  |  |  |  |  |  |  |  |  |  |  |  |  |  |  |  |  |  |  |  |  |  |  |  |  |  |  |  |  |  |  |  |  |  |  |  |  |  |  |  |  |  |  |  |  |  |  |  |  |  |  |  |  |  |  |  |  |  |  |  |  |  |  |  |  |  |  |  |  |  |  |  |  |  |  |  |  |  |  |  |  |  |  |  |  |  |  |  |  |  |  |  |  |  |  |  |  |  |  |  |  |  |  |  |  |  |  |  |  |  |  |  |  |  |  |  |  |  |  |  |  |  |  |  |  |  |  |  |  |  |  |  |  |  |  |  |  |  |  |  |  |  |  |  |  |  |  |  |  |  |  |  |  |  |  |  |  |  |  |  |  |  |  |  |  |  |  |  |  |  |  |  |  |  |  |  |  |  |  |  |  |  |  |  |  |  |  |  |  |  |  |  |  |  |  |  |  |  |  |  |  |  |  |  |  |  |  |  |  |  |  |  |  |  |  |  |  |  |  |  |  |  |  |  |  |  |  |  |  |  |  |  |  |  |  |  |  |  |  |  |  |  |  |  |  |  |  |  |  |  |  |  |  |  |  |  |  |  |  |  |  |  |  |  |  |  |  |  |  |  |  |  |  |  |  |  |  |  |  |  |  |  |  |  |  |  |  |  |  |  |  |  |  |  |  |  |  |  |  |  |  |  |  |  |  |  |  |  |  |  |  |  |  |  |  |  |  |  |  |  |  |  |  |  |  |  |  |  |  |  |  |  |  |  |  |  |  |  |  |  |  |  |  |  |  |  |  |  |  |  |  |  |  |  |  |  |  |  |  |  |  |  |  |  |  |  |  |  |  |  |  |  |  |  |  |  |  |  |  |  |  |  |  |  |  |  |  |  |  |  |  |  |  |  |  |  |  |  |  |  |  |  |  |  |  |  |  |  |  |  |  |  |  |  |  |  |  |  |  |  |  |  |  |  |  |  |  |  |  |  |  |  |  |  |  |  |  |  |  |  |  |  |  |  |  |  |  |  |  |  |  |  |  |  |  |  |  |  |  |  |  |  |  |  |  |  |  |  |  |  |  |  |  |  |  |  |  |  |  |  |  |  |  |  |  |  |  |  |  |  |  |  |  |  |  |  |  |  |  |  |  |  |  |  |  |  |  |  |  |  |  |  |  |  |  |  |  |  |  |  |  |  |  |  |  |  |  |  |  |  |  |  |  |  |  |  |  |  |  |  |  |  |  |  |  |  |  |  |  |  |  |  |  |  |  |  |  |  |  |  |  |  |  |  |  |  |  |  |  |  |  |  |  |  |  |  |  |  |  |  |  |  |  |  |  |  |  |  |  |  |  |  |  |  |  |  |  |  |  |  |  |  |  |  |  |  |  |  |  |  |  |  |  |  |  |  |  |  |  |  |  |  |  |  |  |  |  |  |  |  |  |  |  |  |  |  |  |  |  |  |  |  |  |  |  |  |  |  |  |  |  |  |  |  |  |  |  |  |  |  |  |  |  |  |  |  |  |  |  |  |  |  |  |  |  |  |  |  |  |  |  |  |  |  |  |  |  |  |  |  |  |  |  |  |  |  |  |  |  |  |  |  |  |  |  |  |  |  |  |  |  |  |  |  |  |  |  |  |  |  |  |  |  |  |  |  |  |  |  |  |  |  |  |  |  |  |  |  |  |  |  |  |  |  |  |  |  |  |  |  |  |  |  |  |  |  |  |  |  |  |  |  |  |  |  |  |  |  |  |  |  |  |  |  |  |  |  |  |  |  |  |  |  |  |  |  |  |  |  |  |  |  |  |  |  |  |  |  |  |  |  |  |  |  |  |  |  |  |  |  |  |  |  |  |  |  |  |  |  |  |  |  |  |  |  |  |  |  |  |  |  |  |  |  |  |  |  |  |  |  |  |  |  |  |  |  |  |  |  |  |  |  |  |  |  |  |  |  |  |  |  |  |  |  |  |  |  |  |  |  |  |  |  |  |  |  |  |  |  |  |  |  |  |  |  |  |  |  |  |  |  |  |  |  |  |  |  |  |  |  |  |  |  |  |  |  |  |  |  |  |  |  |  |  |  |  |  |  |  |  |  |  |  |  |  |  |  |  |  |  |  |  |  |  |  |  |  |  |  |  |  |  |  |  |  |  |  |  |  |  |  |  |  |  |  |  |  |  |  |  |  |  |  |  |  |  |  |  |  |  |  |  |  |  |  |  |  |  |  |  |  |  |  |  |  |  |  |  |  |  |  |  |  |  |  |  |  |  |  |  |  |  |  |  |  |  |  |  |  |  |  |  |  |  |  |  |  |  |  |  |  |  |  |  |  |  |  |  |  |  |  |  |  |  |  |  |  |  |  |  |  |  |  |  |  |  |  |  |  |  |  |  |  |  |  |  |  |  |  |  |  |  |  |  |  |  |  |  |  |  |  |  |  |  |  |  |  |  |  |  |  |  |  |  |  |  |  |  |  |  |  |  |  |  |  |  |  |  |  |  |  |  |  |  |  |  |  |  |  |  |  |  |  |  |  |  |  |  |  |  |  |  |  |  |  |  |  |  |  |  |  |  |  |  |  |  |  |  |  |  |  |  |  |  |  |  |  |  |  |  |  |  |  |  |  |  |  |  |  |  |  |  |  |  |  |  |  |  |  |  |  |  |  |  |  |  |  |  |  |  |  |  |  |  |  |  |  |  |  |  |  |  |  |  |  |  |  |  |  |  |  |  |  |  |  |  |  |  |  |  |  |  |  |  |  |  |  |  |  |  |  |  |  |  |  |  |  |  |  |  |  |  |  |  |  |  |  |  |  |  |  |  |  |  |  |  |  |  |  |  |  |  |  |  |  |  |  |  |  |  |  |  |  |  |  |  |  |  |  |  |  |  |  |  |  |  |  |  |  |  |  |  |  |  |  |  |  |  |  |  |  |  |  |  |  |  |  |  |  |  |  |  |  |  |  |  |  |  |  |  |  |  |  |  |  |  |  |  |  |  |  |  |  |  |  |  |  |  |  |  |  |  |  |  |  |  |  |  |  |  |  |  |  |  |  |  |  |  |  |  |  |  |  |  |  |  |  |  |  |  |  |  |  |  |  |  |  |  |  |  |  |  |  |  |  |  |  |  |  |  |  |  |  |  |  |  |  |  |  |  |  |  |  |  |  |  |  |  |  |  |  |  |  |  |  |  |  |  |  |  |  |  |  |  |  |  |  |  |  |  |  |  |  |  |  |  |  |  |  |  |  |  |  |  |  |  |  |  |  |  |  |  |  |  |  |  |  |  |  |  |  |  |  |  |  |  |  |  |  |  |  |  |  |  |  |  |  |  |  |  |  |  |  |  |  |  |  |  |  |  |  |  |  |  |  |  |  |  |  |  |  |  |  |  |  |  |  |  |  |  |  |  |  |  |  |  |  |  |  |  |  |  |  |  |  |  |  |  |  |  |  |  |  |  |  |  |  |  |  |  |  |  |  |  |  |  |  |  |  |  |  |  |  |  |  |  |  |  |  |  |  |  |  |  |  |  |  |  |  |  |  |  |  |  |  |  |  |  |  |  |  |  |  |  |  |  |  |  |  |  |  |  |  |  |  |  |  |  |  |  |  |  |  |  |  |  |  |  |  |  |  |  |  |  |  |  |  |  |  |  |  |  |  |  |  |  |  |  |  |  |  |  |  |  |  |  |  |  |  |  |  |  |  |  |  |  |  |  |  |  |  |  |  |  |  |  |  |  |  |  |  |  |  |  |  |  |  |  |  |  |  |  |  |  |  |  |  |  |  |  |  |  |  |  |  |  |  |  |  |  |  |  |  |  |  |  |  |  |  |  |  |  |  |  |  |  |  |  |  |  |  |  |  |  |  |  |  |  |  |  |  |  |  |  |  |  |  |  |  |  |  |  |  |  |  |  |  |  |  |  |  |  |  |  |  |  |  |  |  |  |  |  |  |  |  |  |  |  |  |  |  |  |  |  |  |  |  |  |  |  |  |  |  |  |  |  |  |  |  |  |  |  |  |  |  |  |  |  |  |  |  |  |  |  |  |  |  |  |  |  |  |  |  |  |  |  |  |  |  |  |  |  |  |  |  |  |  |  |  |  |  |  |  |  |  |  |  |  |  |  |  |  |  |  |  |  |  |  |  |  |  |  |  |  |  |  |  |  |  |  |  |  |  |  |  |  |  |  |  |  |  |  |  |  |  |  |  |  |  |  |  |  |  |  |  |  |  |  |  |  |  |  |  |  |  |  |  |  |  |  |  |  |  |  |  |  |  |  |  |  |  |  |  |  |  |  |  |  |  |  |  |  |  |  |  |  |  |  |  |  |  |  |  |  |  |  |  |  |  |  |  |  |  |  |  |  |  |  |  |  |  |  |  |  |  |  |  |  |  |  |  |  |  |  |  |  |  |  |  |  |  |  |  |  |  |  |  |  |  |  |  |  |  |  |  |  |  |  |  |  |  |  |  |  |  |  |  |  |  |  |  |  |  |  |  |  |  |  |  |  |  |  |  |  |  |  |  |  |  |  |  |  |  |  |  |  |  |  |  |  |  |  |  |  |  |  |  |  |  |  |  |  |  |  |  |  |  |  |  |  |  |  |  |  |  |  |  |  |  |  |  |  |  |  |  |  |  |  |  |  |  |  |  |  |  |  |  |  |  |  |  |  |  |  |  |  |  |  |  |  |  |  |  |  |  |  |  |  |  |  |  |  |  |  |  |  |  |  |  |  |  |  |  |  |  |  |  |  |  |  |  |  |  |  |  |  |  |  |  |  |  |  |  |  |  |  |  |  |  |  |  |  |  |  |  |  |  |  |  |  |  |  |  |  |  |  |  |  |  |  |  |  |  |  |  |  |  |  |  |  |  |  |  |  |  |  |  |  |  |  |  |  |  |  |  |  |  |  |  |  |  |  |  |  |  |  |  |  |  |  |  |  |  |  |  |  |  |  |  |  |  |  |  |  |  |  |  |  |  |  |  |  |  |  |  |  |  |  |  |  |  |  |  |  |  |  |  |  |  |  |  |  |  |  |  |  |  |  |  |  |  |  |  |  |  |  |  |  |  |  |  |  |  |  |  |  |  |  |  |  |  |  |  |  |  |  |  |  |  |  |  |  |  |  |  |  |  |  |  |  |  |  |  |  |  |  |  |  |  |  |  |  |  |  |  |  |  |  |  |  |  |  |  |  |  |  |  |  |  |  |  |  |  |  |  |  |  |  |  |  |  |  |  |  |  |  |  |  |  |  |  |  |  |  |  |  |  |  |  |  |  |  |  |  |  |  |  |  |  |  |  |  |  |  |  |  |  |  |  |  |  |  |  |  |  |  |  |  |  |  |  |  |  |  |  |  |  |  |  |  |  |  |  |  |  |  |  |  |  |  |  |  |  |  |  |  |  |  |  |  |  |  |  |  |  |  |  |  |  |  |  |  |  |  |  |  |  |  |  |  |  |  |  |  |  |  |  |  |  |  |  |  |  |  |  |  |  |  |  |  |  |  |  |  |  |  |  |  |  |  |  |  |  |  |  |  |  |  |  |  |  |  |  |  |  |  |  |  |  |  |  |  |  |  |  |  |  |  |  |  |  |  |  |  |  |  |  |  |  |  |  |  |  |  |  |  |  |  |  |  |  |  |  |  |  |  |  |  |  |  |  |  |  |  |  |  |  |  |  |  |  |  |  |  |  |  |  |  |  |  |  |  |  |  |  |  |  |  |  |  |  |  |  |  |  |  |  |  |  |  |  |  |  |  |  |  |  |  |  |  |  |  |  |  |  |  |  |  |  |  |  |  |  |  |  |  |  |  |  |  |  |  |  |  |  |  |  |  |  |  |  |  |  |  |  |  |  |  |  |  |  |  |  |  |  |  |  |  |  |  |  |  |  |  |  |  |  |  |  |  |  |  |  |  |  |  |  |  |  |  |  |  |  |  |  |  |  |  |  |  |  |  |  |  |  |  |  |  |  |  |  |  |  |  |  |  |  |  |  |  |  |  |  |  |  |  |  |  |  |  |  |  |  |  |  |  |  |  |  |  |  |  |  |  |  |  |  |  |  |  |  |  |  |  |  |  |  |  |  |  |  |  |  |  |  |  |  |  |  |  |  |  |  |  |  |  |  |  |  |  |  |  |  |  |  |  |  |  |  |  |  |  |  |  |  |  |  |  |  |  |  |  |  |  |  |  |  |  |  |  |  |  |  |  |  |  |  |  |  |  |  |  |  |  |  |  |  |  |  |  |  |  |  |  |  |  |  |  |  |  |  |  |  |  |  |  |  |  |  |  |  |  |  |  |  |  |  |  |  |  |  |  |  |  |  |  |  |  |  |  |  |  |  |  |  |  |  |  |  |  |  |  |  |  |  |  |  |  |  |  |  |  |  |  |  |  |  |  |  |  |  |  |  |  |  |  |  |  |  |  |  |  |  |  |  |  |  |  |  |  |  |  |  |  |  |  |  |  |  |  |  |  |  |  |  |  |  |  |  |  |  |  |  |  |  |  |  |  |  |  |  |  |  |  |  |  |  |  |  |  |  |  |  |  |  |  |  |  |  |  |  |  |  |  |  |  |  |  |  |  |  |  |  |  |  |  |  |  |  |  |  |  |  |  |  |  |  |  |  |  |  |  |  |  |  |  |  |  |  |  |  |  |  |  |  |  |  |  |  |  |  |  |  |  |  |  |  |  |  |  |  |  |  |  |  |  |  |  |  |  |  |  |  |  |  |  |  |  |  |  |  |  |  |  |  |  |  |  |  |  |  |  |  |  |  |  |  |  |  |  |  |  |  |  |  |  |  |  |  |  |  |  |  |  |  |  |  |  |  |  |  |  |  |  |  |  |  |  |  |  |  |  |  |  |  |  |  |  |  |  |  |  |  |  |  |  |  |  |  |  |  |  |  |  |  |  |  |  |  |  |  |  |  |  |  |  |  |  |  |  |  |  |  |  |  |  |  |  |  |  |  |  |  |  |  |  |  |  |  |  |  |  |  |  |  |  |  |  |  |  |  |  |  |  |  |  |  |  |  |  |  |  |  |  |  |  |  |  |  |  |  |  |  |  |  |  |  |  |  |  |  |  |  |  |  |  |  |  |  |  |  |  |  |  |  |  |  |  |  |  |  |  |  |  |  |  |  |  |  |  |  |  |  |  |  |  |  |  |  |  |  |  |  |  |  |  |  |  |  |  |  |  |  |  |  |  |  |  |  |  |  |  |  |  |  |  |  |  |  |  |  |  |  |  |  |  |  |  |  |  |  |  |  |  |  |  |  |  |  |  |  |  |  |  |  |  |  |  |  |  |  |  |  |  |  |  |  |  |  |  |  |  |  |  |  |  |  |  |  |  |  |  |  |  |  |  |  |  |  |  |  |  |  |
| --- | --- | --- | --- | --- | --- | --- | --- | --- | --- | --- | --- | --- | --- | --- | --- | --- | --- | --- | --- | --- | --- | --- | --- | --- | --- | --- | --- | --- | --- | --- | --- | --- | --- | --- | --- | --- | --- | --- | --- | --- | --- | --- | --- | --- | --- | --- | --- | --- | --- | --- | --- | --- | --- | --- | --- | --- | --- | --- | --- | --- | --- | --- | --- | --- | --- | --- | --- | --- | --- | --- | --- | --- | --- | --- | --- | --- | --- | --- | --- | --- | --- | --- | --- | --- | --- | --- | --- | --- | --- | --- | --- | --- | --- | --- | --- | --- | --- | --- | --- | --- | --- | --- | --- | --- | --- | --- | --- | --- | --- | --- | --- | --- | --- | --- | --- | --- | --- | --- | --- | --- | --- | --- | --- | --- | --- | --- | --- | --- | --- | --- | --- | --- | --- | --- | --- | --- | --- | --- | --- | --- | --- | --- | --- | --- | --- | --- | --- | --- | --- | --- | --- | --- | --- | --- | --- | --- | --- | --- | --- | --- | --- | --- | --- | --- | --- | --- | --- | --- | --- | --- | --- | --- | --- | --- | --- | --- | --- | --- | --- | --- | --- | --- | --- | --- | --- | --- | --- | --- | --- | --- | --- | --- | --- | --- | --- | --- | --- | --- | --- | --- | --- | --- | --- | --- | --- | --- | --- | --- | --- | --- | --- | --- | --- | --- | --- | --- | --- | --- | --- | --- | --- | --- | --- | --- | --- | --- | --- | --- | --- | --- | --- | --- | --- | --- | --- | --- | --- | --- | --- | --- | --- | --- | --- | --- | --- | --- | --- | --- | --- | --- | --- | --- | --- | --- | --- | --- | --- | --- | --- | --- | --- | --- | --- | --- | --- | --- | --- | --- | --- | --- | --- | --- | --- | --- | --- | --- | --- | --- | --- | --- | --- | --- | --- | --- | --- | --- | --- | --- | --- | --- | --- | --- | --- | --- | --- | --- | --- | --- | --- | --- | --- | --- | --- | --- | --- | --- | --- | --- | --- | --- | --- | --- | --- | --- | --- | --- | --- | --- | --- | --- | --- | --- | --- | --- | --- | --- | --- | --- | --- | --- | --- | --- | --- | --- | --- | --- | --- | --- | --- | --- | --- | --- | --- | --- | --- | --- | --- | --- | --- | --- | --- | --- | --- | --- | --- | --- | --- | --- | --- | --- | --- | --- | --- | --- | --- | --- | --- | --- | --- | --- | --- | --- | --- | --- | --- | --- | --- | --- | --- | --- | --- | --- | --- | --- | --- | --- | --- | --- | --- | --- | --- | --- | --- | --- | --- | --- | --- | --- | --- | --- | --- | --- | --- | --- | --- | --- | --- | --- | --- | --- | --- | --- | --- | --- | --- | --- | --- | --- | --- | --- | --- | --- | --- | --- | --- | --- | --- | --- | --- | --- | --- | --- | --- | --- | --- | --- | --- | --- | --- | --- | --- | --- | --- | --- | --- | --- | --- | --- | --- | --- | --- | --- | --- | --- | --- | --- | --- | --- | --- | --- | --- | --- | --- | --- | --- | --- | --- | --- | --- | --- | --- | --- | --- | --- | --- | --- | --- | --- | --- | --- | --- | --- | --- | --- | --- | --- | --- | --- | --- | --- | --- | --- | --- | --- | --- | --- | --- | --- | --- | --- | --- | --- | --- | --- | --- | --- | --- | --- | --- | --- | --- | --- | --- | --- | --- | --- | --- | --- | --- | --- | --- | --- | --- | --- | --- | --- | --- | --- | --- | --- | --- | --- | --- | --- | --- | --- | --- | --- | --- | --- | --- | --- | --- | --- | --- | --- | --- | --- | --- | --- | --- | --- | --- | --- | --- | --- | --- | --- | --- | --- | --- | --- | --- | --- | --- | --- | --- | --- | --- | --- | --- | --- | --- | --- | --- | --- | --- | --- | --- | --- | --- | --- | --- | --- | --- | --- | --- | --- | --- | --- | --- | --- | --- | --- | --- | --- | --- | --- | --- | --- | --- | --- | --- | --- | --- | --- | --- | --- | --- | --- | --- | --- | --- | --- | --- | --- | --- | --- | --- | --- | --- | --- | --- | --- | --- | --- | --- | --- | --- | --- | --- | --- | --- | --- | --- | --- | --- | --- | --- | --- | --- | --- | --- | --- | --- | --- | --- | --- | --- | --- | --- | --- | --- | --- | --- | --- | --- | --- | --- | --- | --- | --- | --- | --- | --- | --- | --- | --- | --- | --- | --- | --- | --- | --- | --- | --- | --- | --- | --- | --- | --- | --- | --- | --- | --- | --- | --- | --- | --- | --- | --- | --- | --- | --- | --- | --- | --- | --- | --- | --- | --- | --- | --- | --- | --- | --- | --- | --- | --- | --- | --- | --- | --- | --- | --- | --- | --- | --- | --- | --- | --- | --- | --- | --- | --- | --- | --- | --- | --- | --- | --- | --- | --- | --- | --- | --- | --- | --- | --- | --- | --- | --- | --- | --- | --- | --- | --- | --- | --- | --- | --- | --- | --- | --- | --- | --- | --- | --- | --- | --- | --- | --- | --- | --- | --- | --- | --- | --- | --- | --- | --- | --- | --- | --- | --- | --- | --- | --- | --- | --- | --- | --- | --- | --- | --- | --- | --- | --- | --- | --- | --- | --- | --- | --- | --- | --- | --- | --- | --- | --- | --- | --- | --- | --- | --- | --- | --- | --- | --- | --- | --- | --- | --- | --- | --- | --- | --- | --- | --- | --- | --- | --- | --- | --- | --- | --- | --- | --- | --- | --- | --- | --- | --- | --- | --- | --- | --- | --- | --- | --- | --- | --- | --- | --- | --- | --- | --- | --- | --- | --- | --- | --- | --- | --- | --- | --- | --- | --- | --- | --- | --- | --- | --- | --- | --- | --- | --- | --- | --- | --- | --- | --- | --- | --- | --- | --- | --- | --- | --- | --- | --- | --- | --- | --- | --- | --- | --- | --- | --- | --- | --- | --- | --- | --- | --- | --- | --- | --- | --- | --- | --- | --- | --- | --- | --- | --- | --- | --- | --- | --- | --- | --- | --- | --- | --- | --- | --- | --- | --- | --- | --- | --- | --- | --- | --- | --- | --- | --- | --- | --- | --- | --- | --- | --- | --- | --- | --- | --- | --- | --- | --- | --- | --- | --- | --- | --- | --- | --- | --- | --- | --- | --- | --- | --- | --- | --- | --- | --- | --- | --- | --- | --- | --- | --- | --- | --- | --- | --- | --- | --- | --- | --- | --- | --- | --- | --- | --- | --- | --- | --- | --- | --- | --- | --- | --- | --- | --- | --- | --- | --- | --- | --- | --- | --- | --- | --- | --- | --- | --- | --- | --- | --- | --- | --- | --- | --- | --- | --- | --- | --- | --- | --- | --- | --- | --- | --- | --- | --- | --- | --- | --- | --- | --- | --- | --- | --- | --- | --- | --- | --- | --- | --- | --- | --- | --- | --- | --- | --- | --- | --- | --- | --- | --- | --- | --- | --- | --- | --- | --- | --- | --- | --- | --- | --- | --- | --- | --- | --- | --- | --- | --- | --- | --- | --- | --- | --- | --- | --- | --- | --- | --- | --- | --- | --- | --- | --- | --- | --- | --- | --- | --- | --- | --- | --- | --- | --- | --- | --- | --- | --- | --- | --- | --- | --- | --- | --- | --- | --- | --- | --- | --- | --- | --- | --- | --- | --- | --- | --- | --- | --- | --- | --- | --- | --- | --- | --- | --- | --- | --- | --- | --- | --- | --- | --- | --- | --- | --- | --- | --- | --- | --- | --- | --- | --- | --- | --- | --- | --- | --- | --- | --- | --- | --- | --- | --- | --- | --- | --- | --- | --- | --- | --- | --- | --- | --- | --- | --- | --- | --- | --- | --- | --- | --- | --- | --- | --- | --- | --- | --- | --- | --- | --- | --- | --- | --- | --- | --- | --- | --- | --- | --- | --- | --- | --- | --- | --- | --- | --- | --- | --- | --- | --- | --- | --- | --- | --- | --- | --- | --- | --- | --- | --- | --- | --- | --- | --- | --- | --- | --- | --- | --- | --- | --- | --- | --- | --- | --- | --- | --- | --- | --- | --- | --- | --- | --- | --- | --- | --- | --- | --- | --- | --- | --- | --- | --- | --- | --- | --- | --- | --- | --- | --- | --- | --- | --- | --- | --- | --- | --- | --- | --- | --- | --- | --- | --- | --- | --- | --- | --- | --- | --- | --- | --- | --- | --- | --- | --- | --- | --- | --- | --- | --- | --- | --- | --- | --- | --- | --- | --- | --- | --- | --- | --- | --- | --- | --- | --- | --- | --- | --- | --- | --- | --- | --- | --- | --- | --- | --- | --- | --- | --- | --- | --- | --- | --- | --- | --- | --- | --- | --- | --- | --- | --- | --- | --- | --- | --- | --- | --- | --- | --- | --- | --- | --- | --- | --- | --- | --- | --- | --- | --- | --- | --- | --- | --- | --- | --- | --- | --- | --- | --- | --- | --- | --- | --- | --- | --- | --- | --- | --- | --- | --- | --- | --- | --- | --- | --- | --- | --- | --- | --- | --- | --- | --- | --- | --- | --- | --- | --- | --- | --- | --- | --- | --- | --- | --- | --- | --- | --- | --- | --- | --- | --- | --- | --- | --- | --- | --- | --- | --- | --- | --- | --- | --- | --- | --- | --- | --- | --- | --- | --- | --- | --- | --- | --- | --- | --- | --- | --- | --- | --- | --- | --- | --- | --- | --- | --- | --- | --- | --- | --- | --- | --- | --- | --- | --- | --- | --- | --- | --- | --- | --- | --- | --- | --- | --- | --- | --- | --- | --- | --- | --- | --- | --- | --- | --- | --- | --- | --- | --- | --- | --- | --- | --- | --- | --- | --- | --- | --- | --- | --- | --- | --- | --- | --- | --- | --- | --- | --- | --- | --- | --- | --- | --- | --- | --- | --- | --- | --- | --- | --- | --- | --- | --- | --- | --- | --- | --- | --- | --- | --- | --- | --- | --- | --- | --- | --- | --- | --- | --- | --- | --- | --- | --- | --- | --- | --- | --- | --- | --- | --- | --- | --- | --- | --- | --- | --- | --- | --- | --- | --- | --- | --- | --- | --- | --- | --- | --- | --- | --- | --- | --- | --- | --- | --- | --- | --- | --- | --- | --- | --- | --- | --- | --- | --- | --- | --- | --- | --- | --- | --- | --- | --- | --- | --- | --- | --- | --- | --- | --- | --- | --- | --- | --- | --- | --- | --- | --- | --- | --- | --- | --- | --- | --- | --- | --- | --- | --- | --- | --- | --- | --- | --- | --- | --- | --- | --- | --- | --- | --- | --- | --- | --- | --- | --- | --- | --- | --- | --- | --- | --- | --- | --- | --- | --- | --- | --- | --- | --- | --- | --- | --- | --- | --- | --- | --- | --- | --- | --- | --- | --- | --- | --- | --- | --- | --- | --- | --- | --- | --- | --- | --- | --- | --- | --- | --- | --- | --- | --- | --- | --- | --- | --- | --- | --- | --- | --- | --- | --- | --- | --- | --- | --- | --- | --- | --- | --- | --- | --- | --- | --- | --- | --- | --- | --- | --- | --- | --- | --- | --- | --- | --- | --- | --- | --- | --- | --- | --- | --- | --- | --- | --- | --- | --- | --- | --- | --- | --- | --- | --- | --- | --- | --- | --- | --- | --- | --- | --- | --- | --- | --- | --- | --- | --- | --- | --- | --- | --- | --- | --- | --- | --- | --- | --- | --- | --- | --- | --- | --- | --- | --- | --- | --- | --- | --- | --- | --- | --- | --- | --- | --- | --- | --- | --- | --- | --- | --- | --- | --- | --- | --- | --- | --- | --- | --- | --- | --- | --- | --- | --- | --- | --- | --- | --- | --- | --- | --- | --- | --- | --- | --- | --- | --- | --- | --- | --- | --- | --- | --- | --- | --- | --- | --- | --- | --- | --- | --- | --- | --- | --- | --- | --- | --- | --- | --- | --- | --- | --- | --- | --- | --- | --- | --- | --- | --- | --- | --- | --- | --- | --- | --- | --- | --- | --- | --- | --- | --- | --- | --- | --- | --- | --- | --- | --- | --- | --- | --- | --- | --- | --- | --- | --- | --- | --- | --- | --- | --- | --- | --- | --- | --- | --- | --- | --- | --- | --- | --- | --- | --- | --- | --- | --- | --- | --- | --- | --- | --- | --- | --- | --- | --- | --- | --- | --- | --- | --- | --- | --- | --- | --- | --- | --- | --- | --- | --- | --- | --- | --- | --- | --- | --- | --- | --- | --- | --- | --- | --- | --- | --- | --- | --- | --- | --- | --- | --- | --- | --- | --- | --- | --- | --- | --- | --- | --- | --- | --- | --- | --- | --- | --- | --- | --- | --- | --- | --- | --- | --- | --- | --- | --- | --- | --- | --- | --- | --- | --- | --- | --- | --- | --- | --- | --- | --- | --- | --- | --- | --- | --- | --- | --- | --- | --- | --- | --- | --- | --- | --- | --- | --- | --- | --- | --- | --- | --- | --- | --- | --- | --- | --- | --- | --- | --- | --- | --- | --- | --- | --- | --- | --- | --- | --- | --- | --- | --- | --- | --- | --- | --- | --- | --- | --- | --- | --- | --- | --- | --- | --- | --- | --- | --- | --- | --- | --- | --- | --- | --- | --- | --- | --- | --- | --- | --- | --- | --- | --- | --- | --- | --- | --- | --- | --- | --- | --- | --- | --- | --- | --- | --- | --- | --- | --- | --- | --- | --- | --- | --- | --- | --- | --- | --- | --- | --- | --- | --- | --- | --- | --- | --- | --- | --- | --- | --- | --- | --- | --- | --- | --- | --- | --- | --- | --- | --- | --- | --- | --- | --- | --- | --- | --- | --- | --- | --- | --- | --- | --- | --- | --- | --- | --- | --- | --- | --- | --- | --- | --- | --- | --- | --- | --- | --- | --- | --- | --- | --- | --- | --- | --- | --- | --- | --- | --- | --- | --- | --- | --- | --- | --- | --- | --- | --- | --- | --- | --- | --- | --- | --- | --- | --- | --- | --- | --- | --- | --- | --- | --- | --- | --- | --- | --- | --- | --- | --- | --- | --- | --- | --- | --- | --- | --- | --- | --- | --- | --- | --- | --- | --- | --- | --- | --- | --- | --- | --- | --- | --- | --- | --- | --- | --- | --- | --- | --- | --- | --- | --- | --- | --- | --- | --- | --- | --- | --- | --- | --- | --- | --- | --- | --- | --- | --- | --- | --- | --- | --- | --- | --- | --- | --- | --- | --- | --- | --- | --- | --- | --- | --- | --- | --- | --- | --- | --- | --- | --- | --- | --- | --- | --- | --- | --- | --- | --- | --- | --- | --- | --- | --- | --- | --- | --- | --- | --- | --- | --- | --- | --- | --- | --- | --- | --- | --- | --- | --- | --- | --- | --- | --- | --- | --- | --- | --- | --- | --- | --- | --- | --- | --- | --- | --- | --- | --- | --- | --- | --- | --- | --- | --- | --- | --- | --- | --- | --- | --- | --- | --- | --- | --- | --- | --- | --- | --- | --- | --- | --- | --- | --- | --- | --- | --- | --- | --- | --- | --- | --- | --- | --- | --- | --- | --- | --- | --- | --- | --- | --- | --- | --- | --- | --- | --- | --- | --- | --- | --- | --- | --- | --- | --- | --- | --- | --- | --- | --- | --- | --- | --- | --- | --- | --- | --- | --- | --- | --- | --- | --- | --- | --- | --- | --- | --- | --- | --- | --- | --- | --- | --- | --- | --- | --- | --- | --- | --- | --- | --- | --- | --- | --- | --- | --- | --- | --- | --- | --- | --- | --- | --- | --- | --- | --- | --- | --- | --- | --- | --- | --- | --- | --- | --- | --- | --- | --- | --- | --- | --- | --- | --- | --- | --- | --- | --- | --- | --- | --- | --- | --- | --- | --- | --- | --- | --- | --- | --- | --- | --- | --- | --- | --- | --- | --- | --- | --- | --- | --- | --- | --- | --- | --- | --- | --- | --- | --- | --- | --- | --- | --- | --- | --- | --- | --- | --- | --- | --- | --- | --- | --- | --- | --- | --- | --- | --- | --- | --- | --- | --- | --- | --- | --- | --- | --- | --- | --- | --- | --- | --- | --- | --- | --- | --- | --- | --- | --- | --- | --- | --- | --- | --- | --- | --- | --- | --- | --- | --- | --- | --- | --- | --- | --- | --- | --- | --- | --- | --- | --- | --- | --- | --- | --- | --- | --- | --- | --- | --- | --- | --- | --- | --- | --- | --- | --- | --- | --- | --- | --- | --- | --- | --- | --- | --- | --- | --- | --- | --- | --- | --- | --- | --- | --- | --- | --- | --- | --- | --- | --- | --- | --- | --- | --- | --- | --- | --- | --- | --- | --- | --- | --- | --- | --- | --- | --- | --- | --- | --- | --- | --- | --- | --- | --- | --- | --- | --- | --- | --- | --- | --- | --- | --- | --- | --- | --- | --- | --- | --- | --- | --- | --- | --- | --- | --- | --- | --- | --- | --- | --- | --- | --- | --- | --- | --- | --- | --- | --- | --- | --- | --- | --- | --- | --- | --- | --- | --- | --- | --- | --- | --- | --- | --- | --- | --- | --- | --- | --- | --- | --- | --- | --- | --- | --- | --- | --- | --- | --- | --- | --- | --- | --- | --- | --- | --- | --- | --- | --- | --- | --- | --- | --- | --- | --- | --- | --- | --- | --- | --- | --- | --- | --- | --- | --- | --- | --- | --- | --- | --- | --- | --- | --- | --- | --- | --- | --- | --- | --- | --- | --- | --- | --- | --- | --- | --- | --- | --- | --- | --- | --- | --- | --- | --- | --- | --- | --- | --- | --- | --- | --- | --- | --- | --- | --- | --- | --- | --- | --- | --- | --- | --- | --- | --- | --- | --- | --- | --- | --- | --- | --- | --- | --- | --- | --- | --- | --- | --- | --- | --- | --- | --- | --- | --- | --- | --- | --- | --- | --- | --- | --- | --- | --- | --- | --- | --- | --- | --- | --- | --- | --- | --- | --- | --- | --- | --- | --- | --- | --- | --- | --- | --- | --- | --- | --- | --- | --- | --- | --- | --- | --- | --- | --- | --- | --- | --- | --- | --- | --- | --- | --- | --- | --- | --- | --- | --- | --- | --- | --- | --- | --- | --- | --- | --- | --- | --- | --- | --- | --- | --- | --- | --- | --- | --- | --- | --- | --- | --- | --- | --- | --- | --- | --- | --- | --- | --- | --- | --- | --- | --- | --- | --- | --- | --- | --- | --- | --- | --- | --- | --- | --- | --- | --- | --- | --- | --- | --- | --- | --- | --- | --- | --- | --- | --- | --- | --- | --- | --- | --- | --- | --- | --- | --- | --- | --- | --- | --- | --- | --- | --- | --- | --- | --- | --- | --- | --- | --- | --- | --- | --- | --- | --- | --- | --- | --- | --- | --- | --- | --- | --- | --- | --- | --- | --- | --- | --- | --- | --- | --- | --- | --- | --- | --- | --- | --- | --- | --- | --- | --- | --- | --- | --- | --- | --- | --- | --- | --- | --- | --- | --- | --- | --- | --- | --- | --- | --- | --- | --- | --- | --- | --- | --- | --- | --- | --- | --- | --- | --- | --- | --- | --- | --- | --- | --- | --- | --- | --- | --- | --- | --- | --- | --- | --- | --- | --- | --- | --- | --- | --- | --- | --- | --- | --- | --- | --- | --- | --- | --- | --- | --- | --- | --- | --- | --- | --- | --- | --- | --- | --- | --- | --- | --- | --- | --- | --- | --- | --- | --- | --- | --- | --- | --- | --- | --- | --- | --- | --- | --- | --- | --- | --- | --- | --- | --- | --- | --- | --- | --- | --- | --- | --- | --- | --- | --- | --- | --- | --- | --- | --- | --- | --- | --- | --- | --- | --- | --- | --- | --- | --- | --- | --- | --- | --- | --- | --- | --- | --- | --- | --- | --- | --- | --- | --- | --- | --- | --- | --- | --- | --- | --- | --- | --- | --- | --- | --- | --- | --- | --- | --- | --- | --- | --- | --- | --- | --- | --- | --- | --- | --- | --- | --- | --- | --- | --- | --- | --- | --- | --- | --- | --- | --- | --- | --- | --- | --- | --- | --- | --- | --- | --- | --- | --- | --- | --- | --- | --- | --- | --- | --- | --- | --- | --- | --- | --- | --- | --- | --- | --- | --- | --- | --- | --- | --- | --- | --- | --- | --- | --- | --- | --- | --- | --- | --- | --- | --- | --- | --- | --- | --- | --- | --- | --- | --- | --- | --- | --- | --- | --- | --- | --- | --- | --- | --- | --- | --- | --- | --- | --- | --- | --- | --- | --- | --- | --- | --- | --- | --- | --- | --- | --- | --- | --- | --- | --- | --- | --- | --- | --- | --- | --- | --- | --- | --- | --- | --- | --- | --- | --- | --- | --- | --- | --- | --- | --- | --- | --- | --- | --- | --- | --- | --- | --- | --- | --- | --- | --- | --- | --- | --- | --- | --- | --- | --- | --- | --- | --- | --- | --- | --- | --- | --- | --- | --- | --- | --- | --- | --- | --- | --- | --- | --- | --- | --- | --- | --- | --- | --- | --- | --- | --- | --- | --- | --- | --- | --- | --- | --- | --- | --- | --- | --- | --- | --- | --- | --- | --- | --- | --- | --- | --- | --- | --- | --- | --- | --- | --- | --- | --- | --- | --- | --- | --- | --- | --- | --- | --- | --- | --- | --- | --- | --- | --- | --- | --- | --- | --- | --- | --- | --- | --- | --- | --- | --- | --- | --- | --- | --- | --- | --- | --- | --- | --- | --- | --- | --- | --- | --- | --- | --- | --- | --- | --- | --- | --- | --- | --- | --- | --- | --- | --- | --- | --- | --- | --- | --- | --- | --- | --- | --- | --- | --- | --- | --- | --- | --- | --- | --- | --- | --- | --- | --- | --- | --- | --- | --- | --- | --- | --- | --- | --- | --- | --- | --- | --- | --- | --- | --- | --- | --- | --- | --- | --- | --- | --- | --- | --- | --- | --- | --- | --- | --- | --- | --- | --- | --- | --- | --- | --- | --- | --- | --- | --- | --- | --- | --- | --- | --- | --- | --- | --- | --- | --- | --- | --- | --- | --- | --- | --- | --- | --- | --- | --- | --- | --- | --- | --- | --- | --- | --- | --- | --- | --- | --- | --- | --- | --- | --- | --- | --- | --- | --- | --- | --- | --- | --- | --- | --- | --- | --- | --- | --- | --- | --- | --- | --- | --- | --- | --- | --- | --- | --- | --- | --- | --- | --- | --- | --- | --- | --- | --- | --- | --- | --- | --- | --- | --- | --- | --- | --- | --- | --- | --- | --- | --- | --- | --- | --- | --- | --- | --- | --- | --- | --- | --- | --- | --- | --- | --- | --- | --- | --- | --- | --- | --- | --- | --- | --- | --- | --- | --- | --- | --- | --- | --- | --- | --- | --- | --- | --- | --- | --- | --- | --- | --- | --- | --- | --- | --- | --- | --- | --- | --- | --- | --- | --- | --- | --- | --- | --- | --- | --- | --- | --- | --- | --- | --- | --- | --- | --- | --- | --- | --- | --- | --- | --- | --- | --- | --- | --- | --- | --- | --- | --- | --- | --- | --- | --- | --- | --- | --- | --- | --- | --- | --- | --- | --- | --- | --- | --- | --- | --- | --- | --- | --- | --- | --- | --- | --- | --- | --- | --- | --- | --- | --- | --- | --- | --- | --- | --- | --- | --- | --- | --- | --- | --- | --- | --- | --- | --- | --- | --- | --- | --- | --- | --- | --- | --- | --- | --- | --- | --- | --- | --- | --- | --- | --- | --- | --- | --- | --- | --- | --- | --- | --- | --- | --- | --- | --- | --- | --- | --- | --- | --- | --- | --- | --- | --- | --- | --- | --- | --- | --- | --- | --- | --- | --- | --- | --- | --- | --- | --- | --- | --- | --- | --- | --- | --- | --- | --- | --- | --- | --- | --- | --- | --- | --- | --- | --- | --- | --- | --- | --- | --- | --- | --- | --- | --- | --- | --- | --- | --- | --- | --- | --- | --- | --- | --- | --- | --- | --- | --- | --- | --- | --- | --- | --- | --- | --- | --- | --- | --- | --- | --- | --- | --- | --- | --- | --- | --- | --- | --- | --- | --- | --- | --- | --- | --- | --- | --- | --- | --- | --- | --- | --- | --- | --- | --- | --- | --- | --- | --- | --- | --- | --- | --- | --- | --- | --- | --- | --- | --- | --- | --- | --- | --- | --- | --- | --- | --- | --- | --- | --- | --- | --- | --- | --- | --- | --- | --- | --- | --- | --- | --- | --- | --- | --- | --- | --- | --- | --- | --- | --- | --- | --- | --- | --- | --- | --- | --- | --- | --- | --- | --- | --- | --- | --- | --- | --- | --- | --- | --- | --- | --- | --- | --- | --- | --- | --- | --- | --- | --- | --- | --- | --- | --- | --- | --- | --- | --- | --- | --- | --- | --- | --- | --- | --- | --- | --- | --- | --- | --- | --- | --- | --- | --- | --- | --- | --- | --- | --- | --- | --- | --- | --- | --- | --- | --- | --- | --- | --- | --- | --- | --- | --- | --- | --- | --- | --- | --- | --- | --- | --- | --- | --- | --- | --- | --- | --- | --- | --- | --- | --- | --- | --- | --- | --- | --- | --- | --- | --- | --- | --- | --- | --- | --- | --- | --- | --- | --- | --- | --- | --- | --- | --- | --- | --- | --- | --- | --- | --- | --- | --- | --- | --- | --- | --- | --- | --- | --- | --- | --- | --- | --- | --- | --- | --- | --- | --- | --- | --- | --- | --- | --- | --- | --- | --- | --- | --- | --- | --- | --- | --- | --- | --- | --- | --- | --- | --- | --- | --- | --- | --- | --- | --- | --- | --- | --- | --- | --- | --- | --- | --- | --- | --- | --- | --- | --- | --- | --- | --- | --- | --- | --- | --- | --- | --- | --- | --- | --- | --- | --- | --- | --- | --- | --- | --- | --- | --- | --- | --- | --- | --- | --- | --- | --- | --- | --- | --- | --- | --- | --- | --- | --- | --- | --- | --- | --- | --- | --- | --- | --- | --- | --- | --- | --- | --- | --- | --- | --- | --- | --- | --- | --- | --- | --- | --- | --- | --- | --- | --- | --- | --- | --- | --- | --- | --- | --- | --- | --- | --- | --- | --- | --- | --- | --- | --- | --- | --- | --- | --- | --- | --- | --- | --- | --- | --- | --- | --- | --- | --- | --- | --- | --- | --- | --- | --- | --- | --- | --- | --- | --- | --- | --- | --- | --- | --- | --- | --- | --- | --- | --- | --- | --- | --- | --- | --- | --- | --- | --- | --- | --- | --- | --- | --- | --- | --- | --- | --- | --- | --- | --- | --- | --- | --- | --- | --- | --- | --- | --- | --- | --- | --- | --- | --- | --- | --- | --- | --- | --- | --- | --- | --- | --- | --- | --- | --- | --- | --- | --- | --- | --- | --- | --- | --- | --- | --- | --- | --- | --- | --- | --- | --- | --- | --- | --- | --- | --- | --- | --- | --- | --- | --- | --- | --- | --- | --- | --- | --- | --- | --- | --- | --- | --- | --- | --- | --- | --- | --- | --- | --- | --- | --- | --- | --- | --- | --- | --- | --- | --- | --- | --- | --- | --- | --- | --- | --- | --- | --- | --- | --- | --- | --- | --- | --- | --- | --- | --- | --- | --- | --- | --- | --- | --- | --- | --- | --- | --- | --- | --- | --- | --- | --- | --- | --- | --- | --- | --- | --- | --- | --- | --- | --- | --- | --- | --- | --- | --- | --- | --- | --- | --- | --- | --- | --- | --- | --- | --- | --- | --- | --- | --- | --- | --- | --- | --- | --- | --- | --- | --- | --- | --- | --- | --- | --- | --- | --- | --- | --- | --- | --- | --- | --- | --- | --- | --- | --- | --- | --- | --- | --- | --- | --- | --- | --- | --- | --- | --- | --- | --- | --- | --- | --- | --- | --- | --- | --- | --- | --- | --- | --- | --- | --- | --- | --- | --- | --- | --- | --- | --- | --- | --- | --- | --- | --- | --- | --- | --- | --- | --- | --- | --- | --- | --- | --- | --- | --- | --- | --- | --- | --- | --- | --- | --- | --- | --- | --- | --- | --- | --- | --- | --- | --- | --- | --- | --- | --- | --- | --- | --- | --- | --- | --- | --- | --- | --- | --- | --- | --- | --- | --- | --- | --- | --- | --- | --- | --- | --- | --- | --- | --- | --- | --- | --- | --- | --- | --- | --- | --- | --- | --- | --- | --- | --- | --- | --- | --- | --- | --- | --- | --- | --- | --- | --- | --- | --- | --- | --- | --- | --- | --- | --- | --- | --- | --- | --- | --- | --- | --- | --- | --- | --- | --- | --- | --- | --- | --- | --- | --- | --- | --- | --- | --- | --- | --- | --- | --- | --- | --- | --- | --- | --- | --- | --- | --- | --- | --- | --- | --- | --- | --- | --- | --- | --- | --- | --- | --- | --- | --- | --- | --- | --- | --- | --- | --- | --- | --- | --- | --- | --- | --- | --- | --- | --- | --- | --- | --- | --- | --- | --- | --- | --- | --- | --- | --- | --- | --- | --- | --- | --- | --- | --- | --- | --- | --- | --- | --- | --- | --- | --- | --- | --- | --- | --- | --- | --- | --- | --- | --- | --- | --- | --- | --- | --- | --- | --- | --- | --- | --- | --- | --- | --- | --- | --- | --- | --- | --- | --- | --- | --- | --- | --- | --- | --- | --- | --- | --- | --- | --- | --- | --- | --- | --- | --- | --- | --- | --- | --- | --- | --- | --- | --- | --- | --- | --- | --- | --- | --- | --- | --- | --- | --- | --- | --- | --- | --- | --- | --- | --- | --- | --- | --- | --- | --- | --- | --- | --- | --- | --- | --- | --- | --- | --- | --- | --- | --- | --- | --- | --- | --- | --- | --- | --- | --- | --- | --- | --- | --- | --- | --- | --- | --- | --- | --- | --- | --- | --- | --- | --- | --- | --- | --- | --- | --- | --- | --- | --- | --- | --- | --- | --- | --- | --- | --- | --- | --- | --- | --- | --- | --- | --- | --- | --- | --- | --- | --- | --- | --- | --- | --- | --- | --- | --- | --- | --- | --- | --- | --- | --- | --- | --- | --- | --- | --- | --- | --- | --- | --- | --- | --- | --- | --- | --- | --- | --- | --- | --- | --- | --- | --- | --- | --- | --- | --- | --- | --- | --- | --- | --- | --- | --- | --- | --- | --- | --- | --- | --- | --- | --- | --- | --- | --- | --- | --- | --- | --- | --- | --- | --- | --- | --- | --- | --- | --- | --- | --- | --- | --- | --- | --- | --- | --- | --- | --- | --- | --- | --- | --- | --- | --- | --- | --- | --- | --- | --- | --- | --- | --- | --- | --- | --- | --- | --- | --- | --- | --- | --- | --- | --- | --- | --- | --- | --- | --- | --- | --- | --- | --- | --- | --- | --- | --- | --- | --- | --- | --- | --- | --- | --- | --- | --- | --- | --- | --- | --- | --- | --- | --- | --- | --- | --- | --- | --- | --- | --- | --- | --- | --- | --- | --- | --- | --- | --- | --- | --- | --- | --- | --- | --- | --- | --- | --- | --- | --- | --- | --- | --- | --- | --- | --- | --- | --- | --- | --- | --- | --- | --- | --- | --- | --- | --- | --- | --- | --- | --- | --- | --- | --- | --- | --- | --- | --- | --- | --- | --- | --- | --- | --- | --- | --- | --- | --- | --- | --- | --- | --- | --- | --- | --- | --- | --- | --- | --- | --- | --- | --- | --- | --- | --- | --- | --- | --- | --- | --- | --- | --- | --- | --- | --- | --- | --- | --- | --- | --- | --- | --- | --- | --- | --- | --- | --- | --- | --- | --- | --- | --- | --- | --- | --- | --- | --- | --- | --- | --- | --- | --- | --- | --- | --- | --- | --- | --- | --- | --- | --- | --- | --- | --- | --- | --- | --- | --- | --- | --- | --- | --- | --- | --- | --- | --- | --- | --- | --- | --- | --- | --- | --- | --- | --- | --- | --- | --- | --- | --- | --- | --- | --- | --- | --- | --- | --- | --- | --- | --- | --- | --- | --- | --- | --- | --- | --- | --- | --- | --- | --- | --- | --- | --- | --- | --- | --- | --- | --- | --- | --- | --- | --- | --- | --- | --- | --- | --- | --- | --- | --- | --- | --- | --- | --- | --- | --- | --- | --- | --- | --- | --- | --- | --- | --- | --- | --- | --- | --- | --- | --- | --- | --- | --- | --- | --- | --- | --- | --- | --- | --- | --- | --- | --- | --- | --- | --- | --- | --- | --- | --- | --- | --- | --- | --- | --- | --- | --- | --- | --- | --- | --- | --- | --- | --- | --- | --- | --- | --- | --- | --- | --- | --- | --- | --- | --- | --- | --- | --- | --- | --- | --- | --- | --- | --- | --- | --- | --- | --- | --- | --- | --- | --- | --- | --- | --- | --- | --- | --- | --- | --- | --- | --- | --- | --- | --- | --- | --- | --- | --- | --- | --- | --- | --- | --- | --- | --- | --- | --- | --- | --- | --- | --- | --- | --- | --- | --- | --- | --- | --- | --- | --- | --- | --- | --- | --- | --- | --- | --- | --- | --- | --- | --- | --- | --- | --- | --- | --- | --- | --- | --- | --- | --- | --- | --- | --- | --- | --- | --- | --- | --- | --- | --- | --- | --- | --- | --- | --- | --- | --- | --- | --- | --- | --- | --- | --- | --- | --- | --- | --- | --- | --- | --- | --- | --- | --- | --- | --- | --- | --- | --- | --- | --- | --- | --- | --- | --- | --- | --- | --- | --- | --- | --- | --- | --- | --- | --- | --- | --- | --- | --- | --- | --- | --- | --- | --- | --- | --- | --- | --- | --- | --- | --- | --- | --- | --- | --- | --- | --- | --- | --- | --- | --- | --- | --- | --- | --- | --- | --- | --- | --- | --- | --- | --- | --- | --- | --- | --- | --- | --- | --- | --- | --- | --- | --- | --- | --- | --- | --- | --- | --- | --- | --- | --- | --- | --- | --- | --- | --- | --- | --- | --- | --- | --- | --- | --- | --- | --- | --- | --- | --- | --- | --- | --- | --- | --- | --- | --- | --- | --- | --- | --- | --- | --- | --- | --- | --- | --- | --- | --- | --- | --- | --- | --- | --- | --- | --- | --- | --- | --- | --- | --- | --- | --- | --- | --- | --- | --- | --- | --- | --- | --- | --- | --- | --- | --- | --- | --- | --- | --- | --- | --- | --- | --- | --- | --- | --- | --- | --- | --- | --- | --- | --- | --- | --- | --- | --- | --- | --- | --- | --- | --- | --- | --- | --- | --- | --- | --- | --- | --- | --- | --- | --- | --- | --- | --- | --- | --- | --- | --- | --- | --- | --- | --- | --- | --- | --- | --- | --- | --- | --- | --- | --- | --- | --- | --- | --- | --- | --- | --- | --- | --- | --- | --- | --- | --- | --- | --- | --- | --- | --- | --- | --- | --- | --- | --- | --- |
| |  |  |  |  |  |  |  |  |  |  |  |  |  |  |  |  |  |  |  |  |  |  |  |  |  |  |  |  |  |  |  |  |  |  |  |  |  |  |  |  |  |  |  |  |  |  |  |  |  |  |  |  |  |  |  |  |  |  | | --- | --- | --- | --- | --- | --- | --- | --- | --- | --- | --- | --- | --- | --- | --- | --- | --- | --- | --- | --- | --- | --- | --- | --- | --- | --- | --- | --- | --- | --- | --- | --- | --- | --- | --- | --- | --- | --- | --- | --- | --- | --- | --- | --- | --- | --- | --- | --- | --- | --- | --- | --- | --- | --- | --- | --- | --- | --- | | G0VAM7/1-523 | 1 | M | F | R | L | V | N | G | R | T | Q | R | S | N | L | N | K | L | V | - | - | - | - | - | - | - | R | P | P | R | T | T | I | P | Q | S | L | - | - | - | - | C | S | Y | S | - | T | K | S | E | S | I | N | A | N | V | 43 | | Q6CN98/1-532 | 1 | M | L | R | - | Q | S | A | R | S | S | V | N | N | I | A | R | L | S | Y | K | R | P | T | T | F | V | N | N | G | I | A | T | Q | L | N | S | - | - | - | - | M | K | N | Y | Y | S | Q | E | A | I | N | I | D | N | K | 50 | | Q6FKS3/1-511 | 1 | M | - | - | - | - | - | - | - | - | - | - | - | - | L | A | K | S | L | - | - | - | - | - | - | - | - | K | P | I | V | Q | R | G | L | S | L | - | - | - | - | T | M | R | N | S | S | N | L | A | A | V | K | V | N | D | 31 | | Q750F2/1-518 | 1 | M | - | - | - | - | - | - | K | I | N | G | A | L | L | P | S | L | R | - | - | - | - | - | - | - | R | S | N | G | L | K | I | I | A | A | V | - | - | - | - | - | - | A | R | M | S | S | V | A | D | S | S | S | N | T | 36 | | A7TSJ9/1-525 | 1 | M | I | - | - | D | L | L | K | T | R | I | N | V | L | S | K | I | S | - | - | - | - | - | - | - | K | N | Q | R | T | V | S | N | I | I | F | - | - | - | - | K | H | T | M | S | S | S | S | P | I | N | S | S | N | E | 42 | | C5DXE8/1-523 | 1 | M | L | - | - | - | S | T | R | T | K | G | A | I | C | L | G | R | K | - | - | - | - | - | - | - | T | H | I | G | A | K | T | L | T | L | G | - | - | - | - | Q | N | Y | T | T | S | S | H | A | H | T | T | T | N | Q | 41 | | Sbay\_661.8/1-520 | 1 | M | F | - | - | - | - | - | - | - | - | - | - | - | L | A | K | N | L | - | - | - | - | - | - | - | K | N | N | R | I | A | V | Y | L | P | N | E | I | Y | F | V | Q | R | H | F | L | N | T | A | S | S | Q | T | N | E | 37 | | SAKL0B02948g/1-524 | 1 | M | I | S | - | S | G | A | R | A | T | S | S | A | C | K | S | L | H | - | - | - | - | - | - | - | T | I | T | T | V | K | A | F | L | S | S | - | - | - | - | T | S | G | H | - | R | A | K | A | S | I | K | D | N | K | 42 | | Q12428/1-516 | 1 | M | F | - | - | - | - | - | - | - | - | - | - | - | L | A | K | N | L | - | - | - | - | - | - | - | K | N | N | K | I | K | V | C | L | P | K | - | - | - | - | K | K | F | A | A | L | S | T | A | S | I | Q | T | N | E | 33 | |  | | G0VAM7/1-523 | 44 | R | P | K | P | D | I | V | L | D | E | I | A | T | Y | V | H | D | K | K | I | V | S | P | L | A | F | E | T | A | K | L | C | F | L | D | A | L | G | C | G | L | A | S | L | K | Y | N | K | V | Q | D | I | I | K | P | 98 | | Q6CN98/1-532 | 51 | R | P | E | A | D | E | V | L | R | K | I | S | S | Y | V | H | N | K | G | I | T | S | P | E | A | Y | E | T | A | R | L | C | L | L | D | T | L | G | C | G | L | A | A | L | K | Y | D | Q | P | K | N | L | I | K | P | 105 | | Q6FKS3/1-511 | 32 | R | P | D | P | D | L | V | L | V | D | I | A | E | Y | V | H | H | H | K | I | D | S | K | L | A | F | D | T | A | R | Y | C | L | L | D | T | L | G | C | G | L | A | A | L | K | Y | N | Q | V | Q | N | M | I | K | P | 86 | | Q750F2/1-518 | 37 | R | P | Q | A | D | Q | V | L | K | D | I | A | S | Y | V | H | N | T | R | I | T | S | T | E | A | F | K | T | A | R | L | C | L | L | D | T | L | G | C | G | L | A | A | L | R | Y | Q | E | P | R | G | V | I | K | P | 91 | | A7TSJ9/1-525 | 43 | R | P | K | T | D | T | V | I | S | E | I | S | D | Y | V | M | D | K | K | I | N | S | S | L | A | F | E | T | A | K | L | C | F | I | D | A | L | G | C | G | L | A | A | L | K | F | D | Q | A | V | K | I | I | K | P | 97 | | C5DXE8/1-523 | 42 | R | P | E | P | D | K | I | L | K | D | I | A | D | Y | V | H | Q | A | K | I | T | S | Q | V | A | L | E | T | A | K | L | C | L | L | D | T | L | G | C | G | L | A | A | L | K | H | Q | Q | A | R | D | I | I | T | P | 96 | | Sbay\_661.8/1-520 | 38 | R | P | K | A | D | K | V | L | R | D | I | A | K | Y | V | H | E | T | P | I | T | S | T | L | A | L | D | T | A | K | L | C | F | L | D | T | L | G | C | G | L | A | A | L | K | F | E | Q | A | R | N | I | I | K | P | 92 | | SAKL0B02948g/1-524 | 43 | R | P | E | P | D | Q | V | L | K | D | I | A | S | Y | V | H | N | T | P | I | T | S | P | D | A | F | E | T | A | K | L | C | F | L | D | T | L | G | C | G | L | A | A | L | K | Y | D | Q | S | R | A | I | I | K | P | 97 | | Q12428/1-516 | 34 | R | P | N | P | D | K | V | L | K | D | I | A | K | Y | V | H | E | T | P | L | K | S | S | L | A | L | D | T | A | R | L | C | F | L | D | T | L | G | C | G | L | A | A | L | K | F | K | Q | A | Q | N | I | I | K | P | 88 | |  | | G0VAM7/1-523 | 99 | I | V | P | N | M | I | V | P | N | G | T | K | V | L | G | T | K | Y | E | M | D | P | V | K | G | A | F | A | I | G | T | M | I | R | W | L | D | F | N | D | C | W | L | A | K | E | W | G | H | P | S | D | N | L | G | 153 | | Q6CN98/1-532 | 106 | I | V | P | G | T | I | V | P | N | G | T | K | V | L | G | T | K | Y | R | M | D | P | V | K | G | A | F | A | I | G | T | L | I | R | W | L | D | F | N | D | C | W | L | A | A | E | W | G | H | P | S | D | N | L | G | 160 | | Q6FKS3/1-511 | 87 | I | V | P | G | M | I | V | P | N | G | V | K | V | I | G | T | D | H | V | M | D | P | I | K | G | A | F | A | I | G | T | I | I | R | W | L | D | F | N | D | C | W | L | A | A | E | W | G | H | P | S | D | N | L | G | 141 | | Q750F2/1-518 | 92 | I | V | P | G | T | I | V | P | N | G | T | R | V | L | G | T | K | Y | R | M | D | P | V | S | G | A | F | A | I | G | T | L | I | R | W | L | D | Y | N | D | C | W | L | A | A | E | W | G | H | P | S | D | N | L | G | 146 | | A7TSJ9/1-525 | 98 | I | V | P | G | T | I | V | P | N | G | T | R | V | I | G | T | N | L | I | Y | D | P | V | R | G | A | F | A | N | G | S | L | I | R | W | L | D | Y | N | D | C | W | L | A | A | E | W | G | H | P | S | D | N | L | G | 152 | | C5DXE8/1-523 | 97 | I | V | P | D | T | I | I | P | N | G | T | K | V | L | G | T | N | Y | K | L | G | P | V | E | G | A | F | A | I | G | T | L | I | R | W | L | D | Y | N | D | C | W | L | A | A | E | W | G | H | P | S | D | N | L | G | 151 | | Sbay\_661.8/1-520 | 93 | I | V | E | G | T | I | V | P | N | G | T | K | I | L | G | T | S | Y | V | M | D | P | V | R | G | A | F | A | I | G | T | L | I | R | W | L | D | Y | N | D | C | W | L | A | A | E | W | G | H | P | S | D | N | L | G | 147 | | SAKL0B02948g/1-524 | 98 | I | V | P | G | T | I | V | P | N | G | T | K | I | P | G | T | K | Y | R | M | D | P | V | R | G | A | F | A | I | G | T | L | V | R | W | L | D | Y | N | D | C | W | L | A | A | E | W | G | H | P | S | D | N | L | G | 152 | | Q12428/1-516 | 89 | I | V | P | G | T | I | V | P | S | G | T | K | I | L | G | T | S | Y | V | M | D | P | V | K | G | A | F | A | I | G | T | L | I | R | W | L | D | Y | N | D | C | W | L | A | A | E | W | G | H | P | S | D | N | L | G | 143 | |  | | G0VAM7/1-523 | 154 | G | I | L | A | V | A | D | Y | S | S | R | L | F | K | A | T | D | G | K | E | G | K | R | F | T | V | N | D | I | L | E | G | M | I | K | A | H | E | I | Q | G | V | I | A | L | D | N | S | F | N | E | V | G | L | D | 208 | | Q6CN98/1-532 | 161 | G | I | L | A | V | A | D | H | L | T | R | L | S | K | A | T | G | G | D | E | G | R | L | F | T | V | K | D | V | L | E | A | M | I | K | A | H | E | I | Q | G | I | I | A | L | E | N | S | F | N | K | V | G | L | D | 215 | | Q6FKS3/1-511 | 142 | G | I | L | A | V | A | D | Y | E | S | R | L | Y | K | A | T | E | G | R | E | G | K | L | F | K | V | R | D | V | L | T | A | M | I | K | A | H | E | I | Q | G | V | F | A | L | D | N | S | F | N | R | V | G | L | D | 196 | | Q750F2/1-518 | 147 | G | I | L | A | V | A | D | Y | M | T | R | L | S | Q | S | S | G | G | D | E | G | R | L | F | T | I | R | E | V | L | E | A | M | I | K | A | H | E | I | Q | G | I | L | A | L | D | N | A | F | N | K | V | G | L | D | 201 | | A7TSJ9/1-525 | 153 | G | I | I | S | V | A | D | Y | L | T | R | L | Y | K | S | S | D | G | E | E | G | R | L | F | T | I | N | D | V | L | E | C | M | I | K | A | H | E | I | Q | G | V | I | A | L | D | N | S | F | N | K | V | G | L | D | 207 | | C5DXE8/1-523 | 152 | G | I | L | A | V | A | D | Y | L | T | R | L | S | R | A | T | S | G | K | E | G | K | V | F | T | V | G | D | V | L | E | G | M | I | K | A | H | E | I | Q | G | V | I | A | L | E | N | S | F | N | S | V | G | L | D | 206 | | Sbay\_661.8/1-520 | 148 | G | I | L | A | V | A | D | Y | L | T | R | L | N | R | A | T | H | G | K | N | G | K | E | F | L | V | K | D | V | L | E | A | M | V | K | A | H | E | I | Q | G | I | I | A | L | E | N | S | F | N | K | V | G | L | D | 202 | | SAKL0B02948g/1-524 | 153 | G | I | I | A | V | G | D | Y | L | T | R | L | S | K | A | T | G | G | E | D | G | R | F | F | T | V | R | D | I | L | E | A | M | I | K | A | H | E | I | Q | G | I | I | A | L | E | N | S | F | N | K | V | G | L | D | 207 | | Q12428/1-516 | 144 | G | I | L | A | V | A | D | H | L | S | R | L | N | K | A | T | H | G | K | N | G | K | Q | F | L | V | K | D | V | L | E | A | M | I | K | A | H | E | I | Q | G | I | I | A | L | E | N | S | F | N | K | V | G | L | D | 198 | |  | | G0VAM7/1-523 | 209 | H | V | A | L | V | K | I | A | T | T | A | V | V | S | Q | M | L | G | L | S | K | D | E | T | I | A | A | V | S | H | A | F | V | D | G | Q | S | L | R | T | Y | R | H | A | P | N | T | G | S | R | K | S | W | A | A | 263 | | Q6CN98/1-532 | 216 | H | V | V | L | V | K | V | A | T | T | A | V | V | S | K | M | L | G | L | S | E | A | Q | T | T | E | A | L | S | H | A | F | V | D | G | Q | A | L | R | T | Y | R | H | A | P | N | T | G | S | R | K | S | W | A | A | 270 | | Q6FKS3/1-511 | 197 | H | V | I | L | V | K | V | A | T | T | A | V | V | S | Q | M | L | G | L | T | S | E | Q | T | I | E | A | V | S | Q | A | F | V | D | G | Q | S | L | R | T | Y | R | H | A | P | N | T | G | S | R | K | S | W | A | A | 251 | | Q750F2/1-518 | 202 | H | V | V | L | V | K | V | A | T | T | A | V | V | S | K | M | L | N | L | S | E | S | Q | T | V | D | A | L | S | H | A | F | V | D | G | Q | S | L | R | T | Y | R | H | A | P | N | T | G | S | R | K | S | W | A | A | 256 | | A7TSJ9/1-525 | 208 | H | V | I | L | V | K | V | A | T | T | A | V | V | A | K | M | L | G | L | T | K | D | Q | C | E | E | A | I | S | Q | A | F | V | D | G | Q | S | L | R | T | Y | R | H | A | P | N | T | G | S | R | K | S | W | A | A | 262 | | C5DXE8/1-523 | 207 | H | V | V | L | V | K | V | A | T | T | A | V | V | S | K | M | L | G | L | D | E | Q | Q | T | I | A | A | I | S | Q | A | F | V | D | G | Q | S | L | R | T | Y | R | H | A | P | N | T | G | S | R | K | S | W | A | A | 261 | | Sbay\_661.8/1-520 | 203 | H | V | V | L | V | K | V | A | T | T | A | V | V | S | K | M | L | G | L | S | Q | E | Q | T | I | E | A | L | S | Q | A | F | V | D | G | Q | S | L | R | T | Y | R | H | T | P | N | T | G | S | R | K | S | W | A | A | 257 | | SAKL0B02948g/1-524 | 208 | H | V | V | L | V | K | V | A | T | T | A | V | V | S | K | M | L | G | L | T | E | E | Q | T | V | E | A | L | S | Q | A | F | V | D | G | Q | S | L | R | T | Y | R | H | S | P | N | T | G | S | R | K | S | W | A | A | 262 | | Q12428/1-516 | 199 | H | V | V | L | V | K | V | A | T | A | G | V | V | S | K | M | L | G | L | S | Q | E | Q | T | I | E | A | L | S | Q | A | F | V | D | G | Q | S | L | R | T | Y | R | H | A | P | N | T | G | S | R | K | S | W | A | A | 253 | |  | | G0VAM7/1-523 | 264 | G | D | A | V | S | K | A | V | N | I | A | Y | M | V | K | N | A | G | I | E | T | I | P | S | V | L | T | A | P | K | W | G | F | Y | D | V | L | F | K | G | K | P | F | L | F | S | Q | R | K | N | F | D | S | Y | V | 318 | | Q6CN98/1-532 | 271 | G | D | A | V | A | R | A | V | N | I | A | Y | L | V | K | N | G | S | P | G | T | I | P | S | V | L | T | A | K | T | W | G | F | Y | D | V | L | F | K | G | K | P | F | T | F | Q | Q | R | D | T | F | G | S | Y | V | 325 | | Q6FKS3/1-511 | 252 | G | D | A | V | S | R | A | V | N | L | C | Y | L | V | K | N | A | G | V | G | T | I | P | S | V | L | T | A | K | T | W | G | F | Y | D | V | L | F | K | G | E | A | F | H | F | N | Q | R | T | S | F | E | S | Y | V | 306 | | Q750F2/1-518 | 257 | G | D | A | T | S | R | A | V | K | I | A | F | L | V | K | N | G | G | V | D | G | I | P | S | V | L | T | A | K | D | W | G | F | Y | D | V | L | L | K | G | K | P | F | E | F | K | Q | R | K | T | Y | G | S | Y | V | 311 | | A7TSJ9/1-525 | 263 | G | D | A | V | S | R | A | V | N | I | A | Y | L | V | K | N | A | N | V | G | N | I | P | S | V | L | T | A | K | T | W | G | F | Y | D | V | L | F | K | G | K | P | F | V | F | N | Q | R | S | F | Y | D | S | Y | V | 317 | | C5DXE8/1-523 | 262 | G | D | A | V | S | R | A | V | N | L | A | Y | L | I | K | K | A | D | V | G | L | I | P | S | V | L | T | A | P | T | W | G | F | Y | D | V | L | F | K | S | K | P | F | S | F | K | Q | R | N | Q | F | G | S | Y | V | 316 | | Sbay\_661.8/1-520 | 258 | G | D | A | V | S | R | A | V | N | L | A | Y | L | V | K | N | A | N | V | G | T | I | P | S | I | L | T | A | K | T | W | G | F | Y | D | V | L | F | K | G | K | P | F | T | F | N | Q | R | S | A | Y | G | S | Y | V | 312 | | SAKL0B02948g/1-524 | 263 | G | D | A | V | S | R | A | V | N | L | A | F | L | V | K | N | G | H | V | G | T | I | P | S | V | L | T | A | K | T | W | G | F | Y | D | V | L | F | K | G | K | P | F | S | F | K | Q | R | N | S | Y | G | S | Y | V | 317 | | Q12428/1-516 | 254 | G | D | A | V | S | R | A | V | N | L | A | Y | L | V | K | N | A | N | V | G | T | I | P | S | V | L | T | A | R | T | W | G | F | Y | D | V | L | F | K | G | K | P | F | S | F | Q | Q | R | S | K | Y | D | S | Y | V | 308 | |  | | G0VAM7/1-523 | 319 | M | E | N | I | L | F | K | I | S | F | P | A | E | F | H | A | Q | T | A | V | E | A | A | L | T | A | N | K | R | L | K | D | M | G | K | S | F | K | D | I | K | S | V | R | I | R | T | Q | E | P | A | M | R | I | I | 373 | | Q6CN98/1-532 | 326 | M | E | N | V | L | F | K | I | S | F | P | A | E | F | H | A | Q | T | A | V | E | A | A | I | K | V | N | K | Q | L | A | E | L | G | K | T | Y | K | D | I | K | K | V | R | I | R | T | Q | E | A | A | V | R | I | I | 380 | | Q6FKS3/1-511 | 307 | M | E | N | I | L | F | K | I | S | F | P | A | E | F | H | A | Q | T | A | V | E | C | A | M | I | A | H | N | M | L | K | E | K | N | K | T | Y | K | D | I | K | S | V | R | I | R | T | Q | E | A | A | A | R | I | I | 361 | | Q750F2/1-518 | 312 | M | E | N | V | L | F | K | I | S | F | P | A | E | F | H | A | Q | T | A | V | E | A | A | L | K | V | R | K | Q | L | N | R | M | G | K | N | Y | Q | D | I | K | S | I | T | I | R | T | Q | D | A | A | M | R | I | I | 366 | | A7TSJ9/1-525 | 318 | M | E | N | I | L | F | K | I | S | F | P | A | E | F | H | A | Q | T | A | V | E | A | S | M | K | V | H | Q | Q | L | K | D | M | G | K | T | Y | A | D | I | K | S | V | R | I | R | T | Q | N | A | A | V | R | I | I | 372 | | C5DXE8/1-523 | 317 | M | E | N | I | L | F | K | I | S | F | P | A | E | F | H | A | Q | T | A | A | E | A | A | M | K | A | H | V | T | L | K | Q | M | G | K | T | F | R | D | V | K | S | V | R | I | R | T | Q | E | A | A | V | R | I | I | 371 | | Sbay\_661.8/1-520 | 313 | M | E | N | V | L | F | K | I | S | F | P | A | E | F | H | A | Q | T | A | V | E | A | A | V | K | A | Y | N | I | L | N | E | Q | G | K | T | F | R | D | I | K | S | I | R | I | R | T | Q | E | A | A | M | R | I | I | 367 | | SAKL0B02948g/1-524 | 318 | M | E | N | V | L | F | K | I | S | Y | P | A | E | F | H | A | Q | T | A | V | E | A | A | M | K | V | N | A | K | L | N | S | S | G | K | S | Y | K | D | I | K | S | V | R | I | R | T | Q | E | A | A | V | R | I | I | 372 | | Q12428/1-516 | 309 | M | E | N | V | L | F | K | I | S | F | P | A | E | F | H | A | Q | T | A | V | E | A | A | V | K | A | Y | R | I | L | A | K | Q | G | K | T | F | K | D | I | K | S | I | R | I | R | T | Q | E | A | A | M | R | I | I | 363 | |  | | G0VAM7/1-523 | 374 | D | K | S | G | P | L | Y | N | Y | A | D | R | D | H | C | I | Q | Y | M | V | T | I | P | L | I | Q | G | R | L | E | A | D | D | Y | M | D | A | V | A | L | E | P | E | V | D | S | L | R | S | K | I | Y | C | V | K | 428 | | Q6CN98/1-532 | 381 | D | K | E | G | P | L | Y | N | Y | A | D | R | D | H | C | I | Q | Y | M | T | A | I | P | L | I | Y | G | R | L | T | A | D | D | Y | S | D | E | V | A | S | N | P | E | I | D | E | L | R | A | K | M | Y | C | T | K | 435 | | Q6FKS3/1-511 | 362 | D | K | D | G | P | L | Y | N | Y | A | D | R | D | H | C | I | Q | Y | M | T | A | I | P | L | I | Y | G | R | L | T | A | E | D | Y | T | D | E | V | A | G | N | P | D | I | D | A | L | R | A | K | M | Y | C | E | V | 416 | | Q750F2/1-518 | 367 | D | K | S | G | P | L | Y | N | Y | A | D | R | D | H | C | I | Q | Y | M | T | A | V | P | L | I | H | G | R | L | V | A | D | D | Y | H | D | L | V | A | L | N | P | A | I | D | E | L | R | S | K | I | Y | C | V | R | 421 | | A7TSJ9/1-525 | 373 | D | K | S | G | P | L | Y | N | Y | A | D | R | D | H | C | I | Q | Y | M | V | A | I | P | L | I | H | G | R | L | T | A | D | D | Y | S | D | G | I | A | E | N | P | A | I | D | E | L | R | A | K | M | Y | C | V | E | 427 | | C5DXE8/1-523 | 372 | D | K | S | G | P | L | Y | N | Y | A | D | R | D | H | C | I | Q | Y | M | T | A | V | P | L | I | Y | G | R | L | T | A | E | D | Y | T | D | A | I | A | K | Y | P | E | I | D | E | L | R | S | K | M | Y | C | I | K | 426 | | Sbay\_661.8/1-520 | 368 | D | K | S | G | P | L | Y | N | Y | A | D | R | D | H | C | I | Q | Y | M | T | A | I | P | L | I | T | G | N | L | A | A | I | D | Y | S | N | E | V | A | K | N | P | E | I | D | N | L | R | S | K | M | Y | C | V | E | 422 | | SAKL0B02948g/1-524 | 373 | D | K | T | G | P | L | Y | N | Y | A | D | R | D | H | C | I | Q | Y | M | T | A | I | P | L | I | Y | G | R | L | T | A | D | D | Y | S | D | E | I | A | K | N | P | A | I | D | E | L | R | A | K | M | Y | C | V | K | 427 | | Q12428/1-516 | 364 | D | K | S | G | P | L | Y | N | Y | A | D | R | D | H | C | I | Q | Y | M | I | A | V | P | L | I | T | G | N | L | T | A | T | D | Y | S | D | E | V | A | R | N | P | E | I | D | N | L | R | S | K | M | Y | C | I | E | 418 | |  | | G0VAM7/1-523 | 429 | D | E | Q | F | T | K | D | Y | Y | D | P | N | K | R | A | I | P | N | A | L | L | V | E | L | N | D | G | T | V | L | D | E | I | I | V | E | Y | P | I | G | H | R | F | R | R | E | E | G | V | P | L | L | V | E | K | 483 | | Q6CN98/1-532 | 436 | D | D | Q | F | T | L | D | Y | H | D | P | S | K | R | S | I | P | N | A | L | L | V | E | L | N | D | G | T | V | L | D | E | V | V | V | E | Y | P | I | G | H | R | L | R | R | K | E | G | V | P | L | L | M | Q | K | 490 | | Q6FKS3/1-511 | 417 | D | D | T | F | T | A | D | Y | H | H | P | D | K | R | S | I | P | N | A | L | L | I | E | L | E | D | G | T | T | L | D | E | I | V | V | E | Y | P | V | G | H | K | F | R | R | E | E | G | I | P | L | L | M | N | K | 471 | | Q750F2/1-518 | 422 | D | D | Q | F | T | A | A | Y | N | N | P | D | T | R | A | I | P | N | G | L | T | V | E | L | N | D | G | T | T | L | E | E | V | V | V | E | Y | P | I | G | H | K | T | R | R | A | E | A | E | P | K | L | L | E | K | 476 | | A7TSJ9/1-525 | 428 | D | E | N | F | T | Q | D | Y | H | D | P | E | K | R | S | I | G | N | A | L | L | V | E | L | N | D | G | T | V | L | D | E | V | E | V | E | Y | P | V | G | H | R | F | R | R | D | E | G | I | P | L | L | M | K | K | 482 | | C5DXE8/1-523 | 427 | D | D | Q | F | T | L | D | Y | H | N | P | A | K | R | S | I | G | N | A | L | L | I | E | L | N | D | D | T | K | L | D | E | I | V | V | E | Y | P | I | G | H | R | L | R | R | K | E | G | I | P | L | L | L | N | K | 481 | | Sbay\_661.8/1-520 | 423 | D | T | Q | F | S | Q | N | Y | Q | D | P | T | K | R | S | I | G | N | A | L | L | I | E | L | N | D | G | T | H | L | D | E | I | V | V | E | Y | P | V | G | H | K | S | R | R | E | E | G | I | P | L | L | L | N | K | 477 | | SAKL0B02948g/1-524 | 428 | D | D | Q | F | T | L | D | Y | H | N | P | E | M | R | S | I | S | N | A | L | L | V | E | L | E | D | G | T | S | L | E | E | E | I | V | E | Y | P | V | G | H | R | F | R | R | K | E | G | V | P | L | L | L | E | K | 482 | | Q12428/1-516 | 419 | D | T | H | L | T | Q | N | Y | H | D | P | D | K | R | S | I | G | N | A | L | L | I | E | L | N | D | G | T | Q | L | D | E | I | F | V | E | Y | P | V | G | H | K | F | R | R | E | E | G | I | P | L | L | M | N | K | 473 | |  | | G0VAM7/1-523 | 484 | F | Q | R | H | L | S | A | H | Y | I | N | S | P | E | K | A | H | T | I | F | N | A | S | L | N | P | G | L | A | D | L | P | I | D | E | Y | V | D | L | Y | - | - | - |  | | | | | | | | | | | | 523 | | Q6CN98/1-532 | 491 | F | Y | H | H | L | R | E | H | F | A | G | D | I | E | K | V | E | A | L | M | E | A | S | T | D | D | G | F | L | N | L | T | V | D | E | Y | V | N | M | Y | C | D | - |  | | | | | | | | | | | | 532 | | Q6FKS3/1-511 | 472 | F | K | N | L | L | N | G | H | F | K | D | N | - | S | K | A | E | N | I | Y | S | V | S | T | K | D | D | F | D | N | L | D | I | D | D | Y | V | D | L | Y | C | - | - |  | | | | | | | | | | | | 511 | | Q750F2/1-518 | 477 | F | Y | R | H | L | S | G | H | F | E | G | D | I | T | K | V | E | Q | V | M | T | M | S | T | D | P | E | F | E | S | Y | S | I | D | Q | Y | V | D | T | F | C | R | - |  | | | | | | | | | | | | 518 | | A7TSJ9/1-525 | 483 | F | E | R | H | L | S | E | H | F | E | N | N | K | D | H | V | N | K | I | L | E | V | T | K | D | K | D | F | L | N | M | D | V | D | K | Y | I | D | L | Y | V | N | Q |  | | | | | | | | | | | | 525 | | C5DXE8/1-523 | 482 | F | K | T | H | L | N | E | H | F | S | N | S | P | G | R | A | E | E | I | Y | I | K | S | Q | K | S | D | L | E | S | T | P | I | D | S | Y | M | D | L | Y | W | G | - |  | | | | | | | | | | | | 523 | | Sbay\_661.8/1-520 | 478 | F | E | R | H | L | R | E | H | F | T | A | S | P | E | K | V | D | I | I | M | N | A | S | S | K | R | N | F | V | D | M | P | I | D | S | Y | M | N | F | F | V | Q | K |  | | | | | | | | | | | | 520 | | SAKL0B02948g/1-524 | 483 | F | Y | T | H | L | S | Q | H | F | K | G | D | L | E | K | V | E | T | I | M | S | S | S | L | D | E | G | F | E | N | M | S | I | D | E | Y | V | N | L | Y | C | K | - |  | | | | | | | | | | | | 524 | | Q12428/1-516 | 474 | F | Q | R | H | L | R | E | H | F | V | E | S | P | D | K | V | D | L | I | M | K | V | S | S | K | T | N | F | L | N | M | Q | I | D | K | Y | M | D | L | F | T | E | G |  | | | | | | | | | | | | 516 | |
